# Repressing HIF-1α-induced HDAC9 contributes to the synergistic effect of venetoclax and MENIN inhibitor in *KMT2A*r AML

**DOI:** 10.1186/s40364-023-00547-9

**Published:** 2023-12-05

**Authors:** Qing Ling, Yutong Zhou, Yu Qian, Jiejing Qian, Yi Zhang, Jinghan Wang, Yanan Zhu, Yile Zhou, Juying Wei, Chunmei Yang, Jie Sun, Wenjuan Yu, Jie Jin, Xiang Zhang

**Affiliations:** 1 Department of Hematology, The First Affiliated Hospital, Zhejiang University School of Medicine, #79 Qingchun Rd, Hangzhou, 310003 Zhejiang China; 2 Zhejiang Provincial Key Laboratory of Hematologic Malignancy, Zhejiang University, Hangzhou, 310003 Zhejiang P. R. China; 3 Zhejiang Provincial Clinical Research Center for Hematological Disorders, Hangzhou, 310003 Zhejiang P. R. China; 4 Zhejiang University Cancer Center, Hangzhou, 310003 Zhejiang P. R. China

**Keywords:** MENIN inhibitor, Venetoclax, *KMT2A* rearrangement, Acute myeloid leukemia, HIF-1A-induced HDAC9

## Abstract

*KMT2A*-rearranged acute myeloid leukemia (*KMT2A*r-AML) is an aggressive subtype of AML with poor response and prognosis. *KMT2A*r-AML has been demonstrated to be sensitive to BCL2 inhibitor venetoclax (VEN), but these patients are unable to benefit from current VEN-based regimen (VEN plus azacitidine or low dose-cytarabine), so a novel and *KMT2A* rearrangement-specific targeting partner is required, and MENIN inhibitor (MEN1i) is a promising one. Herein, we investigated the effect and mechanism of VEN plus MEN1i in *KMT2Ar*-AML. Our results showed that VEN and MEN1i exhibited a striking synergistic effect in *KMT2Ar-*AML cell lines (in vitro), primary *KMT2Ar-*AML cells (ex vivo), and MOLM13 xenotransplantation model (in vivo). Furthermore, we found that VEN plus MEN1i significantly enhanced apoptotic induction in *KMT2Ar*-AML cell lines. VEN or MEN1i monotherapy disrupted balance of BCL-2/BCL-XL or down-regulated *HOXA9*/*MEIS1*, respectively, but these mechanisms were not further strengthened by their combination. RNA-Sequencing identified that *HDAC9* was specifically repressed by VEN plus MEN1i rather than monotherapy. We demonstrated that HDAC9 was indispensable for *KMT2Ar-*AML proliferation and its repression contributed to proliferation inhibition of VEN plus MEN1i. Moreover, we found that hypoxia induced HDAC9 expression in *KMT2Ar-*AML, and VEN plus MEN1i inhibited hypoxia pathway, especially HIF-1A, and its target HDAC9. As our results indicated, VEN plus MEN1i-mediated HDAC9 down-regulation was partially dependent on HIF-1A repression in *KMT2Ar*-AML. Hypoxia induction sensitized *KMT2Ar*-AML to VEN plus MI-503-mediated proliferation inhibition and apoptosis induction. Therefore, repressing HIF-1A-induced HDAC9 contributed to the synergistic effect of VEN and MEN1i in *KMT2A*r-AML.

**Supplementary Information:**

The online version contains supplementary material available at 10.1186/s40364-023-00547-9.

To the editor,

*KMT2A*r-AML exhibits poor response and prognosis, and new therapy is urgently required. *KMT2Ar-*AML is sensitive to VEN [1], but these patients fail to benefit from current VEN plus AZA/LDAC regimen [2, 3], so a novel partner typically targeting KMT2A rearrangements is needed. MENIN-KMT2A complex is required for *KMT2Ar-*AML initiation and maintenance. Consistently, *KMT2Ar-*AML is sensitive to disruption of MENIN-KMT2A [4], so MEN1i is a promising candidate. Herein, we investigated VEN plus MEN1i in *KMT2Ar-*AML (supplemental Materials and Methods).

Initially, we tested VEN or MEN1i MI-503 monotherapy in AML cell lines, and found that *KMT2Ar-*AML (MV4-11, MOLM13 and THP-1) was sensitive to both of agents (Fig. 1A-B; Fig. S1). To exclude MI-503-specific effects, we tested additional MEN1is (MI-463 and VTP-50469) and demonstrated their sensitivity in *KMT2Ar-*AML (Fig. S2). *NPM1-*mutated AML is also dependent on MENIN-KMT2A complex [5], but OCI-AML3 (*NPM1-*mutated AML) was less sensitive to MEN1is than *KMT2Ar-*AML (Fig. 1B; Fig. S3), so *KMT2Ar-*AML is the best indication of MEN1i.

**Fig. 1:**
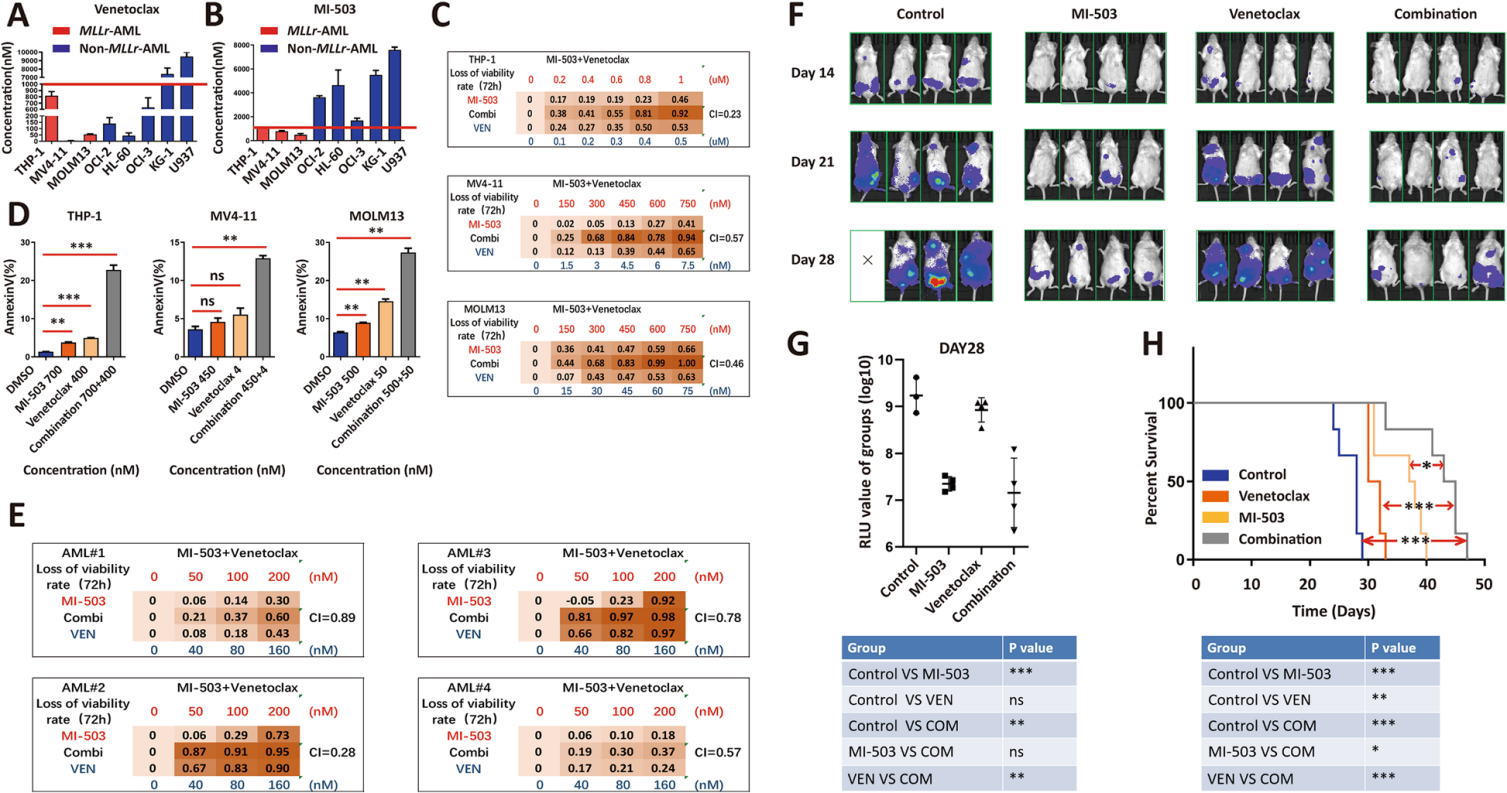
VEN and MEN1i synergistically inhibited *KMT2A*r-AML proliferation. **A** IC50 of VEN in different AML cell lines (72 h); **B** IC50 of MI-503 in different AML cell lines (7 days); **C** Growth inhibition and synergistic index (combination index [CI] was calculated via CalcuSyn software) in THP-1 (expressing *KMT2A-MLLT3*), MV4-11 (expressing *KMT2A-AFF1*), and MOLM13 (expressing *KMT2A-MLLT3*) cells after treatments with different concentrations of VEN, MI-503, and their combination (72 h); **D** Apoptotic induction (Annexin V-positive cells) was determined after treatments with VEN, MI-503, and their combination (72 h) [two-tailed Student's t-tests; **P < 0.01, ***P < 0.001]; **E** Growth inhibition and synergistic index of VEN plus MI-503 in primary bone marrow MNCs from *KMT2Ar*-AML patients (72 h); **F** Detecting the progression of tumor burden via fluorescence imaging after treating the CDX mouse model constructed by MOLM13-Luciferase with DMSO, VEN, MI-503 and their combination. **G** The relative luminescence unit (RLU) value was calculated for indicating leukemic burdens of treated mice (28 days) [two-tailed Student's t-tests; *P < 0.05, **P < 0.01, ***P < 0.001]. **H** Kaplan–Meier survival curves in the MOLM13 xenotransplantation model [two-tailed Student's t-tests; *P < 0.05, **P < 0.01, ***P < 0.001]

Then, we investigated combination therapy of VEN and MEN1i in *KMT2Ar-*AML. Notably, an effective synergy in proliferation inhibition was observed in *KMT2Ar-*AML but not non-*KMT2Ar-*AML cell lines (Fig. 1C; Fig. S4-S5). Furthermore, we found that VEN plus MI-503 consistently enhanced apoptotic induction, but not cell cycle arrest or cell differentiation in three *KMT2Ar-*AML cells (Fig. 1D; Fig. S6). Besides, the synergistic effect of VEN plus MI-503 was selectively exhibited in primary *KMT2Ar-*AML but not non-*KMT2Ar-*AML or NBM cells (Fig. 1E; Fig. S7; Tab. S1). In vivo study, our results revealed that compared to VEN or MI-503 monotherapy, their combination exhibited better effects of reducing the leukemic burden and prolonging the survival duration in MOLM13 xenotransplantation model (Fig. 1F-H; Fig. S8). Therefore, VEN and MEN1i showed a striking synergistic effect in *KMT2Ar-*AML.

As reported, VEN and MI-503 mainly inhibited leukemia via disrupting balance of BCL-2/BCL-XL and down-regulating *HOXA9*/*MEIS1*, respectively. When VEN and MI-503 were combined, imbalance of BCL-2/BCL-XL or down-regulation of *HOXA9*/*MEIS1* were not further enhanced by MI-503 or VEN, respectively (Fig. S9). To uncover their synergistic mechanism, RNA-sequencing was displayed in *KMT2Ar-*AML (Fig. S10). To select possible candidates contributing to their synergy, we adapted indicated strategies, and 34 genes, specifically regulated by combination therapy, were identified. After screening above genes based on their function and correlation with *KMT2Ar-*AML, and we focused on *HDAC9* (Fig. 2A; Tab. S2, S3, S4, S5, S6, S7, S8, S9 and S10). High *HDAC9* expression is associated with *KMT2Ar*-leukemia [6]. Therefore, *HDAC9* was a possible target for VEN plus MI-503 in *KMT2Ar*-AML. First, we demonstrated that *HDAC9*, but not other *HDAC*s, was consistently down-regulated in *KMT2Ar*-AML specifically at 72 h treatment (Fig. 2B; Fig. S11-12), and its protein was down-regulated accordingly, but this phenomenon was not observed in OCI-AML3 (Fig. 2C; Fig. S13). Second, *HDAC9* inhibition with TMP-269 or shRNA impaired *KMT2Ar-*AML proliferation, though their *HDAC9* levels were not all higher than non-*KMT2Ar-*AML cell lines (Fig. 2D-E; Fig. S14-15). Third, combination-mediated proliferation inhibition was not further enhanced by *HDAC9* knockdown in *KMT2Ar*-AML (Fig. 2F). Therefore, HDAC9 was required for *KMT2Ar-*AML maintenance, and VEN plus MI-503 exerted *HDAC9* repression to enhance their proliferative inhibition in *KMT2Ar-*AML.

**Fig. 2:**
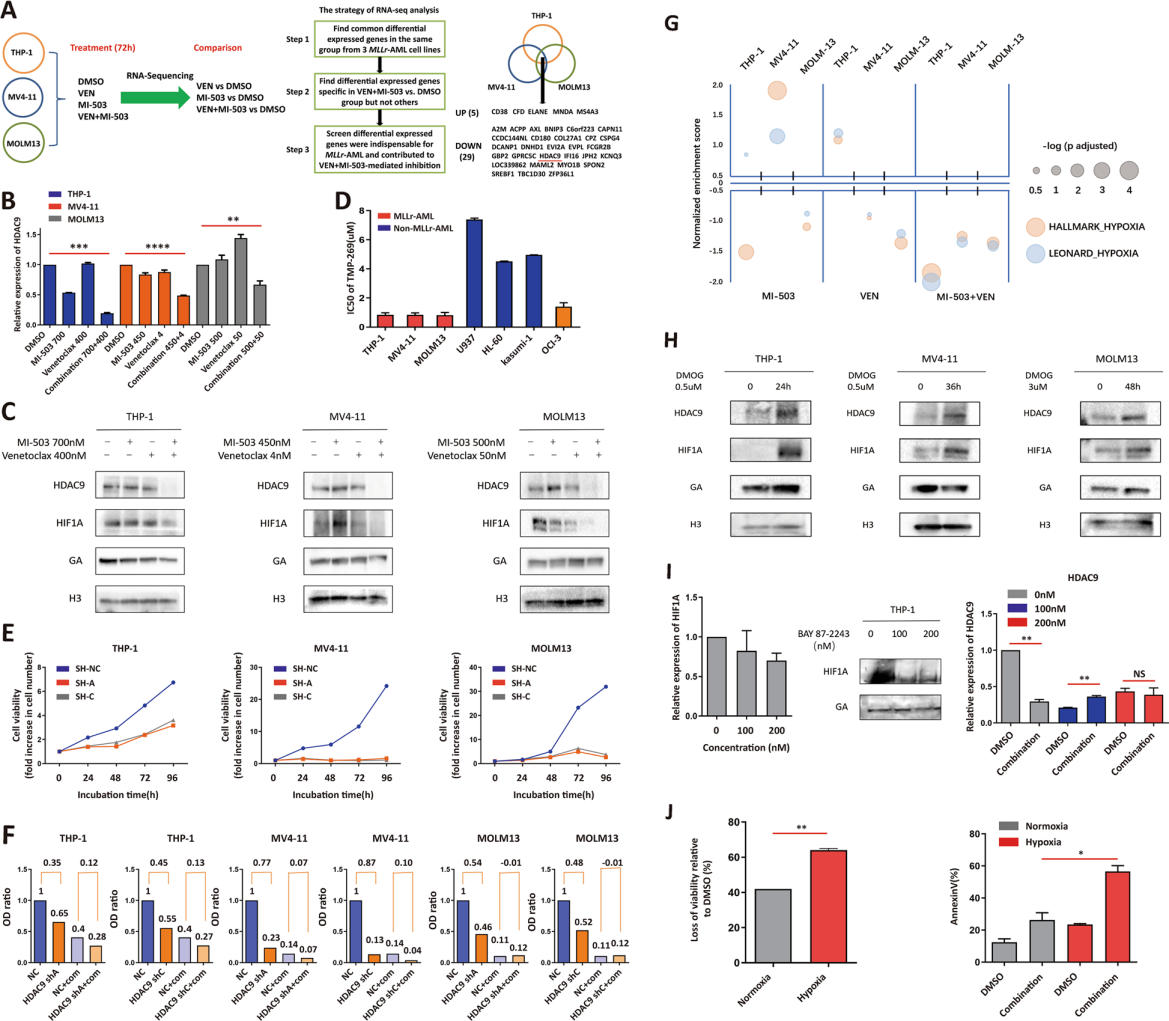
VEN plus MEN1i impaired *KMT2A*r-AML growth via repressing HIF-1A-induced HDAC9. **A** After treating *KMT2A*r-AML cell lines THP-1, MV4-11, MOLM13 with DMSO, VEN, MI-503 and their combination for 72 h, RNA sequencing was performed and differentially expressed genes (DEGs) were analyzed; Overall strategy for screening key DEGs related to the synergistic mechanism of VEN plus MI-503 in *KMT2A*r-AML was shown; Specific DEGs in VEN plus MI-503 group vs. DMSO group shared by three *KMT2A*r-AML cell lines were listed; **B** The mRNA expression of *HDAC9* in THP-1, MV4-11, and MOLM13 after treatments with DMSO, VEN, MI-503, and their combination [two-tailed Student's t-tests; **P < 0.01, ***P < 0.001 ****P < 0.0001]; **C** The protein expression of HDAC9 and HIF1A in THP-1, MV4-11, and MOLM13 after treatments with DMSO, VEN, MI-503 and their combination; **D** IC50 of TMP-269 in THP-1, MV4-11, MOLM-13, U937, HL-60, Kasumi-1, and OCI-AML3 cells (72 h); **E** Growth inhibition was mediated by *HDAC9* knockdown in THP-1, MV4-11, and MOLM13 cells; **F** Different therapeutic effects of VEN plus MI-503 in normal control or *HDAC9* knockdown THP-1, MV4-11, and MOLM13 cells were exhibited; Relative absorbance value (optical density [OD] ratio) to normal control for each group was presented and the difference value of OD ratio between two groups was marked as numbers between the histogram bars; **G** Hypoxia pathway gene set was enriched in the VEN plus MI-503 group compared to DMSO group, the horizontal axis represents the p-value, the vertical axis represents the standard enrichment fraction (NES), the size of the circle represents the -log (p adjusted) value, and different colors represent different gene sets; **H** The protein expression of HDAC9 and HIF1A in THP-1, MV4-11, and MOLM13 treated with DMOG. **I** Under HIF-1A inhibitor BAY 87-2243 treatment, HIF-1A mRNA (left) and protein (middle) levels were evaluated. Furthermore, *HDAC9* mRNA was detected in DMSO or VEN (400 nM) plus MI-503 (700 nM)-treated THP1 cells with or without BAY 87-2243 3-day pre-treatment (72 h) [two-tailed Student's t-tests; *P < 0.05, **P < 0.01, ***P < 0.001]. **J** Under normoxia and hypoxia, THP-1 cells were treated by DMSO or VEN plus MI-503, and cell viability and cell apoptosis were evaluated [two-tailed Student's t-tests; **P < 0.01, ***P < 0.001 ****P < 0.0001]

To explore how HDAC9 was repressed, we conducted GSEA and showed that hypoxia pathway was inhibited by VEN plus MI-503 (Fig. 2G; Fig. S16; Tab. S11). HDAC9 was reported to be induced by hypoxia, especially HIF-1A [7]. In our study, hypoxia inducer DMOG stabilized HIF-1A and up-regulated HDAC9 in *KMT2Ar-*AML (Fig. 2H). VEN plus MI-503 repressed HIF-1A and its target expression, while *HDAC9* repression was attenuated in THP-1 cells with HIF-1A inhibition (Fig. 2C, I; Fig. S17). Moreover, VEN plus MI-503 exerted a more remarkable proliferation inhibition and apoptosis induction in THP-1 under hypoxia than normoixa (Fig. 2J). Thus, VEN plus MI-503 down-regulated HDAC9 via suppressing HIF-1A, which contributed to their synergistic effect.

MEN1i monotherapy exerts its best inhibition taking more than 7 days, and easily induced MEN1 mutations to mediate resistance [8]. VEN works within 48-72 h, so it can overcome the long onset duration of MEN1i and reduce chances of resistance in combination. As reported, both of VEN and MEN1i are oral available and target LSCs [9, 10], so VEN plus MEN1i is possibly a bi-oral and bi-LSCs-targeted regimen, which will make evolutions in therapeutic concept for *KMT2Ar*-AML. How VEN and MI-503 cooperate still lacks in recent studies [11, 12], and we demonstrated that beyond each effect of monotherapy, repression of HIF-1A-induced HDAC9 mainly contributed to enhanced proliferative inhibition in combination.

Collectively, targeted therapy with VEN plus MEN1i is promising in *KMT2Ar-*AML.

### Supplementary Information

**Additional file 1:** **Supplementary Information.** Materials and Methods, Figure S1-S18. **Figure S1.** *KMT2Ar*-AML was sensitive to MI-503. The IC50 of MI-503 in AML cell lines (72 h). **Figure S2.** MEN1i inhibited the proliferation of *KMT2Ar*-AML cell lines. (A-C) IC50 of MENis, such as MI-503 (A), MI-403 (B), and VTP50469 (C), in THP-1, MV4-11, and MOLM13 cells (7 days). **Figure S3.** OCI-AML3 was relatively less sensitive to MEN1i than *KMT2Ar*-AML. (A, B) The IC50 of MI-463 (A) and VTP50469 (B) in OCI-AML3 and *KMT2Ar*-AML cell lines (72 h). **Figure S4.** No significant synergistic effects of VEN plus MEN1i were observed in non-*KMT2Ar*-AML cell lines. Growth inhibition and synergistic index of VEN plus MI-503 in HL-60, OCI-AML2, OCI-AML3, KG-1, and U937 cells (72 h). **Figure S5.** VEN plus MI-463 or VTP-50469 also cooperated in the inhibition of *KMT2A*r-AML cell lines. (A-B) Growth inhibition and synergistic index of VEN plus MI-403 (A) or VTP-50469 (B) in THP-1, MV4-11, and MOLM13 cells (72 h). **Figure S6.** VEN plus MI-503 did not influence cell cycle distribution and cell differentiation of *KMT2Ar*-AML cell lines. (A) Cell cycle analysis for THP-1, MV4-11, and MOLM13 cells after single-agent or combinatorial treatment with VEN and MI-503 (72 h); (B) Cell differentiation was determined by CD11b and CD14 staining in THP-1, MV4-11, and MOLM13 cells after single-agent or combinatorial treatment with VEN and MI-503 (72 h). **Figure S7.** No significant synergistic effects of VEN plus MEN1i were observed in primary bone marrow MNCs from non-*KMT2A*r-AML patients or healthy donors. (A-B) Growth inhibition and synergistic index of VEN plus MI-503 in primary bone marrow MNCs from non-*KMT2Ar*-AML patients (A) or healthy donors (B) (72 h). **Figure S8.** Leukemic burden in treated MOLM13 xenotransplantation model. The relative luminescence unit (RLU) value was calculated for indicating leukemic burdens of treated mice (14 days[A] and 21 days[B]). **Figure S9.** The synergistic mechanism of VEN plus MI-503 was not related to further downregulation of *HOXA9* and *MEIS1* or disruption of BCL2/BCL-XL balance. (A) The mRNA expression of *HOXA9* and *MEIS1* in THP-1, MV4-11, and MOLM13 after treatments with DMSO, VEN, MI-503 and their combination (72 h) [two-tailed Student's t-tests; *P < 0.05, **P < 0.01, ***P < 0.001 ****P < 0.0001]; (B-C) The mRNA (B) and protein (C) expression of BCL2, BCL-XL and MCL-1 in THP-1, MV4-11, and MOLM13 after treatments with DMSO, VEN, MI-503 and their combination (72 h). **Figure S10.** Global transcriptional regulation of VEN plus MI-503 in *KMT2Ar*-AML cell lines. DEGs in three *KMT2Ar*-AML cell lines. **Figure S11.** VEN plus MI-503 specifically downregulated *HDAC9*. The mRNA expression of HDAC family in THP-1, MV4-11, and MOLM13 after treatments with DMSO, VEN, MI-503 and their combination (72 h). **Figure S12.** *HDAC9* was not consistently down-regulated by VEN plus MI-503 until 72 treatments. (A-C) *HDAC9 *mRNA was detected under treatments for 12 h (A), 24 h (B), and 48 h (C), respectively. **Figure S13.** HDAC9 was not repressed by VEN plus MI-503 in OCI-AML3. (A, B) OCI-AML3 was treated by DMSO, VEN, MI-503, or VEN plus MI-503 at 72 h, and *HDAC9* mRNA (A) and protein (B) were detected. **Figure S14.** *HDAC9* knockdown was displayed in *KMT2Ar*-AML cell lines. *HDAC9* was detected in *KMT2Ar*-AML cell lines after shRNA-mediated knockdown. **Figure 15.** *HDAC9* expression in AML cell lines. (A-B) *HDAC9* expression was determined in our AML cell lines (A) and obtained from Cancer Cell Line Encyclopedia (CCLE) database (B). **Figure S16.** VEN plus MI-503 inhibited the expression of hypoxia pathway. (A-B) HALLMARK_HYPOXIA (A) and LEONARD_HYPOXIA (B) gene sets for MI-503, VEN, or VEN plus MI-503 compared to DMSO, respectively. **Figure S17.** VEN plus MI-503 repressed the targets of HIF-1A. The mRNA expression of *ALDOC*, *ADM*, *ENO3*, *PNRC1* and *TMEM45A* in THP-1, MV4-11, and MOLM13 after treatments with DMSO, VEN, MI-503 and their combination (72 h) [two-tailed Student's t-tests; *P < 0.05, **P < 0.01, ***P < 0.001 ****P < 0.0001]. **Figure S18.** The band quantification for western blot. (A-C) The band was quantified for Fig. 2C (A), 2H (B), and Figure S9C (C), respectively.

**Additional file 2:** **Table S1.** The clinical and genetic features of primary sample donors.

**Additional file 3:** **Table S2.** Different expressed genes of MI-503 vs. DMSO in MV4-11.

**Additional file 4: Table S3.** Different expressed genes of VEN vs. DMSO in MV4-11. 

**Additional file 5: Table S4.** Different expressed genes of MI-503 plus VEN vs. DMSO in MV4-11. 

**Additional file 6: Table S5.** Different expressed genes of MI-503 vs. DMSO in MOLM13. 

**Additional file 7: Table S6.** Different expressed genes of VEN vs. DMSO in MOLM13. 

**Additional file 8: Table S7.** Different expressed genes of MI-503 plus VEN vs. DMSO in MOLM13. 

**Additional file 9: Table S8.** Different expressed genes of MI-503 vs. DMSO in THP-1. 

**Additional file 10: Table S9.** Different expressed genes of VEN vs. DMSO in THP-1. 

**Additional file 11: Table S10.** Different expressed genes of MI-503 plus VEN vs. DMSO in THP-1. 

**Additional file 12: Table S11.** FPKM of different groups in three *KMT2Ar-*AML cell lines.

## Data Availability

The datasets used and/or analyzed during the current study are available from the corresponding author on reasonable request.

## Abbreviations

AZA: Azacitidine
GSEA: Gene Set Enrichment Analysis
*KMT2A*r-AML: *KMT2A-*rearranged acute myeloid leukemia
LDAC: Low dose-cytarabine
LSCs: Leukemic stem cells
MEN1i: MENIN inhibitor
NBM: Normal bone marrow
VEN: Venetoclax
