# Supplementary material for: Repressing HIF-1α-induced HDAC9 contributes to the synergistic effect of venetoclax and MENIN inhibitor in KMT2Ar AML
Source: Biomark Res. 2023 Dec 5;11:105. doi: 10.1186/s40364-023-00547-9 (PMC10696732; doi:10.1186/s40364-023-00547-9)
Supplement: Supplementary file 1 — Additional file 1: Supplementary Information. Materials and Methods, Figure S1-S18. Figure S1. KMT2Ar-AML was sensitive to MI-503. The IC50 of MI-503 in AML cell lines (72 h). Figure S2. MEN1i inhibited the proliferation of KMT2Ar-AML cell lines. (A-C) IC50 of MENis, such as MI-503 (A), MI-403 (B), and VTP50469 (C), in THP-1, MV4-11, and MOLM13 cells (7 days). Figure S3. OCI-AML3 was relatively less sensitive to MEN1i than KMT2Ar-AML. (A, B) The IC50 of MI-463 (A) and VTP50469 (B) in OCI-AML3 and KMT2Ar-AML cell lines (72 h). Figure S4. No significant synergistic effects of VEN plus MEN1i were observed in non-KMT2Ar-AML cell lines. Growth inhibition and synergistic index of VEN plus MI-503 in HL-60, OCI-AML2, OCI-AML3, KG-1, and U937 cells (72 h). Figure S5. VEN plus MI-463 or VTP-50469 also cooperated in the inhibition of KMT2Ar-AML cell lines. (A-B) Growth inhibition and synergistic index of VEN plus MI-403 (A) or VTP-50469 (B) in THP-1, MV4-11, and MOLM13 cells (72 h). Figure S6. VEN plus MI-503 did not influence cell cycle distribution and cell differentiation of KMT2Ar-AML cell lines. (A) Cell cycle analysis for THP-1, MV4-11, and MOLM13 cells after single-agent or combinatorial treatment with VEN and MI-503 (72 h); (B) Cell differentiation was determined by CD11b and CD14 staining in THP-1, MV4-11, and MOLM13 cells after single-agent or combinatorial treatment with VEN and MI-503 (72 h). Figure S7. No significant synergistic effects of VEN plus MEN1i were observed in primary bone marrow MNCs from non-KMT2Ar-AML patients or healthy donors. (A-B) Growth inhibition and synergistic index of VEN plus MI-503 in primary bone marrow MNCs from non-KMT2Ar-AML patients (A) or healthy donors (B) (72 h). Figure S8. Leukemic burden in treated MOLM13 xenotransplantation model. The relative luminescence unit (RLU) value was calculated for indicating leukemic burdens of treated mice (14 days[A] and 21 days[B]). Figure S9. The synergistic mechanism of VEN plus MI-503 was not re [file 40364_2023_547_MOESM1_ESM.docx]

**Supplementary Information**

**Materials and Methods**

**1 Cell culture and shRNA-mediated knockdown**

MV4-11 and MOLM13 were cultured in IMDM medium containing L-glutamine (Gibco, C12440500BT) with 10% fetal bovine serum (FBS) (Thermo Fisher Scientific, 10099-141C) at 37°C in a humidified atmosphere with 5% CO_2_. OCI-AML3, THP-1, HL-60, OCI-AML2, KG-1, U937, and Kasumi-1 were cultured in RPMI-1640 medium containing L-glutamine (Gibco, C11875500BT) with 10% FBS at the same condition. In normoxia culture, the concentration of oxygen was 20%; in hypoxia culture, the concentration of oxygen was 1%. For *HDAC9* knockdown, three independent shRNA sequences were designed and added into the psi-LVRU6GP vector. The shRNA-A and shRNA-C sequences were selected for subsequent experiments due to their high knockdown efficiency. THP-1, MV4-11, MOLM13 were transfected with shRNA lentiviral particles with *HDAC9* shRNA or Scramble. Targeted sequences for *HDAC9* shRNA and non-silencing control shRNA were attached below. Scramble: 5’- ACAGAAGCGATTGTTGATC - 3’; ShRNA-A: 5’ - GCAATTGCAGCAGGAATTACT - 3’; ShRNA-C: 5’ - GCAACGAAAGACACTCCAACT - 3’.

**2 Primary cells from patients**

Bone marrow cells were obtained from four *KMT2A*-rearranged AML, four non *KMT2A*-rearranged AML patients and four healthy donors. Mononuclear cells (MNCs) were isolated through mononuclear cell separation medium (Ficoll Paque Premium) and immediately used for subsequent experiments. Gene fusions and mutations have been determined by the First Affiliated Hospital, College of Medicine, Zhejiang University.

**3 Reagents and antibodies**

For treating cells or mouse models, MI-503 (MedChemExpress [MCE], HY-16925), VTP50469 (MCE, HY-114162), MI-463 (MCE, HY-19809), Venetoclax (MCE, HY-15531), DMOG (MCE, HY-15893), TMP-269 (AdooQ, A14128), and BAY 87-2243 (MCE, HY-15836) were used. For western blotting, antibodies, including BCL-2 (Cell Signaling Technology [CST], #3498), BCL-XL (CST, #2764), MCL-1 (CST, #94296), H3 (CST, #4499), GAPDH (CST, #5174), PARP (CST, #9532), Caspase3 (CST, #9662), HDAC9 (Abcam, ab109446; Proteintech, 67364-1-Ig), and HIF1A (Abcam, ab51608) were used. For flow cytometry analysis, Annexin V-FITC/PI apoptosis kit (MULTI SCIENCES, AP101), cell cycle staining kit (MULTI SCIENCES, CCS012), CD11b-PE (MULTI SCIENCES, AH011B01-50) and CD14-FITC (MULTI SCIENCES, AH01404-100) were used.

**4 Cell proliferation, cell apoptosis, cell cycle and cell differentiation**

To analyze the cell proliferation, cells were seeded into 24-well plates with 100,000 cells and 1 mL medium (cell concentration, 1 x 10^5^ cells/mL) per well with or without drugs. Cells from each well were homogeneously transferred into three wells with a total volume of 100μL for each well in 96-well plate and supplemented with 10μL MTS (5mg/mL) (Promega, G3581). Cells were incubated for 4h at 37°C and absorbance was determined at 490 nm. To test cell apoptosis, collected cells were resuspended twice in PBS and AV as well as PI were added. Cells were incubated for 30 minutes at 37°C and then detected by flow cytometry (Becton Dickinson). To determine the cell cycle distribution, cells were collected without synchronization and then fixed with 75% ethanol at -20°C for 24h. Then, cells were stained with 250-300µL buffer (DNA staining solution) as cell cycle staining kit indicated. After incubated for 30 minutes at 37°C, cells were detected by flow cytometry. To explore the state of cell differentiation, cells were collected after 3-day drug treatments, and CD11b-PE as well as CD14-FITC were stained for flow cytometry analysis.

**5 Synergistic effects for combination therapy**

To decide whether synergistic effects existed between two agents, we first treated cells with single agents and found a concentration with 30-40% inhibition rate (IC_30-40_), then chose the gradient around and including the IC_30-40_ for combined treatments, and finally calculated the combination index [CI] via CalcuSyn software. It was widely accepted that concentration with 50% inhibition rate (IC_50_) less than 1μM was recognized as sensitive to this agent *in vitro*, and the IC_50_ was possibly achieved *in vivo* for effective treatments. To determine if an effective synergy was created, not only CI should be less than 1, but also concentrations for each agent at high combined inhibition rate (70%-80%) should be less than 1μM.

**6 Cell derived xenograft (CDX) mouse model and *in vivo* study**

Severe immune-deficient strain NCG (NOD/ShiLtJGpt-Prkdcem26Cd52Il2rgem26Cd22/Gpt) mice were purchased from GemPharmatech Co, Ltd (Nanjing, China). 1 × 10^5^ MOLM13-Luci cells were injected into sixteen 6-week-old female NCG mice through the tail vein. They were randomized into the following four treatment groups: Vehicle, MI-503 (50 mg/kg; intraperitoneal injection, twice daily), venetoclax (75 mg/kg; orally, once daily) or combination of both drugs after being bred for one week. MI-503 was dissolved in the solution of 7% DMSO, 43% PEG300, 5% tween-80, 45% saline, and VEN was dissolved in carboxymethyl cellulose. After 11 days, the four groups were treated as the drug described above. Mice’s body weight was determined once a week. Growth of the leukemia cells was monitored using an IVIS every week 14 days after injection of mouse cells and the tumor burden was analyzed. Mice were euthanized when they developed a bowed back and their lower limbs were paralyzed. The survival curve of mice was generated. Data of 4 mice in each group were included in the final statistical analysis. Mice dying for nonrelated cancer causes were excluded from the studies (such as fights or infections). Each experimental group comprised mice derived from the different brood.

**7 Western blotting**

Cells were lysed using RIPA buffer (Thermo Fisher Scientific, 89900) supplemented with protease inhibitor and phosphatase inhibitor cocktail (Thermo Fisher Scientific, 1861280) on ice for 30 min. The cell lysate was centrifuged at 12,000 g for 15 min at 4°C and the protein concentration of the cellular supernatant was determined using a BCA reagent (Thermo Fisher Scientific, 23228[reagent A] and 23224[reagent B]). Approximately 40-60μg protein was loaded per well on 4-12% SDS-PAGE gel and transferred onto PVDF membrane (Millipore, IPVH00010) preactivated with methanol. Membranes were blocked using 5% non-fat milk for 1 h and incubated with primary antibodies overnight at 4°C. After incubation, membranes were washed thrice with TBST and incubated with secondary antibodies (CST, #7076S; Proteintech, SA00001-2) for 1 h at room temperature. Target proteins were then visualized using a ECL detection kit (Thermo Fisher Scientific, 34096) and analyzed using Image Lab™ software (Bio-Rad).

**8 Reverse transcription-quantitative polymerase chain reaction (RT-qPCR)**

Total RNA was extracted from cells using Trizol reagent (Invitrogen, 9109). RNA was reverse transcribed to cDNA using a Reverse Transcription Kit (TAKARA, RR036A-1). Quantitative real-time PCR was carried out using SYBR Green qPCR Master Mix (TAKARA, RR420) and CFX96/384 Real-Time PCR detection systems (Bio-Rad). A total volume of 10µl containing 1µl of 100ng/µl sample cDNA, 5µl of 2 × PCR Mix, 0.2µl of 0.5µM of each primer and 3.6µl of ddH_2_O was used for PCR reactions. Quantification was carried out using ∆∆CT method and the expression level of GAPDH was used as a control to normalize values across different target genes. The primers used for qPCR were listed below.

| **Gene** | **Primers** |
| --- | --- |
| *HOXA9* | Forward 5' - GTCGTGGACCCTGACATTTTC - 3';  Reverse 5' - CCTTAAAGACTTCCCCATACGTG - 3' |
| *MEIS1* | Forward 5' - TACCCGCACACAGCTCATAC - 3';  Reverse 5' - CATTGAATGACTCTGACGAGCA - 3' |
| *BCL-2* | Forward 5' - GGTGGGGTCATGTGTGTGG - 3';  Reverse 5' - CGGTTCAGGTACTCAGTCATCC - 3' |
| *BCL-XL* | Forward 5' - GAGCTGGTGGTTGACTTTCTC - 3';  Reverse 5' - TCCATCTCCGATTCAGTCCCT - 3' |
| *MCL-1 L* | Forward 5' - GTGCCTTTGTGGCTAAACACT - 3';  Reverse 5' - AGTCCCGTTTTGTCCTTACGA - 3' |
| *MCL-1 S* | Forward 5' - GGCCTTCCAAGGATGGGTTT - 3';  Reverse 5' - ACTCCAGCAACACCTGCAAAA - 3' |
| *HDAC9* | Forward 5' - AGTAGAGAGGCATCGCAGAGA - 3';  Reverse 5' - GGAGTGTCTTTCGTTGCTGAT - 3' |
| *HDAC1* | Forward 5' - CGCCCTCACAAAGCCAATG - 3';  Reverse 5' - CTGCTTGCTGTACTCCGACA - 3' |
| *HDAC2* | Forward 5' - ATGGCGTACAGTCAAGGAGG - 3';  Reverse 5' - TGCGGATTCTATGAGGCTTCA - 3' |
| *HDAC3* | Forward 5' - CCTGGCATTGACCCATAGCC - 3';  Reverse 5' - CTCTTGGTGAAGCCTTGCATA - 3' |
| *HDAC4* | Forward 5' - GGCCCACCGGAATCTGAAC - 3';  Reverse 5' - GAACTCTGGTCAAGGGAACTG - 3' |
| *HDAC5* | Forward 5' - GGTGTGGTCTACGACACGTTC - 3';  Reverse 5' - GATCCGCTCGCACTTGCTAA - 3' |
| *HDAC6* | Forward 5' - AAGAAGACCTAATCGTGGGACT - 3';  Reverse 5' - GCTGTGAACCAACATCAGCTC - 3' |
| *HDAC7* | Forward 5' - GGCGGCCCTAGAAAGAACAG - 3';  Reverse 5' - CTTGGGCTTATAGCGCAGCTT - 3' |
| *HDAC8* | Forward 5' - TCGCTGGTCCCGGTTTATATC - 3';  Reverse 5' - TACTGGCCCGTTTGGGGAT - 3' |
| *HDAC10* | Forward 5' - CAGTTCGACGCCATCTACTTC - 3';  Reverse 5' - CAAGCCCATTTTGCACAGCTC - 3' |
| *HDAC11* | Forward 5' - CACGCTCGCCATCAAGTTTC - 3';  Reverse 5' - GAAGTCTCGCTCATGCCCATT - 3' |
| *ALDOC* | Forward 5' - ATGCCTCACTCGTACCCAG - 3';  Reverse 5' - TTTCCACCCCAATTTGGCTCA - 3' |
| *ADM* | Forward 5' - ATGAAGCTGGTTTCCGTCG - 3';  Reverse 5' - GACATCCGCAGTTCCCTCTT - 3' |
| *ENO3* | Forward 5' - TATCGCAATGGGAAGTACGATCT - 3';  Reverse 5' - AAGCTCTTATACAGCTCTCCGA - 3' |
| *PNRC1* | Forward 5' - ACTTGCCACTAACCAAGATCAC - 3';  Reverse 5' - TTGGAAGAACACTAGGAGAAGGT - 3' |
| *TMEM45A* | Forward 5' - GTTCACTTCCTGTGTCCTTAACC - 3';  Reverse 5' - CATTTCCCGGCCATGAGTGT - 3' |
| *GAPDH* | Forward 5’ - GGAGCGAGATCCCTCCAAAAT - 3’;  Reverse 5’ - GGCTGTTGTCATACTTCTCATGG - 3’ |

**9 RNA-sequencing**

For gene expression analysis, THP-1, MV4-11 or MOLM13 cells were treated with control solvent, MI-503, Venetoclax and combination of both drugs, respectively. RNA sequencing was performed using total RNA samples isolated from these 12 samples using HiSeq X Ten platforms (Illumina). Expression levels of mRNA in sequence data were calculated as RPKM (Reads Per Kilo-base per Million reads). As for enrichment pathway map, we first Performed differential analysis within the cell line to obtain differentially expressed genes, then we used R language software packages such as clusterProfiler, org.Hs.eg.db, ReactomePA, msigdbr, DOSE, enrichplot, ggplot2, ggupset for GSEA analysis based on the differentially expressed genes, finally we extracted the pathway enrichment results of interest for plotting, and the enrichment results of each comparative group within the cell line were displayed in one graph. To further draw a bubble diagram of enrichment pathways, we used R language software packages such as ggplot2 and ggbreak, the horizontal axis represents the pvalue, the vertical axis represents the standard enrichment fraction (NES), the size of the circle represents the -log (padj) value, and different colors represent different gene sets.

**10 Statistical analyses**

All experiments were performed not less than twice. Data were analyzed with GraphPad Prism 5 and expressed as mean ± standard error of mean (SEM). The two-tailed Student's t-tests was used to compare the differences between two groups. P<0.05 was considered statistically significant. *, P < 0.05; **, P < 0.01; ***, P < 0.001.

**11 Ethics approval and consent to participate**

Humans: This study was approved by the Ethics Committee of the First Affiliated Hospital, College of Medicine, Zhejiang University (IIT20220603A). Written informed consents was obtained from all participants before enrollment. All experimental protocols and procedures were carried out following the ethical standards.

Animals: All animal experiments were reviewed and approved by the Institutional Animal Care and Use Committee of the First Affiliated Hospital, College of Medicine, Zhejiang University (ZJCLA-IACUC-20120020). All animal experiments were performed following animal use guidelines and ethical approval.

**Supplemental figures**

**
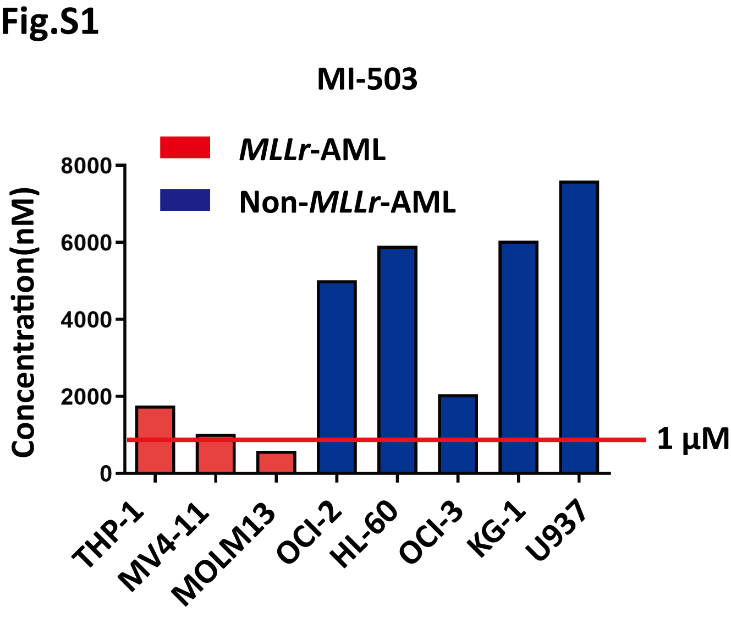
**

**Figure S1. *KMT2Ar*-AML was sensitive to MI-503.** The IC50 of MI-503 in AML cell lines (72 hours).

**
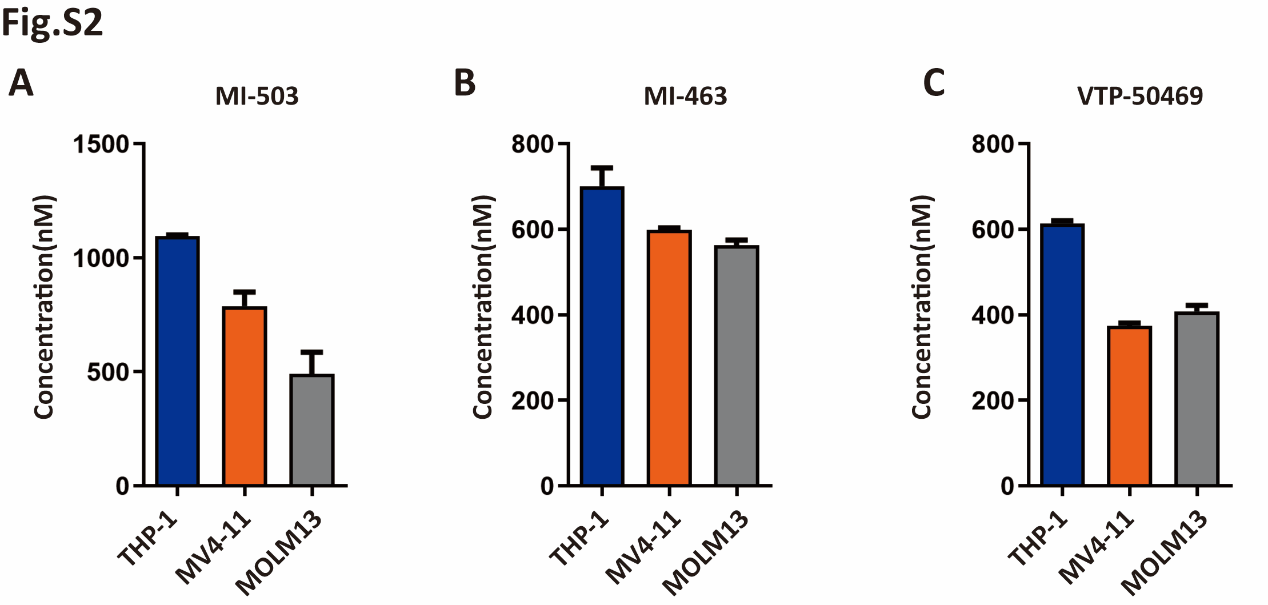
**

**Figure S2. MEN1i inhibited the proliferation of *KMT2Ar*-AML cell lines.** (**A-C**) IC50 of MENis, such as MI-503 (**A**), MI-403 (**B**), and VTP50469 (**C**), in THP-1, MV4-11, and MOLM13 cells (7 days).


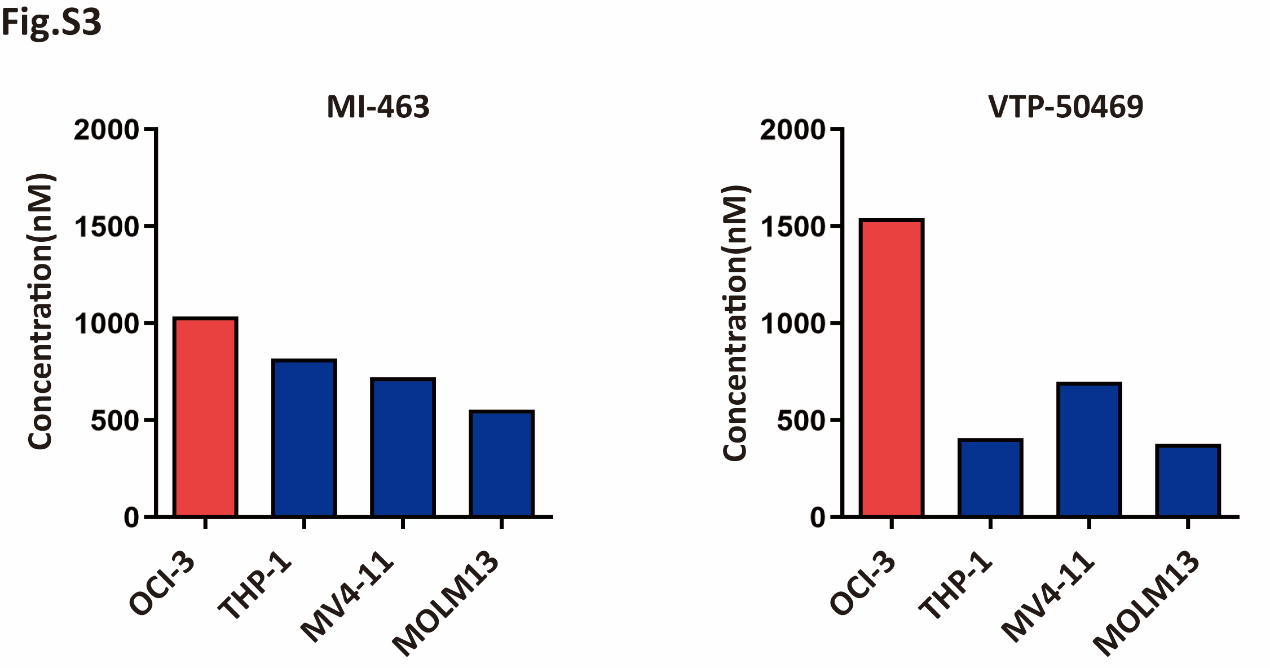


**Figure S3. OCI-AML3 was relatively less sensitive to MEN1i than *KMT2Ar*-AML.** (**A, B**) The IC50 of MI-463 (**A**) and VTP50469 (**B**) in OCI-AML3 and *KMT2Ar*-AML cell lines (72 hours).


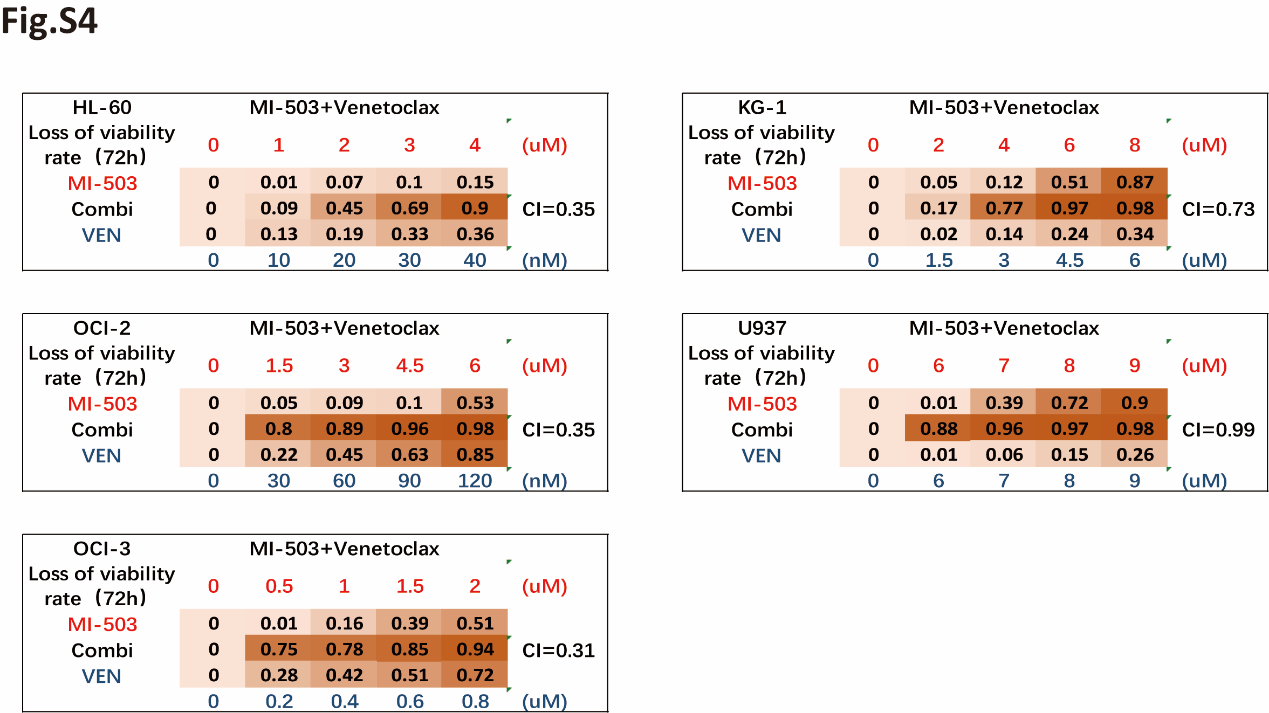


**Figure S4. No significant synergistic effects of VEN plus MEN1i were observed in non-*KMT2Ar*-AML cell lines.** Growth inhibition and synergistic index of VEN plus MI-503 in HL-60, OCI-AML2, OCI-AML3, KG-1, and U937 cells (72 hours).


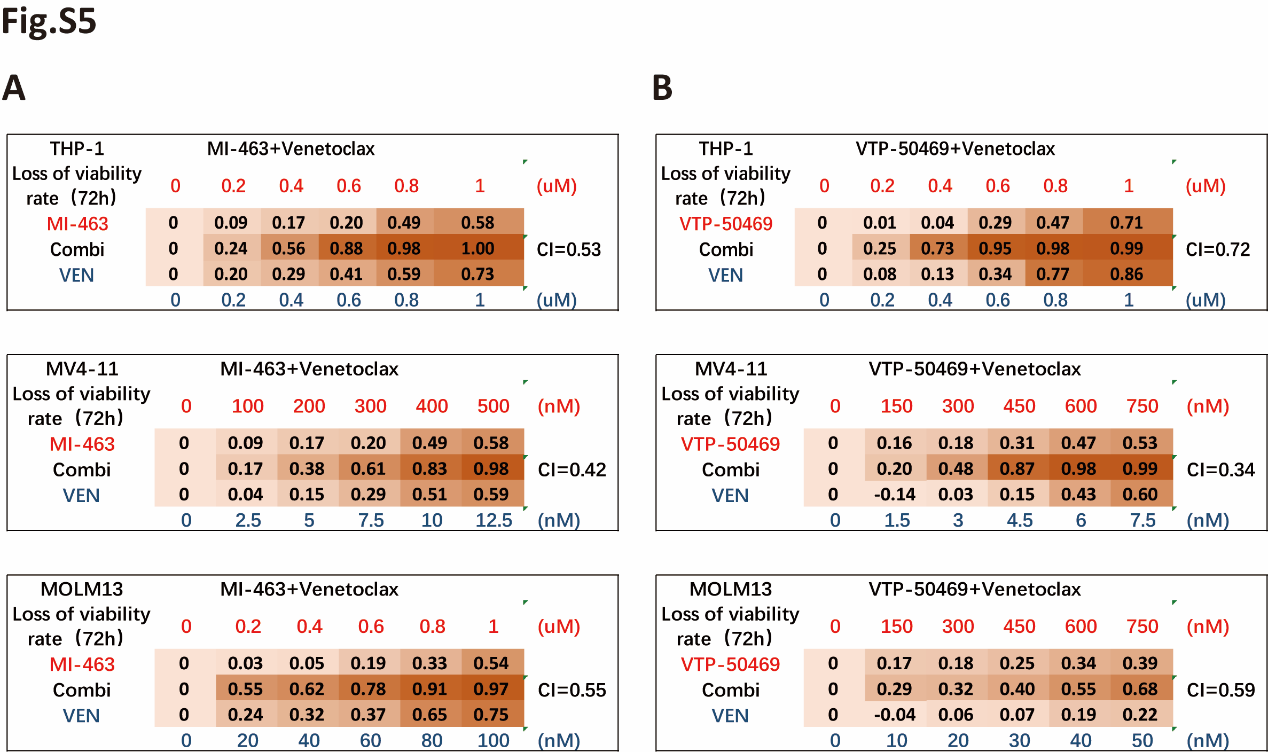


**Figure S5. VEN plus MI-463 or VTP-50469 also cooperated in the inhibition of *KMT2A*r-AML cell lines.** (**A-B**) Growth inhibition and synergistic index of VEN plus MI-403 (A) or VTP-50469 (B) in THP-1, MV4-11, and MOLM13 cells (72 hours).


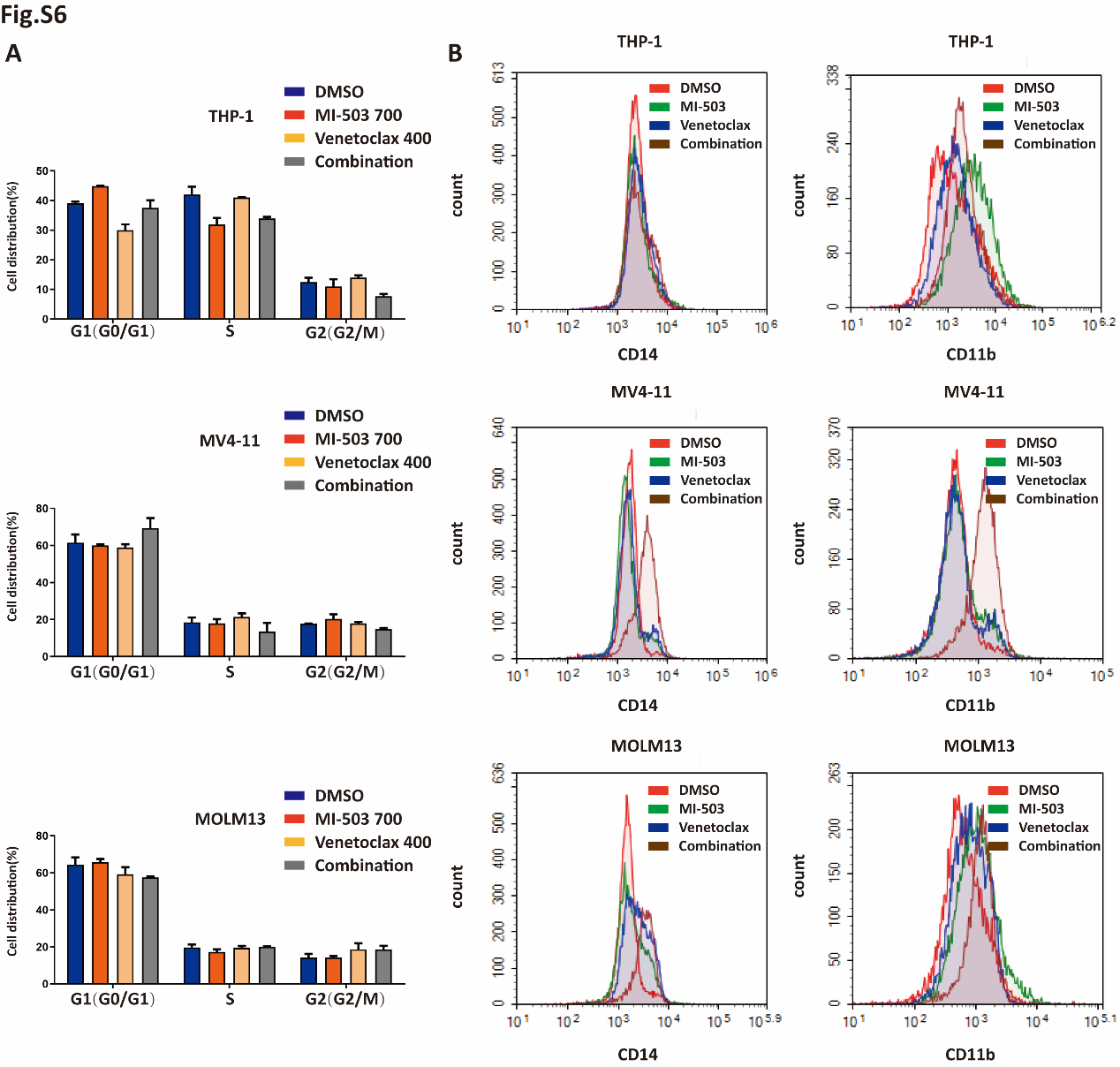


**Figure S6. VEN plus MI-503 did not influence cell cycle distribution and cell differentiation of *KMT2Ar*-AML cell lines.** (**A**) Cell cycle analysis for THP-1, MV4-11, and MOLM13 cells after single-agent or combinatorial treatment with VEN and MI-503 (72 hours); (**B**) Cell differentiation was determined by CD11b and CD14 staining in THP-1, MV4-11, and MOLM13 cells after single-agent or combinatorial treatment with VEN and MI-503 (72 hours).


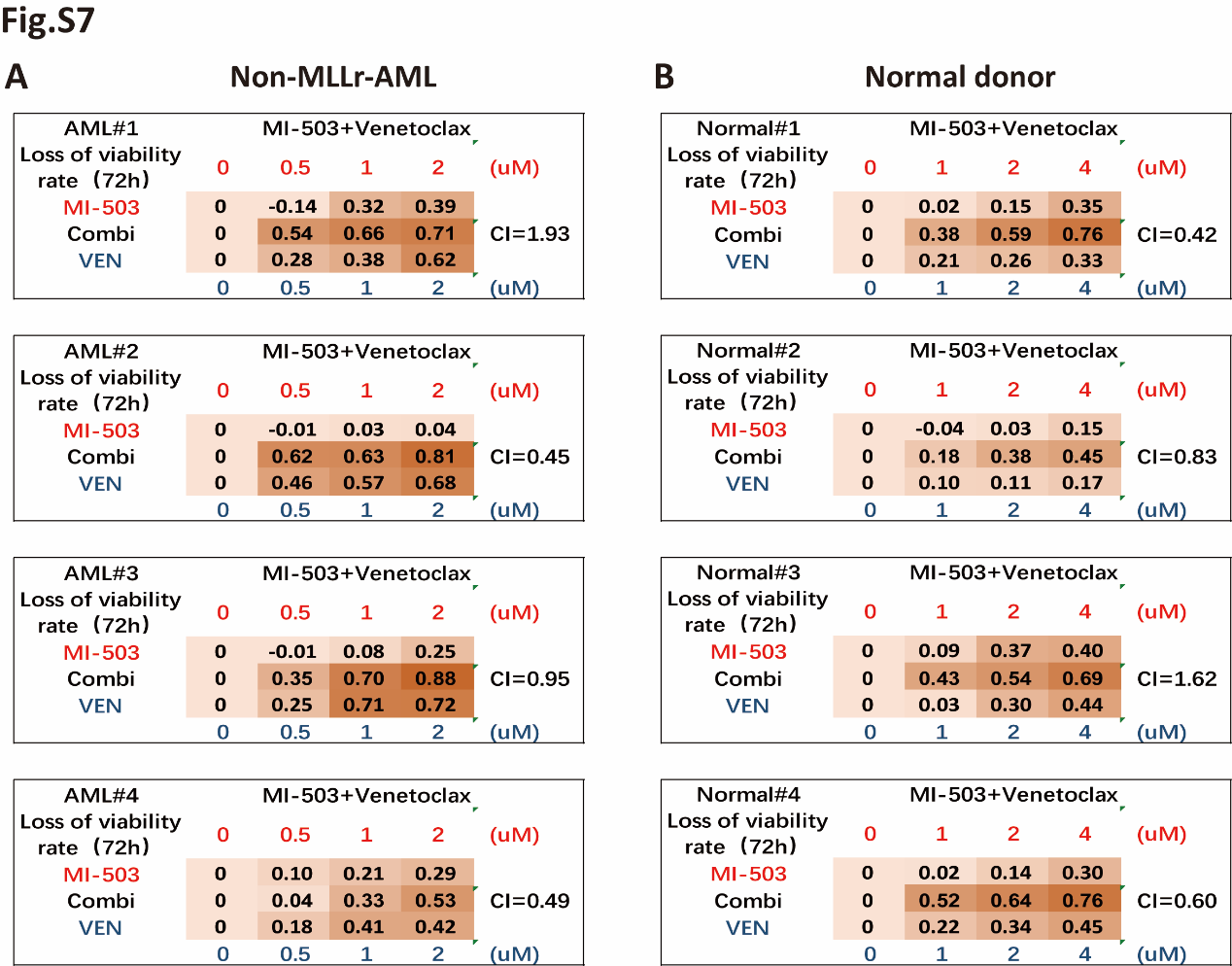


**Figure S7.** **No significant synergistic effects of VEN plus MEN1i were observed in primary bone marrow MNCs from non-*KMT2Ar*-AML patients or healthy donors.** (**A-B**) Growth inhibition and synergistic index of VEN plus MI-503 in primary bone marrow MNCs from non-*KMT2A*r-AML patients (A) or healthy donors (B) (72 hours).


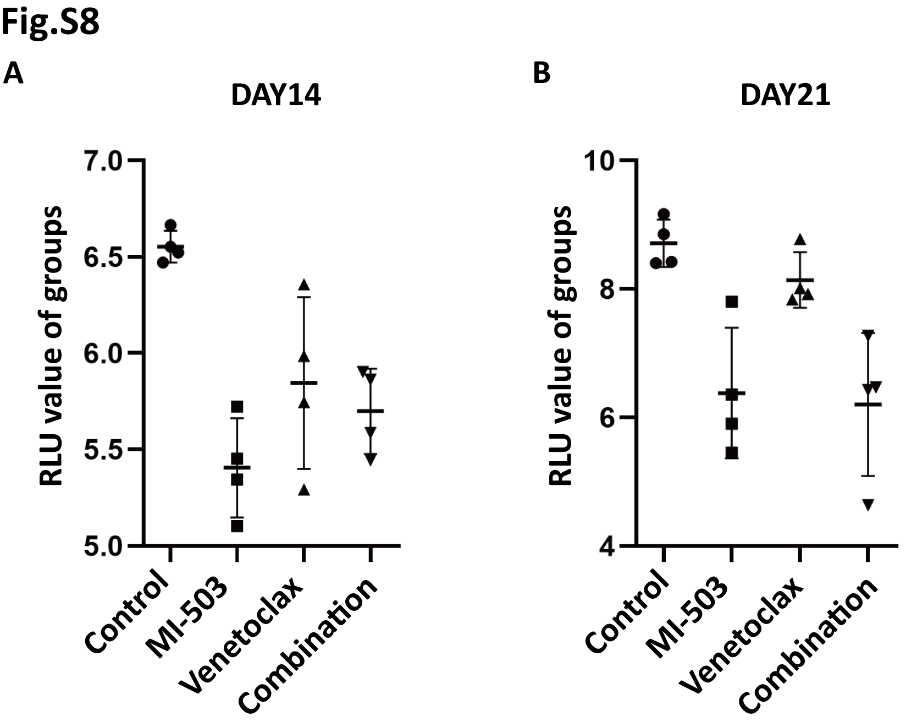


**Figure S8. Leukemic burden in treated MOLM13 xenotransplantation model.** The relative luminescence unit (RLU) value was calculated for indicating leukemic burdens of treated mice (14 days[**A**] and 21 days[**B**]).


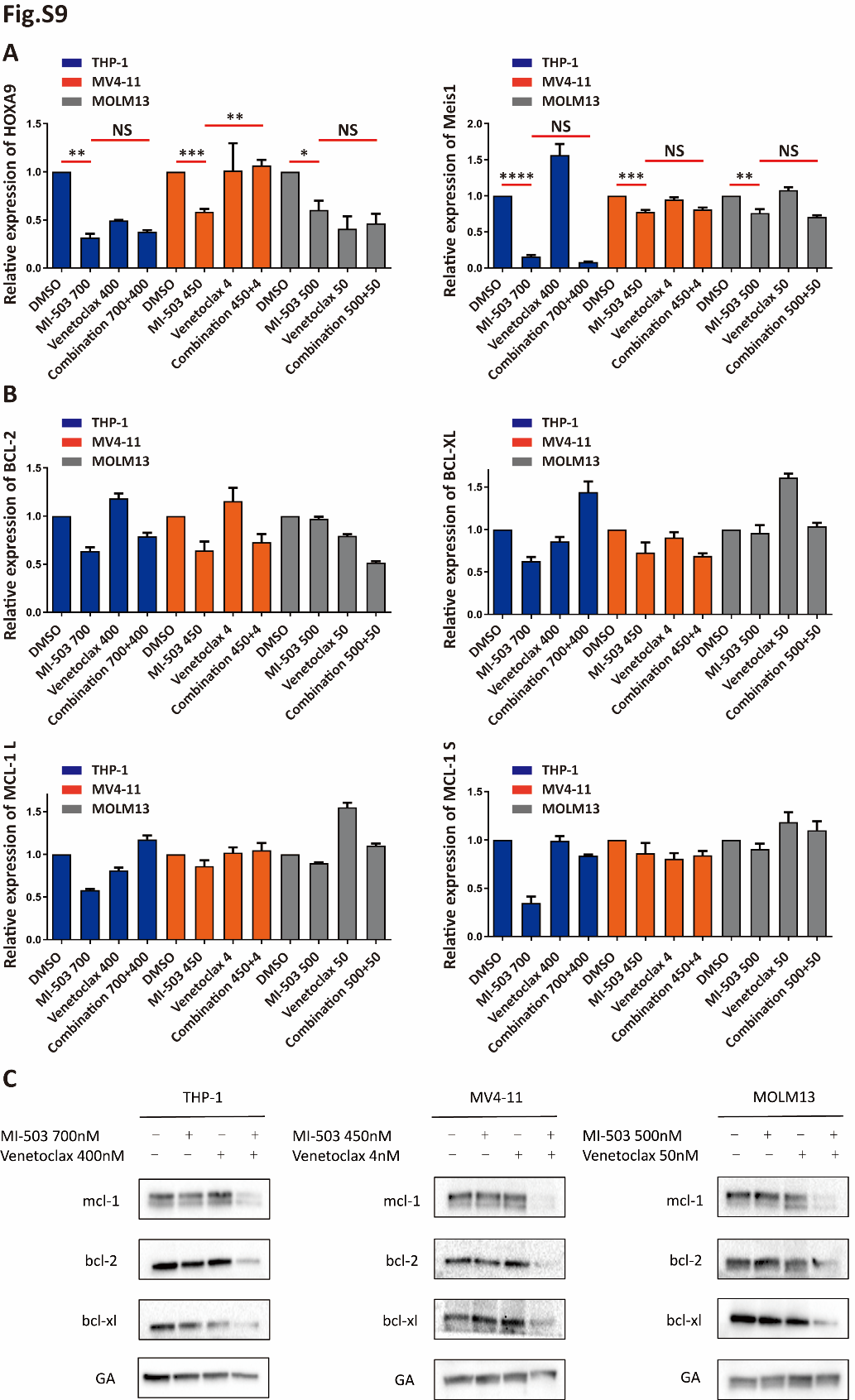


**Figure S9. The synergistic mechanism of VEN plus MI-503 was not related to further downregulation of *HOXA9* and *MEIS1* or disruption of BCL2/BCL-XL balance.** (**A**) The mRNA expression of *HOXA9* and *MEIS1* in THP-1, MV4-11, and MOLM13 after treatments with DMSO, VEN, MI-503 and their combination (72 hours) [two-tailed Student's t-tests; *P < 0.05, **P < 0.01, ***P < 0.001 ****P < 0.0001]; (**B-C**) The mRNA (**B**) and protein (**C**) expression of BCL2, BCL-XL and MCL-1 in THP-1, MV4-11, and MOLM13 after treatments with DMSO, VEN, MI-503 and their combination (72 hours).


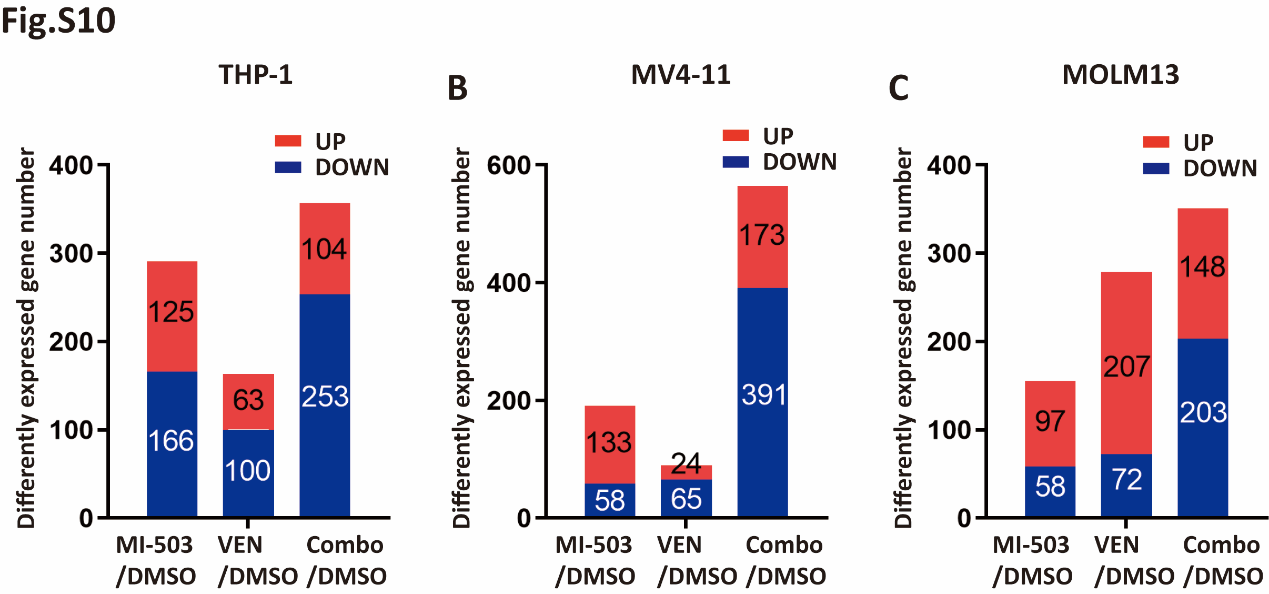


**Figure S10. Global transcriptional regulation of VEN plus MI-503 in *KMT2Ar*-AML cell lines.** DEGs in three *KMT2Ar*-AML cell lines.


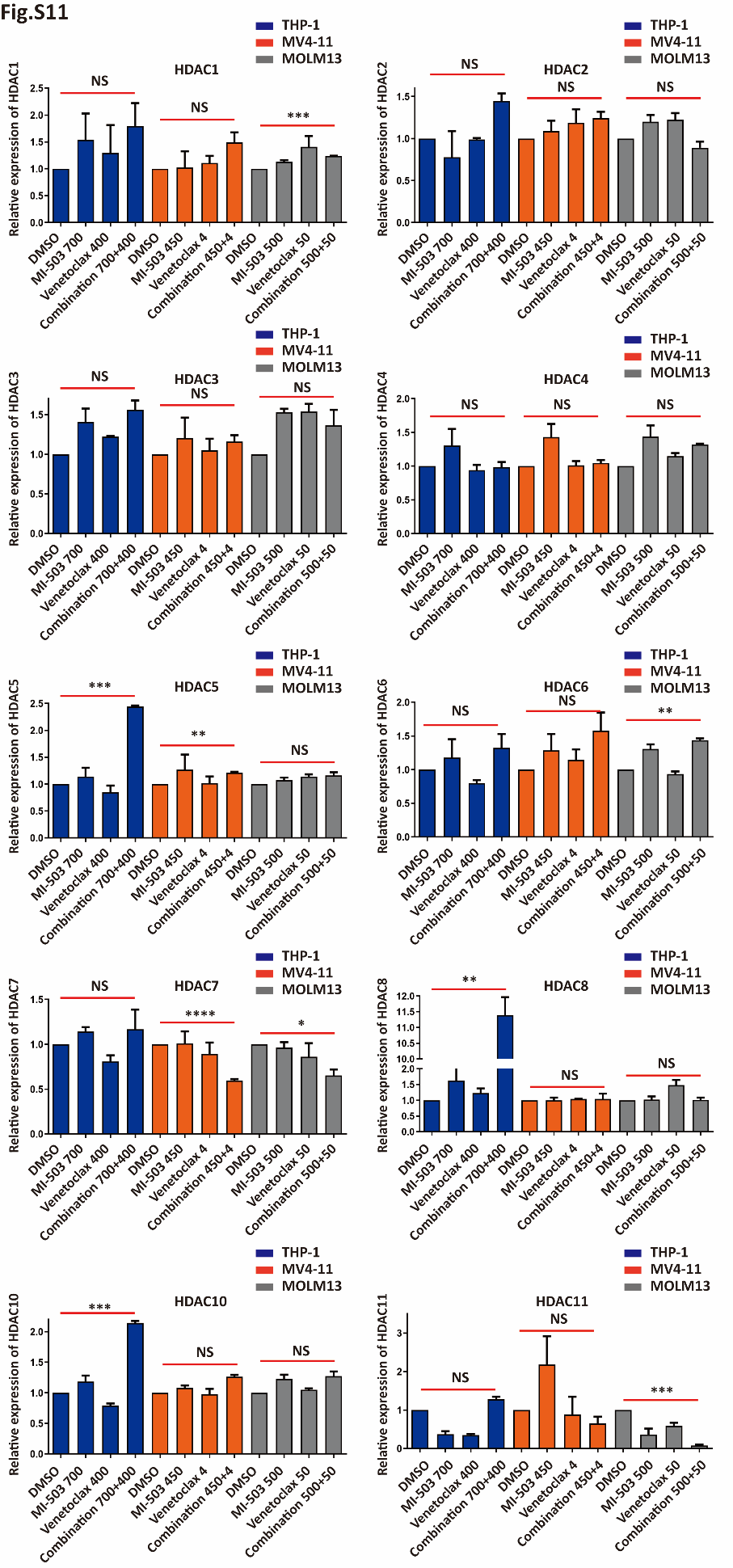


**Figure S11. VEN plus MI-503 specifically downregulated *HDAC9*.** The mRNA expression of HDAC family in THP-1, MV4-11, and MOLM13 after treatments with DMSO, VEN, MI-503 and their combination (72 hours).


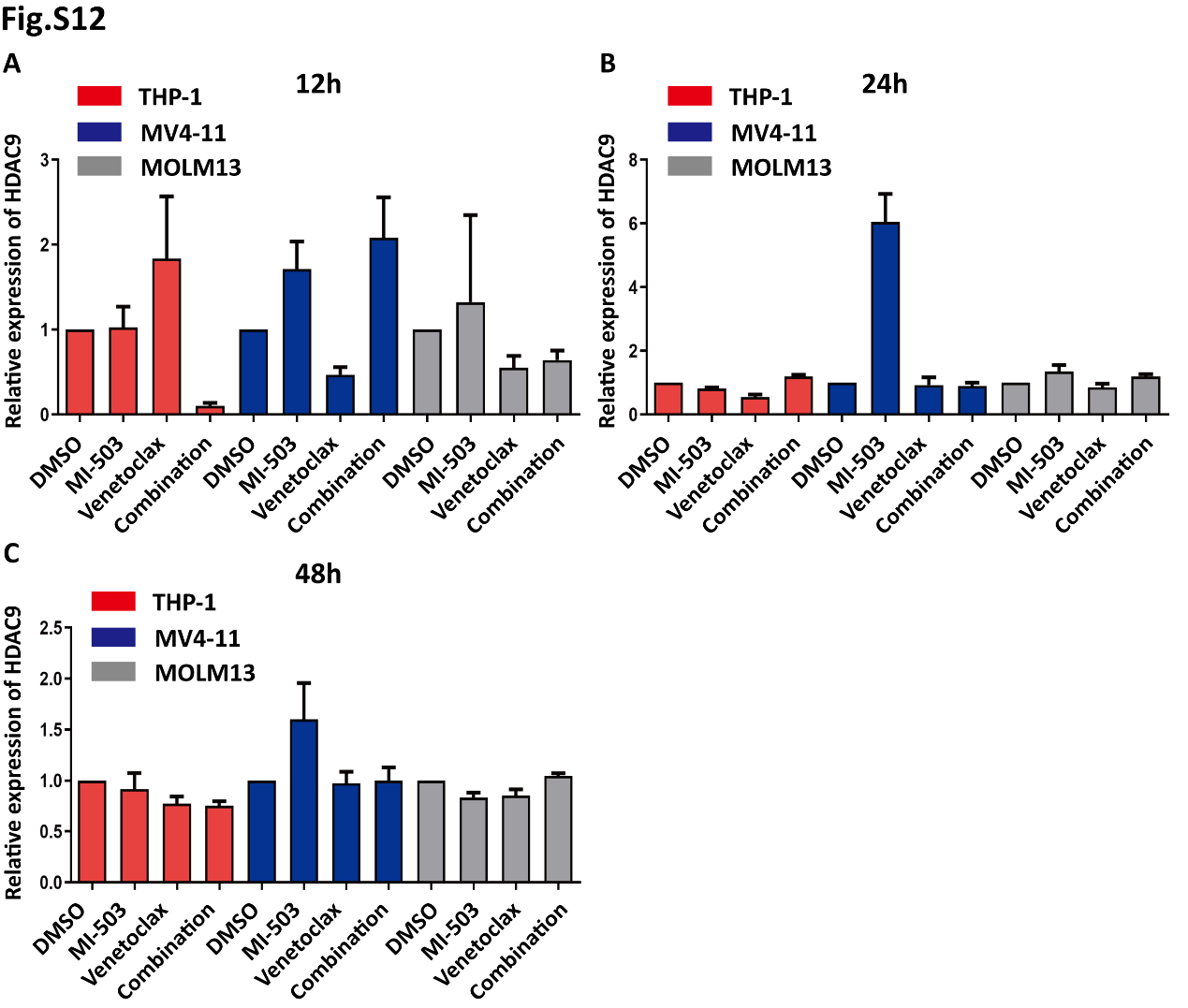


**Figure S12. *HDAC9* was not consistently down-regulated by VEN plus MI-503 until 72 treatments.** (**A-C**) *HDAC9* mRNA was detected under treatments for 12 hours (**A**), 24 hours (**B**), and 48 hours (**C**), respectively.

**
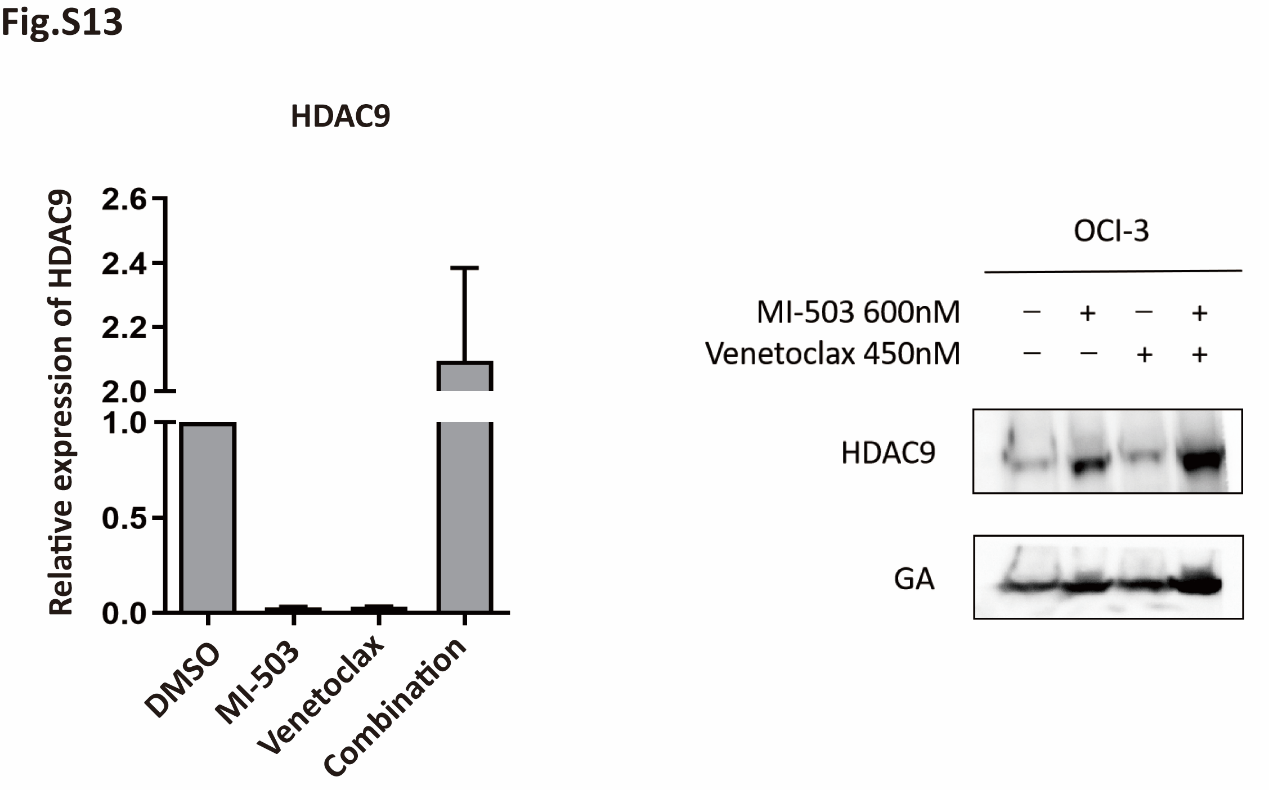
**

**Figure S13. HDAC9 was not repressed by VEN plus MI-503 in OCI-AML3.** (**A, B**) OCI-AML3 was treated by DMSO, VEN, MI-503, or VEN plus MI-503 at 72 hours, and *HDAC9* mRNA (**A**) and protein (**B**) were detected.


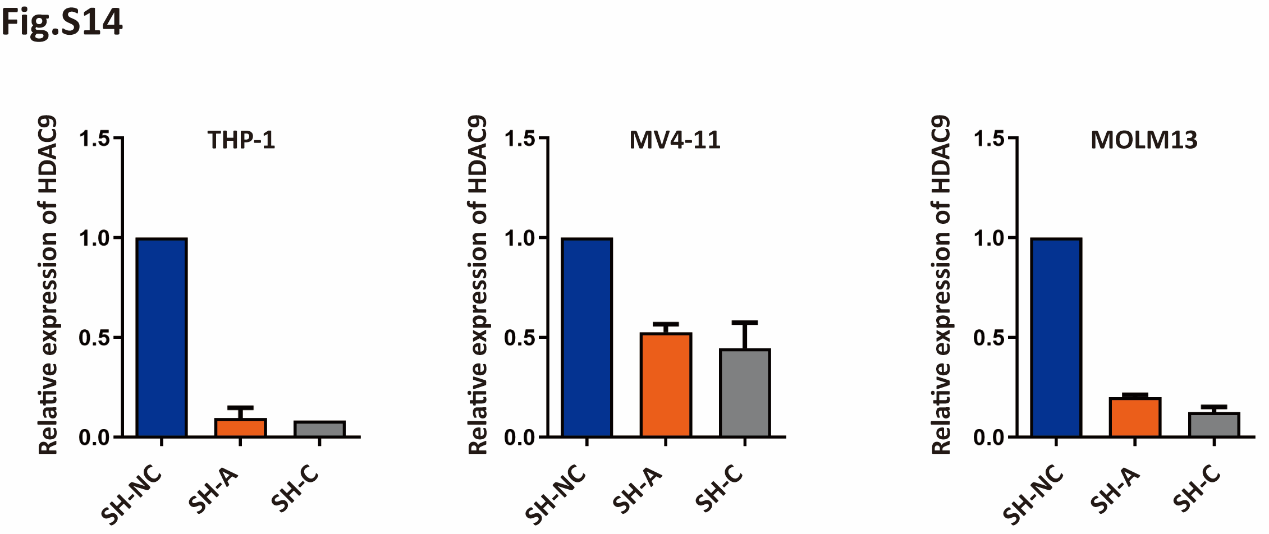


**Figure S14. *HDAC9* knockdown was displayed in *KMT2Ar*-AML cell lines.** *HDAC9* was detected in *KMT2Ar*-AML cell lines after shRNA-mediated knockdown.


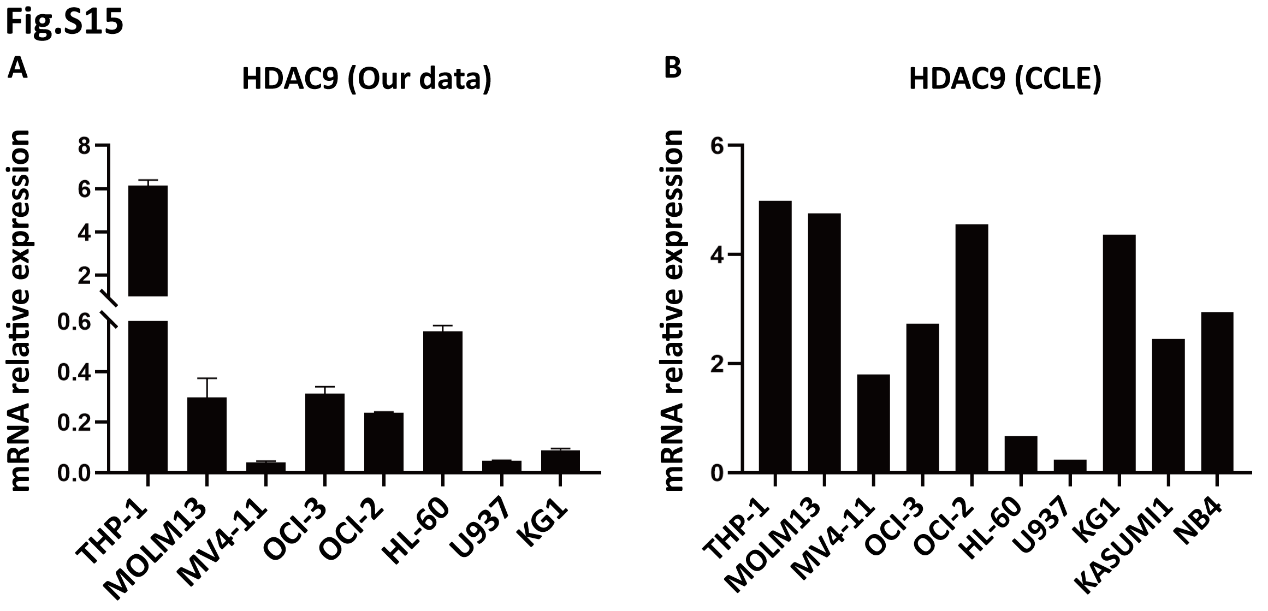


**Figure 15. *HDAC9* expression in AML cell lines.** (**A-B**) *HDAC9* expression was determined in our AML cell lines (A) and obtained from Cancer Cell Line Encyclopedia (CCLE) database (**B**).


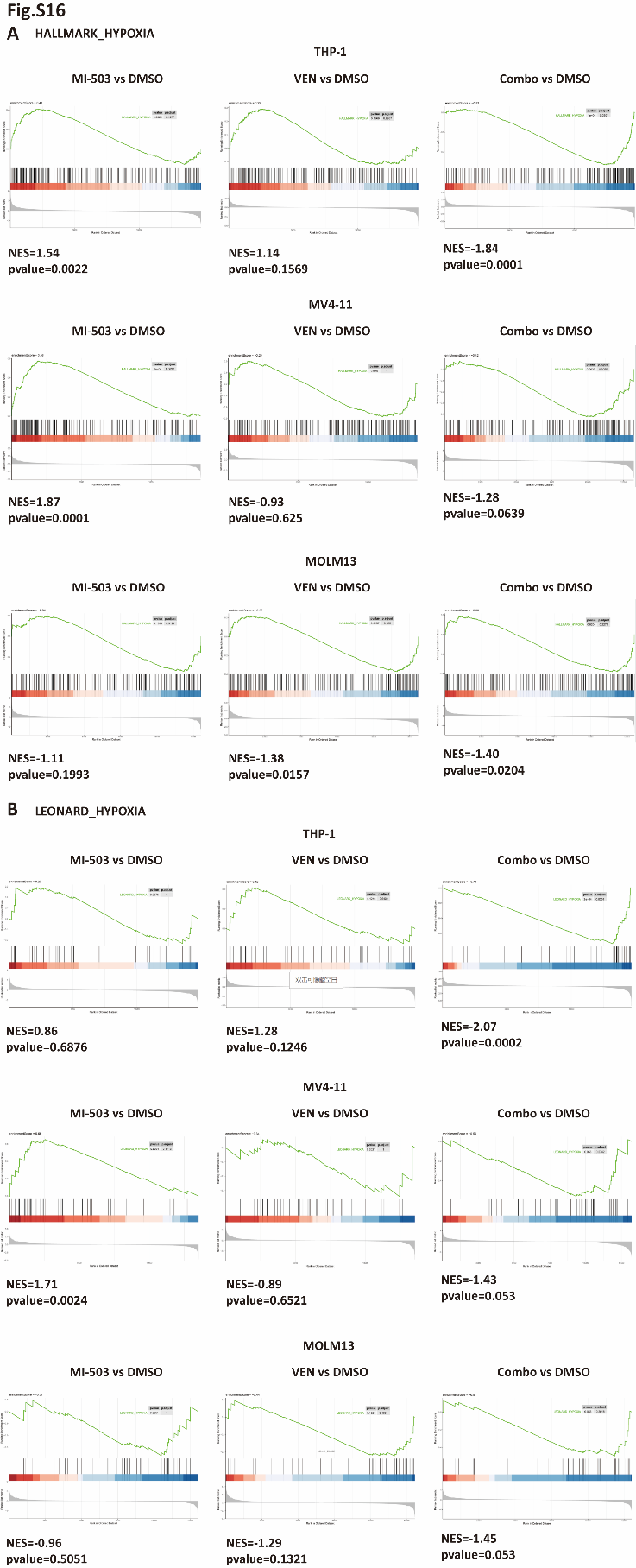


**Figure S16. VEN plus MI-503 inhibited the expression of hypoxia pathway.** (**A-B**) HALLMARK_HYPOXIA (**A**) and LEONARD_HYPOXIA (**B**) gene sets for MI-503, VEN, or VEN plus MI-503 compared to DMSO, respectively.


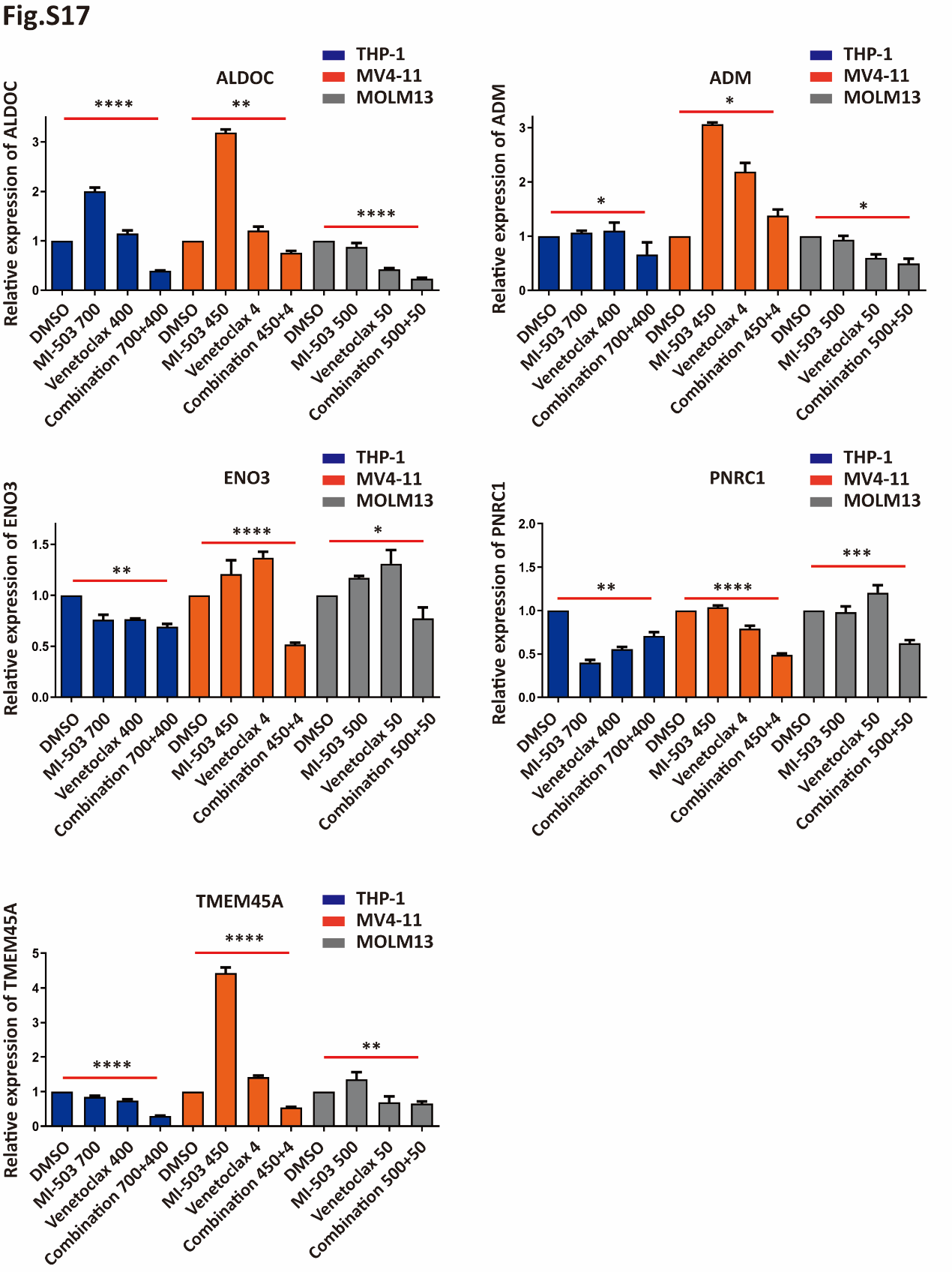


**Figure S17. VEN plus MI-503 repressed the targets of HIF-1A.** The mRNA expression of *ALDOC*, *ADM*, *ENO3*, *PNRC1* and *TMEM45A* in THP-1, MV4-11, and MOLM13 after treatments with DMSO, VEN, MI-503 and their combination (72 hours) [two-tailed Student's t-tests; *P < 0.05, **P < 0.01, ***P < 0.001 ****P < 0.0001].

**
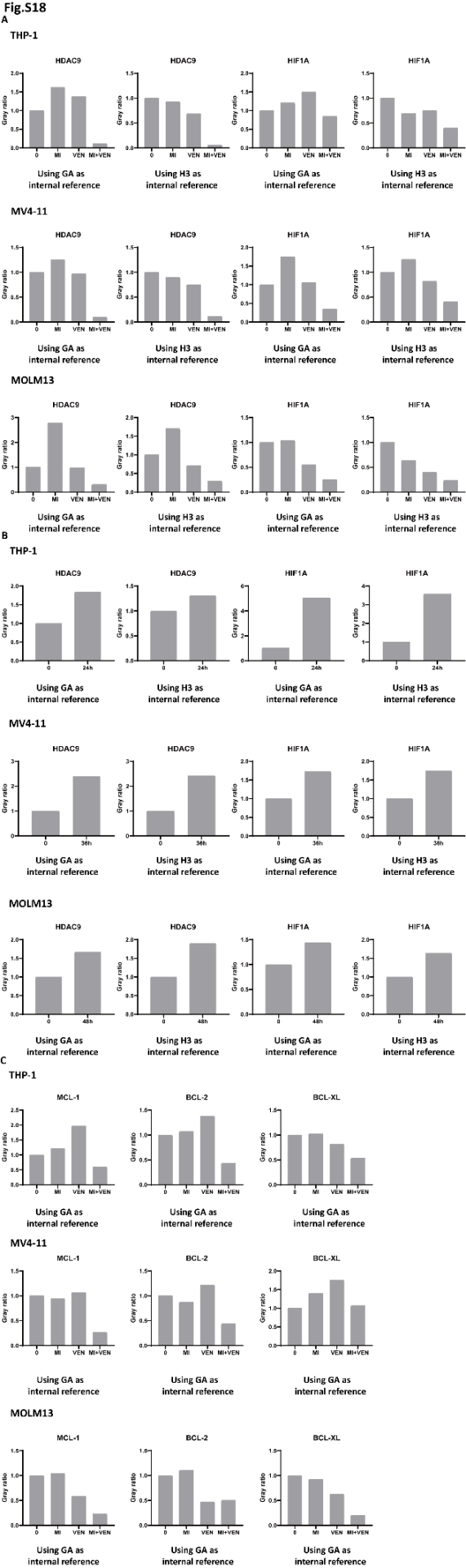
**

**Figure S18. The band quantification for western blot.** (**A-C**) The band was quantified for Figure 2C (**A**), Figure 2H (**B**), and Figure S9C (**C**), respectively.
