# Supplementary material for: Repressing HIF-1α-induced HDAC9 contributes to the synergistic effect of venetoclax and MENIN inhibitor in KMT2Ar AML
Source: Biomark Res. 2023 Dec 5;11:105. doi: 10.1186/s40364-023-00547-9 (PMC10696732; doi:10.1186/s40364-023-00547-9)
Supplement: Supplementary file 2 — Additional file 2: Table S1. The clinical and genetic features of primary sample donors. [file 40364_2023_547_MOESM2_ESM.docx]

**Table S1. The clinical and genetic features of primary sample donors.**

| **No.** | **Gender** | **Age** | **Primary/**  **Relapsed** | **FAB** | **BM blast** | **Karyotype** | ***KMT2A* rearrangement** | **Molecular aberrations** |
| --- | --- | --- | --- | --- | --- | --- | --- | --- |
| *KMT2Ar*-AML #1 | Male | 55 | Primary | M4 | 31% | 46,XY,del(11)(q23)(6)/46,XY[4] | *KMT2A-MLLT4* | / |
| *KMT2Ar*-AML #2 | Male | 70 | Relapsed | M4 | 80% | NA | *KMT2A-MLLT3* | *CSF3R, PML, U2AF1* mutation |
| *KMT2Ar*-AML #3 | Female | 85 | Primary | M1 | 71% | 46,XY[20] | *KMT2A-MLLT3* | / |
| *KMT2Ar*-AML #4 | Male | 21 | Primary | M5 | 51% | 46,XY,t(7;9)(q22;p24)(6)/46,XY[4] | *KMT2A-MLLT3* | *CDKN2A, KRAS* mutation |
| Non-*KMT2Ar*-AML #1 | Female | 53 | Primary | M2 | 58% | 46,XY[20] | Negative | *DNMT3A, SETBP1* mutation |
| Non-*KMT2Ar*-AML #2 | Female | 57 | Primary | M4 | 65% | NA | Negative | *CBFβ-MYH11;*  *WT1, NF1* mutation |
| Non-*KMT2Ar*-AML #3 | Male | 69 | Primary | M5 | 40% | NA | Negative | *CBFβ-MYH11;*  *NF1, FLT3* mutation |
| Non-*KMT2Ar*-AML #4 | Female | 72 | Primary | M3 | 84% | 46,XX,t(15;17)(q24;q21)[10] | Negative | *PML-RARA* |
| Healthy donor #1 | Female | 72 | / | / | / | / | / | / |
| Healthy donor #2 | Female | 68 | / | / | / | / | / | / |
| Healthy donor #3 | Male | 63 | / | / | / | / | / | / |
| Healthy donor #4 | Male | 48 | / | / | / | / | / | / |
