# Supplementary material for: Repressing HIF-1α-induced HDAC9 contributes to the synergistic effect of venetoclax and MENIN inhibitor in KMT2Ar AML
Source: Biomark Res. 2023 Dec 5;11:105. doi: 10.1186/s40364-023-00547-9 (PMC10696732; doi:10.1186/s40364-023-00547-9)
Supplement: Supplementary file 3 — Additional file 3: Table S2. Different expressed genes of MI-503 vs. DMSO in MV4-11. [file 40364_2023_547_MOESM3_ESM.pdf]

gene_id	BaseMean	BaseMean_	BaseMean_	FoldChang	log2FoldCl	pValue	qValue	Regulation	Expression	Expression_MV4_11_MI_503
A2M	36.2195	52.08937	20.34964	0.390668	-1.35599	0.034082	1	Down	0.526571	0.205997
ABCD2	45.3534	24.57046	66.13634	2.691702	1.428519	0.016823	0.719445	Up	0.07162	0.193045
ADGRD1	88.02443	57.00346	119.0454	2.088389	1.062391	0.027286	0.934608	Up	0.317687	0.664367
ADGRE1	19.68491	8.845364	30.52447	3.4509	1.786973	0.024441	0.878691	Up	0.137581	0.475433
ADGRG3	15.64965	6.879727	24.41957	3.549497	1.827615	0.035433	1	Up	0.053367	0.189688
AFF3	477.7863	284.0345	671.5382	2.364284	1.241404	1.49E-06	0.000478	Up	0.780592	1.848084
AIG1	25.75514	10.811	40.69929	3.764618	1.912503	0.008681	0.484352	Up	0.065023	0.245124
AK4	256.2566	123.8351	388.6782	3.138676	1.650156	3.84E-07	0.000139	Up	0.895523	2.814626
ALDH1A3	157.8922	278.1376	37.64684	0.135353	-2.8852	1.43E-11	1.77E-08	Down	4.22065	0.572066
ALDOC	879.1219	489.4435	1268.8	2.592333	1.374251	1.14E-09	9.91E-07	Up	15.42727	40.04767
ANK1	9.105344	2.948455	15.26223	5.17635	2.371935	0.035873	1	Up	0.016821	0.08719
APLN	22.78936	5.896909	39.6818	6.729255	2.750447	0.000675	0.086148	Up	0.100142	0.674808
APOL4	61.06727	85.50518	36.62936	0.428388	-1.22301	0.024483	0.878691	Down	1.352624	0.580245
ARL4D	66.49521	37.34709	95.64332	2.560931	1.356668	0.010577	0.537836	Up	1.260643	3.232865
ASF1A	1927.91	2579.898	1275.923	0.494563	-1.01577	6.38E-07	0.00022	Down	56.29737	27.88094
ASMTL	331.8225	166.0963	497.5488	2.995544	1.582818	7.39E-08	3.77E-05	Up	3.460356	10.37992
B3GNT7	27.22937	13.75945	40.69929	2.957914	1.56458	0.026124	0.911697	Up	0.202237	0.599024
BASP1	283.2199	123.8351	442.6047	3.574146	1.837599	5.72E-09	3.68E-06	Up	3.453899	12.36174
BNIP3	940.4726	587.7253	1293.22	2.200381	1.137754	2.84E-07	0.000117	Up	19.70491	43.41802
BPI	289.3187	95.33337	483.304	5.069621	2.341878	3.33E-13	5.47E-10	Up	2.729878	13.8585
C2orf48	82.02353	51.10655	112.9405	2.209903	1.143983	0.02044	0.804837	Up	1.471134	3.25554
C3orf80	462.5281	629.9865	295.0698	0.468375	-1.09426	2.47E-05	0.005719	Down	13.37934	6.275173
C4A	9.631417	1.965636	17.2972	8.799795	3.13747	0.008102	0.469409	Up	0.019834	0.174775
C6orf223	1835.743	1200.021	2471.464	2.059517	1.042306	3.58E-07	0.000136	Up	10.30288	21.24818
CCR2	394.3284	170.0275	618.6292	3.638405	1.863306	3.12E-11	3.55E-08	Up	1.783002	6.496216
CD180	124.1002	174.9416	73.25872	0.418761	-1.2558	0.003369	0.263613	Down	3.333833	1.398001
CD300A	574.494	339.0723	809.9158	2.388623	1.256179	3.18E-07	0.000127	Up	8.067926	19.29777
CDH3	156.3986	103.1959	209.6013	2.031101	1.022262	0.00849	0.48132	Up	1.280309	2.604017
CEACAM4	46.42288	21.622	71.22375	3.294041	1.719858	0.0042	0.30025	Up	0.32195	1.061977
CFH	35.04603	60.93473	9.15734	0.150281	-2.73426	0.000104	0.019556	Down	0.758916	0.114208
CIITA	9.614085	2.948455	16.27971	5.52144	2.465045	0.026599	0.923906	Up	0.015255	0.084346
COL18A1	25.19441	13.75945	36.62936	2.662123	1.412577	0.049115	1	Up	0.110654	0.294981
CORO2A	43.87917	21.622	66.13634	3.058752	1.612943	0.00798	0.467877	Up	0.190876	0.584645
CPZ	34.72794	50.12373	19.33216	0.385689	-1.37449	0.034088	1	Down	1.080428	0.417283
CR1	69.36822	18.67355	120.0629	6.429571	2.684723	2.02E-06	0.000609	Up	0.100956	0.649998
CSF2RA	96.67303	57.00346	136.3426	2.391831	1.258115	0.007248	0.441144	Up	1.047652	2.509256
CXCL9	6.388318	12.77664	0	0	#NAME?	0.002691	0.230767	Down	0.258318	0
CYP26B1	33.2996	15.72509	50.87411	3.235219	1.693863	0.010705	0.540616	Up	0.180493	0.584739
DBNDD1	116.8667	65.84882	167.8846	2.549545	1.35024	0.002093	0.199769	Up	1.268111	3.237559
DBP	101.5871	66.83164	136.3426	2.040091	1.028633	0.024661	0.880748	Up	2.020458	4.127594
DHRS3	58.61533	22.60482	94.62584	4.186092	2.065604	0.000295	0.04572	Up	0.553003	2.318107
DLX5	373.5087	514.0139	233.0034	0.453302	-1.14146	4.06E-05	0.009099	Down	8.90019	4.040027
DOC2A	25.78981	8.845364	42.73425	4.83126	2.272399	0.002318	0.210462	Up	0.110846	0.536263
EGR3	504.4784	328.2613	680.6956	2.07364	1.052165	3.12E-05	0.007105	Up	3.537873	7.346374
ENO2	248.9661	75.677	422.2551	5.579702	2.480188	5.50E-13	8.13E-10	Up	1.710003	9.554441
EPSTI1	66.73886	110.0756	23.40209	0.2126	-2.23379	5.82E-05	0.011487	Down	0.813975	0.173289
ERV3-1	2285.174	3091.946	1478.402	0.478146	-1.06448	1.39E-07	6.64E-05	Down	51.83248	24.81761
F2R	51.88037	29.48455	74.2762	2.519157	1.332941	0.019799	0.793962	Up	0.40951	1.033039
FADS1	2897.077	1915.513	3878.642	2.024858	1.017821	3.52E-07	0.000136	Up	22.81376	46.25823
FADS2	4671.293	2767.616	6574.97	2.37568	1.24834	2.83E-10	2.72E-07	Up	39.33742	93.58177
FAM114A1	42.33562	22.60482	62.06641	2.745716	1.457182	0.017032	0.726282	Up	0.186024	0.511473
FAM156A	104.7894	29.48455	180.0943	6.108093	2.610722	9.35E-08	4.61E-05	Up	0.324872	1.987078
FAM171A2	63.49476	34.39864	92.59088	2.691702	1.428519	0.008121	0.469409	Up	0.568811	1.533179
FCGR2B	1192.525	574.9487	1810.101	3.148282	1.654565	2.98E-14	7.36E-11	Up	9.343601	29.4568
FGR	109.1316	71.74573	146.5174	2.042176	1.030107	0.020955	0.818112	Up	1.20716	2.468627
FLT1	642.9834	349.8833	936.0836	2.675417	1.419764	3.59E-09	2.80E-06	Up	1.586829	4.251275
GDF15	52.11792	73.71137	30.52447	0.414108	-1.27192	0.02602	0.910225	Down	2.346347	0.972979
GOLGA8K	39.18529	57.00346	21.36713	0.374839	-1.41566	0.0237	0.871597	Down	0.601572	0.225803
GPA33	78.67033	39.31273	118.0279	3.002283	1.58606	0.001785	0.174939	Up	0.75022	2.255475
GPLD1	5.422832	9.828182	1.017482	0.103527	-3.27192	0.040377	1	Down	0.067574	0.007005
GPNMB	30.73856	16.70791	44.76922	2.679522	1.421976	0.034638	1	Up	0.319735	0.857917
GPR141	52.89785	29.48455	76.31116	2.588175	1.371935	0.015979	0.693766	Up	0.138506	0.358971
GRIN3A	12.6492	3.931273	21.36713	5.435167	2.442324	0.013384	0.620845	Up	0.019328	0.105196
GTPBP6	181.0843	116.9554	245.2132	2.096639	1.068079	0.003585	0.27068	Up	1.761578	3.69848
H1F0	40.84406	20.63918	61.04893	2.957914	1.56458	0.011614	0.565314	Up	0.484979	1.436501
H3F3C	17.66728	7.862546	27.47202	3.494036	1.804895	0.029185	0.974831	Up	0.407263	1.424949
HAL	108.5994	44.22682	172.972	3.91102	1.967545	2.19E-05	0.005311	Up	0.543165	2.127255
HILPDA	404.2208	243.7389	564.7026	2.316834	1.212155	7.85E-06	0.002152	Up	9.450971	21.92648
HIST1H2B(	21.79532	33.41582	10.17482	0.304491	-1.71553	0.025123	0.891873	Down	4.176998	1.27361
HUNK	81.09271	46.19246	115.993	2.51108	1.328308	0.007634	0.455508	Up	0.342458	0.861123
IER5L	71.93537	46.19246	97.67829	2.114594	1.080381	0.035577	1	Up	0.933229	1.976116
IFI27	6.388318	12.77664	0	0	#NAME?	0.002691	0.230767	Down	0.844836	0
IFI44	72.7051	117.9382	27.47202	0.232936	-2.102	8.88E-05	0.017057	Down	2.45053	0.571602
IFI44L	29.55387	55.03782	4.069929	0.073948	-3.75735	5.20E-06	0.001451	Down	0.508752	0.037673
IFI6	147.4217	208.3575	86.48598	0.415085	-1.26852	0.001551	0.158213	Down	11.01122	4.576883
IFIT1	147.7979	244.7217	50.87411	0.207886	-2.26614	6.94E-08	3.67E-05	Down	2.833879	0.589934
IFIT2	269.0883	405.9039	132.2727	0.325872	-1.61762	3.95E-07	0.000139	Down	6.358612	2.074946
IFIT3	294.6069	458.9761	130.2377	0.283757	-1.81727	5.14E-09	3.46E-06	Down	8.851379	2.5151
IKZF2	36.72825	52.08937	21.36713	0.410201	-1.2856	0.0432	1	Down	0.141942	0.058305
IL18R1	24.73766	10.811	38.66432	3.576387	1.838503	0.012428	0.592159	Up	0.140863	0.504473

IL32	115.1498	76.65982	153.6398	2.004176	1.00301	0.021771	0.832417	Up	2.72012	5.459107
IL3RA	102.5944	182.8042	22.38461	0.122451	-3.02972	2.92E-09	2.40E-06	Down	5.712659	0.700486
INHBA	48.86259	27.51891	70.20627	2.551201	1.351177	0.020428	0.804837	Up	0.185345	0.473503
IRF7	226.0869	305.6565	146.5174	0.479353	-1.06084	0.001593	0.160376	Down	7.827303	3.757209
ISG15	151.0869	202.4606	99.71325	0.492507	-1.02178	0.009543	0.508577	Down	16.61884	8.196168
JAM2	5.070079	0.982818	9.15734	9.31743	3.219932	0.048289	1	Up	0.010518	0.098135
JPH2	335.0605	213.2716	456.8495	2.142102	1.099027	0.000137	0.0239	Up	1.589096	3.408692
KIAA1755	24.31558	5.896909	42.73425	7.24689	2.857362	0.000349	0.052131	Up	0.032533	0.236088
LAMA5	26.28733	38.32991	14.24475	0.371635	-1.42804	0.044386	1	Down	0.181334	0.067483
LOC10012	4.069929	0	8.139857	Inf	Inf	0.018103	0.756708	Up	0	0.168596
LOC10013	11.15764	1.965636	20.34964	10.3527	3.371935	0.003125	0.255451	Up	0.026008	0.269619
LOC10272	3.931273	7.862546	0	0	#NAME?	0.022192	0.839848	Down	0.067941	0
LOC10537	15.84642	24.57046	7.122375	0.289876	-1.78649	0.039245	1	Down	0.64956	0.188551
LOC10798	7.596452	1.965636	13.22727	6.729255	2.750447	0.030141	0.986703	Up	0.061744	0.416061
LRRC69	53.10073	75.677	30.52447	0.403352	-1.30989	0.021291	0.827081	Down	3.089736	1.247967
LRRC70	12.84596	21.622	4.069929	0.188231	-2.40942	0.014354	0.647407	Down	0.526372	0.099216
LSP1	22.89335	0	45.7867	Inf	Inf	4.97E-08	2.83E-05	Up	0	0.714774
LTA	19.23428	34.39864	4.069929	0.118317	-3.07928	0.000562	0.078439	Down	0.669511	0.079323
LTB	140.022	224.0826	55.96152	0.249736	-2.00152	2.18E-06	0.000645	Down	13.72324	3.431909
MAPK10	21.26925	34.39864	8.139857	0.236633	-2.07928	0.008432	0.480672	Down	0.109166	0.025868
MAPT	25.2464	10.811	39.6818	3.670503	1.875978	0.010386	0.532139	Up	0.083039	0.305216
1-Mar	17.71928	4.914091	30.52447	6.21162	2.63497	0.002669	0.230767	Up	0.037264	0.23179
MARCKS	640.6008	283.0516	998.15	3.526388	1.818191	1.06E-13	2.24E-10	Up	3.598967	12.70883
MARCO	22.7027	10.811	34.59439	3.199925	1.678038	0.025482	0.897742	Up	0.286915	0.91937
MEF2C	3751.163	5007.459	2494.866	0.49823	-1.00512	3.93E-07	0.000139	Down	34.98274	17.45345
MFAP4	853.1536	548.4126	1157.895	2.111357	1.078171	1.64E-06	0.000515	Up	15.31144	32.37243
MGAM2	28.29885	10.811	45.7867	4.235195	2.082428	0.003551	0.27068	Up	0.063089	0.267564
MGLL	209.2271	136.6117	281.8426	2.063092	1.044808	0.002529	0.221389	Up	1.266858	2.617243
MICALCL	33.73901	19.65636	47.82166	2.432884	1.282668	0.048871	1	Up	0.178177	0.434082
MMP25	140.8183	92.38491	189.2517	2.048513	1.034577	0.010625	0.538396	Up	0.95562	1.960296
MNDA	368.6374	213.2716	524.0033	2.456977	1.296884	3.76E-06	0.001069	Up	6.611934	16.26774
MOXD1	100.587	65.84882	135.3251	2.055088	1.0392	0.023738	0.871597	Up	1.182822	2.43415
MPO	416.4652	241.7733	591.1571	2.445089	1.289887	1.67E-06	0.000515	Up	4.11731	10.08105
MS4A3	697.8123	385.2647	1010.36	2.622508	1.390947	3.80E-09	2.81E-06	Up	12.49606	32.81613
MST1	77.60085	42.26118	112.9405	2.672441	1.418158	0.005118	0.344325	Up	0.710848	1.902316
MYH7B	3.561188	0	7.122375	Inf	Inf	0.029435	0.978539	Up	0	0.04941
NAV3	948.1791	612.2957	1284.062	2.097128	1.068415	1.33E-06	0.000436	Up	1.698847	3.567606
NCAM2	400.9175	546.4469	255.388	0.467361	-1.09739	5.23E-05	0.010608	Down	1.461345	0.683916
NDRG1	2616.884	1100.756	4133.013	3.754702	1.908699	1.96E-20	9.65E-17	Up	11.45319	43.06254
NLGN2	111.7099	69.78009	153.6398	2.201771	1.138665	0.010234	0.531366	Up	0.801443	1.767024
NOXA1	46.31888	27.51891	65.11886	2.366331	1.242652	0.03534	1	Up	0.638148	1.512149
NR5A2	30.29915	12.77664	47.82166	3.742899	1.904156	0.005874	0.381223	Up	0.084504	0.316726
NUPR1	10.61423	3.931273	17.2972	4.399897	2.13747	0.039731	1	Up	0.236007	1.039836
OAS1	297.8735	475.684	120.0629	0.252401	-1.98621	2.05E-10	2.17E-07	Down	7.954743	2.010546
OAS2	357.5073	584.7768	130.2377	0.222714	-2.16674	2.16E-13	4.00E-10	Down	5.189932	1.157459
OAS3	164.9911	250.6186	79.36361	0.316671	-1.65894	2.03E-05	0.005	Down	2.064616	0.654704
OASL	28.67505	47.17527	10.17482	0.215681	-2.21303	0.002131	0.200847	Down	1.156159	0.249705
OSBPL6	3.931273	7.862546	0	0	#NAME?	0.022192	0.839848	Down	0.04806	0
P3H2	35.79131	18.67355	52.90907	2.83337	1.502519	0.019599	0.792244	Up	0.25083	0.711672
PCDHGA1:	45.66027	64.866	26.45454	0.407834	-1.29395	0.029694	0.982893	Down	0.737883	0.301348
PCDHGC5	65.8947	100.2475	31.54195	0.314641	-1.66822	0.002011	0.194516	Down	1.139181	0.358926
PCGF2	65.05564	32.433	97.67829	3.011694	1.590575	0.003175	0.256056	Up	0.295116	0.890024
PCOLCE	29.247	14.74227	43.75173	2.967774	1.569381	0.022445	0.843314	Up	0.3537	1.051147
PDE4B	22.7027	10.811	34.59439	3.199925	1.678038	0.025482	0.897742	Up	0.096089	0.307899
PDGFRB	7.087711	1.965636	12.20979	6.21162	2.63497	0.042225	1	Up	0.018723	0.11646
PDYN	4.069929	0	8.139857	Inf	Inf	0.018103	0.756708	Up	0	0.094031
PFKFB4	514.4952	164.1306	864.8598	5.269338	2.397622	2.15E-19	6.36E-16	Up	1.48116	7.815478
PNCK	39.96523	12.77664	67.15382	5.255986	2.393961	0.00026	0.04159	Up	0.216571	1.139861
PNPLA1	31.2993	13.75945	48.83914	3.549497	1.827615	0.007304	0.441144	Up	0.123356	0.438454
PPARGC1A	18.15869	8.845364	27.47202	3.10581	1.63497	0.044245	1	Up	0.040543	0.126092
PPP2R3B	31.22997	17.69073	44.76922	2.53066	1.339514	0.044979	1	Up	0.279208	0.707553
PRAME	143.5353	82.55673	204.5139	2.477253	1.308741	0.001232	0.13325	Up	1.223607	3.035357
PRLR	14.17542	3.931273	24.41957	6.21162	2.63497	0.005966	0.38393	Up	0.016521	0.102765
PRR5L	58.86409	37.34709	80.38109	2.152272	1.10586	0.043373	1	Up	0.485231	1.045787
PRRT4	75.51389	45.20964	105.8181	2.34061	1.226885	0.015708	0.693766	Up	0.521762	1.222923
PRTN3	1992.788	1208.866	2776.709	2.296953	1.199721	4.35E-09	3.07E-06	Up	61.85585	142.2756
PSD3	73.23017	30.46736	115.993	3.807122	1.928701	0.000261	0.04159	Up	0.077796	0.296586
RAB26	88.09376	53.07218	123.1153	2.319772	1.213983	0.011938	0.575736	Up	0.923916	2.146225
RASA4	77.14411	39.31273	114.9755	2.924638	1.548258	0.002404	0.21433	Up	0.359089	1.051653
RNASE13	4.422682	8.845364	0	0	#NAME?	0.014169	0.645108	Down	0.30136	0
RPH3AL	13.17527	2.948455	23.40209	7.93707	2.988606	0.003433	0.264573	Up	0.01628	0.12939
S100A3	25.76126	39.31273	12.20979	0.310581	-1.68696	0.019563	0.792244	Down	2.916507	0.907059
4-Sep	10.84566	19.65636	2.034964	0.103527	-3.27192	0.004451	0.313655	Down	0.34154	0.035407
SIGLEC1	67.86034	104.1787	31.54195	0.302768	-1.72372	0.001318	0.139322	Down	0.687289	0.208375
SIGLEC6	130.3774	78.62546	182.1293	2.316417	1.211895	0.003837	0.285299	Up	0.931364	2.160398
SLAMF7	12.35456	20.63918	4.069929	0.197194	-2.34231	0.018544	0.770761	Down	0.350171	0.069147
SLC13A3	27.27015	40.29555	14.24475	0.353507	-1.50019	0.032973	1	Down	0.520943	0.18441
SLC1A3	187.1198	120.8866	253.3531	2.09579	1.067494	0.003184	0.256056	Up	1.309313	2.747823
SLC22A23	47.15183	66.83164	27.47202	0.411063	-1.28257	0.029494	0.978539	Down	0.378353	0.155741
SLC25A6	1744.793	674.2133	2815.373	4.17579	2.062049	3.06E-22	2.27E-18	Up	24.87423	104.0126
SLC2A5	375.0604	224.0826	526.0383	2.34752	1.231137	9.63E-06	0.002545	Up	4.043698	9.505732

SLC6A8	1171.578	406.8867	1936.269	4.758741	2.25058	9.04E-24	1.34E-19	Up	6.09665	29.05232
SLCO4C1	345.2812	181.8214	508.7411	2.798027	1.48441	2.76E-07	0.000117	Up	1.798838	5.040127
SLX1A	97.70886	142.5086	52.90907	0.371269	-1.42946	0.002371	0.213944	Down	6.668706	2.479294
SLX1B	91.6835	22.60482	160.7622	7.111855	2.830226	5.58E-08	3.06E-05	Up	1.057795	7.53324
SMARCD3	4.069929	0	8.139857	Inf	Inf	0.018103	0.756708	Up	0	0.179082
SMN1	523.8381	701.7322	345.9439	0.492986	-1.02038	4.51E-05	0.009726	Down	20.81258	10.27443
SORL1	1253.298	763.6497	1742.947	2.282391	1.190546	2.34E-08	1.45E-05	Up	3.227328	7.376164
SORT1	121.2037	166.0963	76.31116	0.459439	-1.12205	0.009152	0.497479	Down	1.13361	0.521542
SPATA25	14.6495	5.896909	23.40209	3.968535	1.988606	0.02719	0.933488	Up	0.104248	0.414283
SPP1	106.229	34.39864	178.0594	5.17635	2.371935	7.25E-07	0.000244	Up	1.038222	5.381599
STC1	17.27986	0.982818	33.57691	34.16391	5.094401	1.41E-05	0.003589	Up	0.013879	0.47482
STON1	49.11746	70.76291	27.47202	0.388226	-1.36503	0.019306	0.789129	Down	0.687784	0.267384
SULT1A4	64.99753	6.879727	123.1153	17.89538	4.161515	2.94E-10	2.72E-07	Up	0.269626	4.831696
SYTL1	387.7331	255.5327	519.9334	2.034704	1.024819	0.000179	0.030133	Up	5.710402	11.63497
TBC1D30	1719.567	921.8835	2517.251	2.730552	1.449193	3.30E-12	4.44E-09	Up	5.567325	15.2228
TDO2	73.50849	101.2303	45.7867	0.452302	-1.14464	0.025386	0.897742	Down	3.254483	1.474037
TLR5	5.57882	0.982818	10.17482	10.3527	3.371935	0.032478	1	Up	0.010028	0.103958
TMEM45A	233.1839	105.1615	361.2062	3.434774	1.780215	1.72E-07	7.94E-05	Up	2.71586	9.341209
TNFRSF19	70.70379	29.48455	111.923	3.79599	1.924476	0.000314	0.047408	Up	0.261805	0.995176
TNR	24.703	12.77664	36.62936	2.866901	1.519492	0.036139	1	Up	0.086127	0.247258
TOX2	124.3765	72.72855	176.0244	2.420293	1.275182	0.002862	0.240661	Up	1.409026	3.414952
TSPOAP1	6607.135	4378.455	8835.815	2.018021	1.012941	2.15E-07	9.37E-05	Up	26.07937	52.70119
TTC9	22.72003	9.828182	35.61188	3.623445	1.857362	0.014241	0.646391	Up	0.103143	0.374247
TUBA1A	1441.28	893.3818	1989.178	2.226571	1.154823	3.66E-08	2.17E-05	Up	19.7548	44.04603
U2AF1L5	188.0527	298.7767	77.32864	0.258817	-1.94999	1.96E-07	8.80E-05	Down	8.678041	2.249121
WDR54	51.91504	27.51891	76.31116	2.773045	1.471471	0.01045	0.533198	Up	1.267172	3.518763
XAGE1B	204.4864	116.9554	292.0174	2.496828	1.320096	0.000176	0.030005	Up	6.663203	16.65977
XKR7	3.439864	6.879727	0	0	#NAME?	0.035382	1	Down	0.129439	0
ZNF395	2440.727	1050.633	3830.82	3.646203	1.866395	1.50E-19	5.57E-16	Up	11.97885	43.73745
ZNF703	62.37328	40.29555	84.45102	2.09579	1.067494	0.046986	1	Up	0.658761	1.382527
