# Supplementary material for: Repressing HIF-1α-induced HDAC9 contributes to the synergistic effect of venetoclax and MENIN inhibitor in KMT2Ar AML
Source: Biomark Res. 2023 Dec 5;11:105. doi: 10.1186/s40364-023-00547-9 (PMC10696732; doi:10.1186/s40364-023-00547-9)
Supplement: Supplementary file 4 — Additional file 4: Table S3. Different expressed genes of VEN vs. DMSO in MV4-11. [file 40364_2023_547_MOESM4_ESM.pdf]

gene_id	BaseMean	BaseMean_	BaseMean_	FoldChang	log2FoldCl	pValue	qValue	Regulation	Expression	Expression_MV4_11_Venetoclax
AKAP17A	72.17768	100.1172	44.23815	0.441864	-1.17833	0.003168	0.426208	Down	1.7266	0.762919
ALDH7A1	9.65998	3.888047	15.43191	3.969066	1.988799	0.038141	1	Up	0.040527	0.160853
ANK1	9.173974	2.916035	15.43191	5.292088	2.403837	0.017829	1	Up	0.016821	0.089017
APOBEC3H	21.69656	32.07639	11.31674	0.352806	-1.50305	0.020034	1	Down	1.497231	0.52823
ASMTL	299.725	164.27	435.18	2.649175	1.405543	3.89E-10	4.80E-07	Up	3.460356	9.167057
ATP1B1	12.20357	4.860058	19.54709	4.021987	2.007908	0.01901	1	Up	0.122294	0.491865
ATP6V1C2	3.086383	0	6.172765	Inf	Inf	0.037859	1	Up	0	0.099307
AXL	8.347273	13.60816	3.086383	0.226804	-2.14048	0.042405	1	Down	0.153742	0.034869
BOLA2B	31.3248	19.44023	43.20936	2.222677	1.152298	0.035106	1	Up	1.058202	2.352032
BTNL3	4.88845	8.748105	1.028794	0.117602	-3.08802	0.046665	1	Down	0.198478	0.023341
C4A	6.63038	1.944023	11.31674	5.821296	2.54134	0.036913	1	Up	0.019834	0.115459
CBS	179.4879	106.9213	252.0546	2.357385	1.237187	6.95E-06	0.003116	Up	1.531846	3.611136
CD99	322.5357	492.8099	152.2615	0.308966	-1.69448	2.50E-14	4.62E-11	Down	6.238611	1.927512
CSF2RA	118.2078	56.37668	180.039	3.193501	1.675139	5.53E-07	0.000315	Up	1.047652	3.345665
CYGB	32.07303	46.65656	17.4895	0.374856	-1.41559	0.009908	0.869088	Down	0.655382	0.245673
DACT3	15.77932	23.32828	8.230354	0.352806	-1.50305	0.043585	1	Down	0.30785	0.108611
DEPTOR	48.42353	68.04082	28.80624	0.423367	-1.24002	0.007687	0.790164	Down	0.472014	0.199834
DHRSX	12.77475	2.916035	22.63347	7.761729	2.956378	0.001191	0.223302	Up	0.062642	0.486209
DUSP27	17.75173	26.24432	9.259148	0.352806	-1.50305	0.033157	1	Down	0.349077	0.123156
EPSTI1	60.09102	108.8653	11.31674	0.103952	-3.26601	8.04E-11	1.32E-07	Down	0.813975	0.084614
FABP3	51.7404	77.76093	25.71986	0.330755	-1.59616	0.000539	0.120946	Down	1.480318	0.489622
FAM110B	9.833682	15.55219	4.115177	0.264604	-1.91809	0.044729	1	Down	0.047831	0.012656
FBXO36	14.71878	6.804082	22.63347	3.326455	1.733986	0.024567	1	Up	0.100498	0.334301
FBXO39	52.31157	75.81691	28.80624	0.379945	-1.39614	0.002138	0.340149	Down	2.517784	0.956615
FCMR	28.15659	39.85248	16.46071	0.413041	-1.27564	0.026597	1	Down	0.714209	0.294997
GAPT	30.12901	42.76851	17.4895	0.408934	-1.29006	0.021253	1	Down	0.932869	0.38148
GTPBP6	185.4052	115.6694	255.141	2.205778	1.141287	2.46E-05	0.008456	Up	1.761578	3.885637
HOMER2	32.01625	48.60058	15.43191	0.317525	-1.65506	0.00291	0.406237	Down	0.199872	0.063464
IFI27	7.34687	12.63615	2.057588	0.162833	-2.61853	0.025701	1	Down	0.844836	0.137567
IFI44	73.23822	116.6414	29.83503	0.255784	-1.967	2.08E-06	0.000992	Down	2.45053	0.626805
IFI44L	29.78831	54.43265	5.143971	0.094502	-3.40352	3.99E-07	0.000236	Down	0.508752	0.048078
IFI6	125.6667	206.0665	45.26695	0.219672	-2.18658	1.15E-10	1.70E-07	Down	11.01122	2.418844
IFIT1	136.4474	242.0309	30.86383	0.12752	-2.9712	2.25E-17	6.67E-14	Down	2.833879	0.361376
IFIT2	241.8722	401.4408	82.30354	0.20502	-2.28616	1.26E-18	4.65E-15	Down	6.358612	1.30364
IFIT3	274.8037	453.9295	95.67786	0.210777	-2.24621	5.86E-20	2.89E-16	Down	8.851379	1.86566
IFITM1	159.8224	234.2548	85.38992	0.364517	-1.45594	6.65E-07	0.000365	Down	18.08662	6.59286
IL3RA	101.1994	180.7942	21.60468	0.119499	-3.06493	1.92E-14	4.05E-11	Down	5.712659	0.682653
IRF7	213.9043	302.2956	125.5129	0.415199	-1.26812	7.94E-07	0.000405	Down	7.827303	3.249878
IRF9	341.1358	472.3977	209.874	0.444274	-1.17048	3.46E-08	2.56E-05	Down	15.58489	6.923938
ISG15	148.4705	200.2344	96.70666	0.482967	-1.05	0.000408	0.10075	Down	16.61884	8.026329
KCNE1	8.318882	14.58018	2.057588	0.141122	-2.82498	0.011856	0.920708	Down	0.143136	0.0202
KLHL1	3.888047	7.776093	0	0	#NAME?	0.017299	1	Down	0.081902	0
L1CAM	24.64099	36.93644	12.34553	0.334237	-1.58106	0.010146	0.871165	Down	0.390073	0.130376
LAMC3	57.42715	76.78892	38.06539	0.495715	-1.01242	0.019483	1	Down	0.675398	0.334803
LGSN	35.61703	48.60058	22.63347	0.465704	-1.10252	0.034559	1	Down	0.278546	0.12972
LOC10050	27.80919	16.5242	39.09418	2.365875	1.242374	0.030637	1	Up	0.925873	2.190492
LOC10192	15.77932	23.32828	8.230354	0.352806	-1.50305	0.043585	1	Down	0.753899	0.265979
LOC10798	29.61461	42.76851	16.46071	0.384879	-1.37752	0.014881	1	Down	0.498342	0.191801
LOC10798	4.88845	8.748105	1.028794	0.117602	-3.08802	0.046665	1	Down	0.122449	0.0144
LOC40049	25.18378	35.96443	14.40312	0.400482	-1.32019	0.028576	1	Down	0.142343	0.057006
MMP19	26.55662	41.7965	11.31674	0.270758	-1.88492	0.00192	0.317643	Down	0.682138	0.184694
MROH7	34.13062	46.65656	21.60468	0.463058	-1.11074	0.036312	1	Down	0.588351	0.272439
MST1L	9.833682	15.55219	4.115177	0.264604	-1.91809	0.044729	1	Down	0.184437	0.048803
MX2	167.5701	250.779	84.36113	0.336396	-1.57177	5.28E-08	3.72E-05	Down	3.462925	1.164911
NES	15.83275	3.888047	27.77744	7.144318	2.836796	0.000507	0.117253	Up	0.038726	0.276669
OAS1	282.5514	470.4537	94.64907	0.201187	-2.31339	2.56E-21	1.89E-17	Down	7.954743	1.600384
OAS2	333.926	578.347	89.5051	0.15476	-2.69189	2.76E-30	4.09E-26	Down	5.189932	0.803192
OAS3	160.4537	247.863	73.04439	0.294697	-1.7627	3.10E-09	2.70E-06	Down	2.064616	0.608433
OASL	26.92906	46.65656	7.20156	0.154353	-2.6957	3.16E-05	0.010388	Down	1.156159	0.178455
OCLN	10.7773	18.46822	3.086383	0.167119	-2.58106	0.007307	0.78345	Down	0.155472	0.025982
PCDH17	21.89195	7.776093	36.0078	4.630577	2.211192	0.000946	0.186627	Up	0.037139	0.171974
PDCD1LG2	5.83207	11.66414	0	0	#NAME?	0.002292	0.346095	Down	0.255324	0
PDF	46.4444	30.13236	62.75645	2.082693	1.05845	0.023849	1	Up	1.440498	3.000103
PIK3R6	6.346467	11.66414	1.028794	0.088201	-3.50305	0.012471	0.939818	Down	0.146222	0.012897
PLEKHG1	3.086383	0	6.172765	Inf	Inf	0.037859	1	Up	0	0.043636
PROX1	23.18297	34.02041	12.34553	0.362886	-1.46241	0.019752	1	Down	0.191493	0.06949
PTCHD4	4.88845	8.748105	1.028794	0.117602	-3.08802	0.046665	1	Down	0.018427	0.002167
PTGFRN	17.71999	9.720117	25.71986	2.646044	1.403837	0.044709	1	Up	0.083907	0.222021
PTK6	13.26411	21.38426	5.143971	0.240549	-2.05559	0.01359	0.979511	Down	0.466986	0.112333
PTN	42.95719	26.24432	59.67006	2.273638	1.185002	0.014336	0.988073	Up	0.639744	1.45454
REC8	129.8637	185.6542	74.07318	0.398985	-1.32559	2.98E-05	0.010017	Down	3.436184	1.37098
SAMD9L	707.0614	1011.864	402.2585	0.397542	-1.33082	3.16E-15	7.79E-12	Down	7.816879	3.107527
SEMA3E	3.60078	0	7.20156	Inf	Inf	0.02103	1	Up	0	0.059246
SIGLEC1	57.17499	103.0332	11.31674	0.109836	-3.18658	3.52E-10	4.74E-07	Down	0.687289	0.075489
SIK1	17.86194	4.860058	30.86383	6.350505	2.666871	0.00045	0.106057	Up	0.056749	0.360385
SLAMF7	13.29251	20.41225	6.172765	0.302405	-1.72545	0.034328	1	Down	0.350171	0.105893
SLFN5	473.1707	654.1639	292.1776	0.446643	-1.16281	7.38E-10	8.40E-07	Down	3.419303	1.527201
SLX1A	93.10432	140.9417	45.26695	0.321175	-1.63857	9.04E-06	0.003934	Down	6.668706	2.141814
SMIM11A	32.6375	9.720117	55.55489	5.715455	2.514868	1.70E-05	0.006301	Up	0.180812	1.033417
SPSB1	13.29251	20.41225	6.172765	0.302405	-1.72545	0.034328	1	Down	0.362412	0.109595

TBC1D3D	6.860864	11.66414	2.057588	0.176403	-2.50305	0.037857	1	Down	0.148919	0.02627
TEX29	5.374456	9.720117	1.028794	0.105842	-3.24002	0.029959	1	Down	0.21404	0.022654
TRIM74	499.0108	685.2682	312.7534	0.456396	-1.13164	1.04E-09	1.02E-06	Down	10.79253	4.925646
TSPYL4	14.71878	6.804082	22.63347	3.326455	1.733986	0.024567	1	Up	0.091092	0.303014
TTC39B	5.860461	10.69213	1.028794	0.09622	-3.37752	0.019297	1	Down	0.102194	0.009833
TXLNB	52.2264	78.73295	25.71986	0.326672	-1.61408	0.000452	0.106057	Down	0.777345	0.253936
U2AF1L5	196.0991	295.4916	96.70666	0.327274	-1.61143	2.72E-09	2.51E-06	Down	8.678041	2.840086
ZBTB46	25.49943	42.76851	8.230354	0.19244	-2.37752	0.000215	0.061211	Down	0.332905	0.064064
ZDHHC11I	14.74717	5.83207	23.66227	4.057267	2.020508	0.009958	0.869088	Up	0.027482	0.111501
