# Supplementary material for: Repressing HIF-1α-induced HDAC9 contributes to the synergistic effect of venetoclax and MENIN inhibitor in KMT2Ar AML
Source: Biomark Res. 2023 Dec 5;11:105. doi: 10.1186/s40364-023-00547-9 (PMC10696732; doi:10.1186/s40364-023-00547-9)
Supplement: Supplementary file 5 — Additional file 5: Table S4. Different expressed genes of MI-503 plus VEN vs. DMSO in MV4-11. [file 40364_2023_547_MOESM5_ESM.pdf]

gene_id	BaseMean	BaseMean_	BaseMean_	FoldChang	log2FoldCl	pValue	qValue	Regulation	Expression	Expression_MV4_11_MI_503_Venetoclax
A2M	28.55384	53.11646	3.99123	0.075141	-3.73425	0.004938	0.253599	Down	0.526571	0.040166
ABCA1	354.7054	676.4832	32.92765	0.048675	-4.36068	8.98E-14	3.30E-10	Down	2.702288	0.133525
ABCA6	19.53626	36.0791	2.993422	0.082968	-3.5913	0.016872	0.554369	Down	0.252627	0.021277
ABCC6	351.7631	472.0349	231.4913	0.490411	-1.02794	0.03933	0.849477	Down	3.819214	1.90135
ABCD1	657.137	948.0787	366.1954	0.38625	-1.37239	0.001579	0.130998	Down	13.89297	5.447423
ACACB	120.3697	61.13404	179.6053	2.937894	1.554783	0.031145	0.774847	Up	0.315523	0.94101
ACCS	199.7167	298.6548	100.7786	0.337442	-1.56729	0.009583	0.393125	Down	4.077104	1.396619
ACE	51.07914	87.19117	14.96711	0.171659	-2.54239	0.011254	0.438164	Down	0.862301	0.150263
ACPP	511.4007	694.5227	328.2787	0.472668	-1.0811	0.017307	0.564551	Down	8.627956	4.139917
ACSF2	348.3103	490.0745	206.5462	0.421459	-1.24654	0.01314	0.478889	Down	10.85515	4.644278
ACTA2	404.0223	642.4085	165.636	0.257836	-1.95547	7.49E-05	0.016616	Down	18.03971	4.721725
ACVRL1	82.09458	125.2747	38.91449	0.310633	-1.68672	0.040908	0.869481	Down	1.499709	0.472915
ADAM28	565.7784	921.0193	210.5374	0.228592	-2.12916	3.58E-06	0.001644	Down	6.243811	1.448898
ADAMTS1	111.1978	201.4417	20.95396	0.10402	-3.26507	5.16E-05	0.013286	Down	1.723621	0.182006
ADAMTS4	8.017579	16.03516	0	0	#NAME?	0.022782	0.665208	Down	0.197966	0
ADAMTSL	332.7609	451.991	213.5308	0.472423	-1.08185	0.032997	0.792825	Down	4.453682	2.135883
ADTRP	15.52747	28.06153	2.993422	0.106674	-3.22873	0.041737	0.879472	Down	0.210223	0.022765
AGAP11	9.51868	18.03955	0.997807	0.055312	-4.17626	0.046653	0.923535	Down	0.306996	0.017238
AGMAT	139.6419	204.4483	74.83556	0.366037	-1.44994	0.033668	0.796158	Down	3.48039	1.293243
AGRN	1383.993	2065.529	702.4565	0.340086	-1.55603	7.17E-05	0.016616	Down	14.95436	5.162778
AGXT	14.02637	26.05713	1.995615	0.076586	-3.70677	0.030945	0.774847	Down	0.8755	0.068067
AHNAK	28191.95	37997.31	18386.6	0.483892	-1.04724	0.023801	0.681403	Down	105.1075	51.63089
AK5	6.514283	13.02857	0	0	#NAME?	0.039275	0.849477	Down	0.100941	0
AKT1S1	612.1457	906.9886	317.3028	0.349842	-1.51522	0.000612	0.070392	Down	16.17862	5.745676
ALDH1A3	145.3032	283.6218	6.984652	0.024627	-5.34364	4.41E-10	8.10E-07	Down	4.22065	0.105514
ANLN	1869.591	1229.696	2509.486	2.040737	1.02909	0.006509	0.303478	Up	13.63013	28.23673
ANXA1	763.5879	1032.263	494.9125	0.479444	-1.06057	0.011447	0.441262	Down	30.8596	15.01951
ANXA2	2006.106	2740.007	1272.205	0.464307	-1.10685	0.003371	0.20365	Down	27.93683	13.1677
APOBEC3H	18.53187	33.07251	3.99123	0.120681	-3.05073	0.037455	0.832726	Down	1.497231	0.183424
APOBR	845.7812	1202.637	488.9257	0.406545	-1.29851	0.001736	0.138563	Down	17.02834	7.027619
APOL4	51.57804	87.19117	15.96492	0.183102	-2.44928	0.013805	0.48639	Down	1.352624	0.251419
ARHGAP3	477.0413	268.5889	685.4937	2.552204	1.351744	0.003612	0.210506	Up	1.767925	4.580435
ARHGEF3	484.5587	739.6216	229.4957	0.310288	-1.68832	0.000305	0.043957	Down	6.104759	1.922919
ARRB1	2900.149	3882.512	1917.786	0.493955	-1.01755	0.006624	0.305937	Down	26.48771	13.28187
ARRDC4	85.10556	133.2922	36.91888	0.276977	-1.85216	0.023931	0.682472	Down	1.759693	0.494775
ASF1A	1893.613	2630.768	1156.459	0.43959	-1.18577	0.001767	0.140284	Down	56.29737	25.12252
ASF1B	1891.407	1167.56	2615.253	2.239931	1.163454	0.002146	0.155229	Up	36.21498	82.3475
ASIC1	59.07696	94.20655	23.94738	0.254201	-1.97596	0.033573	0.796158	Down	1.064863	0.274788
ASPHD1	117.1188	171.3757	62.86187	0.366807	-1.44691	0.046233	0.923535	Down	2.638026	0.982301
ASPM	3378.935	1986.355	4771.515	2.402146	1.264324	0.00081	0.087525	Up	9.795238	23.88592
ASRGL1	130.8511	63.13843	198.5637	3.144894	1.653012	0.01869	0.592948	Up	1.182834	3.776221
ATP10A	672.0054	902.9798	441.0309	0.488417	-1.03381	0.015961	0.535193	Down	5.920432	2.935431
AUNIP	329.7506	216.4746	443.0265	2.046552	1.033195	0.042083	0.881711	Up	4.826771	10.02783
AURKB	971.1112	567.2437	1374.979	2.423965	1.277369	0.001644	0.132652	Up	17.2069	42.34054
AXL	7.015381	14.03076	0	0	#NAME?	0.032621	0.78801	Down	0.153742	0
BCL2A1	112.1364	174.3823	49.89037	0.286098	-1.80542	0.015475	0.528405	Down	9.928806	2.883625
BCL3	149.6858	234.5142	64.85749	0.276561	-1.85433	0.006041	0.289022	Down	2.348273	0.659276
BEST1	91.09899	136.2988	45.89914	0.336754	-1.57023	0.047963	0.928099	Down	1.33323	0.455769
BIRC5	1602.037	937.0545	2267.019	2.419303	1.274591	0.000905	0.094229	Up	18.63405	45.76411
BNIP3	440.3479	599.314	281.3817	0.469506	-1.09078	0.020422	0.62619	Down	19.70491	9.391683
BPI	292.0716	97.21314	486.9301	5.008891	2.324491	2.43E-05	0.007758	Up	2.729878	13.88073
BST1	301.7703	197.4329	406.1077	2.05694	1.0405	0.046232	0.923535	Up	2.266029	4.731672
BTBD19	33.55386	58.12744	8.980267	0.154493	-2.69439	0.020675	0.631294	Down	1.024944	0.160744
BTG2	2520.592	3476.623	1564.562	0.450024	-1.15193	0.00217	0.156232	Down	68.82941	31.44393
BUB1	2730.027	1606.522	3853.533	2.39868	1.262241	0.000804	0.087461	Up	23.72297	57.76554
BUB1B	1891.044	1229.696	2552.392	2.075628	1.053548	0.005338	0.266641	Up	17.64413	37.17725
C16orf45	8.518677	17.03735	0	0	#NAME?	0.019141	0.598132	Down	0.243612	0
C19orf66	137.6331	198.4351	76.83118	0.387185	-1.3689	0.045542	0.923535	Down	4.13759	1.626275
C2orf48	133.3214	52.11426	214.5286	4.116505	2.04142	0.003976	0.219742	Up	1.471134	6.14764
C3AR1	64.11648	117.2571	10.97588	0.093605	-3.41727	0.000507	0.06529	Down	1.747355	0.166039
C6orf223	870.2736	1223.683	516.8643	0.422384	-1.24337	0.002554	0.172863	Down	10.30288	4.417676
CA12	8.518677	17.03735	0	0	#NAME?	0.019141	0.598132	Down	0.217335	0
CACNA2D	1559.892	2195.814	923.9697	0.420787	-1.24884	0.001166	0.108408	Down	12.48377	5.332557
CACNB4	263.2289	367.8064	158.6514	0.431345	-1.21309	0.026503	0.725048	Down	0.973485	0.426266
CAMKK1	224.2376	332.7295	115.7457	0.347867	-1.52339	0.008747	0.37258	Down	2.053186	0.725052
CAPN11	9.51868	18.03955	0.997807	0.055312	-4.17626	0.046653	0.923535	Down	0.337953	0.018976
CBLN3	18.53626	35.07691	1.995615	0.056893	-4.13562	0.010254	0.411489	Down	0.802785	0.046364
CCDC144N	290.7718	414.9097	166.6339	0.401615	-1.31612	0.013242	0.479039	Down	3.839565	1.565378
CCDC150	305.7703	201.4417	410.0989	2.03582	1.02561	0.048476	0.933115	Up	1.745034	3.606374
CCDC9B	260.3211	406.8921	113.7501	0.279558	-1.83878	0.001013	0.100539	Down	4.101123	1.163865
CCL5	1111.704	1661.643	561.7656	0.338078	-1.56457	9.80E-05	0.019199	Down	68.00019	23.33754
CCNA2	2780.551	1667.656	3893.445	2.334681	1.223225	0.001152	0.107755	Up	31.24184	74.04429
CCNB1	2374.508	1241.722	3507.293	2.824539	1.498015	7.84E-05	0.016616	Up	30.73769	88.13456
CCNB2	1551.634	931.0413	2172.227	2.333116	1.222258	0.00148	0.127883	Up	32.27184	76.43416
CCND1	59.61757	113.2483	5.986845	0.052865	-4.24155	7.73E-05	0.016616	Down	1.417692	0.076081
CCND2	1199.21	1751.841	646.5793	0.369086	-1.43797	0.000287	0.043075	Down	14.42182	5.403494
CCNF	1369.805	826.8128	1912.797	2.313458	1.210051	0.001851	0.144574	Up	10.34799	24.30218
CCR1	423.8928	603.3228	244.4628	0.405194	-1.30331	0.006331	0.298039	Down	12.10514	4.97921
CCRL2	22.03517	38.0835	5.986845	0.157203	-2.6693	0.046851	0.923535	Down	1.003319	0.160113
CD101	53.0967	97.21314	8.980267	0.092377	-3.43632	0.001051	0.102892	Down	0.739519	0.069349
CD109	2976.665	4877.694	1075.636	0.220522	-2.18101	1.87E-08	1.96E-05	Down	28.6126	6.405245
CD180	106.1583	178.3911	33.92545	0.190175	-2.3946	0.002137	0.155229	Down	3.333833	0.643611
CD1A	20.03956	38.0835	1.995615	0.052401	-4.25426	0.007199	0.322355	Down	0.909188	0.048364
CD209	99.68351	183.4021	15.96492	0.087049	-3.52203	3.58E-05	0.010738	Down	2.259039	0.199624
CD276	14.02637	26.05713	1.995615	0.076586	-3.70677	0.030945	0.774847	Down	0.359007	0.027911
CD36	887.3702	1284.817	489.9235	0.381318	-1.39093	0.000746	0.082356	Down	11.8022	4.56854

CD38	641.2202	286.6284	995.8119	3.474226	1.796691	4.86E-05	0.01297	Up	2.702763	9.532209
CD40	24.54505	45.09888	3.99123	0.0885	-3.49819	0.010902	0.428455	Down	0.928819	0.083445
CD52	77.63517	139.3054	15.96492	0.114604	-3.12527	0.000544	0.068046	Down	14.81095	1.723094
CD70	2053.229	2843.234	1263.224	0.444291	-1.17042	0.001944	0.147139	Down	55.59276	25.07342
CD84	128.6331	189.4153	67.85091	0.358212	-1.48111	0.035186	0.808725	Down	1.22841	0.446695
CD86	76.65054	145.3186	7.98246	0.054931	-4.18624	1.92E-05	0.006727	Down	2.668395	0.148797
CDC20	2193.399	1238.716	3148.083	2.541408	1.345628	0.000377	0.051307	Up	39.70664	102.4389
CDC25B	1206.457	732.6062	1680.308	2.293603	1.197616	0.002358	0.164113	Up	9.168948	21.3484
CDC25C	247.2888	151.3318	343.2458	2.268167	1.181527	0.034058	0.797784	Up	3.161573	7.279571
CDC42EP3	3416.369	4954.864	1877.874	0.378996	-1.39975	0.00022	0.03717	Down	41.07372	15.80251
CDC7	714.374	429.9427	998.8053	2.323113	1.216059	0.0043	0.232564	Up	6.524675	15.3871
CDCA2	1127.958	654.4349	1601.481	2.447121	1.291085	0.001175	0.108554	Up	4.461923	11.08422
CDCA3	658.9057	388.8526	928.9588	2.388974	1.256391	0.003697	0.210637	Up	10.50735	25.48193
CDCA5	1544.645	929.0369	2160.253	2.325261	1.217393	0.00155	0.130998	Up	13.26988	31.3232
CDCA8	1180.406	683.4986	1677.314	2.454013	1.295143	0.001066	0.103666	Up	14.76191	36.77452
CDH2	7.015381	14.03076	0	0	#NAME?	0.032621	0.78801	Down	0.155423	0
CDHR1	39.07692	74.1626	3.99123	0.053817	-4.21579	0.000675	0.07591	Down	0.494401	0.02701
CDK1	2900.775	1890.144	3911.405	2.069369	1.049191	0.005145	0.261472	Up	29.70867	62.40921
CDKN1A	10484.66	18086.66	2882.666	0.159381	-2.64945	3.25E-10	6.83E-07	Down	368.2598	59.58239
CDKN3	572.8111	259.5691	886.0531	3.413554	1.771274	8.93E-05	0.018212	Up	8.335333	28.88399
CENPA	489.9733	250.5493	729.3973	2.911192	1.54161	0.000897	0.0941	Up	9.400692	27.78166
CENPE	1543.107	909.9952	2176.218	2.391461	1.257892	0.001087	0.104358	Up	5.671397	13.76832
CENPF	7713.616	3902.556	11524.68	2.95311	1.562235	7.47E-05	0.016616	Up	20.11659	60.30613
CENPI	606.4791	369.8108	843.1473	2.279942	1.188997	0.006785	0.30851	Up	2.98897	6.917879
CENPM	333.7177	205.4505	461.9849	2.248644	1.169055	0.021367	0.63915	Up	5.352237	12.21753
CENPN	788.4049	518.136	1058.674	2.043235	1.030855	0.013404	0.482622	Up	5.078412	10.53353
CENPO	696.9343	439.9646	953.904	2.168138	1.116457	0.008907	0.378089	Up	5.326358	11.72316
CEP55	1773.778	991.1732	2556.383	2.579149	1.366895	0.00035	0.048946	Up	19.84253	51.95183
CEP85L	564.4996	792.7381	336.2611	0.424177	-1.23726	0.005559	0.273379	Down	3.88281	1.67194
CFD	3512.33	1616.544	5408.117	3.34548	1.742213	5.34E-06	0.002307	Up	75.80334	257.439
CFDP1	613.5471	407.8943	819.2	2.008363	1.00602	0.021165	0.637003	Up	12.31056	25.0985
CFH	33.06373	62.13623	3.99123	0.064234	-3.96053	0.002075	0.152785	Down	0.758916	0.049486
CIB3	38.55606	64.14063	12.9715	0.202235	-2.30589	0.033943	0.796514	Down	4.843617	0.994384
CIT	2130.902	1177.582	3084.223	2.619116	1.38908	0.000249	0.039307	Up	5.962489	15.85294
CKAP2L	865.0736	443.9734	1286.174	2.896961	1.53454	0.000224	0.037411	Up	4.975503	14.63212
CKS1B	1174.558	746.637	1602.479	2.146262	1.101826	0.005188	0.261829	Up	49.1276	107.0375
CLSPN	1685.162	1077.362	2292.962	2.128311	1.089709	0.004241	0.23191	Up	6.231984	13.46446
CNR2	374.4662	587.2876	161.6448	0.27524	-1.86124	0.00021	0.035917	Down	8.460532	2.363938
CNTNAP1	456.3479	615.3492	297.3466	0.483216	-1.04926	0.024343	0.689985	Down	3.869197	1.897972
COL27A1	795.9304	1220.676	371.1844	0.304081	-1.71747	5.11E-05	0.013286	Down	7.287896	2.24967
COL6A1	11.02417	22.04834	0	0	#NAME?	0.008367	0.361447	Down	0.280192	0
COL6A3	551.7717	903.982	199.5615	0.220758	-2.17946	2.49E-06	0.001181	Down	4.276696	0.958413
COL9A2	176.266	297.6526	54.87941	0.184374	-2.43929	0.000194	0.034473	Down	3.700264	0.692564
COX20	456.05	251.5515	660.5486	2.625898	1.392811	0.003042	0.190918	Up	4.547542	12.12222
CPNE5	50.58682	90.19776	10.97588	0.121687	-3.03875	0.00339	0.20365	Down	0.917904	0.113388
CPZ	27.55165	51.11206	3.99123	0.078088	-3.67876	0.006007	0.288333	Down	1.080428	0.085646
CR1	53.92331	19.04175	88.80487	4.663693	2.221473	0.021886	0.652008	Up	0.100956	0.477958
CSF1R	332.3454	490.0745	174.6163	0.356306	-1.48881	0.0037	0.210637	Down	6.150742	2.224732
CSF2RA	119.3653	58.12744	180.6032	3.10702	1.635532	0.024157	0.686247	Up	1.047652	3.304369
CSPG4	718.4701	1389.045	47.89476	0.03448	-4.85808	1.45E-21	2.12E-17	Down	8.996387	0.314896
CSRP3	14.03076	28.06153	0	0	#NAME?	0.003326	0.203551	Down	1.029145	0
CTNNAL1	498.5951	314.69	682.5003	2.168802	1.116899	0.014563	0.508028	Up	6.513571	14.34058
CUX2	12.02417	23.05054	0.997807	0.043288	-4.5299	0.022451	0.659713	Down	0.082245	0.003614
CXCL10	673.2813	1257.758	88.80487	0.070606	-3.82407	2.55E-15	1.87E-11	Down	55.53536	3.980493
CXCL11	35.07032	67.14722	2.993422	0.04458	-4.48746	0.000677	0.07591	Down	2.244857	0.101591
CXCL9	6.514283	13.02857	0	0	#NAME?	0.039275	0.849477	Down	0.258318	0
CXorf21	1260.041	1735.806	784.2767	0.451823	-1.14617	0.003411	0.20365	Down	48.36438	22.18305
CYFIP2	352.9409	554.2151	151.6667	0.27366	-1.86954	0.000252	0.039307	Down	4.151903	1.153418
CYGB	28.04397	48.10547	7.98246	0.165937	-2.5913	0.035103	0.808725	Down	0.655382	0.110399
CYP1B1	339.2752	465.0196	213.5308	0.459187	-1.12285	0.026183	0.721493	Down	4.844322	2.258135
CYTH4	3444.444	4789.501	2099.387	0.438331	-1.18991	0.001596	0.131715	Down	74.84164	33.30221
CYTIP	182.3077	322.7075	41.90791	0.129863	-2.94493	9.35E-06	0.003816	Down	3.863251	0.509293
DAPK2	12.02417	23.05054	0.997807	0.043288	-4.5299	0.022451	0.659713	Down	0.11182	0.004914
DCANP1	406.677	715.5689	97.78513	0.136654	-2.8714	2.34E-08	2.29E-05	Down	12.25519	1.700079
DDIAS	471.6126	295.6482	647.5771	2.190364	1.13117	0.0146	0.508114	Up	4.310025	9.583485
DDN	300.297	435.9558	164.6382	0.377649	-1.40488	0.007721	0.340556	Down	5.194665	1.991468
DENND6B	77.08798	117.2571	36.91888	0.314854	-1.66724	0.047641	0.928099	Down	1.134773	0.362698
DEPDC1	655.9913	424.9317	887.0509	2.087514	1.061786	0.01381	0.48639	Up	4.195523	8.890845
DEPDC1B	808.7678	476.0437	1141.492	2.397872	1.261754	0.002495	0.171486	Up	4.43665	10.79962
DGKG	305.2626	197.4329	413.0923	2.092318	1.065102	0.040702	0.868015	Up	1.82704	3.880638
DIAPH3	771.3675	484.0613	1058.674	2.187065	1.128996	0.00709	0.318444	Up	1.942017	4.311639
DLGAP5	1455.548	795.7447	2115.352	2.65833	1.41052	0.000282	0.043075	Up	12.6218	34.06102
DLX5	364.3499	524.1492	204.5505	0.390253	-1.35752	0.006386	0.299641	Down	8.90019	3.52592
DNAJC9	2472.67	1641.599	3303.741	2.012514	1.008998	0.007081	0.318444	Up	37.17421	75.94656
DNHD1	409.8423	566.2415	253.4431	0.447588	-1.15976	0.015651	0.529492	Down	1.938805	0.880928
DPEP2	25.04176	44.09668	5.986845	0.135766	-2.8808	0.026992	0.730437	Down	0.75426	0.103954
DQX1	17.03296	32.07031	1.995615	0.062226	-4.00633	0.014705	0.510572	Down	0.550306	0.034762
DSN1	930.2099	571.2525	1289.167	2.256738	1.174239	0.00394	0.219178	Up	12.18572	27.91642
DTX4	338.4552	546.1975	130.7128	0.239314	-2.06302	7.47E-05	0.016616	Down	4.606693	1.119142
DYNC1I1	98.62644	156.3428	40.91011	0.261669	-1.93418	0.013529	0.483469	Down	1.317785	0.350046
E2F5	138.1342	199.4373	76.83118	0.38524	-1.37617	0.044177	0.909989	Down	5.769452	2.256281
EGFR	22.54724	44.09668	0.997807	0.022628	-5.46576	0.001455	0.127238	Down	0.299055	0.006869
EIF3CL	413.9204	150.3296	677.5113	4.506839	2.172116	1.15E-05	0.00432	Up	2.468328	11.29282
EIF4E3	672.4999	900.9754	444.0243	0.492826	-1.02085	0.017287	0.564551	Down	7.125467	3.564796
ELANE	10347.87	3922.6	16773.14	4.276027	2.096271	3.63E-07	0.000232	Up	124.8433	541.9174
EMP1	73.14065	137.301	8.980267	0.065406	-3.93444	5.56E-05	0.014089	Down	2.41702	0.160481
EPSTI1	56.62195	112.2461	0.997807	0.008889	-6.81369	1.06E-06	0.0006	Down	0.813975	0.007345

ERBB4	31.54946	54.11866	8.980267	0.165937	-2.5913	0.028295	0.741092	Down	0.23437	0.039479
ERV3-1	2358.737	3152.913	1564.562	0.496228	-1.01093	0.007013	0.316938	Down	51.83248	26.11021
ESAM	22.46164	5.010987	39.9123	7.964958	2.993667	0.028213	0.741092	Up	0.143953	1.163942
ESCO2	1133.03	692.5183	1573.542	2.272203	1.184092	0.002833	0.185459	Up	9.792575	22.58768
ESPL1	1443.398	943.0677	1943.729	2.061071	1.043394	0.006779	0.30851	Up	7.360779	15.40083
ESRP1	13.52527	25.05493	1.995615	0.07965	-3.65019	0.035141	0.808725	Down	0.341171	0.027586
ETV6	2315.898	3182.979	1448.816	0.455176	-1.1355	0.002531	0.172698	Down	17.63847	8.150198
EVI2A	1321.116	1831.015	811.2175	0.443043	-1.17448	0.002583	0.174043	Down	32.66115	14.68942
EVL	2579.953	3472.614	1687.292	0.485885	-1.04131	0.005462	0.271038	Down	30.78767	15.18582
EVPL	21.03956	39.0857	2.993422	0.076586	-3.70677	0.012137	0.45409	Down	0.314158	0.024424
FABP3	42.08351	80.17579	3.99123	0.049781	-4.32826	0.00039	0.052506	Down	1.480318	0.074808
FAM135B	133.1298	192.4219	73.83775	0.383728	-1.38184	0.046107	0.923535	Down	1.025554	0.399494
FAM83D	330.7089	198.4351	462.9827	2.33317	1.222291	0.016536	0.548219	Up	3.641248	8.624306
FAT1	297.8596	462.013	133.7062	0.289399	-1.78887	0.000833	0.088637	Down	1.636293	0.480713
FBN2	302.1748	154.3384	450.0112	2.915744	1.543864	0.003493	0.206834	Up	0.772722	2.287179
FBXO5	1168.56	741.626	1595.494	2.151346	1.10524	0.00508	0.259983	Up	15.94677	34.82655
FCER1G	347.3454	505.1075	189.5834	0.375333	-1.41376	0.005145	0.261472	Down	45.88836	17.48423
FCER2	34.55825	61.13404	7.98246	0.130573	-2.93707	0.012137	0.45409	Down	1.408144	0.18665
FCGR2A	1245.962	1685.696	806.2285	0.478276	-1.06408	0.006532	0.303571	Down	23.13	11.23006
FCGR2B	415.873	586.2854	245.4606	0.418671	-1.25611	0.008752	0.37258	Down	9.343601	3.971134
FCMR	23.53847	41.09009	5.986845	0.1457	-2.77892	0.035519	0.812563	Down	0.714209	0.105636
FGD2	191.277	317.6966	64.85749	0.204149	-2.2923	0.000286	0.043075	Down	3.139634	0.65066
FGD4	265.7498	379.8328	151.6667	0.399299	-1.32446	0.015391	0.528149	Down	1.916353	0.776785
FGL2	65.60442	113.2483	17.96053	0.158594	-2.65659	0.004248	0.23191	Down	1.424667	0.229366
FKBP10	7.51648	15.03296	0	0	#NAME?	0.02721	0.730581	Down	0.274539	0
FLNA	72444.79	99080.23	45809.34	0.462346	-1.11296	0.043688	0.905	Down	625.1204	293.3987
FOXM1	1741.054	1084.378	2397.731	2.211159	1.144803	0.002638	0.176895	Up	15.68902	35.21627
FREM2	7.51648	15.03296	0	0	#NAME?	0.02721	0.730581	Down	0.049451	0
FRMD4A	438.8775	611.3404	266.4146	0.435788	-1.1983	0.011124	0.434531	Down	1.648114	0.729104
FSCN2	40.56265	69.15162	11.97369	0.173151	-2.5299	0.019391	0.600829	Down	0.63762	0.112077
FYB1	471.8556	634.3909	309.3203	0.487586	-1.03627	0.024954	0.703441	Down	5.362319	2.654188
GABRA4	112.6155	165.3626	59.86845	0.362044	-1.46576	0.046497	0.923535	Down	0.74155	0.272539
GAS7	3276.572	4907.76	1645.385	0.335262	-1.57664	3.42E-05	0.01045	Down	28.17938	9.59054
GBP2	425.5454	674.4788	176.6119	0.261849	-1.93319	7.15E-05	0.016616	Down	8.762118	2.329101
GBP5	126.1408	190.4175	61.86406	0.324886	-1.62199	0.022564	0.661476	Down	2.523153	0.832152
GDF15	40.07692	75.1648	4.989037	0.066375	-3.91322	0.001089	0.104358	Down	2.346347	0.158096
GGH	1112.413	618.3557	1606.47	2.597971	1.377385	0.000565	0.06856	Up	13.03002	34.36425
GGT5	44.07254	77.16919	10.97588	0.142231	-2.81369	0.00858	0.369524	Down	1.499036	0.216439
GGT7	292.229	396.8701	187.5878	0.472668	-1.0811	0.04044	0.865942	Down	6.283865	3.015161
GIMAP8	24.04396	44.09668	3.99123	0.090511	-3.46576	0.012056	0.454053	Down	0.53651	0.049295
GINS2	504.0984	321.7053	686.4916	2.133914	1.093502	0.01644	0.5475	Up	14.63802	31.70932
GJA1	6.514283	13.02857	0	0	#NAME?	0.039275	0.849477	Down	0.22074	0
GLIPR1	1954.087	3134.873	773.3008	0.246677	-2.01931	1.98E-07	0.000138	Down	42.31179	10.5954
GOLGA8B	1548.831	2156.729	940.9325	0.436278	-1.19668	0.001849	0.144574	Down	17.28847	7.656795
GPM6A	16.53626	33.07251	0	0	#NAME?	0.001607	0.131839	Down	0.37709	0
GPR183	652.9739	1097.406	208.5418	0.190032	-2.39569	1.11E-07	8.56E-05	Down	27.43085	5.291667
GPR27	204.2865	107.2351	301.3379	2.810067	1.490605	0.012827	0.473709	Up	2.166964	6.181528
GPR68	106.6594	179.3933	33.92545	0.189112	-2.40269	0.002038	0.151148	Down	1.561594	0.299789
GPR84	444.9346	643.4107	246.4585	0.38305	-1.3844	0.003403	0.20365	Down	15.37416	5.978254
GPRC5C	367.0617	623.3667	110.7566	0.177675	-2.49269	1.55E-06	0.000812	Down	6.184321	1.115439
GPSM2	605.9275	345.7581	866.0969	2.504922	1.324766	0.002651	0.176984	Up	2.265873	5.761788
GRAP	186.7035	279.6131	93.7939	0.335442	-1.57587	0.010941	0.428498	Down	7.20733	2.454251
GRIN2C	27.04616	48.10547	5.986845	0.124452	-3.00633	0.018786	0.594623	Down	0.304116	0.038421
GSAP	237.242	347.7625	126.7216	0.364391	-1.45644	0.010499	0.416737	Down	5.256734	1.944515
GSN	3235.089	4646.187	1823.992	0.392578	-1.34895	0.000359	0.049358	Down	44.35653	17.67711
GTSE1	1057.958	584.281	1531.635	2.6214	1.390338	0.000547	0.068046	Up	4.678046	12.44874
HBA1	5.986845	0	11.97369	Inf	Inf	0.048299	0.93215	Up	0	1.076934
HBB	47.39805	1.002197	93.7939	93.58826	6.548256	6.63E-06	0.002782	Up	0.085958	8.166463
HDAC9	628.0557	881.9336	374.1778	0.42427	-1.23695	0.004612	0.244415	Down	3.432329	1.478286
HIP1	3941.557	5338.705	2544.409	0.476597	-1.06916	0.004574	0.244264	Down	33.77044	16.33862
HIRIP3	692.9431	439.9646	945.9215	2.149994	1.104333	0.009734	0.396027	Up	7.75013	16.91507
HIST1H2BD	33.55825	60.13184	6.984652	0.116156	-3.10587	0.00944	0.390916	Down	2.445889	0.288406
HIST1H2BK	254.9517	461.0108	48.89257	0.106055	-3.23711	8.04E-08	6.95E-05	Down	27.90575	3.004367
HIVEP1	592.4492	797.7491	387.1493	0.485302	-1.04304	0.017724	0.572126	Down	3.422212	1.685958
HJURP	965.5991	556.2195	1374.979	2.472007	1.305683	0.001314	0.11989	Up	9.364787	23.5004
HLX	1019.889	1426.127	613.6516	0.430292	-1.21661	0.002499	0.171486	Down	33.59851	14.67612
HMGA2	413.9125	602.3206	225.5045	0.374393	-1.41738	0.003264	0.203099	Down	5.334798	2.027557
HMGB1	15807.05	9469.762	22144.34	2.338426	1.225538	0.003879	0.217419	Up	83.54363	198.3191
HMGB2	9843.45	6186.564	13500.34	2.182202	1.125785	0.004741	0.247747	Up	200.9476	445.149
HMGB3	2150.881	1187.604	3114.157	2.622219	1.390788	0.000244	0.038987	Up	15.27655	40.6651
HMGN2	11201.9	5926.995	16476.8	2.779958	1.475063	0.000296	0.043957	Up	161.62	456.1007
HMGN5	321.7549	210.4614	433.0485	2.057614	1.040973	0.042059	0.881711	Up	5.315149	11.10215
HMMR	1197.602	789.7315	1605.472	2.032934	1.023564	0.009141	0.381755	Up	13.4995	27.8592
HOMER2	29.04616	50.10987	7.98246	0.159299	-2.65019	0.029765	0.766027	Down	0.199872	0.032322
HS6ST2	395.9564	604.325	187.5878	0.310409	-1.68776	0.000597	0.069984	Down	4.458253	1.404838
HSD3B7	40.05497	65.14283	14.96711	0.229758	-2.12181	0.046127	0.923535	Down	1.465279	0.341758
HSH2D	174.7122	271.5955	77.82898	0.286562	-1.80308	0.004757	0.247747	Down	4.572716	1.330211
HSPA1B	425.6455	264.5801	586.7108	2.217517	1.148945	0.015595	0.529492	Up	5.637192	12.68987
HSPG2	51.08353	89.19556	12.9715	0.145428	-2.78163	0.00624	0.29538	Down	0.316988	0.046797
HTR7	228.8506	388.8526	68.84872	0.177056	-2.49772	3.19E-05	0.009974	Down	3.797401	0.682535
HUNK	125.328	47.10327	203.5527	4.321414	2.111503	0.003657	0.210637	Up	0.342458	1.502312
ICAM1	1523.675	2060.518	986.8316	0.478924	-1.06213	0.005641	0.276156	Down	34.0827	16.57022
ICAM2	181.6859	266.5845	96.78733	0.363064	-1.4617	0.018953	0.597355	Down	9.398124	3.463796
IER3	314.2472	199.4373	429.0572	2.151339	1.105235	0.032312	0.78801	Up	8.635567	18.85937
IFI16	856.627	1371.006	342.248	0.249633	-2.00212	2.23E-06	0.001101	Down	14.42231	3.654803
IFI44	62.62636	120.2637	4.989037	0.041484	-4.5913	2.38E-05	0.007758	Down	2.45053	0.103198
IFI44L	28.06153	56.12305	0	0	#NAME?	7.92E-05	0.016616	Down	0.508752	0

IFI6	141.1562	212.4658	69.84652	0.328742	-1.60497	0.018835	0.594897	Down	11.01122	3.674674
IFIT1	127.2681	249.5471	4.989037	0.019992	-5.64441	9.90E-10	1.45E-06	Down	2.833879	0.057514
IFIT2	229.4044	413.9075	44.90134	0.108482	-3.20448	2.76E-07	0.000184	Down	6.358612	0.700238
IFIT3	258.4594	468.0261	48.89257	0.104465	-3.2589	5.98E-08	5.49E-05	Down	8.851379	0.938666
IFITM1	144.7122	241.5296	47.89476	0.198298	-2.33426	0.000822	0.088159	Down	18.08662	3.640848
IGFBP2	118.3741	61.13404	175.6141	2.872608	1.522361	0.035718	0.814581	Up	1.635467	4.769203
IGFBP5	6.013184	12.02637	0	0	#NAME?	0.047509	0.927888	Down	0.103497	0
IL12RB1	80.60445	128.2813	32.92765	0.256683	-1.96194	0.019328	0.600829	Down	1.659668	0.43246
IL18	293.2421	403.8855	182.5988	0.452105	-1.14527	0.02999	0.766027	Down	18.56614	8.520964
IL18R1	32.45289	11.02417	53.8816	4.887588	2.289123	0.046757	0.923535	Up	0.140863	0.698905
IL1B	289.4331	487.0679	91.79829	0.188471	-2.40758	1.40E-05	0.005023	Down	16.72087	3.199128
IL21R	127.1913	214.4702	39.9123	0.186097	-2.42587	0.000941	0.095328	Down	2.188794	0.413497
IL4I1	544.0088	1004.202	83.81583	0.083465	-3.58268	6.62E-13	1.94E-09	Down	21.58414	1.828805
IL7R	171.2792	298.6548	43.90353	0.147004	-2.76607	3.90E-05	0.011225	Down	3.466331	0.517282
IPCEF1	510.0028	739.6216	280.3839	0.379091	-1.39938	0.002252	0.160543	Down	5.711413	2.197934
IQCN	64.60002	110.2417	18.95834	0.171971	-2.53977	0.006195	0.29538	Down	1.331021	0.232363
IQGAP3	1394.97	927.0325	1862.907	2.009537	1.006863	0.009149	0.381755	Up	7.403517	15.10295
IQSEC2	122.1276	180.3955	63.85968	0.353998	-1.49819	0.036517	0.821315	Down	0.667658	0.239929
IRF7	212.2178	311.6834	112.7522	0.361753	-1.46692	0.013038	0.477546	Down	7.827303	2.874427
IRF9	329.3454	487.0679	171.6229	0.352359	-1.50488	0.003447	0.204961	Down	15.58489	5.574642
IRGQ	664.6096	943.0677	386.1515	0.409463	-1.28819	0.002892	0.185459	Down	5.174208	2.150732
ISG15	138.6485	206.4526	70.84433	0.343151	-1.54309	0.024543	0.693193	Down	16.61884	5.789126
ITGA7	1210.184	1750.839	669.5288	0.382405	-1.38683	0.000455	0.060152	Down	19.6911	7.643998
ITGAL	4991.815	6963.267	3020.363	0.433757	-1.20504	0.001566	0.130998	Down	68.51179	30.16747
ITGAX	702.0816	1195.621	208.5418	0.174421	-2.51935	1.82E-08	1.96E-05	Down	12.81845	2.269669
JAK3	13.02418	24.05274	1.995615	0.082968	-3.5913	0.039948	0.859029	Down	0.187653	0.015805
JAML	26.54506	47.10327	5.986845	0.1271	-2.97596	0.02056	0.62909	Down	0.955801	0.123322
JPH2	128.1957	217.4768	38.91449	0.178936	-2.48248	0.000713	0.079358	Down	1.589096	0.288653
JUP	14.53186	29.06372	0	0	#NAME?	0.002868	0.185459	Down	0.358648	0
KCNN2	434.9236	628.3777	241.4694	0.384274	-1.37979	0.003696	0.210637	Down	6.682233	2.606697
KCNN4	903.2495	1245.731	560.7678	0.450152	-1.15152	0.004859	0.25129	Down	21.42386	9.790027
KCNQ3	2066.783	2881.317	1252.248	0.43461	-1.20221	0.001466	0.127387	Down	6.295885	2.77769
KDM1B	3173.527	4328.49	2018.565	0.466344	-1.10053	0.003402	0.20365	Down	50.51152	23.91245
KDM6B	835.1945	1152.527	517.8621	0.449328	-1.15416	0.005304	0.265881	Down	6.43722	2.936222
KIAA1551	6177.055	8942.607	3411.504	0.381489	-1.39029	0.000325	0.046387	Down	71.72732	27.77755
KIAA1614	40.57143	73.1604	7.98246	0.109109	-3.19616	0.004643	0.244415	Down	0.945391	0.104713
KIF11	3846.425	2221.871	5470.978	2.462329	1.300024	0.000599	0.069984	Up	23.49267	58.7227
KIF14	1965.954	1263.771	2668.137	2.111251	1.078098	0.004307	0.232564	Up	8.526496	18.27418
KIF15	1200.721	619.3579	1782.084	2.877309	1.52472	0.000125	0.023851	Up	6.225067	18.18269
KIF18A	425.573	231.5076	619.6385	2.676536	1.420367	0.002992	0.189408	Up	2.966589	8.060427
KIF18B	939.2209	585.2832	1293.159	2.209458	1.143692	0.004894	0.25221	Up	6.146056	13.78509
KIF20A	1192.273	634.3909	1750.154	2.758795	1.464038	0.000227	0.037472	Up	9.813152	27.48245
KIF22	1599.674	997.1863	2202.161	2.208375	1.142985	0.002837	0.185459	Up	19.6191	43.98244
KIF23	1223.501	769.6875	1677.314	2.179215	1.123808	0.004197	0.230866	Up	10.64822	23.55616
KIF2C	2106.557	1223.683	2989.431	2.442979	1.288641	0.000661	0.075231	Up	18.63343	46.21043
KIF4A	1368.102	732.6062	2003.597	2.73489	1.451483	0.000207	0.035722	Up	8.739122	24.26248
KIF4B	21.46384	5.010987	37.91668	7.56671	2.919666	0.034236	0.800664	Up	0.061455	0.47205
KIFC1	2060.493	1148.518	2972.469	2.58809	1.371888	0.000301	0.043957	Up	18.57962	48.81393
KLHL6	339.262	459.0064	219.5176	0.478245	-1.06418	0.03487	0.807489	Down	3.104658	1.507274
KNL1	2085.702	1268.782	2902.622	2.287724	1.193913	0.001572	0.130998	Up	7.110208	16.51252
KRT17	24.04396	44.09668	3.99123	0.090511	-3.46576	0.012056	0.454053	Down	1.504206	0.138209
KRT86	7.51648	15.03296	0	0	#NAME?	0.02721	0.730581	Down	0.123266	0
KYNU	435.3654	602.3206	268.4102	0.445627	-1.16609	0.013608	0.483828	Down	2.311949	1.045869
L1CAM	21.53627	38.0835	4.989037	0.131003	-2.93233	0.033335	0.792969	Down	0.390073	0.051874
LAMC3	43.07912	79.17359	6.984652	0.088219	-3.50276	0.001961	0.14768	Down	0.675398	0.060486
LGALS1	8658.545	12789.04	4528.05	0.354057	-1.49795	0.000164	0.029733	Down	1230.58	442.2937
LGALS3	34.57142	67.14722	1.995615	0.02972	-5.07242	0.000302	0.043957	Down	3.463247	0.104486
LILRB4	1398.707	1950.276	847.1386	0.434369	-1.20301	0.001923	0.146562	Down	26.46952	11.67164
LIMD2	1738.158	2495.471	980.8448	0.39305	-1.34722	0.00043	0.05736	Down	37.28041	14.87496
LMO7	1093.149	706.5491	1479.749	2.094332	1.06649	0.00728	0.324972	Up	2.772675	5.89484
LOC10192	14.03076	28.06153	0	0	#NAME?	0.003326	0.203551	Down	3.019374	0
LOC10537	29.54507	50.10987	8.980267	0.179212	-2.48026	0.038793	0.849477	Down	0.405192	0.073715
LOC10798	83.1275	141.3098	24.94519	0.176528	-2.50203	0.003306	0.203551	Down	0.727505	0.13037
LOC33986	296.5516	548.2019	44.90134	0.081907	-3.60988	8.25E-10	1.35E-06	Down	12.60549	1.048108
LPAR5	15.02638	27.05933	2.993422	0.110624	-3.17626	0.046888	0.923535	Down	0.497554	0.055875
LPAR6	106.6133	158.3472	54.87941	0.346577	-1.52875	0.041982	0.881711	Down	1.879291	0.661181
LPXN	1454.18	1993.37	914.9895	0.459016	-1.12338	0.00359	0.210049	Down	19.26336	8.976098
LST1	3159.712	4398.644	1920.779	0.436675	-1.19537	0.0015	0.128077	Down	263.8773	116.9737
LTB	123.2308	228.501	17.96053	0.078602	-3.6693	4.36E-06	0.001941	Down	13.72324	1.095003
LY86	484.9039	669.4678	300.3401	0.448625	-1.15642	0.012022	0.454053	Down	26.76455	12.18908
LY96	30.55385	55.12085	5.986845	0.108613	-3.20273	0.010052	0.406696	Down	2.245467	0.24758
MAML2	208.209	303.6658	112.7522	0.371304	-1.42933	0.016119	0.539265	Down	1.519789	0.572849
MAP1B	9.520875	19.04175	0	0	#NAME?	0.013638	0.483828	Down	0.083242	0
MAP7	48.57804	84.18458	12.9715	0.154084	-2.69821	0.008742	0.37258	Down	0.378148	0.059149
MAPK10	18.03736	35.07691	0.997807	0.028446	-5.13562	0.004463	0.240102	Down	0.109166	0.003152
MARCKS	648.2092	288.6328	1007.786	3.491583	1.803881	4.38E-05	0.012149	Up	3.598967	12.75639
MDGA1	500.947	249.5471	752.3469	3.014849	1.592086	0.000579	0.06966	Up	1.38903	4.251132
MDM2	4330.599	5975.1	2686.098	0.449549	-1.15345	0.002322	0.163143	Down	40.21216	18.35107
MEF2C	3525.96	5106.195	1945.725	0.381052	-1.39194	0.00024	0.038722	Down	34.98274	13.5321
MELK	907.7943	587.2876	1228.301	2.091481	1.064525	0.009045	0.379572	Up	10.80247	22.9353
MFAP4	884.7833	559.2261	1210.34	2.164313	1.113909	0.006571	0.304457	Up	15.31144	33.6406
MILR1	441.9785	660.448	223.5089	0.33842	-1.56311	0.001024	0.100902	Down	10.22506	3.512768
MIS18A	470.0589	269.5911	670.5266	2.487199	1.314522	0.004755	0.247747	Up	6.293379	15.88993
MKI67	16716.92	9410.633	24023.21	2.552773	1.352065	0.001638	0.132652	Up	38.24919	99.12016
MLPH	49.60218	96.21094	2.993422	0.031113	-5.00633	4.35E-05	0.012149	Down	0.76258	0.024086
MMP19	25.03957	43.09448	6.984652	0.162078	-2.62524	0.040446	0.865942	Down	0.682138	0.112234
MMP9	81.15164	150.3296	11.97369	0.07965	-3.65019	7.60E-05	0.016616	Down	3.455237	0.279376

MNDA	352.7023	217.4768	487.9279	2.243586	1.165806	0.019702	0.607906	Up	6.611934	15.05909
MPEG1	92.11216	143.3142	40.91011	0.285457	-1.80865	0.023347	0.673662	Down	1.705401	0.494191
MS4A14	295.853	457.002	134.704	0.294756	-1.76241	0.001007	0.100539	Down	7.1081	2.126882
MS4A3	867.4562	392.8614	1342.051	3.416093	1.772347	2.32E-05	0.007756	Up	12.49606	43.33415
MS4A7	1461.059	2172.764	749.3534	0.344885	-1.53581	8.12E-05	0.016798	Down	38.77007	13.5737
MUC8	18.03077	32.07031	3.99123	0.124452	-3.00633	0.041636	0.878603	Down	0.78091	0.098658
MVP	688.7194	1017.23	360.2085	0.354107	-1.49774	0.000527	0.06728	Down	18.67238	6.712152
MX2	179.1738	258.5669	99.78075	0.385899	-1.3737	0.027801	0.737023	Down	3.462925	1.356577
MXD3	292.2648	185.4065	399.123	2.152691	1.106142	0.03612	0.814849	Up	3.149247	6.882021
MXD4	680.0471	930.0391	430.055	0.462405	-1.11277	0.009449	0.390916	Down	9.228474	4.331918
MYBL1	276.7439	160.3516	393.1362	2.451714	1.293791	0.01643	0.5475	Up	1.495229	3.721389
MYCL	155.1496	223.49	86.80925	0.388426	-1.36429	0.037649	0.833968	Down	2.423658	0.955667
MYO1B	856.9195	1276.799	437.0397	0.342293	-1.5467	0.000205	0.035722	Down	12.42134	4.316127
MYO1G	4860.457	7352.12	2368.795	0.322192	-1.63401	2.20E-05	0.007501	Down	120.0934	39.2791
MYOF	551.4221	972.1314	130.7128	0.13446	-2.89475	1.46E-09	1.95E-06	Down	6.152201	0.839752
NABP1	927.9344	1582.47	273.3993	0.172767	-2.5331	3.04E-09	3.72E-06	Down	15.49613	2.717771
NCAM2	387.3719	557.2217	217.522	0.390369	-1.35709	0.005562	0.273379	Down	1.461345	0.579103
NCAPG2	2448.932	1509.309	3388.554	2.245103	1.166782	0.001913	0.146562	Up	13.52649	30.82825
NCAPH	1655.109	1023.243	2286.975	2.235025	1.160291	0.002394	0.165862	Up	9.048017	20.52879
NCF1	1093.018	1785.916	400.1208	0.224042	-2.15816	1.51E-07	0.000111	Down	65.72216	14.94753
NCKAP5L	458.9631	670.47	247.4563	0.369079	-1.438	0.00221	0.1583	Down	6.678775	2.502323
NCS1	449.3874	626.3733	272.4014	0.434887	-1.20129	0.010475	0.416737	Down	6.71277	2.963503
NDC80	872.7306	523.147	1222.314	2.336464	1.224327	0.002947	0.187343	Up	12.93213	30.67303
NDUFB6	395.6915	255.5603	535.8226	2.096658	1.068092	0.027121	0.730581	Up	15.93663	33.91967
NEFH	521.525	305.6702	737.3797	2.412338	1.270432	0.005165	0.261563	Up	4.41062	10.80103
NEIL3	777.6338	383.8416	1171.426	3.051848	1.609683	0.000148	0.0275	Up	8.579959	26.58126
NEK2	872.0012	417.9163	1326.086	3.17309	1.665889	6.48E-05	0.016135	Up	6.849385	22.06285
NFKB2	1439.114	1948.272	929.9566	0.477324	-1.06696	0.005664	0.276367	Down	21.53269	10.43371
NKG7	659.1436	953.0897	365.1975	0.383172	-1.38393	0.001436	0.127041	Down	53.41638	20.77763
NOG	70.59565	114.2505	26.9408	0.235805	-2.08434	0.017975	0.577664	Down	3.242225	0.77611
NR5A2	6.514283	13.02857	0	0	#NAME?	0.039275	0.849477	Down	0.084504	0
NRGN	737.295	416.9141	1057.676	2.536916	1.343076	0.001571	0.130998	Up	18.37831	47.33028
NRP1	1064.87	1462.206	667.5332	0.456525	-1.13123	0.004609	0.244415	Down	12.40647	5.749633
NUCB2	2107.223	1072.351	3142.096	2.9301	1.55095	4.71E-05	0.012809	Up	15.09602	44.9027
NUF2	1109.938	627.3755	1592.501	2.538353	1.343893	0.000762	0.083536	Up	15.18016	39.11615
NUSAP1	3463.002	1873.107	5052.897	2.697602	1.431678	0.00016	0.029395	Up	30.04782	82.28458
OAS1	269.9715	485.0635	54.87941	0.113139	-3.14384	9.40E-08	7.67E-05	Down	7.954743	0.913618
OAS2	310.1274	596.3074	23.94738	0.040159	-4.63812	8.47E-14	3.30E-10	Down	5.189932	0.211581
OAS3	154.721	255.5603	53.8816	0.210837	-2.2458	0.000938	0.095328	Down	2.064616	0.44189
OASL	25.05054	48.10547	1.995615	0.041484	-4.5913	0.002305	0.162771	Down	1.156159	0.048689
OCSTAMP	24.04835	46.10108	1.995615	0.043288	-4.5299	0.002882	0.185459	Down	1.247601	0.054824
OIP5	213.8139	129.2835	298.3444	2.307677	1.206441	0.039297	0.849477	Up	5.616046	13.15628
ONECUT2	1487.132	2004.395	969.8689	0.483871	-1.0473	0.006438	0.301123	Down	6.675296	3.278897
OPRL1	242.2003	333.7317	150.6689	0.451467	-1.14731	0.04078	0.868015	Down	4.454035	2.041303
OPTN	904.8165	1277.802	531.8314	0.416208	-1.26462	0.002033	0.151148	Down	19.07345	8.058742
ORC6	683.4595	437.9602	928.9588	2.121103	1.084815	0.011277	0.438164	Up	13.25523	28.54148
P2RX7	936.698	1255.753	617.6428	0.49185	-1.02371	0.011596	0.44583	Down	12.66642	6.324332
PBK	430.1707	280.6153	579.7262	2.065911	1.046778	0.026833	0.728453	Up	7.007757	14.69664
PCBP4	224.1827	307.6746	140.6909	0.457272	-1.12888	0.049658	0.943511	Down	5.700323	2.646069
PCDHGA1:	37.06374	66.14502	7.98246	0.120681	-3.05073	0.008116	0.353688	Down	0.737883	0.090397
PCDHGC5	52.10987	102.2241	1.995615	0.019522	-5.67876	9.67E-06	0.003838	Down	1.139181	0.022576
PDGFA	301.8508	462.013	141.6887	0.306677	-1.70521	0.001337	0.12123	Down	7.427008	2.312184
PDYN	7.483556	0	14.96711	Inf	Inf	0.027732	0.73653	Up	0	0.171887
PEG10	44.59559	88.19336	0.997807	0.011314	-6.46576	1.14E-05	0.00432	Down	0.714322	0.008204
PEX5L	6.013184	12.02637	0	0	#NAME?	0.047509	0.927888	Down	0.051852	0
PHACTR1	30.54726	52.11426	8.980267	0.172319	-2.53685	0.033124	0.792825	Down	0.339451	0.05938
PHF19	807.8709	522.1448	1093.597	2.094432	1.066559	0.010254	0.411489	Up	4.69673	9.985952
PHGDH	2633.875	1668.659	3599.092	2.156877	1.108944	0.003128	0.195496	Up	11.49954	25.17871
PHLDA1	428.3171	573.2569	283.3773	0.494329	-1.01646	0.031647	0.781171	Down	5.205322	2.612107
PIF1	325.6255	155.3406	495.9103	3.192407	1.674644	0.001256	0.115294	Up	2.847553	9.228211
PIM1	2504.621	3473.616	1535.626	0.442083	-1.17761	0.001734	0.138563	Down	68.84605	30.89656
PIMREG	98.87074	40.08789	157.6536	3.932698	1.975519	0.011743	0.448222	Up	0.927351	3.702222
PITPNM2	661.4911	885.9424	437.0397	0.493305	-1.01945	0.017793	0.573096	Down	6.1196	3.064544
PLA2G4C	62.07917	98.21534	25.94299	0.264144	-1.9206	0.035341	0.811031	Down	1.311449	0.351657
PLAU	5619.023	7685.851	3552.195	0.462173	-1.11349	0.003589	0.210049	Down	147.013	68.97442
PLB1	293.864	460.0086	127.7194	0.277646	-1.84868	0.0006	0.069984	Down	2.118051	0.596973
PLEKHA4	13.52527	25.05493	1.995615	0.07965	-3.65019	0.035141	0.808725	Down	0.413665	0.033447
PLK1	2059.963	1361.986	2757.94	2.02494	1.017879	0.006853	0.310647	Up	32.11559	66.01695
POU2F2	586.537	831.8238	341.2502	0.410243	-1.28545	0.003729	0.211485	Down	4.500397	1.874217
PPP2R3B	49.92988	18.03955	81.82021	4.535601	2.181294	0.027902	0.738125	Up	0.279208	1.285553
PRAME	53.06817	84.18458	21.95176	0.260758	-1.93922	0.043768	0.905391	Down	1.223607	0.323897
PRC1	2550.6	1687.7	3413.499	2.022574	1.016193	0.006677	0.306441	Up	28.40605	58.3234
PRTN3	2338.068	1232.703	3443.434	2.793402	1.482023	9.32E-05	0.018494	Up	61.85585	175.4049
PSRC1	262.7921	168.3692	357.2151	2.121618	1.085165	0.046679	0.923535	Up	3.921912	8.446807
PTGIR	50.58243	88.19336	12.9715	0.14708	-2.76533	0.006675	0.306441	Down	1.694183	0.252954
PTN	13.52966	27.05933	0	0	#NAME?	0.003863	0.21737	Down	0.639744	0
PTPN3	253.8266	402.8833	104.7698	0.26005	-1.94314	0.0006	0.069984	Down	1.922624	0.50755
PTPRD	7.015381	14.03076	0	0	#NAME?	0.032621	0.78801	Down	0.05429	0
PTPRE	1483.395	2120.65	846.1408	0.399001	-1.32554	0.000613	0.070392	Down	10.29113	4.16835
PTPRO	92.60668	141.3098	43.90353	0.31069	-1.68645	0.033367	0.792969	Down	0.414508	0.130734
PTPRZ1	8.518677	17.03735	0	0	#NAME?	0.019141	0.598132	Down	0.110719	0
PTTG1	1186.78	632.3865	1741.174	2.753338	1.461182	0.000235	0.038369	Up	20.57808	57.51644
PXMP2	405.6959	267.5867	543.8051	2.032258	1.023083	0.032962	0.792825	Up	15.0127	30.9717
QPRT	1079.971	1523.34	636.6012	0.417898	-1.25878	0.001639	0.132652	Down	31.82511	13.50107
RAB17	111.1342	172.3779	49.89037	0.289424	-1.78874	0.016723	0.551931	Down	2.474003	0.72688
RAB6B	224.6926	312.6856	136.6996	0.437179	-1.1937	0.038057	0.840509	Down	2.952098	1.31014
RACGAP1	1872.418	1153.529	2591.306	2.246416	1.167625	0.002081	0.152785	Up	14.62106	33.34237

RAD54L	900.8316	597.3096	1204.354	2.016297	1.011708	0.013117	0.478889	Up	9.975272	20.4177
RAET1E	19.53846	37.0813	1.995615	0.053817	-4.21579	0.008094	0.353688	Down	0.381921	0.020865
REC8	129.6353	191.4197	67.85091	0.354461	-1.4963	0.032955	0.792825	Down	3.436184	1.23644
RELB	309.8157	453.9954	165.636	0.364841	-1.45466	0.005427	0.270171	Down	10.6958	3.961359
RENBP	58.07916	94.20655	21.95176	0.233017	-2.10149	0.025401	0.709969	Down	3.25489	0.769932
RETREG1	12.47698	2.004395	22.94957	11.44963	3.517229	0.046446	0.923535	Up	0.02478	0.288017
RFC5	1277.031	836.8348	1717.227	2.05205	1.037066	0.007831	0.344353	Up	9.814544	20.44493
RGS1	496.3765	668.4656	324.2874	0.485122	-1.04358	0.02237	0.659713	Down	25.58159	12.59813
RHOBTB3	1639.987	2319.085	960.8886	0.41434	-1.27111	0.000914	0.094528	Down	20.52338	8.632424
RNASE6	96.14071	160.3516	31.92984	0.199124	-2.32826	0.003786	0.213862	Down	7.47355	1.510699
RNF207	161.6902	248.5449	74.83556	0.301095	-1.73171	0.008142	0.353813	Down	3.206336	0.980031
RPH3A	6.514283	13.02857	0	0	#NAME?	0.039275	0.849477	Down	0.130703	0
RRM2	4775.359	3121.845	6428.874	2.059319	1.042167	0.005977	0.288333	Up	48.81094	102.0395
RXFP1	711.1876	1025.248	397.1274	0.387348	-1.3683	0.001385	0.124445	Down	12.70417	4.995453
S100A3	21.04175	40.08789	1.995615	0.049781	-4.32826	0.005706	0.277491	Down	2.916507	0.147385
S100A4	3783.01	5158.31	2407.709	0.466763	-1.09924	0.003541	0.208846	Down	491.0599	232.6798
S100B	20.04175	39.0857	0.997807	0.025529	-5.29174	0.002691	0.178023	Down	1.661578	0.04306
SAMD9L	752.6361	1043.287	461.9849	0.442816	-1.17522	0.005303	0.265881	Down	7.816879	3.513862
SAMSN1	445.3281	595.3052	295.351	0.496134	-1.0112	0.030869	0.774847	Down	8.676134	4.36971
SAPCD2	1155.417	663.4546	1647.38	2.483034	1.312104	0.000948	0.095352	Up	9.080278	22.88807
SDHAF3	390.7896	523.147	258.4321	0.493995	-1.01743	0.035714	0.814581	Down	13.65512	6.847719
SEMA6A	844.8515	1233.705	455.998	0.369617	-1.4359	0.000561	0.06856	Down	8.02612	3.011514
SERPINE1	70.6154	123.2703	17.96053	0.1457	-2.77892	0.00234	0.163667	Down	2.074789	0.306876
SERPINF1	31.06153	59.12964	2.993422	0.050625	-4.30401	0.0015	0.128077	Down	1.704114	0.087577
SESN1	1313.76	1889.142	738.3775	0.390853	-1.3553	0.00054	0.068046	Down	27.93473	11.08373
SGK1	729.0691	989.1688	468.9695	0.474105	-1.07672	0.01091	0.428455	Down	13.21474	6.360047
SGO1	412.6213	240.5274	584.7152	2.430972	1.281533	0.007644	0.338175	Up	2.216666	5.470256
SGO2	1329.02	883.938	1774.102	2.007043	1.005072	0.009609	0.393125	Up	7.318432	14.91084
SH2D3C	65.58027	102.2241	28.93642	0.283068	-1.82078	0.041412	0.876931	Down	1.218059	0.350016
SH3PXD2B	42.06595	72.15821	11.97369	0.165937	-2.5913	0.015719	0.529492	Down	0.480621	0.08096
SH3TC1	167.1694	244.5361	89.80267	0.367237	-1.44522	0.024133	0.686247	Down	2.04478	0.76229
SH3TC2	34.54947	57.12525	11.97369	0.209604	-2.25426	0.04508	0.922121	Down	0.115393	0.024553
SIGLEC1	55.11207	106.2329	3.99123	0.037571	-4.73425	3.88E-05	0.011225	Down	0.687289	0.026213
SIGLEC14	40.55826	67.14722	13.9693	0.20804	-2.26507	0.033755	0.796158	Down	0.694919	0.14676
SKA3	1536.696	944.0699	2129.321	2.25547	1.173428	0.002268	0.160936	Up	17.20591	39.39509
SLAMF7	11.02198	21.04614	0.997807	0.04741	-4.39865	0.029918	0.766027	Down	0.350171	0.016853
SLC22A18	145.6397	209.4592	81.82021	0.390626	-1.35614	0.043243	0.898307	Down	4.893907	1.940637
SLC2A3	3558.996	4927.804	2190.187	0.444455	-1.16989	0.001915	0.146562	Down	67.1868	30.31372
SLC37A2	1136.753	1708.746	564.759	0.330511	-1.59723	6.78E-05	0.016333	Down	21.86494	7.336036
SLC43A2	790.1373	1081.371	498.9037	0.461362	-1.11603	0.007507	0.334128	Down	6.297236	2.949304
SLC44A1	390.1289	221.4856	558.7722	2.522837	1.335047	0.006253	0.29538	Up	0.948772	2.429846
SLC44A2	439.3347	592.2986	286.3708	0.48349	-1.04844	0.025802	0.715234	Down	8.651102	4.246067
SLC48A1	452.1247	281.6174	622.6319	2.210914	1.144643	0.014455	0.506677	Up	2.744688	6.160164
SLC4A8	395.3148	539.1822	251.4475	0.46635	-1.10052	0.022916	0.666476	Down	1.963613	0.929598
SLFN5	459.4708	674.4788	244.4628	0.362447	-1.46416	0.001842	0.144574	Down	3.419303	1.258084
SMAGP	268.7476	381.8372	155.658	0.407655	-1.29458	0.017374	0.564551	Down	16.54677	6.847527
SNX20	946.7859	1305.863	587.7086	0.450054	-1.15183	0.004556	0.244225	Down	12.18524	5.56706
SOCS3	412.8621	578.2679	247.4563	0.427927	-1.22456	0.010681	0.422838	Down	11.34385	4.927849
SORL1	1185.604	778.7073	1592.501	2.045057	1.032141	0.008664	0.372053	Up	3.227328	6.700018
SPAG5	2549.358	1576.456	3522.26	2.23429	1.159816	0.002025	0.151148	Up	22.22753	50.41482
SPC24	608.4462	356.7822	860.1101	2.410742	1.269477	0.003894	0.217435	Up	6.754656	16.53033
SPC25	377.6278	208.457	546.7985	2.623075	1.391259	0.004803	0.249248	Up	6.55676	17.45933
SPON2	199.2748	324.7119	73.83775	0.227395	-2.13673	0.000554	0.068326	Down	7.035633	1.624093
SRC	178.2002	269.5911	86.80925	0.322003	-1.63485	0.009523	0.392314	Down	3.140545	1.026581
SREBF1	2380.381	3240.104	1520.659	0.469324	-1.09134	0.003656	0.210637	Down	30.89988	14.72167
SRPK3	61.60221	108.2373	14.96711	0.138281	-2.85433	0.002943	0.187343	Down	2.902813	0.407481
SSPO	194.2397	303.6658	84.81364	0.279299	-1.84012	0.002845	0.185459	Down	1.045683	0.296481
ST18	69.08577	108.2373	29.93422	0.276561	-1.85433	0.034841	0.807489	Down	0.402956	0.11313
ST5	57.57586	92.20215	22.94957	0.248905	-2.00633	0.03261	0.78801	Down	0.798722	0.201817
ST6GALNA	247.2091	342.7515	151.6667	0.442498	-1.17626	0.034851	0.807489	Down	4.47323	2.009369
STAP1	48.57584	83.18238	13.9693	0.167936	-2.57402	0.011757	0.448222	Down	1.702055	0.290165
STAT6	5965.061	8049.649	3880.473	0.482067	-1.05269	0.005994	0.288333	Down	96.9707	47.45432
STIL	1017.694	651.4283	1383.959	2.124499	1.087123	0.006753	0.30851	Up	5.602469	12.08269
STMN1	5800.931	3270.17	8331.693	2.547786	1.349244	0.000459	0.060152	Up	48.74531	126.0733
STON1	42.06595	72.15821	11.97369	0.165937	-2.5913	0.015719	0.529492	Down	0.687784	0.115857
SUCNR1	196.7803	324.7119	68.84872	0.21203	-2.23766	0.000337	0.047633	Down	4.169887	0.897531
SYTL3	263.2421	373.8196	152.6645	0.408391	-1.29198	0.018349	0.586842	Down	3.739699	1.550388
TACC3	3202.892	2018.425	4387.36	2.173655	1.120123	0.002889	0.185459	Up	32.87002	72.53005
TAF9B	369.2123	238.523	499.9016	2.095822	1.067516	0.030197	0.767297	Up	4.762616	10.13275
TBC1D10C	34.54947	57.12525	11.97369	0.209604	-2.25426	0.04508	0.922121	Down	1.218572	0.259286
TBC1D16	1091.364	1486.259	696.4696	0.468606	-1.09355	0.005963	0.288333	Down	5.836289	2.776336
TBC1D30	524.91	940.0611	109.7588	0.116757	-3.09842	2.36E-10	5.77E-07	Down	5.567325	0.659869
TCF19	1823.349	1073.353	2573.346	2.397482	1.26152	0.00092	0.094528	Up	14.87995	36.21465
TDO2	63.58685	103.2263	23.94738	0.231989	-2.10787	0.020926	0.634288	Down	3.254483	0.766438
TEC	288.3146	431.947	144.6821	0.334953	-1.57797	0.003287	0.203551	Down	4.370062	1.485934
TEDC1	213.3128	128.2813	298.3444	2.325706	1.217668	0.037704	0.833968	Up	3.363098	7.940022
TGM5	196.7935	330.7251	62.86187	0.190073	-2.39538	0.000139	0.026231	Down	5.59633	1.079819
THBS4	133.3653	72.15821	194.5725	2.69647	1.431072	0.039041	0.849477	Up	0.963753	2.638087
THSD7A	1486.445	2374.205	598.6845	0.252162	-1.98758	4.85E-07	0.000285	Down	9.401493	2.406601
TIAM1	235.7453	347.7625	123.7281	0.355783	-1.49093	0.009017	0.379572	Down	1.873562	0.676677
TIFAB	217.8484	376.8262	58.87064	0.156228	-2.67828	1.38E-05	0.005023	Down	15.89348	2.520601
TK1	1027.645	639.4019	1415.889	2.214396	1.146913	0.004266	0.232056	Up	18.9253	42.54275
TMEM106(	1335.334	805.7666	1864.902	2.314445	1.210666	0.00189	0.146562	Up	24.73578	58.11657
TMEM255/	184.6662	260.5713	108.761	0.417394	-1.26052	0.040763	0.868015	Down	3.245299	1.375083
TNFAIP3	2663.244	4144.086	1182.402	0.285323	-1.80933	2.25E-06	0.001101	Down	25.22991	7.307679
TNFRSF10I	3579.422	4914.776	2244.069	0.456596	-1.13101	0.002681	0.178023	Down	63.52482	29.44445
TNFRSF9	22.04176	41.09009	2.993422	0.07285	-3.77892	0.00977	0.396368	Down	0.319877	0.023656

TNFSF13B	908.8033	1275.797	541.8095	0.424683	-1.23554	0.00254	0.172698	Down	25.64566	11.05621
TOP2A	12111.17	6963.267	17259.08	2.478589	1.309519	0.001393	0.124445	Up	63.63725	160.1192
TP53I3	71.09236	113.2483	28.93642	0.255513	-1.96853	0.024382	0.689985	Down	1.675987	0.434722
TP53INP1	400.1035	675.481	124.7259	0.184648	-2.43715	1.42E-06	0.000772	Down	6.320607	1.184759
TPM2	469.9741	686.5052	253.4431	0.369179	-1.43761	0.002091	0.152785	Down	15.97034	5.985196
TPM4	11316.29	15104.12	7528.458	0.498437	-1.00452	0.012837	0.473709	Down	139.1019	70.38355
TPX2	4376.112	2609.722	6142.503	2.3537	1.234931	0.001139	0.107245	Up	38.24238	91.37421
TRANK1	74.622	130.2857	18.95834	0.145514	-2.78077	0.001926	0.146562	Down	0.583618	0.08621
TRIB2	6.013184	12.02637	0	0	#NAME?	0.047509	0.927888	Down	0.146487	0
TRIM22	1638.146	2617.739	658.5529	0.251573	-1.99095	3.79E-07	0.000232	Down	45.47091	11.61249
TROAP	746.6271	349.7669	1143.487	3.269284	1.708975	6.69E-05	0.016333	Up	5.66331	18.79536
TSPOAP1	7518.779	4464.789	10572.77	2.368033	1.243689	0.001445	0.127041	Up	26.07937	62.69204
TTK	1579.553	922.0215	2237.084	2.426282	1.278747	0.000883	0.093252	Up	13.47069	33.17865
TUBA1B	23409.14	13247.04	33571.23	2.534243	1.341555	0.00303	0.190918	Up	405.7358	1043.804
TUBA4A	1287.203	697.5293	1876.876	2.690748	1.428007	0.000283	0.043075	Up	14.46002	39.49749
TUBB	44639.37	28455.39	60823.35	2.137499	1.095923	0.029045	0.757696	Up	339.0623	735.7205
TUBB4B	4946.075	2935.436	6956.714	2.369908	1.244831	0.001096	0.104358	Up	99.06235	238.3239
TXLNB	50.56707	81.17798	19.95615	0.245832	-2.02426	0.039012	0.849477	Down	0.777345	0.19399
TXNIP	2712.772	3978.723	1446.821	0.363639	-1.45942	0.000116	0.022326	Down	61.15773	22.57615
TYMP	347.442	549.2041	145.6799	0.265256	-1.91454	0.000195	0.034473	Down	14.45473	3.892272
TYROBP	229.2859	359.7888	98.78294	0.274558	-1.86482	0.001398	0.124445	Down	32.68635	9.110206
U2AF1L5	202.2244	304.668	99.78075	0.327507	-1.6104	0.0076	0.337214	Down	8.678041	2.885155
UBA7	834.1813	1145.512	522.8511	0.456435	-1.13152	0.006255	0.29538	Down	13.24953	6.139127
UBASH3B	2602.054	3540.763	1663.345	0.46977	-1.08997	0.003667	0.210637	Down	19.65156	9.371519
UBE2C	560.4219	297.6526	823.1912	2.765611	1.467598	0.001101	0.104358	Up	12.69376	35.63762
UPB1	82.60446	130.2857	34.92326	0.268051	-1.89942	0.022088	0.656698	Down	2.81839	0.766914
VAT1L	428.9916	653.4327	204.5505	0.31304	-1.67558	0.000499	0.064852	Down	9.254506	2.940902
VENTX	683.794	1046.294	321.294	0.307078	-1.70332	9.16E-05	0.018434	Down	21.63157	6.743175
VIM	12292.64	18066.61	6518.676	0.360813	-1.47067	0.00036	0.049358	Down	442.5296	162.0886
VWCE	40.05716	66.14502	13.9693	0.211192	-2.24337	0.03606	0.814849	Down	0.987881	0.211792
WDR27	2019.59	2746.021	1293.159	0.470921	-1.08644	0.00398	0.219742	Down	7.630982	3.648009
WDR62	1039.144	650.4261	1427.863	2.195273	1.1344	0.004631	0.244415	Up	6.995674	15.58998
WDR76	985.199	621.3623	1349.036	2.171093	1.118422	0.005565	0.273379	Up	8.231416	18.14181
YAP1	19.03297	34.07471	3.99123	0.117132	-3.0938	0.033711	0.796158	Down	0.29442	0.035008
YPEL5	574.4228	767.6831	381.1625	0.49651	-1.0101	0.02241	0.659713	Down	11.7464	5.920528
ZDHHC21	158.1452	224.4922	91.79829	0.408915	-1.29013	0.047409	0.927888	Down	0.772452	0.320651
ZFP36L1	111.1385	174.3823	47.89476	0.274654	-1.86431	0.012893	0.474574	Down	1.870328	0.521472
ZMAT3	869.7528	1213.661	525.8446	0.433271	-1.20666	0.003388	0.20365	Down	7.148242	3.144031
ZNF358	79.09457	122.2681	35.92107	0.293789	-1.76715	0.034882	0.807489	Down	3.130551	0.933651
ZNF367	400.6981	263.5779	537.8182	2.040453	1.028889	0.032563	0.78801	Up	2.917319	6.042797
ZNF467	128.1254	185.4065	70.84433	0.382103	-1.38797	0.048132	0.930146	Down	2.267085	0.879378
ZNF512B	37.05716	63.13843	10.97588	0.173838	-2.52418	0.023573	0.678595	Down	0.429658	0.075822
ZSCAN30	432.8424	589.292	276.3927	0.469025	-1.09226	0.020797	0.63369	Down	6.934039	3.301488
