# Supplementary material for: Repressing HIF-1α-induced HDAC9 contributes to the synergistic effect of venetoclax and MENIN inhibitor in KMT2Ar AML
Source: Biomark Res. 2023 Dec 5;11:105. doi: 10.1186/s40364-023-00547-9 (PMC10696732; doi:10.1186/s40364-023-00547-9)
Supplement: Supplementary file 6 — Additional file 6: Table S5. Different expressed genes of MI-503 vs. DMSO in MOLM13. [file 40364_2023_547_MOESM6_ESM.pdf]

gene_id	BaseMean	BaseMean_	BaseMean_	FoldChang	log2FoldCl	pValue	qValue	Regulation	Expression	Expression_MOLM13_MI_503
ADAM28	88.72228	122.8052	54.63939	0.444927	-1.16836	0.005886	0.368654	Down	0.865504	0.383753
ADAMTS1	37.09289	51.33659	22.8492	0.445086	-1.16784	0.04889	1	Down	0.456658	0.202548
ADAMTS2	90.27501	56.36958	124.1804	2.202969	1.139449	0.006839	0.400383	Up	0.432503	0.949492
AFF3	785.1817	511.3527	1059.011	2.070999	1.050327	8.34E-09	5.11E-06	Up	1.432732	2.956914
AGXT	32.63555	53.34979	11.92132	0.223456	-2.16194	0.000942	0.109849	Down	1.863517	0.414972
AKAP12	7.549498	15.099	0	0	#NAME?	0.001435	0.144898	Down	0.07666	0
ALDH1A3	34.61586	52.34319	16.88854	0.32265	-1.63196	0.00836	0.450782	Down	0.809788	0.260374
ANGPT1	8.54952	16.1056	0.993444	0.061683	-4.01898	0.005007	0.328621	Down	0.19982	0.012283
APOL4	30.11248	47.31019	12.91477	0.272981	-1.87313	0.004663	0.312527	Down	0.763011	0.207566
ARMC12	23.58273	36.23759	10.92788	0.301562	-1.72947	0.017016	0.722057	Down	0.689644	0.20725
BASP1	56.2677	21.13859	91.39681	4.323694	2.112264	6.65E-05	0.014445	Up	0.601081	2.589894
BBOF1	6.463961	1.0066	11.92132	11.84316	3.565982	0.023368	0.860296	Up	0.012114	0.142977
BPI	244.5614	102.6732	386.4495	3.76388	1.912221	1.41E-11	1.55E-08	Up	2.997413	11.24284
C19orf81	27.91509	15.099	40.73119	2.697609	1.431681	0.032345	1	Up	1.101702	2.961672
C4B	6.039598	12.0792	0	0	#NAME?	0.004854	0.320846	Down	0.124238	0
C8orf76	40.60941	57.37618	23.84265	0.41555	-1.26691	0.027236	0.93455	Down	2.386474	0.988265
CACNB4	351.715	233.5311	469.8988	2.012146	1.008735	1.60E-05	0.004102	Up	0.642578	1.288485
CAMKK1	160.385	219.4387	101.3312	0.461775	-1.11474	0.000697	0.086897	Down	1.407737	0.647807
CBSL	46.88261	29.19139	64.57383	2.212085	1.145407	0.034502	1	Up	0.404222	0.891077
CCL3	46.11282	63.41578	28.80986	0.454301	-1.13828	0.036644	1	Down	4.441366	2.010732
CCR2	214.6725	89.58738	339.7577	3.792473	1.923139	1.42E-10	1.20E-07	Up	0.957791	3.619819
CD1A	10.54298	17.1122	3.973774	0.232219	-2.10644	0.047811	1	Down	0.424711	0.098284
CD36	414.5519	271.7819	557.3218	2.050621	1.036061	2.47E-06	0.000842	Up	2.595455	5.30387
CD38	99.63379	44.29039	154.9772	3.499116	1.80699	1.47E-05	0.003974	Up	0.43418	1.513985
CD69	3.523099	7.046198	0	0	#NAME?	0.042601	1	Down	0.23467	0
CDH3	100.2555	63.41578	137.0952	2.161847	1.112264	0.005869	0.368654	Up	0.802123	1.728063
CHI3L1	2095.099	900.9068	3289.292	3.65109	1.868327	1.65E-30	1.18E-26	Up	27.37462	99.60119
CLCNKA	11.54958	19.12539	3.973774	0.207775	-2.26691	0.027716	0.94249	Down	0.420958	0.087162
COL4A1	25.40517	11.0726	39.73774	3.588837	1.843516	0.009255	0.480952	Up	0.039619	0.141693
COL7A1	49.64908	72.47518	26.82298	0.370099	-1.43402	0.007281	0.413652	Down	0.433132	0.159747
CR1	12.91477	0	25.82953	Inf	Inf	2.73E-05	0.006109	Up	0	0.141875
CST7	297.2368	182.1946	412.2791	2.262851	1.178141	2.84E-06	0.000938	Up	10.93528	24.65924
CUX2	25.41833	13.0858	37.75085	2.884873	1.528508	0.028396	0.957546	Up	0.04854	0.139547
CYP26B1	30.87569	12.0792	49.67218	4.112209	2.039913	0.002106	0.187646	Up	0.14135	0.57925
DACH1	2478.579	3334.865	1622.293	0.486464	-1.03959	3.51E-11	3.59E-08	Down	16.43101	7.965428
DBNDD1	99.22916	58.38278	140.0755	2.399261	1.26259	0.001937	0.179128	Up	1.146266	2.74067
DCANP1	402.0352	256.6829	547.3874	2.132543	1.092575	9.39E-07	0.000383	Up	4.570218	9.712444
DEPTOR	105.7655	70.46198	141.069	2.002058	1.001484	0.011002	0.531321	Up	0.492867	0.983331
DHRS3	85.30122	55.36298	115.2395	2.081525	1.057641	0.013863	0.619409	Up	1.380819	2.86426
DHRS9	939.9022	624.0918	1255.713	2.012064	1.008676	8.56E-09	5.11E-06	Up	15.23876	30.55521
DLX3	78.25166	116.7656	39.73774	0.340321	-1.55503	0.000579	0.075128	Down	2.504869	0.849507
DLX5	716.8836	1005.593	428.1742	0.425793	-1.23178	4.85E-11	4.63E-08	Down	17.75162	7.53234
DOC2A	12.43778	3.019799	21.85576	7.237487	2.855489	0.006477	0.389145	Up	0.038581	0.278263
EIF3CL	318.4446	159.0428	477.8463	3.004515	1.587132	1.78E-10	1.42E-07	Up	2.714836	8.128526
ESRRB	3.477052	0	6.954105	Inf	Inf	0.045456	1	Up	0	0.055522
FABP3	16.55627	25.16499	7.947548	0.315818	-1.66284	0.048134	1	Down	0.483036	0.152023
FAM156A	68.28112	35.23099	101.3312	2.876196	1.524162	0.001358	0.140162	Up	0.395761	1.134346
FCGR3A	3.973774	0	7.947548	Inf	Inf	0.029125	0.971472	Up	0	0.17135
FCGR3B	7.450827	0	14.90165	Inf	Inf	0.001622	0.157156	Up	0	0.30241
FGD6	75.16607	100.66	49.67218	0.493465	-1.01898	0.023764	0.868609	Down	0.60494	0.297483
FOSB	249.8342	377.4749	122.1936	0.323713	-1.62721	3.68E-09	2.40E-06	Down	5.579992	1.800063
FPR1	5.463939	0	10.92788	Inf	Inf	0.008108	0.440486	Up	0	0.443373
FYB1	220.8832	127.8382	313.9282	2.455669	1.296116	6.35E-06	0.001938	Up	1.123382	2.749103
G0S2	5.463939	0	10.92788	Inf	Inf	0.008108	0.440486	Up	0	0.63122
GAPT	31.87571	13.0858	50.66562	3.871803	1.953005	0.002719	0.220338	Up	0.287797	1.110435
GOLGA8Q	228.5904	319.0921	138.0887	0.432755	-1.20838	1.79E-05	0.004434	Down	9.37926	4.044868
GPA33	24.39857	9.059398	39.73774	4.386356	2.133023	0.003646	0.27099	Up	0.176257	0.770449
GPC3	53.86961	34.22439	73.51482	2.148024	1.10301	0.031629	1	Up	0.742749	1.58992
GPR141	9.457448	3.019799	15.8951	5.263627	2.396057	0.036954	1	Up	0.014462	0.075862
GTPBP6	279.7957	97.64017	461.9512	4.73116	2.242194	1.97E-16	4.71E-13	Up	1.499345	7.069082
GYS2	3.523099	7.046198	0	0	#NAME?	0.042601	1	Down	0.072048	0
HAL	54.27423	20.13199	88.41648	4.391839	2.134825	7.18E-05	0.01537	Up	0.252072	1.103226
HBA2	4.470496	0	8.940992	Inf	Inf	0.018855	0.75799	Up	0	0.822056
HBB	57.16905	7.046198	107.2919	15.22692	3.928552	2.49E-10	1.88E-07	Up	0.628287	9.533753
HECW1	16.93459	7.046198	26.82298	3.80673	1.928552	0.022555	0.8517	Up	0.032183	0.122088
HLA-DQB	33.41192	20.13199	46.69185	2.319286	1.213681	0.049899	1	Up	0.675322	1.560842
HLA-DRA	10.45089	3.019799	17.88198	5.92158	2.565982	0.020695	0.804402	Up	0.131688	0.7771
HUNK	130.641	76.50158	184.7805	2.415382	1.272251	0.000458	0.067098	Up	0.578226	1.3918
IFIT2	79.6892	108.7128	50.66562	0.46605	-1.10144	0.012591	0.579548	Down	1.736244	0.806376
IQCN	17.07273	28.18479	5.960661	0.211485	-2.24137	0.009057	0.477298	Down	0.353773	0.074559
ITGA3	5.52972	10.066	0.993444	0.098693	-3.34091	0.044739	1	Down	0.111592	0.010975
KBTBD13	6.03302	11.0726	0.993444	0.089721	-3.47841	0.03083	1	Down	0.197904	0.017695
KLHL29	47.88263	30.19799	65.56727	2.171246	1.118523	0.0373	1	Up	0.251846	0.544926
KRT17	11.04628	18.1188	3.973774	0.219318	-2.18891	0.036423	1	Down	0.642543	0.140433
KRT81	4.026399	8.052798	0	0	#NAME?	0.027086	0.933875	Down	0.235337	0
LOC10272	35.09285	49.32339	20.86231	0.42297	-1.24137	0.040804	1	Down	0.450082	0.189712
LOC10272	60.35652	114.7524	5.960661	0.051944	-4.26691	1.19E-11	1.44E-08	Down	4.103328	0.212404
LSP1	9.457448	3.019799	15.8951	5.263627	2.396057	0.036954	1	Up	0.047996	0.251756
M1AP	8.960726	3.019799	14.90165	4.93465	2.302948	0.049346	1	Up	0.047805	0.235084
MAP3K12	52.37286	33.21779	71.52794	2.153302	1.106551	0.03295	1	Up	0.28778	0.617531

MARCKS	442.0065	216.4189	667.5941	3.08473	1.625144	1.51E-13	2.40E-10	Up	2.805425	8.62402
MEF2C	4076.794	5503.081	2650.507	0.481641	-1.05397	1.21E-11	1.44E-08	Down	39.19526	18.81267
MGLL	89.73882	50.32999	129.1477	2.566018	1.359531	0.001396	0.141959	Up	0.475837	1.216778
MNDA	179.652	51.33659	307.9675	5.998987	2.584719	2.65E-14	4.74E-11	Up	1.622609	9.700307
MPO	395.0121	170.1154	619.9088	3.64405	1.865543	8.94E-16	1.83E-12	Up	2.953521	10.72551
MS4A3	290.7302	98.64677	482.8136	4.894368	2.291122	1.70E-17	6.10E-14	Up	3.26203	15.9103
MS4A6A	62.85665	41.27059	84.4427	2.046075	1.032859	0.032548	1	Up	0.632179	1.289006
MSRB3	870.483	566.7156	1174.25	2.072027	1.051043	3.67E-09	2.40E-06	Up	5.942733	12.27087
MUC8	24.07946	36.23759	11.92132	0.328977	-1.60394	0.024744	0.893951	Down	0.917335	0.300737
NAIP	65.29421	34.22439	96.36402	2.815653	1.49347	0.002	0.180439	Up	0.26861	0.753693
NAV3	76.14315	22.14519	130.1411	5.87672	2.555011	1.59E-07	7.61E-05	Up	0.062642	0.366853
NCAM2	323.7437	437.8709	209.6166	0.478718	-1.06275	1.12E-05	0.003209	Down	1.193829	0.569528
NCR3	6.03302	11.0726	0.993444	0.089721	-3.47841	0.03083	1	Down	0.489355	0.043753
NEUROG3	155.6416	101.6666	209.6166	2.061804	1.043907	0.001702	0.162711	Up	2.96183	6.085572
NR2F2	27.41837	15.099	39.73774	2.631814	1.396057	0.038064	1	Up	0.137938	0.361771
OCSTAMP	23.58931	37.24419	9.934435	0.266738	-1.90651	0.009179	0.47872	Down	1.047838	0.278531
OLIG1	116.2361	76.50158	155.9706	2.03879	1.027713	0.006671	0.393762	Up	1.873715	3.806885
PBX3	1228.385	1670.956	785.8138	0.470278	-1.08841	8.26E-11	7.41E-08	Down	29.44131	13.79767
PCDHGA1	23.57616	35.23099	11.92132	0.338376	-1.5633	0.029771	0.98873	Down	0.408589	0.137778
PCDHGB7	12.04631	19.12539	4.967218	0.259718	-1.94498	0.049176	1	Down	0.214972	0.055639
PCDHGC5	61.76774	102.6732	20.86231	0.203191	-2.29909	8.79E-06	0.002573	Down	1.189509	0.240861
PDYN	8.450848	1.0066	15.8951	15.79088	3.98102	0.00564	0.359502	Up	0.011839	0.186297
PICK1	4.026399	8.052798	0	0	#NAME?	0.027086	0.933875	Down	0.158608	0
PKDCC	27.60913	44.29039	10.92788	0.246733	-2.01898	0.003502	0.262678	Down	0.988492	0.243049
PLBD1	5.960661	0	11.92132	Inf	Inf	0.005375	0.345688	Up	0	0.343233
PLPP2	8.542941	15.099	1.986887	0.131591	-2.92587	0.020612	0.803375	Down	0.543743	0.071304
PMP22	46.64244	68.44878	24.83609	0.362842	-1.46259	0.007587	0.426743	Down	1.682388	0.608327
PNCK	45.6161	63.41578	27.81642	0.438636	-1.18891	0.029914	0.990908	Down	1.095904	0.479038
PORCN	33.89549	18.1188	49.67218	2.741472	1.454951	0.018919	0.75799	Up	0.371142	1.013953
PPARGC1	7.954126	1.0066	14.90165	14.80395	3.88791	0.008015	0.438785	Up	0.004704	0.069394
PRICKLE2	24.07288	35.23099	12.91477	0.366574	-1.44782	0.041499	1	Down	0.093556	0.034176
PRRT4	48.8695	29.19139	68.5476	2.348213	1.231563	0.021184	0.816766	Up	0.343469	0.803746
PRTN3	1720.854	1096.187	2345.52	2.139708	1.097414	9.13E-12	1.31E-08	Up	57.18451	121.9345
PSD3	34.40536	20.13199	48.67873	2.417979	1.273802	0.037616	1	Up	0.052408	0.126283
PSTPIP1	99.77193	65.42898	134.1149	2.049778	1.035468	0.010324	0.515973	Up	1.278761	2.612102
PTAFR	49.86294	29.19139	70.53449	2.416277	1.272786	0.016517	0.713563	Up	0.359457	0.86554
PTGIR	52.87616	34.22439	71.52794	2.08997	1.063482	0.039509	1	Up	0.683489	1.423525
PXYLP1	34.89551	19.12539	50.66562	2.649128	1.405518	0.021593	0.8237	Up	0.249253	0.658016
RNASE6	57.63609	78.51478	36.75741	0.468159	-1.09493	0.02844	0.957546	Down	3.804314	1.774858
ROPN1L	111.2097	67.44218	154.9772	2.297927	1.200333	0.001969	0.179128	Up	2.005602	4.592771
SELL	8.457426	2.013199	14.90165	7.401976	2.88791	0.022856	0.853713	Up	0.046017	0.339438
SEMA4A	89.76514	54.35639	125.1739	2.302837	1.203412	0.004446	0.301848	Up	0.823585	1.890016
SEMG1	32.59609	47.31019	17.88198	0.377973	-1.40364	0.025321	0.901657	Down	1.557063	0.586491
SEZ6L2	33.89549	18.1188	49.67218	2.741472	1.454951	0.018919	0.75799	Up	0.261604	0.714698
SFN	6.03302	11.0726	0.993444	0.089721	-3.47841	0.03083	1	Down	0.468223	0.041864
SH3TC1	111.2655	151.9966	70.53449	0.464053	-1.10764	0.004153	0.288854	Down	1.321322	0.611041
SIGLEC6	20.43137	10.066	30.79675	3.059483	1.613288	0.035359	1	Up	0.121564	0.370635
SIK1	11.54958	19.12539	3.973774	0.207775	-2.26691	0.027716	0.94249	Down	0.225174	0.046623
SIRPB2	123.1573	71.46858	174.8461	2.446475	1.290704	0.000533	0.073227	Up	0.800414	1.951413
SLA2	31.9086	18.1188	45.6984	2.522155	1.334657	0.034771	1	Up	0.312342	0.785047
SLC15A2	169.5366	99.65337	239.4199	2.402527	1.264552	8.84E-05	0.018366	Up	0.921706	2.206756
SLC1A3	99.70615	55.36298	144.0493	2.601907	1.379569	0.000729	0.090105	Up	0.61133	1.585117
SORL1	622.346	296.9469	947.7451	3.191631	1.674294	2.88E-17	8.27E-14	Up	1.279437	4.069354
SPARC	5.470518	1.0066	9.934435	9.869301	3.302948	0.048374	1	Up	0.015651	0.153929
SPIB	87.71568	120.792	54.63939	0.452343	-1.14451	0.007193	0.41191	Down	1.909492	0.860755
SPP1	39.89562	24.15839	55.63284	2.302837	1.203412	0.037113	1	Up	0.743376	1.705946
SRPK3	175.4577	245.6103	105.305	0.428748	-1.2218	0.000116	0.022762	Down	6.847941	2.925878
STXBP1	144.1249	87.57418	200.6756	2.291493	1.196288	0.000541	0.073227	Up	1.232229	2.813868
SYNE1	14.43782	5.032999	23.84265	4.737264	2.244054	0.015626	0.681231	Up	0.009766	0.046103
SYNPO2L	3.523099	7.046198	0	0	#NAME?	0.042601	1	Down	0.069984	0
TFAP2C	52.82354	26.17159	79.47548	3.036708	1.602508	0.002331	0.201404	Up	0.50972	1.542511
TGFB2	16.92801	6.039598	27.81642	4.605674	2.203412	0.010515	0.521872	Up	0.056149	0.25771
THBS4	59.32697	33.21779	85.43614	2.572	1.36289	0.00626	0.38373	Up	0.461235	1.182189
TIFAB	190.5239	118.7788	262.2691	2.208047	1.142771	0.000178	0.031168	Up	5.208203	11.46014
TMEM238	3.477052	0	6.954105	Inf	Inf	0.045456	1	Up	0	0.584325
TMEM269	11.94106	3.019799	20.86231	6.908511	2.788375	0.00866	0.463452	Up	0.050257	0.345996
TNFRSF14	68.32716	42.27719	94.37714	2.232342	1.158558	0.013563	0.611761	Up	0.49535	1.101961
TNFRSF19	112.0716	47.31019	176.833	3.737735	1.902164	1.92E-06	0.000671	Up	0.428281	1.595258
TNS4	3.523099	7.046198	0	0	#NAME?	0.042601	1	Down	0.08715	0
TSPOAP1	7196.985	3645.904	10748.07	2.947984	1.559728	2.44E-23	1.17E-19	Up	22.13975	65.04166
UNC5B	146.8354	198.3001	95.37058	0.480941	-1.05607	0.001973	0.179128	Down	1.409859	0.675711
VAMP7	328.6752	52.34319	605.0071	11.55847	3.530878	8.23E-36	1.18E-31	Up	1.090192	12.55732
VCAN	15.41811	3.019799	27.81642	9.211347	3.203412	0.001138	0.124613	Up	0.013576	0.124621
XAGE1A	89.24531	126.8316	51.65906	0.407304	-1.29582	0.002297	0.199681	Down	7.366844	2.99016
ZBTB16	12.94766	5.032999	20.86231	4.145106	2.051409	0.033235	1	Up	0.022126	0.091397
