# Supplementary material for: Repressing HIF-1α-induced HDAC9 contributes to the synergistic effect of venetoclax and MENIN inhibitor in KMT2Ar AML
Source: Biomark Res. 2023 Dec 5;11:105. doi: 10.1186/s40364-023-00547-9 (PMC10696732; doi:10.1186/s40364-023-00547-9)
Supplement: Supplementary file 7 — Additional file 7: Table S6. Different expressed genes of VEN vs. DMSO in MOLM13. [file 40364_2023_547_MOESM7_ESM.pdf]

gene_id	BaseMean	BaseMean_	BaseMean_	FoldChang	log2FoldCl	pValue	qValue	Regulation	Expression	Expression_MOLM13_Venetoclax
ABTB2	14.91617	2.012987	27.81935	13.81994	3.788679	0.003191	0.237751	Up	0.022919	0.316007
ACTA2	357.5722	215.3896	499.7548	2.320236	1.214272	0.000787	0.090402	Up	6.28869	14.55732
ACVR2B	227.9934	304.9676	151.0193	0.495198	-1.01392	0.015245	0.614036	Down	0.962277	0.47541
ACVRL1	87.22027	44.28572	130.1548	2.93898	1.555315	0.009018	0.454974	Up	0.551219	1.616256
ADAM28	266.5638	122.7922	410.3355	3.341706	1.740585	1.73E-05	0.005398	Up	0.865504	2.885533
ADAMDEC	19.87744	1.006494	38.74838	38.49839	5.266726	0.000103	0.019215	Up	0.023046	0.885181
ADAMTS5	122.6272	65.42208	179.8322	2.7488	1.458802	0.005833	0.356539	Up	0.365507	1.002368
AK4	103.384	163.052	43.71613	0.268112	-1.89909	0.000883	0.096925	Down	1.202254	0.321589
AKAP12	8.045476	15.0974	0.993548	0.065809	-3.92557	0.020991	0.691474	Down	0.07666	0.005033
ALDOC	999.495	1465.455	533.5354	0.364075	-1.45769	5.79E-07	0.000364	Down	47.09728	17.10707
ANKRD36	346.8811	484.1234	209.6387	0.433027	-1.20747	0.000936	0.101947	Down	2.765642	1.194814
ANXA1	556.6443	271.7533	841.5354	3.096689	1.630727	5.82E-07	0.000364	Up	8.44679	26.09626
APLN	19.07807	31.2013	6.954838	0.222902	-2.16552	0.0323	0.845966	Down	0.540258	0.120145
APOBEC3H	8.95488	2.012987	15.89677	7.897106	2.981324	0.042442	0.997173	Up	0.09475	0.746512
AQP9	4.470967	0	8.941935	Inf	Inf	0.036296	0.902932	Up	0	0.162697
ARHGAP33	451.8201	657.2403	246.4	0.374901	-1.41542	3.22E-05	0.008515	Down	4.497975	1.682374
ARHGEF3	287.6678	160.0325	415.3032	2.595118	1.3758	0.000412	0.059906	Up	1.373359	3.555742
ASB2	27.84524	4.025974	51.66451	12.8328	3.681764	0.000237	0.040142	Up	0.062051	0.794431
ASMTL	405.5554	647.1754	163.9355	0.253309	-1.98103	3.23E-08	3.57E-05	Down	13.74738	3.47424
BASP1	56.76818	21.13637	92.39999	4.371612	2.128165	0.002386	0.196055	Up	0.601081	2.621585
BAX	1468.592	869.6104	2067.574	2.377586	1.249498	7.29E-06	0.002758	Up	39.08647	92.71537
BBOF1	6.464537	1.006494	11.92258	11.84566	3.566287	0.049568	1	Up	0.012114	0.14317
BCL2A1	72.65201	19.12338	126.1806	6.59824	2.722081	5.12E-05	0.01194	Up	1.132078	7.45235
BCL3	107.1365	51.33117	162.9419	3.174327	1.666451	0.002865	0.226361	Up	0.534414	1.692459
BEX1	3926.026	2474.968	5377.084	2.172587	1.119414	3.23E-05	0.008515	Up	160.2823	347.4176
BNIP3	905.7051	1326.559	484.8516	0.365496	-1.45207	8.99E-07	0.000461	Down	45.34856	16.53617
BTG2	848.788	355.2922	1342.284	3.777971	1.917612	2.66E-10	3.82E-07	Up	7.3134	27.56556
C1QTNF3	207.0448	291.8831	122.2064	0.418683	-1.25607	0.003885	0.279295	Down	4.258801	1.77894
C21orf58	1186.104	1592.273	779.9354	0.489825	-1.02966	0.000269	0.043204	Down	12.93286	6.320109
C2orf48	156.4952	233.5065	79.48387	0.340393	-1.55473	0.001384	0.135362	Down	6.85349	2.327452
C3	468.2266	41.26624	895.187	21.69297	4.439155	3.12E-28	1.12E-24	Up	0.448881	9.714923
C3AR1	66.22631	24.15585	108.2968	4.483253	2.164546	0.001207	0.12233	Up	0.374267	1.674031
C4B	6.535736	12.07792	0.993548	0.082262	-3.60364	0.046299	1	Down	0.124238	0.010196
CA2	74.70545	106.6883	42.72258	0.400443	-1.32033	0.033366	0.861336	Down	3.57491	1.428219
CBSL	57.31673	29.18831	85.44516	2.927376	1.549608	0.022685	0.702998	Up	0.404222	1.180557
CCDC15	175.3791	234.513	116.2452	0.495687	-1.0125	0.027496	0.772523	Down	2.338607	1.156523
CCDC170	4.470967	0	8.941935	Inf	Inf	0.036296	0.902932	Up	0	0.091045
CCL3	167.3239	63.4091	271.2387	4.277599	2.096801	1.61E-05	0.005368	Up	4.441366	18.9542
CCL3L3	194.8665	97.62988	292.1032	2.991945	1.581084	0.000437	0.062797	Up	7.041498	21.01878
CCL4	10.43226	0	20.86451	Inf	Inf	0.001166	0.120619	Up	0	1.742187
CCL4L2	23.89047	7.045455	40.73548	5.78181	2.531521	0.008155	0.435808	Up	0.21329	1.230337
CD101	53.30371	23.14935	83.45806	3.605201	1.85008	0.008647	0.444064	Up	0.183097	0.658565
CD14	12.94202	4.025974	21.85806	5.42926	2.440756	0.042186	0.997173	Up	0.124445	0.674072
CD209	16.43238	6.038961	26.8258	4.442122	2.151249	0.044664	1	Up	0.077339	0.342751
CD36	526.3411	271.7533	780.929	2.873669	1.522894	3.73E-06	0.001579	Up	2.595455	7.441136
CD38	100.1364	44.28572	155.9871	3.522289	1.816513	0.001596	0.150964	Up	0.43418	1.52575
CD48	36.34866	13.08442	59.6129	4.556023	2.187775	0.006997	0.400819	Up	0.365946	1.663382
CD52	111.6528	58.37663	164.929	2.825258	1.498383	0.006205	0.365644	Up	6.453137	18.18938
CD82	193.7888	84.54546	303.0322	3.584252	1.841672	5.23E-05	0.01194	Up	1.939865	6.936797
CD83	650.7224	300.9416	1000.503	3.324576	1.73317	4.62E-08	4.43E-05	Up	6.661321	22.09457
CD86	62.723	20.12987	105.3161	5.231833	2.387316	0.000533	0.072257	Up	0.384315	2.005994
CDKN1A	7390.817	2674.253	12107.38	4.527387	2.178679	6.75E-15	1.39E-11	Up	56.61294	255.7127
CFH	34.36803	14.09091	54.64516	3.878043	1.955329	0.016515	0.634211	Up	0.178939	0.692319
CFP	66.20042	20.12987	112.271	5.577331	2.479575	0.000274	0.043204	Up	0.408631	2.273772
CHI3L1	2749.477	900.8117	4598.142	5.104442	2.351753	6.05E-17	1.45E-13	Up	27.37462	139.4073
CHRFAM7A	226.987	302.9546	151.0193	0.498488	-1.00437	0.01638	0.63315	Down	4.495541	2.235764
CKAP4	661.4799	428.7663	894.1935	2.085503	1.060396	0.000616	0.079137	Up	7.883918	16.40371
CLEC10A	6.458064	0	12.91613	Inf	Inf	0.01051	0.491059	Up	0	0.398315
CNR2	187.1819	61.39611	312.9677	5.097517	2.349795	6.71E-07	0.000386	Up	0.919612	4.676839
COL6A3	495.6059	281.8182	709.3935	2.517203	1.331821	6.00E-05	0.013062	Up	1.386228	3.481303
COL9A2	109.0201	35.22728	182.8129	5.189527	2.375603	3.65E-05	0.009208	Up	0.455323	2.357414
CR1	12.91613	0	25.83226	Inf	Inf	0.000332	0.049238	Up	0	0.142067
CXCL10	310.0583	11.07143	609.0451	55.01052	5.781636	4.25E-29	2.04E-25	Up	0.508269	27.89511
CXCL8	533.317	43.27922	1023.355	23.6454	4.563488	3.78E-31	2.72E-27	Up	1.255734	29.6233
CYFIP2	200.6531	70.45455	330.8516	4.695958	2.231419	1.11E-06	0.000533	Up	0.548776	2.571035
CYGB	12.43877	3.019481	21.85806	7.239014	2.855793	0.023663	0.717838	Up	0.042771	0.3089
CYP1B1	196.8019	89.57793	304.0258	3.393981	1.762978	9.34E-05	0.017675	Up	0.970242	3.285324
CYP2W1	4.025974	8.051949	0	0	#NAME?	0.047851	1	Down	0.149037	0
CYTIP	198.5187	124.8052	272.2322	2.181257	1.12516	0.010604	0.491059	Up	1.553437	3.380565
DERL3	62.17606	89.57793	34.77419	0.3882	-1.36513	0.038149	0.931312	Down	1.275995	0.49419
DES	10.94845	3.019481	18.87742	6.251876	2.644289	0.043198	1	Up	0.074982	0.46769
DHRS3	35.62696	55.35715	15.89677	0.287167	-1.80004	0.02431	0.723963	Down	1.380819	0.395604
DLX2	122.1886	74.48052	169.8968	2.28109	1.189723	0.023604	0.717556	Up	1.804611	4.106907
DLX3	79.24114	116.7533	41.72903	0.357412	-1.48434	0.015318	0.614079	Down	2.504869	0.893189
DNAJB2	147.0986	85.55195	208.6451	2.438812	1.286179	0.00909	0.45543	Up	1.182441	2.877045
DTX4	103.1624	51.33117	154.9935	3.019482	1.594301	0.004739	0.319886	Up	0.45013	1.355998
DUSP1	671.9251	430.7792	913.0709	2.11958	1.083778	0.000451	0.063283	Up	11.8814	25.12501
DYNC1I1	222.071	311.0065	133.1355	0.428079	-1.22405	0.003881	0.279295	Down	2.725545	1.164036
DYNLT3	42.34878	19.12338	65.57419	3.429007	1.777791	0.018639	0.653633	Up	0.481746	1.648069
EBI3	19.37419	0	38.74838	Inf	Inf	1.68E-05	0.005398	Up	0	1.543685
EMP1	38.86489	18.11688	59.6129	3.290461	1.71829	0.026585	0.756966	Up	0.331594	1.088561
ENO2	136.5012	214.3831	58.61935	0.273433	-1.87074	0.0003	0.04587	Down	4.939244	1.34741
ETV1	73.70543	105.6818	41.72903	0.394855	-1.3406	0.031562	0.83069	Down	0.738375	0.290873
FADS2	3806.282	5175.39	2437.174	0.470916	-1.08646	5.42E-05	0.012181	Down	75.00325	35.2381
FAM117A	55.32317	28.18182	82.46451	2.92616	1.549009	0.024289	0.723963	Up	0.464903	1.357216
FAM57B	8.54225	15.0974	1.987097	0.131618	-2.92557	0.049461	1	Down	0.218569	0.028701

FAT1	179.3759	83.53897	275.2129	3.294425	1.720027	0.000222	0.038476	Up	0.307619	1.011071
FCER1G	175.4341	88.57143	262.2968	2.961415	1.566287	0.000792	0.090402	Up	8.366224	24.71825
FCGR2B	890.4054	1187.662	593.1483	0.499425	-1.00166	0.000625	0.079529	Down	19.67956	9.805609
FCGR3B	7.451612	0	14.90322	Inf	Inf	0.005896	0.357698	Up	0	0.302819
FDXR	293.0029	139.9026	446.1032	3.18867	1.672955	1.91E-05	0.005829	Up	2.204959	7.014537
FGF9	33.1172	51.33117	14.90322	0.290335	-1.78421	0.029188	0.793378	Down	0.576217	0.166907
FGL2	74.74914	36.23377	113.2645	3.125938	1.644289	0.008798	0.447518	Up	0.473928	1.478025
FGR	27.87113	8.051949	47.69032	5.92283	2.566287	0.004761	0.319904	Up	0.138136	0.816253
FOSB	605.5111	377.4351	833.587	2.208557	1.143104	0.000294	0.045514	Up	5.579992	12.29508
FOXA1	100.2351	136.8831	63.58709	0.464536	-1.10614	0.048881	1	Down	1.835993	0.850901
FPR1	5.96129	0	11.92258	Inf	Inf	0.014162	0.580162	Up	0	0.484334
FTH1	8726.181	5615.228	11837.13	2.108042	1.075903	7.70E-05	0.014956	Up	255.0579	536.4224
FUCA1	368.2488	176.1364	560.3613	3.181406	1.669664	4.48E-06	0.001841	Up	2.914253	9.249862
FYB1	277.0285	127.8247	426.2322	3.334506	1.737473	1.38E-05	0.004727	Up	1.123382	3.737215
G0S2	9.935483	0	19.87097	Inf	Inf	0.001513	0.145009	Up	0	1.149225
GADD45A	169.0537	100.6494	237.458	2.35926	1.238335	0.008159	0.435808	Up	4.071507	9.58341
GAPT	32.37446	13.08442	51.66451	3.948553	1.981324	0.017386	0.645988	Up	0.287797	1.133739
GAS7	2760.681	1716.072	3805.29	2.217443	1.148897	2.13E-05	0.006237	Up	10.24473	22.66428
GDF15	14.92912	4.025974	25.83226	6.416399	2.681764	0.020255	0.680556	Up	0.130667	0.836462
GJC2	4.967742	0	9.935483	Inf	Inf	0.026292	0.753091	Up	0	0.232403
GPR183	433.0107	204.3182	661.7032	3.238592	1.695367	1.09E-06	0.000533	Up	5.310019	17.15699
GPR68	49.71625	6.038961	93.39354	15.46517	3.95095	2.70E-06	0.001214	Up	0.054656	0.843305
GPRC5A	4.025974	8.051949	0	0	#NAME?	0.047851	1	Down	0.157662	0
GRIN2C	18.41301	5.032468	31.79355	6.317685	2.659396	0.01233	0.53751	Up	0.033078	0.208492
HBB	43.26466	7.045455	79.48387	11.28158	3.495897	3.59E-05	0.009208	Up	0.628287	7.071587
HILPDA	307.2411	422.7273	191.7548	0.453614	-1.14046	0.002587	0.209005	Down	16.71279	7.563518
HIST1H3H	6.961311	1.006494	12.91613	12.8328	3.681764	0.038088	0.931312	Up	0.118788	1.52084
HJURP	986.5061	1376.883	596.129	0.432955	-1.20771	3.14E-05	0.008515	Down	24.10271	10.41113
HK3	14.43234	4.025974	24.83871	6.169614	2.62518	0.024311	0.723963	Up	0.06243	0.384272
HLA-DRA	11.44522	3.019481	19.87097	6.580922	2.71829	0.035307	0.890105	Up	0.131688	0.864612
HLA-DRB1	4.470967	0	8.941935	Inf	Inf	0.036296	0.902932	Up	0	0.409217
HMOX1	65.71659	22.14286	109.2903	4.935691	2.303252	0.000652	0.081523	Up	0.777427	3.828219
HTR7	178.5183	104.6753	252.3613	2.410895	1.269569	0.005709	0.353844	Up	1.062828	2.556408
ID3	13.56177	23.14935	3.974193	0.171676	-2.54224	0.031975	0.839001	Down	1.043016	0.178645
IL18R1	51.1823	79.51299	22.85161	0.287395	-1.79889	0.011457	0.514821	Down	1.056344	0.302882
IL1B	81.09717	19.12338	143.071	7.481469	2.903322	9.89E-06	0.003556	Up	0.682576	5.094798
IL21R	90.72358	48.31169	133.1355	2.755761	1.462451	0.012604	0.542645	Up	0.512634	1.409411
IL22RA2	5.464516	0	10.92903	Inf	Inf	0.019219	0.661141	Up	0	0.210109
IL32	20.90982	7.045455	34.77419	4.935691	2.303252	0.019738	0.677345	Up	0.254898	1.255172
IL3RA	77.13592	21.13637	133.1355	6.298882	2.655096	5.22E-05	0.01194	Up	0.673472	4.232256
IL4I1	204.2777	16.1039	392.4516	24.36998	4.607033	1.53E-17	4.40E-14	Up	0.359883	8.749956
IL7R	98.14283	43.27922	153.0064	3.535332	1.821846	0.001667	0.155627	Up	0.522272	1.842113
INSIG1	2861.185	3903.182	1819.187	0.466078	-1.10136	4.47E-05	0.011088	Down	68.45504	31.83119
IPCEF1	472.2639	282.8247	661.7032	2.339623	1.226276	0.00025	0.041612	Up	2.270745	5.300334
IQGAP3	1431.117	1917.37	944.8645	0.492792	-1.02095	0.000236	0.040142	Down	15.92085	7.82742
ITGAX	571.6204	360.3247	782.9161	2.172807	1.11956	0.000457	0.063283	Up	4.016548	8.706891
ITGB7	263.6285	129.8377	397.4193	3.060894	1.613953	6.62E-05	0.01371	Up	2.392111	7.304973
JCHAIN	5.96129	0	11.92258	Inf	Inf	0.014162	0.580162	Up	0	0.471272
JPH2	170.9275	237.5325	104.3226	0.439193	-1.18707	0.010759	0.494238	Down	1.804584	0.790718
JUN	323.332	143.9286	502.7354	3.492951	1.804446	2.17E-06	0.001008	Up	2.417909	8.425999
KAZALD1	232.0194	313.0195	151.0193	0.48246	-1.05152	0.011403	0.513987	Down	4.827098	2.323466
KREMEN1	165.4662	83.53897	247.3935	2.961415	1.566287	0.00102	0.108674	Up	0.491983	1.453577
LFNG	104.2077	59.38312	149.0322	2.509674	1.3275	0.017263	0.645988	Up	1.050387	2.629998
LILRB1	76.28477	43.27922	109.2903	2.525237	1.336419	0.030325	0.812011	Up	0.600406	1.512641
LILRB2	20.40658	6.038961	34.77419	5.758307	2.525645	0.012605	0.542645	Up	0.111666	0.641514
LILRB3	6.464537	1.006494	11.92258	11.84566	3.566287	0.049568	1	Up	0.017865	0.211136
LIMD2	1774.243	1122.24	2426.245	2.161966	1.112344	4.95E-05	0.01194	Up	17.43135	37.59835
LIMS4	12.56822	23.14935	1.987097	0.085838	-3.54224	0.008211	0.435808	Down	0.310947	0.026629
LOC10013	641.9018	860.552	423.2516	0.491837	-1.02375	0.001002	0.107474	Down	4.575647	2.245241
LOC10272	29.13006	49.31819	8.941935	0.181311	-2.46346	0.005524	0.349885	Down	0.450082	0.081415
LOC10272	61.34433	114.7403	7.948387	0.069273	-3.85157	7.02E-07	0.000388	Down	4.103328	0.283588
LOC10272	14.43234	4.025974	24.83871	6.169614	2.62518	0.024311	0.723963	Up	0.035471	0.218336
LPXN	1048.27	552.565	1543.974	2.794195	1.482433	3.24E-07	0.000233	Up	5.551935	15.47711
LTB	126.4913	48.31169	204.671	4.236468	2.082862	0.000107	0.019642	Up	3.016741	12.75061
MAP1LC3A	4.967742	0	9.935483	Inf	Inf	0.026292	0.753091	Up	0	0.368655
MDGA1	260.1754	365.3572	154.9935	0.424225	-1.2371	0.002046	0.178273	Down	2.114432	0.894908
MDM2	3602.629	2320.974	4884.284	2.104411	1.073416	6.66E-05	0.01371	Up	16.24051	34.09723
MEI1	19.4195	7.045455	31.79355	4.512632	2.173969	0.030847	0.819877	Up	0.082248	0.37029
MFAP4	710.0667	1031.656	388.4774	0.376557	-1.40906	4.93E-06	0.001967	Down	29.36842	11.03317
MKI67	14501.48	20185.23	8817.741	0.436841	-1.19482	2.10E-05	0.006237	Down	85.301	37.17635
MLPH	53.21956	10.06494	96.37419	9.575241	3.259309	2.55E-05	0.007331	Up	0.082945	0.79237
MMP9	62.63238	6.038961	119.2258	19.74277	4.303252	9.20E-08	7.35E-05	Up	0.144315	2.84256
MNDA	118.0656	51.33117	184.8	3.600151	1.848058	0.00069	0.085516	Up	1.622609	5.828053
MPEG1	35.84541	12.07792	59.6129	4.935691	2.303252	0.005009	0.327392	Up	0.149433	0.73584
MTMR7	8.948408	1.006494	16.89032	16.78135	4.068787	0.013802	0.573601	Up	0.007553	0.126455
MVP	728.5832	434.8052	1022.361	2.351309	1.233464	5.02E-05	0.01194	Up	8.298358	19.46663
MYO15A	5.032468	10.06494	0	0	#NAME?	0.024599	0.725979	Down	0.040347	0
MYO1A	11.44522	3.019481	19.87097	6.580922	2.71829	0.035307	0.890105	Up	0.044405	0.291545
NAV3	57.7682	22.14286	93.39354	4.217773	2.076481	0.002803	0.222695	Up	0.062642	0.263594
NBDY	172.125	114.7403	229.5097	2.000254	1.000183	0.030568	0.815474	Up	1.044739	2.084884
NCAPG	1931.416	2619.903	1242.929	0.474418	-1.07577	7.96E-05	0.015267	Down	30.73214	14.54598
NCF1	657.2322	308.9935	1005.471	3.254019	1.702223	7.27E-08	6.54E-05	Up	11.82272	38.3819
NDRG1	948.1428	1282.273	614.0129	0.478847	-1.06236	0.000257	0.041959	Down	13.60357	6.498885
NFAM1	710.1508	426.7533	993.5483	2.328156	1.219188	7.09E-05	0.013961	Up	3.727617	8.658296
NFE2	634.112	421.7208	846.5032	2.00726	1.005227	0.001266	0.127289	Up	11.02682	22.08223
NFKB2	893.5122	434.8052	1352.219	3.109943	1.636888	3.97E-08	4.07E-05	Up	4.996438	15.5025
NFKBIA	779.1959	425.7468	1132.645	2.660373	1.411628	3.01E-06	0.001312	Up	15.16721	40.2566

NFKBIZ	219.325	115.7468	322.9032	2.789739	1.48013	0.000566	0.075148	Up	1.539548	4.284949
NOD2	164.6022	103.6688	225.5355	2.175538	1.121372	0.017339	0.645988	Up	0.711313	1.543889
OBSCN	230.7556	348.2468	113.2645	0.325242	-1.62041	0.000131	0.023515	Down	0.502797	0.16315
ODF3L1	4.470967	0	8.941935	Inf	Inf	0.036296	0.902932	Up	0	0.213925
OLIG1	122.2016	76.49351	167.9097	2.195084	1.134276	0.030702	0.817533	Up	1.873715	4.103398
OLIG2	172.0861	108.7013	235.471	2.16622	1.11518	0.016159	0.627984	Up	2.207412	4.77062
OPTN	569.7563	379.4481	760.0645	2.003079	1.002219	0.001667	0.155627	Up	5.888914	11.76853
OSM	86.27203	51.33117	121.2129	2.36139	1.239636	0.036208	0.902932	Up	1.353579	3.188895
P2RY4	4.025974	8.051949	0	0	#NAME?	0.047851	1	Down	0.274249	0
P4HA1	1863.136	2508.182	1218.09	0.485647	-1.04202	0.000135	0.023912	Down	46.4403	22.50113
PCDH12	40.3358	15.0974	65.57419	4.343408	2.118828	0.006671	0.385234	Up	0.128437	0.556556
PDK1	664.967	893.7663	436.1677	0.488011	-1.03501	0.000814	0.091694	Down	8.641129	4.207159
PFKFB4	290.6808	474.0585	107.3032	0.22635	-2.14337	8.63E-08	7.30E-05	Down	4.361955	0.985033
PHLDA3	547.5034	318.052	776.9548	2.442855	1.288568	6.94E-05	0.013862	Up	6.513217	15.87384
PICK1	4.025974	8.051949	0	0	#NAME?	0.047851	1	Down	0.158608	0
PINK1	193.0477	123.7987	262.2968	2.118736	1.083204	0.014777	0.601906	Up	2.598111	5.491911
PKDCC	27.60737	44.28572	10.92903	0.246785	-2.01868	0.021744	0.694351	Down	0.988492	0.243377
PLA2G4C	21.38071	3.019481	39.74193	13.16184	3.71829	0.000784	0.090402	Up	0.04192	0.550462
PLAU	2826.496	1521.818	4131.174	2.71463	1.440756	1.20E-07	9.10E-05	Up	30.26521	81.9678
PLCXD1	233.1036	327.1104	139.0968	0.425229	-1.23369	0.003077	0.235321	Down	3.339559	1.416775
PLK2	123.4848	44.28572	202.6839	4.576732	2.194318	5.71E-05	0.01264	Up	0.777183	3.548687
PNCK	36.67229	63.4091	9.935483	0.156689	-2.67403	0.001352	0.133142	Down	1.095904	0.171316
PORCN	47.31005	18.11688	76.50322	4.222758	2.078186	0.005001	0.327392	Up	0.371142	1.563599
POU3F2	238.5357	322.0779	154.9935	0.48123	-1.0552	0.010358	0.486746	Down	4.39926	2.112133
PPP2R3B	75.22164	109.7078	40.73548	0.371309	-1.42931	0.021399	0.691474	Down	1.765454	0.654005
PRICKLE2	21.09106	35.22728	6.954838	0.197428	-2.3406	0.017589	0.649064	Down	0.093556	0.018428
PROCR	133.1565	80.51949	185.7935	2.307436	1.20629	0.018009	0.653633	Up	2.843102	6.54502
PSRC1	229.555	316.039	143.071	0.4527	-1.14337	0.00624	0.366199	Down	7.65408	3.456948
PTAFR	56.32319	29.18831	83.45806	2.859297	1.515661	0.026475	0.755336	Up	0.359457	1.025404
PTGER4	867.0182	563.6364	1170.4	2.076516	1.054165	0.000342	0.050207	Up	7.616705	15.77943
PTPRO	73.74912	35.22728	112.271	3.187046	1.67222	0.008049	0.435105	Up	0.107438	0.341612
PTX3	123.1175	64.41559	181.8193	2.822598	1.497024	0.004662	0.31767	Up	1.855498	5.225147
QPCT	38.3487	15.0974	61.6	4.080171	2.02863	0.01023	0.48611	Up	0.494893	2.014551
RBM47	171.447	86.55845	256.3355	2.961415	1.566287	0.000876	0.096898	Up	0.47541	1.404612
RELB	199.1433	67.43507	330.8516	4.906225	2.294613	6.42E-07	0.000384	Up	1.651828	8.085393
RGS1	212.532	140.9091	284.1548	2.016582	1.011912	0.018212	0.653633	Up	5.606666	11.28001
RGS18	101.2335	60.38961	142.0774	2.35268	1.234305	0.027954	0.781633	Up	0.955287	2.242258
RGS20	34.85834	13.08442	56.63225	4.328222	2.113774	0.009842	0.478906	Up	0.257193	1.1106
RGS8	4.967742	0	9.935483	Inf	Inf	0.026292	0.753091	Up	0	0.066517
ROGDI	116.6918	69.44806	163.9355	2.360548	1.239122	0.02052	0.683001	Up	2.200278	5.181784
RPS10	94.72367	52.33767	137.1097	2.619713	1.389409	0.015979	0.624629	Up	3.320129	8.67756
RPS27L	1224.139	786.0715	1662.206	2.114574	1.080367	0.000128	0.023295	Up	40.48145	85.40197
S100A12	6.961311	1.006494	12.91613	12.8328	3.681764	0.038088	0.931312	Up	0.120573	1.543685
S100A3	38.839	14.09091	63.58709	4.512632	2.173969	0.006129	0.364368	Up	1.065874	4.798714
S100A8	173.214	52.33767	294.0903	5.619095	2.490338	3.80E-07	0.00026	Up	4.024399	22.56089
S100A9	340.5897	123.7987	557.3806	4.502314	2.170667	1.22E-08	1.46E-05	Up	11.97743	53.80074
SAPCD1	346.7516	463.9935	229.5097	0.49464	-1.01555	0.005177	0.336802	Down	28.30832	13.96986
SAT1	608.6584	326.1039	891.2128	2.732911	1.450438	5.16E-06	0.002007	Up	6.940344	18.92324
SCD	47224.21	63864.03	30584.4	0.478899	-1.06221	0.00057	0.075148	Down	652.0053	311.5184
SCIMP	26.4067	12.07792	40.73548	3.372722	1.753914	0.046955	1	Up	0.158682	0.533947
SCUBE1	92.73657	52.33767	133.1355	2.543779	1.346974	0.020197	0.680556	Up	0.685687	1.740181
SDS	4.967742	0	9.935483	Inf	Inf	0.026292	0.753091	Up	0	0.344767
SELL	11.93553	2.012987	21.85806	10.85852	3.440756	0.011304	0.511115	Up	0.046017	0.498515
SEMA4A	115.6011	54.35065	176.8516	3.2539	1.70217	0.001794	0.164342	Up	0.823585	2.673632
SEMA6A	564.4649	329.1234	799.8064	2.430111	1.281022	6.82E-05	0.013811	Up	2.226231	5.397408
5-Sep	622.6376	877.6624	367.6129	0.418855	-1.25548	6.67E-05	0.01371	Down	19.94094	8.332931
SERPINA1	15.92914	5.032468	26.8258	5.330547	2.414283	0.028652	0.787904	Up	0.073447	0.390601
SERPINE1	28.87763	10.06494	47.69032	4.738264	2.244358	0.010655	0.491059	Up	0.176134	0.832629
SESN1	1359.999	900.8117	1819.187	2.019497	1.013996	0.000276	0.043204	Up	13.8494	27.9038
SHANK3	12.94202	4.025974	21.85806	5.42926	2.440756	0.042186	0.997173	Up	0.031847	0.172506
SIGLEC14	9.941956	1.006494	18.87742	18.75563	4.229252	0.008472	0.439794	Up	0.01083	0.202654
SLAMF1	85.71053	41.26624	130.1548	3.154027	1.657195	0.005849	0.356539	Up	0.561184	1.765873
SLC22A4	173.6218	115.7468	231.4968	2.000028	1.00002	0.030072	0.808225	Up	2.25453	4.498637
SLC25A6	1224.416	751.8507	1696.981	2.257071	1.174452	3.26E-05	0.008515	Up	28.28271	63.68765
SLC2A6	50.76158	14.09091	87.43225	6.204869	2.633401	0.000442	0.062884	Up	0.290586	1.798853
SLC43A2	484.1137	194.2533	773.9741	3.984356	1.994347	5.58E-09	7.29E-06	Up	1.176144	4.675282
SLC6A8	564.6688	824.3182	305.0193	0.370026	-1.4343	9.00E-06	0.00332	Down	12.59359	4.649123
SLC7A11	515.0707	295.9091	734.2322	2.481276	1.311082	6.63E-05	0.01371	Up	1.695117	4.196274
SLX1A	106.7433	67.43507	146.0516	2.165811	1.114907	0.042664	0.997516	Up	3.217535	6.952367
SPON2	436.4833	589.8052	283.1613	0.480093	-1.05861	0.001861	0.168287	Down	13.28711	6.364214
SQSTM1	2595.679	1627.5	3563.858	2.189774	1.130782	2.89E-05	0.008147	Up	23.48257	51.30195
SRGN	15868.26	9393.605	22342.91	2.378524	1.250067	1.02E-05	0.00359	Up	177.8804	422.109
STAP1	61.80712	32.20779	91.40645	2.838023	1.504886	0.023218	0.713392	Up	0.685205	1.940105
STC1	5.535715	11.07143	0	0	#NAME?	0.017897	0.651953	Down	0.159416	0
STON1	23.40017	8.051949	38.74838	4.812299	2.266726	0.016639	0.634617	Up	0.079797	0.383112
STOX2	135.6663	84.54546	186.7871	2.209309	1.143596	0.023756	0.719146	Up	0.24574	0.541654
SUCNR1	107.0977	45.29221	168.9032	3.729189	1.898862	0.000771	0.090402	Up	0.604737	2.249935
SYT11	157.1376	101.6559	212.6193	2.09156	1.06458	0.02616	0.753091	Up	1.072956	2.238935
TARP	18.92273	7.045455	30.8	4.371612	2.128165	0.035819	0.898844	Up	0.078099	0.340626
TBC1D30	1703.073	3187.565	218.5806	0.068573	-3.86622	9.98E-36	1.44E-31	Down	19.62757	1.34279
TFAP2C	51.8328	26.16883	77.49677	2.961415	1.566287	0.025763	0.751713	Up	0.50972	1.505982
TFEC	167.5375	96.62338	238.4516	2.467846	1.303252	0.005608	0.350586	Up	0.665753	1.639156
TGM5	190.8535	91.59092	290.1161	3.167521	1.663354	0.000252	0.041612	Up	1.611408	5.0923
THBS1	46.81975	19.12338	74.51612	3.896598	1.962215	0.007865	0.428381	Up	0.147431	0.573144
THEMIS2	175.0603	107.6948	242.4258	2.251044	1.170594	0.011174	0.506828	Up	2.197364	4.934861
TLR4	45.32296	18.11688	72.52903	4.003394	2.001224	0.007423	0.415308	Up	0.174946	0.698749
TLR8	5.464516	0	10.92903	Inf	Inf	0.019219	0.661141	Up	0	0.135024

TNF	3594.046	2222.338	4965.755	2.234473	1.159935	1.70E-05	0.005398	Up	74.02175	165.015
TNFAIP3	1778.031	1170.552	2385.51	2.037935	1.027108	0.000174	0.030463	Up	7.409598	15.06517
TNFAIP6	5.464516	0	10.92903	Inf	Inf	0.019219	0.661141	Up	0	0.427449
TNFRSF19	30.60744	47.3052	13.90968	0.294041	-1.76591	0.035347	0.890105	Down	0.428281	0.125639
TP53I3	57.29084	25.16234	89.41935	3.553698	1.829321	0.007845	0.428381	Up	0.387175	1.372703
TP53INP1	291.3831	119.7727	462.9935	3.8656	1.950692	8.73E-07	0.000461	Up	1.165254	4.493933
TP53INP2	211.3183	106.6883	315.9484	2.961415	1.566287	0.000332	0.049238	Up	1.417036	4.186672
TREM1	9.438709	0	18.87742	Inf	Inf	0.001969	0.174809	Up	0	0.144003
TREM2	8.948408	1.006494	16.89032	16.78135	4.068787	0.013802	0.573601	Up	0.053258	0.891657
TRIM22	1277.308	556.5909	1998.026	3.589756	1.843886	1.40E-10	2.24E-07	Up	10.05218	36.00098
TUBB2A	5.032468	10.06494	0	0	#NAME?	0.024599	0.725979	Down	0.320518	0
TUBB3	75.76858	40.25974	111.2774	2.763987	1.466751	0.01816	0.653633	Up	1.120375	3.089501
TYMP	199.4217	110.7143	288.129	2.602455	1.379873	0.001829	0.166429	Up	3.02968	7.866274
TYROBP	204.9315	117.7597	292.1032	2.480501	1.310632	0.002736	0.219784	Up	11.12327	27.52714
VCAN	16.41297	3.019481	29.80645	9.871383	3.303252	0.004972	0.327392	Up	0.013576	0.133703
WDR54	44.6854	73.47403	15.89677	0.216359	-2.2085	0.003611	0.264411	Down	3.449652	0.744628
XAGE1A	90.2349	126.8182	53.65161	0.423059	-1.24107	0.033313	0.861336	Down	7.366844	3.109364
ZC3H12A	249.4292	162.0455	336.8129	2.078509	1.055549	0.009213	0.456797	Up	3.230741	6.699508
ZNF395	1734.962	2816.169	653.7548	0.232143	-2.10691	1.02E-13	1.84E-10	Down	32.73858	7.58237
