# Supplementary material for: Repressing HIF-1α-induced HDAC9 contributes to the synergistic effect of venetoclax and MENIN inhibitor in KMT2Ar AML
Source: Biomark Res. 2023 Dec 5;11:105. doi: 10.1186/s40364-023-00547-9 (PMC10696732; doi:10.1186/s40364-023-00547-9)
Supplement: Supplementary file 8 — Additional file 8: Table S7. Different expressed genes of MI-503 plus VEN vs. DMSO in MOLM13. [file 40364_2023_547_MOESM8_ESM.pdf]

gene_id	BaseMean	BaseMean_	BaseMean_	FoldChang	log2FoldCl	pValue	qValue	Regulation	Expression	Expression_MOLM13_MI_503_Venetoclax
A2M	8.75317	16.53892	0.967415	0.058493	-4.09559	0.018531	0.78115	Down	0.165988	0.009722
ABCA1	236.9451	405.2037	68.68645	0.169511	-2.56055	5.80E-07	0.000395	Down	1.63866	0.278131
ABTB2	14.09378	2.067366	26.1202	12.63453	3.6593	0.008062	0.526444	Up	0.022919	0.289951
ACPP	579.2522	822.8115	335.6929	0.407983	-1.29342	0.001036	0.131727	Down	10.34815	4.227341
ADA2	535.2	293.5659	776.834	2.6462	1.403922	0.000473	0.079514	Up	2.388065	6.327492
ADAMDEC	33.40894	1.033683	65.7842	63.64061	5.991876	4.02E-06	0.001794	Up	0.023046	1.46858
ADAMTS2	106.3363	57.88624	154.7864	2.673975	1.418986	0.022272	0.837687	Up	0.432503	1.158003
ADAMTS5	125.9828	67.18938	184.7762	2.750081	1.459474	0.013449	0.67959	Up	0.365507	1.006476
ADGRD1	65.06228	98.19987	31.92469	0.325099	-1.62105	0.024487	0.879798	Down	0.543337	0.176868
AFF1	5783.252	8059.625	3506.878	0.435117	-1.20053	0.000458	0.079514	Down	38.87209	16.93584
AGT	46.51548	17.57261	75.45835	4.29409	2.102352	0.009981	0.59971	Up	0.36965	1.589369
AGXT	29.32742	54.78519	3.869659	0.070633	-3.82351	0.000339	0.063026	Down	1.863517	0.131797
AK4	108.3974	167.4566	49.33815	0.294632	-1.76301	0.00468	0.379615	Down	1.202254	0.354682
AKAP12	7.752621	15.50524	0	0	#NAME?	0.006343	0.458134	Down	0.07666	0
ALDH1A3	29.778	53.75151	5.804489	0.107987	-3.21106	0.001404	0.158047	Down	0.809788	0.08756
ALDOC	981.7984	1505.042	458.5546	0.304679	-1.71464	3.57E-06	0.001763	Down	47.09728	14.36816
ANGPT1	8.75317	16.53892	0.967415	0.058493	-4.09559	0.018531	0.78115	Down	0.19982	0.011703
ANK3	30.19544	51.68414	8.706733	0.16846	-2.56952	0.007052	0.484815	Down	0.142123	0.023973
ANKRD36	337.1192	497.2014	177.0369	0.356067	-1.48978	0.000823	0.117985	Down	2.765642	0.98603
ANKRD36E	228.5428	309.0712	148.0145	0.478901	-1.0622	0.02979	0.961623	Down	1.671128	0.801343
ANKRD36C	188.8389	262.5554	115.1224	0.438469	-1.18945	0.02168	0.834376	Down	1.768019	0.776226
ARID5B	124.108	174.6924	73.52352	0.420874	-1.24854	0.033901	1	Down	0.94146	0.39675
ASF1A	1862.766	2565.601	1159.93	0.452109	-1.14526	0.000955	0.126783	Down	55.58216	25.16176
ASMTL	485.6643	664.658	306.6705	0.461396	-1.11592	0.005973	0.438019	Down	13.74738	6.351209
AXL	12.33793	22.74102	1.93483	0.085081	-3.55502	0.012463	0.65765	Down	0.252267	0.021491
BASP1	91.6328	21.70734	161.5583	7.442564	2.8958	3.44E-05	0.01025	Up	0.601081	4.479388
BAZ2B	948.0508	1297.272	598.8297	0.461607	-1.11526	0.002263	0.230958	Down	6.17789	2.855455
BCAT1	4538.063	6109.065	2967.061	0.485682	-1.04192	0.002297	0.231352	Down	25.22899	12.26915
BCL2	1547.182	2287.54	806.8239	0.352704	-1.50347	2.08E-05	0.006622	Down	11.05159	3.90299
BEX1	4161.548	2541.826	5781.271	2.274456	1.185521	0.000548	0.087059	Up	160.2823	365.0277
BNIP3	888.2237	1362.394	414.0535	0.303916	-1.71825	4.45E-06	0.001873	Down	45.34856	13.80003
BPI	254.9075	105.4356	404.3794	3.835319	1.939347	7.14E-05	0.018233	Up	2.997413	11.51094
BST1	212.7587	103.3683	322.1491	3.116518	1.639935	0.001245	0.147116	Up	1.201086	3.74806
BST2	1264.679	838.3168	1691.041	2.017186	1.012344	0.004272	0.361438	Up	44.71785	90.32122
BTG2	550.0626	364.89	735.2352	2.01495	1.010744	0.010627	0.615264	Up	7.3134	14.75525
C18orf32	7.772452	1.033683	14.51122	14.03837	3.811303	0.034256	1	Up	0.033991	0.477794
C3	519.8928	42.381	997.4046	23.53424	4.556689	1.63E-21	5.82E-18	Up	0.448881	10.57778
C4B	6.685804	12.40419	0.967415	0.077991	-3.68055	0.049133	1	Down	0.124238	0.009702
C6orf223	897.626	1308.642	486.6096	0.371843	-1.42723	0.000117	0.028949	Down	11.15454	4.153116
CACNA2D4	1410.861	1897.842	923.8811	0.486806	-1.03858	0.003119	0.286739	Down	10.92323	5.324391
CAPN11	16.40639	28.94312	3.869659	0.133699	-2.90294	0.01575	0.729881	Down	0.548929	0.073486
CBS	206.7097	307.0038	106.4156	0.346626	-1.52855	0.002695	0.256911	Down	4.318706	1.498919
CCDC144N	379.3809	593.3339	165.4279	0.278811	-1.84264	2.60E-05	0.008096	Down	5.558639	1.551818
CCDC9B	168.9472	230.5113	107.383	0.465847	-1.10207	0.03921	1	Down	2.352104	1.097142
CCL3	182.0266	65.12202	298.9312	4.590324	2.198596	5.43E-05	0.014638	Up	4.441366	20.41375
CCL3L3	227.1705	100.2672	354.0738	3.531301	1.8202	0.000288	0.056185	Up	7.041498	24.8979
CCL4	15.96234	0	31.92469	Inf	Inf	0.000197	0.040753	Up	0	2.605017
CCL4L2	24.90101	7.23578	42.56625	5.882745	2.55649	0.012293	0.653504	Up	0.21329	1.256361
CCND1	128.8589	187.0966	70.62128	0.377459	-1.40561	0.016159	0.742749	Down	2.371138	0.896168
CD109	1278.69	1833.753	723.6262	0.394615	-1.34148	0.000168	0.037128	Down	10.88991	4.302894
CD180	88.85681	131.2777	46.43591	0.353723	-1.49931	0.022114	0.834376	Down	2.483719	0.879687
CD1A	9.753719	17.57261	1.93483	0.110105	-3.18305	0.034756	1	Down	0.424711	0.046823
CD300A	410.3382	593.3339	227.3425	0.383161	-1.38398	0.001089	0.134218	Down	14.01618	5.377418
CD38	344.8901	45.48204	644.2982	14.16599	3.82436	2.31E-14	6.49E-11	Up	0.43418	6.158562
CD82	167.2438	86.82936	247.6582	2.85224	1.512095	0.005351	0.409223	Up	1.939865	5.540135
CD83	550.2082	309.0712	791.3453	2.560398	1.356368	0.000675	0.102667	Up	6.661321	17.07775
CDC42EP1	487.6455	681.197	294.0941	0.431731	-1.21179	0.002871	0.271935	Down	14.03605	6.067661
CDHR1	75.81654	116.8062	34.82693	0.29816	-1.74584	0.011685	0.637749	Down	0.788317	0.23535
CEACAM4	19.5471	6.202097	32.8921	5.303384	2.406913	0.028281	0.939767	Up	0.091684	0.486864
CEMIP2	38.8093	18.60629	59.0123	3.171632	1.665225	0.048719	1	Up	0.151311	0.480525
CEP85L	506.8814	738.0495	275.7132	0.37357	-1.42055	0.000462	0.079514	Down	3.659677	1.368918
CFD	3203.976	1309.676	5098.276	3.892776	1.960799	2.43E-08	2.48E-05	Up	62.17351	242.3414
CHI3L1	6429.587	925.1461	11934.03	12.89961	3.689256	1.25E-22	8.94E-19	Up	27.37462	353.58
CHRDL1	4.651573	9.303145	0	0	#NAME?	0.035601	1	Down	0.121587	0
CHRFAM7A	206.3585	311.1385	101.5785	0.326474	-1.61496	0.001581	0.175266	Down	4.495541	1.469579
CNOT6L	3045.522	4288.75	1802.294	0.420238	-1.25072	0.00028	0.055551	Down	24.28829	10.22009
CNR2	111.339	63.05465	159.6234	2.531509	1.339998	0.028254	0.939767	Up	0.919612	2.331026
COL27A1	894.1801	1201.139	587.2208	0.488886	-1.03243	0.004926	0.388684	Down	7.259987	3.553912
CORO2A	17.57914	5.168414	29.98986	5.802526	2.536681	0.027365	0.931719	Up	0.045297	0.26318
CORO2B	35.84078	16.53892	55.14264	3.334113	1.737303	0.045397	1	Up	0.205671	0.68662
CPEB2	954.2198	1308.642	599.7971	0.458335	-1.12552	0.002049	0.213842	Down	8.379372	3.845543
CPM	1848.217	1206.308	2490.126	2.064254	1.04562	0.002522	0.247061	Up	9.747286	20.14697
CPNE5	35.13191	54.78519	15.47864	0.282533	-1.82351	0.036711	1	Down	0.564424	0.159675
CPZ	27.6775	48.58309	6.771903	0.139388	-2.84282	0.00447	0.369521	Down	1.039677	0.145106
CR1	32.40839	0	64.81679	Inf	Inf	6.68E-07	0.000434	Up	0	0.348351
CRYL1	130.6344	76.49253	184.7762	2.415611	1.272388	0.028478	0.939767	Up	1.828418	4.422476
CSF2RA	80.5605	8.269462	152.8515	18.48385	4.208194	1.76E-07	0.000158	Up	0.150888	2.792602
CSPG4	371.0653	635.7149	106.4156	0.167395	-2.57867	1.48E-08	1.62E-05	Down	4.168261	0.698652
CSRP3	14.92213	27.90944	1.93483	0.069325	-3.85047	0.004699	0.379615	Down	1.036232	0.07193
CST3	1830.704	1203.207	2458.201	2.043041	1.030718	0.002908	0.27362	Up	26.6401	54.49739
CTSD	1195.932	730.8137	1661.051	2.272879	1.184521	0.000918	0.126443	Up	18.71129	42.58364
CXCL10	48.73521	11.37051	86.09991	7.572211	2.920715	0.000569	0.089376	Up	0.508269	3.853709
CXCL8	533.9866	44.44836	1023.525	23.02728	4.525272	1.48E-21	5.82E-18	Up	1.255734	28.95364
DACH1	2518.636	3424.591	1612.68	0.470912	-1.08647	0.001558	0.174035	Down	16.43101	7.747589
DCANP1	188.872	263.5891	114.1549	0.433079	-1.2073	0.019832	0.794408	Down	4.570218	1.981833
DDIT4	7766.521	11034.56	4498.479	0.407672	-1.29452	0.000165	0.037128	Down	342.9354	139.9863

DDN	490.8264	780.4305	201.2223	0.257835	-1.95548	2.58E-06	0.001416	Down	9.414344	2.430495
DEPP1	93.57465	142.6482	44.50108	0.311964	-1.68055	0.009647	0.590462	Down	3.76397	1.175745
DHRSX	17.06229	4.134731	29.98986	7.253158	2.858609	0.016446	0.746192	Up	0.087213	0.633388
DLL1	1551.687	2126.286	977.0889	0.459529	-1.12177	0.001341	0.153459	Down	34.85411	16.03723
DLX3	77.85078	119.9072	35.79435	0.298517	-1.74411	0.011124	0.621386	Down	2.504869	0.748716
DLX5	658.0508	1032.649	283.4525	0.274491	-1.86517	2.07E-06	0.001182	Down	17.75162	4.878972
DNAH14	172.1476	239.8144	104.4808	0.435674	-1.19868	0.024411	0.879798	Down	0.610812	0.26646
DNAJB2	139.2219	87.86304	190.5807	2.169066	1.117074	0.049063	1	Up	1.182441	2.568118
DNHD1	341.5983	455.8541	227.3425	0.498718	-1.00371	0.022072	0.834376	Down	1.580153	0.789072
DNMT3B	1116.103	1499.874	732.333	0.488263	-1.03427	0.003898	0.33991	Down	17.60843	8.608697
DOC2A	15.09433	3.101048	27.08761	8.734985	3.126805	0.014322	0.703125	Up	0.038581	0.337442
DYNC1I1	190.1776	319.408	60.94713	0.190813	-2.38977	1.01E-05	0.003628	Down	2.725545	0.520743
DYNLT3	40.77726	19.63997	61.91454	3.152476	1.656485	0.046461	1	Up	0.481746	1.520662
E2F5	170.4778	248.0839	92.87182	0.374357	-1.41752	0.008334	0.539239	Down	7.265534	2.723427
EBI3	14.99493	0	29.98986	Inf	Inf	0.000291	0.056185	Up	0	1.167554
EFNA5	165.1769	233.6123	96.74148	0.414111	-1.27191	0.018546	0.78115	Down	1.445772	0.599487
EIF4E3	759.9544	1148.422	371.4873	0.323476	-1.62827	1.94E-05	0.006317	Down	9.194805	2.978159
ELANE	10845.32	3896.984	17793.66	4.566008	2.190933	6.88E-10	1.09E-06	Up	125.5627	574.0636
EMB	3044.667	4231.897	1857.436	0.438913	-1.18799	0.000548	0.087059	Down	51.61067	22.68199
ENO2	161.8439	220.1744	103.5134	0.470143	-1.08883	0.044257	1	Down	4.939244	2.32516
ESYT3	145.2852	201.5681	89.00216	0.441549	-1.17936	0.035283	1	Down	1.344512	0.594437
ETV1	71.19811	108.5367	33.85952	0.311964	-1.68055	0.016892	0.754933	Down	0.738375	0.230645
EVI2A	1256.856	1680.768	832.9441	0.495573	-1.01283	0.00426	0.361438	Down	30.35207	15.06119
EVI5L	268.8557	163.3219	374.3895	2.292341	1.196822	0.010742	0.616904	Up	2.240665	5.14303
EVPL	90.67241	142.6482	38.69659	0.271273	-1.88218	0.004389	0.367157	Down	1.160745	0.315287
FABP3	14.85586	25.84207	3.869659	0.149743	-2.73944	0.026049	0.911908	Down	0.483036	0.072425
FAM43A	495.536	293.5659	697.506	2.375978	1.248521	0.002098	0.217394	Up	5.073784	12.07083
FBN2	249.408	160.2208	338.5952	2.113303	1.0795	0.023822	0.866866	Up	0.812099	1.718438
FCGR2B	820.7693	1219.746	421.7928	0.345804	-1.53197	4.62E-05	0.012946	Down	19.67956	6.814092
FCGRT	516.2428	335.9469	696.5386	2.073359	1.05197	0.008667	0.545971	Up	8.691437	18.04384
FCRLA	16.92323	29.9768	3.869659	0.129088	-2.95357	0.013349	0.676938	Down	0.689554	0.089129
FDXR	219.8554	143.6819	296.0289	2.060307	1.04286	0.035005	1	Up	2.204959	4.548785
FGD4	212.3352	286.3301	138.3403	0.48315	-1.04946	0.035508	1	Down	1.462483	0.707514
FGR	46.21727	8.269462	84.16508	10.17782	3.347356	0.000171	0.037128	Up	0.138136	1.407746
FLT3	7636.755	11105.89	4167.623	0.375262	-1.41403	4.08E-05	0.011674	Down	148.7607	55.8967
FPR1	4.353366	0	8.706733	Inf	Inf	0.04771	1	Up	0	0.345643
FTCD	50.53775	82.69462	18.38088	0.222274	-2.16959	0.006376	0.458164	Down	0.81063	0.180416
FTL	45772.8	26635.94	64909.66	2.43692	1.285059	0.000335	0.062951	Up	1224.891	2988.833
FUCA1	354.0678	180.8945	527.241	2.914633	1.543314	0.00047	0.079514	Up	2.914253	8.504993
FYB1	204.4629	131.2777	277.648	2.114967	1.080635	0.032552	1	Up	1.123382	2.378997
G0S2	4.353366	0	8.706733	Inf	Inf	0.04771	1	Up	0	0.492083
GABRA4	118.1842	186.0629	50.30557	0.270369	-1.887	0.001992	0.212528	Down	0.844703	0.228677
GBP2	231.0939	313.2059	148.9819	0.475668	-1.07197	0.027853	0.939767	Down	4.119187	1.961904
GLIPR2	457.1045	301.8354	612.3735	2.028833	1.02065	0.012899	0.665882	Up	7.087061	14.39711
GNPDA1	390.8629	257.387	524.3388	2.037161	1.02656	0.015723	0.729881	Up	5.09487	10.39253
GOLGA8J	52.37318	79.59358	25.15278	0.316015	-1.66193	0.030732	0.974777	Down	0.718549	0.227367
GOLGA8M	1148.685	1701.442	595.9275	0.350249	-1.51355	2.86E-05	0.008687	Down	7.530614	2.641007
GOLGA8N	507.4976	742.1843	272.811	0.367578	-1.44388	0.000374	0.067706	Down	7.570299	2.786287
GOLGA8O	248.6201	331.8122	165.4279	0.498559	-1.00416	0.035228	1	Down	3.295444	1.645103
GOLGA8Q	215.5954	327.6775	103.5134	0.3159	-1.66246	0.001008	0.129904	Down	9.37926	2.966753
GOLGA8R	312.98	438.2815	187.6785	0.428214	-1.22359	0.0066	0.467246	Down	4.606188	1.974994
GPR137	266.3179	174.6924	357.9435	2.048993	1.034915	0.027225	0.931719	Up	2.407599	4.93955
GPR141	15.09433	3.101048	27.08761	8.734985	3.126805	0.014322	0.703125	Up	0.014462	0.126494
GPR65	213.5479	309.0712	118.0246	0.381869	-1.38885	0.005709	0.424655	Down	3.715138	1.420535
GPRC5A	4.134731	8.269462	0	0	#NAME?	0.049031	1	Down	0.157662	0
GPRC5C	456.5758	738.0495	175.1021	0.23725	-2.07552	9.95E-07	0.000593	Down	7.412671	1.760935
GRAP	153.9721	216.0397	91.9044	0.425405	-1.23309	0.025276	0.903629	Down	5.637562	2.401358
GRN	3965.738	2197.61	5733.867	2.609138	1.383573	6.00E-05	0.015894	Up	43.53248	113.7296
GTPBP6	158.9678	100.2672	217.6683	2.170882	1.118281	0.040274	1	Up	1.499345	3.259122
H2AFX	945.8156	608.8392	1282.792	2.106947	1.075154	0.00324	0.295126	Up	20.76162	43.80037
HBB	31.18921	7.23578	55.14264	7.620829	2.929948	0.002668	0.256061	Up	0.628287	4.794276
HDAC9	467.1242	629.5128	304.7356	0.484082	-1.04668	0.010406	0.611785	Down	2.480267	1.202209
HILPDA	310.4289	434.1468	186.711	0.430064	-1.21738	0.006991	0.484815	Down	16.71279	7.196895
HLA-A	3606.954	2231.721	4982.186	2.232441	1.158622	0.000734	0.10818	Up	75.29938	168.3194
HLA-B	4979.797	2852.965	7106.629	2.490963	1.316704	0.000128	0.030983	Up	96.26044	240.0921
HLA-C	4974.715	3132.059	6817.372	2.176642	1.122104	0.001041	0.131727	Up	107.343	233.9503
HLA-DQB1	46.61488	20.67366	72.55611	3.509592	1.811303	0.024273	0.878761	Up	0.675322	2.373179
HMGA2	569.1476	884.8325	253.4627	0.286453	-1.80363	7.21E-06	0.002714	Down	7.933999	2.275663
HOMER3	2125.235	1111.209	3139.261	2.825086	1.498295	1.69E-05	0.005632	Up	26.53814	75.06975
HPDL	186.1686	269.7912	102.546	0.380094	-1.39557	0.007632	0.508988	Down	8.13353	3.095513
HRC	23.60903	42.381	4.837074	0.114133	-3.13121	0.003692	0.327947	Down	0.949962	0.108563
HSD11B1	105.0177	62.02097	148.0145	2.386523	1.25491	0.043069	1	Up	2.152751	5.144252
HUNK	130.2169	78.55989	181.874	2.3151	1.211074	0.036954	1	Up	0.578226	1.340386
IER3	164.8915	88.89672	240.8863	2.709732	1.43815	0.008193	0.532534	Up	3.896826	10.57305
IFI16	602.245	861.0578	343.4322	0.398849	-1.32608	0.000716	0.107755	Down	9.169985	3.662184
IFIT1	22.95966	37.21258	8.706733	0.233973	-2.09559	0.038526	1	Down	0.427818	0.100228
IFIT2	66.46043	111.6377	21.28312	0.190645	-2.39104	0.00128	0.148815	Down	1.736244	0.331435
IL4I1	46.48235	16.53892	76.42577	4.620963	2.208194	0.007145	0.48878	Up	0.359883	1.665165
ITGA3	5.168414	10.33683	0	0	#NAME?	0.026146	0.911908	Down	0.111592	0
ITGAX	276.4499	370.0584	182.8414	0.494088	-1.01716	0.028135	0.939767	Down	4.016548	1.987101
ITIH4	64.42596	33.07785	95.77406	2.895414	1.53377	0.033799	1	Up	0.547663	1.587767
JMJD1C	3497.506	5200.458	1794.554	0.345076	-1.53501	9.41E-06	0.00345	Down	22.26319	7.692461
JPH2	140.3555	243.9491	36.76176	0.150694	-2.7303	6.33E-06	0.002447	Down	1.804584	0.272293
JUN	426.0473	147.8166	704.2779	4.764538	2.252336	2.20E-07	0.000175	Up	2.417909	11.53516
KBTBD13	5.685255	11.37051	0	0	#NAME?	0.019392	0.78115	Down	0.197904	0
KCNG1	190.1906	259.4544	120.9268	0.466081	-1.10135	0.032805	1	Down	2.715749	1.267402
KCNQ1	181.5695	96.1325	267.0065	2.777484	1.473779	0.005346	0.409223	Up	1.489984	4.143772

KCNQ3	2058.271	2930.491	1186.05	0.404728	-1.30498	0.000167	0.037128	Down	6.482564	2.627075
KDM7A	411.9151	551.9866	271.8435	0.492482	-1.02186	0.014877	0.718713	Down	3.269095	1.612058
KIAA0930	7822.671	4954.442	10690.9	2.157842	1.109589	0.001187	0.141466	Up	39.86136	86.12604
KIAA1147	344.7987	465.1573	224.4402	0.482504	-1.05139	0.016343	0.746192	Down	3.364479	1.62548
KRTAP5-1	4.353366	0	8.706733	Inf	Inf	0.04771	1	Up	0	0.447898
LIN28B	235.8913	342.149	129.6336	0.37888	-1.40019	0.004159	0.358294	Down	3.196602	1.212701
LOC10192	73.55037	106.4693	40.63142	0.381626	-1.38977	0.044061	1	Down	2.828563	1.080852
LOC10272	59.40363	117.8398	0.967415	0.00821	-6.92848	2.46E-09	3.20E-06	Down	4.103328	0.03373
LOC10537	21.99224	37.21258	6.771903	0.181979	-2.45816	0.019209	0.78115	Down	1.006832	0.18346
LOC10537	9.236877	16.53892	1.93483	0.116986	-3.09559	0.042979	1	Down	0.342733	0.040147
LOC10537	42.22838	19.63997	64.81679	3.300248	1.722575	0.036858	1	Up	0.314726	1.04002
LOC10537	31.64656	51.68414	11.60898	0.224614	-2.15448	0.018698	0.78115	Down	0.34386	0.077336
LOC10798	31.06345	48.58309	13.54381	0.278776	-1.84282	0.042332	1	Down	0.253215	0.070682
LOC10798	4.134731	8.269462	0	0	#NAME?	0.049031	1	Down	0.985733	0
LOC11226	323.118	452.7531	193.483	0.427348	-1.22652	0.006052	0.441557	Down	3.314454	1.418261
LOC11226	11.19154	2.067366	20.31571	9.826859	3.29673	0.023824	0.866866	Up	0.066533	0.654655
LOC11226	5.320781	0	10.64156	Inf	Inf	0.027136	0.931719	Up	0	0.387932
LOC33986	305.2279	573.694	36.76176	0.064079	-3.964	2.72E-14	6.49E-11	Down	13.35489	0.856878
LPAR6	134.2924	205.7029	62.88196	0.305693	-1.70984	0.003362	0.304289	Down	2.471524	0.756508
LRMP	1207.624	1729.351	685.8971	0.396621	-1.33417	0.000196	0.040753	Down	30.5494	12.13225
LRRC25	129.0507	72.3578	185.7436	2.567016	1.360092	0.019949	0.79683	Up	1.512722	3.888216
MAML2	167.3768	241.8818	92.87182	0.383955	-1.38099	0.010499	0.611785	Down	1.225551	0.471167
MAP3K1	3877.581	5211.829	2543.333	0.487993	-1.03507	0.002477	0.244263	Down	24.36308	11.90442
MAP7	61.61006	96.1325	27.08761	0.281774	-1.82739	0.013299	0.676803	Down	0.43716	0.12334
MARCKS	524.207	222.2418	826.1722	3.717447	1.894312	3.84E-06	0.001772	Up	2.805425	10.44255
MARCO	7.288745	1.033683	13.54381	13.10248	3.711768	0.042807	1	Up	0.027235	0.357315
MEF2C	3813.302	5651.144	1975.461	0.349568	-1.51635	1.18E-05	0.004111	Down	39.19526	13.71919
MGAT5B	14.82273	24.80839	4.837074	0.194977	-2.35862	0.049193	1	Down	0.170091	0.033207
MMP2	19.06339	6.202097	31.92469	5.147402	2.363845	0.032241	1	Up	0.081746	0.421326
MMRN2	91.82532	133.3451	50.30557	0.377259	-1.40637	0.029737	0.961623	Down	1.262515	0.476912
MNDA	264.3429	52.71782	475.9681	9.028598	3.174502	7.74E-10	1.11E-06	Up	1.622609	14.66888
MPO	314.6887	174.6924	454.6849	2.602775	1.38005	0.002277	0.230958	Up	2.953521	7.697318
MS4A14	208.83	297.7006	119.9594	0.402953	-1.31132	0.009359	0.579396	Down	4.687659	1.891357
MS4A2	9.223574	1.033683	17.41347	16.84604	4.074338	0.017934	0.774788	Up	0.013717	0.231385
MS4A3	499.0472	101.3009	896.7935	8.852768	3.146129	8.83E-13	1.80E-09	Up	3.26203	28.91544
MSRB1	818.0302	470.3257	1165.735	2.478569	1.309508	0.000464	0.079514	Up	18.20868	45.19001
MSRB3	916.4153	581.9634	1250.867	2.149392	1.103928	0.002616	0.254505	Up	5.942733	12.78982
MUC8	21.99224	37.21258	6.771903	0.181979	-2.45816	0.019209	0.78115	Down	0.917335	0.167152
MXD3	336.7803	215.006	458.5546	2.132752	1.092716	0.013264	0.676803	Up	3.697202	7.895441
MYBPH	98.16624	44.44836	151.8841	3.417091	1.772769	0.005916	0.436074	Up	1.337781	4.577248
MYLIP	1868.113	2521.152	1215.073	0.481951	-1.05304	0.002336	0.233649	Down	42.63833	20.57625
MYO1B	1095.756	1800.675	390.8356	0.217049	-2.2039	3.23E-09	3.85E-06	Down	17.73463	3.854282
MYOF	262.1177	390.7321	133.5032	0.341675	-1.54931	0.001154	0.13863	Down	2.503372	0.856448
MYOM1	19.4743	34.11153	4.837074	0.141802	-2.81805	0.012112	0.651131	Down	0.311834	0.044276
NABP1	411.5177	615.0413	207.9942	0.338179	-1.56414	0.000239	0.048136	Down	6.097236	2.064632
NAV3	83.92662	22.74102	145.1122	6.381077	2.6738	0.000158	0.036955	Up	0.062642	0.40024
NBPF8	329.8568	451.7194	207.9942	0.46045	-1.11888	0.011616	0.637749	Down	3.382975	1.559711
NCALD	191.1708	124.0419	258.2997	2.082358	1.058218	0.040045	1	Up	0.797919	1.663708
NCAM2	324.4697	449.652	199.2874	0.443204	-1.17396	0.008452	0.544403	Down	1.193829	0.529795
NDFIP1	521.3381	298.7343	743.9419	2.490313	1.316327	0.001077	0.134179	Up	4.514313	11.25663
NEB	4.134731	8.269462	0	0	#NAME?	0.049031	1	Down	0.016479	0
NECTIN1	96.59588	55.81887	137.3729	2.461047	1.299273	0.041322	1	Up	0.496252	1.222884
NEU1	1289.951	826.9462	1752.956	2.119794	1.083924	0.002215	0.227836	Up	21.70422	46.06813
NFE2	737.9931	433.1131	1042.873	2.407854	1.267748	0.000825	0.117985	Up	11.02682	26.58541
NFKBIZ	216.158	118.8735	313.4424	2.636772	1.398773	0.005292	0.409046	Up	1.539548	4.064701
NIPA1	1408.132	1948.492	867.771	0.445355	-1.16697	0.000928	0.126443	Down	16.07408	7.167958
NR2F2	33.87282	15.50524	52.2404	3.369209	1.75241	0.047475	1	Up	0.137938	0.465345
NR5A2	12.8879	24.80839	0.967415	0.038995	-4.68055	0.003128	0.286739	Down	0.1629	0.006361
NRIP1	1368.182	1879.235	857.1295	0.456105	-1.13256	0.001329	0.153319	Down	11.84732	5.410635
OBSCN	237.8394	357.6543	118.0246	0.329996	-1.59948	0.001112	0.135936	Down	0.502797	0.166136
OCSTAMP	22.02538	38.24626	5.804489	0.151766	-2.72008	0.010924	0.621386	Down	1.047838	0.159233
ONECUT2	1718.449	2590.409	846.4879	0.326778	-1.61362	4.66E-06	0.001904	Down	8.733663	2.857667
P3H2	9.753719	17.57261	1.93483	0.110105	-3.18305	0.034756	1	Down	0.234341	0.025836
PAN3	2398.775	3352.233	1445.318	0.431151	-1.21374	0.000429	0.076641	Down	16.46609	7.108574
PANK3	857.5308	1144.287	570.7747	0.498804	-1.00346	0.006525	0.466528	Down	18.36398	9.171901
PBX3	1221.705	1715.913	727.4959	0.42397	-1.23797	0.000525	0.085288	Down	29.44131	12.49842
PCDH12	36.29136	15.50524	57.07747	3.681172	1.880165	0.030743	0.974777	Up	0.128437	0.473411
PCDHGA1	60.4107	88.89672	31.92469	0.359121	-1.47746	0.043849	1	Down	0.912054	0.327963
PCDHGA1	21.4754	36.1789	6.771903	0.187178	-2.41752	0.021998	0.834376	Down	0.408589	0.076578
PCDHGC5	56.10377	105.4356	6.771903	0.064228	-3.96066	6.28E-06	0.002447	Down	1.189509	0.076499
PDE3A	65.67852	102.3346	29.02244	0.283603	-1.81805	0.012054	0.650487	Down	0.336998	0.095698
PDK1	638.4106	917.9103	358.9109	0.391009	-1.35473	0.000487	0.080915	Down	8.641129	3.383138
PFKFB4	296.6401	486.8646	106.4156	0.218573	-2.19381	3.52E-06	0.001763	Down	4.361955	0.954644
PIMREG	162.0555	90.96409	233.147	2.563066	1.357871	0.012791	0.665153	Up	2.130305	5.467193
PINK1	237.2224	127.143	347.3019	2.731585	1.449738	0.003044	0.282631	Up	2.598111	7.106166
PKDCC	24.19214	45.48204	2.902244	0.063811	-3.97006	0.000592	0.092057	Down	0.988492	0.063158
PLCXD1	248.7526	335.9469	161.5583	0.480904	-1.05618	0.026902	0.931719	Down	3.339559	1.608091
PLEKHA5	792.9725	1182.533	403.412	0.341142	-1.55155	4.05E-05	0.011674	Down	5.517399	1.884658
PLEKHH2	130.1577	197.4334	62.88196	0.318497	-1.65065	0.004947	0.388684	Down	1.448856	0.462055
PNCK	32.56101	65.12202	0	0	#NAME?	4.30E-07	0.000324	Down	1.095904	0
POLR3G	459.5042	618.1423	300.866	0.486726	-1.03882	0.011261	0.624938	Down	7.126131	3.472972
POU6F1	627.1327	852.7883	401.4771	0.470782	-1.08687	0.004976	0.38884	Down	3.85802	1.81864
PPFIA4	164.5275	228.4439	100.6111	0.440419	-1.18305	0.028368	0.939767	Down	1.908003	0.841411
PRAM1	1034.659	649.1528	1420.165	2.18772	1.129428	0.001809	0.197454	Up	14.39042	31.52304
PRAME	57.89277	85.79567	29.98986	0.34955	-1.51643	0.041298	1	Down	1.262454	0.441863
PRICKLE2	18.57316	36.1789	0.967415	0.02674	-5.22487	0.000347	0.063664	Down	0.093556	0.002505
PRRT4	75.45182	29.9768	120.9268	4.034014	2.012216	0.004244	0.361438	Up	0.343469	1.387356

PRTN3	2468.647	1125.681	3811.614	3.386053	1.759605	4.94E-07	0.000353	Up	57.18451	193.8809
PSTPIP1	138.5592	67.18938	209.929	3.124437	1.643596	0.004416	0.367157	Up	1.278761	4.00059
PTGER4	958.3985	578.8624	1337.935	2.311317	1.208715	0.000951	0.126783	Up	7.616705	17.62745
PTPN3	167.1449	234.646	99.64372	0.424656	-1.23564	0.021585	0.834376	Down	1.133624	0.482024
QPCT	45.9655	15.50524	76.42577	4.929028	2.301303	0.005451	0.41315	Up	0.494893	2.442502
QRICH2	104.1364	49.61677	158.656	3.197629	1.677002	0.007804	0.516673	Up	0.246659	0.789746
RAB5IF	205.0791	135.4124	274.7458	2.028955	1.020737	0.043044	1	Up	6.445246	13.09407
RABAC1	175.2615	110.6041	239.9189	2.169169	1.117142	0.034875	1	Up	7.449799	16.18083
RAET1E	19.05686	36.1789	1.93483	0.05348	-4.22487	0.001079	0.134179	Down	0.377237	0.020201
RBM47	147.478	88.89672	206.0593	2.317963	1.212858	0.030026	0.967043	Up	0.47541	1.103411
RBPMS	21.92597	35.14522	8.706733	0.247736	-2.01312	0.049336	1	Down	0.248065	0.061534
REL	392.9047	532.3466	253.4627	0.476123	-1.07059	0.011681	0.637749	Down	3.626999	1.729139
RHOBTB3	1362.139	1901.976	822.3025	0.432341	-1.20976	0.000624	0.095905	Down	17.04033	7.376791
RUBCNL	318.6188	433.1131	204.1245	0.471296	-1.08529	0.015175	0.72822	Down	5.007928	2.363278
RUNX2	522.0686	774.2284	269.9087	0.348616	-1.52029	0.000171	0.037128	Down	7.556817	2.637846
RXFP1	755.9851	1069.862	442.1085	0.413239	-1.27495	0.000734	0.10818	Down	13.42103	5.553286
S100A9	231.418	127.143	335.6929	2.640279	1.40069	0.004408	0.367157	Up	11.97743	31.66476
SAMD11	92.75961	132.3114	53.20781	0.402141	-1.31423	0.041118	1	Down	2.81594	1.133873
SAMHD1	2365.168	1353.091	3377.245	2.495948	1.319588	0.000135	0.032185	Up	14.72738	36.80644
SCD	45719.28	65589.24	25849.32	0.394109	-1.34333	0.000181	0.038604	Down	652.0053	257.2945
SEMA4A	115.9442	55.81887	176.0695	3.1543	1.65732	0.006568	0.467246	Up	0.823585	2.601203
SEMA7A	210.8178	284.2628	137.3729	0.48326	-1.04913	0.035948	1	Down	4.418464	2.138037
SERPINF1	19.95801	34.11153	5.804489	0.170162	-2.55502	0.019328	0.78115	Down	0.995257	0.169575
SH3TC2	33.91273	62.02097	5.804489	0.093589	-3.41752	0.000494	0.081183	Down	0.126833	0.011886
SHANK1	621.7655	836.2494	407.2816	0.487034	-1.03791	0.007341	0.497542	Down	4.715751	2.299708
SHOX2	904.2122	1242.487	565.9376	0.455488	-1.13452	0.00202	0.212846	Down	14.99146	6.837284
SIGLEC10	130.6344	76.49253	184.7762	2.415611	1.272388	0.028478	0.939767	Up	1.225051	2.963085
SIGLEC9	5.168414	10.33683	0	0	#NAME?	0.026146	0.911908	Down	0.123406	0
SIRPB2	120.3771	73.39148	167.3628	2.280411	1.189294	0.04535	1	Up	0.800414	1.82764
SLC15A2	161.9363	102.3346	221.538	2.164839	1.11426	0.039864	1	Up	0.921706	1.997932
SLC1A3	106.3032	56.85255	155.7538	2.739609	1.45397	0.019322	0.78115	Up	0.61133	1.676978
SLC22A4	233.5714	118.8735	348.2693	2.929747	1.550776	0.001658	0.182408	Up	2.25453	6.613766
SLC25A6	1580.354	772.1611	2388.547	3.093327	1.62916	4.27E-06	0.001849	Up	28.28271	87.60113
SLC2A3	3825.455	5758.647	1892.263	0.328595	-1.60562	3.70E-06	0.001763	Down	79.4862	26.15265
SLC2A6	36.74193	14.47156	59.0123	4.077812	2.027795	0.02023	0.805832	Up	0.290586	1.18649
SLC43A2	351.2782	199.5008	503.0557	2.521572	1.334324	0.002388	0.237149	Up	1.176144	2.969579
SLC44A1	378.3395	243.9491	512.7298	2.10179	1.071618	0.012429	0.65765	Up	1.057929	2.226428
SLC48A1	411.6094	270.8249	552.3938	2.039672	1.028337	0.014358	0.703125	Up	2.672162	5.457401
SLC6A8	592.107	846.5862	337.6277	0.398811	-1.32622	0.000741	0.10818	Down	12.59359	5.028974
SLX1B	238.6269	35.14522	442.1085	12.57948	3.653	2.79E-11	4.98E-08	Up	1.632779	20.56614
SMAGP	427.215	606.7718	247.6582	0.408157	-1.2928	0.002024	0.212846	Down	26.61957	10.87906
SMN1	679.8024	413.4731	946.1316	2.288254	1.194247	0.001827	0.197931	Up	12.17482	27.89521
SORL1	606.1857	304.9364	907.435	2.975817	1.573286	6.73E-05	0.017487	Up	1.279437	3.812308
SP7	4.134731	8.269462	0	0	#NAME?	0.049031	1	Down	0.129351	0
SPIB	82.82039	124.0419	41.59883	0.335361	-1.57621	0.018737	0.78115	Down	1.909492	0.6412
SPON2	361.8814	605.7381	118.0246	0.194844	-2.35961	1.91E-07	0.000161	Down	13.28711	2.592275
SQSTM1	2665.598	1671.465	3659.73	2.189534	1.130624	0.001	0.129904	Up	23.48257	51.48256
SREBF1	3937.428	5283.153	2591.704	0.49056	-1.0275	0.002658	0.256061	Down	51.00723	25.05456
SRPK3	150.7784	252.2186	49.33815	0.195617	-2.3539	4.86E-05	0.013376	Down	6.847941	1.341308
STC1	5.685255	11.37051	0	0	#NAME?	0.019392	0.78115	Down	0.159416	0
STEAP1	5.168414	10.33683	0	0	#NAME?	0.026146	0.911908	Down	0.427927	0
STOX2	163.8578	86.82936	240.8863	2.774249	1.472097	0.006968	0.484815	Up	0.24574	0.682629
STXBP1	194.4308	89.9304	298.9312	3.324028	1.732932	0.000928	0.126443	Up	1.232229	4.101274
SULT1A4	102.3406	23.7747	180.9066	7.609203	2.927745	1.54E-05	0.005253	Up	0.925051	7.04803
SYNPO2	475.5064	664.658	286.3548	0.43083	-1.21481	0.002948	0.275513	Down	2.573044	1.109983
SYT11	176.0301	104.402	247.6582	2.37216	1.246201	0.018807	0.78115	Up	1.072956	2.548525
TBC1D30	1735.513	3273.673	197.3526	0.060285	-4.05206	6.60E-25	9.44E-21	Down	19.62757	1.184778
TCEA3	6.685804	12.40419	0.967415	0.077991	-3.68055	0.049133	1	Down	0.265554	0.020738
TDRKH	60.00632	31.01048	89.00216	2.870067	1.521084	0.039193	1	Up	0.383615	1.102428
TEC	300.5693	443.4499	157.6886	0.355595	-1.49169	0.001145	0.13863	Down	4.541952	1.61719
TERT	196.8036	299.768	93.83923	0.31304	-1.67558	0.001255	0.147133	Down	4.055294	1.271114
TFAP2C	91.31476	26.87575	155.7538	5.795327	2.53489	0.000211	0.043123	Up	0.50972	2.957823
TGFB2	22.44934	6.202097	38.69659	6.239275	2.641378	0.013027	0.667696	Up	0.056149	0.350786
THBS4	94.93265	34.11153	155.7538	4.566015	2.190936	0.000958	0.126783	Up	0.461235	2.108737
THSD7A	1062.585	1701.442	423.7277	0.24904	-2.00555	6.25E-08	5.96E-05	Down	6.820818	1.700862
TLR4	43.64637	18.60629	68.68645	3.691571	1.884235	0.02202	0.834376	Up	0.174946	0.646663
TMEM255/	186.3872	261.5218	111.2527	0.425405	-1.23309	0.01786	0.774788	Down	3.297439	1.404567
TMEM37	36.64253	11.37051	61.91454	5.445186	2.444981	0.006326	0.458134	Up	0.26954	1.469598
TNFAIP6	8.706733	0	17.41347	Inf	Inf	0.004668	0.379615	Up	0	0.665557
TP53INP2	221.1805	109.5704	332.7907	3.037232	1.602757	0.001398	0.158047	Up	1.417036	4.309447
TRANK1	43.20257	65.12202	21.28312	0.326819	-1.61344	0.047251	1	Down	0.295325	0.096643
TREM1	5.320781	0	10.64156	Inf	Inf	0.027136	0.931719	Up	0	0.079329
TSPOAP1	6680.051	3743.999	9616.103	2.568404	1.360872	7.61E-05	0.019098	Up	22.13975	56.93757
TST	431.9715	287.3638	576.5792	2.006443	1.00464	0.015574	0.729881	Up	13.47708	27.07605
TUBA4A	1284.723	784.5653	1784.88	2.274993	1.185862	0.000842	0.119146	Up	16.46555	37.50759
TXNIP	3826.388	5380.319	2272.457	0.422365	-1.24344	0.000296	0.056488	Down	83.72525	35.40845
UNC5B	127.4542	203.6355	51.27298	0.251788	-1.98972	0.000899	0.126019	Down	1.409859	0.355446
VAT1L	800.2284	1257.992	342.4648	0.272231	-1.8771	8.77E-07	0.000545	Down	18.03728	4.916681
VCAN	12.19209	3.101048	21.28312	6.863203	2.778882	0.038789	1	Up	0.013576	0.093296
VIT	16.06175	3.101048	29.02244	9.358913	3.226341	0.010368	0.611785	Up	0.034928	0.327308
WNT7B	1028.821	648.1191	1409.523	2.17479	1.120876	0.001968	0.211572	Up	8.534187	18.58414
ZBED6	776.3869	1057.458	495.3164	0.468403	-1.09418	0.003486	0.311562	Down	16.18218	7.589611
ZC3HAV1L	333.5276	445.5173	221.538	0.49726	-1.00793	0.022319	0.837687	Down	5.094995	2.536823
ZFP36L1	171.4121	247.0502	95.77406	0.38767	-1.3671	0.010701	0.616904	Down	2.68251	1.041278
ZIC1	95.24441	134.3788	56.11006	0.417552	-1.25997	0.048145	1	Down	1.396346	0.583802
ZNF221	102.3145	143.6819	60.94713	0.424181	-1.23725	0.047312	1	Down	1.668439	0.708638
ZNF292	2025.544	2784.741	1266.346	0.454745	-1.13687	0.001001	0.129904	Down	6.821417	3.106024

ZNF395	1914.835	2892.245	937.4249	0.324117	-1.62541	3.59E-06	0.001763	Down	32.73858	10.62488
ZNF573	47.75474	71.32411	24.18537	0.339091	-1.56026	0.047646	1	Down	1.002557	0.340399
ZNF703	66.09571	24.80839	107.383	4.328497	2.113866	0.00405	0.351002	Up	0.402652	1.74514
ZNF93	9.786853	18.60629	0.967415	0.051994	-4.26551	0.011661	0.637749	Down	0.361977	0.018845
