# Supplementary material for: Repressing HIF-1α-induced HDAC9 contributes to the synergistic effect of venetoclax and MENIN inhibitor in KMT2Ar AML
Source: Biomark Res. 2023 Dec 5;11:105. doi: 10.1186/s40364-023-00547-9 (PMC10696732; doi:10.1186/s40364-023-00547-9)
Supplement: Supplementary file 9 — Additional file 9: Table S8. Different expressed genes of MI-503 vs. DMSO in THP-1. [file 40364_2023_547_MOESM9_ESM.pdf]

gene_id	BaseMean	BaseMean_	BaseMean_	FoldChang	log2FoldCl	pValue	qValue	Regulation	Expression	Expression_THP_1MI_503
ABLIM1	4.044772	0	8.089544	Inf	Inf	0.040086	1	Up	0	0.036358
ABTB2	206.2003	276.9007	135.4999	0.489345	-1.03108	0.016499	0.770395	Down	3.344139	1.612602
ACVR1C	26.15745	11.86717	40.44772	3.408371	1.769082	0.038345	1	Up	0.077415	0.260017
ADAM28	276.6083	445.0189	108.1976	0.24313	-2.0402	5.33E-07	0.000502	Down	3.327174	0.797155
ADAMTS4	50.74715	73.18089	28.3134	0.386896	-1.36998	0.044524	1	Down	0.996393	0.379886
ADGRG5	259.8363	362.9376	156.7349	0.431851	-1.2114	0.002576	0.280616	Down	4.397561	1.87143
AHNAK2	106.0335	147.3507	64.71635	0.439199	-1.18705	0.026785	0.928445	Down	0.47036	0.203573
ALDOC	3308.52	1895.781	4721.26	2.490404	1.31638	3.01E-07	0.000345	Up	64.62647	158.6019
AMIGO2	132.5313	84.05913	181.0035	2.153288	1.106542	0.025934	0.914211	Up	1.030315	2.186252
ANK3	17.87867	28.679	7.078351	0.246813	-2.01851	0.041444	1	Down	0.08591	0.020895
ANXA4	6296.193	8938.946	3653.44	0.40871	-1.29085	1.07E-06	0.0008	Down	79.41607	31.98542
APOA1	6.561623	0.988931	12.13432	12.27014	3.617079	0.037537	1	Up	0.044907	0.542985
APOE	45.78666	19.77862	71.7947	3.629915	1.859936	0.009461	0.59242	Up	0.802168	2.869391
AQP1	105.7775	170.0961	41.45891	0.243738	-2.0366	0.00024	0.054083	Down	2.187192	0.525338
AQP3	341.5965	465.7865	217.4065	0.466751	-1.09927	0.002929	0.304641	Down	14.74128	6.7803
ARFGEF3	185.8574	107.7935	263.9214	2.448399	1.291838	0.003868	0.370996	Up	0.428897	1.034817
ARID5A	278.2094	392.6056	163.8133	0.417246	-1.26103	0.001379	0.189975	Down	4.40046	1.809333
ARRDC4	321.4617	457.875	185.0483	0.404146	-1.30705	0.000548	0.098884	Down	6.666455	2.654979
ARTN	187.2881	250.1995	124.3767	0.49711	-1.00836	0.02291	0.890726	Down	7.621008	3.733301
ASMTL	144.8625	246.2438	43.4813	0.176578	-2.50162	1.08E-06	0.0008	Down	5.548328	0.965444
ATF3	143.7465	210.6423	76.85067	0.36484	-1.45467	0.002928	0.304641	Down	4.061514	1.460219
BEST1	302.5718	429.196	175.9476	0.409947	-1.28649	0.000826	0.134956	Down	4.630039	1.870425
BEST4	6.428051	12.8561	0	0	#NAME?	0.008382	0.56935	Down	0.215532	0
BNIP3	2018.823	1259.898	2777.747	2.20474	1.140608	1.11E-05	0.00524	Up	45.68476	99.25598
BOLA2-SM	50.23042	74.16982	26.29102	0.354471	-1.49626	0.029362	0.966612	Down	2.131975	0.744715
BTG2	1151.84	1608.991	694.6896	0.431755	-1.21172	1.15E-05	0.005264	Down	35.13064	14.9469
C1QTNF12	19.62921	7.911447	31.34698	3.962231	1.986313	0.037137	1	Up	0.328057	1.280907
C2orf48	113.9947	73.18089	154.7125	2.114111	1.080051	0.038492	1	Up	2.278292	4.746409
C3AR1	53.62489	87.02592	20.22386	0.232389	-2.10539	0.002351	0.26146	Down	1.430232	0.327529
C3orf80	175.2095	245.2549	105.1641	0.428795	-1.22164	0.007274	0.529579	Down	5.633223	2.380316
C9orf139	478.3049	313.4911	643.1187	2.051474	1.036661	0.002014	0.244198	Up	3.968142	8.021971
CALD1	4.550368	0	9.100737	Inf	Inf	0.027917	0.945296	Up	0	0.093083
CAPS	128.9682	176.0297	81.90663	0.4653	-1.10377	0.027768	0.945296	Down	4.854853	2.226062
CASTOR3	359.4863	493.4765	225.496	0.456954	-1.12988	0.00193	0.238418	Down	3.215999	1.448159
CCL3	119.3952	173.0629	65.72754	0.37979	-1.39673	0.007077	0.52614	Down	12.85784	4.812151
CCL3L3	222.6114	346.1258	99.09691	0.286303	-1.80438	2.78E-05	0.010857	Down	26.47983	7.470838
CCL4	6.439182	11.86717	1.011193	0.085209	-3.55285	0.04256	1	Down	1.053522	0.088462
CCL4L2	41.22986	65.26944	17.19028	0.263374	-1.92481	0.009711	0.598945	Down	2.095903	0.543967
CD180	70.64821	101.8599	39.43653	0.387164	-1.36898	0.025901	0.914211	Down	2.099374	0.800964
CD83	89.5826	126.5832	52.58204	0.415395	-1.26744	0.025547	0.911281	Down	2.972029	1.216585
CD86	145.869	201.7419	89.99618	0.446096	-1.16458	0.015773	0.752741	Down	4.08546	1.795961
CDC20B	71.626	104.8267	38.42533	0.366561	-1.44788	0.018253	0.816585	Down	1.49968	0.541717
CDK6	11970.87	16228.36	7713.38	0.475303	-1.07308	0.000178	0.045355	Down	79.647	37.30504
CERKL	26.67417	10.87824	42.47011	3.904134	1.965003	0.02181	0.871094	Up	0.195787	0.753247
CFLAR	2961.147	3964.624	1957.67	0.493784	-1.01805	6.69E-05	0.021863	Down	12.69728	6.178402
CHN1	18.85647	31.64579	6.067158	0.191721	-2.38292	0.016484	0.770395	Down	0.566122	0.106957
CMPK2	662.2473	905.8607	418.6339	0.462139	-1.1136	0.000323	0.067256	Down	13.61405	6.199952
COL15A1	39.22504	18.78969	59.66039	3.175167	1.666832	0.025453	0.911281	Up	0.196608	0.615172
COL4A2	11.59533	2.966793	20.22386	6.816742	2.769082	0.02484	0.902122	Up	0.027182	0.182591
COLCA2	55.79841	28.679	82.91783	2.891239	1.531688	0.021192	0.861442	Up	0.414698	1.181528
CRYZ	247.9169	164.1625	331.6713	2.020384	1.014629	0.012365	0.663223	Up	3.995343	7.954556
CTGF	267.0306	397.5502	136.5111	0.343381	-1.54212	0.000129	0.035121	Down	10.04284	3.398289
CX3CR1	714.0168	978.0527	449.9809	0.460078	-1.12005	0.000218	0.050901	Down	15.83651	7.179914
CXCL8	55.15281	86.03699	24.26863	0.282072	-1.82586	0.006928	0.524719	Down	2.647905	0.736021
CXorf21	1935.155	2674.069	1196.241	0.447349	-1.16053	8.35E-06	0.004613	Down	82.16997	36.22324
CYP19A1	17.35082	30.65686	4.044772	0.131937	-2.92208	0.006318	0.49848	Down	0.308201	0.040071
DACH1	2683.742	3635.31	1732.174	0.476486	-1.06949	2.96E-05	0.011273	Down	19.00081	8.921749
DBNDD1	82.85574	50.43548	115.276	2.285613	1.192581	0.039975	1	Up	1.050465	2.365986
DDB2	526.5862	336.2365	716.9358	2.132237	1.092368	0.000857	0.135878	Up	10.77371	22.6375
DEPP1	130.8904	184.9301	76.85067	0.415566	-1.26685	0.011484	0.637822	Down	5.315725	2.17686
DPPA4	7.078351	0	14.1567	Inf	Inf	0.005256	0.440753	Up	0	0.255984
EGR2	33.26276	54.3912	12.13432	0.223093	-2.16428	0.00738	0.533272	Down	1.016316	0.223431
EGR3	73.24767	140.4282	6.067158	0.043205	-4.53267	2.66E-09	1.06E-05	Down	1.636865	0.06969
EIF3C	624.6392	878.1706	371.1078	0.422592	-1.24266	8.04E-05	0.025753	Down	13.95966	5.813317
ELOVL6	6.572754	0	13.14551	Inf	Inf	0.007229	0.528683	Up	0	0.109957
EMP1	63.8323	33.62365	94.04095	2.796869	1.483813	0.019687	0.832726	Up	0.652781	1.79915
ENAH	187.2518	118.6717	255.8318	2.155795	1.10822	0.01256	0.667219	Up	0.463522	0.984704
ENHO	62.16438	91.97057	32.35818	0.351832	-1.50704	0.019044	0.832726	Down	5.051872	1.751521
ENO2	337.0149	64.28051	609.7494	9.485758	3.245763	2.68E-15	4.30E-11	Up	1.570898	14.68413
EPAS1	628.851	863.3367	394.3653	0.456792	-1.13039	0.000314	0.067073	Down	9.362886	4.214598
EPHA2	4.044772	0	8.089544	Inf	Inf	0.040086	1	Up	0	0.110418
EPHB1	194.8879	293.7125	96.06333	0.327066	-1.61235	0.000296	0.064594	Down	2.45024	0.789718
EPSTI1	179.8758	279.8674	79.88425	0.285436	-1.80876	8.70E-05	0.026811	Down	2.238248	0.629571
ETV1	340.7621	225.4762	456.048	2.022599	1.01621	0.005876	0.470581	Up	1.670999	3.330535
ETV4	229.9334	99.88202	359.9847	3.604099	1.849639	1.46E-05	0.005992	Up	2.142117	7.607953
EVA1B	121.0011	165.1515	76.85067	0.465334	-1.10366	0.031191	0.989057	Down	6.00321	2.752813
EVPL	7.922578	14.83396	1.011193	0.068167	-3.87477	0.018575	0.826366	Down	0.131493	0.008833
EXPH5	40.7307	19.77862	61.68277	3.118659	1.640926	0.025859	0.914211	Up	0.086785	0.266711
FAM114A1	192.2855	120.6496	263.9214	2.187504	1.129285	0.01035	0.60945	Up	1.073817	2.314767
FAM131B	115.764	46.47975	185.0483	3.981267	1.993228	0.000203	0.050236	Up	0.558603	2.191557

FAM13B	1493.492	2069.832	917.152	0.443104	-1.17428	1.04E-05	0.005061	Down		19.24661	8.404046
FAM162A	668.1022	385.6831	950.5214	2.464514	1.301303	2.77E-05	0.010857	Up		27.25265	66.18629
FAM189B	1395.251	915.75	1874.752	2.047231	1.033674	0.000112	0.0315	Up		15.05412	30.37038
FAM222A	80.10983	114.716	45.50368	0.396664	-1.33401	0.023673	0.899324	Down		1.588206	0.620808
FAM234B	183.1959	119.6606	246.7311	2.061924	1.043991	0.01938	0.832726	Up		1.504044	3.056053
FAM46C	61.44902	20.76755	102.1305	4.917792	2.298011	0.000599	0.105396	Up		0.214987	1.04186
FAXDC2	4.450189	8.900378	0	0	#NAME?	0.031248	0.989057	Down		0.103217	0
FERMT1	116.0025	70.21409	161.7909	2.304251	1.204298	0.020622	0.85196	Up		0.796635	1.80891
FGFR3	169.3204	229.432	109.2088	0.475997	-1.07098	0.019505	0.832726	Down		3.044032	1.427845
FKBP4	638.4056	373.8159	902.9953	2.415615	1.272391	4.97E-05	0.016692	Up		4.392741	10.45662
FMNL2	114.4523	73.18089	155.7237	2.127929	1.08945	0.036596	1	Up		0.617809	1.295505
FOSB	26.76792	47.46868	6.067158	0.127814	-2.96788	0.001271	0.181524	Down		0.744385	0.093757
FOXD4	42.27445	62.30265	22.24625	0.357067	-1.48573	0.040361	1	Down		1.686863	0.593551
GAPT	82.12109	115.7049	48.53726	0.419492	-1.25329	0.031763	0.995847	Down		2.699494	1.115922
GEM	81.59323	117.6828	45.50368	0.386664	-1.37085	0.019487	0.832726	Down		2.909575	1.108642
GFRA2	39.24087	62.30265	16.17909	0.259685	-1.94516	0.010167	0.605947	Down		0.706332	0.180752
GJA1	14.1567	0	28.3134	Inf	Inf	9.99E-05	0.029185	Up		0	0.521339
GLIPR1	528.266	815.868	240.6639	0.294979	-1.76132	1.41E-07	0.000188	Down		12.14443	3.530176
GNAI1	178.1844	115.7049	240.6639	2.07998	1.05657	0.019045	0.832726	Up		1.8843	3.862223
GNAL	33.73026	12.8561	54.60442	4.247354	2.086565	0.009008	0.579428	Up		0.083636	0.350059
GOLGA8J	85.09901	120.6496	49.54846	0.410681	-1.28391	0.026177	0.917385	Down		1.186532	0.480188
GOLGA8S	73.13166	105.8156	40.44772	0.382247	-1.38742	0.022524	0.886349	Down		1.071615	0.403656
GPA33	144.3634	200.753	87.97379	0.438219	-1.19028	0.013993	0.708904	Down		4.143369	1.789258
GPR3	25.66298	10.87824	40.44772	3.718223	1.894613	0.028447	0.9511	Up		0.304558	1.115922
GPRIN3	64.8101	36.59044	93.02975	2.54246	1.346225	0.03274	0.998777	Up		0.140101	0.351012
GRAP	60.70324	87.02592	34.38056	0.395061	-1.33985	0.037509	1	Down		2.473897	0.963105
GTPBP6	72.33839	41.5351	103.1417	2.483242	1.312225	0.031	0.989057	Up		0.676602	1.655694
GUCA1B	61.31545	32.63472	89.99618	2.757682	1.463456	0.023025	0.890726	Up		0.851287	2.313387
GULP1	7.06722	0.988931	13.14551	13.29265	3.732556	0.02812	0.945701	Up		0.00815	0.106758
HBB	107.0272	193.8305	20.22386	0.104338	-3.26067	4.95E-08	7.93E-05	Down		18.33453	1.885124
HBG2	12.63991	0	25.27982	Inf	Inf	0.000219	0.050901	Up		0	2.530205
HDC	16.15683	1.977862	30.33579	15.33767	3.939007	0.001178	0.17471	Up		0.036036	0.544656
HELZ2	640.8851	872.2371	409.5332	0.46952	-1.09074	0.000472	0.089202	Down		4.900226	2.267246
HERC6	235.9906	325.3583	146.623	0.450651	-1.14992	0.005399	0.450379	Down		4.610114	2.047292
HILPDA	472.0614	239.3213	704.8015	2.945001	1.558268	5.20E-06	0.003201	Up		10.03619	29.1261
HK2	1605.042	828.7241	2381.359	2.873525	1.522822	1.17E-08	3.12E-05	Up		6.857437	19.41801
HLA-DRA	663.4341	441.0632	885.8051	2.008341	1.006004	0.001119	0.17071	Up		20.40389	40.38112
HMGA2	8.089544	0	16.17909	Inf	Inf	0.00283	0.302045	Up		0	0.155736
HMOX1	349.7511	549.8456	149.6566	0.272179	-1.87737	6.30E-07	0.000531	Down		20.47698	5.492231
HR	22.85672	35.60151	10.11193	0.284031	-1.81588	0.043126	1	Down		0.37371	0.104599
HSD17B7	371.5701	228.443	514.6972	2.253066	1.17189	0.001177	0.17471	Up		7.734109	17.17166
IFI44	232.5007	365.9044	99.09691	0.270827	-1.88455	9.80E-06	0.004905	Down		8.222602	2.19447
IFI44L	108.6553	183.9411	33.36937	0.181413	-2.46265	1.22E-05	0.005413	Down		1.838905	0.328743
IFI6	307.6945	423.2624	192.1267	0.453919	-1.13949	0.002802	0.302045	Down		24.19202	10.82127
IFIT1	226.2396	338.2144	114.2648	0.337847	-1.56556	0.000224	0.051354	Down		4.235813	1.410215
IFIT2	501.0926	759.4989	242.6863	0.319535	-1.64596	1.10E-06	0.0008	Down		12.86774	4.051803
IFIT3	492.003	758.51	225.496	0.297288	-1.75007	2.81E-07	0.000345	Down		15.82042	4.634722
IGF2BP1	9.089606	0.988931	17.19028	17.38269	4.11958	0.00924	0.584915	Up		0.006462	0.11069
IGFBP3	5.43912	10.87824	0	0	#NAME?	0.01592	0.756584	Down		0.244827	0
IL2RB	151.4083	203.7198	99.09691	0.486437	-1.03967	0.028686	0.955158	Down		2.822415	1.352931
IL31RA	325.5228	186.9079	464.1376	2.483242	1.312225	0.000494	0.092057	Up		1.141098	2.792351
IL3RA	57.9749	104.8267	11.12312	0.10611	-3.23637	9.60E-06	0.004905	Down		3.542908	0.370461
INHBE	26.82358	42.52403	11.12312	0.261573	-1.93472	0.02373	0.899324	Down		1.021918	0.263412
INSRR	20.32874	35.60151	5.055965	0.142015	-2.81588	0.004888	0.424689	Down		0.502766	0.070361
IQCD	162.6141	241.2991	83.92902	0.347821	-1.52358	0.001212	0.178065	Down		4.057999	1.390901
IRF7	271.8593	372.827	170.8916	0.458367	-1.12542	0.004434	0.410068	Down		10.32575	4.66405
IRX5	552.3916	738.7314	366.0519	0.495514	-1.013	0.00172	0.216962	Down		15.87192	7.750212
ISG15	160.2976	222.5095	98.08572	0.440816	-1.18175	0.011562	0.638549	Down		19.75353	8.580846
JCHAIN	4.550368	0	9.100737	Inf	Inf	0.027917	0.945296	Up		0	0.376891
JPH2	359.4821	134.4946	584.4695	4.345673	2.11958	2.01E-08	4.59E-05	Up		1.083822	4.641335
KCNJ11	25.66298	10.87824	40.44772	3.718223	1.894613	0.028447	0.9511	Up		0.163529	0.599181
KCNJ9	9.089606	0.988931	17.19028	17.38269	4.11958	0.00924	0.584915	Up		0.005396	0.092432
KIF26A	36.7908	55.38013	18.20147	0.328664	-1.60531	0.034805	1	Down		0.485313	0.157182
KIRREL3	20.351	33.62365	7.078351	0.210517	-2.24799	0.018955	0.832726	Down		0.430296	0.089265
KLF2	96.96618	144.3839	49.54846	0.343172	-1.543	0.005695	0.465808	Down		3.05667	1.033684
KY	6.933647	12.8561	1.011193	0.078655	-3.66832	0.032146	0.995847	Down		0.061402	0.004759
L1CAM	66.18688	93.94844	38.42533	0.409005	-1.28981	0.039349	1	Down		1.061243	0.427731
L1TD1	10.08967	1.977862	18.20147	9.202601	3.202042	0.018867	0.832726	Up		0.02936	0.266252
LAMC3	41.75772	63.29158	20.22386	0.319535	-1.64596	0.024513	0.902122	Down		0.595445	0.187494
LGI2	230.0382	135.4835	324.5929	2.395811	1.260514	0.002542	0.278894	Up		0.916017	2.16264
LHX9	181.1876	253.1663	109.2088	0.431372	-1.213	0.007046	0.52614	Down		1.741914	0.740469
LILRB2	153.3417	211.6312	95.05214	0.44914	-1.15476	0.014942	0.734436	Down		4.150867	1.837168
LIN28A	12.63991	0	25.27982	Inf	Inf	0.000219	0.050901	Up		0	0.366578
LINGO3	8.428175	14.83396	2.022386	0.136335	-2.87477	0.045506	1	Down		0.412382	0.055403
LOC10192	32.81282	49.44655	16.17909	0.327204	-1.61174	0.041131	1	Down		0.265378	0.085568
LOC10272	7.572816	0.988931	14.1567	14.31516	3.839472	0.021161	0.861442	Up		0.066468	0.93764
LOC10272	60.58253	7.911447	113.2536	14.31516	3.839472	4.06E-07	0.000433	Up		0.300106	4.233498
LOC10537	59.28193	33.62365	84.94021	2.526204	1.336971	0.039145	1	Up		0.991031	2.467082
LOC10537	197.7469	129.5499	265.9438	2.052828	1.037613	0.017229	0.783899	Up		0.938939	1.899406
LOC10798	108.9669	156.2511	61.68277	0.394767	-1.34093	0.011952	0.650832	Down		1.372527	0.533937
LOC11226	160.6555	100.871	220.4401	2.185367	1.127876	0.015671	0.752741	Up		0.804437	1.732384

LOX	185.6143	39.55724	331.6713	8.384592	3.067741	3.69E-10	2.95E-06	Up	0.422803	3.4934
LPL	466.1993	266.0224	666.3762	2.504963	1.324789	0.0001	0.029185	Up	4.203942	10.37734
LRGUK	68.68147	96.91523	40.44772	0.417352	-1.26067	0.041427	1	Down	0.279365	0.114895
MAP7	107.0113	150.3175	63.70516	0.423804	-1.23853	0.020633	0.85196	Down	0.744654	0.310991
MAPT	44.7866	18.78969	70.78351	3.767147	1.913472	0.008186	0.568577	Up	0.15609	0.579448
MARCKS	536.7824	283.8232	789.7417	2.782513	1.476389	7.49E-06	0.004285	Up	3.902977	10.70191
MATK	111.4299	72.19196	150.6678	2.087044	1.061461	0.043408	1	Up	1.446617	2.975178
MB	10.39491	19.77862	1.011193	0.051126	-4.28981	0.00502	0.429908	Down	0.797796	0.040194
MESP1	29.68549	12.8561	46.51488	3.618117	1.855239	0.024092	0.900675	Up	0.655127	2.335801
METTL25	134.9129	186.9079	82.91783	0.443629	-1.17257	0.017846	0.802859	Down	0.698706	0.305452
MS4A2	10.07854	2.966793	17.19028	5.79423	2.534617	0.048544	1	Up	0.042889	0.244891
MSMO1	2438.215	1417.138	3459.291	2.44104	1.287496	6.09E-07	0.000531	Up	34.79021	83.68738
MX1	526.9876	839.6023	214.3729	0.255327	-1.96958	5.44E-09	1.74E-05	Down	8.341604	2.098813
MXD4	1387.913	1971.928	803.8984	0.407671	-1.29452	1.56E-06	0.00109	Down	21.57922	8.669096
MYBPH	305.7659	459.8529	151.6789	0.329842	-1.60015	3.65E-05	0.013587	Down	15.07729	4.900696
MYO7A	11.39497	20.76755	2.022386	0.097382	-3.3602	0.010972	0.625377	Down	0.139773	0.013413
MYRF	60.77646	35.60151	85.9514	2.414263	1.271583	0.047649	1	Up	0.341116	0.811549
NCF1	92.40468	145.3728	39.43653	0.271278	-1.88215	0.001067	0.164311	Down	5.899975	1.577225
NEK8	90.61605	124.6053	56.62681	0.454449	-1.13781	0.043543	1	Down	2.18812	0.979907
NFIA	18.66255	3.955724	33.36937	8.435718	3.076511	0.003472	0.345354	Up	0.022152	0.184145
NOS3	61.78765	35.60151	87.97379	2.471069	1.305135	0.041194	1	Up	0.411497	1.002026
NR4A3	9.417106	16.81183	2.022386	0.120295	-3.05535	0.028079	0.945701	Down	0.142784	0.016926
NTN1	47.6913	75.15875	20.22386	0.269082	-1.89388	0.007639	0.543781	Down	0.729818	0.19352
NTRK1	38.66848	68.23623	9.100737	0.133371	-2.90648	0.00034	0.069307	Down	1.414253	0.185873
NUAK1	45.24767	22.74541	67.74993	2.97862	1.574644	0.026714	0.928098	Up	0.197455	0.579577
NUGGC	8.92264	15.82289	2.022386	0.127814	-2.96788	0.035704	1	Down	0.187536	0.023621
NUTM2B	23.81226	40.54617	7.078351	0.174575	-2.51808	0.006451	0.501563	Down	0.72931	0.125465
NUTM2D	26.34024	40.54617	12.13432	0.299272	-1.74047	0.041245	1	Down	0.433139	0.127738
OAS1	603.0369	910.8054	295.2684	0.324184	-1.62512	4.41E-07	0.000441	Down	16.47286	5.262455
OAS2	1333.808	2017.419	650.1971	0.322292	-1.63356	2.41E-09	1.06E-05	Down	19.36438	6.150075
OASL	76.53727	117.6828	35.39175	0.300739	-1.73342	0.004308	0.405808	Down	3.119263	0.924419
OLFML3	95.50505	139.4393	51.57084	0.369844	-1.43501	0.010215	0.605947	Down	3.495645	1.274015
OSMR	78.57078	116.6938	40.44772	0.346614	-1.5286	0.010477	0.612644	Down	0.980124	0.334777
P3H3	61.33771	30.65686	92.01856	3.001565	1.585715	0.014187	0.713025	Up	0.679381	2.009505
P4HA1	3506.086	2221.139	4791.032	2.157016	1.109037	1.46E-05	0.005992	Up	43.6225	92.72396
P4HA2	456.1653	259.0999	653.2307	2.521154	1.334084	9.89E-05	0.029185	Up	2.835908	7.045624
PADI2	37.21378	17.80076	56.62681	3.181146	1.669547	0.027744	0.945296	Up	0.22959	0.719722
PAPPA2	9.405975	17.80076	1.011193	0.056806	-4.13781	0.00839	0.56935	Down	0.087867	0.004919
PFKFB4	914.3688	561.7128	1267.025	2.255645	1.17354	4.49E-05	0.016331	Up	5.482296	12.186
PGR	73.39411	37.57937	109.2088	2.906085	1.539077	0.011627	0.639921	Up	0.132186	0.378548
PIK3IP1	65.65903	95.9263	35.39175	0.368947	-1.43851	0.022479	0.886349	Down	2.296867	0.83508
PIK3R3	65.17569	93.94844	36.40295	0.387478	-1.36781	0.030023	0.975362	Down	0.900167	0.343715
PLEKHA8	213.848	136.4725	291.2236	2.133937	1.093517	0.010166	0.605947	Up	0.503963	1.059761
PLEKHH2	163.6334	105.8156	221.4513	2.092803	1.065437	0.021474	0.868489	Up	0.845921	1.74456
PLEKHO1	147.4081	199.764	95.05214	0.475822	-1.07151	0.025508	0.911281	Down	3.552183	1.665589
PLGLB1	42.22992	66.25837	18.20147	0.274705	-1.86405	0.011372	0.637822	Down	1.303023	0.352733
PLK2	116.023	158.2289	73.81709	0.466521	-1.09999	0.034165	1	Down	2.945401	1.354077
POU2F2	367.9812	502.3769	233.5856	0.464961	-1.10482	0.002257	0.256803	Down	2.997541	1.373439
POU5F1	13.65111	0	27.30221	Inf	Inf	0.000129	0.035121	Up	0	0.45182
POU6F1	137.0706	85.04806	189.0931	2.223368	1.152746	0.019102	0.832726	Up	0.419144	0.918337
PPP1R27	706.8439	402.4949	1011.193	2.512313	1.329016	1.34E-05	0.005812	Up	29.8288	73.84778
PRDM1	157.9032	210.6423	105.1641	0.499254	-1.00215	0.032394	0.996122	Down	1.464988	0.720749
PRDM11	44.20778	70.21409	18.20147	0.259228	-1.94771	0.007549	0.539758	Down	0.349675	0.089325
PRF1	14.36176	26.70113	2.022386	0.075742	-3.72277	0.002847	0.302045	Down	0.620759	0.046332
PRLR	394.7608	234.3766	555.145	2.368602	1.244036	0.000471	0.089202	Up	1.065268	2.486445
PROC	3.955724	7.911447	0	0	#NAME?	0.044443	1	Down	0.205919	0
PTCRA	8.417044	15.82289	1.011193	0.063907	-3.96788	0.014202	0.713025	Down	0.561037	0.035332
PTPRF	5835.06	3551.251	8118.869	2.2862	1.192951	5.41E-06	0.003212	Up	25.25918	56.90644
PTPRZ1	4.044772	0	8.089544	Inf	Inf	0.040086	1	Up	0	0.057133
PYROXD2	140.8576	197.7862	83.92902	0.424342	-1.2367	0.011416	0.637822	Down	1.175987	0.491752
RBM20	12.90063	21.75648	4.044772	0.185911	-2.42731	0.035275	1	Down	0.103327	0.01893
RCVRN	4.450189	8.900378	0	0	#NAME?	0.031248	0.989057	Down	0.448531	0
RGS13	4.450189	8.900378	0	0	#NAME?	0.031248	0.989057	Down	0.337188	0
RHBDL3	6.439182	11.86717	1.011193	0.085209	-3.55285	0.04256	1	Down	0.1003	0.008422
RHOBTB1	311.8049	192.8415	430.7682	2.233794	1.159496	0.002267	0.256803	Up	1.057106	2.326964
RIMBP3	155.8363	214.598	97.07453	0.452355	-1.14447	0.01529	0.746579	Down	2.192399	0.977298
ROBO4	61.16431	90.98164	31.34698	0.344542	-1.53725	0.017456	0.792009	Down	1.404055	0.47671
RSAD2	278.998	412.3842	145.6118	0.353097	-1.50186	0.000154	0.040683	Down	6.372336	2.217283
RTL5	249.8802	349.0926	150.6678	0.431598	-1.21224	0.002878	0.303295	Down	4.764016	2.026193
RUFY4	326.3893	199.764	453.0145	2.267748	1.18126	0.001633	0.210863	Up	2.697553	6.028269
S100A9	1123.061	707.0856	1539.036	2.17659	1.12207	5.00E-05	0.016692	Up	72.56352	155.6407
SALL2	6.572754	0	13.14551	Inf	Inf	0.007229	0.528683	Up	0	0.094547
SAMD11	457.8691	646.7608	268.9773	0.415884	-1.26575	0.000213	0.050901	Down	14.99495	6.145326
SCML1	123.4529	82.08127	164.8245	2.008064	1.005805	0.047531	1	Up	0.530604	1.049969
SCN3B	3.955724	7.911447	0	0	#NAME?	0.044443	1	Down	0.072653	0
SEMA3G	136.4742	182.9522	89.99618	0.491911	-1.02353	0.037314	1	Down	2.142234	1.03844
SEMA5B	4.550368	0	9.100737	Inf	Inf	0.027917	0.945296	Up	0	0.082103
SEMA6A	416.4055	602.2589	230.552	0.382812	-1.38529	8.34E-05	0.026204	Down	4.321093	1.630074
SEMA6B	23.64059	10.87824	36.40295	3.3464	1.74261	0.048397	1	Up	0.159797	0.526955
SERPINB2	11.59533	2.966793	20.22386	6.816742	2.769082	0.02484	0.902122	Up	0.077561	0.52101
SH2D2A	75.55947	114.716	36.40295	0.317331	-1.65594	0.006434	0.501563	Down	3.661864	1.145098

SIGLEC1	288.3975	475.6758	101.1193	0.21258	-2.23392	3.65E-08	6.49E-05	Down	3.393966	0.710982
SIGLEC10	86.59354	122.6274	50.55965	0.412303	-1.27822	0.025972	0.914211	Down	2.13943	0.869245
SLC16A14	151.4276	67.2473	235.608	3.503605	1.80884	0.000204	0.050236	Up	0.764586	2.639789
SLC26A1	7.428113	13.84503	1.011193	0.073037	-3.77524	0.024386	0.902122	Down	0.20015	0.014405
SLC27A5	37.22491	16.81183	57.638	3.42842	1.777544	0.019666	0.832726	Up	0.376652	1.272512
SLC2A3	181.5742	84.05913	279.0893	3.320154	1.73125	0.000155	0.040683	Up	1.263954	4.135401
SLC43A2	244.6731	407.4395	81.90663	0.201028	-2.31453	6.58E-08	9.58E-05	Down	2.616703	0.518368
SLC7A2	285.075	186.9079	383.2421	2.050433	1.035928	0.007729	0.547762	Up	1.360983	2.749958
SLITRK5	495.2901	286.79	703.7903	2.454027	1.295151	0.000109	0.031296	Up	2.840257	6.868547
SOCS3	107.0559	146.3618	67.74993	0.462894	-1.11125	0.037218	1	Down	3.166468	1.444389
SP9	489.949	671.4841	308.4139	0.459302	-1.12249	0.000787	0.129881	Down	27.32719	12.36861
SPAG4	105.5185	58.34692	152.6901	2.616936	1.387878	0.010093	0.605947	Up	2.153951	5.554651
STARD4	2737.678	1673.271	3802.086	2.272247	1.18412	3.93E-06	0.002622	Up	9.725223	21.77625
STON2	192.1215	269.9781	114.2648	0.423237	-1.24046	0.005018	0.429908	Down	1.112098	0.463826
STS	866.1199	1164.961	567.2793	0.486951	-1.03815	0.000342	0.069307	Down	9.13906	4.38546
SULT1A4	47.15231	78.12554	16.17909	0.207091	-2.27166	0.0018	0.22527	Down	3.311455	0.675784
SYNC	59.21984	84.05913	34.38056	0.409005	-1.28981	0.046626	1	Down	0.823945	0.332089
TBC1D3B	5.933585	11.86717	0	0	#NAME?	0.011509	0.637822	Down	0.306454	0
TBC1D3H	4.944655	9.889309	0	0	#NAME?	0.022202	0.881939	Down	0.147651	0
TDGF1	7.078351	0	14.1567	Inf	Inf	0.005256	0.440753	Up	0	0.376681
THBD	635.3211	423.2624	847.3797	2.00202	1.001456	0.001324	0.184355	Up	6.216005	12.26331
THY1	5.561561	0	11.12312	Inf	Inf	0.013968	0.708904	Up	0	0.160934
TIGD1	149.5769	96.91523	202.2386	2.086758	1.061263	0.026107	0.916947	Up	2.189511	4.502432
TLE6	68.33814	37.57937	99.09691	2.637003	1.398899	0.024279	0.902122	Up	0.746215	1.939111
TMEM119	100.5388	141.4171	59.66039	0.421875	-1.24511	0.022645	0.887836	Down	2.877605	1.196309
TMEM56	128.2926	56.36906	200.2162	3.551881	1.828583	0.000381	0.07589	Up	0.464425	1.625555
TNF	236.2179	350.0815	122.3544	0.349502	-1.51663	0.000284	0.063194	Down	12.36851	4.259862
TNFRSF18	4.044772	0	8.089544	Inf	Inf	0.040086	1	Up	0	0.177457
TNFRSF19	12.88949	22.74541	3.033579	0.133371	-2.90648	0.015444	0.749534	Down	0.21843	0.028708
TP53INP1	374.9132	605.2257	144.6006	0.23892	-2.0654	2.95E-08	5.90E-05	Down	6.24567	1.470482
TPD52L1	14.10105	4.944655	23.25744	4.703552	2.233751	0.041248	1	Up	0.099929	0.463174
TREML2	46.73577	70.21409	23.25744	0.331236	-1.59407	0.023752	0.899324	Down	1.074602	0.350763
TRIB2	70.855	38.56831	103.1417	2.67426	1.41914	0.020879	0.857432	Up	0.518097	1.365346
TUBB4A	102.0573	51.42441	152.6901	2.969215	1.570082	0.004228	0.400668	Up	1.118674	3.273204
TXNIP	12861.64	17853.17	7870.115	0.440825	-1.18172	4.74E-05	0.016692	Down	302.6488	131.4718
UCHL1	5.055965	0	10.11193	Inf	Inf	0.019654	0.832726	Up	0	0.52495
ULBP2	264.635	161.1957	368.0742	2.283399	1.191183	0.002848	0.302045	Up	6.764703	15.22154
UNC5B	1140.345	1597.123	683.5665	0.427999	-1.22432	9.70E-06	0.004905	Down	12.0458	5.080494
UNC5CL	72.38292	37.57937	107.1865	2.852268	1.51211	0.013492	0.69257	Up	0.711385	1.999509
UNC79	117.9118	170.0961	65.72754	0.386414	-1.37178	0.00838	0.56935	Down	0.504762	0.192206
UNC80	18.35088	31.64579	5.055965	0.159767	-2.64596	0.009754	0.598945	Down	0.112693	0.017742
USH1C	91.38409	56.36906	126.3991	2.242349	1.165011	0.038193	1	Up	0.364033	0.8044
VAMP7	80.53925	31.64579	129.4327	4.090045	2.032117	0.000786	0.129881	Up	0.699203	2.818119
WSB1	6829.735	4389.864	9269.606	2.111593	1.078332	4.84E-05	0.016692	Up	59.33351	123.4634
XAGE1B	551.5345	814.8791	288.19	0.35366	-1.49957	4.74E-06	0.003036	Down	50.2102	17.4987
YAP1	154.5884	100.871	208.3058	2.065072	1.046192	0.026593	0.925896	Up	0.961207	1.956051
YPEL3	392.749	547.8677	237.6304	0.433737	-1.20511	0.000712	0.121388	Down	20.22523	8.644656
YPEL4	14.61777	3.955724	25.27982	6.390695	2.675973	0.016373	0.770395	Up	0.126067	0.793923
ZNF235	87.627	120.6496	54.60442	0.452587	-1.14373	0.04479	1	Down	1.845069	0.822892
