# Supplementary material for: Repressing HIF-1α-induced HDAC9 contributes to the synergistic effect of venetoclax and MENIN inhibitor in KMT2Ar AML
Source: Biomark Res. 2023 Dec 5;11:105. doi: 10.1186/s40364-023-00547-9 (PMC10696732; doi:10.1186/s40364-023-00547-9)
Supplement: Supplementary file 10 — Additional file 10: Table S9. Different expressed genes of VEN vs. DMSO in THP-1. [file 40364_2023_547_MOESM10_ESM.pdf]

gene_id	BaseMean	BaseMean_	BaseMean_	FoldChang	log2FoldCl	pValue	qValue	Regulation	Expression	Expression_THP_1Venetoclax
AARD	29.58723	16.88274	42.29172	2.505028	1.324827	0.031032	1	Up	0.557385	1.363041
ABCG1	40.88331	57.59992	24.1667	0.419561	-1.25305	0.021783	0.988313	Down	0.296058	0.121259
ADAM28	326.6599	446.896	206.4239	0.461906	-1.11433	9.26E-05	0.029824	Down	3.327174	1.500271
ADM2	62.23683	27.80686	96.66679	3.476365	1.79758	0.000213	0.053454	Up	0.400594	1.359473
ALDH1L2	1516.457	939.4746	2093.44	2.228309	1.15595	6.65E-08	7.50E-05	Up	6.749039	14.68109
ARMC12	38.39363	53.62752	23.15975	0.431863	-1.21135	0.029879	1	Down	1.078127	0.454524
AS3MT	26.42103	37.73788	15.10419	0.400239	-1.32106	0.039552	1	Down	0.901992	0.352422
ATF3	154.0988	211.5308	96.66679	0.456987	-1.12978	0.001426	0.218526	Down	4.061514	1.811893
ATOH8	59.85608	80.44127	39.27088	0.488193	-1.03448	0.030242	1	Down	0.728941	0.347396
BAALC	21.57319	10.92412	32.22226	2.949643	1.56054	0.025622	1	Up	0.226094	0.651027
BMP8A	70.18164	43.69649	96.66679	2.212232	1.145503	0.01162	0.76764	Up	0.381261	0.823368
BOLA2B	100.5769	16.88274	184.2711	10.91476	3.448209	2.91E-13	2.30E-09	Up	0.97885	10.42969
C15orf62	9.958709	15.88963	4.027783	0.253485	-1.98003	0.048436	1	Down	0.376429	0.093149
C2orf91	15.55921	6.951715	24.1667	3.476365	1.79758	0.026263	1	Up	0.03987	0.135306
C3AR1	62.82846	87.39299	38.26394	0.437838	-1.19153	0.011518	0.764107	Down	1.430232	0.611309
CACFD1	33.91083	46.6758	21.14586	0.453037	-1.1423	0.049855	1	Down	0.581131	0.257009
CAMK4	23.42096	34.75858	12.08335	0.347636	-1.52435	0.024342	1	Down	0.168478	0.057176
CBSL	44.77448	4.965511	84.58344	17.03419	4.090361	9.79E-10	1.95E-06	Up	0.072635	1.207835
CCDC154	41.89718	56.60682	27.18754	0.480287	-1.05803	0.049777	1	Down	1.523423	0.71427
CCDC194	32.39349	47.6689	17.11808	0.359104	-1.47753	0.013503	0.830743	Down	2.119756	0.743099
CCL3L3	259.8867	347.5858	172.1877	0.495382	-1.01339	0.0008	0.143568	Down	26.47983	12.80549
CCL4L2	38.31057	65.54474	11.0764	0.16899	-2.56499	2.33E-05	0.010808	Down	2.095903	0.345759
CDC42EP1	68.18852	40.71719	95.65985	2.349373	1.232276	0.007306	0.63736	Up	0.910117	2.087324
CDHR2	6.462086	11.91723	1.006946	0.084495	-3.56499	0.014183	0.830743	Down	0.16058	0.013245
CEL	26.40719	39.72409	13.09029	0.32953	-1.60152	0.013561	0.830743	Down	0.981698	0.315801
CERKL	21.06972	10.92412	31.21532	2.857467	1.514737	0.031555	1	Up	0.195787	0.546144
CERS1	20.0697	9.931022	30.20837	3.041819	1.604934	0.026022	1	Up	0.156072	0.463446
CES4A	46.87653	64.55164	29.20143	0.452373	-1.14441	0.027596	1	Down	1.052915	0.464976
CHAC1	146.4534	80.44127	212.4656	2.64125	1.401221	0.000113	0.035666	Up	0.779878	2.010838
CHDH	24.58019	12.91033	36.25005	2.807833	1.489457	0.024577	1	Up	0.090058	0.24685
CIB3	13.04876	5.958613	20.13892	3.379799	1.756938	0.043962	1	Up	0.494163	1.630428
CILP	4.46896	8.937919	0	0	#NAME?	0.011513	0.764107	Down	0.081507	0
CLEC5A	9.041746	2.979306	15.10419	5.069699	2.3419	0.029436	1	Up	0.049654	0.245739
CLU	11.45528	17.87584	5.034729	0.28165	-1.82802	0.049595	1	Down	0.342224	0.094094
COL15A1	37.62895	18.86894	56.38896	2.988454	1.579399	0.005493	0.555982	Up	0.196608	0.573574
COL5A2	70.82866	95.33781	46.3195	0.485846	-1.04143	0.021166	0.972438	Down	0.793562	0.376375
CPNE7	165.4262	103.2826	227.5697	2.203369	1.139711	0.00101	0.175299	Up	1.405831	3.023856
CSF2RA	67.27156	27.80686	106.7363	3.838486	1.940537	4.81E-05	0.018065	Up	0.550397	2.062417
CTXN1	11.55219	3.972409	19.13197	4.816214	2.267899	0.017212	0.910796	Up	0.185458	0.87195
CXCL8	51.25551	86.39989	16.11113	0.186472	-2.42297	7.67E-06	0.004323	Down	2.647905	0.48201
DHCR7	2045.204	1358.564	2731.844	2.010832	1.007793	1.54E-06	0.00143	Up	28.62011	56.18082
DHRSX	29.6426	8.937919	50.34729	5.632999	2.493903	0.000157	0.045165	Up	0.204511	1.124597
EGR2	32.34504	54.62062	10.06946	0.184353	-2.43946	0.000129	0.03932	Down	1.016316	0.182902
EGR3	82.5936	141.0205	24.1667	0.17137	-2.54481	4.94E-08	6.00E-05	Down	1.636865	0.273835
EIF3C	633.264	881.8747	384.6533	0.436177	-1.19702	9.45E-07	0.000994	Down	13.95966	5.943992
ENO2	134.9843	64.55164	205.4169	3.182211	1.670029	9.23E-06	0.004857	Up	1.570898	4.879978
EPAS1	636.8921	866.9782	406.8061	0.469223	-1.09165	7.13E-06	0.004179	Down	9.362886	4.288741
EPSTI1	189.8643	281.0479	98.68068	0.351117	-1.50998	7.15E-06	0.004179	Down	2.238248	0.767186
ETV4	199.1796	100.3033	298.0559	2.971546	1.571214	2.27E-06	0.001987	Up	2.142117	6.213931
FAS	28.40724	41.71029	15.10419	0.362121	-1.46545	0.019516	0.950888	Down	0.25821	0.091278
FAXDC2	4.972433	8.937919	1.006946	0.11266	-3.14995	0.049407	1	Down	0.103217	0.011352
FBXO32	153.6091	209.5446	97.67374	0.466124	-1.10121	0.001881	0.26758	Down	1.778836	0.809428
FERMT1	111.783	70.51025	153.0558	2.170688	1.118152	0.004215	0.45893	Up	0.796635	1.688097
FOSB	33.90391	47.6689	20.13892	0.422475	-1.24306	0.033203	1	Down	0.744385	0.307001
GAL	118.8039	74.48266	163.1252	2.19011	1.131003	0.003186	0.384888	Up	5.369033	11.47897
GAL3ST1	8.455212	14.89653	2.013892	0.135192	-2.88692	0.013759	0.830743	Down	0.157415	0.020775
GAL3ST4	49.36621	68.52405	30.20837	0.440843	-1.18166	0.020718	0.967818	Down	1.590756	0.684587
GOLGA8S	78.30461	106.2619	50.34729	0.473804	-1.07764	0.013805	0.830743	Down	1.071615	0.495653
GPNMB	140.9498	75.47576	206.4239	2.734969	1.451525	7.98E-05	0.027392	Up	1.55555	4.153145
GPR3	23.58709	10.92412	36.25005	3.318348	1.730465	0.01102	0.743554	Up	0.304558	0.986582
GPR65	18.06965	7.944817	28.19448	3.548789	1.827327	0.016336	0.901644	Up	0.103597	0.358896
GPR84	38.09782	23.83445	52.36118	2.196869	1.135449	0.041819	1	Up	0.625455	1.341348
GPR89B	324.3211	204.579	444.0631	2.170619	1.118106	8.99E-05	0.029824	Up	3.571048	7.566936
GPT2	631.0344	407.1719	854.8969	2.099597	1.070113	1.10E-05	0.005601	Up	5.537105	11.34905
GRIN3A	35.86243	55.61372	16.11113	0.289697	-1.78738	0.002281	0.307782	Down	0.294474	0.083278
GTF2IRD2	61.82844	86.39989	37.25699	0.431216	-1.21352	0.010526	0.740588	Down	1.356743	0.571127
GTPBP6	75.23022	41.71029	108.7501	2.607274	1.382542	0.001999	0.276852	Up	0.676602	1.722108
HAPLN4	3.020837	0	6.041675	Inf	Inf	0.049252	1	Up	0	0.101096
HEATR4	3.475858	6.951715	0	0	#NAME?	0.0313	1	Down	0.094798	0
HERC6	241.4036	326.7306	156.0766	0.477692	-1.06585	0.000561	0.105663	Down	4.610114	2.149812
HHAT	19.43471	28.79996	10.06946	0.349634	-1.51608	0.037564	1	Down	0.269639	0.092032
HIST1H2A(	26.41411	38.73098	14.09724	0.363978	-1.45808	0.023721	1	Down	4.182733	1.486198
HIST2H2BI	141.6435	192.6618	90.62512	0.470384	-1.08809	0.002721	0.343678	Down	5.110347	2.346628
HMOX1	356.1346	552.1648	160.1044	0.289958	-1.78609	4.26E-10	1.35E-06	Down	20.47698	5.79617
HSD11B1	34.0908	20.85515	47.32645	2.269294	1.182243	0.041912	1	Up	0.785264	1.739591
IFI27	26.39335	41.71029	11.0764	0.265556	-1.91291	0.00377	0.419166	Down	2.970346	0.770023
IFI44	222.9948	367.4478	78.54177	0.213749	-2.22601	2.41E-11	9.51E-08	Down	8.222602	1.715754
IFI44L	106.9592	184.717	29.20143	0.158087	-2.66121	7.81E-10	1.95E-06	Down	1.838905	0.28379
IFI6	302.6455	425.0477	180.2433	0.424054	-1.23768	2.24E-05	0.01072	Down	24.19202	10.01462
IFIT1	236.7824	339.6409	133.9238	0.39431	-1.3426	1.84E-05	0.009079	Down	4.235813	1.630479

IFIT2	543.973	762.7025	325.2435	0.426436	-1.2296	1.12E-06	0.001108	Down	12.86774	5.35669
IFIT3	569.657	761.7094	377.6047	0.495733	-1.01236	4.62E-05	0.017778	Down	15.82042	7.65609
IL3RA	57.16567	105.2688	9.062512	0.086089	-3.53802	9.88E-10	1.95E-06	Down	3.542908	0.297748
INMT	6.958637	12.91033	1.006946	0.077995	-3.68047	0.009439	0.713067	Down	0.297482	0.02265
IRF9	601.0574	807.392	394.7227	0.488886	-1.03243	2.65E-05	0.011971	Down	28.37179	13.54052
ITGB1BP2	24.42099	35.75168	13.09029	0.366145	-1.44951	0.029078	1	Down	1.626618	0.581406
KCNJ9	7.041699	0.993102	13.09029	13.18122	3.720412	0.008387	0.679419	Up	0.005396	0.069434
KCTD8	10.45526	16.88274	4.027783	0.238574	-2.06749	0.035903	1	Down	0.271843	0.063312
KIF19	4.027783	0	8.055566	Inf	Inf	0.017306	0.910796	Up	0	0.125833
LGI4	14.44843	21.84825	7.04862	0.322617	-1.6321	0.049378	1	Down	0.420595	0.132462
LOC10537	8.462134	13.90343	3.020837	0.217273	-2.20242	0.045794	1	Down	0.478864	0.101568
LOC10798	299.7977	400.2202	199.3753	0.498164	-1.00531	0.000544	0.105663	Down	6.472567	3.147673
LOC10798	6.462086	11.91723	1.006946	0.084495	-3.56499	0.014183	0.830743	Down	0.138109	0.011392
LOC10798	33.90391	47.6689	20.13892	0.422475	-1.24306	0.033203	1	Down	0.669716	0.276205
LOC11226	25.42101	36.74478	14.09724	0.383653	-1.38213	0.034159	1	Down	0.634081	0.237478
LOC11226	5.468984	9.931022	1.006946	0.101394	-3.30196	0.032446	1	Down	0.919282	0.090992
LOX	60.64335	39.72409	81.56261	2.053228	1.037894	0.028888	1	Up	0.422803	0.847455
LRRC7	4.965511	9.931022	0	0	#NAME?	0.007106	0.631713	Down	0.036869	0
MAPT	31.58728	18.86894	44.30561	2.348071	1.231476	0.039523	1	Up	0.15609	0.357788
MATK	115.7969	72.49646	159.0974	2.194555	1.133928	0.003357	0.395556	Up	1.446617	3.099137
MGAT3	49.12577	30.78617	67.46537	2.191418	1.131865	0.026653	1	Up	0.102693	0.219689
MS4A4E	91.27031	124.1378	58.40285	0.470468	-1.08783	0.009033	0.697365	Down	1.405758	0.645627
MX1	547.4401	843.1437	251.7364	0.298569	-1.74386	1.18E-11	6.20E-08	Down	8.341604	2.43128
NBEA	57.80066	86.39989	29.20143	0.33798	-1.56499	0.001463	0.219981	Down	0.380504	0.125543
NEO1	126.1742	172.7998	79.54871	0.460352	-1.11919	0.002951	0.366903	Down	1.371166	0.616199
NFIA	10.04177	3.972409	16.11113	4.055759	2.019972	0.043256	1	Up	0.022152	0.087704
NGEF	47.14648	25.82066	68.47231	2.651842	1.406995	0.007058	0.631713	Up	0.415647	1.076002
NOS3	55.6363	35.75168	75.52093	2.112375	1.078866	0.027413	1	Up	0.411497	0.848551
NPIPA2	35.13235	15.88963	54.37507	3.422047	1.774859	0.002578	0.330922	Up	0.184544	0.616492
NPIPA7	76.81496	103.2826	50.34729	0.487471	-1.03661	0.018472	0.945388	Down	2.295537	1.09238
NPIPB6	14.44151	22.84135	6.041675	0.264506	-1.91863	0.022803	1	Down	0.220685	0.056983
NR4A2	90.28413	121.1585	59.4098	0.490348	-1.02812	0.0138	0.830743	Down	1.979525	0.947559
NR4A3	8.441368	16.88274	0	0	#NAME?	0.000307	0.070356	Down	0.142784	0
NUTM2B	26.40027	40.71719	12.08335	0.296763	-1.75262	0.007358	0.637755	Down	0.72931	0.211282
OAS1	666.2648	914.6471	417.8825	0.456878	-1.13012	2.78E-06	0.002312	Down	16.47286	7.347009
OAS2	1420.274	2025.928	814.6191	0.402097	-1.31439	1.21E-09	2.13E-06	Down	19.36438	7.601073
OASL	84.76669	118.1792	51.35423	0.434546	-1.20242	0.004971	0.516389	Down	3.119263	1.323208
OTUD7A	4.972433	8.937919	1.006946	0.11266	-3.14995	0.049407	1	Down	0.046364	0.005099
PLS1	45.6153	28.79996	62.43064	2.167733	1.116187	0.033024	1	Up	0.351592	0.744022
PPP1R27	612.4267	404.1926	820.6608	2.030371	1.021743	2.96E-05	0.012973	Up	29.8288	59.12239
PPP4R4	4.46896	8.937919	0	0	#NAME?	0.011513	0.764107	Down	0.106815	0
PSAT1	2546.239	1491.639	3600.838	2.414014	1.271434	1.38E-09	2.17E-06	Up	38.98692	91.87547
PTH1R	5.53128	0.993102	10.06946	10.1394	3.3419	0.029545	1	Up	0.012238	0.121132
RBPMS2	5.034729	0	10.06946	Inf	Inf	0.006405	0.612895	Up	0	0.287651
RGPD2	133.1052	189.6825	76.52788	0.403452	-1.30953	0.000441	0.090459	Down	1.026207	0.404174
RPS10	71.67822	45.6827	97.67374	2.13809	1.096323	0.014901	0.849333	Up	3.061	6.388961
RSAD2	286.6105	414.1236	159.0974	0.384179	-1.38015	3.56E-06	0.002443	Down	6.372336	2.389861
SCARF2	65.29738	94.3447	36.25005	0.38423	-1.37996	0.003242	0.384888	Down	1.55392	0.582855
SEMA6A	430.2817	604.7992	255.7642	0.422891	-1.24164	3.29E-06	0.002358	Down	4.321093	1.783869
SEMA6B	20.06278	10.92412	29.20143	2.673114	1.418521	0.047665	1	Up	0.159797	0.416991
SIGLEC1	310.8377	477.6821	143.9932	0.301442	-1.73005	4.88E-09	7.00E-06	Down	3.393966	0.998738
SIGLEC11	22.41402	34.75858	10.06946	0.289697	-1.78738	0.010408	0.740259	Down	0.647359	0.183076
SLC24A3	15.93809	24.82755	7.04862	0.283903	-1.81653	0.023672	1	Down	0.376435	0.104328
SLC26A4	6.965559	11.91723	2.013892	0.16899	-2.56499	0.040874	1	Down	0.117646	0.019408
SLC27A5	39.15321	16.88274	61.42369	3.638254	1.863246	0.001075	0.180627	Up	0.376652	1.337746
SLC2A3	131.825	84.41368	179.2363	2.123309	1.086314	0.003398	0.397153	Up	1.263954	2.619904
SLC7A5	3349.092	2023.942	4674.242	2.309474	1.207564	8.66E-09	1.14E-05	Up	22.24864	50.16
SMN1	389.068	522.3717	255.7642	0.489621	-1.03026	0.000152	0.044333	Down	16.68561	7.975229
SPDYE18	18.93124	28.79996	9.062512	0.314671	-1.66808	0.024501	1	Down	1.367302	0.420012
SSC4D	10.54524	3.972409	17.11808	4.309244	2.107435	0.031835	1	Up	0.072675	0.305724
SULT1A4	47.78657	78.45507	17.11808	0.21819	-2.19635	5.57E-05	0.020001	Down	3.311455	0.705332
SULT1B1	4.027783	0	8.055566	Inf	Inf	0.017306	0.910796	Up	0	0.27967
TBC1D3B	6.462086	11.91723	1.006946	0.084495	-3.56499	0.014183	0.830743	Down	0.306454	0.025278
TBC1D3H	5.468984	9.931022	1.006946	0.101394	-3.30196	0.032446	1	Down	0.147651	0.014615
TGM4	21.42784	31.77927	11.0764	0.348542	-1.5206	0.03007	1	Down	0.596962	0.203115
TLDC2	3.972409	7.944817	0	0	#NAME?	0.018863	0.946561	Down	0.177853	0
TLE6	58.13983	37.73788	78.54177	2.081245	1.057447	0.028194	1	Up	0.746215	1.516101
TMEM74B	4.027783	0	8.055566	Inf	Inf	0.017306	0.910796	Up	0	0.095273
TNFSF4	39.89713	54.62062	25.17364	0.460882	-1.11753	0.041857	1	Down	0.2483	0.111714
TP53INP1	432.2748	607.7785	256.7712	0.422475	-1.24306	3.12E-06	0.002349	Down	6.24567	2.575851
TRIM73	15.94501	23.83445	8.055566	0.33798	-1.56499	0.048585	1	Down	1.034903	0.341453
TRPM8	3.475858	6.951715	0	0	#NAME?	0.0313	1	Down	0.045194	0
TSKU	65.16076	41.71029	88.61123	2.124445	1.087086	0.019239	0.949253	Up	0.604732	1.25415
TTC21A	40.88331	57.59992	24.1667	0.419561	-1.25305	0.021783	0.988313	Down	0.754919	0.309198
TTC39A	5.027807	0.993102	9.062512	9.125458	3.189897	0.045373	1	Up	0.013007	0.115872
TTN	57.82142	83.42058	32.22226	0.386263	-1.37235	0.004888	0.511152	Down	0.04207	0.015863
TUBB4A	80.19573	51.64131	108.7501	2.105875	1.07442	0.013289	0.830743	Up	1.118674	2.29973
UBQLNL	7.46211	12.91033	2.013892	0.155991	-2.68047	0.02839	1	Down	0.32616	0.049667
ULBP2	249.6012	161.8757	337.3268	2.083864	1.059261	0.000535	0.10549	Up	6.764703	13.76128
UNC5C	143.6504	193.6549	93.64596	0.483571	-1.0482	0.003693	0.413523	Down	0.6736	0.317982
VASN	9.448314	16.88274	2.013892	0.119287	-3.06749	0.006716	0.631713	Down	0.354772	0.041313

VPS37D	9.545219	2.979306	16.11113	5.407679	2.435009	0.02106	0.972254	Up		0.085779	0.452826
XAGE1B	450.4429	818.3162	82.56955	0.100902	-3.30898	5.81E-29	9.18E-25	Down		50.2102	4.945744
