# Supplementary material for: Repressing HIF-1α-induced HDAC9 contributes to the synergistic effect of venetoclax and MENIN inhibitor in KMT2Ar AML
Source: Biomark Res. 2023 Dec 5;11:105. doi: 10.1186/s40364-023-00547-9 (PMC10696732; doi:10.1186/s40364-023-00547-9)
Supplement: Supplementary file 11 — Additional file 11: Table S10. Different expressed genes of MI-503 plus VEN vs. DMSO in THP-1. [file 40364_2023_547_MOESM11_ESM.pdf]

gene_id	BaseMean	BaseMean_	BaseMean_	FoldChang	log2FoldCl	pValue	qValue	Regulation	Expression	Expression_THP_1MI_503_P_Venetoclax
A2M	78.15091	124.4927	31.80908	0.25551	-1.96855	0.002184	0.176799	Down	1.29745	0.341205
ABCB11	78.89022	131.7548	26.02561	0.197531	-2.33985	0.000339	0.049137	Down	1.321645	0.268699
ABCC3	27.83912	10.3744	45.30385	4.36689	2.126606	0.017791	0.658493	Up	0.082037	0.368723
ABI3BP	13.37642	23.86111	2.891735	0.12119	-3.04465	0.012741	0.526391	Down	0.202471	0.025255
ABTB2	456.103	290.4831	621.723	2.140307	1.097818	0.001832	0.158015	Up	3.344139	7.366773
ACAT2	1169.305	1593.507	745.1037	0.467587	-1.09669	0.000229	0.037319	Down	47.11654	22.6753
ACPP	551.6181	741.7693	361.4669	0.487304	-1.03711	0.002066	0.169833	Down	9.687449	4.858766
ADAM19	85.33087	41.49758	129.1642	3.112571	1.638107	0.007877	0.388485	Up	0.262505	0.840956
ADAMTS14	118.6599	165.9903	71.32946	0.429721	-1.21853	0.023738	0.780477	Down	1.493119	0.660385
ADAMTS4	41.277	76.77052	5.78347	0.075335	-3.73055	2.92E-05	0.007327	Down	0.996393	0.077258
ADCK1	166.8348	111.006	222.6636	2.005869	1.004228	0.034538	0.933216	Up	1.835306	3.789029
ADM	12.37575	22.82367	1.927823	0.084466	-3.56549	0.007203	0.364356	Down	0.81024	0.070439
AGMAT	124.5904	170.1401	79.04075	0.464563	-1.10605	0.036206	0.950753	Down	3.044881	1.4559
AHNAK	26159.23	34915.03	17403.42	0.498451	-1.00448	0.026901	0.827363	Down	101.5342	52.08965
AHNAK2	90.30205	154.5785	26.02561	0.168365	-2.57034	4.67E-05	0.010689	Down	0.47036	0.081508
AIF1L	25.01286	39.4227	10.60303	0.268957	-1.89455	0.037388	0.959437	Down	0.60206	0.166663
ALDOC	1296.09	1988.772	603.4087	0.303408	-1.72067	9.85E-09	1.11E-05	Down	64.62647	20.18153
ANK3	16.48874	30.08575	2.891735	0.096116	-3.37907	0.003795	0.247788	Down	0.08591	0.008499
ANOS1	240.6803	127.6051	353.7556	2.772269	1.471067	0.000538	0.065784	Up	1.071506	3.057363
ANXA4	6850.28	9377.416	4323.144	0.461017	-1.11711	0.000488	0.061579	Down	79.41607	37.68268
AP5B1	3807.739	2265.768	5349.709	2.361102	1.23946	3.23E-05	0.007835	Up	19.39215	47.12568
APOL3	113.0643	157.6908	68.43772	0.433999	-1.20423	0.028045	0.841445	Down	1.264685	0.564922
AQP1	98.37696	178.4396	18.31432	0.102636	-3.28439	3.21E-07	0.000179	Down	2.187192	0.231049
AQP3	341.1901	488.634	193.7462	0.396506	-1.33459	0.000465	0.060095	Down	14.74128	6.015916
ARHGAP24	36.28972	17.63647	54.94296	3.115303	1.639373	0.042541	1	Up	0.062245	0.199583
ARL4C	117.0262	174.2898	59.76252	0.342892	-1.54418	0.004749	0.282618	Down	2.451479	0.865171
ARRDC4	348.6073	480.3345	216.8801	0.451519	-1.14714	0.002353	0.185706	Down	6.666455	3.098046
ASAH1	2467.951	1478.351	3457.551	2.338788	1.225761	2.39E-05	0.006076	Up	25.83453	62.18825
ASS1	1084.174	1503.25	665.099	0.442441	-1.17644	9.13E-05	0.018633	Down	39.42886	17.95504
ATF3	140.3686	220.9746	59.76252	0.27045	-1.88657	0.000278	0.042748	Down	4.061514	1.130554
ATG16L2	2640.484	1627.743	3653.225	2.244351	1.166298	5.84E-05	0.012791	Up	10.19733	23.55559
ATP6V0D2	26.87521	10.3744	43.37602	4.181065	2.063871	0.022413	0.759074	Up	0.247081	1.06327
AVIL	69.18562	102.7065	35.66473	0.347249	-1.52596	0.020266	0.718531	Down	1.085834	0.38808
AXL	76.33338	127.6051	25.0617	0.196401	-2.34813	0.00038	0.052473	Down	1.469932	0.297137
BAZ2B	1186.023	1602.844	769.2015	0.479898	-1.0592	0.000362	0.051815	Down	7.926451	3.915115
BCAT1	1720.238	2519.941	920.5356	0.365301	-1.45284	6.87E-07	0.000339	Down	10.80672	4.063132
BICDL1	244.683	131.7548	357.6112	2.714217	1.440536	0.000648	0.074624	Up	1.570955	4.388591
BNIP3	875.8013	1321.698	429.9046	0.325267	-1.6203	2.60E-07	0.000152	Down	45.68476	15.29425
BOLA2B	60.86946	17.63647	104.1025	5.90268	2.56137	0.000414	0.055816	Up	0.97885	5.946786
BTG2	1192.411	1687.914	696.9081	0.412881	-1.2762	1.89E-05	0.004982	Down	35.13064	14.92891
C12orf75	146.009	203.3381	88.67987	0.43612	-1.1972	0.016545	0.625299	Down	8.40221	3.771522
C16orf71	9.712644	2.074879	17.35041	8.36213	3.063871	0.030258	0.881059	Up	0.039393	0.339045
C3	646.1815	282.1835	1010.179	3.579866	1.839906	3.82E-08	3.55E-05	Up	3.103633	11.43547
C3AR1	51.43081	91.29468	11.56694	0.126699	-2.98052	0.00014	0.026072	Down	1.430232	0.186508
C4orf47	8.781472	16.59903	0.963912	0.05807	-4.10605	0.011808	0.505227	Down	0.284005	0.016975
C6orf223	1108.241	1855.979	360.5029	0.194239	-2.3641	4.02E-14	2.11E-10	Down	16.4279	3.284238
C9orf50	4.668478	9.336956	0	0	#NAME?	0.026367	0.822237	Down	0.202858	0
CACNB4	336.5624	466.8478	206.2771	0.441851	-1.17837	0.001985	0.165786	Down	1.298985	0.590741
CADM1	99.37709	57.05917	141.695	2.483299	1.312258	0.023047	0.76901	Up	0.699545	1.787975
CAMP	28.3946	12.44927	44.33993	3.561648	1.832545	0.03689	0.955892	Up	0.94322	3.45765
CAPN11	15.37777	25.93599	4.819558	0.185825	-2.42798	0.02784	0.840101	Down	0.510801	0.097695
CBSL	30.06508	5.187198	54.94296	10.59203	3.404907	0.00043	0.056562	Up	0.072635	0.791847
CCDC144N	675.3473	1064.413	286.2817	0.268957	-1.89455	1.15E-08	1.21E-05	Down	10.35518	2.866541
CCL3	102.8249	181.5519	24.09779	0.132732	-2.91341	2.24E-06	0.000843	Down	12.85784	1.756553
CCL3L3	196.9745	363.1038	30.84517	0.084949	-3.55727	2.74E-12	7.22E-09	Down	26.47983	2.3152
CCL4	6.706593	12.44927	0.963912	0.077427	-3.69102	0.035738	0.941602	Down	1.053522	0.083956
CCL4L2	41.46484	68.47101	14.45867	0.211165	-2.24356	0.004813	0.283441	Down	2.095903	0.455522
CD180	63.54921	106.8563	20.24214	0.189433	-2.40024	0.000656	0.074994	Down	2.099374	0.40932
CD1D	1351.117	712.7209	1989.514	2.791434	1.481007	6.17E-07	0.000314	Up	10.54509	30.29665
CD300A	418.8062	666.0362	171.5763	0.257608	-1.95675	1.20E-07	9.05E-05	Down	16.33829	4.331944
CD38	1212.694	682.6352	1742.752	2.552977	1.352181	5.99E-06	0.002053	Up	6.767006	17.78117
CD86	157.8701	211.6377	104.1025	0.49189	-1.02359	0.034411	0.931528	Down	4.08546	2.068359
CD99	694.2534	945.1074	443.3993	0.469152	-1.09187	0.000698	0.078714	Down	12.19908	5.890577
CDK6	12174.09	17024.38	7323.8	0.430195	-1.21694	0.000778	0.084715	Down	79.647	35.26561
CFAP74	4.149758	8.299516	0	0	#NAME?	0.037705	0.961337	Down	0.063255	0
CFD	4913.517	3181.827	6645.207	2.088488	1.062459	0.000492	0.061592	Up	156.8543	337.1672
CLEC5A	19.38852	3.112319	35.66473	11.45922	3.518436	0.001867	0.160112	Up	0.049654	0.58563
CNR1	37.03304	11.41183	62.65425	5.490288	2.456882	0.003438	0.232896	Up	0.05276	0.298135
COCH	79.85815	118.2681	41.4482	0.35046	-1.51268	0.015668	0.607873	Down	2.065483	0.745035
COL27A1	1025.821	1502.212	549.4296	0.365747	-1.45108	2.16E-06	0.000832	Down	9.428706	3.549358
CPZ	55.95223	96.48188	15.42259	0.15985	-2.64521	0.000382	0.052473	Down	2.144062	0.352749
CRB1	17.08904	6.224637	27.95344	4.490774	2.166964	0.039954	0.983716	Up	0.060328	0.278841
CRB2	6.187873	11.41183	0.963912	0.084466	-3.56549	0.047739	1	Down	0.080457	0.006995
CRISPLD2	32.76897	13.48671	52.05123	3.859445	1.948393	0.021174	0.737688	Up	0.156896	0.623236
CSPG4	250.517	459.5857	41.4482	0.090186	-3.47095	9.82E-14	3.87E-10	Down	3.129229	0.290465
CTGF	256.7209	417.0507	96.39116	0.231126	-2.11325	7.30E-07	0.000349	Down	10.04284	2.389032
CTRC	6.784145	1.03744	12.53085	12.07863	3.594385	0.04108	1	Up	0.016788	0.208711
CXCL8	49.46622	90.25724	8.675205	0.096116	-3.37907	3.59E-05	0.008584	Down	2.647905	0.261949
CYP51A1	1643.684	2386.111	901.2574	0.37771	-1.40465	1.60E-06	0.000665	Down	40.96228	15.92426
DCANP1	61.62138	106.8563	16.3865	0.153351	-2.70509	0.000192	0.032537	Down	1.923924	0.303662
DCHS1	187.2125	278.0338	96.39116	0.346689	-1.52829	0.000941	0.098334	Down	1.45865	0.520484
DDIT4	2490.204	3683.948	1296.461	0.351922	-1.50667	2.53E-07	0.000152	Down	118.891	43.06376
DENND1B	1999.54	2676.594	1322.487	0.494093	-1.01715	0.000422	0.056397	Down	6.184212	3.144924
DEPP1	108.5675	194.0012	23.13388	0.119246	-3.06799	4.97E-07	0.000261	Down	5.315725	0.652414
DES	54.28578	90.25724	18.31432	0.202912	-2.30107	0.001763	0.152895	Down	2.266267	0.4733
DHCR7	959.7437	1419.217	500.2701	0.352497	-1.50432	1.16E-06	0.000524	Down	28.62011	10.3835

DNHD1	479.4631	650.4746	308.4517	0.474195	-1.07645	0.001958	0.165279	Down	2.341434	1.142762
DOC2A	380.6807	215.7874	545.574	2.528294	1.338164	0.000309	0.045191	Up	2.78785	7.254607
DPP4	142.521	213.7125	71.32946	0.333764	-1.5831	0.001904	0.16159	Down	3.089908	1.061455
DRAM1	432.6096	253.1352	612.0839	2.418011	1.273821	0.00038	0.052473	Up	3.898558	9.702401
DUSP27	348.3746	514.57	182.1793	0.354042	-1.49801	8.46E-05	0.018031	Down	6.978591	2.542958
EGR1	1682.383	3151.741	213.0245	0.067589	-3.88706	1.40E-32	2.21E-28	Down	56.72831	3.946351
EGR2	30.93937	57.05917	4.819558	0.084466	-3.56549	0.000214	0.035866	Down	1.016316	0.088354
EGR3	76.54994	147.3164	5.78347	0.039259	-4.67084	3.89E-09	4.73E-06	Down	1.636865	0.06614
ELANE	19142.82	11737.59	26548.05	2.261798	1.17747	0.004124	0.261396	Up	392.7256	914.2393
EPSTI1	203.1865	293.5954	112.7777	0.384126	-1.38035	0.002021	0.167852	Down	2.238248	0.88491
ERICH4	13.85838	23.86111	3.855646	0.161587	-2.62962	0.023897	0.782441	Down	0.773601	0.128659
EVI2A	469.1261	753.1811	185.071	0.245719	-2.02492	1.98E-08	1.95E-05	Down	14.12402	3.572023
EVPL	8.744708	15.56159	1.927823	0.123883	-3.01295	0.037966	0.963323	Down	0.131493	0.016766
FADS1	5227.202	7260.002	3194.403	0.44	-1.18442	0.00012	0.022875	Down	89.14307	40.36988
FADS2	9445.575	15836.51	3054.636	0.192886	-2.37418	2.04E-11	4.59E-08	Down	232.0591	46.06971
FAM72A	220.7208	298.7826	142.6589	0.477467	-1.06653	0.013425	0.546076	Down	3.086526	1.516808
FASN	10590.15	14828.12	6352.178	0.428387	-1.22301	0.000482	0.061315	Down	98.10962	43.25785
FBLN1	162.2404	226.1618	98.31898	0.434728	-1.20181	0.012483	0.519814	Down	2.62831	1.176011
FCER1G	1140.776	1536.448	745.1037	0.484952	-1.04409	0.000461	0.060095	Down	146.7424	73.24391
FCGR2B	262.1121	365.1787	159.0454	0.435528	-1.19916	0.003423	0.232879	Down	6.118286	2.7426
FCRLA	26.1606	44.6099	7.711293	0.172861	-2.53232	0.006596	0.341317	Down	1.065597	0.189586
FDFT1	7298.665	10062.13	4535.204	0.45072	-1.1497	0.00039	0.053017	Down	129.4635	60.05809
FDPS	8023.738	11713.73	4333.747	0.369972	-1.43451	1.57E-05	0.004606	Down	350.3875	133.424
FFAR2	6.187873	11.41183	0.963912	0.084466	-3.56549	0.047739	1	Down	0.232962	0.020253
FGL1	20.9734	34.2355	7.711293	0.225243	-2.15045	0.027498	0.832013	Down	0.7596	0.176097
FIGN	2263.789	3210.875	1316.703	0.410076	-1.28604	9.30E-06	0.002878	Down	9.079595	3.832198
FOS	2139.805	3370.641	908.9687	0.269672	-1.89072	1.57E-10	2.47E-07	Down	88.16301	24.47033
FOSB	27.30833	49.7971	4.819558	0.096784	-3.36909	0.000619	0.072332	Down	0.744385	0.074151
FRMD3	91.66982	43.57246	139.7672	3.207696	1.681537	0.005225	0.297671	Up	0.214968	0.709714
GAL	46.61527	77.80796	15.42259	0.198213	-2.33487	0.002502	0.192623	Down	5.369033	1.095333
GBE1	1672.434	2326.977	1017.891	0.437431	-1.19287	4.13E-05	0.009585	Down	42.3834	19.08191
GBP2	130.1045	203.3381	56.87079	0.279686	-1.83812	0.000555	0.067133	Down	2.777019	0.799404
GDF5	7.266101	1.03744	13.49476	13.00776	3.7013	0.031851	0.895628	Up	0.022583	0.302347
GEM	68.95699	123.4553	14.45867	0.117117	-3.09398	1.66E-05	0.004753	Down	2.909575	0.350724
GIMAP4	39.38996	64.32125	14.45867	0.224788	-2.15336	0.007399	0.372823	Down	1.807174	0.41811
GIPC3	460.8955	629.7258	292.0652	0.463797	-1.10843	0.001595	0.144661	Down	8.133836	3.882761
GLIPR1	608.6772	855.8876	361.4669	0.42233	-1.24356	0.000176	0.030859	Down	12.14443	5.278931
GPAT3	144.8607	75.73308	213.9884	2.82556	1.498537	0.003099	0.214514	Up	0.753526	2.19139
GPER1	16.89717	28.01087	5.78347	0.206472	-2.27598	0.031285	0.893183	Down	0.506592	0.107656
GPNMB	47.61595	78.8454	16.3865	0.207831	-2.26652	0.003022	0.21131	Down	1.55555	0.332744
GPRC5C	234.281	327.8309	140.7311	0.42928	-1.22001	0.004066	0.25874	Down	3.419144	1.510687
GRAP2	37.27832	59.13405	15.42259	0.260807	-1.93894	0.016442	0.625299	Down	0.821516	0.220522
GRM8	4.149758	8.299516	0	0	#NAME?	0.037705	0.961337	Down	0.067658	0
GRN	9210.77	5960.09	12461.45	2.090816	1.064066	0.001677	0.148651	Up	122.601	263.8315
GSDME	265.1009	163.9154	366.2864	2.234606	1.16002	0.004516	0.273095	Up	3.326934	7.651773
GSG1L	76.43508	35.27294	117.5972	3.333921	1.73722	0.006904	0.352606	Up	0.370484	1.271281
HAL	370.8985	198.1509	543.6462	2.743596	1.456068	0.000103	0.020018	Up	2.508889	7.084652
HDAC9	2363.408	3329.143	1397.672	0.419829	-1.25213	1.58E-05	0.004606	Down	13.62086	5.885648
HGF	16.89717	28.01087	5.78347	0.206472	-2.27598	0.031285	0.893183	Down	0.207572	0.044111
HMGCR	3837.282	5261.893	2412.671	0.458518	-1.12495	0.000156	0.028336	Down	58.96515	27.82713
HMGCS1	6399.526	9795.504	3003.549	0.306625	-1.70545	1.15E-07	9.04E-05	Down	78.23788	24.69118
HOMER3	1161.978	679.5229	1644.433	2.419982	1.274996	2.05E-05	0.005312	Up	16.85222	41.97457
HOXA7	34.64796	52.90942	16.3865	0.309709	-1.69102	0.03852	0.97394	Down	1.479916	0.471745
HPSE	476.8679	278.0338	675.702	2.430287	1.281127	0.000255	0.039858	Up	3.324918	8.316786
HPX	16.08836	5.187198	26.98953	5.203103	2.379372	0.029475	0.869494	Up	0.180401	0.966093
ID1	551.2855	365.1787	737.3924	2.019264	1.01383	0.002615	0.196521	Up	12.1536	25.25894
IDI1	3010.286	4134.196	1886.375	0.456286	-1.13199	0.000102	0.020018	Down	36.66792	17.22028
IFI16	836.3413	1119.397	553.2853	0.494271	-1.01663	0.001104	0.111004	Down	12.37938	6.297682
IFI44	237.7121	383.8526	91.5716	0.238559	-2.06758	2.14E-06	0.000832	Down	8.222602	2.018936
IFI44L	125.8812	192.9638	58.79861	0.304713	-1.71448	0.001381	0.131333	Down	1.838905	0.576723
IFIT1	241.9842	354.8043	129.1642	0.364043	-1.45782	0.000572	0.06804	Down	4.235813	1.587109
IFIT2	572.3628	796.7535	347.9721	0.436737	-1.19516	0.000364	0.051815	Down	12.86774	5.784149
IFITM1	466.814	674.3357	259.2922	0.384515	-1.37889	9.21E-05	0.018633	Down	53.08629	21.00935
IL12RB2	392.5595	537.3937	247.7253	0.460975	-1.11724	0.002227	0.178033	Down	5.738399	2.72261
IL3RA	59.80385	109.9686	9.639116	0.087653	-3.51205	6.38E-06	0.002054	Down	3.542908	0.319628
IL7R	44.05844	73.65821	14.45867	0.196294	-2.34891	0.002815	0.203761	Down	0.898754	0.181579
INHBE	23.75082	44.6099	2.891735	0.064823	-3.94736	0.000291	0.043351	Down	1.021918	0.06818
INSIG1	5670.842	8069.205	3272.48	0.405552	-1.30204	3.06E-05	0.007543	Down	143.0933	59.72864
INSRR	23.97543	37.34782	10.60303	0.283899	-1.81655	0.048104	1	Down	0.502766	0.146909
IRF7	284.7192	391.1147	178.3236	0.455937	-1.13309	0.004514	0.273095	Down	10.32575	4.845555
ITGA6	186.6358	98.55675	274.7148	2.787377	1.478908	0.001395	0.131835	Up	0.915424	2.626244
ITGB7	965.3275	597.5652	1333.09	2.230869	1.157606	0.000158	0.028418	Up	11.13187	25.55989
JAML	12.82094	21.78623	3.855646	0.176976	-2.49837	0.035388	0.940599	Down	0.464748	0.084654
JPH2	75.8474	141.0918	10.60303	0.07515	-3.73409	3.29E-07	0.000179	Down	1.083822	0.083831
KCNMB1	293.2065	399.4142	186.9989	0.468183	-1.09486	0.005605	0.311339	Down	14.43337	6.955049
KCNN4	291.4177	430.5374	152.298	0.353739	-1.49924	0.000182	0.031509	Down	7.784009	2.834025
KCNQ3	838.2243	1145.333	531.1153	0.463721	-1.10867	0.000382	0.052473	Down	2.630975	1.255714
KIF26A	33.86786	58.09661	9.639116	0.165915	-2.59148	0.00273	0.200389	Down	0.485313	0.082875
KIRREL1	240.5097	326.7934	154.2259	0.471937	-1.08333	0.009846	0.451728	Down	2.344414	1.138768
KLF2	101.2767	151.4662	51.08732	0.337285	-1.56796	0.006551	0.340087	Down	3.05667	1.061116
KLF6	612.7127	874.5615	350.8638	0.401188	-1.31765	7.13E-05	0.015413	Down	10.58872	4.372286
KLHDC7B	7.262077	14.52415	0	0	#NAME?	0.005118	0.293498	Down	0.170051	0
KLHL30	9.300192	17.63647	0.963912	0.054654	-4.19352	0.00905	0.423812	Down	0.270147	0.015196
KYNU	273.6302	404.6014	142.6589	0.352591	-1.50393	0.000229	0.037319	Down	1.632665	0.592496
LAMC3	39.94545	66.39613	13.49476	0.203246	-2.2987	0.004412	0.268846	Down	0.595445	0.124561
LARGE1	49.89477	20.74879	79.04075	3.809415	1.929569	0.009477	0.436036	Up	0.148738	0.583174
LDHA	11255.07	15176.7	7333.44	0.483204	-1.0493	0.003039	0.21131	Down	335.5459	166.878

LDLR	1431.177	2019.895	842.4588	0.417081	-1.2616	1.76E-05	0.004864	Down	21.57701	9.262498
LGALS1	7465.677	10572.55	4358.808	0.412276	-1.27832	9.00E-05	0.018633	Down	1069.475	453.8119
LGI4	52.37808	22.82367	81.93249	3.589803	1.843905	0.011512	0.501883	Up	0.420595	1.554001
LHX9	152.0705	265.5845	38.55646	0.145176	-2.78413	1.44E-07	9.89E-05	Down	1.741914	0.260278
LOC10192	14.48739	28.01087	0.963912	0.034412	-4.86094	0.000768	0.084164	Down	1.788544	0.063347
LOC10272	7.266101	1.03744	13.49476	13.00776	3.7013	0.031851	0.895628	Up	0.066468	0.88988
LOC10272	173.6557	113.0809	234.2305	2.071353	1.050574	0.025039	0.813561	Up	1.042949	2.223486
LOC10537	50.6381	14.52415	86.75205	5.97295	2.578444	0.000804	0.086359	Up	0.478864	2.943867
LOC10798	691.4818	418.0881	964.8755	2.307828	1.206536	0.000192	0.032537	Up	6.472567	15.37435
LOC10798	5.187198	10.3744	0	0	#NAME?	0.018654	0.681492	Down	0.148061	0
LOC10798	31.64593	49.7971	13.49476	0.270995	-1.88366	0.026091	0.822237	Down	0.669716	0.186796
LOC10798	120.0322	163.9154	76.14902	0.464563	-1.10605	0.038795	0.975835	Down	1.372527	0.65627
LOC33986	17.52618	32.16063	2.891735	0.089915	-3.47529	0.002578	0.1955	Down	0.777433	0.071947
LOC64517	7.225313	13.48671	0.963912	0.071471	-3.80649	0.026901	0.827363	Down	0.314569	0.02314
LOXL2	179.6235	240.686	118.5611	0.492597	-1.02152	0.027126	0.827363	Down	3.565752	1.807838
LRMP	1954.72	2921.43	988.0094	0.338194	-1.56408	9.02E-08	7.49E-05	Down	53.59118	18.65417
LSP1	59.17887	92.33212	26.02561	0.28187	-1.8269	0.008979	0.421724	Down	1.483965	0.430515
LSS	1984.728	3115.431	854.0257	0.274128	-1.86708	2.63E-10	3.77E-07	Down	34.87021	9.838395
LY9	7.744032	14.52415	0.963912	0.066366	-3.91341	0.020351	0.718531	Down	0.19177	0.013099
LYZ	4473.656	2506.454	6440.857	2.569709	1.361605	7.35E-06	0.00232	Up	93.32239	246.8236
MAML2	167.836	234.4613	101.2107	0.431673	-1.21199	0.010799	0.482784	Down	1.23361	0.548088
MAMLD1	11.67723	3.112319	20.24214	6.503879	2.7013	0.032974	0.914572	Up	0.033053	0.221256
MANEAL	230.2203	145.2415	315.1991	2.170172	1.117809	0.008793	0.416672	Up	2.416197	5.396888
MAP2K6	362.098	493.8212	230.3749	0.466515	-1.10001	0.003182	0.218357	Down	1.949894	0.936253
MARCKS	597.0915	297.7451	896.4378	3.010755	1.590126	2.34E-06	0.000859	Up	3.902977	12.09451
MLPH	8.781472	16.59903	0.963912	0.05807	-4.10605	0.011808	0.505227	Down	0.138313	0.008267
MMAB	466.7157	630.7632	302.6682	0.479844	-1.05936	0.002451	0.189581	Down	6.24074	3.082148
MNDA	554.6466	283.221	826.0723	2.916706	1.54434	6.11E-06	0.002053	Up	9.052324	27.175
MORN4	8.744708	15.56159	1.927823	0.123883	-3.01295	0.037966	0.963323	Down	0.314041	0.040042
MPO	94.38633	133.8297	54.94296	0.410544	-1.28439	0.028467	0.844486	Down	2.349616	0.992827
MROH7	487.4956	319.5314	655.4599	2.051316	1.03655	0.002765	0.202029	Up	4.108415	8.674089
MS4A2	16.97875	3.112319	30.84517	9.910673	3.308983	0.004372	0.26746	Up	0.042889	0.437491
MS4A3	553.5764	266.622	840.5309	3.15252	1.656505	1.37E-06	0.000599	Up	8.915564	28.92834
MSMO1	1089.37	1486.651	692.0885	0.465535	-1.10304	0.000236	0.038018	Down	34.79021	16.66966
MTSS1	147.896	215.7874	80.00466	0.370757	-1.43145	0.004239	0.264406	Down	0.622006	0.237356
MTUS2	4.668478	9.336956	0	0	#NAME?	0.026367	0.822237	Down	0.054137	0
MUC1	235.3225	316.4191	154.2259	0.48741	-1.03679	0.014068	0.563505	Down	9.71723	4.874768
MVD	955.8795	1323.773	587.9861	0.444174	-1.1708	0.000135	0.025373	Down	34.65693	15.84384
MX1	646.6702	880.7862	412.5542	0.468393	-1.09421	0.000801	0.086359	Down	8.341604	4.021397
MYO1B	80.89559	120.343	41.4482	0.344417	-1.53777	0.013682	0.555083	Down	1.230795	0.436302
MYRFL	11.67723	3.112319	20.24214	6.503879	2.7013	0.032974	0.914572	Up	0.02699	0.180669
NATD1	144.5218	93.36956	195.6741	2.095694	1.067428	0.033075	0.915763	Up	1.100949	2.374718
NCF1	106.615	152.5036	60.72643	0.398197	-1.32845	0.018049	0.665523	Down	5.899975	2.418047
NECTIN1	944.5666	596.5277	1292.605	2.166882	1.115621	0.00028	0.042748	Up	5.507196	12.28239
NEO1	319.1862	180.5145	457.858	2.536406	1.342786	0.000557	0.067133	Up	1.371166	3.579529
NES	10.78282	18.67391	2.891735	0.154854	-2.69102	0.037264	0.959383	Down	0.189645	0.030226
NFIA	12.67791	4.149758	21.20606	5.110191	2.353377	0.048555	1	Up	0.022152	0.11651
NKG7	76.55799	120.343	32.77299	0.27233	-1.87657	0.003584	0.239643	Down	7.090561	1.987435
NLRC4	92.42174	132.7923	52.05123	0.391975	-1.35117	0.022409	0.759074	Down	2.07114	0.835574
NMB	75.07938	105.8188	44.33993	0.419017	-1.25492	0.048002	1	Down	4.278612	1.845236
NPTX1	23.97543	37.34782	10.60303	0.283899	-1.81655	0.048104	1	Down	0.387732	0.113296
NPTXR	670.9904	383.8526	958.1281	2.496083	1.319666	5.25E-05	0.011687	Up	3.725977	9.572297
NR4A1	24.71473	44.6099	4.819558	0.108038	-3.21039	0.001353	0.130166	Down	0.31175	0.034666
NREP	2568.045	3854.088	1282.002	0.332634	-1.58799	6.05E-08	5.31E-05	Down	63.83331	21.85403
NTNG2	894.6351	574.7415	1214.529	2.113174	1.079411	0.00048	0.061315	Up	4.152749	9.03208
NTRK1	43.98491	71.58333	16.3865	0.228915	-2.12712	0.00617	0.327871	Down	1.414253	0.33321
NYAP2	6.706593	12.44927	0.963912	0.077427	-3.69102	0.035738	0.941602	Down	0.049735	0.003963
OAS1	681.1262	955.4818	406.7707	0.425723	-1.23201	0.000145	0.02666	Down	16.47286	7.21794
OAS2	1459.176	2116.377	801.9745	0.378937	-1.39997	2.00E-06	0.000811	Down	19.36438	7.552453
OASL	88.71718	123.4553	53.97905	0.437236	-1.19352	0.045966	1	Down	3.119263	1.403732
OBSCN	552.8026	747.9939	357.6112	0.478094	-1.06463	0.001568	0.143004	Down	1.091957	0.537323
OLFML3	95.79141	146.279	45.30385	0.309709	-1.69102	0.004223	0.264406	Down	3.495645	1.114288
OTOF	6.187873	11.41183	0.963912	0.084466	-3.56549	0.047739	1	Down	0.084223	0.007322
PCBP3	128.6988	68.47101	188.9267	2.759221	1.464261	0.00557	0.310623	Up	0.528779	1.501681
PCDHGA9	23.04828	38.38526	7.711293	0.200892	-2.31551	0.015434	0.601414	Down	0.464151	0.095971
PCDHGB5	24.60444	41.49758	7.711293	0.185825	-2.42798	0.010066	0.459118	Down	0.50503	0.096591
PCOLCE2	28.24755	8.299516	48.19558	5.807035	2.537802	0.005689	0.312825	Up	0.230771	1.379283
PCSK5	144.7872	73.65821	215.9162	2.931326	1.551553	0.002244	0.178033	Up	0.245462	0.740569
PDGFD	207.801	111.006	304.5961	2.74396	1.456259	0.001087	0.111004	Up	1.569179	4.431668
PEX5L	31.21281	10.3744	52.05123	5.017278	2.326905	0.007982	0.392454	Up	0.047023	0.242828
PFKFB4	416.5676	589.2656	243.8696	0.413853	-1.27281	0.000428	0.056562	Down	5.482296	2.33521
PHACTR1	49.87867	74.69565	25.0617	0.335518	-1.57554	0.030801	0.887541	Down	0.511488	0.176631
PIK3IP1	62.36471	100.6316	24.09779	0.239465	-2.06211	0.003028	0.21131	Down	2.296867	0.566103
PIP5K1A	81.67568	115.1558	48.19558	0.418525	-1.25661	0.041533	1	Down	1.376237	0.592832
PLCXD1	211.486	310.1944	112.7777	0.363571	-1.45969	0.000965	0.09958	Down	3.20207	1.198221
PLOD2	233.6439	350.6546	116.6333	0.332616	-1.58807	0.000221	0.036735	Down	4.72492	1.617538
PLXNC1	708.522	463.7355	953.3086	2.055716	1.039641	0.001191	0.117462	Up	2.415386	5.110539
PNCK	54.94753	108.9311	0.963912	0.008849	-6.8203	1.11E-09	1.46E-06	Down	1.903597	0.017337
PNPLA3	461.0466	620.3888	301.7043	0.486315	-1.04004	0.003009	0.21131	Down	12.48408	6.248718
POU2F2	392.6738	527.0193	258.3283	0.490169	-1.02865	0.004781	0.282618	Down	2.997541	1.512264
PPARG	251.6142	136.942	366.2864	2.674756	1.419407	0.000692	0.078537	Up	0.651141	1.792569
PPP1R3A	19.41724	31.12319	7.711293	0.247767	-2.01295	0.04265	1	Down	0.402647	0.10268
PPP2R3B	77.74651	113.0809	42.41211	0.37506	-1.41481	0.024675	0.806257	Down	1.839968	0.710277
PRAM1	273.96	169.1026	378.8173	2.240162	1.163603	0.004019	0.256802	Up	3.892739	8.975354
PRDM1	162.0566	220.9746	103.1385	0.466744	-1.0993	0.022046	0.751471	Down	1.464988	0.703768
PRDX2	151.3065	216.8249	85.78813	0.395656	-1.33768	0.006888	0.352606	Down	11.79063	4.801447
PRF1	14.96935	28.01087	1.927823	0.068824	-3.86094	0.002392	0.187775	Down	0.620759	0.043972

PRKN	59.27252	26.97343	91.5716	3.394882	1.763361	0.011738	0.505227	Up	0.288957	1.009659
PRLR	386.5664	245.8732	527.2597	2.144438	1.100599	0.002716	0.200274	Up	1.065268	2.351197
PRRT4	103.0857	52.90942	153.2619	2.896686	1.534403	0.007512	0.372823	Up	0.629526	1.87686
PRUNE2	6.706593	12.44927	0.963912	0.077427	-3.69102	0.035738	0.941602	Down	0.04582	0.003651
PTPRO	101.873	141.0918	62.65425	0.444067	-1.17115	0.039527	0.978436	Down	0.435092	0.19886
QRICH2	88.14505	52.90942	123.3807	2.331923	1.22152	0.042198	1	Up	0.273136	0.655557
QSOX1	8550.962	5497.392	11604.53	2.110916	1.077869	0.001233	0.12086	Up	77.22753	167.7876
RAB17	48.91476	74.69565	23.13388	0.309709	-1.69102	0.021766	0.751155	Down	1.127023	0.359255
RAB3IL1	231.4628	329.9058	133.0198	0.403205	-1.31041	0.002168	0.176799	Down	4.913331	2.03901
RAB3IP	304.7276	180.5145	428.9407	2.376212	1.248664	0.001488	0.138161	Up	0.766389	1.874352
RALGPS1	200.3063	130.7174	269.8953	2.064724	1.045949	0.019058	0.691422	Up	0.431734	0.917478
RASAL2	2624.425	3636.226	1612.624	0.443488	-1.17303	5.26E-05	0.011687	Down	12.47624	5.694861
RASGRF1	146.8218	212.6751	80.96858	0.380715	-1.39322	0.005444	0.305776	Down	1.184571	0.464171
RASL12	4.149758	8.299516	0	0	#NAME?	0.037705	0.961337	Down	0.133125	0
RBM20	12.37575	22.82367	1.927823	0.084466	-3.56549	0.007203	0.364356	Down	0.103327	0.008983
RELB	107.3583	146.279	68.43772	0.467858	-1.09586	0.049275	1	Down	3.622955	1.744591
RGS1	5.705917	11.41183	0	0	#NAME?	0.013332	0.545754	Down	0.459117	0
RGS13	4.668478	9.336956	0	0	#NAME?	0.026367	0.822237	Down	0.337188	0
RGS16	102.9472	144.2041	61.69034	0.427799	-1.225	0.030862	0.887541	Down	3.346874	1.473655
RHOB	223.1673	299.82	146.5146	0.488675	-1.03305	0.016171	0.619837	Down	7.125615	3.58393
RIN3	279.7933	374.5157	185.071	0.494161	-1.01695	0.011017	0.487048	Down	3.924175	1.995877
ROBO4	63.62676	95.44444	31.80908	0.333273	-1.58522	0.019182	0.692757	Down	1.404055	0.481617
S100A8	329.4297	143.1667	515.6927	3.602045	1.848816	2.53E-06	0.000907	Up	11.13091	41.26641
S100A9	1961.339	741.7693	3180.908	4.288272	2.100396	1.90E-12	6.01E-09	Up	72.56352	320.271
S1PR3	483.6848	293.5954	673.7742	2.294907	1.198436	0.000581	0.068464	Up	3.7758	8.918485
SCD	23728.07	39724.6	7731.535	0.194628	-2.36121	2.31E-07	0.000146	Down	410.0684	82.14469
SERPINE1	17.93461	30.08575	5.78347	0.192233	-2.37907	0.02248	0.759512	Down	0.532348	0.105327
SERPING1	9.855675	19.71135	0	0	#NAME?	0.001178	0.116901	Down	0.561073	0
SH2D2A	80.41364	120.343	40.48429	0.336408	-1.57172	0.012013	0.506943	Down	3.661864	1.2679
SH2D3C	266.4824	379.7029	153.2619	0.403637	-1.30887	0.001368	0.130879	Down	4.756396	1.975993
SHOX2	3226.04	4551.247	1900.834	0.417651	-1.25963	1.75E-05	0.004864	Down	57.02443	24.51273
SIGLEC11	65.38686	36.31038	94.46334	2.601552	1.379372	0.039127	0.975835	Up	0.647359	1.733383
SIGLEC16	73.06139	35.27294	110.8498	3.142631	1.651973	0.011125	0.489377	Up	0.513934	1.662332
SIPA1L2	203.1693	102.7065	303.6322	2.956309	1.563797	0.000524	0.06461	Up	0.819286	2.492885
SKIDA1	843.9669	1157.783	530.1514	0.457902	-1.12689	0.000303	0.044712	Down	5.969219	2.813244
SLAMF6	33.64728	51.87198	15.42259	0.29732	-1.74991	0.034203	0.931528	Down	0.988492	0.302492
SLC12A7	88.37423	154.5785	22.16997	0.143422	-2.80166	1.43E-05	0.00434	Down	1.326015	0.195741
SLC16A14	112.8678	70.54589	155.1898	2.199841	1.1374	0.038253	0.969043	Up	0.764586	1.731149
SLC1A3	530.986	309.157	752.815	2.435057	1.283956	0.000173	0.030722	Up	3.452099	8.651858
SLC22A4	175.8937	67.43357	284.3539	4.216801	2.076149	1.84E-05	0.004911	Up	1.328083	5.764018
SLC2A5	1864.343	2519.941	1208.745	0.479672	-1.05988	0.000246	0.039205	Down	46.88135	23.14523
SLC40A1	415.7653	172.215	659.3155	3.828445	1.936759	1.76E-07	0.000116	Up	2.693453	10.61326
SLC6A8	981.9056	1446.191	517.6205	0.35792	-1.48229	1.52E-06	0.000649	Down	22.33996	8.229723
SLC8A1	834.8519	519.7572	1149.947	2.212469	1.145657	0.00025	0.039484	Up	3.460857	7.880935
SLCO4C1	299.9706	467.8852	132.0559	0.28224	-1.82501	5.65E-06	0.001983	Down	4.772276	1.386312
SMAD7	506.0305	325.756	686.3051	2.106807	1.075058	0.001762	0.152895	Up	5.338935	11.57701
SMIM11A	39.5939	2.074879	77.11293	37.16502	5.215874	9.70E-07	0.00045	Up	0.039354	1.505347
SMN1	395.7453	545.6932	245.7975	0.450432	-1.15062	0.001614	0.144689	Down	16.68561	7.735493
SMPD1	491.4288	308.1195	674.7381	2.189858	1.130838	0.0011	0.111004	Up	6.8688	15.48153
SPON2	21.60241	38.38526	4.819558	0.125558	-2.99358	0.003562	0.239192	Down	0.874357	0.112992
SPP1	23.75082	44.6099	2.891735	0.064823	-3.94736	0.000291	0.043351	Down	1.388095	0.092611
SPSB2	125.7296	79.88284	171.5763	2.147849	1.102892	0.036477	0.954701	Up	2.997996	6.627529
SPTLC3	18.41656	30.08575	6.747381	0.224272	-2.15668	0.034266	0.931528	Down	0.245155	0.056589
SQLE	4358.556	5813.811	2903.302	0.49938	-1.00179	0.000852	0.090491	Down	99.32218	51.04984
SREBF1	1832.534	2519.941	1145.127	0.454426	-1.13788	8.57E-05	0.018031	Down	25.2643	11.81646
SRGAP1	126.8853	58.09661	195.6741	3.36808	1.751927	0.001102	0.111004	Up	0.232177	0.804855
STC2	59.62003	104.7814	14.45867	0.137989	-2.85738	0.000114	0.022003	Down	1.106941	0.157212
STS	800.4605	1222.104	378.8173	0.309971	-1.68979	1.26E-07	9.07E-05	Down	9.13906	2.915682
SULF2	145.2576	236.5362	53.97905	0.228206	-2.13159	3.96E-05	0.009317	Down	1.735736	0.407688
SULT1A4	54.95558	81.95772	27.95344	0.341071	-1.55185	0.02832	0.844397	Down	3.311455	1.162468
SYN3	20.60173	37.34782	3.855646	0.103236	-3.27598	0.002199	0.177065	Down	0.194187	0.020633
TBC1D2	168.7707	84.0326	253.5088	3.016791	1.593015	0.000935	0.098334	Up	0.677893	2.104861
TBC1D30	214.6518	385.9275	43.37602	0.112394	-3.15336	5.14E-11	1.01E-07	Down	2.402788	0.277956
TBC1D3C	11.15851	2.074879	20.24214	9.755819	3.286263	0.015952	0.615523	Up	0.030861	0.309875
TBC1D3H	5.187198	10.3744	0	0	#NAME?	0.018654	0.681492	Down	0.147651	0
TBXA2R	55.43754	81.95772	28.91735	0.352833	-1.50294	0.032936	0.914572	Down	0.99808	0.362452
TCN1	34.69679	13.48671	55.90687	4.14533	2.051487	0.014065	0.563505	Up	0.485806	2.07271
TCP11L2	36.20412	56.02173	16.3865	0.292503	-1.77348	0.028411	0.844397	Down	0.64037	0.192787
TESK1	254.0895	165.9903	342.1886	2.061497	1.043693	0.011449	0.500524	Up	3.723896	7.901275
TF	159.3114	102.7065	215.9162	2.102264	1.071944	0.026664	0.827363	Up	1.501105	3.247994
TGM5	95.86896	134.8671	56.87079	0.42168	-1.24578	0.032446	0.907925	Down	2.399172	1.041265
TIFAB	38.46281	65.35869	11.56694	0.176976	-2.49837	0.002518	0.192902	Down	2.898013	0.527877
TLR1	130.6272	190.8889	70.36555	0.36862	-1.43979	0.005967	0.321501	Down	1.23762	0.469552
TLR5	87.77741	42.53502	133.0198	3.127301	1.644918	0.007053	0.359087	Up	0.447426	1.440151
TM6SF1	100.7127	53.94685	147.4785	2.733773	1.450894	0.011939	0.5058	Up	0.464465	1.306869
TMEM119	94.90103	148.3539	41.4482	0.279387	-1.83966	0.002049	0.169307	Down	2.877605	0.827475
TMEM45A	207.0301	334.0555	80.00466	0.239495	-2.06193	6.25E-06	0.002054	Down	8.894226	2.19241
TMPRSS9	16.89717	28.01087	5.78347	0.206472	-2.27598	0.031285	0.893183	Down	0.392716	0.083456
TNF	204.8329	367.2536	42.41211	0.115485	-3.11423	1.56E-10	2.47E-07	Down	12.36851	1.470138
TNFAIP3	303.4258	442.9867	163.865	0.369909	-1.43476	0.000282	0.042748	Down	2.835285	1.079466
TNNC2	4.337602	0	8.675205	Inf	Inf	0.037776	0.961571	Up	0	0.661404
TPD52	2731.497	3747.232	1715.763	0.457875	-1.12698	0.000103	0.020018	Down	45.93104	21.64561
TRIB1	444.2189	607.9396	280.4983	0.461392	-1.11594	0.001634	0.145669	Down	9.148264	4.344356
TSHZ3	162.2399	103.744	220.7358	2.127698	1.089293	0.023444	0.775663	Up	1.136171	2.488116
TUBA1A	1706.408	2401.672	1011.143	0.421016	-1.24805	1.81E-05	0.004911	Down	54.75055	23.7249
VNN1	47.28507	69.50845	25.0617	0.360556	-1.4717	0.046409	1	Down	1.020656	0.378765
WNT4	4.819558	0	9.639116	Inf	Inf	0.027494	0.832013	Up	0	0.089541

XAGE1B	460.68	854.8502	66.5099	0.077803	-3.68403	2.07E-20	1.64E-16	Down	50.2102	4.020733
ZFP36	2113.054	2819.761	1406.347	0.498747	-1.00362	0.000498	0.061898	Down	90.84552	46.63379
ZFP36L1	123.6225	183.6268	63.61817	0.346454	-1.52927	0.004317	0.26716	Down	2.070478	0.7383
ZIC5	471.9805	629.7258	314.2352	0.499003	-1.00288	0.003991	0.256802	Down	7.662182	3.935254
ZNF280A	22.52956	37.34782	7.711293	0.206472	-2.27598	0.017816	0.658493	Down	0.976423	0.207499
ZNF365	42.14671	19.71135	64.58208	3.27639	1.712107	0.027156	0.827363	Up	0.126504	0.426598
ZNF366	172.5821	232.3865	112.7777	0.485302	-1.04304	0.026154	0.822237	Down	2.089368	1.043624
ZNF556	49.87867	74.69565	25.0617	0.335518	-1.57554	0.030801	0.887541	Down	2.561479	0.884551
ZNF723	6.187873	11.41183	0.963912	0.084466	-3.56549	0.047739	1	Down	0.177547	0.015435
ZYG11A	103.4659	145.2415	61.69034	0.424743	-1.23534	0.029222	0.863628	Down	1.531221	0.669393
