# Supplementary material for: Repressing HIF-1α-induced HDAC9 contributes to the synergistic effect of venetoclax and MENIN inhibitor in KMT2Ar AML
Source: Biomark Res. 2023 Dec 5;11:105. doi: 10.1186/s40364-023-00547-9 (PMC10696732; doi:10.1186/s40364-023-00547-9)
Supplement: Supplementary file 12 — Additional file 12: Table S11. FPKM of different groups in three KMT2Ar-AML cell lines. [file 40364_2023_547_MOESM12_ESM.pdf]

gene_id	MV4_11_DMSO	MV4_11_MI_503	MV4_11_Venetoclax	MV4_11_MI_503_Venetoclax	MOLM13_DMSO	MOLM13_MI_503	MOLM13_Venetoclax	MOLM13_MI_503_Venetoclax	THP1_DMSO	THP1_MI_503	THP1_Venetoclax	THP1_MI_503_Venetoclax
A1BG	0.54845532	0.789695947	0.354745523	0.554322217	0.509053668	0.500661006	0.43867064	0.447221928	1.525300014	1.503508973	1.345656767	1.617182243
A1CF	0	0	0	0	0	0	0	0	0	0	0	0
A2M	0.526570666	0.205997495	0.347016577	0.040166299	0.165987588	0.081625493	0.234990558	0.009721737	1.297450304	0.72992856	0.995280362	0.341204517
A2ML1	0	0	0	0	0	0	0	0	0	0.019790164	0	0
A3GALT2	0	0	0	0	0	0	0	0	0	0.057677785	0	0
A4GALT	0	0	0.014952161	0	0	0	0	0	1.860212419	1.549077825	1.826044746	1.867120019
A4GNT	0	0	0	0	0	0	0	0	0	0	0.021971836	0
AAAS	21.44936726	24.13650396	23.97533263	33.14050987	31.48733313	31.26230306	22.79373067	34.69096608	23.3422749	27.60236565	27.97832103	26.07718812
AACS	4.002753215	3.661443227	3.738149298	3.299916881	2.442191656	2.305205827	3.809538979	3.747758025	8.763239753	7.693977635	10.73678	7.367416387
AADAC	0	0	0	0	0	0	0	0	0	0	0	0
AADACL2	0	0	0	0	0	0	0	0	0	0	0	0
AADACL3	0	0	0	0	0	0	0	0	0	0	0	0
AADACL4	0	0	0	0	0	0	0	0	0	0	0	0
AADAT	0.017153191	0.053347901	0.090775871	0	0	0	0	0	1.549357774	1.655207186	1.866156222	1.80296104
AAED1	10.90088763	13.01179271	9.974780906	10.0957133	9.93132276	9.901389671	8.842908184	8.584254658	9.31071561	8.857799539	10.85320036	9.717323043
AAGAB	18.18530638	15.40052439	18.65511215	22.94940964	14.9271943	15.99295747	19.51269457	20.89825483	20.58676522	17.70603391	19.49578776	26.40865648
AAK1	3.555251964	3.856226612	3.559381441	3.20454344	3.162736796	2.965068574	2.896216405	2.609743073	4.739184595	3.238095691	4.238441062	5.019228835
AAMDC	2.488303392	2.805328663	1.74479619	2.514921091	2.630711381	1.820720311	2.398923463	2.373947057	5.88967441	6.446142631	5.528139366	6.37675322
AAMP	46.6896534	43.84802089	48.68860497	50.66675323	42.20228308	44.19749416	49.40153945	56.92628453	37.64665124	37.29494394	38.77418444	49.05756952
AANAT	0	0.010585222	0	0.010319766	0	0.010485856	0	0.009991068	0.011111624	0	0.010998426	0
AAR2	14.11618711	12.0732031	13.71348981	14.84467125	13.2131978	13.3405167	14.67194176	16.41087522	14.84987656	12.12519623	14.10123896	17.43625926
AARD	0	0	0	0	0	0	0	0	0.557385426	0.991114911	1.363041183	1.097402833
AARS	86.00840395	88.16360143	90.67482286	101.3379143	115.9020744	113.7433422	90.06857619	100.109844	49.20714783	61.05117681	92.59312827	44.00870399
AARS2	13.33046523	14.58855902	14.19517963	16.3176044	15.38347017	16.32979941	14.20993737	16.82189477	13.83239796	16.21060511	15.33314706	15.73382958
AARSD1	0.078899649	0	0	0	0	0	0.08113661	0	0.085862547	0.129775017	0	0
AASDH	4.088749358	3.407817245	3.136430629	3.551484076	4.024212481	3.866402834	3.630173517	3.359139574	4.846079053	5.025049034	4.299596735	4.735386871
AASDHPPT	15.76563192	14.3395692	16.28477896	14.57944555	18.79086718	16.11763375	18.61584351	14.78832246	16.16290517	16.24701578	17.58273903	17.30830162
AASS	0.054991886	0.08551475	0.126109037	0	0	0	0	0	7.580356901	9.979789427	5.242320668	9.433301133
AATF	47.19960546	43.27763949	51.23356226	50.0414676	52.95887886	50.25320543	50.68298188	55.94834747	47.15293563	43.13024094	45.914549	48.15225257
AATK	0	0	0	0	0	0	0.013978822	0.013301232	0.21449911	0.186321758	0.190350423	0.212197419
ABAT	3.139865698	2.928643072	2.190125347	2.082294534	1.53125701	1.820148861	2.238941753	1.936740383	5.72756736	3.620566023	3.682569368	6.600773061
ABCA1	2.702288043	4.274804356	1.389815659	0.133525077	1.638659703	2.512025905	1.259769464	0.278130936	13.31839832	13.85446064	10.39265723	9.594930736
ABCA10	0.067995022	0.079301389	0.044979293	0.017180593	0	0.017457103	0	0.024950053	0.175739517	0.12115888	0.128173093	0.132677852
ABCA12	0	0	0	0	0	0	0	0	0	0	0	0
ABCA13	0.005682308	0	0.004009489	0.003828728	0	0.001945174	0.001947804	0	0.014428801	0.006230882	0.004080517	0.00788467
ABCA2	21.78772291	19.47106066	21.6025442	26.59408723	25.84189482	27.55751815	20.67125948	30.18725903	44.46504974	52.21463653	49.13571697	52.08701404
ABCA3	0.178742313	0.151610175	0.171984845	0.213500586	0.195122508	0.166874403	0.050130003	0.230550343	3.890326605	5.719584528	4.39329235	7.406769648
ABCA4	0	0	0	0	0	0	0	0	0	0	0	0
ABCA5	0.229465078	0.327092398	0.297514529	0.440647421	0.119801276	0.235652275	0.112086161	0.286279248	1.691821713	1.320993456	0.963968632	1.993990641
ABCA6	0.252627046	0.247347436	0.23024729	0.021277453	0.043964631	0.079272958	0.079380131	0.041199474	0.045820232	0.038474423	0.022676723	0.029211736
ABCA7	8.671212357	8.949238417	7.119097663	10.07190766	5.024769866	5.156793945	6.637988889	9.379561108	11.13989885	6.610801158	6.786126883	18.08835399
ABCA8	0	0	0.009157868	0	0	0	0.00889776	0	0	0	0	0
ABCA9	0.089285776	0.092562149	0.031500432	0.015040146	0	0.02292331	0.022954301	0.014561097	0	0.016317581	0.024043848	0.023229632
ABCB1	0	0	0	0.011065141	0	0	0.022516854	0	0.17871289	0.060024796	0.058964093	0.1822955
ABCB10	31.28607537	44.31338401	33.85995959	43.30690242	28.02301693	49.57360467	30.87209694	37.08738736	24.75945577	17.65923942	24.49605244	19.46849364
ABCB11	0.066939208	0.069395566	0.02024268	0.038660152	0.069896497	0.078564718	0.0295016	0.009357194	1.321645397	0.786445366	1.431789019	0.268699177
ABCB4	0.013548926	0.007023054	0.007170185	0	0	0	0.013933065	0	1.356505011	1.82740477	1.452143904	0.97291301
ABCB5	0	0	0	0	0	0	0	0	0	0	0	0
ABCB6	1.413715885	1.978550129	1.421481569	1.196652314	1.291650543	1.107023965	1.417452576	1.469786177	2.634640794	2.344673004	1.979644394	3.107984862
ABCB7	19.9895848	18.25662165	18.94503676	16.54028268	22.54437251	21.60181562	18.6813356	16.36157112	14.32499512	18.21333373	14.84969267	10.80639907
ABCB8	6.500331323	6.232270407	7.144657253	8.958908449	6.407786961	6.698944713	5.971757294	9.329631861	4.093512796	4.236850534	4.027328512	5.629456356
ABCB9	0.817581468	0.971017312	0.756121894	0.93062097	0.538743459	0.87223323	0.881575178	0.900979449	0.751522107	0.757247465	0.709665349	0.768239798
ABCC1	7.510196208	7.380832976	6.661782664	4.536442635	4.563590137	6.161075644	5.184119275	4.673208814	10.99621565	10.13443164	10.534883	12.19534984
ABCC10	9.33262159	8.687831819	9.220425659	8.905173806	7.548641921	7.823887639	8.047358209	9.010473422	4.695114178	4.79444593	4.477804746	6.756407616
ABCC11	0	0.005846779	0.011938535	0	0.005888984	0	0.005799724	0	0.012275077	0.024737187	0.006075013	0.011738581
ABCC12	0	0	0	0	0	0	0	0	0	0	0	0
ABCC2	0.324464941	0.51984655	0.218538835	0.357748128	0.284899486	0.378651902	0.121332422	0.27419642	0.280874194	0.291100106	0.222410265	0.161158952
ABCC3	0	0	0	0.007619105	0.007871504	0.015483457	0	0	0.082037345	0.115727268	0.097441923	0.368723486
ABCC4	7.118350912	7.139690294	6.770660734	6.981275595	7.020685095	7.135566991	6.746313625	5.9664884	8.250145234	8.268223951	8.649906452	10.16294673
ABCC5	3.670890052	3.006062447	3.484019931	3.845152044	2.865801163	3.172531731	3.495832129	3.566688875	1.805182878	1.606332555	1.54545979	2.515444171
ABCC6	3.819213859	3.95936126	3.115409827	1.901350078	2.328416158	2.839634776	2.701717057	1.975674978	4.068016351	3.307659322	3.074520749	2.236242946
ABCC8	0	0	0	0	0	0	0	0	0.018493054	0.009316971	0.064066308	0.02652719
ABCC9	0	0	0	0	0	0	0	0	0	0	0	0
ABCD1	13.89297068	14.40277815	11.4247528	5.447422728	10.27433272	9.72789186	10.39044637	7.975539088	9.477359836	8.245152747	8.352559916	10.34695811
ABCD2	0.07162003	0.193045196	0.133414384	0.083967944	0.107689121	0.102971626	0.106056862	0.05606445	0.545583506	0.537174467	0.450535523	0.420371865
ABCD3	14.06769573	14.38963529	13.99025833	20.9105733	17.89837189	18.20631314	16.21383373	18.17852436	23.18129285	22.59071588	21.0476212	29.76119792
ABCD4	5.411704407	6.439081476	4.995572531	4.925742413	5.217345587	5.731298988	4.806254581	5.370598689	14.33840752	14.78481037	13.132466	13.99972471
ABCE1	56.45256743	45.68871198	59.43784544	55.57816705	69.26221958	64.00937237	68.42350515	53.44385214	37.71241114	40.5883691	41.95797146	36.51811834
ABCF1	53.3746106	48.48902726	55.5059439	62.64342457	53.7213271	52.42086225	52.57160436	63.186554	17.09076152	16.25004998	18.05446832	20.12662502
ABCF2	25.5522586	22.13244736	28.7406727	26.66681894	24.81257697	25.18346884	30.6526017	28.7188536	18.13811443	19.38291416	20.95676447	23.67934875
ABCF3	16.77181118	15.24919133	16.14594212	15.36635109	13.9354651	14.56341512	15.88015904	16.36126788	14.96880375	13.90537045	15.11006719	20.66478212
ABCG1	0	0	0.004964503	0	0.004897733	0.004816986	0.004823498	0	0.296058134	0.26745358	0.121258794	0.234305016
ABCG2	0	0	0	0	0.010053104	0	0	0	0.031432237	0.063343397	0.031112026	0.010019484
ABCG4	0	0.012262942	0.012519846	0	0	0	0	0	0.102982206	0.220504367	0.152899636	0.332374207
ABCG5	0	0	0.011728332	0	0	0	0	0	0	0	0	0
ABCG8	0	0	0	0	0	0	0	0	0	0	0	0
ABHD1	0.026704497	0	0	0	0.027884267	0.027424545	0.027461622	0	0.929957432	0.937042173	0.373946475	0.639193386
ABHD10	9.380521041	9.685291285	9.686806155	9.230863129	10.70887004	10.51277465	9.529075939	9.365071763	14.63956427	16.12814051	15.72016503	14.0195263
ABHD11	3.93580239	4.647363993	4.728640978	4.745839338	4.220753583	4.432072927	4.938128509	6.408758008	4.216988295	3.699229343	3.944866021	5.487607426
ABHD12	9.770515642	11.33528065	10.81360569	12.11423187	9.33672809	9.231901253	9.326336055	12.80451841	16.32207343	15.46767515	16.34465103	16.80298712
ABHD12B	0	0	0	0	0	0	0	0	0	0	0	0
ABHD13	6.821723368	5.88781431	6.726371241	7.650836246	7.383494692	6.584330315	6.783500921	6.738063484	5.086312955	4.736933626	4.462591925	5.609098852
ABHD14A	3.642343921	4.3298145	3.906508465	3.8285588	4.208938887	3.541058266	4.444792366	6.272731533	7.663309814	6.816389816	6.957496919	7.227294706
ABHD14A-AC'	0	0	0	0	0	0	0	0	0	0	0	0
ABHD14B	20.13718486	25.63734768	19.98138203	22.78508705	22.43321915	27.23340613	19.07326263	22.12416849	14.41729759	21.11276751	17.48126885	13.4884504
ABHD15	11.99321565	12.36699994	11.20438075	14.61045837	10.31900195	12.62861594	10.1790398	12.67425615	14.77443098	10.87153406	11.52344123	18.62192208
ABHD16A	25.18053211	21.51769628	24.81497646	24.41962024	20.20097718	19.66746671	24.13246741	25.50939937	28.1343984	23.06902181	24.53034617	32.36695725
ABHD16B	0	0	0	0.03062228	0	0	0	0	0.032971992	0.066446368	0.032636094	0
ABHD17A	21.12295083	22.06856085	20.63790375	21.23462934	23.15089296	22.85365669	20.48511086	23.3443069	13.8593468	13.86349197	14.62605741	17.15801982
ABHD17B	5.406640841	5.618353068	6.279757748	5.866848737	7.724008595	6.765779295	6.66927441	5.8936094	11.3063556	8.252163095	10.90067362	9.208410811
ABHD17C	7.113806351	6.642092456	8.977302816	6.590745008	8.499443691	8.335899682	7.385838085	6.514684352	10.52063601	11.17582853	9.922257745	11.88790183
ABHD18	4.668598322	4.918553855	4.138437396	4.537324716	4.025172425	4.312907762	4.396745365	3.778756871	4.337648671	4.484120721	4.040903087	4.356187568
ABHD2	8.689742748	9.137813808	8.825612599	12.30379275	8.304691576	7.981613765	7.91084935	9.971851569	16.66311915	15.88937673	16.13335556	20.24425925
ABHD3	12.90926735	13.3085242	13.09324869	20.30597386	16.0105192	15.98625878	13.99534777	17.39285921	21.21930837	14.64773257	18.41159222	19.13342423
ABHD4	2.976992014	3.335986811	2.240227726	2.295760235	2.659304828	2.3150368	2.176599184	1.986902956	5.393277273	3.170046335	3.429138208	4.548679343
ABHD5	2.198290509	1.885402637	2.093096474	2.471647531	1.686986735	2.003701615	1.979174175	2.064651332	2.286606264	2.420195831	2.253802095	2.949244713
ABHD6	1.295055616	1.364954442	1.416394882	1.090757493	0.833899538	1.24130994	1.309576777	1.2883388	3.382426401	3.59753907	3.789714279	5.525763784
ABHD8	0	0	0.081750404	0.026021612	0	0.026440411	0.026476157	0	4.735093514	5.053484693	5.574307385	8.57399408
ABI1	18.12973235	17.0034187	17.24602474	16.49021669	16.72266179	15.66429516	17.2529159	14.12690776	22.65000208	20.78630064	18.80026534	20.16317822
ABI2	5.221082926	5.233494287	5.350756488	5.546239089	7.512126851	6.286320902	4.524864504	4.876315096	13.7696544	15.28017457	13.87760812	14.0821642
ABI3	0.126313523	0.052379457	0.080215185	0.051065886	0	0	0.051957904	0	0	0.110806337	0.027212067	0.052581126
ABI3BP	0	0.008386068	0	0	0	0	0	0	0.202471424	0.097571876	0.226549052	0.025255066
ABL1	15.67404634	15.11575599	16.16377701	15.70026974	15.83678622	14.92894703	16.04647911	15.91056465	15.22019718	12.14231761	14.30199385	14.66421099
ABL2	3.321112387	2.51561517	2.669888346	2.514881045	2.809339932	2.601113305	2.520041085	1.861297785	3.028618136	3.465097653	2.88331787	3.395602019
ABLIM1	0.008289234	0.00859341	0.00438672	0.004188952	0.017310883	0.072358299	0.012786375	0.004055529	0	0.036357929	0	0.012939744
ABLIM2	0.019959036	0.062074319	0.010562459	0.040345081	0.020840801	0	0.030787371	0.039060036	0.640752442	0.864492873	0.666473595	0.6335187
ABLIM3	0.006722834	0.020908591	0.01423108	0	0	0.006904105	0.006913439	0.006578327	0.124374109	0.272758852	0.159315028	0.132930919
ABO	0	0	0	0	0	0	0	0	0	0	0	0
ABR	21.61595232	20.84829849	20.3957408	18.64856051	18.28654052	18.02447582	18.72869109	18.22986612	12.2305808	8.979739914	10.71850465	15.00731428
ABRA	0	0	0	0	0	0	0	0	0	0	0	0
ABRACL	112.6637681	103.3967412	106.9533188	89.49121981	90.97432825	88.9887658	118.2356907	93.22242095	82.1188058	76.00270485	85.58407806	87.93577083
ABRAXAS1	11.35999279	12.98755612	10.39421033	9.192363255	14.11598624	14.2103514	10.71766323	8.466715538	6.874481345	5.277602704	5.641783717	6.850243462
ABRAXAS2	19.56711304	17.78678212	18.50203992	18.74034386	20.28133106	18.96807853	22.93302412	18.75936551	16.22486467	15.6582464	15.53652618	15.64675115
ABT1	22.34142545	20.89440601	22.53961663	21.33128521	22.360559	23.33436325	24.56353521	23.11705422	7.75948636	6.854306737	6.937995447	7.791366225
ABTB1	5.303176169	7.696889327	4.265845643	5.717231916	2.43648404	3.001444092	4.144684059	4.381978266	10.38812707	6.228670266	7.997344132	11.43034904
ABTB2	0.504841893	0.523367244	0.487868002	0.332766685	0.022919355	0.056353721	0.316007485	0.28995086	3.344138686	1.612601706	2.387979633	7.366773353
ACAA1	21.28596521	21.86576534	20.44854033	20.36070814	13.30539886	15.27950433	19.21879985	19.64094349	28.05091069	24.08613387	25.46445619	33.3416989
ACAA2	4.201263542	5.768781975	5.418465233	5.680353701	5.374642061	6.343237747	5.579295624	5.907800127	19.74145998	21.44781549	27.69214797	15.0276259
ACACA	17.1004361	16.3886936	15.02539424	14.26222813	17.86575767	18.25875662	14.584087	12.90834079	22.78092187	22.47884164	26.79960661	13.39498763
ACACB	0.315522754	0.62202808	0.290156437	0.941010319	0.610315125	0.823355866	0.287234362	0.885731275	3.79956008	2.921008631	3.393124822	3.725006056
ACAD10	16.48220783	14.22107997	14.28326388	12.09000704	15.06249459	14.46432748	13.81021356	13.12797629	16.19726808	12.62840152	13.36492169	15.47571224
ACAD11	8.54960683	9.255520754	7.703680486	6.438739027	7.299859665	7.723410548	6.893554379	6.041168379	3.85338362	4.148226543	3.634831247	4.472353001
ACAD8	3.60294069	2.931385012	3.820297959	3.1871197	3.442368338	2.662993402	3.751991042	3.225860483	6.055061331	5.186726695	5.460007924	7.248192621
ACAD9	18.57328252	22.23521758	20.31883059	22.05992428	20.5787853	23.75519888	19.23602145	24.98469358	25.97557835	23.12473722	28.60394796	28.65883891
ACADL	0	0	0	0	0	0	0	0	0	0	0	0
ACADM	35.34819726	33.09504221	35.246564	31.92691865	41.55439167	37.77680946	38.69818617	31.52627786	46.56388107	40.85416263	47.97634798	46.37216787
ACADS	6.342669848	8.339552453	6.986062297	8.182531356	7.269015635	7.78465492	6.840667615	8.653547875	6.229004824	6.389548923	6.221093253	6.708061672
ACADSB	8.332737922	7.267616595	7.276074905	5.620685662	5.513702003	6.343651362	7.441979319	5.016252724	5.766130345	5.25388218	5.287871298	6.494401835
ACADVL	108.796896	106.2836327	111.4903284	100.7911179	89.67981847	92.41574637	99.75091309	105.0357493	50.56757835	55.99765349	55.62261986	60.42288845
ACAN	0	0	0	0	0	0	0	0	0	0	0	0
ACAP1	3.22899852	4.668864508	2.450790826	1.309709943	2.26255565	3.228799	3.517158322	1.642156075	5.317172967	6.522394322	6.132545038	4.642624854
ACAP2	18.69459209	17.32465364	16.29780849	17.12277698	17.41437381	16.31399953	17.19218423	13.19303563	21.7807253	22.23611391	19.2039154	25.01988537
ACAP3	7.429568159	7.500173899	7.837775245	8.55128198	9.046523938	8.663892629	6.955514538	9.60576284	5.522696645	4.656599937	4.994135022	9.937332886
ACAT1	22.37112301	19.08445738	26.22634449	19.60700074	21.052148	21.84752199	25.65382889	21.71131794	19.90085202	23.27756824	24.70471934	15.11383478
ACAT2	47.29829394	69.25530038	49.82249595	79.42684654	74.11124094	76.10253828	59.91502986	77.25555382	47.11654418	67.44240169	90.47976789	22.67530058
ACBD3	17.16170286	16.88388985	17.44528392	19.05387814	18.42422654	17.40743197	18.54853453	15.86234082	26.16642454	30.45387063	26.02992638	27.04911748
ACBD4	1.019281342	1.518983582	0.899017858	0.933604907	1.019965563	1.133995203	1.211958097	0.748028974	2.438001626	2.491502784	1.692646437	2.839723165

ACBD5	3.739528623	3.586172733	3.632358813	3.537696141	3.262275057	3.987795765	3.733067481	3.164125918	4.553125819	4.400402457	3.807165645	4.67428237
ACBD6	32.66533367	33.31168158	32.1064256	38.43308325	37.86350477	36.76051545	32.22177783	38.74030432	18.44440151	20.99474954	19.36839107	20.79167036
ACBD7	0.063868915	0.033106304	0.050699805	0.064552129	0.050017922	0.016397762	0.016419931	0.015624014	1.00782757	1.120557851	0.756770018	0.564974054
ACCS	4.077103772	3.701921119	2.664457605	1.396618873	1.900036669	2.191871736	2.208904489	1.43245953	20.59142373	22.2035276	17.84684036	24.53244411
ACCSL	0	0	0	0	0	0	0	0	0	0	0	0
ACD	9.428393529	12.87948986	11.21964278	12.39855489	12.34695381	14.76465301	12.10624196	16.23422814	10.85566915	11.50956576	11.08173908	14.41985198
ACE	0.862300938	0.893943376	0.587466523	0.150262945	0.558866665	0.508937729	0.611550944	0.242461449	1.251198714	1.249862415	1.281157568	0.876755646
ACE2	0	0.011614433	0	0	0	0	0	0	0	0	0	0.01165915
ACER1	0	0	0	0.030848082	0.063739987	0	0	0	0	0	0	0.031763414
ACER2	1.337332495	1.039804787	0.937258198	1.013728583	1.009569682	0.992925116	1.142943014	0.760394935	2.310863209	1.763690787	1.596258505	2.144038444
ACER3	4.226456497	3.976902703	4.176408195	4.629180734	3.158636759	3.595092737	3.894722799	3.83722463	4.114940426	4.76154534	4.132144577	5.591638884
ACHE	0.01165971	0	0	0	0	0	0	0	0	0	0	0.012134106
ACIN1	47.88938913	45.33392941	47.02880573	51.42576157	49.34514117	48.35084726	49.79013321	53.23222013	46.63939683	41.34517043	42.58797792	43.79337019
ACKR1	0	0	0	0	0	0	0	0	0	0	0	0
ACKR2	0.036357811	0.018845987	0.019240804	0.018373368	0	0.056007222	0.037388627	0.053364454	1.167208502	1.156166795	1.429460935	0.870253158
ACKR3	0	0.02173972	0	0.042389064	0	0	0	0	0.31949166	0.160962829	0.22588349	0.480115267
ACKR4	0	0	0	0	0	0	0	0	0.157960616	0.045475433	0.06700775	0
ACLY	86.39062976	100.7543291	84.18508815	85.85812237	86.309337	98.21924754	88.4876757	86.40628743	122.5802373	137.1284977	154.6833084	94.47893251
ACMSD	0	0	0	0	0	0	0	0	0	0	0	0
ACO1	8.865682005	10.98363834	9.368050084	11.50797277	8.813615471	10.91062976	8.408775511	10.62522328	13.23620734	16.29369161	12.84880904	17.24802685
ACO2	40.23991206	40.25779266	42.24811069	48.07537747	39.09601408	40.74358427	40.43276018	51.07033354	41.99579241	44.74542726	44.32058134	44.45238708
ACOD1	0	0	0	0	0	0	0	0	0	0	0	0
ACOT1	0.084940112	0.286185313	0.202279018	0.128773091	0.332597466	0.283498801	0.545927071	0.290900156	3.05039066	3.399620621	4.757708903	2.408792413
ACOT11	1.793123084	2.105681975	1.797094544	1.764190075	1.524383802	2.151100127	2.10505357	2.096179689	5.197887165	4.054266949	5.606440224	7.480831959
ACOT12	0	0	0	0	0	0	0	0.011674707	0.064920462	0.039249029	0.012851819	0.012416607
ACOT13	9.978337225	9.836790515	10.9110903	8.88476702	9.281368784	8.336219347	10.94112876	8.793458337	2.025222997	3.128104488	2.914570309	2.599266294
ACOT2	0.93409548	0.792304727	0.778943879	0.40052202	1.418709276	1.511595921	1.717398687	1.523361945	8.65590228	7.91484243	8.628701807	9.573720212
ACOT4	0	0	0	0	0	0	0	0	4.485160621	3.036989856	6.037677464	5.249896766
ACOT6	0	0	0	0	0	0	0	0	0.032496258	0.065487652	0	0
ACOT7	7.455405879	7.985839104	9.631194494	12.88489135	9.125356998	10.73288103	7.643618836	13.42219897	17.03557499	18.99303636	23.99502927	14.74395453
ACOT8	11.04121565	12.44171408	11.9633174	12.74721308	10.93660314	9.859935835	12.38646094	13.1098532	13.86781108	14.93054728	14.71371715	15.30545735
ACOT9	5.515107346	5.331169525	5.429708836	4.406554519	4.176396389	4.056515657	5.007245108	4.521442598	7.13729449	6.279089699	7.024444696	7.588031768
ACOX1	7.004754703	6.465886833	7.09752464	6.640209348	5.20011659	5.00274606	6.113418168	5.438143355	7.371538168	7.539447833	6.835382729	7.494802117
ACOX2	0.23954074	0.225755248	0.069145424	0.066028129	0.090953944	0.111818006	0.223938356	0.021308348	0.331774828	0.358181147	0.703703403	0.249286883
ACOX3	6.586714919	6.603797777	6.638949139	3.930798375	5.294024916	5.551634553	5.982481407	5.045861462	6.932203574	6.070311112	5.694646947	6.358642832
ACOXL	0.012810275	0.016600441	0	0	0.016720271	0.016444607	0.013173472	0.009401189	0.006970391	0	0.010349072	0.006665742
ACP1	41.46451025	39.63842529	41.76940572	42.89005596	48.93592658	43.75642258	47.78278437	42.83817642	38.56199615	41.51927563	41.61668792	33.24841684
ACP2	5.483184958	5.395355466	4.967384207	7.209087547	5.337260664	4.605038214	5.447503574	7.388688128	15.09468025	10.44550669	11.26198905	20.96331835
ACP4	0	0	0.042281204	0	0	0	0	0	0	0	0	0
ACP5	0.076361723	0	0.107763068	0	0	0.052280472	0	0	0.027700216	0.027911246	0	0
ACP6	0.01279961	0.013269296	0.013547284	0.019404794	0.00668254	0	0	0	1.302377982	2.19652345	1.59243025	1.83821314
ACP7	0	0	0	0	0	0	0	0	0	0	0	0
ACPP	8.627955964	9.009096933	8.657560693	4.139916814	10.34814709	10.13918178	8.501284468	4.227340613	9.68744882	7.740740457	7.992867973	4.858766095
ACR	0	0	0	0	0	0	0	0	0	0	0	0.040875109
ACRBP	0.256779453	0.147890036	0.060395314	0.057672501	0.178749094	0.380904539	0.176039771	0.307095552	1.987130674	2.784405788	2.028352302	1.306443027
ACRV1	0	0	0	0	0	0	0	0	0.54820496	1.004329776	0.493291095	0.667220922
ACSBG1	0.018812339	0.013001776	0.013274159	0	0.019643444	0.025759449	0.032242843	0.01227198	0.027296705	0.034380826	0.013509312	0.013051835
ACSBG2	0.071106126	0.147430781	0.075259704	0	0	0.0365117	0.018280531	0.034788853	0.116071867	0.136448834	0.076592935	0.184998014
ACSF2	10.85514656	12.61126184	7.847454189	4.644278012	6.212071796	6.292032411	9.313840138	5.669312827	13.26257555	15.53002922	14.75345345	9.679677646
ACSF3	2.620227967	2.540348569	2.597091933	2.308391638	2.430056444	2.608818827	2.376104845	2.554132892	3.036246716	2.599375977	2.721998087	3.291607097
ACSL1	38.63881917	26.06070787	38.72018578	19.96431279	19.7872331	18.87827444	29.40712961	17.12843968	15.82214745	13.58298072	14.369336	25.93820861
ACSL3	17.3786633	19.51611075	16.42233599	18.86422822	23.99120026	25.46972111	19.29098104	18.50392842	41.7141406	39.96869849	42.80671947	29.40163587
ACSL4	25.98176972	26.27350695	25.14028795	21.03951778	26.67847628	28.33611105	24.15359883	18.97619223	23.29507307	27.83326783	26.79142251	19.98792813
ACSL5	32.76864283	29.35222215	32.43144491	30.40281995	31.89021023	31.99911169	35.61905838	31.67051137	24.05518145	22.29999725	24.19715353	20.93975918
ACSL6	0	0	0	0	0	0	0	0	0.059269491	0	0.016761626	0.008097007
ACSM1	0	0	0	0.010076926	0	0	0	0	0	0	0	0.010375931
ACSM2A	0	0	0	0	0	0	0	0	0	0	0	0
ACSM2B	0	0	0	0	0	0	0	0	0	0	0	0
ACSM3	0.097525258	0.202207963	0.025805519	0.123210622	0.012729225	0.075116165	0.075217718	0.119286199	0.119398349	0.200513279	0.144444662	0.088806591
ACSM4	0	0	0	0	0	0	0	0	0	0	0	0
ACSM5	0	0.035610675	0.018178353	0	0	0	0	0	0	0.037666374	0	0
ACSM6	0	0	0	0	0	0	0	0	0	0	0	0
ACSS1	7.776953945	8.811670965	7.296189645	6.42610823	7.575434044	8.893909625	7.474377154	7.072781417	9.366407784	10.1422942	8.939367231	6.867526648
ACSS2	8.818015152	15.27563978	7.841199253	8.779817045	9.77236126	10.41919703	7.97244423	8.419977877	36.17305675	48.90371691	49.87566641	23.16372361
ACSS3	0	0.013592622	0.013877383	0.013251747	0	0	0.013483228	0	8.64676018	7.461810499	6.920378848	7.668465192
ACTA1	0.252288631	0.149455115	0.19073269	0.218560624	0.225800932	0	0.185315365	0.141066117	0.235331241	0.158082718	0.271756146	0.225045809
ACTA2	18.03971156	13.30416292	13.31479772	4.721724889	6.288689805	5.202344426	14.55731665	6.499001613	1.776348802	1.049240966	1.121643847	1.669423239
ACTB	3430.037543	3459.979673	3712.805743	3026.780809	2943.667892	2882.370568	3096.322216	3259.742245	3256.74702	3173.802931	3585.483244	2956.578698
ACTBL2	0	0	0	0	0	0	0.019804999	0	0	0	0	0
ACTC1	0	0	0	0	0	0	0	0	0	0.015977356	0.031390037	0.015163525
ACTG1	2448.884618	2759.313216	2432.032569	2116.216045	2236.341354	2268.015229	2214.353065	2276.332419	1373.46236	1425.747504	1411.01641	1399.041373
ACTG2	0.04042792	0	0.17115787	0	0.253283841	0	0.124722392	0	0.043995685	0	0	0
ACTL10	0.716399479	0.522632304	0.533581284	0.509525741	0.69263805	0.354233709	0.354712615	0.493296666	0.461998083	0.174569155	0.457291548	0.3589673
ACTL6A	34.14943868	32.13463537	36.5247025	53.54675387	48.62208062	48.30320659	42.08430034	52.59881432	46.25079193	49.30170231	50.69415192	45.17896772
ACTL6B	0	0	0	0	0	0	0	0	0	0	0	0
ACTL7A	0	0	0	0	0	0	0	0	0	0	0	0
ACTL7B	0	0	0	0	0.039905397	0	0	0	0	0	0.041165983	0
ACTL8	0	0	0	0	0.028377171	0	0	0	0.118299509	0	0.029273588	0.028282272
ACTL9	0	0	0	0	0	0	0	0	0	0	0	0
ACTN1	37.68708721	37.31182127	37.11644455	36.71242979	35.58609329	35.46237686	34.19675845	38.04286982	80.0634937	72.5990387	82.26561371	91.57769253
ACTN2	0	0	0.022273282	0	0	0	0	0	0	0.011537813	0	0
ACTN3	0	0	0	0	0	0.01658975	0	0	0	0	0	0
ACTN4	147.799889	148.6176783	148.4949036	158.5595761	133.3856589	133.1457673	131.1086044	157.7669943	108.6840113	98.45047502	109.1391574	125.9227303
ACTR10	16.29798311	16.44306632	17.66623017	19.54156484	15.55801732	16.31114632	18.26532338	16.90964933	18.2831099	19.7879063	20.07362291	22.05397153
ACTR1A	46.80476596	39.82361072	51.28327366	51.24283028	41.01069152	38.5649911	49.46074177	53.91747829	29.73772957	26.96580004	28.78919287	35.68882136
ACTR1B	15.00297725	19.31155926	15.43580054	15.92587492	17.30675924	19.51761523	13.72605216	18.33010823	13.59062352	19.23156594	13.42960034	17.248882
ACTR2	262.3318678	232.2258671	255.5781396	247.0972302	244.0283786	228.8519042	273.7782683	209.8889736	331.1205969	293.6420033	311.9530126	315.3674641
ACTR3	83.06959574	82.32496083	90.0614864	81.77650883	74.13257368	74.19723988	87.08022849	72.25345748	79.91913071	75.72378486	82.97696213	81.73764139
ACTR3B	2.800914084	2.414214666	3.091577212	2.216171356	3.091778033	3.180517172	3.349407098	2.72504745	1.811439243	2.009518401	2.206751359	1.715611713
ACTR3C	0.006092338	0	0	0.004105006	0	0.004171073	0.004176712	0	0.002209996	0.006680497	0.006562445	0.006340216
ACTR5	8.856111671	8.078458931	8.45446693	10.06970246	8.612740411	8.247829637	8.236658712	9.770208564	4.936940591	5.283973822	6.008938217	7.002686018
ACTR6	14.27645144	15.47924513	13.88585053	18.55493987	17.893159	15.91680551	18.29540033	16.38328439	11.99672204	14.14668772	11.19260484	10.15476989
ACTR8	5.817494348	5.186821236	5.448698203	6.2592094	5.488781842	5.444746317	5.535842772	5.068314664	4.21895135	3.179638836	3.381708477	4.858416444
ACTRT1	0	0	0	0	0	0	0	0	0	0	0	0
ACTRT2	0	0	0	0	0	0	0	0	0	0	0	0
ACTRT3	0	0.055645009	0.056810754	0.054249545	0	0.027561326	0	0.026260812	1.16824453	0.47085785	0.838348831	0.670311009
ACVR1	2.569731618	2.268169122	2.097225549	1.773202659	1.831943952	1.483786766	2.249914766	1.807609162	1.942957129	1.821960926	1.634133187	2.212461289
ACVR1B	3.826632994	3.95682828	3.622180189	3.728015589	3.563165749	3.717116623	3.722141974	3.445215435	4.872699543	4.725973532	4.886800528	6.076126853
ACVR1C	0.011856243	0.012291312	0.006274406	0.011983071	0.012380037	0.006087965	0.006096196	0	0.077415345	0.260017073	0.127711146	0.203587492
ACVR2A	0.464642543	0.450946418	0.533638039	0.619489336	0.485169858	0.446713242	0.396485678	0.62877831	2.474444079	1.50681757	1.45889278	1.728424583
ACVR2B	0.68128768	0.876553574	0.492526314	0.642465535	0.962276734	1.058856854	0.475409536	0.544623891	1.92965543	2.407933432	2.080357327	1.737700399
ACVRL1	1.499709054	2.17663797	1.688900761	0.47291451	0.551219429	0.739270349	1.61625574	0.575249384	4.974514145	4.841384562	5.712167897	4.594781443
ACY1	0.256541411	0.565154987	0.305467838	0.226874958	0.267875083	0.362255688	0.395722297	0.282405456	0	0.070327025	0.103626407	0.033372407
ACY3	0.879816234	1.240457906	0.633222552	0.142276438	0.477716404	0.433698825	0.289523442	0.17218093	1.18725047	0.926164149	1.47842148	0.732490501
ACYP1	3.284712372	3.529073182	3.286952972	5.492841668	3.92871067	4.293265371	3.746332121	4.908819067	8.189057037	13.55594394	12.6730917	10.37937415
ACYP2	0.496053077	0.48053422	0.430351982	0.575330482	0.348142473	0.359105292	0.434853972	0.628620423	1.008862219	0.918460112	0.867191879	1.362524212
ADA	26.52486697	32.33187541	21.23848905	24.99344077	16.19247278	24.1542826	22.69523253	24.87327038	14.32008725	18.7844478	16.70398263	11.03444284
ADA2	7.771060496	12.63115476	5.148081399	7.333288972	2.388065072	4.622956651	4.148895407	6.327491985	14.03049529	10.40214801	9.333551801	14.80006815
ADAD1	0	0	0	0	0	0	0	0	0	0	0	0
ADAD2	0	0	0	0	0	0	0	0	0	0	0	0
ADAL	1.910527075	1.688040802	2.01063884	1.876107289	1.72289558	1.906301879	1.62980321	1.752619159	3.118696765	2.916294476	3.285704807	3.72798802
ADAM10	35.44237115	30.54946811	32.64446781	26.49265992	30.59730582	28.10789364	31.23896627	22.19999571	44.20552252	39.85005382	37.53148349	43.27383212
ADAM11	0.771813903	0.924100602	0.540763782	0.549345137	0.329175184	0.580513908	0.424795228	0.457389074	2.129391166	1.52576968	1.45196995	2.805601396
ADAM12	0.019464482	0.025223423	0.010300738	0	0	0.004997329	0	0	0.015886668	0.010671799	0.015724825	0.010128215
ADAM15	18.95303241	24.88359359	22.22928484	29.29706828	21.09144909	26.61998324	20.1310757	35.39072513	32.27662615	48.14555591	39.66544376	51.59679162
ADAM17	8.078604488	6.557979364	7.462720085	8.664264349	7.412264966	5.784593457	7.996316827	7.000641048	13.7335326	10.16895051	11.81718477	16.15129626
ADAM18	0	0	0	0	0	0.016909565	0	0	0	0	0	0
ADAM19	0.066334772	0.025006891	0.08935772	0	0	0.012386072	0.006201408	0.01180162	0.262504795	0.317405578	0.409233142	0.84095623
ADAM2	0	0	0	0	0	0	0	0	0	0	0	0
ADAM20	0.008000827	0.00414721	0.012702278	0.004043206	0.004177147	0.008216558	0	0.011743274	0.030474151	0.026319697	0.0215455	0.024979064
ADAM21	0.040397568	0	0.085514687	0	0.021091141	0.020743415	0.020771459	0	0.043962655	0.044297578	0.043514793	0.021020608
ADAM22	2.754612143	2.196268175	2.55386153	2.858462359	3.518536335	2.936859072	2.870552378	2.299321899	3.117843894	3.85061763	2.938855432	3.52865277
ADAM23	0	0	0.00887668	0	0	0	0	0	0.027380731	0	0	0
ADAM28	6.243811418	5.536151471	3.466065731	1.4488978	0.865503716	0.383753184	2.885533375	1.003862649	3.327173697	0.797155057	1.50027137	3.061555959
ADAM29	0	0	0	0	0	0	0	0	0	0	0	0
ADAM30	0	0	0	0	0	0	0	0	0	0	0	0
ADAM32	0.018333752	0.019006515	0	0.01852987	0	0	0.01885355	0	0.059855118	0.12062223	0.078993804	0.057239079
ADAM33	0.010185418	0	0	0	0	0	0	0	0	0	0	0
ADAM7	0	0	0	0	0	0	0	0	0	0	0	0
ADAM8	4.958560216	4.686510618	3.842705381	3.840800828	3.112474274	2.509860681	4.096749105	3.49729348	1.521991979	1.332206929	1.004324611	1.837716554
ADAM9	15.70701313	16.18724945	16.36686994	16.06248732	15.79572861	15.46388102	16.49803368	13.35038704	19.3890653	24.04917627	22.13832412	19.08450193
ADAMDEC1	4.966017717	5.971967089	3.177021434	2.944562041	0.023046267	0.135997847	0.88518111	1.468580445	0.048037947	0	0.071322851	0.114845978
ADAMTS1	14.43868672	21.06147446	13.33510618	15.59875616	7.272511885	9.7902855	10.77911462	10.69813315	1.787846163	2.461158595	1.707321738	2.564559332
ADAMTS10	5.904120425	9.712888802	4.89786681	6.278313257	7.200384065	9.247143035	5.684718581	7.717819615	2.567582972	1.849745267	1.350516289	2.194407177
ADAMTS12	0.005652265	0.011719353	0	0	0	0	0	0	0.055359697	0.0805732	0.091326214	0.017646712
ADAMTS13	0.33332559	0.270842044	0.276516099	0.18210336	0.169322346	0.175782473	0.166755905	0.176303125	2.137234227	1.511412904	1.669079697	2.381336063
ADAMTS14	1.723620993	1.049008185	1.443106331	0.182006142	0.456657646	0.202548294	0.961200526	0.201381718	1.493118905	1.466881657	1.367064883	0.660385421
ADAMTS15	0.009092525	0.009426178	0	0	0.009494221	0	0	0	2.256046403	2.652106045	3.663007294	4.210799254

ADAMTS16	0	0	0	0	0	0	0	0	0	0.01185541	0	0
ADAMTS17	0.019115297	0.027248017	0.032876828	0.036224579	0.059879359	0.053984462	0.046685976	0.08650792	0.065006946	0.047161578	0.064344698	0.06216574
ADAMTS18	0.018175835	0.009421402	0.009618777	0	0.037957641	0.027998881	0.009345578	0.008892574	0.049449634	0.019930544	0.019578349	0.018915352
ADAMTS19	0	0	0	0	0	0	0	0	0.012922489	0.026041873	0.006395421	0.018536544
ADAMTS2	1.442318126	2.72211178	1.448283786	2.100650498	0.432503192	0.949492415	0.380310432	1.158002547	6.938449153	8.386326196	6.310057635	13.6321718
ADAMTS20	0	0	0	0	0	0	0	0	0	0.00792963	0.007789505	0
ADAMTS3	0.008155435	0	0	0	0	0	0.008386658	0	0.532509158	0.697535799	0.632501161	0.28856662
ADAMTS4	0.197965733	0.102615078	0.209529662	0	0.090436328	0.114358272	0.305367677	0.036320714	0.996392509	0.379885602	1.346086933	0.077257621
ADAMTS5	1.130905502	1.13332433	1.020271002	1.649189961	0.365506596	0.685778309	1.002368433	1.006476112	0.052744627	0.047241293	0.029004055	0.117691841
ADAMTS6	0.078211571	0.131274926	0.055186804	0.045170406	0.066111266	0.095619564	0.065109209	0.058308891	0.214810882	0.245034777	0.17250507	0.259684816
ADAMTS7	0.018126852	0.009396012	0.009592855	0	0	0	0	0	0.147949108	0.218645147	0.126916313	0.179211568
ADAMTS8	0	0	0	0	0	0	0	0	0.350298646	0.735348633	0.664565894	0.432693386
ADAMTS9	2.23685307	2.654570539	2.172819087	3.190378412	1.598093001	1.987351375	1.520903698	1.48318108	0	0	0	0
ADAMTSL1	0	0	0	0	0	0	0	0	0	0	0	0
ADAMTSL2	0	0	0	0	0	0	0	0	0.550536239	0.554730417	0.611382336	0.423747687
ADAMTSL3	0	0	0	0	0	0	0	0	0	0	0	0.00562803
ADAMTSL4	4.453681767	6.592948132	3.720901109	2.135882929	4.67106254	5.222818161	4.326074787	2.782903901	9.81165144	11.77055513	8.764992442	12.13703423
ADAMTSL5	4.220357715	3.883012393	4.150480468	3.074717048	2.680808039	1.697543498	3.90601185	3.596229418	2.392085664	1.639010399	2.083590654	2.616942005
ADAP1	24.94649057	22.70167735	27.99650884	20.16287714	15.5324567	15.5933958	22.7372185	23.938298	19.12749092	14.47077521	19.14045503	28.51145082
ADAP2	1.477529449	0.997417246	1.09104945	0.451473303	0.358791818	0.317588842	0.689039447	0.285791757	3.683262018	3.221503197	3.516195305	3.575919466
ADAR	52.6042347	41.78494625	44.39130254	49.36064424	43.82037618	44.24329307	43.80936518	43.48674794	100.9080849	85.48203206	81.90275869	107.9172013
ADARB1	4.41936826	4.429396882	4.091506448	4.352027066	3.670791902	4.661841202	3.796939865	4.268891728	1.110694022	1.005777161	1.0311169	1.260001257
ADARB2	0	0	0	0	0	0	0	0	0	0.007002655	0	0
ADAT1	9.018710316	7.549869548	8.895338443	9.322143393	7.966923631	7.488139571	8.514511272	8.162771979	9.359244924	7.761164396	7.921304967	11.25722404
ADAT2	13.53403342	10.6340043	11.22509798	7.391318307	11.66668478	12.33752552	10.28966558	7.01445339	4.590976675	5.841145406	4.786410709	5.49904618
ADAT3	2.232010933	2.970567019	2.394315357	2.042492318	2.834535805	2.106340349	1.954100659	3.659732686	1.739679162	1.455264838	1.85191542	2.448382348
ADCK1	4.255589172	3.218943168	3.30306113	3.520539619	3.275094589	3.658132289	3.322703402	4.102424995	1.835305655	1.72830622	2.003362927	3.789029183
ADCK2	4.054144996	5.879833223	5.591254785	7.822524251	4.960174004	5.971830378	4.842879989	7.873896816	6.573320332	7.16225092	6.748965495	9.162681317
ADCK5	5.959534832	5.962074356	6.307653854	6.971556969	6.150250157	6.459246368	5.413805575	8.65364563	4.991727434	3.696108693	4.192257546	6.14776416
ADCY1	0.016633558	0.017243933	0	0.004202872	0.034736815	0.038434631	0.034210305	0.048828063	5.701963207	6.794166753	6.024612938	6.794302255
ADCY10	0.018561421	0.019242539	0.01309711	0.0250133	0.025841922	0.006353968	0.012725116	0.018162445	0.060598403	0.04749116	0.053316503	0.019316626
ADCY2	0.016422878	0	0.0086911	0.008299278	0	0	0.00844425	0	0	0.027012532	0	0.008545536
ADCY3	26.65900304	24.73150274	26.2126698	24.29098493	15.36536942	17.82941909	19.41301808	19.21908637	15.68382473	18.59010512	17.7208474	21.50706008
ADCY4	1.808116964	2.234364014	1.530988745	1.28653087	1.011966534	0.906152681	1.383379197	1.089859	4.014074073	3.616397137	2.695530842	5.042911589
ADCY5	0	0	0	0	0	0	0	0	0.027392472	0.048302026	0.040670123	0.085134567
ADCY6	0.020418503	0.049391456	0.079241158	0.006878974	0.007106855	0.013979372	0.013998272	0.013319739	4.96997097	4.836179431	4.743387588	5.057325293
ADCY7	19.96860737	23.64468415	17.53741661	22.66912201	20.39787487	21.95677901	18.75864624	19.44677992	25.67877842	21.64560296	22.5373589	26.92018001
ADCY8	0	0	0	0	0	0	0	0	0	0	0	0
ADCY9	13.12262477	9.149329032	11.80219375	7.002588679	9.002383202	7.373647239	10.61394805	7.338410405	19.75619742	13.28604942	15.52676418	19.74184076
ADCYAP1	0	0	0	0	0	0	0	0	0	0.047405764	0	0
ADCYAP1R1	0.023813052	0.00822896	0.016802709	0	0	0	0	0	0.051829111	0.008703994	0.03420074	0
ADD1	28.37707708	29.80947029	28.33775194	34.08207839	31.09695846	29.50811634	29.95750082	33.70402637	46.33343689	40.21719771	43.27656706	45.86784197
ADD2	2.751679184	3.369081587	2.671387732	2.747549075	2.377857815	3.303349578	2.092998327	2.516124771	2.793159678	4.947377888	3.802107875	2.676018618
ADD3	7.453947365	9.016908851	7.067746795	9.441621893	9.957037593	9.693227978	8.082563046	7.923834131	12.27803455	13.13572793	10.01502224	12.25549671
ADGB	0.005220175	0	0.005525105	0	0.005450795	0.005360929	0	0	0.028404277	0.022896536	0	0.021730267
ADGRA1	0	0	0	0	0	0	0	0	0	0	0	0
ADGRA2	27.19132979	47.11898203	22.45262851	31.14470986	24.0563829	38.72668873	24.9157859	37.46010401	41.44078523	38.29531817	37.628096	60.742702
ADGRA3	3.806250468	3.168545985	3.958263509	3.875746713	3.78609227	3.743167417	3.348026562	3.399359987	12.84996856	10.58519116	11.23653419	11.9722465
ADGRB1	0.484459069	0.600890057	0.302161058	0.148641136	0.135498711	0.213223634	0.275786206	0.126976515	4.161213766	4.724144441	4.258601057	2.889975326
ADGRB2	0	0.019642296	0.040107592	0	0	0	0.009742107	0	0.618573138	0.914152279	0.673498669	1.054908834
ADGRB3	0	0	0	0.003586702	0	0	0	0.003472461	0.173786293	0.186784275	0.152902997	0.217894548
ADGRD1	0.31768674	0.664367085	0.243487054	0.166078487	0.543337324	0.545629528	0.298530527	0.176867532	0.417251425	0.264270406	0.289100517	0.245109187
ADGRD2	0	0.015308486	0	0	0	0.01516478	0	0	0	0	0.031812132	0
ADGRE1	0.137581264	0.475432855	0.129438139	0.216304648	0.079810794	0.109892957	0.188642618	0.149582183	0.615527134	0.301726914	0.493991793	0.986344241
ADGRE2	31.11853106	26.90170362	28.41388599	17.49875276	20.26932413	18.13713179	31.12915771	14.53479158	2.638712939	1.744609648	2.746539089	1.793121615
ADGRE3	0.111039127	0.115113747	0.070515206	0.022445385	0	0.022806628	0.114187305	0	0	0.024351785	0	0
ADGRE5	38.16903845	38.59519989	35.081739	24.18676026	37.65094754	32.56758984	37.61904025	28.73896947	27.33389433	22.30405903	29.89376526	26.58825151
ADGRF1	0	0	0	0	0	0	0	0	0	0	0.009533175	0
ADGRF2	0	0	0	0	0	0	0	0	0	0.011510803	0	0
ADGRF3	0.072646903	0.075312706	0.053823339	0.198244843	0.144127065	0.111908584	0.05229461	0.241690234	0.442724773	0.430165548	0.320835676	0.567020037
ADGRF4	0	0	0	0	0.017724542	0	0	0	0	0	0	0
ADGRF5	0	0	0	0	0	0	0	0	0	0	0	0
ADGRG1	0	0.019012993	0.009705655	0.009268093	0	0	0.009429988	0.017945783	0	0.010055279	0.019755182	0.028629294
ADGRG2	0	0.029489404	0	0	0.009900757	0	0	0	0.020637271	0.020794493	0	0.029602942
ADGRG3	0.053367374	0.189688144	0.096831025	0.046232787	0.031842901	0.101783219	0.062720508	0.029840141	0.016593442	0.008359928	0.057485396	0.015868206
ADGRG4	0	0	0	0	0	0	0	0	0.005704097	0.005747553	0.005645987	0
ADGRG5	6.331184263	6.574924104	6.316432876	5.976025279	5.518654255	4.907517704	6.997571874	6.744573999	4.397560946	1.871429921	4.839037261	3.002191835
ADGRG6	0	0.006753526	0.00689501	0	0	0	0	0	0.850725284	0.992930755	0.87012727	0.616937021
ADGRG7	0	0	0	0	0	0	0	0	0	0	0	0
ADGRL1	4.423392205	7.328016921	5.147297143	4.502461996	7.367792487	9.382146982	6.609980936	5.662464618	3.494076291	4.368142613	4.101447036	4.315655445
ADGRL2	0.016142868	0.005578412	0.011390556	0.016315551	0.117992277	0.071838596	0.077469235	0.031591757	37.46557287	27.08890826	29.53148801	31.30338445
ADGRL3	0.260393548	0.263597045	0.230210502	0.133137617	0.179132407	0.235954136	0.248874367	0.158873054	11.19491188	6.056640305	7.718327632	6.650367377
ADGRL4	0	0	0	0	0	0	0	0	0	0	0	0
ADGRV1	0.005522611	0.01145053	0.020458227	0	0	0	0.002839594	0	0	0.009083652	0.005948756	0.002873654
ADH1A	0	0	0	0	0	0	0	0	0	0	0	0
ADH1B	0	0	0	0	0	0	0	0	0	0	0	0
ADH1C	0	0	0	0	0	0	0	0	0	0	0	0
ADH4	0	0	0.026465047	0	0	0	0	0	0	0	0	0
ADH5	41.45284044	41.27015338	40.71737306	36.89250313	40.12737429	39.92422271	44.13042235	37.48443766	47.47370011	47.43488918	48.73853664	39.99318039
ADH6	0	0	0	0	0	0	0	0	0	0	0	0
ADH7	0	0	0	0	0	0	0	0	0	0	0	0
ADHFE1	0.028395547	0.088312594	0.150271191	0.086097892	0.20755018	0.058322384	0.146003081	0.027785186	0.154507275	0.342505603	0.152933255	0.443263051
ADI1	26.06546319	19.78306049	26.11980893	33.3780725	22.66460551	20.90224451	29.69350321	35.05252356	22.61901481	14.57213751	18.55879506	23.00128081
ADIG	0	0	0	0	0	0	0	0	0	0	0	0
ADIPOQ	0	0	0	0	0	0	0	0	0	0	0	0
ADIPOR1	49.45909412	51.63846627	50.13891241	51.36500407	44.23362506	44.72283744	49.48306152	53.25492179	50.09373154	60.59452899	50.71944914	50.57965056
ADIPOR2	47.16463926	45.43076127	45.63929986	52.07202465	50.49751187	46.29966405	49.22396157	47.89406047	30.69426282	25.26721879	29.7092251	25.72593202
ADIRF	0	0	0	0	0	0	0.082344	0	0.348561054	0.965845413	0.17250507	0
ADK	35.07208635	30.87375612	35.48952069	29.68247813	29.29722112	28.32815118	33.98233246	30.44702486	13.3122966	14.85089801	16.37934476	11.27808224
ADM	5.245586122	8.560582198	4.15504568	3.454655364	7.06752184	8.793016363	4.350249047	3.841345714	0.810240035	1.818373801	0.947801443	0.070438866
ADM2	0.315521491	0.354357968	0.250464223	0.225885137	0.590283983	0.283525441	0.365025539	0.16723383	0.400593989	0.259486621	1.359473137	0.259950911
ADM5	1.656070765	1.743253803	1.833706974	2.266048692	2.287909414	1.936209249	2.384233086	3.066434746	1.497228164	1.704198312	1.783859251	1.829509479
ADNP	9.997647559	10.64090183	10.05088602	11.88171903	14.33005292	13.60488109	11.35816823	11.7455099	18.65132642	17.3317084	18.0715799	15.86756577
ADNP2	12.9506473	13.14126301	12.97569525	13.998153	14.84739119	15.01093385	13.2691474	12.94091148	23.16986631	19.89996228	23.77000901	20.07842291
ADO	16.90579651	15.84844274	17.5109921	17.56857084	15.61582615	16.04096555	14.44301374	15.22749834	15.72455882	15.63837741	14.49042591	18.67632448
ADORA1	0.352904678	0.470384536	0.231226158	0.186832247	0.315853334	0.258871605	0.120970073	0.082218814	0.603504835	0.755521345	0.995594535	0.454706861
ADORA2A	0.800683354	0.905525141	0.693371703	0.55176025	0.646043677	0.953088734	0.767244465	0.641023138	0.217835924	0.379128547	0.411631984	0.530256736
ADORA2B	2.631252262	3.168380631	2.574407355	1.964848135	2.586990896	2.488624247	2.045662398	2.141154202	0.511683644	0.723797568	0.896063979	0.470499906
ADORA3	0.116521353	0.048318858	0	0.023553559	0.048667647	0.047865274	0.023964993	0.022803347	0.202886989	0.204432653	0.200820108	0.218272018
ADPGK	27.85374749	26.76772971	26.74352502	28.46789542	24.15161228	25.23132566	27.05632336	26.76523856	23.8318488	23.08717671	22.24111885	28.08626942
ADPRH	0	0	0	0	0.134597069	0.058834664	0.029457103	0.084087722	4.909728766	3.345204082	4.273460717	4.874005834
ADPRHL1	1.087061836	0.845213965	1.202859477	0.84898794	0.644935705	0.380581671	0.711379569	0.435148168	0.403293781	0.243819729	0.425797639	0.668490069
ADPRHL2	12.02839385	12.30483583	12.05741911	13.54000945	11.46330309	12.94094713	11.97674243	14.26081678	12.81286524	12.03814852	14.12195264	14.80278402
ADPRM	8.542888121	7.496680386	6.640739322	5.374028518	7.328712865	7.899551719	7.58215739	5.896573001	2.777466738	2.837496263	2.023695846	2.471624652
ADRA1A	0	0	0	0	0	0	0	0	0	0	0	0
ADRA1B	0	0	0	0	0	0	0	0	0.034547644	0.040612647	0.056992826	0.022025132
ADRA1D	0	0	0	0	0	0	0	0	0	0.154925214	0.043482148	0.084019353
ADRA2A	0.013882756	0.014392188	0.0146937	0.042093784	0	0	0.028552718	0.013584347	0.468345193	0.213122095	0.358895998	0.260056806
ADRA2B	0.063849968	0	0	0	0.033335389	0.016392898	0	0	0	0	0	0
ADRA2C	0.164891402	0.284903584	0.116348889	0.083327633	0	0.056445821	0.197827464	0.107564716	1.28600871	0.934185702	0.799267619	1.80180318
ADRB1	1.034076117	1.656761127	1.114380067	1.140150285	1.393872363	0.907491807	1.546755217	0.993451478	3.048630434	2.700759273	3.058077307	3.208881694
ADRB2	5.560145613	9.233610741	4.490400485	5.513090059	3.35689982	5.737128901	3.224723299	3.480972245	3.584614169	3.87198147	2.44109036	2.056766608
ADRB3	0	0	0	0	0	0	0	0	0	0	0	0.02115561
ADRM1	26.86382947	23.34951821	30.42682837	32.65593457	27.87959871	24.32890052	29.56262394	36.78492253	25.78078517	20.54376048	30.94378624	31.00395655
ADSL	30.82433841	30.39467702	33.00953533	31.12783528	35.07270498	36.12056023	31.15105688	35.39404516	28.94015165	30.37031714	33.59430147	28.64777242
ADSS	30.95240227	27.43365783	31.82443658	30.98098226	36.97957948	34.65131157	34.75412314	28.8492783	16.90860419	16.38098686	19.43292517	14.71121514
ADSSL1	0.808094112	1.182702227	0.70436302	0.720651527	0.645254755	0.805474882	0.366619929	0.907007158	4.009067953	4.795408516	3.174580942	5.045837174
ADTRP	0.210222925	0.202370192	0.150984072	0.022764826	0.031358615	0.007710403	0.154416542	0.029386314	0.057193777	0.041163928	0.016174607	0.039067182
AEBP1	0.026076841	0.067584349	0.082800264	0	0	0	0.013408085	0	0.014189062	0.057188635	0	0
AEBP2	4.86348164	4.627068456	4.829897061	4.93236009	5.832840887	5.240068274	5.390048891	4.28032792	3.006244578	2.931629383	2.999567558	2.597201279
AEN	10.49729291	8.354581933	11.63128332	8.244530741	8.791675461	6.692792787	12.69526611	9.832969138	5.461946757	4.460399768	6.819716809	5.62151108
AES	85.15543749	103.189918	92.99919135	93.11241918	87.97385985	95.69817417	106.607821	108.3124298	66.51026606	77.32291091	77.84779681	66.61289358
AFAP1	0.019569485	0	0.006904204	0	0.013622693	0.006699049	0.006708106	0	1.597236979	1.902674697	1.904184955	1.615679225
AFAP1L1	0	0	0	0	0	0	0	0	0.117732345	0.200757228	0.143425187	0.043302582
AFAP1L2	0	0	0	0	0	0	0	0	0	0	0	0
AFDN	9.023434003	7.903790546	8.133078016	7.933705151	9.113071366	8.375606416	9.150315792	6.934991398	1.735787262	2.134013539	1.966634986	2.515983337
AFF1	36.70222665	31.26286714	31.60449816	19.88653769	38.8720913	28.37553449	29.76904373	16.93583542	31.65886959	22.03109204	26.11105319	23.88524461
AFF2	0.007749631	0.032136025	0.004101158	0.046995177	0.012137999	0.019896471	0	0.037915265	4.258935669	4.966955686	3.063576761	8.335113696
AFF3	0.780592465	1.848083542	1.020587437	1.266675948	1.432732341	2.956914408	1.227695221	1.250117212	2.051684742	2.168015361	1.934772258	1.956391607
AFF4	7.93182746	7.430388804	7.207324264	6.285117217	6.278202487	5.851762413	6.484110856	4.450962908	14.60858887	14.89823296	12.48001619	14.72051658
AFG1L	1.470377535	1.70595628	1.675471357	1.454487067	2.02533809	1.734921374	1.660055036	1.481628913	1.695465815	2.13376284	1.80624844	1.679966965
AFG3L2	37.49407711	37.4245727	41.19502534	46.09822434	37.72938329	40.51638187	42.65348073	44.4837664	38.70769429	34.78018488	41.60298717	39.10595526
AFM	0	0	0	0	0.028135603	0	0	0	0	0	0.029024388	0
AFMID	10.56009699	9.998811172	10.4566598	8.704473125	9.507404217	12.04981652	10.69057103	10.03459646	12.20708542	11.63104108	12.96744613	10.7699583
AFP	0	0	0	0	0	0	0	0	0	0	0	0
AFTPH	20.10682184	17.15185895	18.39503024	19.12184638	19.25119943	16.02606897	19.8718195	16.55937149	16.03110384	15.79554541	13.56281378	15.49339078
AGA	7.957571768	8.30262962	7.609953184	9.852947806	7.854930479	7.988197529	7.025435009	9.163562419	9.049659318	6.930138119	9.839433289	10.43821605
AGAP1	1.401674878	1.290973367	1.380484197	1.65526562	1.904219444	1.718271456	1.107613725	1.388870772	2.064873007	2.320051352	2.221838819	2.315501493

AGAP11	0.30699591	0.335942413	0.288825515	0.017237771	0.249323354	0.315273615	0.333238729	0.100132352	0.482572007	0.355335376	0.238827939	0.230740312
AGAP2	8.218216804	8.294042075	7.878809524	7.491002689	7.309673443	7.975992446	7.422808041	8.041566841	8.504638953	7.517044094	7.966259331	8.955459391
AGAP3	7.215510409	7.856540024	8.167469681	7.179156671	8.553891939	8.927576894	8.379807822	8.743074978	5.538913347	6.680276845	6.990402995	7.113397941
AGAP4	5.718890526	6.082740668	5.188245088	6.136628755	5.58378123	6.654899945	5.670995466	5.105408164	5.31429317	4.947571762	4.540133447	5.796987457
AGAP5	7.142076916	6.668341011	5.845524544	6.613200633	7.156521342	7.630772308	5.611068136	7.010262098	4.248249482	5.545341035	4.467781774	4.570396525
AGAP6	17.71137568	14.06662371	15.71165989	13.6531804	18.85431485	17.35656244	16.03716581	16.12633621	6.746041888	6.089712276	6.56414273	9.309719305
AGAP9	19.13880066	19.72425585	16.36766149	16.60946289	16.61829905	21.02073581	17.20096128	17.00687379	10.35256995	10.60447281	9.008710539	12.92643203
AGBL1	0	0	0	0	0	0	0	0	0	0	0	0
AGBL2	0.152119	0.178727868	0.064401939	0.030749247	0.148250128	0.07290298	0.093859124	0.10915609	0.419376888	0.244646859	0.196628471	0.443263051
AGBL3	0.321431015	0.260332843	0.180734979	0.172586871	0.377585356	0.391991306	0.227249149	0.206404983	0.360728483	0.297389977	0.335414003	0.428589668
AGBL4	0	0.005408583	0	0	0	0.005357811	0	0	0.039742854	0.074370454	0.089915383	0
AGBL5	3.764757469	4.84814176	3.190850005	4.786013744	4.975272686	5.467145669	4.098341212	5.698431161	9.906411894	12.56201356	10.61337564	8.325608354
AGER	6.430668082	9.019576795	6.072294199	7.104799413	8.327627448	8.449314403	7.747571835	9.222744914	14.61383568	20.42857952	13.37838971	12.40045915
AGFG1	8.042049717	7.113303888	7.49694592	8.836678094	7.530688952	7.110059001	7.506667725	7.561481084	10.81627894	10.36166106	9.417805078	12.6450857
AGFG2	2.427813714	2.082954369	2.071211611	1.713418381	1.671835899	1.837716533	1.904769489	2.49850535	1.617128064	1.354048264	1.228670887	1.992959644
AGGF1	15.07240527	13.70787466	14.33608489	16.18649285	16.99684926	15.96903471	15.49973764	13.76753216	22.62330535	17.60055079	20.7345815	23.72097955
AGK	0.87268784	0.745905699	1.041579506	0.614601198	0.455621038	0.46717779	0.630110607	0.35428989	1.74280527	1.924055685	2.020059375	1.739096237
AGL	4.722160429	4.325713636	4.107551495	4.368093859	5.398353367	4.264203838	4.549051753	3.12691076	10.4033015	8.76398348	8.8795196	7.526771676
AGMAT	3.480390113	2.281595339	3.015572124	1.293242754	2.173363802	1.787116923	2.842199492	1.46907346	3.044880836	2.824876514	4.171625469	1.45590035
AGMO	0	0	0	0	0	0	0	0.006670839	0	0	0	0
AGO1	3.103366038	3.32847969	2.987629604	3.612052765	2.740609244	3.136186773	2.957942481	3.077040523	4.342805014	4.579525028	3.98295011	5.535898505
AGO2	14.79640936	11.32226933	13.57667785	8.926396958	12.34808825	10.58517665	11.93955055	6.976576197	8.296299715	7.453051606	7.649077445	7.21381238
AGO3	1.364767771	1.13560201	1.349456857	1.515488596	1.153174742	0.922083407	1.13569592	0.843430856	1.065050896	1.260230255	1.141244958	1.261446912
AGO4	7.112458311	7.927644142	6.375620296	8.2559506	8.951495057	9.00476336	7.052652848	6.953197465	18.53800893	17.0350647	15.49109744	17.53781845
AGPAT1	21.08283664	24.75293191	21.75490748	19.20194839	17.51993637	19.20079924	21.05935662	20.49666337	29.68178845	30.23912084	29.42821272	28.33736026
AGPAT2	24.78437784	30.41459865	27.33586338	30.38964524	26.71413682	28.9510542	27.7390764	36.462493	56.13724385	50.39004063	62.71695971	62.7636237
AGPAT3	8.483416119	8.525148166	8.267986574	9.658254043	7.755169729	8.372796753	8.066578384	9.790592833	14.9682286	14.63080379	14.79240096	17.09902952
AGPAT4	2.659232066	3.370204535	2.730130856	1.861217161	1.707678832	1.863862761	2.085152731	1.574253602	4.094881826	3.703264414	3.824011583	3.812131611
AGPAT5	27.01197079	25.1240693	29.621222	27.75396688	38.0853291	34.8455119	30.07258942	27.0415981	12.58758622	13.16432256	14.98900993	11.06388965
AGPS	9.170155723	9.136583717	10.10842187	11.58087658	10.44909469	10.4450981	9.905566757	10.22136781	13.15092994	12.16664424	12.85936655	13.14253656
AGR2	0	0	0	0	0	0	0	0	0	0	0	0
AGR3	0	0	0	0	0	0	0	0	0	0	0	0
AGRN	14.95435611	13.93098616	13.19375381	5.162777602	7.758263457	7.391906	9.707531474	6.21952066	14.26054642	10.43078948	12.28638055	11.69664163
AGRP	0	0.073015866	0	0	0	0	0	0	0.076646933	0	0	0.073296988
AGT	0.770492938	0.561295342	0.727338151	0.568266081	0.369649991	0.23524189	0.877996085	1.589368579	28.01006414	34.89113158	31.89688181	45.68331221
AGTPBP1	3.695323469	3.830924669	3.154178485	5.452795867	3.167076687	3.445844151	2.946547748	4.237981842	6.933013205	7.85240361	6.62460233	9.538374958
AGTR1	0	0	0	0	0	0	0	0	0	0	0	0
AGTR2	0	0	0	0	0	0	0	0	0	0	0	0
AGTRAP	28.0496647	36.83617155	27.35118373	33.06089277	27.06871606	29.72979078	28.00396794	36.20889068	23.18301818	27.66390499	26.33822096	31.74405664
AGXT	0.875499739	1.640709462	0.677160729	0.068066544	1.863517101	0.414972154	0.934949645	0.131797067	0.219868299	0.073847778	0.108814212	0.035043116
AGXT2	0	0	0	0	0	0	0	0	0	0	0	0
AHCTF1	20.47718663	15.28265746	19.69516566	18.27596678	23.90668085	20.53148265	20.90760006	15.6878637	33.71882706	25.25282189	29.60683571	26.36136158
AHCY	59.00396522	61.86397719	60.77170353	50.65938375	52.86248109	57.77281905	56.26565337	58.35872625	35.50937697	47.29414352	45.61611347	34.29453012
AHCYL1	18.90049994	20.94150301	18.84669333	24.12739766	22.61948415	24.38223132	18.62326906	21.79034232	47.00691006	61.18972155	46.3413621	51.60285002
AHCYL2	3.872872341	4.374649544	3.529573683	3.785493972	3.038522926	3.511947564	3.349233845	2.972119244	3.944977318	3.8508118	3.40906336	5.850411273
AHDC1	2.789356773	3.296679606	2.919992871	2.4552288	3.212130956	2.645180753	3.025357409	2.813013752	1.653925364	1.057474041	1.439838147	1.448247384
AHI1	8.078606724	7.521894339	7.927200653	8.195548067	6.748407412	6.264971309	4.782722522	4.255379082	6.12121531	7.227559816	5.717283243	5.892966764
AHNAK	105.1074544	100.7562977	81.15991401	51.63088623	60.28574283	69.4243319	71.88155193	39.6890775	101.5341649	87.38787993	92.74768439	52.08965062
AHNAK2	0.02900785	0.018043382	0.024561846	0.00586363	0.012115752	0.008937001	0.020881195	0.002838433	0.470360124	0.203573044	0.671793331	0.081507831
AHR	3.850307941	3.116481267	3.172653737	2.663976563	2.98607606	2.671467662	3.658303879	2.486411695	2.409071885	2.474651205	1.614428756	2.859556384
AHRR	87.2207261	76.82264843	79.96126229	50.17730712	86.54811589	74.19797661	74.3471625	53.3412019	55.17571863	54.18896701	48.92086559	44.12843803
AHSA1	73.49685703	67.78548652	84.63500967	93.3503724	81.22089019	79.0011735	94.90752897	101.9145326	78.31097591	82.47291368	85.78793988	72.32261503
AHSG	0	0	0	0	0	0	0	0	0	0.034424956	0	0
AHSP	0.093581845	0	0	0	0	0	0	0	0	0	0	0
AICDA	0	0	0	0	0	0	0	0	0	0	0	0
AIDA	8.323834265	9.779851009	8.866900083	12.44743712	11.34577071	10.53368023	9.793183404	11.64810291	15.68176877	19.11851651	15.23278306	15.64839488
AIF1	124.8437525	144.1049701	127.664647	93.0976484	94.38800426	120.1998413	138.3140478	100.7174651	50.97953527	34.66952453	51.7104414	37.73523727
AIF1L	0.029117728	0.135837959	0.046227907	0	0.015202056	0	0	0	0.602060002	0.287358966	0.705702561	0.166663389
AIFM1	28.44266892	24.51484143	29.80657239	26.00593803	24.24285964	25.6093336	30.5244703	29.27140361	20.94708396	21.0885333	23.85086699	19.91110201
AIFM2	6.506817667	5.183632232	6.46633117	3.698191465	6.310209692	6.35920354	5.839988487	4.260836969	1.459451945	0.889604413	1.605093332	0.947673673
AIFM3	2.171113611	1.434322749	1.509428935	1.032629784	1.311321645	1.092967931	1.13822339	1.083050741	5.4898366	4.574706239	4.746073045	5.604320176
AIG1	0.065023088	0.24512412	0.031282424	0.215079212	0.049378709	0.078917495	0.054709053	0.133034972	17.07922361	21.55866737	17.42098442	21.66627724
AIM2	0.144261557	0.130860875	0.152688422	0.036451185	0.056488068	0	0.166895614	0	0.019624081	0.019773584	0	0
AIMP1	17.15561469	16.72673139	17.57885271	17.22857281	19.65538526	19.74323346	19.97615496	17.3309571	16.5169402	18.88179962	18.12591986	16.1745083
AIMP2	22.86308453	19.21453081	25.86921889	25.33140297	22.94918575	23.18483239	25.55255086	26.25626026	11.00895233	11.40751237	11.10288635	12.86730253
AIP	20.7621365	22.00161421	22.16997537	21.97756813	23.57151655	24.7915071	23.53007995	24.61344081	25.66937296	26.43746225	25.07703846	23.62054009
AIPL1	0	0.015521458	0	0	0	0	0	0	0	0	0	0
AIRE	0.023405638	0	0	0	0	0	0.024069234	0	0.025471186	0.051330469	0	0
AJAP1	0	0	0	0	0	0	0	0	1.473049324	0.778784444	1.147533729	1.517361467
AJM1	0.46005641	0.370952044	0.703343404	0.585527464	0.373629757	0.209982743	0.38548882	0.416821789	0.797341689	0.579206968	0.532263903	1.188070368
AJUBA	1.200828984	1.470262538	1.183320581	1.297376088	1.567350096	1.190683217	1.479720726	1.428253731	1.588440601	1.577839176	2.051741722	1.648293867
AK1	0.769664117	0.693832359	0.531275945	0.236751356	0.489188545	0.223378719	0.550598685	0.163721792	7.228740067	6.586620146	7.515556724	6.91279939
AK2	97.36581676	106.3609775	96.78250418	132.5951789	96.99685147	115.3181213	95.27627196	127.5688595	84.81967753	95.89817705	83.28534392	107.1646432
AK3	6.053866648	6.167392079	5.840679	5.871527289	7.233047422	7.400740773	6.943833269	6.630031217	25.45295077	27.79154543	23.62610335	27.6965562
AK4	0.895523005	2.814626138	1.000491337	0.481284678	1.202253517	1.510885606	0.321588613	0.354682254	6.079354121	7.505113261	7.249997803	3.79440432
AK5	0.100941456	0.064397254	0.073964651	0	0.202694079	0.127585474	0.151712581	0.045586951	0.042249825	0.085143397	0.050183293	0.040403246
AK6	16.1882946	15.62640401	18.88227285	19.03272209	20.39628224	20.73857578	20.08713308	20.44695451	23.3693735	25.44931572	25.2664986	27.2043768
AK7	0.014662006	0	0.015518469	0	0	0	0.015077702	0	0.079779642	0.016077486	0.01579338	0.137327
AK8	0.008372423	0	0.008861488	0.008461984	0.008742306	0	0	0	0.054667736	0.027542107	0.036073878	0.052278418
AK9	0.405539259	0.416857779	0.37466542	0.489768715	0.423455466	0.395297398	0.346352842	0.474168927	0.733053483	0.667035464	0.610824622	0.704591114
AKAIN1	0	0	0	0	0	0	0	0	0	0	0	0
AKAP1	20.36517037	19.21873911	20.53835508	21.35316804	21.71175613	21.35379893	18.94598861	21.99064106	14.7332802	14.5135881	16.83194382	17.07667681
AKAP10	10.66720945	10.4723064	10.53856159	9.212642835	9.929859548	9.360913661	11.26721947	7.966796048	5.925963927	5.92784108	5.511391524	5.571144421
AKAP11	12.8612182	9.966420514	10.18614319	10.14960208	11.19746505	10.07965186	10.02425609	6.790014936	8.877571115	11.28907335	8.2754764	8.983895496
AKAP12	0.004894448	0.020296206	0.020721405	0	0.076660176	0	0.005033215	0	0.825589398	1.143162792	1.049152176	1.227554541
AKAP13	6.236783587	6.047448148	5.742996909	4.544755012	4.150208189	4.902202961	4.729883893	4.043973989	5.17753809	4.61418891	4.741391752	4.679608112
AKAP14	0	0	0	0	0	0	0	0	0	0.064556208	0	0.061267942
AKAP17A	1.726599616	1.216476174	0.762918877	0.813236166	1.242760967	1.29113221	0.948110353	1.39423642	1.860729664	2.389585254	1.877886972	1.517727165
AKAP2	0	0.015716051	0	0.015321924	0.007914748	0	0.02338435	0.00741695	0.008248804	0	0	0.00788828
AKAP3	0.029638976	0	0	0.044934042	0	0.015219074	0.030479299	0	0	0	0.015963014	0.030844891
AKAP4	0	0	0	0	0	0	0	0	0	0	0	0
AKAP5	0.282029454	0.292378625	0.351181016	0.293430076	0.173228915	0.221484804	0.272965218	0.178567045	0.153459283	0.081862089	0.089350553	0.172649603
AKAP6	0.002909411	0.012064692	0.003079361	0.002940534	0	0.002987859	0.002991899	0	0	0	0	0
AKAP7	2.163671154	2.455848969	2.090923789	1.771926186	2.741472851	3.434017689	2.471262265	1.933062054	3.871620302	5.00768212	4.265141258	3.079405435
AKAP8	19.32932526	16.68353135	18.96861944	18.03504046	19.6509133	18.29115487	19.82036188	18.48003911	12.54389029	10.25740308	12.28477715	13.07986638
AKAP8L	29.62464202	29.89941443	28.0378016	28.23581702	30.27258487	31.9554318	31.05339581	32.39386265	26.71279775	30.88187501	25.36940432	29.85771587
AKAP9	4.122805541	3.352041748	3.164043406	3.617562581	3.511674914	3.590153759	3.12498882	2.272440433	9.80339967	8.188279223	7.142090092	8.179361659
AKIP1	8.88166258	8.601595108	8.369343541	9.288474782	8.087236858	8.38745041	8.365395023	10.23189072	9.206050673	9.418622218	8.814936223	10.40896852
AKIRIN1	36.5770786	33.41726733	38.04512647	34.95334387	28.27890232	27.22479672	39.23803357	32.83564862	34.60651215	28.13854976	30.70311815	41.96836035
AKIRIN2	24.92730692	26.79485763	25.46956679	28.14967319	23.96372778	24.45532716	24.23061699	29.3243017	30.37027618	28.03623999	30.15088622	28.98493729
AKNA	19.85670631	19.90377462	18.20003789	12.00033466	17.69373368	16.43747458	19.81602666	13.29534306	8.721986216	7.65554937	8.346485327	7.697303186
AKNAD1	0	0.01821225	0	0	0	0	0	0	0	0.057790768	0	0
AKR1A1	45.61983063	42.51416716	42.120659	35.37795071	34.93463297	38.53280003	34.99777811	38.34700304	41.29446425	60.06871481	45.57867903	37.248422
AKR1B1	2.358278268	2.02167487	2.712037205	1.558445624	1.112844305	1.024635557	1.352481979	1.442242032	46.21981268	45.27895731	53.05217882	27.96405188
AKR1B10	0	0	0	0	0	0	0	0	0	0	0	0
AKR1B15	0	0	0	0	0	0	0	0	0	0	0	0
AKR1C1	0.222879838	0.33008356	0.235899094	0.305715445	0.398959517	0.408731202	0.212827569	0.249244631	0.017324928	0.052370746	0.051445299	0.016567722
AKR1C2	0.02589488	0.040267653	0.013703749	0.026171881	0	0.026593098	0	0.025338272	0.070450261	0.042592186	0.097625584	0.040422689
AKR1C3	0.027176546	0.056347598	0.057528062	0.054934514	0	0.11163729	0.027947054	0	0.295748773	0.268201702	0.087820763	0.282822721
AKR1C4	0	0	0	0	0	0	0	0	0	0	0	0
AKR1D1	0	0	0	0	0	0	0	0	0	0	0	0
AKR1E2	0.464961102	0.421770137	0.338333354	0.323080209	0.384356138	0.497393866	0.348646422	0.388617473	0.137040026	0.148705894	0.135643951	0.060484859
AKR7A2	34.45537746	38.488701	36.44630067	45.12370041	32.52355354	35.4899165	32.22074495	46.40051874	30.00579648	31.81144581	31.18290968	31.77333474
AKR7A3	3.467727256	3.520597903	3.94872617	2.731344023	3.920589949	4.715559131	4.008725495	3.95481995	0.780776761	0.57693166	0.850104987	0.846205582
AKR7L	1.731382991	1.251718226	1.639622832	1.703853716	1.807873284	1.801462875	1.757043857	1.872500415	0.545419837	0.524594353	0.564402703	0.49787336
AKT1	34.77995624	36.38244194	36.16300026	44.59282522	39.77520371	39.76575935	37.21370939	46.96466183	62.2530686	67.52178019	69.37921377	69.90813595
AKT1S1	16.17862212	9.044070229	13.28267524	5.745675619	5.973347363	5.030354036	9.890471867	6.734676879	11.30310543	9.095690929	10.57175259	7.441714131
AKT2	16.76633692	17.65551472	16.9400065	18.43360269	19.1493649	20.70732722	17.112302	20.82600918	15.2814252	15.94973851	15.1664086	13.24515051
AKT3	11.87189653	11.82206131	11.4200273	13.27809126	14.80825776	12.97837654	12.51435115	12.00682442	11.8659152	8.819776526	9.175167955	11.49218846
AKTIP	1.697180704	2.315078092	2.212309234	2.455638423	1.697542721	2.036491003	1.763670689	2.290017799	2.488531509	3.25190111	2.771077466	3.346547739
ALAD	15.96421766	18.25392774	16.15553	18.50395776	15.63977821	15.98370236	15.8730362	17.01955213	38.96107143	33.5220071	31.94436523	48.41339063
ALAS1	62.43918999	68.42218094	72.79226778	50.52101736	33.12352075	37.04223187	53.56030866	48.20639601	28.93438849	27.09457872	47.4659993	35.24088234
ALAS2	0	0	0	0.050970167	0	0.025895248	0.051860513	0	0.082321823	0.1382483	0.027161061	0.07872385
ALB	0	0	0	0.069873878	0.024062869	0	0	0.022549434	1.128531721	0.631738483	0.645397985	0.911331116
ALCAM	6.060433678	5.215268476	5.574672241	10.43342136	6.310548678	6.87396763	5.103855484	8.085837934	34.7859011	32.28349141	30.99473691	37.1129957
ALDH16A1	16.90770002	16.29747268	17.6620034	20.36230075	16.55677804	18.39112646	19.44491402	24.22731653	9.910426739	8.144946727	9.389321047	11.06565067
ALDH18A1	43.11800441	40.35003713	44.83884627	42.46182671	42.45166481	45.49343379	42.84179703	41.56421916	23.94011397	29.72949798	29.48833814	27.70526889
ALDH1A1	0.02271404	0	0	0.022957015	0	0.023326492	0.046716056	0.06667741	0	0.049813739	0	0.070914604
ALDH1A2	0.011624446	0.024102018	0	0.011748794	0	0.011937883	0	0.022749159	0.012650304	0	0	0.04838963
ALDH1A3	4.220650145	0.57206555	4.009425291	0.105514462	0.809787563	0.260373558	1.794405389	0.087560302	0.275911965	0.57238168	0.433748891	0.279373664
ALDH1B1	15.25229785	12.76985918	15.09591794	12.41513043	13.66368882	14.43710141	13.63203089	13.13819106	13.57199934	15.56488252	16.99891436	14.70104256
ALDH1L1	0	0	0.014595792	0	0	0	0	0	0	0	0	0
ALDH1L2	13.3212753	13.02852842	14.90430973	15.74307512	21.30279207	22.26441721	13.87424165	12.94512785	6.749038887	9.45306436	14.68108941	4.536947822
ALDH2	3.186779818	3.249560487	3.124108877	1.689636306	3.873070585	4.211598056	6.607986067	4.60074131	36.41413946	31.73633339	32.329125	40.74871328
ALDH3A1	0.075984318	0.052515059	0.093826658	0.025599044	0.185129485	0.078033126	0.078138623	0.07435104	0.124034906	0.083319898	0.027282515	0.065896563
ALDH3A2	13.54424784	15.11832448	14.08062641	19.47724074	17.91309756	19.47843336	14.61880348	19.30318093	7.045742726	6.321401066	7.001260649	9.638614328
ALDH3B1	17.07124092	18.87502945	13.73735428	12.55322652	10.84207572	12.97741422	14.55212958	13.51160573	17.30264078	19.74719262	16.40791946	24.68829175
ALDH3B2	0	0	0	0	0	0	0	0	0	0	0	0

ALDH4A1	5.379453445	5.218564861	4.914384089	6.150793991	3.640138867	4.367134816	4.342134089	6.851791287	5.281853402	5.701064933	5.001442734	6.474041921
ALDH5A1	8.909281257	7.272436755	7.799336395	8.97302685	6.944557897	7.770667965	6.85000144	7.098470221	7.486848727	7.669427381	7.612378488	8.210283427
ALDH6A1	1.653118468	1.851802803	1.232998358	1.682015524	1.494452995	1.629328985	1.266433739	1.682722452	6.556110018	7.518495536	6.286155902	6.500490731
ALDH7A1	0.040526877	0.063021038	0.16085327	0	0.010579326	0	0	0	0	0.055549213	0	0
ALDH8A1	0.042071588	0.02180771	0	0.021260816	0.043930257	0.064808982	0.0216322	0.020583631	0.251814241	0.691998135	0.453179857	0.350266764
ALDH9A1	28.72709813	29.12321726	27.34947108	35.01510855	34.78535821	33.30767366	32.78419193	32.9581686	36.63233433	36.55217711	38.44263679	42.85151658
ALDOA	322.1416935	410.4901413	328.2399144	314.2856958	390.4896878	377.3298644	307.0956158	344.3959618	310.8828499	405.2156881	400.1589937	183.8545171
ALDOB	0	0	0	0	0	0	0	0	0	0	0	0
ALDOC	15.42726615	40.04766825	14.26278634	9.925214995	47.09728198	44.06202367	17.10707263	14.36815661	64.62647023	158.601856	111.4853494	20.18152601
ALG1	7.774222614	7.359844324	7.981081992	7.831149458	6.369463424	6.584338286	7.647624504	8.940584067	5.296513844	3.856774107	5.913603629	5.213597432
ALG10	1.996059462	2.352473699	1.856914189	2.585472189	3.06054604	3.128764737	2.274121991	1.98399359	4.081471646	5.402782423	4.022917933	4.444270281
ALG10B	2.39094699	2.440211826	2.328611255	2.384374387	2.911749367	2.771189499	2.426024602	1.986805079	3.115414661	4.069267196	2.758177622	3.03442309
ALG11	3.62926083	3.10999197	3.441591073	3.519663933	3.001010297	2.714548842	3.473279543	2.853316584	4.337648671	3.427544553	3.502559091	4.388217804
ALG12	5.383002855	5.325908918	5.870750754	6.019811359	4.744568009	5.654265255	5.11466177	5.968266505	5.791231204	4.892717234	5.974751499	6.730944098
ALG13	3.656912404	3.683373127	3.325824547	2.760770008	3.560116264	3.597977254	3.551953943	2.856835361	4.47507004	4.444048427	4.120323425	3.342218627
ALG14	1.375384434	1.367257885	1.754847603	1.542436504	1.731245896	1.664005405	1.74375529	1.806718129	1.45575499	1.528824826	1.927997846	1.156839997
ALG1L	0.018069026	0.009366038	0.047811267	0.018262313	0.02830094	0.04639058	0.009290659	0.026520951	0.147477142	0.079253693	0.077853195	0.056412591
ALG1L2	0.315830144	0.286492188	0.250709235	0.47881293	0.494674676	0.486519078	0.446578758	0.386301749	0.214813856	0.649351148	0.552826227	0.328680257
ALG2	10.07740892	8.219780476	9.619586383	9.012948436	7.365831199	7.107705625	9.287704856	8.186321012	10.59428538	7.90199925	8.294872785	12.05064643
ALG3	24.72134469	24.27036855	29.96568791	26.04014836	26.72546103	27.48074202	26.47006903	28.86652878	13.93992774	14.92400992	16.54182075	15.19897976
ALG5	32.13503429	34.78466238	39.54793628	42.28962039	43.59799367	39.55629201	40.15674045	44.06538318	24.16613409	20.17035861	25.16129969	23.47894523
ALG6	6.911759078	6.503458863	6.994497615	9.663813702	9.083893579	7.639090386	7.485267444	9.449724603	6.045171598	4.795965165	5.003516987	6.910573099
ALG8	18.80247022	17.85886603	21.79396103	21.26337419	23.59089567	22.69899327	21.54721219	23.75320075	19.39582951	18.63296032	21.65249238	16.19912744
ALG9	3.411114366	3.846906165	3.447741187	4.107617674	4.380067398	4.094828383	3.499925667	3.818915989	3.001486905	3.917337817	3.335032349	2.934265544
ALK	0	0.006065469	0	0	0	0	0.006016654	0	0	0	0	0.006088822
ALKAL1	0	0	0	0	0	0	0	0.044964072	0.750105769	0.906984405	0.940454628	1.052071541
ALKAL2	0	0	0	0.031600911	0	0	0	0	0	0	0	0
ALKBH1	4.870962182	5.610782066	5.706294142	5.343842485	6.107733236	6.220809843	6.100782532	5.356951702	6.27490801	6.14010704	6.076449397	7.842012139
ALKBH2	14.13504882	14.02929031	14.23819533	9.172423958	18.32360523	18.0627469	16.60055036	13.24181854	3.233814196	5.107841361	4.065970257	3.635749402
ALKBH3	14.33549805	13.43938687	13.46684631	11.57721244	10.31344678	11.83397965	12.27319211	10.57085559	17.95189398	15.83227635	17.65818633	21.59294697
ALKBH4	4.820281265	5.523180366	5.262963289	5.435952731	5.854447193	6.2268975	5.635264621	6.528863814	4.058493064	3.672125136	4.427060817	4.013122406
ALKBH5	22.21955658	24.77308841	23.45114524	24.72197093	23.65932173	25.3612355	21.70543714	28.10507211	11.98790178	11.76994654	10.56611138	12.58915256
ALKBH6	10.0318327	12.53040322	9.644767487	10.38510757	9.797396545	9.385947234	10.53870785	10.84809076	7.856811375	9.013733569	9.058336602	8.160643549
ALKBH7	17.68701691	22.44585216	17.45847752	17.79215974	16.38419785	17.9646313	17.53278117	20.42640651	5.581286737	6.778967274	5.912631285	4.7891897
ALKBH8	3.350987268	2.954041606	2.834972168	3.121876867	4.367836284	3.58180477	3.23501511	3.167428859	4.291708736	3.512016351	3.744613348	2.787490198
ALLC	0.016076953	0.008333451	0.017016069	0	0	0	0	0	0	0	0	0
ALMS1	3.288940277	3.029822316	2.771628583	4.190918988	3.995022665	4.091625443	2.680063063	3.226392204	3.488577016	3.43298178	3.367832839	3.370765439
ALOX12	0.011616917	0	0	0.02348237	0	0	0.01194628	0.011367213	0	0	0	0
ALOX12B	0	0.022295812	0.022762902	0	0	0.022086514	0	0	0.023404579	0.07074865	0	0.089526617
ALOX15	0.119932156	0.186499664	0.10578154	0.040405029	0	0.020527659	0.020555412	0.039118075	0.304537741	0.372613062	0.538277337	0.062405905
ALOX15B	0.019812062	0.020539073	0	0	0	0	0.020373773	0.019386203	0	0	0	0
ALOX5	0.123747553	0.36348412	0.087317411	0.20845216	0.02153576	0.148264933	0.212093399	0.10090634	4.174716678	2.646037485	2.732575527	6.632295799
ALOX5AP	17.92358841	14.3582779	17.1638519	11.92003695	8.588032667	5.777048661	16.95562102	10.66724792	25.33161805	15.39984392	22.73335746	29.63460112
ALOXE3	0	0	0	0.014533717	0	0	0	0	0	0.015768139	0.015489499	0.014964965
ALPI	0	0	0	0	0	0.016878576	0	0	0	0	0	0
ALPK1	3.498733005	3.308855536	2.593855769	2.482266204	3.078501558	2.962517206	2.765125446	2.382485445	6.998650633	7.034556533	5.513374723	7.144459157
ALPK2	0.014714127	0	0.007786817	0	0	0	0.003782825	0	0.008006324	0	0.019811903	0
ALPK3	0.054218665	0.056208238	0.052168894	0.109597299	0.051467252	0.030371233	0.055755871	0.057876261	0.123370881	0.097286685	0.063711683	0.153885405
ALPL	0	0	0	0.038272462	0	0.03888843	0.019470502	0	0.041209189	0.103807837	0	0.019704046
ALPP	0.018111599	0	0	0.036610682	0.018911746	0	0	0	0	0	0	0
ALPPL2	0	0	0	0	0	0	0	0	0	0	0	0
ALS2	2.878286219	2.834710651	2.498062619	2.923620992	2.569655674	2.630746623	2.772431519	2.426813225	4.964687748	4.334457026	3.973660525	5.541479692
ALS2CL	0.007053292	0.007312115	0	0	0.007364897	0	0	0	1.466067256	2.034100204	1.0940471	2.554413002
ALS2CR12	0.18296914	0.172439325	0.193657062	0.268983832	0.295262929	0.119574408	0.102630914	0.227864296	0.669754408	0.401266224	0.662931387	0.65779232
ALX1	0	0	0	0	0	0	0	0	3.74258712	2.856893529	3.592203713	2.169098728
ALX3	0	0	0	0.03679646	0	0	0	0	0	0	0	0
ALX4	0	0	0	0.00994972	0	0	0	0	8.666964867	8.538686445	8.440818901	9.968336731
ALYREF	66.51391479	75.00599772	92.51582159	118.1402523	97.77629771	94.1996878	89.18192963	118.1691062	99.71451613	101.9264261	119.3577836	91.68096838
AMACR	3.923613813	4.648676808	4.15280697	4.299682133	3.706767965	3.73421368	3.473227685	4.134604949	7.601312215	7.86409797	7.353581527	9.57246134
AMBN	0	0.027850285	0	0	0	0	0	0	0	0	0	0
AMBP	0	0	0	0	0	0	0	0	0	0	0	0
AMBRA1	6.700271438	6.102680375	6.289626752	6.691326703	5.788589542	6.135500069	6.537522799	6.829426665	8.871411017	7.487065586	7.947609826	11.00719535
AMD1	55.4897157	46.5664083	53.20733727	51.77035253	54.38584018	51.62630849	54.84588206	48.46280858	68.55798153	57.3250567	63.71142779	50.69145968
AMDHD1	0.403067872	0.679020208	0.293296158	0.40737954	0.289351491	0.310452014	0.284965752	0.36975371	0.219319315	0.138118854	0.352763178	0.419467407
AMDHD2	4.539058168	5.482125545	4.677358153	4.678456876	4.927294423	5.046055237	4.328836908	5.672796008	2.983341047	1.806926831	3.275675346	4.474299545
AMELX	0	0	0	0	0	0	0	0	0	0	0	0
AMELY	0	0	0	0	0	0	0	0	0	0	0	0
AMER1	1.771770451	1.638571856	1.571715983	1.384911909	2.056344993	2.140254643	1.559844837	1.378218306	2.732672425	3.256666877	3.05495181	2.175487246
AMER2	0	0	0	0	0	0	0	0	0	0	0	0
AMER3	0	0	0	0	0	0	0	0	0	0	0	0
AMFR	8.133277019	7.758228209	7.881090703	9.584012762	7.344594295	6.902745927	7.644398627	9.19317439	15.17320986	13.16535959	13.24223738	17.63048684
AMH	11.29623269	11.62043766	12.97035818	15.49669132	17.73841187	12.52530631	12.66168979	19.74839999	0.979657835	0.573167155	0.844558013	1.359930085
AMHR2	0	0	0	0	0	0	0	0	0	0	0.026442383	0
AMIGO1	0.010434276	0	0	0.021091785	0	0	0	0.010209992	0.965183605	0.594963635	0.910393251	1.270481125
AMIGO2	0.100245508	0.15011252	0.106101228	0.090060308	0.058152348	0.080071043	0.034362555	0.065393825	1.030315018	2.186251916	1.883665385	1.3446227
AMIGO3	2.335610145	2.440086077	2.816978079	3.147459286	2.835807468	2.194055892	2.662492936	3.667280001	3.566300312	2.561091619	3.861513232	3.919169232
AMMECR1	3.324951704	3.588617952	3.98197032	3.710381403	4.242307802	3.938488752	3.775194287	3.200000215	8.495755257	8.150934394	8.222770893	7.356550787
AMMECR1L	5.291016901	5.02317494	5.677268668	5.118315069	5.778585072	5.025947444	5.724327712	4.89979065	6.45454663	5.419766353	6.179323323	6.467573469
AMN	2.167421687	2.556485643	2.785606648	2.145900611	3.313935719	3.463715549	3.195475169	2.921555826	0.012034167	0	0	0
AMN1	1.184073466	1.218430694	1.197540145	0.700314314	1.126483493	1.152948443	1.190585519	0.875403209	2.567591059	3.597006684	2.418613877	2.482754765
AMOT	0.040785418	0.035235044	0.079141055	0.006870284	0.021293633	0.013961713	0.006990294	0.006651456	1.538670726	1.982713943	1.471741086	1.492643716
AMOTL1	7.224727999	6.710456177	7.268646682	5.976332935	9.93791474	9.35791951	7.001883609	5.556417823	10.98982747	9.945148709	10.24036045	9.039061257
AMOTL2	0	0	0.010132144	0	0.029987617	0	0.009844364	0	0.802167905	0.661319264	0.680567949	0.697371094
AMPD1	0	0	0.023661313	0	0	0	0	0	0.024328316	0	0.024080475	0
AMPD2	10.84387167	12.42915111	10.56534705	9.685466464	10.45616463	11.90241863	9.768207725	11.3099436	13.08155557	16.13085805	14.92901526	16.62142672
AMPD3	4.050693219	3.716312755	3.522437437	3.113764733	1.707745072	2.324083608	2.483677795	2.465634565	2.783560903	2.773487114	2.253326525	2.275975744
AMPH	0	0	0	0	0	0	0	0	0	0	0	0.04008511
AMT	3.518881364	3.825341679	2.353634468	2.29692129	3.087467136	3.463189501	2.487820899	2.893280803	4.308100656	4.742858429	3.790411138	4.043517219
AMTN	0	0	0	0	0	0	0	0	0	0	0	0
AMY1A	0	0	0	0	0	0	0	0	0	0	0	0.027681117
AMY1B	0	0.110957975	0.084961882	0	0	0	0	0	0.029118974	0.058681625	0	0
AMY1C	0	0.052750943	0	0.051428055	0	0.026127876	0	0.024895002	0	0	0.027405061	0
AMY2A	0	0	0	0	0.033464442	0	0	0.031359695	0	0	0	0
AMY2B	2.856082847	5.001163453	2.311642774	3.274753685	2.731650781	4.190132898	2.616203298	3.029539587	2.66411339	4.763511042	2.48185707	3.097173709
AMZ1	0.037890245	0.039280641	0.045832638	0.005470795	0.028260134	0.022235372	0.038964508	0.010593085	0.029452901	0.005935457	0.017491712	0
AMZ2	12.13384565	13.68606233	12.31180089	10.3832177	10.10213637	11.04876878	14.47432233	10.32162034	9.031986131	11.53122169	9.637048143	10.5061073
ANAPC1	12.5252349	11.08637778	12.9338401	12.33910075	15.0268418	14.61350526	13.23566997	11.15155663	13.15430025	14.41641552	15.23479711	11.80627469
ANAPC10	3.388054203	3.299128403	3.611577317	3.81564484	3.81569894	3.814922595	3.795193659	3.386268833	3.594873888	4.192800156	4.209946094	3.563680767
ANAPC11	15.27884617	15.73575659	18.92541276	25.11900224	17.76519982	17.26675209	17.66745906	24.09042846	14.70309616	16.60755484	16.8890271	13.33141795
ANAPC13	16.32982873	15.26340789	17.62382483	16.4159358	14.79402678	16.80013941	18.86562727	17.80823797	26.29081357	27.67631891	24.70137751	24.10810357
ANAPC15	8.75732231	8.761239144	9.441716779	12.4615485	11.27572704	10.85922517	8.291878343	12.90356286	6.931022839	6.916673624	7.542057798	4.100071121
ANAPC16	32.88278292	31.33851611	30.2077935	38.43076358	33.23349171	31.81850444	31.55143363	36.36577986	21.96960107	19.95468754	20.80385049	21.10353571
ANAPC2	15.94357359	14.75180107	15.10303386	17.16154209	14.23398899	15.20685952	15.04296786	20.39813445	43.37648671	37.87206694	40.31558499	52.16193725
ANAPC4	16.14954853	16.20544317	15.25457798	19.03987281	17.4210465	17.32249012	16.53872336	15.96583208	22.00932629	20.47389526	20.07612082	25.27076316
ANAPC5	130.7225679	121.0425598	133.0426978	131.0260541	136.8884816	132.8718713	133.6996498	135.0821283	43.76865334	42.13672561	44.82909218	43.16752597
ANAPC7	24.97490167	22.04688917	26.62831467	29.60205379	25.82989658	25.92589326	27.25065229	29.44205335	20.91954612	18.75525823	20.67149367	24.17955266
ANG	0.162566649	0.379197202	0.086031392	0.410764113	0.127311477	0.125212519	0.041793933	0.159072291	0.221141454	0.356521899	0.131333165	0.126885722
ANGEL1	10.10188853	10.25059352	8.798884752	9.700725298	7.509986427	9.675754449	8.19924187	8.76316293	7.443149247	9.488765257	7.665815208	12.34807179
ANGEL2	9.941533588	9.175157831	9.335294419	10.02745692	11.57283479	9.638934662	10.7324875	8.847985514	6.563890249	6.181832686	6.627615007	7.727974211
ANGPT1	0.047841352	0.012399227	0.050635947	0	0.199819688	0.012282831	0.098395497	0.011703251	0	0	0	0
ANGPT2	0	0	0	0	0	0.010182506	0.010196272	0	0.140272221	0.032617122	0.04272099	0.061911442
ANGPT4	0.011383448	0	0	0	0.011886355	0	0.011706192	0	0.037164115	0.024964829	0.024523674	0.011846604
ANGPTL1	0	0	0.016061134	0	0	0	0	0	0	0	0	0
ANGPTL2	0.10187587	0.125416911	0.188696944	0.045047471	0.186159077	0.189628837	0.196432971	0.143298704	0.048504059	0.04189164	0.048009931	0
ANGPTL3	0	0	0	0	0	0	0	0	0	0	0	0
ANGPTL4	0.027467872	0	0.029072374	0	0	0	0.02824664	0	0	0.120478559	0.059174787	0.057170902
ANGPTL5	0	0	0	0	0	0	0	0	0	0	0	0
ANGPTL6	0.167735539	0.121723458	0.106520165	0.067811931	0.052543764	0.189484116	0.12074382	0.049239023	0.073015283	0.128750193	0.108407176	0.017456016
ANGPTL7	0	0	0	0	0	0	0	0	0	0	0	0
ANGPTL8	0	0	0	0	0	0	0	0	0	0	0	0
ANHX	0	0	0	0	0	0	0	0	0	0	0	0
ANK1	0.016820744	0.087189937	0.089016538	0.011333785	0.011709242	0.034548583	0.028829409	0.005486395	0.433222492	0.307410516	0.452967362	0.472638408
ANK2	0.492866126	0.557685439	0.524837136	0.416127791	0.483259815	0.472206097	0.441939626	0.358763321	0.484033624	0.583288146	0.796346222	0.322138318
ANK3	0.062610406	0.056441667	0.072030128	0.013756556	0.142122726	0.083867747	0.114774214	0.023973104	0.085910328	0.020894957	0.043983688	0.008498846
ANKAR	0.738293273	0.640787614	0.699643293	0.555304848	0.708161629	0.625956056	0.768053543	0.495616269	0.644626633	0.517747382	0.480856508	0.61645246
ANKDD1A	0.43255274	0.968598898	0.38456862	0.419692619	0.632327316	0.621902267	0.320267853	0.440185253	1.468663682	1.517797411	1.136869429	2.052692755
ANKDD1B	0.237207288	0.075665136	0.096562871	0.018441902	0.019052831	0.018738711	0.037528089	0.053563508	0.238283854	0.140057857	0.078618791	0.246858489
ANKEF1	1.316441856	1.378965292	1.523965861	1.164208502	1.532106894	1.351937824	1.226850052	0.925854244	0.776001368	0.887170766	0.605614131	0.713543563
ANKFN1	0	0	0	0.002424014	0.002504314	0	0	0	0.334081695	0.215651573	0.328094864	0.197179221
ANKFY1	12.15687112	10.11141654	10.62565165	13.20984886	11.34850488	11.40879639	10.84235148	11.88459642	8.742586218	8.78461794	8.007827252	11.32804377
ANKH	4.568070506	4.669184861	4.868861833	6.179690673	5.419711469	5.495078389	4.723975224	5.94519174	1.284692894	1.364832315	1.720813665	1.295312552
ANKHD1	0.547000264	0.494540086	0.525096565	0.6492792	0.518034314	0.489897682	0.758732798	0.715731295	0.712943319	0.599808061	0.48643983	0.589113711
ANKHD1-EIF	0.006445031	0.006681533	0.020464528	0.006513974	0.013459528	0.019856435	0.0331388	0.025225981	0.02805522	0.021201716	0.013884706	0.020121775
ANKIB1	5.710946579	5.684382419	5.309556217	6.260526842	5.841431574	5.927691147	5.770030927	4.424889909	6.142389962	6.676522226	6.037926801	7.793363167
ANKK1	0	0	0	0	0	0	0	0	0.008783299	0	0	0.01679883
ANKLE1	25.14903031	23.52363367	27.82155755	23.15341236	24.12994273	27.13223261	26.32633375	26.62098023	2.45660805	3.208752489	3.368190983	2.210024908
ANKLE2	25.90314915	24.07723986	25.80619754	27.81514994	27.95357551	26.82162014	25.6681149	26.85582368	20.38988485	18.16061373	20.02061633	19.36493874
ANKMY1	5.242708859	4.354997213	5.04707532	4.603535433	4.783932464	4.931397506	4.519118836	4.842474853	2.740035114	1.926045731	2.100635158	2.78013647

ANKMY2	7.721461398	9.106763065	8.214955782	9.202708365	11.07820219	11.43106765	9.63157786	9.910446815	14.90692865	14.31675278	15.27354621	14.79806906
ANKRA2	7.301161582	8.300758045	6.748814429	6.100190779	6.530984735	7.048145326	7.082701271	6.334544973	6.939060765	8.166141362	6.344067067	7.395603103
ANKRD1	0	0	0	0	0	0	0	0	0	0	0	0
ANKRD10	30.51837251	26.20029887	25.631962	23.16836795	29.97473857	28.81601364	22.98665711	19.81855431	32.31377101	34.67089054	26.55652353	38.52791582
ANKRD11	11.43188541	10.56898888	11.15067183	12.59883216	11.29544066	10.27367933	10.91933653	11.3811236	14.39223999	9.977442611	13.8342599	15.70284635
ANKRD12	4.102210122	3.888991257	3.566999183	3.817732009	3.672812791	3.465456225	3.256320055	2.314321274	7.429384409	5.79023767	5.249309018	6.933368734
ANKRD13A	32.22804595	35.73500388	31.66818882	38.20478584	36.78701947	36.24784465	35.77100399	34.74272171	24.02829067	22.21290409	22.0745962	25.67980692
ANKRD13B	0.897230172	1.181547418	1.077970624	0.894573526	1.266038725	1.867748712	0.885262938	1.281324078	1.636147786	2.220308814	2.050470364	2.561463827
ANKRD13C	5.159696685	4.935400049	4.837243306	5.581491014	5.917888295	5.13119526	5.744264141	4.862454453	8.022895685	7.368089213	8.312337346	7.606186799
ANKRD13D	46.90751752	50.24672726	45.36679883	46.67615981	57.1278357	47.56282116	42.38294108	51.92163765	59.73037904	59.39002385	54.64690035	77.29586568
ANKRD16	3.56962591	3.150808997	3.778141292	2.886248523	3.258749125	3.309762113	3.482046197	3.5527753	2.175401513	2.885354166	2.043380758	2.50487872
ANKRD17	20.47684016	17.41857752	19.47695591	16.98982021	19.91910754	18.33273703	19.1822564	14.43407059	19.58310391	18.31471979	17.47962693	18.28704957
ANKRD18A	3.171261693	2.759498564	2.60163001	3.269546231	2.972248401	3.067118943	2.226503796	1.80079246	3.71446459	3.344745007	2.846639884	2.92917317
ANKRD18B	3.979294236	5.002715108	4.007439082	6.93320858	7.317927415	7.380259919	3.786733343	5.869697308	8.58014197	6.138148214	7.021299091	4.604765964
ANKRD2	0	0	0	0	0	0	0	0	0	0.038793146	0.076215258	0
ANKRD20A1	0	0	0.016152235	0.046272123	0.015934997	0.03134456	0.015693468	0	0.348758763	0.184074906	0.26301397	0.301752433
ANKRD20A2	0	0	0.02576466	0	0.02541814	0	0	0.011909733	0.066227389	0.053385546	0.065552707	0.025333137
ANKRD20A3	0.018181977	0.037698342	0	0	0	0.028008342	0	0.008895578	0.039573075	0.109655029	0.03916993	0.085147844
ANKRD20A4	0.198898007	0.12689024	0.194322825	0.293806711	0.335491263	0.235685777	0.094401765	0.164680754	0.033300118	0.100661429	0.065921756	0.143301134
ANKRD22	0.13499639	0.097965091	0.07144102	0	0.014096036	0.055454549	0.111059042	0.052837861	0.014690983	0.014802904	0	0.028097792
ANKRD23	1.229575558	0.993830208	1.191111615	1.200602095	0.935721279	1.498153408	1.264436446	0.876869062	1.179329732	1.211166476	0.853038241	1.843495895
ANKRD24	0.211610633	0.334286872	0.181310352	0.295350167	0.168349958	0.196619609	0.155435865	0.27608277	0.789549533	0.883960664	0.629546594	0.954288351
ANKRD26	1.108948875	1.026649226	1.02897296	1.059191794	1.703360199	1.661739659	1.431841526	1.048028032	0.980872324	0.997379184	0.796937927	0.939716945
ANKRD27	30.6730073	29.1279816	29.58602511	25.98765794	29.27568666	31.46314686	27.88320751	24.13991971	15.07311182	16.17332105	16.64898635	13.99037286
ANKRD28	9.054124521	8.636368403	8.492449769	6.093265987	8.824612293	8.217587035	7.929983826	5.545236085	8.982352326	7.65049588	8.843617038	5.777845236
ANKRD29	0	0.009190135	0	0	0.009256474	0.018207729	0.009116173	0	0.164001708	0.252737024	0.257819769	0.239863488
ANKRD30A	0	0	0	0	0	0	0	0	0	0	0	0
ANKRD30B	0	0	0	0	0	0	0	0	0	0	0	0
ANKRD30BL	0	0	0	0	0	0	0	0	0	0	0	0
ANKRD31	0	0.016416752	0	0	0	0.008131321	0	0	0.129248654	0.069457769	0.042643984	0.057679855
ANKRD33	0	0.019382947	0	0	0	0	0	0	0	0	0	0
ANKRD33B	11.99703555	12.12337797	11.19222828	10.31811193	5.219603865	7.392311052	9.288130419	7.775796082	5.415037892	6.31451421	5.415256314	6.801437549
ANKRD34A	0.372229945	0.5711158	0.330937578	0.421357145	0.528597441	0.351685265	0.382783397	0.378798045	0.518501446	0.897963638	0.497181188	0.526829707
ANKRD34B	0	0	0.01336606	0	0	0	0	0	0.054971375	0.152322957	0.122425565	0.091995375
ANKRD34C	0	0	0	0	0	0	0	0	0	0	0	0
ANKRD35	0	0	0	0	0.033414688	0.049295681	0.032908218	0.062626141	7.086889868	5.824993245	7.255984906	9.191612298
ANKRD36	2.092471667	1.798198765	1.661025652	2.393125511	2.765641644	2.346816445	1.194813797	0.986030074	2.648664513	2.994900704	2.117512096	2.859542624
ANKRD36B	2.09821425	1.587014703	1.563609633	2.007052377	1.671128299	1.940410014	1.122886128	0.801342687	1.310614527	1.602327086	1.331856515	1.459436138
ANKRD36C	2.066521787	1.762258552	1.277063109	2.169477736	1.768018688	1.827867002	1.117397468	0.776226272	2.444763705	3.377108641	2.089551011	2.469723487
ANKRD37	2.513397747	2.447710931	2.136233127	1.231652792	2.703964831	2.933145359	1.566459105	1.788634508	1.864912645	3.632965724	2.174076641	0.871886606
ANKRD39	7.832280525	8.429600612	7.846827646	9.789330348	9.739045096	9.517078975	8.669176301	11.29112796	7.157120308	7.867249913	7.663825262	9.022044813
ANKRD40	9.786965425	7.956202258	9.763333938	10.52330905	10.71529888	9.25500363	9.820224915	9.931284042	13.67039451	13.78824636	15.20064191	16.142772
ANKRD42	3.916798466	3.611394011	3.883569543	3.449338659	3.73901634	3.168895833	4.164230511	2.958807343	2.136038953	2.743713102	1.685708437	2.898398477
ANKRD44	4.491098735	3.896066274	4.0245452	3.947511095	4.355645419	3.768562217	3.986115028	2.916873075	4.64654604	3.60315587	3.830908835	3.491292532
ANKRD45	0.045724028	0.094803776	0.048394942	0.023106572	0.015914686	0.031304608	0.007836732	0.007456866	0.190743508	0.342611436	0.172382917	0.166545373
ANKRD46	5.725476075	4.894921779	5.063052414	3.907916307	4.982016921	4.072627071	6.117199581	3.674306145	7.889585532	7.52842547	7.341993785	8.641009263
ANKRD49	9.798239196	8.957830448	9.572853686	11.12733064	12.31106443	10.20065493	11.26633983	10.9309481	15.08629434	12.83987136	13.62781427	16.05171037
ANKRD50	2.694319231	2.81025455	2.125710581	3.261113516	2.985245981	3.251609687	2.381342122	2.36256256	11.34618483	10.61428884	7.808220532	11.89534023
ANKRD52	13.50126618	13.87747759	12.90231848	18.4228451	12.16439987	11.97565826	12.55951021	17.07647333	17.86533703	21.04056392	17.67714279	28.01740162
ANKRD53	0.059001711	0.01223336	0.012489645	0	0	0	0	0.011546695	0.025683446	0.064697779	0.0635545	0.036841381
ANKRD54	4.960290561	4.90082047	5.946177829	5.06874757	5.107890785	5.108109615	5.326379796	5.966527401	8.007584426	6.911640489	7.58653315	9.711038989
ANKRD55	0.186968592	0.206751436	0.263853515	0.163772803	0.208243868	0.166408608	0.230723424	0.182949716	0.827438888	1.079765016	0.993552483	0.609670661
ANKRD6	0.073011616	0.096333749	0.028100546	0.033542105	0.041583914	0.034081941	0.027302414	0.032473744	1.422964924	1.564813184	1.379870335	2.258744285
ANKRD60	0	0.016373385	0	0	0	0	0	0	0	0	0	0
ANKRD61	0.075628336	0.039201772	0.12006911	0.114656013	0.276393247	0.077667545	0.116658822	0.222008131	0.12345381	0.207323873	0.32585638	0.118058114
ANKRD62	0	0	0	0.011454332	0	0	0	0	0	0	0	0
ANKRD63	0.727673777	0.714672022	0.689108386	0.86448548	0.759821476	0.825957032	0.498869841	0.612098099	1.819960065	1.357870537	1.485139738	2.76340948
ANKRD65	0.011831478	0	0	0	0	0	0.012166923	0	0	0	0	0
ANKRD66	0	0	0.03083529	0	0	0.014959518	0	0	0	0	0	0
ANKRD7	0	0	0	0	0	0	0	0	0.301846686	0.086898931	0.256090001	0.123708908
ANKRD9	1.234654784	1.693176594	1.759516821	1.955311391	2.020085443	2.246360104	1.479603405	2.454282838	3.173889272	3.091466768	3.319577247	3.7838461
ANKS1A	4.088019669	3.68127006	4.097749645	3.435023311	3.925459341	4.062421615	3.878325113	3.729549742	6.39840333	6.012064957	5.577723903	7.507673016
ANKS1B	0.005716212	0.002962985	0.012100235	0.011554718	0.005968747	0.014675853	0.011756556	0.027966712	0	0	0.003078648	0.002974393
ANKS3	2.880146208	2.530676539	2.732395187	2.360713911	2.548944194	2.398707895	2.81732578	3.24784708	2.19784581	1.925730233	2.213289598	2.832842467
ANKS4B	0	0	0	0.018714786	0	0	0	0	0.020150811	0	0.019945528	0
ANKS6	3.970653889	3.673796583	4.001175135	4.132690598	4.586458233	4.695712834	3.93513333	3.724256066	3.173631405	4.043790519	3.32966774	4.207143423
ANKUB1	0	0	0	0	0	0	0	0	0	0	0	0
ANKZF1	18.13202892	21.28170323	17.6658269	15.3281151	18.42563032	19.82561453	18.1463506	17.16836374	20.13339016	21.5179453	19.33260187	20.31725853
ANLN	13.63012617	13.63509498	15.40217661	28.23672565	23.36090265	21.23032894	14.74766757	23.08728732	36.14557981	36.68892971	34.62864789	36.01085258
ANO1	0	0	0.010364473	0	0.010225077	0	0	0.009581971	0.010656644	0	0	0
ANO10	0.486588442	0.627479093	0.314031657	0.539773398	0.235454163	0.438768529	0.292907814	0.522580894	6.418935545	6.532906026	5.599300001	8.275090918
ANO2	0	0	0	0	0	0	0	0	0	0	0	0
ANO3	0	0	0	0	0.004920466	0.009678686	0.014537657	0	0.010256285	0.00516721	0.025379501	0.053944118
ANO4	0	0	0	0	0	0	0	0	0	0	0	0
ANO5	2.061084045	2.516576919	1.753263377	2.291444896	1.538381634	2.532150393	1.56216462	1.755346596	0.274141365	0.443641912	0.19734443	0.556096314
ANO6	19.56711304	19.52444259	19.54123667	22.57059408	22.84620242	19.88158647	20.54870697	17.23911992	68.17278081	58.54924419	56.75616409	62.36241007
ANO7	0.043351106	0.078648309	0.057354261	0.01095371	0.090532606	0.06678001	0.066870294	0.031814458	0.436385803	0.368405963	0.490310484	0.225574618
ANO8	4.507109356	5.953986483	4.297373673	4.007530536	5.460812731	5.429371791	4.595781746	4.801009192	1.831562379	3.127992985	2.427447211	2.365035664
ANO9	0	0	0.022658755	0	0	0	0	0	0.011648748	0	0	0.011139626
ANOS1	0.144089868	0.340248288	0.110143731	0.105178101	0.025075929	0.106870866	0.0987834	0.023498778	1.07150634	1.983782892	1.302025774	3.057363266
ANP32A	113.7482205	115.3444689	126.3257476	137.8036842	139.4534933	144.859905	130.0724112	146.5403348	112.487872	110.1877392	121.6326469	101.0567887
ANP32B	180.7628538	206.1287119	215.3075771	310.6042251	301.0528283	287.30654	238.8617636	315.5268247	143.4441906	178.4362341	174.7095508	132.9151353
ANP32C	1.984466074	1.582528275	1.858034	2.391404577	1.912742081	1.959590729	1.883750396	2.838030179	1.162859005	0.920635628	1.31544292	1.429759119
ANP32D	0	0	0	0	0	0	0	0	0.44362316	0	0	0.282822721
ANP32E	59.52636025	63.33548131	67.28485314	87.12187203	100.0296459	92.73946583	78.89674422	80.06750432	72.72269539	94.35789684	87.54548583	67.86662664
ANPEP	0.014156685	0	0	0.042924364	0.029564219	0.0436152	0.072790276	0.055409554	124.6038367	111.923583	103.1904364	169.4994294
ANTXR1	0	0.014092831	0.023980118	0.004579804	0	0.004653512	0	0	2.96366421	2.335331027	2.269658291	4.937334491
ANTXR2	5.741564662	5.870475634	5.039277508	5.05127168	5.65398047	5.630201695	5.649401956	4.267368222	4.243174229	4.806848507	3.55053368	5.154244197
ANTXRL	0	0	0	0	0	0	0	0	0.346020822	0.19757226	0.331079261	0.408107004
ANXA1	30.85960339	37.48966308	29.45942864	15.01951204	8.44679044	13.38435383	26.09626236	13.95478501	26.11646097	36.69704128	28.65812519	27.59411218
ANXA10	0	0	0	0	0	0	0	0	0	0	0	0
ANXA11	26.52311973	29.98479965	25.67790755	26.42574506	24.82359844	26.18992507	24.87292626	29.20163261	32.91712656	28.39793508	31.43265864	39.28823786
ANXA13	0	0	0	0	0	0	0	0	0	0	0	0
ANXA2	27.9368286	22.52127663	26.72433016	13.16769654	13.97734641	10.97653621	27.67809195	12.21835898	45.33650096	49.31223918	51.29159496	43.05726194
ANXA2R	30.13505423	36.61659173	29.19617304	33.63632465	35.86013688	44.08614783	30.8146077	40.96614092	25.99505566	28.33701391	22.61696809	37.28836629
ANXA3	0	0	0	0	0.034726081	0.034153559	0	0	0.615259808	0.729349498	1.182160829	0.415319398
ANXA4	120.1410465	109.8859315	118.0670036	61.03541802	101.5914644	78.75755378	135.3449222	72.06301758	79.41606686	31.98541703	60.15320393	37.68267986
ANXA5	100.1210682	98.52581994	96.38846984	112.1667568	79.47734514	74.32768939	82.29000375	101.6476907	112.9337815	102.5782527	104.0912563	101.0253358
ANXA6	51.90938375	78.67083358	51.54089582	66.08278111	40.33184004	57.26509157	46.50641076	56.96320015	72.88294145	101.2261241	91.47343024	66.71329489
ANXA7	34.67433995	34.64703573	35.48927656	41.16956151	26.64078742	29.55581853	35.34077245	37.08195197	16.55141417	16.17103331	15.64835695	17.94528375
ANXA8	0.564576956	1.128781878	0.967471678	0.42116918	0.266687278	0.483166637	0.718818069	0.486674581	0.702172455	0.943362468	0.550223517	0.741429338
ANXA8L1	0	0.050392161	0.02572393	0	0.050755916	0.22463601	0.049986602	0	0.449634313	0.159903454	0.602131519	0.607034134
ANXA9	0.167481959	0.198431736	0.202588817	0.072545801	0.12491507	0.221140119	0.123021716	0.117058533	0.364524499	0.07870748	0.206177692	0.298793591
AOAH	0.250134152	0.413990791	0.167207644	0.323774042	0.169540984	0.297438449	0.22112406	0.283403463	0.821401094	0.620744111	0.529416964	0.954474789
AOC1	0.018352511	0	0.01942455	0.037097659	0	0	0.056618521	0.017958025	1.238271466	0.623852526	0.691903011	1.22234977
AOC2	0.261113957	0.062468221	0.361402494	0.284207675	0.083892197	0.226899979	0.227206736	0.353771075	0.349732032	0.26429731	0.216355743	0.229932022
AOC3	0.063342626	0.010944501	0.02234757	0.04268014	0.044094015	0.032525285	0.032569257	0.061981081	0.080421385	0.127339242	0.079602104	0.076906473
AOX1	0.062363795	0.075427632	0.03300335	0.042020605	0.032559474	0.074719569	0.02137731	0	0.045244935	0.056987033	0	0.043267451
AP1AR	5.252241612	4.674797163	5.15674565	5.875938107	6.160793621	5.766463007	5.418919956	5.156250807	3.337322404	3.978461587	3.517342107	4.162384036
AP1B1	41.58714026	39.66841205	43.48477079	45.03893809	36.02786825	37.37818476	42.54106252	46.26349925	43.33703467	33.88020442	42.27125683	47.24655778
AP1G1	20.03129638	17.67990193	20.07065899	21.56352086	19.58745622	17.7478845	20.211473	20.10035147	28.98847441	27.98616237	26.34930587	30.43560589
AP1G2	4.658555785	5.664824751	3.76417694	3.060920849	2.514383415	3.148229223	3.533450479	2.773114642	9.947848489	11.04935616	8.589505482	15.81655645
AP1M1	49.9161882	50.06805737	53.92768585	55.88639032	46.5050918	48.35001226	52.02570363	65.18304803	43.2513099	44.5679214	44.55609006	56.58441639
AP1M2	0	0	0	0	0	0	0	0	0	0.059093014	0	0
AP1S1	43.50121587	40.822863	46.60963251	34.29807951	42.19391775	40.693717	52.62141252	41.3557483	34.86572087	38.65803837	38.50789048	34.15718274
AP1S2	11.58301045	12.62030581	10.74732959	9.136400248	8.265397356	6.52482313	9.589861808	8.714903166	20.43163828	22.96877663	18.96144952	29.08492993
AP1S3	6.494257345	6.495602376	6.389754824	4.457355148	3.467800915	4.874298319	5.309521356	3.85489955	12.49593991	8.359690219	11.6444802	7.961862428
AP2A1	29.17902822	34.50760775	30.00110056	29.15659643	24.46410849	26.47818281	26.4257261	32.78661497	31.03360809	32.81605782	33.84731066	42.62755093
AP2A2	11.4593397	10.88017043	11.68962067	11.07195452	11.54410991	10.96227476	11.61127611	12.51864789	15.41135209	15.99597646	17.48875743	18.98524868
AP2B1	36.17663218	37.95497881	36.72664107	44.71558845	38.28893002	41.59280626	35.49787302	43.42782698	46.72695322	56.97098176	52.16376563	46.80237337
AP2M1	111.1708198	120.9255799	120.4063764	130.4434342	107.3544291	110.767738	119.7601326	146.173936	163.5562634	160.9662758	166.1185303	170.3311419
AP2S1	89.62588517	84.62429212	97.6526345	96.99046669	68.97873381	76.79674795	84.64312241	95.39961401	76.3803353	76.2625708	91.22668141	81.45294376
AP3B1	20.87716411	20.26047093	21.50695711	23.6089993	22.99833035	21.32738125	19.9498299	21.2049054	32.78858165	29.07636332	31.16328184	28.4035833
AP3B2	0.191537075	0.172944228	0.255041735	0.355948399	0.116128416	0.13324947	0.15249099	0.320427742	0.309298407	0.29132944	0.352735135	0.527260272
AP3D1	48.9720405	46.76357195	49.68268507	50.36369281	43.59150927	43.95889368	51.70645576	51.55663268	49.68165028	45.73154758	51.0139688	61.91588698
AP3M1	37.39016495	31.44025056	36.17725114	36.34230475	33.69314513	32.18793686	37.51789647	33.85401424	24.19289744	23.51227386	21.20216658	25.29621698
AP3M2	4.517917919	4.155286568	4.52434366	5.737079142	5.503766066	5.888897092	4.860441011	5.769718088	5.988196363	7.05003827	6.700847118	8.451071708
AP3S1	9.549617516	10.8143142	9.766120277	10.96217684	11.51774395	10.58437844	10.57838398	10.47777818	7.341268773	10.69526453	8.897008084	6.102619724
AP3S2	0.018136016	0.009400762	0	0	0	0.018625028	0.009325104	0	0.069077823	0.029830321	0.039070916	0.056621738
AP4B1	7.588707983	7.298993202	7.481652911	8.962403078	8.77506022	8.890165181	7.933927178	8.800698388	13.73343297	12.88264742	12.74582253	16.43843361
AP4E1	7.072530357	5.926118077	7.115239073	7.096601268	7.621000736	6.941252722	8.135319397	5.69336542	7.688746761	6.292200896	7.029230999	7.754861059
AP4M1	3.750595281	3.967576223	4.779820906	5.286372481	4.102781882	4.742599811	4.01435784	5.817037601	4.664669126	4.252567521	4.149937057	4.805974722
AP4S1	0.969541637	0.950151848	1.066261249	0.81149041	0.775099418	0.770099296	0.895769187	0.948701411	1.796973542	1.14619984	1.746031051	1.67113831
AP5B1	19.38612837	19.93679679	17.73783363	20.61606088	13.06055531	12.78657464	14.98538436	18.93748991	19.39215063	17.3389655	16.24158125	47.12568439
AP5M1	6.064975928	5.379858516	5.902817927	5.834883343	5.990084125	5.30594064	6.307271254	4.836930026	5.434007753	4.530335861	4.165383449	5.604635553
AP5S1	4.063170922	4.696112286	5.026954676	4.300867925	3.927342557	4.144534393	4.178369819	5.31902031	3.077296162	1.565422177	1.744765569	2.057102406
AP5Z1	9.851632957	9.517478923	9.727160371	8.915118005	7.443506849	7.340762163	8.630806051	9.183097024	8.667849726	6.963646566	7.741496296	10.19173886
APAF1	25.79920223	25.6515268	23.16915242	24.51802206	26.12715928	25.64513063	23.61866922	20.37299811	21.33060583	17.41035754	17.29477664	20.65070739

APBA1	0	0	0	0	0.007772416	0	0	0	2.97287043	4.105574809	3.399607463	4.446447355
APBA2	7.209358452	7.515476306	7.104244106	6.062608959	5.493075991	4.9907359	7.49622468	7.996025926	7.417961029	6.6478848	7.7051922	10.27341944
APBA3	8.688351899	10.09824925	9.077367973	6.427543854	7.692657823	7.404855455	8.91165625	7.274561882	5.848515062	6.506932668	6.729636005	6.734782257
APBB1	0.011679957	0.012108557	0.024724455	0	0.012195962	0	0	0	1.665103468	2.100437897	2.012996003	1.458621198
APBB1IP	27.22822588	27.90222189	28.17554718	21.97200436	34.20332988	34.30376007	28.74606535	22.2504178	47.97723866	35.61757679	43.56101809	41.41266471
APBB2	0.30192397	0.387527742	0.349994826	0.59577625	0.360300098	0.506931531	0.216846044	0.417359336	1.846243587	2.123064414	1.81194852	2.538603447
APBB3	13.51472752	16.69904662	12.82625195	11.61796234	11.53417599	13.82791827	10.94908095	13.29742629	11.28833871	11.10091566	9.212634085	14.01269943
APC	5.194952367	4.460392412	4.832236682	4.557416363	4.821196146	4.153217076	4.897857911	3.424094937	8.536338102	5.814938778	6.865749988	7.825963429
APC2	0.015342015	0.021206661	0.010825467	0.01033742	0.026699676	0.031511381	0.015776991	0.025020399	0.038957213	0.033646288	0.066103445	0.101119471
APCDD1	0	0	0	0	0	0	0	0	0.290863469	0.28626357	0.227642131	0.310494068
APCDD1L	0	0	0	0	0	0	0	0	0	0	0	0
APCS	0	0	0	0	0	0	0	0	0	0	0	0
APEH	43.01109137	44.15210468	44.70503137	42.71800805	39.1707287	47.13094768	43.17502255	49.0482596	33.15802013	34.01192835	33.16857356	40.1518104
APELA	0	0	0	0	0	0	0	0	0	0.01993391	0.039163313	0
APEX1	214.2680775	211.7138527	216.8459729	192.1515905	227.135043	228.5854384	204.0951974	215.4599868	128.7678351	160.8277634	160.7849449	123.6807618
APEX2	14.74304913	14.93789607	14.08188101	17.05539655	15.58211456	16.5121943	14.44789347	17.84419051	16.82676408	16.11002488	17.09801091	19.75331085
APH1A	57.70333527	72.35753624	64.36009236	74.75576889	63.36910856	69.14163209	66.50098275	76.63795688	104.8523207	118.2720771	117.4939348	112.961757
APH1B	6.10889566	7.83543288	6.300556291	6.162976429	5.73857297	5.895822698	6.190385468	4.635901085	11.35498832	10.40246998	9.498178495	13.84020848
API5	28.91775032	27.57009178	29.73037451	36.5978005	33.87062252	32.53255727	32.04030028	32.513202	28.96618359	30.52385334	30.59571759	34.9703826
APIP	5.544446165	6.558503079	5.416909186	5.927049418	6.605845546	7.445927046	6.140229989	6.851140608	12.84104448	19.05755552	17.72540871	13.27847073
APLF	2.069060797	1.449314671	1.627645088	1.568395353	0.978050275	0.933211174	1.552662544	1.299565651	0.486844434	0.352585246	0.256001289	0.378272634
APLN	0.100141863	0.674807924	0.088326274	0.084344244	0.540257679	0.839876697	0.120144595	0.359294176	0.417754315	0.713762593	0.539345878	0.451604023
APLNR	0	0	0.02937982	0	0	0	0.04281803	0.013580843	0.060416051	0	0.059800571	0.028887748
APLP1	0.150426089	0.311892054	0.136468326	0.152035216	0.089755269	0.132413239	0	0.147192691	4.770720623	7.564059172	6.180421277	5.993492368
APLP2	173.8566423	191.4574778	169.7312723	206.3285016	142.859721	166.9026425	174.6966735	190.0227301	427.9212112	476.9273916	388.1488537	579.6839919
APMAP	36.76948993	39.68849314	37.17119788	48.58347485	30.04023295	34.53488549	32.90827288	42.21522057	30.6440942	32.73615225	30.13716469	42.33432083
APOA1	0	0.085558469	0.043675446	0.083412835	0.086176072	0.042377652	0	0.16151205	0.044906639	0.54298504	0.311144115	0.257663645
APOA2	0	0	0	0	0	0	0	0	0	0	0	0
APOA4	0	0.038208303	0	0	0	0	0	0	0	0	0	0
APOA5	0	0	0	0	0	0	0	0	0	0	0	0
APOB	0	0	0	0	0	0	0	0	0	0	0	0
APOBEC1	0	0	0	0	0	0	0	0	0	0	0	0
APOBEC2	0	0	0	0	0	0.03381913	0	0	0	0	0	0
APOBEC3A	0	0.038631663	0.039440984	0.037662859	0.038910525	0	0.076641507	0	0	0.040861755	0.040139684	0.038780401
APOBEC3B	1.434455133	1.958610255	1.407155845	3.50073582	5.479856824	3.844518249	2.986228176	5.648721179	13.78289275	14.42499659	14.47158271	12.67075214
APOBEC3C	9.539645139	9.986284944	8.479811621	6.967628941	8.171210327	8.419183438	9.733471358	6.818627144	14.57873505	12.74886759	14.02881955	10.29619643
APOBEC3D	0.076002205	0.039395566	0.100552227	0.057611407	0.099199856	0.019512874	0.039078508	0.037184271	0.661675221	0.770890484	0.327467252	0.355925204
APOBEC3F	0.110185803	0.141106547	0.068601278	0.026203406	0.033839315	0.053250261	0.026661126	0.038053188	1.326060266	1.414342387	1.12404653	1.335555525
APOBEC3G	0.692255607	0.648652578	0.648151395	0.228735246	0.236312612	0.259759701	0.438081487	0.09118518	6.823587105	6.802582495	6.266517689	5.333888187
APOBEC3H	1.497230614	1.316994441	0.528229843	0.18342384	0.094750082	0.093187957	0.74651154	0.088790773	1.283739194	0.646759584	0.879688588	1.274848456
APOBEC4	0	0	0	0	0	0	0	0	0	0	0	0.020150737
APOBR	17.02834204	19.18314733	14.35834887	7.02761936	10.28313245	9.210076428	18.50342639	7.984027897	17.24936001	12.93054723	16.09537815	16.58406208
APOC1	0.661864217	0.548921247	0.350263108	0.468260984	0.20733136	0.271884175	0.408377623	0.194291248	5.402053235	6.023816815	8.127471362	2.410776703
APOC2	63.03616595	61.37018601	65.23398133	33.57109173	57.80534839	48.21077044	75.67786334	38.49658546	157.0389473	142.0475679	168.1604983	107.5424669
APOC3	0	0	0	0	0	0	0	0	0.106083799	0	0	0
APOC4	0.174990442	0.090705889	0.185212297	0	0	0	0.179951765	0	0.190433356	0	0.094246673	0
APOD	0.04761908	0	0	0	0.049722831	0	0	0	0	0	0.051293543	0
APOE	0.110567591	0.114624908	0.156035017	0.149000462	0.153936435	0.227097775	0.0758016	0.180318248	0.802167905	2.869390807	0.793995941	0.460264922
APOF	0	0	0	0	0	0	0	0	0	0	0	0
APOH	0	0	0	0	0	0	0	0	0	0	0	0
APOL1	0.7630209	0.636255395	0.772479155	0.318532122	0.329084209	0.459935999	0.818769438	0.422002508	7.816191525	6.420636296	6.700258592	7.785296972
APOL2	4.4136737	3.78826925	3.928301027	2.998063888	3.695909257	3.237630062	4.03777257	3.295189922	4.366527824	5.766872273	4.90860766	4.429207178
APOL3	0.107038058	0.039630663	0.040460913	0.015454723	0.007983347	0.03140691	0.062898742	0.014962469	1.26468529	1.140181147	1.268272572	0.564922111
APOL4	1.352624038	0.580245126	1.365813594	0.251419445	0.763010556	0.20756601	0.943303931	0.425969949	0	0.01704836	0.066988389	0.016179977
APOL5	0	0	0	0	0	0	0	0	0	0.019753724	0	0
APOL6	8.078996779	6.796611512	7.209492331	6.43112394	6.290176642	6.325734377	6.800910426	4.84323243	14.20110101	13.84603958	15.30926068	12.14205066
APOLD1	3.66832688	3.733582948	2.71428506	2.389070813	4.28444714	4.225260922	3.221960666	2.814847805	5.884947094	5.061709693	5.332572819	3.074949895
APOM	1.59654741	1.16472342	0.813611161	1.852681568	1.605337104	1.639596023	1.337773289	3.124459483	2.445289855	2.528758901	2.038213755	3.630697837
APOO	4.908102325	5.336412087	6.113396436	6.079765789	5.624929785	5.593661546	5.662775801	6.061822115	3.321992336	4.266167222	3.868411813	2.958784978
APOOL	3.976669547	4.19755111	3.877346712	3.759380869	4.135576549	4.191148545	3.478068928	3.25445093	7.291368526	8.377599284	6.559416449	6.178439272
APOPT1	5.164554	5.052431435	5.620213633	5.054383699	6.266185721	5.415861096	6.900532932	6.156780399	4.848520777	4.965221093	5.034186496	5.204358628
APP	77.08252974	80.53400001	77.96736835	84.54667029	67.75876956	71.2078316	72.30645014	77.41156336	99.30201706	130.8930786	112.7592448	96.810007
APPBP2	6.165678849	6.374839888	6.037273998	6.973098364	6.05075359	6.272671538	6.348964574	6.025082331	9.131787024	8.921157702	7.387275957	9.959362983
APPL1	15.75128601	15.07950174	14.43610973	17.36945777	16.44715773	16.92605706	13.96274146	13.61312832	25.51090708	24.88508282	24.38846501	24.11203608
APPL2	2.282426243	2.385009704	2.242740038	2.692335079	3.0659988	2.779188229	2.571267364	2.274833987	4.486743144	3.697731381	3.860635527	4.832488232
APRT	151.0268344	151.4535448	166.0593487	135.3226996	133.6786287	142.5678745	156.7486912	165.9839525	65.66942087	72.75012698	78.07233194	60.37056009
APTX	4.508022387	4.300329073	4.422702096	4.691884515	4.554309151	4.391518133	5.100044381	4.787172488	3.133374599	3.217447462	3.29858012	3.339238271
AQP1	0.046740118	0.048455263	0.037102789	0.047240103	0.036603778	0.024000199	0.036048969	0.011433861	2.187192229	0.525337535	1.850243309	0.23104866
AQP10	0	0	0	0.029381507	0	0	0	0	0	0.063754051	0	0
AQP11	0.249349216	0.206799339	0.211131719	0.378024805	0.130182573	0.281679817	0.153851255	0.585574736	0.515573162	0.246079411	0.376025881	0.700634972
AQP12A	0	0	0	0	0	0	0	0	0	0	0	0
AQP12B	0	0	0	0	0	0	0	0	0	0	0	0
AQP2	0	0	0	0	0	0	0	0	0	0	0	0
AQP3	5.435599681	4.740606814	6.240149742	3.342754884	3.8739161	3.810047651	4.613728588	3.29256687	14.74128224	6.780299564	13.35194777	6.015915909
AQP4	0	0	0	0	0	0	0	0	0	0	0	0
AQP5	0	0	0	0	0	0	0	0	0	0	0	0
AQP6	0	0	0	0	0	0	0	0	0.022064151	0	0.021839376	0
AQP7	0	0	0	0	0	0	0	0	0.036798234	0.012359525	0	0.011729975
AQP8	0	0	0	0	0	0	0	0	0	0	0	0.041236303
AQP9	0	0	0.01860594	0.088835623	0	0.090265368	0.162697325	0.068804872	0.019130434	0.115657055	0.056806635	0.03658863
AQR	18.39905654	17.32821733	18.87962278	21.73265596	21.5456464	20.09174547	22.03603241	18.55290191	25.140858	23.05584313	24.73641211	24.8247087
AR	0.018046302	0.009354259	0.033425796	0	0.009421782	0	0.004639488	0	18.65694448	14.89579703	14.96789195	17.29688466
ARAF	8.542842675	9.416550457	8.907999127	11.67886633	9.808678308	10.47350999	9.151585457	12.45444266	13.56349373	12.51951789	12.68221892	14.96893642
ARAP1	27.02867775	29.64087232	25.88395976	24.71702845	24.21227082	22.78539631	26.26607331	26.0093902	35.64731306	33.99596701	33.63133921	43.76959628
ARAP2	2.342524162	2.229660669	2.25462258	2.395273464	2.138474133	2.356447762	1.95814367	1.293535535	0.238526505	0.247854424	0.184450432	0.220973252
ARAP3	0.432012531	0.70663205	0.35563735	0.47544572	0.160390505	0.295774087	0.217194236	0.338181163	1.295490433	1.831714743	1.406385673	2.237957777
ARC	0	0.056768102	0.057957376	0.018448158	0.019059294	0.018745067	0	0	0.297955853	0.280210733	0.432550027	0.151964447
ARCN1	45.75688505	43.18546672	45.12543578	48.15579172	42.36395076	41.22518274	46.11723279	44.2751342	103.9059113	108.1208144	100.0907408	101.3027889
AREG	0.042170502	0	0	0.042621605	0.132100624	0	0	0	0.091784102	0.138725018	0.045424533	0.131658853
AREL1	5.262714616	4.903215308	5.190581293	5.573696133	4.149241253	4.62826247	4.993321165	4.884052249	19.34363175	17.31234255	17.63280214	21.89726394
ARF1	116.9393163	116.1426923	125.234465	128.5079586	111.4004024	113.1829906	124.1334794	137.6528519	147.1008774	168.7093951	156.1436531	165.4657585
ARF3	84.42145035	79.29010275	82.75543451	73.37846221	66.19105114	67.8311583	73.19237568	71.15179112	50.71323865	46.82064667	49.49973738	45.95329485
ARF4	58.78849149	45.87763112	58.81847586	49.54051278	44.27132237	38.90463319	59.27859364	46.69164851	70.03432928	70.29659082	67.32218569	69.38714132
ARF5	45.9050542	47.99673974	48.74243509	52.20182241	39.42303663	47.74785973	47.1560646	59.6669133	56.63481066	60.40411316	56.05785208	63.86735725
ARF6	47.62125645	42.74091883	47.77151293	45.72827581	57.15676621	47.7408836	47.8475709	45.87581874	57.27975742	49.44743329	52.38478422	55.89938315
ARFGAP1	17.28106462	16.70994096	16.84732848	16.43595519	15.15200144	15.80133678	17.68245695	18.01912698	16.15077029	18.09950133	16.77472619	20.13510686
ARFGAP2	29.50242463	30.07292157	29.53107179	30.60994928	30.64572777	32.93936166	29.60317623	33.51853082	45.65653281	40.21412408	42.07223377	45.59327883
ARFGAP3	13.92248786	13.27161949	14.1929628	17.12374528	11.22545995	11.85963954	13.22340581	14.47816137	19.65721937	21.99386492	19.02731536	26.62566775
ARFGEF1	16.59651889	15.7991574	15.73495947	18.82442577	19.13613068	17.3827278	17.23344595	14.86971793	19.11083629	18.11005083	16.69071841	19.29879941
ARFGEF2	13.08703875	10.59286115	12.03325587	10.5743915	10.56344202	9.893098391	11.23142583	8.15971553	12.3335205	11.30900823	10.80718164	13.37120365
ARFGEF3	0	0	0.003826957	0	0.003775487	0	0	0	0.428897327	1.034816671	0.408948991	0.688603581
ARFIP1	5.224308835	4.327711926	5.156218883	5.329928884	5.754843573	4.57345359	5.296522699	4.67063013	6.756378692	6.03443609	5.927801121	7.621858964
ARFIP2	13.30548424	12.57535973	14.94159031	12.71249781	10.85459994	12.24178645	14.4164946	14.99088833	13.67272877	13.54676613	13.29230958	13.20618857
ARFRP1	4.616322048	6.504144034	5.43305764	7.908277249	5.601929907	6.150219966	5.883600162	8.388889872	6.769404344	6.918697754	6.700442059	10.48008541
ARG1	0	0	0	0.070584255	0	0.107580387	0	0.034168026	0	0	0	0
ARG2	0.407442409	0.197117038	0.402493154	0.301987307	0.31199131	0.27895234	0.558658939	0.345526536	1.330197662	1.519042449	2.048116737	1.187255806
ARGFX	0	0	0.011244379	0	0	0	0	0	0	0	0	0
ARGLU1	87.33682008	88.50273562	92.63365	94.42085239	103.0358712	102.6310744	90.35022557	93.57847684	102.0672762	102.8448604	91.95751728	118.2341114
ARHGAP1	25.04471241	29.08827532	26.2807655	27.37739446	23.55710868	25.2537835	25.96227	29.54092112	29.12130806	24.82138551	28.72955314	43.28201743
ARHGAP10	0.497086013	0.225455457	0.460357355	0.204101383	0.275743527	0.239291824	0.383384535	0.744801815	2.468102059	2.469871328	2.14177196	3.750503617
ARHGAP11A	22.29204242	21.14570196	25.50896562	40.95352243	51.99696087	45.99755187	29.43097361	36.26810216	67.07700925	72.31702477	63.21667647	55.98997613
ARHGAP11B	6.54770061	6.174164926	6.377237	9.046594388	12.18289808	11.55283373	6.661709531	6.509196964	19.36781188	19.43898158	18.27012924	13.3020038
ARHGAP12	5.22811796	5.572948495	5.221136464	5.624949857	5.734245267	5.553107785	5.365505994	4.713494269	6.882459204	6.414775272	5.699662142	7.228849037
ARHGAP15	15.64634227	16.71428754	15.47563505	19.66502341	18.02559312	18.02487061	15.40716316	17.78166222	29.67182071	24.84894521	26.91301208	28.57025365
ARHGAP17	17.91012364	18.74521627	17.12634532	19.94256244	17.65398309	20.33129454	15.6219716	20.68923011	27.69200874	26.90437378	28.20603877	28.28102347
ARHGAP18	9.937750504	8.744665576	9.590525474	6.500448542	10.6501738	8.265103492	10.04391243	6.271123048	8.436254085	9.424223108	7.700433657	5.721909909
ARHGAP19	24.16353865	23.15567316	24.90980587	34.60518994	39.14397454	34.8642354	25.22647276	31.86613883	18.88806315	24.30153208	19.01852127	14.94310239
ARHGAP20	0	0	0	0	0	0	0	0	0.344750042	0.322563863	0.365612092	0.57301929
ARHGAP21	2.789284117	2.665581501	2.832012511	3.250713328	3.557627604	3.265708822	2.961797926	2.600419001	5.779198185	5.489474085	5.328855659	4.774916659
ARHGAP22	0.03095706	0.038511648	0.013106151	0	0.045254584	0.025433418	0.031834753	0.012116656	0.101067064	0.054313082	0.126714115	0.057989885
ARHGAP23	2.667833304	2.464948039	2.756262956	2.982458615	3.044313655	2.710698454	2.772580084	2.652034659	3.819686415	2.289096585	3.323422251	4.293445295
ARHGAP24	0.171593047	0.174401681	0.135322059	0.183630282	0.056211391	0.10365871	0.10033889	0.131689935	0.06224538	0.051651425	0.028993537	0.199583395
ARHGAP25	3.909641523	6.122778961	3.719814508	3.10021734	3.919504214	4.420836685	5.046995022	3.561067616	6.483841797	6.487630725	6.630594892	6.157173611
ARHGAP26	6.454489572	7.045586723	5.480286153	5.266794419	4.038828796	4.498624383	5.139171968	4.51854818	6.352677385	5.693313242	5.470014369	7.423385514
ARHGAP27	9.962811654	11.21599747	9.785040696	11.1794649	9.563134	9.636386619	10.43203985	12.24506385	13.51489186	9.758196915	10.99241567	14.73323648
ARHGAP28	0	0	0	0.008703019	0	0	0	0	0	0	0	0
ARHGAP29	0	0	0	0	0.005996457	0	0	0	0.006249547	0	0.006185881	0.011952807
ARHGAP30	64.3137318	70.99682727	60.86458553	47.87271054	56.91039726	57.09581496	62.41146815	52.70051344	56.54721886	56.39143775	61.36659362	50.22946868
ARHGAP31	4.133112172	3.963590663	4.095465224	3.517747032	2.91843717	2.525070568	3.42328308	3.250897449	1.599717178	0.83847941	0.830763117	2.353010205
ARHGAP32	2.266939521	2.77666093	2.578671657	2.629567747	2.514506655	2.72574076	2.480542008	1.807724226	3.432725414	3.494262049	2.993674199	4.059290493
ARHGAP33	1.767924765	2.851781139	1.906106325	4.58043531	4.497974685	4.674477922	1.682373622	5.054216364	4.924722857	5.215416686	4.917187561	6.789617623
ARHGAP35	11.81494587	10.86897564	11.18813336	10.85840268	11.18202105	10.73144972	10.68079517	10.19125301	24.21695183	21.45785887	21.78605519	28.2422452
ARHGAP36	0	0	0	0	0	0	0	0	0	0	0	0
ARHGAP39	0.006860839	0.0071126	0.014523213	0	0	0	0	0	0.283719721	0.225695682	0.155195177	0.521218872
ARHGAP4	139.1995253	136.1185508	133.8356581	109.5445033	118.7054429	121.4025733	109.6857242	121.0923663	39.54245295	39.6337212	41.42577278	41.11900164
ARHGAP40	0	0	0	0.019163202	0.019798026	0.038943241	0	0	0	0	0	0
ARHGAP42	0.397934304	0.27960817	0.336943324	0.174282792	0.152355329	0.118058498	0.268264166	0.203343273	0.611084523	0.378171011	0.576283167	0.524558296
ARHGAP44	5.869225715	7.13322141	6.410326974	5.704826226	5.137525797	6.065954259	5.31649095	5.25429547	1.073590696	1.328248854	1.358582518	1.546500107

ARHGAP45	37.39094404	32.28917932	36.72336726	29.83419015	33.05709159	28.85159422	32.78693478	33.42901395	26.57990518	22.60462275	27.11026614	34.41424048
ARHGAP5	0.191201485	0.246760799	0.169330242	0.260291597	0.248542039	0.248451663	0.184584316	0.297819319	0.038217862	0.085575597	0.012609508	0.044669172
ARHGAP6	0	0	0	0	0	0	0	0	0.329031786	0.407318686	0.325679825	0.314651061
ARHGAP8	0	0	0	0	0	0	0	0	0	0	0	0
ARHGAP9	20.84641182	23.20142449	20.8330839	17.7882131	22.14106863	20.80470946	22.18664213	20.73599885	20.40497936	16.73425724	19.64640169	17.14550608
ARHGDIA	90.67656478	89.7651295	99.65541246	117.7432051	93.45595977	97.18138578	94.82710129	141.9772286	130.6375223	126.8272026	145.5369226	152.2220277
ARHGDIB	461.8869903	471.9115827	492.5851845	444.7420636	391.6240908	422.1851728	434.7171054	456.1000051	225.2409363	213.1923702	230.8234138	200.4155347
ARHGDIG	0	0	0	0	0	0	0	0.111789657	0.186491265	0	0	0.356680884
ARHGEF1	59.20991943	58.34871623	59.0405936	54.9898114	57.97640219	61.50382768	58.79361587	66.90782268	50.8689667	52.5507709	53.15478352	68.97220142
ARHGEF10	1.861281531	2.139915488	1.876640827	2.157575541	1.602705404	2.618077463	1.551198968	1.838536678	3.023909996	4.952498306	4.351878734	3.598617761
ARHGEF10L	0.103597116	0.195844595	0.141898209	0.215569751	0.038179025	0.012516525	0.01880017	0.053666631	4.907487002	4.296694531	4.325794192	5.371581802
ARHGEF11	7.635101369	6.568120801	6.74851031	7.477449928	5.626519601	4.99995348	5.535298536	6.408545746	16.46695648	13.87555911	14.55108134	18.00715906
ARHGEF12	9.470176295	6.799915181	7.910249276	9.765038725	6.784009764	6.701664296	7.611724312	7.364570097	12.35876731	9.806937564	9.680053187	12.24212959
ARHGEF15	0	0	0	0	0	0	0	0	0	0.013328298	0	0
ARHGEF16	0	0.014321982	0.014622023	0.013962816	0.100977559	0.042562613	0.014206718	0.027036163	0.796813254	0.636247426	0.62500425	1.308318304
ARHGEF17	0.507537615	0.668664064	0.397292933	0.486249788	0.458194565	0.602663677	0.347951537	0.387990727	3.429035293	3.820385409	2.938518283	3.768839232
ARHGEF18	10.42036274	13.60965185	10.71356417	15.93582724	10.79642466	13.2586836	11.01594513	15.13050104	10.15054843	8.35526455	9.478561349	11.84604519
ARHGEF19	0.765583183	0.728886608	1.107966419	1.184346006	0.864663277	0.850407754	0.915825949	1.284217764	2.686470562	2.073034058	2.591783497	3.121889831
ARHGEF2	32.9523085	31.60914468	32.41358592	34.09803378	31.53982233	30.48434088	31.43214431	34.87401746	55.73856876	41.44453107	48.15279996	66.5721175
ARHGEF25	0	0.059662162	0	0.019388652	0	0	0	0	2.943570141	2.818747295	2.644954748	2.016359637
ARHGEF26	0.009857036	0.010218744	0	0	0	0	0	0	0.632888288	0.832265401	0.860028942	0.738582289
ARHGEF28	0.025030613	0.01297456	0.006623187	0.006324592	0.013068217	0.006426382	0.01287014	0.030615728	0.299635226	0.253885551	0.276361187	0.377710912
ARHGEF3	6.104758788	3.961916099	4.473923328	1.922919105	1.373359106	1.002418772	3.555741764	1.473148802	7.498697638	6.168020685	6.798582612	4.743334859
ARHGEF33	0	0	0.013232524	0	0	0.025678652	0	0	0.122449887	0.219347117	0.067334693	0.03903269
ARHGEF35	0	0	0	0	0	0	0	0	0.071008396	0.023849788	0.117141681	0.135809779
ARHGEF37	0.040603328	0.021046641	0.053718903	0.071815918	0.031797849	0	0.062631769	0.009932641	0.176746296	0.089046405	0.109341075	0.137329878
ARHGEF38	0	0	0	0	0	0	0	0	0	0	0	0
ARHGEF39	5.142860684	5.303370356	6.004882378	9.15815987	9.887740037	9.962252075	5.498538806	9.944813517	13.38474447	14.94876943	13.11649173	12.60152212
ARHGEF4	0.002849781	0.008863064	0.009048742	0	0.008927041	0.014633105	0.002930578	0.019519675	0.046519111	0.053123311	0.049114885	0.041520209
ARHGEF40	12.65891726	11.90000471	12.32774411	12.00163099	13.38655644	12.27006504	10.77155086	12.72391974	9.492484117	6.414043145	8.22722997	8.65042246
ARHGEF5	0	0	0	0	0	0	0	0	0.565522526	0.2902912	0.327407583	0.265300906
ARHGEF6	25.55574725	25.96033241	24.60338735	26.96249976	23.48893431	26.88557252	23.36701307	23.18312889	49.7864467	43.63845925	43.94808587	48.34752939
ARHGEF7	10.85618833	10.78582777	10.14123003	10.51534099	10.58242466	10.37337651	9.942226462	9.893309675	12.08120652	13.83467184	11.50218844	12.65944535
ARHGEF9	2.418271598	2.532039323	2.401956828	1.923591172	2.583928898	2.929758852	2.242702534	1.799326123	5.092593507	5.824106344	5.703851194	3.559340762
ARID1A	24.56318996	24.42464515	23.76184433	27.36683481	32.75108395	29.28166906	23.91861503	25.64246072	36.51448116	27.92447496	32.81322592	32.02174013
ARID1B	9.987221996	10.24517721	9.296378115	11.84517437	12.99183077	12.12182238	9.667505983	10.57156071	16.84973973	11.45075164	14.2423408	14.34290862
ARID2	7.242436979	6.24123089	6.429330536	7.094775854	8.901376914	7.994156738	6.94630324	5.96159806	25.17127138	15.78423127	19.09143607	18.63915015
ARID3A	27.92759468	23.79129322	26.25306706	22.79449225	24.70565461	21.28645815	24.32364785	24.46470712	16.45280366	10.78946319	14.17648972	13.33016436
ARID3B	6.181678611	5.664555175	5.969781475	4.759020369	5.113866324	4.059846511	6.188631316	4.274816595	4.137714935	3.741269408	3.471735175	5.240888988
ARID3C	0.121658514	0.277470196	0.167394564	0.331991759	0.279473111	0.26237161	0.175150882	0.309513031	0.516340046	0.667017571	0.445556845	0.94956306
ARID4A	7.33253974	6.044741699	6.24600078	6.135433722	6.883471792	5.568041488	7.286667154	4.505026493	11.79298233	8.527492781	9.356501429	9.369836716
ARID4B	12.44877136	10.98227168	10.37080198	10.43111158	11.81851827	9.998596661	12.24860735	8.237746283	25.40663189	18.75352706	19.89153643	21.62727282
ARID5A	6.447369303	6.736753671	6.177161402	5.929558416	5.658030829	4.759323992	5.750332607	6.587845092	4.400459598	1.809333455	3.10489535	4.261131428
ARID5B	0.949643251	1.078515143	0.847007455	0.447547987	0.941460338	0.630076615	0.779059472	0.396750204	1.242461532	1.088123501	1.080388686	1.321409669
ARIH1	17.83535548	17.38862075	18.75940456	19.73366712	18.0634378	17.12149572	20.16160093	16.28390056	23.14146575	23.20683421	19.81093763	25.85698356
ARIH2	12.81067365	10.79138868	12.85162234	13.97587648	12.9011016	12.19641688	12.82720038	13.22978612	10.83244083	8.722893328	10.12323006	11.36227382
ARIH2OS	0.606115204	0.453813255	0.356400384	0.374365992	0.4219284	0.311229116	0.311649882	0.263594134	0.879473197	0.406162777	0.725428081	0.665819198
ARL1	22.99482931	21.48058744	23.95144737	26.99961008	26.83652091	25.49039974	25.50778797	26.31834122	18.32091103	23.52164507	17.93754667	20.80304665
ARL10	0.009598566	0	0	0	0	0	0	0	0.062673839	0.021050437	0.031017679	0
ARL11	11.53262935	10.61443821	9.809692336	7.022026474	9.619015448	9.431541935	9.820329062	7.637839838	8.70874236	7.094096236	7.286873879	8.093933888
ARL13A	0	0	0	0	0	0	0	0	0	0	0	0
ARL13B	4.706448962	4.070310096	4.808221247	5.672540972	5.282294913	5.246900416	4.568127751	5.023946388	4.190558153	4.981426366	4.690072373	5.736089264
ARL14	0	0	0	0	0	0	0	0	0	0	0	0
ARL14EP	9.933255559	9.747077532	9.951275707	10.27215754	9.558596542	9.819232519	11.3620088	9.303923169	11.02182397	9.707860209	9.59352976	10.17156701
ARL14EPL	0	0	0	0	0.034491589	0	0	0	0	0	0	0
ARL15	2.936422019	2.982364401	3.234160711	3.464983041	3.066149399	2.525755031	2.866392353	3.456715793	5.920710202	5.213959949	4.351141743	6.406522222
ARL16	11.28264986	10.73694385	10.99249919	13.04074473	10.81444834	10.72528255	11.81078583	12.54045551	14.79697894	11.48364705	13.71137076	16.31795869
ARL17A	0.763654417	0.512261579	0.576481247	0.669670238	0.580454249	0.640082532	0.433072899	0.379114271	0.67828097	0.985150777	0.659274309	0.765507226
ARL17B	0.231372628	0.3358081	0.155095721	0.226052729	0.169116063	0.190089008	0.277587914	0.369785508	0.520368631	0.71038662	0.506759914	0.618018519
ARL2	23.06124037	24.36286131	24.98948567	25.97169276	22.9333872	24.86720636	22.86810509	29.81875022	51.32810492	68.69795013	59.44031855	62.05592259
ARL2BP	14.57982863	14.98202126	14.88908426	17.58453785	10.83602545	10.9468337	13.01693949	15.67051438	23.08868421	25.73714614	22.85347173	31.8927062
ARL3	2.243658543	2.894272128	2.280270088	3.002071614	2.688873092	2.644542201	2.884088352	3.692316225	3.412776886	4.794716987	4.751184427	4.285156201
ARL4A	5.256985386	4.343645521	5.215273645	4.409179606	3.998127403	3.771054915	4.615298342	3.900216913	5.36229009	6.383967012	6.068314848	4.589002792
ARL4C	0.080452869	0.125107674	0.141920711	0.013552247	0.140011958	0.13770361	0.179256711	0.078723541	2.451479488	2.043759789	2.614270711	0.865171126
ARL4D	1.260643226	3.232865252	2.106761331	1.944722428	2.113067026	2.418922662	2.592769888	2.7917089	4.115685145	3.928774618	4.502573774	3.038164672
ARL5A	25.79259165	21.82780401	24.72926067	22.37875035	29.20153277	26.88912184	28.46207751	20.88505235	26.38724869	27.25856865	26.06356192	23.30784082
ARL5B	4.533008357	4.361536093	3.908749625	3.864267127	4.680342935	4.588306141	4.021126451	3.124739792	5.776235019	7.654449855	5.756390432	5.727245743
ARL5C	0.228733521	0.059281745	0.060523678	0.057795078	0.119419338	0.176175745	0.117609283	0	0.373378897	0	0.061595859	0
ARL6	1.084836004	1.010422743	1.022620423	1.182100058	0.955768507	1.079271832	0.897703936	0.937120948	1.116010254	1.375436668	1.086382538	1.569980153
ARL6IP1	88.18847053	86.56156815	92.19245265	151.3766181	108.9122641	99.98695913	110.584578	146.3714687	157.4334544	156.3924544	153.089862	142.568293
ARL6IP4	60.76876504	64.10664886	64.81831553	74.93346787	69.88962989	70.47303746	64.63863259	85.77684905	23.17987358	23.51998848	26.15639006	22.70994604
ARL6IP5	51.83721793	48.52274871	52.24240089	37.69648669	45.86300245	43.05537608	52.99486473	34.09574024	50.13775581	51.04654754	47.44797358	45.34121367
ARL6IP6	8.418961008	10.62232517	9.325197666	10.53733255	12.74339763	12.19818491	9.597245611	10.60083214	13.54758949	14.20541942	13.95439439	11.39334782
ARL8A	22.45312595	25.07954663	22.20999828	24.39000597	17.59163277	19.11796366	22.07241237	25.40247127	9.591157424	14.43059629	10.30071502	17.18963413
ARL8B	55.39590637	56.53164762	50.65649392	47.97190919	36.17789848	43.07909115	40.93134026	41.50497164	59.36257483	47.28256732	54.29176664	61.42056777
ARL9	0.053809561	0.027892061	0.085429172	0.081577753	0.112373597	0.055260459	0.055335168	0.026326464	0	0.029502187	0.057961704	0.027999449
ARMC1	30.07307093	32.7389944	34.85684228	37.23185794	39.68125594	39.67778311	36.08585582	34.50425825	26.93083433	34.44112748	28.42743009	29.1935698
ARMC10	9.431409728	9.05416328	9.116519431	10.73599461	8.441209184	9.895736041	9.179229726	9.452336563	8.548746224	11.0014863	8.617154691	9.639873039
ARMC12	0.733850131	0.779798496	0.485448187	0.278137582	0.689643625	0.207250271	0.452793736	0.197470921	1.078126792	0.92540103	0.4545241	0.40094677
ARMC2	1.203930258	1.163491351	1.05828073	0.897138731	1.012086816	1.016356557	1.24855612	1.01831602	1.532241084	1.387285252	1.659502702	1.550216009
ARMC3	0	0	0	0	0	0	0	0	0	0	0.013410852	0
ARMC4	0	0	0	0	0	0	0	0	1.058494303	1.137662169	0.814886374	1.113454707
ARMC5	2.903115432	2.24448197	2.998297958	3.154410824	2.378121849	2.136785857	2.378219495	3.377215912	3.235812245	2.235306148	3.043841263	3.51136139
ARMC6	29.66374337	25.43755949	30.54740088	29.58553172	32.40445914	31.68936115	31.3901316	34.77007921	10.51919236	9.054489445	12.26680419	10.9350577
ARMC7	4.612792677	3.952116309	4.236657483	5.49053243	3.323838247	3.797566051	5.429628587	6.080359417	6.554873975	4.493779839	5.153520233	7.042015263
ARMC8	7.277460889	7.068589407	7.806115479	9.24928182	8.290499658	7.690091787	7.692749213	8.343464027	8.624034222	8.211098248	7.57150087	9.891833459
ARMC9	1.240810948	1.300540989	1.243713066	1.752395608	1.730364631	1.718714186	1.456262754	1.533086027	0.500778172	0.522614464	0.41896472	0.612866544
ARMCX1	0	0	0	0	0	0	0	0	12.41730439	14.05522229	11.72228757	11.06377123
ARMCX2	0	0	0	0	0	0	0	0	0	0.035247535	0.017312337	0.016726075
ARMCX3	6.915143093	6.634341593	6.175242499	5.946816152	4.587580566	4.903658439	5.571288234	4.368161027	9.877574209	9.90635269	8.18676728	10.71021208
ARMCX4	0	0	0.023014324	0	0	0	0	0	3.162966202	2.328701735	2.97459713	2.496718146
ARMCX5	4.366126649	4.543819436	4.870961559	4.515059423	5.192702254	5.176339947	4.541921674	4.783630706	5.063307942	4.917031196	5.992281393	3.859573228
ARMCX5-GPR/	0.068252931	0.048985959	0.05556911	0.037144715	0.060303911	0.080876854	0.070188036	0.051373723	0.045708465	0.103627548	0.090485634	0.054638403
ARMCX6	6.08692863	6.022149501	5.677627475	6.994786664	6.907261402	5.651637808	6.05942954	6.935173927	11.37288464	13.56247241	9.879835851	10.67334384
ARMH1	2.011314871	3.12768117	2.274611849	0.765792185	2.071402714	2.631450408	2.578341156	1.36148125	13.26780786	22.2512656	20.13672091	10.16467467
ARMH2	0	0	0	0	0	0	0	0	0	0	0	0
ARMH3	11.42883152	10.715594	12.29753735	11.06139022	11.25918369	10.8686707	11.71378289	11.32261065	7.909086627	7.292207282	7.88991411	8.186279703
ARMH4	0.003779292	0.003917975	0.004000055	0.00381972	0	0	0	0.003698056	0.057579407	0.078738805	0.040709161	0.169121551
ARMS2	0	0	0	0	0	0	0	0	0	0	0	0
ARMT1	19.76804213	18.58756915	20.63855107	19.91687172	23.72571151	19.643449	24.06708069	19.34312952	15.15096555	14.94928486	15.5751219	13.84387365
ARNT	23.8262835	25.96388501	22.27652491	23.22767431	22.65776743	22.4488752	22.35830979	22.5296797	60.73354393	55.33564785	50.70785517	63.84141447
ARNT2	0.016407855	0.008504974	0.04341575	0	0	0	0	0	3.222980759	3.355485841	3.198984102	4.80673579
ARNTL	2.910659389	3.22556824	3.080682038	3.512401889	2.227035618	2.293392778	2.631936173	2.185176321	6.294091048	5.640427485	6.675934159	5.14422153
ARNTL2	3.387229369	2.741294086	3.52317162	3.636320797	2.993208451	3.334372092	3.230591993	2.988130962	2.928549494	2.649359274	2.766382682	3.214548661
ARPC1A	21.99930397	23.3967007	25.30447159	28.63089772	23.88026351	23.38339255	25.10226346	30.37290887	72.84253632	70.71712819	76.68113378	69.83310098
ARPC1B	120.5133475	114.6890334	124.4730251	121.2700519	85.39345619	93.18278724	111.8570107	126.097013	115.5058208	114.5702653	127.0833997	131.0295913
ARPC2	103.7490202	113.0674327	107.9143691	101.8799716	89.26323499	97.86207137	101.9175501	101.1011693	109.1428789	125.4088366	122.3322775	108.6582166
ARPC3	500.0957823	501.4132072	523.8102079	555.493422	491.1186071	510.0430153	567.2290189	536.1427245	348.1543384	350.3113852	351.115336	313.7231069
ARPC4	72.14256358	70.52764257	79.09348292	63.24464585	57.18579505	59.9872061	77.19269214	72.81841793	74.40078408	66.17789992	81.83156432	72.19848418
ARPC4-TTLL3	0.094473771	0.111932022	0.028569241	0.040921873	0.028185001	0.124741441	0.08327339	0.132061521	0.07343649	0.118393528	0.116301387	0.112362977
ARPC5	158.4879875	166.7105535	169.6297365	162.7383616	136.8307464	147.8204021	160.0369453	155.2832287	127.1101572	129.096819	132.9284709	117.205749
ARPC5L	18.1120244	14.70109926	19.15144081	18.21708028	21.71603274	18.22189289	20.39423996	18.34094176	13.61775515	12.93246561	17.03310339	14.26455968
ARPIN	0.023968624	0.00828272	0.033824963	0	0	0	0	0.007817807	4.025608466	3.451777802	3.72641688	3.774832973
ARPP19	20.96543132	19.87026331	21.21112657	24.87712471	23.73384746	22.31351001	22.20276273	21.93941849	28.82710315	31.89222589	30.12015955	28.11977174
ARPP21	0	0	0	0	0.002136868	0	0	0	0.002227058	0.002244024	0.00440874	0.004259443
ARR3	0	0	0.042062615	0	0	0	0	0.038886949	0	0	0	0
ARRB1	26.48770505	21.92379776	23.48307183	13.2818671	17.67706651	15.34232554	21.63485534	13.66166196	16.24303369	12.45314686	14.0669446	16.39408678
ARRB2	64.588859	76.55672275	62.68026214	59.32727802	60.56646428	64.656522	62.97839381	64.00860834	17.57220719	17.277129	20.45982593	14.54252502
ARRDC1	11.48113254	12.50667924	10.97618491	12.39310505	10.4611023	11.93752395	9.533526777	12.88075929	17.0061891	15.01337545	15.03237317	24.27420078
ARRDC2	14.69028419	14.16859865	13.74989049	14.44115513	13.54588354	13.79165944	13.67877834	15.39360655	12.32805412	9.697153543	12.22214532	12.2265847
ARRDC3	3.577540788	5.01016023	3.199718856	5.212264409	5.111862706	4.254109952	3.431554929	3.592763966	10.64385116	13.43198335	9.464974937	6.749478476
ARRDC4	1.75969304	1.015004919	1.862483382	0.494775323	1.533497578	1.168527031	2.190548814	0.724997292	6.666455132	2.654979224	3.634185993	3.098045793
ARRDC5	0	0	0.050356128	0	0	0.024429911	0	0	0.465980825	0.130425231	0.307489144	0.371345483
ARSA	7.397033631	7.262275331	6.773510398	6.204133311	4.240045263	4.157947125	5.372346396	6.262120116	8.579585766	6.379555038	6.688872686	14.58047234
ARSB	6.05458662	3.988858176	5.70302348	6.045391657	2.585935976	2.768758213	4.321141519	5.016179772	6.098508627	4.451407241	4.418381962	7.31100075
ARSD	4.862551803	4.965995001	3.964151768	3.8666677	3.247840339	3.3263577	3.595339511	3.877205924	11.79911707	7.015307241	7.679168207	14.09382293
ARSE	0	0	0	0	0.039470881	0	0	0	0	0	0	0
ARSF	0.067010661	0	0.023641669	0	0.046647404	0.022939169	0.045940364	0	0	0.024493306	0	0
ARSG	2.198081143	2.036321304	1.541557776	1.863708286	1.541753476	1.598669834	1.600831154	1.660521951	1.74496917	1.794893434	1.604849754	2.614301709
ARSH	0	0	0	0	0	0	0	0	0.034670371	0	0	0
ARSI	0	0	0	0	0	0	0	0	0.036898713	0	0	0
ARSJ	0	0.018102911	0.018482162	0	0	0	0	0	0.076012665	0.028721909	0.018809574	0.027258916
ARSK	1.594240436	1.750924324	1.871138605	2.249430558	2.406357466	2.334264017	2.158866919	1.899768314	1.545979213	1.852000016	1.615242548	1.708385297
ART1	0	0	0	0	0	0.021205088	0	0	0.15729386	0	0.200174725	0.042976899
ART3	0.818762618	1.410518181	0.9940293	0.900537587	0.653773446	1.187067358	1.002942179	0.765817933	0.104825701	0.092421262	0.025939451	0.22554938
ART4	0	0	0	0	0	0	0	0	0	0	0	0
ART5	0	0	0.029509213	0	0	0	0	0	0.030341066	0.061144429	0.090095912	0.058029947
ARTN	0.802714642	0.860866076	0.761714153	1.17498821	1.705257777	2.615206887	1.252442071	1.923023617	7.621007735	3.733301463	6.082401001	4.868217029

ARV1	16.18332657	17.09175157	18.77015223	20.61593171	25.20660891	25.27577366	25.03257595	27.15122808	23.29855466	23.43908104	23.89649186	27.64857913
ARVCF	0.166949531	0.26259777	0.121863232	0.186190799	0.21039242	0.248308469	0.136162283	0.180260373	0.269391789	0.315632686	0.254245196	0.335502122
ARX	0.449036669	0.232757114	0.65349158	0.226920036	0.19536439	0.038428692	0.134682258	0.109846165	1.058772381	0.594967613	0.644914644	0.681488685
AS3MT	0.436234786	0.384406189	0.16160092	0.19840556	0.318854958	0.201598754	0.381312466	0.320143464	0.901991799	1.435047613	0.352422195	0.658276476
ASAH1	32.90213087	35.16299436	26.36049785	38.89465618	16.24720431	21.7277964	22.9906487	32.16229956	25.83452517	26.61590509	20.90569188	62.18825081
ASAH2	0.252577638	0.261846063	0.200498741	0.138276392	0.153846114	0.108078347	0.227271372	0.102978542	0	0	0	0
ASAH2B	0.583173331	0.473854578	0.600556569	0.27081074	0.526648376	0.598897764	0.372791113	0.262185056	0	0	0	0
ASAP1	10.92233633	11.92154398	10.42573901	14.22136549	9.275753187	10.75492625	8.341132857	11.37708161	20.39504634	20.64906497	17.66790298	23.13133673
ASAP2	3.51860056	4.41083753	3.474820857	4.359741286	4.127539106	3.84782126	2.649426643	2.967579544	6.064104049	6.118374248	6.208483443	6.457875748
ASAP3	0.012282484	0.025466387	0	0.012413871	0	0	0	0.012018473	0.454458055	0.363642572	0.264604901	0.549635391
ASB1	6.076028788	5.635086894	5.94325831	5.588490474	5.700228192	5.052843091	5.605797849	5.326428308	6.63773567	6.7910695	7.259934725	6.900299726
ASB10	0	0	0	0	0	0	0	0.022744245	0	0	0	0
ASB11	0	0	0	0	0	0	0	0	0	0.019733904	0	0.037457457
ASB12	0.249696338	0.517718067	0.396423072	0.799163344	0.478000607	0.427381737	0.256775721	0.447936745	1.086928205	1.323377306	1.210337199	1.559134074
ASB13	13.18107321	14.25798783	14.64022724	15.23662223	12.13568918	15.09682495	12.6822442	15.65879167	6.914469664	7.746082148	8.841976062	10.5549813
ASB14	0.040621209	0.126335454	0.085988095	0.027370493	0.028277201	0.083433002	0.0417729	0.039748059	0.058941376	0.133628427	0	0.042273955
ASB15	0	0	0	0	0	0	0	0	0	0	0	0
ASB16	0.02515641	0.0782386	0.026625891	0.101702045	0.052535576	0.025834716	0	0.024615675	0.082129393	0.193095194	0.081292712	0.15707966
ASB17	0	0	0	0	0	0	0	0	0	0	0	0
ASB18	0	0	0	0	0	0	0	0	0	0	0	0
ASB2	1.634194283	1.509343988	1.823998518	0.570578798	0.062050578	0.106798236	0.794430903	0.31981348	0.921540765	0.570169272	1.040174141	0.572048387
ASB3	0.190949471	0.296934644	0.202103553	0.135094457	0.219323912	0.215707965	0.215999591	0.112107016	0.332481232	0.314075804	0.267388981	0.317949745
ASB4	0	0	0	0	0	0	0	0	0	0	0	0
ASB5	0	0	0	0	0	0	0	0	0	0	0	0.011416697
ASB6	4.819809216	4.489764879	5.323150481	5.577362595	4.740989067	4.112851092	5.590982994	6.276885224	6.562781731	4.991499431	5.906525838	7.523867628
ASB7	5.127216536	4.369683734	4.439032058	4.70518606	4.237001378	4.220985923	4.808940934	3.960255326	6.583810274	6.105090175	5.59061639	7.19081729
ASB8	10.33232449	9.962821459	11.11225981	13.30621296	9.377356275	9.755201392	10.91089167	12.30259406	8.22153093	6.456775995	7.000880242	9.076215194
ASB9	0.15589443	0.134679193	0.055000272	0.105041369	0.027130275	0.053365967	0	0.05084783	0	0	0	0
ASCC1	8.013766824	7.679078098	8.113561416	6.903660537	7.215414828	7.576359988	7.699072693	7.034010935	4.804114216	4.927933704	4.59452529	4.407895585
ASCC2	13.19857153	13.28689616	14.22489606	14.53860943	12.83699883	12.93505597	13.6037887	14.91160832	17.7436004	16.35764833	17.50870069	21.45595769
ASCC3	14.4607831	11.51418674	14.01868035	11.51817732	11.05962902	10.67164358	13.42929513	9.667732653	9.985412521	10.14382751	10.34975397	9.50433198
ASCL1	0	0	0	0	0	0	0	0	0	0	0	0
ASCL2	0.727549498	0.693907409	0.523632927	0.794158763	0.850854712	0.896600193	0.718249881	1.053628095	0.443383234	0.414849576	0.783689882	1.090297652
ASCL3	0	0	0	0	0	0	0	0	0	0	0	0
ASCL4	0	0	0	0	0	0	0	0	0	0	0	0
ASCL5	0	0	0	0	0	0	0	0	0	0	0	0
ASDURF	0	0	0	0	0	0	0	0	0	0	0	0
ASF1A	56.29736838	27.88094404	57.79265891	25.12252308	55.58215791	32.22241964	76.19888602	25.16176208	40.306939	24.3637034	34.4905634	23.94851274
ASF1B	36.21498464	49.14545672	48.95767682	82.34750254	62.32157907	59.72981974	45.13764127	84.68269928	59.47164409	50.68717773	69.14553325	71.17133301
ASGR1	0.660026892	0.864311763	0.882418819	0.421318285	0.217637696	0.606473722	0.928802043	0.441890296	0.415843013	0.266643396	0.224512732	0.072303291
ASGR2	3.138469213	2.387749329	3.000334669	1.432535015	2.114272761	1.559561389	3.669924116	1.857464546	1.459824847	0.94362593	1.608532698	0.711180747
ASH1L	10.99892003	9.650744055	8.789124162	9.191359652	9.486678072	9.010249321	8.377004207	6.759845773	36.02813483	30.94601363	27.67600434	33.49885188
ASH2L	26.05854221	27.5802962	26.95893287	32.89687739	29.67808027	31.5158425	28.62411855	33.83364669	28.54087133	29.87796237	28.47611643	27.70104094
ASIC1	1.064862889	1.679395664	1.067115271	0.27478822	1.253852769	1.361152348	1.08340434	0.609665486	2.219049741	3.8508118	2.281860755	1.968803391
ASIC2	0	0	0	0	0	0	0	0	0	0	0	0
ASIC3	0.976664458	0.747324016	1.033715132	1.386657281	1.286905932	1.194046209	0.956528399	1.865834107	0.936324767	1.274943266	0.751448189	1.645602904
ASIC4	0	0.018265921	0	0	0	0.018094453	0.018118915	0.034481289	0.287614229	0.405727525	0.227747362	0.38506119
ASIC5	0	0	0	0	0	0	0	0	0	0	0	0
ASIP	0	0.038604929	0	0	0.019441799	0.019121266	0	0	0.162098981	0.122500431	0.080223811	0.038753563
ASL	10.39559133	12.59889777	10.58368153	11.60750609	9.045712751	9.557466615	9.061324639	11.140914	13.30624609	14.05899996	13.19735203	18.28850882
ASMT	0	0	0	0	0	0	0	0	0	0	0	0
ASMTL	3.460356083	10.37992219	9.167057265	7.09821645	13.74737881	13.52072864	3.474239992	6.351209391	5.548328009	0.965444481	3.462704518	7.62846491
ASNA1	55.57045212	52.00574511	60.33354001	63.65630845	50.88454366	52.31659852	56.85119988	68.20036835	31.99758643	31.34326735	37.97947249	39.16513549
ASNS	14.33370743	14.47010806	17.00818012	21.95663275	27.95325931	22.21812252	17.66603298	21.99270967	12.36597713	12.04811635	20.91401678	16.66744744
ASNSD1	12.65210638	11.82957498	12.79190353	14.81231018	14.21155368	14.53634078	13.5706644	14.02089309	10.8063801	10.96034311	11.75184684	10.96862284
ASPA	0	0	0	0	0	0	0	0	0	0	0	0
ASPDH	0.042807924	0	0.045308497	0	0	0.043962179	0	0.041887771	0	0.046940632	0	0
ASPG	0	0	0	0	0	0	0	0	0	0	0	0
ASPH	3.00549546	3.203511859	2.886354937	3.412867977	3.320699087	3.204276793	2.532077994	2.647070412	7.313840751	8.575869141	7.50691099	8.13052194
ASPHD1	2.638026063	2.65486359	2.873764194	0.982300924	1.304796643	2.154650907	1.745088437	1.268017769	3.072293877	4.652007725	5.018473195	3.75680686
ASPHD2	1.575468579	1.852174279	1.856595346	2.134039215	1.526354324	2.752181003	1.904077615	2.081960039	1.679153161	1.88785502	2.116923074	2.062138744
ASPM	9.795237842	8.766222651	9.640335777	23.8859181	23.62962444	20.41307534	12.5734024	16.79013291	24.73451729	25.25352539	24.35477537	16.10328364
ASPN	0	0.022083975	0	0	0	0	0.043812484	0	0	0	0	0
ASPRV1	0.123586497	0.051248619	0.130805653	0.074945111	0.154855669	0.329988958	0.025418084	0.193488023	0.322783227	0.271035252	0.239621191	0.360121536
ASPSCR1	8.831025864	8.429505765	9.216153975	9.195961944	8.94173995	9.449665256	8.658027415	13.15316075	7.706212865	6.726588965	7.428144884	8.890414314
ASRGL1	1.182834031	2.004802699	1.748724618	3.776220709	1.274299341	1.523229667	0.868835504	2.553648652	4.352026781	4.405769752	5.076199448	6.955899507
ASS1	170.5302998	172.4071531	187.4254634	116.5540881	203.1027447	182.3441061	174.7223354	135.1310981	39.42886361	37.70028565	56.91128245	17.95503727
ASTE1	1.479604846	1.350355942	1.244796395	2.364572548	1.954323431	2.337692724	1.547564037	2.314006486	3.344220086	3.203292708	2.983222821	3.895575571
ASTL	0.571361548	0.789770482	0.483789553	0.166825671	0.145836429	0.078235666	0.182796685	0.16151205	0.013817427	0.013922693	0.013676664	0.01321352
ASTN1	0	0	0	0.005484034	0	0	0	0	0.053143523	0.059498209	0.029223406	0.062114338
ASTN2	0.332786632	0.267549744	0.280343111	0.144148497	0.085099279	0.153443151	0.202539426	0.126266015	0.118254716	0.096813943	0.146312517	0.113086253
ASXL1	10.37785394	10.46354726	9.609001386	11.43574245	12.7171544	12.72542688	9.70867124	11.65394268	15.07328741	14.58082317	14.79149671	13.62266402
ASXL2	7.324581259	6.625101924	6.902079008	7.534886803	7.053935525	6.958266028	6.582110301	5.467073371	9.446407899	8.163850227	8.477539213	9.876625495
ASXL3	0.004865241	0.005043772	0	0	0	0	0.00250159	0.004760663	0	0	0	0
ASZ1	0	0	0	0	0	0	0	0	0	0	0	0
ATAD1	15.41239515	14.90870073	15.45594708	13.95421953	13.76211894	13.52852182	15.40630963	12.13003893	8.361405867	9.828097292	8.241067171	9.755479647
ATAD2	34.03587677	35.95645005	39.52410447	57.86425575	57.08538355	53.48298829	40.47968885	47.71405526	59.47372308	62.91679378	62.90901889	59.41015251
ATAD2B	3.708705388	3.091842947	3.342299372	3.663268073	3.704020176	3.178124965	3.384480143	2.619599916	3.803079293	3.185682183	2.861046536	3.443333438
ATAD3A	16.04028504	14.65662539	17.15492789	19.15262783	17.95640497	18.84793457	18.81587511	21.42618296	10.08785226	10.49193901	12.79778344	12.94671249
ATAD3B	9.569998079	8.254023281	10.59625436	9.815781968	10.56897882	10.71855364	11.43020608	10.42876635	5.576159843	5.082709071	6.572276389	5.775450451
ATAD3C	0.041831739	0	0	0	0	0.014319891	0	0.01364419	0.015174464	0.015290069	0.015019877	0.029022492
ATAD5	6.568366266	8.257609627	7.847712251	11.23408063	14.43941817	15.06350992	8.225684591	9.345040449	10.98515481	12.40181807	10.38854299	10.93138764
ATAT1	3.526120236	4.076146774	3.466246474	3.456436209	3.934084644	3.571591577	4.023472709	4.017503508	0.946183687	1.366528598	1.071823245	1.266761446
ATCAY	0	0	0.011309131	0	0	0	0	0	0	0.035149548	0.034528418	0
ATE1	8.477251048	7.66585731	9.034624643	9.212649056	9.50031036	9.360919982	9.131899488	8.138987985	4.311261478	3.94835074	3.94186598	4.341203045
ATF1	5.350725996	5.150107115	5.631286241	5.886634354	6.660345231	6.685066711	6.010186773	5.570955899	2.313818772	2.364594777	1.877785531	1.845656591
ATF2	13.3204478	14.08281452	12.24059777	12.92952303	12.95051798	12.23035309	11.94012578	10.55303894	20.00220209	19.17327644	17.27728395	18.24441736
ATF3	7.026256563	5.68558453	5.953058547	3.86062089	2.415062657	2.375246021	3.459574156	2.86324944	4.061513759	1.460218968	1.811893048	1.130554128
ATF4	51.45195999	50.65287127	54.84735735	59.61112705	62.71185254	55.20543197	57.0364109	63.18482474	32.06345193	31.68345419	53.24338723	27.67184819
ATF5	13.23760784	7.743250519	10.9488892	6.857953812	5.162814341	3.043016454	9.105330655	6.502267786	27.74314297	20.6294212	46.12006528	21.12959428
ATF6	13.57735593	14.01955958	13.54827022	15.43621218	12.51965448	13.27749406	13.46910501	13.65565527	12.79813276	12.1697448	11.03789913	15.45136576
ATF6B	84.25227878	80.34960648	74.80495281	87.64826866	73.5196528	71.71607677	74.54171697	92.60555169	85.88992062	79.41682037	79.14342717	94.46603233
ATF7	6.017508511	5.497071258	4.862149486	6.043507094	5.279074801	5.536442933	5.407281809	4.959430337	11.43070399	11.20555792	9.010509435	10.93111149
ATF7IP	5.585088087	4.705882794	4.50962785	4.855571363	6.919039702	5.7169226	5.358349024	3.936792825	13.02564642	9.844912159	10.68427568	9.457507315
ATF7IP2	2.428482177	2.246470445	2.53079535	2.463900192	3.013664429	2.570700034	2.554965224	2.467677599	4.909501503	4.895693619	4.40673949	4.82129037
ATG10	0.98627055	1.219903053	1.151870182	1.03806857	1.072456907	1.096687144	1.021227975	1.151429225	2.390888261	2.364351747	1.802373796	2.236841364
ATG101	16.65621959	13.05585827	16.02652048	13.92290178	15.42533936	13.69185006	17.16643506	17.0932293	9.485071151	7.006010132	8.035871063	12.4911751
ATG12	10.78676647	10.68014709	9.812188188	12.32144351	12.45711933	12.35383924	10.95201822	11.41826783	11.58993679	11.48745671	10.66870157	11.74295615
ATG13	13.57896276	13.14863375	12.92257921	13.12066794	11.21024909	11.91866103	14.03737733	13.59830151	21.36069594	15.85075292	18.66800347	20.65677113
ATG14	4.307002273	3.676417168	4.179694761	3.584220051	4.181886468	3.860190037	4.095492675	3.765614832	7.511526945	5.814568283	5.880522118	7.555776579
ATG16L1	9.450926922	8.119043175	9.558047788	8.812411579	9.592074998	8.554498071	9.959049311	9.110662945	9.793597391	7.460911918	9.106830176	10.17572583
ATG16L2	9.30469432	11.2620774	8.261629874	11.58325622	9.603514971	11.80034654	7.382116736	12.10257653	10.19732579	11.95149647	8.311489581	23.55558564
ATG2A	5.216575258	5.381915731	5.041954455	5.043512362	4.264802203	4.211715125	4.674510753	5.243955923	6.56224857	4.561435423	5.74558035	8.693095892
ATG2B	3.934777178	4.091464206	3.645614207	4.36456266	4.063188789	4.235809384	3.485135509	3.393592867	7.031982953	6.135899883	5.669657351	7.152648356
ATG3	25.00561946	23.27984425	28.51361469	28.82769394	24.91682541	25.35479864	27.21549923	26.87708302	24.41841046	24.27663637	24.85155399	25.87661535
ATG4A	5.359729435	3.777622968	5.373257145	5.452819337	4.303591085	4.069146193	5.056928557	4.863732038	5.620976681	5.159488355	4.325215888	6.001196917
ATG4B	7.424911245	6.859561898	7.439858008	8.099918727	7.717773654	7.849889922	7.072720926	9.439964934	11.61637515	12.87720623	11.1806603	17.07475811
ATG4C	4.103938378	4.066671312	4.140584536	3.910819374	4.852900991	4.324065201	4.571070722	3.671519566	3.387284283	3.822193029	3.628347535	3.172679292
ATG4D	8.076678726	7.883720903	10.38028275	9.991817038	7.803722032	8.698405515	9.303427348	12.44477107	11.8143852	12.56574639	12.08947815	11.89840151
ATG5	15.13937131	15.87512891	16.1240767	18.76727551	16.6992776	15.35283225	18.1037818	18.2622932	16.25185108	15.69984234	15.62667958	19.27480453
ATG7	7.682947729	6.453468976	7.390818948	6.560601996	3.842038913	4.05199793	5.562668742	5.802521399	6.107032507	5.049723899	4.966721623	8.158102131
ATG9A	13.67061319	15.20854391	12.88962738	14.1441281	12.40755051	13.89463649	12.34979021	15.01614125	24.42225582	21.1193051	23.94597857	29.86021869
ATG9B	0	0.025298921	0	0	0.012740771	0	0.025095314	0	0.066392582	0.093657737	0.157718922	0.101585304
ATIC	52.48908084	42.83962864	54.19865908	47.81027452	53.51027123	55.99832828	58.37051451	51.28482625	30.81123739	35.17691318	39.28259815	32.11196328
ATL1	0.220155039	0.190194755	0.330104767	0.148340044	0.210724441	0.169568403	0.150931245	0.197470921	3.453998797	2.414089643	3.774526219	3.207574158
ATL2	10.90128198	9.806645985	11.20743925	11.77886578	13.44033721	11.54892585	12.01756623	11.12937753	10.87834248	10.13561298	9.878855417	9.119201448
ATL3	6.543930101	6.236371244	6.44903205	7.845589187	6.332875196	6.763781746	6.207911883	6.286093812	11.09226312	10.37346181	11.14618964	14.73462321
ATM	12.38776231	11.26330056	10.42698457	9.204838962	11.01995187	10.85979407	9.566808548	6.846965879	13.56462126	15.12746051	12.57975559	12.00106246
ATMIN	10.52206335	10.84330893	11.4347057	14.50271163	11.20469298	10.68797241	11.74263737	11.58160342	14.19697279	12.01813904	12.13151937	16.44153716
ATN1	17.20590846	18.40535176	17.64389174	17.19304832	23.05197237	20.2083958	17.91912768	19.83867971	12.74791656	12.4711667	13.43127055	11.9879969
ATOH1	0	0	0	0	0	0	0	0	0	0	0	0
ATOH7	0	0	0	0	0.036989334	0	0.036428682	0	0.115651594	0.194221114	0	0.073731269
ATOH8	0.04961693	0.077156462	0.061267784	0.175516911	0.060443767	0.067939706	0.034015779	0.0890091	0.728940959	0.353645397	0.347396103	0.860594726
ATOX1	45.44652073	44.71855972	51.4787921	49.26918137	35.50454147	43.39471593	51.9403723	50.93013312	40.11659737	40.54288299	45.1603458	44.20362976
ATP10A	5.920431596	4.441476047	5.786385889	2.935431008	5.619365988	4.791174207	5.203086978	2.893371321	4.254751856	3.163135955	4.933362737	2.277156345
ATP10B	0	0	0	0	0	0	0	0	0	0	0.006924935	0
ATP10D	5.515678109	4.962239986	5.22556447	5.406478469	4.17884143	4.280854375	5.101592797	4.016821336	5.493609683	5.448562992	4.225485024	7.282331027
ATP11A	17.18458028	15.96616094	15.46596064	15.22284244	15.07602496	15.27949796	15.07383535	12.32875316	15.89196053	13.63495767	13.85826009	18.84284692
ATP11AUN	0	0	0	0	0	0	0	0	0	0	0	0
ATP11B	7.233123719	6.122835586	6.380223109	6.339172541	7.3600512	6.428974555	6.541696927	4.850416289	10.22543411	9.357176977	8.339306216	9.363598014
ATP11C	7.003714558	5.897914061	7.188665393	6.333126311	8.319733619	7.417561553	6.984487802	5.252429739	8.527824352	9.265480608	7.906014139	6.96186093
ATP12A	0	0	0	0	0	0	0	0	0	0	0	0
ATP13A1	46.04495394	40.96894379	43.07910145	43.34499344	40.78519363	41.9276404	42.78566122	48.25497233	32.45160199	26.1377019	30.38230497	33.65436796
ATP13A2	9.150289186	12.73093074	9.332361689	14.51168608	8.664449393	11.28462335	9.61517026	15.71768108	9.015652449	7.244101405	9.095970327	15.74622502
ATP13A3	18.30567986	17.41226577	18.46563182	18.55671143	18.77214684	19.10527714	17.39980775	13.53863021	35.86997251	38.70817655	34.90540991	39.46539839
ATP13A4	0.016217469	0.033625149	0.02288639	0.016390949	0.005644645	0.033309499	0.027795443	0.0211585	0.017648661	0.035566229	0.034937736	0.028128842
ATP13A5	0	0	0	0	0	0	0	0	0	0	0	0
ATP1A1	35.87531967	37.02874863	39.41103519	47.82716034	43.9642066	43.99069542	39.42367043	47.36087416	76.40848053	83.07845898	84.50477608	77.31690404

ATP1A2	0.009848016	0.030628178	0.010423276	0.009953362	0.041132356	0.020227108	0.020254454	0.019272668	0.203624979	0.14038376	0.159118879	0.051243502
ATP1A3	0.391500443	0.435930889	0.276246312	0.644825497	0.378515216	0.31271076	0.283311288	0.595910264	5.096824853	4.881256514	6.2631752	5.236221476
ATP1A4	0	0	0	0	0	0	0	0	0	0.014363285	0	0
ATP1B1	0.122294457	0.456415541	0.491864929	0.568572217	0.280933993	0.427012634	0.276675839	0.358997239	4.924217071	4.773990288	5.45366939	5.2689873
ATP1B2	0.015583423	0.016155263	0	0	0	0	0	0	0	0	0	0
ATP1B3	44.56237223	39.9340207	48.69657948	53.12373085	43.74691702	40.14175641	44.57150054	48.76710336	54.26748335	54.27655943	59.21443421	57.327286
ATP1B4	0	0	0	0	0	0	0	0	0	0	0	0
ATP23	15.99476855	18.76530088	19.33259549	22.85174974	23.19638476	22.20557876	20.06957465	20.70693144	8.165922086	7.831161611	8.968997569	7.535020027
ATP2A1	0.539895259	0.684086219	0.571432588	0.469883006	0.375831429	0.354233709	0.508935491	0.631013355	1.958470136	1.989835367	1.809283949	3.542851181
ATP2A2	53.14852425	42.60445754	53.67941754	50.93725509	45.93796172	41.91357585	50.30359454	44.23261797	53.81167903	49.43242418	53.81658676	54.18693491
ATP2A3	17.60149112	23.31945069	18.99891331	31.85201137	22.9153322	25.74499289	20.24457361	33.96674706	8.21695827	8.643894915	8.097459372	14.93765008
ATP2B1	16.27686131	12.63853077	13.93401834	12.39558544	12.10283062	10.79041476	13.42531015	10.61799425	13.37859758	9.217175364	10.41875228	12.60337171
ATP2B2	0.021618948	0.011206131	0.011440896	0.016387656	0.033861068	0.011100936	0.033347831	0.010577125	0.088225578	0.148162852	0.168831801	0.044997106
ATP2B3	0.09087534	0.111874426	0.090172231	0.045923723	0.023722524	0.099158518	0.052566657	0.061133862	0.339951883	0.311401596	0.305898795	0.195056329
ATP2B4	24.6245098	24.36490338	19.96539766	21.08949531	21.05745155	22.88476838	18.3685462	16.88199271	56.15553037	43.06260953	45.6870783	36.76340264
ATP2C1	12.20917479	13.4597185	12.15645762	13.66645284	13.44265862	13.9641753	12.4428415	11.8232377	17.2057803	19.05301533	16.63170074	19.264672
ATP2C2	0.009572952	0	0	0	0	0	0	0	0.04167106	0	0.010311636	0.019924888
ATP4A	0	0	0	0	0	0	0	0	0	0	0	0
ATP4B	0	0	0	0	0	0	0	0	0	0	0	0
ATP5F1A	282.30724	274.8788755	299.2168006	285.2671109	280.2727289	288.7607927	304.2111399	293.9385064	243.8599282	247.041485	253.2280536	192.7111793
ATP5F1B	783.0269196	791.5412732	846.3807526	754.5757712	788.2614571	786.0298	886.6837644	794.3649875	423.888067	489.0671853	490.6459787	368.0188078
ATP5F1C	172.5206023	178.898823	186.9642412	166.120285	181.9131359	183.7622063	203.1469962	183.2985673	192.5339627	198.1220134	190.2269542	167.4243057
ATP5F1D	82.22696028	94.70447204	104.3521928	86.51502396	87.15426921	85.6664436	98.07329754	114.3320729	40.37011639	35.8376799	46.47620478	41.85724144
ATP5F1E	411.60024	383.8811655	441.0921559	435.3291172	319.8423913	347.6751402	423.3959656	444.0117099	273.3163297	302.683032	305.7842954	266.0904396
ATP5IF1	21.95050809	23.14918178	23.20763115	25.08426058	20.12328955	21.76337003	25.3761717	26.53522027	26.72526622	27.65667591	28.31697329	31.13242746
ATP5MC1	120.8428005	125.2771697	148.9398856	146.6802461	117.0630673	143.4892896	139.663569	155.3546216	74.57805845	93.29439715	95.49795379	66.55819661
ATP5MC2	54.11356968	58.13353001	54.97830121	57.44381059	57.32669516	57.020165	56.54304905	60.13034727	34.94848213	40.0181183	36.61679276	32.24179024
ATP5MC3	52.78489567	54.16037278	63.67680391	61.53098917	50.14931619	54.53888566	62.78644865	61.34725028	45.7187698	54.52607717	55.4861902	39.45515451
ATP5MD	120.8006584	121.5519788	148.3065714	114.4278547	119.2869617	120.2254943	151.7693866	120.7954765	68.18696376	75.50447903	86.09971192	58.62971959
ATP5ME	200.495898	206.0191673	232.9602808	208.1536453	170.5615695	190.9135295	218.6118139	228.4992835	139.0959147	159.2309828	176.1082996	123.1975774
ATP5MF	54.3360732	53.92828986	58.73603864	54.7044553	40.13342759	48.77195071	53.27769588	62.4416332	51.45334133	53.92376286	71.91334661	48.76616435
ATP5MF-PTCl	0	0	0	0	0	0	0	0	0	0	0	0
ATP5MG	164.9677269	148.2156597	170.6455159	169.1712046	141.3488522	148.7654497	172.3330677	171.8500284	161.2321517	171.4125584	181.3360923	139.2857514
ATP5MGL	0.202038401	0.069817424	0.142560153	0.204199632	0	0.138324051	0.138511059	0.263594134	0.513026032	0.221543333	0.435256849	0.420517388
ATP5MPL	78.40654797	83.64589801	87.37706838	92.17725101	76.92893928	79.56066022	91.0837291	93.01445644	58.43109472	63.1686353	67.59052088	53.87081908
ATP5PB	122.7753118	125.2242807	130.5661352	121.7497843	132.129258	124.7799958	142.9143159	123.8189846	139.0066754	141.906078	153.9162632	105.6992448
ATP5PD	142.9154253	143.1373926	144.6710522	181.5170579	139.2023419	148.1904259	148.5686983	183.7773431	129.6378135	132.7124111	150.8681643	166.2861192
ATP5PF	34.40687734	33.4747346	41.81178215	36.78996699	35.99131448	36.68551451	44.09058831	37.2472205	34.22235802	33.42379189	35.0249867	28.02083937
ATP5PO	244.6131898	256.9114885	260.7391601	257.0137806	246.8454764	253.8141658	277.9802664	275.4153174	195.7269783	202.4157507	217.0327812	166.8850558
ATP5S	2.517528703	1.778498437	1.963379967	2.45283031	2.395730527	2.141378578	2.043872842	1.787852154	1.259806982	0.902347871	1.18687781	1.43698574
ATP6AP1	72.43678623	74.19039452	72.00475269	70.27965849	46.82612808	48.45090064	60.51653139	72.33107989	53.28872603	57.23448229	54.29651283	62.5816636
ATP6AP1L	0.444063265	0.444205399	0.272106817	0.204721932	0.390468559	0.408032921	0.288412631	0.289678772	1.220846825	1.845221494	1.216801365	1.078305132
ATP6AP2	69.5007141	59.56331109	72.35827099	62.28797169	40.8505885	42.04894982	61.25714461	54.90172791	57.49460684	59.9892289	55.03089586	69.66186041
ATP6V0A1	23.23747605	19.90463033	19.24144612	13.37526031	13.61926778	10.43475937	16.88426131	12.43346557	22.02952355	19.07373585	22.03462849	24.63811493
ATP6V0A2	14.94037034	12.90159561	14.86430837	16.42247625	17.91096507	16.75313317	15.33073989	14.53230304	3.664834638	2.603524847	3.218644808	4.336699955
ATP6V0A4	0	0	0	0	0	0	0	0	0	0	0	0
ATP6V0B	53.24622794	48.07526066	58.40760528	58.64397368	41.15188895	43.20405199	51.81299639	64.66961423	83.66866545	71.43285018	86.67556272	96.14551308
ATP6V0C	60.93462863	64.84734212	64.67911313	65.38590538	47.83409014	43.13996806	63.38146768	72.92708594	68.69059561	62.50341523	71.66345489	87.34190558
ATP6V0D1	32.26976139	25.8632964	31.5621549	32.31894482	17.90415553	19.46830604	28.14673557	31.49796652	35.32039355	23.96961472	33.18243005	51.62095425
ATP6V0D2	0	0	0	0	0	0	0	0	0.247081253	0.771787175	0.122282075	1.063270231
ATP6V0E1	89.86562715	85.88983817	98.21449021	92.43012214	66.76163828	66.85137108	91.09599066	94.56036107	87.83738559	75.99656379	78.33373102	112.4422334
ATP6V0E2	0.019624202	0.040688637	0.124623154	0	0.020491174	0	0.020180586	0.019202381	5.70206223	6.778401867	6.743174131	8.965542155
ATP6V1A	20.51394319	18.98336212	18.68666262	18.51913862	13.67664726	13.89632694	17.07161613	14.46723178	25.01443509	23.21378281	21.15040613	30.3342609
ATP6V1B1	0.022309105	0	0	0	0	0	0	0	2.403510551	1.957027342	1.537955653	2.600280543
ATP6V1B2	84.30282383	78.70985634	79.19879213	71.14380465	61.05976671	57.05769424	70.95044686	65.07237725	92.18500915	101.3192722	85.171715	104.492032
ATP6V1C1	20.07481513	17.92641919	19.39030528	18.91658038	17.07077876	15.9174147	18.50973007	16.12634393	22.69851093	18.9922916	22.41646534	23.58158398
ATP6V1C2	0	0.081058009	0.099307378	0	0.048985875	0.016059418	0.03216226	0.076508178	1.174228346	0.771635236	0.960132842	0.634686735
ATP6V1D	11.47103042	9.513571142	11.32117856	12.49714561	9.271132886	9.087683386	11.06090565	11.04953479	21.85396748	18.29593003	18.77497045	25.42585662
ATP6V1E1	77.84398776	72.40826607	85.46967891	70.70845533	60.5426742	55.29976188	77.92576984	74.71023614	74.884599	75.87475734	77.08989036	84.67543309
ATP6V1E2	2.384340498	2.181928174	1.760302048	2.975118632	2.458940859	3.038116021	2.17950333	2.246678561	2.546707569	1.807573831	2.171978501	2.481351644
ATP6V1F	108.6070574	114.7773667	116.8333678	93.32681319	77.0935144	91.24401298	95.43115244	117.7440637	65.43903145	83.77610067	71.37022507	86.91138767
ATP6V1G1	49.2335771	45.04974694	45.74605773	47.97102705	45.23543006	45.96462724	49.66769258	50.75729217	53.57844253	52.66808834	56.05483197	60.17013897
ATP6V1G2	0.268544284	0.556797222	0.42634646	0.237489819	0.350510285	0.310258345	0.724914863	0.229925451	3.762632862	4.564280985	4.628258308	2.026161031
ATP6V1G3	0	0	0	0	0	0	0	0	0	0	0	0
ATP6V1H	22.69340766	21.30926303	22.67949361	19.76038567	13.89859982	15.66385261	21.0480079	17.97800716	19.13947899	18.97423717	18.87398542	24.8202743
ATP7A	1.419527634	1.373509855	1.14853774	1.345441502	1.370249046	1.237512907	1.141867692	0.938356795	2.437352709	2.51126602	1.875651332	2.777293259
ATP7B	0.103554604	0.071569718	0.109603621	0.047970239	0.049559361	0.022155584	0.044371075	0.021110147	1.122237466	1.220682269	1.013202742	1.023795119
ATP8A1	0.025730812	0.093362547	0.088510001	0.182042405	0.053735133	0.11891073	0.052920663	0.075533191	2.170120704	2.842649475	1.669906824	2.476938741
ATP8A2	0.053326813	0.055283659	0.050500588	0.011346791	0.026376027	0.043235285	0.049066235	0.008239035	0	0	0	0.002920869
ATP8B1	1.347659968	1.405478771	1.323887388	0.970581143	1.365066193	1.259686518	1.344375704	0.892288677	0.070255857	0.088488864	0.095617687	0.058787086
ATP8B2	3.696579383	4.991630119	3.930582241	2.830134111	3.966861081	4.690519647	4.609069195	3.216147454	26.38512614	29.99365626	26.50255909	29.86074869
ATP8B3	3.941178665	5.885623075	3.432162789	6.35317193	6.146896197	7.439104936	3.909271085	6.580395641	6.580067454	8.041575217	6.169111422	7.299689576
ATP8B4	24.73496529	23.22495916	20.61017417	14.93881764	19.52450102	19.61208244	19.20949435	13.27438121	22.00854018	17.824344	18.53815647	18.18540839
ATP9A	0.103999924	0.122191737	0.088059963	0.021022485	0.159271946	0.163766338	0.163987741	0.061058672	3.448154037	3.573258078	2.733408522	4.083929486
ATP9B	1.615565331	1.498126387	1.377790663	1.390073789	1.51298601	1.253297174	1.219134666	1.300306315	2.54234495	2.10714736	2.353690033	2.624759388
ATPAF1	5.075901884	5.184397927	5.213614274	7.55630363	5.378476077	6.137197768	5.116960227	6.703950911	36.21795546	37.17933625	40.26614636	33.85435288
ATPAF2	3.61102177	4.241437352	3.972574167	4.314856352	3.95629359	4.85926677	3.896327523	4.891065408	4.161991824	4.193699334	3.65973071	4.22074345
ATR	9.123578201	7.046486488	8.339095627	8.714009696	9.485817766	9.403045208	8.631669641	7.071829661	6.836642825	6.888726762	6.535345296	6.680261034
ATRAID	33.67908852	36.94999763	35.27976295	40.22479566	34.08183493	36.87983394	30.27997384	43.01119038	50.34837267	62.38087646	49.91837711	58.64262368
ATRIP	6.455081677	6.888250472	7.324062087	9.46434156	8.501713292	8.980266459	8.001118324	10.89777499	8.467156811	7.00275844	7.694851893	8.527023623
ATRN	14.12370198	13.74417602	12.67257826	10.52104525	8.763635474	9.478678786	11.63126341	8.616243942	11.80905875	9.540888788	10.6557377	9.115396473
ATRNL1	0	0	0	0	0	0	0	0	0	0	0	0.0022485
ATRX	11.4796817	9.788625141	9.474158886	8.050128226	10.28479524	9.368597537	9.971759168	5.754666004	17.67834473	13.9335631	13.97665178	13.2382939
ATXN1	3.541434054	3.047252229	2.987932678	2.311216266	1.790835346	1.750919005	2.377601704	1.554439591	3.352950221	2.452043387	2.326969487	4.101461282
ATXN10	55.70016705	58.29691997	62.40861831	57.32490744	58.19467522	60.18909406	65.10606239	56.95370806	36.68243971	36.25313811	37.89268134	40.20821835
ATXN1L	5.506507179	5.574167879	5.272068267	6.86193796	5.521781504	5.486804344	5.662627502	5.962346563	15.37841116	14.55281614	13.28870913	14.8127952
ATXN2	12.40199424	11.43241682	11.54077712	12.1589617	14.19666442	12.58601192	13.2517128	12.12439212	12.46676731	11.15364246	11.07788056	13.19965671
ATXN2L	30.67029991	32.57053687	30.57561176	35.83068444	32.62556096	33.94739709	28.93863747	37.23019558	36.17140202	38.35914721	36.90723865	40.91230091
ATXN3	4.298235903	4.310920859	3.471626998	3.888582761	4.350155145	3.991077464	3.50091052	3.224735174	4.389966115	4.542731695	3.382425	3.542903032
ATXN3L	0	0	0	0	0	0	0	0	0	0	0	0
ATXN7	6.155996798	5.203355492	5.73614247	5.650970216	5.464886606	5.330732517	5.396759663	3.742933385	9.49256891	9.000401508	7.216332225	8.690767186
ATXN7L1	1.327611642	1.1063313	1.129508589	1.152418577	1.164063648	1.066590128	1.009241345	1.025585311	2.402195057	1.636882065	1.806385287	2.131937773
ATXN7L2	1.233230053	0.891419943	1.209468232	1.212116878	1.039621169	1.208386815	1.396177491	1.450280406	2.203937766	1.253036545	1.84024753	1.813252822
ATXN7L3	20.52988287	19.76461017	19.99491677	16.86718881	17.1313651	17.39495379	19.33773864	18.34729989	20.53494838	17.06236596	19.64783703	20.91348269
ATXN7L3B	36.28711016	37.83078541	35.73083238	35.15391126	33.26649131	34.84920825	34.38535147	31.53169662	40.93670659	42.14598168	35.10445387	47.19298835
AUH	2.841338266	3.070946951	2.925875702	2.577338191	3.041466987	2.940526904	2.972760525	2.635066375	3.755958067	3.72430838	3.178984257	3.282950458
AUNIP	4.82677124	4.957558988	5.723659915	10.02783009	7.070016605	7.389479925	5.790889662	7.871700248	3.112731272	2.915913849	3.899416941	4.372007052
AUP1	64.61704677	59.9350273	67.28903925	58.69518499	66.31605815	63.61805968	67.58723772	70.14019853	112.5112066	102.945321	114.2403088	107.8986647
AURKA	16.78350029	15.00261718	19.18350489	28.71765216	26.83108521	22.29267433	21.41538147	30.35875631	38.29621666	42.12939421	41.2898722	27.4760094
AURKAIP1	36.19292756	33.47047302	40.37480611	37.58465473	39.18760796	37.97836611	38.27642823	47.95776274	28.34667221	25.78152917	33.66947372	28.14212177
AURKB	17.20689912	17.2079833	19.46691112	42.34054369	36.37857132	36.12223196	18.53884438	50.09465045	15.7147865	23.06837686	21.97305263	14.8697641
AURKC	0.537682007	0.600290318	0.39398542	0.292618125	0.345499146	0.339802974	0.425327962	0.607066814	1.575356647	1.723417544	1.247446351	1.463460846
AUTS2	0.105787487	0.147270332	0.095971659	0.223002713	0.148333401	0.15519985	0.071488449	0.150834092	0	0.013257176	0.003255727	0.009436426
AVEN	4.653470331	4.023703642	5.269422741	5.093475005	5.453174943	4.611994465	5.140653813	4.831820839	2.012387233	1.515218072	2.363996977	1.649514392
AVIL	0.48377204	0.376143169	0.373355937	0.193541525	0.126286118	0.103503387	0.279836961	0.157791132	1.085833948	0.707300985	0.966209332	0.388080026
AVL9	8.864012683	9.152227206	8.410828103	7.97745471	9.380016864	9.280429054	9.605396129	6.819874476	12.90706186	12.91394311	12.31357349	10.65671506
AVP	0	0.025958177	0	0	0	0	0.025749264	0	0	0	0.053942954	0
AVPI1	1.750183421	2.632669009	1.852418269	2.427909312	1.827504292	1.72688933	2.470319995	2.484895852	1.456487261	1.467583291	0.813238189	1.642824838
AVPR1A	0	0	0	0	0	0	0	0	0	0	0	0
AVPR1B	0	0	0	0	0	0	0	0	0.374732874	0.352415205	0.432734562	0.167232206
AVPR2	0.058078317	0.120419043	0.076838615	0.088049383	0.060644143	0.014911079	0.119449904	0.099452374	0.015800933	0.01592131	0	0.015110334
AWAT1	0	0	0	0	0	0	0	0	0	0	0.020955063	0
AWAT2	0	0	0	0	0	0	0	0	0	0.027367521	0	0
AXDND1	0	0	0	0.024873162	0	0	0.050615292	0.024080919	0.013390866	0.013492882	0.013254449	0.025611205
AXIN1	5.284064915	6.316746656	5.296703812	8.672147731	6.560515378	6.842161846	5.403062614	8.454572675	5.946049121	5.531317811	5.315218416	8.274572763
AXIN2	0.040216413	0	0	0	0	0	0	0	0.120355162	0.066148402	0.075809403	0.094168552
AXL	0.153741602	0.102460632	0.03486905	0	0.252267259	0.078943512	0.203272045	0.021490991	1.469931759	0.698419125	1.159234073	0.297137014
AZGP1	0	0.04409812	0	0	0	0	0	0	0	0	0	0
AZI2	11.2662497	13.39437339	10.69030538	14.78498285	11.73566542	13.51925051	10.66550855	14.00894238	14.73915314	12.79989073	13.3038831	18.53931798
AZIN1	60.1220854	56.26028295	59.33360451	74.21569845	59.17506566	56.17965201	62.67443506	58.73757401	42.06351492	43.4945793	41.83797301	53.16770041
AZIN2	0.239097675	0.287009045	0.133191724	0.228936631	0.118260334	0.1033872	0.258817436	0.123135941	0.547785379	0.620953425	0.460874164	0.720285181
AZU1	1298.932319	1367.744021	1281.9339	1517.689814	977.3320267	1176.963492	901.9391252	1620.65484	1919.409537	1563.294546	1406.044857	3056.942067
B2M	291.4906727	293.927529	276.8957578	299.712844	216.8866106	227.4360057	279.4498554	270.2625243	401.5618397	423.0739182	316.2468938	470.8249008
B3GALNT1	0	0	0	0	0	0	0	0	0	0	0	0
B3GALNT2	19.91128485	18.0023909	19.86786371	18.70304855	18.55806431	19.24332035	20.47580899	18.13989861	20.12006296	16.13108611	18.30180901	19.66499138
B3GALT1	0	0	0	0.007423583	0	0	0	0.007187132	0	0	0	0.007643857
B3GALT2	0	0	0	0	0	0	0	0	0	0	0	0
B3GALT4	1.404282727	0.99260003	1.283633268	1.516075281	1.333020134	0.950506108	1.509737676	1.03057333	5.10561316	2.449766432	3.644092875	6.244242575
B3GALT5	0	0	0	0	0	0	0	0	0	0	0	0
B3GALT6	18.03284961	15.5788095	18.35213146	14.70132556	14.32330849	13.98823638	15.77042378	17.41901388	11.80390216	9.696744657	11.99493903	14.67640313
B3GAT1	0	0	0	0.015319766	0	0	0.015587371	0	0	0.008310475	0	0
B3GAT2	0	0	0	0	0	0	0	0	0	0.091954869	0	0.058180674
B3GAT3	13.79435102	13.69967659	14.72282153	13.70759312	13.70173547	12.45205508	13.32716883	15.51684747	17.28238091	14.49051847	19.15408303	20.99053942
B3GLCT	1.410308115	1.105459902	1.371332684	2.004822963	1.81981534	1.118632765	0.966862085	1.783894226	2.657768752	1.458450335	2.531887758	2.648999689
B3GNT2	17.12294394	17.8297351	15.1994237	19.54347479	15.64695658	17.23488986	16.03241151	16.82894228	40.00657295	41.24497054	36.64386188	47.72901926
B3GNT3	0	0	0	0	0	0	0	0	0	0	0	0.020172514
B3GNT4	0.092171224	0.038221392	0.117066354	0	0.019248646	0.075725192	0.018956892	0.054114006	0.020061068	0.0404278	0.059570096	0
B3GNT5	3.335606293	5.479611954	2.979546052	6.542520964	3.490624002	4.637662463	3.136162103	4.648378413	5.026117432	3.947022862	4.31159267	3.93670733

B3GNT6	0	0	0	0	0	0	0	0	0	0.023048584	0	0
B3GNT7	0.202237276	0.599024126	0.198761384	0.642401992	0.301674087	0.445050673	0.222826179	0.650210659	13.26796482	16.23610301	17.50521251	12.56780656
B3GNT8	0.54980105	0.404499234	0.506830948	0.250953316	0.092595252	0.036427461	0.364767092	0.173542941	1.389648816	0.680670106	1.241763592	2.510168175
B3GNT9	0.755919894	0.4949423	0.884294571	0.844427756	1.287830505	0.61287015	0.715981839	1.070577101	1.298889251	0.741644631	0.664256123	1.242119752
B3GNTL1	6.241234332	6.583411006	5.513592142	4.663305071	5.532684947	5.553558901	5.75493909	5.019650368	4.222068691	4.460961365	4.948602027	3.934276315
B4GALNT1	0.031108288	0.024187363	0.074082242	0.023580793	0.016241306	0.015973539	0	0	2.208947116	3.402622542	3.325740187	3.099808969
B4GALNT2	0	0	0	0	0	0	0	0	0	0	0	0
B4GALNT3	0.031302828	0.08112874	0.082828361	0	0	0.016073432	0.048285487	0	0.834599941	0.652171677	0.724942774	1.009869613
B4GALNT4	0.032770744	0	0.011561669	0	0	0	0	0.01068878	0	0.011978152	0	0
B4GALT1	10.12373257	12.03629775	9.209559063	10.5299219	9.139219687	10.62043107	8.68441906	9.843426705	22.46662351	23.56521452	22.61044787	27.83274634
B4GALT2	11.82817463	13.40340509	14.1867939	13.15453537	16.45261252	18.81914534	13.62847697	15.84023114	8.949336519	14.55583242	14.6938615	8.423420403
B4GALT3	18.26795599	17.74581842	19.37632668	18.48307542	18.19963695	17.4591014	19.52770055	20.96578559	16.82489053	17.1027172	16.50648517	14.99390806
B4GALT4	3.056744802	2.831395757	2.622699509	3.948637451	3.569514266	2.934841161	3.403810327	3.132628004	3.405236461	5.335185445	3.506926608	3.802278173
B4GALT5	13.93039393	17.47383338	13.5877194	22.52245349	10.77864348	13.72161133	12.33571636	18.99870891	18.04555426	20.54170901	18.9037536	30.65252754
B4GALT6	0.884887062	0.755728509	1.159571115	1.08174102	0.844781937	0.701033225	0.71931385	0.750417616	0.953807163	0.979555784	0.826079097	1.438342899
B4GALT7	8.72803889	7.415746337	7.797106964	7.74233303	6.131495882	5.975585301	6.477727986	8.435960242	6.564566516	5.941412695	6.440189295	10.11091229
B4GAT1	2.313551167	3.449452936	2.338640778	3.021398255	2.877198384	2.963241981	2.619732586	4.069791561	7.892634648	9.634530691	7.280213986	12.22777888
B9D1	1.486201238	1.481931127	1.464946093	1.559431358	1.457089655	1.969010647	1.271670525	1.543064946	0.592619932	0.733978091	0.611023652	0.484072286
B9D2	1.27707104	1.288151624	1.534327655	2.023309672	1.477651536	1.098828875	1.774700702	2.060185136	2.967352346	1.968074064	2.156368706	2.729901419
BAALC	0.056661524	0.039160493	0	0	0	0	0	0	0.226093657	0.434921678	0.651026507	0.37345703
BAAT	0.061008572	0.063247304	0.048429236	0.030830594	0	0	0.047053714	0.059697198	0.082990727	0.033449192	0.065716217	0.031745408
BABAM1	63.07716213	62.93402248	70.18346748	68.79832743	61.85048795	65.73707421	72.10563932	75.1481714	37.33186615	44.50930775	46.39257856	37.3076776
BABAM2	13.48715091	14.46255007	11.62601996	13.23325593	12.94570942	13.18445049	10.72386974	13.71878623	17.55234579	22.38710327	18.17231708	20.7403329
BACE1	17.17079176	21.2109393	18.41031768	23.22662267	19.44137899	21.99402969	19.12831966	23.17778406	28.45230929	29.42377605	22.88620757	48.20370329
BACE2	0	0.023796149	0.006073668	0.011599695	0	0.005893192	0.011802318	0	0.087428346	0.02516983	0.043268841	0.083607186
BACH1	9.040877635	9.382046109	8.118269265	8.834837611	7.847953655	7.821107412	7.719666639	6.315153853	11.43901175	10.18243505	10.09049902	11.72311631
BACH2	0	0	0.003087374	0.002948185	0	0	0.002999684	0	0.085708947	0.054376016	0.050273067	0.051606293
BAD	5.5392195	7.155316263	5.53707596	7.464630984	6.610211604	6.004608651	6.871687514	8.130231579	11.8647449	14.12441314	14.15894558	16.88202097
BAG1	27.91205984	27.55358244	30.99831216	30.23270539	27.43331686	28.36503786	31.53233037	31.0506812	18.24937162	11.16710725	20.63753921	17.79877213
BAG2	14.34595208	13.24809442	16.68680217	15.12623585	19.37652109	17.83352378	17.82406681	15.7304017	5.346082917	4.921505291	5.572902657	3.651732863
BAG3	7.995041715	7.550709588	9.126622286	8.884391603	8.107857757	7.759247586	9.405471942	8.970043799	10.90992032	12.76018364	13.70700732	12.45872039
BAG4	11.36702865	11.24679873	11.54620678	12.91404093	13.41736724	13.54277367	11.94961959	12.25501625	11.47815299	10.94435976	11.14048874	12.61982353
BAG5	10.42598648	9.269040396	10.53637294	11.88316969	12.02016781	10.37053905	11.49042028	10.30209561	12.28229906	11.17422011	12.21233459	13.4827954
BAG6	67.78109141	66.2498544	66.73608116	73.61855397	75.22337082	72.97705126	64.70918405	82.70458977	67.01808838	67.43523884	71.9789611	62.67044784
BAGE2	0	0	0	0	0	0	0	0	0	0	0	0
BAHCC1	14.87561411	17.56391368	14.1195437	17.43872254	20.50003849	22.55968177	14.48538362	19.26315014	28.46758747	22.15870798	24.14020955	36.45508025
BAHD1	12.76388205	12.33180486	13.96912707	14.4527515	13.72594825	13.26033445	12.23255778	15.35019426	8.887483503	6.469574117	7.587506483	9.226590222
BAIAP2	1.408273079	1.249564326	1.490535648	1.373613973	1.072365185	0.846274451	1.32172001	1.365967398	1.927403204	1.294724557	1.742163207	2.067159351
BAIAP2L1	0.117296045	0.182400398	0.217258566	0.14818847	0.153097544	0.180688148	0.150777024	0.215202704	0.957355701	0.964649172	0.742288848	0.930771888
BAIAP2L2	0.152391846	0.157983919	0.145164268	0.0308044	0.222774053	0.06260035	0.047013737	0.074558097	0.24876065	0.467890819	0.27905663	0.15859218
BAIAP3	0.019303878	0.020012241	0.020431491	0	0.050391748	0.01982438	0.059553544	0.009444471	1.144905833	0.624440912	0.84213417	0.984375262
BAK1	24.70061766	21.0852857	25.89660494	25.81350655	18.65586812	18.85131378	25.5688292	25.83576717	35.02834623	33.19795314	35.80209264	42.52712213
BAMBI	2.496963381	1.94144275	2.180326894	2.271306345	1.825093227	1.698842404	2.56775721	2.595997046	1.664358814	0.924082429	1.445680544	2.371183071
BANF1	55.45009625	50.81797428	58.06405508	69.77219191	62.21660141	59.10173435	62.38702769	78.1125455	46.0253618	54.25524178	54.28608343	41.52113475
BANF2	0	0	0	0	0	0	0	0	0	0.057847426	0	0
BANK1	7.81558434	7.032254311	8.13165225	6.051076583	11.74856875	10.29792048	9.220000567	6.060353493	3.915652158	4.705473537	3.621613712	4.296983194
BANP	4.319594615	3.182045718	3.860122815	2.81467865	3.709174978	3.284991208	3.546557787	3.054055448	2.148668622	1.42760143	1.625268089	2.036813017
BAP1	22.18038663	24.57756661	24.6543605	29.3131731	25.66410272	24.85987738	23.27893269	30.34875683	41.68975118	41.06829663	40.52706919	48.02239786
BARD1	4.087887659	4.465100586	4.619155983	7.357936747	7.342988736	6.350989483	4.360571933	6.051310516	12.67189483	11.21156254	12.14249963	12.44530158
BARHL1	0	0	0	0	0	0	0	0	0	0	0	0
BARHL2	0.035968958	0	0.038070041	0	0	0	0	0	0.900294059	0.35497284	0.54242236	0.636351123
BARX1	100.9383125	124.1403734	113.4444065	130.2748641	117.8709561	119.2089649	107.7206203	146.6252599	45.47945606	54.10725016	50.49160555	36.99252494
BARX2	0	0	0	0	0	0	0	0	0	0	0	0
BASP1	3.453899475	12.36173863	2.582168893	7.341859235	0.601081391	2.589894136	2.621584622	4.479388197	119.9504593	142.8669339	121.7107196	170.3073999
BATF	0.914934085	0.889226169	0.907855175	0.751336017	2.68693511	3.817141134	4.05752028	5.76328546	7.529807763	8.026099805	8.130653436	9.938168019
BATF2	0.045912595	0.047597373	0	0.046403727	0	0	0.023607154	0.044925707	0.724483556	0.37758772	0.544009164	0.59725788
BATF3	8.040509094	8.378304868	9.20845736	6.376192181	9.213773875	9.146558659	9.837363178	6.536225585	1.884633561	1.763349118	2.043094535	2.14555168
BAX	88.16622895	85.33802836	86.52970879	59.28946722	39.08646861	42.22397357	92.71536583	66.13411287	35.4084147	30.92741361	37.38109874	42.83329621
BAZ1A	32.83081383	23.51880197	32.46940651	27.21383847	30.25848386	26.37332812	33.35507384	24.41278496	38.84050097	24.04633483	31.68548971	29.78962838
BAZ1B	26.05231355	24.97689062	27.69660705	32.09309734	34.34846012	30.56556977	31.90796347	30.01405635	33.60698251	28.49128786	34.69392298	33.85625181
BAZ2A	31.98501788	32.94933265	28.23589564	28.36920014	27.18243621	27.99033549	29.10130193	23.71605154	35.53425671	40.68354995	33.28673379	33.9695124
BAZ2B	5.001922558	4.251987548	4.690346454	3.921411754	6.177889634	4.536450822	4.445623703	2.855454935	7.926450601	4.761085402	7.058591912	3.915114883
BBC3	4.487760946	4.132993632	3.873711441	3.060541877	2.934452236	2.975563154	4.166939767	4.519198706	3.105721326	3.057716565	3.074082259	1.791057898
BBIP1	4.549751487	4.784735641	4.167276681	5.372193484	6.189683914	5.526045857	4.498794136	5.950208973	4.403771364	4.365364281	4.146853595	3.960897723
BBOF1	0.034805667	0.072165746	0.061397996	0.164163941	0.012114446	0.142976607	0.143169904	0.07946755	0.391398441	0.521599687	0.299931196	0.458809434
BBOX1	0	0	0	0	0	0	0	0	0	0	0	0
BBS1	2.235412024	2.350547561	1.825192991	2.598223188	1.950698944	2.672835236	2.34805015	2.624834409	7.193803687	7.581274212	5.950964233	8.408143268
BBS10	4.385261449	4.857562369	4.387096746	4.477707155	5.049441574	4.704002194	5.019237946	3.438678545	7.73040904	7.32820167	6.341330682	7.580090967
BBS12	0.918920272	0.90578921	0.924765206	1.06577878	0.786489343	0.74258175	0.975956208	0.972870456	1.13116454	0.825929092	1.038507568	1.222820299
BBS2	11.3629575	13.43906964	9.071791338	11.17897385	6.758755685	9.167465367	7.863226103	10.6837072	15.44265999	15.79429717	11.56935526	21.3188141
BBS4	4.509608084	3.981750164	4.591009004	5.098609556	5.686519038	4.552708234	4.696415172	4.711838756	6.52957838	5.322127516	5.145747839	6.025449697
BBS5	0.835181654	1.060198702	1.028289051	1.154199017	1.139041847	1.085254492	0.946499546	1.267539611	1.83632165	1.794241349	1.413700089	2.146299259
BBS7	3.644125748	3.985224146	3.755735376	4.307213935	4.023072861	4.10858428	4.525552763	3.523244274	5.262387413	5.874687991	5.236882551	5.032388517
BBS9	0.494886191	0.496496336	0.495633382	0.575475973	0.394507783	0.568343324	0.514389417	0.468627724	0.642797323	0.671034716	0.641980895	0.581475908
BBX	6.426453793	6.131944453	6.134715175	5.858142683	6.634057966	6.205719946	6.054412308	4.384392045	5.850358082	7.292281559	4.885491192	5.813314089
BCAM	0.031366692	0.048776556	0.06639788	0.031702226	0.016376216	0	0	0	1.860346843	1.840124761	2.196158984	1.403644797
BCAN	0	0	0.013247914	0	0	0.051417035	0	0.012247715	0	0	0.040447804	0.026052058
BCAP29	9.441957517	8.915969513	9.282467305	9.893638848	8.340529033	8.134788317	9.451541228	9.34735756	9.544136286	7.641669555	8.349538873	10.30244524
BCAP31	120.9592215	116.5273494	128.2863776	112.6971461	102.4541416	96.72886612	120.4625357	114.5794525	69.77622033	68.9779852	75.57277222	66.30587479
BCAR1	0.32912464	0.24508875	0.451383174	0.238942419	0.295261433	0.204703628	0.548203334	0.385552973	1.034152541	0.74212945	0.933738679	1.075788632
BCAR3	0.136832958	0.165496441	0.132757066	0.092197785	0.130971559	0.199073489	0.105534332	0.033472936	2.183990939	1.925550678	2.505655339	2.147870458
BCAS1	0.029420208	0	0	0	0	0	0.015127165	0	0	0	0	0.015308611
BCAS2	22.61322036	22.63021501	26.11791658	30.32056562	27.66251896	27.24883041	30.21368062	31.85825188	46.61309113	42.12658935	48.89407517	49.08492436
BCAS3	2.139787033	2.418622646	1.923926912	2.30733725	1.957832324	1.822661753	1.825125899	2.338885232	2.601204663	2.369905713	1.757583247	2.904584459
BCAS4	0.046886054	0.016202185	0.016541615	0.031591733	0	0.01605009	0	0.030585494	0.3061425	0.771187001	0.505039532	0.309026744
BCAT1	14.27617281	14.01596189	14.43507662	12.74716951	25.22899103	21.2485322	15.80344143	12.26914839	10.80671679	13.12154716	19.02845476	4.06313238
BCAT2	12.04079411	13.70838506	12.2078159	13.77920191	10.783834	14.05052915	12.15884855	14.09587364	8.035348279	10.71604108	10.57866071	10.04464553
BCCIP	35.8464522	30.23224858	39.57821505	39.27728156	42.71062902	42.80924347	46.31223804	41.55413436	16.73589084	17.35319874	18.49000686	16.63530882
BCDIN3D	1.821807231	2.077525	1.805520614	2.410422985	3.043667762	2.874428285	2.690968459	2.592941992	2.072699157	2.179293605	2.105103426	2.895603264
BCHE	0.043836709	0.068167969	0.162390822	0	0	0	0	0	0	0	0.023609655	0
BCKDHA	30.08967552	35.47039982	30.69157775	29.73709895	35.28302913	38.53280003	29.85104603	36.80362521	31.82083416	43.07252806	37.73718587	32.10308913
BCKDHB	5.435554288	4.949504372	5.265082375	5.628589039	5.770707278	5.887388201	5.408747525	5.419630616	4.87875445	5.614395936	4.541531459	5.448370879
BCKDK	24.04684525	26.15604046	23.63185946	18.7301618	18.79110361	22.6774247	21.42228698	22.98376788	18.36931217	24.92383946	21.91000499	18.28677734
BCL10	3.826230922	4.151745488	4.144229405	4.408563086	3.968633798	3.903203758	4.052753466	4.492783645	5.343666501	3.957866305	3.970356093	4.804835596
BCL11A	3.61744947	3.489018896	3.288629989	2.813926491	4.755305286	4.736610734	3.075652188	2.869679407	2.755682685	1.905423373	2.261411007	1.902483598
BCL11B	0.02006821	0.055478987	0.028320627	0.040565765	0.006984933	0.041218641	0.048153428	0.01309123	0.072797435	0.080687235	0.129700481	0.174039342
BCL2	6.561791619	4.987896758	6.712237846	4.427767704	11.05158523	10.49609812	5.931402761	3.902990154	17.6960336	13.26300442	18.47912461	9.864884677
BCL2A1	9.928805504	9.050870222	9.482064332	2.883625058	1.132077597	1.933823046	7.452350287	2.512599973	0.062097834	0.062570917	0.245860885	0.178151322
BCL2L1	5.497933392	5.474466601	6.774197279	6.13099882	4.86835926	4.942550331	6.04906183	6.704254849	8.838295942	8.997250871	9.054266131	12.39975617
BCL2L10	0.122387174	0.084585476	0.043178758	0	0.042598028	0.125687169	0.041952364	0	0.177583797	0	0	0.042455572
BCL2L11	10.6072235	8.218707868	9.794241138	7.105249183	8.032866231	6.779902257	8.365609312	6.703247548	9.634456565	6.375986037	7.341914978	10.54291383
BCL2L12	10.41475372	11.28769618	10.20891131	12.91847113	12.00766261	13.63280374	12.15020732	16.5601952	11.69877203	12.99913086	14.74538942	13.30179122
BCL2L13	13.18158584	11.61663361	12.0381695	11.3992852	11.75398481	11.74810891	12.75749586	11.36564163	12.97489129	12.27117633	12.85059534	13.82453773
BCL2L14	0	0	0	0	0	0	0	0	0	0	0	0
BCL2L15	0.16230513	0.078521788	0.057261996	0.109360886	0.101685338	0.144457261	0.13352544	0.105877596	0.117752377	0.10678451	0.116552792	0.090084695
BCL2L2	9.708668717	9.805683502	10.15123385	9.619246606	8.325107944	8.474881699	8.925027077	8.650740821	14.95172556	13.79134502	13.27826891	16.83947204
BCL2L2-PABI	0	0	0	0	0	0	0	0	0	0	0	0
BCL3	2.348272518	2.08072069	2.241148239	0.659275636	0.534413788	0.329790129	1.692459443	0.716833974	1.113939243	0.71527123	1.167449458	0.751943438
BCL6	1.033058368	1.02680323	0.710148485	0.462852499	0.405901663	0.442958649	0.492841673	0.411635956	1.014121226	1.232055714	1.078357277	0.85342211
BCL6B	0.015265124	0.015825283	0.048470452	0.04628525	0.047818552	0.031353452	0	0.014937001	0	0.033477659	0.016443036	0
BCL7A	7.63831197	9.216250611	8.716801579	9.991459735	11.62946427	10.97028929	10.18061772	11.27382288	5.35584376	5.225076766	5.469819774	5.684265703
BCL7B	20.5198489	20.48156787	23.1788188	24.47777637	20.66006671	19.65620239	21.46388672	26.36955166	26.38795436	20.08659552	24.85428209	29.23674096
BCL7C	3.020238811	3.754086082	3.343447929	4.9837497	3.459381932	3.861269155	3.89818193	5.051182986	5.399701825	6.623629652	6.572976704	5.580646276
BCL9	0.261826462	0.385261553	0.241363223	0.213409075	0.317489366	0.425013729	0.364789994	0.165289368	4.035014103	3.834219272	3.593607431	3.64770727
BCL9L	2.165791818	2.684259319	2.208268346	2.710565006	2.114086447	2.192479871	1.995858174	2.222826304	3.979407747	2.815029582	3.036111865	4.870467388
BCLAF1	64.26973718	59.96234496	66.44998075	72.89355241	73.50043431	68.77676833	70.31038621	64.82847864	47.55024292	41.69694972	44.41151099	44.80659556
BCLAF3	1.878674073	1.558090145	1.598379387	1.840346782	1.712690115	1.380212877	1.768466491	1.562548296	3.019520708	1.640110849	2.420584106	3.278571222
BCO1	0	0	0	0	0	0	0	0	0	0	0	0
BCO2	0.64464033	1.039570987	0.739154233	0.579143226	0.766608567	1.158538732	0.662917153	0.770958019	0.74050375	0.706874368	0.424345251	0.838585839
BCOR	22.61225233	23.18162424	21.16557582	28.14619617	23.83315552	24.3860336	21.0876331	23.73320504	27.74070394	20.40965193	23.20549357	36.14626708
BCORL1	3.071672615	3.432771046	2.711417948	3.129366934	2.758342666	3.091405948	2.943964863	3.005647239	1.691430419	2.229757725	1.568321015	2.026675526
BCR	2.975178549	3.317894924	3.239410399	2.779320006	2.465827455	2.991579611	2.947694088	2.956834	10.30496829	7.15514284	8.559957102	10.98635824
BCS1L	12.74915733	12.98486058	14.91585196	12.7723816	13.61927083	14.71695958	13.25453566	14.58397104	7.949911622	8.01047683	9.044739193	8.665629339
BDH1	4.643188536	5.469735145	4.835909752	5.447512753	5.447259011	6.109017796	4.957892729	6.382026508	2.017951332	2.873765705	2.732434661	2.660488026
BDH2	5.976974623	6.335544555	5.295453613	4.394932501	5.943003038	5.13810192	6.129168361	4.369946625	6.522713813	6.682866736	6.854129699	5.748404904
BDKRB1	0	0	0	0	0	0	0	0	0	0.045353093	0.044551655	0
BDKRB2	0	0	0	0	0	0	0	0	0.181381306	0.098410917	0.220964322	0.213481625
BDNF	0	0	0.005989355	0	0	0	0	0.011074336	0.067740123	0.049640866	0.012190915	0.029445209
BDP1	8.734602312	7.22205147	7.977724196	7.884734997	10.11559753	8.780039194	8.297845359	5.298432117	10.32397843	9.461254698	9.408596217	9.680137519
BEAN1	0	0	0	0	0	0	0	0	0.020470143	0	0	0
BECN1	39.79983219	38.63287626	39.31803437	33.56084325	31.90371209	32.48630534	41.38674789	36.09699229	45.99364194	38.85591159	39.71122392	46.47444592
BECN2	0	0	0	0	0	0	0	0	0	0	0	0
BEGAIN	0.072735281	0.13572779	0.069285622	0.058810672	0.182276719	0.194210857	0.134635445	0.163695235	0.973596326	0.829474849	1.190886585	0.984030393
BEND2	0	0	0	0	0	0	0.017769803	0	0	0	0.009306632	0
BEND3	2.468693779	2.022659437	2.486468919	1.963886509	3.725275386	3.541183744	2.53868611	2.072765863	1.109287687	0.873233304	1.321015593	1.707232967
BEND4	0	0	0	0	0	0	0	0	8.55159548	7.703756127	8.490662937	5.167280365
BEND5	0.097853357	0.213032652	0.051784671	0.019780021	0.051088197	0.090442649	0.060376615	0.038300002	0.606986844	0.954971689	0.737828561	0.274953677
BEND6	2.890856743	2.940920223	3.250359569	4.623877096	2.529581394	3.098285125	2.93577376	3.947868099	1.66609267	1.086273001	1.280492867	1.60264631

BEND7	0.008036075	0	0	0	0	0.008252757	0	0	0.865780682	1.145544902	0.891585346	1.062105758
BEST1	1.333230147	1.239872991	0.975325459	0.455769312	1.064570424	0.674521903	1.209732218	0.498807118	4.630038908	1.870424692	3.220681293	2.948384362
BEST2	0	0	0	0	0	0	0.026212775	0	0	0.027950912	0	0
BEST3	0	0.007534322	0.007692164	0	0.022766126	0.007463595	0.014947371	0	0.007909003	0	0	0.030253322
BEST4	0.07617435	0.047381757	0.080623983	0.015397839	0.031815854	0.015645656	0	0	0.215531524	0	0.180514932	0.079273639
BET1	11.38881968	13.57594159	12.16467173	12.46106776	11.38282717	11.83897203	11.35355872	13.25695089	10.23348182	13.29030568	11.36724674	10.83732735
BET1L	22.85968732	21.49530876	22.5316015	20.72159759	20.69941802	21.12845943	22.97520578	22.94500328	23.24449899	26.1436574	25.27768955	26.41036683
BEX1	299.073789	387.8355468	309.342137	357.478382	160.2822945	248.3515272	347.4175505	365.0277258	108.1493564	97.33668251	89.29598893	141.1016337
BEX2	0	0	0.048429236	0	0	0	0	0	0	0.100347575	0	0
BEX3	0.317929459	0.164797996	0.378563539	0.040166299	0	0	0	0	83.90178634	109.3367616	100.9834999	78.00142039
BEX4	0	0	0	0	0	0	0	0.041070927	1.507349831	1.656909108	1.311146183	1.441469315
BEX5	0	0	0	0	0	0	0	0	0	0.058018067	0	0
BFAR	18.19587188	17.45201573	18.32296185	20.10624866	19.38749213	18.61385804	19.4573216	20.40019799	18.89720948	21.63946164	20.49516699	20.40840579
BFSP1	0.574479297	0.411220013	0.680422146	0.912410048	0.671270853	0.519734867	0.450108127	0.883348569	0.565636443	0.20997998	0.265203525	0.313161102
BFSP2	0	0	0	0	0	0	0.024084948	0	0	0.012840996	0.025228163	0.01218692
BGLAP	0.07242202	0.225238715	0	0.439180366	0.075621532	0.223124328	0.074475327	0.141730628	0.472879599	0.079413694	0.390051842	0.376843195
BGN	0	0	0.023189243	0	0	0	0	0	0	0	0.023600042	0
BHLHA15	0	0	0	0	0	0	0	0	0	0	0	0
BHLHA9	0	0	0	0.07681521	0	0	0	0	0	0	0	0
BHLHB9	0	0	0	0	0	0	0	0	0	0	0	0.013080799
BHLHE22	0	0	0.016765611	0.048029292	0.04962037	0.065069719	0.130315379	0.061999327	17.20375053	14.69464254	14.57147334	11.6217909
BHLHE23	0	0	0	0	0	0	0	0	0	0	0	0
BHLHE40	33.8596504	45.66384874	32.6159832	39.97596801	17.80126885	24.84453208	23.74287391	31.52694389	14.38212761	13.03906748	12.48588868	13.39246819
BHLHE41	0	0	0	0.014387611	0	0	0	0.041788038	0.077458012	0.015609623	0.015333784	0
BHMG1	0.067198952	0.104497262	0.035562149	0.084897235	0.052625787	0.120769032	0.034552087	0	0.255952419	0.221059161	0.579074155	0.157349388
BHMT	0	0	0	0	0	0	0	0	0	0	0	0
BHMT2	0	0	0	0	0	0	0	0	0	0	0	0
BICC1	0	0	0	0.006441069	0	0	0	0	0.655386427	0.78616594	0.569766259	0.865500835
BICD1	1.721777217	1.526389694	1.481726069	1.95568676	1.634020983	1.784727729	1.414819633	1.574549292	1.71612117	1.945344579	1.343311015	2.29421326
BICD2	9.219322038	8.243454422	9.130566744	8.119921149	7.684105549	7.286907646	8.498777505	8.030437439	8.348829063	8.367302283	9.114981086	10.09128084
BICDL1	0.670630353	0.471348726	0.697773831	0.987985741	0.130556566	0.070038604	0.151955467	0.478258538	1.570954509	0.810157231	1.395782471	4.388591354
BICDL2	0.119488292	0.247745914	0.126468056	0.060383237	0.020794522	0.061355061	0.040958673	0.0389733	0	0	0	0
BICRA	1.646395258	1.923057774	1.718912685	2.093185224	1.711352028	1.683137323	1.769683481	2.0702522	1.783582522	1.347877858	1.749363339	1.75214608
BICRAL	7.176670376	6.181310409	6.040694539	6.706305642	7.307998385	6.790412773	5.749831327	5.887320816	8.559032399	7.309826198	7.34725821	7.702061826
BID	18.67151304	19.16792753	20.23322494	20.77268094	19.20067667	19.90207493	22.55012105	24.05011576	23.24718777	19.52024415	22.81424952	26.86262967
BIK	18.06826808	27.62865209	19.90060459	31.84357196	18.86650111	29.74671065	21.01923481	32.43155186	31.15323854	16.03581632	27.24747454	25.61964312
BIN1	0.228544619	0.263256827	0.30908777	0.269487622	0.119320714	0.143432054	0.221967403	0.136664042	4.186680484	4.5944888	4.499622582	4.67758617
BIN2	68.07173869	61.71204126	59.96134346	44.98134491	75.66926044	54.56461127	62.15009488	43.2413791	41.81448024	36.08037756	43.6314913	42.17116092
BIN3	12.19136788	13.26697561	13.0167006	13.43866703	12.45080102	13.3986935	12.04213491	14.8593226	14.12070591	10.92528826	12.99770566	16.61974606
BIRC2	12.17496512	11.41841459	11.36384921	12.43271732	12.64696991	11.14278883	12.46825236	10.7034206	21.22651006	18.77349027	17.34094581	18.65754637
BIRC3	0.030801122	0.039914226	0.040750416	0.023347954	0.016080938	0.007907908	0.055430191	0.030139055	0.368712552	0.320859506	0.199067099	0.176298766
BIRC5	18.63405163	20.41285638	21.97955486	45.76411224	34.9606747	34.24101747	31.72549625	49.31824286	51.422825	59.09178824	60.17283532	45.54577105
BIRC6	11.62325915	9.528061872	9.701607717	8.636473179	9.205423837	8.859769443	9.372594539	5.855382602	15.33782071	13.8764991	13.48097389	14.79844794
BIRC7	0.039334474	0	0	0	0.041072221	0	0	0	0.042805743	0	0	0
BIRC8	0	0	0	0	0	0	0	0	0	0	0	0
BIVM	8.338058079	7.380447504	6.890040467	7.699316018	7.968835533	7.510301705	8.104429993	6.952626057	6.360768206	7.517931867	5.908065547	7.019681785
BIVM-ERCC5	0	0	0	0	0	0	0	0	0	0	0	0
BLCAP	15.12342646	14.93540633	14.70648063	13.23042291	9.926996225	10.42423437	13.85259299	13.06662777	17.04699465	15.50889644	14.99851641	20.00069396
BLID	0	0	0	0	0	0	0	0	0	0	0	0
BLK	0	0	0	0	0	0	0	0	0	0	0	0.032642902
BLM	12.01891708	12.99834862	13.69693637	19.64593248	24.69031864	22.78119751	16.27248486	19.05129591	16.14763835	14.15547133	16.95354145	15.49703622
BLMH	27.46684325	29.86000681	29.36312099	35.67083979	34.15058868	36.52809973	31.32097007	38.51944618	35.38423653	41.16584257	38.77059957	41.58530761
BLNK	0.57989648	0.939337478	0.409180288	0.158735337	0.126149076	0.334987064	0.236050334	0.118214927	0.86772451	0.887582641	0.819844484	0.339463464
BLOC1S1	49.06165843	45.65032796	41.48290818	54.94972052	43.54962939	47.70755852	47.00617202	62.21790665	58.25432147	51.85851586	53.6496738	59.1960678
BLOC1S2	16.54172139	15.76427529	16.82952803	17.81645166	13.01481107	12.10536849	19.31786966	17.02247219	14.53298901	13.7065092	14.07805774	16.69589936
BLOC1S3	4.903062639	4.203647771	5.211365609	6.042796511	4.039573758	4.461628719	4.55275891	6.174218815	9.793480132	8.280121737	10.00569202	8.870260023
BLOC1S4	10.23286604	10.89702765	11.12531692	7.809513214	9.667327578	9.793897573	9.699760993	7.696999871	8.219367259	7.289673674	7.8357025	7.715242854
BLOC1S5	12.26016188	13.59074942	13.03762988	14.20610073	16.22905378	16.49684303	13.48137029	14.07478708	4.601456152	6.097330396	4.346607845	5.90731118
BLOC1S6	19.70544096	20.07654765	19.01975333	22.77593413	20.75983375	20.01722617	19.11319891	19.09729955	23.85301287	26.84774874	25.01875827	25.50639258
BLVRA	0	0	0.081071575	0.07741661	0.159962416	0	0.039384461	0.037475394	23.965123	25.86953352	26.56749976	27.26209737
BLVRB	19.36138405	31.80216284	18.56288082	24.71475536	14.11233844	22.46570045	21.07390739	27.98724578	16.41820291	25.57315757	18.01146399	22.56964965
BLZF1	9.035032812	8.679121373	8.451529549	10.22078136	8.323435895	7.760583013	9.291193785	8.083812872	7.71255093	6.165540529	6.011945643	6.929773874
BMF	9.640222708	10.61275986	6.412849799	5.19038026	6.467714367	5.493662164	6.161220032	5.554013369	7.083857803	3.556563376	4.694679639	8.438511336
BMI1	1.81979856	1.837785933	2.374416247	2.521061751	2.735625473	3.061074964	1.952056943	2.04164416	1.75845495	1.066551373	1.487064117	1.257118137
BMP1	6.340983002	6.541178316	6.280174681	6.736116786	6.097538426	6.157931124	6.123285904	7.727744868	5.672795627	5.326545151	5.603752363	6.446776744
BMP10	0.1750189	0.163297152	0.21302878	0.026533665	0.109650607	0.10784282	0.161982928	0.025688532	0.028569649	0	0.0188524	0.009106993
BMP15	0	0	0	0	0	0	0	0	0	0	0	0
BMP2	4.825360052	4.209790049	4.56773175	3.38297386	6.511068866	4.623941118	5.958096699	4.106496147	1.885993754	1.415955936	1.427538012	2.387070206
BMP2K	6.049631504	5.711770692	5.863541873	6.145008901	5.258800503	5.464977691	4.61126399	4.488679952	17.78274541	14.43837306	15.16363921	14.94061735
BMP3	0	0	0	0	0	0	0	0	0	0	0	0
BMP4	0	0	0	0	0	0	0	0.02043187	2.158725037	1.121930284	1.844338262	1.868802988
BMP5	0.004920406	0.015302886	0.026039128	0	0	0	0	0	0	0	0	0
BMP6	0	0.014742104	0	0.028744804	0	0.014603715	0	0.013914622	0.170227491	0.296269322	0.0612703	0.162787497
BMP7	0.013348936	0	0.014128698	0	0	0	0	0	0	0.029275304	0	0
BMP8A	0.00796235	0	0.042137305	0.040237621	0.024942349	0	0.090069081	0.023373599	0.381261218	0.532593494	0.823368386	0.480606116
BMP8B	3.300089962	3.894616022	4.459553252	3.187345349	4.885417399	4.69868751	3.925296937	4.097569762	20.00076258	29.38408394	26.79510624	37.90349806
BMPER	0	0.022176157	0	0.010810012	0.011168117	0	0.010998841	0.010465698	0.03491846	0	0.034562733	0.044523076
BMPR1A	3.786002037	4.075247214	3.973053306	4.608084645	5.458867112	5.195144907	4.638876349	3.381453032	1.472722633	1.810762985	1.726703447	1.500569295
BMPR1B	0.016857632	0.017476229	0.01784235	0	0	0.008656087	0	0	0	0	0	0
BMPR2	4.447402275	3.978316613	3.717692103	4.490477229	3.453858806	3.520356838	3.685348734	3.07980643	7.213803654	5.491844225	5.059121152	7.473006025
BMS1	25.47515134	21.92690018	25.99320293	26.19777343	27.59804971	27.056878	28.81915544	25.82437614	13.33838673	14.65731789	15.05182781	14.19955285
BMT2	4.149697235	5.023684222	3.23628184	3.973345651	4.689359906	4.149440826	3.719893325	3.245728458	3.669175417	3.487574638	2.99954022	3.878158139
BMX	0	0	0	0	0	0	0.020609001	0	0	0	0	0
BNC1	0	0	0	0	0	0	0	0	0	0	0	0
BNC2	0	0	0	0	0	0	0	0	0	0	0.002340847	0
BNIP1	6.549785149	5.664695613	7.391988427	7.131881558	7.405926383	7.172339276	7.107610669	7.966986465	4.568095373	5.713940757	5.028284985	3.125695093
BNIP2	20.1662738	18.77906598	20.69272805	21.14517919	19.31742589	19.36981906	20.76334853	18.73692523	25.85816873	24.34515446	23.83538027	28.01653267
BNIP3	19.70490961	43.41801511	20.47231024	9.391682514	45.3485613	44.53322932	16.53616774	13.80003265	45.68476393	99.25598061	70.88152003	15.29424916
BNIP3L	19.14250291	32.24995517	15.58393823	21.41537777	23.31697954	25.00349214	13.06757753	18.23773241	25.2692837	33.67265342	24.3250688	16.4493872
BNIPL	0.216023784	0.183232517	0.103928433	0.496215042	0.205061309	0.322688809	0.282734434	0.34589515	0.277831147	0.301482205	0.275000784	0.163500435
BOC	0.059122104	0.017511889	0.026818136	0	0	0	0	0	0.367655044	0.287103375	0.28202995	0.29005865
BOD1	7.119489487	6.190299463	6.830444535	6.684988714	7.386058036	7.995431265	6.967082597	7.550740041	9.897170208	11.40801604	11.42906833	10.92253383
BOD1L1	11.31754623	10.49999208	10.06946751	11.78707248	11.83841082	10.71957451	10.51825505	8.365601297	17.31353798	18.42619658	14.7635763	18.23123218
BOD1L2	0	0	0	0	0	0	0	0	0	0	0	0
BOK	0	0.019000041	0.019398086	0.018523559	0	0	0	0	0.199449104	0.160774862	0.217158971	0.17165875
BOLA1	6.933718369	7.086912012	5.891667031	6.958537906	8.004834288	7.371404183	7.280943142	6.498002044	10.04311305	12.85031746	12.67583537	11.63679477
BOLA2	0	0	0	0	0	0	0	0	0	0	0	0
BOLA2-SMG1I	1.227693864	1.1102665	1.714112835	0.897619288	3.163916816	6.035729698	1.531118723	1.635819132	2.131975378	0.744715404	1.885162206	1.603851957
BOLA2B	1.058201787	2.303769038	2.352032266	2.085566937	2.099408405	2.825510173	2.720509729	3.572322571	0.978849921	1.392433613	10.42968709	5.946785717
BOLA3	35.25788916	34.1350492	42.5603817	36.12588817	32.75872917	41.19597362	46.74523207	40.20250664	28.43352193	36.53158917	42.47734961	27.69620628
BOLL	0	0	0	0	0	0	0	0	0	0	0	0
BOP1	64.71780311	61.59927736	70.09392573	62.20569758	74.29066205	75.68428689	76.1286055	81.33326258	34.93710599	33.57438854	45.42363426	39.17846322
BORA	14.16306666	12.52047495	15.43546777	23.25692737	24.18543974	20.40093828	20.49770989	21.28172439	24.05300087	26.48649002	25.43864706	16.55984942
BORCS5	1.263102827	1.073321993	1.015448512	0.885956441	1.318905099	1.119952854	1.050488051	0.905014417	0.758643402	0.59791503	0.587349229	0.646472665
BORCS6	4.470073238	4.925557245	4.314604644	5.228250273	4.755622455	5.572240597	4.307701157	6.189607562	2.753523059	2.558705889	2.543773828	2.92575229
BORCS7	9.480206	7.857886547	8.116063701	8.688226113	8.745296378	7.920287491	9.044516146	8.757468571	6.661452651	4.652500975	5.379607814	4.760481339
BORCS8	5.305167972	3.849890089	5.080295046	4.876792114	3.956816805	3.346760167	4.338484993	4.301225131	1.951941902	1.662095052	1.904844721	2.287278967
BORCS8-MEF	0	0	0	0	0	0	0	0	0	0	0	0
BPGM	4.520756572	3.970631766	4.253183201	6.515220509	5.223121304	5.588377762	5.638978601	6.614895313	6.810159028	6.448941732	6.673148301	7.710466818
BPHL	3.832516524	5.386420929	3.865819763	4.289461194	3.142378317	3.830194308	3.623765774	3.926317791	1.06367771	0.846143036	0.942016216	1.284869572
BPI	2.729878349	13.85850304	2.651044736	13.88073341	2.997412898	11.24284434	2.749393801	11.51094354	0.061253407	0.092580085	0.060629397	0.351457524
BPIFA1	0	0	0	0	0	0	0	0	0	0	0	0
BPIFA2	0	0	0	0	0	0	0	0	0	0	0	0
BPIFA3	0	0	0	0	0	0	0	0	0	0	0	0
BPIFB1	0.027384	0	0	0	0	0	0	0	0	0	0	0
BPIFB2	0	0	0	0	0	0	0	0	0	0	0	0
BPIFB3	0	0	0	0	0	0	0	0	0	0	0	0
BPIFB4	0	0	0	0	0	0	0	0	0	0	0	0
BPIFB6	0	0	0	0	0	0	0	0	0	0	0	0
BPIFC	0	0.024065626	0	0	0	0	0	0	0	0	0	0
BPNT1	16.06656268	15.19121171	17.09480804	11.26835557	12.88105967	12.21883039	16.92895761	10.59142413	10.34918776	10.86188719	11.99417607	9.514518789
BPTF	9.535088813	9.329406088	8.530045771	11.14118906	10.93776567	10.76167055	9.063555422	9.382571921	16.48226741	15.11611337	13.69002776	14.20028769
BPY2	0	0	0	0	0	0	0	0	0	0	0	0
BPY2B	0	0	0	0	0	0	0	0	0	0	0	0
BPY2C	0	0	0	0	0	0	0	0	0	0	0	0
BRAF	4.628438217	4.004049847	4.068741242	4.283920189	4.643576197	4.301656589	4.274839844	3.304669891	7.306215561	6.357758618	5.659445201	6.524555779
BRAP	13.67954127	11.83973502	13.00938297	14.47635234	12.99253833	12.60985605	15.11854376	13.01505311	9.846686284	8.012085541	9.039524609	11.12360866
BRAT1	14.03727675	14.21834292	14.44207472	14.10248058	15.5790004	14.99125171	15.40049322	16.73762705	12.07449612	11.33695086	14.23011886	15.62895588
BRCA1	10.55031401	11.48203357	13.10078415	19.80089735	20.27143677	18.83551173	12.54608715	18.17116047	20.71640251	18.28624499	20.55317389	21.61266452
BRCA2	3.445210774	3.997878386	3.381352552	5.79866456	5.556502306	5.809482602	3.853924831	4.189667404	7.467643532	5.544939448	6.806147707	6.088761353
BRCC3	14.43671145	13.58068726	15.12567092	15.40176184	14.73613418	12.79593274	14.16287043	13.24646842	10.20052746	7.648715193	10.01807224	9.400598043
BRD1	4.093299656	3.911483462	4.08629821	4.345492477	4.484865541	4.446968822	4.367259886	4.580568652	7.008848053	5.402520374	5.893048876	6.907976677
BRD2	89.95591357	77.89499351	87.2641661	89.20086217	91.12432148	80.63339906	81.54495916	88.62163519	110.5586774	88.25199886	94.01352154	102.5395662
BRD3	10.75515324	11.97313461	11.45494	12.20229328	13.69161744	13.38779833	11.41629936	12.86355102	18.55402147	16.50899593	17.70066158	20.89078091
BRD3OS	3.179120664	4.467612372	3.863317769	4.069962289	3.417927794	4.087097373	4.237923142	4.401185694	12.24982358	11.87834232	10.55232767	16.37079406
BRD4	13.62587459	11.80306662	13.48816143	13.04649673	14.02520744	12.63441304	12.92968185	14.06955298	11.61565427	9.273037193	11.57967151	10.89377486
BRD7	14.36591195	15.23530804	16.08461245	21.3997067	18.66157687	16.95106091	17.31345743	19.77130268	27.27846035	24.11602523	24.22938998	30.16145472
BRD8	14.24606691	12.24993173	13.13735083	18.61490222	14.77970102	13.45318512	15.6046352	18.60515614	29.67201141	18.83867032	22.97480948	25.17863162

BRD9	13.57891289	12.84313201	13.34606837	14.07717836	14.31278497	14.47210579	13.71467732	14.78662181	20.8898379	20.33771123	21.58303735	22.97371701
BRDT	0	0	0	0	0	0	0	0	0	0	0	0
BRF1	4.036744749	4.346814678	4.368265273	4.678204724	4.189328912	4.230021351	3.999012132	4.850987896	6.513431802	5.652519896	6.881015087	8.188074284
BRF2	10.0354831	8.53497062	10.79255206	10.2244117	9.945063358	10.38896621	11.5373825	10.39895334	10.86255669	7.405048981	9.5057194	10.38779573
BRI3	5.303149263	6.028421066	4.96278041	5.846198679	2.779407847	3.553659626	4.59020799	5.539777276	8.979824047	9.093139882	8.866288003	11.9754114
BRI3BP	24.20931078	21.39771273	25.49138866	27.94933525	23.06369053	23.58052963	21.30249969	21.12466744	14.61240366	13.38271756	13.81832824	14.41530095
BRICD5	1.409173757	1.839631559	2.209613242	2.215496684	1.907408295	2.78714243	1.449126608	3.421674295	3.975827347	3.605504931	5.509453888	6.680760967
BRINP1	0.016836533	0	0	0	0	0	0	0	0	0	0	0
BRINP2	0	0.007808528	0	0	0	0	0	0	0.040984222	0	0.040566702	0
BRINP3	0	0	0	0	0	0	0	0	0	0	0	0.007337382
BRIP1	2.296365926	2.52505378	2.459043589	3.656260291	4.199698077	4.070463041	2.301395825	3.350720844	3.94170329	3.005901817	3.649835022	3.37658301
BRIX1	58.16335879	53.54925115	69.03231858	73.61009073	88.5835859	82.60852526	79.11233742	77.6351504	62.69827524	74.53184128	77.26709435	56.96667242
BRK1	72.84423545	78.22110262	77.97106964	74.59462326	62.58903589	66.16218169	72.60388098	75.35278773	71.30563275	77.71919716	72.79713971	79.42700958
BRMS1	28.66629259	25.60456585	34.26587049	34.29526483	29.8165644	28.52521259	34.1316163	39.62577376	21.34894417	16.79449111	21.77059166	22.65427541
BRMS1L	2.122187551	2.140999906	2.653173507	2.749488407	2.379536205	2.23791693	2.607109551	2.773407289	1.673978762	1.655495945	1.472822539	1.912085217
BROX	11.05228797	10.3610751	10.29224075	12.3307933	11.199753	10.00952878	11.02999686	10.67155146	23.4306517	20.48677294	20.29447645	25.54561773
BRPF1	11.7638613	11.25827006	11.93317813	11.92195137	11.81710886	10.37703808	12.04270011	13.14501848	16.85329557	12.89955288	16.08583355	16.45984633
BRPF3	6.246875129	6.533128738	5.871592231	9.538053079	6.309528059	6.504078505	6.617918004	8.419240145	16.59777701	9.745027364	11.67190265	19.29868593
BRS3	0	0	0	0	0	0	0	0	0	0	0	0
BRSK1	0.228837744	0.271125743	0.276805741	0.066081614	0.085338394	0.167862875	0.201707781	0.223918893	1.067282936	0.860331096	0.880341793	0.935583061
BRSK2	0.008768056	0.009089803	0	0	0.027466253	0.027013423	0.027049944	0	0.39121534	0.499955592	0.472231576	0.364992008
BRWD1	3.683106998	3.510809734	3.201228224	3.396318075	3.342609029	3.197922469	3.108062193	2.212720322	6.395465342	6.358106314	5.353165936	5.630299401
BRWD3	4.735774495	5.043413123	4.236521622	4.020267514	5.766986221	5.325433154	4.338921361	3.109697679	7.488059499	7.978995432	6.335004688	6.328538765
BSCL2	7.89748628	6.792026143	7.633124063	8.366949015	6.734991436	6.832581475	7.442436454	8.473170627	10.08672196	9.495276854	9.901905014	10.28011876
BSDC1	10.99835253	10.55809687	10.03058169	11.00827112	8.226160145	8.418935341	9.725756141	10.50593275	13.00240068	13.20771427	11.24162702	14.93505658
BSG	187.2644734	193.9728933	196.8217616	206.9866308	154.9778072	161.2976748	206.5985126	238.8650412	184.9324128	187.8219545	186.4665096	225.6116238
BSN	0.131497768	0.122341266	0.096354727	0.081787333	0.091538114	0.086566293	0.086683326	0.135269758	0.396283712	0.366027512	0.330504106	0.417561812
BSND	0	0	0	0	0	0	0	0	0	0	0	0
BSPH1	0	0	0	0	0	0	0	0	0	0	0	0
BSPRY	0	0	0	0	0	0	0	0	4.014573696	5.693185506	3.851031517	3.199259984
BST1	2.266028963	2.826172903	2.106205893	4.731672428	1.201085905	1.760112939	2.152843214	3.748060097	13.79461293	14.22764734	12.78676318	19.79952516
BST2	83.85631271	79.43352363	66.45422666	81.33758287	44.71785445	58.29734343	73.09238073	90.32121561	79.07376027	90.50425624	69.28101576	98.75370089
BSX	0	0	0	0	0	0	0	0	0	0	0	0
BTAF1	21.73852583	18.80668822	20.00550138	19.73650104	20.02334434	20.9991055	18.28734975	14.49346727	26.23682507	35.13216162	24.20749457	28.80741358
BTBD1	29.30466072	28.85677412	30.22160634	34.43623995	31.09366526	29.89362792	29.83331108	33.06782816	30.50501437	32.47388807	28.62914246	41.26860597
BTBD10	6.74298541	5.263703944	6.141687849	6.434989994	5.371828227	4.841842476	7.113732266	5.888295529	7.425760007	7.379229044	6.843705899	7.199059636
BTBD11	0.086421229	0.083193023	0.0653353	0.118540576	0.045119604	0.082412064	0.063479601	0.108724643	0.013435415	0.013537771	0.006649272	0.006424102
BTBD16	0	0.029911057	0.030537684	0	0.03012697	0.014815136	0	0	0	0	0	0
BTBD17	0	0	0	0	0	0	0	0	0.043089225	0.043417494	0	0.041205959
BTBD18	0.034020797	0.011756401	0	0	0	0	0	0.011096508	0.012341045	0	0	0
BTBD19	1.024944016	0.934315339	0.935185243	0.160744341	0.073808602	0.090739669	0.34527691	0.034583204	1.903864515	1.104515382	1.18017262	0.99308392
BTBD2	40.48777638	48.76423782	40.83246456	40.35225168	43.51434666	47.29535886	43.14407413	48.95660625	30.28498358	34.92230521	36.31805031	34.48177154
BTBD3	6.431537561	6.164713647	6.53001062	7.578823747	8.579451675	8.009628392	6.424346154	7.119108751	7.210255917	5.690885213	5.736609935	5.128437103
BTBD6	9.182772346	10.93101925	12.20790988	16.16045028	12.50892849	11.41303859	10.97031157	17.77702364	18.45096876	16.47454242	20.34258505	24.49629843
BTBD7	8.4694537	7.342000007	7.64975501	7.779809959	8.195246749	6.761779783	7.518771641	5.884384858	10.8605812	6.385648581	7.996172238	10.60713106
BTBD8	0.223740378	0.30926748	0.236809901	0.150755838	0.155749962	0.114886608	0.268431168	0.145954063	2.394273851	1.880943326	2.128877543	2.056785612
BTBD9	2.287477129	1.817639703	1.937789773	1.806888557	1.407931148	1.477623342	1.488481019	1.323594548	2.306513688	1.464362825	1.758665094	2.331232679
BTC	0	0	0	0	0	0	0	0	0.01367864	0.027565697	0.040617872	0.078484792
BTD	1.658595007	1.277311461	1.36425861	1.62844186	1.484459673	1.153388699	1.189060411	1.15924545	1.361460138	1.267905534	1.133201076	1.390725625
BTF3	510.9969587	579.1901442	544.0381375	440.2629621	547.9674893	572.3373035	592.4717374	506.2100587	391.5761113	449.4064157	454.6443641	333.3032938
BTF3L4	14.48674372	15.88339627	14.48658943	16.1761615	16.43370074	17.56718433	15.55296017	16.14856126	16.96335467	18.61757666	17.51459622	18.85123388
BTG1	8.600331522	12.06272033	8.190004662	10.74920533	10.81715931	10.14289186	9.045171672	11.12686883	12.44988585	15.23018893	12.17443476	16.57232369
BTG2	68.82941247	80.40565329	56.74277842	31.44393229	7.313399674	6.703794568	27.56556482	14.7552454	35.13063578	14.94690456	23.38130671	14.92890998
BTG3	7.703505508	7.056453001	7.131267061	5.833622786	7.059367007	6.895749531	6.550359616	6.255347895	8.233190843	10.46445101	10.37861275	7.466519845
BTG4	0.035611887	0.012306226	0	0	0	0	0	0	0.129182125	0.195249419	0.05114644	0.086475246
BTK	65.78849057	61.9483122	61.9580015	46.5480813	62.81302852	62.13453823	61.82520534	48.68907298	41.18944285	35.09759392	36.42504479	36.06003405
BTLA	0	0	0.013077562	0	0.025803352	0	0.012706124	0	0	0.027097302	0.013309232	0.01285853
BTN1A1	0.013595139	0	0	0.013740568	0	0	0	0	0	0.029815247	0.02928838	0.014148282
BTN2A1	14.17500911	15.30052822	13.70513213	14.63648596	14.95367592	13.86758659	14.26164121	14.07030233	7.927447172	7.347533322	8.129734344	7.064429723
BTN2A2	2.998253286	4.014855529	3.221474333	3.018847476	3.308593672	3.359014788	3.024864601	2.900467348	2.756118895	2.677488494	2.446673856	2.659297644
BTN3A1	5.674976354	7.373992703	5.503668916	5.722705644	8.44611862	8.979330061	6.113143099	5.527866491	3.632819574	6.969020592	3.402190193	4.43608552
BTN3A2	12.34952195	13.43675579	12.27205034	11.73193963	14.44415162	16.98574192	12.92715167	12.36420639	7.109128185	9.703258659	6.125577872	9.114210137
BTN3A3	3.190573562	4.088110184	2.978543193	2.572516303	2.75131892	3.55272102	2.801780652	2.332724673	3.257571262	4.638585059	2.741692845	2.984618193
BTNL10	0	0	0	0	0	0	0	0	0	0	0	0
BTNL2	0	0	0	0	0	0	0	0	0.125354067	0.108264907	0.159527625	0.051375137
BTNL3	0.198477888	0.205761105	0.023341304	0.089156014	0.115136882	0.113238645	0.226783475	0.064737207	0	0	0	0
BTNL8	0.082577496	0.021401927	0.010925145	0.010432605	0.021556416	0.03180153	0.053074207	0	0.022466241	0.011318698	0.02223737	0.021484327
BTNL9	0	0	0	0	0	0	0	0	0.975357519	0.91788703	1.020067695	0.835935793
BTRC	4.003431328	3.793320275	3.758883566	4.749638058	3.925584331	3.93454465	3.939863953	4.142030362	4.270848882	4.169642454	3.408148175	5.241496931
BUB1	23.72297196	22.52230216	25.75092752	57.76554486	50.51557886	47.11425235	34.05943501	53.63763655	62.92274762	72.911621	63.73236832	51.65574992
BUB1B	17.64412912	19.2754862	19.63364187	37.1772478	42.8983655	38.94223122	25.02060504	36.73885512	34.58411227	43.53583046	39.54468986	27.0267267
BUB1B-PAK6	0.055488075	0.014381057	0.07341168	0.084122457	0.043454601	0.014246058	0.028530636	0.054295363	0.03019245	0	0.059769738	0.04330928
BUB3	139.643987	152.3366681	152.6845548	200.2845988	214.7051256	231.7780377	160.8003142	205.239743	80.33752199	82.99891732	87.38293023	70.66720664
BUD13	9.080477129	9.602339514	9.880546773	14.23541444	14.85900458	14.277643	11.3028209	14.06689664	11.7631399	13.52890288	13.13302044	12.80191262
BUD23	31.34688189	27.30888149	31.16594161	31.83904607	27.30346235	28.44698444	31.35792498	32.15359069	49.18555838	58.40880027	58.21243922	50.30614849
BUD31	15.92341099	15.59063028	17.69983687	20.22034722	18.24338988	15.47921792	20.60854498	22.40620978	17.14351756	15.18481211	18.54481318	18.44794581
BVES	5.536792974	5.207785126	6.131883779	7.597246721	6.71944338	5.657842521	4.920776428	6.476220486	7.132734889	4.613800311	6.013403327	6.458476067
BYSL	27.93229968	22.25758423	30.70871072	26.208732	27.95222645	25.32539609	31.1990243	28.6283761	14.2128835	14.76591881	17.53414352	14.35147659
BZW1	62.59748099	62.66618454	68.09571678	84.84381873	72.61532997	66.74802672	74.35663184	71.43914976	73.2137339	80.85844017	78.35855318	79.82886503
BZW2	70.41231769	66.18135539	72.51785818	55.96078913	64.92274704	70.65653997	74.73320355	61.39875514	32.37527974	40.77028474	40.60957605	25.227896
C10orf105	0.01725495	0.026832189	0.027394315	0	0	0.026580307	0.053232485	0	0.06572195	0.07568302	0.027879607	0.008978499
C10orf111	0.031394143	0.065092324	0	0.06345994	0.032781096	0.064481282	0.064568457	0.092157984	0.034164677	0.068849912	0.06763326	0.098014409
C10orf113	0	0	0	0	0	0	0	0	0	0	0	0
C10orf120	0	0	0	0	0	0	0	0	0	0	0	0
C10orf126	0	0	0	0	0	0	0	0	0	0	0	0
C10orf142	0	0	0	0	0	0	0	0	0.052755187	0	0.156653253	0.050449458
C10orf143	2.031585461	1.394603042	1.743452489	1.72034717	0.917335488	1.071376238	1.12928914	1.370050686	0.149383309	0.180625636	0.118289191	0.399992135
C10orf25	0.616012918	1.190935766	0.810590328	0.67308377	0.695381171	0.82069988	0.616357068	0.55389838	1.540053172	1.588298443	1.775435848	1.715312805
C10orf53	0	0	0	0	0	0	0	0	0	0	0	0
C10orf55	0.471074548	0.577153667	0.838540751	0.757453602	0.44716911	0.703674761	0.572508701	0.670471033	0.39613624	0.4226338	0.415165406	0.557092109
C10orf62	0	0	0	0	0	0	0	0	0	0	0	0
C10orf67	0	0	0	0	0	0	0	0	0.005871679	0.005916412	0.011623725	0
C10orf71	0	0	0	0	0	0	0	0	0	0	0	0
C10orf82	0	0	0	0	0	0	0	0	0	0	0	0
C10orf88	6.205443365	5.872963897	6.863114559	6.906053155	7.553456894	6.390665276	7.098388882	6.24262124	4.666450028	3.402260652	3.886644852	4.553198448
C10orf90	0	0	0	0	0	0	0	0	0	0	0	0
C10orf95	0	0.037974215	0.193848813	0.148087593	0.038248331	0	0.037668596	0.107528104	0.079725333	0.040166354	0.23673943	0.152481685
C10orf99	0	0.129429517	0.066070512	0	0.130363802	0	0.192581791	0	0.203799039	0.06845055	0	0
C11orf1	5.218186952	4.168920127	4.205587943	4.79015275	3.999060401	5.211395559	5.169210522	4.73126847	7.137438806	8.97664414	6.858457817	6.526562053
C11orf16	0	0	0	0	0	0	0	0	0	0	0	0
C11orf21	10.96980009	14.08553883	8.015431104	9.229907882	8.450308452	8.158494745	8.264962984	8.027800731	4.34288557	3.887490687	3.898769304	5.060956018
C11orf24	10.00200616	9.155409286	9.955867877	9.299448676	9.3287242	8.183609895	9.123966612	9.425276101	7.800317448	7.927305256	9.291570816	9.19065897
C11orf40	0	0	0	0	0	0	0	0	0	0	0	0
C11orf42	0	0	0	0.009291845	0	0	0	0	0	0	0.009902905	0.009567555
C11orf44	0	0	0	0	0	0	0	0	0.016509235	0	0	0
C11orf45	0	0.028666044	0	0	0	0.014198473	0.014217669	0	3.249892992	3.47173734	3.216785198	2.302113005
C11orf49	0.881221058	0.799363054	0.774471218	0.98607412	0.739408083	1.026184857	0.93047877	1.231827538	3.450647404	3.9255725	3.771451693	4.208719696
C11orf52	0	0	0	0	0	0	0	0	0	0	0	0
C11orf53	0	0	0	0	0	0	0	0	0	0	0	0
C11orf54	4.245398323	3.73027241	3.735357209	3.610561225	3.513927432	3.571193843	3.833353516	2.972070361	5.277379806	5.014804106	3.913065625	5.019785832
C11orf58	21.53710861	21.07074987	21.71429141	22.07797849	24.42188115	21.17990728	24.09440576	22.22780619	29.03969113	27.79514811	28.12676408	28.64316458
C11orf65	0.050692003	0.105104327	0.107306229	0.128085654	0.079397266	0.1301471	0.052129221	0.074403573	0.151705329	0.180653996	0.150159854	0.171452116
C11orf68	6.105315559	8.939763092	6.096868255	8.332086728	7.203435722	7.934835076	7.484436168	9.41677372	16.51643148	15.84796978	13.04138332	19.59961459
C11orf71	1.948401623	2.144584026	1.7057829	1.87199731	1.130266401	1.679799366	1.781015685	1.223939326	2.277410981	2.848668945	2.150568352	2.928865071
C11orf74	4.853685243	4.561193598	4.656749153	5.187942754	3.609664545	4.159774947	4.632210679	3.860986957	7.410032529	8.232278056	7.409770033	8.067330008
C11orf80	1.804447871	2.470234108	1.860881934	2.548574109	2.343129608	1.80558678	1.546339601	2.625855411	1.510531137	2.105487133	1.993523772	1.54081235
C11orf86	0	0	0	0	0	0.04643736	0	0	0	0	0	0
C11orf87	0	0	0	0	0	0	0	0	0.017312976	0.02325983	0.005712201	0.005518764
C11orf88	0	0	0	0	0	0	0	0	0	0	0	0
C11orf91	0	0	0	0	0	0	0	0	0	0	0	0
C11orf94	0.729126841	0.151176482	0.617374323	0.736926404	0.456803241	0.748786701	1.349638241	0.570763451	0.952166782	0.319806907	0.628311151	1.365826801
C11orf95	6.091284697	6.055020195	5.886038834	5.279735157	6.269814714	5.265728812	6.263984614	5.979214859	5.380688854	3.677865354	5.107855669	4.423341284
C11orf96	0.081038495	0.084012231	0.04288613	0	0.126928009	0.208058955	0.166672192	0.158593158	1.014186681	1.821671196	1.09114653	1.560210284
C11orf97	0	0	0	0	0	0	0	0.087173722	0.193901513	0	0.095963086	0
C11orf98	0.247590004	0.256675407	0.349403566	0.333651341	0.344704286	0.847553046	0.763829004	0.16151205	0.179626555	0.090497507	0	0.085887882
C12orf10	17.58221865	19.55887724	18.93738572	18.93409573	21.27236821	21.55840112	21.08657015	22.31790789	16.91682982	19.4738717	18.46187598	16.13137004
C12orf29	15.91444911	15.68321863	16.6260903	16.80376817	18.22063198	20.20833053	16.19237167	16.63495439	7.661066338	10.03936641	8.530897929	9.527996358
C12orf4	9.031305909	7.316151286	9.033371727	9.71334404	9.405610843	8.279356929	10.38142209	9.149487325	4.180894891	3.564631579	4.252901806	4.920817121
C12orf40	0	0	0	0	0	0	0.011348476	0	0.012009487	0	0	0.011484598
C12orf42	0	0	0	0	0	0	0	0	0	0	0	0
C12orf43	7.920262836	7.076372556	7.931376645	10.10465559	9.819616307	9.657722335	9.785226171	11.50705155	3.492098749	2.888183784	3.11686514	3.281562497
C12orf45	34.51770866	28.6992266	31.13174541	29.72822721	29.71938384	30.65089744	34.33983087	33.86040407	7.437463518	7.778711731	6.802579368	10.62358531
C12orf49	17.80135435	14.94442748	17.89699307	18.01226948	11.9931505	12.07651318	17.16974783	15.84150839	10.85556235	8.292626839	9.674843527	12.31173982
C12orf50	0.011808111	0	0	0	0.012329778	0	0	0	0	0	0	0
C12orf54	0	0.028948688	0	0	0	0	0	0	0.212718111	0.183718861	0.180472352	0.145300724
C12orf56	0	0	0	0	0	0	0	0	0.053551218	0	0	0
C12orf57	11.96867071	13.12567569	10.78333911	13.9961831	12.03265081	12.2405979	13.78293244	16.06688622	20.82908592	21.42162488	21.149629	21.10252622
C12orf60	0.38622602	0.486198446	0.510983683	0.474005571	0.662546203	0.552462929	0.709243372	0.6748645	0.270199597	0.19663083	0.222872483	0.157905124

C12orf65	6.192798015	5.493769786	6.570847657	7.831588836	6.434216849	6.91348995	6.87531144	8.486572759	2.363788791	2.635179616	2.903892969	2.677302063
C12orf66	3.167677777	3.227297576	3.078138142	3.422360267	4.020471254	4.220603407	3.103037833	3.153029963	2.228809581	2.395508728	1.911956731	1.904047816
C12orf71	0	0	0	0	0	0	0	0	0	0	0	0
C12orf73	2.613135247	2.568903176	2.829359495	2.89912565	2.822669203	3.254244141	3.413081803	3.027212736	2.124636734	2.634859028	2.183876638	2.375616138
C12orf74	0	0	0	0	0	0	0	0	0	0	0	0
C12orf75	7.24813997	10.53609326	9.797879653	9.117279362	13.03895693	15.53441881	12.55776138	10.36869526	8.402209652	11.87862588	13.19625903	3.771521737
C12orf76	5.229987378	5.95291479	4.308552093	2.888190316	3.94095782	3.460700061	5.57232903	3.376540494	4.224643796	5.912261959	3.716983	5.330556503
C12orf77	0	0	0	0	0	0	0	0	0.067502314	0.034008285	0.16703661	0
C12orf80	0	0	0	0	0	0	0	0	0	0	0	0
C13orf42	0	0	0	0	0	0	0	0	0.111894759	0.018791202	0.018459141	0.017834044
C13orf46	1.016126831	0.643752978	0.788687278	0.399387516	0.565876277	0.405815369	0.592130416	0.33142842	0.073720005	0.037140815	0.097291991	0.140995969
C14orf119	14.54045119	14.35620779	16.19245743	16.12893481	13.44420641	13.49343918	15.74950091	15.42162974	16.57715365	13.66645435	15.30728816	17.07074275
C14orf132	0	0	0	0	0	0	0	0	0.245725417	0.323781267	0.236985662	0.078328565
C14orf177	0	0	0	0	0	0	0	0	0	0	0	0
C14orf178	0.249889602	0.08635313	0	0.084187567	0	0.171085011	0.085658155	0	0.362589827	0.182676082	0.269171998	0.866856019
C14orf180	0	0	0	0	0	0	0	0	0	0	0	0
C14orf28	1.123261211	1.589607179	1.170004122	0.9550742	1.265971606	1.043686593	1.026762558	0.732744529	1.746270091	1.779124608	1.421194854	1.391622735
C14orf39	0	0	0	0	0	0.016349248	0	0	0	0.017456915	0.017148433	0.016567722
C14orf93	2.253562805	2.517437287	2.268375734	3.030694866	3.025443003	2.843315902	1.872882605	3.285182707	2.242230698	2.551813113	2.714787488	2.517557332
C15orf32	0	0	0	0	0	0	0	0	0	0	0	0
C15orf38-Al	0	0	0	0	0	0	0	0	0	0	0	0
C15orf39	27.87109172	28.52840558	27.46649832	25.13486672	21.98227586	20.57132047	22.64904545	26.49892123	11.85186319	8.676721866	13.52483395	14.15467987
C15orf40	2.603688429	2.407107053	2.564903222	2.642932366	2.459790723	2.3382096	2.758644733	2.823449864	2.220160144	1.989883592	2.051849166	2.909033706
C15orf41	2.168987121	1.785952594	2.207812737	2.234144824	1.712153169	1.897080352	1.803595636	1.655241527	2.812151593	2.753916797	2.995899051	2.926846979
C15orf48	0.395243364	0.234141119	0.358569452	0.513605999	0.353746896	0.347914744	0.348385107	0.22099865	0.491569839	0.309571744	0.182460767	0.117521299
C15orf53	0	0	0	0	0	0	0	0	0	0	0	0
C15orf56	0	0	0	0	0	0	0	0	0.13758989	0.138638098	0.054475285	0.052630544
C15orf59	0	0	0	0	0	0	0	0	0	0	0	0
C15orf61	7.675226491	6.881618714	7.860098576	5.786548394	5.718317208	5.240583195	5.46098804	6.779521226	5.643625392	4.958733079	5.452064646	5.09473405
C15orf62	0.194570529	0.179298101	0.251699716	0.13110125	0.451480905	0.155413102	0.266782649	0.253851	0.376429133	0.213354507	0.09314858	0.269982638
C15orf65	0.787107938	0.593448103	0.378675408	1.157131247	0.5977319	0.367423262	1.103759998	1.190292263	1.479530431	1.412338748	1.695688139	1.638265657
C16orf45	0.24361186	0.089135747	0.197173411	0	0.059852781	0	0.058945585	0	2.105290201	2.40417291	2.130150494	2.043102301
C16orf46	0.79294418	0.516165622	0.936851783	0.596410348	0.423615343	0.549195784	0.493048103	0.487192963	0.602038284	0.424637375	0.297952555	0.537343786
C16orf47	0	0	0	0	0	0	0	0	0	0	0	0
C16orf54	8.740429054	10.56419857	8.724867959	8.101254907	5.947409401	5.934437236	6.943519911	6.809616596	10.59368007	6.99512983	10.59730918	12.71722491
C16orf58	25.86225606	25.28430685	23.48568301	21.57619913	23.68913728	25.2434018	20.91051669	25.08173621	37.84532931	41.32194009	37.33615607	48.19528407
C16orf70	5.39849179	5.609576483	5.196808722	5.367623716	5.205387769	5.376832328	4.804473375	5.932033824	6.583714656	4.477520027	4.735697855	6.999862355
C16orf71	0.018099415	0.093817897	0.01915667	0.018293027	0	0	0.037225138	0.035420739	0.039393378	0.079386982	0.097480161	0.339044796
C16orf72	6.284587445	6.502932915	6.758171234	7.314754338	7.161607788	6.466196845	6.815725066	6.994912286	12.84121462	12.71841787	12.2641942	12.0952202
C16orf74	13.12282164	26.15268957	13.31857915	16.41245066	19.89688415	24.3072173	12.44733175	21.10807817	2.934433817	5.190807988	2.839994389	5.799440526
C16orf78	0	0	0	0	0	0	0	0	0	0	0	0
C16orf82	0	0	0	0	0	0	0	0	0	0	0	0
C16orf86	1.080929538	1.304801966	0.783610089	0.61359161	1.159606466	1.398998951	0.898397057	0.825871468	0.902383708	1.769806495	0.845341303	1.109499372
C16orf87	1.778408527	1.648013308	1.567296288	1.636030298	2.152576664	2.146905715	2.015445208	1.448967675	1.816862151	1.878461129	1.806172069	1.699683291
C16orf89	0	0	0	0	0	0	0	0	0.02606064	0.026259179	0	0
C16orf90	0	0	0	0	0	0	0	0	0	0	0	0
C16orf91	14.80038024	14.08862787	17.41194439	11.7334055	13.6158193	14.12588409	17.82267677	15.45131946	15.80713688	12.30766091	16.77214942	12.76866507
C16orf92	0	0	0	0	0	0	0	0	0	0	0	0
C16orf95	1.251385136	1.249256817	1.471648096	1.826891968	1.839016665	1.142335061	1.620495872	1.814054378	2.168824336	2.236169226	1.647490285	1.109366644
C16orf96	0	0.012960995	0	0	0.026109107	0.025678652	0	0.012233487	0.095238801	0.068545974	0.026933877	0.065054483
C16orf97	0	0	0	0	0	0	0	0	0	0	0	0
C17orf100	1.448718946	2.074025037	1.752393271	1.045868627	1.116532537	0.991854385	1.383379197	0.843796928	0.412910787	0.718643021	0.222929629	0.753831331
C17orf102	0	0	0	0	0	0	0	0	0	0	0	0
C17orf107	0.017459299	0.054299924	0.092395817	0.017646064	0.018230629	0.035860129	0.07181722	0.136672105	0.1140005	0.134013829	0.188065229	0.054508987
C17orf112	0	0	0	0	0	0	0	0	0	0	0	0
C17orf113	0	0	0.025222667	0	0	0.024473188	0	0	1.063281018	0.679412636	0.462050782	1.116009941
C17orf47	0	0	0	0	0	0	0	0	0	0	0	0
C17orf49	66.7405406	62.76485896	68.49470708	73.8385567	58.42680389	60.76814547	61.5243727	80.69950476	23.28063598	26.39841333	18.74287649	23.93751379
C17orf50	0.052191621	0	0.055240331	0	0	0.053598893	0	0	0.113595067	0	0.112437834	0.10863026
C17orf51	0.657225752	0.734262719	0.614574066	0.741645249	0.706249337	0.570100782	0.643050688	0.668073441	0.506907716	0.552750572	0.597968483	0.345303301
C17orf53	2.347822821	3.470061815	2.955097143	4.842075742	4.687754721	4.056560785	3.409507691	5.495028487	4.574863833	5.479474623	5.75857728	4.67207794
C17orf58	6.803507695	7.516251391	7.528241207	8.230705593	9.18507053	9.068925831	7.950455155	8.540130137	7.665672221	9.532635192	8.846007136	7.795504436
C17orf64	0.195197923	0.168633983	0.137733449	0.328809967	0.237791772	0.200461156	0.434919699	0.286503238	0.708080497	1.105886097	0.770953737	0.677132997
C17orf67	1.211571121	0.982980118	1.142958411	1.091430207	1.017577851	1.054898622	1.083410042	1.262845564	2.006401368	2.108330557	1.305060386	2.275040922
C17orf75	1.916199683	2.055812352	1.886634577	2.285753463	2.535967309	2.288217746	2.268398186	2.09303566	3.346186118	3.4938408	2.760081127	2.68980218
C17orf77	0	0	0	0	0	0	0	0	0	0	0	0
C17orf78	0	0	0	0	0	0	0	0	0	0.022932131	0	0.021764049
C17orf80	8.830471244	8.533442798	8.826349541	9.603081429	8.770195021	9.043962825	8.477086506	9.097900775	11.42218508	9.249405375	9.227926546	11.72098974
C17orf82	0.035169648	0	0.03722404	0	0	0	0	0	0.076546741	0.115694852	0.113650399	0.14640235
C17orf97	3.30640586	3.731075024	3.778270429	4.909156056	2.933079211	3.996542137	3.460328606	4.867318007	2.642922967	2.213867227	2.86814303	4.324004152
C17orf98	0	0.098558519	0	0	0	0.097633319	0	0	0.103459818	0	0	0
C17orf99	0	0	0	0	0	0	0	0	0	0	0	0
C18orf12	0	0	0	0	0	0	0	0	0	0	0	0
C18orf21	9.778447613	9.671189767	8.803145309	8.519869754	8.743428716	9.205266988	9.76672937	10.14565564	9.051302397	8.812141548	8.111611791	9.708425282
C18orf25	7.667958033	6.958147376	7.063440183	7.566380066	8.096569597	6.401886813	7.177447148	5.912695576	9.832543164	12.70669895	9.958949438	9.05454832
C18orf32	0.032552668	0.2024832	0.172270966	0.394810661	0.033990804	0.267443236	0.133902403	0.477794268	0.141701771	0.071390653	0.070129103	0.169385659
C18orf54	1.589985734	1.777893943	1.822489002	2.856098532	2.76221552	2.823631173	1.863545654	2.269171363	3.54371904	5.032502122	3.731985823	3.294902951
C18orf63	0	0	0	0	0	0	0	0	0	0	0	0
C18orf65	0.181438789	0.268709642	0.384074633	0.261970947	0.297714251	0.159712308	0.319856462	0.101450729	0.535937805	0.397910039	0.697997395	0.215795371
C19orf12	4.591611617	3.9731192	4.00955069	3.657460318	3.547748825	3.300035603	4.009355404	3.504906611	2.221700754	2.44875023	2.341967207	2.822571534
C19orf18	0	0	0	0	0	0	0	0	0.319990476	0.451399585	1.013537987	0.734411788
C19orf24	28.61826924	29.48144085	34.29893757	35.73014429	29.44313805	30.93350809	31.90273366	48.24067176	9.159950825	9.559367911	10.94472393	9.072447298
C19orf25	4.270229678	4.741408436	5.013622986	7.664865479	4.580710383	5.272029872	5.975046315	8.790710063	5.129561113	4.78483001	5.931900288	5.026787537
C19orf33	0	0	0	0	0	0	0	0	0	0	0	0
C19orf38	10.23987912	20.43336298	8.606670309	9.470054234	7.757132647	12.02808489	9.601064576	10.3144729	5.280439847	7.74248942	6.308021232	3.169091912
C19orf44	2.838083737	2.574449695	2.816125282	2.90685973	3.519116655	3.370016192	3.205192206	3.520939874	1.723518279	1.528250807	1.746903242	2.004198875
C19orf47	2.162911823	2.224087689	2.405343719	2.376718334	2.070642722	1.905843781	2.079862405	2.38258257	1.332063084	1.390319135	1.40355695	1.839996431
C19orf48	49.44921354	57.17499917	59.9449577	65.88692954	66.79374548	72.23718003	57.74961421	80.50318605	35.22359986	37.01565604	44.3099934	37.17469197
C19orf53	76.51601292	77.23955312	87.55707807	74.82442976	63.53430307	68.07359786	83.36759908	83.83966279	35.19930397	39.94142264	39.42669732	31.50707275
C19orf54	5.997310847	7.715389455	5.982207542	5.880525597	7.664263882	8.588483817	6.219946389	7.307345566	9.07319002	12.39540564	9.903627598	8.09109565
C19orf57	0.223740378	0.304435176	0.222009283	0.296800556	0.379640531	0.459546433	0.273224582	0.383129416	0.547842322	0.628684862	0.48201001	0.509345494
C19orf66	4.137589534	3.856145111	3.229167406	1.626274945	2.203831713	2.18895797	3.932557562	2.637758351	8.70983008	8.47829845	7.878289818	10.1559168
C19orf67	0.019252079	0	0	0.019458021	0	0	0	0.018838257	0	0.042221377	0	0.020035384
C19orf70	31.44060076	31.95128008	33.23613318	31.54183049	27.64811489	30.25788798	32.37187055	35.16517619	10.46285861	11.77536866	11.73432185	10.85281253
C19orf71	0.538095609	0.796916024	0.488166697	1.010010274	0.722401697	0.868378635	0.237150719	0.75218469	1.505783753	2.444466937	0.993629206	1.999960673
C19orf73	0.650922107	0.749786582	0.84204381	0.29239338	0.604159125	0.668473289	0.743752256	0.495390455	0.236122004	0.237920864	0.389527578	0.376336686
C19orf81	1.758482381	2.187612616	1.563409683	2.13275171	1.101701934	2.961671636	1.519004608	2.408957505	1.760575049	2.313897033	2.424541852	1.976431724
C19orf84	0	0	0	0	0	0	0	0	0	0	0	0
C1D	12.5723273	11.73030596	13.3067246	14.01985265	15.70954884	14.2024543	14.65261455	14.43444764	16.19017232	19.85193903	19.32056789	17.31430128
C1GALT1	3.516964762	3.556590322	3.265882761	3.661894445	4.794828543	4.109268284	3.527966678	3.402408983	6.390930756	7.050837186	6.497371886	5.096459162
C1GALT1C1	8.845794406	7.353894194	9.392422967	9.968710001	7.790606533	7.342907572	8.835026804	9.512924025	8.826796101	8.026330334	6.057972179	9.793925059
C1GALT1C1L	0.505901854	0.476787365	0.681486272	0.23241525	0.432206143	0.377849289	0.472950153	0.225012514	3.203186711	2.975434262	1.981596706	3.398223776
C1orf100	0	0	0	0	0	0	0	0	0	0	0	0.016292959
C1orf105	0	0	0	0	0	0	0	0	0	0	0.01521704	0
C1orf109	4.060197668	2.884531814	3.905550249	4.296742126	4.912915815	4.16967508	4.863010759	4.311791068	4.015646123	4.779537647	4.733667764	4.511229534
C1orf112	4.381413714	4.5851434	5.898093617	8.710001978	9.052617989	8.435330826	6.15663658	7.601481471	4.508816713	4.327366049	4.607927548	3.945254471
C1orf115	0.054316515	0	0.057489349	0	0	0.018593694	0.018618832	0.017716329	1.241308949	1.786808106	1.306673669	1.413161983
C1orf116	0	0	0.010317533	0	0	0	0	0	0	0	0	0
C1orf122	7.716242035	6.094775056	8.080553948	5.07539889	4.689338277	4.402388581	6.801439451	6.591603318	4.665111526	6.222767856	5.760988755	8.370093377
C1orf123	20.85302765	22.47490359	21.81389275	23.92554391	21.16521683	24.80979937	21.79415826	26.92100942	23.43388246	27.87650206	22.5144829	25.24250273
C1orf127	0.894605363	1.440481388	0.82598657	0.442468651	0.755252334	0.625516333	0.841673937	0.316625321	1.304977076	0.667895288	1.250677016	0.653683644
C1orf131	24.55713338	23.06310295	26.58322341	28.36013293	26.30351515	26.32908275	30.27339517	30.55620079	21.45243628	19.89967473	21.39445156	25.40116256
C1orf141	0	0	0	0	0	0	0	0	0	0	0	0
C1orf146	0	0	0	0	0	0	0	0	0	0	0.122282075	0
C1orf158	0	0	0	0	0	0	0	0	0	0	0	0
C1orf159	2.681539578	2.517922322	2.733785699	2.479699519	3.18621438	2.842472895	2.599085672	2.756603075	1.355111459	1.399233073	1.454188693	1.141918203
C1orf162	2.956080791	3.840294761	3.458099484	5.009455844	2.576098574	3.279258935	3.131316811	4.059705337	3.007328912	3.217091043	3.375712605	3.777978856
C1orf167	0	0	0.007473138	0	0	0.007251077	0.00726088	0.006908926	0.015367605	0	0.015211049	0
C1orf174	6.921248474	6.901262951	7.072480559	9.030278229	6.70141892	7.637729423	6.651608113	8.570261486	7.641606043	7.465240066	7.495982723	8.512461236
C1orf185	0	0	0	0	0	0	0	0	0.176247576	0.088795146	0	0.084272233
C1orf189	0.076217508	0	0.080669662	0	0.079584701	0.156545208	0.078378425	0.149158437	0.497662241	0.334302404	0.246296191	0.158637107
C1orf194	0.039594968	0	0	0	0	0	0	0	0	0	0	0
C1orf195	0	0	0	0	0	0	0	0	0	0	0	0
C1orf198	1.956266698	2.306909827	2.303475363	3.21293884	1.991624764	2.410817552	1.835704274	2.23724099	5.468630687	5.939317845	6.189956992	7.074616622
C1orf21	0.720384109	0.898347323	0.57461091	0.612017807	0.103565112	0.225159028	0.209358901	0.188994795	2.965406499	4.298824712	3.109509325	2.477250472
C1orf210	0	0	0	0	0	0	0	0	0.102673741	0.06897063	0.067751845	0.130915018
C1orf216	8.236321423	8.938800965	9.636853279	10.89329237	9.188095322	8.821848991	9.230799305	11.19169867	5.969616041	5.168395122	6.463293114	8.13616288
C1orf226	0.162039447	0.103375718	0.211082812	0.088185356	0.07809145	0.025601324	0.102543744	0.073179886	2.59083324	2.077522562	2.336190974	3.320759347
C1orf229	0	0	0	0	0	0	0	0	0.207469467	0.287443808	0.308033855	0.248002209
C1orf232	0	0	0	0	0	0	0	0	0	0	0	0
C1orf35	11.61925528	14.03374131	16.43710599	17.25427431	16.09824419	15.75560244	14.92618784	19.42749557	14.36334607	14.22537327	15.43215171	14.79216196
C1orf43	98.89429969	92.5697423	101.5585789	87.19735682	99.5622751	99.40082486	103.1000522	92.3284248	145.5796843	166.6345035	157.522415	141.0815544
C1orf50	6.467750172	6.869965726	7.294444896	7.447821118	6.089209704	8.275457835	7.468884728	8.299968997	44.82735541	36.15834566	41.74384766	45.51634635
C1orf52	4.871766679	5.254721335	4.708766933	5.269313353	5.631382229	5.514742716	5.200516908	4.857741369	6.814670673	7.813045586	5.466083799	7.704445335
C1orf53	2.048387383	1.55182769	2.334813875	1.672164774	0.740382412	1.051809607	1.377302861	2.158538789	1.88621033	1.036680076	2.37617526	1.63979206
C1orf54	8.045687928	12.14439013	10.62755249	8.912402031	7.863463445	10.11345713	11.18617628	10.0141335	37.47448269	51.94643483	38.47936068	37.57820843
C1orf56	0.475220307	0.530555505	0.580361223	0.554196691	0.419874175	0.375410724	0.300734608	0.572314438	1.273005588	1.483126257	1.378165508	2.168435621
C1orf61	0.010919148	0.02263966	0.011556977	0.022071903	0.011401542	0	0.033686182	0.021368883	0.047531053	0.01197329	0.070570256	0.011363413

C1orf68	0	0	0	0	0	0	0	0	0	0	0	0
C1orf74	2.327499028	2.619728063	2.592495297	2.81167401	3.217287337	3.414652435	2.872185851	2.841414464	2.979173943	3.18417015	2.984639735	2.941239795
C1orf87	0	0	0	0	0	0	0	0	0.020061068	0	0	0
C1orf94	0	0	0	0	0	0	0	0	0	0	0	0
C1QA	0	0.041754582	0	0.040707462	0.042055987	0	0	0	0.482141338	0.132494853	0.303691561	1.089798929
C1QB	0	0	0	0	0	0	0	0	0	0	0	0
C1QBP	338.8197886	309.5706977	362.2843303	289.2748521	329.5841275	340.7807471	398.5749796	330.0881345	56.09105621	70.01378899	77.44839853	43.04644502
C1QC	0	0	0	0	0	0	0	0	0	0	0	0
C1QL1	0	0	0.037968521	0	0	0	0	0	1.171165141	1.337432485	1.236516345	1.007980179
C1QL2	0	0	0	0	0	0	0	0	0	0	0	0
C1QL3	0	0	0	0	0.022537825	0.022166249	0	0.021120308	0	0	0.023249781	0.044924909
C1QL4	0	0	0	0	0	0.049784197	0	0	0.553929459	0.823934957	0.731048515	0.378370938
C1QTNF1	0	0	0	0.011227326	0	0	0.011423445	0	0.024177645	0	0	0
C1QTNF12	0.979725898	1.132870819	0.837540902	0.685527335	0.944315943	1.006142803	1.085003292	1.327384748	0.328057463	1.280907286	0.24353657	0.823513218
C1QTNF2	0	0	0	0	0	0	0	0	0	0.083280698	0	0
C1QTNF3	1.898664589	2.362004107	1.741629747	3.169862153	4.258800733	4.116369757	1.778940316	3.275325912	5.249733971	3.747533447	3.469218541	4.859287615
C1QTNF4	0.23635239	0.204187854	0.16677242	0.218973958	0.143963247	0.222498186	0.121526723	0.308362684	0.385815749	0.302365022	0.297021907	0.184476607
C1QTNF5	0	0	0	0	0	0	0	0	0	0	0	0
C1QTNF6	0.356674991	0.28202286	0.249540329	0.171080184	0.119935869	0.142791883	0.14298493	0.177461841	1.197348925	1.70364276	0.950725619	1.774147789
C1QTNF7	0	0	0	0	0	0	0	0	0.011697614	0	0	0
C1QTNF8	0	0	0	0	0	0	0	0	0	0	0	0
C1QTNF9	0.027272965	0	0	0	0	0	0	0.080060205	0	0	0	0
C1QTNF9B	0	0	0	0.044872251	0	0	0	0	0	0	0	0
C1R	0.115306202	0.278920608	0.081361116	0.17480947	0.080266855	0.138151146	0.13833792	0.094023086	0.794719203	0.948284588	0.786623121	0.999980336
C1RL	11.96087487	12.42959057	9.875061466	9.182855077	7.325449568	8.105260955	10.18592858	8.426156577	6.257895492	4.807997375	4.351386249	6.612747338
C1S	0.126271179	0.093503389	0.095462255	0.07292681	0.11301401	0.222301543	0.092750868	0.052952995	0.569289123	1.246153397	0.893811052	0.488089631
C2	0.136641851	0.155821569	0.115698895	0.151913879	0.185482068	0.168391443	0.098361141	0.106963795	0.460971551	0.599333411	0.44155691	0.568805473
C20orf141	0	0	0	0	0	0	0	0	0	0	0	0
C20orf144	0	0	0	0	0	0	0.097079242	0	0	0	0	0
C20orf173	0	0	0	0	0	0	0	0	0	0	0	0
C20orf194	6.04372552	6.14150503	5.302089473	3.875512145	3.69533992	3.858405449	4.616087488	2.781352385	7.051798477	6.789206253	6.66165501	4.978981591
C20orf196	1.29728387	1.137982289	1.807279687	1.412019626	1.736661816	1.708029834	1.505098323	1.486390105	0.627452991	0.838924754	0.859930489	0.565412331
C20orf197	2.352532254	2.34144405	2.180928509	2.052078521	1.60493662	2.009283232	2.253577686	1.536832385	0.785208012	0.441594419	0.618152135	0.712471957
C20orf202	0.033547108	0.104334392	0.035506722	0.033905965	0.070058352	0.13780663	0	0.032826015	0	0	0	0.279296254
C20orf203	0.046839799	0.029135161	0.029745533	0.03787268	0.029345473	0.05772332	0.01926712	0	0.050973413	0.133540541	0.030272478	0.087742007
C20orf204	0.127467657	0.188778754	0.154186887	0.239257933	0.171127305	0.093503314	0.131081616	0.178182499	0.257616698	0.31948223	0.274607056	0.322159486
C20orf27	30.73162759	36.19002466	36.42257132	40.30171289	25.19560025	34.14028331	30.79182795	47.62202964	9.410013078	13.19750393	13.97122499	10.67079994
C20orf78	0	0	0	0	0	0	0	0	0	0	0	0
C20orf85	0	0	0	0	0	0	0	0	0	0	0	0
C20orf96	8.198596027	9.436120088	8.464996724	8.9627299	10.48261168	11.8562551	9.084878308	11.19857058	14.45748013	18.12696575	14.2741509	14.87035436
C21orf140	0	0	0	0	0	0	0	0	0	0	0	0
C21orf2	2.260477446	2.960117639	1.96078748	3.658888475	2.715281172	3.665412602	2.30708849	4.490300268	2.552444559	2.460068672	2.617979666	2.352449004
C21orf58	6.443368047	7.572615385	6.968905729	11.22836523	12.93285544	11.82716929	6.320108655	12.45651993	11.48501826	12.37950063	12.07640908	12.93849139
C21orf59-T(	0	0	0	0	0	0.011436353	0	0	0	0	0	0.034767528
C21orf62	0	0.026122277	0	0	0	0	0	0	0	0	0	0
C21orf91	7.012179107	6.335137693	6.268684561	8.108225026	7.19786708	6.896114653	7.210988194	6.386578275	5.85259223	4.365867566	4.320723354	5.978163322
C22orf15	0	0	0	0	0	0.01858119	0	0	0	0.019840072	0	0.018829489
C22orf23	0.023590338	0.024455994	0.024968339	0.095370747	0.024632529	0	0.097036682	0	0.128360932	0.155206596	0.101642619	0.122750765
C22orf24	0.033673067	0.034908712	0.035640038	0.034033272	0.1406428	0	0	0	0.183223583	0.036923889	0.036271404	0.175215578
C22orf31	0	0	0	0	0	0	0	0	0	0	0	0
C22orf34	0.386109047	0.246324588	0.288636204	0.261978834	0.256560724	0.31056106	0.25822523	0.295906868	0.881503192	0.651360447	0.700926819	0.733389161
C22orf39	4.28776831	4.985730905	4.742658027	6.285723004	5.203228301	6.208371691	4.826434241	6.690285939	6.768039761	8.280944139	7.011160852	8.040042906
C22orf42	0	0.022852979	0.023331742	0	0	0	0.022669057	0	0.023989454	0.024172214	0	0
C22orf46	5.194822385	5.6310387	4.584877992	4.372476343	4.599778798	4.124260639	4.860678269	3.984844261	6.801105747	5.343792394	5.850851218	7.102585702
C2CD2	0.322212939	0.255857865	0.35554673	0.214796818	0.479617213	0.401305407	0.345448239	0.382369336	2.887252578	3.127254393	2.857839128	3.05357736
C2CD2L	3.162813829	2.837653833	2.923599661	3.837663107	3.110838572	2.888147416	2.892052043	4.246203897	7.882842297	7.311491176	7.245212957	10.92534348
C2CD3	4.948142645	4.684729032	4.606196582	5.742196507	5.390845071	5.081562221	4.450845544	5.285126641	6.396902444	5.112056364	5.818003935	8.065429702
C2CD4A	0	0	0	0	0	0	0	0	0	0.01712754	0	0.016255123
C2CD4B	0	0	0	0	0	0	0	0	0	0	0	0
C2CD4C	0.012367171	0	0	0.012499464	0.090394758	0.06350317	0.012717805	0.01210134	0.444132954	0.650933112	0.546180154	0.321758784
C2CD4D	0	0.023667425	0	0	0	0	0	0	0	0	0	0
C2CD5	11.01520293	11.32781015	10.90010356	16.39195375	15.83809595	14.76031953	11.31740981	13.22807559	36.59223325	35.55764431	34.68607699	37.46541909
C2CD6	0	0	0	0	0	0	0	0	0	0	0	0
C2orf15	0.329110464	0.409424746	0.174167527	0.332631001	0.240555101	0.236589119	0.304597254	0.193221755	0.035815448	0.072176605	0	0.102750273
C2orf16	0	0	0	0	0	0	0	0.008491038	0	0	0	0
C2orf27A	2.403655309	2.743138116	3.612995511	2.572271488	3.522220485	3.464150368	3.074175993	2.865868846	1.077085059	1.129588248	1.000840228	0.861844915
C2orf27B	0	0	0	0	0	0	0	0	0	0	0	0
C2orf40	0	0	0	0	0	0	0	0	0	0	0.052884766	0.102187772
C2orf42	2.091706965	1.671868336	2.061792083	2.32387664	2.45087816	2.459664328	2.397309944	1.874881721	3.21462242	2.64381618	3.233471896	3.938201491
C2orf48	1.471134156	3.255540225	2.575152046	6.147639984	6.853489633	5.549288952	2.327451856	5.758048944	2.27829181	4.746408662	3.656889821	3.003095521
C2orf49	3.091689681	3.151313541	3.067456818	2.590451452	2.720623934	2.675769573	3.077411968	2.540272858	3.569999005	3.648954729	3.42177426	3.954308207
C2orf50	0	0.012009499	0	0.02341665	0	0	0	0	0.063033646	0.088919402	0.1372613	0.024111474
C2orf54	0.019411818	0	0	0	0	0	0	0	0	0	0	0
C2orf66	0.143110534	0.049454009	0.050490054	0.096427604	0.074716488	0	0.024528	0.070017192	0.077870023	0.052308843	0.205537956	0.099288828
C2orf68	4.969374917	5.163849596	4.343856201	5.967950922	6.727276411	6.796484039	5.062169856	6.590197026	13.80610798	14.3087531	11.41097425	14.1518295
C2orf69	6.986005219	5.701113296	6.920496226	8.111092742	9.223798488	8.375042674	7.584836584	7.146561623	9.990088919	9.67051307	9.452981713	9.928989306
C2orf70	0	0.025466387	0.01299995	0	0	0	0	0	0	0	0	0
C2orf71	0	0	0	0	0	0	0	0	0	0	0	0
C2orf72	0	0	0	0	0	0	0	0	0.555984569	1.15339464	0.874038536	0.688062426
C2orf73	0	0	0	0	0	0	0	0	0.019716585	0	0.019515725	0.018854848
C2orf74	0.052962166	0	0.056055887	0	0	0.054390215	0	0	19.19281457	17.94522803	17.85631225	15.15718226
C2orf76	2.128644819	1.885773474	2.348568301	2.477387205	1.980210836	2.278781797	2.202262735	2.53733843	1.51625312	1.640975168	1.20898303	1.369428838
C2orf78	0	0	0	0	0	0	0	0	0	0.03678577	0	0
C2orf80	0	0	0	0	0	0	0	0	0	0	0	0
C2orf81	0.366051434	0.36050963	0.309947109	0.27747535	0.229333872	0.488697933	0.319965256	0.322364867	0.936135402	1.284448965	0.788594608	0.990456714
C2orf83	0	0	0	0	0	0	0	0	0	0	0	0
C2orf88	0.066856039	0.049506675	0.040435059	0.019306059	0.009972808	0.029425164	0.06875154	0.009345568	0.041574907	0.115202009	0.061727054	0.039757827
C2orf91	0	0	0	0	0	0.005375008	0.005382275	0	0.039870421	0.114783337	0.13530599	0.021787335
C2orf92	1.205100447	1.76040832	1.120889486	0.719722285	0.838893498	1.143837113	1.314374534	0.857597747	1.231968761	1.601747522	1.081742009	1.121118097
C3	23.40275523	25.4572122	17.57856695	21.16274	0.448881283	0.452248492	9.714923277	10.57778042	3.103633266	2.241982268	1.863538797	11.43547272
C3AR1	1.747354599	1.749543645	1.217142426	0.166038539	0.374266769	0.322084271	1.674030891	0.306886343	1.430232201	0.327529139	0.611308559	0.186507573
C3orf14	0.025538472	0	0	0	0	0	0	0	2.417925185	1.79225437	2.338274709	1.807273432
C3orf18	0.612608848	0.777659689	0.741021318	0.391714737	0.404691161	0.449376406	0.424270572	0.428172047	0.925176924	1.343501089	1.117755902	0.689577518
C3orf20	0.185763731	0.146727922	0.196614895	0.116226729	0.138550383	0.14535054	0.054580143	0.173115004	0.385061694	0.213397376	0.219154888	0.349820509
C3orf22	0	0	0	0.007680436	0.015869736	0	0	0	0.008269772	0.008332774	0.008185525	0.015816664
C3orf30	0	0	0	0	0	0	0	0	0	0	0	0
C3orf33	0.857458426	1.1908216	1.061657378	0.948388388	0.996699074	1.046725463	1.214512103	0.633228242	1.12679749	1.224083533	0.923623059	1.582650779
C3orf35	0.090248869	0.159603347	0.123614955	0.096579818	0.144125569	0.190816501	0.087348331	0.109087559	0.109767846	0.186280582	0.114368002	0.066297039
C3orf36	0	0	0	0	0	0	0	0	0	0	0	0
C3orf38	12.45534875	12.99721269	10.45541367	13.80816899	13.41136813	12.64416422	13.10293029	11.72733409	19.38587682	14.9809662	17.0294173	18.53859403
C3orf49	0	0	0	0	0	0	0	0.014010891	0	0	0	0
C3orf52	0	0	0	0	0	0	0	0	0	0.076728705	0	0
C3orf56	0	0	0	0	0	0	0	0	0	0.03391056	0	0
C3orf58	4.686223458	4.931058874	4.897963992	5.269192255	6.361231284	5.402121902	4.397416999	5.055506508	9.052114634	6.962850184	7.407689973	10.88765876
C3orf62	7.119649418	6.774896117	7.361028001	4.575019754	5.055246785	4.340793681	6.735785059	4.971961755	3.784266195	2.021598341	2.922303165	3.291300182
C3orf67	0	0	0	0	0	0	0	0	0.193322339	0.129863424	0.127568598	0.061624319
C3orf70	0	0	0	0	0	0	0	0	0.507991037	0.569651858	0.592025237	0.63465941
C3orf79	0	0	0	0	0	0	0	0	0	0	0	0
C3orf80	13.37933612	6.275172728	12.41561796	6.792872106	10.63582534	6.494925891	13.15766405	5.371497343	5.633222557	2.38031611	4.991271611	5.62595609
C3orf84	0	0	0	0	0	0	0	0.032342094	0	0	0	0
C3orf85	0	0	0	0	0	0	0	0	0	0	0	0
C4A	0.01983397	0.174775169	0.115459011	0.080184546	0.051775524	0.1731345	0.061188907	0.135853483	0.312972623	0.369728848	0.128186591	0.144486654
C4B	0.039660631	0.010278998	0.01049434	0.020042443	0.124238361	0	0.010196272	0.009702032	0.172642733	0.250064605	0.202924704	0.123822883
C4BPA	0	0	0	0	0	0	0.046598036	0	0.024656108	0.024843947	0	0
C4BPB	0	0	0	0	0	0	0	0	0	0	0	0
C4orf17	0	0	0	0	0	0	0	0	0	0	0	0
C4orf19	0	0	0	0	0	0	0	0	0.034090093	0.034349803	0.033742805	0.06520029
C4orf3	18.14302628	19.08404163	17.23571013	14.15087775	13.12257468	12.27000314	16.56414565	13.47499284	15.66048125	16.96375763	14.98615101	16.5233593
C4orf33	4.05759626	4.096517562	4.196372994	4.570069614	4.721463364	3.812944406	4.349906001	4.13905474	5.800990473	5.205413015	4.656361605	4.885069046
C4orf36	0.825300013	0.484831324	0.727924096	0.333651341	0.746859287	0.480280059	0.594089226	0.3230241	0.688568462	0.54298504	0.503757138	0.458068702
C4orf45	0	0	0	0	0	0	0	0	0.03012256	0	0	0.028806018
C4orf46	8.096593034	7.793021252	9.505578971	10.23285689	12.4664957	11.66592282	8.200706376	8.907282009	10.17177999	11.60358056	11.776294	11.90115447
C4orf47	0.065243481	0.118365824	0.1381092	0.032970699	0.136251709	0.083753347	0.016773316	0.047880808	0.284004884	0.751192398	0.263542151	0.016974507
C4orf48	3.165268286	3.90916863	4.194987472	3.894590079	2.672824692	2.346096195	4.019229585	5.197961722	3.714181011	3.410483019	3.913526794	5.098620968
C4orf50	0	0	0	0	0	0	0	0	0	0	0	0
C4orf51	0	0	0	0	0	0	0	0	0	0	0	0
C4orf54	0.009929795	0	0	0	0	0	0	0	0	0	0	0
C5	2.15788461	2.299457325	2.04735194	3.058568357	2.962397116	2.834096054	1.768179194	2.776077065	12.71459839	9.653375832	8.982721289	13.45619809
C5AR1	0.159537029	0.141763969	0.144733879	0.230348025	0.071393645	0.117027655	0.11718587	0.111505566	0.347232359	0.24991264	0.540092114	0.284619562
C5AR2	0.576451826	0.965361809	0.422393928	0.224083925	0.532466571	0.318766551	0.387596973	0.216946553	0.820346411	0.680726198	0.716461108	0.946005296
C5orf15	31.44592049	28.71133664	29.1130833	29.4457182	30.8399263	27.30317265	34.1083937	28.83099846	44.23317708	44.337351	38.95453385	54.08398691
C5orf22	18.7085765	17.43700448	21.27521269	23.07502926	18.80091187	17.34411011	20.57169577	20.15461822	32.87244203	36.16153304	30.29512566	42.68427467
C5orf24	6.831490782	5.882619405	6.182213143	6.530336777	7.326611623	6.720995047	7.177484359	6.032487573	12.01789105	9.815453293	10.19041134	12.38854015
C5orf30	3.241896514	3.575708488	4.136323044	3.306498559	5.502751118	4.72792369	3.927502975	3.403971982	4.301252995	4.739828696	4.815525311	4.067045198
C5orf34	2.706023283	3.378323655	3.339409817	5.178985668	5.951736641	5.770833251	3.777427467	5.036563013	8.959801798	11.04832603	9.339859374	7.645473456
C5orf38	0	0	0	0	0	0	0	0	0	0	0	0
C5orf46	0	0	0	0	0	0	0	0	0	0	0	0
C5orf47	0	0	0	0	0	0	0	0	0	0.018415847	0.018090419	0

C5orf49	0	0	0	0	0	0	0	0	0	0	0	0
C5orf51	2.610760175	2.933831586	2.204283573	2.497689225	2.892579633	3.243993586	2.090439675	1.608839477	6.354655303	6.916623882	5.463427252	6.865050191
C5orf52	0	0	0	0	0	0	0	0	0.032370512	0	0.096122228	0
C5orf56	1.403727675	1.178049775	1.627222323	1.756539607	1.116756246	2.059395971	1.649744136	0.850295737	2.909727059	2.125623424	1.800052909	2.365170883
C5orf58	0	0	0	0	0	0	0	0	0	0	0	0
C5orf60	0	0.021646923	0	0.021104062	0.043606363	0	0	0	0	0.022896536	0.04498386	0.021730267
C5orf63	0	0	0	0	0	0.008539709	0	0	2.425222206	2.352515621	1.845172455	2.232686741
C5orf64	0	0	0	0	0	0	0	0	0.023493784	0.02959096	0.04650889	0.02246696
C5orf66	0.043500049	0.067644448	0.046041052	0.021982687	0.045421826	0.044672966	0.022366681	0.148977566	0.236694653	0.143098725	0.093713345	0.339524447
C5orf67	0.087816501	0.113798698	0.069709647	0.022188971	0.091696122	0.06763826	0.022576568	0.021482223	0.047783155	0.120367961	0.11824093	0.137084208
C6	0	0	0	0	0	0	0	0	0	0	0	0
C6orf10	0	0	0	0	0	0	0	0	0	0	0	0
C6orf106	30.25418635	31.38828017	30.52044269	37.3016766	29.30333573	29.95692311	29.65357936	35.97818479	44.63075217	51.53242551	41.10847206	61.266419
C6orf118	0	0	0	0	0	0	0	0	0.03363484	0	0	0
C6orf120	21.8684488	18.40206134	20.59794492	16.27585338	22.90071778	18.35943184	20.92495399	16.30294343	36.29040089	36.27511689	29.69735511	38.31711797
C6orf132	0.023943738	0.008274121	0.008447461	0.024199867	0.016667695	0.008196449	0.00820753	0.01561938	0.112912688	0.052510568	0.025791325	0.033223909
C6orf136	10.25073494	12.03629104	11.91432615	13.90545493	11.75408521	13.43116754	12.52660698	14.60659811	2.633494128	3.428753436	2.694530943	3.56536698
C6orf141	0.071976406	0.014923521	0.030472328	0	0	0	0	0	0	0	0	0
C6orf15	0	0	0	0	0	0	0	0	0	0	0	0
C6orf163	1.196685019	1.411714736	1.615991494	1.042660441	1.034112858	0.720420089	1.188178451	0.726804225	2.155518665	1.49320886	1.644618892	1.460093988
C6orf201	0.018993844	0	0	0	0	0	0	0	0.020670052	0.041655047	0	0
C6orf203	13.11208711	12.03808099	12.7607161	13.89812001	13.19824129	12.86652907	14.39799928	12.8846092	7.981094406	7.493586004	8.707721097	10.55219312
C6orf222	0	0	0	0	0	0	0	0	0	0	0	0
C6orf223	10.30288126	21.24817808	8.162904518	4.417675605	11.15453772	13.60497412	7.74883251	4.153116347	16.42790056	20.19861208	24.19539833	3.284238383
C6orf226	2.047102859	1.920105638	2.785733868	2.955715685	1.933965895	2.702957213	2.205387126	2.766186452	5.092022353	4.917031196	4.410129626	5.782494989
C6orf47	12.50121111	13.16279882	11.11442156	13.97534029	11.30546494	10.51623272	12.92271799	12.99835928	13.29686484	10.87110897	11.80311056	17.53500873
C6orf48	261.914909	255.3096546	245.2280804	203.3119966	239.3165621	250.0214468	239.6141456	227.8157515	121.3381559	145.3154243	125.2242388	126.7789024
C6orf52	0.168154878	0.104595228	0.177977442	0.305916573	0.175583746	0.069075573	0.13833792	0.131632321	0.731978213	1.106332019	1.014329814	0.524989677
C6orf58	0.089607928	0.139344184	0.047421133	0.045283238	0.046783346	0	0	0.131522719	0.146273748	0.294776225	0.096522404	0.046626893
C6orf62	77.51066247	75.82790452	71.13158332	72.38981486	64.76290271	64.96299642	73.87465245	68.75629833	29.19030296	24.5683743	27.36530946	24.23008858
C6orf89	20.21793821	18.98200631	18.40669108	23.06883175	16.46287664	17.08379728	18.84866296	19.37672122	25.13359313	19.33901855	20.23165949	32.74356395
C6orf99	0.248953685	0.184349378	0.075284575	0.143781014	0.03713602	0.073047533	0.036573145	0.139201397	0.77406817	0.428980911	0.574636851	0.51816562
C7	0	0	0	0	0	0	0	0	0.049142545	0	0.029185148	0.03759577
C7orf25	2.09101567	1.861434261	2.044767227	1.86069637	2.04099881	1.913984204	2.138613669	1.968229844	2.547624278	2.342729119	2.595117461	3.074913136
C7orf26	8.888442948	7.745036476	9.52930125	12.95253748	9.22111576	9.344506166	8.923756158	13.62715518	8.401202419	8.108112673	7.902930759	12.36002751
C7orf31	3.58903163	3.59984219	3.071857215	3.391706879	3.869353337	3.499518566	3.584194597	3.765451412	2.918747703	1.903825226	2.372619702	2.750728476
C7orf33	0	0	0	0	0	0	0	0	0	0	0	0
C7orf43	10.61033594	9.559250901	10.00461612	10.97916549	8.243368344	7.999362156	9.35242274	11.96844732	8.018540678	5.355639612	6.848370307	10.95440118
C7orf50	2.84536326	3.951074596	3.997009555	5.602094502	4.497487924	4.843332559	4.151927215	5.926008751	7.32925145	7.012971401	7.592008403	7.334913981
C7orf55-LU(	0	0	0	0	0	0	0	0	0	0	0	0
C7orf57	0	0	0	0.025485084	0	0	0.025930257	0	0.109762431	0.02764966	0.054322122	0.104965134
C7orf61	1.286284323	1.166799358	0.623984656	0.541684946	0.895407149	0.660483569	1.543211853	0.681761024	2.274673332	2.468310481	1.270077172	1.840601257
C7orf65	0	0	0	0	0	0	0	0	0	0	0	0
C7orf66	0	0	0	0	0	0	0	0	0	0	0	0
C7orf69	0	0	0	0	0	0	0	0	0	0	0	0
C7orf71	0	0	0	0	0	0	0	0	0	0	0	0
C7orf77	0.080432826		0	0	0	0	0	0.078703929	0.262593081	0.088197869	0	0
C8A	0	0	0	0	0	0	0	0	0	0	0	0
C8B	0	0	0	0	0	0	0	0	0	0	0	0
C8G	1.043058762	0.699686817	1.363749609	1.178241965	0.384402271	0.630107851	1.325015422	0.780488105	1.468964259	1.412875555	0.859181468	1.787878184
C8orf33	8.158082474	7.153806096	8.687600171	9.889362131	9.674764419	9.328936849	10.82770463	10.02738014	7.67296311	8.87706783	10.02081774	7.897531018
C8orf34	0.018424777	0.019100881	0.009750519	0.009310935	0.01442907	0	0.009473578	0.018028738	0	0	0	0
C8orf37	1.19123	1.387840261	0.924597124	1.375968845	1.066163162	1.269953612	1.085003292	1.087916293	1.123508622	1.592359249	1.197606359	1.345957088
C8orf44	3.384427982	3.066357641	1.926521143	1.753433026	2.464854274	2.891535623	2.632222574	1.753242328	4.271162513	3.898280546	2.787798643	4.883624899
C8orf44-SGI	0.011792584	0.012225317	0	0.023837462	0	0	0	0.011539103	0.03849984	0.051724194	0.012702543	0.061361932
C8orf46	0.062117819	0	0	0	0.016215526	0	0	0	0.01689993	0	0.016727764	0.032322597
C8orf48	0	0	0	0	0	0	0	0	0	0	0	0
C8orf49	0.027342257	0	0	0	0	0	0	0	0	0	0	0
C8orf58	2.635676841	3.002927987	2.706776221	2.479246289	2.397884714	2.465549073	3.48863813	3.064185935	3.720238446	4.20642242	3.85099583	5.730246195
C8orf59	35.20368938	35.16188662	34.66979806	37.07070676	38.75742436	36.76512141	38.97660805	40.61797328	37.9006795	42.69645981	33.59413028	34.74217397
C8orf74	0	0	0	0	0	0	0	0	0	0	0	0
C8orf76	1.92463407	1.704283897	1.824865571	1.70206936	2.386473563	0.988264535	2.061667954	1.687090848	1.70176753	1.27505727	1.598049952	1.210110333
C8orf82	7.8820423	8.431140546	7.251751657	7.234466009	8.026685519	7.350899509	7.103065028	9.892863862	8.668561863	8.123790671	8.790258371	9.304164869
C8orf86	0	0	0	0	0	0	0	0	0	0	0	0
C8orf87	0	0	0	0	0	0	0	0	0	0	0	0
C8orf88	0.138565393	0.143650099	0.19554603	0	0.048229012	0.04743387	0.047497998	0.045195647	8.243394127	9.065908162	10.19926974	5.527788297
C8orf89	0	0.062051303	0	0	0	0	0.061551911	0	0	0	0.06447353	0
C9	0	0	0	0	0	0.019519766	0	0	0	0.062526712	0.020473933	0.039561214
C9orf116	0.819813254	2.163373141	1.498757403	1.657165802	0.856031558	0.994994406	1.609471642	1.385603377	1.865429242	1.634470204	2.408381038	1.551216034
C9orf129	0.071035724	0.036821202	0	0	0.185434979	0.0729511	0	0.034754408	0.15460926	0.077893563	0.03825855	0
C9orf131	0	0.015384479	0	0	0.015495532	0	0.015260664	0.01452094	0.032299094	0	0.015985026	0.015443712
C9orf135	0	0	0	0	0	0	0	0	0	0	0	0
C9orf139	5.130197551	5.735819268	5.076808423	5.254830314	5.573038599	6.308055766	4.8024964	6.066679856	3.968141832	8.021971374	4.398547413	6.021258255
C9orf147	0.111908965	0.135351422	0.098704994	0.037702023	0.116852961	0.057463215	0.153442407	0.018250582	0.121784937	0.061356368	0.080362847	0.116462181
C9orf152	0	0.020963593	0	0	0	0	0.020794877	0	0	0	0	0
C9orf153	0.02730064	0	0	0.027592678	0.028506747	0	0	0.106855258	0.059419845	0.059872526	0	0.028411415
C9orf16	29.08624911	29.96511942	27.96330253	36.5017573	22.96825214	28.50145273	26.48361075	42.2172128	25.91598539	27.11011794	25.32560926	36.76504282
C9orf163	0.04182632	0.043361152	0.044269554	0.021136871	0.109185384	0.021477053	0.064518268	0.081854533	0.159311232	0.137592789	0.135161377	0.195876444
C9orf170	0	0	0	0	0	0	0	0	0	0	0	0
C9orf24	0.007976514	0.016538429	0.016884904	0.024185518	0.008328906	0.008191589	0.02460799	0.007805059	0.052082648	0.04373286	0.034368042	0.074709471
C9orf3	0.403392421	0.362435708	0.426956102	0.392607281	0.433694192	0.355964749	0.362591615	0.350863583	0.390214952	0.386634616	0.399114352	0.435353501
C9orf40	7.452334881	7.221944357	9.308411568	10.76007637	11.84151885	10.22021384	6.592620001	12.38759217	7.480345096	9.491456347	9.024574935	8.935738266
C9orf43	0.080916633	0.167771795	0.08564328	0.102227761	0.12673714	0.083098434	0.187224252	0.039588668	0.132086294	0.088728382	0.217901142	0.08420887
C9orf47	0.241791327	0.330420655	0.255915275	0.544296009	0.229521236	0.395040043	0.203438117	0.322628237	0.131564712	0.313340223	0.142062999	0.400318926
C9orf50	0	0	0	0	0	0	0	0	0.202857703	0.045422921	0.044620249	0
C9orf57	0	0	0	0	0	0	0	0	0	0	0	0
C9orf62	0	0	0	0	0	0	0	0	0	0	0	0
C9orf64	8.293440133	7.523049491	8.4673528	7.571617439	8.087957927	7.673389739	8.307315279	7.215613951	7.045722678	7.077951121	7.627094411	7.348456009
C9orf66	0.096571358	0.240276188	0.286194881	0.312334062	0.181507964	0.396701067	0.218480562	0.283486692	1.156031636	0.931870952	1.081840844	1.507508045
C9orf72	5.201669518	5.551510577	4.431880176	5.233469753	4.507752806	5.06332128	5.167203314	4.062654706	2.46452989	3.582685597	2.718940066	2.958293428
C9orf78	52.34095931	46.28376909	55.02534001	61.65829431	45.39108186	50.58503767	60.62100542	64.06297884	26.33843003	27.5697295	29.07576728	33.10415253
C9orf84	0	0	0	0	0	0	0	0	0	0.00653933	0	0
C9orf85	4.701542439	4.169722228	4.889885263	3.488341622	3.944426773	3.823577184	5.449675624	4.520705967	4.110907945	3.754823822	3.629924876	3.733259923
C9orf92	0	0	0	0	0	0	0	0	0	0	0	0
CA1	0	0	0	0	0	0	0	0	0	0.078099768	0.076719661	0
CA10	0	0	0	0	0	0	0	0	0	0	0	0
CA11	0.729522246	1.028557558	0.617709125	0.088479125	0.457050965	0.869063611	0.480131608	0.45685838	1.778341863	2.879823043	2.828933476	2.065035315
CA12	0.217334886	0.212056533	0.419466909	0	0.013349204	0.013129118	0	0	0.055650518	0.238316551	0.096396276	0.013304561
CA13	1.968131798	1.635176589	1.891039172	1.523631183	2.098806486	1.691500418	2.052640469	1.720951743	2.187390147	1.132639093	1.5185816	2.411366331
CA14	0.204017292	0.141002519	0.197940156	0.120283153	0.071010172	0.104759163	0.122384258	0.066543985	0.18501819	0.149142178	0.073253338	0.123852225
CA2	1.453439519	1.439806262	0.957189602	0.816103971	3.574910357	3.81449744	1.428218619	1.643428735	1590.035939	1493.596924	954.0719084	3156.509359
CA3	0.153478496	0.54097551	0.324887515	0.093072165	0.41667335	0.252186919	0.063131966	0.210251283	0.334045962	0.201954504	0.264514335	0.766670606
CA4	0	0	0	0	0	0	0	0	0	0	0	0
CA5A	0	0	0	0	0	0	0	0	0	0	0	0
CA5B	0.919749433	0.866022902	0.839510968	0.904003116	0.740111508	0.979211513	0.867730404	0.776129099	0.881541897	1.147332981	1.03616657	1.413803154
CA6	0	0	0	0	0	0	0.033577165	0	0	0	0	0
CA7	0	0	0	0	0	0	0	0	0.057241698	0.028838893	0	0
CA8	0	0	0	0.005639275	0	0	0	0	0.139655735	0.097891952	0.048081048	0.1161321
CA9	0	0	0	0	0.072219536	0	0	0	0	0.037920549	0	0
CAAP1	13.61097346	12.34428021	14.36767706	12.78573516	14.87464592	13.94074889	13.71730669	12.18341588	10.8280509	10.19509735	11.24484382	9.958712895
CAB39	18.45658842	16.59525035	18.94970321	17.45190432	18.89208962	16.48257599	21.19586589	15.24614112	19.52957084	18.33577619	18.75032099	21.01323912
CAB39L	1.223929222	0.988124539	1.318351419	1.521646223	1.266691112	1.357040247	1.247491707	1.409589265	4.856280578	5.000169404	3.663441012	5.004732505
CABCOCO1	0	0	0	0	0	0	0	0	0	0	0	0
CABIN1	7.056058741	6.96471949	6.482001943	8.389622175	7.609170548	7.932962063	6.27229431	8.181562095	10.19060746	10.12004967	9.002643992	11.01472062
CABLES1	4.312980069	3.20439325	4.141032381	2.667364185	4.128228525	3.016124265	3.548209238	3.215446005	3.24081957	2.049388575	3.008699121	3.25947789
CABLES2	3.340884228	3.846672593	3.460803091	5.445701162	3.948662731	3.868962093	3.172452161	5.80086423	9.205327068	7.046229116	8.789965099	11.37731921
CABP1	0.026385859	0.045590162	0.065163365	0.00888937	0.018367701	0.009032438	0.045223249	0.017212464	0.067000294	0.077155115	0.056843776	0.036612552
CABP2	0	0	0	0	0	0	0	0	0	0	0	0
CABP4	0	0.007962335	0	0	0	0	0	0	0	0	0.008273152	0
CABP5	0	0	0	0	0	0	0	0	0	0	0	0
CABP7	0.018044789	0	0	0	0	0.018531341	0	0.088284588	0.019637242	0	0.019437191	0.018778973
CABS1	0	0	0	0	0	0	0	0	0	0	0	0
CABYR	0.041664391	0	0.044098166	0.02105504	0.021752535	0.042787812	0	0.040768818	0.090682551	0.068530052	0.089758736	0.086719162
CACFD1	0.102256345	0.05889371	0.060127514	0.057416774	0.011863767	0.058340856	0.05841973	0.111175947	0.581131352	0.274091266	0.257009243	0.804038243
CACHD1	3.284885983	2.9189366	3.233297434	3.031731355	2.853536114	2.797040994	3.226623161	2.088830262	3.46463012	2.673761833	2.814829658	2.527994065
CACNA1A	0.33234774	0.40196725	0.469015241	0.211494422	0.231353626	0.284424183	0.424048524	0.204758042	1.734712179	1.464480068	1.637486081	1.172114662
CACNA1B	0.01099276	0.03418843	0.011634889	0.011110351	0.005739203	0.005644582	0	0.005378236	0.005981436	0.006027005	0.005920501	0.01144002
CACNA1C	0	0.003466575	0	0	0	0	0	0.003271994	0	0.00366669	0	0.027839373
CACNA1D	0.005319253	0	0	0.005376153	0	0.005462679	0	0.015614747	0.133139572	0.087491658	0.131783233	0.332140563
CACNA1E	0.500611547	0.576646293	0.320529063	0.190518307	0.141975486	0.215799184	0.206557509	0.117927078	0.72302448	1.050442546	0.718987423	1.009800393
CACNA1F	0.026278477	0.063566474	0.018542335	0.026559581	0.027439426	0.035982718	0.027023523	0.017142415	0.009532518	0.00960514	0	0
CACNA1G	0.005097533	0.010569178	0.005395299	0.015456187	0.015968207	0	0	0	0.077663471	0.173279235	0.098835796	0.100793774
CACNA1H	0	0	0	0	0	0	0.005825981	0	0.129471825	0.273340964	0.146460401	0.047166897
CACNA1I	0.003523874	0	0	0.007123139	0.003679555	0.003618891	0	0	0.015339426	0.030912573	0	0.007334499
CACNA1S	0	0	0	0	0	0	0	0	0.009489265	0.009561558	0.009392595	0.009074526
CACNA2D1	0.011098569	0.007670556	0.007831252	0	0	0.007598551	0.003804412	0.003620002	0.136884204	0.202833875	0.183309616	0.123201427
CACNA2D2	0	0	0	0	0.009580017	0	0.009434811	0	0.03993743	0.070422953	0.108709078	0.105027773
CACNA2D3	0	0.006991368	0	0.034080191	0	0.020777212	0.013870201	0.032994691	0.939398033	0.591596685	0.602935381	1.150998798

CACNA2D4	12.48377254	11.05758956	12.71245902	5.332556769	10.92322769	9.970756184	12.32209424	5.324390782	3.168495273	3.055182026	3.136216706	2.792829453
CACNB1	4.200708758	5.645571383	4.006836006	3.611355251	5.295256982	5.98758919	4.413489688	4.466980939	4.945947596	6.404349887	6.323887909	4.814051311
CACNB2	0	0.005294118	0.005405028	0	0	0	0	0.004996956	0.011114787	0.027998659	0.005500779	0.021258005
CACNB3	4.847208164	4.72779302	3.984834449	2.795455632	3.670445845	3.229939129	3.719451717	2.869344727	1.832147634	1.75481464	1.763661947	2.531839504
CACNB4	0.973484612	1.526184933	0.710295458	0.42626648	0.642577999	1.288484517	0.692848893	0.376351898	1.298985294	1.835342611	1.348611068	0.590740627
CACNG1	0	0	0	0	0	0	0	0	0	0	0	0
CACNG2	0	0	0.011630137	0	0	0	0	0	0	0	0	0
CACNG3	0	0	0	0	0	0	0	0	0	0	0	0
CACNG4	0	0.016263592	0	0	0	0	0	0	0	0	0	0
CACNG5	0	0	0	0	0	0	0	0	0	0	0	0
CACNG6	0	0.022171749	0	0	0	0	0	0	0	0	0	0
CACNG7	0.020176063	0	0	0	0	0	0	0	0.043913204	0	0	0
CACNG8	0	0.0063746	0.006508146	0	0	0	0.006323297	0.00601679	0.020074822	0.013485173	0.026493751	0.012798286
CACTIN	12.183062	12.07537879	12.93423479	15.10559378	13.48795027	13.84067452	13.82099475	16.57276491	8.626644253	6.454456306	8.431524422	8.741733745
CACUL1	11.48043747	11.14854776	10.29206048	10.18309833	11.07896964	10.9291085	10.61548547	9.249416489	11.59870522	12.7375912	11.24835436	11.90599733
CACYBP	35.56779995	40.4894476	42.06075205	54.81120044	54.25926664	52.05111265	51.26948293	56.20863661	32.50600169	32.1479803	35.36885158	31.08528825
CAD	26.99452286	25.49918625	27.21879421	34.24197607	33.80203803	36.85750932	25.99534704	35.93325262	12.88359785	15.33562658	16.08543611	14.08377909
CADM1	0	0.060582235	0	0.011812591	0.048815637	0.036008118	0.072113598	0.091490753	0.699544774	0.884296662	0.553934614	1.787975267
CADM2	0	0	0	0	0	0	0	0	0.156790256	0.234051465	0.086218322	0.249895919
CADM3	0	0	0	0	0	0	0	0	0	0.014608659	0	0
CADM4	0.146753348	0.177494933	0.258876279	0.173043718	0.051078908	0.226065512	0.25152349	0.31113094	0.399260844	0.885065615	0.685001952	0.941799662
CADPS	0.00687223	0	0	0	0	0	0.014134142	0.006724512	0	0.00753568	0	0
CADPS2	0.007364111	0	0	0	0	0	0	0	2.860996	2.559790156	1.935494142	4.291690618
CAGE1	0	0.081436674	0.049885648	0.047636644	0.065619619	0	0.032312507	0.076865589	0.034194603	0	0.03384625	0.032700087
CALB1	0.16981321	0.132033424	0.449331608	0	0	0	0	0	0	0.093103549	0	0
CALB2	0	0	0	0	0.038248331	0	0	0	0	0	0	0
CALCA	0	0	0	0	0	0	0	0	0	0	0	0
CALCB	0.052191621	0	0	0.052749921	0.05449738	0	0.053671356	0	0	0	0	0.05431513
CALCOCO1	14.02123414	13.67507898	9.779606161	8.204391149	10.38653106	8.90482818	9.691559403	8.589949164	20.52885183	10.87366327	12.35412312	23.69553405
CALCOCO2	25.62897083	24.77183293	24.08220828	26.88624919	24.37656236	24.03258088	24.58696482	23.38137635	35.14722739	37.91923769	35.18398412	32.99489743
CALCR	0	0	0	0	0	0	0	0	0	0	0	0
CALCRL	0.051312359	0.026597642	0.045258091	0.034574169	0.017859758	0	0.035178111	0	0.018613559	0.018755364	0.055271809	0
CALD1	0.075456001	0.107559218	0.119795508	0	0.009848694	0	0	0.00922926	0	0.09308315	0	0
CALHM1	0	0	0	0	0	0	0	0	0	0	0	0
CALHM2	7.443689723	8.032240666	6.053782257	4.161398125	7.221899108	7.686056996	8.572466641	5.993661649	14.70026355	11.34271802	15.18408746	10.04729583
CALHM3	0.032572373	0.067535256	0	0.032920804	0.034011379	0.133802563	0.100487593	0	0	0	0	0
CALHM4	0	0	0	0	0	0	0	0	0	0	0	0
CALHM5	0	0.005700983	0	0	0	0	0	0	0.017953477	0.006030084	0.011847052	0.028614665
CALHM6	0	0	0	0.044541498	0	0	0	0	0.623470386	0.724869464	0.901942973	0.45863144
CALM1	44.34888456	47.40998661	44.01199163	53.876896	44.51403497	45.49089151	46.27424962	50.75635672	43.40562396	42.23132401	41.5076219	53.94909491
CALM2	57.94364247	53.61250501	61.16411982	74.76140375	74.37951695	66.68816205	67.74711388	78.96312485	91.65482913	99.83953353	90.97181913	89.81738934
CALM3	89.51861039	115.095925	99.65109506	155.1997326	123.8678109	128.6463475	110.2276544	167.9879142	255.5593878	275.1514462	258.7852064	238.6513072
CALML3	0	0	0	0	0	0	0	0	0	0	0	0
CALML4	5.42158898	4.72991582	5.037894355	5.151044016	7.515817724	7.081922845	4.656033543	4.702980006	6.55700872	4.964769363	4.764489556	7.249048393
CALML5	0	0	0	0	0	0	0	0	0	0	0	0
CALML6	0.359849939	0.124351586	0.30469611	0.04849324	0.125249217	0.123184259	0.246701596	0.046948665	1.096498795	0.473508131	0.568505341	1.02360894
CALN1	0.011686721	0.028269663	0.004123129	0	0.004067675	0.012001837	0.020030105	0	0.012718075	0.017086621	0.008392341	0
CALR	664.6086159	681.728089	756.9266036	757.465017	413.0361838	517.4125511	712.6393598	772.4358166	541.3957539	632.6219916	630.2994054	529.952949
CALR3	0	0.043176565	0	0.042093784	0.043488234	0	0	0	0.045323728	0.04566902	0.044862	0.043342801
CALU	22.11178606	20.94874419	22.96010937	22.5240381	19.68064543	18.85299534	20.61761212	20.61575957	25.18976868	34.99187305	29.67462167	32.0252184
CALY	0.020221556	0.06289078	0.064208322	0.102189343	0.042229837	0.041533603	0.041589754	0	0.154042766	0.110868798	0.043563851	0.399841818
CAMK1	12.37842648	13.85531113	14.61709467	7.332831721	11.76234578	9.673031186	15.41269312	10.71118116	47.51148947	43.65137334	46.75326067	44.34212037
CAMK1D	4.385775422	4.98948191	4.141313448	5.091792264	3.519767022	4.461585201	3.612819273	4.192641643	5.1126651	3.776843967	4.437283279	5.964549437
CAMK1G	0	0	0	0	0	0	0	0	0	0	0	0
CAMK2A	0	0	0	0	0	0	0	0.010727981	0.083518297	0.012022081	0.011809638	0.011409719
CAMK2B	0.0317773	0.098830137	0.089689419	0.032117226	0.03318118	0.065268258	0.032678249	0	0.610942446	0.360066064	0.376522878	0.352748969
CAMK2D	5.034035735	5.241717882	4.874970581	3.991229794	5.125407557	4.813496734	4.243121926	3.770209682	14.94078987	12.35935249	12.05349009	12.98959368
CAMK2G	14.78039957	15.20186016	12.71477856	16.72812772	14.27089329	15.34226327	15.02499731	15.84256582	12.3116572	16.66074255	12.66591711	16.19830217
CAMK2N1	0.022887946	0.118639136	0.193799341	0.046265562	0.430183911	0.352576299	0.188294914	0.17916777	2.092255888	2.760732105	2.933833575	2.953578671
CAMK2N2	1.186975607	1.558673989	1.465696578	1.279651026	1.032845563	1.097082633	0.773064845	1.084030877	0.21528771	0.130156708	0.383570098	0.205878305
CAMK4	0.00442331	0.009171249	0.037453535	0.026823758	0.013856177	0	0.009097438	0.008656462	0.168478339	0.179462544	0.05717554	0.253180389
CAMKK1	2.053186324	1.134787903	1.276381146	0.725052242	1.407737283	0.647806778	0.915787171	0.695906994	0.491294422	0.58997593	0.632842414	0.508437307
CAMKK2	35.53748863	25.60755154	32.66212014	25.72629654	38.1232861	31.71764412	31.81271953	27.39839534	7.39227801	7.680493941	7.234961909	8.935526225
CAMKMT	0.535209088	0.548614518	0.639668588	0.547012201	0.5651332	0.633012629	0.544199237	0.535473455	0.337030648	0.422025029	0.369224085	0.319171194
CAMKV	0.21937503	0.227425074	0.265359494	0.079186326	0.245428649	0.32184309	0.306164295	0.21465958	0.170524919	0.395195285	0.438848077	0.342451041
CAMLG	15.48044904	17.46674997	14.07307798	15.25792643	16.13929452	18.38728907	15.6231763	16.06360525	6.895352304	10.79027363	8.453825645	9.116680676
CAMP	0	0.074878016	0	0	0	0.222525337	0.074275393	0.141350143	0.943220248	1.346408542	0.700208501	3.45765013
CAMSAP1	5.897896198	5.256383177	6.162225552	5.990438261	6.450558491	6.09882024	6.047133585	5.662770262	10.36328301	9.828736895	10.03799027	10.88682155
CAMSAP2	0.622346619	0.554996858	0.658700244	0.615476973	0.405277244	0.357361503	0.543648584	0.314306748	0.93215482	1.350180933	0.677577434	1.051589818
CAMSAP3	0	0	0	0	0.040152548	0	0.026362634	0.012542384	2.65032607	2.501852938	2.51287043	2.667884652
CAMTA1	0.607035457	0.587947684	0.720921282	0.714343616	0.613022643	0.608769418	0.729752492	0.764096306	1.404951562	1.518782158	1.491943646	1.334648826
CAMTA2	11.84042694	11.67271204	11.87626368	10.90051778	9.553171052	9.326072353	10.45374708	11.67117807	9.050295577	10.14900717	8.843383358	10.57913706
CAND1	42.190704	41.67017471	43.7866222	47.35674337	52.80422527	49.25446556	45.87426664	40.87808017	28.70097268	30.08474762	28.25039851	28.42647586
CAND2	0.031351463	0.021667944	0	0	0.141858299	0.311235832	0.214935591	0.132936117	2.513376347	2.452308431	2.870505813	2.816802253
CANT1	10.96599395	10.50800775	9.489730184	13.98475764	10.484414	9.766639833	9.947738145	14.13840958	21.4096687	18.73817805	20.41483014	24.91986396
CANX	118.418807	122.1768383	124.3547806	127.6073189	123.5517941	123.2339965	124.1421715	115.6381958	128.4862331	158.9013783	145.4405991	118.4839496
CAP1	174.0863087	175.3866151	179.27799	151.7395066	159.259668	149.4274093	173.4930012	145.945947	2446.644651	2450.793998	2649.305913	2247.822937
CAP2	1.249498247	1.362639885	1.648813934	0.672434232	0.711654265	0.983222876	0.717554951	0.508110285	5.597999846	6.975185387	6.135270803	3.597034214
CAPG	135.199771	136.2264421	124.8559041	109.0593405	77.00577184	88.81717181	126.3887313	107.162699	121.161843	79.49913046	108.4751884	131.4215703
CAPN1	43.29028312	44.65595358	41.51181605	37.56053311	38.05116243	36.57220417	41.65929591	41.75870086	55.48328009	45.88667811	51.92402619	53.87336986
CAPN10	7.855707448	7.975188352	7.302190946	8.433403603	6.651462924	7.398714952	7.680789187	9.518948464	10.16574886	10.35477674	9.864889049	13.38551591
CAPN11	0.33795258	0.25303335	0.317949931	0.018975983	0.548928947	0.269939435	0.30891929	0.073486292	0.510801265	0.164701673	0.444925848	0.097695218
CAPN12	0.442746901	0.639312591	0.384929171	0.063926146	0.280686329	0.243581216	0.357735437	0.139252529	0.739936836	0.93630215	0.800528966	0.362026381
CAPN13	0	0	0	0	0	0	0	0	0	0	0	0
CAPN14	0	0	0	0	0	0	0	0	0	0	0	0
CAPN15	9.571584762	8.285129547	10.13069731	8.937525811	12.69087587	9.87756003	9.399365533	11.01427847	11.11407934	8.71866175	11.59736395	11.93260037
CAPN2	37.26387888	45.17211679	36.30797112	38.26077598	22.13099554	30.15824329	34.57891226	34.54156419	69.98080949	74.75681665	74.99096728	84.63691901
CAPN3	6.377211746	6.291327761	4.556844599	5.09396855	3.590308815	4.466711015	3.853213492	5.204904438	5.88460912	4.41485488	4.463462707	8.379955369
CAPN5	0.163483909	0.121059292	0.086516812	0.106221032	0.024386631	0.023984574	0.048034	0.022852833	1.131008003	1.792667623	1.220113988	2.163151908
CAPN6	0	0	0	0	0	0	0	0	0.765573875	1.263793275	1.305952155	0.389422106
CAPN7	9.559291614	9.22153213	10.16789792	13.28163253	10.16737088	8.793927945	10.08644122	10.20099713	10.59650188	11.00235854	10.29692157	13.63870029
CAPN8	0	0	0	0	0	0	0	0	0.015389818	0.015507063	0	0.014717188
CAPN9	0	0.012360763	0	0	0	0	0	0	0	0.013074313	0	0.024816707
CAPNS1	82.68735852	90.61200834	87.28962948	90.9320019	70.9264688	81.47233608	84.84725741	100.7426029	57.95315507	63.78372867	63.00437188	55.92423364
CAPNS2	0.052962166	0	0.112111774	0.321172256	0	0.054390215	0	0.103647497	0.11527216	0.174225515	0.228195684	0.165351079
CAPRIN1	77.48702249	68.4109605	73.5878744	77.83238165	82.70375738	77.53861458	79.36939034	71.1324869	90.57283544	84.89887532	84.62358046	90.04224632
CAPRIN2	2.999501409	2.622661544	2.293476414	2.546101922	2.708468968	2.543230785	2.382013773	2.162101996	10.71031799	11.06112552	8.38208331	16.68525016
CAPS	6.917297996	6.079871671	5.570602736	4.686192916	3.689957432	3.4489527	4.587638511	5.689557229	4.854853171	2.226061631	3.050615982	7.876882836
CAPS2	0.027085349	0.056158512	0.028667507	0.05475017	0.028281946	0.027815667	0.009284424	0.008834384	0.451959702	0.38610245	0.233402834	0.357039959
CAPSL	0	0	0	0	0	0	0	0	0	0	0	0
CAPZA1	130.8936699	121.2816076	131.1909194	130.5206318	134.2987268	127.3693192	135.8907349	125.1945278	189.1007191	185.7727041	187.9784701	164.6752218
CAPZA2	29.46279012	30.5439376	31.37721143	30.47046798	28.85654768	28.89681025	31.51943773	30.28213832	51.02720787	47.48399566	47.01392856	53.4318865
CAPZA3	0	0	0	0	0	0	0	0	0	0	0	0
CAPZB	47.67294963	48.52629769	49.15085142	46.29852582	41.29562093	43.21423653	49.78641424	47.16774488	55.49463095	54.18437498	56.19279439	51.69425655
CARD10	0.262216378	0.457833263	0.467424725	0.18133039	0.576422658	0.63778421	0.823144329	0.513160112	0.901127321	1.225789772	0.698691991	0.689393984
CARD11	0.011965657	0	0	0	0	0.024576588	0.024609814	0	0	0.065604152	0.051555885	0.037357504
CARD14	0	0	0	0	0	0	0	0.010131408	0.011267704	0.02270709	0.022305832	0.010775235
CARD16	5.020133958	4.553805853	4.815249731	3.937507348	3.358103125	3.571254125	5.646445702	3.325986726	8.280103404	7.91312309	6.618561885	7.020270117
CARD17	0	0	0	0	0	0	0.005597893	0	0.005923951	0.005969082	0	0.005665038
CARD18	0	0	0	0	0	0	0	0	0	0	0	0
CARD19	17.40247605	18.38470541	16.13857047	15.80185084	13.03718326	11.68753025	14.48713327	14.43360561	15.93217466	17.5074581	19.87598462	20.87022616
CARD6	6.359085476	5.212022471	6.230033662	5.546683476	4.495983424	4.255719866	6.142664334	5.175183751	9.005837407	5.130815161	6.836371153	8.275507946
CARD8	17.96078694	16.1993898	15.22336617	13.112775	15.86565767	14.95257866	14.47548313	11.36708947	14.7414244	15.91430313	13.4990052	14.58417398
CARD9	0.684342036	0.499245555	0.804796721	0.179319915	0.370520575	0.338382459	0.62555065	0.471222627	26.86563466	24.48556467	30.96023172	37.4293158
CARF	2.487153264	2.2303335	2.099368758	1.633943129	2.291881201	2.228553275	1.720032373	1.399372951	2.375080683	2.488629147	1.868652105	2.161270189
CARHSP1	11.55394971	13.09247975	11.42224471	11.96638763	15.4606062	13.94788509	12.51128985	12.75708279	27.22055551	18.35492677	24.24599339	20.06839647
CARM1	19.4044708	20.35430868	21.16914974	21.85297766	25.80217863	27.7480052	22.0617336	25.2716101	17.85535943	22.92099453	23.27362525	20.44858909
CARMIL1	0.056866115	0.117905673	0.052664395	0.086211628	0.037111492	0.058399428	0.095027369	0.041732836	0.696201207	0.475464575	0.574257302	0.954274498
CARMIL2	0.229025584	0.213686782	0.157562493	0.0462951	0.155443367	0.223440884	0.188415128	0.089641078	0.162004116	0.138124732	0.148018822	0.166840729
CARMIL3	0	0.012126983	0	0.011822863	0.012214521	0	0	0.022892578	0.585582571	0.230886682	0.453613333	1.22954104
CARNMT1	3.363560599	2.803415175	3.160122658	2.81547892	3.759711433	3.385777786	3.481780493	2.711303207	2.604201712	3.387694355	2.729299553	2.891310349
CARNS1	0	0.00799887	0.008166444	0.01559655	0.00805661	0.03169513	0.007934495	0	0.016793306	0.008460622	0.008311113	0
CARS	17.21557572	15.55996273	18.1904756	17.95722859	20.24967979	18.0375384	19.03219289	18.23748583	17.66067747	16.68103165	25.70516342	17.93112838
CARS2	15.9665216	16.57712385	17.12619066	16.96826895	16.34841749	15.56494759	14.3361706	18.9578524	15.39164108	14.39832164	16.27445532	17.08735221
CARTPT	0	0	0	0	0	0	0	0	0	0	0	0
CASC1	0.015076929	0.015630183	0.01595763	0	0	0.046450371	0.04651317	0.014752852	0.082037345	0.049597401	0.032480641	0.062761444
CASC10	14.04080547	8.469987306	11.9403267	7.546659896	15.70631475	10.08524206	13.13132189	8.285754935	26.4969489	15.51497626	17.7760881	19.90508873
CASC3	26.3898564	24.80925412	26.93675805	33.72400885	31.52106287	28.899369	27.64110778	33.56939091	34.28039594	33.94868907	31.1856152	35.38112245
CASC4	16.39134339	16.61890418	14.67651117	19.33454852	16.12510904	16.86075064	16.10050068	16.06515613	37.17315375	39.28742105	35.55694383	44.39038828
CASD1	2.714503932	2.604366014	2.382326901	2.862825732	2.808019546	2.796354084	3.112223918	2.235460375	3.944861435	3.069003961	2.942126271	4.29006134
CASK	0.02947823	0.030559944	0.024960132	0.029793562	0.030780541	0.018163841	0.030313996	0.028844598	0.025663748	0.032324079	0.031752878	0
CASKIN1	0.01875878	0.087512131	0.069490931	0.03791889	0.039175038	0.028896876	0.096453143	0.073422246	0.142899703	0.123418597	0.181856487	0.234264175
CASKIN2	0.093491515	0.032307406	0.054973727	0.062994404	0.043387489	0.032004127	0.085459719	0.081317264	4.11490455	3.360287728	3.938710364	4.756663222
CASP1	14.0545945	14.64898508	14.25325158	13.47647992	11.22943772	11.20012819	13.63386759	11.80375834	22.12705378	21.94205673	22.18766661	19.6203403
CASP10	3.397312191	3.738971652	2.862976439	3.043095909	2.488224476	2.513342221	3.087973911	2.694090588	4.783484195	4.652200072	4.630692662	3.594184261
CASP14	0	0	0	0	0	0	0	0	0	0	0	0
CASP2	17.12515531	19.06010863	18.25627617	24.61381569	21.7006189	24.83231169	17.40738921	23.7935621	19.18774577	17.1932379	18.97896428	22.74673985
CASP3	18.51875288	16.76444286	19.08365447	23.53843248	20.37629451	18.43944097	21.74778326	22.38438628	16.35134578	14.47821123	15.29460662	19.01812515

CASP4	46.23177572	46.94628522	45.3848501	42.78643596	42.56806206	45.00902617	48.44168725	44.66765009	61.09564938	50.05584364	46.97370297	62.86132314
CASP5	0.024129848	0.050030602	0	0	0.025195874	0.024780475	0	0	0.210074465	0.291052968	0.129958977	0.125558069
CASP6	4.081881197	4.869775825	3.874572118	4.911363811	4.769619872	4.125404493	4.464125524	5.135324737	3.34920796	2.983965951	4.152584428	2.865687177
CASP7	13.77227574	12.45194023	13.10598394	14.04475078	12.01026941	11.65683414	13.75243753	12.68193773	9.357941873	8.026163936	8.521600512	9.478719261
CASP8	13.90911901	12.11175938	13.30793948	11.35804377	12.53582783	11.09939025	14.71394478	10.06489643	22.42087902	18.78712747	19.92691952	18.77281202
CASP8AP2	12.47573435	12.40563641	13.53291728	18.30262365	19.73142788	18.12327327	14.98132373	13.97486416	18.45152237	15.05863524	18.8034863	16.51069116
CASP9	2.597613365	2.679469036	2.501908327	3.045464426	1.77660406	1.640607387	2.67126082	2.821373422	4.042399594	3.005050202	2.812045	5.163306627
CASQ1	0	0	0	0	0	0	0	0	0	0	0	0
CASQ2	0	0	0	0	0	0	0	0	0	0	0.021451408	0
CASR	0	0	0	0	0	0	0	0	0	0.007526068	0	0.014285433
CASS4	0.353410251	0.472746794	0.21719281	0.092178252	0.190463724	0.222446761	0.386877233	0.122708096	1.290266681	1.075079702	1.31396235	0.78303545
CAST	21.93491787	25.48837153	19.70593559	26.52518926	20.06301077	21.00692834	21.46420426	22.94031441	36.53542483	35.24583129	32.72980498	39.22713046
CASTOR1	1.625566265	1.513256076	1.404507554	0.938831517	1.143134621	1.022079998	1.228154158	1.266007524	1.732920061	1.746122052	1.608062062	2.831017081
CASTOR2	0.556924977	0.508628019	0.589457076	0.743808984	0.401532075	0.57875049	0.559078828	0.752555407	1.789360245	1.054168837	1.635439889	1.952647655
CASTOR3	0.977171202	0.792004369	0.714573747	0.873897712	0.51944652	0.547374122	0.730818858	0.904012417	3.21599937	1.448159309	1.939286585	3.149398779
CASZ1	0.661281172	0.612745654	0.619388595	1.088294837	0.849370855	0.871426481	0.421257395	0.979189858	1.350119684	1.219231226	1.166168045	2.174182369
CAT	50.18326437	51.97624904	45.41365257	58.33400475	31.12260063	38.27387413	42.68025557	51.25648109	184.1020682	156.8746735	156.194191	243.9117255
CATIP	0.050078698	0	0	0	0	0	0	0	0.027249073	0.109826662	0.053942954	0.182406837
CATSPER1	0.246550107	0.298196909	0.413174053	0.083062495	0.429070627	0.253197977	0.485952219	0.100521054	4.80718796	3.154109358	3.895097306	3.527994772
CATSPER2	2.481508875	3.032923121	2.294699441	2.930462968	3.600319136	3.353183165	2.57872627	2.402609351	2.615223131	2.807004217	2.053982702	1.957243066
CATSPER3	0.826628221	0.938577029	0.999902525	0.51719619	1.150863469	0.848917066	0.526230565	0.654791354	1.199437599	1.165411929	0.720811238	0.94219069
CATSPER4	0	0	0	0	0	0	0	0	0	0	0	0
CATSPERB	0	0	0	0	0	0	0	0	0.129303355	0.048858163	0.047994786	0.046369499
CATSPERD	0.015929414	0.01651395	0	0	0	0.016358928	0.016381044	0.015587013	0.017335186	0	0.017158586	0.033155061
CATSPERE	0.193816428	0.285530102	0.237528187	0.391779414	0.213030516	0.178090577	0.188821426	0.299447934	0.543953478	0.402683882	0.428532027	0.48833163
CATSPERG	0.205486953	0.232393494	0.177946548	0.122722998	0.117035512	0.172658958	0.211312913	0.146232747	0.264279584	0.256050921	0.171037834	0.379093396
CATSPERZ	0.072324679	0.074978658	0.153098874	0	0	0.07427481	0.148750451	0.14154013	0.078707335	0.15861391	0	0.075267337
CAV1	0.029420208	0.04574969	0.031138754	0.11893968	0.122879822	0.120853928	0.015127165	0.071969558	2.417248993	3.306696758	1.948958324	2.051353867
CAV2	0	0	0	0	0.016995402	0	0	0	1.753559422	2.15956724	1.49024344	1.304269574
CAV3	0	0	0	0	0	0	0	0	0	0	0	0
CAVIN1	0.02382008	0	0.012605751	0	0.049744842	0	0.012247713	0.011654035	0.012961102	0.039179531	0	0
CAVIN2	0.016551695	0	0	0.033457501	0	0.067991951	0.034041937	0.016195918	0.198136213	0.254094507	0.267433268	0.155026173
CAVIN3	0.103779288	0	0	0	0.054182062	0	0	0	0.338813445	0.22759643	0.279468195	0.324005201
CAVIN4	0	0	0	0	0	0	0	0	0.113411731	0.076183827	0.093546972	0.090379114
CBARP	0.795030884	0.659363858	0.722675654	0.444307826	0.615290859	0.51869716	0.923374955	0.49422182	0.162859745	0.25640698	0.191425755	0.379620555
CBFA2T2	3.402998711	3.640150314	3.052765608	4.234438445	2.958139716	3.038154449	2.907441024	3.112323517	3.963848121	4.794105416	3.622606479	5.303285547
CBFA2T3	6.566830575	6.115483819	4.851374564	5.22580315	6.149062957	6.546462749	5.858160872	5.732614799	5.736903334	4.593420643	6.125324832	4.527929015
CBFB	33.81972081	27.87427254	37.22997502	42.47107076	40.74259569	34.8021064	37.4606382	39.20426902	56.63494696	50.46228699	57.33329233	52.14183183
CBL	12.50014641	11.13596303	11.49096273	10.14970291	12.61213777	11.0855501	10.87389507	7.801128371	27.3369194	30.88128505	27.30098944	29.06896805
CBLB	1.882165769	2.050658367	1.516287705	1.441870349	1.721217514	1.871357848	1.53486207	1.319695764	1.898234689	2.385940502	1.556062224	1.796555961
CBLC	0	0	0	0	0	0	0	0	0	0	0	0
CBLL1	4.027241737	3.829629037	4.311980075	4.123489863	4.900934097	4.586029801	4.417917488	3.946395888	6.316353385	5.505622156	5.376851502	6.216692876
CBLL2	0	0	0	0	0	0	0	0	0	0	0	0
CBLN1	0.066295147	0.091637161	0.09355693	0	0	0	0	0	0	0.024231776	0	0
CBLN2	0	0	0	0	0	0	0	0	0	0	0	0
CBLN3	0.802785435	0.59445995	0.509807505	0.046364168	0.143700252	0.235551827	0.283044337	0.089774814	0.624022347	0.628776368	0.667078431	0.262569432
CBLN4	0	0	0	0	0	0	0	0	0	0	0	0
CBR1	17.31168249	17.54684976	17.24338819	14.04207222	16.49335841	15.57031363	18.11433006	16.93956915	12.2396357	15.44632956	15.64847225	11.30305643
CBR3	1.807459007	2.537416312	2.231879463	2.397666635	3.224154995	3.673718379	3.330178053	3.647753607	1.106419133	1.156138896	1.500757897	0.66618704
CBR4	7.38340896	7.73639847	7.575354185	6.193898168	9.03191867	9.777118326	7.953194963	6.97023426	5.094162308	5.976068173	5.736491369	4.671477798
CBS	1.531845677	1.992290061	3.611136496	1.463783006	4.318705795	4.462024605	4.238925904	1.498918767	3.212823628	3.725949105	5.010147262	2.637629293
CBSL	0.120140424	0.179904138	0.197801771	0.067458656	0.404221573	0.891076608	1.180556794	0.8359681	0.072634901	0.146376518	1.207835055	0.79184749
CBWD1	14.14380317	12.31522521	13.47692583	11.5749048	16.31917366	14.239242	14.56389551	11.55131508	30.94558462	29.85837088	32.54972527	27.06259306
CBWD2	8.709824689	8.584803523	8.031354813	11.3538626	9.421881929	10.9097901	8.49874896	9.232824954	7.593544677	9.568765797	9.239756561	8.429018801
CBWD3	1.906964406	1.890987176	2.398627667	2.87707877	3.405260523	3.973530662	4.007323406	2.704310648	1.563959613	1.303126912	1.577796761	1.351796736
CBWD5	4.073293574	4.67464707	4.883939626	4.861244121	5.069367586	4.754251743	4.142409215	4.426964085	2.289987706	2.686512016	2.849513923	3.378704511
CBWD6	1.625408039	1.72052776	2.390387005	2.248035586	2.161717849	2.442354131	2.005789869	2.009014753	1.247504999	1.500906184	1.769260271	1.709346356
CBX1	31.26826126	35.85526507	35.4784329	47.12974812	38.9066285	42.0442054	40.32271202	46.35661403	35.29688664	40.90176174	35.78203828	42.33399565
CBX2	3.881513377	4.203231643	3.979440855	4.4344531	4.226884506	4.683424176	3.668811869	4.901153428	4.426198457	3.30104225	4.71917692	4.746008329
CBX3	64.71780311	67.05816854	71.71896904	87.59440628	86.83256488	84.90574783	79.90767599	83.11222431	62.06885715	59.42375031	63.20978128	60.98273161
CBX4	13.48826326	15.28484934	14.52928732	15.4091311	13.16019683	12.83270649	12.77627543	16.21710192	17.94485255	10.93547738	14.59346894	21.05558596
CBX5	17.85037421	18.46779695	16.65201813	24.94715688	30.72094172	27.86304214	18.48701893	20.90056836	64.27691395	72.43299038	64.21793406	63.40219108
CBX6	15.68463599	17.21028377	17.1089296	17.46676948	13.80746146	17.25488771	12.4851936	17.48265114	20.62124511	21.31431333	21.78382277	31.36483336
CBX7	1.153530172	1.467645589	0.721448143	1.483834076	0.875993937	0.996169046	0.768356777	1.475051585	6.904310942	7.560609234	5.91618851	9.767408421
CBX8	25.51255376	22.58787851	23.17160378	18.99611322	18.09898171	16.76400716	19.00580475	19.20844216	13.78732572	10.99175926	11.28491126	13.83672662
CBY1	3.429248174	3.598975593	2.82299388	2.567356503	3.448127689	4.086926125	3.265253809	3.521242256	9.721315689	11.88443732	9.759090939	14.18697516
CBY3	0	0	0.070312076	0	0.069366418	0	0	0.13000723	0.072294145	0	0	0.207403329
CC2D1A	15.76883656	15.96559776	14.74054345	16.12367031	15.07486777	16.5868675	15.60399284	19.3809357	8.526254912	8.91745942	7.87473383	10.92553556
CC2D1B	4.587398649	4.178210679	4.368024427	4.647961272	3.579192856	3.747857004	4.08612744	4.327485552	10.45462227	10.12910504	9.956236018	13.68322977
CC2D2A	0.013325795	0.05525916	0.028208411	0.006734171	0	0	0	0	0.072508986	0.109592077	0.086124374	0.069339895
CC2D2B	0.075624793	0.036586607	0.074706169	0.035669088	0.005264387	0.031065563	0.031107562	0.024666414	0.04937921	0.044227021	0.01086137	0.057714596
CCAR1	50.01044078	42.30403345	49.52103573	48.68266826	53.28241694	45.85491718	52.78875019	42.91672809	44.07557823	39.27838998	44.12591981	34.74779329
CCAR2	51.63992591	50.01194879	53.30072052	60.2239455	60.40387922	57.19329948	56.42386076	69.35555752	59.12719811	52.7413402	60.60848537	70.60730723
CCBE1	0.006890711	0.042861407	0.029172893	0.006964422	0.007195134	0.007076509	0	0	0	0.007555945	0.007422423	0.007171072
CCDC102A	1.525198149	1.551611332	1.76515905	1.469480052	2.574918189	1.69806972	1.934898585	1.855056812	2.249257556	1.266054125	2.272405865	1.097726759
CCDC102B	0.06808464	0.053777547	0.099513807	0.108134637	0.060936457	0.103215895	0.080017113	0.06344873	0.059980139	0.092433195	0.066353701	0.148457646
CCDC103	0.269197358	0.49613449	0.411554284	0.272076997	0.218625676	0.12286928	0.430623875	0.409750415	0.976513459	0.45917801	0.934346529	0.809322607
CCDC105	0	0	0	0	0	0	0	0	0	0	0	0
CCDC106	2.178164793	2.972388145	2.234826197	1.931897769	2.367225717	2.807532591	2.217063727	2.196590566	4.571462447	5.776140735	6.631719089	4.9730538
CCDC107	5.10925029	4.531311751	4.219882259	4.865412998	4.348155108	4.42811578	4.616325751	4.826036789	3.921024897	3.659437048	3.72201939	4.395083718
CCDC110	0	0	0	0	0	0	0	0	0.032272393	0.032518255	0.047915434	0.046292835
CCDC112	4.993796576	5.390332232	4.751014082	5.897870213	5.487831218	7.452574878	5.693155958	5.76491916	5.780516165	6.214225036	5.620895141	5.041263399
CCDC113	0.774542226	0.88326076	0.909217393	0.619145468	0.507313413	0.715883983	0.629960692	0.489185804	1.747092726	1.976592493	1.888571606	2.564723187
CCDC114	0.021285428	0.033099756	0.04505758	0.02151312	0.033338686	0.087437434	0.010944456	0.031241848	0.243220609	0.350105069	0.217814947	0.14398451
CCDC115	12.85215233	12.76250903	13.95168746	12.79930915	12.16643277	12.61852991	13.16812394	13.31294513	11.14297543	11.84733501	9.812414399	13.00761823
CCDC116	0.045549854	0.078702203	0.096421187	0.030691404	0.11097844	0.077963401	0.046841282	0.089141527	0.066092841	0.066596359	0.081774413	0.063204175
CCDC117	11.83599218	11.17597534	13.04411421	16.40356973	17.00208669	14.54008633	15.55029485	15.15805244	13.92876639	9.925699069	11.37061376	11.68589086
CCDC12	3.734417473	3.790797965	3.922615326	4.553542639	4.416365087	4.728517157	4.007581165	5.079817217	4.664340665	3.955340552	4.738719726	4.357432717
CCDC120	0	0	0.025793832	0.012315482	0	0	0	0	0	0	0.013125386	0
CCDC121	0.497213601	0.706369782	0.68218595	0.949227789	1.057588612	0.794298172	0.587060303	0.864935168	2.745544565	2.503950178	2.142321696	3.487953316
CCDC122	0.402274899	0.428011164	0.235295772	0.331682122	0.309508236	0.369635174	0.315703299	0.227889912	0.875551355	0.487543522	0.513137255	0.738132249
CCDC124	58.03284126	55.32054174	66.68155743	66.1861978	58.67707225	57.91377698	73.93350685	82.21246703	23.84505204	23.86326497	31.52303181	24.66433495
CCDC125	6.269171511	6.128425482	5.646906123	6.316150489	7.562809491	8.305393884	7.632084639	6.241355241	2.627336866	3.606064976	2.889523631	3.112198772
CCDC126	1.671828842	1.652564299	2.016391824	2.023725566	1.603597215	1.637051157	1.999102886	1.711981051	1.586657977	1.811911785	1.36109492	2.144466502
CCDC127	8.109328186	9.389017666	8.583024788	8.808865336	9.179814988	8.600395312	9.235517456	9.23280222	14.84575531	12.71502715	15.87824135	16.79966965
CCDC129	0	0	0	0	0	0	0	0	0.006706168	0.013514515	0	0
CCDC13	0.094666515	0.069275531	0.064832929	0.067538241	0.110478027	0.131531672	0.080170998	0.098080587	0.121200987	0.146549207	0.161954465	0.237602696
CCDC130	18.95342795	17.80964722	18.7552585	17.01313276	15.65639383	15.10941368	16.70957422	19.20233454	12.19669367	9.893375192	11.11690094	13.8252207
CCDC134	3.576466281	4.136696748	3.378687383	4.354099601	4.151125743	4.082686997	3.373910263	3.738198839	10.11621634	9.139925057	9.52762418	7.259401332
CCDC136	2.904031505	3.945183009	2.799930748	3.413076359	3.117202231	3.468007356	2.614020564	2.740381741	3.192478447	2.94130567	2.324199048	3.691221016
CCDC137	13.29531899	12.20737277	15.01579921	14.91239275	16.10476601	14.4863575	15.21456593	18.2252509	12.94677849	10.55633816	12.81488514	14.55338614
CCDC138	2.977370352	2.861772458	3.380854002	4.16507887	4.395705939	4.221988132	3.84243837	3.328189863	1.748808337	1.902669455	2.283211119	1.877573926
CCDC14	18.0904488	19.95111154	18.49346374	20.29671761	23.30166955	23.92525325	16.63260463	18.63355616	25.61688182	27.38810639	22.67607093	28.05351401
CCDC140	0	0	0	0	0	0	0	0	0	0	0	0
CCDC141	0.021786717	0.015057459	0.026902589	0.018349811	0.026540765	0.011187083	0.011202207	0.003553069	0.106692283	0.055743386	0.046935721	0.052904014
CCDC142	3.521990155	2.944126266	3.373326756	3.522063235	2.797038143	2.789131234	2.9586907	3.397746008	5.762704717	3.889202826	4.929216098	5.665710207
CCDC144A	0.059805013	0.042624711	0.035605379	0.02644458	0.035126507	0.049901776	0.030750302	0.014629877	0.056947458	0.049183974	0.020131184	0.027229251
CCDC144NL	3.839565356	3.643947281	3.631942275	1.565378	5.558638817	4.219300783	4.072408942	1.551818467	10.355183	7.189950476	8.111841326	2.866541357
CCDC146	1.097824824	2.108850685	0.956902416	1.370643313	1.550910733	1.459022008	1.394585689	1.390176325	1.704215702	1.593277435	0.939073507	1.864949826
CCDC148	0.077113157	0.079942851	0.016323526	0.015587609	0.032207967	0.079192403	0.015859893	0.030182246	0.050351038	0.067646173	0.099676189	0.144451158
CCDC149	0.210102407	0.217812189	0.214139163	0.14943141	0.17063236	0.135853622	0.136037289	0.121828901	2.6590445	2.380653716	2.103888303	3.830438055
CCDC15	1.01890201	1.205766117	1.14963635	1.777864568	2.338607374	2.42838028	1.156522801	1.787076881	2.88711664	3.541527292	2.868058577	3.421154591
CCDC150	1.745034162	2.115080445	2.572891056	3.606373716	3.635189779	3.138380351	2.830146534	3.236651451	1.823450083	1.732622803	1.814225639	1.59919411
CCDC151	0.047745839	0.173242614	0.10106971	0.19302633	0.024927595	0.147099712	0.073649292	0	0.675472353	0.890039365	0.540015873	1.416121222
CCDC152	0	0	0.016314174	0	0	0	0	0.015082477	0	0.033803709	0.016603181	0
CCDC153	0.350779406	0.436381656	0.445523713	0.850876171	0.293021114	0.288190136	0.432869631	0.617830971	1.297901395	0.538501461	1.284679221	1.387195669
CCDC154	0.761340204	0.636514396	0.623855204	0.992883042	0.692397792	0.554874527	0.883948369	1.057384229	1.523423392	0.807910192	0.714270196	2.044688228
CCDC155	0.035254135	0.024365198	0.024875642	0.011877084	0	0.012068237	0.012084553	0.022997566	0.42201845	0.966439851	0.544300777	0.807147264
CCDC157	0.245664211	0.179218517	0.202233413	0.404624183	0.304020554	0.345728266	0.280699195	0.462961155	0.435671848	0.189564273	0.333225892	0.34087933
CCDC158	0	0	0	0	0	0	0	0	0	0	0	0
CCDC159	11.19989697	10.02168233	9.138934674	7.999678869	8.656686998	7.967787049	10.19840013	8.26528526	4.664233266	3.293267405	4.24603178	7.976587334
CCDC160	0	0	0	0	0	0	0	0	0.035425443	0	0	0.033877132
CCDC163	0.73679169	0.689909632	0.905609597	1.201085879	0.794159698	0.97633319	0.586591886	0.953520345	1.732951954	2.501952269	1.920109441	1.657211229
CCDC166	0	0	0	0	0	0	0	0	0	0	0	0.042423408
CCDC167	27.12140507	26.58953305	31.18187704	42.29957558	30.85297638	36.12841575	32.07835819	45.70036774	35.73845318	43.23186848	46.29469407	34.08628625
CCDC168	0.079489481	0.097389353	0.114726494	0.216673978	0.16348726	0.20037142	0.086697295	0.084851847	0.110096548	0.081880819	0.083028538	0.090243984
CCDC169	2.657262265	2.428548508	2.183374982	1.872913538	3.066725851	2.800724996	2.552824509	1.265859875	0.608792796	0.958485613	1.05453392	0.545798234
CCDC169-SOl	0	0	0	0	0	0	0	0	0	0	0	0
CCDC17	0.193238754	0.231149676	0.173060938	0.225352909	0.139690936	0.03053064	0.122287664	0.116360063	0.954402532	1.744051949	0.768552977	0.525956508
CCDC170	0.039348856	0.030594582	0	0.009942444	0	0.040409842	0.091045066	0.028877292	1.113356259	1.208133442	0.974858636	1.668705761
CCDC171	0.141092322	0.173942411	0.165478282	0.184996676	0.135380282	0.215386948	0.129406884	0.190298302	0.406683381	0.351241404	0.328604372	0.341287315
CCDC172	0	0	0	0	0	0	0	0	0	0	0	0
CCDC173	0.023704652	0.073723509	0.050178662	0.071874672	0	0.024343814	0.024376726	0.023195123	0.051593178	0.051986233	0.05106758	0.098676474
CCDC174	6.181125545	5.692567944	6.013840754	5.87626924	6.576844915	6.634270606	6.054407181	6.321224683	8.643933716	7.244739006	7.7967585	8.357816552
CCDC175	0	0	0	0	0	0	0	0	0.015345455	0.046387087	0.030378251	0.029349528
CCDC177	0	0	0	0	0	0	0	0	0.084075028	0.042357771	0.152567298	0
CCDC178	0	0	0	0	0	0	0	0	0	0	0	0
CCDC179	0	0	0	0	0	0	0	0	0	0	0	0

CCDC18	3.137744214	3.395555234	3.214214938	4.942420267	4.909758619	4.682770317	3.689011192	3.909693143	6.464871517	6.644908654	6.058060417	5.93884315
CCDC180	0	0	0	0	0	0	0	0	3.158549513	2.843869044	2.367994836	3.28217845
CCDC181	0	0	0	0	0	0	0	0	0	0.028740562	0	0
CCDC182	0	0	0	0	0	0	0	0	0	0	0	0
CCDC183	0.407648188	0.422606983	0.265514132	0.380315864	0.458400455	0.450842902	0.225726209	0.521619919	3.651359852	3.232174352	2.499514027	6.983545659
CCDC184	0	0.023798687	0	0.023201864	0	0	0	0	0.049964383	0	0	0
CCDC185	0	0	0	0	0	0	0	0	0	0	0	0
CCDC186	3.223016726	2.585976496	2.888482999	3.444706032	2.894764496	2.656813785	3.04772009	2.471032437	2.983346659	1.976968137	2.134906124	2.416016748
CCDC187	0	0.01137291	0	0	0	0	0	0	0	0	0	0
CCDC188	0.020078194	0	0.021251038	0	0	0	0	0	2.075759112	1.10082788	1.319277583	1.901455147
CCDC189	2.759927191	2.89860505	2.176539413	4.010974553	3.691790993	4.390455474	2.318771701	4.342145614	7.224082668	6.567030601	7.811131368	9.142294245
CCDC190	0	0	0	0	0	0	0	0	0	0	0	0
CCDC191	0.707174635	0.970094244	0.665318566	0.512590863	0.686205426	0.608870047	0.558273299	0.26560617	1.134940333	1.879868557	0.931019002	1.122505473
CCDC192	0	0	0	0	0	0	0	0	0	0	0	0
CCDC194	1.136250154	0.715181047	0.47245897	0.328115648	0.381358362	0.41674554	0.375578063	0.039708091	2.119755912	1.735422775	0.743098765	1.140249071
CCDC196	0	0	0	0	0	0	0	0	0	0	0	0
CCDC197	0	0	0	0	0	0	0	0	0	0	0	0
CCDC198	0	0	0	0	0	0	0	0	0	0	0	0
CCDC22	14.10789694	14.21665197	12.59886883	13.64905913	11.70255444	13.70192483	14.3169904	17.18769588	9.949095853	10.10122321	8.423067759	10.50432126
CCDC24	0.602834827	0.516268053	0.416118714	0.34437759	0.082104431	0.161501584	0.404299814	0.435995997	2.310384229	2.557910042	2.738570509	3.900556519
CCDC25	21.97413741	21.15541903	23.42364125	22.943743	26.44172171	26.97166979	24.78065913	22.28267465	16.4695098	18.4701193	17.14598066	15.58655791
CCDC27	0	0	0	0	0	0	0	0	4.715245944	3.338658831	3.200823135	4.66149702
CCDC28A	11.63209979	11.25348	10.38495435	13.93283403	10.43071757	10.46392347	11.23139546	11.9685493	16.95870316	18.69445524	14.9447462	17.14157712
CCDC28B	2.420499277	3.385355269	3.304686272	2.055548028	2.826538446	3.397701871	3.10772436	2.256353862	0.826081348	1.413466514	0.971942329	0.357725199
CCDC3	0.042358615	0	0	0.028541154	0	0	0	0	0.09219353	0.046447946	0.182508644	0.14694017
CCDC30	0.192069821	0.192895457	0.158819803	0.133460536	0.19428787	0.209576753	0.129619467	0.217307122	1.783201805	1.961327781	1.383581102	2.179990596
CCDC32	8.700643776	7.548857551	8.853173499	5.661190347	5.790242607	6.135789987	8.256114569	6.467431168	9.9358042	8.598833181	10.73960783	10.31763195
CCDC33	0	0	0	0	0	0	0	0	0	0	0	0
CCDC34	8.867698613	11.31604507	11.53376067	17.05657853	13.45963066	13.83858578	11.71383268	16.87112178	4.914675329	6.656286879	5.553924714	5.841543304
CCDC36	0	0	0	0	0	0	0	0	0.029876662	0	0	0
CCDC38	0	0	0.016300166	0	0	0.015815815	0.031674395	0	0.016759661	0.016887342	0.016588925	0
CCDC39	0	0.014474344	0	0	0	0	0.014357854	0	0.030388302	0	0	0
CCDC40	0.091802809	0.061181708	0.076344211	0.066274883	0.054776309	0.020202459	0.060689314	0.038498363	0.235488969	0.273234977	0.233089961	0.307086333
CCDC42	0	0	0	0	0	0	0	0	0	0	0.042001235	0.040578912
CCDC43	18.09591874	17.48146003	18.99313988	18.06055646	19.47353498	17.31735605	19.87717911	18.37188238	17.36479653	19.34614893	18.02831288	17.88926469
CCDC47	33.93705101	32.55105658	35.36634977	34.45098222	35.17131457	31.34083721	35.08347908	30.56324252	24.95712621	21.56180938	26.34330483	29.25802622
CCDC50	5.805752642	5.652327218	5.624887765	5.552967332	6.913084566	5.913072117	6.25377969	4.731200663	7.706921261	6.688169813	6.176300011	5.848676886
CCDC51	7.677049135	7.740380523	7.679583386	5.653786557	8.016211375	7.403315023	7.84657013	6.435612379	4.68669826	4.51708129	5.168398978	4.165207352
CCDC54	0	0	0	0	0	0	0	0	0	0	0	0
CCDC57	3.572701051	3.022553478	3.237770414	2.610854528	2.843253917	2.76147154	2.609856318	2.742032062	2.539935631	2.422624859	2.575081304	3.218914805
CCDC58	15.70873592	15.6929854	20.91896213	20.38000479	23.26205039	22.29190473	21.7933623	21.63129858	8.951580697	14.90768694	12.55221608	8.738681666
CCDC59	26.98797394	27.87481158	33.17842918	32.72527461	33.80937234	33.79875914	39.21713197	31.52012036	17.92599706	21.96699651	20.75437626	18.87408773
CCDC6	15.07074023	15.5280325	15.90220957	17.97594555	17.96396188	16.55822218	16.20075449	15.49678601	16.06910126	13.44736728	14.43323014	16.56806274
CCDC60	0	0	0	0	0	0	0	0	0	0	0	0
CCDC61	1.808252828	2.282130631	2.104078959	2.80382731	2.603521039	3.079637328	1.882619988	3.044220652	2.554513823	2.647869022	2.092960704	3.0039067
CCDC62	1.193201993	1.024172001	1.086366835	1.024422584	1.04496192	1.067262075	1.134674401	0.941575971	0.18151105	0.154756347	0.096741041	0.146873602
CCDC63	0.036235395	0	0.038352041	0	0	0	0	0	0.03943317	0.019866793	0.03903145	0.037709696
CCDC65	0.029647141	0	0	0	0.030956914	0	0.030487696	0.029009878	0.032263503	0	0.031934823	0
CCDC66	3.933858235	3.708484491	4.312351587	4.937155294	4.491332769	4.7724437	4.512166732	4.769325301	3.728252157	3.318181325	3.504011408	4.172643396
CCDC68	0	0	0	0	0	0.00959383	0	0.009141133	0.030499092	0.030731445	0.060376775	0.038888124
CCDC69	8.675386688	10.33951064	9.014591451	10.73623069	8.992532643	9.185689641	9.08414936	9.898564636	11.49113194	7.516591378	11.22059459	11.64790276
CCDC7	0.355507141	0.302213133	0.353697241	0.294634238	0.378637253	0.27747059	0.241287069	0.229591257	0.61900906	0.483386788	0.451868461	0.591954533
CCDC70	0	0	0	0	0	0	0	0	0	0	0	0
CCDC71	12.97725061	12.52987722	13.54671564	12.03556987	11.16294013	11.58883789	13.10087584	13.04700045	11.95726	9.11767374	11.19569331	12.36178782
CCDC71L	1.458146881	1.511654096	1.326423352	1.346285849	1.366194313	1.270820565	1.791280519	1.048893841	2.07574311	1.763130565	1.833854986	1.98502029
CCDC73	0.093419376	0.096847434	0.056500775	0.107907081	0.125416961	0.123349238	0.178412	0.13058762	0.232374036	0.117072171	0.086252535	0.166663389
CCDC74A	0.018967064	0.058989202	0.040150004	0	0.03961001	0.077913935	0	0.111356211	0.350895442	0.623944741	0.510765806	0.296081594
CCDC74B	0.065541487	0.045297703	0.092493352	0	0.022812342	0	0.022466572	0.021377559	0.142651053	0.143737818	0.164730786	0.022736053
CCDC77	8.653333211	8.92634988	10.15877623	12.21821625	12.66781374	12.87793607	10.13521231	12.14421092	5.678234823	5.862765049	6.05984292	4.469185861
CCDC78	4.274215616	3.430945732	3.304282381	2.085487043	3.385758284	3.687699924	2.962415611	2.700822519	0.874874359	1.190078267	1.09688483	0.739029292
CCDC8	0	0.01673189	0.017082418	0	0	0	0	0	0	0.017697773	0.017385034	0
CCDC80	0.068518117	0	0.012086753	0.011541844	0	0.011727602	0.011743457	0.011174221	0.037282422	0.012522151	0.024601742	0.035652949
CCDC81	0.10560097	0.060820019	0.049675343	0.118589552	0.036755429	0.096398532	0.084462751	0.103331085	0.076613507	0.115795763	0.126388364	0.073265022
CCDC82	3.499742512	3.40404927	3.164523097	3.834005973	3.939720189	3.380472261	3.494102095	3.173065344	4.847302662	5.161540934	4.446425417	4.877405607
CCDC83	0	0	0	0	0	0	0	0	0.039419897	0	0	0
CCDC84	4.469466468	5.873933024	4.656048177	6.331301511	4.409689886	4.300846677	5.681897552	6.749492447	9.727794175	6.907640947	8.491446448	11.5367254
CCDC85A	0.052728624	0.02733176	0.069760879	0.026646334	0.041293581	0	0.013555896	0.03869642	0.502091866	0.448097894	0.241388771	0.480147344
CCDC85B	28.78287387	33.14446192	32.96049762	25.82412632	25.31142308	29.06556587	30.00315921	35.04496853	12.6907911	15.80474313	16.18411206	18.95417274
CCDC85C	0.194499712	0.113420783	0.167262206	0.147435225	0.019039918	0.006242004	0	0.017842402	2.824395772	3.145833581	3.050960568	2.776857177
CCDC86	21.23007488	15.4202262	21.05698366	18.86612052	23.08817333	21.63796616	23.2050704	24.43200396	9.56112485	10.30746391	12.65664993	10.7551235
CCDC87	0.166135865	0.07654768	0.097689162	0.317169079	0.038550119	0.094786378	0.189829049	0.108376525	0.421860514	0.141691465	0.338027088	0.403422599
CCDC88A	17.34114111	14.42243365	15.27520214	16.47504302	18.33811805	16.27271802	15.80431599	11.95423397	21.56068601	18.66823865	18.60453008	15.54271725
CCDC88B	6.437312942	6.930559796	5.35133094	4.716304731	3.910978414	3.87377906	5.34502938	4.756698642	19.96588919	13.49614618	15.41321591	23.14780877
CCDC88C	7.098201662	6.465916671	6.24617465	6.118171473	5.679508122	6.860412307	5.840862037	6.43136498	2.528933907	4.603424358	3.423955664	5.067445659
CCDC89	0	0	0	0	0	0	0	0	0	0	0	0
CCDC9	5.965635944	5.519277058	5.483969224	6.605972334	5.881745263	6.346165736	5.499298928	7.581649573	5.871344679	4.404478474	6.118748752	4.872695703
CCDC90B	9.148720273	7.693491801	9.206701025	6.811979065	8.73988638	6.709216878	10.24632659	6.999756241	8.791020278	7.150428363	8.190383176	8.153581472
CCDC91	7.715160781	6.826550052	8.027134777	6.721576393	8.808584869	7.855900807	8.557158381	7.084503598	11.74730332	10.9387105	11.41589719	10.84179594
CCDC92	7.911667024	7.724974572	7.413331153	7.531247813	5.498565567	5.80169185	7.071334993	8.479497721	3.505149829	3.545868577	3.510744521	3.4716657
CCDC92B	0	0	0	0	0	0	0	0	0	0	0	0
CCDC93	11.05283782	10.56815681	9.478733442	12.49955509	10.254045	10.29496764	8.987233867	10.15216597	7.298325818	7.437203194	6.512904493	8.651224953
CCDC96	0.774777699	0.777298491	1.058110196	0.858846137	0.652424508	0.693001571	0.693938473	1.17386928	1.849494417	0.849575293	1.346068362	2.028757134
CCDC97	10.91409624	9.91351424	10.52989372	11.94912632	10.18667371	9.167311193	9.646865485	13.6355304	11.97613995	9.115914315	11.41373354	12.8828197
CCDC9B	4.101122904	3.74896106	3.014958575	1.163865069	2.352103641	2.458556163	2.565756799	1.097141927	3.352781754	2.580818326	2.393762869	3.658272348
CCER1	0	0	0	0	0	0	0	0	0	0	0	0
CCER2	0.747621259	0.695562615	0.71013443	0.774993496	0.540450147	0.708719811	0.413978812	1.012917039	0.563260756	0.567551873	0.557522622	0.458843845
CCHCR1	9.011170479	12.40344952	10.7878474	15.33744411	11.27531309	14.61999185	10.96424379	15.41205895	5.751993165	6.194379857	6.704829779	6.351690468
CCIN	0	0.057420609	0	0.027990308	0	0	0	0.081296338	0.060276127	0	0.029831036	0.028820843
CCK	0	0	0	0	0	0	0	0	0	0	0	0
CCKAR	0	0	0	0	0	0	0	0	0	0	0	0
CCKBR	0	0	0	0	0	0	0	0	0	0	0	0
CCL1	0	0	0	0	0	0	0	0	0	0	0	0
CCL11	0	0	0	0	0	0	0	0	0	0	0	0
CCL13	0	0	0	0	0	0	0	0	0	0	0	0
CCL14	0	0	0	0	0	0	0	0	0	0	0	0
CCL15	0	0	0	0	0	0	0	0	0.218297324	0	0	0.052189095
CCL16	0	0	0	0	0	0	0	0	0	0	0	0
CCL17	0	0	0	0	0	0	0	0	0.059149755	0	0.058547175	0
CCL18	0	0	0.072922895	0	0	0	0	0	0.074978562	0	0	0
CCL19	0	0	0	0	0	0	0	0	0	0	0	0
CCL2	2.011572369	0.893737597	2.281152789	3.557908226	0.075015753	0.073778983	0.369393644	0.702976346	0.54727343	0.236332608	0.154770904	1.046708389
CCL20	0.447347893	0.927526964	0.067639883	0.129180923	0	0.39377999	0.131437453	0.187599507	0.625919612	0.770840995	0.344190639	0.133014011
CCL21	0	0	0	0	0	0	0	0	0	0	0	0
CCL22	0	0	0	0	0	0	0	0.018044184	2.187405764	2.102966049	1.787715329	1.266596067
CCL23	1.117450944	1.158456201	1.63761991	0.521263597	0.269265808	1.059305915	0.707158695	0.841101091	4.490089998	2.356404726	3.611032656	3.130928849
CCL24	0.230659477	0.37576567	0.348761674	0.033303839	0.378478129	0.236878879	0.101656769	0.096729201	0.896482809	0.361325011	0.851856024	0.274336308
CCL25	0	0	0	0	0	0	0	0	0.24298032	0.244831429	0.096201998	0.139416346
CCL26	0	0	0	0	0.142968953	0.070305927	0	0	0	0	0	0
CCL27	0	0	0.248160267	0	0	0	0	0	0.127577902	0	0.126278221	0
CCL28	3.706139916	4.493573197	3.461143764	3.331026771	4.780430675	4.359202046	4.088155873	3.250025843	7.1592912	6.552916694	7.058729878	5.765377573
CCL3	65.62470911	61.17355378	58.52487815	44.76370213	4.441365513	2.010731868	18.9542043	20.41374511	12.85783562	4.812150982	7.490659319	1.756552661
CCL3L3	56.9380237	63.13551758	52.83216664	66.96261715	7.04149802	5.925863128	21.0187847	24.89789634	26.47983201	7.470838096	12.80549267	2.315199952
CCL4	13.71458073	7.02528669	7.770169563	5.707588908	0	0.24854779	1.742186695	2.605017443	1.053521866	0.08846233	0.260697318	0.08395637
CCL4L2	6.390614875	4.174733619	4.200422048	4.158518857	0.21329045	0.119870843	1.230337247	1.256360546	2.09590291	0.543966575	0.345758536	0.455522496
CCL5	68.000192	63.35239431	67.37097301	23.33753805	47.15065952	43.55130652	70.51859809	26.04554153	13.25594691	21.90177615	20.10104814	8.066914543
CCL7	0	0	0	0	0	0	0	0.06460482	0	0	0	0
CCL8	0	0	0	0	0	0	0	0	0.140539817	0	0	0
CCM2	12.24060873	13.39998119	12.28527447	11.20885771	9.663643483	8.933530775	12.45473272	11.37040177	13.54590477	10.65848895	12.96236995	14.3528285
CCM2L	0.03595694	0	0.019028661	0	0	0.018463234	0.018488195	0	0.058695213	0.039428249	0.096828773	0.112259737
CCNA1	0.539444219	0.403895061	0.459936134	0.318041922	0.375517451	0.307771978	0.493100911	0.703798541	3.294003878	4.518575034	4.002924675	3.446325994
CCNA2	31.24183855	30.59757129	38.6706603	74.04428746	63.75417626	59.00104788	43.21008573	71.48379069	63.54367741	76.64804107	72.17910689	56.91723388
CCNB1	30.73768829	30.19389528	35.68410779	88.13456322	65.92687991	60.76357477	59.54462048	91.51753794	91.73856962	97.17087375	99.19494882	70.58597945
CCNB1IP1	83.6186533	89.46634939	83.19970365	63.64290489	89.27902345	93.18237468	82.31352375	69.7666915	22.99262525	33.94676342	30.18409004	23.44912371
CCNB2	32.27184121	35.43678228	37.64986963	76.43415884	67.43141295	64.67863872	53.8705185	76.92290303	69.40797932	79.49782393	80.11362656	52.38373429
CCNB3	0.021785247	0.033876997	0.046115612	0	0.022747692	0.011186328	0.011201451	0.010658488	0.663818299	0.441935597	0.527991228	0.249387809
CCNC	37.73632395	38.74008739	40.08394888	35.46178858	42.65511097	43.32245152	43.73904895	36.09242786	41.38229094	43.2034186	42.98166594	38.40614629
CCND1	1.417691858	1.495727208	1.580177425	0.076080907	2.371137923	2.035708196	3.922100964	0.896167817	2.07527514	3.934542099	2.446040653	3.877750747
CCND2	14.42182036	15.0793325	14.74030893	5.403494212	11.76804154	13.21777298	13.99075312	6.539232129	0.170592899	0.162845559	0.053322634	0.094447698
CCND3	110.9896668	79.25545414	116.9685088	80.79492304	76.61028222	56.01218262	105.1910863	84.46788976	41.68177108	38.22871201	52.66245268	60.87067903
CCNDBP1	12.02973213	12.12817599	11.87800259	11.54313834	10.17055676	9.853377388	12.20748785	11.92654869	15.68370436	13.27816098	13.59947499	15.37008635
CCNE1	8.500590136	8.784002803	10.24917128	11.26073276	8.014374651	8.221264541	7.044200819	11.46735556	14.16056012	11.76467587	16.65361833	17.26346421
CCNE2	10.33143569	11.22880385	13.88912989	16.91203307	14.39832027	11.22322088	11.80959583	14.70205637	18.34573524	14.34377307	18.1737987	22.91980737
CCNF	10.34799247	10.22058733	11.53658903	24.30218373	19.44927645	18.13676589	12.24080986	25.41823183	19.28736998	17.10989317	20.37441704	16.52554917
CCNG1	95.5403143	109.127123	87.32144427	86.27144857	92.04344687	95.85495264	108.6748617	84.39829192	113.6286439	125.850497	107.9384076	84.38407811
CCNG2	4.392802057	7.737100872	3.115578846	3.304670235	5.315095242	5.887875824	3.781531419	2.738554932	9.560934077	10.21974435	8.341568192	5.231339649
CCNH	11.53249234	10.82804311	11.56810025	10.1591389	11.41251584	10.10730745	10.88914166	7.74534065	11.42356647	11.97044419	12.74705758	11.97443575
CCNI	109.9796608	127.3357394	112.1453072	127.8024351	123.6053551	127.9034442	117.428619	122.4981139	86.72810655	85.32985543	86.16241292	99.93821428
CCNI2	0	0.04218081	0	0.0205615	0	0	0.041841337	0.059719767	0	0.044615784	0	0.021171606

CCNJ	4.228194579	3.51104128	4.297062858	3.965142277	4.700537493	4.795864661	4.596842523	3.735929043	4.658563453	4.105855604	5.007637415	4.925626364
CCNJL	0	0	0	0	0	0	0	0.009981598	0.055505457	0.078299644	0.010988001	0.053079525
CCNK	24.34899898	23.04048929	24.74777467	25.91847733	23.76578966	23.40943688	25.4300258	27.10375386	30.18118127	29.18027627	30.72937562	29.81456135
CCNL1	42.23041662	37.43117719	36.56267682	33.82804627	32.09010489	30.45729062	34.10614926	28.04782898	32.52868698	29.14084514	27.20606818	30.43166087
CCNL2	24.41350271	27.06285169	22.32385103	22.23396686	28.88603641	29.66548003	25.4514529	26.02237132	35.6827251	35.69759062	31.93875358	34.92906131
CCNO	1.464205738	1.138451463	1.046071493	1.183894827	0.535112368	0.488697933	0.639930513	1.00291292	3.226679471	3.652651745	2.562932475	3.695165434
CCNQ	7.774532684	7.19249199	7.213587014	10.02320513	6.434741443	6.265785784	7.680193949	9.783820584	4.641136036	4.631742696	4.945537855	6.073449021
CCNT1	13.06320157	11.13343477	13.18152423	11.64473491	13.67172836	12.47318527	12.79166564	9.743183382	8.716218556	8.592950115	8.279086113	8.202214673
CCNT2	6.259570344	5.705019521	5.552412107	5.76683677	7.042064994	6.093223402	6.477717897	4.373632933	10.07806813	9.010867423	8.936850017	9.31857078
CCNY	15.34417359	15.06943799	15.11853625	17.26917388	15.9343876	15.70401563	14.50564963	16.49319346	17.66912886	17.26283626	16.09858904	21.25489704
CCNYL1	12.73387512	14.35589332	13.12839477	15.76099698	9.677971448	13.20432629	13.15147103	14.35744302	11.97204336	10.13313048	11.21313817	15.09809309
CCNYL3	0	0	0	0	0	0	0	0	0	0	0	0
CCP110	7.340233906	7.994246791	7.649481644	8.829131703	10.82297051	10.70252023	7.896729099	8.200628471	18.60065158	18.8042722	17.53361085	17.15810961
CCPG1	8.984401274	7.800354827	8.610333266	11.1586349	6.005289844	5.722666899	7.768220993	7.814473095	18.32225681	16.16636534	17.18067389	28.61580169
CCR1	12.10514037	17.61494127	11.93965258	4.97921013	8.335634924	11.2131648	8.58150025	4.722235724	17.24361232	18.60975035	16.74304818	16.63644416
CCR10	0.259531644	0.58295304	0.412037807	0.437179812	0.225831184	0.488637495	0.533779754	0.16930202	0.188290216	0.284587015	0.186372037	0.090030384
CCR2	1.783002112	6.496216432	1.996238073	1.697908922	0.957790668	3.619819351	1.006864767	1.250519654	7.077238117	9.651357153	10.70198857	8.301694637
CCR3	0.263084554	0.418199087	0.167071392	0.017726587	0.146510557	0.432285204	0.27054352	0.068647886	0.095433926	0.11539317	0.151138732	0.146020597
CCR4	0	0	0.027055953	0	0	0	0	0	0	0	0	0
CCR5	0.293960999	0.380935002	0.311132375	0.089131661	0.168821302	0.196226703	0.181377223	0.071910582	0.159951535	0.064468041	0.158322053	0.107072464
CCR6	0.029188804	0.015129949	0	0.059002081	0.015239164	0.014987919	0.015008182	0.028561393	0.079411794	0	0.078602799	0.015188201
CCR7	0.079688354	0.020653137	0	0	0	0.020459259	0.040973838	0	0	0	0	0
CCR8	0.036186658	0.03751454	0.038300458	0.036573752	0.075570677	0	0	0.035408829	0.078760265	0.039680144	0	0
CCR9	0.045860421	0	0	0	0	0	0	0	0	0	0	0
CCRL2	1.003318603	0.875903775	0.698635688	0.160113352	0.165417464	0.244035391	0.543034032	0.542547348	0.172399187	0	0.085321448	0.164864275
CCS	20.43929056	24.21636291	21.19173258	15.33535277	15.24448025	17.45630765	19.94639772	16.8366922	23.03745384	18.63897872	19.76988342	29.30174939
CCSAP	7.403407545	7.480567242	7.405599196	9.615974166	9.648815339	9.842993201	8.441366595	9.309692543	12.36163701	14.84753398	11.68834007	13.79836299
CCSER1	0	0	0	0	0	0	0	0	0	0	0	0
CCSER2	3.879735848	4.060409878	3.894064015	3.585131773	3.384215652	3.480205454	3.104935847	2.907945718	2.136911088	2.402081161	2.16631441	1.686449082
CCT2	207.5747433	184.390723	234.0005598	215.6340953	234.7189971	229.4931489	256.3798116	227.2474545	97.4089149	101.0210739	111.2244962	95.06561529
CCT3	297.4210469	280.0590599	331.4451848	302.4610373	307.6807226	307.1191124	348.8190317	327.9875103	344.7349884	384.4665664	409.913311	346.5446806
CCT4	129.9080543	121.5899119	137.4237396	139.771504	145.884753	147.408273	160.7758709	140.3405637	127.6365586	156.3653599	157.8710004	119.7694626
CCT5	147.8161449	137.3158254	165.3777687	178.7076138	188.3762758	186.1477054	185.1779316	185.7870068	164.5208175	178.4326231	200.9109053	153.4553158
CCT6A	153.4703616	140.9051633	173.4665419	186.326364	175.4993812	166.1340138	182.9342431	177.4664577	108.4338897	119.1383369	126.3887149	112.3131068
CCT6B	2.663839647	2.912222375	2.434974898	2.692335079	1.087323286	1.467311906	1.643618848	2.108960675	1.765708021	1.062184957	1.512951761	2.36899032
CCT7	193.9021564	180.2660868	221.6889026	185.3352186	203.872514	207.4677188	230.1066387	206.4411843	172.72063	193.7046009	205.0658294	152.6042599
CCT8	273.9290172	277.4747997	287.9129156	230.9079145	286.9288697	284.2273348	288.0775133	241.339189	145.0423525	171.6351865	179.6186877	133.1878778
CCT8L2	0	0	0	0	0	0	0	0	0.028523262	0.028740562	0.028232686	0.027276619
CCZ1	11.46326351	11.36456368	16.40589429	14.56426476	15.23136107	12.28682704	15.63688205	14.47523002	6.221413949	5.654855154	6.221519122	5.397484225
CCZ1B	7.942478755	8.628916428	9.119889817	9.153059421	8.56879282	12.58108479	13.38170726	9.205659037	21.40119308	22.27125895	24.21384897	21.19784025
CD101	0.739519326	0.750848904	0.532570638	0.069349181	0.18309668	0.125271654	0.65856533	0.052220246	0.315275399	0.50995563	0.468095368	0.357034634
CD109	28.61260054	22.01379303	19.50071196	6.405245466	10.88991377	9.653826423	12.46598013	4.302894175	15.87272324	15.75512847	17.18650319	8.969123314
CD14	0.446923263	0.370658616	0.473029745	0.331249643	0.124444737	0.275384345	0.674071812	0.37900779	1.199698511	1.241509538	1.347946597	1.798414248
CD151	32.1068131	31.29269388	34.92916883	28.7330509	24.32224103	23.37680729	28.17868463	25.48349856	35.40899534	32.11814466	37.61800225	40.48196258
CD160	0.0256725	0	0.027172128	0	0	0	0.026400366	0	0	0.056301884	0.027653485	0.080151096
CD163	0.396946207	0.205756144	0.370705888	0.129797538	0.073144021	0.035969055	0.192094312	0.068543626	0.038115594	0.051207962	0.100606125	0.109349119
CD163L1	0.361872242	0.392600168	0.285031123	0.187124446	0.105449106	0.112353138	0.242318533	0.115286362	1.474488487	1.291931875	1.495727417	1.488866563
CD164	63.23856195	60.47798142	59.72055046	58.44782074	63.66068826	53.61845105	58.36239034	51.67327095	113.3112844	122.0395946	105.5575819	124.6096019
CD164L2	0	0	0	0	0	0	0	0	0	0	0	0
CD177	0	0	0	0	0	0	0	0	0	0	0	0.039311266
CD180	3.333832869	1.398000961	2.259873675	0.643611463	2.483718561	1.596455991	2.754239125	0.879686933	2.099373643	0.800964358	1.977113456	0.409320179
CD19	0.024728658	0.076908256	0	0.049986368	0	0.076186294	0.025429765	0.048394236	0.161465782	0.352507751	0.34627856	0.12867394
CD1A	0.909187778	0.496079339	0.759708065	0.048363867	0.424711239	0.098284497	0.541295551	0.046823413	0.729048999	0.236122441	0.566988653	0.248994659
CD1B	0	0	0	0	0	0	0	0	0	0.012680932	0.049827383	0.024070019
CD1C	0.062279584	0.064564956	0.065917571	0.020981932	0.043354011	0.042639243	0.064045333	0.02031363	0.384062643	0.250404366	0.290702989	0.345672215
CD1D	2.665794758	3.494732657	2.955871743	3.977316393	2.489008904	2.53488342	3.655167053	4.319886387	10.54509111	9.047852947	10.05783691	30.29665195
CD1E	0	0	0	0	0	0	0	0	0.043521559	0	0.021539095	0
CD2	0.032750798	0	0	0.066202275	0	0.033633876	0.033679347	0	0	0	0	0
CD200	0	0	0	0	0	0	0.00441551	0	0	0.01412489	0	0
CD200R1	0.019819359	0	0.020977083	0.060094109	0.020694953	0	0	0	0	0	0.042697388	0.020625745
CD200R1L	0	0	0	0	0	0	0	0	0.022883258	0	0	0
CD207	0	0	0	0	0	0	0	0	0	0	0	0
CD209	2.259038687	1.458910271	1.803047447	0.199624386	0.077339021	0.152127897	0.342750524	0.096633041	3.788352487	2.585417642	3.856135366	2.004089979
CD22	0.032661342	0.101579584	0.017284607	0.066021449	0.017052139	0.100626025	0.033587355	0.063918578	0.071087414	0.196979702	0.105544832	0.101970681
CD226	1.426794895	1.737644156	1.114277611	1.13404522	0.947415449	1.451040488	1.210835183	0.887827727	0.150748506	0.197466049	0.1342915	0.115327891
CD24	0	0.015727128	0	0.015332723	0	0.046738476	0.03120111	0.044533066	0.874989463	1.497150745	0.735347241	0.947260764
CD244	9.173691563	12.6734486	9.30321437	5.411967585	4.916081142	6.370619895	9.392225265	6.109558713	5.29948928	5.738690558	5.2019704	7.128661925
CD247	0	0	0	0.085021108	0.029279207	0.057592974	0.201847929	0.137188453	0.061029971	0.092242378	0	0.145906459
CD248	0	0.02180771	0.022264574	0	0	0	0.0216322	0	0.022892204	0.023066605	0.045317986	0
CD27	0	0	0	0	0.025005251	0.024592994	0.073878729	0.04686509	0	0	0	0.024921628
CD274	0.042447721	0.117347613	0.089854506	0.185907755	0.014774336	0	0.043651197	0.055380414	0.123183291	0.062060872	0.12192838	0.117799419
CD276	0.359006565	0.200404851	0.233832307	0.0279113	0.115343697	0.028360512	0.198791981	0.013511144	16.90480862	12.76384079	16.0632897	16.53957725
CD28	0	0	0	0	0.009807436	0	0.009658783	0.018381193	0	0.02059849	0	0.019549275
CD2AP	5.797137255	6.267022721	6.709479102	6.332710429	7.185233426	6.89694301	7.48257691	5.330917961	12.62746776	13.32811802	12.75612703	11.30112659
CD2BP2	12.13960214	12.44744382	13.19218756	16.14559693	14.16991632	14.16352214	14.07649007	19.0664798	21.76672055	17.96980809	19.60656313	24.16180558
CD300A	8.067926335	19.29776657	7.945172881	3.876213668	14.01617662	15.99455254	7.623315175	5.377417712	16.33828815	16.66790236	22.3937221	4.331944369
CD300C	4.881915804	4.926097843	4.478142078	2.171020843	2.650748161	2.361938955	4.172450159	2.929880689	4.486318081	3.901902175	4.510729357	4.764422441
CD300E	0.013302734	0.055163532	0.028159595	0.040335107	0.013890432	0.068307118	0.027359786	0.013016793	0.028953403	0.02917398	0.028658444	0.05537592
CD300H	0.036505808	0	0	0	0.038118588	0	0.075081639	0	0	0	0	0
CD300LB	0.422219745	0.534982858	0.595844268	0.189660571	0.1959435	0.457693423	0.55479898	0.229524535	0.612640876	0.334375269	0.429533113	0.512631594
CD300LD	0	0	0	0	0	0	0	0	0	0	0	0.042976899
CD300LF	13.73035887	17.55819304	12.28822624	16.35997864	10.28696313	12.08241646	12.65715517	15.53527054	19.16298561	12.95771219	16.93451025	22.30806686
CD300LG	0	0	0	0	0	0	0	0	0.188340682	0.110702391	0.155351658	0.1050636
CD302	0.027046776	0.042058901	0.057253361	0.054672198	0.042362502	0.027776054	0.083440816	0	0.044150483	0.059315782	0.072834511	0.056294445
CD320	19.14089095	17.92583287	23.08158283	23.1278255	25.91509417	26.15043283	23.22219275	30.17757762	9.829923248	14.10256429	14.50200899	11.23559041
CD33	21.6149948	21.60084063	21.22913415	17.81483814	15.78704261	16.89218395	19.64955132	19.3475815	26.66436696	27.8880541	25.8607813	29.29416452
CD34	0.076569991	0.178604445	0.081042734	0.038694535	0.059964566	0.058975943	0.157481801	0.074924124	0.10415912	0.083962112	0.061858809	0
CD36	11.80219966	15.87151315	9.383323934	4.568540254	2.595455198	5.303869505	7.441136348	4.107739147	1.522815239	0.908536132	1.170142178	1.849065436
CD37	22.96093049	31.17990273	20.1650996	16.41727143	21.50690857	25.21453721	23.73226426	19.46715488	77.6871179	70.37407328	67.0631282	84.74126536
CD38	2.702763331	4.124537273	2.930657696	9.532209038	0.434179687	1.513985166	1.52575015	6.15856169	6.767006173	7.585388483	4.794513949	17.78117476
CD3D	0.072617491	0	0.153718708	0	0.151651278	0.074575518	0.14935268	0	0	0	0.078220923	0
CD3E	0	0	0	0.107835761	0	0.036523766	0.109719434	0	0	0.038998265	0	0.14804732
CD3EAP	11.09863057	9.319947428	11.57394006	10.95302758	13.43226322	11.81754642	12.18651176	12.04363761	3.522033687	3.369630127	4.59538416	3.912438254
CD3G	0	0	0	0	0	0	0.036143153	0	0	0	0	0.036576681
CD4	38.05787199	42.28696462	35.33474445	21.05086298	36.43411443	40.28002904	45.61619971	32.76928919	21.64250788	18.53300548	18.88097672	13.61127508
CD40	0.928818657	0.727525945	0.851997879	0.083444831	0.107761409	0.063590861	0.382060998	0.060590251	1.707106842	1.516414684	1.645249739	2.040619635
CD40LG	0	0	0	0	0	0	0	0	0	0	0	0
CD44	28.25075096	32.5179872	24.78328615	26.44586026	21.60090627	27.08183158	25.82458283	24.10038196	19.02586112	15.66551701	17.26401478	12.72840659
CD46	53.55581698	70.03841981	47.85510743	59.46624683	52.6099202	55.40703803	53.16612063	49.37860829	58.04050059	61.73460918	55.43345959	56.31247947
CD47	8.616112596	9.323036913	8.158587061	9.336049069	7.816040457	9.197355328	8.521921572	8.415325956	11.70471871	11.75896174	10.23593622	12.53014035
CD48	1.455769683	1.089970314	1.398139421	0.817412354	0.365946083	0.332227206	1.6633818	0.896893569	0	0.088683929	0.02903893	0.028055561
CD5	0	0	0	0.015485526	0	0	0.015756027	0	0.10004258	0.03360158	0.132031216	0.063780067
CD52	14.81094844	20.98808539	12.51833411	1.723094451	6.453137268	7.331585592	18.18938192	2.710843835	4.522320842	3.738891048	4.935352985	3.437556166
CD53	48.60492509	44.94883237	45.75559019	33.00039642	41.90164335	34.03328676	47.23041175	33.54987221	75.11084769	58.25895785	68.80807063	56.94953155
CD55	7.794699245	11.73060388	7.3550449	7.862541517	6.517668641	8.368012297	8.442565613	8.710298714	4.483889398	6.911940995	4.388528991	4.111919143
CD58	1.798112463	1.421914199	1.699554556	1.732819329	1.33611753	1.399977424	1.487874424	1.342101375	3.849882303	2.604482797	3.270142746	3.977540685
CD59	1.025578264	1.173675828	1.007951451	0.93558644	0.354644397	0.424028271	0.62320548	0.690743862	5.623911817	4.739336504	4.124749951	6.771153981
CD5L	0.038559341	0	0.040811739	0	0.020131422	0	0	0.037730511	0.146867717	0.105704719	0.145371525	0.120384591
CD6	0.147154679	0.135604064	0.051916847	0.033050847	0	0.016791388	0.100884536	0.015999067	0.053380362	0	0	0
CD63	85.23329956	76.2292731	80.92271402	73.34077591	46.14954528	45.20090907	69.30326851	73.22846585	129.0840026	122.9353275	127.4313378	153.8610521
CD68	60.07188044	64.32005411	48.85173914	37.06747752	32.33162766	28.22570836	47.64310252	33.02155634	46.60050953	31.63219422	34.60213774	61.33931107
CD69	0.128423773	0.099852246	0.101944119	0.064898769	0.2346704	0	0.09904863	0.062831657	0	0.035205474	0	0.033412231
CD7	0	0	0	0	0	0.041055318	0	0.039118075	0.043505392	0.043836831	0.129186561	0.083207873
CD70	55.59276189	66.95719778	55.04467647	25.07342442	29.5461534	30.06523128	53.84397981	31.36933385	53.35497421	43.74832708	47.34453799	46.16952184
CD72	0.556171172	0.648652578	0.404703226	0.070265076	0.290371052	0.107093912	0.178731162	0.170067598	2.307528218	2.934972109	1.909591012	1.808749962
CD74	79.56687198	72.63411731	88.87262913	46.52619792	42.09076285	49.0772763	75.46461931	53.70968554	55.32624087	88.87663412	72.38669363	40.03935316
CD79A	0.256643375	0.576465487	0.226362406	0.389083082	0.357308735	0.571054023	0.571826059	0.376690266	0.744779104	0.844259728	0.921489724	0.400628052
CD79B	0.506046027	0.174871855	0.535606094	0.042621605	0.176134165	0.043307569	0.650491785	0.04126405	4.956341507	3.421883779	3.997358873	4.73971871
CD80	0.152665673	0.08440949	0.086177842	0.041146335	0.01062735	0	0.052331348	0.029876827	0.055379475	0	0.043852244	0
CD81	29.66705323	35.29714439	33.22889547	36.13379135	32.09585105	34.84083186	35.53864242	40.34104956	79.98459355	83.31599128	88.73829348	88.92452909
CD82	12.18621785	10.54693628	11.25946889	8.807133756	1.939864811	2.498417773	6.936796631	5.540135492	64.33465727	36.78982157	49.05184868	90.89175977
CD83	38.68228939	31.05507806	35.79308421	21.43491578	6.661321487	6.90207948	22.09457338	17.07775391	2.972028907	1.216585038	2.505085527	1.576495566
CD84	1.228410074	1.20610675	1.066273838	0.44669543	0.481853207	0.83434682	0.655012254	0.375229227	7.801637582	7.412072632	5.90188624	6.331074926
CD86	2.668394776	1.850567649	1.77247028	0.148796631	0.384314628	0.170090331	2.005994458	0.198078732	4.085459795	1.795960779	2.3390838	2.068358781
CD8A	0.043950091	0.015187618	0.108540558	0.074033717	0	0.060180189	0.075326937	0.071675644	0	0	0.01578048	0
CD8B	0	0	0	0.01021126	0	0	0	0	0	0	0	0.010514251
CD8B2	0	0	0	0	0	0	0	0	0	0	0	0
CD9	0.213784509	0.243792348	0.226272472	0.108035694	0.200906312	0.263458682	0.10992286	0.167351381	0.0465302	0.140654051	0.092112362	0
CD93	28.03300656	27.89622322	29.93399429	23.78793153	22.3120536	25.69640186	28.37362137	22.63738047	20.38746661	28.07132449	23.18987129	21.60232107
CD96	0	0	0	0.012911958	0	0.006559883	0.019706256	0.006250348	0.01390272	0	0.013761088	0
CD99	6.238611332	7.143633238	1.927512379	9.028957787	9.340910726	7.316671738	10.55328836	6.94734499	12.19907894	8.648937562	14.39433116	5.890577056
CD99L2	19.0478047	19.8983579	19.0048955	26.9856842	15.38011825	17.36900283	17.43258278	22.73996035	14.09853689	13.85320093	12.58987005	19.86337752
CDA	0.392361381	0.464867681	0.118651628	0.339907304	0	0.172688933	0.1729224	0.109693601	2.134936455	3.626310507	2.294317437	1.808297775
CDADC1	2.782886882	2.973097682	3.15904689	4.669867418	3.604561697	3.4578692	3.211318467	4.105390187	2.450523193	2.620604862	2.150967289	3.526184116
CDAN1	6.818020297	6.711229658	6.475220229	10.68445825	8.940988007	8.86430564	7.271003288	10.83832676	9.668108141	10.00607457	8.778329692	11.75403508
CDC123	111.9989697	98.27086555	125.1268793	101.3222392	108.4172917	102.6673636	122.8240548	101.4283154	98.08886341	93.69040836	108.9215736	95.86374801
CDC14A	2.054243319	2.340329401	2.112776859	2.134909492	2.978741651	2.407814396	2.224454341	2.074012555	6.280023521	6.240311546	5.45761084	5.899789558
CDC14B	4.304239962	4.101673623	4.375106965	3.760076893	3.370796845	3.557800527	3.508631545	2.844195494	5.255319741	5.302551383	4.996820447	6.760018272

CDC14C	0.167341412	0.11565471	0.216475672	0.169131485	0.038829854	0.076379348	0.095603262	0.109162947	0.2428124	0.203885191	0.240338785	0.232199995
CDC16	42.82891064	42.2235913	42.8534952	41.18681671	45.68032409	41.80476154	42.55859253	42.12234266	47.75116409	50.00980509	47.71237801	57.38748942
CDC20	39.70663715	35.76844579	44.10015364	102.4389295	75.23999363	74.56037992	66.76559659	111.0898201	422.7731359	485.7733267	493.1416351	354.8157095
CDC20B	0.013000619	0	0	0	0	0	0	0	1.499679934	0.541716894	1.13430732	0.920010901
CDC23	16.42428118	15.38262627	17.47394368	20.47847236	19.58969207	20.14248094	18.71398546	21.32819093	25.63199778	27.80966867	26.30781605	29.02003157
CDC25A	9.246748222	8.925459471	11.06082963	11.09171202	12.84903369	12.53539876	12.45041277	11.25109942	12.97527696	14.26974211	17.40376418	14.11761713
CDC25B	9.168948482	10.81873875	10.6603924	21.34839718	15.29747804	14.45273532	12.78255277	22.86536258	16.74848052	19.20049105	17.4830873	16.00341491
CDC25C	3.16157342	3.494647313	3.457055986	7.279571205	6.340144593	6.966688162	4.327769939	7.805745405	14.2863919	16.18602487	15.71957488	9.870623025
CDC26	7.879764189	8.168914919	10.40627996	9.363145781	9.339758084	9.331581394	8.541180266	10.55837085	10.3519874	11.71524847	11.50822744	11.70953228
CDC27	14.54129235	13.76593995	13.6238228	19.00483293	18.14789354	16.61294731	15.49384701	15.45591471	31.76900718	29.86457624	29.72933367	28.76667843
CDC34	23.1375179	25.03212517	25.21118158	23.0552341	23.10658863	22.63424516	28.70473703	27.16821439	18.43261565	18.54051454	19.04364685	19.38660886
CDC37	197.4095973	194.0091843	205.1421122	168.9240803	161.4640862	171.189589	197.5910232	197.7435564	92.27167679	106.4272449	111.3283023	113.4061899
CDC37L1	2.601713694	2.538526585	2.316338944	2.846095285	2.892437586	2.37324495	3.493858723	2.515839579	6.11230954	4.32967252	5.060933738	6.211481953
CDC40	19.10497775	17.76462946	18.18113704	19.11173586	18.73865462	18.70221593	19.77589571	17.13645786	21.55089762	23.06270121	20.02258696	21.21941145
CDC42	101.2574843	99.38591064	100.4210214	104.7539365	93.67434574	89.15237214	107.408104	97.79593026	107.5578763	108.5643084	107.3256015	94.40828619
CDC42BPA	0.018208125	0.00471907	0.01927173	0	0	0	0	0	9.942172568	8.465562888	8.796488991	8.58387655
CDC42BPB	1.861689684	2.007515287	1.96252463	1.790924685	2.365513406	2.58757462	3.052391503	3.240968077	9.340680715	7.591781388	8.174396167	13.33640581
CDC42BPG	0.018709861	0.009698213	0.019802775	0.028365004	0.00976822	0.019214346	0.038480645	0.027461541	0.315595614	0.143612872	0.372841279	0.253124369
CDC42EP1	5.283046347	7.633839247	6.260919855	2.556391549	14.03605015	13.82558857	11.5998286	6.067661187	0.91011696	2.057771964	2.08732443	0.636833573
CDC42EP2	1.861922521	2.040546221	1.238715955	2.150673993	1.249829925	1.420436898	1.64118145	2.056145001	1.418366088	1.341671389	1.575824865	2.10376486
CDC42EP3	41.0737176	28.55092843	40.27230792	15.80251464	31.65441226	23.61601811	38.65003852	17.80298179	13.91402773	13.82872321	14.02284848	12.69204624
CDC42EP4	3.324967196	4.158510206	2.695894126	4.979141003	3.965564869	4.542384633	3.921142849	4.148955236	5.84254719	5.050827963	5.618736579	5.587191721
CDC42EP5	0.966277187	1.189560395	1.022721101	0.244153394	0.756724561	0.434145017	0.496836524	0.177282587	0.722941445	0.463558497	0.520419337	0.314247468
CDC42SE1	68.4495482	66.26445622	65.02243395	64.46598841	58.62279556	59.13198928	67.62607699	61.10584896	132.5701541	125.9427331	122.6246033	151.26564
CDC42SE2	18.60207723	19.24001207	18.70045942	19.65763969	18.89892319	17.43669568	19.24765717	17.09182083	30.81108507	30.57327566	27.97510951	33.99399251
CDC45	13.48577084	17.62575226	17.38261067	23.99874892	25.95560549	27.69879065	16.5685118	24.73685963	18.21166638	20.05855902	23.01209078	22.5107236
CDC5L	19.41728712	19.32323121	21.224474	26.50769645	24.11000688	22.37223789	23.65853726	23.99342863	28.71466772	26.5574726	27.00734233	27.08528829
CDC6	14.40584034	17.48972927	19.03714138	26.0564039	23.65027736	22.33678703	17.74287648	22.55390059	30.88309193	25.34759257	33.74372163	30.08792584
CDC7	6.524675413	9.208006521	8.547746431	15.38709829	15.00749709	15.80655287	8.868015881	14.0338359	23.00621179	25.81649844	23.91862276	24.4540132
CDC73	15.54343533	13.19634096	15.49233688	15.84179377	15.01104562	13.39457683	15.70637132	12.48972474	11.10293378	10.92956675	10.93351437	11.16169761
CDCA2	4.461922951	4.363557964	5.575948493	11.08421531	9.475056328	8.210125273	5.64945714	10.1294205	15.58579134	15.06016412	15.71406188	11.69043679
CDCA3	10.5073526	10.52795452	12.98420228	25.48192852	23.21558212	19.91267656	13.97999712	29.59905432	10.3147408	10.27454128	10.70555875	8.736617332
CDCA4	12.54752092	13.73567448	14.97535424	19.13346943	16.94995148	15.74686528	12.06861591	20.37000774	22.1771793	21.48019009	27.07872499	23.2624859
CDCA5	13.26987574	15.62667735	16.77220774	31.323195	25.82888748	24.69741165	19.04860528	32.6787129	27.07482064	28.92925297	30.25295626	31.04594445
CDCA7	30.42825345	32.69624499	34.47568719	35.43745328	56.8466436	56.97136956	39.22692328	38.44975406	53.99446407	62.09108005	63.18031968	47.50938606
CDCA7L	34.9889312	38.26918815	36.74435013	34.90418822	47.63168513	50.31649452	35.75675602	37.50765657	25.87871657	30.13964159	29.15696774	30.68406915
CDCA8	14.76191493	15.50556239	17.84642664	36.7745247	29.42687523	26.85222605	20.18865538	39.8179828	44.02469125	49.34436616	48.19261523	37.25751355
CDCP1	0.025758526	0.008901248	0.018175453	0	0.044827508	0.017635379	0	0	0.168190303	0.150641454	0.166476889	0.044677596
CDCP2	0	0	0.061042638	0.097151069	0	0.019742929	0.019769621	0.037622671	0.020921135	0.063241559	0.124248025	0.10003376
CDH1	0	0.011419472	0	0	0	0	0	0	0	0.084550792	0	0
CDH10	0	0.015961122	0	0	0	0	0	0	0	0	0	0
CDH11	0.014138087	0	0	0.014289324	0	0	0	0	0	0	0	0
CDH12	0	0	0	0	0	0	0	0	0.012181872	0.012274677	0	0
CDH13	0	0	0	0	0.023069923	0	0	0	0	0	0	0
CDH15	0	0	0.03984105	0	0.019652605	0.019328597	0.019354728	0	0.081928306	0.041276232	0.081093674	0
CDH16	0	0	0	0	0	0	0	0	0	0	0	0
CDH17	0	0	0	0	0	0.014511675	0	0	0	0	0	0
CDH18	0	0	0	0	0	0	0	0	0	0	0	0
CDH19	0	0	0	0	0	0	0	0	0	0	0	0
CDH2	0.155422705	0.218670995	0.352503288	0	0	0	0	0	8.626077067	8.983954667	8.215327092	11.05651871
CDH20	0	0	0	0	0	0	0	0	0	0	0	0
CDH22	0	0	0	0	0	0.011992287	0	0	0.012707955	0.038414306	0.012578495	0.012152539
CDH23	0.098056638	0.101654861	0.078876219	0.027749558	0.049146555	0.080560476	0.060502042	0.026865697	3.376308866	3.720299133	2.902521351	5.306404876
CDH24	1.478976234	1.72898155	1.920629857	2.957789871	3.428161398	2.305212112	1.855292178	2.863580312	1.267050007	1.173186391	1.107260616	1.822966103
CDH26	0.051428425	0.079973414	0.043546043	0.181924964	0.096660841	0.08450419	0.074041131	0.135872031	0.335801914	0.310163489	0.227127005	0.465631673
CDH3	1.280309062	2.604017464	1.419625185	1.133794587	0.80212289	1.728063286	1.705321285	0.966437162	0.331737237	0.347635108	0.433432182	0.241101083
CDH4	0	0	0	0	0	0	0	0	0	0	0	0
CDH5	0	0	0	0	0	0	0	0	0	0	0	0
CDH6	0.044187691	0.034356881	0.081845508	0	0	0	0	0	0	0	0	0.005748193
CDH7	0	0	0	0	0	0	0	0	0	0	0	0
CDH8	0.004324485	0	0	0	0	0	0	0	0	0	0	0
CDH9	0	0	0	0	0	0	0	0	0	0	0	0
CDHR1	0.49440123	0.429428302	0.417210591	0.027010265	0.788317388	0.528315782	1.003095916	0.235349568	1.505035909	2.644720529	2.64116529	0.88302212
CDHR2	0.036889553	0.063738713	0	0	0.012839762	0	0	0	0.16058023	0.053934529	0.013245362	0.102374587
CDHR3	0.031414762	0.024425654	0.049874726	0.011906554	0.053304268	0.052425451	0.036343612	0.034581942	0.055554064	0.034447566	0.03383884	0.024519696
CDHR4	0.03302213	0.102701666	0.069902156	0	0	0.084781311	0.13583349	0.016156161	0.125777171	0.14484044	0.088925596	0.017182847
CDHR5	0	0.031181734	0	0	0	0	0	0.014715743	0.016366198	0	0	0
CDIP1	6.204495764	5.717486157	6.823290281	4.915003118	4.961091984	4.841030477	5.978037527	5.797656235	10.76677095	11.29827528	11.86127661	10.64522004
CDIPT	16.7118786	17.90572766	17.52210882	19.01895988	14.12857051	14.42477416	15.82650305	21.08747587	38.32372194	26.6035543	31.49043434	39.42303827
CDK1	29.70867441	29.99866263	36.86258766	62.40920987	65.57867861	55.13104297	43.76921066	62.5330592	42.70158617	49.84971439	46.91572325	35.55667786
CDK10	18.06564445	16.88491903	17.22001634	16.30131362	20.04044846	21.08432418	16.73092118	20.36510513	15.67429787	14.32632388	13.8265975	17.7012226
CDK11A	12.71651884	10.21737447	12.6717313	13.27038226	10.91274024	11.63288694	13.40015743	13.23604652	11.35533504	10.17254272	10.0462203	12.39065985
CDK11B	10.67795771	10.52581916	11.69045389	13.80179437	11.88935671	11.85499842	12.27571984	14.50458533	14.88938618	13.24793485	14.2007587	16.20445951
CDK12	13.50717137	10.78821624	12.84774229	13.05829058	12.25619065	11.57009217	12.33402397	11.11314592	13.31789788	11.88701264	12.29598646	13.06712684
CDK13	20.96341028	18.80235095	20.33842486	18.78940647	25.69893	20.62192363	20.19582301	16.33704693	17.54212145	12.1827628	14.37154366	13.83382095
CDK14	0.015382071	0.031893043	0.045585669	0.055967814	0.064246525	0.104259055	0.053781822	0.105360042	1.496514831	1.612491621	1.441503693	2.28272911
CDK15	0	0	0	0.005399103	0	0	0	0	0	0	0.005754165	0
CDK16	10.81830647	12.20917109	11.23138692	13.35642743	12.53284574	12.94397816	9.984494047	14.59566764	13.81872582	19.39672184	16.18877976	16.05138742
CDK17	6.377512753	5.935225323	6.095280942	6.729937251	6.600539471	5.960367187	6.211326328	4.732600163	6.695519777	4.896475045	5.439967588	6.742342335
CDK18	0.617318098	0.411409802	0.528925038	0.445658306	0.537158687	0.618868834	0.287180604	0.359552911	5.406361892	5.366964397	4.955480264	5.674840609
CDK19	10.98275459	10.40720982	9.86494557	10.23021982	11.92667703	9.90713208	9.621384	8.632198973	26.45587629	21.00696337	22.3559263	20.01108047
CDK2	27.28992044	34.86222411	32.56441238	46.13285876	42.76631176	44.05015695	29.37629771	43.50057882	38.51193348	43.24574185	45.77670747	42.66909961
CDK20	2.446856267	2.470471186	2.49970689	3.849325891	3.799107381	4.829008044	2.144265106	5.100817493	12.10991239	11.52556778	10.08428217	13.32990791
CDK2AP1	24.30499266	26.63848508	29.18030143	27.74254668	21.6540134	20.92446577	34.3824133	28.92953028	6.87566052	9.281586976	8.954757588	7.109972549
CDK2AP2	9.515307435	10.24255592	12.59768853	17.659263	10.55882537	10.55498592	11.38752068	20.27608146	13.85481868	12.76065027	13.60653672	13.59431062
CDK3	5.243861664	4.79672384	4.389354483	5.092114512	6.083920871	6.159605254	4.617138212	6.472621125	5.855825706	4.058899637	4.540948758	4.458511054
CDK4	91.73428133	95.62992291	99.90917479	101.8703208	101.5403783	111.4318038	92.20685317	114.9580168	54.78502272	67.75777054	76.25930441	56.33455657
CDK5	18.04176391	20.2936369	16.94739092	14.63339407	10.64393671	11.81174932	17.44008644	14.65278475	15.80533007	16.42032882	15.54714599	18.02479225
CDK5R1	0.637050839	0.59438488	0.685501117	0.858487271	0.709541261	0.708747002	0.807972065	0.706471809	1.906494162	2.025801329	1.898508841	1.679524985
CDK5R2	0.08514171	0.088266015	0.02252879	0.02151312	0.044451581	0.021859359	0.065666734	0	0.46327735	0.443466421	0.458557782	0.664543894
CDK5RAP1	8.260494748	7.628855562	8.850770067	10.31848334	9.536570146	9.483889511	9.631310301	10.11790055	8.577993332	9.265281486	9.49318714	10.17061082
CDK5RAP2	9.049131972	10.02543304	9.271383003	15.09121748	15.08447987	14.44237898	10.15484808	13.31941661	28.19986308	28.27467962	27.06896839	27.44575045
CDK5RAP3	43.27632146	52.43559275	40.5703478	45.70376453	50.70989157	58.34271522	42.54855538	52.26135276	42.4139089	43.47360809	35.03873507	63.59928059
CDK6	39.82308901	25.22191329	35.38483619	20.85703896	30.1820673	23.3637463	33.07726736	17.67462575	79.64699531	37.30504497	56.31389062	35.26561404
CDK7	19.18894564	20.41476274	21.58808668	21.69981789	21.15762243	21.63564089	25.08021636	22.5186464	15.8078088	16.00180975	16.62243372	17.35127975
CDK8	10.03468649	9.563753433	10.45766209	9.804394053	11.4439683	11.54558291	11.42906404	9.894425643	3.10409099	3.775829113	2.975588894	3.957897339
CDK9	19.83030338	20.08408912	18.73082977	15.70833753	16.41054555	16.47530662	19.38521658	16.7202867	26.910267	25.65926473	26.00304586	28.36190184
CDKAL1	4.244496691	4.257384736	4.229888999	5.710581985	5.727081916	5.443961856	4.355388833	5.618589756	2.099578962	2.216315922	2.078189819	2.285083972
CDKL1	0.640243558	0.678822465	0.592937285	0.301486197	0.676125619	0.597733462	0.591059798	0.377312764	1.29847947	1.180725717	1.136350329	1.113012186
CDKL2	0	0	0.011498644	0	0	0.011156967	0	0	0.011822786	0.011912856	0.011702343	0.03391817
CDKL3	0.611081065	0.508588442	0.419038378	0.461038697	0.350493466	0.477297627	0.513346087	0.370558037	0.693107969	0.745576707	0.760214283	0.644900946
CDKL4	0.213597812	0.230293294	0.189902891	0.207247387	0.303326636	0.228131458	0.219653731	0.133764188	0.009297913	0.018737496	0.018406384	0.017783074
CDKL5	0.117165568	0.158432609	0.113226205	0.221391863	0.085107335	0.068009653	0.120487443	0.11963176	0.182942581	0.296055272	0.170104403	0.206755378
CDKN1A	368.2598198	233.7136809	297.3120923	59.58238608	56.61294045	40.17227875	255.7126871	59.14219708	33.04311206	20.36176741	32.44272833	30.28230176
CDKN1B	25.63578762	25.4267697	26.70437585	31.21273251	40.57564293	33.22636371	27.52502405	33.09851142	23.62754883	19.99553709	20.67599417	18.53312744
CDKN1C	0	0	0	0.027620705	0	0	0.056206367	0.026740949	2.200767406	1.55826687	2.060598911	1.251372041
CDKN2A	0	0	0.012815657	0	0	0.012434847	0	0	0	0.026554624	0	0
CDKN2AIP	7.620645921	7.0293903	7.271919107	8.930266045	9.664693269	9.243455573	7.435614976	8.777934524	10.3501352	8.438596053	10.06694769	9.944605118
CDKN2AIPNL	10.43091042	11.37806448	11.86996454	10.10230368	10.86899625	10.84634658	12.98842467	10.80333252	10.52200167	12.0587653	12.36172312	11.44453416
CDKN2B	0	0	0	0	0	0	0	0	0	0	0	0
CDKN2C	34.54843072	34.17283968	35.55526742	67.44375428	58.95305748	54.32384062	44.59416072	70.33139336	145.3558673	88.41131637	122.8470007	136.6978769
CDKN2D	10.81485201	11.29011036	12.16700317	16.20471643	10.85830613	11.53363049	10.22709006	15.94758405	6.254993024	8.583208356	7.616892891	6.768665214
CDKN3	8.335332693	8.841382924	9.810048454	28.88398906	24.29608577	20.95402556	13.99926795	29.06617993	34.4975378	38.28932187	36.46872742	30.57834608
CDNF	1.296169739	1.266948394	1.29349056	0.598873157	0.541373146	1.140959227	0.99016818	0.253661733	1.128445422	1.29947693	1.15684061	1.310366525
CDO1	0	0	0	0	0.033624655	0	0	0.031509831	5.431795239	4.978825125	4.821470262	5.462489833
CDON	0.992567413	1.080439526	0.97175601	1.059614191	0.485820832	0.694418951	0.688978341	0.570598947	4.219380985	5.0610162	3.962565168	3.17632675
CDPF1	2.012390081	2.358353112	2.708729842	2.343425964	2.877860418	2.246360104	2.721770453	2.547031899	3.07073787	3.238044934	2.780276842	4.006425283
CDR1	0	0	0	0	0	0	0	0	0	0	0	0
CDR2	6.432453056	5.945901943	7.377303278	8.836080316	6.467096363	6.360474691	7.331602554	8.554639354	6.479984775	7.206307748	7.314930032	6.880693714
CDR2L	0.015217636	0.031552105	0	0.030760842	0.031779863	0.015627958	0.046947258	0.07445267	2.401285994	1.985724137	2.311255717	3.864177408
CDRT1	0.071270942	0.044331752	0.060347318	0.028813334	0.01488392	0.029277064	0.029316645	0.069738978	0.046536363	0.015630298	0.015354094	0.044502436
CDRT15	0	0	0	0	0	0	0	0	0	0	0	0
CDRT15L2	0	0	0	0	0	0.052729445	0	0	0	0	0	0
CDRT4	0.043082114	0	0	0	0	0.044243762	0	0	0	0	0	0
CDS1	0.071145299	0.122926667	0.025100388	0.179765872	0.037144204	0.097418173	0.048774939	0.069616036	0.812950682	1.625285763	1.034574261	1.345059492
CDS2	5.564675329	6.192731094	5.097313266	5.248539065	3.869980802	4.231509359	4.717740778	4.541016317	5.472140264	4.657933637	5.11897817	5.840816664
CDSN	0	0	0	0.02105504	0	0	0	0	0	0	0	0
CDT1	19.43178908	26.44271638	23.84565049	35.25378779	41.42069791	42.28716213	26.12647366	43.69700967	31.3478221	36.28493301	39.37062263	38.6026054
CDV3	52.11908351	47.77441042	51.06850058	52.30361237	58.98370785	56.50748661	56.60836643	50.46293927	45.24014019	45.57174134	47.57476119	47.07872026
CDX1	0	0	0	0	0	0	0	0	0.297417784	0.199789078	0.228968355	0.189612524
CDX2	0.035408792	0.012236043	0.012492385	0.035787564	0.012324369	0.02424236	0	0.011549228	0	0	0	0
CDX4	0	0	0	0	0	0	0	0	0	0	0	0
CDY1	0	0	0	0	0	0	0	0	0	0	0	0
CDY1B	0	0	0	0	0	0	0	0	0	0	0	0
CDY2A	0	0	0	0	0	0	0	0	0	0	0	0
CDY2B	0	0	0	0	0	0	0	0	0	0	0	0
CDYL	13.92235814	12.95328699	13.44905448	12.51416015	14.92115929	14.35581653	14.54964619	12.58580635	8.336899219	8.229924554	8.054042878	7.707754923
CDYL2	0.493719619	0.530116722	0.454129223	0.87919224	0.122745601	0.150902399	0.10275236	0.132279339	0.454138313	0.393147661	0.335551097	0.795178247

CEACAM1	0	0.015444109	0	0	0	0.030598261	0	0	0.194545705	0.032671304	0.04814095	0.062014284
CEACAM16	0	0	0	0	0	0	0	0	0	0	0	0
CEACAM18	0	0	0	0	0	0	0	0	0.0739839	0	0	0
CEACAM19	0.765972397	0.868525026	0.506697343	0.556431885	0.59985906	0.368730818	0.393844611	0.491864544	1.19825615	1.548602352	1.186049096	2.241948797
CEACAM20	0	0	0	0	0	0	0	0	0.010602618	0.010683392	0	0.04055687
CEACAM21	0.485066086	0.407728993	0.527276328	0.132501324	0.184802481	0.175023989	0.458373909	0.205249568	0.806076629	0.503143648	0.854350852	0.532088453
CEACAM3	0	0	0	0	0	0.047515442	0	0	0	0	0	0.096300772
CEACAM4	0.321950051	1.061976752	0.63504597	0.946600704	0.091683653	0.225430209	0.225734979	0.486864173	2.309205133	1.219562809	1.324118332	3.746458829
CEACAM5	0	0	0	0	0	0	0	0	0	0	0	0
CEACAM6	0	0	0	0.020885241	0	0	0	0.020220019	30.5609337	20.48385346	25.06331733	42.96689703
CEACAM7	0	0	0	0	0	0	0	0	0	0	0	0
CEACAM8	0	0	0	0.016963559	0	0.017236575	0.051779633	0	0	0	0	0
CEBPA	230.2705609	246.671407	241.0373317	258.9858523	160.1462573	178.5725651	199.6987529	269.2283712	129.2465978	113.3215901	125.264252	190.8985647
CEBPB	4.4671125	4.260551989	4.701036028	5.856467397	2.931948695	3.722478708	4.305013061	4.920600987	6.694753298	6.605802821	8.138835049	9.589944251
CEBPD	18.06556	23.18764451	16.25271217	17.30239577	10.10553932	13.60529276	11.05818703	18.0139515	33.18769325	34.10084944	32.24727877	54.7455262
CEBPE	0.382614848	1.009667361	0.331334862	0.527328719	0.254238908	0.250047324	0.286154714	0.37439057	0.302822273	0.495835079	0.224802988	0.217190299
CEBPG	24.93368826	24.41694174	27.5462239	29.10862734	31.17410339	29.68025563	27.39645969	30.233051	18.22601841	17.07079426	20.48809637	17.40329754
CEBPZ	34.43488715	31.84553013	37.21599163	39.89878886	41.10241485	41.17153084	41.54291883	35.40237433	27.85473849	25.83434769	30.16445419	27.37723943
CEBPZOS	13.81655302	12.46956781	15.127367	12.71076575	13.97516728	13.28561547	14.87568303	12.3764723	12.11658388	14.62853023	12.47473815	12.12734127
CECR2	0.279841698	0.294255025	0.224256865	0.214146652	0.204543323	0.184748933	0.250776021	0.136913261	1.144191798	0.889887667	0.908612145	0.61157787
CEL	0.96974481	0.981950172	0.71608694	0.501455871	0.470970651	0.486366148	1.205963424	0.507551279	0.98169752	0.494588218	0.315801403	0.680623666
CELA1	0.847839719	0.761757964	0.658067851	0.171381834	0.472157972	0.290233501	0.813752469	0.221230791	0.184532323	0	0.182652427	0.176467118
CELA2A	0	0	0	0	0	0	0	0.057231444	0.063650279	0	0	0
CELA2B	0	0	0	0	0	0	0	0	0.126886798	0	0	0
CELA3A	0	0	0	0	0	0	0	0	0	0	0	0
CELA3B	0	0	0	0	0	0	0	0	0	0	0	0
CELF1	15.85612555	16.1340764	15.96437907	15.0453423	16.3117578	14.64708668	15.41777211	14.46703875	23.47318407	24.96698566	22.06287908	22.10336885
CELF2	25.04189214	19.67584703	23.50475864	17.17932789	21.05141092	17.71356557	22.25866584	14.54889117	29.52635881	23.77731563	25.89282379	25.28169024
CELF3	0.011132629	0	0.005891464	0	0	0.005716402	0	0.005446667	0.006057542	0.00610369	0	0.00579279
CELF4	0	0	0	0	0	0	0	0	0	0	0	0
CELF5	0.074220084	0.034974371	0.035707073	0.013638914	0.0211361	0.013858422	0.034692895	0.03301124	0.036713641	0.022196003	0.043607551	0.014043611
CELF6	0.36123901	0.327682953	0.334547806	0.486804306	0.220032218	0.247319535	0.185740424	0.382930388	0.524157825	0.412618002	0.437752727	1.268786239
CELSR1	0.051312359	0.044329404	0.081464563	0.021608856	0.026789637	0.026347962	0.013191791	0.008368234	7.989870262	8.130450184	7.328120671	16.14017849
CELSR2	0.1786845	0.111144835	0.140490732	0.092877897	0.277202422	0.157287833	0.147000446	0.134879418	4.572433165	5.279044119	5.543206574	4.930453332
CELSR3	0.598283865	0.685526324	0.447547353	0.463742451	0.535470243	0.595937064	0.508850398	0.510595192	0.504221742	0.739897689	0.566921863	0.454095735
CEMIP	0.007191869	0	0	0	0.007509596	0	0	0	0.046959308	0.031544707	0.061974556	0.02993793
CEMIP2	0.297868604	0.191956134	0.281185186	0.471923965	0.151310948	0.198421754	0.157296258	0.480524929	1.839801613	1.783196233	1.482862257	2.907184006
CEMP1	0.039162708	0.040599797	0.124351051	0	0	0.040218674	0.040273048	0.038320909	0	0	0	0
CEND1	0	0.033890718	0	0	0	0	0	0	0	0.035847129	0	0
CENPA	9.400691974	9.628705841	9.870223454	27.78166195	21.75226166	18.76770291	14.07547247	27.70625788	27.04892237	26.84266087	29.77068637	24.61447056
CENPB	35.7340957	37.26143622	39.24527923	49.29134647	37.74803232	40.86160669	38.16957531	53.7838539	24.59859179	26.53119227	27.28690063	32.53457151
CENPBD1	3.061760494	3.101144764	3.054367604	3.272358084	3.270519999	3.831006284	2.967615282	3.374746224	8.100112079	7.312837763	8.245042868	9.082882218
CENPC	6.131323883	5.583766466	6.005304926	7.419887941	7.56553356	7.19833205	6.107885663	5.732404373	10.86374905	10.85751685	10.71333834	9.621091491
CENPE	5.671396548	4.979453231	6.022516978	13.7683172	12.53523238	10.56459376	7.181046743	10.57947985	24.56523982	25.22496932	25.71440875	15.87989668
CENPF	20.11659274	20.75302146	21.1659194	60.30613454	47.91197633	48.59694705	27.51352097	45.67702192	49.2426818	44.04934859	47.06049614	33.40256898
CENPH	14.10128161	16.38583272	14.80198276	20.20356155	30.33844419	29.63933883	18.8434373	20.28028557	18.67624757	27.01712173	24.07768395	15.84418088
CENPI	2.988970037	3.518522805	3.395047628	6.917878589	5.396233257	5.38213408	3.931687376	5.897001155	4.451591121	4.991789611	5.575422044	3.742813651
CENPJ	9.170048982	9.699648906	10.09241804	15.19106427	16.59945495	17.01000935	11.47812429	13.29116657	8.287501965	9.254047794	9.175404826	8.484455715
CENPK	5.758618938	6.164160781	6.606397391	9.86646819	11.6450924	10.10627408	6.185532785	9.03117847	7.694020582	8.844709221	8.762764434	6.783080838
CENPL	6.718954855	6.585208482	7.724489176	11.10339467	9.676951325	9.834656422	6.710903029	10.44745941	11.30403384	9.27302331	11.0640927	10.14691206
CENPM	5.352236767	5.169707542	7.709765155	12.21753181	10.08690708	10.51048023	6.255746781	13.33567617	11.64914381	10.64998897	13.52720983	10.89546746
CENPN	5.07841237	5.468432521	6.601864937	10.53352754	8.349042367	8.302182804	7.060840151	10.611324	9.503156358	8.853887498	9.247632165	10.36562311
CENPO	5.326357885	5.484076	6.241048864	11.72316148	9.032962215	8.996178373	6.83735558	11.36163527	15.65954744	14.00938133	16.7154496	14.3564257
CENPP	2.817800898	2.737273621	2.88298602	4.261366973	3.923050331	3.954831112	3.198192403	3.911185327	2.430462958	2.277321663	2.484394203	2.899865398
CENPQ	8.207226421	9.900676064	9.760626413	13.93563388	12.40285405	14.00667197	10.74171312	12.96611213	15.78442204	18.22819551	18.38829423	16.21265956
CENPS	0.346320585	0.448786176	0.412369293	0.35002522	0.632836026	0.577945263	0.311622023	0.55067423	0.423993816	0.61710126	0.932609874	0.540616883
CENPS-CORT	0.625401684	1.115163753	0.608979066	0.935495694	0.940364831	1.001933001	0.926111596	1.272874139	1.197844403	0.9052275	1.347319936	1.822372347
CENPT	13.22779648	14.59636345	13.47587399	16.59420804	17.54826612	19.10414817	14.65410318	19.80926233	18.57547475	18.73397378	18.01918248	24.19526401
CENPU	11.57044962	13.99764416	14.33304637	21.73796524	24.3087963	21.80149846	15.42094057	21.18198856	25.31311778	25.15656523	29.13101168	28.53829893
CENPV	16.27230706	15.95885956	20.89247218	19.53006914	25.48679526	25.87366832	21.44000061	24.01950596	6.238164842	8.009184834	7.917449211	4.666574903
CENPVL1	0	0	0	0	0	0	0	0	0	0	0	0
CENPVL2	0	0	0	0	0	0	0	0	0	0	0	0
CENPVL3	0	0	0	0	0	0	0	0	0	0	0	0
CENPW	21.49011436	22.54080407	25.68896009	38.79787674	32.73530006	34.61892471	29.89919016	40.0772485	19.67227273	23.93442906	22.73986964	16.48832104
CENPX	11.87364871	13.68141846	15.92916863	18.26852466	17.33380506	15.92184681	15.2434194	21.38678325	15.10251605	12.2735733	15.80403445	19.00735639
CEP104	7.317199699	6.727104933	7.038247181	6.712813697	6.532177127	6.127205657	6.854879583	5.775141869	5.145286185	4.629004264	4.5298821	6.9873103
CEP112	0.011043522	0	0.011688616	0.011161656	0	0.005670647	0	0	0.03605434	0.030274179	0.023791361	0.034478542
CEP120	7.035907431	7.843110256	8.577949662	9.529352037	9.845033082	9.537042234	9.374886087	7.671226288	6.895260498	7.279625798	7.375091995	6.544686771
CEP126	0.055466626	0.064689743	0.051368312	0.08408994	0.057917071	0.085443307	0.049909313	0.054274375	0.490437664	0.197669596	0.246454867	0.570018427
CEP128	1.46656173	1.742007138	1.602009105	2.424320984	2.236756942	2.283308578	1.903863941	2.213222017	2.722016717	2.419249675	2.634413547	2.576349815
CEP131	2.500157096	3.247157175	2.809730009	4.698901281	3.534591091	4.529309316	3.148803602	5.250174529	5.777870313	5.80648633	5.249989864	7.791024036
CEP135	3.659237792	3.20017019	3.912710695	4.921691805	5.192502751	5.03944548	4.158580184	4.764929346	6.034512628	5.257408072	5.396957237	4.891970064
CEP152	4.434701149	4.434097858	4.615274475	6.894158472	8.525761211	7.642808858	5.280000518	5.785836767	5.330354609	6.692108252	5.540603777	4.779099961
CEP162	2.478180574	2.492044737	2.509279741	3.105815585	3.553724445	2.969168022	3.117593892	2.683569574	6.139897079	4.338823352	4.671460611	6.077866361
CEP164	11.26462057	10.28225633	11.19707499	10.80462835	10.74984429	11.24489763	12.71445037	12.6299346	7.379429158	6.819396875	7.277643039	8.027387197
CEP170	22.04490507	19.69532281	20.37243758	18.58339061	22.38588657	21.29602652	19.00642723	14.56800388	22.14451085	21.39783141	19.20410992	18.67552209
CEP170B	0.206889638	0.164015285	0.231855719	0.202952682	0.190614493	0.112483123	0.168952791	0.238167718	3.158689203	2.929200536	3.126510533	4.559448961
CEP19	3.268577133	2.806907302	3.666044853	3.574727764	2.44511907	2.104206038	3.637171053	3.055110982	5.892988698	4.119072371	4.519226215	6.650821167
CEP192	7.789436797	7.237080044	6.964296859	8.736439862	10.02796394	9.963894068	6.955764445	7.184622641	12.46848791	11.31649984	10.98907453	12.45712128
CEP250	7.477860747	7.415427136	7.22175228	7.097127265	8.451312808	8.262175627	8.173606719	7.654035093	7.747253187	7.317052911	7.610868983	7.363229398
CEP290	3.64235413	3.641154044	2.939228045	4.26438257	4.547386477	4.786064522	3.512974711	2.994033448	5.114768932	6.425113709	4.50826789	5.874175009
CEP295	3.288393825	3.37348977	3.498640407	4.219489114	4.920599646	4.2345404	3.446318246	3.189729955	9.111413364	8.227626649	8.340965751	8.6536719
CEP295NL	0	0.077227672	0.026281856	0	0	0.051001808	0.076606139	0	0.108090922	0.217828793	0.347716727	0.10336668
CEP350	18.46156187	16.69980897	15.6984964	17.13059366	17.71038736	16.66405974	16.12915726	11.59986405	20.60802667	18.20654722	16.70011273	18.26841951
CEP41	1.386676825	1.510562587	1.576607093	2.271979562	2.098379534	2.119411588	1.665513911	2.067107112	2.894312887	3.041095195	2.847323473	2.89746668
CEP44	2.541695847	2.579877913	2.577685131	2.434622426	3.763664754	3.742540932	2.978044749	2.47839656	2.717812457	3.792167245	2.508875586	2.4561729
CEP55	19.84252636	21.44423172	24.10194138	51.95182772	42.65336391	37.23178547	30.80365609	46.94002671	29.27912853	34.6282197	37.19317375	26.49612328
CEP57	12.79582333	14.68066329	13.23714397	16.4172816	19.49806343	19.80631787	14.55061499	14.41134761	16.95935761	19.34673638	16.73673791	15.84488301
CEP57L1	4.120788585	4.223594419	4.207055436	5.350618063	5.369407701	5.322842739	4.615765712	4.728997142	7.584180381	8.877217401	7.594938499	6.305115196
CEP63	4.946391415	4.739668742	4.566461949	4.975647584	5.482632373	5.328143388	4.789777472	5.023289378	6.741373946	6.382088338	6.084424163	6.877057752
CEP68	3.603055118	3.320240443	2.966073621	2.819087286	3.166032561	3.26211269	2.962817258	2.844889285	3.328228783	3.180867601	3.223629329	3.189594555
CEP70	3.86669732	4.229920176	3.657364501	4.115850379	4.87144911	5.497623872	4.691901817	3.52051174	7.839319939	9.962678343	8.423824382	6.509203377
CEP72	6.779462312	6.887672333	7.42149274	10.49332267	12.499439	11.93530423	7.569245426	10.78456307	10.79259453	11.0447353	12.45613286	10.88531511
CEP76	3.451415012	3.837346043	4.089800005	4.992363838	5.183862202	4.85439306	4.745218905	5.249385603	3.728785135	3.661205659	4.175721156	3.669925511
CEP78	3.639269034	3.751407263	3.90102512	7.215530331	5.088290283	5.518624076	4.183049913	5.889611898	5.971549046	6.345347625	6.160933198	7.107304601
CEP83	3.828050591	3.721855651	3.748822949	3.716796757	4.267630457	4.416877579	3.499873486	3.415691477	1.501157603	1.595159118	1.570215189	1.216140868
CEP85	16.86256969	16.1717484	16.3510785	20.65066815	21.75831659	20.55455782	17.04270732	19.93621538	19.14525829	18.51591071	18.2261657	21.60112596
CEP85L	3.882809947	3.160184234	3.283539298	1.671939593	3.659676539	2.500381997	3.952511991	1.368918498	2.959430251	2.502922284	2.204892394	2.733024163
CEP89	2.490344873	2.947865064	2.779587105	3.523778174	3.082614667	3.096892074	2.560951055	3.535597173	3.429531043	3.028666135	3.306802009	3.468124331
CEP95	8.788029835	8.056709966	8.47729636	9.158917059	10.5463163	9.750539112	9.066842218	8.782542305	6.598002973	7.035623415	6.41430429	6.969852227
CEP97	3.121815277	3.016617778	3.2701788	4.310821529	4.326188984	4.089946796	3.058395933	3.199276652	5.795009562	6.451952597	5.777488666	6.002985694
CEPT1	25.7126154	19.77459117	22.83360356	17.08091435	23.02443786	20.03950695	21.3808341	14.61440726	19.05582417	16.98388939	17.11524252	20.50580025
CER1	0	0	0	0	0	0	0	0	0	0	0	0
CERCAM	1.388451017	2.232539889	1.089670557	1.326933199	1.34130325	1.716886285	1.019868813	1.303153044	5.848630554	11.144235	7.020111554	6.182781704
CERK	12.16136813	14.17271941	12.93516743	18.07995028	11.88542835	14.11350389	11.70527933	16.71856508	17.02896543	14.08432182	16.09402014	21.58407791
CERKL	0.01635549	0	0	0.016530446	0	0	0.0504576	0	0.195787486	0.753247332	0.546143712	0.680837676
CERS1	0.028683135	0.029735673	0.015179313	0	0	0	0.014748179	0	0.156072114	0.330248364	0.463446458	0.343276832
CERS2	84.46961922	82.74544639	84.45850202	98.17430327	89.8343648	84.88339545	86.22914751	93.59261102	126.9917853	131.1984599	128.1313442	138.4199548
CERS3	0	0.012390964	0	0.012080224	0.012480408	0.03682394	0	0	0	0	0	0.049754686
CERS4	0	0	0.05001708	0	0	0	0	0	3.685604587	4.490965261	4.089218554	3.229444693
CERS5	9.177050174	8.894652864	8.649298947	7.670458267	8.076662175	7.464799357	8.134000043	8.423898386	4.866644537	4.680097911	4.973974028	4.502347849
CERS6	8.731231811	8.727988099	8.061186561	9.580884753	8.392576423	9.246546939	7.684449135	7.748354311	17.81176196	17.89312135	17.9078659	20.1944643
CES1	218.3123118	208.9479769	218.898991	169.3423928	127.362326	127.486125	225.1616295	178.0730929	106.7022786	76.94311111	70.97645282	158.2601766
CES2	18.80124661	18.32305336	18.59597753	16.27450017	15.14457903	15.99821895	18.05432797	16.62791528	20.67432986	16.86660226	21.5786328	21.9523319
CES3	0.224403349	0.330590712	0.137506715	0.095496345	0.08632739	0.145549935	0.133601152	0.04622733	2.62201151	3.030514398	2.150028077	1.720773889
CES4A	0	0	0	0	0.015542683	0	0.0153071	0	1.052914719	0.718172191	0.464976323	1.363182046
CES5A	0	0	0	0	0	0	0	0	0	0	0	0
CETN1	0	0	0	0	0	0	0	0	0	0	0	0
CETN2	28.20019578	27.27223727	29.79474207	44.71669418	25.3360842	28.55123693	27.56511141	43.97494569	19.13987476	21.03396678	21.25262467	20.84403457
CETN3	11.85315501	14.82375262	11.74900034	15.28869775	16.34525051	15.76662035	14.43357875	15.02265368	16.74848052	24.50950934	17.06424982	15.19410682
CETP	0.093941272	0.116866177	0.079542991	0.056967705	0	0.096474264	0.096604693	0.018384402	0.204463188	0.082408344	0.3238084	0.254184946
CFAP100	0	0	0	0	0.013791556	0	0	0	0	0	0	0
CFAP126	0	0.356220445	0.14547326	0.069457431	0	0	0.070670712	0.067245119	0.074787046	0	0	0
CFAP157	0.06496124	0.022448339	0.022918624	0.02188538	0	0.022237609	0	0.063564903	0.047129382	0	0.023324629	0.112673841
CFAP161	0	0	0.082860497	0	0	0.026799446	0	0	0	0	0	0
CFAP20	20.74083693	21.63145896	22.39319884	24.79323863	26.44084641	24.67901284	21.20039327	26.89700673	24.1122235	23.83922865	27.45554384	26.74250819
CFAP206	0.048586511	0.10073882	0	0.024553124	0.025366501	0.074844865	0	0.023771074	0.555179864	0.133192719	0.26167812	0.632041748
CFAP221	0	0	0	0	0	0	0	0	0.008793851	0	0.008704265	0
CFAP298	1.312674594	1.214291259	1.538975513	1.602265241	1.708061809	1.845817531	1.754858955	1.877285866	1.637301614	2.037306226	1.740265075	2.028107989
CFAP299	0	0	0	0	0	0	0	0	0	0.020138012	0.019782151	0.019112252
CFAP300	0	0	0	0	0	0	0	0	0	0	0	0
CFAP36	7.13164639	8.684953079	6.866163654	8.098071013	9.26353207	8.515184434	7.488471821	9.038227217	6.405174625	8.126350958	6.548168517	6.7735195
CFAP43	0.009631208	0	0	0.009734235	0	0	0	0.009424186	0.052405814	0.073927084	0.062246326	0.08018457
CFAP44	2.241426816	2.106659117	1.940036862	2.244762154	2.244486405	1.903363361	1.863932498	1.988411184	2.511465243	1.528436683	2.023902358	2.24229389
CFAP45	0.659504367	0.466162577	0.60284281	0.212087008	0.313018377	0.338643479	0.215791741	0.557329046	0.163114922	0.591687319	0.258325142	0.031197158
CFAP46	0.058502784	0.040433043	0.027520068	0.013139688	0.013574969	0.006675581	0.013369212	0	0	0	0.007001897	0
CFAP47	0.004814312	0	0	0	0	0	0	0	0.099544322	0.073907242	0.082972824	0.035071333
CFAP52	0	0	0	0	0	0	0	0	0.026702352	0	0	0.051070587

CFAP53	0.194559363	0.144070562	0.205924313	0.280915127	0.203154747	0.199805377	0.142911074	0.271967605	0.151235168	0.335252127	0.179633379	0.260325459
CFAP54	0.003944115	0.004088846	0.004174506	0.003986306	0	0	0	0	0.004292183	0	0.004248457	0.004104588
CFAP57	0	0	0	0.009409199	0	0.009560633	0	0.009109503	0	0	0.010027976	0
CFAP58	0.217465086	0.297587458	0.24858149	0.228582991	0.290652693	0.232261869	0.313082909	0.161720924	0.132527578	0.162152338	0.103068015	0.036210087
CFAP61	0.020921291	0.016266754	0.011071692	0.010572544	0.021845567	0	0.010757225	0	0.011383798	0.005735262	0.011267827	0
CFAP65	0	0	0	0	0	0	0.0051279	0	0	0.005467925	0	0
CFAP69	0.005244597	0	0.027754767	0.010601397	0.010952592	0.010772019	0	0.010263729	0.176930406	0.270292943	0.101687199	0.158281488
CFAP70	1.198171759	0.922978355	0.730954206	0.622313666	0.278023435	0.33325466	0.51339262	0.431514644	1.475954214	0.821152573	0.842492677	2.424569611
CFAP73	0.055492844	0.115058347	0.078312522	0.09347743	0.212462972	0.132974634	0.152176467	0.325800175	0.12078018	0.101416938	0.179324625	0.077000892
CFAP74	0	0	0.007690087	0	0	0.029846318	0	0	0.063254936	0.05576973	0.062610536	0
CFAP77	0	0	0	0	0	0	0	0	0	0	0	0
CFAP97	13.03446632	9.898674994	12.41690268	11.10551757	14.82784835	13.55698812	13.69767174	8.929750365	5.522409428	6.190729824	5.876272223	5.503927149
CFAP97D1	0.133412134	0	0.047068414	0.02247321	0.023217685	0.0228349	0.045731544	0.087029634	0.096790508	0.195055783	0.263462289	0.231400408
CFAP97D2	0.029641697	0.03072941	0.031373181	0	0.010317077	0.030440943	0.040642797	0.009668184	0	0.04333777	0.042571945	0
CFAP99	0.089533379	0.046409419	0.023690841	0.022622782	0.070116637	0	0.092071827	0	0	0	0	0
CFB	0	0	0	0.041404772	0.064164597	0.021035576	0	0.040085975	0.178327391	0.179685952	0.154446869	0.149216708
CFC1	0	0	0	0	0	0	0	0	0	0	0	0
CFC1B	0	0	0	0	0	0	0	0	0	0	0	0
CFD	75.8033377	119.5582835	78.29142163	257.438964	62.17351429	83.63875519	68.9635673	242.3414256	156.8543008	102.9098127	100.1294758	337.1671691
CFDP1	12.31056283	15.64714824	12.99765554	25.09849546	19.23426665	18.29590225	15.21017262	25.24617643	19.22317151	27.36290547	22.05737683	22.56953933
CFH	0.758915554	0.11420771	0.388667752	0.04948605	0.178938849	0.037711869	0.692318979	0.047909853	0.093245632	0.053689149	0.039555303	0.038215809
CFHR1	0	0	0	0	0	0	0	0	0.04511422	0	0	0
CFHR2	0	0	0	0	0	0	0	0	0	0	0	0
CFHR3	0	0	0	0	0	0	0	0	0	0	0	0
CFHR4	0	0	0	0	0	0	0	0	0	0	0	0
CFHR5	0	0	0	0	0	0	0	0	0	0	0	0
CFI	0	0	0.021211464	0	0	0	0	0	0	0	0	0
CFL1	652.9747505	632.5742308	729.2216038	731.912146	527.6207948	562.2532372	667.7110257	816.3293292	538.2712169	565.645903	622.0762846	539.5449459
CFL2	2.179627782	2.008542216	1.858374861	2.386522166	2.576216083	2.502653679	2.303686314	2.236453496	6.47352884	5.593382322	6.032582378	5.749535324
CFLAR	10.22989921	8.870426621	8.107508269	9.345106855	4.49761815	4.507154066	6.07851836	6.754975714	12.69728231	6.178401684	7.401567545	13.64460162
CFP	2.171949547	2.839918923	1.843199104	1.384349746	0.408631263	0.763599064	2.273772353	1.148791163	3.59867107	1.99542066	3.878164899	4.805723682
CFTR	0	0	0	0	0	0	0	0	0	0	0	0
CGA	0	0	0	0	0	0	0	0	0	0.069910396	0	0
CGAS	17.76730783	16.25231071	17.47774032	20.46234423	17.5232968	14.22910809	16.45929522	16.24585358	18.16540827	14.17807663	18.33416819	16.47026436
CGB1	0	0	0	0	0	0	0	0	0	0	0	0
CGB2	0	0	0	0	0	0	0	0	0	0	0	0
CGB3	0	0	0	0	0	0	0	0	0	0	0	0
CGB5	0	0	0	0	0	0	0	0	0	0	0	0
CGB7	0	0	0	0	0	0	0	0	0	0	0	0
CGB8	0	0	0	0	0	0	0	0	0	0	0	0
CGGBP1	26.57995169	24.62908454	26.03420228	29.89825776	31.26297101	27.23912695	26.48117214	24.6495495	51.634633	43.52862254	43.10931873	47.17475928
CGN	0	0.010635676	0	0	0	0.010535836	0	0	0.97131904	1.0349671	1.248745951	1.334578142
CGNL1	0.506084255	0.341341934	0.464657253	0.246505013	0.413840443	0.463373817	0.501621918	0.16705745	0.524204228	0.561628039	0.683061437	0.260164856
CGREF1	0.199442405	0.186084913	0.105546296	0.040315173	0.728887306	1.085546443	0.799878261	0.780621621	0.173634565	0.262436061	0.236315323	0.062267123
CGRRF1	2.173399928	2.426473114	1.921176822	2.848402084	2.269417965	1.986727538	2.677111983	2.781046813	3.300887105	2.671303233	2.907089442	3.380315243
CH25H	0	0	0	0	0	0	0	0	0	0	0.042062194	0
CH507-42P1	0	0	0	0	0	0	0	0	0	0	0	0
CHAC1	0.663550981	0.816308259	0.561849207	0.625939132	0.369528436	0.417951512	0.336632886	0.640630828	0.779878136	0.514178205	2.010838453	0.81024385
CHAC2	4.917428034	4.638230343	6.655156467	8.188328755	8.501672901	7.781997161	6.217434593	7.572555984	5.000480378	5.259565953	5.817878869	4.907768662
CHAD	0	0	0	0	0	0	0.020336335	0	0.021520859	0.021684812	0.021301618	0
CHADL	0.079765136	0.016538429	0.050654712	0.048371036	0.033315623	0.032766356	0.049215981	0.062440472	0.225691474	0.087465719	0.27494434	0.249031569
CHAF1A	22.59180036	27.73076047	29.1416961	37.95513617	38.22469923	37.4922334	26.80357195	40.53410721	19.39750731	16.84279388	23.27585416	20.29846738
CHAF1B	5.063134338	7.003324918	7.251977778	10.06772234	11.0764566	10.66776942	7.909066439	10.44711644	9.208214803	8.961543943	12.00434159	9.607584025
CHAMP1	19.51663156	19.57288337	20.38430783	27.33631555	23.44770001	23.99302568	20.59922308	23.92110134	24.57000114	19.32159351	25.92447707	30.38852904
CHAT	0	0	0	0	0	0	0	0	0	0	0	0
CHCHD1	27.73360068	30.46976315	34.00971775	34.86063532	34.75060294	31.88607029	35.60070635	40.23831608	19.56567357	18.80589656	19.70972624	19.63942424
CHCHD10	64.40728297	71.02610779	72.67206491	83.42579261	53.38135515	62.5416563	72.44992854	92.67207496	25.01517777	39.11801795	34.96995991	39.76620833
CHCHD2	412.2057457	420.6330667	421.3090841	343.3134142	282.4462672	296.7818417	357.5018455	348.6757583	194.8910936	228.3823558	254.4674795	164.8083534
CHCHD3	33.05578608	33.87936187	38.54003656	41.88037634	38.26683799	38.76911753	40.270096	42.17747662	39.3198768	46.39000112	48.65546154	41.90144481
CHCHD4	15.36457536	12.93746463	17.22075993	17.19032449	20.56212643	18.91395995	19.284513	17.5290921	9.966586146	11.58751741	12.61136819	10.05466513
CHCHD5	4.981125808	4.330274287	4.893825542	5.395622788	3.218670905	4.243746299	4.915618898	5.09262445	3.524677158	4.580248237	3.705314389	3.463609766
CHCHD6	0.308248031	0.382630216	0.300497075	0.319743963	0.262574924	0.354046806	0.379550786	0.444495965	0.22951906	0.382481058	0.253394046	0.189941241
CHCHD7	95.52589365	70.10818646	99.8989397	73.17472167	63.77437025	54.98810539	84.37999478	61.76144377	23.11737994	20.29820271	20.02521156	24.03895559
CHD1	15.19597671	13.37735734	13.59540301	15.34532973	15.88777194	16.19587469	14.72022912	12.41561162	12.72789301	12.00137517	12.09880222	11.29492353
CHD1L	32.44044505	34.67336513	31.82805927	34.292463	36.03426493	36.92967573	31.99794613	31.87560027	52.66560226	56.78839695	50.54593852	50.80654811
CHD2	14.50066511	15.91153902	12.14777345	13.27359082	15.10588159	13.87605088	12.4245525	10.82144305	29.83260922	31.8383192	27.67211771	22.43014074
CHD3	32.91676266	36.98326472	30.70735696	25.53784956	37.93543278	41.53724047	34.41577514	27.7144571	21.15685735	22.27745841	20.29873411	15.49316664
CHD4	67.81975715	65.71492138	68.36002702	72.68089711	82.53902463	74.95180439	72.01274155	69.7178774	67.61011951	53.55781672	64.09333004	58.37013426
CHD5	0.322979875	0.248237321	0.218074398	0.236383844	0.162809724	0.240188271	0.166068495	0.168916566	0.042420346	0.085487037	0.095973017	0.098518191
CHD6	5.161292741	4.760751105	4.283462401	3.989800862	4.898996863	5.153338134	4.260014034	3.375986456	7.392319539	5.821020217	5.572416414	5.574213714
CHD7	11.16224971	9.64623092	11.00013921	10.04924127	14.25662434	12.6705876	10.78208802	8.163940885	11.69548035	10.42353104	11.42097897	10.7977084
CHD8	17.44012607	16.8623362	16.52262485	17.57478489	17.98272465	17.57418377	17.98079414	16.06030511	26.90061409	25.31071575	24.20659961	26.68013861
CHD9	8.594779621	6.918928265	7.07674442	7.015723609	7.44700396	6.600127064	7.450808055	4.71453662	11.28477896	8.731349921	9.506784439	8.514329148
CHDH	0.038194412	0.013198656	0.020212746	0.00643383	0	0	0	0.006228904	0.090057653	0.181487488	0.246849797	0.092746313
CHEK1	4.943321528	6.061494949	6.282246209	9.034348883	11.17445715	9.316218237	7.552330288	9.232033037	11.1351312	10.84110665	13.14014915	12.00371523
CHEK2	15.16019535	15.98160174	16.39374017	19.56832271	21.22739541	21.34636858	17.46833202	19.24888113	13.79478289	16.48357097	14.10676902	13.24889087
CHERP	30.66947864	27.58555469	31.44572369	32.64438203	34.76858308	33.60159752	32.5795091	38.3164167	21.75020977	19.86984914	22.85913343	22.93288238
CHFR	0.239224485	0.380271132	0.168798996	0.6125182	0.299751741	0.409458051	0.114803253	0.358926305	28.34191873	23.6612088	27.89857935	30.22347208
CHGA	0	0	0	0	0	0	0	0	0	0.023966033	0	0
CHGB	0.284062538	0.273451577	0.343606524	0.123043368	0.14830603	0.041674554	0.10432724	0.039708091	0.066242372	0	0.065567538	0.147810065
CHI3L1	383.6384424	673.8430378	413.7369986	485.3809685	27.37462426	99.6011858	139.4072711	353.5800347	311.6944135	297.527229	326.7247912	413.1785927
CHI3L2	0.351505025	0.182201813	0.403040913	0.029605427	0.152930862	0.120327618	0.391593458	0.057324908	0.223139793	0.160599821	0.15776185	0.030483886
CHIA	0	0	0	0	0	0	0	0	0	0	0	0
CHIC1	0.004340181	0	0.009187414	0	0	0	0	0.004246889	1.029658013	1.284979922	0.939692082	1.097574804
CHIC2	0.87666041	1.152936944	1.062065465	1.127684929	1.017520455	0.948661434	0.83446059	0.925165901	1.273348393	1.064573335	1.158921906	1.451432345
CHID1	7.755180876	7.015202858	8.134353596	8.284689685	6.883732676	7.152336794	7.723966821	9.715989799	10.42610281	12.91517528	12.09831585	12.14842144
CHIT1	0.139946842	0.193442988	0.148121668	0.141443872	0	0	0.071957305	0.022823116	0	0.025576235	0.025124276	0.024273472
CHKA	9.50972647	10.24902196	10.4068057	14.38456179	11.98546864	11.41224584	9.635532032	13.4106019	14.30591566	11.65460253	13.47650167	16.22199209
CHKB	33.42865291	31.57333346	29.22806947	27.97714628	25.55826752	26.32419635	27.1750364	29.57484924	45.9047847	43.64657525	36.96882143	46.02978855
CHL1	0	0	0	0	0	0	0	0	0	0	0	0
CHM	3.073212011	2.886196391	2.892649166	2.710662249	2.687966973	2.521367604	2.64139421	2.291428809	3.81338281	3.22068134	3.456936172	3.841441849
CHML	3.045680536	2.620677572	4.110077609	2.839695375	3.768578258	3.737724522	4.220412549	2.190457185	4.234225182	6.120023546	4.91284286	2.32173162
CHMP1A	13.98082305	15.35472037	16.87489816	17.85371696	15.37096764	15.44315845	16.59744514	20.68296848	18.97721294	16.87021814	19.56451445	21.85591142
CHMP1B	21.45348505	20.92816069	19.89624942	17.96843315	19.20633705	20.38858095	18.69822339	18.46293859	23.0597058	21.2107228	18.96067496	23.75378127
CHMP2A	26.29720288	28.1666208	26.74505248	32.30989171	28.23980383	29.37411752	26.75440948	33.87226599	28.44839472	27.84514481	29.26612947	31.58247103
CHMP2B	12.3724853	13.83964952	12.57802072	16.15948707	14.26609962	13.81009644	15.51716295	15.20489576	10.89060211	12.94538398	9.790117036	13.36406703
CHMP3	0.067430527	0.034952457	0.01784235	0.01703796	0.052807142	0.017312174	0	0	0.036690637	0.092425398	0.108950571	0
CHMP4A	39.96034498	38.38243772	46.57863899	50.25414178	40.52410698	37.73686182	41.78702943	48.14869552	33.89987595	27.3700232	32.78512294	34.73088047
CHMP4B	45.11900641	42.74411754	50.24431769	45.66769167	39.74354609	39.18895908	49.85876693	49.71178114	56.38943273	48.86579636	53.31632122	58.20893431
CHMP4C	0	0	0	0	0	0	0	0	0	0	0	0
CHMP5	22.38165792	21.45128544	19.17685438	18.39111982	19.29894388	18.34006523	21.7865516	19.4841271	45.2341319	52.16328747	40.23718269	42.01270043
CHMP6	7.840177107	7.45542507	8.148904243	7.63900691	7.95096207	6.864113564	7.685439983	8.9410633	10.09730952	8.225976717	9.083097167	11.00607288
CHMP7	12.4086524	11.34096443	11.89481195	14.95738942	12.80951541	12.73471003	13.3067294	15.82917743	13.40435809	13.75221497	12.8207748	14.36467203
CHN1	0.048769995	0.101119254	0.034412557	0.065722258	0.016974864	0	0.033435147	0.031814458	0.566122123	0.10695657	0.367732863	0.372198119
CHN2	1.193316762	1.060376521	0.912772865	0.902027582	0.680607885	0.751772918	1.082779096	0.971420034	2.291694742	1.352504295	1.63105055	2.160225878
CHODL	0.0625329	0	0	0.063201823	0	0	0.016076458	0.01529719	0.034025716	0.119997275	0.050518626	0.032538582
CHORDC1	17.40897557	13.93836356	17.51791157	19.59839363	19.67619061	15.63029463	20.4730995	16.90451355	16.03189226	16.02427759	16.72891392	14.22291988
CHP1	25.16719656	27.40676696	23.6981346	28.32326976	19.99925271	22.70062483	26.23973753	25.61997397	35.41816138	39.37685024	34.7325809	35.95092975
CHP2	0	0	0	0	0	0	0	0	0	0	0	0
CHPF	5.732857728	6.508432989	6.67975513	5.176359308	3.018940669	2.969168022	4.70612266	4.898322776	2.64294252	2.699309726	2.491445659	4.865731769
CHPF2	15.1625217	14.009467	14.848355	11.54887349	10.20966726	9.009425972	13.00912972	11.43313526	13.92107955	9.43617957	12.19735157	19.3991876
CHPT1	18.67795063	22.04831084	21.92972327	25.99155283	24.05278582	24.37593274	23.53154648	23.34498464	11.97028924	15.66990463	16.50891106	12.08130264
CHRAC1	17.90070455	20.82993362	21.76669797	27.3229002	27.98120523	26.94609421	21.19266214	28.0507214	14.47586307	15.62296334	16.41168047	14.17863493
CHRD	0	0.011736613	0.011982491	0	0	0	0	0	0.012320273	0.012414133	0.012194762	0.035345402
CHRDL1	0.025876201	0.026825738	0	0	0.121587206	0.013286958	0.066524607	0	0.281597774	0.226994467	0.278729039	0.228896677
CHRDL2	0	0	0	0	0	0	0	0	0	0	0	0
CHRFAM7A	1.644896199	1.631114669	1.438201548	1.821512824	4.495541305	2.849688719	2.235764359	1.469579339	1.089600743	0.799899812	1.032279145	0.952666009
CHRM1	0	0	0	0	0	0	0	0	0	0	0	0
CHRM2	0	0	0	0	0	0	0	0	0	0	0	0
CHRM3	0	0.002714028	0	0.002645965	0.002733619	0	0	0	0.005697991	0.025836303	0.016919832	0.024520292
CHRM4	0.088843524	0.030701223	0.062688807	0.059862596	0	0.030413021	0.030454138	0.086933839	0.225595927	0.227314596	0.382796062	0.369833124
CHRM5	0	0.030811445	0.031456935	0	0	0	0.030563473	0	0.145546621	0.048885149	0.080035492	0.046395111
CHRNA1	0	0	0	0	0	0	0	0	0	0	0	0
CHRNA10	0.343133432	0.307216902	0.462225471	0.425623059	0.390864686	0.272297912	0.400979477	0.793609354	0.39038838	0.256540758	0.336009876	0.616799465
CHRNA2	0	0	0	0	0	0	0	0	0	0	0	0
CHRNA3	0	0	0	0	0	0	0	0	0	0	0	0
CHRNA4	0	0	0	0	0	0	0	0	0	0	0	0
CHRNA5	1.143620256	0.954627531	1.430500441	1.45607545	2.031595534	2.364165354	1.802249464	1.409697501	1.794084056	1.628605419	2.19176191	2.009344976
CHRNA6	0	0	0.023730326	0	0.023411166	0	0.02305632	0.04387744	0	0.024585156	0	0
CHRNA7	0.170676231	0.160853869	0.238124374	0.172501977	0.445541223	0.215114242	0.199449135	0.151825053	0.101311863	0.051041846	0.133706352	0.064589272
CHRNA9	0.023642162	0.049019439	0.125115952	0	0	0	0	0	0	0	0	0
CHRNB1	2.936672792	2.243267922	2.453853934	2.477125037	2.051959408	1.904751136	2.679339274	2.635887261	1.802162199	1.719044144	1.641098708	1.516588971
CHRNB2	0.020430778	0.010590246	0.032436325	0.020649328	0.005333346	0.02622708	0.010505015	0.034985334	0.055584487	0.044806359	0.027509114	0.037208571
CHRNB3	0	0	0	0	0.020725488	0	0	0	0	0	0	0
CHRNB4	0	0	0	0	0	0	0	0	0.007294252	0.022049467	0.007219943	0
CHRND	0	0	0	0	0	0	0	0	0	0	0.041351988	0.013317217
CHRNE	0.127309687	0.175975147	0.134746328	0.042890512	0.066467033	0.021790402	0.065459583	0.062286587	1.062176587	0.372267346	0.594244596	0.971589727
CHRNG	0	0	0	0	0	0	0	0	0	0	0	0

CHST1	0	0	0	0	0	0	0	0	0	0	0.014688724	0
CHST10	6.58200177	5.965313392	6.736659377	8.682424142	7.7371985	8.334364243	7.801351537	9.640864051	8.551130098	7.857329043	8.683291797	11.19977977
CHST11	45.43316071	38.39783624	43.43904831	32.96901069	28.38117385	29.41656653	32.66215199	28.61206987	33.81727054	31.91487654	30.95184175	43.2475123
CHST12	5.665315554	5.238265086	5.209095851	4.907929849	3.974188196	4.3130114	4.521288107	6.592317855	4.308554686	5.46869811	5.701923693	7.716921425
CHST13	14.23661027	13.28937877	14.42974055	7.895604634	12.69242144	12.08048141	13.36852991	10.21183475	5.941168458	5.027278532	6.010602678	7.470705114
CHST14	6.365098191	6.903221611	6.607351789	7.348678378	5.879419329	6.360735222	7.40151927	8.024900359	13.72042875	13.44913173	14.58272162	17.73213539
CHST15	0.005177481	0.005367471	0.005479917	0.010465731	0	0.010634169	0.015972819	0	0.02817197	0.028386594	0.005576994	0.016164408
CHST2	0.546289958	0.458462687	0.454300649	0.723032215	0.095070725	0.120218547	0.307640527	0.330910354	2.590321741	2.709893915	1.961478973	5.184331219
CHST3	0.021965327	0	0	0	0	0	0	0	3.304696005	2.553102839	2.998937009	3.66315891
CHST4	1.81930895	1.499183158	1.999642517	0.636497422	1.680489426	2.395338473	1.463131877	0.890101519	0	0.025576235	0.025124276	0
CHST5	0.049670364	0.03432869	0.017523933	0.016733898	0.017288246	0.051009654	0	0.032401802	0	0.054465576	0.035668741	0.034460861
CHST6	0.019814494	0.03423599	0.062915801	0	0	0	0	0	0.32344686	0.289698659	0.355724215	0.20620682
CHST7	2.109752386	2.119526047	2.163929443	1.626718867	1.567052993	1.652899731	1.476200922	1.596188207	1.183473263	1.907983002	1.124560136	1.969241794
CHST8	0	0	0	0	0	0	0	0	0	0.01472165	0	0
CHST9	0	0	0	0	0	0	0	0	0.006061931	0	0	0
CHSY1	11.99519275	12.84712749	12.63281844	15.89704873	13.95597545	14.35813354	12.51908393	14.0887149	33.73664487	38.10948703	32.60145281	35.04193873
CHSY3	0.002731034	0.0056625	0.011562256	0.002760248	0.014258437	0.025242051	0.011233856	0.018706314	0.166434675	0.21262298	0.158855606	0.272846485
CHTF18	11.31900697	15.22295601	13.13901687	22.31061225	20.91453546	23.95942683	12.98108068	26.23193467	14.66584715	17.84961773	16.0946921	20.86134921
CHTF8	45.44435949	42.65516895	45.9186539	47.99436816	46.66646557	42.23635582	45.97755627	51.93429648	54.52311751	42.19087753	52.36619964	57.21059272
CHTOP	75.59671916	71.54584029	82.85236098	90.59173052	80.44102611	79.4975018	75.05758228	95.3635447	62.69464364	52.43925236	60.69120217	54.91304761
CHUK	11.78344255	9.250905555	12.59031094	13.96987886	13.16171469	11.74073856	13.26170343	11.96126057	7.915035023	7.033688255	7.005917831	9.923757121
CHURC1	10.56840614	8.595229816	9.358216347	7.94339392	7.75579876	7.429206878	11.14356908	6.408655176	6.835846329	4.194778481	5.611783204	6.908854462
CHURC1-FNTI	0	0	0	0	0	0	0	0	0	0	0	0
CIAO1	28.19260652	25.88614749	27.71764693	27.35288013	27.67607223	26.67252032	29.59211524	27.10241512	22.10605271	21.09187256	21.82618268	27.37225119
CIAPIN1	29.07491854	25.71898553	30.90380067	29.63518124	28.91740369	30.92255725	33.0945894	31.6834532	23.42867022	21.06526472	23.61501125	25.6640233
CIART	0.19702443	0.499288315	0.208533373	0.331886708	0.182869971	0.157373153	0.247635007	0.235631494	3.930883814	7.249520363	4.315293641	2.77944087
CIB1	28.50347028	28.29629361	29.13671275	35.2715405	24.99905388	27.38996642	30.23385259	40.67247942	32.80111066	29.50218717	29.02285306	41.06585915
CIB2	0.020297835	0.105213357	0.064450526	0.041029927	0.021194568	0.104225686	0.104366594	0.099307673	0.08835648	0.178059221	0.109320452	0.274607954
CIB3	4.843617293	2.353760409	5.767370259	0.994384236	1.975625831	2.487109246	3.657880296	0.814602267	0.494162507	0.746890815	1.63042767	0.315043031
CIB4	0	0	0	0	0	0	0	0	0	0	0	0
CIC	8.728433342	9.559066596	7.929034882	9.300208283	8.632970468	8.555454528	7.457202427	9.745052885	8.942393936	7.764826181	8.620154849	9.970247295
CIDEA	0	0.035195029	0.035932354	0.034312409	0	0	0	0	0	0	0	0
CIDEB	8.461553445	11.58915128	9.568067259	14.27610676	16.12561121	15.98408764	9.711321963	16.26975485	2.415528104	3.171485121	2.69521922	2.981941363
CIDEC	0	0	0	0	0	0	0	0	0.207506226	0.209087081	0.287549203	0.634998144
CIITA	0.015255026	0.084345676	0.032292259	0.041115229	0.084954524	0.093998134	0.057520965	0.019902827	0.608713691	1.159791142	0.827085357	0.550357728
CILP	0	0	0	0	0.017379152	0.008546313	0.008557867	0	0.081507008	0.036501314	0	0.060623615
CILP2	0.4741495	0.305557228	0.244141477	0.492173471	0.107047961	0.184245397	0.210850842	0.413800104	0.209186439	0.126468056	0.220859076	0.080017479
CINP	17.58841622	18.46175223	21.46637007	23.38728905	20.66113126	20.99784534	22.4958088	23.01884916	17.34616399	18.26182373	16.57740248	16.53082918
CIP2A	5.553797378	5.12518265	6.275028927	9.415287809	8.97077843	8.176825057	6.136008814	7.274908614	7.890289479	7.922528771	8.29588862	6.851069815
CIPC	5.841332274	5.388530615	6.51001203	5.34095377	6.577525415	7.21893755	6.376016354	5.812650781	6.437637276	5.957433381	5.19895686	6.285065003
CIR1	13.35485476	14.68494447	13.24557156	14.89298259	13.53747742	11.92738286	15.33830365	15.09403523	26.48675208	24.68113818	24.66524255	25.14172536
CIRBP	74.87205236	77.75671496	74.8030557	83.98552916	69.57212873	76.28894649	68.59538731	86.04665131	75.26567264	64.51918938	63.54199417	104.4814311
CISD1	15.13712979	15.11040488	18.26379514	16.97601563	20.97676021	17.11815913	20.34381172	16.90988258	6.722532357	8.313234906	7.396441311	5.923982183
CISD2	4.557191254	4.60133794	5.065047081	5.538204542	6.141259049	5.017709663	5.569204778	5.379678013	5.148197074	5.097288958	5.872901745	4.333926998
CISD3	9.022695713	9.523086026	9.960255	12.72976063	9.46393725	9.28694353	9.278506905	13.0633593	5.553704198	6.289920027	7.300184224	8.200142243
CISH	7.053727008	7.29083476	7.58778777	6.705456121	6.566435368	7.082466252	6.089670793	6.902107667	2.281194276	2.965159443	2.607937971	2.912291778
CIT	5.962489062	6.996688217	6.72970907	15.85293815	13.22541016	13.27835236	7.149111655	14.11658574	17.84248666	19.85360314	18.25104946	15.43085462
CITED1	0.199664419	0.482979409	0.211327574	0.470867261	0.416970676	0.717668293	0.307987947	0.683804264	0.325927219	0.291920219	0.143380838	0.173156768
CITED2	264.574265	314.8627634	246.2393988	251.6309525	137.0565083	193.5290693	202.520964	240.6564641	138.5537599	150.0333241	122.4122707	170.3244731
CITED4	10.42013185	15.33955322	11.02881125	17.65095712	10.61934846	12.32766233	9.215384271	18.14915035	118.2958439	130.2118222	133.7923136	218.4000431
CIZ1	19.47315925	21.65388196	19.5599772	22.47150636	21.14282112	20.93389623	20.48673767	26.57263024	30.61840633	33.17784507	33.42648667	35.35133922
CKAP2	37.66047184	36.10167783	39.29412703	71.82614637	58.74605859	53.39501881	47.8121439	62.50176509	42.13486612	43.46541888	39.69539104	38.8302191
CKAP2L	4.975503123	4.669052974	5.361240736	14.63211904	8.4086693	8.154695096	6.121402416	11.51748463	14.15371774	17.58677657	15.35241118	13.73381468
CKAP4	10.38616689	13.81741091	13.6003842	7.201198214	7.883918385	8.864243518	16.40370589	9.573259695	1.041550027	1.788011344	1.030939393	1.162032486
CKAP5	31.2967434	29.49562586	31.6913302	53.23493119	51.27442884	48.01750302	38.68681531	48.34752802	64.40187223	77.75852014	65.61914053	57.09986021
CKB	2.650559971	2.350547561	2.467390525	0.742349482	1.600573492	1.738162792	1.31359449	1.124929032	3.927052254	6.373172778	5.194194215	4.386857357
CKLF	0.605963523	1.19357918	1.02617624	1.837336777	1.265468438	0.995683938	0.872401295	1.007995249	1.318879664	1.129588248	1.501260343	1.26123646
CKLF-CMTM1	0	0	0	0	0	0	0	0	0	0	0	0
CKM	0.253221463	0.098442568	0.368517997	0.127965103	0.16525529	0.097518456	0.227850691	0.030972311	1.584517544	1.006545209	1.807041349	1.350561678
CKMT1A	0	0	0	0	0	0	0	0	0	0	0	0
CKMT1B	0	0	0	0	0	0	0	0	0	0.02616602	0	0
CKMT2	0	0	0	0	0	0	0	0	0	0	0	0
CKS1B	49.12760152	57.97173431	63.09474791	107.0374764	98.74003583	93.79379303	68.89770903	118.9852939	120.0587795	167.6129163	147.3193301	106.7135879
CKS2	78.44315853	83.58562391	88.48021381	153.0024305	117.9375497	131.7818403	99.35177869	155.7364211	119.1128183	147.7982948	123.262714	104.907028
CLASP1	6.90877487	6.425396216	6.174663201	5.961998749	6.358634883	5.804239869	5.80317266	5.165627604	9.900425419	9.714453578	8.833148878	9.733840007
CLASP2	12.6794941	10.95512841	13.06049418	15.13841741	15.02071548	13.49351465	12.91565025	11.9114537	14.81358583	14.78762428	14.68420576	14.12453652
CLASRP	24.15844471	23.62139063	22.14556678	24.15811771	23.59748333	23.63866502	25.15452484	25.2788713	19.62917354	19.98288284	20.80805105	19.01346695
CLBA1	1.136212899	1.075479968	0.862722875	0.699006069	0.70926645	1.078067243	0.825518915	0.77341919	1.330564023	1.083394526	1.463343448	2.210651962
CLC	0.085007205	0	0	0.085916538	0	0	0.087417327	0	0	0	0	0
CLCA1	0	0	0	0	0	0	0	0	0	0	0	0
CLCA2	0.024102827	0	0.025510764	0.012180329	0.025167659	0	0.024786189	0.011792369	0	0	0.012981345	0
CLCA4	0	0	0	0	0	0	0	0	0	0	0	0
CLCC1	6.052881459	6.344076585	6.241883771	8.418756749	6.760970816	7.276815803	6.110281696	7.694174046	9.669062054	10.55867751	8.924547148	12.35558779
CLCF1	0.654551171	0.735117671	0.663919904	0.909635359	0.825857659	1.092325334	0.420693116	0.640481642	0.237438447	0.358871005	0.440661711	0.312208762
CLCN1	0	0	0	0	0	0	0	0	0	0	0	0
CLCN2	0.3658855	0.51839279	0.593795997	0.505392546	0.42025484	0.46342633	0.263381352	0.560899282	1.234342227	1.404229217	1.326865836	2.132324344
CLCN3	8.804280125	8.564865149	8.231277212	9.739317636	8.792878947	8.269009194	8.309946561	8.16906148	10.53387308	9.257610226	9.943416761	10.79623836
CLCN4	1.616902403	1.610500494	1.283513852	0.961293303	0.720025259	0.936066097	1.20630503	0.876385462	2.242619949	2.268396186	2.868630495	2.119858153
CLCN5	3.441544525	3.33697342	3.546157	3.918269295	3.498463195	3.4095994	3.325731027	3.090239586	3.205502785	3.524057347	3.287331387	4.171475534
CLCN6	6.308316366	7.577555316	5.637305836	5.249533649	5.748842331	6.313541335	4.865377169	4.592577286	13.14426304	19.95462081	15.70601447	7.901564525
CLCN7	34.20489304	32.56011139	30.88117468	29.75139515	27.71750606	30.46052476	28.41404431	35.56007157	26.78086554	20.5654641	21.71497913	39.89568402
CLCNKA	0.424365622	0.395944081	0.359323541	0.107226279	0.420957876	0.087161607	0.436397223	0.083048783	0.554179089	0.418800764	0.50282235	1.015752897
CLCNKB	0	0	0	0	0	0	0	0	0	0	0	0
CLDN1	0	0	0	0	0	0	0	0	0	0	0.01679076	0.016222161
CLDN10	0	0	0	0	0	0	0	0	0	0.03565219	0	0
CLDN11	0.018821113	0	0.03984105	0	0	0	0	0.018416554	0.081928306	0.020638116	0.020273419	0.078347532
CLDN12	6.138754487	5.724571441	4.958498154	4.081856268	4.569777836	4.690502897	3.624574315	3.305178361	10.40431915	8.438398515	8.811317942	10.5609277
CLDN14	0	0	0	0	0	0	0	0	0	0	0	0
CLDN15	6.906039897	6.040793927	6.091206557	5.889303017	6.3598248	5.368440271	5.622289784	6.616811848	8.846367711	5.94250829	7.438935229	8.30999702
CLDN16	0	0	0	0	0	0	0	0	0	0	0	0
CLDN17	0	0	0	0	0	0	0	0	0	0	0	0
CLDN18	0	0	0	0	0	0	0	0	0	0	0	0
CLDN19	0	0.015486985	0.015811433	0	0	0.030683209	0	0	0.113800055	0.04914301	0.080457667	0.046639838
CLDN2	0	0	0	0	0	0	0	0	0	0.017162413	0	0
CLDN20	0	0	0	0	0	0.045707575	0	0	0	0	0	0
CLDN22	0	0	0	0	0	0	0	0	0	0	0	0
CLDN23	0.16539824	0	0.02917663	0	0.028784221	0	0	0.026973836	1.409958034	1.209106032	1.544061778	1.491773944
CLDN24	0	0	0	0	0	0	0	0	0	0	0	0
CLDN25	0	0	0	0	0	0	0	0	0	0	0	0
CLDN3	0	0	0	0	0	0	0	0	0	0	0	0
CLDN34	0	0	0	0	0	0	0	0	0	0	0	0
CLDN4	0.029388073	0	0	0.059404881	0.0613728	0	0.030221282	0.028756378	0.031981571	0.128900872	0	0.122335115
CLDN5	0	0	0	0	0	0	0	0	0	0	0.016977652	0
CLDN6	0	0.081436674	0	0	0.041012262	0.080672202	0	0	0.085486507	0.172275546	0.084615626	0
CLDN7	0.552837954	0.436666315	0.278633959	0.106428901	0.137443245	0.297389943	0.433151999	0.437915744	5.156793675	3.839325728	4.735618645	5.725914803
CLDN8	0	0	0	0	0	0	0	0	0	0	0	0
CLDN9	1.12868835	1.496647167	0.694546113	1.830525187	1.480042501	1.671291918	0.566848061	1.79790487	0.199955024	0.17269573	0.084822005	0.40974804
CLDND1	31.32893394	27.58483122	30.51521957	22.74249969	25.66202723	21.63027391	25.53428478	19.44968517	26.0310112	33.63388609	25.99384035	25.51011993
CLDND2	0.706371538	0.68504744	0.687340363	0.264844141	0.32120338	0.327608054	0.398347599	0.289853088	0.07439118	0.137422849	0.196355549	0.082996462
CLEC10A	0.5661028	0.30888218	0.504565061	0.090340812	0	0.091794782	0.398315162	0.029154445	0.129697136	0.065342607	0.1925638	0.062014284
CLEC11A	162.7289847	166.0836431	187.818696	241.6216694	168.1610583	195.0022578	154.836216	260.6450929	446.7824375	346.0164128	385.257891	490.6968186
CLEC12A	0.912377911	1.719741709	1.009567607	0.461068867	0.259823346	0.319424616	0.639712923	0.365222624	0.699539872	1.182361259	0.692413416	1.57530621
CLEC12B	0.227825608	0.295232187	0.165779464	0.158305598	0.014868166	0.073115187	0.043928421	0.013933032	0.077478509	0.093682521	0.030675683	0.222276656
CLEC14A	0	0	0	0	0	0	0	0	0	0	0	0
CLEC16A	4.040703125	4.048380663	3.968540728	3.829941451	3.427852874	3.686761506	3.572789564	3.860173915	4.39729536	3.669730918	3.79823173	6.151991703
CLEC17A	0.023079374	0.01196314	0	0.023326257	0	0	0	0.011291642	0	0.012653737	0.062150658	0
CLEC18A	0.028903972	0.019976409	0.010197454	0.00973772	0	0	0	0.037710244	0.031454749	0.010564794	0.041512411	0.060159963
CLEC18B	0.045853908	0	0.024266204	0.115861032	0	0.023545146	0.023576978	0.112170704	0.199602069	0.100561354	0.123480408	0.190878224
CLEC18C	0.020145848	0	0	0	0	0	0	0	0.02192372	0	0.043400752	0
CLEC19A	0	0	0.022835919	0	0	0	0	0	0	0.02365853	0	0.022453448
CLEC1A	0.035447669	0.1102453	0.018759151	0	0	0	0.03645268	0.017342862	0	0	0	0
CLEC1B	0	0.015245729	0	0.014863397	0	0	0	0	0	0	0	0
CLEC2A	0.024162353	0	0	0	0	0	0	0	0	0	0	0
CLEC2B	0.158418727	0.164231958	0.055890855	0.080056676	0.027569577	0.08134513	0.298668718	0.025835588	0.086199593	0.086856292	0	0.054954758
CLEC2D	3.900842187	3.960818182	3.523727803	2.989866705	3.444922987	4.119303658	3.908316926	3.296940722	1.571447823	1.75935518	1.090967587	1.492329954
CLEC2L	0	0	0	0	0	0	0	0	0.040778731	0	0.12108991	0
CLEC3A	0	0	0	0	0	0	0	0	0.029293775	0	0.02899535	0
CLEC3B	0.174706366	0.362234556	0.061637209	0.206004427	0.121616447	0.149514228	0.179659636	0.142459222	0.728809476	0.510860384	0.690020282	0.696955992
CLEC4A	0.224473734	0.33244411	0.254556531	0.3889285	0.301359468	0.411654191	0.280303294	0.07844596	0.157039426	0.298889858	0.051813204	0.100117221
CLEC4C	0	0	0	0.032449385	0	0	0	0	0	0	0	0
CLEC4D	0	0	0	0	0	0.026491111	0.079580778	0	0	0	0	0
CLEC4E	0	0.021962253	0	0.021411484	0	0.130536516	0.174283993	0.041458999	0	0.023230069	0	0
CLEC4F	0	0	0	0	0	0	0	0	0.014942143	0.045167931	0	0.028578157
CLEC4G	0.196504787	0.169763	0.173319481	0.033101137	0.068395373	0.033633876	0.067358695	0.064093644	0.39205163	0.825989416	0.52916954	0.477166515
CLEC4M	0.052548399	0.081715022	0.027808975	0	0	0.053965292	0	0.051418875	0	0.02881073	0	0.082029637
CLEC5A	0	0.031534269	0	0.030743453	0	0	0	0	0.049653695	0.250159869	0.245739275	0.58563009
CLEC6A	0	0.03458408	0	0	0	0	0	0	0	0	0	0
CLEC7A	0.190881734	0.395772414	0.20203186	0.077169448	0	0.019602859	0.078517443	0.018677875	0	0.020930959	0	0.099324049

CLEC9A	0	0	0	0	0	0	0	0	0	0	0	0
CLECL1	0.025227173	0.07845868	0.053401576	0	0.105366711	0.12953694	0.129712067	0	0.027453473	0.027662623	0.027173794	0.052507172
CLGN	0.019067881	0	0.020181708	0.115631117	0	0	0	0.018658019	5.768673091	7.61076976	6.079611723	9.445597394
CLHC1	2.439205299	2.546646932	2.188026801	1.258875467	1.607659565	1.510091297	2.143670705	1.252633562	1.863773493	1.716732319	1.602541879	1.72830551
CLIC1	503.3844874	429.0549023	522.5890483	375.0119383	454.2352458	396.7201565	527.9282266	398.0068387	478.6348556	458.8658094	507.0150333	428.908278
CLIC2	0.878849546	0.600497227	0.676499258	0.908438228	0.54226309	0.656397429	0.821606056	0.254078719	1.304192808	1.314128605	1.07575545	1.12247236
CLIC3	0	0.042583299	0.043475406	0	0	0	0.042240586	0	0	0.045041507	0	0.085494502
CLIC4	36.26138201	33.12575382	36.96664515	34.65000029	39.29527798	31.41894814	38.15056568	28.63017821	63.10134241	47.75974279	67.88342966	52.58895236
CLIC5	0.006725354	0	0	0	0	0	0.006916031	0	0.153696213	0.10324475	0.094175996	0.1679757
CLIC6	0.009942639	0.010307487	0	0	0	0	0	0	0.108200771	0.141732607	0.267746229	0.031041518
CLINT1	39.3913748	34.56470524	36.20361761	33.84348409	37.20717427	35.20853749	40.30619554	30.80497437	54.61066952	46.26724121	47.72072122	59.46050027
CLIP1	16.61256887	13.97593003	15.85607296	16.25725101	15.24701488	12.55842581	16.2446427	13.6671843	5.954469018	5.539001757	5.334607029	6.84335701
CLIP2	3.279065456	4.963713867	3.255614678	4.878338869	4.080436212	4.966785778	2.695637335	5.01636743	6.073721791	6.809420876	6.355678453	6.76456229
CLIP3	0.919568019	1.122447873	0.675019183	0.779500763	0.588505609	0.502644744	0.625342305	0.740159687	0.758610276	0.764389635	0.543191269	0.756324709
CLIP4	0.00768269	0	0.03252586	0	0	0	0	0	0.016721376	0.025273147	0.008275515	0.007995274
CLK1	13.66592022	14.42371401	13.70292482	17.44687113	18.0483075	18.02774358	17.03509554	15.76948605	48.06473481	38.42431897	35.85684341	33.63676547
CLK2	44.0798835	48.05268308	42.02848662	49.99242437	54.63966329	53.71347005	43.24726521	53.66780808	69.79155282	75.92852945	68.94756121	74.8365275
CLK3	6.349790029	7.003553965	5.861563623	6.02735871	6.23386379	6.850414511	6.186498696	6.488736787	7.765024355	9.252632425	6.973737825	7.997896257
CLK4	7.555231521	7.543205926	7.24688362	7.701130775	7.956247911	8.287966662	8.409532893	6.531735424	15.67315137	13.36836244	12.48477063	12.28535874
CLLU1OS	0	0	0	0	0	0	0	0	0	0	0	0
CLMN	0.043116635	0.081270574	0.033189266	0.079232472	0.057300057	0.076482278	0.024184951	0.122734099	0.345514192	0.425512315	0.236440516	0.269225475
CLMP	0	0	0	0.018404456	0.019014145	0	0	0	0	0	0.01961479	0
CLN3	16.89716504	18.56301621	16.03996099	15.91930585	15.41725536	15.96360925	16.90469338	17.00507868	10.10485476	9.452767258	10.05130524	11.3572543
CLN5	9.04712279	8.947435629	10.25670912	12.39591602	9.446813136	7.697200701	10.78286423	11.11211994	14.83008972	10.10732688	11.43739562	17.51073551
CLN6	28.17681733	29.68352238	30.8896441	35.73120131	35.51146015	33.81683727	28.1612072	38.32719849	34.45064276	28.92319688	39.01169082	31.62114448
CLN8	3.714551249	3.661277316	3.199662416	3.396185125	3.240169037	3.26304322	3.155797064	3.304787664	2.108246511	2.412562035	2.314528523	2.688137455
CLNK	0	0	0	0	0	0	0	0	0	0	0	0
CLNS1A	105.6379676	112.1183827	113.1566766	117.6378729	147.651962	141.1485086	118.3830613	123.4107097	83.19815484	98.0690719	93.9353054	60.45377508
CLOCK	2.833480249	2.047039516	2.48354238	1.910685124	2.112668008	1.859595817	2.249100949	1.568237622	3.411160606	3.912911447	3.619625891	3.418724941
CLP1	13.14632283	11.09034272	13.8216934	13.02180093	11.13996116	10.35759408	11.630577	12.20772119	11.73702877	9.90864358	11.71165518	12.71047596
CLPB	12.57564816	10.87494075	13.63727235	10.69590597	13.64743946	13.83494685	13.40880898	11.88205618	9.431863971	10.7065072	10.76693146	7.781644283
CLPP	41.70124091	45.46090891	50.12438528	41.86382222	49.88958141	47.81878083	50.67182185	54.52848876	26.9133953	32.96247846	36.81147296	26.95342307
CLPS	0	0	0	0	0	0	0	0	0	0	0	0
CLPSL1	0	0	0	0	0	0	0	0	0	0	0	0
CLPSL2	0	0	0	0	0	0	0	0	0.115045692	0	0	0
CLPTM1	26.44074163	23.93553127	25.61973055	31.0498921	22.32836876	21.53232229	25.12578901	32.95209664	34.09199478	29.32565174	32.50017386	41.1171077
CLPTM1L	54.23678233	51.81912552	59.5366504	66.26971584	52.63927675	52.96229205	59.74942891	67.74212291	104.5414328	102.0139901	112.5925268	111.9342829
CLPX	13.55103544	11.59393436	14.29487366	12.0779968	15.13853405	14.33321762	14.72358332	11.39544835	14.05987065	15.06941805	14.70597593	13.89119971
CLRN1	0	0	0	0	0	0	0	0	0	0	0	0
CLRN2	0	0	0	0	0	0	0	0	0	0	0	0
CLRN3	0	0	0	0	0	0	0	0	0.203327281	0	0.201255915	0.048610155
CLSPN	6.231984263	6.719095891	9.12398037	13.46445997	10.60539444	9.656589506	7.469830355	10.93107012	9.564136792	8.270274836	9.947532205	10.201911
CLSTN1	26.6679607	31.22245617	26.14422548	31.48012534	27.56658088	29.78735396	26.44991377	33.16751627	84.7111411	70.43040321	75.99571249	86.47361531
CLSTN2	0	0	0	0	0	0	0.003804935	0	4.968774614	8.276760206	4.742792227	8.644538808
CLSTN3	9.375477869	10.65219501	8.038741747	6.64260947	7.693299777	7.605364062	8.190228121	6.671965574	3.102082959	3.406095527	3.162289358	2.798959393
CLTA	83.30259422	81.26353056	84.7615767	93.7781444	71.58479761	74.51450678	82.66725161	96.11181258	98.65433122	92.96647179	106.2709328	101.4951748
CLTB	12.78371955	11.09132439	15.00165237	13.79318562	11.01745053	10.03555882	14.61887997	15.9298292	17.62477483	15.93464513	19.19201616	22.66256806
CLTC	49.77759446	47.15857965	51.43481899	54.33462476	48.7979213	46.40132371	50.59747983	46.68910338	78.11622512	76.44932471	75.19265158	77.75962335
CLTCL1	12.04720293	10.68947074	11.8880048	13.15520032	8.683414334	11.16044555	10.43575893	12.36076767	11.84734791	11.4432726	11.73698669	12.88673412
CLTRN	0.07568152	0.130764467	0.133503941	0.076491095	0.105366711	0.025907388	0.07782724	0.049369834	0.466709034	0.387276719	0.271737945	0.183775102
CLU	0.349412733	0.561463563	0.462279069	0.141260178	0.018242467	0.071766829	0.107795782	0.068380426	0.34222358	0.574717932	0.094093675	0.81816573
CLUAP1	3.098848574	2.952435445	3.133797246	2.776953118	3.27505238	3.68488952	3.535051527	3.486461097	4.915125331	5.750484607	5.121819932	5.992887518
CLUH	27.72479812	23.37798456	29.44845885	28.04623751	29.3692044	31.51244071	29.8179143	33.71648961	7.040912042	7.121527657	8.824099658	7.85964429
CLUL1	0	0	0	0	0	0.009969413	0.009982892	0	0	0.010644845	0	0
CLVS1	0.048906667	0.164779273	0.064704364	0.0865022	0.038300476	0.050225366	0.075439902	0.071783133	0.079834025	0.06703519	0.079020728	0.076344784
CLVS2	0	0	0	0	0	0	0	0	0	0	0	0
CLYBL	1.301571577	1.343491936	1.168873836	1.531896372	1.312005874	1.53919183	1.129880391	1.334241744	1.569729195	2.038894611	1.620499987	1.524577351
CMA1	0.345301995	0.178986487	0.121824131	0	0.060092833	0.177306284	0.355091986	0.112626585	0.501033216	0.063106283	0.185973381	0.179675611
CMAS	25.52811758	23.08308484	25.68893528	21.41529442	23.90595159	21.7292237	25.32861133	21.0553145	77.83884003	86.43911125	81.75243631	77.30215068
CMBL	0.066480802	0.082704406	0.084437037	0	0.152719245	0.150201395	0.191423857	0.143113964	0.506434148	1.151802712	0.630174193	0.553485533
CMC1	1.648377101	1.888745395	1.825686654	1.753694739	2.008955148	2.085893637	1.742344057	2.00244919	2.204820567	2.406286131	2.16586791	2.347450046
CMC2	12.4600715	10.48412294	14.72223917	14.5120142	12.86638044	13.00871603	14.58803977	14.01601363	11.94453223	11.54351134	12.97539101	12.93111198
CMC4	5.291008934	7.075862044	4.872066837	9.037456729	5.745749319	4.835969334	5.930711208	8.024782586	4.0305585	2.842885293	3.134605409	5.065780426
CMIP	7.726162403	8.508447734	7.513493676	7.479867596	6.28456829	6.936621377	6.805333146	7.14931504	5.554000188	6.20145905	5.868317648	6.459901023
CMKLR1	0.038135762	0.108721711	0.060545125	0	0.029865414	0.00979101	0.107846713	0.018658019	0.093377802	0.031363062	0.09242653	0.029765538
CMPK1	45.15962804	41.10509172	46.08704589	41.31787894	44.9494389	43.41623741	50.02827976	37.30630689	65.31344167	82.02212175	72.28398129	61.52233971
CMPK2	5.763359057	3.440492785	3.902855572	3.80972247	3.265679413	3.225864333	2.907202982	3.514649784	13.61405164	6.199952025	7.841012196	12.46472951
CMSS1	29.20380324	29.37362528	32.3565455	38.14079183	34.81937867	35.86186803	35.69736003	41.91156881	15.32702648	22.16638544	23.42563081	19.14050122
CMTM1	1.588296519	1.896061381	1.477308281	1.799866939	1.708721962	2.421970009	2.128275688	1.930921343	1.414197621	1.952738686	1.451634796	1.903361499
CMTM2	0.151861414	0.209912029	0.10715481	0.204647859	0.369997733	0.259926898	0.41644529	0.346726715	0.11017546	0.055507408	0.163579596	0.263400277
CMTM3	28.81581432	31.96568187	29.52181281	25.19600189	28.51939659	27.21125534	31.95131202	27.7762056	24.44211368	21.5674409	23.29107565	27.03857446
CMTM4	2.9111095	3.164065217	2.620606338	3.183824221	2.828538154	3.128069238	2.388613739	2.638643333	6.336030092	7.970294758	5.525506374	7.704632095
CMTM5	0	0	0	0	0	0	0	0	0.027376464	0.027585028	0	0
CMTM6	63.56114497	61.58460969	62.8071076	69.13670126	54.01677967	52.37102282	60.95745766	56.89206783	63.3250314	53.06186606	54.53497192	70.23985469
CMTM7	3.532612739	4.217654503	3.703525604	3.147368189	2.395019108	3.146441791	3.804938426	4.505150992	9.292069416	10.94169481	10.92868464	10.38436332
CMTM8	0	0	0.053754395	0	0	0	0	0	0.497427384	0.66828928	0.629126562	0.924946418
CMTR1	18.40125925	16.34772563	16.39761785	17.37118407	13.85531063	15.2438388	14.72061312	16.84141018	27.87305021	24.38197287	24.22299476	30.11957749
CMTR2	7.615366593	6.26713237	6.692347466	8.765533327	7.929498576	7.030955526	7.666523855	7.78611187	6.078993339	5.563135732	5.51084876	6.602490256
CMYA5	0.058033576	0.025784202	0.035099164	0.054464771	0.0346271	0.025542158	0.046890598	0.016224614	4.474985826	3.349990285	3.116652735	5.258659401
CNBD1	0	0	0	0	0	0	0	0	0	0	0	0
CNBD2	0.08772702	0.090946194	0.150883668	0.144081351	0.160304704	0.10135401	0.078937472	0.107301668	0.131269784	0.144294374	0.106308403	0.159768613
CNBP	94.2780397	93.2208062	100.1386078	91.28479836	104.13818	109.3125029	109.6238078	98.28857808	78.25072058	86.64087947	86.15465999	81.06932784
CNDP1	0	0	0	0.024743025	0.025562693	0	0	0	0	0.053689149	0	0
CNDP2	10.4737623	9.314135843	10.45162352	11.15844915	8.434781142	8.777834574	10.27962279	10.76343706	10.77267575	10.23346023	10.63677474	14.41247231
CNEP1R1	5.135352281	4.835138257	4.647599028	5.842205755	5.569461367	4.993568409	5.127878628	4.660840128	10.1511778	10.77258287	8.444397582	12.57328895
CNFN	0	0	0	0	0	0	0	0	1.38599425	1.483837816	1.457616808	1.988126586
CNGA1	0	0	0.026998237	0	0	0.013097999	0	0	0.027759307	0.027970787	0.027476513	0
CNGA2	0	0	0	0	0	0	0	0	0	0	0	0
CNGA3	0	0	0	0	0	0	0	0	0	0	0.012467564	0
CNGA4	0	0.028710305	0	0.027990308	0	0.014220396	0	0.027098779	0.105483222	0.10628683	0.074577591	0.057641687
CNGB1	0	0	0	0	0	0	0	0	0.086191135	0.060793438	0.059719153	0.140120883
CNGB3	0	0	0	0	0	0	0.011735985	0	0.012419567	0	0	0
CNIH1	50.25502908	51.43272499	53.51080014	51.02192557	57.17391737	53.00809975	57.43502667	51.72789445	57.48832405	62.23862677	60.81294699	57.41559621
CNIH2	0.714286206	0.740497191	1.008013828	1.042783469	0.911585229	0.774298461	0.571307043	0.931910236	0.993244773	0.870271008	0.769403146	0.660753969
CNIH3	1.066091404	0.853146109	0.712652112	0.548199475	0.605419102	0.749099021	0.84627994	0.658847904	1.709730133	2.2149713	2.034804334	1.537682658
CNIH4	19.93289276	20.01707848	21.14619813	24.4464488	19.77524198	20.04289945	21.63945113	25.29693763	32.53795719	33.69867362	34.79694887	40.83804526
CNKSR1	0.084176083	0.305427338	0.178186254	0.191422181	0.197763468	0.25933731	0.281328581	0.226508491	0.183209252	0.115378127	0.294682107	0.109501171
CNKSR2	0.828914049	0.733811034	0.921693648	1.007219912	1.361514809	1.214725788	1.024814014	0.7700861	0.714557702	0.791490958	0.632035886	0.639710484
CNKSR3	1.08302259	0.932758189	1.049292611	1.136708122	0.78290941	0.983891119	1.09659413	1.21462863	2.003618952	1.653474494	1.740915081	2.427572639
CNMD	0	0	0	0	0	0	0	0	0	0.161766619	0	0
CNN1	0	0.02252084	0.022992645	0	0	0	0	0	0.023640798	0	0	0
CNN2	145.4843268	151.0856451	149.2216515	110.8620745	119.6505374	115.0536628	135.6449994	124.1311383	125.9416204	91.79487438	116.310122	125.5579706
CNN3	0.043836709	0.045445313	0.139192134	0.011076409	0.01144334	0	0	0	4.782456432	6.068678013	5.784365538	2.817052548
CNNM1	0.008935497	0	0	0	0	0	0	0	0	0	0	0
CNNM2	2.44708089	2.618419875	2.099769588	2.579254382	2.09890591	2.234830954	1.788484393	2.42868794	2.15896124	2.232908758	1.54388816	2.928641453
CNNM3	3.718201232	3.253606293	3.436312045	5.304917322	3.390095593	3.945474488	2.583526729	4.447625608	6.359724514	5.365575199	5.145860201	7.208018009
CNNM4	2.502770273	3.541848997	2.522825307	3.683897541	2.28148684	2.713045769	2.747353296	3.498533443	5.782330662	5.848163349	5.348994431	6.439149866
CNOT1	50.89914452	50.09951355	51.32765777	56.21221794	54.49427357	55.89165305	51.10912258	48.98550151	77.47657415	72.0936986	75.44757484	66.32075634
CNOT10	14.10931876	15.35581577	14.29576812	16.13793183	18.05317667	16.13983533	14.2167492	16.29538528	20.56369276	16.24226168	20.26406063	20.72743067
CNOT11	27.56724921	25.72095524	25.98838194	32.85140645	28.11571337	27.5424446	27.53572889	30.48767653	34.65123234	32.92340983	34.16011447	41.43206852
CNOT2	26.74082364	25.52888447	25.63826797	24.5572503	26.65200415	25.01765993	26.28066947	23.85787297	20.62834611	19.3704158	18.48120372	19.08828201
CNOT3	13.98847102	14.68081726	15.26255332	17.26536377	15.65836966	15.68102341	15.76142118	20.02472963	21.50320592	20.3414385	21.37715682	23.49886929
CNOT4	3.314072808	3.710132001	3.9227681	4.11258108	4.064533356	3.675303822	4.194502521	3.434720907	6.647557243	4.956453457	6.115128721	6.867211902
CNOT6	11.03630379	11.15053792	11.58470435	11.85916904	13.77166215	13.17875142	11.69217521	10.90291656	16.51410489	14.59525023	15.57838154	14.00958506
CNOT6L	16.80777906	15.16365634	14.47258112	10.2673396	24.28829209	17.23794675	16.32727604	10.22008809	9.511598537	7.832003847	7.826460507	8.121532317
CNOT7	14.5509245	14.78568762	14.82447396	15.15351454	17.5802466	16.28653826	16.58140325	14.97146846	16.44798103	16.62432935	16.57624501	16.25227557
CNOT8	24.48944105	26.82515142	23.69698082	23.73248185	27.76443679	27.00472058	24.91018486	22.82585964	36.14368612	35.7127699	32.17078166	35.14684986
CNOT9	20.87178417	20.59341369	23.66154199	23.91940286	22.18971395	24.05965618	24.06990696	24.80029233	17.76314279	17.66093089	18.79555245	17.16713425
CNP	10.31317818	9.683356844	10.76273033	11.52327123	9.270620676	9.167223531	10.4570396	11.28815464	18.6321727	13.94860031	16.35748151	20.84425726
CNPPD1	20.43426681	22.77974108	20.17033015	25.50392174	17.84782011	19.21741201	20.34718066	27.28757332	23.33955372	17.74350587	21.18209042	27.90460747
CNPY1	0	0	0	0	0	0	0	0	0.026212291	0.10564794	0.025945257	0.100266605
CNPY2	38.51968107	40.54478649	39.52091128	40.52179533	33.16278841	37.71545231	39.23260875	44.35449969	31.40584458	31.8876998	36.93767775	35.50775312
CNPY3	31.28414111	33.36620363	30.18408698	35.30376793	30.78458944	29.45933325	29.32677706	37.89042506	45.53771003	50.3421278	50.33278783	46.01155629
CNPY3-GNMT	0	0	0	0	0	0	0	0	0	0	0	0
CNPY4	4.063170922	4.553805853	4.997897135	7.362345953	6.383126103	5.826783046	5.194730046	7.342755311	2.151119647	2.40834181	2.562932475	2.818992186
CNR1	0	0	0	0	0	0	0	0	0.052759508	0.091824319	0.066464399	0.298134853
CNR2	8.460531973	7.10959426	6.601449295	2.363938104	0.919612213	1.408570852	4.676838713	2.331025805	0	0	0	0
CNRIP1	0.628398851	0.651458169	0.886808013	0.58807492	0.291626983	0.69314589	0.502611819	0.250511337	3.697883015	3.164594472	3.008392923	3.826914367
CNST	7.226590262	6.533425666	7.044652363	6.418327337	8.166978648	6.893110682	7.861330244	6.520656944	6.797096764	6.23186326	6.147715805	6.901567305
CNTD1	0.015887086	0.016470068	0.050445333	0.048171097	0	0.081577293	0.065350065	0.108819161	0.051867367	0.05226251	0.051338976	0.049600442
CNTD2	0.240435929	0.199407048	0.330824914	0.121503951	0.175740657	0.098767576	0.074175828	0.211740999	0.418647057	0.158188671	0.129494423	0.450393288
CNTF	2.471716893	2.503511269	2.555959034	3.531877369	1.957934905	2.48001002	2.424930799	2.696586086	1.453135207	0.591912942	1.162906409	1.80355482
CNTFR	0	0	0	0	0	0	0	0	0.018151971	0.018290259	0	0.017358617
CNTLN	1.380014657	1.381190654	1.390702933	1.780566191	1.433317147	1.390840271	1.264394055	1.479640302	4.521379646	4.483382649	4.459504927	3.918891631
CNTN1	0	0	0	0	0	0	0	0	0.853510718	1.576690612	1.065202412	0.733403407
CNTN2	0	0	0	0	0	0	0	0	0.006266266	0	0	0
CNTN3	0	0	0	0	0	0	0	0	0	0	0	0
CNTN4	0.628023184	0.669538751	0.641137179	0.562713854	1.288282694	0.933129174	1.003095916	0.836798464	0.896720268	0.835174904	1.055506564	0.533057973

CNTN5	0	0	0.006760777	0	0	0	0	0	0.02780544	0.014008636	0.006880544	0.066475426
CNTN6	0	0	0	0	0	0	0	0	0.009008963	0	0	0
CNTNAP1	3.86919667	4.697128879	3.901789093	1.897971688	3.888792363	3.345784879	3.862250856	2.608290043	0.802355788	1.478737102	0.834909187	0.773845891
CNTNAP2	0.005273896	0	0	0	0	0	0	0	0.005739318	0.040481292	0.005680849	0
CNTNAP3	0	0	0.010891716	0.010400682	0	0	0.010582361	0	1.959781026	1.636189382	1.662699474	1.670649879
CNTNAP3B	0.008435423	0	0.008928168	0	0	0	0	0	0.807826716	0.786231669	0.717820127	0.86908465
CNTNAP4	0.022194086	0.011504253	0	0	0	0	0	0	0	0	0	0
CNTNAP5	0	0	0	0	0	0	0	0	0	0	0	0.004999455
CNTRL	14.37743242	11.52778775	12.80097075	13.50069195	21.05916836	18.30161058	14.98289851	11.89726518	27.37045456	24.68184411	24.28022743	17.79204675
CNTROB	12.20224752	13.90172215	12.42873444	17.98079939	15.00620416	15.8122162	12.4603019	18.43270704	8.779087387	9.584110051	9.011916991	9.062570006
COA1	2.879026022	2.63353491	2.696673167	3.411910493	3.26557768	3.424308735	3.103847016	3.119109686	5.782924813	5.286375964	5.849680961	5.338264031
COA3	19.77900938	17.81225668	22.06215414	23.01946493	19.19252032	16.27721428	22.3943068	23.45922549	27.75719363	23.51411948	26.03972578	32.29639463
COA4	14.74643621	12.01481479	14.26829722	12.84892725	14.13579078	14.4430744	13.8922858	14.34597492	10.46125311	11.18026861	11.85580302	11.42472249
COA5	9.744798798	11.14419635	11.24873836	11.17250492	12.33756529	12.04033761	11.68082491	11.56159377	12.99085273	12.52215075	9.676685104	12.64491264
COA6	19.63524366	20.12268457	23.55846246	20.03464776	23.24161448	23.55111465	25.74087197	22.47649378	13.41620235	15.69614972	14.93442225	15.16961814
COA7	18.83589169	14.14394003	18.15807692	19.01165544	16.69125333	15.6057208	18.45378115	16.81207503	14.1825107	14.87669419	18.64425662	15.0195722
COASY	23.21724288	23.97076524	25.59524995	23.91347855	27.72983768	26.3949955	23.69647189	25.67604564	23.40607981	24.62564918	24.07115458	26.2536014
COBL	0	0	0	0	0	0	0	0	0.01265714	0	0.018792296	0
COBLL1	0	0.011432344	0.005835924	0	0	0	0	0	0	0.00604615	0	0
COCH	8.274551902	7.145614599	9.691840878	6.613048038	7.353655885	7.112732291	8.594756892	7.265843451	2.065483077	1.533529531	1.918905412	0.745034855
COG1	5.513928642	6.396334218	5.160466181	6.64807168	6.627635555	6.008550684	5.560865306	6.661853205	10.86270139	12.94791213	9.01414304	13.63531672
COG2	21.94193918	20.65520182	22.83883493	25.22663395	21.62052826	22.72026286	23.64830654	23.46257178	19.9810269	19.39569468	20.93279095	25.89949071
COG3	12.38798041	11.2221255	12.34614813	13.03923617	12.91090539	12.93763175	13.37496471	12.16735781	12.47837995	11.06411962	10.03932392	13.24404913
COG4	12.87652347	10.50123854	12.00839021	9.297969532	11.5032797	10.75529265	12.10870597	11.08777432	23.21466665	22.16782264	20.77436228	24.88012761
COG5	9.145721457	8.560241974	9.262010431	8.880083878	8.268611566	8.030906637	8.810408724	7.803755987	8.19554692	9.031202888	7.101846799	9.899425309
COG6	5.278280599	6.228898394	5.393408265	6.295611743	6.75829903	6.373503066	5.951952333	5.440182418	2.317499926	3.027362246	2.089079072	2.643622927
COG7	8.766325809	8.940236722	10.20246844	10.08466702	10.26990492	10.02739447	10.22418003	10.7398026	8.182643868	8.752966791	8.367981058	8.381292144
COG8	12.29316621	11.3133329	12.00687894	10.57186644	10.98964238	10.56482514	10.82307091	12.02892551	11.07795484	9.317724847	11.26710472	12.63622447
COIL	13.23788741	12.05055628	13.05978736	14.45315794	15.18792734	14.72773042	15.04175406	13.53304194	18.02989616	15.36712255	17.14205283	19.58048057
COL10A1	0	0	0	0	0	0	0	0	0	0	0	0
COL11A1	0	0	0	0	0	0	0	0	0	0	0	0
COL11A2	0.682878348	0.428658986	0.563626314	0.569876024	0.542962427	0.373164117	0.476749613	0.65593938	1.022673019	0.72819463	0.715326649	1.512602693
COL12A1	0	0	0	0.00412728	0.012792016	0	0	0.003995821	0.328854142	0.304492484	0.224333831	0.246485249
COL13A1	0.015610549	0	0.01652242	0	0	0	0	0	0.01698818	0.102705612	0.033630231	0.048737075
COL14A1	0	0.018719504	0.006370557	0.006083352	0.006284877	0.012362519	0.006189616	0.005889589	0.032750703	0.052800335	0.025933648	0.025055436
COL15A1	0.142630043	0.207009464	0.191218032	0.086493465	0.049643752	0.1367108	0.107560849	0.046521407	0.196608391	0.615171954	0.573574025	0.207806481
COL16A1	0.008739575	0	0.018500173	0	0	0	0	0	0.028532527	0.009583299	0.047069761	0
COL17A1	0	0	0	0	0.030046416	0	0	0	0	0	0	0
COL18A1	0.110654209	0.294980667	0.184042478	0.095861049	0.181567211	0.162339773	0.130047398	0.193349472	1.608459764	1.820052675	1.430312311	1.965883787
COL19A1	0.019852996	0.012348905	0.037822833	0	0	0	0	0.003885251	0.246297274	0.18721872	0.128309557	0.26445761
COL1A1	0.036253704	0.037584047	0.028778566	0.018320757	0.037855347	0.05584685	0.083883529	0.070948868	0.35507786	0.457167125	0.370986144	0.433880638
COL1A2	0	0	0	0	0	0	0	0	0	0.021809046	0	0
COL20A1	0.006304577	0.006535925	0	0.006372017	0	0.025898282	0.019449971	0	0.006860956	0.020739675	0	0.019683268
COL21A1	0.032144302	0.022215899	0.011340657	0	0.044752528	0.011003676	0.033055656	0	0	0	0.011541558	0.022301433
COL22A1	0.028903972	0.009988204	0.015296181	0	0.020120608	0.029683326	0.009907819	0.004713781	0	0	0	0.00501333
COL23A1	4.12906018	5.043339982	3.823972459	2.142109702	2.545605979	3.902065033	4.697842824	2.804574345	8.580577259	10.59670396	7.771599858	16.47967594
COL24A1	0.09916125	0.042329415	0.080258662	0.011790822	0.036544259	0.023961174	0.059983922	0	0.56495229	0.428541255	0.521498255	0.394572187
COL25A1	0	0.003964475	0	0	0	0	0	0.003741946	0.016646509	0.008386664	0	0.067655553
COL26A1	0	0	0	0	0	0	0.015487033	0	0.049167302	0.016513959	0.048666418	0.031345591
COL27A1	7.2878956	7.003255131	7.447622686	2.249670045	7.259986657	7.699472321	7.549900032	3.553911653	9.428706352	7.781517622	9.642022537	3.549357538
COL28A1	0	0.004200928	0	0	0.004231252	0.004161492	0	0	0.035278715	0.013330305	0.00872983	0.067473634
COL2A1	0	0	0.009570288	0	0	0	0	0	0	0	0	0
COL3A1	0	0	0	0	0	0	0	0	0	0	0.010557687	0
COL4A1	0.162118549	0.286072419	0.175239327	0.073210804	0.039618896	0.141693484	0.117055163	0.081004505	0.566813898	0.525744105	0.587048024	0.545630296
COL4A2	1.307148546	1.225645255	1.91223171	0.530135482	0.521616574	0.735324066	0.804812901	0.399194412	0.027181614	0.182591287	0.098650586	0.025993609
COL4A2-AS2	0	0	0	0	0	0	0	0	0	0	0	0
COL4A3	0	0	0	0	0	0	0	0	0	0.006833956	0	0
COL4A3BP	7.112995164	7.656308683	6.934597202	7.455963921	7.117051935	6.65227112	6.389722652	5.619757417	9.240369041	8.686428366	8.346272004	8.364196821
COL4A4	0	0	0	0	0	0	0	0	0.086268658	0.169276719	0.121343413	0.021710048
COL4A5	0.037120282	0.023089454	0.031430895	0	0.031008167	0.007624235	0	0	4.532447877	4.265768786	3.18277567	5.153320299
COL4A6	0.006376296	0	0.013497519	0	0	0	0	0	0.020817013	0.013983736	0.027473257	0
COL5A1	0.02506968	0.020791696	0.015920457	0.005067571	0.020941781	0.010298259	0.025780455	0	0	0	0	0
COL5A2	0.091151148	0.070871978	0.088435996	0.076771836	0.134835626	0.039003712	0.085924174	0.081759205	0.793561925	0.458108496	0.376374699	0.553348803
COL5A3	0	0	0	0	0	0	0	0	0.084526754	0.028390236	0.055777101	0.035925516
COL6A1	0.280191796	0.158440109	0.417878395	0	0.039895952	0	0	0.012462232	6.943831193	7.331904505	6.831935721	7.197018241
COL6A2	0.034434446	0.059496717	0.133634939	0	0	0	0.011803577	0	8.031774593	7.274856735	7.035027599	7.836023387
COL6A3	4.276696087	3.907690259	2.920655451	0.958413404	1.38622818	1.748039882	3.481303187	0.858295157	0.103195448	0.062388976	0.112358576	0.103619427
COL6A5	0	0	0	0	0	0	0	0	0.011014438	0.005549175	0.005451115	0.005266519
COL6A6	0.002952676	0	0	0	0.009249363	0	0	0	0.009639748	0.003237729	0.003180515	0.006145621
COL7A1	0.59340308	0.388219262	0.365863691	0.267849867	0.433131638	0.159746508	0.379170315	0.264955849	1.548596306	1.200303118	1.464556966	2.212376196
COL8A1	0	0	0	0	0	0	0	0	0.113668568	0.038178178	0.018751764	0.054350274
COL8A2	0	0	0	0	0	0	0	0	0.055642586	0.112132981	0.055075735	0.021284264
COL9A1	0	0	0	0	0	0	0	0	0	0	0.013209139	0
COL9A2	3.700263852	2.634860113	2.808738694	0.692564083	0.455322517	0.383842037	2.357413959	0.731459991	181.9522597	115.4950175	152.6275656	119.7383262
COL9A3	3.521568119	5.069387275	2.811431239	3.640592812	3.361962521	4.649814203	3.228229689	3.760923452	4.883504609	5.207566322	4.638670375	3.85332737
COLCA1	0	0	0	0	0	0	0	0	0.027982601	0.028195783	0.018465022	0.017839726
COLCA2	0	0	0	0	0	0	0	0	0.41469828	1.181528375	0.792638683	0.382898454
COLEC10	0	0	0	0	0	0	0	0	0.015951582	0.016073107	0	0.015254399
COLEC11	0.024425584	0.101287556	0.025852375	0	0.153028049	0	0.050236194	0.09560223	0.026581143	0.053567294	0	0
COLEC12	0	0	0	0	0	0	0	0	0.11210898	0.018827178	0	0.017868187
COLGALT1	64.29649432	67.42444569	69.3933688	77.09560849	61.47894199	72.25941726	60.78274327	76.98776118	64.66052032	69.02565837	76.96768314	65.66403944
COLGALT2	0.04932778	0.029221646	0.05966766	0.014244413	0.022074436	0.014473666	0.021739851	0.048267483	1.104294004	0.942710014	0.797011378	1.136698444
COLQ	1.374813498	1.541272525	1.032121111	1.147161904	0.884700038	1.149207397	0.756214415	1.110623265	0.782864399	0.771299011	0.654350784	1.48066013
COMMD1	4.039984654	4.089917981	3.79417542	4.543279858	3.663925889	3.759347375	4.096011722	4.324332851	4.458436209	3.889255553	4.004403919	5.526856424
COMMD10	3.098102035	3.095898726	2.991732391	4.091598231	3.960339717	3.92784681	3.292684719	3.508111711	5.291879886	5.209616076	4.850927565	4.761443691
COMMD2	8.363277673	8.519647296	8.913279319	10.81539915	9.460486332	9.707112393	10.28762295	10.14414215	9.654369958	9.409494128	9.759337044	10.713227
COMMD3	40.78559072	44.7102037	42.56343741	48.78499731	52.48872908	50.86074053	51.45818159	47.51060411	27.47637964	20.73295956	22.33556096	19.0229805
COMMD3-BMI	0	0	0	0	0	0	0	0	0	0	0	0
COMMD4	26.18411667	28.76368775	31.23080344	34.80003348	27.59173147	28.53479036	27.99695994	38.7845231	29.62768959	22.78517134	31.30967028	33.83266805
COMMD5	13.70192865	13.57758555	14.3422406	15.13246681	11.55951	10.93405363	13.12616124	16.6926653	14.05529835	12.23868136	13.4559773	18.32004448
COMMD6	46.64728631	47.37266523	39.13405749	39.50671137	35.57209403	39.38257632	43.67564577	40.75688361	26.34546339	24.28569042	24.59671511	23.04866268
COMMD7	19.72022337	18.43262975	21.09780131	22.45219718	19.67873613	18.54421886	20.38894854	22.73838822	22.3653565	21.90455577	23.19386715	23.49557602
COMMD8	10.76899704	10.77856466	10.61068934	11.79717778	12.59486829	11.20488561	12.42218054	10.90147858	8.673869545	8.196130999	8.299322282	8.27633495
COMMD9	12.21884326	12.68581894	13.61625348	11.75111346	11.10996052	11.22161363	12.49146671	13.09739397	7.966578487	6.669717541	8.001382232	8.290600561
COMP	0	0	0	0	0	0	0	0	0.167172838	0.096255097	0.212746873	0.11419025
COMT	24.41853009	22.92119852	25.41527935	25.80795483	24.88802325	24.178079	23.3889571	29.15646597	28.58890862	24.59211076	33.38058511	22.98304641
COMTD1	2.682470668	2.822410919	2.627286042	1.699536519	3.302646646	2.919265296	3.376103993	2.546458587	2.091366324	1.975592891	1.768176972	2.249955757
COP1	8.514830468	8.515239747	8.987707635	9.044693487	8.667369754	7.852739433	8.399087982	8.484733932	8.308771629	7.61039639	7.912371121	9.150422476
COPA	65.86115506	72.35395523	64.48148466	66.31611893	60.02447796	64.75987379	61.33160153	62.96792134	60.53224702	66.27318008	64.95884544	55.95936555
COPB1	63.19228837	60.63972855	64.10619273	61.60157316	60.83849843	54.52407026	67.08624266	55.2057071	82.9976544	92.1953805	82.43150143	75.2420702
COPB2	38.36063812	35.9488028	39.12803415	38.43246791	37.5307268	33.68727429	40.395426	36.82939705	54.97026815	61.82776981	54.88379175	50.58527255
COPE	116.138601	124.7019948	127.6882291	144.2389254	113.9407434	121.1287492	135.4100129	173.8540086	94.53867916	86.78822248	101.56374	103.6370105
COPG1	44.78344097	45.91264578	44.84484746	43.14055463	41.58340949	43.37648898	47.4301439	46.27957847	73.14834815	90.63651974	78.76025047	71.94697233
COPG2	12.81497852	14.48920253	12.81472901	18.26780409	13.8968589	17.62960797	12.40957374	14.92986779	9.965508396	13.01287244	8.210402577	12.72512809
COPRS	16.56338955	18.68367712	20.02884893	22.33521677	18.77373894	18.24388345	22.19584485	24.94086079	18.21189813	16.93905484	19.82900388	21.16704789
COPS2	24.19334443	22.107242	25.70361649	23.96230652	28.70888275	25.65279807	30.3616038	21.55847747	32.98534733	35.23313785	32.71986144	30.8348367
COPS3	46.31512082	43.79389602	48.74609137	55.30250879	49.33607089	48.97541364	53.97512279	56.02779072	24.60857839	26.38846236	28.38028478	24.82842744
COPS4	21.11514414	19.83043145	22.19836782	24.35285239	22.37396253	23.20006594	26.12076754	24.9657081	23.13298235	22.998013	23.01960066	24.01218605
COPS5	54.42349545	55.55948765	60.20176547	65.62720345	60.03314323	64.90322315	66.17937527	66.50524235	65.23940049	62.1327942	64.26358016	72.23482119
COPS6	30.90464961	35.98975593	38.78061055	45.04129321	37.58920469	43.4023237	38.02837624	50.88059974	50.42744718	52.96325327	53.53125085	51.32577895
COPS7A	19.14266373	17.81219678	21.99044484	20.26761815	19.9883622	18.8436965	18.94119195	21.92920225	16.1066963	15.94782005	14.63501585	17.41918025
COPS7B	10.10770494	9.688765709	10.71963809	11.44788805	12.30445279	11.4339178	11.27178519	12.41523645	10.34746623	11.57363507	10.00132096	11.19555104
COPS8	13.20337912	12.96746695	14.48949365	17.16070968	13.18200829	13.98758056	15.76921271	16.90877509	24.67866279	28.93068549	23.52900842	27.62580201
COPS9	9.589092408	7.991008523	12.14003769	10.80993756	6.970397911	7.12060741	11.07461893	10.57389171	7.987041231	9.463347222	9.097484673	10.47820383
COPZ1	53.5431769	50.06763002	56.30430904	54.89671226	52.59863179	51.15695917	56.62266931	58.46560308	64.55902895	75.56649329	64.93432445	61.29384422
COPZ2	0.174895647	0.302189175	0.061703988	0.235688705	0.121748209	0.059870486	0	0.114090852	4.314151116	4.474871294	3.579433487	3.700902307
COQ10A	6.886431335	7.509995331	7.951302431	7.442180966	5.883271436	6.735346751	6.039350732	7.526014132	5.580066601	5.557198692	5.683779251	6.422034382
COQ10B	8.576331037	5.469834922	8.261604339	7.569584612	5.364879781	5.174960314	8.45370174	7.115783189	7.483758156	6.782361062	6.066502219	9.1103607
COQ2	3.854289552	3.718363612	4.690020834	5.60244978	4.530911822	4.902691399	4.496627222	5.743063344	4.312711909	4.712282227	4.71906972	4.916000286
COQ3	11.51061122	10.08496663	12.42557131	12.99787513	16.59278862	14.20086559	14.58669593	12.21009696	4.37870545	6.98111386	5.650786064	3.524776879
COQ4	11.35845714	11.0881093	10.61885564	12.54573878	10.60187634	11.32437169	10.56511324	14.06240023	10.59032309	8.95307136	10.25526224	12.19684863
COQ5	24.47421802	22.72146993	24.89267778	26.55359407	27.55060257	27.73122751	26.98853619	28.15488178	20.79353253	26.25155454	21.51998501	19.88472648
COQ6	3.24264551	2.774934991	3.448249692	3.091823115	2.922735688	2.874549152	2.79978962	3.038244585	6.008962706	5.283222831	5.502901939	7.799732925
COQ7	3.41531672	3.87495261	3.428647417	4.99259107	3.596812225	3.974056405	3.210675772	4.718826864	4.897135636	3.50393724	4.373574479	4.164450935
COQ8A	23.76900769	24.70328838	23.2405369	22.85841161	25.99127848	30.61998149	24.58143619	27.3297258	18.0805745	15.378887	16.92900941	19.65802791
COQ8B	9.374112525	10.47767444	9.191818528	9.779311454	10.0357678	9.272780192	9.307477176	11.2601777	6.8947247	6.640059748	5.571008762	6.256985493
COQ9	27.81275703	28.50307698	26.40262153	30.10665047	27.61103781	30.55847738	28.47025511	31.45459128	22.60508206	23.58078904	24.4681437	30.27057113
CORIN	0	0.020971474	0	0.020445552	0	0	0.010401347	0	0	0.022182096	0.021790114	0
CORO1A	161.1864955	195.8835239	167.1065445	141.1700121	145.8465837	179.5738797	160.4357609	159.4668476	96.00774797	85.51288623	116.9194759	112.7539203
CORO1B	27.35342002	29.42830877	22.96529534	17.39555923	19.30622692	21.24972661	23.73903867	24.82323503	48.64566681	41.23448698	43.78612783	57.83817545
CORO1C	62.11980079	57.1659014	63.47205257	47.05761164	58.70671698	54.2027429	59.07332897	46.1021887	42.0314005	44.06781946	45.57319166	32.97514441
CORO2A	0.190875579	0.584644939	0.183659404	0.473524525	0.045297322	0.053460618	0.124910086	0.263179745	4.909753898	6.688177228	5.971868532	9.670238738
CORO2B	0.553976261	1.008223659	0.403920435	0.671882659	0.205671192	0.417203188	0.202553806	0.686620205	1.245920363	1.147419771	0.861933365	1.94734217
CORO6	0.348787945	0.4239294	0.572837541	0.388986454	0.502340623	0.382895444	0.408149428	0.282447537	2.827130873	3.930108081	3.303583914	2.828732932
CORO7	2.825797945	3.10828686	2.260699652	2.654897284	2.050208629	2.384265347	2.673987402	3.050650433	3.089612182	3.636857393	3.401105885	4.349026907
CORO7-PAM1	0	0	0	0	0	0	0	0	0	0	0	0
CORT	0	0	0	0.176718663	0.045643216	0	0	0.085544969	0.047569665	0.047932067	0	0.045490576
COTL1	122.0840574	130.4928567	137.4827893	115.2614702	96.6050441	111.0854057	139.438673	123.6777655	132.41722	137.9623785	133.6858649	135.6016346

COX10	9.607569827	8.826601534	9.789352149	9.72845954	10.23790102	8.964638015	9.75093398	10.10262715	3.842763705	3.734454073	3.861539218	4.010580696
COX11	4.262894447	4.377383856	4.212182961	4.431156957	3.954883204	4.414189435	4.482559413	4.097042069	3.791621005	3.193921589	3.491537642	3.867981453
COX14	12.65790471	11.16799233	14.30945756	11.65007616	9.673803168	9.735576284	12.57365677	13.966993	4.747966789	4.843201898	6.266130125	6.053935009
COX15	15.34579851	15.07802579	14.74795307	13.07235742	15.07169797	15.71426814	15.38012182	12.46891566	8.914170315	9.490044904	7.944190868	8.792393381
COX16	0.846160829	0.714764518	0.663398734	0.601816077	0.490857297	0.547133253	0.773467693	0.674644393	0.409259805	0.412377689	0.438848077	0.13045754
COX17	46.17699904	40.22259366	53.0482171	51.81376583	48.48269858	46.37698059	51.54150389	51.78160327	61.88071136	77.41708691	68.92372799	54.8074329
COX18	5.770811746	5.134940073	6.045197223	5.018142621	6.384582271	6.425351712	6.348738706	5.136588251	5.016331644	6.288948951	4.697182767	4.661436635
COX19	5.577887417	5.8966246	5.170115498	5.882182414	5.583067703	5.51361762	5.113779571	6.243855741	7.554745493	8.673920498	7.608140206	7.659632656
COX20	4.547542012	5.634759767	4.717301078	12.12221603	9.496893237	8.651889978	5.682567745	10.97379213	21.64881019	16.01263492	17.01771232	23.87023769
COX4I1	287.0531524	291.7642282	301.548149	268.5450305	243.8929647	259.6350939	300.0754384	292.6015764	180.9328802	191.6849705	196.7939962	175.9903996
COX4I2	0	0	0	0	0	0	0	0	0	0	0	0
COX5A	172.6797726	182.0590881	204.956048	180.6717679	165.9334806	165.4225416	193.0981767	195.2261174	128.5239668	140.5376916	153.7114271	132.7246628
COX5B	170.059523	189.7764774	195.7822343	206.3405605	179.7977556	197.1868244	211.9172568	235.5306239	140.539817	149.4388016	160.5711355	133.8429127
COX6A1	248.6340373	265.3646768	291.5345169	306.0591918	235.0612048	250.7008566	293.1216061	319.7836941	161.0352069	174.0216425	181.3836531	149.8851645
COX6A2	0	0	0	0	0	0	0	0	0	0	0	0.127270225
COX6B1	418.1641297	433.6033595	452.8228764	427.9836234	361.7858441	391.7872851	444.5571114	472.6269634	246.1431822	261.3193732	269.7675225	179.7659566
COX6B2	0	0	0	0	0	0	0	0	0.070424843	0.141922728	0.139414801	0.235713946
COX6C	126.8890914	126.5889906	134.5593364	138.847357	137.0285479	132.063979	142.3262659	138.0050914	140.104479	176.9596286	161.7462592	112.8608813
COX7A1	0	0	0	0	0	0	0	0	0	0	0	0
COX7A2	123.6428056	118.8901954	138.1069906	123.3146891	121.7764741	113.603535	149.2097595	130.4013048	150.8871907	162.2499285	172.6963968	115.8566225
COX7A2L	66.29443927	62.76400687	67.86639309	63.36006095	55.52252121	58.71763996	65.80250998	60.7453981	38.13498317	44.00621811	43.37134377	36.35790575
COX7B	262.7936229	275.4952677	308.3693356	319.5128655	257.7692604	281.6344423	304.5861214	327.2333309	232.0499353	267.2018268	293.7488973	193.2944447
COX7B2	0	0	0	0	0	0	0	0	0	0	0	0
COX7C	490.367718	535.6372177	538.7425258	462.7416188	457.6394041	483.6308438	548.1852503	492.6347692	497.9610017	594.5379342	543.7292004	427.2151865
COX8A	231.7950314	248.8830045	270.8346267	320.7330456	246.1358575	251.8874157	231.6000518	339.388106	254.9074425	293.5089391	312.5587728	254.3697515
COX8C	0.202672546	0.315164529	0	0.10242028	0.634879081	0.20813732	0.416837423	0	0.220558407	0.444477396	0	0.21091864
CP	0.009851622	0	0.010427093	0	0	0	0	0	0.010721028	0	0	0
CPA1	0	0	0	0	0	0	0	0	0	0	0	0
CPA2	0	0	0	0	0	0	0	0	0	0	0.043580228	0.042104435
CPA3	0	0	0	0	0	0.032698496	0	0	0	0	0	0
CPA4	0	0	0	0	0	0	0	0	0	0	0	0
CPA5	0	0	0	0	0	0	0	0	0	0	0	0
CPA6	0	0	0	0	0	0	0	0	0	0	0.018412231	0
CPAMD8	0.026004379	0.040437928	0.006880848	0.026282551	0	0	0.020056241	0.050890833	0.219319315	0.171089161	0.161063089	0.074421637
CPB1	0	0	0	0	0	0	0	0	0	0	0	0
CPB2	0.218405118	0.113209785	0.086686121	0.082778034	0.028506747	0.084110287	0.112298666	0.053427629	0	0	0.058814514	0.05682283
CPD	10.71749942	11.81353576	9.846744986	17.40585272	8.841732321	10.04026359	9.350729933	12.29406993	23.87991478	27.27222168	20.59000138	40.27201435
CPE	0.040641662	0.126399067	0.086031392	0	0	0	0	0	3.095980358	4.523371598	3.130107108	4.589033628
CPEB1	0	0	0.027198081	0	0	0	0	0	0.097876743	0.112711317	0.055359793	0.040113824
CPEB2	6.928242434	4.92193511	6.608374318	4.215507237	8.379371783	5.949824376	6.994302643	3.845543122	12.40284441	11.03073885	10.62415018	8.846097696
CPEB3	0.932972597	0.891249584	0.806520868	0.888646546	0.95888872	0.827702947	0.843891449	0.707392283	1.546882063	1.135523544	1.073364883	1.408105935
CPEB4	3.301767985	2.725546031	2.9590102	2.326604873	1.946142125	1.844339195	2.621106128	1.600301182	3.908808993	3.207715729	3.011429265	3.982029737
CPED1	0.181152575	0.127704022	0.130379381	0.109854232	0.083228492	0.05953187	0.126676253	0.028361394	0.16559701	0.119184704	0.187325732	0.128195702
CPHXL	0	0	0	0	0	0	0	0	0	0	0	0
CPLANE1	0.810073466	0.779047952	0.67132042	0.686347099	0.964642026	1.012459395	1.070545849	0.806153099	7.821522484	5.926896796	6.063514547	7.78818766
CPLANE2	0	0.060242032	0	0	0	0	0	0	1.675803253	1.720429922	1.314466282	2.328247954
CPLX1	0.023466882	0.072984023	0.074513015	0.02371791	0.073510857	0.024099633	0.144793287	0.022962463	0.153227014	0.051464784	0.075833018	0
CPLX2	0	0	0	0	0	0	0	0	0	0	0	0
CPLX3	0.080232944	0.16635424	0.141532757	0.027030402	0.027925844	0.109861747	0.110010274	0.052338895	0.058209003	0.02932623	0.086424011	0.083497364
CPLX4	0	0	0	0.027662853	0	0	0	0	0	0	0	0
CPM	19.79763091	21.30361359	20.57310221	26.12945814	9.747286161	15.84620553	14.42810829	20.14696559	14.37188679	11.84999402	10.39123155	25.98075863
CPN1	0	0	0	0	0	0	0	0	0.033347527	0	0.033007804	0
CPN2	0	0	0	0	0	0	0	0	0	0	0.018359741	0
CPNE1	38.77835314	41.50177221	36.16776305	40.94188938	31.20986966	34.33788945	34.69568404	44.61108236	63.07722224	63.96093152	64.83417254	61.49075226
CPNE2	40.58558478	46.96035982	41.53125071	60.72767486	57.81867616	56.00121538	39.14011204	54.0410082	58.61058794	54.49599722	65.05799803	53.646845
CPNE3	1.894631497	1.638546975	1.458402986	1.167371346	2.359189624	2.060171492	2.073375712	1.57631625	11.73149793	13.65399662	10.55337365	12.49457637
CPNE4	0	0	0.012540522	0	0	0	0	0	0.058023155	0.038976797	0.031906696	0.024660971
CPNE5	0.917903805	0.634391073	0.464171644	0.113388335	0.564423868	0.450379022	0.66074973	0.159675294	0.310771645	0.257221495	0.307605706	0.244119536
CPNE6	0	0	0	0.021319157	0	0	0	0.010320057	0.034432531	0	0	0.021951744
CPNE7	0.037264239	0.051508884	0.013146995	0.012554286	0.038910525	0.012756339	0	0	1.405830733	2.710496421	3.023856193	0.840242019
CPNE8	10.86887067	8.520082896	9.506003544	5.985389061	6.019427565	5.556737137	7.634668003	5.071365064	11.99066328	10.81430655	9.691345949	10.86912272
CPNE9	0.082403615	0.192211752	0.021804281	0.062463823	0.043022051	0.042312755	0.04236996	0.120948534	0.179351476	0.203307569	0.022190545	0.150073619
CPO	0.08240011	0.061694975	0.077523034	0.083281555	0.035850184	0.044661564	0.077674956	0.062711387	0.067253943	0.030118359	0.054241247	0.114336941
CPOX	13.00687487	13.92350049	13.75515477	14.12345049	14.35304933	14.22800579	11.7777195	12.56780317	6.740348652	7.280224703	6.89407178	8.0063046
CPPED1	5.947695234	6.120543387	5.915004798	4.408890438	4.719581327	4.65976193	6.900006283	5.091297318	6.234266664	4.572046588	5.566889982	5.633618669
CPQ	19.57464271	18.71873555	17.64981011	17.44008742	12.56923766	13.5528472	14.70683271	18.53253403	13.91096615	11.68583299	10.88454774	19.42291206
CPS1	0.017456467	0.036194077	0.046190415	0.044108004	0.091138359	0.035854312	0.071805571	0.034162484	2.251144675	2.306578138	2.641887872	1.507837341
CPSF1	28.28366422	29.3101074	29.03681162	33.31342431	33.20757287	37.56533018	29.13093709	40.30464009	36.87801675	35.78002036	36.3240935	42.30339119
CPSF2	21.48317501	20.00643005	21.95290399	26.55064416	27.27102408	26.32046771	24.98704181	23.55708918	24.40756815	21.98595479	22.06813873	25.46654361
CPSF3	33.81719356	33.11216969	35.41416025	38.24427692	35.26452727	35.83056311	37.36502615	37.11360895	38.14717023	38.32343915	41.70610958	42.7123384
CPSF4	8.541966164	8.817893676	8.811080516	11.82816447	10.62006082	10.34584818	9.379683165	12.33684083	9.53212884	11.35106574	10.669632	10.91099621
CPSF4L	0.020421086	0.063511334	0.043227917	0.061918598	0	0.020971711	0.063000191	0.039964272	0.133339485	0.067177656	0.021996851	0.042503908
CPSF6	35.66782333	32.90682959	34.9417584	37.6251627	47.45405103	41.88463583	38.17551032	34.82290995	33.45564612	32.93899471	30.29855475	30.39583617
CPSF7	29.54065343	29.91180667	29.98190767	37.44686628	33.95704292	33.79874201	31.77855294	38.85313804	40.4816415	34.12229917	37.78911374	47.43980881
CPT1A	16.50684183	18.59154702	17.50978609	29.35542071	12.2705704	18.01318143	13.09084869	20.4025623	27.09961488	18.9828825	29.30592597	22.75550087
CPT1B	7.831672133	9.804794748	8.30671155	7.630358222	7.779177485	7.804283078	7.115252854	9.157031013	12.71199907	15.39244548	13.06506487	13.77956253
CPT1C	0.057504206	0	0	0.014529834	0	0.014763681	0	0.028134079	0.046934216	0.094583555	0.046456081	0
CPT2	15.18285282	14.42381944	15.64636849	14.20272018	14.52793759	14.21697641	16.2035107	15.31597786	34.86241419	30.30315153	34.03891905	35.94499454
CPTP	3.662801413	4.164089321	3.239997095	4.166966223	2.586699049	2.453193656	3.275346997	4.432472756	4.197862558	3.608948908	4.53629907	6.003170346
CPVL	10.42393477	10.99085505	9.9408491	13.77158319	7.57825251	9.188763854	9.43899062	12.32339612	24.58478892	23.07509913	19.23753735	26.91649551
CPXCR1	0	0	0	0	0	0	0	0	0	0	0	0
CPXM1	0.224206504	0.209190456	0.237303255	0	0.046822332	0.138151146	0.230563199	0.04387744	0.219593464	0.147510936	0.169054969	0.023332875
CPXM2	0	0	0	0	0	0	0	0	0	0	0	0
CPZ	1.080428191	0.4172828	0.941738903	0.085645935	1.039676983	0.543902151	0.806063469	0.145106495	2.144062168	1.788715285	2.94372432	0.352748969
CR1	0.100956024	0.649997666	0.101229393	0.477957935	0	0.141875375	0.142067183	0.348350568	0	0.029132208	0	0.016588989
CR1L	0	0	0	0	0	0	0	0	0	0	0	0
CR2	0	0	0	0	0	0	0	0	0	0	0	0.01321352
CRABP1	0	0	0	0	0	0	0	0	17.06684953	16.50899593	18.54474197	11.67852683
CRABP2	1.061423585	0.478422999	0.586134971	1.026135257	0.289125893	0.426538702	0.332200836	1.083765249	0.753322346	0.809665514	0.944487452	1.873033495
CRACR2A	10.81854354	8.543240557	8.949575142	8.871759494	8.686472951	9.114817064	7.35994884	5.808924047	1.710738412	1.830837963	2.050904049	1.879839645
CRACR2B	0.011862778	0.012298087	0	0	0.01238686	0.012182641	0	0.011607788	1.458792559	1.027633504	1.060586729	1.28392537
CRADD	1.629861054	1.46438013	2.006189514	1.720509036	2.269154982	1.909380888	2.172684402	1.665708525	0.919694412	0.86050804	0.806288002	0.716162718
CRAMP1	8.625799539	7.444668398	7.986601418	8.143953958	9.827014696	8.591910214	8.300635765	7.692419217	11.77575794	8.412718042	9.986364165	10.78670305
CRAT	4.069630653	5.011033824	3.977287828	5.610292675	4.477360076	4.515632952	4.361392542	6.301263227	15.86553763	16.08899341	15.21683672	21.15982732
CRB1	0.027717837	0.009578318	0.00977898	0.037352451	0.009647459	0.009488403	0.009501231	0.009040681	0.060327875	0.040524982	0.08956994	0.278840671
CRB2	0.006721154	0.006967789	0.014227525	0	0.014036173	0.006902381	0	0.006576684	0.080457261	0.036850096	0.036198916	0.006994616
CRB3	0.032010447	0.066370163	0	0.032352867	0.13369851	0	0.032918006	0.093967153	0	0	0.03448049	0.033312849
CRBN	6.207193214	6.449364263	6.217039095	8.491103743	7.786402804	7.857680689	6.426019496	7.908130138	10.10979974	9.455926831	8.196906737	11.95124886
CRCP	15.3193113	13.29425376	14.44646005	17.48164	16.11637035	14.78606633	15.8918338	16.3049382	13.83002718	12.81971601	14.12338337	16.50199445
CRCT1	0	0	0	0	0	0	0	0	0	0	0	0
CREB1	5.283930026	4.637427961	4.912405225	5.83714049	5.806831737	5.862068784	4.945653518	5.059382934	9.635532424	7.728463052	8.089573503	8.877820899
CREB3	9.157218623	10.33709008	9.322902612	10.92992043	8.833256835	7.702430205	8.908633186	9.472406878	9.775527516	10.26440224	10.17696039	11.04246249
CREB3L1	0.017125895	0.106526012	0.072505132	0.06923637	0.053647484	0.01758767	0.052834342	0.083788874	0.055911767	0.093896204	0.092236957	0.089113461
CREB3L2	7.516184466	8.71041917	6.981732523	8.549010878	5.037721563	7.046635656	5.91882039	6.206154723	13.71845074	13.07427045	12.09561559	16.30169287
CREB3L3	0	0	0	0	0	0	0	0	0	0	0	0.021389954
CREB3L4	3.411755135	3.893019557	2.714345322	2.198549369	4.231941854	4.13865562	3.343656953	3.047148192	7.326011735	5.47359728	6.141474132	5.123303511
CREB5	0.047869726	0.024813161	0.067554637	0.008063632	0.008330758	0.008193411	0.041022439	0.011710192	0.034729487	0.013122776	0.008593922	0.008302898
CREBBP	9.666472927	9.214210528	8.967841068	10.95237	10.69450639	9.515302527	8.863523734	9.41714657	17.92274903	11.63430106	13.22254541	17.79730039
CREBL2	7.80400922	7.636850649	6.796096379	6.874810193	6.247889488	6.072418605	6.690142248	6.075868991	5.69764316	5.04469605	5.289974528	5.610170301
CREBRF	1.722571216	1.77346584	1.068768387	0.924529856	1.04819174	0.896709064	1.032304158	0.900894568	4.395586136	5.073894207	3.01357351	3.535861589
CREBZF	10.03120884	9.965390231	9.851291901	11.04911599	13.48186862	12.02106218	10.30831802	10.71801204	21.46235394	21.04243014	20.21209047	20.52431625
CREG1	35.96157226	43.55897361	36.43178903	55.45891329	29.70190589	37.42218616	36.95384114	50.62581657	35.60896974	37.33643036	29.86772884	53.870609
CREG2	0	0.02037404	0	0	0.02052111	0.010091391	0	0.009615217	0.010693619	0.043100346	0.021169359	0.071583691
CRELD1	2.958072459	2.534836162	2.768915011	2.67864669	2.678112423	2.616399214	2.813354579	3.028338108	5.805585066	10.66841087	5.396497989	8.576888859
CRELD2	3.412673555	4.188111982	4.627291453	5.742417528	2.619610769	2.273313345	3.699128466	4.963851659	6.303494247	7.646093076	7.371886204	11.49925965
CREM	1.212238268	0.872542583	1.023780591	1.314080763	1.141181623	0.838550206	1.020538873	1.118575109	0.676697496	0.695627619	0.717163603	0.535999732
CRH	0	0	0	0	0	0	0	0	0	0	0	0
CRHBP	0	0	0	0	0	0	0	0	0.06371954	0.032102489	0	0
CRHR1	0	0	0	0	0	0	0	0	0	0	0	0
CRHR2	0	0	0.015800466	0	0	0	0	0	0	0	0	0
CRIM1	0.256530086	0.236394265	0.256430815	0.201657728	0.243061105	0.248811105	0.195409792	0.106914218	0.935732721	1.151228633	0.849443172	1.028319145
CRIP1	19.50596581	27.07854241	22.06920276	16.08894571	25.98639437	27.51510333	35.73729592	33.89532261	85.03146911	81.25394051	109.4027157	63.8154118
CRIP2	0.31396961	0.125188783	0.255622896	0.02440986	0.075655474	0	0.149017508	0.023632374	0.841052166	0.344280461	1.06662022	0.955097916
CRIP3	0.011537213	0.011960575	0	0.023321256	0	0	0	0.033867664	0.112998352	0.164463308	0.049709866	0.036019875
CRIPAK	2.385884198	2.460879553	2.307337527	1.982983865	2.111905328	2.11439972	1.992713676	2.192390668	2.069242935	2.417014659	1.65679414	2.230881183
CRIPT	2.037088308	2.199468198	1.923475963	1.973448625	2.241823257	2.361112901	1.964459306	2.109094678	3.210309726	2.40058639	2.704151113	2.990830287
CRISP1	0	0	0	0	0	0	0	0	0	0	0	0
CRISP2	0	0	0	0	0	0	0	0	0	0	0	0
CRISP3	0	0	0	0	0	0.024571124	0	0	0	0	0	0
CRISPLD1	5.410296087	7.214827517	5.316421025	6.618740257	6.31105808	7.243519215	5.913682063	5.799286544	9.680950677	9.432978495	8.92496462	12.3722758
CRISPLD2	0.022180363	0.01149714	0.011738001	0.056044073	0	0.011389212	0.03421383	0.075962592	0.156895577	0.048643342	0.071675643	0.62323589
CRK	9.343675684	8.008697161	9.53627966	10.91076096	8.780820623	8.139137308	10.02038389	9.534669811	18.21207189	13.88661089	15.31267334	19.3608596
CRKL	21.23077697	20.50131947	20.05379623	23.70478429	23.26608281	22.46739771	21.31313227	21.58522864	37.65323421	37.91792845	34.39605325	34.55631579
CRLF1	0.02158426	0	0.022845079	0	0.022537825	0	0	0	0.117445361	0.284016242	0.162748466	0.022462454
CRLF2	0.031578381	0.229160124	0	0.031916179	0.065946947	0.032429847	0.03247369	0	0.274921395	0.761793566	0.612271518	0.230042425
CRLF3	10.50210402	10.1477406	10.14731636	12.63008166	11.95951579	11.33017903	10.95038004	12.56795501	6.411342664	9.289025951	6.700774989	7.178369555
CRLS1	8.136478653	8.079502461	10.55191091	9.753471995	10.00248503	9.339624756	10.19140015	10.1834235	4.041163235	4.149758196	4.26751051	3.716849989
CRMP1	0.011730883	0	0.024832257	0	0	0	0	0	0.012766134	0.038590173	0	0

CRNDE	8.483079544	8.134202023	8.930465707	12.50445128	13.06117466	11.09413264	9.004243294	9.81400735	13.48869404	13.11762507	12.78783149	12.70980925
CRNKL1	12.26658016	11.19980327	12.14424818	13.12490473	11.07693515	11.68321048	11.97486638	11.96711695	7.789190323	5.963279166	6.934914918	7.347238257
CRNN	0	0	0	0	0	0	0	0	0	0	0	0
CROCC	0.233809389	0.24931451	0.205044154	0.209303566	0.132532486	0.109766274	0.13739334	0.209173644	2.377225333	1.933849141	1.770152588	3.13538217
CROCC2	0	0	0	0	0	0	0	0	0	0	0	0
CROT	0	0.016428839	0	0.008008418	0	0	0	0	1.603863321	1.598704886	1.169305463	1.805884089
CRP	0	0	0	0	0	0	0	0	0.05812234	0.029282568	0	0
CRTAC1	0	0	0	0	0	0	0	0	0	0	0	0
CRTAM	0.039755863	0.082429437	0.063117231	0.040181137	0.041512227	0.061241735	0.04088302	0	0.06489648	0.261563536	0.214117856	0.103433504
CRTAP	23.05777303	29.0117692	21.77581159	26.7305843	21.73637541	26.27177417	22.04754095	27.79498291	17.8729908	26.59676955	19.547151	22.64036069
CRTC1	3.949062396	3.96015222	4.027935488	4.334384383	3.838973625	3.780591124	3.60379192	4.593973844	2.591027278	1.965938728	1.91574889	3.248980981
CRTC2	27.23266536	29.91267731	26.37488315	24.87283777	25.6017031	25.591167	25.94421378	28.12671662	34.59179369	28.9628784	32.47346634	37.66750575
CRTC3	5.164938229	4.436799296	4.839181388	5.195564703	5.393118613	4.595308281	5.035784218	4.362580388	6.62823616	5.325175952	5.930883652	7.268193933
CRX	0	0	0	0	0	0	0	0	0	0	0	0
CRY1	7.027143435	6.442629268	7.119097663	10.06794788	6.143467076	7.232073437	6.824051743	8.319044883	0.655014061	0.709504501	0.729383855	1.190133197
CRY2	5.013848734	5.402171126	5.137224321	5.179539867	4.270608321	4.642991826	5.20667445	5.06278157	11.89129442	10.59052873	9.129499111	11.94848299
CRYAA	0	0	0	0	0	0	0	0	0	0	0	0
CRYAA2	0	0	0	0	0	0	0	0	0	0	0	0
CRYAB	0	0	0	0	0	0	0	0	0	0	0.049709866	0
CRYBA1	0	0	0	0	0	0	0	0	0	0	0	0
CRYBA2	0	0	0	0	0	0	0	0	0	0	0	0
CRYBA4	0.115223899	0.119452081	0	0.116456464	0.060157172	0.059165373	0	0	0.18808862	0.315869242	0.124114997	0.059955994
CRYBB1	1.877077704	1.097719747	1.528249946	0.583740629	1.45743932	1.383982862	2.029286121	1.224486705	2.356996036	1.477748195	1.866387595	1.00176921
CRYBB2	0	0	0	0	0.024567905	0	0	0.023022706	0.025604835	0	0	0
CRYBB3	0	0	0	0	0	0	0	0	0	0	0.008616278	0
CRYBG1	19.14156336	17.5007704	16.9227238	17.07486618	23.26589394	20.10610241	17.9740247	15.5759677	1.572268239	2.295396902	1.680751073	1.750800894
CRYBG2	0	0	0	0	0	0	0	0	0	0.021205525	0.0104154	0
CRYBG3	6.551033777	5.141561068	5.641249264	5.796633483	5.439960693	4.877434448	5.161939489	3.819687881	9.356685792	7.710300033	7.428499595	8.760244405
CRYGA	0	0	0	0	0	0	0	0	0	0	0	0
CRYGB	0	0	0	0	0	0	0	0	0	0	0	0
CRYGC	0	0	0	0	0	0	0	0	0	0	0	0
CRYGD	0	0	0	0	0	0	0	0	0	0	0	0
CRYGN	0	0.048656015	0	0	0.049007238	0	0	0	0	0.025732392	0.050555346	0
CRYGS	1.932225798	2.549437553	2.354957225	2.899753277	2.139867195	2.044456574	1.685946355	2.177161345	2.803659751	1.861944348	2.270534637	2.620187867
CRYL1	3.715084017	6.230944112	4.307774136	4.280978531	1.828418249	3.037624152	3.382404721	4.42247551	5.948530071	8.640482259	5.582063809	10.98307339
CRYM	0	0	0	0	0	0.063444843	0	0	0.100846597	0.033871627	0.066546158	0
CRYZ	6.192633391	6.855508585	6.928903932	6.057698599	7.159024897	6.90471821	7.619104496	5.540135492	3.995343474	7.954556016	5.765200282	3.153246667
CRYZL1	12.54822606	14.31281673	11.58361654	11.56995989	12.21594921	12.17603324	11.38397375	10.18592594	18.515498	24.34663255	15.88780772	18.29537373
CRYZL2P-SE(	0.639381806	0.652322811	0.644505258	0.5641615	0.529864189	0.479438154	0.563579605	0.466746064	1.148634239	0.979325715	1.049476339	0.771014639
CS	195.2404698	190.3708132	205.0950797	198.9554202	180.4170187	189.8092767	199.5673242	195.3430718	76.75932823	91.83745362	90.70149662	82.38010051
CSAD	3.510861067	3.124989243	2.877666894	3.452836934	3.801742965	3.909021893	2.868870742	3.921208854	6.303503067	5.664523636	4.520299824	7.713380837
CSAG1	0	0	0	0	0	0	0	0	0	0.067356592	0	0.063925684
CSAG2	0	0	0.058533177	0	0	0	0	0	0.060183204	0	0	0
CSAG3	0	0	0.059511788	0	0	0	0	0	0.122378803	0.030827782	0.030283022	0.029257523
CSDC2	0.021268601	0	0.022510981	0	0	0	0	0	0	0	0	0
CSDE1	151.135873	138.6907343	149.3343389	145.3129337	148.6676898	143.2953788	158.3845693	133.0545481	240.5328268	270.000077	243.2563872	212.2063535
CSE1L	44.15216073	42.43997259	54.01521679	65.47688062	62.60392247	58.79421947	58.97563945	61.92261492	57.17135097	62.73096641	67.54063784	54.4957705
CSF1	0.16078554	0.166685623	0.10210658	0.054168495	0.022385179	0.055040297	0.044091767	0.020977262	1.108174187	0.693477706	0.946784801	0.725085344
CSF1R	6.15074223	8.528007384	5.072232276	2.224732236	2.941992261	2.906405605	3.751098337	2.387720452	39.57267909	22.48179761	32.47643845	28.15652908
CSF2	0	0	0	0	0	0	0	0	0	0	0	0
CSF2RA	1.047651739	2.509255558	3.345665233	3.304369089	0.150887677	0.185500029	0.297201304	2.792602442	0.550396508	0.297101583	2.062417116	0.432351351
CSF2RB	9.374186221	8.719425178	7.641823603	6.684638506	3.673447909	4.791240725	7.035169201	6.386987684	7.798343628	6.931916037	7.462379872	10.7807174
CSF3	0	0	0	0	0	0	0.069516543	0	0	0	0.036408105	0
CSF3R	21.60822009	31.91974081	18.11153889	24.35420206	16.72841138	25.46344548	17.1482159	27.23771008	32.95287532	34.12935552	26.45737041	55.1811756
CSGALNACT1	0.044916161	0.062085834	0.015846628	0.055484776	0.0156335	0.035876758	0.015396541	0.034183871	0.369315663	0.519886437	0.295668123	0.644023693
CSGALNACT2	20.45708525	19.70992305	18.96520472	23.06422458	15.39673378	15.18449238	17.49618859	16.67452837	8.229014794	8.424965873	7.461569379	10.27231996
CSH1	0	0	0	0	0	0	0	0	0	0	0	0
CSH2	0	0	0	0	0	0	0	0	0	0	0	0
CSHL1	0	0	0	0	0	0	0	0	0	0	0	0
CSK	44.54614279	44.73321658	47.32848218	47.6902855	48.82561678	47.65340177	49.32885061	54.95232836	41.91733464	36.65366672	43.3979085	48.68005541
CSKMT	1.691157627	1.530078767	1.724855662	2.765874289	1.669550588	2.052531318	2.276646907	2.286641459	0.702699085	0.842919634	0.761782391	0.735985528
CSMD1	0.003388085	0.014049647	0.003585996	0	0	0.006958879	0.003484144	0.003315258	0	0.003715173	0	0
CSMD2	0	0	0	0	0	0	0	0	0	0.002967281	0	0
CSMD3	0	0	0	0	0	0	0	0	0	0.004156116	0	0.007888836
CSN1S1	0	0	0	0	0	0	0	0	0.117116514	0	0	0
CSN2	0	0	0	0	0	0	0	0	0.152495461	0	0	0.194440621
CSN3	0	0	0	0	0	0	0	0	0	0	0	0
CSNK1A1	58.98837012	55.8839144	57.60483461	52.8090689	55.58372029	50.04086606	59.19576099	47.71730499	71.52697303	71.9452264	71.15084267	66.51747554
CSNK1A1L	0	0	0	0.022613376	0	0.022977322	0.023008386	0.021893109	0	0.024534043	0	0.023284365
CSNK1D	12.10178058	13.07375911	12.46342102	16.00273333	13.40075502	13.7681314	13.36127889	17.01897107	25.12731953	20.13634423	22.02596162	31.16662062
CSNK1E	0.03722557	0.057887363	0.039400056	0.018811888	0.058305222	0	0	0.036425409	0.020255364	0.020409676	0.020049015	0.038740158
CSNK1G1	4.628011688	4.400822146	4.431187448	4.697199121	4.0665958	4.106205365	4.599026619	3.975962983	7.565246479	7.936052762	6.712091135	7.997912693
CSNK1G2	26.07028758	26.18077525	26.69057218	27.93750228	29.02836189	27.31120241	26.60898723	32.50725278	22.63050596	19.93084141	24.8013629	24.10095239
CSNK1G3	5.072169215	4.631259167	5.256143098	5.512517582	5.927812516	4.783936366	5.543337662	5.285020808	10.36981164	10.72806806	9.624088835	9.08836398
CSNK2A1	30.76265872	30.35675916	32.55962223	34.79433496	33.75923993	33.35726911	34.73123617	34.07892959	30.67857394	33.45736498	30.83903188	28.63268481
CSNK2A2	17.47223274	17.60374453	16.65007082	18.36301658	18.67189768	18.80580509	19.18931783	17.17684034	15.44761026	15.52037407	14.69451642	15.66775434
CSNK2A3	7.75222487	7.721530969	7.762631915	6.91336889	6.705910289	7.219763301	7.620309136	6.916274483	6.782171016	6.917179479	6.344678014	5.655256027
CSNK2B	164.1857096	171.1468681	182.6815803	161.2818118	177.8917723	173.6896745	196.1172204	188.471436	194.8148375	204.847342	223.3983331	188.8726111
CSNKA2IP	0	0	0	0	0	0.075699258	0	0	0	0	0	0
CSPG4	8.996387377	6.258049841	8.06544816	0.31489603	4.168260695	2.866344654	7.883092071	0.698651642	3.129228936	1.95732243	5.998931452	0.290464735
CSPG5	0.041551784	0.064614813	0.021989491	0.083992538	0.086774979	0.085344338	0.042729859	0.040658632	0.949593358	1.047954139	1.118951808	1.124302216
CSPP1	8.632280368	7.418974708	8.763138681	8.404451396	9.840583276	9.397383307	9.469317597	7.481590832	5.688158232	4.792033079	5.606945644	6.316172293
CSRNP1	5.304547097	6.595992544	4.790130159	3.727656286	3.544279215	3.636747819	4.246090162	3.594547264	3.438018915	2.964720066	3.434650381	3.165421098
CSRNP2	6.813667871	6.490965564	7.01676977	6.998228403	6.739723792	6.231458281	7.556034218	6.478004015	6.633395992	5.57330846	5.474822112	6.860919159
CSRNP3	0	0	0	0	0	0	0	0	0	0	0	0
CSRP1	33.94355259	36.07725254	33.45118179	40.55486109	29.54400513	27.74912012	31.3819928	41.98187443	39.58477704	34.04684423	43.79550414	47.20389106
CSRP2	0.116597098	0.302189175	0.123407977	0.058922176	0.060874105	0.059870486	0.059951428	0	1.395754773	2.173508914	1.193144496	0.970728474
CSRP3	1.029144607	0.685870349	1.050358672	0	1.036231942	1.585341024	0.944931145	0.07193023	0	0	0	0
CST1	0	0	0	0	0	0	0	0	0	0	0	0
CST11	0	0	0	0	0	0	0	0	0	0	0	0
CST2	0	0	0	0	0	0	0	0	0	0	0	0
CST3	37.91875368	44.26373485	37.02510752	41.07132488	26.64009516	37.16293975	36.67222735	54.49738936	56.91242418	51.26530773	48.73032845	65.00890496
CST4	0	0	0	0	0	0	0	0	0	0	0	0
CST5	0	0	0	0	0	0	0	0	0	0	0	0
CST6	0	0	0	0	0	0	0	0	0	0	0	0
CST7	24.41681149	41.20827054	25.59813181	26.66627622	10.93528016	24.65923689	18.68305668	19.13622556	1.637112563	2.15714917	2.866922977	0.782780306
CST8	0	0.060767017	0	0	0	0	0	0	0	0	0	0
CST9	0	0	0.033719823	0	0	0	0	0	0	0	0.034317172	0
CST9L	0	0	0.059387676	0	0	0	0	0	0	0	0	0
CSTA	126.2055159	138.7192108	127.9374073	102.7275407	89.23386498	98.7747327	137.1349813	109.8207817	68.31796657	73.82672926	78.40230447	80.68440376
CSTB	87.64089115	97.91893702	86.88328552	77.81707635	48.17719117	56.436213	69.63989745	73.43403089	72.26338106	92.71219246	94.34192191	78.93461804
CSTF1	14.08002895	13.05678121	14.39319901	14.23064482	14.85498564	15.89919881	13.70470526	15.59935124	15.43643013	15.23285559	16.78906269	16.08988579
CSTF2	13.72527867	12.67820948	14.78376104	16.87533781	14.1416811	15.96367116	16.75437465	15.7444519	14.80454809	15.29414662	14.87146642	16.429429
CSTF2T	18.55186076	17.84820439	18.15283297	20.45729213	16.8833811	18.13780015	17.65070683	18.63990528	13.86306183	12.63354001	12.00131626	15.77779194
CSTF3	20.79937031	17.92636259	20.505083	18.73579826	22.84781486	22.47112768	20.93398675	18.68433186	29.80531451	31.05682573	32.89145718	23.6583834
CSTL1	0	0	0	0	0	0	0	0	0	0	0	0
CT45A1	0	0	0	0	0	0.145167579	0.096909226	0.046105892	0	0	0	0
CT45A10	0.105993882	0.036627788	0	0	0	0	0.036333006	0	0	0	0	0
CT45A2	0	0	0	0	0	0	0	0	0	0	0	0
CT45A3	0	0	0	0	0	0	0	0	0	0	0	0
CT45A5	0	0	0	0	0	0	0	0	0	0	0	0
CT45A6	0	0	0	0	0	0	0	0	0	0	0	0
CT45A7	0	0	0	0	0	0	0	0	0	0	0	0
CT45A8	0	0	0	0	0	0	0	0	0	0	0	0
CT45A9	0	0	0	0	0	0	0	0	0	0	0	0
CT47A1	0	0	0	0	0	0	0	0	0	0	0	0
CT47A10	0	0	0	0	0	0	0	0	0	0	0	0
CT47A11	0	0	0	0	0	0	0	0	0	0	0	0
CT47A12	0	0	0	0	0	0	0	0	0	0	0	0
CT47A2	0	0	0	0	0	0	0	0	0	0	0	0
CT47A3	0	0	0	0	0	0	0	0	0	0	0	0
CT47A4	0	0	0	0	0	0	0	0	0	0	0	0
CT47A5	0	0	0	0	0	0	0	0	0	0	0	0
CT47A6	0	0	0	0	0	0	0	0	0	0	0	0
CT47A7	0	0	0	0	0	0	0	0	0	0	0	0
CT47A8	0	0	0	0	0	0	0	0	0	0	0	0
CT47A9	0	0	0	0	0	0	0	0	0	0	0	0
CT47B1	0	0	0	0	0	0	0	0	0	0	0	0
CT55	0	0	0	0.023152477	0	0	0.023556904	0.02241504	0.049858031	0	0.04935011	0.071518389
CT62	0	0	0	0.026009167	0	0	0	0	0.028004905	0	0	0
CT83	0	0	0	0.09474768	0	0	0	0	0	0	0	0
CTAG1A	0	0	0	0	0	0	0.073977497	0	0	0	0	0
CTAG1B	0	0	0	0	0	0	0	0	0	0	0	0
CTAG2	0	0	0	0	0	0	0	0.053024097	0	0	0	0
CTAGE1	0.0249407	0.025855908	0	0.012603747	0.026042549	0.012806595	0.012823909	0	0	0.027348493	0.013432608	0.012977729
CTAGE15	0	0	0	0	0	0	0	0	0.022635584	0.091232121	0.067214964	0.064938808
CTAGE4	0	0	0	0	0	0	0	0	0	0.022469297	0.110361203	0
CTAGE6	0.04042792	0	0	0.020430191	0.042213973	0	0	0.019779462	0	0.02216543	0	0

CTAGE8	0	0	0	0	0	0	0	0	0.056468907	0.094831846	0.018631213	0.036000578
CTAGE9	0	0	0	0	0	0	0	0	0	0.022896536	0	0.021730267
CTBP1	13.166454	13.32442233	12.95172726	10.26463515	13.40462758	13.01470813	11.34475184	11.40869592	14.59481405	15.88046927	15.43502822	16.6081898
CTBP2	9.62846431	9.012678945	10.19708321	10.67035827	11.84742269	11.04009161	10.15980118	10.37051162	6.300893892	5.958096433	7.029665905	7.22331073
CTBS	7.478852219	7.591389667	6.519908856	5.19123183	7.446879793	6.914239898	6.870054703	4.873070116	15.80563082	16.72519995	14.74743121	17.26376888
CTC1	5.353579826	5.439661386	4.386555372	5.134156674	4.938975233	4.935642992	4.269785424	4.73251306	2.987497287	2.376518752	2.244418712	3.553350138
CTCF	21.86474798	21.62347845	24.15059322	30.68576986	28.81518034	27.67847363	23.05056881	30.73281424	29.85609315	23.68122682	27.88928071	28.99818259
CTCFL	0	0	0	0	0	0	0	0	0	0	0	0
CTDNEP1	50.12556397	59.90402718	53.79044358	63.79629481	64.15290539	66.52108031	58.91480138	76.18353618	30.30508182	32.2217958	32.46480329	29.10131454
CTDP1	10.53091153	9.550917806	11.64943254	11.7834532	10.72657065	10.21481204	12.01723343	13.20312066	11.93346299	8.329152844	10.14329814	14.33911198
CTDSP1	15.45606747	17.12348691	16.33309189	19.56682443	22.87723653	20.59582722	15.61829155	21.59354961	23.8029823	17.33612994	22.18076531	23.02924563
CTDSP2	36.98226989	44.60434371	35.22070827	41.76791479	41.31562247	40.28001234	35.70702107	40.52038863	60.03939899	39.83134456	49.08822488	50.02152636
CTDSPL	0	0	0	0.012690507	0.013110909	0	0	0	2.104298585	2.850053784	2.860553103	2.142998345
CTDSPL2	15.22981426	14.87449609	15.19583427	18.2800268	19.99820334	19.49869714	14.76423137	14.66875709	28.43944031	28.01146553	27.76383415	24.0514219
CTF1	0.058648023	0.030400066	0	0.059275388	0.061239018	0.060229383	0.120621619	0.028693694	0.127647427	0.12861989	0.126347038	0.122068444
CTGF	0.527994838	0.356980301	0.29156714	0.092807455	0.215734295	0.282903372	0.141642921	0.06738856	10.04284102	3.398289478	7.888132879	2.38903152
CTH	1.238668863	1.538632971	1.145669802	1.285754712	1.596348281	1.157467092	0.940996219	1.157447201	1.068669975	1.370487335	1.081823586	1.056802114
CTHRC1	0	0	0	0	0	0	0	0	0	0.039179531	0	0
CTIF	3.679221106	4.17749156	3.42651926	3.233546264	2.879334811	3.864477142	3.039360863	3.451062544	3.382186988	3.282661257	2.683108309	5.049730827
CTLA4	0.027245347	0	0	0	0	0	0	0	0	0	0	0
CTNNA1	25.74572939	25.92351027	26.12206395	33.54832123	26.8110425	25.81185122	27.42920146	30.74236053	37.94746814	34.88342938	34.84077476	45.31239954
CTNNA2	0.172383664	0.241257614	0.209821235	0.095825221	0.089999677	0.150476982	0.159543973	0.118074803	0.056278959	0.037805141	0.055705626	0.04484935
CTNNA3	0	0	0	0	0	0	0.004060701	0	0.030080561	0.012989882	0.004253446	0.004109408
CTNNAL1	6.51357059	10.55898371	8.035743237	14.34057645	14.23081214	16.08390371	8.724781674	11.93520503	17.20177019	25.27134152	20.55696506	18.1122113
CTNNB1	31.90988394	32.08074053	31.86438498	37.07561668	32.16841393	30.51869796	34.29620882	34.05584049	120.3614187	95.14884674	108.3931091	111.3198133
CTNNBIP1	3.428437735	4.861187748	3.400894655	4.614970021	3.820702307	4.026119124	3.4940206	5.039637425	10.65760279	10.8062297	9.174509665	13.45573541
CTNNBL1	21.20411704	21.17798242	22.61689742	23.35544811	19.48987248	21.14817025	23.23158424	23.7386728	24.45683432	25.44225316	26.15746609	27.96275289
CTNND1	3.635323088	2.802383436	3.094284968	2.612201011	2.327106778	2.645540064	2.980256286	2.280244927	3.956140517	3.168581363	3.605491802	3.58922076
CTNND2	0	0	0	0	0	0	0	0	0.01811547	0.01825348	0	0.008661856
CTNS	4.251067178	4.059135674	4.588191841	3.900807311	2.51146813	3.188103379	3.968946553	3.844977354	1.582655596	1.303371055	1.61172097	1.80453832
CTPS1	22.25500967	18.84001999	24.56933969	25.63803902	24.21418762	24.05798328	24.35818076	25.95599501	103.6872568	110.7305046	127.2149793	112.6134408
CTPS2	3.186779818	3.704498955	3.162814651	4.065687361	4.560404238	4.528138547	4.512770973	4.498502614	5.50333911	6.70243864	5.548566972	5.686878464
CTR9	26.02706902	24.43436735	25.70183149	31.53346226	26.79809687	25.73132705	27.99251859	28.57091261	25.36930145	22.48274551	24.19062839	27.07384779
CTRB1	0	0	0	0	0	0	0	0	0	0	0	0
CTRB2	0	0	0	0	0	0	0	0	0	0	0	0
CTRC	0.030854106	0	0	0.031184156	0.016108601	0.063372086	0.01586444	0.030190899	0.016788491	0.084581959	0.049852383	0.208711492
CTRL	1.092579916	0.755115015	0.481834021	1.288311947	1.093313341	1.122039768	0.936297257	1.470005613	2.080750251	1.447653855	1.618220153	2.274066958
CTSA	27.41766639	29.32496478	28.90882696	33.000112	16.10264633	15.67647258	22.70619167	30.1627276	34.20786067	31.89477166	32.75444901	50.58898906
CTSB	68.09823511	70.04893556	60.82182306	58.55613111	35.27729143	36.07689839	47.41494517	53.37281455	46.82408806	34.33623493	35.66112082	66.63402404
CTSC	33.14933806	31.71494182	34.82477307	32.75640277	27.51601174	26.1436441	33.01710894	31.54764406	21.93155825	18.53010788	20.91143543	28.96343099
CTSD	30.82073764	35.89312776	28.54345705	46.57194372	18.71128904	21.55235972	24.89170293	42.58364481	72.81855801	75.59674905	65.0055659	145.4177717
CTSE	0	0	0	0.02424662	0.025049843	0	0	0	0.078321342	0	0.077523456	0
CTSF	0.026020097	0	0.02754003	0	0	0	0	0	4.389037643	2.682017016	3.05504144	3.791027968
CTSG	268.4907695	359.1319388	305.7527162	229.9265306	130.1718999	229.7942671	243.9673706	201.6727068	277.5152183	225.6947859	218.2864161	507.468282
CTSH	1.169310884	0.947734979	0.922585553	0.386777177	0.466188373	0.676836909	0.918244587	0.083212846	28.87424133	34.92238355	34.12206181	25.26698636
CTSK	4.875562421	6.379731045	4.027600004	4.266681735	3.197370309	2.992002729	3.85206141	3.636251956	16.11160885	12.74624882	12.29684763	9.838480569
CTSL	0.110085026	0	0.023303102	0	0.160927819	0.090442649	0.045282461	0.129262508	28.75200838	38.3383578	27.60532858	40.44110329
CTSO	7.320155057	5.078199964	5.359347985	4.876678944	2.605320359	3.410209001	3.867613166	3.536524678	12.17883969	8.067082889	8.616195975	13.40307773
CTSS	32.37482953	30.27582354	29.46789878	29.88734491	26.96473828	27.21984604	30.59804389	27.5379815	61.33859798	56.18848504	53.40323512	53.380981
CTSV	0.047160001	0.048890554	0.037436096	0.047664477	0.036932602	0.084755305	0.072745619	0.0922926	2.732889736	3.994818509	3.5686325	1.680948541
CTSW	0.249118337	0.172173215	0.043945047	0.041963865	0	0.127917728	0.170787555	0.121881779	0.316286882	0.318696466	0.402511831	0.216045134
CTSZ	94.16673151	86.16787331	92.71912796	88.79285193	60.3820796	57.83927997	84.66280685	97.4079174	74.05667577	65.57352803	77.93917085	94.11548265
CTTN	7.35673162	7.779458303	7.270567826	6.175185565	8.238047577	8.335051261	7.425426994	6.998946234	2.676878404	2.187649017	2.637397934	2.913783024
CTTNBP2	0	0.016233618	0.005524569	0	0.005450267	0	0	0.005107472	0.278334911	0.337691152	0.247387231	0.37481075
CTTNBP2NL	1.859157429	1.775218299	1.674320287	1.854320796	1.404882827	1.247735767	2.104732101	1.372375159	2.963851774	2.771837558	3.056625682	2.732481501
CTU1	2.426154856	2.356329773	3.567995791	2.503731065	2.933340223	2.806297611	3.440392497	3.248638198	1.167274227	1.372194752	1.898128881	2.391979542
CTU2	9.781065798	8.663012789	11.1933674	11.18717317	11.21447305	9.763431325	11.07266852	13.91388483	5.605372876	6.369107618	6.433631058	6.500890496
CTXN1	0.255627368	0.353343605	0.541119062	0.430603077	0.266920659	0.218766661	0.306687391	0.125066338	0.185457663	0.23358818	0.87194962	0.177352015
CTXN2	0	0	0	0	0	0	0	0	0.056705865	0.057137871	0.037418789	0.054227468
CTXN3	0	0	0	0	0	0	0	0	0	0	0	0
CTXND1	0	0	0	0	0	0	0	0	0	0	0	0
CUBN	0.018968401	0.023597344	0.024091701	0.034508356	0.023767681	0.038959714	0.015604954	0.025984948	0.103211818	0.108158047	0.081728291	0.118440987
CUEDC1	2.735417037	3.362441613	2.771122983	2.238072782	2.339416452	2.44799417	3.415751104	2.128549753	2.041246434	1.385481557	1.852080582	1.775806281
CUEDC2	34.23835771	47.43516034	39.57170674	39.28828233	39.13344754	51.51790243	40.48350493	47.59014675	35.54623297	46.96669263	39.7396296	38.01931928
CUL1	14.33716096	13.67152863	14.73718153	17.55834176	18.47717574	15.95735277	16.19144735	15.78576752	23.45984902	24.54502658	26.4208534	25.47237477
CUL2	11.02654936	9.591792907	11.00224184	11.92461689	11.03220674	10.50031068	11.62780726	10.04407575	13.7232279	13.86075187	13.98293708	14.44830009
CUL3	13.30333326	12.23851091	14.25397172	15.78073798	14.86382069	12.87369686	15.45859191	13.41847481	17.94056297	15.90339508	16.68205266	17.74591143
CUL4A	24.99744141	22.861763	24.19605244	24.89798288	25.45103937	26.54358796	24.95963281	22.75796673	26.43580728	25.17279659	27.06650003	26.69872407
CUL4B	8.736347688	9.084320964	7.89509096	9.470674204	10.00511242	9.433168295	7.824809337	8.643353046	14.29933217	14.78489315	12.34176374	11.91465933
CUL5	9.847553031	9.003522802	9.300976044	10.36060246	10.34867833	9.430750021	9.890866403	7.654526843	12.47257305	12.24668184	11.36570817	12.9152245
CUL7	11.02157789	11.51444005	9.869943079	8.524621355	8.945555823	9.547465631	10.12561455	8.522022038	7.373926347	10.77624801	7.441719262	7.623661289
CUL9	7.525604698	7.968279185	6.460315491	7.70832351	6.180858445	7.331404122	5.76905067	7.066960197	8.299799398	9.980839442	8.490796832	9.478641694
CUTA	42.67540219	47.68698235	44.35914591	50.99239403	47.89239167	50.65257598	41.59536708	58.70199853	48.06913134	53.39185202	56.35066186	48.66612147
CUTC	41.69454277	40.3293102	43.58886113	38.20478584	50.43668766	48.83764211	44.73735064	39.25876235	12.45647135	18.33103735	13.60066292	13.83598524
CUX1	4.054922836	4.262585587	3.620089885	4.199574782	3.278435178	3.639438022	3.173786165	3.738978895	6.401060874	5.200882159	5.612678939	8.283387705
CUX2	0.08224481	0.088969891	0.102188005	0.003614113	0.048539898	0.139546613	0.066190392	0.010496995	0.272400186	0.301922968	0.381326998	0.171182173
CUZD1	0.495831846	0.685368762	0.524795264	0.668181097	1.060128276	0.654687311	0.922657472	0.785519773	1.104872775	0.69904261	0.966452277	1.154869787
CWC15	22.10942534	23.81898367	23.78039508	26.02999186	24.52127914	23.65675378	28.42033886	29.1769142	30.79104355	34.03972512	34.82207849	31.12431857
CWC22	16.0339296	15.89060096	14.98928347	17.35300931	21.2282601	18.51469598	19.45487938	17.05552511	18.80142499	18.91101134	18.09753793	16.59049213
CWC25	12.76307986	11.68536226	12.22383253	15.94924377	15.21009051	14.51410691	13.60642381	16.44237433	16.00302997	12.47401533	12.72056725	16.3864003
CWC27	3.458672899	3.35151599	3.731314205	4.123231836	4.442004202	4.316070529	4.248026907	4.278113192	3.981693428	5.368126371	4.913976211	4.170812511
CWF19L1	19.99042246	20.86962852	22.68764283	29.06898419	26.34345613	27.61992334	24.16914643	30.42125549	15.42041383	16.9684344	14.98226805	16.54275564
CWF19L2	4.53367847	4.607583532	4.87717188	6.324904967	7.170801372	6.808332741	6.037953401	6.050723255	5.386712597	5.721142375	5.299813235	6.15678501
CWH43	0	0	0	0	0	0	0	0	0	0	0	0
CX3CL1	0.048608456	0	0.017149287	0	0	0	0	0	0	0	0.017453088	0.016862059
CX3CR1	12.86014662	19.03707516	12.16300853	13.86026173	13.05955123	14.12866523	14.08724126	12.26696607	15.83650977	7.179914297	12.56867132	10.9486499
CXADR	0.022781355	0.053138981	0.036168151	0.005756263	0.041628661	0.023395622	0.017570439	0.005572918	0.316095587	0.312258543	0.233122856	0.29635319
CXCL1	0	0	0	0	0	0.094543128	0.047335473	0	0.100185213	0.252371148	0.198329183	0.191612999
CXCL10	55.53536092	33.44294796	42.66774981	3.980493425	0.508268738	0.136333368	27.89511341	3.853709392	0.240782307	0.048523334	0	0.23025863
CXCL11	2.244857147	1.215718717	1.418500157	0.101591224	0.5247833	0.378496292	1.619397815	0.131140544	0	0.073479918	0	0
CXCL12	0	0	0	0	0	0	0	0	0.032671522	0.043893899	0.053897809	0
CXCL13	0	0	0	0	0	0	0	0	0.142477511	0	0.047008681	0.045416787
CXCL14	0.081736042	0.028245125	0.028836851	0	0	0	0	0	0	0.059751265	0	0
CXCL16	2.128188294	2.299179037	1.470055138	1.562271704	0.818708556	1.12729495	1.359190219	1.293306732	1.950316638	1.645833922	1.037615844	2.144837091
CXCL17	0	0	0	0	0	0	0	0	0	0	0.125730377	0.060736333
CXCL2	0.669828558	1.249934677	1.181592973	1.98584848	0.466280487	0.504452319	0.688819517	1.179775157	0.728940959	0.244831429	0.192403996	0.185888461
CXCL3	0	0	0	0	0	0	0	0	0.050524812	0.050909728	0.050010098	0
CXCL5	0	0	0	0	0	0	0	0	0	0.071146754	0	0
CXCL6	0	0	0	0	0	0	0	0	0.035297322	0.248963605	0.034937736	0.101263832
CXCL8	78.67271034	89.01104657	87.56046107	81.7190865	1.255734065	1.780742428	29.62329673	28.9536373	2.647904555	0.736021302	0.48201001	0.261949111
CXCL9	0.258317685	0	0.105156539	0	0.020748449	0.020406373	0.081735846	0	0.021624172	0	0	0
CXCR1	0	0.044878618	0.022909405	0.043753153	0	0.066685992	0.089034864	0.063539334	0.023555212	0.047469328	0.093260987	0.135154221
CXCR2	0.325679925	0.618989918	0.42130504	0.658327528	0.170034024	0.297299037	0.446551456	0.336383873	8.171377499	5.059218379	7.308553757	7.494136428
CXCR3	0.46246842	0.535843434	0.604655413	0.164970178	0.198841047	0.111750169	0.783308747	0.21295421	1.746682086	0.656267055	1.640978466	0.651149986
CXCR4	43.13085935	56.81449852	44.89190451	53.0769343	31.71368273	35.81960136	39.62359315	45.39279517	46.08192738	26.58745219	32.41672602	41.90295609
CXCR5	0	0	0	0.017504077	0	0	0	0.016946549	0	0	0	0
CXCR6	0	0	0	0	0	0	0	0	0	0	0	0
CXorf21	48.36437956	30.19348154	44.80561311	22.18305268	29.88659502	25.63718213	30.58222823	19.78241832	82.16996736	36.22323552	61.84185922	57.68438723
CXorf36	0	0	0	0	0	0	0	0	0.026633531	0	0.017574804	0
CXorf38	4.627738412	3.904286315	4.631640365	4.051000324	3.790940894	3.827865518	4.898327203	3.656716505	7.301344394	8.174409544	7.925673764	8.00991806
CXorf40A	3.281135532	2.983225847	2.888379831	3.316234257	2.2729736	2.644432403	2.435074975	3.065143336	1.565790593	1.391420372	1.246734228	1.696266595
CXorf40B	4.226887666	3.465759525	4.080644411	3.948459039	3.096225633	3.72261599	3.615687594	3.785095062	2.543890006	2.5211343	2.483481709	2.805943396
CXorf49	0	0	0	0	0	0	0	0	0	0	0	0
CXorf49B	0	0	0	0	0	0	0	0	0	0	0	0
CXorf51A	0	0	0	0	0	0	0	0	0	0	0	0
CXorf51B	0	0	0	0	0	0	0	0	0	0	0	0
CXorf56	10.96980772	10.75328939	11.68082116	12.89775679	11.03875452	11.56086042	12.37251596	12.53022832	10.10870036	8.997378908	9.410140956	9.989117179
CXorf57	5.678672919	7.087326937	5.339323247	8.595196984	8.851147838	9.030781907	5.400281496	7.72800408	4.304871869	4.307440238	4.09770241	4.647449596
CXorf58	0	0	0.034854823	0	0	0.03381913	0	0	0.035837367	0	0	0.034271052
CXorf65	0.157614414	0.098038878	0	0.063720174	0.131662094	0.032372852	0.097249856	0.092535902	0.171523893	0.138264498	0.067910608	0.098416342
CXorf66	0	0	0	0	0	0	0	0	0	0	0	0
CXorf67	0	0	0	0	0	0	0	0	0	0	0	0
CXXC1	23.14939496	26.74055375	25.69593049	32.39588352	28.1486288	29.67624233	24.65787297	36.26358301	21.96893982	21.18898895	21.72614318	28.82512126
CXXC4	0	0	0	0	0	0	0	0	0.02009549	0.020248584	0.009945385	0.019217192
CXXC5	25.31955605	26.06606668	26.78303191	23.78053062	23.40350287	20.80180928	24.89025964	23.59758899	17.98597857	14.4211455	16.53790017	19.73556391
CYB561	4.074210863	4.153084776	3.7930062	3.126217592	4.652591327	4.058124331	4.049598257	3.519972924	2.342923428	1.972291065	2.304377684	1.871829488
CYB561A3	23.59396819	17.71274483	22.85614912	19.87674014	16.5081108	14.93998168	18.48879918	19.70149097	13.62501739	8.566906526	12.90583388	16.65948795
CYB561D1	3.042745414	3.144449243	2.80395427	3.376032585	2.986740277	3.085359174	2.92172845	2.958557335	8.743000564	7.125572857	5.976072907	11.24772745
CYB561D2	13.69697913	11.68423785	12.75742301	15.38605861	11.44167536	10.24830323	11.18776485	15.16404336	9.922962833	7.080524922	8.641563091	10.01871208
CYB5A	0	0.031822089	0.032488751	0	0	0	0	0	11.39096729	16.02172401	12.53136662	13.44868021
CYB5B	23.48989463	24.85946627	24.75572365	27.56056607	26.55960197	26.93399392	26.95749662	27.19868953	29.70267169	32.53087648	33.98454533	25.46473491
CYB5D1	1.255418765	1.301486799	1.372461499	1.67788471	1.828334813	1.687924982	1.715687477	1.907304848	1.698773689	1.204540566	1.467947982	1.779243601
CYB5D2	2.688451572	1.848733132	1.75877281	1.856987931	1.495305211	1.33191163	1.611569037	2.022570433	1.573119133	1.185124265	1.164181846	1.673077821
CYB5R1	9.530482173	12.05317298	9.393915845	8.209082053	6.907932637	7.634889893	9.733331416	7.370769029	11.72590784	10.91742532	9.278105942	18.6435774
CYB5R2	0.07750747	0.020087908	0	0	0	0.019899337	0.01992624	0	0.442824414	0.446198006	0.313080862	0.262148248
CYB5R3	24.70925243	23.71261357	25.37532224	25.12820557	24.42695909	21.88735251	26.36955967	28.89398683	46.27051361	49.54220001	52.95165023	43.9456812
CYB5R4	26.48570285	25.22326108	26.46137235	29.69764213	27.33073916	25.89642317	28.81270424	27.81443076	22.12548298	17.93501899	19.96544665	21.43260036
CYB5RL	3.233192498	2.873001976	2.947568838	3.185397403	3.716470902	4.255097163	3.045459883	3.070645403	7.864931269	8.833525369	7.623844384	9.118730056

CYBA	83.34302945	74.19787615	90.76087453	83.32425987	64.86961648	58.6160956	81.50575136	91.16396465	79.18699301	61.69161133	77.09425512	97.69835134
CYBB	0.648007681	1.059355715	0.329740512	0.365254722	0.117110048	0.243156256	0.384449985	0.256070286	2.902146135	2.391330595	3.79878697	1.85452583
CYBC1	30.04332198	32.95741562	29.28281481	31.82005708	30.83048031	35.26102526	28.32171492	35.27978752	38.62806305	35.44248207	34.49993845	53.14730513
CYBRD1	6.552004685	8.984766534	5.110483556	7.091569009	5.782058105	6.866758482	5.405298089	7.069032403	4.855466567	6.903056063	5.543361481	4.337942868
CYC1	73.08429639	77.28377289	83.52761578	78.30477903	79.3933824	81.11397072	79.14799363	93.10843467	66.4513115	76.4430126	78.12645135	68.16354009
CYCS	46.72628714	42.14531693	54.15553383	58.70612818	53.05178224	51.77799197	62.22958208	57.72997125	32.38785507	43.76850304	41.55073375	33.78337324
CYFIP1	21.95792276	20.73440999	21.50703892	16.81620098	17.08823943	17.33299461	19.7801658	16.95812576	17.85295043	18.24448546	16.27259411	21.27847294
CYFIP2	4.151902771	4.187506275	3.504419779	1.153417836	0.548775764	0.747909095	2.571035427	1.013827837	1.250092553	1.901773472	1.083698659	2.320590617
CYGB	0.655381609	0.537882929	0.245672997	0.110398718	0.042770971	0.042065815	0.308899694	0.160323557	0.029717461	0.044915789	0.029414719	0.07104656
CYHR1	11.68833216	10.58057516	10.45904736	10.61174021	8.415927944	8.150323014	9.733273432	11.37665854	15.27387537	11.00512004	13.53825733	18.23780493
CYLC1	0	0	0	0	0	0	0	0	0	0	0	0
CYLC2	0	0	0	0	0	0	0	0	0	0	0	0
CYLD	11.36846304	8.985564388	10.1472556	7.065835946	5.391355516	4.848142784	9.667492008	5.086441475	5.97365171	7.212780508	4.721458552	6.736345252
CYP11A1	0	0	0	0	0	0	0	0	0	0.024301637	0	0
CYP11B1	0	0	0	0	0	0	0	0	0	0	0	0
CYP11B2	0	0	0	0	0	0	0	0	0	0	0	0
CYP17A1	0	0	0	0	0	0	0	0	0	0	0	0
CYP19A1	0.009135749	0.05682593	0.009669403	0.036933901	0.009539355	0	0.018789531	0.008939377	0.308201353	0.040070882	0.246017418	0.085567078
CYP1A1	0.020040805	0.083104837	0	0	0	0.041162353	0.041218002	0	0.043618813	0.043951117	0.021587227	0.104281003
CYP1A2	0	0	0	0	0	0	0	0	0	0	0	0
CYP1B1	4.844322126	3.46350775	3.900724059	2.258134668	0.970241575	1.415285318	3.285324286	1.675413319	6.430728269	4.831169184	4.498386004	11.15849226
CYP20A1	1.727340277	1.438616664	1.797427724	2.393143578	1.585795128	1.544701727	1.641593348	1.908609304	1.568241871	1.074741535	1.196864755	1.676432319
CYP21A2	0	0	0	0	0	0.025931703	0.025966761	0	0.274792384	0.027688585	0.135996489	0.131391128
CYP24A1	0	0	0	0	0	0	0	0	0	0	0	0.013229128
CYP26A1	0.021706156	0.022502671	0.068922285	0.02193835	0	0	0	0	0.047243451	0.047603368	0.09352433	0.02258931
CYP26B1	0.180493286	0.584739221	0.226855942	0.296439074	0.141350437	0.57925009	0.185610626	0.176613596	2.074705544	1.23698898	2.466714013	1.737492039
CYP26C1	0.0685909	0	0	0.034662313	0.035810579	0	0.035267793	0	0.037322025	0.037606357	0	0
CYP27A1	0.512693954	0.990536591	0.715301281	0.683053222	0.535344119	0.622248559	0.383439881	0.684100411	0.228247862	0.102216326	0.100410054	0.218272018
CYP27B1	2.466541994	2.512192051	2.290019353	3.192695865	3.388829833	2.488609312	2.358475189	2.836949093	1.153741293	1.32860674	1.491575808	1.148348951
CYP27C1	0	0	0	0	0	0	0	0	0.011739827	0	0	0
CYP2A13	0	0	0	0	0	0	0	0	0	0	0	0
CYP2A6	0	0	0	0	0	0	0	0	0	0	0	0
CYP2A7	0	0	0	0	0	0	0	0	0	0	0	0
CYP2B6	0	0	0	0	0	0	0	0	0	0	0	0
CYP2C18	0	0	0	0	0.022077328	0	0	0	0	0	0.022774736	0
CYP2C19	0	0	0	0	0	0	0.013382145	0	0	0	0	0
CYP2C8	0.717815288	0.826839748	0.703468149	0.886714705	1.082650764	1.092103924	0.683487746	0.572314438	0.144659726	0.058304718	0.143186027	0.110669761
CYP2C9	0	0	0	0	0	0	0	0	0.031466017	0	0	0.030090757
CYP2D6	0.227044561	0.26900119	0.137318339	0.229473285	0.203207228	0.29978549	0.233481721	0.158688753	0.282378575	0.391228522	0.489128301	0.168773053
CYP2D7	0.229074333	0.451212564	0.242455433	0.300982202	0.215275091	0.164675696	0.212012137	0.134490238	0.822657507	0.3014272	0.56752626	0.452949799
CYP2E1	9.26415355	8.834437989	8.097066088	5.350430504	7.549995737	8.254261658	6.904448062	6.190746222	1.545628861	0.672515364	0.695401364	1.209334348
CYP2F1	0.019688826	0.040822628	0.020838925	0	0.020558653	0.06065912	0.040494086	0.019265616	0	0	0.021208088	0
CYP2J2	0	0	0	0	0	0	0	0	0.123021548	0.12395877	0.24353657	0.205878305
CYP2R1	1.660025836	1.926904762	1.672882812	1.713480862	2.083724399	1.872544256	1.784273136	1.455861244	0.557331623	0.997284306	0.722856823	0.601891676
CYP2S1	5.618833718	7.929678884	5.447846077	5.222965887	4.860671985	5.959874152	5.778138719	6.802340969	12.54182183	15.47065913	14.26953528	15.04543585
CYP2U1	0.011159179	0.023137338	0.011811029	0	0.023304354	0	0.011475564	0	2.5866671	1.994548531	1.995363502	2.891689301
CYP2W1	0.017841366	0.129472431	0.037767096	0.018032218	0.149036601	0.183224332	0	0.087289337	0.019415868	0.019563785	0.153744572	0
CYP39A1	0	0	0	0.009534567	0	0	0	0	0	0	0	0
CYP3A4	0	0	0	0	0	0	0	0	0	0	0	0
CYP3A43	0	0	0	0	0	0	0	0	0	0.053285106	0	0.016856983
CYP3A5	0	0	0.019557961	0.018676225	0	0	0	0	0	0	0.019904431	0.019230391
CYP3A7	0	0	0	0	0	0	0	0	0	0	0	0
CYP3A7-CYP:	0	0	0	0	0	0	0	0	0	0	0	0
CYP46A1	0.274438562	0.284509182	0.186730431	0.336811609	0.020468779	0.040262629	0	0.095906973	0.341323174	0.408409148	0.358961371	0.95881539
CYP4A11	0	0	0	0	0	0	0	0	0	0	0	0
CYP4A22	0	0	0	0	0	0	0	0	0	0	0	0
CYP4B1	0	0	0	0	0	0	0	0	0	0	0	0
CYP4F11	0	0.032527185	0	0	0	0	0	0	0	0	0.016898456	0
CYP4F12	0	0	0	0	0	0	0	0	0	0	0	0
CYP4F2	0.044729477	0	0	0	0	0	0.022998823	0	0.024338428	0	0.024090484	0
CYP4F22	0	0	0	0	0	0	0	0	0	0.06057944	0.019836312	0
CYP4F3	0.011318797	0	0.023959942	0	0.011818847	0.023247984	0.011639707	0.0110755	0	0	0	0
CYP4F8	0	0	0	0	0	0	0	0	0	0	0	0
CYP4V2	9.105529431	9.878438217	8.475116265	9.734760196	11.76530541	14.09705576	8.634356552	9.234410261	6.323893407	4.139337486	4.460488249	7.690324968
CYP4X1	0.036957116	0	0.019557961	0	0.019294917	0	0	0	0	0	0	0
CYP4Z1	0	0	0	0	0	0	0	0	0	0	0	0
CYP51A1	17.8546931	27.07769411	17.40801253	23.58675499	20.95043039	27.98317017	18.73115628	19.47261583	40.96228444	57.53285406	60.20049209	15.92426107
CYP7A1	0.056149107	0.038806346	0.019809663	0.01891658	0	0.019221029	0.019247015	0.018314062	0.142576626	0.020523261	0.040321185	0.155823023
CYP7B1	0	0	0	0	0	0	0	0	0.492068293	0.493670648	0.527116257	0.499080764
CYP8B1	0	0	0	0	0	0	0	0	0	0	0	0
CYR61	0	0	0	0	0.073446796	0.024078631	0	0	1.250263441	1.208368451	1.035481416	1.244419974
CYREN	7.412489383	7.255221604	7.282770628	8.54063117	8.215037345	8.242344666	7.349284124	8.463685609	10.18622161	8.61017661	10.03289227	13.00753445
CYS1	0	0.041032822	0.020946223	0.040003802	0.041329017	0.060971451	0	0.058094441	0.258440267	0.434015258	0.277124732	0.370717241
CYSLTR1	10.43791345	12.65305258	9.333007952	9.00527595	12.8597223	14.34919194	10.12805844	7.529566897	15.54608535	11.6676457	14.83248523	9.981329557
CYSLTR2	0.010530247	0	0	0	0	0	0	0	0.034378624	0.011546844	0.011342799	0
CYSRT1	0.290413975	0.258060702	0.263466991	0.419315101	0.129961755	0.17042547	0.554631598	0.324767484	0.496639034	0.272957784	0.580957708	0.56128426
CYSTM1	20.16380248	22.62015484	19.65183882	21.57477567	15.86813983	19.37152339	18.97205756	20.94545061	15.44393593	21.72139056	17.26112274	18.33810094
CYTH1	9.583838706	9.209501995	8.123056702	9.133683156	7.753357486	6.857065281	9.184710518	8.232593714	10.65929407	9.29931662	9.041984436	12.57953513
CYTH2	12.03987584	12.38165204	10.07652317	13.59900111	10.43357169	10.3936786	9.768272313	14.35130623	16.26423796	12.13239975	13.40775811	16.21167563
CYTH3	5.622972683	6.291160737	5.199537877	6.121221706	5.318195529	6.207373059	5.708103019	5.596361816	8.372952845	7.538934382	7.496501391	9.385360311
CYTH4	74.84164475	58.88504348	67.87591369	33.30221034	63.31643897	37.52114372	61.5676213	36.50153529	56.99034098	32.07804752	49.51036095	47.14924748
CYTIP	3.863250523	2.524900046	3.542882048	0.509292549	1.553436573	1.133547868	3.38056544	0.821784834	0.221959949	0.565705261	0.426474074	0.099886553
CYTL1	0	0.049985772	0.051032958	0	0	0	0	0	0	0	0	0
CYYR1	0	0	0	0	0	0	0	0	0	0	0	0
D2HGDH	11.66765418	13.12848802	10.3186647	8.995832653	11.73046293	12.65477762	8.783058871	10.14430775	7.016150443	8.404634056	6.835387747	9.013192665
DAAM1	1.921137662	2.074236838	1.995877331	3.122807476	2.218629758	2.191143553	1.920980655	2.47758103	3.50694399	3.989930546	3.137446611	4.717248471
DAAM2	0	0	0	0	0	0.004306122	0	0	0	0	0	0
DAB1	0	0	0	0.006994877	0	0	0	0	0	0.015177974	0	0.007202431
DAB2	7.075588855	8.171810046	6.288201022	7.174917918	5.263314542	5.873150237	6.446348616	5.790563298	23.86366703	18.73622928	18.39254235	22.69896682
DAB2IP	0.103410754	0.125849873	0.147521409	0.109061167	0.20187436	0.235484909	0.106342652	0.136383755	3.855607166	3.081361461	3.457942548	3.76195811
DACH1	15.49358174	8.572703317	14.20677553	8.156095105	16.43100573	7.965427561	15.3223075	7.747588624	19.00080792	8.921748897	14.18728963	10.68175659
DACH2	0	0	0	0.007961524	0	0	0	0	0.017144856	0.034550944	0.042425489	0.01639552
DACT1	0	0	0	0	0	0.012827404	0	0	0.203893653	0.246536383	0.12108991	0.623943163
DACT2	0	0	0	0	0	0	0	0	0	0	0	0
DACT3	0.307849693	0.478719519	0.108610787	0.2333571	0.133937541	0.144902275	0.131907432	0.075308122	0.795666425	0.8298589	0.759926985	0.574005399
DAD1	109.7807684	108.2868245	127.9599075	108.5362547	119.1450335	107.5874351	122.5789085	118.8279143	93.50837893	103.5327831	108.9380657	97.29507388
DAG1	3.134062092	3.105228676	2.945684578	4.79262596	3.008333068	3.470018178	2.870412167	4.120872291	11.06682668	9.45072339	10.1364848	17.20821615
DAGLA	0.10873998	0.147416457	0.115091894	0.092995003	0.087341518	0.077311383	0.051610603	0.130957073	2.940201622	2.228829934	2.784107948	3.656076094
DAGLB	12.99935439	14.08980393	14.32626664	12.61511643	11.21805155	12.51431003	13.71019108	13.93221815	13.30137729	13.50409392	13.40488885	13.4897691
DALRD3	14.26768659	14.06128687	13.31643888	12.64118072	13.05994803	11.17007203	12.728613	13.59838025	10.8889321	8.533690505	9.760080265	13.61405736
DAND5	0.031067876	0.032207922	0	0.031400213	0.06488083	0	0.031948711	0	0	0	0.033465187	0.096995783
DAO	0	0	0	0	0	0	0	0.02864686	0	0	0	0
DAOA	0	0	0	0	0	0	0	0	0	0	0	0
DAP	43.63123658	45.75631844	43.62651138	55.52303079	41.86420308	43.69870592	44.11219403	57.10437145	139.2196308	134.0573339	132.0843776	174.5243222
DAP3	89.64685707	82.41998371	88.86813025	73.28667	80.08130407	80.7432607	88.68182943	75.00261691	72.49180807	73.42034943	82.10278789	74.14441687
DAPK1	0.035873041	0.05206518	0.053155929	0.029005423	0	0.007368061	0.044268134	0.00702039	7.682843327	6.175791182	6.043473635	10.04246919
DAPK2	0.111819651	0.080642026	0.113205745	0.00491373	0.04061207	0.039942507	0.124989085	0.009514443	0.354481679	0.170594505	0.246133003	0.354167232
DAPK3	16.2127508	16.05906534	17.30772834	17.72815236	15.00746959	15.3341792	17.10359734	20.58250834	9.328761298	8.173766143	10.13702523	11.29674756
DAPL1	0	0	0	0	0	0	0	0	0	0	0	0
DAPP1	11.16831928	12.68860145	9.9700712	8.979284277	4.901541567	6.632549184	9.314321291	6.704925006	8.588315805	8.083737469	6.787084738	9.294899192
DARS	38.75924656	43.43280682	35.75559472	35.3667251	49.47713025	52.07471151	43.20595148	39.16030273	48.11533697	65.63932934	58.09022185	37.24982184
DARS2	27.07008382	26.06183715	30.84884001	34.71080925	37.71102164	36.05714365	33.12766502	34.34280456	16.34853281	18.40170829	21.69468535	12.1934196
DAW1	0	0	0	0	0	0	0	0	0.02661739	0	0.026346229	0
DAXX	35.61925425	38.54757473	38.07015744	40.49251368	41.06041001	39.4959071	40.39573059	46.14933527	44.9655756	45.08250094	44.99512751	45.86984371
DAZ1	0	0	0	0	0	0	0	0	0	0	0	0
DAZ2	0	0	0	0	0	0	0	0	0	0	0	0
DAZ3	0	0	0	0	0	0	0	0	0	0	0	0
DAZ4	0	0	0	0	0	0	0	0	0	0	0	0
DAZAP1	42.03406139	42.57803621	47.09860308	53.05248281	59.58697992	54.71880481	51.75070356	62.54849252	32.79162573	35.84518885	39.08987686	26.59738877
DAZAP2	114.765704	120.6445322	114.5246146	145.0148008	142.0701525	141.8677126	115.2001508	143.0249551	122.7654764	142.0394025	120.9635555	109.9054475
DAZL	0	0	0	0	0	0	0	0	0	0	0	0
DBF4	8.759375661	6.924826983	9.664626057	14.36536831	14.00715174	13.11145254	11.14634684	12.30410124	11.67568013	13.36546488	14.46446406	9.689085133
DBF4B	2.628246011	2.580626335	2.871300113	4.555505926	4.264796303	4.144844635	3.740374025	4.646889924	4.83273287	4.710544595	4.705402172	4.357426105
DBH	0	0	0	0	0	0	0	0	0.02121676	0	0.021000617	0.040578912
DBI	108.0669606	122.24651	103.8057416	112.1824669	108.0930409	121.2758335	107.9425458	120.6095887	131.3249517	122.9194841	139.2268323	101.2067167
DBN1	14.85867982	16.89015742	14.78792018	9.013030876	14.70320605	16.03289439	19.16553342	12.0427673	7.483398848	6.781040087	7.917550398	8.218397888
DBNDD1	1.268111353	3.237558945	1.04169702	1.587748503	1.146266029	2.740669947	2.179929231	1.555696792	1.050464689	2.365986168	1.834876303	0.571216344
DBNDD2	2.935497381	2.675085466	3.106970911	2.153394269	4.548337417	4.132986342	3.968162061	3.26619573	4.766070196	3.660192162	4.615516217	3.720120424
DBNL	42.82483477	39.73030614	41.4352119	38.23851606	29.73363505	33.17915732	36.20271853	38.17609834	50.77473718	38.31008653	50.32947933	59.15246001
DBP	2.020458387	4.127593764	1.792550267	2.282315192	2.575098999	3.387029763	3.055503472	3.110912938	7.275321833	11.43596654	6.49708771	7.20471752
DBR1	9.881755786	11.57157617	11.22117996	15.83032974	15.8116753	15.05796139	11.99271482	15.24781828	12.84363259	10.50672688	12.32494219	14.36402981
DBT	3.226727031	3.063013275	2.993454233	2.386994666	2.806041691	2.984354215	3.263240732	2.239639595	6.515286977	5.840223452	5.375839398	6.114211929
DBX1	0	0	0	0	0	0	0	0	0	0	0	0
DBX2	0	0	0	0	0	0	0	0	0.021512953	0.021676846	0.042587585	0.082290814
DCAF1	7.638865111	6.834517105	8.233921863	9.107781737	10.06733983	8.982857315	7.751319774	8.658065676	6.415330692	7.34008002	6.531749587	6.22275031
DCAF10	5.943696289	5.096124759	5.300528262	5.940677246	6.075550232	6.313740858	6.003791176	5.119572017	12.42014465	12.23296666	10.3629791	13.26939374
DCAF11	18.07655558	15.90276768	15.1179589	20.47784176	16.406094	16.85055696	14.9848079	22.30379453	20.80823188	19.91381182	18.54050481	23.6462002
DCAF12	31.01122727	30.28402931	30.45331211	34.35510268	28.03603658	31.77687931	29.95556178	32.06480015	93.8307141	81.72964257	88.6882117	101.9643043

DCAF12L1	0	0	0	0	0	0	0	0	4.923039311	4.229517099	4.530929637	4.245344248
DCAF12L2	0	0	0	0	0	0	0	0	3.492316216	4.358922614	3.322929529	3.792153211
DCAF13	40.89689561	36.52783139	43.26428163	41.70490219	46.02128397	43.1290937	48.38161915	43.1068448	25.99880225	27.64852969	28.14718614	25.85868909
DCAF15	17.92870821	22.40111507	19.12482334	23.28424248	23.61509611	25.10307417	20.89529205	26.9915055	24.25474846	24.2082407	24.66401763	23.0239375
DCAF16	10.80819167	10.16528432	10.88848193	11.37206446	14.57776105	13.85224539	11.36249819	10.11358881	9.590081877	9.93802399	8.796553127	10.66597912
DCAF17	4.087607127	3.080803541	3.628773967	3.371681775	3.682598814	3.770322466	3.870548435	2.84564553	3.290638062	4.00040402	3.238431923	3.730452055
DCAF4	3.490021236	2.596955722	3.513697016	2.101664142	3.022024424	3.634077614	3.576465088	2.641570641	2.474665327	2.61352709	3.392560734	3.657329212
DCAF4L1	0.293178664	0.175348245	0.274499994	0.205141038	0.294356657	0.231602928	0.255107647	0.28687681	0.184068285	0.197835287	0.255070364	0.082144236
DCAF4L2	0	0	0	0	0	0	0	0	0	0	0	0
DCAF5	3.441163885	3.405576603	3.238958082	3.212865694	3.28017174	2.700168646	3.500193419	2.90275545	11.30930127	10.15592934	9.350831951	12.73234016
DCAF6	22.08857403	20.20430303	21.46552841	17.5904947	22.40307371	18.5646955	22.93193568	17.21097436	18.23663229	17.18911151	17.24069312	14.28122972
DCAF7	32.98250242	31.43814352	32.66816535	37.84539323	35.20593104	36.59197006	32.4602913	36.2190076	58.76939359	54.54597829	53.73655987	58.50958783
DCAF8	42.44622326	38.21420647	38.07608042	40.12709452	33.98990213	37.38584204	37.20837583	37.77776418	31.72973805	33.88610734	27.37619483	35.11648691
DCAF8L1	0	0	0	0	0	0	0	0	0	0	0	0
DCAF8L2	0	0	0	0	0	0	0	0	0.013281528	0	0	0
DCAKD	8.19434937	6.879319875	9.064148234	8.606789895	8.120158417	7.623270187	8.211877705	9.134473382	7.431250896	7.382153733	8.151675851	8.46086677
DCANP1	12.25519185	18.82602895	11.95372571	1.700078636	4.57021807	9.712444233	5.012819036	1.981832709	1.923923597	2.57850057	3.67922776	0.30366229
DCBLD1	6.080042709	6.180522606	6.197323971	6.730896883	6.002810318	5.697330197	6.459216123	5.544241079	6.1145685	5.175367194	4.829080169	8.580178608
DCBLD2	0.673591036	0.841551507	0.712938042	0.995010307	0.640228327	0.842520232	0.506195566	0.802764916	5.150044113	4.96201126	5.46036271	5.059762485
DCC	0	0	0.005098727	0	0	0	0	0	0	0	0	0
DCD	0	0	0	0	0	0	0	0	0	0	0	0
DCDC1	0	0	0	0	0	0.003266757	0	0	0.069234165	0.048833131	0.061675967	0.089381075
DCDC2	0	0.011573469	0	0	0	0.011464825	0	0	0.012149016	0	0	0.011618029
DCDC2B	0	0	0	0	0.025016384	0	0.024637207	0.023442978	0	0	0.025806636	0.074798173
DCDC2C	0	0	0	0	0	0	0	0	0	0	0	0
DCHS1	0.165044661	0.191840552	0.127044033	0.192084432	0.193225351	0.164358646	0.133721941	0.230010933	1.458649772	0.992637955	1.551535519	0.520484235
DCHS2	0	0	0	0	0	0	0	0	0.81261166	0.805002379	0.587434433	0.971723704
DCK	16.03760214	18.18814862	19.63964342	26.09737089	27.41450244	25.69070801	20.24923289	23.85236224	32.77195898	39.24571735	38.26317299	35.48530145
DCLK1	0	0.003997715	0.004081466	0	0.004026573	0	0	0	0	0.008456984	0	0.004013107
DCLK2	0.044822625	0.023233703	0.023720442	0	0.011700708	0	0.011523359	0.010964791	1.182871915	1.204170905	1.182891911	1.935821992
DCLK3	0	0	0	0	0	0	0	0	0.107446343	0.194876833	0.095716575	0.102750273
DCLRE1A	7.112692953	8.042964294	7.324384209	10.38251903	11.01029457	11.02650278	8.735292761	9.253882372	7.099464548	6.83064742	7.380936795	7.684967805
DCLRE1B	5.092343692	5.610058654	5.360434548	7.404685152	7.592027458	7.409860354	5.636253562	7.71192967	11.08348651	9.783345205	10.9257363	12.34632762
DCLRE1C	4.751177597	4.681812927	4.511435539	5.964986301	5.400340728	5.635321539	4.854073706	5.278610428	8.368350601	5.589745282	7.223555618	7.790143435
DCN	0.125430212	0.205885453	0.177009421	0.073950307	0	0	0.064493203	0.061367049	0	0	0	0
DCP1A	7.68454773	7.929780436	6.970172378	7.399464547	7.9777665	7.727907976	7.118558082	6.790848769	12.46207678	12.70280304	11.7527355	11.55767093
DCP1B	4.477486916	4.944814738	4.612472165	3.518250413	4.064872098	4.257101993	5.109963651	4.030224143	2.139906678	1.748277758	1.545645431	1.963418181
DCP2	9.893915189	9.733054541	8.828563407	10.86860844	10.39629862	9.97342126	9.365402155	9.451832796	14.01021042	12.21449965	11.2073511	11.3537659
DCPS	6.198008834	5.960256227	6.757948164	9.297256063	7.155129152	7.641995313	7.508124759	9.705482445	14.85320627	16.38099204	15.3967069	15.91629578
DCST1	0.140556102	0.145713857	0.099177677	0.11838304	0.097843794	0.144345995	0.216811716	0.045844953	0.076480092	0.128437907	0.050467308	0.170654023
DCST2	0.133937924	0.225635838	0.124041527	0.338426687	0.367119717	0.189130381	0.154952244	0.458706283	0.419054111	0.477322257	0.270511996	0.679513707
DCSTAMP	0.039844177	0.185878229	0.063257439	0.100675988	0	0.040918518	0.020486919	0	0.021680214	0.021845381	0.107296749	0
DCT	0.020393997	0.010571181	0	0.020612154	0.010647489	0.020943892	0	0	0.332906521	0.2795356	0.197709052	0.477534669
DCTD	25.70718959	23.48715612	28.00757203	24.44016418	26.03778473	24.86720636	29.57394795	23.30855242	17.85312715	20.14783514	21.71796762	19.88985279
DCTN1	26.57812767	31.54184102	26.89040551	32.6793131	27.78479963	29.11583999	26.58519436	34.96665851	29.92802038	28.75738345	30.56142247	31.23160787
DCTN2	38.51717806	41.71092382	39.81501428	43.74369374	35.82121888	37.72912008	41.50137591	45.55118457	24.49568428	26.63267353	26.69057175	29.97813391
DCTN3	31.93575563	31.97382585	32.4700308	44.32612319	35.34515076	30.55049742	34.80941963	44.6265673	75.8757904	77.95293359	72.3932254	73.29937165
DCTN4	19.73176102	15.29245852	17.93331599	15.39066552	18.88652797	16.28690663	18.26942766	13.79282866	31.69096109	30.82181556	29.68676275	29.07825903
DCTN5	15.39102005	12.91865546	14.90868188	15.21886604	13.8237937	12.6048885	15.20628404	12.71658906	29.19224684	22.51011933	25.6269847	30.44502125
DCTN6	32.68715775	29.92327665	31.96639235	26.80618879	30.88776941	27.97376677	36.16934597	28.94508227	35.88383426	40.6113589	39.01862467	27.60158868
DCTPP1	44.92137447	42.28635654	50.6472948	52.29716659	48.26044668	51.16526387	52.86484788	53.45212456	25.93294241	25.97246121	30.99916115	27.69945532
DCUN1D1	7.225680211	5.959624846	6.590435741	6.844602654	6.892161973	6.293901887	7.26037738	5.930950582	6.596140801	5.174643308	5.631131346	6.907381258
DCUN1D2	4.704530562	4.351074842	4.372719923	5.599626812	5.548235968	5.714273536	4.463404754	4.685193445	6.282684409	5.446759066	5.800671996	6.946274149
DCUN1D3	3.481485603	2.791024342	2.756677974	1.923334678	1.50173321	1.413932854	2.470964146	1.819168971	1.135663721	1.577039992	1.14298666	1.432827105
DCUN1D4	6.025041509	7.002706335	5.927903432	5.446235934	7.363385848	7.3901386	5.899159988	5.264462476	5.873371943	7.062658907	5.941508812	7.162136407
DCUN1D5	4.685831469	3.609342685	5.199323265	3.884367977	5.183345261	4.9999307	5.251916	3.729533811	3.010307033	3.599794166	3.656052509	2.601618094
DCX	0	0	0	0	0	0	0	0	0	0	0	0
DCXR	43.94339289	48.98774499	48.77741221	82.00417461	46.56252191	52.59409145	46.05701548	84.28279359	59.54717817	59.92965404	56.63326896	82.27582483
DDA1	12.78982456	11.52315966	13.18803684	12.00711515	10.02303097	10.14214248	12.56939491	12.87616331	9.26945211	9.643694607	9.870959089	9.591578115
DDAH1	0.009636383	0	0.020398561	0	0.010062106	0	0	0.018858499	2.820947555	3.730040141	3.248927873	2.767853882
DDAH2	32.46766741	32.28079864	29.03184691	19.30708846	27.50888125	23.2108298	29.57445253	21.49937515	32.93426031	32.8015215	31.58121435	18.89689759
DDB1	72.01784661	68.89774443	71.67473533	72.57598372	73.05831668	74.30222815	76.06680102	74.97844683	76.82746536	79.16013859	80.67534648	77.72482607
DDB2	26.5553677	30.12584061	22.22021392	14.62631428	17.05670673	19.73587806	28.89525812	18.20628853	10.7737053	22.63750076	15.90183104	9.605871716
DDC	0	0	0	0	0	0	0	0	0	0	0	0
DDHD1	4.067384263	3.858501233	3.560637046	3.624569476	4.466086103	4.362890072	3.755550984	2.937682488	6.364249583	5.876085278	5.382411338	5.555378378
DDHD2	8.718551478	9.121100467	8.207206192	8.562119997	9.827696858	9.010924894	8.523189365	7.798123271	19.44425538	27.07280091	21.46952323	15.70822192
DDI1	0	0	0	0	0	0	0	0	0	0	0	0
DDI2	4.276655397	2.39372164	3.612676426	2.698965457	2.914166048	2.659925058	2.66352114	1.689680894	3.168263908	3.104334621	3.33063521	3.593959179
DDIAS	4.31002456	4.96801301	5.984449189	9.583484773	8.085528991	8.072258134	7.046482144	9.192460742	6.391642503	5.495112789	6.436692044	7.054979381
DDIT3	11.63094097	12.60375343	11.75289859	15.39286583	7.679332928	6.941362658	8.349923375	14.30132556	19.1532309	15.68958078	19.24177275	16.85448097
DDIT4	209.0235314	216.0558264	206.3520727	109.3300473	342.9354348	235.9232957	245.2274936	139.9863446	118.8910068	89.36683113	134.8462963	43.06376154
DDIT4L	0	0	0	0	0	0	0	0	0	0	0.044010405	0
DDN	5.194664664	6.066182785	5.460186756	1.991467559	9.414343756	10.24023145	5.56298958	2.430494694	0.077973711	0.052378495	0.077179366	0.062138148
DDO	0.188915954	0.097924146	0.099975625	0	0.09863101	0	0.097136047	0	0.274117061	0.172628363	0.067831134	0.098301168
DDOST	129.5124755	135.3670197	142.6230516	151.4138349	106.8500597	117.0450823	131.5706837	144.1392488	199.803197	232.4372696	210.8268365	196.7863468
DDR1	0.116356432	0.043864063	0.167936253	0	0.011045174	0	0	0	0	0.046396206	0	0.033024709
DDR2	0.16365731	0.12974212	0.173217154	0.068109165	0.175913584	0.133467428	0.222746449	0.070649783	0.162385363	0.216403913	0.16591596	0.065120823
DDRGK1	20.16837865	20.40056651	22.253932	27.02753068	19.43943868	19.70592984	22.71344903	24.40890683	8.530489648	7.744883735	8.399609559	10.02711694
DDT	29.77015815	42.51321031	31.50914595	31.21693633	32.25106699	31.107893	30.00191675	39.1073617	21.22028126	27.01306765	26.21504439	21.22226595
DDTL	0.466220022	0.590734381	0.493453701	0.615187226	0.500339723	0.957581954	0.466120548	0.69697017	0.662391837	0.369221115	0.488245398	0.687351105
DDX1	51.33600936	46.60548657	54.08540304	57.46057945	59.93805151	55.36126203	64.52068462	56.49480562	54.43503794	51.57770191	54.30341299	51.52470736
DDX10	18.84323776	16.30455028	18.32120722	19.97788516	21.72418501	21.70462864	20.97107925	20.8095213	10.15440365	13.66645435	11.9510498	10.03656735
DDX11	5.349411644	5.852291223	6.287898669	9.11290319	10.24510141	10.72409241	5.933595188	10.28236839	15.26176363	15.02492923	17.3432064	17.18676512
DDX17	142.7254845	134.9705897	130.4950195	137.9360281	152.7329637	143.0941691	128.0116113	128.9425861	209.215124	188.7008119	172.9061915	211.2101921
DDX18	53.53801195	48.6277523	56.12080176	56.79747223	63.05158678	63.33303552	61.86074943	52.00962561	31.37105033	36.57801055	37.11704317	30.25279156
DDX19A	20.45279472	19.21341602	20.36710674	23.05174712	20.69951304	20.78893151	20.78386252	23.37499606	14.02519406	14.04360708	15.46340415	16.51766081
DDX19B	10.20972427	10.16696178	9.877700445	10.20266926	10.52564026	11.22339618	11.03154336	10.24354137	11.61149833	10.09160865	12.39159886	10.53533532
DDX20	9.82676026	10.28271417	11.39055626	12.70536702	12.90215376	13.03580048	12.54894405	13.45574835	18.21812442	15.29743039	17.86739697	20.35743445
DDX21	186.0346877	132.1664831	186.3527672	156.2666456	217.4398893	187.9031685	210.3860496	144.4311456	38.19523837	43.04581224	48.79588157	32.60003611
DDX23	46.20012801	42.9778495	49.61359522	51.72167702	50.25339014	48.19009686	51.15157851	56.21469666	30.73994823	25.57398043	29.75261005	27.7166267
DDX24	66.03900651	63.9587284	69.87727611	66.81922682	68.97558489	67.50098755	70.16379156	66.47700109	67.99352575	65.96961257	73.6711342	65.45868568
DDX25	0	0	0	0	0	0	0	0	0.073781928	0.123906708	0.146060572	0.258709738
DDX27	40.0676644	39.55710495	43.12314251	44.28060714	41.67657804	42.33728103	44.35943336	46.15629728	34.9081145	31.99950287	35.77908608	35.37089701
DDX28	9.811843596	8.365467749	9.309167029	8.931411651	9.725471313	10.84332051	8.874105566	10.87971071	5.779072401	4.867747151	6.189434045	5.137909762
DDX31	5.879064844	5.235480256	6.084386421	4.995585306	7.020344544	6.975539271	6.164137229	5.474823099	2.330302913	1.868345615	2.372701319	2.156568633
DDX39A	56.14992759	53.96443621	65.24299675	78.68776586	72.01212283	71.32882761	65.13673216	90.97450892	37.81331156	35.99039179	46.21078654	31.09303994
DDX39B	201.8855006	198.3435438	207.9303858	196.5167782	232.134718	218.1764772	203.3823601	218.3390181	138.9674193	144.6699845	140.3961266	123.7420112
DDX3X	64.03392754	56.46034225	62.51746813	64.85393627	58.04136508	53.94492552	61.65503078	53.80641733	61.25226188	52.83420756	57.41146111	57.7409414
DDX3Y	40.67088993	40.46211259	38.47179108	33.30102411	41.75092392	38.84864209	34.90847246	29.72156528	42.60267855	53.99423612	37.24706649	39.05524159
DDX4	0.016705856	0.017318883	0.053045124	0.067538241	0	0	0.017179499	0.016346764	0.018180148	0.036637302	0.035989881	0.069542253
DDX41	35.86702363	37.1644635	37.86662615	39.73528923	38.41284656	44.19353681	36.8096739	47.76032143	28.42462193	33.70830242	32.70432256	31.85525865
DDX42	51.19055972	45.94186133	49.6416658	50.74552033	52.59749797	49.69070641	52.94490409	48.0400577	48.30967141	45.41099182	48.42876071	52.39888851
DDX43	0	0	0	0.02017254	0	0	0	0	0.021720422	0	0	0
DDX46	35.18890924	35.68001525	39.44455872	45.71545468	46.27740725	44.57767065	39.05481286	40.89506169	36.6335111	32.52694279	36.25086503	36.63888833
DDX47	34.76743228	32.51259487	40.21486979	35.52845081	44.15781422	38.01627584	41.26535634	37.93213163	11.69876022	14.32081953	11.86667791	10.26286919
DDX49	52.65569475	45.48506576	59.42510392	53.6171772	50.39767764	49.94250649	59.06855525	58.5460908	18.89667605	15.49173427	21.76595357	20.20880763
DDX5	180.1997304	145.7678575	184.1074761	172.5708131	160.6570375	140.9434678	181.9558269	161.3884607	166.8169002	141.6309284	157.0773458	193.8693754
DDX50	11.73653315	12.37281099	12.66200443	13.00748339	15.30215695	14.98440761	13.50419919	13.59813681	7.715784562	9.99587071	8.316211266	8.049334939
DDX51	9.195592467	8.390007319	9.624466633	11.22397755	12.61651182	12.53691012	11.11612688	13.26889385	8.436149412	7.902148994	10.01535141	8.954619016
DDX52	8.519044966	8.207949928	8.897810794	9.996558271	8.376088049	8.534560983	9.461752766	8.76763878	9.524009907	8.294741355	9.461548227	11.53704207
DDX53	0	0	0.026750954	0	0	0	0	0	0	0	0	0
DDX54	32.09412977	30.81432881	33.14987271	35.54091251	38.15469663	38.48428648	35.16391403	42.32906039	18.88674212	18.34374751	20.20258422	17.43494591
DDX55	19.70385783	19.82968481	20.62901099	20.22731489	23.63766588	23.68618387	23.84985229	20.8461336	5.769915496	5.533122337	6.664906448	5.761980273
DDX56	29.93999939	28.55245783	34.82210445	30.92304329	30.22583701	30.49715419	34.29547313	35.85972417	28.52472202	25.33161336	30.4137491	28.7208837
DDX58	9.4924772	6.017084615	6.155115557	5.191519458	7.28916206	6.727461209	7.655706574	4.627612288	7.005813345	3.746703333	4.216726128	4.332967782
DDX59	11.22791767	11.72098808	10.89070912	12.29632699	12.50772675	12.14092028	12.47895678	12.77686578	8.679079076	8.436545242	8.354840167	8.560133911
DDX6	23.6204525	21.4933703	22.8623853	29.41539986	24.47223989	24.33448765	24.70212706	26.4285522	49.03016467	44.7086038	41.71588413	48.78498577
DDX60	2.921713867	2.129628224	2.123548467	2.108494322	2.35616681	2.557797904	2.391600075	1.671604193	5.113929481	4.003263852	3.238884637	3.959965648
DDX60L	18.57084418	16.66387984	12.54903897	10.5510228	10.0197677	11.54551342	11.24884714	8.002928158	19.49738807	17.04866798	13.72356365	20.42197113
DEAF1	9.03340489	11.42096223	9.147379725	10.15423917	10.41504016	13.27618765	8.94711754	11.38901113	7.420214979	8.651435034	9.028739957	9.883044831
44166	0	0.029252292	0	0	0	0	0	0	0	0	0	0
DECR1	27.75223311	27.54828031	27.06466052	25.81470753	27.43934866	27.89510412	31.40357925	25.38185534	33.40916173	34.77880405	32.37028588	28.55928503
DECR2	17.33599244	16.63304197	20.18351883	16.59383224	18.20835609	16.16272414	21.39300236	21.28734942	7.842293428	7.827491228	8.311627751	9.303670495
DEDD	12.51058233	12.91137195	13.49429797	12.99958967	12.0651903	13.20880867	13.13993407	13.67215537	11.25884984	9.618267919	10.37193495	11.19100251
DEDD2	10.83179472	11.80884654	11.46452091	8.287263787	8.488828018	8.301029923	8.83925409	8.456379398	7.959866977	6.717814049	6.272911651	7.975600744
DEF6	38.88068879	46.64874288	36.53808661	40.57569417	43.87758273	48.49731011	42.41720186	45.79612276	74.72808937	72.31633685	73.66387251	87.04706879
DEF8	7.118975025	9.04636245	7.393066384	8.7674348	6.35772506	7.871679336	7.045354516	8.457937366	10.4155889	11.5681561	10.1763129	12.7801431
DEFA1	0	0	0	0	0	0	0	0.092373558	0	0	0	0
DEFA1B	0	0	0	0	0	0	0	0	0	0	0	0
DEFA3	0	0	0	0.188510116	0	0.478860126	0.575409025	0	0	0	0	0.097051818
DEFA4	0	0.090119744	0	0.263579169	0	0.178547524	0	0.085061274	0	0	0	0
DEFA5	0	0	0	0	0	0	0	0	0	0	0	0
DEFA6	0	0	0	0	0	0	0	0	0	0	0	0
DEFB1	0.58109677	0.240968128	0.369024501	0.234925134	0	0	0.119514401	0.341163682	1.897135758	1.529271041	2.628932563	3.023698641
DEFB103A	0	0	0	0	0	0	0	0	0	0	0	0
DEFB103B	0	0	0	0	0	0	0	0	0	0	0	0
DEFB104A	0	0	0	0	0	0	0	0	0	0	0	0
DEFB104B	0	0	0	0	0	0	0	0	0	0	0	0

DEFB105A	0	0	0	0	0	0	0	0	0	0	0	0
DEFB105B	0	0	0	0	0	0	0	0	0	0	0	0
DEFB106A	0	0	0	0	0	0	0	0	0	0	0	0
DEFB106B	0	0	0	0	0	0	0	0	0	0	0	0
DEFB107A	0	0	0	0	0	0	0	0	0	0	0	0
DEFB107B	0	0	0	0	0	0	0	0	0	0	0	0
DEFB108B	0	0	0	0	0	0	0	0	0	0	0	0
DEFB109B	0	0	0	0	0	0	0	0	0	0	0	0
DEFB110	0	0	0	0	0	0	0	0	0	0	0	0
DEFB112	0	0	0	0	0	0	0	0	0	0	0	0
DEFB113	0	0	0	0	0	0	0	0	0	0	0	0
DEFB114	0	0	0	0	0	0	0	0	0	0	0	0
DEFB115	0	0	0	0	0	0	0	0	0	0	0	0
DEFB116	0	0	0	0	0	0	0	0	0	0	0	0
DEFB118	0	0	0	0	0	0	0	0	0	0	0	0
DEFB119	0	0	0	0	0	0	0	0	0	0	0	0
DEFB121	0	0	0	0	0	0	0	0	0	0	0	0
DEFB123	0	0	0	0	0	0	0	0	0	0	0	0
DEFB124	0	0	0	0	0	0	0	0	0	0	0.066015608	0
DEFB125	0	0	0	0	0	0	0	0	0	0	0	0
DEFB126	0	0	0	0	0	0	0	0	0	0	0	0
DEFB127	0	0	0	0	0	0	0	0	0	0	0	0
DEFB128	0	0	0	0	0	0	0	0	0	0	0	0
DEFB129	0	0	0	0	0	0	0	0	0	0	0	0
DEFB130A	0	0	0	0	0	0	0	0	0	0	0	0
DEFB130B	0	0	0	0	0	0	0	0	0	0	0	0
DEFB131A	0	0	0	0	0	0	0	0	0	0	0	0
DEFB131B	0	0	0	0	0	0	0	0	0	0	0	0
DEFB132	0	0	0	0	0	0	0	0	0	0	0	0
DEFB133	0	0	0	0	0	0	0	0	0	0	0	0
DEFB134	0	0	0	0	0	0	0	0	0	0	0	0
DEFB135	0	0	0	0	0	0	0	0	0	0	0	0
DEFB136	0	0	0	0	0	0	0	0	0	0	0	0
DEFB4A	0	0	0	0	0	0	0	0	0	0	0	0
DEFB4B	0	0	0	0	0	0	0	0	0	0	0	0
DEGS1	34.15259883	34.10800584	34.79931168	40.60019321	29.40060239	28.17155622	34.60141926	36.21232009	60.75717937	54.8138596	60.54040803	74.03282993
DEGS2	0.710692313	0.666602712	0.573109749	0.273636068	0.45938892	0.486570075	0.174009962	0.364265542	1.509992792	1.335948098	1.713333379	1.267899596
DEK	76.9487635	89.05092044	89.13415584	152.4864942	157.9287009	144.9966538	107.9765345	142.8388047	49.73464255	51.85751011	53.17495039	51.92595662
DELE1	10.87416962	9.668224668	9.479073642	9.587848384	8.804144169	9.374768303	9.856814636	9.391100008	10.16244855	10.10460056	9.86624598	10.16119405
DENND1A	2.904182002	2.881645983	2.7891151	2.260594399	2.371892258	2.036073182	2.448640089	2.446932977	7.08532142	6.866182055	6.626800965	9.196634562
DENND1B	1.555036839	1.349505359	1.174957093	0.910500776	1.131555666	1.144567828	1.21633177	0.661663897	6.184212166	3.644600937	4.571928078	3.144923832
DENND1C	5.446378045	7.341800819	4.604692956	2.843236778	3.159440045	3.7624142	4.860748789	3.424840931	5.855825706	5.129255642	5.584759897	5.633931768
DENND2A	0	0.00979528	0.020000977	0	0	0.009703329	0	0	0.082259185	0.031082199	0.142487068	0.068830956
DENND2C	0	0.012358024	0.012616921	0	0	0	0	0	0.570793822	0.535928079	0.552138515	0.595469017
DENND2D	0.912981657	0.654359199	0.870874117	0.524029693	1.176933361	0.902873014	1.460458976	1.080851901	4.207264804	3.633700473	4.285815122	4.351820584
DENND3	32.67318516	37.69630952	29.65553726	18.92909135	26.94303698	32.73422609	21.2519123	18.2777246	19.51941902	31.2939004	21.85397568	22.32484281
DENND4A	7.217348453	5.822653738	6.556584354	6.161611821	6.599823507	6.062857124	5.961847632	4.9762617	9.750435611	5.994889288	7.651402442	6.577752389
DENND4B	27.61302826	29.9156903	27.41595363	23.85789178	28.10241806	28.18904291	22.92456955	24.83899781	44.02678589	42.16492369	43.34567855	48.61028008
DENND4C	6.10943735	6.30756101	4.962828507	6.308196755	5.69021778	5.589949436	4.957607028	4.514338207	4.822283755	4.052630781	3.473233732	5.455330175
DENND5A	27.40490927	26.34776725	25.81829062	24.61083784	20.74295764	20.87592778	22.25351944	21.29060044	13.00384241	17.5727599	12.53539144	14.21519119
DENND5B	2.688479174	2.056676849	2.469998966	1.651555547	2.097612653	1.878280817	1.983597214	1.320235016	5.655700905	3.5507833	4.413874164	4.659640914
DENND6A	5.625644304	5.945323567	5.838642825	7.684140752	7.425417359	7.516140325	5.672809537	6.317068745	8.987016309	9.199220361	9.80148176	10.79962523
DENND6B	1.134772643	1.498167652	1.252386323	0.362698493	0.6785356	0.916359443	1.176919576	0.597897348	1.963200399	1.871804233	1.713359661	2.059079914
DENR	53.13743556	53.36585338	55.4044691	59.97952834	57.83813731	55.97100818	61.63711573	57.41142221	18.97442422	18.98891695	21.16602992	19.54406829
DEPDC1	4.195522951	3.415982443	4.293975788	8.890844959	8.544774268	7.347059865	6.156266379	6.981015322	14.00961612	17.57761796	15.35910536	11.35836437
DEPDC1B	4.436650132	4.40579333	4.77489904	10.79962383	10.99158515	9.95666655	6.262546339	10.09919906	10.82529835	13.35561606	12.39521245	9.117682192
DEPDC4	0.413404018	0.613903333	0.650415899	0.54204487	0.980002302	0.745832601	0.666411906	0.5138471	0.486364261	0.673845637	0.794325673	0.290691958
DEPDC5	4.993500527	5.090171254	5.196808722	5.139752898	5.240264739	5.779880365	5.074966514	5.000557439	4.452753873	4.34932927	4.677232449	4.310281475
DEPDC7	1.49540234	0.800976273	1.529995978	0.93202929	1.639540673	1.766082279	2.127289918	0.731629388	5.560186521	5.903170384	5.798855021	3.605301963
DEPP1	2.429266583	4.603544024	2.681757178	1.16162496	3.763970005	3.487310492	2.659311466	1.175745001	5.315725277	2.176860413	4.867657637	0.652414355
DEPTOR	0.472013692	0.643125213	0.19983432	0.626996931	0.492866655	0.983331468	0.318974652	0.508054572	1.518992382	1.464018311	1.038662108	1.649090379
DERA	13.30442036	12.80553726	13.26446891	16.02164699	15.82688876	14.90525999	14.50199522	15.9897702	10.19378554	8.916969055	9.286069214	9.105512006
DERL1	32.12700625	31.79201272	32.17244136	36.353036	32.3364142	29.27663233	31.38924125	33.26919542	35.91227623	35.73332759	37.66655845	38.58803177
DERL2	5.748013252	5.879662282	6.218671762	7.020349807	5.549477743	5.117678659	6.697363995	6.522378378	4.216416425	3.479888492	3.967534902	4.50962236
DERL3	0.727712867	1.096039135	0.552234164	0.957534226	1.275995172	1.692078343	0.494190068	1.182306122	0.896528559	1.806717255	1.405042574	1.557509562
DES	0.311176286	0.148890005	0.380023007	0.024192691	0.074982383	0.073746164	0.467690476	0.023422121	2.266267066	1.128642392	2.5783676	0.473300302
DESI1	15.22890544	15.92986065	16.08220788	14.9068477	15.26944846	17.56367441	18.1043511	16.81316437	11.78624792	14.10592898	13.9920291	14.10078429
DESI2	12.88916253	11.1102988	12.53056189	15.80299645	16.1484327	13.82663684	13.90071106	14.88685797	19.13521695	17.32238513	17.85742563	19.79343897
DET1	3.567716594	2.39323455	3.087533776	2.799860474	3.681506307	3.944096692	2.719278921	2.772287568	3.037538295	2.531388915	2.71271624	2.599012856
DEUP1	0	0	0	0	0	0	0	0	0	0	0.031609727	0.010179767
DEXI	17.89184651	17.78422809	17.58940195	19.53937876	14.65894684	17.37642066	18.12347341	21.83490054	17.46538302	16.42276173	18.18972518	23.71061719
DFFA	10.98863785	9.135433413	10.46267387	8.875781729	10.24304278	10.43118768	10.78724902	9.066360973	7.434924773	9.546775175	9.622294063	7.17289266
DFFB	5.818138224	4.719670651	6.331952472	4.667755766	6.023691606	5.688080188	6.422530175	5.484009945	2.951164452	2.703315868	2.903396274	3.061638024
DGAT1	9.690106859	12.03493405	9.762919464	12.32878249	11.03413697	12.49905431	9.837989602	14.92682354	13.93107797	14.33779244	14.14348245	19.91198746
DGAT2	14.50186747	15.53954848	15.61825681	15.94269718	14.12418342	15.39367384	13.3214293	17.57172194	25.56444004	18.71494143	23.88807802	31.26494864
DGAT2L6	0	0	0	0	0	0	0	0	0	0	0	0
DGCR2	10.83631433	11.1966346	11.59631787	13.53564856	9.999345519	10.77110633	11.30397274	14.31398743	23.91172361	18.31714743	21.0311534	33.11977692
DGCR6	2.94681016	3.55712704	3.418021384	4.324702079	2.613339471	3.150633797	3.113381539	4.18695454	5.403350052	6.374065946	4.56562557	6.511499865
DGCR6L	12.46860819	13.75122145	14.69447274	16.93671232	11.77291179	13.07724903	12.91305479	20.98329518	15.68610666	15.07835696	14.76427949	22.86890117
DGCR8	12.50670373	13.43132631	14.06303299	20.97986732	18.09530905	16.92290845	13.04363689	21.64183412	16.20900789	10.92721608	15.44541884	16.96451702
DGKA	9.059242493	11.57453153	8.295296407	6.436891936	6.975848912	8.529331648	9.035404224	6.282740723	6.251871889	8.736638038	6.258183946	8.04815092
DGKB	0	0	0	0	0	0.005282522	0	0	0	0	0	0
DGKD	9.550427728	10.1303562	8.845480632	10.87485111	10.21475661	10.82255595	8.438793536	10.23792265	9.952612367	12.25434483	10.21177402	14.37194574
DGKE	5.998461527	4.122095802	5.613279631	4.554167728	5.198736185	4.422708731	5.043783564	3.779238345	5.845906882	4.866018168	4.51617763	5.483694836
DGKG	1.827039554	2.836317804	1.894499619	3.880637677	2.043332387	2.933509348	1.869302465	3.566459985	3.007645744	3.366157861	2.767216807	4.459064334
DGKH	9.645253488	7.053056043	7.976931823	5.806004994	7.984021768	7.030592693	6.771009516	4.069921008	6.781301129	5.675229899	5.525780739	5.386153629
DGKI	0.005227782	0.005419617	0.005533157	0	0	0	0	0	0	0	0	0.016321451
DGKK	0.007153624	0	0.007571494	0	0	0	0	0	0	0.00784424	0	0
DGKQ	7.80043063	7.032390835	8.39802175	8.934302879	6.604720254	5.990853452	7.826220005	9.513613217	7.746959449	6.703092995	6.404076084	12.6884317
DGKZ	28.96778049	31.4269227	29.37975455	32.01728481	26.01987088	27.44083029	27.18683045	35.78183221	32.99911346	30.69205699	30.78724639	45.69717251
DGLUCY	7.926032431	8.817563324	6.769072951	6.939025981	6.404062176	8.005019699	6.351964618	7.616595889	9.196573082	9.014890189	8.808483205	8.658098743
DGUOK	27.86722786	29.4794139	31.94912122	28.38640507	28.8242845	30.37078861	36.35025662	31.76298603	29.51717023	34.01146456	33.78743213	28.3636699
DHCR24	35.29133821	53.11595909	37.2199581	63.58471298	39.66898102	55.78908171	36.65341615	58.84449992	91.34869499	94.52263982	118.4252541	67.92773566
DHCR7	23.56931819	35.91389328	23.27759264	25.6478823	23.82769917	34.96472745	20.97556739	28.04770137	28.62011278	52.40617171	56.1808153	10.38350429
DHDDS	10.5115605	10.81410054	10.49711269	10.44558562	7.406670145	8.108602927	9.703870776	10.63108633	8.592502975	10.59368725	8.712406396	10.60521943
DHDH	0	0.094442644	0	0	0.095124377	0	0	0	0	0.133192719	0.06541953	0.063204175
DHFR	25.70275424	29.56544208	30.50470645	42.38905278	41.18261396	41.12439028	29.92082858	40.31302835	43.10019278	37.9001802	49.15720693	38.32857998
DHFR2	1.408823072	1.47344529	1.411943373	2.179943042	1.822556026	1.94615146	1.743647551	2.025112628	2.021589505	2.078003916	1.678686969	1.816461037
DHH	0	0	0.020710102	0.009888212	0	0	0	0	0.010646956	0.010728068	0.021076983	0.010181618
DHODH	6.253259514	5.320555878	6.84100791	5.983784826	7.279409458	7.501175528	6.286449736	7.455736921	3.526457538	4.033502153	3.90562261	3.645761643
DHPS	35.47810802	34.69874726	37.62018582	41.17971787	47.59554502	45.05332799	36.24707327	46.85627566	41.2593958	47.13132287	42.8243046	33.49658903
DHRS1	6.488341252	7.631914621	6.504172705	5.328170141	4.26693949	6.342939592	6.415671638	5.829976412	5.737011851	7.148935791	5.94737481	9.057213203
DHRS11	2.963229885	3.002149228	3.314523568	4.390292083	4.324766102	4.841341802	3.670543051	4.217506149	3.041511474	4.283171104	4.026125849	3.644484029
DHRS12	3.966080077	3.863007399	3.045812096	2.255949743	2.099540984	2.443811846	3.414580123	3.068562844	2.308604581	2.043003706	1.867809443	2.726034842
DHRS13	3.19327951	4.326826722	3.557695865	5.209198866	4.56280093	5.436869687	3.11098289	5.838143536	6.035676678	5.252341496	5.370735685	9.065933128
DHRS2	0	0.021613375	0	0	0	0.064231451	0	0	0.22688205	0.114305258	0.157199506	0.10848295
DHRS3	0.553002636	2.318106934	0.91613053	1.822559269	1.380819448	2.864259693	0.395604417	1.646874434	4.264966891	5.984804726	5.542361297	4.028517745
DHRS4	8.627185373	10.35812551	8.791368926	9.611696464	11.56417332	12.40372709	12.17291165	14.37059789	19.43208381	17.55611138	18.67222668	21.38063848
DHRS4L1	0.253072597	0.393538777	0.368301349	0.639449366	0.528506042	0.617253799	0.325309629	0.340495127	0.791793011	0.346880508	0.78372674	0.954713737
DHRS4L2	4.21981692	5.03229391	4.11601341	6.467124098	7.401336365	7.307636243	6.324829542	9.148817377	14.85716927	13.85136005	15.38911455	14.66706166
DHRS7	14.83133682	16.05328939	14.16251775	13.5653211	12.79881518	12.46192574	15.08377014	12.35364833	10.76013531	12.18617299	11.22265333	11.37411195
DHRS7B	4.35056024	4.254566401	4.53012303	4.503910853	3.862267664	4.395512744	4.799940913	5.170500326	2.30016067	2.085915689	2.314673599	3.281113876
DHRS7C	0	0	0	0	0	0	0	0	0	0	0	0
DHRS9	15.93572734	28.62413594	14.89840911	15.79691686	15.23876428	30.55521419	13.67645225	18.44925441	37.60434006	33.52873241	40.94844769	68.07565174
DHRSX	0.062642096	0.259763081	0.486209231	0.084416249	0.087212726	0.042887434	0.085890831	0.633387962	0.204510793	0.137379219	1.124596501	0.217302673
DHTKD1	15.07946674	14.78808517	14.84680517	12.23848724	16.41339488	16.51352404	14.37208213	11.15228787	17.6112527	16.59179934	17.54294232	13.42814308
DHX15	77.48505399	74.6501045	88.39388902	92.0725036	97.60034417	92.47332042	91.2222993	85.57846639	92.20595583	91.69545841	96.49393703	83.01428273
DHX16	20.95582258	20.14116829	21.83835157	21.54966097	18.28710679	19.33342713	21.49464838	24.42119027	6.47395365	5.992447455	6.025606938	7.803125248
DHX29	11.21454752	9.548317964	10.29654803	13.28049629	10.62834609	10.05997049	12.22723107	10.89636872	23.31792492	21.21144908	22.04946166	23.76350007
DHX30	20.8713953	19.3279888	24.14654903	23.08995583	24.19659074	23.07126856	25.49087192	26.30063871	19.2666661	18.85778415	20.43499735	20.88560068
DHX32	19.91129027	21.78669769	21.40830932	18.88423118	23.38981389	22.71618851	21.86369988	18.66006059	0.476858771	0.8456653	0.641921148	0.510739142
DHX33	18.8783632	15.23190507	17.67989509	15.21777947	21.22256794	21.27596509	18.35969258	15.49221974	5.368013953	6.333150184	6.541068017	6.030497294
DHX34	8.292506481	7.008676729	7.456484153	7.732224789	7.748912392	7.630578152	7.952189997	8.661792675	4.761726665	4.043600151	4.436550451	5.708718293
DHX35	6.446659914	5.372159613	6.235586832	7.139125027	6.763673094	7.206508641	6.899053605	6.640094142	6.91487587	7.593282913	7.575390332	8.249766123
DHX36	15.40822097	13.39375063	15.26731994	14.36816695	17.92500471	16.17565902	17.23956352	12.20451426	19.89780709	17.95785306	18.74806698	19.57092504
DHX37	28.19330246	22.67443015	28.0451068	25.14451409	29.18585651	27.09866122	28.93601186	27.45797408	4.098838296	3.912692855	4.934930152	3.759238399
DHX38	19.94716241	19.45324199	20.6951616	22.94921321	22.16291702	21.02473385	20.56183597	23.10651261	24.65816063	22.22650905	24.78007575	25.55687814
DHX40	6.441372514	8.101114307	6.864015988	8.340830689	14.51949845	15.28512684	13.83379197	14.63534164	21.80834112	21.73423887	18.34509948	21.03758849
DHX57	5.718182217	5.439440438	6.273895332	6.954273213	6.517581157	7.24903641	6.515721403	6.620045095	7.704433977	7.94687175	7.603384248	9.37311269
DHX58	1.405998231	0.832909618	0.786581899	0.466912608	0.796975867	0.969481729	1.074068208	0.864773739	5.661287264	4.603178141	4.08912355	4.682252086
DHX8	16.9815812	14.97671779	16.14825796	17.41087359	15.53747023	15.43615261	17.1723148	17.73232595	22.67465961	18.85866606	21.25600831	23.51757608
DHX9	108.7223601	105.2224306	121.8784498	140.2432357	129.8142967	127.6375274	130.3475019	134.4646212	88.85724942	77.04649675	88.73149696	89.13404974
DIABLO	17.29426545	17.51006072	17.20260157	18.21738249	17.08481056	16.05313928	16.84183279	18.91430633	7.440263675	6.917636727	6.058947942	8.602776258
DIAPH1	74.56790659	62.52379394	74.09719381	59.72678012	88.02028689	73.84071705	67.31924378	57.93881552	69.78128677	65.91941692	70.47263202	65.37666605
DIAPH2	9.979113178	8.093484115	9.495832296	7.434478001	8.075208774	7.597235431	8.19020913	5.965552137	9.101195569	9.251075853	8.714600921	8.266608116
DIAPH3	1.942017328	1.992438928	2.281005065	4.311638936	4.395694401	3.592363368	2.501515099	3.898905475	7.32908022	8.165291885	8.220228166	6.284864833
DICER1	5.307122989	4.60785418	4.688758066	5.068387282	4.742881246	4.149083827	4.607214581	3.265521899	7.183189052	7.480796089	6.521486965	7.317968064
DIDO1	10.24925209	10.0357371	9.769425272	11.68818069	11.43614156	10.32685904	10.39362115	10.76324173	12.13373163	9.956771626	10.79336775	11.8418106

DIEXF	6.80118496	5.728330769	6.710549516	6.478223932	7.602790614	7.153184579	6.273180633	6.852728261	3.442986362	3.143760821	3.210647122	3.055919254
DIMT1	13.79408829	12.51273382	15.42332061	14.64297818	14.46936315	14.42516074	16.39080002	15.20535914	7.505708604	8.162387073	8.154049753	8.425000411
DIO1	0	0	0	0	0	0	0	0	0.125864067	0.063411472	0.093436384	0
DIO2	0	0.007895842	0	0.00769783	0	0	0	0.014905288	0.024865502	0.100219744	0.049224377	0.047557451
DIO3	0	0	0	0	0	0	0	0	0	0	0	0
DIP2A	4.781673926	5.038671033	4.345125963	5.26205519	5.458271289	5.449048433	4.5506072	4.745586934	6.601569075	7.554491659	5.754942612	7.338840391
DIP2B	30.18645359	21.40471635	27.05502176	17.44194449	28.41897973	20.68383429	24.24207355	14.2341135	7.754013614	6.783979701	6.378115182	7.479370998
DIP2C	0.00529257	0.005486783	0.022406917	0	0	0.010870553	0	0	0.069115677	0.092856299	0.057009643	0.044063263
DIRAS1	0.500965244	0.702647754	0.686178088	0.104243204	0.415400757	0.680920218	0.909121049	0.288351196	3.960539292	5.735638799	5.808867344	2.714075875
DIRAS2	0	0	0	0	0	0	0	0	0	0	0	0
DIRAS3	0.027272965	0.113095026	0.028866083	0.220517663	0	0	0	0	0	0	0	0
DIRC1	0	0	0	0	0	0	0	0	0	0	0	0
DIRC2	0.838506792	1.120028814	0.631481243	0.635607305	0.454612995	0.861115926	0.630127773	0.694254973	1.281016711	1.64441319	1.38955238	1.929839187
DIS3	23.09144896	19.18903636	23.15711726	19.90465825	24.19256053	21.65827771	25.79021841	18.31886005	24.24713788	24.43959021	23.55216201	22.13841717
DIS3L	9.328658509	7.549661106	8.634654489	11.30691317	12.05469011	11.69077121	9.960697772	10.32598943	10.20053332	9.102468245	9.836742135	11.7633024
DIS3L2	6.022053908	5.384617949	5.723163614	7.126244988	6.668006646	6.403461858	6.115381109	6.371384892	7.372687996	6.740998701	6.540793791	7.977460291
DISC1	2.686242975	2.868443706	2.322338133	1.899669333	2.19423747	2.655119852	2.231490917	1.495799403	4.099648609	3.029607708	3.145511839	3.983431152
DISP1	0.54314992	0.523978151	0.654721445	0.892060105	0.543514525	0.480323627	0.442184855	0.597909322	1.305308127	0.860290892	1.072612489	1.397421
DISP2	0.278345736	0.233303613	0.256995821	0.191539225	0.272091035	0.273687061	0.359319272	0.115899028	0.34802398	0.240277553	0.236031591	0.197222624
DISP3	0.036206137	0.030027787	0.01532843	0.051230814	0.045366814	0.037182384	0.022339591	0.0637702	0.070922395	0.031761203	0.046799922	0.052750948
DIXDC1	3.664684101	3.731560249	3.31281326	2.557131049	4.160219821	4.634056874	3.493652747	2.424637998	1.13540004	1.880532047	1.741941651	1.106134332
DKC1	83.53342402	72.78953771	87.38528996	97.14957468	98.32245104	97.87346175	89.68536231	96.40536164	33.42213912	32.44400071	40.36585883	34.33677336
DKK1	0	0	0	0	0.02952538	0	0	0	0	0	0	0
DKK2	5.6812954	4.862129936	4.619485973	4.216831879	2.765311233	3.37306071	3.970986752	4.053566107	1.674473119	1.411432656	1.035884173	3.156385555
DKK3	0	0.016188079	0.016527215	0	0	0	0	0	0	0.017122569	0	0
DKK4	0	0	0	0	0	0	0	0	0	0	0	0
DKKL1	0	0	0	0	0	0	0	0	0.409259805	1.030944223	0.438848077	0.358758234
DLAT	24.3665936	22.8091082	27.02177521	27.06744585	30.56272249	31.35579632	29.70477374	26.54453888	19.95508024	22.45587556	25.0598346	18.94118299
DLC1	0	0	0	0	0.006070311	0	0	0	0	0.012749433	0.006262068	0
DLD	38.12689615	33.87499668	41.42593669	36.59295457	38.97152432	35.83594089	41.24484005	34.05754041	33.66329917	34.03407588	34.47542241	32.03701187
DLEC1	0.008575229	0.044449499	0	0.008666959	0	0	0	0	0.167975877	0.094030875	0.110843099	0.0981654
DLEU7	0.030036037	0.01556911	0	0.015178668	0.015681496	0	0	0.029390415	0.081716797	0.082339345	0.048530592	0.031258107
DLG1	6.883093442	6.421498657	6.661071742	8.656074898	8.302747069	7.452397743	6.826091557	7.489203541	10.70529057	9.90341403	9.678101544	12.14514471
DLG2	0.016225153	0.021626409	0.012266375	0.028112084	0.045985319	0.02618415	0.016685168	0.027216676	0.105942141	0.09658265	0.067411846	0.084426511
DLG3	0.01812787	0.012528719	0.012791192	0	0.006309579	0.024822216	0.012427887	0	0.552374351	0.265039301	0.312426926	0.716886521
DLG4	8.45501634	9.904108621	7.56701947	5.27834635	7.766113733	8.479558318	7.426172128	5.638867826	2.420329567	3.139791004	2.647848912	1.771049511
DLG5	0.688372654	0.642269459	0.818254878	0.877698057	0.834895355	0.739561343	0.789568918	0.943006589	7.594940249	5.974759024	6.274146234	8.910669103
DLGAP1	0	0	0	0	0	0	0	0	0	0	0.00554923	0.010722623
DLGAP2	0	0	0	0	0	0	0	0	0	0	0	0
DLGAP3	0	0	0.005395299	0	0.015968207	0	0	0.004987962	0.005547391	0	0.005490878	0.015914806
DLGAP4	6.536556934	6.129333172	6.285267471	4.372192546	7.259837684	5.858124978	6.15132364	4.979421448	5.151088828	5.485020783	5.621547544	4.98910763
DLGAP5	12.6217995	12.8048043	14.7219154	34.0610214	32.79914744	29.45047777	20.87533512	33.14723494	37.08977936	41.99159167	38.40712003	25.11265242
DLK1	0	0.006752708	0	0	0	0	0	0	0	0.03571261	0	0
DLK2	0	0.019607776	0.040037105	0.038232104	0.039498628	0.038847423	0.038899942	0	0.020582867	0.082958699	0.122239094	0.098416342
DLL1	19.57006345	19.44705811	17.75910008	13.72749883	34.85411483	24.13062244	22.94507112	16.03723088	22.21540633	22.98964163	22.93297805	11.58481442
DLL3	0	0	0.023899614	0	0.023578178	0	0.023220801	0	0.172013344	0.321887061	0.170260984	0.070497984
DLL4	0.047201369	0.032622293	0.06661144	0.015902096	0.016428888	0	0	0	0.171222974	0.17252741	0.118635066	0.212861311
DLST	17.23233834	17.89597174	17.83966818	18.6062551	18.84447873	18.4718078	16.92951995	18.69282312	36.15964163	36.68331406	39.82225854	34.70487
DLX1	15.39996664	12.61755837	14.60266445	10.01892111	10.44513294	12.15127163	14.65232519	11.46742333	2.088733466	1.510393133	2.724175749	2.58492609
DLX2	3.713420216	2.953846722	3.979773346	1.439885106	1.80461072	1.918765922	4.106906991	1.234053008	0.152495461	0.076828612	0.176098926	0.194440621
DLX3	0.744482779	0.600290318	0.875523156	0.606137544	2.504868809	0.849507434	0.893188721	0.748715737	8.169349467	4.036425301	6.237231754	13.98896397
DLX4	4.090658084	5.57164499	4.306579096	4.178399528	4.952981036	4.781940208	4.251387746	4.23693196	5.304104158	4.628729731	4.031303287	5.955402505
DLX5	8.900189858	4.040026524	8.213304325	3.525920165	17.75161665	7.532339545	10.67503239	4.878971537	0	0	0	0
DLX6	5.108320978	3.59993532	5.042219571	4.553851451	8.989887781	5.835504425	7.112413578	5.363578299	1.624015663	2.108422977	1.947513243	1.164777096
DMAC1	13.22693116	11.68661767	13.06777703	11.74077263	11.32255918	12.54716717	11.96794134	12.18623241	5.117421507	6.592667525	5.782292863	6.279206135
DMAC2	21.87195934	22.41302967	24.13751256	24.75761777	23.76019333	25.18198112	25.60516207	26.41286137	19.27233777	20.85761756	22.74446599	19.42765361
DMAP1	9.320944531	9.219005131	9.945406098	11.12655369	9.180333671	8.511559395	9.688835574	11.46236501	20.09513222	15.60743048	17.25826008	22.12690572
DMBT1	0	0	0	0	0.006842036	0	0	0	0	0	0	0
DMBX1	0	0.017503647	0.017870342	0	0	0	0	0	0.0183741	0.074056322	0	0.017571038
DMC1	0	0.020160507	0	0	0	0	0	0	0.232793938	0.149270192	0.418949792	0.121428765
DMD	0	0	0.002348568	0.002242687	0	0	0	0.002171255	0.212500067	0.277381389	0.286820802	0.143172442
DMGDH	0.012407093	0.012862375	0.013131838	0	0.006477611	0.025483264	0.006379429	0	0.114767163	0.054419529	0.053457877	0.109751128
DMKN	0	0	0	0.02090933	0.043203997	0	0	0	0	0.068055795	0	0
DMP1	0.014762568	0	0	0	0	0	0	0	0	0	0	0.01536321
DMPK	8.714098925	7.754068198	7.363126112	9.174286201	10.67625	9.232449083	8.96116079	11.28378545	5.83211791	3.057716565	3.957978684	6.650341562
DMRT1	0.017758931	0.018410601	0	0	0.018543498	0	0	0.034754408	0.135283102	0.116840345	0.095646376	0
DMRT2	0	0	0	0	0.007201589	0	0.007092434	0	1.523625504	1.73942657	1.574965544	1.715423843
DMRT3	0.024683285	0.127945233	0.130625645	0.074841975	0.077321283	0.050697668	0.076149314	0.096610878	0.644678059	1.272112658	0.664698436	0.693564343
DMRTA1	0	0	0	0	0	0	0	0	0.686825778	0.820217193	0.629471152	0.583828659
DMRTA2	0	0	0	0	0	0	0	0.012521505	24.55129779	17.06285831	21.37897797	30.17681445
DMRTB1	0	0	0	0	0	0	0	0	0	0	0	0
DMRTC1	0	0	0	0	0	0	0	0	0	0	0	0
DMRTC1B	0	0	0	0	0	0	0	0	0	0	0	0
DMRTC2	0	0	0	0	0	0	0	0	0	0	0	0
DMTF1	13.18099925	12.05960342	12.01087759	10.9897491	12.69173745	10.82546961	11.81004134	8.776455476	23.14313213	19.18894326	18.52397519	20.0758923
DMTN	0.013325795	0	0	0.026936686	0	0	0.027407215	0.039118075	0.058007189	0.131510493	0.028708125	0.041603937
DMWD	8.702574081	6.745359335	6.955539192	8.286011177	10.12313542	8.001741129	7.761643862	9.645608998	5.912472146	3.638721997	4.257767228	7.143753117
DMXL1	4.055543293	3.801427817	3.423981599	3.876718935	3.484516185	3.1004883	3.302507466	2.293433402	7.387073286	6.522079902	6.339164876	5.527981185
DMXL2	8.155056762	7.840566477	7.174577933	6.073268117	4.981446252	4.549728779	5.606065876	4.10815458	13.13225422	9.602343859	9.905621676	15.44372309
DNA2	6.988992593	8.282204341	8.27529325	12.62287229	11.89000469	12.39421478	7.596168769	11.27562182	9.448470622	11.65133897	11.61682297	10.1945197
DNAAF1	0.012481921	0.012939949	0.039633112	0.025230883	0	0	0	0.024427246	0	0.013686934	0.013445072	0.01298977
DNAAF2	9.152891891	6.307013422	9.072464656	6.699489213	8.343635301	7.665220544	8.478625115	6.877044638	4.683870038	3.823435665	4.65571565	4.157866265
DNAAF3	0.158196945	0.257717488	0.119598449	0.22841314	0.188783868	0.232089284	0.255643363	0.265365451	0.295127713	0.421282807	0.121717143	0.517419477
DNAAF4	0.793985932	0.682014979	1.032449239	0.825407281	0.639563054	0.745503657	1.003124881	0.821314649	0.296247506	0.17412758	0.244357941	0.259691352
DNAAF5	11.68034291	10.69362456	12.93187192	11.38715601	15.36425273	14.81354169	13.7132515	14.6543004	12.90622785	14.72841123	15.9684345	13.38979308
DNAH1	3.47704568	3.267124262	2.673968919	2.977890706	3.083338962	3.266545742	2.025518911	2.564472852	1.923834317	1.538654728	1.308066037	2.249713748
DNAH10	0.074990678	0.046645492	0.047622701	0.030317145	0.035236652	0.026954443	0.015423362	0.033020441	0.040804304	0.016446066	0.020194308	0.035118813
DNAH11	0	0.004014401	0	0.007827457	0	0.007953434	0	0	0.021070185	0.004246141	0.004171107	0.020149287
DNAH12	0	0	0.008062398	0.00384946	0.003976982	0	0.003916702	0.003726849	0.070462229	0.062646207	0.045128733	0.043600502
DNAH14	0.345434133	0.371179667	0.416317599	0.328742175	0.610811925	0.507523072	0.445979517	0.266459691	1.042694236	1.144642284	0.86368126	1.104706265
DNAH17	0	0.012120835	0	0	0	0	0	0	0.046653207	0.038461604	0.037781946	0.028390837
DNAH2	0	0.003257657	0	0	0	0	0	0	0	0	0.003384823	0
DNAH3	0.010950257	0.003784027	0.015453203	0	0.003811342	0	0.003753573	0.003571627	0	0.008004935	0	0
DNAH5	0	0.002460811	0	0	0	0	0	0	0	0	0	0
DNAH6	0.003786739	0.003925695	0.004007937	0.003827246	0.015816129	0	0.0038941	0.01111603	1.520619061	1.457462026	1.040129095	1.48962588
DNAH7	0.042453302	0.044011141	0.031453213	0.030035202	0.026597301	0.017439198	0.026194162	0.037386695	0.060059751	0.051206952	0.054874986	0.101615359
DNAH8	0	0.017516838	0.010730286	0.00341551	0.007057313	0	0.003475172	0	0.036775895	0.018528033	0.021840747	0.024617992
DNAH9	0.01465003	0.030375236	0.015505794	0	0	0	0	0	0	0	0	0
DNAI1	0.007246103	0	0	0	0	0.007441484	0	0.007090349	0.007885572	0.031782588	0.015610478	0.052786465
DNAI2	0	0	0	0.017347741	0	0	0	0	0	0	0	0
DNAJA1	77.54973547	62.08873152	84.29679141	105.9930285	86.19699329	78.53045493	104.2774909	101.6472605	141.7752025	119.2189682	143.6305089	135.5787488
DNAJA2	28.51129947	26.59262757	30.06464079	29.74411944	28.01966336	27.57583907	32.15176585	27.81207828	25.30223903	27.54698973	27.63069403	29.06504526
DNAJA3	32.85440795	28.7501228	34.25824196	31.04068436	35.62767687	35.58029526	36.0289421	34.66365422	21.82953484	24.1527135	24.73284423	25.37481771
DNAJA4	0.379832194	0.344548987	0.469022905	0.239934567	1.883910307	1.381511464	2.35988216	1.254378587	8.83540761	10.04809787	9.529585984	10.24450443
DNAJB1	21.00793015	22.25777143	22.00417498	37.83485256	22.61944099	22.82640454	21.66953152	34.98475037	22.41497796	20.47492599	21.57839719	27.99277175
DNAJB11	36.2442873	34.67126665	43.48080465	50.17200651	34.88831376	34.73794646	44.86369909	52.06132239	47.82315376	46.5824008	47.64445185	66.33104339
DNAJB12	14.92104566	12.79452332	16.13226718	16.78954976	14.83279936	14.01790532	14.96334012	16.71368184	12.75925326	8.16046737	11.15363976	12.45847061
DNAJB13	0.024447779	0.063362246	0.012937933	0.061773249	0.038291776	0.062767445	0.037711382	0.035883413	0.013302648	0.080423954	0.052668518	0.07632744
DNAJB14	9.767058111	8.115764447	9.726413977	8.862706457	9.733417515	8.022635944	10.79896807	7.52300003	11.62547032	10.96325337	9.925375285	12.67980585
DNAJB2	3.490493921	2.872764871	2.622732687	2.598746605	1.182440674	1.03981056	2.877045124	2.568117574	3.595555274	2.468863398	2.496988472	6.73816906
DNAJB3	0	0	0	0	0	0	0	0	0	0	0	0
DNAJB4	4.373470209	3.830669301	4.124799075	3.136483704	4.657113942	3.82435577	4.245133587	2.810604275	4.586644598	4.605759908	3.49822514	3.094359753
DNAJB5	1.371718396	1.656062987	1.084289802	1.456588898	0.688963649	1.105565805	1.124916288	1.376214003	0.888105222	1.408946015	0.879057784	1.373319236
DNAJB6	27.97918098	25.74175777	29.07655862	25.50341656	23.69942338	20.47364743	31.53460066	23.37696568	23.4330463	18.71908049	21.94057834	24.80039973
DNAJB7	0	0	0	0	0	0	0	0	0	0	0	0
DNAJB8	0	0	0	0	0	0	0	0	0	0	0	0
DNAJB9	2.39577635	2.439731091	2.782562995	2.485689345	2.25809829	1.98136396	2.354687995	1.950108456	4.637592462	3.998720405	3.722520763	3.927424741
DNAJC1	12.06177316	12.80788866	12.55977187	11.46092962	17.30235475	12.44713267	13.54778321	11.15317742	50.06231842	33.42939163	42.80378261	38.61222586
DNAJC10	22.96586717	21.36586416	22.99392688	25.09290876	18.12477375	20.31029553	22.27622143	20.81216317	16.76145703	18.10351699	16.37919403	25.28165145
DNAJC11	30.24643096	28.06993309	32.75692918	29.28178072	30.16649255	29.33352007	32.04499397	30.65158324	23.61535541	21.80635396	25.34614433	27.07265319
DNAJC12	1.15047931	1.635698132	1.078519377	0.631226758	0.75510664	0.978957421	1.115492083	0.611121343	0	0	0.035407272	0
DNAJC13	14.91624927	14.51626002	13.64485969	16.54644777	13.04297076	13.56425887	13.48862485	12.71786603	17.9767347	18.03660823	16.5291181	18.25907923
DNAJC14	19.71401329	19.83435438	20.55773067	20.41485308	19.52195976	20.72682065	20.14000705	20.91886612	20.59210781	18.9416445	20.92191225	23.84577734
DNAJC15	5.097951912	4.822834729	5.92916203	4.839026169	5.262452321	4.598402712	5.501623319	5.253912516	13.66921851	12.00917561	14.61570337	11.72023063
DNAJC16	4.619341112	4.347650282	4.326121631	3.638223505	3.231041806	3.487354693	4.112071299	3.027825339	4.544560731	3.218066882	3.724676953	4.927238751
DNAJC17	2.487149068	2.76662142	2.690077389	3.082560164	3.544848632	3.523693208	3.584464316	3.179782109	3.161039518	3.822145707	3.148392135	3.117347607
DNAJC18	2.752703917	3.640947182	2.478149245	2.206532713	3.557102305	3.509288235	2.62467868	2.280732489	2.892332571	2.636808575	2.658376844	2.458595323
DNAJC19	13.70651824	14.24347775	15.2012908	15.74220298	15.40771443	16.70273458	14.70209214	16.26754093	10.74103314	12.36898524	10.73757338	11.80720231
DNAJC2	30.28895883	24.52356708	30.27327113	30.02206758	36.44041432	36.13983187	35.47185438	30.05595491	19.01369233	19.45443015	22.11409755	17.83165939
DNAJC21	11.72948548	11.7845575	13.08856842	14.56790653	13.37532608	12.85350573	13.37801506	13.61524098	18.70875173	19.11139364	19.30487833	19.93742698
DNAJC22	0.035753861	0.074131723	0.050456506	0.15659074	0.024888947	0.048957217	0.012255851	0.069970669	1.387759359	1.110824323	1.360784183	1.128659977
DNAJC24	7.246354196	6.78706814	6.625506892	8.194032069	7.397928486	7.533842518	8.134269678	6.967737515	8.998452169	9.126009899	7.960073969	8.101174032
DNAJC25	2.609811153	2.459617359	2.611591383	3.285171119	3.047167651	3.338043573	3.318158215	3.760923452	3.279055841	3.928421749	3.501214022	3.876466983
DNAJC25-GN(	0	0	0	0	0	0	0	0	0	0	0	0
DNAJC27	0.791161942	1.235758822	0.982522006	1.151460147	0.848144186	0.996659907	1.008855247	1.094140444	1.836761641	2.128367944	1.545342423	1.84430798
DNAJC28	0.788831259	1.022222125	1.013819144	0.541004295	0.823680818	1.215151445	1.622392357	0.689174455	0.490540373	0.339815768	0.424850184	0.703651085
DNAJC3	14.94403507	12.02786394	15.17461684	14.66575295	11.69495252	10.34464128	13.76287023	11.60724434	16.10845273	13.93218858	14.36519739	18.41475434
DNAJC30	4.140830453	3.170052693	4.068055807	2.833007993	2.904684539	2.507874008	3.515770335	3.594694791	4.136514607	3.027059457	2.927820864	4.066218384

DNAJC4	11.81846239	14.25321171	10.3941514	10.74697479	9.723958227	12.72833722	11.17847004	13.61885056	15.69906703	16.96979174	13.78824669	20.12295232
DNAJC5	23.29743557	22.23327755	22.27203738	22.619177	16.67761635	17.38567794	21.03745136	22.98243814	27.24741012	21.33942383	25.53825726	40.38144646
DNAJC5B	0.507039443	0.876075724	0.335410962	0.427052757	0.397079849	0.715977673	0.499689384	0.434123084	3.563615958	5.003904538	4.210017737	3.03409817
DNAJC5G	0	0	0	0	0	0	0	0	0.028412546	0.057258005	0.028123097	0
DNAJC6	0.017028342	0.008826602	0.027034548	0.043026241	0	0.02623123	0.008755565	0.033324638	0.676384931	1.129672357	0.871259786	0.974664379
DNAJC7	52.36117953	50.87002289	56.1166067	56.82496001	58.24911995	53.63780236	60.41282398	56.77583299	52.94602606	47.93503821	57.41801721	48.0055813
DNAJC8	71.91884083	72.40873813	77.50741116	88.74180485	80.63521866	79.80674724	87.78383573	89.34590387	74.25120641	80.09885606	77.59971997	74.65462264
DNAJC9	37.1742137	41.26754511	47.65681911	75.94655978	60.16798048	60.59771922	45.78979307	76.39227053	32.50217895	29.46485838	37.37808726	37.36409024
DNAL1	0.496251216	0.533515453	0.525239131	0.582056911	0.518174962	0.478173158	0.573323497	0.569512489	2.006835407	1.968380016	1.656425779	2.269794829
DNAL4	7.092585023	7.690480799	6.549378348	8.155946602	6.574648932	5.908818367	7.070398048	8.533527724	7.876026505	8.293150127	7.05519056	9.075813463
DNALI1	0.08125264	0.063175676	0.085998915	0	0	0	0	0	0.022105797	0.044548414	0.021880598	0.021139637
DNASE1	1.862429806	1.525479009	1.143653227	1.064654158	1.167959689	0.953535662	1.144672998	1.004177947	6.535361484	4.411931523	4.298875095	7.295113865
DNASE1L1	7.606296878	8.11530804	6.736224288	5.973067147	4.955275049	5.340439948	5.906370691	6.216799491	6.045268244	5.604990022	5.720926448	8.100397071
DNASE1L2	0.870969119	0.761846882	0.576153579	0.797759175	0.682085568	0.726743512	0.783704957	0.878880442	0.888592672	1.014743919	0.967494295	0.793105295
DNASE1L3	0	0	0	0	0	0	0	0	0	0	0.053867754	0
DNASE2	11.44257096	13.27928793	10.26738388	9.506570806	9.233886229	10.32005576	11.84966244	11.93081792	20.38457255	14.60417034	20.55215787	37.84387735
DNASE2B	0.022571125	0.04679876	0	0.045625141	0	0.069539168	0	0	0	0.024750157	0	0.070468413
DND1	0.537088568	0.591597049	0.603990819	0.508906755	0.736071598	0.275785196	0.483276575	0.821162326	0.365304161	0.404895894	0.578532289	0.524007163
DNER	0	0	0	0	0	0.016888893	0	0	0.035793556	0.018033122	0	0
DNHD1	1.938805043	1.636419615	1.394673045	0.880928055	1.580152936	1.543529166	1.330358924	0.789072008	2.34143404	4.056292044	2.95334489	1.14276179
DNLZ	12.62288626	11.51575703	14.58174427	13.41403622	10.39380457	12.81509294	14.46428651	16.79811922	6.672187467	5.062037478	4.428709261	8.557472477
DNM1	37.13617581	42.92158569	35.41112277	41.32630372	42.49110692	48.50668149	32.5700636	43.84953095	26.312638	27.71945619	25.91291068	26.26936077
DNM1L	31.19428346	26.29788633	31.07071589	29.89197707	28.72118477	26.46660123	30.49735832	25.6815959	40.61582591	42.04093368	41.79619198	43.17233542
DNM2	61.43199274	61.56045388	60.39539358	68.96410998	59.98180711	57.71486527	60.78869954	69.70113008	59.48164614	51.74706891	56.95632325	74.54255522
DNM3	0.214454769	0.216395615	0.174019445	0.309228315	0.174664703	0.249602199	0.160255421	0.268601701	0.435643435	0.432691429	0.289523615	0.511826692
DNMBP	3.101184479	2.799252908	2.928636376	3.161378575	3.370789186	2.889660049	2.852328241	2.635589382	2.276578619	1.93480994	1.742234789	2.629186904
DNMT1	64.0950906	67.88581702	74.17300217	102.8115452	103.1855603	103.8900664	74.20196007	105.0554495	59.69364672	57.20197257	67.67323111	62.96419081
DNMT3A	3.950652438	4.356038361	3.577172853	3.254339555	4.117238887	4.440223577	3.984382579	3.523105828	3.959661462	3.489012375	2.804201818	4.594618946
DNMT3B	11.23841152	8.2652068	11.05843421	8.093170878	17.60843301	11.42208614	11.48533398	8.60869692	5.198151977	5.734766405	5.583352016	4.257367687
DNMT3L	0	0	0	0	0	0	0	0	0	0	0	0.032824677
DNPEP	8.092309586	8.04554197	8.720975868	8.054763674	6.41109067	7.679967884	8.725833182	8.639309782	7.737387229	8.900477281	8.610924026	9.392786546
DNPH1	20.80083245	25.31173804	24.33665104	24.67697077	26.85580463	32.13383494	23.88919142	29.39981791	20.25032238	36.06544908	31.78956875	18.37849323
DNTT	0	0	0	0	0	0	0	0	0	0	0	0
DNTTIP1	25.24399152	29.77583838	27.46306956	27.48879981	22.03403868	25.52334905	25.83915247	28.44864101	37.50790322	38.99345001	40.24065991	43.59536642
DNTTIP2	34.31792713	30.39230436	39.08442156	42.27737574	39.21929368	36.0959204	42.73127218	38.39679013	42.42354259	37.84777282	44.69567082	45.13651141
DOC2A	0.110845971	0.536263015	0.091249592	0.385886983	0.038581002	0.278262781	0.139319489	0.337441518	2.787850187	3.538371728	2.759449385	7.254606716
DOC2B	0	0	0	0	0.040553445	0	0	0	0	0	0.020917251	0.020208913
DOCK1	0.027470209	0.028478238	0.04845808	0	0.004780635	0	0	0	0	0.025101835	0	0
DOCK10	13.36942393	12.60398951	12.46660979	16.49390625	14.83463494	13.82187681	12.86549424	12.52536621	16.5728753	16.40454461	16.63100866	23.94549096
DOCK11	10.29756943	9.498562167	8.612296179	10.28188365	9.80420251	9.988551651	8.736735367	7.782915497	16.61023652	14.07045922	14.78429735	13.57479772
DOCK2	23.84895834	26.85535016	22.76255838	26.76977646	24.28271462	26.63598292	22.98871812	23.68719032	20.40824197	22.25005765	21.33330586	22.80008476
DOCK3	0.003366253	0.003489779	0.007125778	0.010206788	0.010544911	0.006914039	0.003461693	0.006587792	0.018316627	0.003691234	0.007252012	0.024522508
DOCK4	0.79292544	0.708639757	0.654030924	0.563748699	0.4510932	0.555974126	0.494867355	0.470879847	0.309454204	0.407753806	0.176712511	0.449584655
DOCK5	13.55048737	11.44428748	10.92264085	10.89289716	7.957747853	8.11310573	8.635191487	7.615092939	10.00168578	8.481101936	7.964920548	11.92392693
DOCK6	7.385160383	8.993733066	6.702298476	5.096109502	6.083183847	7.421712073	7.260807553	5.553420066	5.133968992	7.152045376	5.473876692	4.926057885
DOCK7	2.336295626	2.215347197	2.479361557	2.31091315	2.374456582	2.124171847	2.203924713	1.706937586	4.935789179	5.513086731	4.56338526	3.857745144
DOCK8	53.97371449	51.67263793	48.18226147	42.45958617	64.01661242	52.30019727	48.52884094	36.9245124	122.0326924	93.56459361	104.3098481	84.24776373
DOCK9	3.543041888	3.846737174	2.961912404	3.317545455	2.436143483	3.103302966	2.87778606	2.440783808	0.297112928	0.251748368	0.213880825	0.34870163
DOHH	7.588241681	6.478454614	7.913389356	7.962656373	6.921393278	6.096757353	8.032894549	10.43886343	2.914554478	2.643082717	3.413754425	2.787170411
DOK1	5.74802723	6.521811113	6.021329368	5.821443798	5.373424918	6.460588817	5.413354875	6.88333961	11.31603959	11.97171447	12.10343099	13.62201773
DOK2	36.28318967	34.19227537	35.19446831	27.32907803	32.5275499	29.74140687	38.916702	31.71509347	39.97507342	33.0069088	42.1539663	38.94625033
DOK3	27.77693932	28.3167435	27.34348906	22.67829476	22.57406743	22.90757713	24.09362786	25.62821806	7.247878576	4.665866537	6.092241937	6.972357984
DOK4	0.298113911	0.515088843	0.315527874	0.384998116	0.32857777	0.306152125	0.289534581	0.356529524	4.25353914	3.087332607	3.871249521	3.085196335
DOK5	0	0	0	0	0	0	0	0	0	0	0	0
DOK6	0	0	0	0	0	0	0	0	0.261483326	0.25877048	0.180249298	0.263450684
DOK7	0	0	0	0	0	0	0	0	0.021485326	0	0.010633224	0
DOLK	3.833076704	2.961316805	3.307602544	3.874079614	2.7022689	2.758008368	4.569419489	4.347927838	6.934530442	5.006269511	7.021676441	9.121417733
DOLPP1	3.956585357	2.717425046	3.428683066	3.099154828	5.474081484	4.621968501	4.984233871	5.371760151	6.189521656	5.477428133	6.099830027	5.738857739
DONSON	12.21587891	13.52610241	14.14793735	18.5306042	17.07419752	17.34004881	14.14072238	17.85693607	15.40518332	16.71478909	15.50085181	16.55118008
DOPEY1	6.068623474	5.578982035	4.654430998	5.666857899	4.64922943	4.22822387	4.652246722	4.259997879	6.807569367	5.858846855	4.772411191	8.151847057
DOPEY2	7.466496903	10.20206995	6.893022504	11.90911038	5.388658662	7.498677199	6.047983532	9.474975369	8.692999773	7.427281913	7.865227993	11.37689655
DOT1L	19.27040424	18.27459016	19.48399625	19.74326959	27.15142266	23.91614682	17.73982011	22.45562282	11.44086856	13.80764961	12.7340546	11.10897983
DPAGT1	14.05595753	13.871793	14.37895315	14.39242485	16.55694636	15.84273223	13.781192	16.97706586	16.56552975	22.47999357	21.57585851	18.03462636
DPCD	5.697482915	5.883115355	5.74313761	5.347113215	7.129585372	7.569289703	6.835520738	6.902400688	3.666042144	5.950020943	4.822024086	3.858747651
DPCR1	0	0	0	0	0	0	0	0	0	0.011162386	0	0
DPEP1	0	0	0	0	0	0	0	0	1.005527361	0.552647902	0.524785953	0.437081633
DPEP2	0.754259534	0.835251265	0.399159347	0.103953811	0.340092123	0.316880616	0.405450418	0.234833067	0.354446284	0.263160637	0.258510306	0.321115062
DPEP2NB	0	0	0	0	0	0	0.046696344	0.088865702	0	0	0	0
DPEP3	0	0	0	0	0	0	0	0	0	0.067899165	0.033349657	0
DPF1	0.524577646	1.129487278	0.619284086	0.856459348	0.316011241	0.642322541	0.580946644	0.434332367	0.680654657	0.730087872	0.912782735	0.923866348
DPF2	14.3781209	15.80469127	13.48163617	15.52677614	14.10787866	15.43971436	14.48451041	16.28204665	22.86475499	23.20598869	19.85491286	25.59705764
DPF3	0.021086904	0.026232834	0.026782404	0.034099957	0	0	0.008673904	0.020633642	0.569106033	0.439330321	0.377053171	0.610067163
DPH1	8.701636193	7.714290703	7.696321798	6.859390634	9.997200657	10.40489715	8.773864456	9.320991366	4.35230289	4.225988974	3.472480444	4.414327611
DPH2	19.14766796	15.62015198	20.70871496	18.08467155	20.02369833	20.49316041	20.35776675	20.96523352	10.23043505	9.517854597	11.63272134	12.10908665
DPH3	7.108421693	5.351982155	7.229430542	6.676063747	5.584124636	5.723161884	7.582211195	6.813146445	6.439247456	5.922210267	6.116991604	8.375727059
DPH3P1	0	0	0	0	0	0	0	0.064763749	0	0.072576106	0.035646804	0
DPH5	1.739747472	1.611205387	1.722121913	1.363145931	1.747403209	1.677756255	1.877674437	1.501312096	1.056630701	1.460756158	1.199355366	1.27254549
DPH6	1.301492677	1.142667462	1.22592493	1.309121059	1.74913191	1.585996265	1.280758428	1.163836282	0.589580878	0.785268262	0.79151499	0.706385832
DPH7	4.530630105	4.291086284	4.566095142	5.446092568	5.45258052	5.508084711	5.035919937	5.598601108	5.483322322	6.45660001	5.876012366	7.315132428
DPM1	45.10900998	42.9250953	46.04391523	52.40096676	50.96878748	48.79965868	55.19751821	47.76310413	43.06896684	42.41856763	45.03326395	46.15497964
DPM2	17.78569573	18.61876248	19.78243439	22.05659813	18.93487845	20.23118988	19.50689941	25.40691108	16.66599813	17.28912133	21.48257193	21.08108611
DPM3	7.892068926	9.948168369	7.403861569	10.78639177	8.147085801	8.104867256	8.208049902	10.96936007	30.74308496	38.15616208	35.54984491	32.10603533
DPP10	0	0	0	0	0	0	0	0	0	0	0	0
DPP3	27.14040452	26.38120319	28.94745647	29.35352596	22.89024297	23.96472044	26.77913411	30.7412491	17.20849202	14.75587824	17.40919156	17.21925271
DPP4	0.013783187	0.028577931	0.02917663	0.055722509	0.028784221	0.014154831	0.042521902	0.013486918	3.089908031	5.15381446	4.275863385	1.061454537
DPP6	0	0	0	0	0	0	0	0	0	0	0	0
DPP7	40.87634963	47.47085939	43.13351645	47.46493125	43.58395156	52.97993525	47.74429082	64.8453909	55.08996136	59.9513303	57.65162959	70.25005154
DPP8	12.52638088	10.61923018	11.71844461	12.25197791	12.12340937	11.812874	11.43408788	10.22745241	13.63183456	13.24090653	11.91152909	13.95416866
DPP9	24.06697375	22.1414375	24.72977255	28.41180344	21.9089281	21.46648464	24.53578833	29.79021799	18.0882819	16.93583843	19.89571158	21.37260435
DPP9-AS1	0.035043674	0.036329614	0	0	0.036591858	0.017994288	0.018018615	0.017145206	0.01906814	0	0.037747772	0.018234744
DPPA2	0	0	0	0	0	0	0	0	0	0	0	0
DPPA3	0	0	0	0	0	0	0	0	0	0	0	0
DPPA4	0	0	0	0	0	0	0	0	0	0.25598427	0	0
DPPA5	0	0	0	0	0	0	0	0	0	0	0	0
DPRX	0	0	0	0	0	0	0	0	0	0	0	0
DPT	0	0	0	0	0	0	0	0	0	0	0	0
DPY19L1	8.607064707	8.369215433	9.544673026	12.18709447	10.73544291	9.60913872	8.301859506	10.04013655	13.3234286	12.287416	11.00818766	16.18292286
DPY19L2	0	0	0	0	0	0	0	0	0.254048838	0.301157965	0.221877135	0.285818037
DPY19L3	18.89290285	18.24318344	18.24807971	17.4078234	19.1374651	18.75051915	17.35426739	14.76902304	8.837196978	8.199024388	8.484941592	7.998550101
DPY19L4	7.726542712	8.085876012	7.197565748	7.505366767	9.764608972	8.78593732	7.862055409	6.829060159	11.2200556	10.20081664	9.031628896	10.10398371
DPY30	7.228654127	8.211786904	7.525775558	8.961774487	8.835400538	8.377527146	8.788322331	9.172120282	7.95848252	10.22298011	9.532935567	8.384015928
DPYD	5.048813984	5.75419832	4.597628043	5.532870923	3.468157168	4.682758085	4.003358658	4.207014337	7.23598432	7.576626849	5.8146404	9.279175495
DPYS	0.038614683	0.006671944	0.013623438	0.006504625	0.006720105	0.006609312	0	0	0.007003738	0.014114191	0.020797167	0.026790527
DPYSL2	30.30062917	36.84500228	28.19907617	21.15382632	24.74064496	29.11277548	25.13698002	22.52101574	35.42344059	34.39762362	39.21296851	24.65790734
DPYSL3	4.787645944	8.043378266	4.811577067	8.483833053	4.260964606	5.569239629	3.837479628	6.278421688	0.253203839	0.480827265	0.221706167	0.288702123
DPYSL4	0	0	0	0	0	0	0	0	0.012981214	0.026160219	0.03854691	0
DPYSL5	0.080133374	0.093458134	0.116619619	0	0	0	0	0	0	0	0	0
DQX1	0.550305512	0.35656198	0.291225472	0.034762013	0.143654327	0.264911115	0.247584644	0.033654796	0.280720312	0.264001675	0.185240344	0.125277179
DR1	33.08390887	29.06545699	33.59663914	35.13258165	37.53957503	33.16660742	34.59094633	30.2889703	62.64583873	59.42727887	59.93048699	57.56945069
DRAM1	9.352439364	9.878279667	8.966372391	6.514348981	3.679353798	4.116263353	7.337760324	5.40177382	3.898557905	3.300381103	3.083910563	9.702401299
DRAM2	35.19855839	33.31924365	30.13242911	25.94412722	31.7417332	29.01959368	32.85752426	27.53515622	62.47579678	69.96933241	64.01891712	53.18303816
DRAP1	26.23550729	27.47576143	29.41143631	35.44506013	25.27008257	30.68192625	27.52993427	39.13605717	35.65935654	41.86081484	36.33420229	40.0628938
DRAXIN	0.054930136	0.06833498	0.058138813	0.033310638	0.045885503	0.056411248	0	0.053749416	0.13151099	0.048186504	0.106503743	0.114330132
DRC1	0.021549684	0	0.022808483	0.021780204	0.045003443	0	0.02216066	0	0.046902889	0.023630106	0.046425073	0.022426471
DRC3	0.009405622	0.039003057	0.019910079	0	0.00982115	0.009659231	0.009672289	0.018406897	0	0	0	0.019576612
DRC7	0.389155273	0.311745585	0.280832255	0.089390481	0.09235174	0.127160819	0.072761562	0.121160585	0.481248003	0.368534883	0.419183919	0.478623067
DRD1	0	0	0	0	0	0	0	0	0	0	0	0
DRD2	0	0	0	0.019776425	0	0	0	0	0	0	0	0.020363236
DRD3	0	0	0	0	0	0	0	0	0	0	0	0
DRD4	0.315830144	0.409274554	0.083569745	0.438911852	0.329783117	0.243259539	0.162392276	0.618082798	0.128888313	0.389610689	0.127575283	0.205425161
DRD5	0	0	0	0	0	0	0	0	0	0	0	0
DRG1	54.29199141	52.01388451	55.93155902	57.19819456	57.19428605	58.53797542	61.55560399	59.73383937	55.69092172	59.28922305	61.47937947	58.31609465
DRG2	21.79857451	20.44880376	23.86749505	22.03175664	20.8670625	21.8805862	23.37617641	23.79019594	9.08273544	8.299266527	8.655167695	7.687710101
DRGX	0	0	0	0	0	0	0	0	0	0	0	0
DRICH1	0.108299864	0.096234828	0.049125458	0.093821452	0.096929497	0.063554294	0.031820108	0.090833114	0.067347047	0.03393006	0	0.064403564
DROSHA	22.53318644	21.86134499	22.64917558	22.20552733	23.81792381	24.98988884	22.25513509	20.21919883	40.95121355	39.6684814	39.0514181	39.60282485
DRP2	0.088733644	0.107321383	0.101743322	0.291469228	0.162144121	0.167064737	0.212915309	0.253243437	0.490868996	0.729750404	0.597379109	0.577149569
DSC1	0.01259882	0	0	0	0	0	0	0.012328009	0.013710667	0	0	0
DSC2	4.439161502	4.813161683	3.70704953	2.963657248	3.210674207	4.454296187	4.428907476	2.630155738	4.243673123	2.378038171	3.684982484	2.574783807
DSC3	0	0	0	0	0	0	0	0	0.387903768	0.49815357	0.414065944	0.254573511
DSCAM	0	0	0	0	0	0	0	0	0	0	0	0
DSCAML1	0	0.005637607	0	0	0	0	0	0	0.005917964	0	0	0
DSCC1	9.533470016	9.735792063	12.47489304	18.04849359	21.27124726	17.51091658	13.09607102	18.37863342	16.5945171	17.70911368	17.77935026	19.69463256
DSE	0.064344512	0.072769808	0.024764771	0.041384518	0.097726794	0.126151715	0.096245536	0.062961432	1.145829576	1.007031935	0.913626158	1.004437255
DSEL	0.023191277	0.012021145	0.018409476	0.035159036	0.00605396	0.017862448	0.011924398	0.005673196	0.006309477	0.031787725	0	0
DSG1	0	0.007681647	0	0	0	0.007609537	0	0	0.008063654	0	0	0
DSG2	5.272222214	7.076029701	5.212693008	5.614906182	6.478321662	7.291115012	5.431096457	4.434683724	13.30462692	15.13934618	12.7557086	11.0210093
DSG3	0	0	0	0	0	0	0	0	0	0	0	0

DSG4	0	0	0	0	0	0	0	0	0	0	0	0
DSN1	12.185717	17.10979021	16.35949975	27.91642345	24.57753885	24.41383787	17.2359045	26.1277344	20.05451632	20.98089592	21.99182638	23.62766411
DSP	0.016479041	0	0	0	0.034414127	0.005641125	0.005648751	0	0	0.036139878	0.005916875	0
DSPP	0	0	0	0.02511437	0	0	0	0	0	0.01362373	0	0
DST	3.816587086	2.75157907	3.002146373	2.525822147	2.455897688	2.302108603	2.523904519	1.653715613	5.13302604	4.179783338	4.248871544	4.266392602
DSTN	20.4759919	21.60108687	21.01071789	24.72715571	17.69168961	18.43660677	21.03181236	22.62265165	15.29994658	16.81321609	17.62926315	16.03184196
DSTYK	2.909902225	3.170665888	2.536792643	3.3845726	2.509724004	2.60701787	2.659142948	2.920024381	4.95945088	3.916350568	4.261676078	6.393851687
DTD1	9.238511589	10.49572105	10.54253814	10.43908782	11.6812767	11.27878693	12.33095663	11.67991016	4.908276295	6.454770757	6.340708022	4.452685296
DTD2	5.3187369	4.099019759	5.140832926	4.868497003	6.014774788	6.142341161	5.882328551	5.008018173	3.691288864	3.98351054	4.475222923	4.135688911
DTHD1	0	0	0	0	0	0	0	0	0	0	0	0
DTL	15.79530277	21.25052771	20.32380215	30.53731275	30.86620765	32.03009739	21.397995	27.27491278	19.2614348	19.40817515	21.95650126	21.15328261
DTNA	7.905646305	6.8124545	7.385225953	5.024303354	5.955475906	5.630621909	6.762785969	4.216357363	5.186011393	5.50604817	4.376711099	5.283013482
DTNB	0.162095769	0.310234949	0.263945227	0.264648022	0.371063321	0.460984013	0.378261488	0.500236372	1.214451166	1.558683507	1.463984637	1.563635108
DTNBP1	7.29114038	7.395436678	7.617038647	8.944824335	7.531037101	6.808502504	8.339950675	9.64610276	3.204680735	3.142755672	3.087219803	3.27766455
DTWD1	2.574400178	2.873799691	2.642064096	2.764560044	3.422570476	3.479449634	2.82703378	2.474080357	2.801591111	2.228101962	1.995605859	2.808317268
DTWD2	0.882270044	0.914645255	0.943239174	0.936743547	0.716525918	0.860298626	0.888955165	0.732501818	0.998923564	0.850178961	0.959948719	1.020185305
DTX1	0.013692	0	0	0	0	0	0.014080195	0	0	0	0	0
DTX2	6.613465581	6.124986289	6.632661682	4.300331337	4.64802696	4.678259707	7.133884986	4.672466566	3.070093409	2.611710596	3.101088142	2.815587092
DTX3	0	0.017525643	0.0178928	0	0	0	0	0	0	0.037074693	0.018209772	0
DTX3L	12.23029374	10.05816341	9.06426027	10.87137646	10.96850473	11.14214863	9.123395397	8.571619226	19.02534219	12.89953468	12.73187908	18.30061101
DTX4	4.606693476	3.741724782	3.14018634	1.119141861	0.450129867	0.633681036	1.355998459	1.050411859	3.477068987	2.901094749	3.02282212	3.624182583
DTYMK	16.18243411	17.47355577	20.8128914	28.95210446	25.20143174	25.72049285	21.23893944	32.48221093	26.73873356	26.89905301	31.49536691	25.77596373
DUOX1	0	0	0	0	0	0	0	0	0.010175197	0	0	0
DUOX2	0	0	0	0	0	0	0	0	0.027639203	0.046416279	0.045596054	0.017620799
DUOXA1	0	0	0	0	0	0	0	0.012536412	0.027884884	0.042145982	0.041401217	0.026666142
DUOXA2	0	0	0	0	0	0	0	0	0	0	0	0
DUPD1	0	0	0	0	0	0	0	0	0.010267974	0	0	0
DUS1L	35.330436	31.18396577	36.32650772	39.64764528	31.95968307	33.71664962	36.63862415	42.42296015	30.88842021	31.65168611	33.24092592	39.29705428
DUS2	6.989456938	7.053992163	8.157108032	7.06422405	8.313229561	8.770369553	7.8778239	7.926247275	4.483169788	5.989261224	6.107792429	5.58784711
DUS3L	21.37640089	19.02509961	21.79109724	25.22731959	25.24026712	24.40648501	22.55751057	28.40323295	11.03672393	8.55660979	12.59441837	10.05176266
DUS4L	3.408653457	3.933780693	3.562385125	2.881763914	4.566576462	4.733465574	4.254855538	3.440271014	3.826117195	4.866097805	4.503000882	4.328201743
DUSP1	39.69252686	41.2041808	45.97867819	30.44387155	11.88139813	6.962162517	25.12500953	15.81668993	3.587561203	2.157274556	2.749171715	2.10272545
DUSP10	5.547113875	5.93617257	5.370606693	3.772082953	3.142990755	3.150239918	3.351655062	2.982832163	3.790290986	3.293593342	3.297346325	2.786643541
DUSP11	13.78890516	13.44400736	14.97660082	15.59550289	13.84958307	14.53157879	14.38241703	15.29151549	13.18364665	11.70007423	12.0591464	13.05160424
DUSP12	19.94340368	20.41516808	23.36524361	27.04468369	29.33762912	26.96469929	27.17313605	27.32879263	14.01394186	14.53332297	14.63679385	14.35869201
DUSP13	0	0	0.014477067	0	0	0	0	0	0	0.01499857	0.014733529	0.028469191
DUSP14	6.128024206	5.728311921	6.850366529	6.054394969	7.171635526	6.752877534	7.116038863	6.670044663	5.469933161	4.983094955	6.52671775	5.374174553
DUSP15	0.025783211	0.013364667	0.013644653	0.013029509	0	0.013239209	0.013257108	0.025229003	0.02805858	0.042408511	0.041659107	0.026832247
DUSP16	3.058665986	3.247414582	3.063750655	2.287459088	1.6183031	1.776890698	2.200926366	1.693046004	0.767450489	0.854223646	0.688968742	1.416613412
DUSP18	2.056071707	1.935195775	1.842112752	2.278580886	1.459519656	1.528066979	1.650688769	1.994228557	3.395534933	2.590773601	2.729552326	3.697597812
DUSP19	0.880320014	0.86077005	0.847038949	0.687523976	0.898320261	0.760229398	0.967002376	0.851608991	0.903576006	0.658163685	0.765064317	0.572585878
DUSP2	21.28467074	28.79080503	22.0217421	17.79603572	12.23623615	13.90721541	13.21895677	15.0353362	0.520517841	0.852285407	0.901626501	0.684430986
DUSP21	0	0	0	0	0	0	0	0	0	0	0	0
DUSP22	8.205393838	7.366695499	7.557773108	6.696528449	5.564903919	5.473156505	6.414809224	6.279395366	2.034016242	1.833773974	1.564510982	2.42085787
DUSP23	20.42647949	22.40959189	21.75960072	21.31310661	19.32715432	20.97725024	20.25783787	26.71456928	13.88420591	11.23547669	15.45170724	17.81783145
DUSP26	0	0	0	0	0	0	0	0	0.057325753	0.144406203	0.226967024	0.109640526
DUSP27	0.349076921	0.17424161	0.123155942	0.130670756	0.026999903	0.039832142	0.066476655	0.050603487	6.978590943	3.785239777	5.180623201	2.542958164
DUSP28	0.79323841	0.841695905	0.958102634	1.075253073	0.877005182	0.690036944	0.815728281	0.977083477	1.208538431	0.839118747	1.186174303	1.427651427
DUSP3	33.04027136	28.79878966	30.59035852	26.90231896	30.72417373	29.19875758	27.10376127	26.40310598	30.28777391	34.2402871	30.28857586	34.00418336
DUSP4	1.145677915	1.607563816	0.936241008	0.850946389	0.294900013	0.492517456	0.465784241	0.568347117	3.264834495	2.582683052	2.956058366	2.096215465
DUSP5	3.04381614	3.905771179	2.861156339	4.087492893	3.044933312	3.628653529	2.539113716	4.040612379	4.053676814	2.730819541	3.347471809	3.94296044
DUSP6	69.6592106	78.7550207	70.27251867	61.51371058	57.37838906	49.61233511	58.41568238	52.21867737	17.40361749	16.80625331	15.99708876	17.43470871
DUSP7	32.41880498	30.12515011	31.91820479	20.056107	36.7168137	28.03165765	27.45376953	21.67966013	32.14821161	30.31383931	38.93378116	27.60996712
DUSP8	0.423016022	0.378738007	0.325618903	0.126318955	0.080309878	0.029619685	0.257050985	0.141110224	0.104624365	0.168674288	0.10355852	0.040020653
DUSP9	0	0	0	0	0	0	0	0	3.452917916	3.784418493	4.377107966	2.394435674
DUT	21.89400478	26.46590961	26.19086689	34.17822185	40.06378173	39.43750731	25.51556548	37.36448896	27.01990851	29.2553452	30.98355712	30.40287287
DUX4	0	0	0	0	0	0	0	0	0	0	0	0
DUXA	0	0	0	0	0	0	0	0	0	0	0	0
DUXB	0	0	0	0	0	0	0	0	0	0	0	0
DVL1	11.80757563	12.11353931	13.55576471	11.50830597	13.97800044	14.28808074	13.42333384	15.10745906	7.828133484	10.75430233	10.33748024	9.896121021
DVL2	15.29005095	20.32717167	15.23902338	20.28624492	17.00880211	18.35907808	14.14569444	19.41314864	10.91171766	11.50350534	12.20347541	11.97589183
DVL3	8.54242572	9.5180839	9.05627973	11.71984066	9.447532407	9.580113414	9.037259351	11.36715319	12.66496089	12.62290294	11.32619777	14.38322878
DWORF	0	0	0	0	0	0	0	0	0	0	0	0
DXO	12.92848047	13.30262459	14.22966435	14.63088119	13.29765456	13.83994708	11.60413946	16.31010422	15.01673698	16.43920556	16.84327518	16.84328773
DYDC1	0	0	0	0	0	0	0	0	0	0	0	0
DYDC2	0	0	0	0	0	0	0	0	0	0	0	0
DYM	8.012106525	8.89130349	7.760746157	8.313211385	8.847225518	9.008957225	7.564012993	7.653893884	5.390719447	7.90767252	6.185808023	7.594798205
DYNAP	0	0	0	0	0	0	0	0	0	0	0	0
DYNC1H1	24.40942771	21.80404478	21.23615911	23.08904508	20.22959842	20.10417101	20.47092508	19.1151426	44.04936673	39.42677232	40.45597573	43.15507887
DYNC1I1	1.317785164	0.858217257	1.207005569	0.350045826	2.725544863	2.03865114	1.164036499	0.520743247	0.487219407	0.370514125	0.491355102	0.562626299
DYNC1I2	24.38137704	25.34773153	22.76891706	24.59560516	19.61123807	20.64871525	21.69565147	22.19991775	17.32923153	17.49915599	17.1278696	17.90285995
DYNC1LI1	19.5939278	20.88082237	19.71270896	26.19176091	22.72551409	20.81462848	24.07101469	24.12056623	21.20845254	21.00038226	21.35550632	20.76388301
DYNC1LI2	16.28340262	14.59321293	14.55473625	17.06799809	16.39640659	15.58934154	16.41064551	13.60361649	33.30477172	29.20289885	27.46081147	32.22384293
DYNC2H1	0.674728036	0.589684162	0.568821976	0.46784646	0.311306896	0.294088611	0.290452146	0.234149495	2.787675283	2.568025916	2.176152029	2.55969305
DYNC2LI1	5.546825796	5.047751151	5.115745354	4.398402764	4.935201073	5.861235247	5.539019125	5.096007647	5.02704295	5.573830523	4.764501991	5.902436139
DYNLL1	125.555642	127.7252299	138.793465	179.1836551	131.3309318	132.8722002	148.4602065	187.7098298	168.4144995	151.8882929	171.823465	159.8017357
DYNLL2	21.56624975	27.15902377	25.18345717	40.9139409	24.99241963	29.15515652	25.1226025	35.18267287	27.93251947	28.99820763	27.00055709	53.93849257
DYNLRB1	30.01659229	29.66207904	35.03441306	32.00764784	28.58223021	26.72524736	36.10662182	37.85944949	28.17029767	31.97831752	30.46400699	28.42932838
DYNLRB2	0	0	0	0	0	0	0	0	0	0	0	0
DYNLT1	39.67846014	40.91797801	38.97544801	44.74640004	32.85437643	34.81480378	42.59304643	42.77624707	56.81590271	69.91980194	59.53634244	77.29731573
DYNLT3	0.16997605	0.226560061	0.154204282	0.245420436	0.481746017	0.872796051	1.648069079	1.52066219	7.531183783	10.49084995	9.023821192	9.223645341
DYRK1A	5.10535716	4.758746234	4.982770623	5.607471624	5.727185016	5.345092214	5.008506379	5.119403107	11.30847953	8.976987936	9.10548678	9.333149806
DYRK1B	0.832192404	0.621165599	0.827955682	1.026135257	0.729924387	0.615334522	0.701745092	1.026023657	0.362253369	0.237258542	0.322706671	0.363741687
DYRK2	5.820115315	6.184094794	5.368669315	5.431185042	6.922111042	7.134633508	5.663777942	4.559286026	11.95698519	11.8094413	11.09585243	9.436711668
DYRK3	1.346695784	1.389401178	1.363687099	1.799533313	1.581964972	1.436200102	1.331612751	1.4951379	0.190238592	0.092294172	0.118559615	0.437965158
DYRK4	5.057817731	5.185155959	4.193390164	4.799526628	5.486648221	5.223051175	4.709990791	6.076395384	1.253727697	2.002759443	2.058170156	1.813019179
DYSF	17.06765509	15.52353812	15.29111005	14.19535578	12.26959852	12.98570941	12.98900746	13.22089118	0.030176891	0.129228854	0.067206304	0.028857974
DYTN	0	0	0	0	0	0	0	0	0	0	0	0
DZANK1	0.826717157	0.907468767	0.797802178	0.737259403	0.736293339	0.873979236	0.808481892	0.721707558	1.975756828	1.146492588	1.457991341	1.619489167
DZIP1	3.545411754	3.84008705	3.640497391	2.941545689	4.199332952	4.15727113	4.537007618	3.165000854	5.873821483	9.240512959	6.868479696	6.518863823
DZIP1L	0.400053167	0.604611136	0.377508583	0.915837665	0.62407408	0.856329213	0.312264025	0.754610223	1.431960245	1.765268813	1.339494764	1.855929109
DZIP3	3.340783162	2.414668143	3.041080581	1.958897068	2.00603736	1.807095565	2.858546587	1.389105692	2.266472825	3.262485154	2.444830154	1.203135899
E2F1	10.52275857	11.7973087	12.8249226	17.48382457	17.5219572	17.06934165	12.95252818	18.17501081	17.97955374	18.11652827	21.44583035	22.5654822
E2F2	2.226291714	2.466835532	2.69977171	3.079093298	4.178716682	3.489647049	2.428444553	3.49255605	3.815605025	3.370266609	4.66023748	5.543609583
E2F3	10.67953233	9.765625032	10.98745137	11.98235465	11.97413359	10.21956783	11.50411633	10.28642187	4.729355574	3.619860108	4.745477846	5.746503375
E2F4	59.52362375	54.8012586	64.79056883	67.30812057	52.57479015	54.60259596	65.58534898	69.57708386	54.96110699	49.28270124	57.02327608	70.82527396
E2F5	5.769451838	3.757012507	5.308637481	2.25628124	7.265534306	5.597503346	5.664699298	2.723427327	4.322457553	5.245539745	5.340221619	2.685292024
E2F6	3.236274111	2.578303053	3.359233861	3.397101036	3.151067802	3.323535605	3.723967979	2.98342835	3.227442132	2.624445194	2.981592182	3.541218318
E2F7	5.740058619	6.818702289	6.754773163	11.17980039	8.7524733	9.057212089	4.706933367	9.103203376	13.57652537	11.17043394	12.96722762	12.1408401
E2F8	7.586443626	11.17929528	10.48149122	9.201116138	12.40579617	12.9247145	7.634358509	7.144997071	11.21924311	10.71051206	12.7101319	9.445937115
E4F1	10.55710069	10.17239866	11.39055626	12.34486872	10.63466396	11.14769804	11.25850474	13.17234158	9.300429063	8.33002932	9.767249025	10.50221563
EAF1	10.69035402	10.59932032	10.61481417	9.829437081	9.849388436	9.408641444	10.03458616	8.773719872	11.55118551	10.92585533	10.42913588	11.92642274
EAF2	3.734986273	4.192487844	4.075845287	4.230536093	3.119994563	4.007639767	3.1654153	3.843739382	5.129804233	5.310111239	5.882183425	7.747095148
EAPP	14.13594663	13.37911862	14.96168086	15.2820113	14.13235027	14.29246616	13.77755704	14.55446799	17.55557503	16.07029708	16.22809894	18.12565984
EARS2	7.639260029	6.395158142	7.617323782	7.153081495	10.27365814	9.146643331	8.56889847	7.849392336	7.10348997	7.039624311	7.147021891	6.494429059
EBAG9	8.862751201	8.362930513	8.921007088	11.00499882	13.06933266	12.53808724	9.932430134	11.9110658	11.25893211	10.51170367	11.37802855	10.42803024
EBF1	0.00216677	0.00112314	0.005733348	0.003284922	0.001131247	0.002225194	0.003342303	0.002120195	0.027116854	0.021383562	0.019838708	0.036078859
EBF2	0	0	0	0	0	0	0	0	0	0.060554571	0	0.022988054
EBF3	12.9228894	11.02105009	13.29872893	6.659830496	10.98308214	12.24157335	11.68993564	6.597885485	5.634241885	6.260589634	5.896465385	4.174095521
EBF4	0.035227208	0.073039767	0.074569926	0.089010096	0.091958754	0.271327947	0.126790892	0.051705003	0.287520084	0.560106991	0.360481954	0.348274657
EBI3	3.772057914	2.992710391	2.97392921	1.517182815	0	0	1.543684941	1.167554205	0.041887165	0.084412553	0.082920892	0.040056437
EBLN1	0	0	0	0	0	0	0	0	0	0.100862178	0	0.047862307
EBLN2	2.884371934	2.59151965	3.052859034	2.623703786	2.442904288	2.435541354	2.636577386	2.132459277	2.44138058	2.705977859	2.071293758	2.001151835
EBNA1BP2	42.56919583	44.13128751	50.60542504	53.72274267	54.23634338	55.76527396	60.18794752	60.66951303	42.18904879	43.33695217	51.21950792	49.6896441
EBP	30.4161217	43.29657846	35.73700884	41.50570776	39.91836515	47.71408652	38.45244164	43.96087188	40.33788321	68.64294544	73.29124672	25.31324468
EBPL	49.04031269	49.22760643	47.78985791	42.96323531	51.53163034	58.02880524	58.32049485	45.3992012	19.63224803	25.29570962	22.00087788	14.94382946
ECD	31.37355389	24.23839192	31.37766675	27.98414925	28.45418783	26.25818023	33.82646393	25.9207481	22.03454964	20.45956474	21.95895023	22.41394281
ECE1	5.166690396	5.576836792	5.363951586	7.280025393	7.013432645	6.546671101	6.117966175	8.066708166	8.475319612	6.531972534	8.037050691	9.741689264
ECE2	0	0	0	0	0	0	0	0	0	0	0	0
ECEL1	0	0	0	0	0	0	0	0	0.444848638	0.346365457	0.420302409	0.522089181
ECH1	74.69035565	84.17065769	79.27767325	75.953548	60.57696871	73.98297769	68.92371206	71.77116166	95.50801743	103.8678448	125.3997463	86.8846999
ECHDC1	24.80249501	23.62594348	26.15661818	29.72422972	30.1834735	27.80248757	29.01301508	30.11239791	20.5538022	15.42549936	19.71806087	20.86575761
ECHDC2	0	0.007593809	0.015505794	0.014806743	0.007648625	0	0.007532694	0	14.46817813	11.3173378	12.14307949	13.14975504
ECHDC3	0	0	0	0.03280167	0	0.033329589	0	0	0.529779165	0.854104333	0.734134968	0.574174475
ECHS1	96.36324669	85.46009915	102.8921203	92.15605695	69.96926391	75.2190892	99.0953345	100.9478265	49.17307365	54.16503891	56.43524796	50.68974816
ECI1	24.67764827	31.26246211	29.04537657	38.5508569	33.84038395	39.17130507	29.16810942	45.38872794	22.78813239	23.69157562	25.03769121	29.94422564
ECI2	0.107475821	0.222839368	0.075835927	0	0.037407988	0.110373752	0.073681981	0.070110424	13.21654404	17.00991617	12.6188263	12.82531372
ECM1	26.72388769	33.33045012	27.46803796	29.5937224	37.37391715	41.58776779	29.49059373	30.2559633	80.23872337	85.29319017	89.86532181	66.96775531
ECM2	0.010824695	0	0	0	0.011302917	0	0.011131597	0	0.011779975	0.011869719	0	0.011265117
ECPAS	16.43607461	14.99645504	16.34715288	15.34185274	18.54207476	16.95195297	16.04058589	13.38115205	15.20935325	14.2190127	15.1433768	13.48246704
ECSCR	0.77834466	0.75311254	0.988572867	0.524447142	0.866912997	0.746042835	0.9071339	0.40619424	1.185847057	0.796587503	0.950191863	0.648010401
ECSIT	14.11058853	12.45099143	13.77637371	15.99563782	18.81130311	18.31888758	17.27893093	18.84390123	6.502896056	7.752636322	8.125472476	6.095537092
ECT2	5.360672461	5.527344315	6.276971409	10.22101445	10.58986512	8.619877669	7.201220017	8.818018691	17.1859182	20.2824227	18.51944579	13.6403708
ECT2L	0.009185654	0	0.019444446	0.009283914	0	0	0.009446085	0.008988209	0.019992577	0.020144887	0.019788905	0.019118777
EDA	0	0	0.004999366	0	0	0	0	0	0.179910375	0.155383711	0.122110331	0.181878446
EDA2R	0	0.011407796	0.046587142	0	0	0.011300707	0.011315985	0	0	0	0	0
EDAR	0	0	0	0	0	0	0	0	0	0	0	0
EDARADD	0.285367244	0.536207998	0.320913915	0.288419853	0.204857403	0.311378123	0.44018695	0.226877053	1.630392308	1.271224505	1.440877618	1.466328475

EDC3	10.77057193	10.59348439	10.87269955	11.31246516	11.28031081	11.10545124	11.02028087	10.77208371	11.69751544	12.74807847	11.99810764	13.31536882
EDC4	20.83708416	22.68005447	21.54522178	27.52606226	24.80581174	26.23464713	21.64639076	28.356628	30.06420597	27.39883024	29.74588367	33.44103853
EDDM13	0.129661592	0.067209785	0.205853426	0.0655243	0	0.066578866	0.400013262	0	0.776073286	0.284358431	0.139666756	0.269874211
EDDM3A	0	0	0	0	0	0	0	0	0	0	0	0
EDDM3B	0	0	0	0	0	0	0	0	0	0	0	0
EDEM1	51.85850878	70.43847195	42.93153365	33.94469327	32.5325369	49.30405474	31.45392091	27.0915956	56.85636641	50.37182127	65.70574053	36.27129579
EDEM2	0.337540371	0.437408168	0.44657173	0.341151084	0.205597277	0.259981248	0.23140687	0.440379955	0.24488555	0.308438967	0.302988519	0.409819437
EDEM3	32.3493412	26.83044813	28.54371072	26.44044899	25.23756912	26.39568712	26.66684154	21.3412401	21.09758481	20.5817859	18.4915648	30.96487574
EDF1	102.9486705	117.7493095	110.7502839	120.8224521	102.4643835	114.2967933	119.1519958	141.7691034	155.9390705	145.5223305	161.8967254	159.9820803
EDIL3	0	0.011719353	0	0	0	0.01160934	0.023250071	0	0.086115084	0.161146401	0.097414628	0.164702644
EDN1	0	0.020554208	0.020984813	0.02003875	0	0.02036126	0	0.038800979	0	0	0.04271312	0
EDN2	0	0	0	0	0.044311355	0	0.087279445	0	0.046181591	0	0	0
EDN3	0	0	0	0	0	0	0	0	0	0	0	0
EDNRA	0	0	0	0	0	0	0	0	0	0.011338273	0	0
EDNRB	0	0	0	0	0	0	0	0	0.115842249	0.081707343	0.091729699	0.110779226
EDRF1	14.05947826	13.04609679	14.3042519	15.28794385	15.77069358	15.44076499	14.81983619	13.76837434	10.01491912	9.593499077	8.739480834	9.612632571
EEA1	9.98059631	7.80385687	8.496212069	7.627828157	9.025665192	7.384055486	8.5218294	6.057753693	9.456042717	8.780921122	8.772544391	8.752360455
EED	6.896290393	6.423460122	8.22200722	9.306767128	9.283995944	8.28974716	8.083580871	8.428605271	6.742897757	5.867117999	7.286126264	6.874404473
EEF1A1	8484.627819	8401.514415	7875.311244	6532.956436	8145.91791	7869.690368	7820.299322	6984.713786	3336.003212	3924.294918	3412.858389	2838.866728
EEF1A2	0.509407901	0.528100803	0.454033134	0.216781938	0.839862461	0.550677215	1.654265108	0.288581072	12.42940583	19.9033191	15.45067835	13.39286071
EEF1AKMT1	27.92777588	24.1271629	29.99224415	33.80979113	27.77294229	28.15551881	28.03578373	36.13632533	4.119116942	4.206585624	4.187347413	6.387707092
EEF1AKMT2	7.439245299	9.236387363	7.920482427	10.59470634	12.6343539	11.64093267	10.35644535	10.17093452	8.687741417	10.39831187	8.963476577	7.940827458
EEF1AKMT3	5.4567442	5.637339059	4.772803504	5.036373008	6.706804484	7.160510124	4.403432373	4.449543239	4.763013162	7.250889665	5.632897961	5.166095597
EEF1AKMT4	5.81578082	5.240326584	6.040446502	5.43851686	6.186223284	5.860957727	5.701199115	6.328988354	1.774492638	1.311208319	2.81026442	3.054485391
EEF1AKMT4-[illegible]	0	0	0	0	0	0	0	0	0	0	0	0
EEF1B2	707.3581413	705.3562691	696.5728399	589.8432804	687.1149312	713.2423865	685.4165186	671.5447469	255.1966048	319.9004067	325.4985411	182.2971313
EEF1D	36.72212936	35.90090899	36.00645475	36.5790788	37.78482474	38.86638443	37.20618871	42.85205123	34.91419268	32.40595819	35.75039642	34.58173404
EEF1E1	24.72214362	23.97149005	27.76733996	31.5390295	37.187086	35.33118475	29.64542729	33.19883432	9.077705713	9.431221281	12.57000802	11.19977977
EEF1G	1853.141099	2013.777777	1769.165201	1487.423753	1622.001087	1778.071725	1771.804732	1697.629005	807.7460491	1010.478684	928.8947151	722.1236415
EEF2	2479.073603	2518.446421	2210.193528	1594.976813	2206.942013	2317.194568	2210.200149	1895.85555	619.7570063	778.7157423	723.1998129	600.3301837
EEF2K	8.601528316	8.820882759	7.916829796	7.668222126	8.28777659	9.89728606	8.037264161	7.493884647	10.23136833	8.88355822	9.526897123	10.2600213
EEF2KMT	6.850854646	6.090852933	7.847643516	8.195025405	8.081662817	8.393711877	9.274548686	9.249265411	5.025345993	5.848137022	6.281909059	6.858849817
EEFSEC	6.527586984	6.863203949	6.838818227	8.06945292	7.825228353	8.525666221	6.086323829	9.380098451	4.942293842	5.473584924	4.678011516	6.545146887
EEPD1	1.531543686	1.793944771	1.480660036	1.876274915	1.10767469	1.463898299	1.581784	2.017626997	1.017337881	0.697932471	0.921273999	1.304065042
EFCAB1	0	0	0	0	0	0	0	0	0	0	0	0
EFCAB10	0.022089311	0	0	0	0	0	0	0	0	0	0.023793803	0
EFCAB11	1.010734149	1.209986539	1.490043697	1.909318307	1.181028415	1.51990975	1.212622194	1.954469164	1.558236268	1.662466719	1.697894271	1.97849419
EFCAB12	0.352505976	0.454332436	0.44368314	0.548858819	0.268598365	0.391363021	0.382094821	0.372896093	0.269567047	0.292514604	0.400231311	0.604803971
EFCAB13	0.96170279	1.139420358	0.860350526	0.925704997	1.219373077	1.140481804	0.941875198	0.873814555	0.423612923	0.62770611	0.480958818	0.381269098
EFCAB14	19.42630583	18.17554442	19.38586955	21.72766229	17.2447778	16.64425464	19.36299094	18.67092742	40.35209183	39.8921546	37.11679757	49.91058436
EFCAB2	4.84107896	4.476701864	4.623775537	4.172634931	5.572578704	5.047180216	4.333141115	4.070047864	2.853313663	2.046934535	2.515539607	1.858031695
EFCAB3	0.028805975	0	0	0	0.030078586	0	0	0.028186792	0	0.031586924	0	0
EFCAB5	0.272568481	0.235475398	0.17629959	0.267831842	0.213457656	0.186611933	0.179078213	0.19262363	0.502610621	0.398509915	0.456712453	0.55943743
EFCAB6	0	0	0	0.015752402	0.016274236	0.008002963	0.016027565	0	0.050883352	0.042725832	0.050364985	0.105428774
EFCAB7	1.042262254	1.287414307	1.23223615	1.871486329	1.979794471	1.844672221	1.448087023	1.616584859	1.785827745	1.726482827	1.660143583	1.869322401
EFCAB8	0.260003018	0.25233899	0.222494674	0.240419682	0.300371495	0.28405705	0.307196367	0.324784178	0.174585119	0.285103896	0.214518488	0.155440554
EFCAB9	0.305736141	0.237716428	0.161797674	0.07725166	0.159621587	0.07849497	0.078601091	0	0.33271737	0.167626063	0.082331965	0.07954389
EFCC1	0.0132438	0	0	0	0	0	0	0	0	0.014522366	0.014265741	0
EFEMP1	0	0	0	0	0	0	0	0	0	0.017384907	0	0.016499381
EFEMP2	1.248964662	1.537570085	0.936361008	0.420774999	1.249803064	1.042145979	0.990039271	0.483755144	4.077557552	6.56238206	4.93291095	2.518325721
EFHB	0.270205829	0.133391013	0.163422615	0.221077921	0.120918505	0.13213883	0.172012717	0.113313332	0.112019621	0.169309539	0.124738243	0.200856882
EFHC1	2.600981892	2.773742711	2.328630698	2.110581734	2.384921286	2.680687526	2.291251754	2.079845402	5.874087117	4.15034234	3.815912576	6.354691396
EFHC2	0	0	0	0	0	0	0	0	0	0	0	0
EFHD1	0	0	0	0	0	0	0	0	1.017938595	0.836750048	1.087113381	0.614809502
EFHD2	43.39911254	42.29354245	47.6764705	47.97765597	36.53651684	36.36818935	47.99222084	53.78478127	35.87570772	31.88041227	40.39827713	48.66047348
EFL1	7.277151757	6.874190351	7.250774463	8.491558873	7.369202991	8.455659886	5.96816968	7.550756236	6.667454684	5.910358901	5.94514712	7.815363974
EFNA1	0	0	0	0	0	0	0	0	0	0.0753568	0	0
EFNA2	0	0	0	0.022788673	0	0	0	0	0.417134033	0.284328642	0.582895826	0.281578373
EFNA3	1.268238808	1.439994163	0.926840998	1.037651926	1.891811402	2.666890817	1.366300441	1.477354891	1.807353613	2.350903804	1.82146768	1.571237341
EFNA4	3.296210172	4.349120069	3.534062802	4.240052922	3.39713341	4.703953116	3.609772288	3.769899403	5.962972875	4.694063194	6.409448535	5.925102264
EFNA5	1.304956901	0.762164933	1.238526839	0.600633195	1.445772116	0.811636019	1.241150868	0.599486831	0.200016818	0.342619047	0.138585424	0.21040233
EFNB1	2.891597537	3.349391876	3.231484739	4.489919353	3.694058511	3.434078332	3.222762704	4.726276064	10.96978457	7.404331575	9.552979678	10.70888579
EFNB2	0.020942462	0.017368762	0.039898422	0.025399783	0.008747069	0.017205716	0.004307244	0.016393844	0.164092571	0.050521376	0.112792293	0.178715253
EFNB3	0.03343247	0.017329643	0.017692694	0	0	0	0	0.016356921	0.018191444	0.018330032	0	0
EFR3A	24.7163146	20.83412394	22.6492417	24.98070817	21.97291584	20.09018025	22.75472623	19.06303584	16.78124161	16.80262893	15.64294964	18.51474644
EFR3B	0.070210805	0.065508494	0.081743293	0.163212275	0.058650103	0.043262363	0.028880568	0.075571792	0.580692528	0.538923043	0.574776811	1.176386053
EFS	0.033246562	0.017233278	0.03518862	0	0	0	0	0	0	0	0	0
EFTUD2	47.16742476	46.3909611	50.60943272	51.32071161	50.54471273	50.7291252	50.51866675	53.58800554	47.63234679	48.24102432	51.9634743	50.8299586
EGF	0.026087376	0	0.018407492	0	0.009079961	0.017860523	0	0.017017753	0	0	0	0
EGFL6	0	0	0	0	0	0	0	0	0	0	0	0
EGFL7	3.116230563	4.897315	3.487333713	2.547737518	2.279803707	3.200255255	3.08211374	2.602542381	6.480072711	8.096505812	7.076843936	4.647640073
EGFL8	6.163943504	9.369314557	6.215435111	6.187786211	9.567411532	11.07775447	6.895481446	9.495458436	11.19496091	9.864508405	10.36312194	12.17932707
EGFLAM	0	0	0.041746587	0	0	0	0	0	0.010730852	0	0	0
EGFR	0.299055283	0.169106849	0.359686632	0.006869416	0	0	0	0	0.85799644	1.035948975	0.73943818	1.358063481
EGLN1	15.05363965	14.54520485	14.73760519	12.84743312	19.65232019	16.48706173	15.58956695	12.39372351	38.53841062	28.24297282	30.68320642	33.75394959
EGLN2	20.34069658	22.10136238	20.01173838	23.93867252	19.6475613	21.33310726	21.57252634	28.38582817	25.57745435	22.75348389	27.10598065	27.22990853
EGLN3	0	0	0	0	0	0	0	0	0.086051811	0.303475842	0.149056549	0
EGR1	129.5478905	189.8012049	124.9837503	168.8264083	82.11227619	89.69251725	63.61073986	135.1581865	56.72831153	32.53142961	32.40014876	3.94635097
EGR2	3.769555857	7.199654709	2.947382813	3.312192344	0.460983517	0.3138808	0.733378684	0.681214914	1.016315601	0.223430891	0.182902189	0.088354211
EGR3	3.537872702	7.346373507	2.578570807	4.303708224	0.176966295	0.141414559	0.370353486	0.570061232	1.636864666	0.069690206	0.27383482	0.066140432
EGR4	0.09070301	0.117539237	0.096001317	0.045836636	0.023677538	0	0.04663731	0.044376678	0.370153332	0.099459544	0.14655298	0.11799178
EHBP1	3.007547298	3.323810046	3.100645752	3.842664167	3.784794899	4.174036746	3.428358674	3.636980549	6.762066067	7.256931356	6.433397638	5.957172602
EHBP1L1	16.9270083	17.79812451	15.71076007	14.51505934	13.56580351	14.61990264	14.61983105	14.83606441	16.50001436	14.67970453	16.96566805	16.90305532
EHD1	12.41339831	10.83474838	12.25876528	10.03970319	8.223846444	7.878358616	11.75644615	11.71990985	10.92869979	8.023638649	10.69994479	15.20152432
EHD2	0.120110627	0.264601024	0.095345058	0.0455233	0.125416961	0.061674619	0.0771975	0.014691107	2.646885503	3.868869412	3.153608319	3.984296653
EHD3	0	0	0.011306885	0	0	0	0.021971478	0	0	0.02342838	0	0
EHD4	11.69530538	11.98953825	12.98854031	14.86477829	11.28007257	12.64078216	11.60623597	12.91761545	14.14382228	10.54086439	12.25727804	13.81589971
EHF	0	0	0	0	0	0	0	0	0.007274318	0	0	0
EHHADH	1.222944565	1.295685144	1.109469766	1.276774687	1.16471136	1.380131333	1.24379748	1.05200656	6.30334885	6.262951823	5.993043285	6.545325838
EHMT1	5.710402377	5.737795375	5.523257485	6.446307331	6.334201544	6.121505492	5.971680974	6.67940005	9.742181869	8.544796742	9.392161777	10.98492685
EHMT2	27.50504171	31.98930416	28.60848789	32.01600935	32.59256311	34.20362379	29.99613474	37.63748091	25.443802	27.44935109	26.72102398	27.37476544
EI24	34.68855339	28.72531424	38.23716606	37.1332696	31.42838618	28.7163232	40.23980369	36.92327993	81.48436714	86.74385223	94.96241386	75.67775716
EID1	61.91573574	58.55865477	56.84383289	53.16273774	59.49190443	59.25105122	59.75457153	54.79329126	83.79067677	86.9968848	77.67034608	103.2939994
EID2	8.652793706	10.29183006	10.18036076	13.07898886	10.48712681	12.09938253	10.76513316	12.28442333	8.659727893	9.191636205	8.238634117	11.73846264
EID2B	1.894418997	1.796791935	1.791772895	2.85165678	2.104374481	2.483616115	2.072478198	2.248102558	1.798418382	2.563485927	2.214267331	3.104058812
EID3	0.073011616	0.113536204	0.115914752	0.184481576	0.152474352	0.074980269	0.075081639	0.071442237	0.158909789	0.240180628	0.196613649	0.075982224
EIF1	307.842631	286.4510442	286.8684965	347.7615721	251.9083845	235.6696027	274.4641019	324.1768528	214.1836703	215.5484083	249.2440681	224.71353
EIF1AD	8.557833438	9.559888431	8.928332804	11.36775351	9.10003652	9.954415613	8.441262221	11.22783963	14.00760644	11.49062792	14.93722581	15.54010338
EIF1AX	24.79781613	22.04423315	26.8376004	27.56693493	30.91478562	29.45727897	28.63541863	27.86529382	23.19335165	23.58310697	25.35088731	24.68197911
EIF1AY	21.5774806	21.01837634	23.85201952	20.49127791	24.05147149	26.01649794	25.34224569	24.33885362	24.85806354	31.51943074	25.63690739	24.01092386
EIF1B	29.24432656	31.36478779	35.11712997	44.76565755	32.20192016	30.36048909	34.01035022	41.93503975	34.42901477	37.89806653	32.58913971	39.95178356
EIF2A	26.88807976	26.69742882	26.62038754	24.48626668	34.50494896	32.68976848	30.87915327	24.43627524	21.86998841	26.85901584	24.80132488	17.38016724
EIF2AK1	36.10299596	38.64361593	37.36005098	44.91424714	40.867492	42.75600865	40.02340668	43.02239272	42.46724882	50.26718815	45.61387918	43.97349428
EIF2AK2	11.85545794	7.494602711	6.821719385	7.903653801	7.777947158	8.111380697	7.310649729	6.685193056	17.94178578	12.72486022	11.64977316	13.76308666
EIF2AK3	3.316954438	3.693798474	3.703233145	5.309826562	3.932740727	4.164588148	3.14691947	4.250763352	4.750799141	4.552335901	4.448840249	4.799269318
EIF2AK4	11.09811191	10.94879719	10.40501418	10.41626307	9.961807281	10.51159851	11.2589056	8.861606681	14.77844139	18.4231846	15.03553517	16.42231364
EIF2B1	84.91551556	75.74111497	92.40360346	79.42561254	84.79098702	80.96986977	94.0106034	85.4554211	17.25433136	17.10180262	19.37222716	20.69264123
EIF2B2	8.380463729	9.954986026	9.387414958	8.434818491	9.334082057	9.000892343	9.982593556	9.430375222	21.81209575	24.81170317	22.56782751	19.25984191
EIF2B3	13.05626565	12.92211692	16.21152982	14.71196748	14.95667431	14.57990897	17.11979281	14.67749474	14.23147276	15.49820433	15.54293035	13.98323505
EIF2B4	21.5601471	20.62990421	20.7051095	19.58810074	17.88008057	18.03824998	21.39768787	20.76668051	17.13831342	16.955934	15.31485709	18.63029903
EIF2B5	26.58256749	26.22187756	28.6848007	28.16956338	31.40180363	30.13036979	29.08501838	30.25003567	34.88950047	35.43010502	36.73221739	33.30872625
EIF2D	17.14214484	18.16350513	16.10829473	15.53714283	17.66450579	18.38163895	15.88217135	16.94381442	7.145866002	9.477037887	7.745120257	8.494035596
EIF2S1	17.07952928	14.21055297	17.55759212	16.34819474	17.591257	17.61966122	18.70634247	16.44692911	30.77647652	31.74761174	33.96987242	32.49683997
EIF2S2	66.35817292	65.51391036	70.51033938	75.78088415	71.52201983	71.77076999	75.63902793	75.44198645	58.59608539	62.30145341	70.46765264	60.03241125
EIF2S3	170.6221198	171.2966969	173.2745225	133.6478241	168.8847064	172.0649603	165.5902903	135.8035268	81.50836185	101.6441877	96.70320602	79.17420069
EIF2S3B	6.57012896	6.896097205	6.867261953	5.026852784	6.368834535	6.873400514	6.545925147	4.946851767	3.519286655	4.376513122	4.475551861	2.747758825
EIF3A	151.5403958	128.7364164	142.3311479	143.409954	152.0034178	142.4398707	145.9561721	128.8376293	85.6172911	84.7518972	85.43448262	91.99938034
EIF3B	90.64060426	88.52591969	99.53924419	99.42461301	104.0719774	103.8319153	104.9681632	107.0482177	80.96167733	76.02914883	91.47686003	79.87767457
EIF3C	6.789394256	8.29648414	7.155409839	7.154022178	8.22061886	11.77900781	12.15145432	6.643456728	13.95965967	5.813316882	5.94399216	7.366298102
EIF3CL	2.46832848	3.360694793	4.023269872	11.29282247	2.714836142	8.128526168	3.333647728	2.850020891	1.66541832	1.876591722	1.630726831	1.900880052
EIF3D	190.0314045	195.7453095	180.0923505	156.4864164	204.797854	203.4911807	200.9839166	176.3832576	162.4548466	168.8088206	163.3871729	126.9672314
EIF3E	592.3340448	619.4349496	544.13813	476.260342	642.5357493	659.0734607	628.3864016	521.0457689	308.6598884	386.350109	331.6013089	258.5113885
EIF3F	198.1014406	203.9051773	199.0626671	179.7395182	160.7318212	177.57262	194.2898808	191.4537609	104.5283538	117.1161666	107.2937588	112.8449148
EIF3G	203.6939937	204.9374116	206.201382	189.0945312	196.1058743	204.8752108	219.5746555	231.0100552	91.83471343	97.55599128	98.1443741	102.7142923
EIF3H	470.3588673	432.2285006	450.5300017	329.8513794	459.0571618	418.7659221	478.8336527	355.8099062	267.852357	300.6677805	286.4899266	224.1273575
EIF3I	116.171449	118.7774472	131.4751543	116.9323635	96.91417586	105.0207881	133.9981892	125.499997	72.35103048	82.92589766	84.63194041	73.74112421
EIF3J	66.68346674	56.41654528	67.84015818	68.75748055	73.40745935	67.42294417	78.26529999	66.48512842	42.48793007	41.28922208	46.42827631	45.25008753
EIF3K	486.2142739	494.0611629	502.7533875	446.1045465	422.1245599	452.8511039	492.4396183	489.7370914	191.3471379	203.9714486	216.3341078	178.5944724
EIF3L	300.1723326	314.9491973	291.4358316	253.8812842	289.2699922	307.8401499	296.421645	282.4591195	184.7687895	216.1447332	191.6868365	176.9953635
EIF3M	152.7497212	158.9133878	156.2242032	146.942797	173.5915831	175.3397754	170.6526729	159.3058566	93.04835396	114.0051148	104.7050131	94.24508781
EIF4A1	794.0135361	733.8420527	807.0788652	891.9515344	785.806953	764.0487058	807.8580743	938.3834621	257.5377795	261.0086948	270.0762175	273.6099634
EIF4A2	167.546639	178.8572871	155.9801451	173.3365474	170.2243095	186.2666118	156.7878527	158.8497917	161.8635694	203.1610161	145.5939145	188.7260615
EIF4A3	56.63347668	60.22368848	64.53991653	75.46177373	56.96447058	62.14411431	62.96210278	75.30522621	65.17729881	59.58284861	67.18744196	75.21420726
EIF4B	313.3491139	316.6492649	304.3033714	253.8821031	366.5329031	362.0924911	330.9638684	268.8159088	134.6019608	170.0381651	134.512747	119.7184618
EIF4E	5.778655717	5.469574102	6.55879831	5.372873386	6.507746766	6.258831699	6.738483985	5.697973146	6.167594205	6.71701583	6.464148333	5.768405089
EIF4E1B	0	0	0	0	0	0	0	0	0	0	0	0
EIF4E2	23.09160291	19.86920506	23.38186909	24.07150237	20.0256852	19.46441803	22.47348361	22.66865153	25.36073215	23.71690698	24.64449761	26.84147962
EIF4E3	7.125466963	4.420659519	6.769906394	3.564795997	9.194805314	5.0140584	7.433447209	2.978159446	0.103505536	0.13905877	0.085375908	0.049490852

EIF4EBP1	93.46200792	95.00834696	117.9499394	112.759005	127.3523829	127.6330033	110.6703358	136.934391	50.51416587	66.69761058	87.92095444	55.67294483
EIF4EBP2	43.52779774	44.46581895	45.98723451	58.79750933	47.8456964	46.91745745	44.12999844	54.28893752	53.38746421	49.508517	50.23450269	58.52706583
EIF4EBP3	0.233616038	0	0.412104062	0.314820079	0.406561495	0.15994344	0.320319351	0	0.508465329	0.256169498	0.251642708	0.405201873
EIF4ENIF1	5.359936726	5.502144816	6.062356604	6.647666279	6.438070676	5.999141579	6.61968561	6.924408543	7.453215661	6.597657093	6.867858116	8.521967841
EIF4G1	119.3509325	100.6560941	122.9403323	114.5771096	107.2918714	104.05455	122.1944359	119.5753312	114.2293774	107.2884086	131.6037786	118.6402155
EIF4G2	242.0998182	225.0561056	249.643678	294.2213916	262.3210688	237.4843245	277.7421744	259.95496	277.7990819	236.5048012	274.6366202	251.0379633
EIF4G3	26.2316935	24.56465493	24.46581722	25.23360961	23.19966842	23.13719686	23.40560958	21.13679055	35.72908249	37.3680703	33.35114012	46.40631471
EIF4H	133.6094648	138.9757745	139.9085507	140.8920909	140.2123736	146.4986477	145.6309611	143.1650619	150.4867596	168.9690977	167.6686745	149.7040049
EIF5	34.74878978	33.43488506	34.67125458	54.04688765	41.61586808	43.06261572	39.99664766	49.05875714	60.03388778	36.1460609	49.2258488	73.03078083
EIF5A	103.4500668	98.16991975	118.6376799	113.0711681	99.31388184	99.12335528	115.8150428	126.0406727	29.92838519	34.9801514	40.49421722	27.61794476
EIF5A2	0.855199811	0.634711878	0.637723034	0.864347993	0.578408438	0.50899104	0.569641425	0.523010847	5.84842264	6.159387461	4.919992906	6.199625246
EIF5AL1	3.250994302	2.527718014	3.589211738	3.229119386	3.145875444	2.705459951	3.544909192	3.647312222	0.808225944	0.968040517	1.313194849	0.874982794
EIF5B	60.78487431	58.89511358	66.03273728	65.78776355	66.99839207	68.76386564	73.15596588	65.50795361	40.24789529	44.00837116	44.1930231	43.18518742
EIF6	60.02352388	59.09216886	64.4185017	58.18737764	51.45195605	53.67370383	66.66471536	62.77596323	35.34696291	39.85189329	40.30545435	42.22638564
EIPR1	13.33533148	11.92184153	12.98436191	11.49035758	12.47726332	12.57930383	13.94453935	12.84709459	10.3134278	10.3919991	10.95482252	9.180466882
ELAC1	4.217050224	4.271869597	4.233838646	5.017171846	4.428516148	4.231768211	4.857005331	5.30537791	7.00181578	4.809133959	6.67091708	9.078191392
ELAC2	33.27817438	28.56571638	34.05495657	31.2326633	29.66290125	33.23177125	33.73568786	34.02722789	10.42876986	10.40745612	11.43962387	10.34183128
ELANE	124.8432847	210.3064695	135.6806331	541.917418	125.5626739	202.9274101	121.691448	574.0636104	392.7256197	353.4316555	295.5806382	914.2392838
ELAVL1	24.00023662	24.54943355	28.16614935	32.02243255	28.33454437	29.27217094	29.10165812	34.10533026	20.76314562	21.96349689	22.69481035	21.92627733
ELAVL2	0	0	0	0	0	0	0	0	0.636113258	0.812483735	0.833598553	0.634014461
ELAVL3	0	0	0	0	0	0	0	0	0	0.011644834	0.011439057	0
ELAVL4	0.671315985	0.750004543	0.676038344	0.335954893	0.551251295	0.448455756	0.630027347	0.357141956	0.056742497	0.042881086	0.112328883	0.013565625
ELF1	69.6452374	53.3163177	65.25514588	51.44078513	59.27606647	44.72523316	62.24769254	40.11915773	58.48424199	39.85001562	46.69171156	47.44629685
ELF2	11.02040116	9.379101739	10.39391312	10.00696433	12.41098061	10.12061583	11.90246052	9.394646443	25.81938163	17.63930899	19.85092017	23.44195808
ELF3	0.016091376	0	0.051094002	0.081317537	0.067209089	0.033050513	0.016547598	0	0.052534322	0.070579395	0.051999136	0.117222575
ELF4	16.99597355	15.02583368	17.42881099	16.50938547	16.65348169	14.03583954	17.51184645	17.4179597	34.74872403	24.00300849	28.13790202	37.43965707
ELF5	0	0	0	0	0	0	0	0	0	0	0	0
ELFN1	0.028002478	0.023224031	0.011855283	0.016981214	0.09941461	0.063266553	0.092148489	0.076721586	0.146273748	0.079835228	0.102555054	0.128223957
ELFN2	0.012871561	0.05337555	0.006811719	0.019513875	0.060480946	0.052874497	0.019854743	0.012594888	0.427228045	0.543396343	0.519929168	0.261207637
ELK1	12.44737957	12.10121626	12.17907318	13.12102765	10.78293599	9.828710759	12.17449547	13.44291693	1.505095709	1.658107847	1.46989927	1.247405217
ELK3	6.962442613	6.914551228	5.730123476	9.279862215	7.104517026	6.77450897	6.971754666	7.982055076	10.68193903	9.854118263	8.405957476	11.61092056
ELK4	5.84560689	5.612785774	5.614995236	6.440650928	6.848462668	6.894146853	5.581626202	5.622178976	6.168709784	9.17414177	5.993338987	6.414324785
ELL	8.356991563	7.325638208	7.638480163	6.315767131	6.727142191	6.229641941	7.377285024	7.293319874	7.420734555	6.604527211	6.047573318	7.980654582
ELL2	1.272358072	1.456750482	1.243090458	1.462612693	0.547487319	0.653332692	1.056810404	1.087672548	4.001772537	4.53054245	3.403753417	5.289229499
ELL3	0.432921218	0.501608288	0.35039572	0.823627731	1.037049288	1.150714707	0.523759279	0.921986913	1.108533025	0.418866832	0.822930009	0.848066618
ELMO1	6.975084423	7.307963541	6.760770293	6.880955025	4.913600754	6.515425246	6.346184503	6.365304596	4.205804489	3.566571007	3.849904012	4.568974739
ELMO2	17.68304611	16.39451912	18.63413156	19.64379263	14.9517445	14.29492102	16.73971665	16.67237635	14.99371899	11.84327322	14.71602536	19.55602264
ELMO3	1.517757452	1.510514014	1.499321057	1.309007443	1.373502035	1.392422235	1.331873164	2.297006275	1.013042431	0.821046202	0.87193236	1.200427692
ELMOD1	0	0	0	0	0	0	0	0	0.036069145	0.072687865	0.035701696	0.137970801
ELMOD2	4.028875578	4.097910616	4.006755287	5.547102806	5.281072239	4.736015944	4.315079959	4.457994214	4.340304041	3.923251864	3.641029981	4.588344508
ELMOD3	3.123076429	2.943344383	3.052453983	4.394913652	2.668131787	3.038481199	3.565053859	4.00636	4.878499616	5.489160069	4.329824572	7.526650108
ELMSAN1	5.188734213	4.617200827	4.733875822	3.853817107	3.364911008	2.986877459	3.856536916	2.870525044	9.591085064	8.086754084	7.984451356	9.407239718
ELN	0	0	0	0	0.013987254	0	0	0.013107525	0	0	0	0.013940478
ELOA	16.0895268	13.03472801	15.79653311	14.89733326	13.69716616	12.33103091	15.91879602	15.06195441	16.360298	12.64123658	14.64579564	21.6495844
ELOA2	0	0	0	0	0	0	0	0	0	0	0	0
ELOA3	0	0	0	0	0	0	0	0	0	0	0	0
ELOA3B	0	0	0	0	0	0	0	0	0	0	0	0
ELOA3C	0	0	0	0	0	0	0	0	0	0	0	0
ELOA3D	0	0	0	0	0	0	0	0	0	0	0	0
ELOB	69.02842656	69.02580715	76.16715399	75.31521833	58.570481	64.86126551	75.12168245	84.61697875	71.15715479	69.4940409	74.23781842	73.47734302
ELOC	31.77678594	29.35769056	35.84295897	30.46800472	31.18208348	30.04256943	36.97212308	31.51939646	32.45083688	37.3010677	40.95273161	30.76091492
ELOF1	34.06478775	34.65112631	36.12596585	40.93360843	31.41816605	32.04206928	34.96129267	42.36631991	25.33735481	20.80116641	24.46223436	30.436471
ELOVL1	27.11568017	29.04772294	29.59248659	34.43988	30.07535246	31.46690166	29.89834099	34.16838966	163.2155676	144.4384796	160.6765928	186.683899
ELOVL2	0.036493429	0.018916284	0.038625148	0	0	0	0	0	0.238283854	0.19007852	0.206374326	0.151912916
ELOVL3	1.069064123	1.760231343	1.397750379	2.224558903	1.510280135	2.045089039	1.918515754	2.379329835	0.387803027	0.229857321	0.225795495	0.174519357
ELOVL4	0	0.018230105	0.01861202	0	0	0	0	0	0.248776909	0.347084555	0.303067732	0.567309106
ELOVL5	66.18630449	67.45757024	66.00297024	79.6511528	76.13368084	78.82166117	67.19470411	70.54586424	55.92076472	74.18844538	65.5149115	56.65070788
ELOVL6	0.763639123	1.17549683	0.938871825	1.512431581	1.52226275	2.130886375	1.142239704	2.143553848	0	0.109956546	0.016617461	0.048164191
ELOVL7	0.008971251	0.018600908	0.018990591	0.027201652	0.009367589	0	0.018451206	0.017556828	0.478385228	0.767312637	0.43485773	0.578848237
ELP1	14.72057758	14.9077476	14.60090775	19.14908581	16.79313371	17.97780617	14.96734137	17.73608295	18.70861726	18.94688574	17.80326221	19.05398197
ELP2	13.87047181	12.73847153	12.97694252	12.55459518	15.12757417	15.17205876	13.43604459	11.32339858	12.71016848	12.48339181	11.18390194	12.26633682
ELP3	18.82632155	18.74035768	16.88591311	19.2801497	21.10876247	21.22565917	17.77082415	19.36870122	20.13099351	18.67530386	20.02680274	19.83598941
ELP4	9.075372914	9.186310305	9.419985905	11.00300733	11.2454939	11.84009825	9.813330532	10.34764389	12.75862134	12.85582098	13.38384616	12.60633915
ELP5	13.73930896	14.51542959	16.97130412	19.80203427	15.8357065	15.32206499	17.16368096	22.0269563	6.904949873	6.454165262	7.488044592	7.3539078
ELP6	6.977141836	7.2104247	7.129257435	8.016407115	7.881043315	8.246820728	7.084706509	8.705713531	7.139212584	7.289837075	7.491889933	6.610267569
ELSPBP1	0	0	0	0	0	0	0	0	0	0	0	0
EMB	39.81690189	30.78953093	36.58420751	25.57579389	51.61066942	39.42747549	39.43111806	22.68198842	151.3291463	108.1195255	118.8884664	110.0126449
EMC1	16.40869393	14.60575282	15.14193707	14.15805512	13.17193513	13.6248466	14.73671612	13.04499946	14.22755258	14.75109359	14.71602536	16.92541568
EMC10	15.75701512	15.93353851	16.48947914	17.29621324	9.99663114	10.7934469	12.48486236	16.65052099	11.89437145	10.285491	12.86878509	14.83559186
EMC2	12.68929946	11.26184275	13.24712946	13.72764102	12.31739331	11.16981278	14.20045428	12.82086414	12.67771035	14.10434017	12.47677	13.69108575
EMC3	16.54075586	16.85309029	18.04841661	18.49861721	12.85965462	11.86932385	15.72376073	17.74255366	18.45409862	16.22620295	16.51092192	23.62207071
EMC4	38.96730284	40.45009772	47.82953956	48.86932685	45.96133386	45.46547681	47.72986045	55.14832777	47.84570387	52.68447454	47.30334299	49.25779917
EMC6	15.54359705	13.71952877	16.31941645	16.34078732	13.3622897	14.9369917	16.62622794	18.14143818	9.714420835	9.651527591	9.144769951	9.484732286
EMC7	38.69795743	37.2070565	41.28266121	37.40915297	33.05105802	34.39360609	41.10762471	40.21404867	50.50232986	55.47754742	54.82715232	62.1619655
EMC8	13.09761468	13.1441186	15.21695583	16.22385055	15.47383948	13.66579434	16.60294272	17.71032349	10.35465938	11.73454916	12.9304968	13.50946198
EMC9	2.347065327	2.125841185	1.856587453	3.171219657	1.599440549	1.776047796	1.753042509	3.67458453	3.253236503	3.467658364	3.592667582	3.599561909
EMCN	0	0	0	0	0	0	0	0	0	0	0	0.006036966
EMD	56.71818577	60.95330672	63.3663828	62.25946907	47.25620709	57.07402574	58.04139231	68.78085232	0	0	0	0
EME1	5.666550217	7.871811613	6.117505995	10.7899808	11.83378236	12.98876827	7.925002982	9.625682769	13.41857281	19.56042233	15.69897871	13.91716525
EME2	5.274796657	4.195846392	4.494424172	3.587677583	3.221561204	3.202517328	3.377423933	3.473405259	4.982145175	5.42025387	5.360206874	7.422788687
EMG1	32.62706582	29.76540822	35.01108478	33.58487091	42.55940344	35.87805892	38.50749556	40.8649593	14.20256235	15.13638334	20.11357627	12.27587801
EMID1	2.551052186	2.841607006	2.42718952	1.755466292	1.438144007	1.860363439	1.758222457	2.038135039	1.262572432	0.937404006	0.92083907	0.917898985
EMILIN1	2.732837017	2.946444214	3.225106712	2.499673823	3.994998379	4.111557735	3.302124035	3.663509995	0.371751251	0.404550053	0.44155691	0.327063147
EMILIN2	40.74333338	36.87002302	39.71953971	33.88515916	24.19337447	25.71886754	37.86227487	33.04126114	41.63836537	33.97263624	39.29121606	51.12370059
EMILIN3	0	0	0	0	0	0	0	0	0.290725603	0.169597104	0.333200282	0.131693256
EML1	0.030812881	0	0.032612778	0.02076166	0.010724718	0	0	0.050250934	1.91133078	1.71190397	1.725906809	2.030881996
EML2	14.9921598	15.00364272	13.96322311	11.73264589	11.60523373	12.61125397	13.47540358	12.48739963	11.70889313	14.27166567	12.38136251	16.15606478
EML3	16.14016018	21.40684086	17.67055343	16.91496437	19.96370399	21.97069062	17.2522076	19.03932008	12.99657341	17.2677623	15.54785508	17.76915235
EML4	13.01295007	11.97350312	12.76981294	13.5056037	13.83769284	12.64554362	12.10427223	12.36402781	15.05268691	14.68297668	15.44455863	15.92717099
EML5	0	0	0.009220136	0	0	0	0	0	0.8674244	0.998212258	0.980572775	1.028958235
EML6	0.31165913	0.345378025	0.312802374	0.434473086	0.274930411	0.26487937	0.248660131	0.39434488	0.163733892	0.341746926	0.231522683	0.39144427
EMP1	2.417019619	1.9021471	1.661900831	0.160480825	0.331594221	0.163063648	1.08856068	0.138106042	0.652780571	1.799149775	1.216245585	0.403926483
EMP2	0	0.031123743	0	0	0	0	0	0	4.290859826	4.027265275	4.699888933	3.717985286
EMP3	122.5026173	121.1258813	122.6429994	98.84336913	103.4390783	93.81282776	123.9309471	103.4780394	76.00114215	78.89274689	101.8386484	40.82338538
EMSY	5.608571867	5.097222068	5.61339965	6.886482504	7.137913733	6.348001254	5.476911833	5.735486248	6.702548638	7.626360245	6.19358542	6.757953924
EMX1	0	0	0	0	0	0	0	0	0.005483496	0.005525271	0.005427634	0.005243834
EMX2	0	0	0	0	0	0	0	0	0	0	0	0
EN1	0	0	0	0	0	0	0	0	0	0	0	0
EN2	0	0	0	0	0	0.016277013	0	0	1.414367328	1.842257343	1.79263001	1.649452101
ENAH	0.046142763	0.055195371	0.045081357	0.025111885	0.029650026	0.032806341	0.014600308	0.024312038	0.463521824	0.984703609	0.63849634	0.735077894
ENAM	0	0	0	0	0	0	0	0	0	0	0	0
ENC1	4.020686461	4.966812511	4.056692523	4.149147814	5.287130667	4.968424609	4.39551351	3.83314789	8.781693928	7.900899987	7.154141845	10.54867525
ENDOD1	1.90334266	2.020733153	2.330051995	2.630605854	1.747980524	2.225490447	1.957306173	2.356086714	3.456347157	4.086175509	3.297629422	6.324188447
ENDOG	13.19708938	13.11130452	14.88255161	17.62555829	13.16493603	11.33444442	15.10609692	18.67833808	8.505907413	8.527639504	8.969256333	9.074274118
ENDOU	0	0	0	0	0.022840162	0	0	0	0.095216678	0.071956554	0	0.04552756
ENDOV	1.110857637	1.191332017	0.932489034	0.967879847	0.849951572	0.96378803	0.718894333	0.955792612	3.564143783	3.486288002	3.197744818	3.458198593
ENG	17.05414694	15.2195865	16.24692008	10.54798577	11.26277643	11.74890157	15.91062079	12.17701311	20.14854364	16.96563435	20.7954204	25.44253052
ENGASE	7.155472182	6.227756775	6.900737075	8.27848156	7.407366099	7.91691081	6.47281255	7.904459425	6.637866823	7.835025514	5.874571603	11.15923951
ENHO	0	0	0	0.100900127	0.104242669	0	0	0	5.051871899	1.751521316	6.613441141	3.532398071
ENKD1	4.180570153	4.942255216	3.544993657	4.151162346	4.084456057	5.398000269	4.776775055	5.454278809	5.773349288	4.745013294	4.45048974	7.022124525
ENKUR	0	0.014923521	0	0	0	0.014783429	0	0	0	0	0	0
ENO1	546.2888339	600.31061	614.9782599	609.9781287	698.1996772	665.7144702	600.0686439	663.3202243	466.5011387	620.8440642	629.8358346	345.9381168
ENO2	1.710002553	9.554440984	2.444527138	2.379210843	4.939243957	6.682341874	1.347410196	2.325160269	1.570898353	14.68412618	4.879978352	0.716453101
ENO3	4.11497227	5.441291621	4.066468583	2.90704959	3.441797653	3.880953001	3.368039871	3.143151788	2.604620096	3.959716109	3.052996486	1.813464145
ENO4	0.005705605	0	0	0	0	0	0	0	0	0.006256428	0	0
ENOPH1	24.19097099	20.8776722	24.33035294	27.25463949	28.41395385	29.61012248	27.4276551	25.7376934	23.33243196	26.84954871	27.48617531	22.77271514
ENOSF1	15.65817939	18.06484115	14.67486541	17.0076777	17.8153264	20.61758234	13.40284313	15.71577382	13.080509	12.62445682	13.0872236	13.03620828
ENOX1	0	0.004082263	0	0	0	0	0	0	0.085705462	0.133855514	0.127248526	0.094253544
ENOX2	4.123987342	3.738463449	4.165575255	4.634396694	4.0407304	3.751716172	4.579796047	4.256457198	4.405958099	3.871678107	4.219084641	5.408817526
ENPEP	0	0	0	0	0	0	0	0	0	0	0	0
ENPP1	0	0	0	0	0	0	0	0	0.055080328	0.103071334	0.085673037	0.045148266
ENPP2	0.014630114	0.030333943	0.046454145	0	0.015276454	0.015024594	0.030089814	0	0.286582008	0.064170065	0.189108332	0.076126834
ENPP3	0	0	0	0	0	0.01440575	0.014425226	0.027451996	0.106858133	0.307634903	0.135989399	0.072991265
ENPP4	0	0	0	0.034862288	0	0	0.023647508	0.011250626	4.867342307	5.686105306	4.074658227	8.172488869
ENPP5	0	0	0	0	0	0	0	0	0.015100118	0.030430312	0	0.014440149
ENPP6	0	0	0	0	0	0	0	0	0	0	0	0
ENPP7	0	0	0	0	0	0	0	0	0	0	0.041910126	0
ENSA	48.27931408	51.47750675	51.05827149	70.37926281	50.87311345	54.39388925	52.86544386	74.52707611	65.9186556	68.54187274	69.79176429	66.22595949
ENTHD1	0	0	0	0	0	0	0	0.012751981	0	0	0	0
ENTPD1	0.503015996	0.719157431	0.368851642	0.206017013	0.243738175	0.378149408	0.27047189	0.196238078	0.665475396	0.674150303	0.6091167	0.660340165
ENTPD2	0.025611405	0	0	0.025885373	0	0	0	0.025060889	0	0.028083948	0	0
ENTPD3	0	0	0	0	0	0	0	0	0	0	0	0
ENTPD4	17.40119257	16.37482019	14.6897033	15.0972564	13.66319641	14.00956174	15.17330277	13.16992865	20.06888885	21.07227614	17.43667327	24.12976123
ENTPD5	1.237215784	1.295506466	1.223941921	1.545782955	1.395743698	1.398271569	1.138031182	1.411378321	2.692800267	2.692862839	2.189887591	3.50033556
ENTPD6	6.983342632	8.195952733	6.961654906	7.300460657	6.858392015	7.228481037	6.166271065	8.322646994	8.914749232	9.710989092	9.171721666	11.19401955
ENTPD7	2.796704814	2.208717734	2.896550109	2.723504428	2.143194862	1.805857056	2.863653569	2.337259143	3.422320622	2.540228474	3.018973412	4.078438651
ENTPD8	0	0	0	0	0	0	0	0	0.023072599	0	0.045675101	0
ENY2	31.28334756	28.43448578	31.40078287	30.54179035	30.63340301	29.20452075	36.11606146	31.36540868	37.38058648	37.36339774	36.72292177	29.46103787

EOGT	6.939264831	5.332690172	6.447887518	6.36107689	5.887239069	5.520866807	6.275122131	5.408398261	6.091814933	4.844220424	5.52999197	6.623299937
EOMES	0	0	0.015560869	0.029718671	0	0.015098486	0.060475593	0.014386046	0.239992857	0.145092724	0.205874904	0.290704666
EP300	22.47322468	19.62762893	19.63687815	21.38881702	21.87991327	19.85853929	18.17210692	17.54298477	37.12133564	26.26148022	30.15855975	33.33161548
EP400	17.46615445	16.29546723	15.39301851	19.67527087	18.96310156	18.70429908	15.09059259	17.48827878	13.23111386	12.53190252	14.02337232	15.09883438
EPAS1	9.25406184	10.09426964	9.0540319	13.79550522	7.388666926	9.493463395	9.66845241	13.32716977	9.362886157	4.214598168	4.288741443	6.56397349
EPB41	6.380434564	6.63542204	6.089536589	5.489686157	4.852321198	5.887929725	5.716599685	4.566818881	2.813160708	3.216949813	3.069814201	3.007729503
EPB41L1	0.010232873	0.031825115	0.01624592	0	0.021369896	0.031526362	0.021045989	0.005006459	0.272830141	0.252467134	0.248005768	0.25025656
EPB41L2	36.63163699	33.96799907	36.9270909	32.28848711	42.25692695	40.8659452	42.72294062	32.2477057	47.07665224	45.94217787	50.40237335	34.24742424
EPB41L3	76.94568377	52.56112239	70.0765999	40.43720883	49.99624776	33.82081139	73.93202225	36.81460205	2.321086441	1.486505904	1.431032993	2.727524465
EPB41L4A	0.019564742	0.047326246	0.013805062	0.052730742	0	0.020092277	0.01341296	0.038288398	0.23420464	0.121570036	0.147521001	0.291837674
EPB41L4B	0.01344063	0.006966919	0.007112874	0	0.01403442	0.006901518	0.006910849	0	0.065820446	0.110536482	0.101344305	0.167849828
EPB41L5	0.037306221	0.019337593	0.049356774	0.028278968	0.024346477	0.014367049	0.014386472	0	0.827194376	1.002240868	0.708258967	0.742510748
EPB42	0	0	0.016825046	0	0	0	0.016347169	0	0	0.03486226	0	0
EPC1	7.212335412	6.078958887	7.327780096	9.711202627	8.084160107	7.889051816	7.231089291	7.882033799	11.00669858	7.050421996	7.794805079	9.924172719
EPC2	6.58651374	5.747724999	6.050095777	8.1447437	6.686767569	6.433076546	6.397576318	6.728808732	12.76869343	7.446238935	9.120172221	10.51097542
EPCAM	0	0.032470385	0	0	0.032704772	0	0	0	0	0.103034414	0	0
EPDR1	8.351117791	8.514633509	8.776398583	10.29177605	6.992500562	8.090843128	9.175267587	9.058153848	14.10370906	15.31262863	13.09017978	15.27056356
EPG5	4.377286245	4.227147687	3.627638731	4.029882954	3.518510177	3.95008983	3.483749746	2.922610358	3.949999889	4.083059934	3.123917581	3.867566072
EPGN	0	0.015582157	0	0	0.031389273	0	0	0	0.016357055	0	0.03238084	0.046926452
EPHA1	1.748590317	2.467784739	1.617446547	1.425717145	1.166083205	1.70519711	1.344847062	1.351549771	0.223870999	0.370590008	0.379869167	0.412881013
EPHA10	0	0	0	0	0	0	0	0	0	0	0	0
EPHA2	0.025174064	0.052195669	0.093256016	0.012721677	0.052572443	0	0	0	0	0.110417543	0	0.013099158
EPHA3	0	0	0	0	0	0	0	0	0	0	0.008684702	0.008390605
EPHA4	0	0	0	0	0	0.016527728	0	0.007873924	0	0.017647487	0	0
EPHA5	0	0	0.005759788	0	0	0	0	0	0.011844308	0.017901813	0.017585468	0
EPHA6	0.002356038	0	0	0	0	0	0	0.002305396	0.064098972	0.085255237	0.078673007	0.093172125
EPHA7	0	0	0	0	0	0	0	0	0.228110828	0.333651275	0.182086277	0.379987502
EPHA8	0	0	0.009549427	0	0	0	0	0	0.009818621	0	0	0.009389487
EPHB1	0	0	0	0.007662041	0	0	0	0	2.450239835	0.789717605	1.380040563	1.719887285
EPHB2	0.122626749	0.107568646	0.114814092	0.100104176	0.044323007	0.062966602	0.04850133	0.059995448	0.420876245	0.506830457	0.574079456	0.230690529
EPHB3	1.542880774	1.850658066	1.363087894	0.683036478	1.94390346	1.532102761	1.271922105	0.885866804	5.356276594	5.341154266	4.573755289	3.344010073
EPHB4	12.15448651	14.36077049	12.2438783	11.90177129	13.22717598	15.00374766	12.82569952	12.64626519	2.419433095	3.964879638	3.907974117	2.898467409
EPHB6	0.089039538	0.133332153	0.104711861	0.149986676	0.061982128	0.060960241	0.081390208	0.212973786	1.636487419	1.258412838	1.53456248	1.606146023
EPHX1	14.11509744	15.47862577	14.41205118	21.63957235	13.39020127	14.65856375	13.9328128	20.37391205	30.42563756	20.17328504	23.94123423	36.09865858
EPHX2	0	0	0	0	0	0	0.015061287	0	1.769179786	2.087797677	1.325199539	1.691855681
EPHX3	0.18464065	0.054690315	0.05583606	0.079978189	0	0	0.054250165	0	0.114820112	0.173542277	0.0568252	0.054900881
EPHX4	0	0	0	0	0	0	0	0	1.663817422	1.512932676	2.088710042	1.435869201
EPM2A	1.452258664	1.540562595	1.447724866	2.07368523	1.824990949	1.777560647	1.267681549	1.941540586	1.81932134	2.083160871	1.773502623	2.548200324
EPM2AIP1	6.748799608	6.38904042	5.719011646	7.245019772	7.42460318	7.227910497	6.828563532	5.995030285	12.0595846	12.54804681	9.427834578	12.87244482
EPN1	17.08498304	19.68553675	16.87744955	18.17224412	18.45142525	19.33364093	17.06739378	22.43361839	35.02516857	33.02603475	36.91180765	39.50572452
EPN2	3.561335983	3.726633156	3.628015851	3.441956896	3.858121436	4.708853967	3.524970363	3.419445604	2.228483826	3.258359452	1.966022627	2.860750365
EPN3	0	0	0	0	0	0	0	0	0.014911703	0	0	0.014259969
EPO	0	0.041942949	0.04282164	0.040891104	0.12673714	0.083098434	0.124816168	0.079177336	0.088057529	0.044364191	0.26148137	0
EPOP	13.48568821	12.58249527	12.89895913	13.56263796	14.48131289	15.14875195	14.74120654	15.63948366	4.185320972	5.458634879	5.093169505	5.215243982
EPOR	5.819632965	9.8953399	5.646281473	10.44926017	8.424567103	10.16466444	5.916623849	10.95767619	3.862302084	5.100337152	3.870445595	5.941710282
EPPIN	0	0	0	0	0	0	0	0	0	0	0	0
EPPIN-WFDC(	0	0	0	0	0	0	0	0	0	0	0	0
EPPK1	0.009942026	0.003435618	0.003507593	0	0.01384167	0	0	0	0.010819411	0	0	0.003448845
EPRS	119.4093374	115.4910839	127.5611063	128.1627444	144.3958311	140.4783648	129.3792066	115.765318	88.47717585	102.9470441	118.5601237	76.40056361
EPS15	11.50898675	11.49364131	10.71446662	10.52825624	10.98244435	10.22643656	10.05150158	9.249960118	25.75844192	21.80837101	21.18572931	27.06820387
EPS15L1	8.357879538	7.950421525	7.908661858	8.674629766	8.83721823	8.590456655	8.732185865	9.299380679	6.509872862	6.551759399	6.276976138	8.324852629
EPS8	4.746955877	3.561575952	3.900175514	3.976427549	4.696235113	4.11478893	4.476125272	3.857645765	1.891235575	1.773307303	1.351977538	1.029884055
EPS8L1	1.888328557	2.038514888	2.031668243	1.861209366	2.151002732	1.778976479	2.102351216	2.412750196	1.80022484	1.369011006	1.79869556	1.542893094
EPS8L2	0.03155986	0	0	0.03189746	0.049431201	0.016205413	0.016227322	0.046322224	0.240415132	0.103820014	0.067990268	0.14779768
EPS8L3	0	0	0	0	0	0	0	0	1.861875564	1.487120718	1.43836721	0.998817118
EPSTI1	0.813974989	0.173289411	0.084613803	0.007345377	0.045532252	0.01492719	0.074736856	0.02133425	2.238247805	0.629571255	0.767186245	0.884909666
EPX	0.019775656	0.041002662	0.020930827	0.019987199	0	0.020308879	0.04067267	0	0.021520859	0.021684812	0	0.020580264
EPYC	0	0	0	0	0	0	0	0	0	0	0	0
EQTN	0	0	0.026161131	0	0	0	0.025418084	0	0	0	0	0.051445934
ERAL1	28.40105888	26.83842581	31.19554233	31.13023029	30.56962759	28.64498062	30.62885735	33.37079613	37.05207661	36.68425163	38.95537376	39.2814941
ERAP1	10.96304983	10.49651584	10.1952852	11.26031866	10.44102001	10.73149176	9.857233254	9.574191581	9.986767874	9.597616036	9.174119551	10.53147791
ERAP2	9.952209881	9.066814617	9.382506431	10.56668357	10.9835267	10.72736144	10.25317054	8.083941441	2.436612259	2.53534421	2.136156538	1.483666477
ERAS	0.130289494	0.067535256	0.137900197	0.131683217	0.068022759	0	0.200975186	0.063744465	0	0.428603203	0.210514662	0.271181108
ERBB2	0.197760024	0.322169411	0.179410232	0.142768206	0.176997266	0.154737021	0.213051049	0.193509187	2.039393272	1.786446756	1.754878322	1.862056489
ERBB3	0	0.018015218	0.018392631	0.017563432	0.00907263	0.017846103	0	0.008502007	0.056733335	0.171496648	0.112310745	0.072338316
ERBB4	0.23436976	0.107986685	0.188341993	0.039479474	0.090638488	0.075772527	0.040169101	0.016987556	0.066124826	0.090424513	0.03740068	0.031617381
ERBIN	27.20529137	22.45327789	24.38753198	20.97535906	25.99518419	22.26481132	24.75971379	16.74322024	33.28624382	23.77326023	26.07535666	29.35213443
ERC1	5.739826402	5.297751027	5.203271849	5.477757329	4.870326051	4.81697055	4.915204557	4.74369206	3.088925221	2.151680578	2.740973701	3.155944182
ERC2	0	0	0	0	0	0	0	0	0	0	0	0
ERCC1	12.87625874	16.64245627	12.20755042	16.41731077	16.16684601	15.99221587	14.01210696	18.09465499	13.95171779	13.87400154	14.58080279	15.99635904
ERCC2	12.84730509	12.37929454	13.06356698	10.1327302	10.46839949	11.87434283	12.49199377	11.98749253	13.48175605	15.59697808	15.27193897	14.44439727
ERCC3	23.96276447	24.39979423	23.91377107	26.50086676	26.69197768	26.7265647	26.72613658	26.70044433	31.0298792	27.73809868	27.61175311	33.35514192
ERCC4	2.036766011	1.606918062	2.219145389	2.535350888	1.923455673	1.768703795	1.917402839	1.853769951	2.754340508	3.054498644	2.532698991	4.099000945
ERCC5	1.040625534	1.310960999	0.961993124	0.945250176	1.182879971	1.109267467	1.137859021	0.850696027	1.605514025	1.386638908	1.149301835	0.932172612
ERCC6	4.259459707	3.703218657	3.401695312	3.155386682	2.218764469	2.624585961	2.90687578	2.254456587	3.539727332	3.041243726	2.51304679	3.374944879
ERCC6L	2.230767411	2.082641282	3.052439493	4.247673498	4.748723934	4.493234721	3.244572372	4.811845717	5.083275178	5.054428769	5.522690958	5.091974997
ERCC6L2	2.378020899	2.071638534	2.125256281	2.110750773	2.412517726	2.389266328	2.174094325	1.914422941	3.641943987	3.51796204	3.434998704	3.335420598
ERCC8	4.009339829	3.740817573	4.868904441	3.988245697	5.486475631	5.222655104	4.94761501	4.25353068	5.718041572	6.363216841	5.705250046	4.721475784
EREG	0	0	0	0	0	0	0	0	0	0	0	0
ERF	17.0113137	16.98238135	18.47360786	15.33455229	21.42566214	21.70197123	19.2097086	20.09475682	20.53245216	14.17225321	18.81921138	17.20725657
ERFE	0.315326623	0.461502558	0.47117089	0.168723377	0.426097749	0.47621905	0.343341269	0.326698625	4.582118012	6.834012334	6.593368565	3.667628673
ERG	0	0	0	0	0	0	0	0	0.381881428	0.281554196	0.322675303	0.347376659
ERG28	17.54051919	23.72636345	18.89796834	24.68521614	15.7979765	19.95117855	17.85816782	26.08713267	22.5867563	38.00801257	33.76081051	17.49056516
ERGIC1	15.32990023	16.18793029	15.04487337	14.96782047	14.27315497	15.95960733	16.78406635	17.83796812	11.70625682	16.40218329	14.64394033	13.0517931
ERGIC2	7.869347153	7.630890466	7.507455005	7.854332922	8.421964179	7.568419628	7.431849773	6.879540295	14.69053937	16.88634557	15.42505959	16.03549963
ERGIC3	107.3039852	117.408016	107.318052	112.2341071	84.6092568	92.64015382	104.8370531	121.2636256	127.705013	128.6347195	117.0266315	151.8037499
ERH	79.33719273	78.55741482	94.70799588	96.82053736	93.99539346	93.82549769	100.9987708	101.821955	173.1160458	188.6519385	206.3755042	152.6861362
ERI1	5.12411159	4.815371227	5.822410912	7.445511683	7.365260709	6.745161649	5.985554255	7.152095702	5.910059191	5.864006332	5.97373056	6.669068574
ERI2	6.344079436	6.139303148	6.32207009	9.579132382	10.16357351	10.12736239	7.602502266	8.034984546	12.63867302	10.92569708	11.10461924	12.43236784
ERI3	19.98900823	21.28852897	22.85816251	22.41012302	21.12547615	23.54955281	23.36304439	25.43605387	29.27912853	32.09651705	31.62735577	32.26092143
ERICH1	3.661444416	3.313548827	2.752961882	2.431685876	2.527909758	2.856085424	2.51531284	2.019778926	1.776362088	1.511995091	1.483480532	1.81671512
ERICH2	0.026664797	0.027643271	0.028222389	0.013475017	0.069607035	0.054767551	0	0.01304582	0.0870539	0.146195179	0.086167052	0.069374255
ERICH3	0	0	0.009342648	0	0	0	0	0	0.01440902	0.004839598	0.009508153	0
ERICH4	0.185443633	0.064082851	0.098138049	0.031237891	0.032272716	0.031740642	0.095350663	0.030242923	0.773601328	0.610039482	0.632551619	0.128659159
ERICH5	0	0	0	0	0.036249548	0	0	0	0	0.076134677	0	0.036128322
ERICH6	0	0	0	0	0.042182281	0	0	0	0.021981328	0.022148789	0.087029585	0.147144254
ERICH6B	0	0	0	0	0	0	0	0	0	0	0	0
ERLEC1	7.186723664	6.242957433	7.711445058	7.489023063	6.253519958	5.946819382	7.585861977	7.21815551	9.987446054	10.39868311	8.987000414	11.20149911
ERLIN1	65.97545936	66.6387435	65.09114171	94.97111531	51.5278601	69.96908809	54.47868103	85.09167272	47.66557962	42.77735597	38.01634129	89.43107725
ERLIN2	15.21136631	15.259414	13.52320172	15.05997128	12.07029093	13.52032718	12.70409518	13.06819832	41.10257896	45.62828243	37.50353776	50.76107467
ERMAP	4.524442188	4.380720292	4.111081252	5.148040814	3.639811122	3.813585322	3.730953942	4.413267657	29.09332761	15.28934079	18.43678728	28.28077652
ERMARD	13.22576889	12.17052089	12.50413177	11.00920425	14.26005743	11.88840023	11.56827453	10.03328377	20.8616823	15.87137824	14.63048803	16.76410007
ERMN	0.12931882	0.201096329	0.068436411	0.06535108	0.040509588	0.146086288	0.093089684	0.025307824	0.014073121	0.02836067	0	0.013458039
ERMP1	9.834899832	9.115646379	8.162613607	8.72957737	8.093501938	7.600063067	7.279889078	7.241445083	23.87472913	35.53502675	22.33088438	26.5199273
ERN1	1.451776718	1.505050177	1.332748392	1.841619456	1.170450885	1.080157628	1.41168915	1.401243389	1.950688016	1.743544878	1.632948795	2.158349776
ERN2	0	0	0	0	0	0	0	0	0.014285986	0	0	0.0136616
ERO1A	16.7598896	18.29711343	16.32659752	15.83922603	20.02012909	19.84556039	16.69008317	14.41797562	12.89393912	23.88815859	17.32938731	13.19706726
ERO1B	3.0458242	3.069880911	2.837995392	2.512715821	2.915352759	3.261623583	2.797544772	2.228897546	5.524363874	5.502222136	4.479623686	5.343871696
ERP27	0.982823029	0.955207563	0.552624019	1.241670516	0.481051358	1.009323444	0.631679999	0.811432115	1.9719961	1.987019456	1.554908717	2.077584717
ERP29	254.3592485	299.7265782	266.1388078	301.1394977	218.1034852	259.0147305	232.9685345	319.2617424	142.9533667	148.3481601	153.4810248	126.7921014
ERP44	19.05340595	18.89277282	22.34337433	20.64311681	16.92762984	15.09271464	22.10677484	20.24529981	24.17664168	25.24601467	23.38241354	31.02610589
ERRFI1	2.558622643	3.360391228	2.50040653	1.681688411	2.335653094	1.523370284	2.219540719	1.727962204	1.674069193	1.531910536	1.597835763	1.649909213
ERV3-1	51.83247963	24.81761446	34.63203419	26.11020954	18.51102123	15.27868772	19.2978433	11.59138257	46.99362883	24.49783577	27.41911578	47.56305737
ERV3-1-ZNF	0	0	0	0	0	0	0	0	0	0	0	0
ERVFRD-1	0	0	0	0	0	0	0	0	0	0	0	0
ERVH48-1	0	0	0	0	0	0	0	0	0	0.025721175	0.050533307	0.024411028
ERVMER34-1	0	0	0	0	0	0	0	0	0.039235013	0.039533919	0.038835312	0
ERVV-1	0	0	0	0	0	0	0	0	0	0	0	0
ERVV-2	0	0	0	0	0	0	0	0	0	0	0	0
ERVW-1	0	0	0	0	0	0	0.01546107	0	0.016361625	0	0	0
ESAM	0.143952811	0.328317463	0.396140266	1.163941543	0.300624926	0.266101727	0.177640988	0.845151337	0.626626614	0.820820617	0.74429154	1.498097882
ESCO1	7.688704955	7.322329227	6.560825578	6.43747504	6.187554869	7.405438751	6.818939521	5.175145143	8.763315578	8.069294061	8.000209784	7.788475044
ESCO2	9.79257481	10.48982431	13.36447936	22.58767735	17.32808565	16.43114504	13.02861208	19.372173	14.14219693	12.556106	14.91403331	13.99603766
ESD	45.26469497	56.07878857	47.26949231	51.26102884	54.27618778	63.92626174	52.3391403	53.12226773	37.98843682	44.90098477	39.68721472	32.46420151
ESF1	12.71343601	10.21032811	13.21449475	12.98784726	16.09933509	12.90387378	15.78489943	10.87676611	5.17895932	5.143329344	6.18340126	3.848068552
ESM1	0	0	0	0	0	0	0	0	0.028031717	0	0	0
ESPL1	7.360778714	8.149882585	8.063964092	15.40082983	15.70681985	14.64454368	8.253217363	17.14530592	14.2671375	16.46873074	17.97242534	10.32222761
ESPN	0	0	0.007032076	0	0	0	0	0	0.997782377	1.034525393	1.216630402	1.078630475
ESPNL	0.063107382	0.065423129	0.044529149	0.010630408	0.021965128	0	0.0324483	0.030875447	0	0.034599907	0.011329496	0.021891673
ESR1	0	0	0	0	0	0	0	0	0	0	0	0
ESR2	0.025684755	0.046597715	0.054370197	0.051919015	0.026819474	0.065943268	0.06603242	0.069114822	0.076866447	0.091534232	0.083000053	0.046777123
ESRP1	0.341171449	0.438576661	0.375544589	0.02758568	0.427492762	0.812859903	0.856060168	0.387252072	0.861369239	1.271968506	0.57329608	0.72430734
ESRP2	0	0	0.008228982	0.015715986	0	0	0.007995256	0.022823116	0.033843813	0.034101647	0.008374759	0.016182314
ESRRA	15.31180016	13.0366013	15.14002174	11.53881892	12.30674965	11.53486639	14.19204618	13.17555336	10.21343704	8.247725338	10.86894973	14.55447199
ESRRB	0.015446982	0.048041443	0	0.04683666	0	0.055522206	0.007942467	0.01511495	0.033620357	0.042345611	0.049916782	0.128603758
ESRRG	0	0	0	0	0	0	0	0	0.018975456	0.006373339	0.009391073	0.006048704
ESS2	3.649937765	3.378766333	3.968742949	4.134348416	3.30765919	3.534893363	3.633721624	4.539606614	3.592031529	2.99033294	3.662907416	4.862697952
ESX1	0	0	0	0	0	0	0	0	0	0	0	0

ESYT1	85.51404844	91.32579381	86.54106282	86.30164585	96.05748693	90.24884337	82.23294997	88.95722704	63.84315003	54.3138232	70.55146814	45.99582222
ESYT2	45.81881004	39.352899	42.86238731	36.88405898	48.00504162	44.17012432	41.18225729	32.96881022	52.8679074	47.98784252	49.72204971	47.46504127
ESYT3	0.990481547	1.0199821	0.866627179	0.52723381	1.344511686	1.146032335	1.154372136	0.594437281	1.868345421	1.889819819	1.579027882	1.910380891
ETAA1	3.039349627	4.082591354	3.6838574	5.070223735	6.330185937	5.672042209	4.453027479	4.397071148	4.356748552	4.784138461	4.294763344	4.013282759
ETDA	0	0	0	0	0	0	0	0	0	0	0	0
ETDB	0	0	0	0	0	0	0	0	0	0	0	0
ETDC	0	0	0	0	0	0	0	0	0	0	0	0
ETF1	54.55411154	47.18886655	58.70250324	53.68367135	54.38154736	48.88425181	61.52533558	48.49156884	64.3536568	60.27745184	70.76656408	69.70969865
ETFA	74.6582837	73.07514097	85.70237718	80.39372155	80.61043287	78.79203391	89.10799728	76.39531888	91.48917358	98.02128905	114.8967794	72.69524949
ETFB	26.61511528	29.9234649	27.9894606	28.24308946	24.16139092	28.55765307	28.59626155	31.17826978	25.62619103	24.51353361	28.6321272	29.50670287
ETFBKMT	0.416053306	0.416186475	0.370826574	0.479521969	0.426812361	0.374799638	0.487898252	0.407110271	0.667240042	0.584280972	0.652580223	0.600096718
ETFDH	11.48387646	8.294304481	9.67459957	12.3534769	12.25573167	9.452161607	11.46212971	11.2576877	11.80813814	10.83328646	12.37001443	12.08293227
ETFRF1	5.838397543	3.66070299	4.756682704	3.710844837	5.321195694	3.997214378	5.137381358	4.515351795	13.53695726	10.97806965	9.357724715	14.99150237
ETHE1	38.30671822	45.51197946	40.3436442	44.2268816	33.71207918	33.54577617	40.66037886	46.0655135	21.61754159	21.52230042	18.63966682	32.71125104
ETNK1	8.846833024	8.395620784	8.309220373	7.804374383	10.03465069	9.98627795	9.393268343	7.255114744	28.2031064	25.24411153	24.3709291	25.60878076
ETNK2	0.031275537	0.064846407	0.033102459	0	0	0	0	0	0.068071208	0.085737248	0.067377743	0.162740188
ETNPPL	0	0	0	0	0	0	0	0	0	0	0	0
ETS1	10.25999702	12.35561298	10.65283566	13.01372195	11.53736834	11.04662759	11.46280587	10.27374211	18.45144817	16.02491907	16.84977674	24.08654021
ETS2	18.46499189	16.46607944	18.3428136	10.6432929	14.10934881	13.14480431	18.00131762	10.22601135	20.88596302	14.8966308	18.29173916	14.13028033
ETV1	0.404076802	0.607411588	0.356400384	0.27907283	0.7383747	0.843776714	0.290873223	0.230644868	1.670999075	3.330534772	2.248827051	0.897103761
ETV2	0.096045624	0.24892513	0.355796046	0.145609554	0.150433196	0.073976517	0.197537413	0.469905652	0.104521655	0.131647422	0.077592642	0.049976706
ETV3	7.291086298	7.210311078	6.925728479	7.725648365	6.297552516	7.487679993	7.394146691	7.191900138	17.22194135	14.03725671	14.14207662	18.11266288
ETV3L	0	0	0	0	0	0.027727275	0.027764761	0	0.235055723	0.029605808	0.116330564	0.028097792
ETV4	7.815151722	10.84971871	8.911119711	10.34125806	14.30616423	16.11179614	9.980772765	10.56491209	2.142116611	7.60795265	6.213931287	2.474417116
ETV5	7.319779366	7.42519026	6.164562384	10.10275438	8.711556297	8.500572734	7.257513486	10.64097454	9.164895425	13.43493068	9.693254196	9.938371125
ETV6	17.63847304	13.77186697	15.8649558	8.150197627	12.0329866	10.87642631	12.82720477	7.379778787	13.31446386	6.820610927	10.95343992	8.721471601
ETV7	0.045028921	0	0	0	0	0.023121531	0.02315279	0.022030514	0.465525893	0.197504182	0.266769347	0.538901537
EVA1A	0	0	0	0	0	0	0	0	0	0	0	0
EVA1B	1.585548755	1.712219819	1.328548612	0.834640402	0.862289727	0.780227469	1.256845434	1.422178543	6.003209901	2.752813045	4.340901072	5.63770375
EVA1C	0	0	0.015693795	0	0	0	0	0	0.177498161	0.438996447	0.415267097	0.138878504
EVC	0.011823679	0.012257553	0.006257172	0	0	0	0	0	2.464053225	2.067940608	2.031397876	3.266910051
EVC2	0	0	0	0	0	0	0	0	0	0	0	0
EVI2A	32.66115206	27.35460586	26.41398096	14.68941597	30.35207129	22.1592603	24.3033528	15.06118647	14.12401815	9.64456883	10.91836743	3.572022783
EVI2B	66.99993302	63.65485051	58.6302504	50.17387051	43.61483561	34.10134712	46.42370854	42.97279376	67.65495543	49.16564019	52.17930092	57.46205296
EVI5	1.572734805	1.180012127	1.379613604	1.626668866	1.118240619	1.244350142	1.485169978	1.191622056	3.769358978	2.731124799	2.841066107	5.158547488
EVI5L	5.351076976	5.054644041	4.743669316	5.751485523	2.240664862	2.60820438	4.036310832	5.143029597	5.498150335	5.152830269	5.354362327	8.833748555
EVL	30.78766981	30.50809298	24.68643509	15.18581904	19.55775619	21.38879044	20.30296285	16.44099859	13.46955946	12.83169766	14.51913878	10.75408179
EVPL	0.314157616	0.258878409	0.45187087	0.024424477	1.160745241	0.678346946	1.457932567	0.315286996	0.131493092	0.00883299	0.156184231	0.016766137
EVPLL	0	0	0	0.025236737	0	0	0	0	0.027173205	0	0	0
EVX1	0.051149773	0.017675577	0	0	0.017803168	0	0.017533323	0	0.074218323	0.168263425	0.110193353	0.088718154
EVX2	0	0	0	0	0	0	0	0	0	0	0	0
EWSR1	58.22832838	52.00023698	60.47071901	60.13057824	62.21984498	57.87633826	62.42675816	59.90989853	66.17277425	50.49732139	62.33664536	58.8679433
EXD1	0	0	0	0	0.014559937	0	0.014339251	0	0.030348928	0.076450343	0.015019877	0.029022492
EXD2	0.030167947	0.05212495	0.074503732	0	0	0	0	0	4.158500782	4.03580658	3.596203628	3.662794729
EXD3	1.108767392	1.556340376	1.21507575	1.110710956	1.342171886	1.491347168	0.867765215	1.382571422	1.377464471	2.42085781	1.014646891	2.471140321
EXO1	12.28810618	14.12053758	15.58926496	22.76918164	22.92292488	21.78149462	16.34378134	23.62244256	18.60858065	19.90397821	19.71846279	21.83898662
EXO5	2.769146992	2.696776254	2.620049978	4.897845593	3.395303621	2.886901149	2.804511432	3.694942339	139.9234142	108.8993014	138.8143258	144.5055405
EXOC1	10.50469114	9.006762754	9.509581902	8.835431034	10.42409268	9.577986885	10.74702742	7.647567226	20.36766387	20.44393582	16.08163598	21.21836932
EXOC1L	0	0.065783162	0.067161299	0	0	0	0.065253736	0	0	0.06958063	0.273404263	0
EXOC2	4.974188347	4.641046151	4.507019237	5.450263117	4.329902754	4.239420061	4.622817049	4.780840403	2.281621938	2.436638526	2.023026201	2.709233983
EXOC3	82.36912156	75.44176805	75.57988488	62.41308061	85.926772	77.35264624	75.43520715	64.25409813	96.22706352	87.95152761	83.06306375	98.40327484
EXOC3L1	0	0	0	0.021780204	0	0	0	0.021086475	0.023451445	0	0.023212537	0.112132357
EXOC3L2	0	0	0	0	0.028622923	0	0	0	0.268479019	0.12023306	0.206689723	0.028527203
EXOC3L4	0	0	0.009578335	0	0	0	0	0	0.482568886	0.337394673	0.409416676	0.357880618
EXOC4	10.05564566	9.793448711	8.974336549	8.122796739	9.368927833	10.27412766	8.890798174	8.403397327	12.26821989	12.76928873	10.79705866	12.15221459
EXOC5	13.56312873	11.88120946	13.3517129	13.178551	13.88221077	12.02595719	13.33818134	11.2387294	14.6786031	15.07775549	14.36106211	15.43946451
EXOC6	6.218731828	5.32780449	6.289024547	7.721351097	7.919332729	7.561359043	6.983313367	7.321934992	5.773490708	4.492102574	4.899711918	6.375731966
EXOC6B	1.188681032	1.136120582	1.043316037	1.183815092	0.823427221	0.869399456	0.816909267	0.902135302	1.558608782	1.729438558	1.09927369	2.093924342
EXOC7	16.26088567	15.36295373	15.34045277	15.67401836	13.50881181	13.01093903	15.49515904	15.78027301	20.30621244	16.13089357	16.35433962	25.82521831
EXOC8	7.000893858	6.107829738	7.13294266	7.847879249	6.326751538	5.910784172	6.811972242	6.072187183	18.91584305	16.76495351	16.29961561	16.36840625
EXOG	2.697110281	2.422834256	2.640726449	3.102616732	2.156718295	2.458470923	2.676146163	2.589690922	2.880139654	2.431099354	2.660291834	3.017196216
EXOSC1	9.901629549	9.092247191	9.593136534	10.54391927	9.551609941	9.193343873	10.55576133	11.80693747	8.845290841	9.03518839	9.612646934	9.679539742
EXOSC10	35.77772861	39.07297	38.32159911	45.91401481	49.13312547	47.001672	40.68816396	49.40037281	31.66152192	35.4888482	34.9002221	28.21332764
EXOSC2	21.6366411	21.69962652	23.39094013	25.10554582	27.62009233	29.4198323	24.46680392	26.86856657	20.87138178	20.91993355	23.3713047	22.60082851
EXOSC3	22.4474431	22.27122729	25.89325255	27.29597816	30.24503935	27.79532028	27.89301238	29.62978476	31.29892719	31.15277125	32.17645907	31.08684118
EXOSC4	26.22268741	24.82786261	32.72779545	23.43924168	25.90396068	24.23158136	27.27789966	25.85679015	14.73578675	11.96708437	17.19802663	16.56305459
EXOSC5	31.560336	31.09094911	34.72171576	29.65468952	37.25060392	38.58225176	31.0634041	36.54981946	12.2536393	15.61182138	18.30983395	14.19690393
EXOSC6	13.54734826	11.2880811	12.63011679	13.30837067	12.42719781	11.79973321	11.55528506	13.72073368	12.64169432	12.91154545	14.86547223	17.52436128
EXOSC7	13.39260181	13.31381038	13.84585394	15.17950484	16.50643284	17.33950389	16.03490198	15.53846418	10.30625324	15.00022317	12.70005329	11.27444497
EXOSC8	36.49780854	44.12562888	41.91205312	63.06475219	68.60554006	72.26906076	51.48027968	69.59256581	29.52018532	36.62082765	36.30406898	26.6695199
EXOSC9	23.49232924	24.64675817	25.52129351	33.43490711	34.39527296	35.13151795	28.21861549	33.17024101	31.12058316	40.23306658	37.51714047	32.31382999
EXPH5	0	0	0	0.004030024	0	0	0	0	0.086785116	0.26671114	0.180392112	0.124488104
EXT1	1.024029704	0.663504272	0.965301378	0.51749194	0.852074556	0.394365449	0.82270544	0.720200625	1.880550628	2.403686972	2.068214225	2.347857489
EXT2	11.46231196	11.47798449	11.53311641	15.4919454	7.510325524	8.465431951	9.086325439	12.48118226	17.48685874	20.85461241	15.52416093	32.00137323
EXTL1	0	0.012217285	0.012473233	0	0	0	0	0	0.012824848	0.012922552	0	0
EXTL2	6.943661427	7.609802194	7.753072894	7.310995438	7.154813552	8.071224096	7.799653555	7.197592584	5.380849486	4.585138563	5.243841029	5.606242567
EXTL3	7.530176678	5.701763483	7.468062246	7.79574011	7.384997501	5.9729489	6.751112951	7.506726444	13.88121356	11.02169232	12.25199457	18.51332082
EYA1	0.004726771	0	0.015008639	0	0	0	0	0	0.113165992	0.093295743	0.096738613	0.10330085
EYA2	0	0	0	0	0	0	0	0	0.975248545	1.179213995	1.22273024	0.538849508
EYA3	8.375440648	8.210248134	8.097960131	8.917489451	7.734077561	8.392300772	8.495718559	8.283020036	6.528125163	6.336878806	5.988177825	7.826801169
EYA4	0.005501438	0.022813259	0.040759582	0	0	0	0	0	0	0	0	0
EYS	0.003863964	0.01201726	0	0.003905297	0	0.0079363	0.00794703	0.007561817	0.012614877	0.008473987	0	0.020105881
EZH1	10.77568301	10.65953786	9.206698279	9.568727681	9.41838851	10.00557247	9.794879364	9.117973512	9.703511567	7.663396029	6.984082441	9.649628974
EZH2	10.18638161	10.28736997	12.19197416	15.55455287	17.89574798	15.41260305	11.57508008	15.10065053	18.76192145	14.53002498	19.23827489	14.78816164
EZR	51.31485908	51.24534606	53.94514287	57.59117178	66.00266858	59.94326071	56.88122262	59.5881774	94.87624271	92.50117759	107.5592683	92.12222904
F10	0	0	0	0	0	0	0	0	0	0	0	0
F11	0.007493324	0.015536589	0.007931038	0	0.00782437	0.015390742	0.007705775	0	0.00815461	0	0	0
F11R	22.21383319	26.21334529	17.63357055	20.21306059	15.73462779	16.16096968	18.87232509	15.87755315	12.08710249	11.95952158	8.895053592	15.50827829
F12	1.211927046	2.135878533	1.103139458	1.053406419	1.037684119	1.269497021	1.420767824	1.423052117	0.184643153	0.398678205	0.208871004	0.479269855
F13A1	0	0.014440622	0	0.05631392	0.014544861	0.042915189	0.114595222	0.095410432	9.246838418	4.841932868	4.5613145	5.523059916
F13B	0	0	0	0	0	0	0.015143724	0	0	0	0	0
F2	0	0.027358569	0	0	0	0	0	0	0	0	0	0
F2R	0.409509596	1.033039291	0.505669037	0.344908477	0.356334339	0.462606579	0.224597333	0.320565774	2.644183094	4.759865815	2.823096677	2.471793099
F2RL1	0	0	0.018642482	0.017802019	0.018391751	0	0.018112985	0.017235001	0	0.019314034	0	0
F2RL2	0	0	0.016609152	0	0	0	0	0	0.017077357	0	0	0
F2RL3	0.015520496	0	0.032854215	0	0	0	0	0	0.456034883	0.153169706	0.468107212	0.129215803
F3	125.5856237	138.8891755	128.1854075	108.6794301	103.0991361	87.63620571	120.6829257	88.01158098	15.53886052	8.432718774	12.64354756	8.705219544
F5	0	0	0	0	0	0	0.005964127	0	0	0	0.00624722	0.01207133
F7	0	0	0	0	0	0	0	0	0	0	0	0
F8	1.098989402	1.042354039	1.014693768	0.915774159	0.830136297	0.822453321	1.009919414	0.663524137	1.323206678	1.820450007	1.297133183	1.61821913
F8A1	4.457783832	4.260831857	5.521274333	5.208450342	4.687735256	4.675385449	4.324075986	5.290041565	19.85200607	17.36850267	17.87890389	21.32037982
F8A2	0.096432905	0.199943089	0.102065916	0.19492892	0.151039781	0.04951654	0.148750451	0.14154013	0.367300896	0.475841729	0.467433094	0.602138697
F8A3	0.916112595	0.849758126	1.428922828	1.267037978	0.8055455	0.594198479	0.644585289	1.179501083	2.466163156	1.691881702	1.506173303	2.759802362
F9	0	0	0	0	0	0	0	0	0	0	0	0
FA2H	0	0	0	0	0	0	0	0	0	0.01326239	0	0.012586851
FAAH	2.106149876	2.529590244	1.196144333	2.024841599	1.984641097	1.978298039	1.241409494	2.010613015	2.40382344	2.253150333	2.130334693	2.967006096
FAAH2	0.023925994	0.041339945	0.016882401	0.016121289	0.058293699	0.032761499	0.032805791	0.023411707	0.052074928	0.07870748	0.017181474	0.08299822
FAAP100	10.73674792	12.42062673	10.77205113	12.53303314	10.16004465	11.93074593	9.574752353	13.67963843	8.641488701	9.044578037	9.577459997	11.14449377
FAAP20	2.079920682	2.462291656	2.44286244	2.2581373	2.248873112	2.342712707	2.690862278	2.580124792	2.460615041	2.398432174	2.442775042	3.086223602
FAAP24	3.009857351	4.645787637	4.35375519	6.185510164	5.307888994	6.25071688	6.087212377	5.759425348	4.003361266	4.217217558	3.962577627	3.584764811
FABP1	0	0	0	0	0	0	0	0	0.205467569	0	0	0
FABP12	0	0	0	0	0.025504675	0	0	0	0	0	0	0
FABP2	0	0	0	0	0	0	0	0	0	0	0	0
FABP3	1.480318043	0.978332258	0.489621572	0.07480766	0.483036439	0.15202327	0.646972389	0.072424936	0.020136952	0.020290363	0	0
FABP4	0	0	0	0	0	0	0	0	0	0	0	0
FABP5	105.1163514	83.71433858	110.7891313	132.8753914	99.69121453	110.3697257	114.3038352	139.9516275	83.87208638	108.6455374	116.7163171	75.61000431
FABP6	0.248256336	0.128683095	0	0	0	0	0	0	0	0	0.066853176	0
FABP7	0.016610452	0.008609989	0.008790366	0	0	0	0	0	0	0.009107019	0	0
FABP9	0	0	0	0	0	0	0	0	0	0	0	0
FADD	17.53965179	15.35342799	17.91876498	15.93693823	19.16354924	16.70040842	18.21611031	17.5320328	17.25461865	16.49447702	17.32907924	17.79997378
FADS1	22.81375552	46.25822752	20.13231882	28.94942518	27.17060112	39.39276109	14.13171353	18.84143291	89.14306785	94.07071125	116.1755792	40.36988269
FADS2	39.33741521	93.58177425	35.67680719	43.18905263	75.00324989	93.06194048	35.23810146	40.44652516	232.0591366	200.0358579	283.1426214	46.06970675
FADS3	1.673856422	1.735279174	1.276617696	0.671728981	0.668278483	0.556143682	1.544119506	0.698506368	3.669936513	3.940822806	4.268908641	5.251497832
FADS6	0	0	0	0	0	0	0	0	0	0	0	0
FAF1	30.49085848	33.80220361	33.30355735	37.78709766	39.69176786	38.75408783	32.81152299	36.9074354	70.12581434	66.24380374	69.17370785	67.46278824
FAF2	20.95753262	19.33161769	21.17861022	21.4331686	20.77006493	20.07480331	22.80656853	21.93292391	30.64574572	28.20310363	30.51220463	28.39398151
FAH	35.60966888	39.4584395	37.37255928	37.86309206	28.36366493	36.03821298	38.83267984	40.01622553	25.08368885	28.02334336	26.00206048	29.97004358
FAHD1	6.459496211	5.519563878	6.049549706	5.262442651	4.026482114	5.065709552	5.958242885	5.382129087	3.472170208	2.275177767	3.500051949	4.72598928
FAHD2A	2.215274621	2.419795218	1.990116266	2.561165185	2.431620666	2.62458047	1.90581008	2.749224092	3.069325252	3.922170481	2.63065469	3.65491357
FAHD2B	3.266480962	3.344538898	3.37192335	3.926383644	3.228319682	3.948161666	2.972036246	4.524758267	5.266293416	5.851795308	5.038889052	6.449036314
FAIM	1.046693492	1.405700679	1.284081276	1.394491503	1.912636381	2.125402253	1.883646298	1.769063905	5.954199436	4.069267196	6.841633456	4.406633065
FAIM2	0	0	0	0	0	0	0	0	0.21586087	0.181254478	0.261142224	0.172022012
FAM102A	3.07766861	3.705218415	3.362525833	5.543875581	3.252510714	3.81062664	3.483971594	5.258006911	3.214221675	2.490267644	3.234947483	5.527566583
FAM102B	4.292672833	5.526179846	4.426105663	5.295934021	5.797551694	6.540189104	4.124231613	5.117391348	35.03626055	43.65660731	32.72556864	30.47430712
FAM103A1	11.94627019	12.70401754	12.39049059	14.42660007	11.86642312	9.983441624	13.41138611	13.19672631	12.70252268	11.5606535	12.79434553	13.03791156
FAM104A	13.83308044	14.29083844	13.82678486	14.62907574	13.84166889	13.61346417	15.18131952	13.86511428	16.9552058	16.22312705	16.67888166	18.86647441
FAM104B	1.190232056	1.119125898	1.259758023	1.692542541	1.661903766	1.506586576	1.437461923	1.841769098	1.792292333	2.367459465	2.176545456	2.232466389
FAM106A	2.712888864	3.228191172	3.370725768	2.932650477	2.611048073	3.319019212	2.49869456	1.731244903	1.026887454	0.853636279	0.940194229	1.104756882

FAM106B	0.394866691	0.260499568	0.189969251	0.217685798	0.26237998	0.184324411	0.110744165	0.175626846	0.039064881	0.078724982	0.077333827	0.224145025
FAM106CP	0.061181991	0.031713543	0.064755863	0.030918231	0.031942467	0.28274254	0	0	0	0	0	0
FAM107A	0	0	0	0	0	0	0	0	0	0	0	0
FAM107B	34.93805906	43.23322885	35.56001562	50.13274453	21.89110035	32.10994833	29.19465641	40.86706779	47.61645932	30.40681537	45.02084801	56.71793414
FAM110A	3.509720756	3.657661258	3.304160673	3.341896732	4.417019185	4.173464087	3.666216066	3.813857836	1.909726887	1.478654077	1.810681439	1.595574529
FAM110B	0.047830721	0.068180593	0.012656174	0.096684744	0.015607444	0.027630229	0.021519232	0.073129067	0.143142406	0.170457081	0.125583691	0.248883995
FAM110C	0	0	0	0	0.012300087	0	0	0	0	0	0	0
FAM110D	0	0	0	0	0	0	0	0	0	0.038716781	0	0
FAM111A	15.75913518	16.92963767	16.86370146	26.29515998	24.30049865	21.7445377	14.82670896	24.61916289	39.20551667	35.29640856	33.44201688	43.35940586
FAM111B	0	0	0	0	0	0	0	0	4.111082355	2.352884346	4.313353403	3.664067236
FAM114A1	0.186024335	0.511473234	0.308176781	0.408726654	0.219578651	0.282407274	0.282789074	0.316566531	1.07381743	2.314766527	1.568180769	1.363568557
FAM114A2	4.092643389	3.576206874	3.715182135	4.0523182	4.004879363	3.736859352	4.519855551	3.908441748	4.264470288	4.321844374	3.968416938	4.794507155
FAM117A	3.482356332	4.121167383	3.298683551	1.542841662	0.464902589	0.587877219	1.357215997	0.575696911	7.198650986	6.294497382	6.679983578	11.66643726
FAM117B	2.973367385	3.043824413	2.782034354	2.157319177	2.949003981	3.072684427	3.364393543	2.644959157	4.148679567	3.546599324	3.534127782	2.910041512
FAM118A	2.31657475	2.485848323	2.580941751	2.867133511	2.732953045	3.313958014	2.72496446	3.237121122	4.024774466	4.367393267	4.027550405	4.364868852
FAM118B	4.181602556	4.112022964	4.938184686	5.367851473	3.594158033	3.396819792	5.115945056	5.32844477	6.745466393	5.528895647	6.227769259	9.361135263
FAM120A	21.85301443	20.26892178	21.72699641	24.01819724	22.87671262	21.4968102	22.87900962	21.25589572	21.58048616	21.79587837	21.35562986	26.10507727
FAM120AOS	8.517675645	7.715903031	7.774123598	9.761018456	7.142389656	6.690128156	8.993639573	9.035777954	18.91091413	14.76895205	16.15700032	21.60974456
FAM120B	11.47962794	11.02202262	10.96283696	11.09993432	10.77545925	9.58319253	10.77600284	9.804596461	17.91707865	16.53688104	15.72971607	20.6629436
FAM120C	2.912732172	2.474772168	2.694165461	2.534305338	2.499248045	2.171922001	2.135789017	2.137592407	1.743379505	1.402551614	1.793825378	2.985114283
FAM122A	5.264280735	5.428656138	5.351268042	6.02251867	5.322807201	5.449017856	5.013589036	5.857876125	10.85307914	8.011435443	8.363601535	10.47991783
FAM122B	16.40146588	17.02780629	16.59716778	23.88078174	21.66342006	20.23912779	16.33219241	21.83762004	20.32898018	21.14420525	21.03766905	20.69390951
FAM122C	0.917423822	0.833185416	0.754341474	0.965553226	0.965871415	1.027811826	0.912246673	0.830932503	1.584216621	2.09512503	1.641581293	1.62544359
FAM124A	0.700469503	0.831162415	0.69672474	0.904144898	0.431799425	0.684688869	0.650899873	0.842314725	0.670444286	0.471960962	0.690886054	0.948538437
FAM124B	0	0	0	0	0	0	0	0	0.045149003	0.045492964	0.044689054	0.064763568
FAM126A	15.21014809	12.46852279	14.38066108	14.0364311	19.04861449	14.15663013	17.97822994	11.20308039	16.48351174	15.74954901	15.11302809	14.10684444
FAM126B	4.997497175	4.250713698	4.435260845	4.665928298	4.192419721	3.608531109	4.37114321	3.134161267	5.716725981	5.485455575	4.638016157	6.124145993
FAM129A	18.13068103	15.4174532	18.40157006	22.95981152	15.37476526	15.21972071	15.4450174	16.63711328	12.30862789	10.07391757	13.04098622	15.95255011
FAM129B	13.24209705	14.1772531	14.69009447	8.295606011	12.762468	11.949976	13.47336631	10.72513462	7.350989162	10.70519914	9.747231075	6.35326209
FAM129C	0.253221463	0.218761262	0.196542932	0.221806178	0.176272309	0.216707681	0.199640606	0.198222789	0.146969743	0.111067058	0.109104383	0.149330397
FAM131A	2.559215076	2.499073878	2.114540208	1.251576448	1.534404749	1.848232582	2.360106884	1.664084571	1.868689394	2.389867264	2.187569669	3.12727818
FAM131B	0.152899097	0.135865529	0.092474579	0.033114574	0.079826992	0.056079215	0.056155031	0.074806271	0.558603223	2.191556831	0.870543144	0.78423463
FAM131C	0	0	0	0	0	0	0	0	0	0	0.034075076	0
FAM133A	0	0	0	0	0	0	0	0	0	0.008741389	0.008586919	0.033184533
FAM133B	5.313649855	4.774151297	5.324091471	6.408785503	6.103240141	5.250774316	6.277876189	6.135331345	7.710106262	7.22502543	6.652177093	6.820142386
FAM135A	4.48279352	4.472706032	4.286312155	4.66042801	5.074396419	5.822525217	5.112936525	4.566915691	6.807070121	7.659137117	6.746362229	7.185551699
FAM135B	1.025554465	1.029962938	1.204183285	0.399493992	0.71948551	0.674710781	0.576751303	0.350183264	0.017438433	0.03514257	0.028767969	0.033352531
FAM136A	19.95120032	19.12192903	22.97985723	22.8730062	22.34400498	24.12497664	26.16904886	23.67563516	18.75311686	18.44556959	20.58472718	22.71711054
FAM13A	0.004619639	0	0.029336941	0	0	0.004744201	0.019002461	0	0.251366145	0.238084271	0.223924851	0.17307352
FAM13B	13.6044901	10.34564192	12.58363117	12.09318447	10.30561884	7.757985232	11.67008958	10.30237888	19.24661307	8.404046409	12.8247551	20.3312585
FAM13C	0	0	0	0	0	0	0	0	0	0	0	0
FAM149A	0.017175091	0	0	0	0	0	0	0	0.018690794	0	0	0
FAM149B1	6.500546636	6.836051764	6.147692889	6.021789049	6.113842507	5.868962313	6.655994468	6.049641165	4.88579235	5.353777752	3.757989825	5.149212148
FAM151A	0	0	0	0	0	0	0	0	0	0	0	0
FAM151B	0.728738914	0.728498808	0.771307316	0.815448719	0.43481924	0.58801939	0.749400097	0.585739952	1.132928794	0.684935905	0.827023099	1.245924714
FAM153A	0.008109956	0	0.00858369	0	0	0	0	0	0	0	0	0
FAM153B	0.029994181	0	0	0	0	0	0	0	0	0	0	0
FAM153C	0	0	0	0	0	0	0	0	0	0	0	0
FAM155A	0	0	0	0	0	0	0	0	0.032251656	0.054162268	0.04256413	0.051403432
FAM155B	0.00687223	0.007124409	0.014547326	0	0	0	0	0	0.523509322	0.565176	0.673628995	0.765246766
FAM156A	0.324871569	1.98707779	0.286540457	0.317401869	0.395761311	1.134346302	0.334082318	0.296695913	0.259263766	0.154368458	0.186634586	0.293010942
FAM156B	0.334579173	0.382738399	0.28085634	0.373140093	0.481876489	0.331752324	0.177964734	0.237073648	0.238552076	0.126510237	0.186411997	0.192105999
FAM160A1	0	0	0	0	0	0	0	0	0	0.013333317	0	0.004218055
FAM160A2	7.232702108	8.717680557	6.609904443	8.425664447	5.595932171	5.787060168	6.272812553	8.015182611	14.46175579	14.41267444	11.96780116	24.42489083
FAM160B1	5.776563137	5.344314847	4.863404389	6.581419232	6.20540603	6.507575147	5.103292158	5.275569917	5.152898027	5.604839723	3.969075673	5.711013268
FAM160B2	9.933508851	9.614020974	9.660246965	9.916278471	8.331058017	8.996764031	10.56695645	10.77209092	12.06002251	11.02647595	10.47627523	15.5128648
FAM161A	0.734780641	1.020192423	0.722151823	1.432235603	1.109761202	1.145364296	1.011981856	0.898733232	1.77059836	1.812863001	1.936296855	1.597627692
FAM161B	0.479016862	0.537977435	0.394331819	0.753108171	0.430709881	0.437273658	0.492597934	0.585900538	1.781074584	1.269381941	1.218285067	2.714091042
FAM162A	46.42519392	60.24418871	61.16647157	53.41168706	55.44926309	65.15194876	56.0615245	51.08213691	27.25264947	66.18629104	41.15419293	26.4624868
FAM162B	0	0	0	0	0	0	0	0	0	0	0	0
FAM163A	0	0	0	0	0	0	0	0	0.027485687	0.124627874	0.122425565	0.026284393
FAM163B	0	0.00816871	0	0	0	0	0	0	0.017149878	0.008640266	0.025462749	0
FAM166A	0.028231669	0.1463382	0.059761575	0.085601	0.117915632	0.115971581	0.058064185	0.193373819	0.399400494	0.526271964	0.121640511	0.264422922
FAM166B	0	0.043683729	0.089197778	0.042588229	0	0	0.04333216	0	0.045856114	0.046205462	0.045388961	0.043851918
FAM167A	0.090183622	0.020776209	0.095451589	0.030382776	0.010463091	0.010290588	0.010304501	0.009805015	0.556139872	0.527413402	0.539680667	0.510976917
FAM167B	0.337716909	0.408461142	0.119148078	0.113776503	0.352636811	0.057803827	0.11576395	0.330457709	0.428773849	0.740640682	0.485035177	0.644338794
FAM168A	6.821033998	8.438255233	6.291703561	8.591442103	7.999215685	8.434690117	6.923524085	7.503312578	17.29091862	17.64073286	14.48844228	19.78551101
FAM168B	28.34374425	24.35520057	28.8153415	30.04519471	29.63160321	26.04722456	29.22638291	26.87380059	32.62943034	27.5310047	32.00266101	36.9089711
FAM169A	0	0.013739931	0.028055557	0	0	0	0	0	0.641833113	0.581323885	0.699537803	0.717227276
FAM169B	0	0	0	0	0	0	0	0	0	0	0	0
FAM170A	0.039134226	0	0	0	0.040863126	0	0	0.038293039	0.042587823	0.042912272	0	0.040726472
FAM170B	0	0	0	0	0	0	0	0	0	0	0	0
FAM171A1	0.011349834	0	0.024025641	0	0.011851255	0	0	0	1.457471912	1.692595425	1.638234189	1.098480825
FAM171A2	0.568811426	1.53317882	0.980461653	1.806816233	1.544246051	2.019485197	0.885763787	1.765169146	1.945457045	1.639505418	1.838108996	3.230380452
FAM171B	0.2269169	0.188194957	0.304217837	0.198765024	0	0.015535693	0	0.05921049	4.198019553	3.350824745	3.519743601	3.998796825
FAM172A	0.990447572	0.906950631	0.868551062	1.084703129	1.190676111	1.056725828	0.953953403	1.016638336	1.29062995	1.350540395	1.063799569	1.265411145
FAM173A	8.496246452	8.105930727	9.579014704	10.01832053	7.52157373	8.029837801	9.750132555	12.59091764	6.164027059	5.738411693	6.499138397	10.50780253
FAM173B	9.068352333	8.155937436	9.322627892	12.4632678	8.758284459	9.374542078	10.08713998	10.98894821	10.60932708	7.595057115	9.962167816	11.08311539
FAM174A	3.805485209	4.655252023	3.343055762	3.038492446	4.172287027	4.103499399	4.069913338	3.649212854	5.756434039	7.177335494	4.509043425	7.445398148
FAM174B	0.368278363	0.296949697	0.433100999	0.082715085	0.256365621	0.189104231	0.273519841	0.100100624	1.202336837	0.650618576	0.991740177	0.958156064
FAM177A1	3.206996624	2.78844003	2.846856962	3.510166059	2.916590206	2.656023139	3.434701439	2.892223473	4.503244158	4.813045642	4.393691351	4.537125833
FAM177B	0.009522131	0.009871549	0.010078355	0	0.009942806	0.009778881	0.009792102	0	0.010362459	0.020882808	0.010256893	0.009909556
FAM178B	0	0	0	0	0	0	0	0.012548362	0	0	0	0.013345781
FAM180A	0	0	0	0	0	0	0	0	0	0	0	0
FAM180B	0.191766076	0	0.081187144	0.116290453	0.080095223	0.157549419	0.354965439	0.112586447	0.375640993	0.252335172	0.330501518	0.319309473
FAM181A	0	0	0	0	0	0	0	0	0	0	0	0
FAM181B	0	0	0.030603321	0	0	0	0	0	0.47199025	0.475586037	0.685200151	0.601815141
FAM183A	0	0	0	0	0	0	0	0	0	0.015634439	0	0
FAM184A	0.103455056	0.214502751	0.136872822	0.06535108	0.189044744	0.318733719	0.239373473	0.063269561	4.770788066	5.601232364	4.527169836	3.364509663
FAM184B	0	0	0	0	0	0.014334749	0	0	0.075951047	0.137753403	0.120283691	0.087157819
FAM185A	0.496292509	0.606851034	0.525282836	0.668801884	0.704245082	0.601154332	0.52999274	0.448268989	0.540090345	0.606997821	0.555149343	0.576079484
FAM186A	0	0	0	0	0	0	0	0	0	0	0	0
FAM186B	0.068775001	0.014259745	0.029116964	0	0.014362679	0.042377652	0.070724908	0.1076747	0.029937759	0.015082918	0.029632773	0.085887882
FAM187A	0	0.071472289	0.109454416	0.034839954	0.035994105	0.070801356	0.035448538	0	0.037513297	0.11339726	0.148524545	0.215242404
FAM187B	0	0	0	0	0	0	0	0	0	0	0	0
FAM189A1	0.021807319	0	0.034621752	0.011020298	0.01138537	0.011197661	0	0	0.011865908	0	0	0
FAM189A2	0	0	0	0	0	0	0	0	0.167596614	0.101324054	0.165889249	0.30451605
FAM189B	9.24711776	11.6307261	11.0521916	8.576006597	12.43222613	12.53409069	12.42813737	10.39317936	15.05412161	30.37038036	23.84765264	13.96085818
FAM192A	19.71677532	19.01403631	19.076973	20.5604372	22.77494193	21.01040417	19.61608652	19.64085821	19.63997849	17.9081073	17.97462198	21.87029675
FAM193A	7.75964696	6.74602904	7.415726732	8.090501969	6.895320014	6.808620953	7.313176484	7.892797361	11.9708937	8.595201016	10.15084524	12.14950068
FAM193B	6.014357958	7.634262639	6.353148541	8.866756861	9.877503214	10.26178813	7.25627284	9.151994142	11.46685342	13.02120736	10.18952722	11.42155758
FAM196A	0	0	0	0	0	0	0	0	0	0	0	0
FAM196B	0.008848801	0.009173511	0	0	0	0	0	0	0.038518834	0.038812284	0.019063214	0.027626492
FAM198A	0	0	0	0	0	0	0	0	0.733470741	0.773433395	0.928602884	0.831909071
FAM198B	0	0.01066618	0	0	0	0.06339632	0.042321352	0	0.011196607	0.011281907	0	0.021414493
FAM199X	7.354398018	7.169308984	7.416897328	7.719231376	7.745825436	6.992970421	7.380933995	6.088124704	9.767412517	11.06817125	8.752964457	12.19865515
FAM19A1	0	0	0	0	0	0	0	0	0	0.0158316	0	0
FAM19A2	0.288716624	0.233836862	0.200537962	0.227972705	0.094209924	0.166782068	0.19484214	0.1500838	0.255284152	0.138507921	0.223527697	0.169010761
FAM19A3	0	0	0	0.036921364	0	0	0	0	0	0	0	0
FAM19A4	0	0	0	0	0	0	0	0	0	0	0	0
FAM19A5	0	0	0	0	0	0	0	0	0.060871369	0.163560289	0.160670003	0.310458206
FAM200A	2.498750052	2.11797191	2.395210429	2.366644311	2.116850766	2.291759672	2.26253607	2.21437549	3.967732371	3.532680123	3.18247672	3.843382368
FAM200B	1.021949293	1.548427045	1.22031769	1.648637324	0.937130681	1.110053039	1.279971013	1.487153564	2.744695516	2.277134973	2.363911703	3.136047415
FAM204A	9.625189598	9.795969049	9.237599584	9.630336428	11.53868854	12.73086758	11.26427142	9.736831579	5.658588935	5.817468563	5.411401699	5.48452263
FAM205A	0	0	0	0	0	0	0	0	0.040873609	0.027456666	0.026971477	0.02605812
FAM205C	0	0	0	0	0	0	0	0	6.241833852	8.358263407	6.666002384	9.346239778
FAM206A	13.25851569	11.77405451	12.15310396	15.72644113	12.85165269	13.30772549	13.91739927	15.02970618	12.14178645	11.24676591	9.808489136	12.31403029
FAM207A	13.69988226	14.4343484	15.14234185	13.36229068	13.50483884	15.80744275	15.8288136	16.7801914	7.645588459	7.913939824	8.668456571	9.13928616
FAM208A	16.46330722	15.68223286	14.93570692	14.68399552	18.19442176	16.43532964	16.18398225	12.47105314	24.7129004	23.88682705	23.71871064	21.95911876
FAM208B	7.819685269	5.315967269	7.097513363	7.098785467	7.891998323	7.123486191	7.51204553	5.457959063	7.169024466	6.22429835	6.838450871	7.107439957
FAM209A	0.158730268	0.575942259	0.336004609	0.882355243	0.165742769	0.244515303	0.244845876	0.310636745	1.036429329	0.174054202	0.598424669	1.734479168
FAM209B	0	0	0.093826658	0.044798327	0.046282371	0	0	0	0.192943186	0.340222916	0.047744402	0.322893156
FAM20A	0.010751161	0.016718518	0.017068766	0.021732335	0.005613067	0.022082101	0.011055978	0.015780098	0.011699951	0.023578172	0.00579038	0.027971478
FAM20B	16.54937487	16.97501196	17.62735872	19.51561569	18.76247541	18.67807098	16.54108893	16.40796236	16.98971249	20.5775594	17.92075468	17.47800213
FAM20C	8.542339571	7.47906114	9.542781926	8.394295634	4.152546217	4.091456044	6.237755717	8.11287783	8.616563172	9.327665902	10.12164756	17.31662854
FAM210A	4.850678075	4.608805433	4.34647517	4.274276335	5.60589449	5.290997871	4.804173511	3.815563159	3.505500423	3.532206551	3.96692213	3.783576921
FAM210B	5.870456376	7.501622166	4.936908861	5.825701976	3.129719502	3.988806996	4.15834485	5.050099585	5.732301367	3.675618573	3.725290776	9.782273034
FAM212A	10.67870151	9.872447654	10.32391482	10.55931968	11.48836604	11.77370595	8.74713994	12.93705971	5.181701441	4.866340152	5.925638089	6.879589806
FAM212B	2.684531297	3.196974452	2.706470978	3.030938509	1.667685214	1.805954361	2.428666983	2.402383373	2.181835912	1.676789925	1.912534901	3.191601276
FAM213A	0.035009474	0.065329485	0.029643608	0.01415359	0.007311229	0.014381382	0	0	4.869060021	6.641351179	6.659750726	2.623240544
FAM213B	0.812148514	0.704888867	0.799617849	0.859014597	0.670533925	0.504307449	0.660370554	1.016465798	1.767641315	1.677555045	1.912390363	1.80831825
FAM214A	3.669062155	3.45980355	2.989675237	2.458660714	2.875991561	2.343382046	2.997795383	1.81968835	7.690352086	5.456223669	5.262355311	5.63859639
FAM214B	5.972034359	6.111806162	4.829803309	3.528690507	2.542316728	2.453224683	4.928829694	3.026719272	8.265479654	8.311657741	6.614297999	9.673116561
FAM216A	23.13565971	26.43074559	22.41137351	21.80362112	30.46118012	32.53947276	24.10672177	23.41795958	15.87342276	27.41888922	22.21425203	14.31862504
FAM216B	0	0	0	0	0	0	0	0	0	0	0	0.016928325
FAM217A	0	0	0	0	0	0	0	0	0	0	0	0
FAM217B	7.038424048	5.55639626	6.946505369	9.290757049	6.884757133	6.490845068	7.061187557	6.748599345	8.65002632	6.365065002	7.167600265	9.355952089
FAM218A	0	0	0	0	0	0	0	0	0	0	0	0

FAM219A	2.785894945	2.917005587	3.361437778	3.435149143	2.254453479	2.460470845	2.306228845	3.652856534	8.701123364	6.995600169	7.577183626	9.799444891
FAM219B	12.78439528	14.50358754	12.28393322	12.99039678	12.44780107	12.04556978	11.92094526	13.0861365	14.80732092	15.92682374	15.1730663	17.39716236
FAM220A	10.37790537	12.04372636	9.466406287	9.629171676	10.25269496	9.883984459	11.14701214	10.96341461	12.74845524	13.29863722	11.91173223	14.64469848
FAM221A	0	0	0	0.0231033	0	0.046950262	0.023506868	0	0.29851278	0.250655796	0.270849083	0.309254751
FAM221B	0	0	0	0	0	0	0	0.012965508	0.014419664	0	0	0.027578872
FAM222A	0.956167086	0.899954266	1.09191678	1.869212014	0.985272363	0.801063463	1.060903382	1.846607259	1.588206177	0.620808241	1.233227737	2.238908511
FAM222B	2.101662317	1.902804307	1.675736047	1.968373502	1.748293825	1.366942732	1.801040486	1.700029452	3.148621295	2.3045583	2.475125478	3.594252978
FAM227A	0.035100822	0.041587268	0.042458508	0.005068043	0.015707799	0.041196875	0.046409143	0.034346333	0.16916466	0.153957924	0.09182266	0.266139581
FAM227B	0.217632198	0.133960858	0.129569017	0.144348906	0.191739581	0.153656482	0.188833359	0.066548191	0.259041835	0.201354665	0.168493325	0.212331517
FAM228A	0.088020001	0.060833284	0.031053861	0.148269271	0.030636204	0	0.030171847	0.057418679	0.031929257	0.160862525	0.031603982	0.030533751
FAM228B	2.917585555	3.402728311	2.702011189	2.680723036	2.250241473	2.723867335	2.216134265	3.081964381	6.963489595	5.235138574	4.46408685	9.039871531
FAM229A	1.539615475	2.633585144	1.711027764	2.02291042	1.366488665	1.660185451	1.899919927	2.787064876	2.513229917	2.78561424	2.487626766	2.964176333
FAM229B	0.886417326	1.420187299	1.023486897	1.791798891	1.367331303	1.634435128	1.388040527	2.306399331	1.534660425	2.231165035	1.627528182	2.159448364
FAM231AP	0	0	0	0	0	0	0	0	0	0	0	0
FAM231BP	0	0	0	0	0	0	0	0	0	0.014533097	0	0
FAM231D	0.062081985	0.032180053	0	0.015686521	0	0.015938984	0.015960533	0.015186884	0	0.068075425	0.033436229	0.032303951
FAM234A	9.356601584	9.284772623	8.695767466	10.90401779	6.500637731	7.664677947	7.924635003	10.43796211	15.71590571	16.11508736	13.50348216	23.80647819
FAM234B	3.015437076	3.63526329	3.106954955	3.163136531	3.077094894	3.131934289	3.077438757	3.062386406	1.504043537	3.056053352	1.759397925	1.497741722
FAM236A	0	0	0	0	0	0	0	0	0	0	0	0
FAM236B	0	0	0	0	0	0	0	0	0	0	0	0
FAM236C	0	0	0	0	0	0	0	0	0	0	0	0
FAM236D	0	0	0	0	0	0	0	0	0	0	0	0
FAM237A	0	0	0	0	0	0	0	0	0	0	0	0
FAM240A	0	0	0	0	0	0	0	0	0	0	0	0.030667524
FAM241A	6.316602889	5.709380027	5.578280487	5.466447614	4.245957637	4.439486583	5.399543161	4.596990807	4.983681452	5.562774838	4.805335667	7.477475854
FAM241B	6.187438448	6.524138066	4.309940207	3.394062119	1.739443889	3.149981913	2.90951497	3.208335682	3.510617868	4.523185454	3.161547472	4.017611415
FAM24A	0	0	0	0	0	0	0	0	0	0	0	0
FAM24B	4.460896237	4.48007195	5.090339262	4.226826575	6.550274452	4.795920627	4.58737143	5.592668553	7.812824453	7.643053672	7.507992702	6.963593638
FAM25A	0	0	0	0	0	0	0	0	0	0	0	0
FAM25C	0	0	0.161797674	0	0	0	0	0	0	0	0	0
FAM25E	0	0.173242614	0	0.337796078	0	0	0	0.163518411	0	0	0	0
FAM25G	0	0	0	0	0	0	0	0	0	0	0	0
FAM32A	95.86321411	78.21006523	98.69792237	78.14849271	75.94698714	69.78718657	103.2661037	87.06905199	62.23331642	53.83406801	60.91298046	66.57536345
FAM35A	15.44709591	13.89679466	15.41947543	14.24430607	20.05962934	18.96072663	17.24034798	14.12752887	11.02166665	11.99095957	11.66396155	8.575294747
FAM3A	12.39092947	11.70029284	11.76198744	11.29740217	10.83473291	11.45697913	11.13831882	12.16698102	7.307995044	7.434931228	8.050236619	9.896745809
FAM3B	0	0	0	0	0	0	0	0	0	0	0	0
FAM3C	12.60984079	10.34420554	10.72123082	14.29476458	10.6956573	9.585997915	11.99382949	9.745052885	9.436904199	13.12131055	9.932213118	10.9160415
FAM3D	0	0	0	0	0	0	0	0	0.145215765	0.073161035	0.0359341	0.104151703
FAM43A	9.889324699	13.83517168	9.561548022	13.3152877	5.073784042	7.766334717	7.548103981	12.07083031	14.44871462	10.07483276	13.17730306	22.41736523
FAM43B	0	0	0.022057623	0	0	0	0	0	0	0	0	0
FAM45A	21.56466056	22.87702597	21.51106217	24.01773499	15.33410879	20.04925569	20.4801497	20.93207483	14.39172576	14.31191917	12.8747392	18.84609759
FAM46A	6.644694525	6.769242137	5.00315773	8.74116573	5.886998859	5.927796874	5.837209441	7.402558882	111.0853893	76.41550121	81.30337334	86.97191772
FAM46B	0.067998603	0.09399178	0.119951098	0.045817328	0.165672953	0.139664175	0.093235329	0.155252948	0.345330918	0.546797066	0.70804103	0.16511891
FAM46C	0.065850861	0.078019751	0.02987034	0.02852369	0.009822867	0.057965516	0.038695922	0.009205057	0.214986608	1.041860456	0.222929629	0.078320138
FAM46D	0	0	0	0	0	0	0	0	0	0	0	0
FAM47A	0	0	0	0	0	0	0	0	0	0	0	0
FAM47B	0	0	0	0	0	0	0	0	0	0	0	0
FAM47C	0	0	0	0	0	0	0	0	0	0	0.017521676	0.03385665
FAM47E	0.02569702	0.079919945	0.081594243	0	0.080496846	0	0	0	0.083894351	0.056355658	0.027679897	0.080227649
FAM47E-STBI	0	0	0	0	0	0	0	0	0	0	0	0
FAM49A	3.574294174	3.460553455	3.054730205	1.868549408	2.134218921	1.983005576	3.633383804	1.688431372	4.850979875	3.86304646	4.060306403	5.19478237
FAM49B	38.07144991	41.60656259	43.6104921	46.43751022	41.11229168	40.21928436	47.06721597	44.17061603	39.894988	44.02048015	45.44036001	40.80264099
FAM50A	82.50533999	86.60567414	83.91267778	91.59395629	65.70364544	67.52239464	73.05715582	89.80595615	31.96449239	31.85887076	34.29686607	49.45464883
FAM50B	0	0	0	0.030953425	0	0	0	0	0	0	0	0
FAM53A	0.29189794	0.245870006	0.347567406	0.156729593	0.17144641	0.206090878	0.19698907	0.142811808	0.377218811	0.180043861	0.216165025	0.303774328
FAM53B	17.50216901	16.28818606	17.69108172	16.77981421	20.08629108	19.24488792	18.41292172	20.10718099	9.773311893	7.205934916	8.28024338	10.46808684
FAM53C	15.13108407	15.01151662	15.48313091	12.00351769	12.78273517	12.51335087	14.62060357	12.24694598	21.06456828	15.87808715	19.25086295	16.62615863
FAM57A	5.915738881	5.298755623	6.536796799	6.266013269	7.412506416	7.48470591	6.545804819	7.085222545	1.236058197	1.920107169	2.217532198	1.649923581
FAM57B	0.055819047	0.159135202	0.073849561	0.296184787	0.218568978	0.24362754	0.028700813	0.204822076	2.429803198	3.748981254	2.314860571	2.309083225
FAM69A	0.017363524	0	0.036755587	0.035098528	0.072522489	0.071326826	0.017855814	0.050970889	0.188958558	0.475995275	0.168330214	0.325259819
FAM69B	0.789818365	1.492330907	0.795505231	0.592262726	1.409990685	2.067034198	1.02181418	0.623259095	4.547137386	8.073988725	7.011939055	3.340843397
FAM69C	0	0	0	0	0	0	0	0	0	0	0	0
FAM71A	0	0	0	0	0	0	0.023728631	0.022578443	0	0	0	0
FAM71B	0	0	0	0	0	0	0	0	0	0	0	0
FAM71C	0	0	0	0	0	0	0	0	0	0	0	0
FAM71D	0.019094947	0	0	0	0	0	0	0.056053508	0.020780077	0	0	0.019871859
FAM71E1	0.45537005	0.275380008	0.321313294	0.191767167	0.079247953	0.311765634	0.390233906	0.297054603	0.206481866	0.457720817	0.32700538	0.315931728
FAM71E2	0	0	0	0	0	0	0	0	0	0	0	0
FAM71F1	0.021811739	0.045224258	0	0	0.022775354	0.067199585	0.08972058	0.021342898	0.047473253	0.02391746	0.023494813	0.022699189
FAM71F2	0.656858678	0.508764966	0.471476652	0.297603701	0.197091338	0.100797806	0.395972157	0.192083084	0.336872252	0.173858827	0.268378872	0.330006139
FAM72A	2.02869135	1.888737649	2.303544046	3.175122398	5.03871364	4.004967349	2.866005218	2.833082161	3.086525995	4.61106659	3.415751936	1.516807655
FAM72B	3.353304026	3.032334319	3.394390188	4.994017617	6.501132204	5.846816136	3.824368038	5.192717451	7.491728092	12.30260106	8.990759313	5.066101118
FAM72C	1.912208986	1.98237817	2.529885452	2.783036346	5.211355523	4.791596265	2.811986143	3.012480974	4.328399953	8.492100786	5.252393189	2.98501593
FAM72D	2.726383541	2.473125504	3.323641162	3.86268784	5.452191046	5.087312064	3.704865345	4.239864846	4.26504383	7.020199255	4.929563577	2.811978182
FAM76A	1.523117372	1.860974541	1.301185663	2.089199764	1.613126851	1.687086382	1.678179374	1.958782615	1.468100258	1.765597938	1.453144208	2.026648381
FAM76B	6.107379947	6.623216463	5.405164214	6.246446829	7.519867878	7.171122843	6.044748147	5.710950971	9.538542357	8.891226659	7.858452946	8.037029641
FAM78A	21.82649646	21.69934938	21.20642283	21.43523183	26.39633321	24.43981065	19.89172702	23.81843698	34.60840544	29.28598972	30.51341024	43.34396844
FAM78B	0.246717365	0.344507536	0.271787391	0.167933991	0.331221888	0.330931912	0.207112072	0.206926452	0.07123209	0.011042271	0.021694284	0.031439449
FAM81A	0.319344575	0.292863456	0.480998153	0.186208064	0.294977487	0.214432275	0.277875757	0.144221671	0.681687083	0.875435821	1.177491811	0.792497541
FAM81B	0	0	0	0	0	0.025360467	0	0	0.026873913	0.027078648	0	0
FAM83A	0	0	0.023126468	0.007361284	0	0	0	0	0.023778394	0.01597303	0	0.01515942
FAM83B	0	0	0	0	0.007396893	0	0	0	0.292945467	0.139820792	0.213655569	0.162187437
FAM83C	0	0	0.017988876	0	0.035493872	0	0	0	0.018495975	0	0	0
FAM83D	3.641248479	4.137099934	4.651987263	8.624305615	7.412202417	6.553444676	4.444212048	9.159378167	13.06853789	10.54657819	12.97502252	9.990234174
FAM83E	0	0.015333733	0	0.029898388	0.046333259	0.045569372	0	0.028946085	0.016096277	0.016218904	0.063729196	0.01539277
FAM83F	0	0	0	0	0	0	0	0	0	0.028490765	0	0
FAM83G	3.401579818	3.51593789	3.653695593	2.285176846	4.131537247	3.75244532	4.037775334	3.130928208	0.867773834	1.283906851	1.1416205	1.039934531
FAM83H	4.521254277	3.753661175	4.233587055	2.399768317	1.895327098	2.010090663	2.924176566	2.045089078	3.636037628	2.671150997	3.200809247	3.718792472
FAM84A	0	0	0	0	0	0	0	0	0	0	0	0
FAM84B	0	0	0.010304466	0.009839907	0.040663505	0.009998274	0.010011791	0	2.924204623	3.87526468	4.257726015	3.758927351
FAM86B1	0.077059518	0.068474781	0.081560857	0.011126262	0.068969065	0.135663974	0.033961846	0.07540313	0.083860024	0.072427628	0.035573877	0.137476838
FAM86B2	0.104552256	0	0.147546066	0	0.109171241	0.178952262	0.071677679	0.068203275	0.037926332	0.114645805	0.037539964	0
FAM86C1	4.295927532	2.952079433	3.975262564	3.324640781	4.536981459	4.134450367	4.190528224	3.651115468	3.018742266	3.041740101	4.072126991	3.729853663
FAM89A	13.61403442	12.81023889	13.68691674	13.94119142	14.02794572	13.57533295	13.076535	13.77833638	8.521829132	9.059416622	10.64049967	9.083933523
FAM89B	12.36365082	10.36865362	12.49992457	14.47287066	10.25081511	8.906864035	12.10694002	16.612035	11.32608264	8.012939726	11.01192861	15.97773794
FAM8A1	11.55573077	11.52127276	9.842208782	8.928566141	11.2136772	9.701151104	11.7317553	8.710758427	7.096100698	7.709739114	5.59461755	7.635677039
FAM90A1	0.284884003	0.277965103	0.319261932	0.084685719	0.139985795	0.258146023	0.310194027	0.245965097	0.419445628	0.735028021	0.541529464	0.662708862
FAM90A26	0	0	0	0	0	0	0	0	0	0	0	0.08028516
FAM91A1	19.41230993	15.92605185	17.82818847	20.03859958	19.77975224	16.78798741	19.56513719	16.39211932	27.31583425	27.84691564	24.9852024	28.17187162
FAM92A	0.039098682	0.060800133	0.010345646	0	0.163304047	0.130496996	0.050259008	0.047822823	4.765503935	5.712866763	5.317104514	6.998590808
FAM92B	0	0	0.029912175	0	0	0	0	0	0	0	0	0
FAM96A	19.90237087	22.04122899	21.63383951	21.26212613	21.96648148	23.47496173	24.3665126	23.56525344	19.74147678	21.51269364	21.69543236	17.77545512
FAM96B	52.42390693	50.83617385	55.81209613	55.08540682	47.58595821	45.29934586	50.74369472	60.11021002	41.13319631	46.17366634	50.16713979	49.75009468
FAM98A	22.13212297	18.20470093	24.31578043	22.33020609	19.89408156	21.05908695	26.59550195	20.77666208	20.44230659	22.35999398	21.51156012	21.61919806
FAM98B	12.10347233	12.05181116	13.35560892	15.31906755	16.08263881	15.77970706	15.59927135	15.50309558	13.22498467	14.50905194	13.40727648	11.2048845
FAM98C	5.867187058	7.191447014	5.352189086	6.552429952	5.991002023	6.72446544	5.966864488	6.946380303	5.644169355	5.5805342	4.713753008	5.093875738
FAM9A	0	0	0	0	0	0	0	0	0	0	0	0
FAM9B	0	0	0	0	0	0	0	0	0	0	0	0
FAM9C	0	0.044830529	0.015256571	0	0	0.01480323	0.014823243	0.014104722	0	0.063224618	0.031053685	0
FAN1	9.295418987	8.021305901	9.083749676	8.389532797	10.23917776	10.13364535	8.780486315	7.950049541	9.732568162	9.748800604	9.718754499	9.462925323
FANCA	7.938876738	9.762976361	9.127245948	12.91905399	14.70834712	15.92440664	8.238858239	13.07771027	15.97908043	15.44592041	16.6792883	14.81833341
FANCB	1.5922819	1.40275033	1.69252614	2.203310742	2.219214424	2.287898081	1.517957361	1.511247371	2.179015662	2.345487576	2.200984175	2.311359172
FANCC	1.728775253	1.732868461	1.920641667	3.234181835	2.414836877	2.433811686	1.636508156	2.671856044	2.547907142	2.291127302	2.898085183	2.639097041
FANCD2	9.88856786	10.97719747	10.84594355	18.67085557	18.94214239	19.74422304	12.19371483	17.69939874	19.37022197	22.46369171	22.27411055	19.4070017
FANCD2OS	0	0	0	0.06312846	0	0	0.032115594	0	0	0.034245139	0	0
FANCE	10.91187648	14.21707539	11.7581283	15.67537313	15.37049937	15.2792027	12.31699556	17.70544014	12.66936988	11.72730873	12.45528359	14.57983798
FANCF	5.675290644	5.175498749	4.956905716	4.766303685	5.382657953	6.329318162	5.468298548	5.171411818	4.937368909	4.315218674	4.326546027	4.873884214
FANCG	10.02154497	13.7605074	11.36458008	18.6038205	17.25539084	17.58000905	10.91560324	20.15260313	17.27145856	19.98209713	21.89811229	23.64681741
FANCI	22.91368949	26.46632438	27.57926255	46.15874522	49.24697289	45.93316648	27.90425637	42.83199823	40.39090295	41.73269957	41.74500334	39.4550973
FANCL	9.858985301	9.208807391	10.95146452	11.0003561	15.00862784	13.2073749	10.74079449	13.18114124	15.45619302	15.60070306	16.79706514	15.03462499
FANCM	2.794760138	2.33514928	2.825564344	3.913764717	3.985342694	3.833962048	2.638071957	3.006795132	3.692045148	3.354253839	3.347400715	3.87072492
FANK1	0.598208242	0.514278779	0.633151853	0.457142144	0.411345869	0.389580239	0.330090481	0.399751083	0.031756105	0.079995085	0.047148891	0.015184083
FAP	0.211722369	0.13967647	0.14939325	0.032422302	0.060293453	0.046121761	0.098965961	0.031389608	0.013964053	0	0.013821797	0
FAR1	28.20826714	24.58766454	27.75424146	31.25809179	26.94499389	26.61579619	28.05505578	25.52929644	44.99366459	42.77928806	42.88990562	47.23449889
FAR2	6.753611664	5.940326818	6.896625861	7.296923097	8.416568681	7.583296624	7.339804027	7.377490806	30.87038552	28.78066629	29.6994667	27.08642389
FARP1	0.216334337	0.366312244	0.190809372	0.32797274	0.112945856	0.177733986	0.111233921	0.261077238	5.885646314	5.036958786	5.825687181	5.943597947
FARP2	1.775985161	1.561608383	1.623848093	1.926552542	1.310734027	1.527850937	1.874147728	1.710515038	4.887443954	4.751345852	4.106497062	5.525379512
FARS2	2.373832878	1.918700163	2.274023121	2.13083807	2.445096217	2.388256696	2.581811332	2.409420677	1.190956028	0.997083042	1.152819887	1.08865812
FARSA	94.23036628	95.89380239	106.4699109	88.11220551	87.57592313	103.3584925	105.2519915	108.4192039	44.68044874	43.0670528	51.82006619	44.71435253
FARSB	18.90606192	17.50267911	19.80211518	21.15285769	21.4768072	24.27821813	20.2966832	21.33508211	17.42111123	21.9542795	22.10813204	18.73078388
FAS	3.253995491	2.91658715	3.061398848	2.59221696	2.44213487	2.448284883	4.101483309	2.780516845	0.258209637	0.09911496	0.091278273	0.329232371
FASLG	0	0	0	0	0	0	0	0	0	0	0	0
FASN	69.27563087	109.6130855	58.55501639	55.83866976	91.66661157	134.1317918	59.74589513	71.5946511	98.10962	130.2301447	139.8595323	43.25785293
FASTK	20.29035422	21.01114767	21.98518102	24.44667783	19.79824562	21.8263507	21.2192804	26.02360326	18.98714684	16.81888642	17.85526704	22.69064883
FASTKD1	13.5294612	11.83959875	12.28044347	12.20833733	12.70055761	11.94431787	12.65936067	10.37975696	9.279348808	10.07991395	9.109345875	7.641516404
FASTKD2	6.190617585	4.988040255	6.166809437	6.178531033	6.593555203	6.031307069	6.429874799	5.921085244	5.278253264	5.598831202	5.750268369	5.241077645
FASTKD3	11.2388455	10.46095347	12.38611986	10.89042358	15.00927612	11.79131656	12.60197709	11.32135184	11.77412637	9.200517953	12.1774281	10.84590901
FASTKD5	12.53614289	11.30100963	14.08402695	13.80999566	13.18809943	12.41092381	15.15290661	14.8046131	6.156491574	4.513432757	5.688871369	6.239486109

FAT1	1.636293374	1.446118722	1.198412746	0.480713232	0.307619016	0.382740643	1.011071339	0.343841682	25.52073908	21.07577092	22.03385872	31.47167666
FAT2	0	0	0.003606204	0	0	0	0	0.003333941	0	0.003736109	0	0.003545805
FAT3	0	0	0	0	0	0	0	0	0	0.004273047	0	0
FAT4	0.006259473	0.003244583	0.003312556	0	0	0	0	0	0.504078523	0.590283975	0.377578713	0.46901887
FATE1	0	0	0	0	0	0	0	0	0	0	0	0
FAU	458.6446222	512.5348086	504.4530699	451.4441908	449.1035244	480.5257267	480.116787	512.7937367	358.0710119	436.6323702	453.8149389	311.9382139
FAXC	0.004381173	0.004541941	0.004637093	0	0	0	0	0	0.061981545	0.100886815	0.099104037	0.086629138
FAXDC2	0.031615488	0.065551259	0.055770448	0.031953683	0.011004073	0.021645303	0.03251185	0.020623944	0.103216669	0	0.011351685	0.021934547
FBF1	3.05906031	3.373991545	3.980243532	4.695791433	4.082819306	4.393436757	3.772583576	4.838802988	5.031079149	5.54860541	4.893112406	7.300561721
FBH1	14.34989041	13.41922116	14.49240103	12.82717282	13.39993375	13.12708506	14.71514049	12.92327089	13.5693161	10.83391726	11.88427339	15.11265152
FBL	306.922345	339.6846003	328.0773522	333.7802193	368.6625654	427.0471317	357.4224363	380.5473446	72.4815508	97.95232617	95.02734248	57.39286284
FBLIM1	0	0.022295812	0	0	0.022456754	0	0	0	0	0.058957209	0	0.011190827
FBLL1	0	0	0	0	0	0	0	0	0.186253998	0.187672946	0.294970502	0.605586056
FBLN1	0.199417767	0.149308952	0.105533258	0.067183655	0.069409263	0.068264927	0.113928696	0.086725021	2.628309664	2.769816214	2.995344373	1.176011464
FBLN2	0	0	0	0	0	0	0	0	0	0.012648312	0.024849605	0
FBLN5	0	0	0	0	0	0	0	0	0.054254099	0	0.017900464	0
FBLN7	0	0	0.007608922	0	0	0	0	0.007034459	0.007823414	0.015766032	0.007743715	0.007481483
FBN1	0.04601074	0.062008857	0.053568242	0.162760231	0.148934652	0.212631093	0.052046759	0.135065229	2.648765967	2.472179857	2.264942161	3.313487644
FBN2	0.772722153	0.931122509	0.913453784	2.287179321	0.812099383	1.185183278	0.804950223	1.718437608	36.23032783	32.68789519	32.36429332	37.79560143
FBN3	0	0	0	0	0	0	0	0	0.011404861	0.057458734	0.005644338	0.010906398
FBP1	0.415976727	0.184817631	0.125793001	0.300304631	0.031025289	0.152568908	0.091665104	0.116295811	0.355682401	0.78194643	0.35205894	1.020410636
FBP2	0	0	0	0	0	0	0	0	0	0	0	0
FBRS	18.84784464	17.99349268	18.76504265	16.31764393	14.47923852	14.67656521	17.2416545	17.05444156	20.08291264	20.28133422	18.87436539	20.32600524
FBRSL1	6.875282359	6.736105752	7.1111774	5.659594781	8.203224516	8.134188167	7.084416872	7.412414813	5.302065552	4.746607778	5.570474387	6.388235529
FBXL12	16.74997288	16.23263606	16.17976742	13.64043596	14.77639834	13.56841617	14.30539851	13.48376533	16.32691664	14.72714701	15.97240129	15.27242696
FBXL13	1.215107569	1.046909821	1.086221798	1.053847484	0.943019202	0.657661851	1.038484231	0.610561879	0.035738942	0.027008411	0.035374857	0.068353859
FBXL14	1.204877055	1.358546823	1.183229528	1.506514366	1.549935927	1.57540781	1.194330147	1.610460065	1.074649454	1.116887972	0.996802152	1.409021231
FBXL15	6.244179671	7.934373075	7.147584412	6.528594266	6.417844587	6.251728852	6.139405676	8.235998246	3.514773523	2.597136885	3.436797998	3.870422255
FBXL16	0.01247035	0	0.013198791	0	0	0	0	0	0.013570859	0.013674247	0	0.012977729
FBXL17	1.404815721	1.314856689	1.324343292	1.370982212	1.422337837	1.197378079	1.204845638	1.313401446	1.71756858	1.484308328	1.433573476	2.249189468
FBXL18	2.484031158	1.924186373	2.282110828	2.982394354	1.990299693	1.95177908	2.33158613	2.756897104	3.374523127	2.467909905	2.705637721	4.684406492
FBXL19	12.70656981	14.28966978	13.59499143	11.23718191	12.66222002	13.84348243	15.53816059	13.94707958	12.23470771	14.17558891	14.10361783	12.40427354
FBXL2	0	0	0	0	0	0	0	0	0.512341084	0.516244277	0.449494222	0.233839108
FBXL20	3.614370577	3.880456018	2.976553163	2.99748951	3.076108317	3.488100348	3.340069022	2.872946862	6.713616884	5.136008293	5.663905897	5.884315649
FBXL22	0.069188214	0.117371618	0.113173265	0.139856658	0.131354293	0.200242457	0.10349067	0.135402037	0.068449161	0.055176504	0.027100738	0.065457509
FBXL3	15.94714701	15.31777112	15.14267085	18.24912164	16.32065309	15.81094575	15.77562547	16.68331771	27.01419595	19.75376979	20.62213278	24.84377377
FBXL4	4.026300716	3.797036524	3.739115741	3.550853996	4.516100397	4.921822161	3.819902972	3.405982329	5.738511312	4.6357528	4.343979963	5.399845543
FBXL5	9.185899929	9.581331363	8.566746811	9.341050925	9.027502374	8.763060166	8.566532266	8.503778382	24.93013664	25.66319964	20.50486211	27.06222935
FBXL6	24.58669207	25.9850439	27.19424077	27.38908435	22.86199922	24.32811739	27.0677864	34.03855233	15.91716937	12.85698429	16.36717368	23.15907767
FBXL7	0	0	0	0	0	0	0	0	0	0.00610369	0	0
FBXL8	3.735372893	4.221312524	4.167276681	4.217486495	3.970674325	3.455938622	2.699276482	4.675869807	2.929743943	2.140246224	1.449948809	3.887353203
FBXO10	3.607920747	2.943676223	3.590544554	2.718311283	4.413139356	3.406862127	4.008956781	2.943502263	7.067375852	5.148239559	6.157547759	8.777256871
FBXO11	10.938575	10.87583394	11.84648588	12.0339568	13.78452829	11.34535612	11.99530062	10.71529124	29.73342578	21.70702866	22.99177991	22.0368952
FBXO15	0.221960475	0.342810075	0.215748393	0.206021768	0.264875892	0.223293376	0.209620553	0.146270447	0.034506928	0.099342326	0.04879342	0.032998762
FBXO16	2.716444052	3.561569759	2.579153422	2.139876715	2.002202178	2.256366163	2.259416654	2.345342019	2.608385615	2.628257209	2.323631773	2.494383022
FBXO17	0	0.02320471	0.023690841	0	0	0	0	0	0.097434704	0.098176996	0	0.046588102
FBXO2	0.031375837	0	0.033208619	0.063422937	0.032761982	0.096665525	0.16132702	0	0.034144756	0.103214649	0.135187647	0.032652419
FBXO21	1.56601129	1.75774913	1.831960361	1.356654096	1.438480403	1.499408504	1.368243757	1.094535709	6.317116134	7.101182908	6.607887879	6.567923365
FBXO22	12.95802025	11.12881131	13.22094923	12.3890432	11.83757479	11.08740997	14.6137387	10.44405266	7.819351833	8.687016995	7.832305082	8.155055343
FBXO24	0.092953176	0.149899751	0.229560155	0.135701956	0.194119458	0.127279367	0.138072396	0.161698052	0.449583548	0.70216338	0.545129267	0.666397645
FBXO25	9.963983419	11.04345051	9.088437814	11.25775037	10.57335315	11.45973379	8.809475353	11.01807608	0.088156955	0.066621424	0.087258869	0.063227963
FBXO27	0	0	0	0	0	0	0	0	0.493295251	0.642027239	0.467925302	0.766569693
FBXO28	12.23079176	11.04220644	12.14073232	11.98249633	12.80205538	11.38460741	12.45574225	10.78945532	17.89727688	14.12597845	16.03490169	18.14236935
FBXO3	3.551451311	3.48190539	3.501151557	4.430396537	4.460615166	3.834781964	3.57909911	4.070846806	9.684252582	9.157194058	8.503527333	9.303230224
FBXO30	4.300018462	3.327247834	4.072754811	3.866298345	4.35428514	3.5049162	4.061669888	3.123900504	9.069981011	7.267891537	7.230757529	7.838552153
FBXO31	4.158852583	3.434555389	4.038079958	3.847125075	3.937768104	4.053821097	4.421739303	4.24189303	1.84103248	1.85505811	2.173445249	1.934790839
FBXO32	0.534532062	0.489897988	0.336173918	0.469782625	0.339741656	0.310273234	0.446122862	0.43207845	1.778835623	1.010872523	0.809427765	1.967136671
FBXO33	3.814427989	2.968508321	4.60135114	4.541779471	3.590106186	3.616775012	4.040789109	3.936954244	9.302904307	6.944387426	8.420150386	8.352525599
FBXO34	6.994800763	7.599841723	7.000933511	7.704193152	7.553130859	8.146036374	7.229491941	7.086729383	7.833429951	6.904045117	6.991600737	6.451146768
FBXO36	0.100498113	0.327441483	0.334301278	0.290209011	0.359787398	0.442319572	0.177167026	0.224772373	0.812441133	1.605775396	0.78869981	1.015988559
FBXO38	9.409580502	8.627421282	9.018357813	8.898522314	9.163681745	8.565856671	8.810836924	7.782269371	10.99125106	10.34909764	10.78760003	11.14072996
FBXO39	2.517783892	1.539333892	0.956615403	1.011361864	0.53928541	0.729292195	1.493750783	0.915977757	1.159221646	0.601724273	0.521551023	0.604667174
FBXO4	1.598163758	1.705682361	1.696508292	2.010736039	2.599144005	2.793524145	1.905267301	2.191181089	2.97049508	2.946600086	3.006249217	3.287126531
FBXO40	0	0	0	0	0	0	0	0	0	0	0	0
FBXO41	6.443602791	5.776645584	6.43525319	4.051458516	6.619081497	6.433246056	6.165421259	4.843328776	4.03177339	3.947821826	4.688000092	3.363250541
FBXO42	6.118840484	6.556811366	6.528563391	7.482746644	6.199981912	6.140050952	6.512510575	6.7931464	6.990425545	6.294161145	5.996650088	8.518963184
FBXO43	1.804176391	1.901554302	1.814086915	3.525386732	3.642173032	3.999010553	2.056322249	3.457233349	4.041321457	4.632642971	3.80581178	3.504820715
FBXO44	2.684315075	3.549511434	2.391755896	2.4223478	2.745702555	2.869211898	2.492828891	2.264786176	6.9304122	7.058298789	6.535259537	8.836680397
FBXO45	7.931114413	7.356147916	8.493742895	8.385921689	9.428170292	9.359481121	9.140485606	8.146371433	10.19381485	9.839208421	11.3638505	8.009610509
FBXO46	7.096395157	8.810361151	7.026346856	5.292467883	8.530354166	7.552213694	7.135750178	6.635864936	5.791989265	4.863428888	5.686750505	5.315503027
FBXO47	0	0	0	0	0	0	0	0	0	0	0.022509399	0.021747145
FBXO48	0.886229789	1.0385873	0.79525903	1.324092898	1.005850316	0.811198998	0.931168239	0.754069865	1.824048824	1.732316039	1.369664318	1.283182788
FBXO5	15.94676613	18.0734299	21.30312284	34.82654569	35.53021828	29.92076091	17.86149191	32.78946878	37.89753441	33.57838044	37.3257587	34.71617757
FBXO6	4.098516921	3.01535793	3.554301339	3.287162525	2.319258518	3.041361847	3.562116458	3.182462007	5.179600133	5.103081025	4.528703136	5.943863464
FBXO7	39.24233168	39.35471399	37.6377284	45.53284409	45.32194807	42.88380678	43.60025552	47.46147938	50.8682862	50.65398903	49.36474889	51.61989442
FBXO8	2.859445263	3.897218461	2.687849582	3.253813506	3.173687622	3.532069444	2.878827017	3.150175199	7.202817944	7.476957136	7.107901229	6.575864746
FBXO9	16.11164414	16.8323462	16.03931326	17.78826308	16.5082683	15.63753402	16.38648782	16.33564739	21.99615768	17.35892715	20.38189115	27.00295064
FBXW10	0	0.027027191	0	0.026349403	0	0	0.013404837	0.01275507	0.014185624	0	0	0
FBXW11	7.810196521	6.323875658	8.138147679	6.471508426	7.220200475	5.891129967	8.052402433	6.153382765	11.78931877	12.59527811	12.25666656	12.08684941
FBXW12	0.028821404	0.059758031	0	0	0	0	0	0	0	0.063207685	0.031045369	0
FBXW2	11.7608746	10.46837932	11.80287318	12.94007822	12.41128797	11.89667366	12.77956566	11.70312759	11.28647186	10.72920355	10.03655241	13.00397799
FBXW4	11.47543422	10.03548811	10.91915655	10.8862204	11.55530867	10.36133598	11.1698523	10.6284205	12.11720691	9.443689982	10.91677358	11.75314726
FBXW5	27.92339763	28.25936371	29.36701028	30.03493673	27.53846797	27.51652464	27.00720539	34.25690522	54.36519668	48.97605887	53.50127626	58.09597694
FBXW7	3.20888278	2.734392231	3.145460815	3.237066168	3.160269451	3.070718954	3.143617511	2.961504213	4.616406531	3.851878063	4.327161296	4.686603122
FBXW8	6.915735034	6.000791332	6.070683623	6.449993041	6.161135111	6.899094251	6.528763375	6.39292477	3.615947263	3.932661069	3.40867652	3.684318632
FBXW9	7.090137528	6.595717796	7.875234288	6.375816359	6.840376787	8.194937743	7.09709568	8.283075899	3.197820651	2.985692289	3.833138719	3.14222278
FCAMR	0.174192222	0.114917261	0.083803386	0	0.033070511	0.032525285	0.1139924	0.061981081	0.034466308	0.069457769	0.068230375	0.016479958
FCAR	0.739904584	0.728702862	0.469875129	0.224345831	0	0.018996376	0.095110292	0.126700068	0.02013003	0.060850163	0.059774875	0.019250223
FCER1A	0.045912595	0.047597373	0.097189047	0.046403727	0.095881909	0.047150562	0.047214307	0	0.049964383	0.050345029	0	0.04778063
FCER1G	45.88835647	39.54914888	44.61783036	17.48423356	8.366223823	10.37886785	24.71824758	13.72005239	146.7424344	82.7658652	144.0706306	73.2439066
FCER2	1.408143806	0.813668013	0.952877937	0.186650085	0.168729125	0.142240563	0.284865729	0.045176258	0.276336691	0.227816117	0.422715128	0.120117758
FCF1	14.95279004	14.14245339	15.60944139	18.0317784	15.95559641	16.51292728	17.31462048	17.47748515	24.7206346	25.87477703	26.63105303	24.5568068
FCGBP	0.025172101	0.0347944	0.017761666	0.02544137	0.013142086	0.004308472	0.021571483	0.024631028	0.03652472	0.023001861	0.063267102	0.034928364
FCGR1A	19.56885886	19.01278951	20.50869248	12.20851929	17.22500358	16.38880838	16.6281982	13.8491107	29.98968554	20.51044276	32.06304092	16.06820652
FCGR1B	1.928456774	2.232141265	2.060921801	1.362467689	2.130953741	1.692039093	1.906117474	1.337391708	2.791399171	2.586830608	3.892348228	1.59774172
FCGR2A	23.12999779	25.71800551	18.52821124	11.23005781	16.15388409	19.07919231	18.35549397	12.91766195	27.1166782	19.10517309	19.74519575	19.66329849
FCGR2B	9.343601396	29.45679801	8.3848559	3.971134306	19.67955547	20.09322106	9.805608556	6.814092238	6.118286283	5.709535778	3.802177652	2.742599676
FCGR3A	0	0	0.066224164	0.105397614	0	0.171350259	0.128686437	0.163264894	0.068090997	0.09147965	0.06739733	0.043409999
FCGR3B	0	0	0	0.198413603	0	0.30241039	0.302819233	0.076837546	0.042727659	0.021526587	0.021146189	0.040860196
FCGRT	17.74870332	22.46233553	16.75241259	17.99033421	8.691437199	14.01895498	14.01157035	18.04384026	34.00336679	30.38683153	30.26367868	41.47276849
FCHO1	8.839505442	11.53570048	8.380958706	6.87697604	10.14526955	12.52590737	7.898476475	7.806356298	6.709463468	8.976093392	8.401406522	6.910962943
FCHO2	2.368877946	2.034582584	2.182689581	1.983559362	2.913804631	2.141451023	2.380854922	1.515300117	7.40008178	5.92649721	5.747449173	5.337407511
FCHSD1	7.343504834	7.9034566	5.49880401	6.065084977	6.704868572	6.354533096	6.207047471	6.76297105	13.09905902	13.58291195	10.85288571	15.5275749
FCHSD2	8.499084778	9.582479915	7.682746406	9.287665594	8.052427545	10.34541357	7.098518229	8.73854974	15.00049159	13.94390807	12.51247842	11.83059518
FCMR	0.714209128	0.451473954	0.294996601	0.105636456	0.345597013	0.35778866	0.51949494	0.15340769	0.208527299	0.191014485	0.337750296	0.072513951
FCN1	0.035055089	0.087219474	0.029682231	0.127548278	0.036603778	0.115200956	0.122566496	0.089184113	0.015259481	0.053815065	0.022656041	0.02188882
FCN2	0	0	0	0	0	0	0	0	0	0	0	0
FCN3	0	0	0	0	0	0	0	0	0	0	0	0
FCRL1	0	0.007372026	0	0	0	0.007302823	0	0.00695823	0.185727259	0.116963871	0.099577395	0.140607781
FCRL2	0	0	0	0	0	0	0	0	0	0.019967639	0	0
FCRL3	0	0	0.011463925	0	0	0.022246561	0.011138319	0.010598416	0	0.011876887	0	0
FCRL4	0	0	0	0	0	0	0	0	0	0	0	0
FCRL5	0	0	0	0	0	0.010201303	0	0	0	0	0	0
FCRL6	0.050883745	0.026375471	0	0	0.079697587	0.078383629	0.0523264	0.024895002	0.110748477	0.111592197	0.027405061	0.052954041
FCRLA	0.637608	0.472146608	0.40973224	0.138091837	0.689554448	0.280628651	0.562016094	0.089128952	1.065596722	0.998804475	0.833981348	0.189585777
FCRLB	1.993931356	2.370273946	1.80087846	1.531598128	1.859938492	1.501642896	1.339635981	1.300714633	0.636502794	0.962027843	0.544106902	0.553348803
FDCSP	0	0	0	0	0	0	0	0	0	0	0	0
FDFT1	60.04052893	78.97770227	56.22577976	67.89778172	68.36679533	83.22449276	59.47237669	66.25123418	129.4635366	178.3446555	165.799275	60.05808503
FDPS	99.00731071	133.3852449	104.0361617	109.8078921	162.7243532	157.347347	119.1744156	121.0431389	350.387536	421.0667223	539.6550989	133.4239794
FDX1	6.549246076	5.622615119	6.426367716	6.067695514	5.716628323	5.674925064	6.962935546	6.174828361	7.461305654	5.36743437	7.072981271	6.389731722
FDX1L	14.31105342	10.87988189	12.7234937	12.79272878	11.75539404	13.45585634	14.32435197	13.87896338	4.845245856	4.672923264	6.303163991	5.957329664
FDXACB1	2.579910452	2.41985916	2.823490789	4.132989003	4.031668525	3.947175612	3.122303993	4.121559947	2.75028996	2.174655676	3.02474644	3.433722434
FDXR	5.393109572	5.465017004	5.000653581	3.347252048	2.204959065	2.855071688	7.014537091	4.548784886	3.537963583	3.181771739	3.125546414	3.620482167
FECH	4.748540159	4.810397062	4.918823154	5.588267828	4.331930479	4.742972871	5.217634365	4.731337683	5.804700297	6.324444692	5.543147482	6.370861963
FEM1A	22.07535835	19.62022178	21.79436558	21.29284046	18.73867738	18.54474286	21.47764581	24.51840315	17.19882212	14.49014812	16.14905947	16.98613841
FEM1B	12.23974847	11.99178466	10.72362816	12.85939679	12.34657959	12.34476124	11.57672004	10.94163633	18.12987318	17.2323924	16.86277029	18.9493606
FEM1C	3.090821176	3.695140221	3.070893968	4.324708607	4.063429277	4.288211585	3.151891162	3.723615091	7.055098811	8.090679811	6.445341447	6.872184865
FEN1	32.15449369	40.13735586	45.14549737	60.07002943	56.62728747	58.75033944	41.86288615	65.1281866	72.91727219	67.30507684	82.82767844	87.68120267
FER	1.824387804	1.724050273	1.582408979	1.927112155	1.598951124	1.646991635	1.395231895	1.417826711	1.118125839	1.393860985	0.986129352	1.120663398
FER1L5	0.006625977	0	0	0	0	0	0	0	0.00721072	0.007265654	0.007137262	0
FER1L6	0	0.006972144	0.007118208	0	0	0	0	0	0.095145275	0.051622375	0.036221537	0.02799595
FERD3L	0	0	0	0	0	0	0	0	0	0	0	0
FERMT1	0.061861921	0.053443305	0.13095102	0.010420611	0.075360719	0.052941616	0.05301319	0.020177401	0.796634653	1.808909733	1.688097137	0.536490696
FERMT2	0.138446555	0.111632033	0.081407635	0	0.01606255	0	0	0	1.439682707	1.534990871	1.524435888	0.640353331
FERMT3	51.94321976	50.06110666	52.41984727	39.53299404	34.99623164	31.59469811	49.27436088	39.92702889	27.51532561	24.06275507	31.7424445	32.93692078
FES	47.31180257	43.84268364	43.80382013	35.5975649	40.55472532	35.54489187	44.02135654	40.37025551	70.51097163	60.84636339	59.05532071	82.77847516
FETUB	0	0	0	0	0	0	0	0	0	0	0	0
FEV	0.028637339	0	0.030310155	0	0	0.029409504	0.029449264	0	0.342810446	0.25121607	0.277623913	0.119210003

FEZ1	0.060613417	0.015709412	0.048115557	0	0.03164562	0	0.015582982	0	1.731517035	2.675219451	1.958717105	1.781998189
FEZ2	13.99670658	16.12257853	15.27013211	20.2402784	14.76496131	13.75982961	15.54994491	16.99689905	27.70386195	23.97954564	24.04547333	36.07932611
FEZF1	0.01271793	0	0.040382497	0	0	0	0	0	0.041520863	0.083674367	0.068496459	0.026470763
FEZF2	0	0	0	0	0	0	0	0	0	0	0	0
FFAR1	0	0	0	0	0	0	0	0	0	0	0	0
FFAR2	0.077843849	0.060525268	0.102988755	0.019669139	0.040641446	0.059957098	0.300190784	0.114255902	0.232962325	0.08535895	0.062887924	0.020252766
FFAR3	0	0	0	0	0	0	0	0	0	0	0.026883907	0
FFAR4	0	0	0	0	0	0	0	0	0.376062201	0.284195381	0.545052718	0.462376229
FGA	0	0	0	0	0	0	0	0	0	0	0	0
FGB	0	0	0	0	0	0	0	0	0	0.016263609	0	0
FGD1	0.012587032	0.039146752	0.06661144	0	0	0	0.025887798	0.012316474	1.013640005	1.173186391	0.989755408	0.82524693
FGD2	3.139633866	3.973579072	2.683584026	0.650660033	0.982467489	1.495175301	1.863857118	0.83345331	4.052623717	3.23638939	3.275214987	3.731198487
FGD3	8.327372876	8.477633344	8.443811428	7.558402782	7.913082777	7.308227699	8.833084803	8.075077871	15.54307334	10.8836375	13.04474537	16.66974181
FGD4	1.916352525	1.661676995	1.782115784	0.776784968	1.462482919	1.537032112	1.715899774	0.707514448	0.110051225	0.155245488	0.136162619	0.221006742
FGD5	0	0	0	0	0	0	0	0	0.069440589	0.039982636	0.049095124	0.01897303
FGD6	0.544584272	0.408410882	0.490549365	0.714361603	0.604939692	0.297483089	0.399166263	0.419500075	0.119789716	0.088938549	0.062404935	0.217049995
FGF1	0	0	0	0	0	0	0	0	0	0	0	0
FGF10	0	0	0	0	0	0	0	0	0.071703988	0.228792472	0.13011811	0.091426774
FGF11	1.297027157	1.185390471	1.011530527	1.793865377	1.336508055	0.771157684	1.140750369	1.519637322	0.798606106	0.673694093	0.588257062	0.710420537
FGF12	0	0	0	0	0	0	0	0	0	0.008626371	0	0
FGF13	0	0	0	0.014345864	0	0	0	0	0.16991317	0.342415256	0.076446457	0.280659213
FGF14	0	0	0	0	0	0	0	0	0	0	0	0
FGF16	0	0	0	0	0	0	0	0	0	0	0	0
FGF17	0.272964248	0.45522993	0.251225326	0.539773398	0.322200433	0.329076396	0.231885353	0.522580894	0.258307265	0.169178841	0.242892009	0.716351154
FGF18	0.054298245	0	0.057470011	0	0.028348536	0.02788116	0	0	0.059090068	0.059540236	0	0.141268665
FGF19	0	0	0	0	0	0	0	0	0	0.027610844	0	0
FGF2	0.031774172	0.016470068	0.050445333	0	0	0.008157729	0.008168758	0	0.23340315	0.261312552	0.179686415	0.190135027
FGF20	0	0	0	0	0	0	0	0	0.172908239	0.116150343	0.114097842	0.110234053
FGF21	0	0	0	0	0	0	0	0	0	0	0	0
FGF22	0	0	0	0	0	0	0	0	0	0	0	0.261270135
FGF23	0	0	0	0	0	0	0	0	0	0	0	0
FGF3	0	0	0	0	0	0	0	0	0	0	0	0
FGF4	0	0	0	0	0	0	0	0	0	0	0	0
FGF5	0	0	0	0	0	0	0	0	0.030858031	0.051821864	0.03054367	0.029509344
FGF6	0.025901112	0	0.013707047	0	0.013522695	0	0	0.012672185	0.056373773	0	0.013949869	0
FGF7	0	0	0.014469711	0	0	0	0	0	0.014877606	0	0	0.014227363
FGF8	0.145431246	0.150767896	0.307852872	0.146986942	0.151856213	0	0.249257513	0.237175353	0	0	0	0
FGF9	0.259688208	0.336521949	0.458095969	0.513996164	0.576216917	0.566716949	0.1669068	0.25410623	0	0	0	0.033781761
FGFBP1	0	0	0	0	0.041344223	0	0	0	0	0	0	0
FGFBP2	0	0	0	0.09288671	0	0.047190827	0.189018507	0.179856288	0.200028205	0.100776045	0	0.095642867
FGFBP3	1.427086638	2.088641062	1.954697642	1.951417906	2.388596354	2.392320944	1.942342086	2.423184689	0.616643113	0.713391422	0.497331311	0.720734657
FGFR1	14.14961776	16.99318138	14.04285423	23.28226857	17.11584643	18.53247086	13.0254149	21.76481565	19.7202667	19.97543923	18.69471108	29.12341106
FGFR1OP	11.32074808	8.824522463	11.00639938	12.34438516	14.33244622	13.19387822	11.07888265	12.13441416	21.17564381	22.1582273	19.87391392	21.79400399
FGFR1OP2	12.504317	13.37047996	12.69233412	14.88254455	14.71559012	13.33268207	13.61420797	12.8540006	35.00482478	39.16766564	31.66733079	39.30589376
FGFR2	0	0	0	0	0	0.012060336	0	0.005745627	0	0.045070997	0.006324935	0
FGFR3	3.279453407	3.562284267	2.34804207	4.0213098	4.028629971	3.107859086	3.422026963	3.98760694	3.044032184	1.427845044	2.428599279	3.701473259
FGFR4	0.048130198	0.033264235	0.118863885	0.048645052	0.033504352	0.032951973	0.032996522	0.031397095	0.611073046	0.545359453	0.604847832	0.567669219
FGFRL1	5.924613113	4.259407731	5.237589675	3.44137325	2.619127293	2.587602152	3.443128986	3.409502003	5.582858509	5.899192879	5.843850316	9.839080939
FGG	0	0	0	0	0	0	0	0	0	0	0	0
FGGY	0.652523673	0.509802167	0.510473084	0.262845718	0.394986282	0.276787886	0.379274437	0.259100526	0.84904327	0.777737799	0.799647405	0.969400973
FGL1	0.211515569	0.372771253	0.111935498	0.085511271	0.110430029	0.086887513	0.130507471	0.124181435	0.759600033	0.255128977	0.387322705	0.176097166
FGL2	1.42466738	1.150188076	1.227660703	0.229365753	0.473928011	0.453166835	1.478024634	0.357763571	1.783639508	1.023037418	1.208667204	2.965265027
FGR	1.207159786	2.468627389	1.050143478	1.621192795	0.138135952	0.373610968	0.816253245	1.407745779	2.321455182	1.233035481	2.743116891	1.68650648
FH	60.58624451	56.57505587	71.17910157	70.21538031	74.15813914	74.17990664	70.12659516	72.26054509	37.43528277	42.66621565	43.60421743	34.18781814
FHAD1	0	0	0.014688016	0.007012917	0.01449047	0	0	0.020368638	0.045306195	0	0.037370537	0.007221006
FHDC1	0.239734779	0.256063202	0.192226209	0.337750473	0.166883971	0.179053734	0.074706585	0.227473161	0.442724773	0.438131577	0.344311457	0.514098167
FHIT	0.007180352	0.007443838	0.007599784	0	0	0	0	0	0.382886922	0.488160023	0.340314246	0.313844909
FHL1	0.051566422	0.05345867	0.068223265	0	0.0673057	0.039717627	0.172342401	0.063072506	3.87208408	6.488502114	5.165729219	3.421111453
FHL2	0	0.039619405	0	0	0	0	0	0	0.85258826	0.81717706	1.234979481	0.377833479
FHL3	10.64191433	12.636544	8.723401362	6.861978432	8.836890864	9.66409428	9.12510691	5.62372826	1.890084588	1.904483914	2.551131323	1.01875931
FHL5	0	0	0	0	0	0	0	0	0	0	0	0
FHOD1	14.83243025	14.07043748	16.00368074	14.17847058	19.03384191	16.08271597	15.33934292	15.05375872	11.23106625	9.448068863	11.27176753	11.15232307
FHOD3	0.013540403	0.014037273	0.021497024	0	0	0	0	0	0.07367672	0.037119008	0.021877845	0.056365273
FIBCD1	11.91809714	13.48018349	11.80874701	11.37456882	10.3310665	13.83590175	10.6240858	14.35556818	10.67899602	12.55374441	11.86095651	6.690208498
FIBIN	0	0	0	0	0	0	0	0	0	0	0	0
FIBP	30.20989354	33.69288816	33.75434237	40.87678351	28.0328532	33.58502008	33.57079689	44.82307476	29.37378089	27.27680668	30.72969634	38.22769657
FICD	1.531828807	1.72319213	1.27634943	1.614096464	1.191119293	1.271894261	1.139549187	1.594576872	1.383265915	1.429542686	1.685137357	1.899417526
FIG4	8.525194481	9.38446681	8.149972112	8.929081131	6.161882729	7.119373389	7.777089182	8.611041089	16.6596745	16.48503815	13.23146216	19.90250472
FIGLA	0	0	0	0	0	0	0	0	0	0	0	0
FIGN	0	0	0	0	0	0	0	0	9.079595492	5.057686715	6.855747825	3.832197576
FIGNL1	2.432588702	2.983664978	2.639399247	4.259531507	3.848848149	4.265294708	2.532995267	3.589681016	3.878219119	3.893397966	3.89516197	4.331381429
FIGNL2	0.017654055	0.009150939	0.009342648	0.053528709	0.018433989	0.054390215	0.045386457	0.069098331	0.124878173	0.116150343	0.085573381	0.091861711
FILIP1	0.017609761	0.012170639	0.006212805	0	0	0.006028194	0	0	0.070267353	0.057929461	0.07587438	0.024434995
FILIP1L	1.564808037	1.496563576	1.562906552	0.891012387	0.897515931	0.826134236	1.597841219	0.787152113	5.024761358	2.622148113	3.822172553	3.222545684
FIP1L1	13.40875529	12.96414047	13.73560686	15.75208661	15.79148147	14.6665327	14.61245418	14.36628268	14.77084828	15.16483348	14.56507608	17.18111607
FIS1	71.21211167	66.20118366	74.29293799	52.91331018	51.32082419	54.43790776	66.41717714	57.21285716	56.65650041	49.18361921	56.07932086	54.10448413
FITM1	0.160945845	0.16685181	0	0.216890004	0.22407497	0.495856557	0.220678636	0.472458978	0.52544797	0.294139453	0.173365016	0.614145451
FITM2	1.356736791	1.132369992	1.192600976	1.499078365	1.260601251	1.357895884	1.336084182	1.316323208	1.789280077	2.294614558	2.154986418	2.476874372
FIZ1	2.355024038	2.072387218	2.362163703	2.61546994	2.473362891	1.996688325	2.773798492	2.733129104	1.892340623	1.591466585	2.448255167	1.795384543
FJX1	0.160207103	0.145325215	0.10597838	0.08096043	0.167284849	0.267356145	0.144155629	0.058786299	0.370483949	0.373306425	0.301996223	0.187566092
FKBP10	0.274538576	0.170767719	0.251832026	0	0	0.018796074	0	0	0.199177745	0.521807392	0.433727034	0.171425201
FKBP11	13.80454703	12.85079243	12.05507637	9.681129489	12.3972368	11.77952931	13.078469	11.42060525	5.124394971	4.148400291	3.858333303	3.978979349
FKBP14	3.187694906	3.150962985	3.284228483	4.003159428	3.372756068	3.328026042	3.245400517	3.025911252	2.028390719	3.065765563	2.190247412	2.623054108
FKBP15	20.37395199	19.35084056	19.69715486	18.06445123	19.164128	18.01124075	18.67848924	16.66978602	18.07773579	17.84821116	15.81320338	18.01956268
FKBP1A	98.61942551	92.71281918	104.6653517	98.91183393	83.42206206	82.15738682	106.0706172	99.76899388	50.40643159	56.46337475	52.82438691	44.08125637
FKBP1B	0.012421413	0	0	0.012554286	0	0.025512677	0.012773584	0.036463247	0.702915366	0.735511592	0.57533547	0.581706013
FKBP2	15.53103943	15.23356482	14.99922617	17.7584224	14.52447856	14.9831564	16.45535605	16.62993363	16.9585623	13.18853848	14.30735931	14.80243001
FKBP3	19.06691602	17.16868944	20.64195971	21.52832288	20.57765062	17.72083042	22.57663645	21.26906444	19.20817544	18.74221458	20.08341491	16.22848372
FKBP4	20.71652075	21.60956649	23.169915	17.76503027	28.96870467	29.97158826	28.85906354	24.6911827	4.392740855	10.45661963	8.431420675	4.122959212
FKBP5	12.72838126	14.18747468	12.6627232	14.58390389	14.28988678	18.26590054	13.31834394	13.71314505	6.898281694	7.078526279	7.14977357	7.044648828
FKBP6	0	0	0	0	0	0	0	0	0	0	0.033368856	0
FKBP7	1.024843394	0.920060126	1.017613018	1.131912011	1.114248314	1.291182912	1.097359504	1.033829341	1.046299115	1.564027201	1.206350007	0.956588297
FKBP8	106.0045939	125.4081016	111.3550574	92.59848232	105.9321192	114.703191	119.9878562	114.3839477	88.03406581	93.80453602	90.64141029	74.90761793
FKBP9	0.890641007	1.269569666	0.91320839	0.815777529	0.377807784	0.304885289	0.562892225	0.41759219	15.91169192	12.93009652	14.82020802	10.30186639
FKBPL	4.283945694	4.233616379	3.940929063	4.491632228	4.974872795	6.208578306	3.828995992	6.307382039	5.838397654	5.970680738	6.339561338	4.70824075
FKRP	2.75946466	2.249840378	2.66205575	3.108553974	1.785851773	1.844967233	2.054377226	3.558010381	4.17602955	2.474275313	3.761937497	5.354595562
FKTN	3.475972194	3.348927401	3.159155823	3.497880525	3.408607921	3.899505748	3.508471886	2.902159018	4.411810829	5.170443701	4.077912306	4.769461051
FLAD1	20.30789697	18.2812206	22.11742143	21.23289635	20.5237197	20.39782676	22.16025357	23.29748026	16.38453451	16.07306382	16.68049029	20.12382789
FLCN	7.609529049	8.629260595	6.763772788	7.73905762	5.608735297	6.856209107	6.052590044	7.846684186	3.119652279	2.600369772	2.995542915	3.439224406
FLG	0	0	0	0	0	0	0	0	0	0	0	0
FLG2	0	0	0	0	0	0	0	0	0	0	0	0
FLI1	27.2210592	29.49869601	26.4678678	28.41795546	28.8529779	30.49935576	26.65932167	27.60564972	38.1592404	33.94138775	33.17126497	44.02891478
FLII	35.98572669	38.59932511	35.73120768	37.21896612	30.97820678	34.64553013	35.38765502	40.16268644	25.13082913	22.08829265	23.89539039	31.25068531
FLJ20712	0.019383848	0	0.061548395	0	0.020240201	0.019906505	0.019933418	0	0.021094473	0.021255178	0.020879576	0
FLJ44635	0.0237151	0	0	0	0	0	0	0.023205345	0.051615916	0	0	0.074039972
FLJ45513	0.461224807	0.584405084	0.542407441	0.466158588	0.722401697	0.289459545	0.658751999	0.376092345	0.139424422	0.028097321	0.138004056	0.453324419
FLNA	625.1204013	529.5558486	565.4922634	293.398718	232.1287269	244.5875949	400.0322988	274.4634431	2.181312278	2.475271638	1.729996795	2.230743444
FLNB	24.07921562	30.99636553	21.6789928	29.7923774	21.46970454	28.33543597	21.46263624	27.42798986	61.32691106	63.55314254	55.11212199	58.06078298
FLNC	0.017665647	0.048837051	0	0.035709237	0.043040883	0.036283952	0.018166503	0.017285925	0.076898565	0.071027371	0.057086379	0.134818973
FLOT1	21.67329535	25.03339178	22.58472362	25.99444361	14.23506536	16.59401701	18.84257872	22.94739692	8.441587824	11.69914319	10.21547268	15.71616605
FLOT2	47.73963309	53.75889588	48.57139955	62.97044481	41.17936846	42.02302619	44.8600502	62.7056268	67.0050721	67.03738479	71.28256197	78.38030638
FLRT1	1.48812012	1.819970944	1.552220092	1.817925075	3.067191172	2.848294578	1.938394258	2.283385509	3.295222161	3.675062034	4.358162568	4.785156406
FLRT2	0.002456609	0.007640265	0.002600109	0.002482888	0	0.002522848	0	0.004807609	0.058814904	0.04848789	0.060861906	0.107375536
FLRT3	0	0.009450131	0	0	0	0	0	0	0	0	0	0
FLT1	1.58682933	4.251275013	1.622908944	2.090351079	1.410255135	1.574685035	1.049681365	1.225602457	0.121268756	0.283486888	0.201656027	0.120607304
FLT3	128.1590453	99.05874346	124.150888	65.65622005	148.7607108	101.8056156	104.6704654	55.89670163	3.650872114	3.99857145	4.527811745	5.326657208
FLT3LG	0.870626603	1.053003646	0.793499425	0.733283172	0.707069825	0.769920996	1.06939875	1.11221916	1.368552525	1.591129196	1.458811418	1.132561999
FLT4	0.361090474	0.491781073	0.464614795	0.672658664	0.754085982	0.967058025	0.444137532	0.727441773	0.084755372	0.031054934	0.06101232	0.029473105
FLVCR1	6.243194168	7.237442931	6.231077251	7.485968826	7.25341922	8.40008907	6.41127167	7.009628186	5.887008899	6.350801628	6.032806013	5.503199837
FLVCR2	4.497052637	5.451264709	4.640375946	4.245947142	3.105950881	3.807571549	4.117156845	3.365814648	1.672216406	1.28303984	1.305922587	1.921890424
FLYWCH1	5.373077673	6.427833642	5.228315355	4.91297936	4.746673911	5.372319837	5.363379376	5.897436335	4.741246875	4.88103536	4.871159283	6.993713134
FLYWCH2	10.24582612	10.30708031	10.40251788	10.04859956	8.439906988	8.066935771	9.911941214	13.9244345	9.209091202	9.15441633	9.851037082	10.82066169
FMC1	0.088941423	0.04610258	0.047068414	0.134839261	0.139306112	0.137009401	0	0.087029634	0	0.097527891	0.143706703	0
FMN1	0.004140152	0.002146038	0.01533698	0.00836888	0.004323059	0.004251786	0.002128767	0	0.662311594	0.385886883	0.517316121	0.581661256
FMN2	0	0	0	0	0	0	0	0	0	0	0	0
FMNL1	16.17954935	15.73165139	14.55891342	13.7494217	13.67200998	13.58581813	14.53081172	14.47409337	18.40575922	16.80592879	18.85387367	16.45610794
FMNL2	1.081714868	1.686089792	1.055583343	0.852918241	0.736981106	1.079367383	0.71792134	0.795724323	0.617808814	1.295505225	1.032964493	0.590806746
FMNL3	4.43698309	4.286450947	3.879974875	4.69593959	3.322005018	3.139889227	3.191953728	3.704588144	1.556099442	1.058050498	1.460938807	1.758283155
FMO1	0	0	0	0	0	0	0	0	0	0	0.019154562	0
FMO2	0	0	0	0	0	0	0	0	0	0	0	0
FMO3	0	0	0	0	0	0	0	0	0	0	0	0
FMO4	0.835502289	0.499708465	0.544189012	0.682047501	0.436206857	0.594021054	0.710484389	0.4244936	2.692735619	1.444717437	2.146088759	2.05669291
FMO5	1.088225713	1.187884581	0.975636511	0.9122423	1.009303663	0.92035025	1.020336786	0.845603774	4.360869489	2.77263001	3.316628534	2.471519328
FMOD	0	0	0	0	0	0	0	0	0	0	0	0
FMR1	6.197065726	6.399176055	6.623617504	7.373006307	8.101293115	7.566836766	6.623660993	6.5532663	17.86883111	15.89145244	15.6106334	16.80855635
FMR1NB	0	0	0	0.053266571	0	0	0	0	0.057353827	0	0	0
FN1	0.005679109	0.005887506	0	0.017219578	0.01186001	0	0	0.005557037	0.407899205	0.348732978	0.354805152	0.212766265
FN3K	0.557460009	0.790832696	0.527915639	0.948923334	0.827177515	0.964195569	0.392234014	1.205792252	3.863440079	5.0510833	3.855685849	3.480847583

FN3KRP	8.617836086	10.81776021	10.76965281	16.29193907	12.49498995	13.59519241	9.849820042	15.59522334	20.28211701	19.15578579	20.52286082	25.85187949
FNBP1	23.52076238	22.0902075	21.63704512	18.2604124	22.18146716	20.08861458	22.86112307	17.39919252	16.46312401	12.02289103	13.55165913	15.70959854
FNBP1L	2.354866583	2.067068557	2.365073173	2.04128967	1.740974115	2.109447308	2.518850478	1.589427161	3.778555479	4.222002828	3.582683168	3.595521056
FNBP4	26.73353032	24.20222861	22.86733039	27.70247449	30.60165224	29.62181127	26.92032595	23.93067893	28.34731384	30.26854366	24.95753385	29.86351048
FNDC1	0	0	0.008693754	0	0	0	0	0	0.008938827	0.009006926	0	0
FNDC10	3.683469221	4.512932775	4.171266414	3.228224464	3.953786213	4.523474588	4.185235292	4.940150288	5.662406858	6.129224142	5.743452684	8.2832263
FNDC11	0.025538472	0.026475615	0	0	0	0	0	0.024989525	0	0.112015898	0.055018228	0.02657755
FNDC3A	15.20606051	13.9308662	13.52086689	12.94415635	15.03609508	14.07382465	12.14071099	9.942796541	11.29140731	10.52198339	9.432694796	10.27018587
FNDC3B	5.532913093	4.967608897	4.869403599	4.046852029	2.238905697	2.307306049	3.369769839	2.540512914	5.757416993	5.366821991	5.512989485	8.362967914
FNDC4	0	0	0	0	0	0	0	0	0	0.030827782	0.060566044	0.029257523
FNDC5	0	0.016757021	0	0	0	0	0	0	0	0.017724354	0.017411146	0
FNDC7	0.017561867	0.018206306	0	0	0.018337728	0.054106193	0.018059781	0	0.076446811	0.096286512	0.03783401	0.018276403
FNDC8	0	0	0	0	0.045643216	0	0	0	0	0	0	0
FNDC9	0	0	0	0	0	0	0	0	0	0	0	0
FNIP1	4.187234092	4.357486072	3.626810058	3.997362489	3.954226418	4.160361856	3.334435797	3.658520686	10.35072302	10.37690381	9.313887807	9.306765363
FNIP2	1.818158278	1.556277959	1.477054052	1.619586138	1.46178532	1.347264718	1.407938903	1.425846296	2.203779616	2.003339226	2.589142616	2.959608005
FNTA	56.1919204	54.03880974	57.08726249	58.14140516	68.72434874	61.81733706	58.373049	57.99903439	61.66938052	54.26730533	54.8822743	61.48639424
FNTB	0.095440867	0.118731724	0.141422302	0.212215982	0.279040504	0.176425728	0.215922968	0.093389373	0.228499762	0.293033431	0.472904996	0.317836957
FOCAD	5.751983674	5.598118715	5.551461739	5.841976111	5.079821779	5.567258026	5.017306207	5.476786343	9.914220154	11.25583904	10.24548759	9.715365676
FOLH1	0	0	0	0	0	0	0	0	0.416706698	0.375683282	0.347336052	0.461414148
FOLH1B	0	0	0	0	0	0	0	0	0	0	0	0.021848966
FOLR1	0	0	0	0	0	0.132625101	0.088536269	0	0	0	0	0.044799119
FOLR2	0.042038719	0.130744035	0.222471802	0	0.175583746	0.1295167	0.1296918	0	0.914972767	0.875846182	0.996216782	0.743735375
FOLR3	0.054243509	0	0	0.054823759	0	0	0.111562838	0	0	0.118960432	0	0
FOPNL	25.6171461	26.97871881	23.69446107	26.87294015	32.12696043	30.04294452	27.80696723	27.52010737	32.64708874	36.04171481	33.16616376	31.03213538
FOS	161.6530969	190.4621912	170.5152549	151.6635517	64.57055635	44.47980377	102.7213543	104.1103082	88.16301075	61.41048415	71.60607134	24.47032512
FOSB	35.83978961	43.37451835	37.17918589	20.30802111	5.579991919	1.800062607	12.2950757	10.1513061	0.744384624	0.093756951	0.307000549	0.074151084
FOSL1	6.415401152	6.71446074	6.205351528	5.118969092	6.859084374	5.548112212	6.060668701	5.346389756	1.336183846	1.514658782	2.479821891	0.830559823
FOSL2	16.50682726	15.13704874	14.30628963	9.283007029	15.99487549	13.47846758	12.94220392	9.53892154	24.53819016	24.5773174	21.73398886	24.90682064
FOXA1	1.15066096	1.474352087	1.163139455	2.417408984	1.83599342	1.938497585	0.850901188	1.669915074	0.01406974	0.028353856	0.041779219	0.05381922
FOXA2	0.020600904	0	0	0.020821274	0.086044102	0.021156378	0.06355494	0.020158089	0	0	0	0
FOXA3	0	0	0	0	0	0	0	0	0	0	0	0
FOXB1	0	0	0	0	0	0	0	0	0	0	0	0
FOXB2	0	0	0	0	0	0	0	0	0	0	0	0
FOXC1	0	0.048479828	0.032996976	0	0	0	0	0	1.475830928	1.367424666	1.561540684	1.881770645
FOXC2	0.031972407	0	0.033840036	0	0	0	0	0	0.034793973	0	0.034439515	0
FOXD1	14.28132271	12.15367088	15.54169208	11.29832728	21.07004949	14.86097719	17.31659523	14.25244319	56.08396451	34.56638304	46.35405614	44.68574102
FOXD2	0.322281862	0.501162163	0.35328998	0.51186041	0.480742662	0.496457596	0.378764785	0.58565824	1.052169753	1.224261885	0.942265129	1.090032042
FOXD3	0	0	0	0	0	0	0	0	0.339796463	0.599174014	0.532530159	0.270787712
FOXD4	0.467481324	0.280578573	0.390622643	0.273542229	0.488134053	0.353747791	0.632546501	0.216678717	1.686863372	0.593551091	2.173232602	1.946006544
FOXD4L1	0.078098056	0.13493982	0.08266006	0.052622321	0.054365553	0.106938478	0.08031229	0.025473115	0.113320285	0.057091799	0.084124388	0.243826845
FOXD4L3	0.024260397	0.075451923	0	0	0	0	0.024948227	0	0	0	0.026132418	0
FOXD4L4	0.086026476	0	0	0	0	0	0.044232748	0	0.046809158	0	0	0
FOXD4L5	0.017307675	0.017942786	0	0	0	0	0	0	0	0	0	0
FOXD4L6	0.019746628	0.020471237	0	0	0.020619009	0.020279067	0.020306484	0	0	0	0.02127035	0.041100109
FOXE1	0	0	0	0	0	0	0	0	2.774791724	2.335625335	2.39483491	3.252175286
FOXE3	0	0	0	0.027453392	0	0	0	0.026578964	0.059119896	0.119140584	0.058517621	0
FOXF1	0	0	0	0	0	0	0	0	2.674206119	2.280028517	2.692210279	2.557326762
FOXF2	1.599278215	1.811007152	1.796864156	1.467181046	2.954495584	2.981588528	3.036223203	2.816823326	2.168824336	2.266285983	3.021323373	2.688561673
FOXG1	0	0	0.017764436	0	0	0	0	0	0	0	0	0
FOXH1	0.246493637	0.255538808	0.286981491	0.174391287	0.180168388	0.151883991	0.076044665	0.120597637	0.160948027	0.135145154	0.079654197	0.282174937
FOXI1	0	0.02429622	0	0	0	0	0	0	0	0.051397539	0	0.024389764
FOXI2	0	0	0	0	0	0	0	0	0	0	0	0
FOXI3	0	0	0	0	0	0	0	0	0	0	0	0
FOXJ1	0	0	0.021564855	0	0	0	0	0	0	0	0	0
FOXJ2	4.91651753	5.247393781	4.608451623	6.582695727	5.143194817	4.704405188	4.962628003	5.29902195	5.222069789	4.764513707	4.211310566	4.974952746
FOXJ3	11.72440768	10.42264123	11.06350223	11.8199391	12.58201426	12.0481313	10.90891138	10.5175653	58.35713652	55.83488536	50.914567	61.48415984
FOXK1	9.189926718	8.770726995	9.149983402	7.211215713	7.966065168	8.024433221	7.954549107	6.263041423	10.03109175	8.65417746	9.958747917	9.741655333
FOXK2	17.98765062	15.43551524	16.53816987	15.59620285	15.6003255	16.43531312	16.38392086	16.49031279	15.602181	14.52122098	16.6769326	15.90998741
FOXL1	0	0	0	0	0	0	0	0	0	0.018496669	0	0
FOXL2	0.202915725	0.095618995	0.117146619	0.074576851	0.192618439	0.35994128	0.417337571	0.198554066	2.830549964	2.771203046	3.695878259	2.898811698
FOXL2NB	0.101357482	0.116752034	0.143037542	0.056912064	0.199911171	0.138787255	0.17371861	0.110198678	2.242813111	2.049963613	2.498976759	1.804903814
FOXM1	15.68901775	16.0843466	19.09168956	35.2162652	32.06780908	28.03973147	18.29594476	37.9963735	26.3677843	26.58456317	29.34789576	24.88336949
FOXN1	0	0	0	0	0	0	0	0	0	0	0	0
FOXN2	5.866800503	4.70843214	5.997814974	5.264167628	7.1788925	6.196154871	6.17882237	4.582769973	7.772096376	7.109401153	6.902981278	7.432407667
FOXN3	16.55781099	17.16540571	14.70975197	14.03986866	16.12974953	16.1506407	14.01842488	11.58858939	22.86753489	24.4417527	22.03289674	19.34218021
FOXN4	0	0	0	0	0	0	0	0	0.02731262	0	0.009011459	0.043531482
FOXO1	1.250439446	1.638849598	1.215882163	1.772424255	1.640064309	1.84793145	1.557706407	1.656284256	0.968042224	0.824924183	0.870575371	1.047400526
FOXO3	5.903282652	5.93699444	5.077080374	5.368301703	4.978091251	4.854494823	4.985797283	4.870009219	12.81249384	8.827076974	9.379826215	12.32901503
FOXO4	3.475321477	3.903087094	3.157231687	3.571039058	4.475589988	3.955673299	3.722764254	3.315604206	4.160220631	3.477383741	3.64990276	2.893376904
FOXO6	0.067571654	0.07005122	0.047679181	0.136588954	0.023518961	0	0.023162481	0.022039736	0.661813705	0.518665492	0.315404834	0.679768969
FOXP1	2.372562533	1.847777649	2.16134056	1.797462431	2.629390986	2.145605696	2.237521778	1.778711091	6.097320712	3.878687667	4.717123148	4.68022385
FOXP2	3.958876615	3.362612232	3.657930676	2.762921174	4.747555241	3.796520054	4.071118563	2.619479719	13.38716669	8.620012573	9.467027407	7.340734831
FOXP3	0	0	0	0.018650607	0	0.018950775	0.03795279	0.090282799	0.040163414	0.101173481	0.178894147	0.115224072
FOXP4	5.652979143	5.478535385	5.818240955	7.932960349	6.235580733	5.958177405	5.842391343	8.456631458	9.713454963	7.340591595	8.798031111	11.59640177
FOXQ1	0	0	0	0	0	0	0	0	0	0.050887774	0.024994266	0.120739325
FOXR1	0	0	0	0	0	0	0	0	0.030325353	0	0	0
FOXR2	0	0	0	0	0	0	0	0	0	0	0	0
FOXRED1	11.66322586	13.53099186	12.50951267	14.00808398	14.57571679	15.86355013	12.63513051	15.97476256	20.09518276	22.12858285	19.50883784	23.39063828
FOXRED2	6.10507054	7.34175398	7.046052462	11.2998387	8.326023238	8.723040026	7.228074415	11.12693099	6.043008769	6.10068906	7.239494557	7.292674276
FOXS1	0	0	0	0	0	0	0	0.039949111	0	0	0.043977013	0
FPGS	20.70450181	19.68513759	20.41003308	19.86289594	18.61332218	19.97411637	19.52671048	23.72631955	15.28217888	14.91297116	15.50415941	19.39605207
FPGT	2.200317932	2.07739336	2.010016621	2.779818767	2.112902614	2.10496818	2.053940149	2.281178348	2.879096489	2.635342021	2.221971115	3.482467726
FPGT-TNNI3I	0	0	0	0	0	0	0	0	0.016180784	0	0.080079723	0.046420751
FPR1	0.156993613	0.203443186	0.124623154	0.357014236	0	0.443373482	0.484334073	0.345642856	1.153226069	0.301262305	0.760985168	0.735215302
FPR2	0.11765939	0.040658981	0.06226616	0.059459004	0	0.080554604	0.120995265	0.057565155	0.128042836	0.215030519	0.337969118	0.224485382
FPR3	0.019696033	0.020418785	0.020846552	0	0.041132356	0	0	0	0	0	0	0
FRA10AC1	6.753309573	7.915618451	6.275366927	7.746342208	8.788152863	8.717519263	7.524750053	7.117555213	5.256236995	5.375566489	4.532882495	5.658582629
FRAS1	0	0	0	0	0	0	0	0	0.003415073	0.00688218	0.003380282	0
FRAT1	13.98520839	12.20474937	12.93307218	8.267530345	7.905573704	7.212417452	9.893953065	7.984336924	6.759270713	6.45464676	6.471770759	8.597341315
FRAT2	24.9403044	22.81962711	27.24951668	23.75188996	24.00361193	24.58526524	27.35389251	25.59815835	20.52858626	19.18645642	22.68523821	28.85022634
FREM1	0	0	0.00351062	0	0	0	0	0	0	0.018185408	0	0
FREM2	0.049451257	0.04101271	0.097701132	0	0	0	0	0	0	0.003615021	0	0
FREM3	0	0	0	0	0	0	0	0	0	0	0	0
FRG1	29.92286222	32.09057816	35.1654757	40.67155466	44.4430574	45.77664065	41.75338171	44.27288412	37.95338617	39.93968197	38.90047225	40.21397433
FRG2	0.025783211	0	0	0	0	0	0	0	0	0	0	0
FRG2B	0	0	0	0	0	0	0	0	0	0	0	0
FRG2C	0	0	0	0	0	0	0	0	0.01806796	0	0	0
FRK	0.012218338	0.012666694	0.012932058	0	0.012758129	0	0	0.023911412	0.039889821	0.066989526	0.039483449	0.025430926
FRMD1	0	0	0	0	0	0	0	0	0	0	0	0
FRMD3	0.018812887	0.039006466	0.039823639	0.042781795	0.044199037	0.043470337	0.048365674	0.041419138	0.214967817	0.355851921	0.233042424	0.709714215
FRMD4A	1.648113684	1.997091724	1.472719541	0.729104239	1.455733485	1.692552708	1.830983914	0.840712553	0.044103929	0.032589281	0.064026787	0.025305789
FRMD4B	2.828544627	2.537489454	2.95820109	2.400261422	3.000294337	2.899060177	2.488268192	2.345732624	5.455359641	3.893907915	4.168995236	4.400871096
FRMD5	0	0	0.00930449	0	0	0	0	0	0	0.009639663	0.037877277	0
FRMD6	0.008407744	0	0.017797744	0.016995365	0.008779187	0	0	0	0	0.009219433	0.009056516	0
FRMD7	0.026420406	0.009129971	0	0.00890101	0.009195875	0	0.018112985	0	0.009584003	0	0	0
FRMD8	10.71315084	9.783036268	9.866553264	9.914566983	8.266288071	8.468753645	11.38559424	10.60917213	13.18809361	8.256209097	11.58616145	16.08923586
FRMPD1	0	0	0	0	0	0.006305392	0	0	0	0	0	0.006389651
FRMPD2	0	0	0	0	0	0.007740644	0	0.007375393	0	0	0	0
FRMPD3	0	0	0.014378385	0	0	0.006975569	0	0	0.421335604	0.268133991	0.307295071	0.360507932
FRMPD4	0	0	0	0	0	0	0	0	0	0.006464108	0.01269976	0
FRRS1	3.681002298	3.76404055	3.116818877	3.027035193	3.197196564	3.281948876	3.320798321	2.766898286	8.048118663	7.100339824	6.920800437	5.502379363
FRRS1L	0	0	0	0	0	0	0	0	0	0	0	0
FRS2	3.869765089	3.357095752	3.396338077	3.851788005	3.849041063	3.046564583	3.783149441	3.27643768	5.21814163	4.831144188	4.318653138	5.020643632
FRS3	1.261510452	1.469830592	1.394279708	1.65863481	1.247300302	1.364314226	1.159512854	1.594881231	1.336391759	1.579162716	1.418979467	1.661378119
FRY	4.590181611	4.761861513	3.597400969	2.825285649	1.230892154	1.711536081	2.295851581	1.94591565	15.64933606	8.787739639	10.90255301	15.0597306
FRYL	7.518039489	6.62916595	6.911417781	6.982369491	7.384241638	6.832886555	6.686435212	5.161593151	17.71142308	13.35964584	14.11920665	14.16350055
FRZB	0	0	0	0	0	0	0	0	0	0	0	0
FSBP	0	0	0	0	0	0	0	0	0	0	0	0
FSCB	0	0	0	0	0	0	0	0	0	0	0	0
FSCN1	53.9827676	59.53498539	61.69884641	31.35511151	63.28048243	57.41474683	65.23058417	40.97738628	44.42133878	56.00772873	61.87971582	29.78196086
FSCN2	0.637619732	0.411938388	0.557497602	0.112076597	0.347368152	0.180310615	0.266080148	0.144675743	0.16090196	0.182393738	0.209032419	0.076934774
FSCN3	0.056911222	0.0884994	0	0.028760005	0.029712744	0.087668628	0.029262384	0.11137584	0.185800921	0.093608209	0.122605402	0.177680271
FSD1	0.058266985	0.030202556	0.061670581	0	0	0	0	0.02850727	0.063409049	0.191676365	0.03138154	0
FSD1L	1.540275742	1.389270779	1.300668045	1.101528681	1.358655666	1.364808267	1.555354517	0.9358586	0.835076932	0.804854543	0.65287028	0.763858081
FSD2	0.012684951	0.019725644	0.006712963	0.006410322	0	0	0.013044594	0	0.006902199	0.013909565	0.03415942	0.052804242
FSHB	0	0	0	0	0	0	0	0	0	0	0	0
FSHR	0	0	0	0	0	0	0	0	0.008679798	0	0.017182748	0.004150219
FSIP1	0.025033524	0.012976069	0.019871871	0.044277295	0.026139474	0.012854259	0.012871637	0.036743146	0.040864101	0.020587709	0.033706504	0.045591102
FSIP2	0	0	0	0	0	0	0	0	0	0	0	0
FST	0.032389383	0.022385282	0.125698354	0.163679279	0.101460912	0.088700576	0.088820494	0.095079525	0.035247747	0.082871312	0.046518221	0.157300278
FSTL1	0.135514198	0.032420063	0.121363928	0.073749744	0.076192869	0.096347177	0.032159144	0.112201126	17.92368194	18.88322868	18.20145712	12.10669723
FSTL3	3.450155037	4.198804859	3.583647728	3.811943319	3.378815848	4.093367301	3.085194545	4.487346341	1.375920815	1.715380059	0.969490862	1.181975961
FSTL4	0	0.007081	0.007229345	0	0	0	0	0	0.014866275	0.014979531	0	0
FSTL5	0.02225835	0.011537564	0.011779272	0.011248225	0.046483391	0.034287772	0	0.043559817	0	0	0	0
FTCD	0.669586961	0.684097435	0.472466716	0.215775241	0.810630097	0.74743632	0.488985253	0.180415805	0.137287167	0.117051058	0.125435608	0.171682828
FTCD-AS1	0	0	0	0	0	0	0	0.071248888	0	0	0	0
FTCDNL1	0.462715952	0.379294098	0.330293102	0.304526492	0.286524021	0.229307972	0.296013547	0.263238318	0.462563974	0.501487033	0.373815607	0.554333665

FTH1	1003.817577	804.898153	887.0082888	555.1800856	255.0578922	194.4682533	536.4224492	380.3092309	485.3331216	402.0843248	407.7127161	392.7213255
FTH1P18	0	0	0	0	0	0	0	0	0	0	0	0
FTHL17	0	0	0	0	0	0	0	0	0	0	0	0
FTL	2636.668482	2729.127247	2497.538465	3717.378111	1224.891224	1394.134412	1737.533634	2988.833231	838.4928095	1425.73937	1062.484123	1150.488375
FTMT	0	0	0	0	0	0	0	0	0	0	0	0
FTO	2.488115636	2.704011211	2.379048935	2.843025233	2.157117224	2.865431239	2.187886935	2.917746481	2.84908579	3.508348952	2.550650849	3.508804599
FTSJ1	20.20613915	19.60188528	20.47915213	19.40736103	21.43128687	23.79444415	20.57752943	21.56924692	15.13932181	17.4032019	18.12457461	17.153964
FTSJ3	33.74960618	31.45690748	36.98697177	35.98960541	34.96912135	32.75039982	39.85362055	36.91683933	19.73635385	17.23515066	21.38379355	20.72730443
FUBP1	35.01928682	30.24430726	36.47062039	44.69129992	49.06305787	44.36792392	42.36351617	42.7108874	42.6715258	41.35197934	37.50609383	39.1015753
FUBP3	16.15844929	14.72754022	15.433806	16.4146685	16.01098861	15.17285968	16.13588953	14.92326226	18.31254641	19.30631973	20.84093961	20.12564751
FUCA1	14.64053849	11.83800567	12.64304481	10.960852	2.914253041	3.767014069	9.249862088	8.504992868	7.688591547	7.834605723	4.724205248	14.87107687
FUCA2	30.98062236	28.63643296	30.44336269	34.08677953	32.74623076	30.10263599	32.05443791	35.29482009	46.51566417	49.48679277	41.67419196	55.6472574
FUK	4.737948755	5.039388924	4.265760063	4.301476076	5.011515487	5.013146232	4.872278933	5.188024384	1.741011645	1.529368194	1.590715709	1.515502884
FUNDC1	6.162804406	6.150557008	5.841310903	5.71741516	7.299481532	4.581422633	6.337532048	5.355297838	0	0	0	0
FUNDC2	22.80189219	20.38265985	23.76875522	27.9265272	27.90383522	24.01331668	23.50437799	29.11543506	13.61222642	14.63165153	14.04065561	11.16577234
FUOM	6.08503619	5.614742859	6.069568167	3.284361869	4.474318778	4.907678381	5.209172109	3.709708982	4.541818635	4.698690556	5.181892986	4.277002931
FURIN	20.70694487	19.90355798	18.83764766	16.05634501	16.86099871	13.65660053	19.04740995	15.54492897	20.60909533	16.2483361	15.61589589	26.73910351
FUS	127.5914143	142.2010166	146.6931865	194.1249855	158.4294299	179.1889728	151.3258478	199.1533499	147.5796199	138.6412609	162.4150571	132.5967284
FUT1	0	0	0	0	0	0	0	0	0.156503137	0.118271574	0.103272523	0.02494383
FUT10	3.134437751	3.07899373	2.947708887	3.479570565	3.219258898	3.134521809	2.779440251	2.694993276	4.048527322	3.684956152	3.453791356	4.812739587
FUT11	6.276243805	8.922588934	6.764974452	7.346195587	8.673776804	9.052099131	6.003343685	6.118757966	3.590836518	5.743564741	3.305706083	3.938172989
FUT2	0	0	0	0	0	0	0	0	0	0	0	0
FUT3	0.019746628	0	0.020900103	0.03991572	0	0.020279067	0	0.019322176	0.279360492	0.411406647	0.10635175	0.719251912
FUT4	27.45639961	25.15266202	26.74370022	27.98390289	18.75680331	21.89220548	20.17427996	21.69495658	43.49267373	36.86797795	42.25415864	36.84186813
FUT5	0	0.028060423	0.057296561	0.027356725	0	0.055594023	0	0	0.324014501	0.207761882	0.08746736	0.338021522
FUT6	0.087353183	0.075465533	0.061637209	0.058858408	0.045606168	0.089708537	0.014971636	0.056983689	0.031687369	0.031928774	0.047046837	0.015151217
FUT7	17.81020676	17.90915257	20.69079917	26.26889254	17.24706619	21.24345715	19.3008332	26.7408824	6.719402322	13.22343269	9.003992905	6.611303301
FUT8	0.958260507	0.77401764	1.020458444	1.259658663	1.025149718	0.845238086	0.828244073	1.000940624	7.280596147	6.388433845	7.472392693	8.987477591
FUT9	0.005695339	0	0	0	0	0	0	0	0	0.003122585	0	0
FUZ	0.043836709	0.022722656	0.046397378	0	0	0	0	0	0.691726866	0.504721736	0.920776555	0.593063694
FXN	2.616992299	2.122222021	3.08732329	2.425202613	2.763926967	2.741460877	3.022768368	2.502041326	0.873151269	0.994917683	1.235805555	0.87400734
FXR1	20.44198259	20.02663393	18.53363863	17.7992151	23.42243378	20.61840124	20.44653217	17.67000482	21.56060963	23.59658637	20.95421468	17.16964706
FXR2	15.00614804	15.1085887	16.98859328	16.76891205	14.23950672	14.33774871	15.70948188	16.79887536	6.704694983	7.585430114	6.519963987	8.980071156
FXYD1	0.573750078	0.594804039	0.520512853	0.497046476	0.3423415	0.6313076	0.295008511	0.481214882	0.267592949	0.179754377	0.176577924	0.04264958
FXYD2	0.080432826	0.083384337	0.085131213	0	0	0	0	0	0	0	0	0
FXYD3	0.06522371	0.045078078	0.069033674	0.021973805	0	0	0	0	0.141959411	0.047680303	0.117094351	0.022625818
FXYD4	0	0	0	0	0	0	0	0	0	0	0	0.072631516
FXYD5	104.2940749	123.2202793	112.8646502	140.4831857	109.781865	110.8575779	105.7580754	147.5565093	47.44798723	43.18771003	45.09814018	77.68689708
FXYD6	0.311681362	0.230799014	0.141380508	0.202509937	0.046493007	0.091452972	0.068682459	0.217844139	0.484553224	0.463832483	0.719425366	0.393868962
FXYD6-FXYD[illegible]	0	0	0	0	0	0	0	0	0	0	0	0
FXYD7	0.07546923	0	0	0	0	0.077504149	0	0.073847024	0	0	0	0
FYB1	5.362319273	6.946668807	4.025786966	2.654188014	1.123382151	2.749103417	3.737214581	2.378997234	3.595358982	2.861043341	2.837860491	3.676250128
FYB2	0	0	0	0	0	0	0	0	0	0	0	0
FYCO1	3.502625206	3.539948863	2.863352585	3.417829418	4.122432191	4.189991851	3.013861075	2.765543139	3.787796518	3.828712212	3.725517229	3.936975516
FYN	4.663420179	7.695569646	4.554314938	6.944725318	2.64643703	3.481975723	2.409402328	4.662379879	12.61386781	13.31520108	13.15270814	17.02122694
FYTTD1	23.10184114	22.46236892	22.51754834	27.53454336	17.8339386	17.67889922	22.40684615	23.12173362	16.9223937	16.7396716	14.86945114	24.36571806
FZD1	0.816420923	1.25674573	1.152148219	1.837843629	1.692062212	1.664165534	1.31023501	1.936659433	7.215454204	5.140840891	5.076645769	9.680729178
FZD10	0	0	0	0	0	0	0	0	0	0	0	0
FZD2	3.8606493	5.813584822	3.743165255	3.090228224	3.972358112	5.223625437	4.115000701	3.639793945	5.980026251	4.851367778	4.674575734	4.648245858
FZD3	3.272177227	3.174545207	3.079402494	3.125806185	3.564251613	3.31335326	2.532546887	2.264079654	1.632966368	1.821251887	1.517623547	1.589410313
FZD4	0.058219703	0.082989632	0.023107701	0.08090842	0.022796916	0.022421068	0.02245138	0.06408931	1.211714002	1.189025125	1.050428495	1.151181042
FZD5	0.863222706	1.026317698	0.90086854	1.604588214	0.9896037	1.140669102	0.831827742	1.228607706	1.622603713	1.383432156	1.072883229	2.726444031
FZD6	3.584567746	3.148760492	3.518821728	2.350744638	2.828626018	2.852243348	3.348530373	1.967958419	10.43715693	11.31179717	10.15398266	8.799216751
FZD7	0.26548472	0.478025452	0.251414511	0.338936392	0.189672392	0.286992774	0.143690387	0.232433078	3.740672874	3.61595231	3.220930819	3.271813097
FZD8	0.067557515	0.017509141	0.107255709	0.034140093	0.01763553	0.034689553	0.017368226	0	0.588155752	0.222238698	0.272889377	1.072169752
FZD9	0	0	0.048636022	0.046443355	0.023990947	0	0	0.067446108	0.950133975	0.327522146	0.470227314	1.123803691
FZR1	6.882842432	8.174742305	7.427736437	12.50020984	10.88103356	9.850260887	8.297301239	14.06404804	10.58496621	9.027183034	9.854112643	10.85467727
G0S2	0	0	0	0.508272604	0	0.631220191	1.14922467	0.492083442	3.344448742	2.144499587	3.85207584	2.267868177
G2E3	5.425486883	5.219456751	5.232614935	7.678770638	7.79080589	7.447702404	6.51386793	6.144768362	8.515227045	8.928883536	8.203328435	7.69854816
G3BP1	63.81225524	56.00056802	66.57549109	76.00495167	66.31429567	64.65571113	63.25971044	64.07499214	68.98926934	59.62875926	68.79757771	71.10971281
G3BP2	45.62048516	38.34036454	46.68985346	45.31582665	46.95527465	42.5514945	47.45857385	38.13176297	50.99877735	51.2644479	52.24562423	53.15814455
G6PC	0	0	0	0	0	0	0	0	0	0	0	0
G6PC2	0	0	0	0	0	0	0	0	0	0	0	0
G6PC3	23.21196743	22.24072826	23.79236561	26.68901792	20.10606029	21.70086635	21.82062176	28.41996295	18.30742895	21.7570596	24.1191403	24.64439557
G6PD	64.8496139	57.44766409	60.77982704	46.1463021	36.92913644	37.00143627	53.79502404	45.74419419	30.24539798	29.70160554	31.94231462	30.57116823
GAA	15.3221394	20.22573699	15.12020743	12.38600274	9.037855688	13.23259289	11.68058853	12.36151519	20.789553	27.67461806	21.6338061	31.80364322
GAB1	0.264220229	0.225276615	0.206473761	0.269542389	0.252687177	0.271344549	0.215845499	0.190885289	1.64192075	1.188698102	1.101195233	1.200104895
GAB2	16.51161021	13.91905551	14.43097553	9.565117384	9.982899011	10.52834655	11.21534834	9.733298991	12.56437873	11.12751741	10.75470683	9.199045416
GAB3	4.871692946	5.073366026	4.068890966	4.276230666	2.929880282	4.118157762	3.237635567	3.913028137	4.724572988	3.561339778	3.081930045	5.173373943
GAB4	0	0	0	0	0	0	0	0	0	0	0	0
GABARAP	107.2575793	113.1299489	110.078948	128.1370602	84.6891724	88.05781919	103.4203754	130.2467174	73.96832471	70.83167895	66.33467093	81.77218822
GABARAPL1	7.064807427	7.144321667	6.399446631	7.227992605	3.303774708	3.360583666	6.32911304	5.386163429	3.561134441	3.588264409	2.917927087	4.262501764
GABARAPL2	44.78759856	42.97938318	49.39318059	43.20418583	38.29898549	35.73730255	50.25449378	48.18636255	72.5841869	74.31489065	74.15858692	75.61527957
GABBR1	1.251773283	1.401524149	0.803769091	0.700057226	0.845241853	1.002709934	1.012647303	0.685925245	0.953569633	0.951683457	0.620247759	1.354812069
GABBR2	0.01674224	0.008678301	0.044300546	0	0	0	0	0	0.245966543	0.22030258	0.090170665	0.557549708
GABPA	10.49418347	9.319301246	10.69351296	12.31492741	14.88587964	12.38267766	11.19364028	10.15387868	13.14291007	11.71087364	11.75653223	11.72448345
GABPB1	3.247128673	3.358769181	3.682291895	4.930121022	5.842700504	5.255102876	4.226163818	4.978765582	6.168178478	5.42039107	5.925772236	5.031151067
GABPB2	7.450764906	7.058051983	6.786294317	8.138430972	8.522235463	7.97457837	6.312370523	7.999606488	10.60773305	8.304985617	9.159598495	10.62783979
GABRA1	0	0	0	0	0	0	0	0	0	0	0	0
GABRA2	0	0	0	0	0	0	0	0	0.01721964	0.005783609	0	0
GABRA3	0	0	0	0	0	0	0	0	1.103980069	1.954629153	1.170785827	0.754092362
GABRA4	0.741549949	0.58705415	0.727785479	0.272539056	0.84470256	0.715390552	0.577707841	0.228677213	0.024454296	0.064065553	0.053251377	0.009354197
GABRA5	0	0	0	0	0	0	0	0	0	0	0	0
GABRA6	0	0	0	0	0	0	0	0	0	0	0	0
GABRB1	0.009483532	0.013108711	0.010037501	0.003194993	0.013203337	0.009739242	0.006501606	0.006186456	0	0	0	0
GABRB2	0.007262729	0	0	0.00734042	0.015167175	0.007458558	0	0.014213235	0.221302632	0.334482889	0.289456477	0.136048074
GABRB3	0.00826187	0.008565042	0.008744477	0.008350249	0	0	0	0.016168564	0	0.027178431	0	0
GABRD	0	0	0	0	0	0	0	0	0.925778159	0.932831061	0.872185636	0.618654501
GABRE	0	0	0	0	0	0	0	0	0.013755757	0.006930277	0.027231244	0.013154545
GABRG1	0	0	0	0	0	0	0	0	0	0	0	0
GABRG2	0	0	0	0	0	0	0	0	1.335765982	1.070964852	1.151557026	1.002678346
GABRG3	0	0	0	0	0	0	0	0	0	0	0	0
GABRP	0	0	0	0	0	0	0	0	0	0	0	0
GABRQ	0	0	0	0	0	0	0	0	0.178096889	0.233289801	0.211539064	0.102187772
GABRR1	0.015339099	0	0	0	0	0	0	0	0	0	0	0
GABRR2	0.061906996	0.070596565	0.085180184	0.050055378	0.084034558	0.069933852	0.031831091	0.078749203	0.633280737	0.936792874	1.13362743	0.618487608
GABRR3	0	0	0	0	0	0	0	0	0	0	0	0
GAD1	1.212944797	1.60263775	1.271211251	1.730710338	1.341032984	1.331135908	0.917157479	1.186872638	0.077646308	0.104317126	0.064046081	0.037126342
GAD2	0	0	0	0	0	0	0	0	0	0	0	0
GADD45A	8.149419	8.933544127	8.831808115	4.847373723	4.071507147	4.645082024	9.583409509	5.112675649	12.13598661	12.27119959	12.81037653	11.48383215
GADD45B	20.43543961	20.5064585	18.90459505	25.56080102	18.83896438	17.9718235	18.06578288	24.32684603	9.116707247	7.452923491	7.856328276	8.85924669
GADD45G	0	0	0	0.050874807	0	0	0.155290462	0	0	0	0	0
GADD45GIP1	40.9037291	40.43069371	45.30843807	39.27421743	49.8538174	46.42457922	49.67836173	53.13598271	26.32435409	26.60223431	32.36131816	24.66006554
GADL1	0	0	0	0	0	0	0	0	0	0	0	0
GAGE1	0	0	0	0	0	0	0	0	0	0	0	0
GAGE10	0	0	0	0	0	0	0	0	0.292791285	0	0	0.093331498
GAGE12B	0	0	0	0	0	0	0	0	0	0	0	0
GAGE12C	0	0	0	0	0	0	0	0	0	0	0	0
GAGE12D	0	0	0	0	0	0	0	0	0	0	0	0
GAGE12E	0	0	0	0	0	0	0	0	0	0	0	0
GAGE12F	0	0	0	0	0	0	0	0	0	0	0	0
GAGE12G	0	0	0	0	0	0	0	0	0	0	0	0
GAGE12H	0	0	0	0	0	0	0	0	0	0	0	0
GAGE12J	0	0	0	0	0	0	0	0	0	0	0	0
GAGE13	0	0	0	0	0	0	0	0	0	0	0	0
GAGE2A	0	0	0	0	0	0	0	0	0	0	0	0
GAK	19.3418074	17.89371024	17.95118808	18.14027505	17.2677016	16.53918986	17.79504989	20.2205886	17.66057908	13.82990581	15.75159886	22.46931722
GAL	0.394691155	0.340978739	0.556995416	0.066485536	1.373760358	1.68888932	1.96176023	0.514943064	5.369033349	9.665753255	11.47896821	1.095332985
GAL3ST1	0	0.009997154	0	0	0	0	0	0	0.15741467	0.074019824	0.020774804	0.06021387
GAL3ST2	0.482763486	0.192491793	0.432353757	0.112598693	0.504091361	0.190684812	0.267319652	0.218024548	0.121238627	0	0	0.077293166
GAL3ST3	0	0	0	0	0	0	0	0	0.016448949	0	0	0
GAL3ST4	0.063554599	0.197660274	0.04484471	0	0.044241574	0.043512172	0.065356497	0.062188498	1.590755802	1.277653775	0.684587051	2.777898131
GALC	5.301657786	4.791562453	5.335576354	6.331578576	5.393932669	4.700047423	5.347124643	4.954917676	10.78704735	11.3661058	9.554529256	11.54166778
GALE	11.55544391	10.62840639	11.83195564	8.078744098	10.40272267	10.82605324	13.1636944	10.74029055	4.664489798	6.065573466	5.615235015	4.098950615
GALK1	15.10305088	16.64096503	15.85973851	14.94493244	16.67852068	20.26081471	15.53125212	20.23327077	13.38105654	14.61019409	16.22587002	16.21129033
GALK2	2.716769359	2.593457834	2.639357499	2.447896247	2.49571211	2.757295608	2.66270796	2.190623758	2.366953535	2.647072169	2.617459227	2.860470847
GALM	8.930878233	11.75292598	9.128846719	10.36594915	7.749903259	9.862704049	10.35830729	11.09322779	3.860983982	5.321349411	5.183388429	4.456145796
GALNS	3.528495794	3.588166506	3.314108786	3.872501681	3.269535898	3.353938481	3.206129737	4.256512539	4.16231886	4.075887203	3.953088286	4.681174374
GALNT1	28.89565006	27.57229777	29.6041685	34.43354864	28.07875971	26.7087417	29.34003821	30.29121046	32.68154941	26.56580481	28.99595389	38.41717057
GALNT10	7.426369884	7.005796345	7.248188084	9.688157133	5.726223433	6.299840726	6.707856146	8.159612633	5.771272145	5.161396749	5.157774167	9.402098529
GALNT11	6.327809655	7.473125931	7.372091657	7.098301135	5.893381635	6.712663931	7.126950332	7.382523442	6.520486518	9.658265024	7.327917305	9.190428799
GALNT12	6.044029941	6.893364986	5.93381349	7.16287873	5.834558529	6.627812009	5.717392735	6.469986	9.577285034	8.629057862	7.794434708	13.48121639
GALNT13	0	0.017811022	0	0	0	0	0	0	0	0	0	0
GALNT14	0	0	0	0	0	0	0	0	0.132747537	0.083599284	0.213517185	0.15868206
GALNT15	0	0	0	0	0	0	0	0	0.00327617	0.003301129	0	0.003132981
GALNT16	0	0	0.008215923	0	0	0	0	0	0.059132689	0.102142598	0.125422036	0.113096449
GALNT17	0	0	0	0	0	0	0	0	0	0.008811884	0	0
GALNT18	0.72028546	0.922414611	0.773571242	0.549561415	0.523525289	0.783219098	0.686243224	0.466413735	0.049951269	0.050331815	0.049442398	0.033070216

GALNT2	16.40669883	17.71231611	17.20345719	18.08266129	14.89928982	19.08807579	14.35940191	17.19956921	15.13116559	15.07899903	13.85305735	22.44193613
GALNT3	3.695763268	3.538908804	3.51351482	3.811333906	3.417162867	3.360824813	3.926263222	3.22064399	0.184209826	0.20623689	0.111425855	0.274024671
GALNT4	0.019947937	0.10339967	0.095009274	0.07056463	0.062487635	0	0.092310753	0.019519158	0.151958406	0.098431764	0.182641142	0.145316883
GALNT5	0	0	0	0	0	0	0	0	0	0	0	0
GALNT6	6.880342176	7.102379991	5.811296892	6.340649886	4.506798508	5.668770213	5.696563299	6.071654521	3.962108335	3.713261827	4.30126866	4.593580097
GALNT7	8.455888633	7.526371237	7.959540229	7.494047288	6.882864468	6.18420088	7.979433935	6.092017462	8.222194808	5.92435083	6.99420218	7.211258381
GALNT8	0.050407083	0.18289876	0.160054655	0.280204616	0.184219012	0.258831188	0.311017337	0.345265104	0	0.027636709	0	0
GALNT9	0	0	0	0	0	0	0.0189504	0	0.08021679	0.202069775	0.059549696	0.095888525
GALNTL5	0	0	0	0	0	0	0	0	0	0	0	0
GALNTL6	0	0	0	0	0	0	0	0	0	0	0	0
GALP	0	0	0	0	0	0	0	0	0	0.06290445	0	0
GALR1	0	0.038142989	0	0	0.019209162	0	0.018918006	0	0.020019917	0.020172436	0.079263868	0.019144923
GALR2	0.018940359	0	0	0	0	0.019451059	0	0	0	0	0	0.019710982
GALR3	0.093015663	0.096428905	0.04922453	0.141015995	0	0.095523697	0.04782642	0.045508149	0.101224299	0.050997731	0.150289638	0.145200256
GALT	10.99170389	14.92870445	10.78251945	10.79961711	9.397791942	12.74333706	8.664581447	10.942815	39.26112324	41.28206405	33.78692904	49.78122751
GAMT	17.680627	21.58743541	19.65289912	14.47523212	12.95076901	16.01395366	19.78546796	18.87336352	14.69830527	15.57315631	16.4866549	14.18072873
GAN	1.059938822	0.708137242	0.997203437	1.023664806	1.106765567	1.185275758	1.017324163	0.749056761	1.409807021	1.665914706	1.268586204	1.40947108
GANAB	80.22718162	78.7728567	82.79887504	101.4284018	86.08955967	88.17861851	78.81107797	102.2888426	154.3210726	180.610456	168.0929261	138.5533149
GANC	10.32716787	9.921946475	9.287062	10.01916764	9.46977858	10.09864847	8.605691246	8.866385385	9.921456694	11.5511671	9.288624602	9.438109763
GAP43	0.01427688	0.014800775	0.030221693	0	0	0	0	0	0	0.03131036	0.015378536	0
GAPDH	2645.773812	2900.987477	2722.977137	2489.576089	2900.428792	2666.437782	2688.044722	2827.738162	971.101751	1287.113435	1150.786598	802.4165357
GAPDHS	0	0.037388822	0	0.072902371	0	0	0.037087914	0	0.039248162	0	0	0
GAPT	0.932868667	1.318773563	0.381480411	0.535708911	0.287796841	1.110434747	1.133738664	0.414916693	2.69949412	1.115921973	1.827004055	3.133114119
GAPVD1	9.7215231	9.049301692	9.642203422	9.588162547	9.387600002	9.059912138	10.19308058	8.545695136	10.54569402	10.09157489	9.650738208	10.43062493
GAR1	25.69438384	22.49856842	27.77965181	29.10271848	36.27529698	33.4092433	34.76463586	31.87434572	13.54188581	17.97607616	16.83496997	11.75667489
GAREM1	0.621169138	0.722019299	0.517994046	0.488299671	0.393097938	0.438165949	0.516186268	0.540281914	1.283693135	1.135228751	1.236822734	1.1688203
GAREM2	1.352226786	2.067528868	1.359254924	1.656827402	1.498735328	1.67573481	1.250731718	1.478391923	3.493929419	3.272038168	3.499021841	3.097510339
GARNL3	0	0.009115053	0.00930601	0.026659396	0	0.036117947	0.009041694	0	0.516690503	0.684527872	0.596664596	0.667960721
GARS	73.75310442	66.36029347	85.42917195	78.14077198	91.6742971	81.55539796	86.45862847	78.78365669	56.42490792	56.6573602	92.58789614	51.60642016
GART	45.48389885	41.68195569	48.71464948	38.846549	49.61951048	48.97467421	49.23913058	38.59957173	28.09904722	29.26437286	33.69202479	24.50108561
GAS1	0.81817932	1.045459141	0.825694496	1.480784294	0.993401673	1.34829266	0.469605385	1.452237768	5.984206609	5.174358147	4.918956463	6.415715426
GAS2	0.02794576	0.02897124	0.044367267	0.042367049	0.014590184	0.014349639	0.028738077	0.027345068	0.121647898	0.061287327	0.030102157	0.043624175
GAS2L1	1.894807095	1.642063555	1.833143167	1.750499237	1.483887942	1.383411755	1.918082848	2.042383189	2.319776896	1.444673815	2.630998928	2.449470403
GAS2L2	0	0.055525005	0	0.018044183	0.037283874	0.018334591	0	0	0.038857503	0.078307066	0.09615412	0.018579595
GAS2L3	4.808249372	4.913070675	4.094692198	5.760401091	5.900731966	5.980814812	3.523718483	5.151050377	3.886842843	5.605756454	4.249086248	2.465993363
GAS6	59.26481023	50.65682373	59.85097017	61.55070955	49.95859728	53.32034779	55.05139779	65.98751609	24.87859679	24.99830298	20.60098422	35.32238234
GAS7	28.17938397	25.9085061	25.42197724	9.590540372	10.24473229	10.38312755	22.66428114	10.02269409	8.122132329	6.284713598	8.479479265	5.671153588
GAS8	1.886769283	1.823072573	1.628607193	1.647754895	1.606703348	1.683680355	1.912006652	2.007532927	2.930404694	2.550997631	2.890685817	2.640288507
GAST	0	0	0	0	0	0	0	0	0	0	0	0
GATA1	0	0	0	0	0	0	0	0	0	0.154360692	0	0
GATA2	0.385987817	0.681740063	0.302618392	0.577950782	1.089701461	0.895560035	1.087885872	0.797347746	3.764903881	3.668178427	4.080725683	4.478126607
GATA3	0	0.016721859	0	0	0	0.016564886	0	0	0	0	0	0
GATA4	0	0	0	0	0	0	0	0	0	0	0	0
GATA5	0	0	0	0	0	0	0	0	0	0	0	0
GATA6	0.199505787	0.265920072	0.180994008	0.216042778	0.089279871	0.204885175	0.146544406	0.15338512	0.961496806	0.890691032	0.874951565	1.156756915
GATAD1	8.624727496	9.384336801	8.072918515	9.379401539	8.50984523	8.925563917	8.302717157	8.643817006	15.87740209	16.9461813	13.19869333	16.90346284
GATAD2A	18.96830355	17.62774823	19.83169406	18.0449639	18.78926538	18.21670892	19.77771762	18.68981073	14.02200326	12.2461909	15.41733305	13.88161076
GATAD2B	13.51760379	14.20085534	12.36041717	12.1952572	13.62590977	13.52490279	12.59399532	11.0868938	24.06384063	23.05901218	21.26103014	22.49004874
GATB	9.566679652	7.544471452	8.799242969	6.892522487	9.536406933	9.478170905	8.45019805	7.969856594	6.923143692	8.032839141	8.046631498	6.093924056
GATC	15.18777389	15.24608411	14.60018334	17.1297918	16.46717615	16.54691697	16.64744457	16.96842252	12.5580443	11.80643084	11.50228724	14.72475753
GATD1	14.78813779	13.91963112	15.31873983	13.74837595	15.4994731	15.85255282	14.01709983	15.57569847	11.0747762	11.97146057	11.58037136	12.69154186
GATD3A	4.888863885	4.985176202	5.632506224	4.131134344	4.46883385	4.493924691	4.500000248	5.113153004	2.91308577	3.023161271	2.848877302	3.402971511
GATD3B	3.197607349	3.070685598	3.26564113	2.426694345	2.624237467	2.949682526	2.930594796	2.887347821	2.82046651	2.964982114	3.867336358	3.479497884
GATM	0.032691106	0.118617513	0.051901077	0.066081614	0.904586977	0.520374914	0.554696397	0.447837786	6.083512733	10.7541387	8.97948629	4.814000113
GBA	23.60314941	24.17797442	23.49488106	25.47846765	17.91857994	15.89176186	18.65833337	22.54590142	45.51861534	32.02214274	40.47158122	53.76395584
GBA2	19.23667336	18.17440598	18.49596691	21.80210838	16.71444127	16.92701498	18.24378489	23.0638704	23.80973559	19.48416726	20.01327726	26.59547599
GBE1	17.20725228	25.02095165	16.889192	14.6009875	24.94773634	22.21830635	16.3380699	14.11893625	42.38340451	74.6360592	59.2522353	19.08190938
GBF1	28.04732891	25.10580541	27.31874382	33.93627945	25.66913892	24.74486002	26.77696956	31.69360942	33.30130291	33.02889566	30.57070361	37.44825863
GBGT1	20.0185156	31.23802444	22.43259137	36.3448237	26.76991232	34.54114142	26.87477799	36.21007601	3.071535582	2.607769823	2.702439801	6.010566607
GBP1	0.016857632	0	0.01784235	0	0	0.017312174	0.052006737	0	0.018345319	0.055455239	0.018158428	0.017543515
GBP2	8.762118185	10.28490219	7.689245576	2.329101099	4.119187028	4.71980205	7.43068429	1.961904419	2.777018724	2.812451427	2.370076922	0.799403589
GBP3	3.172771144	3.256139844	3.324354938	2.787743457	2.96333338	2.66887548	3.328307875	2.449336221	6.714680143	7.342766586	5.907800314	6.188915338
GBP4	0.096323054	0.054467816	0.074145199	0	0	0	0.063034365	0.008568418	7.080355573	4.56095652	4.810491272	5.513317135
GBP5	2.52315315	3.097588199	1.939655434	0.832152136	1.234112803	1.841106097	2.403501862	0.805646978	0.115613538	0.160179693	0.085826807	0.193480894
GBP6	0	0	0.011701825	0	0	0	0	0	0.012031694	0.012123356	0	0.034517505
GBP7	0	0	0	0	0	0	0	0	0	0	0	0
GBX1	0	0	0	0	0	0	0	0	0	0	0	0
GBX2	0	0	0.031640434	0	0	0	0	0	0.715712031	0.590043743	0.740621769	1.088867477
GC	0	0	0	0	0	0	0	0	0	0	0	0
GCA	1.721105433	1.62865776	1.826937197	1.81536732	1.546377721	1.377015611	1.301701299	1.312039497	1.099838952	0.965576	0.824559801	0.973667511
GCAT	7.3045829	8.192450723	7.841324962	7.671724266	7.084422436	9.663906281	8.179981339	10.47971327	4.639398144	6.299500836	4.760139914	5.978626398
GCC1	5.183011913	5.251699743	5.182537698	6.962670423	5.901517572	5.509997321	5.597797719	6.06553579	9.140870234	7.596884597	9.188024176	10.69291655
GCC2	7.84010822	7.541893346	6.183564067	6.336523063	6.98560833	6.532990216	6.933791188	4.880748974	9.897976035	10.1103001	8.990151887	9.506408083
GCDH	11.624255	10.41347267	12.269839	12.54673981	12.48412285	14.17600258	12.53852351	14.72798377	7.356305848	6.598375881	7.162276854	8.234648758
GCFC2	2.217120318	2.535163743	2.405699229	2.486970278	2.998465776	3.042816123	2.713019734	2.492152557	2.89312905	3.015309343	2.672381019	3.463631684
GCG	0	0	0	0	0	0	0	0	0	0	0	0
GCGR	0	0.02320471	0.047381682	0	0	0.02298688	0	0	0.146152056	0.049088498	0.096442103	0.046588102
GCH1	2.898080958	3.264425558	3.155850647	3.942995134	1.585800376	1.659816984	2.306826004	2.699451012	1.69821978	1.695879221	1.410771658	2.24749594
GCHFR	13.6599306	17.91663937	13.97859289	18.76402731	17.65195357	18.75600417	13.9696076	20.75101383	3.53156389	4.634284661	4.227221024	3.691372013
GCK	0	0	0	0	0	0	0	0	0	0	0	0
GCKR	0	0	0	0	0	0	0	0	0.026188845	0	0	0
GCLC	7.313513599	5.697286857	6.823085286	11.05228738	6.278670325	6.505454192	5.852758141	9.277205147	11.2307676	8.586915186	8.902122366	20.78129614
GCLM	4.102905298	3.589678099	4.449575367	8.089229712	4.081834126	3.96262565	3.933328044	5.317228553	9.07666737	10.48535539	8.367103613	12.53772121
GCM1	0.038253242	0.026437972	0.01349592	0.090212365	0.093200851	0.039284686	0.039337797	0.024953994	0.041629093	0.083892475	0.068675004	0.079619287
GCM2	0	0	0	0	0	0	0	0	0	0	0	0
GCN1	56.06300694	51.63313433	55.17218558	55.98510145	50.92981966	53.17575968	52.06663927	54.75467189	30.68431052	31.54433211	31.24101056	33.38743761
GCNA	0.660168956	0.852490883	0.600664288	0.491643797	0.483743423	0.499556449	0.571693512	0.54398204	0.504160629	0.495301464	0.573878254	0.506231974
GCNT1	6.142570796	8.177907408	5.33164153	7.624769879	7.216729754	9.153875634	6.193190392	5.963518151	5.203047993	5.295376951	3.916447903	4.392233917
GCNT2	0.058605439	0.060755987	0.015507201	0.019744116	0.071393645	0.045139238	0.075333774	0.01911524	0.462385947	0.56230344	0.426111635	0.35069196
GCNT3	0	0	0	0	0	0	0	0	0	0.053109248	0.104341501	0
GCNT4	0	0	0.016575315	0	0	0	0	0	0.08521283	0.171724023	0.20242737	0.179274705
GCNT7	0	0	0	0	0	0	0	0	0	0	0	0
GCOM1	0	0	0	0	0	0	0	0	0	0	0	0
GCSAM	0	0	0.016647992	0.007948724	0.008212043	0.016153306	0.024262716	0	0.068469169	0.060366942	0.059300194	0.024553741
GCSAML	0.011748812	0.012179939	0	0	0	0	0	0	0	0.064415256	0	0
GCSH	7.409233854	8.385220817	9.247066614	7.894117989	9.896355238	9.003998986	9.96858446	8.277052416	3.393220862	5.314794477	5.187622358	3.309171876
GDA	0	0	0.007277381	0.006949293	0.028718016	0	0.007070683	0	0.127202961	0.143251101	0.1036882	0.11448791
GDAP1	2.554287073	2.261360111	2.440320812	2.261765046	1.687610209	1.555324816	2.266405742	1.681000862	3.997360543	3.705588727	3.944463765	4.551895369
GDAP1L1	0	0	0.019247307	0	0	0	0	0	0.039579761	0.019940647	0.039176549	0
GDAP2	1.230401544	1.109527391	1.203053089	1.231719846	1.219501509	0.926623543	1.269302631	1.142800927	3.285825545	3.353689396	2.579161175	3.634074881
GDE1	15.42880774	16.99583938	16.34934277	24.30210648	15.55883591	14.85335278	16.78412675	21.4960838	34.37373654	38.29092269	32.17914489	47.03225051
GDF1	0	0	0	0	0	0	0	0	0	0	0	0
GDF10	0	0	0	0	0	0	0	0	0	0	0	0
GDF11	7.395678545	7.203422972	7.077246085	8.059103638	7.346540668	8.05438267	7.381013676	7.983861826	5.650462267	5.262907909	5.874893945	5.846226009
GDF15	2.346347131	0.972978867	1.357595368	0.15809642	0.130666974	0.192769042	0.83646184	0.061224335	0.340454983	0.171524344	0.269589319	0.130229997
GDF2	0	0	0	0	0	0	0	0	0	0	0	0
GDF3	0	0	0	0	0	0	0	0	0	0.046978005	0	0
GDF5	0	0.021513352	0	0	0	0	0.042680423	0	0.022583208	0.091021017	0.111765722	0.302346542
GDF5OS	0	0	0	0	0	0	0	0	0	0	0.098073949	0
GDF6	0	0	0	0	0.015193834	0.029886673	0	0	0.585899273	1.132858458	0.548582917	0.984296491
GDF7	0	0	0	0	0	0	0.005677149	0	0.552719775	0.774859948	0.535195786	0.413657609
GDF9	0.091136445	0.094480729	0.1378001	0.03947629	0.08156806	0.053482176	0.107108963	0.127396391	0.368380035	0.442568499	0.280482476	0.338730334
GDI1	47.88855719	57.98760575	48.48217417	57.43214096	44.11603536	46.66290234	44.96400582	60.08079004	47.535804	61.76314397	54.62090413	56.02146994
GDI2	94.82524012	97.08257636	96.35507848	106.6278912	105.1439672	105.780626	102.0927398	104.7903734	98.88018978	97.06150811	102.3990098	90.42059959
GDNF	0	0	0	0	0	0	0	0	0.02576821	0	0.01275285	0
GDPD1	0.867437424	1.253078844	1.294381393	1.092302541	0.920608223	1.036863784	1.169876699	0.960108894	0.851137457	0.888807965	0.765878748	0.93232839
GDPD2	0	0	0	0	0	0	0	0	0.02310902	0.023285073	0.045747201	0.088396052
GDPD3	0.539075746	0.863688587	0.829913024	0.297186714	0.358203634	0.956237443	0.503963278	0.767255786	2.453260314	4.621471943	2.692210279	2.754044205
GDPD4	0.030293912	0.015702779	0.016031747	0	0.007908065	0	0	0	0.008241838	0.074741642	0.016315751	0
GDPD5	0.839978954	1.04672193	0.844144031	0.883265904	0.885947629	0.923621666	0.837618435	1.079293706	3.314792539	3.628461999	3.436392396	5.138971795
GDPGP1	1.464205738	1.593832048	1.43350538	2.515776507	1.14666936	1.766830988	1.693933711	1.934189204	1.712928608	1.284448965	1.537759485	2.399952807
GEM	0.022467458	0.023291909	0	0.04541559	0.046920082	0.069219781	0.023104454	0.021984521	2.909575194	1.108641689	1.476268861	0.350723792
GEMIN2	13.04356215	13.96621281	12.65159469	17.62992441	16.7503336	15.99434401	16.93688568	17.10648684	13.98279655	17.03527161	15.76960968	15.55975192
GEMIN4	10.5810539	10.51373426	12.10257784	12.82113053	13.45765163	14.28596117	11.31559425	14.28997332	4.111126262	3.984903561	4.797521255	4.802167232
GEMIN5	16.6785741	12.77956008	16.76755645	15.12600081	17.37382319	17.63958013	17.77606429	14.76113738	9.535763548	10.19801733	10.12506444	10.56974034
GEMIN6	18.4800512	17.38725871	19.641724	16.24492049	22.53958156	21.60978971	21.39945886	20.05825841	13.68894321	12.26064922	14.88771032	12.28258676
GEMIN7	11.85370036	11.15682434	9.822291268	8.722645398	9.255892915	8.596071333	10.10468283	9.055286224	11.99454154	9.377989933	10.97632263	13.93209319
GEMIN8	2.367806388	2.599087637	2.399649831	2.400955818	2.957199928	2.995857475	2.243962804	2.188193311	2.78728546	2.766095202	2.467164838	2.762096966
GEN1	6.752601931	6.202581114	6.811654859	10.88160903	11.01362093	9.871259705	7.556990117	9.656482088	12.28311169	11.61555547	12.27175722	11.15738015
GET4	9.457906531	10.87362909	10.63773214	10.78326619	11.51721734	11.35380111	11.58116301	13.79365507	13.85430028	13.81855319	15.15665239	14.93840357
GFAP	0	0	0	0	0	0	0	0	0.013483366	0	0.026692012	0.025788118
GFER	6.366012804	5.696510559	6.997934108	6.953354889	6.157457384	5.780670635	7.717981074	8.392994801	8.726614481	6.735659172	8.854257761	9.042578517
GFI1	30.11180289	34.54147141	29.4992235	47.40982991	27.53974682	33.33895434	25.53051372	44.85373424	65.81754513	60.72986781	57.72219249	104.5217845
GFI1B	0	0	0	0.018092205	0.0373831	0	0	0.017515944	0	0.078515468	0.019282004	0
GFM1	28.04319558	22.34297016	29.38194736	28.3574703	29.51837331	26.62087268	31.13603007	25.26787047	25.17774206	24.81130065	28.30298171	24.40109705
GFM2	13.24191473	11.44609381	14.25728991	10.41754347	12.69665923	11.43745745	14.27286181	10.21518592	15.39215697	16.84143631	16.23290449	15.32021729
GFOD1	2.848624073	2.213573529	2.787972235	2.969855767	1.948253541	1.700952368	2.288103548	2.460325967	0.901230361	0.73304155	0.789947194	1.308336965
GFOD2	1.398742846	1.418374685	1.359100463	1.660910689	1.755837457	1.695491342	1.776384651	1.892214612	2.212570508	1.785217576	1.753670864	3.030622224

GFPT1	4.781316969	5.233894674	4.912715921	4.881586207	5.411238482	5.584070274	4.398324284	3.822494401	7.722258581	10.44584611	8.095136889	8.345776952
GFPT2	0	0	0.018413444	0	0	0	0	0	0.113595067	0.228920948	0.224875669	0.144840346
GFRA1	0	0	0	0	0	0	0	0	0	0	0	0
GFRA2	0.010302424	0.02136095	0	0	0	0	0	0	0.706331648	0.180752439	0.499382857	0.664738987
GFRA3	0.055732326	0.057777443	0.029493931	0.028164251	0.029097255	0.028617534	0	0.081801546	0.181952119	0.244451059	0.270147764	0.202999633
GFRA4	0	0	0	0	0	0	0	0	0	0	0	0
GFRAL	0	0	0	0	0	0	0	0	0	0	0	0
GFY	0	0	0	0	0	0	0	0	0.030578724	0	0.030267208	0
GGA1	9.370010685	9.256838741	8.974257329	8.484351734	7.991707806	7.763627262	8.892979743	8.792313985	7.869690813	9.163830843	9.14334004	8.745862534
GGA2	35.93490034	35.13726305	34.25737919	34.83583616	31.99097862	31.68878467	31.97451463	33.51991565	69.64751471	52.85326776	57.85266769	73.13479013
GGA3	15.52708206	14.47229572	14.46011962	15.77125275	15.82126221	16.24040958	15.49066943	16.31636068	20.07986173	19.13164804	17.85448706	22.81989581
GGACT	3.596615684	3.466145135	3.612676426	2.196934942	2.597864635	2.904670437	3.00734608	2.451555878	5.652524814	6.241215457	5.942861245	5.569001458
GGCT	39.9716572	43.66811336	44.96950379	47.28966772	53.98339476	51.71812148	47.1141814	49.55569725	30.38316807	42.62910304	43.4494219	33.02499162
GGCX	2.991339049	3.215697861	3.648663226	3.88216122	2.90708433	2.759830323	3.296381807	3.562471848	5.766360658	5.090632888	4.293735141	6.55681034
GGH	13.03002318	16.85783897	16.80867015	34.36425488	23.83749188	23.14085922	18.8504418	30.19070969	17.46635611	23.18028992	21.54228154	17.07658728
GGN	0	0	0	0	0	0.019863572	0	0.018926286	0.084195912	0.084837346	0.083338179	0.020129007
GGNBP2	24.76409163	25.41863239	23.73531615	31.60554137	27.7290615	24.48272682	26.22262425	29.17780571	37.86890441	35.48948699	36.00004868	41.55263542
GGPS1	11.38090821	11.81884195	12.68842452	14.3337685	12.00642548	12.15046122	13.53667012	13.09135422	13.70693822	15.35789139	15.17090096	12.7001507
GGT1	8.425825054	9.55977892	7.042548226	4.769667004	5.701751743	7.093244346	8.925027077	6.386998362	9.995831515	10.82539933	11.60792183	12.94598199
GGT2	0	0.010131515	0	0	0.010204649	0	0	0	0.031906061	0.04286551	0.010527008	0.010170523
GGT5	1.499036247	2.159515565	1.936889089	0.216438804	0.548858018	0.639773761	0.940938093	0.342891718	0.127116332	0.128084749	0.230672482	0.162080749
GGT6	0	0	0	0	0	0	0	0	0	0	0	0
GGT7	6.283864966	5.971582476	4.988197006	3.01516122	5.451329033	5.377750318	6.217251242	3.990505037	4.455331856	4.17606896	4.085180529	3.616561146
GGTLC1	0	0	0	0	0	0	0	0	0	0	0	0
GGTLC2	0	0	0	0	0	0	0.048327658	0	0	0	0	0
GGTLC3	0	0.052527422	0.053627854	0.05121014	0	0	0	0.148737086	0	0.055559675	0.054577875	0.2636483
GH1	0	0	0	0	0	0	0	0	0.553742384	0.348725617	0.34256326	0.860503174
GH2	0	0	0	0	0	0	0	0	0	0	0	0
GHDC	14.07326976	15.33788277	13.83941352	10.31923712	13.76410333	13.95135838	11.89651538	11.81834959	9.563360325	9.682769124	10.01468489	9.145382295
GHITM	191.2667323	175.6208976	202.5556248	172.8600051	178.9305196	165.5557307	207.6480983	171.0951423	86.65378669	88.85591368	94.69366122	75.66368161
GHR	0	0	0	0	0	0	0	0	2.240141691	2.718254589	2.302239247	2.880620549
GHRH	0	0	0	0	0	0	0	0	0	0	0	0
GHRHR	0	0	0	0	0	0	0	0	0	0	0	0
GHRL	0.157108207	0.101795843	0.06235706	0.039697203	0.061518393	0.060504152	0.06058595	0.057649192	0.149601387	0.258413318	0.29615469	0.224813098
GHSR	0	0	0	0	0	0	0	0	0	0	0	0
GID4	4.147949617	4.353778049	4.007058431	3.795043484	4.806444712	4.610349677	4.297464018	3.906964693	2.318915985	2.132414913	2.050164066	2.529746487
GID8	16.26320136	15.49908961	17.9979537	19.90150737	18.1747223	16.73916308	18.46172192	19.44602164	17.98943519	15.66074991	16.83730538	19.15119332
GIF	0	0	0	0	0	0	0	0	0	0	0	0
GIGYF1	15.17842001	17.62474846	14.59371022	15.50212593	18.12006893	19.93884778	13.89193152	18.39923713	14.62870674	12.44999592	13.86352245	14.61232082
GIGYF2	17.96783092	15.87273887	16.11295022	17.35985432	17.33243639	17.3498547	16.842038	15.26409705	19.95507688	18.96674278	16.78865805	20.99609586
GIMAP1	0.487405443	0.492658683	0.42559823	0.24630964	0.318086496	0.200219053	0.400979477	0.250387567	1.219963689	1.549933746	0.971278549	1.268091006
GIMAP1-GIM.	0	0	0	0	0	0	0	0	0	0	0	0
GIMAP2	9.620836245	11.52021363	9.669737643	7.424725027	9.072435261	9.995134917	9.510132802	8.173427543	12.41775929	8.586915186	12.01008274	10.01227713
GIMAP4	0.160705508	0.055534218	0.085046463	0.054141532	0.027967545	0.220051602	0.11017455	0.078625577	1.807173687	1.027950773	1.327147023	0.418110245
GIMAP5	0.028667853	0	0	0	0	0	0	0	0	0	0	0
GIMAP6	0.550059401	0.541731823	0.771402353	0.166783037	0.157949089	0.141222741	0.438382368	0.107647183	13.24407296	8.489512588	10.70950977	11.63483382
GIMAP7	0	0	0	0.088503122	0	0.089927516	0.13507364	0.04284209	0.047647077	0.048010069	0	0
GIMAP8	0.536510464	0.467711875	0.387170505	0.049295417	0.076392656	0.11269978	0.225704288	0.095450584	1.194254053	0.842346609	0.774924205	0.609097472
GIMD1	0	0	0.035977752	0	0	0	0	0	0	0	0	0
GIN1	1.061332561	0.80320333	0.853730055	1.083412629	1.141467507	1.068150876	1.135080367	1.069674875	1.131895304	1.361639408	1.234687179	1.027198736
GINM1	16.19634676	16.76427699	14.17754991	20.7193851	16.83210768	17.86223057	16.47222934	19.13745809	29.79182506	28.51086901	25.78514029	39.22308106
GINS1	9.489430612	11.02522773	11.56750967	18.38393933	17.99076723	19.71316704	11.85981015	17.96500949	16.40844142	22.0842034	19.32612616	17.02843385
GINS2	14.63802461	22.31364868	21.18836525	31.70931864	30.90273925	35.02951102	24.47877765	33.5104654	20.79314382	23.05170896	26.279247	28.1418195
GINS3	6.52646284	7.395887318	7.884303894	11.66858699	10.90366983	11.74082516	9.095240893	12.02362981	8.79231045	7.551375387	10.59358616	10.21142614
GINS4	12.66107314	13.57578183	16.33575351	19.378786	19.05554363	18.05098269	16.31825747	18.76154578	18.09178843	17.75352856	21.13595701	17.88408352
GIP	0	0	0	0	0	0	0	0	0.082360418	0	0	0
GIPC1	10.541155	10.01253466	10.68959895	7.223465983	4.696640091	6.206174577	9.052265925	7.236402457	14.56448958	11.80087487	14.83225135	17.86221286
GIPC2	0	0.014026684	0	0.027349846	0	0.013895011	0	0.013239358	0	0.029672806	0.029148455	0
GIPC3	4.358943832	4.480601079	4.105292016	3.447297872	7.367285038	6.196252791	4.989028866	4.686954032	8.133835706	7.021115483	9.377328258	3.882761178
GIPR	2.76960975	3.507723677	2.166054056	1.958086699	2.6497831	3.13852503	2.104532247	1.748867548	4.305754196	4.832254796	3.629954565	3.691610978
GIT1	8.590766753	10.40655222	8.936550989	12.86822171	11.06942907	10.99703771	9.895554306	15.42212795	27.30287851	24.32185294	25.32125236	34.57565883
GIT2	28.73025262	27.14549464	27.21786358	26.59168997	24.43854687	24.45700967	24.76563069	24.28622464	20.29399914	18.48362987	18.40956072	23.20651534
GJA1	0.220739757	0.123221474	0.269577696	0	0.017730135	0.017437822	0.034922794	0	0	0.521338745	0.018290219	0
GJA10	0	0	0	0	0	0	0	0	0	0	0	0
GJA3	2.470482589	1.947311401	2.398694261	1.547671275	2.099942958	1.415321933	2.005125605	1.118787322	0.388833048	0.291047948	0.604798653	0.499326171
GJA4	0	0	0	0	0	0	0	0	0	0	0	0
GJA5	0	0	0	0	0	0	0	0	0.010302297	0.010380784	0.010197344	0
GJA8	0	0	0	0	0	0	0	0	0	0	0	0
GJA9	0	0	0	0	0	0	0	0	0	0	0	0
GJB1	0	0	0	0	0	0	0	0	0	0	0	0
GJB2	0	0	0	0	0	0	0	0	0	0	0	0
GJB3	0	0	0	0	0.071666835	0	0	0	0	0	0	0
GJB4	0	0	0	0	0	0	0	0	0	0	0	0
GJB5	0	0	0	0	0	0	0	0	0	0	0	0
GJB6	0	0	0	0	0	0	0	0	0.023348587	0	0	0
GJB7	0	0	0	0	0	0	0	0	0	0.025247914	0	0
GJC1	0.067052412	0.090366802	0.120647636	0.06776968	0.112023524	0.061974346	0.048267436	0.045927788	0.569164368	0.830840411	0.628369871	0.516376139
GJC2	0.180796509	0.234288625	0.119598449	0.182730512	0	0.092835714	0.232403057	0.08845515	0.172157833	0.099125366	0.194747429	0.235190671
GJC3	0	0.049985772	0	0	0	0.04951654	0.049583484	0	0.052471557	0	0.05193701	0
GJD2	0	0	0	0	0	0	0	0	0	0	0	0
GJD3	0.079875647	0	0.014090248	0	0	0.054686253	0.013690046	0	0.231799137	0.145978165	0.100379002	0.235522336
GJD4	0	0	0	0.034420993	0	0	0	0	0.074124376	0	0	0
GJE1	0	0	0	0	0	0	0	0	0	0	0	0
GK	9.517709829	6.335750032	10.30552057	6.652856977	9.846699292	8.435852621	7.726424825	6.055140343	7.258697039	5.860804942	6.111166801	8.001470992
GK2	0	0	0	0	0	0	0	0	0	0	0	0
GK5	2.169029614	1.96437262	2.072894679	2.296152705	2.3210925	2.35322062	2.114719792	1.935558057	2.424386439	3.264300237	2.589553821	2.49676619
GKAP1	1.91599895	1.914512971	1.294936683	2.239801023	1.614978768	1.825420553	1.25815697	1.671521534	2.411665671	2.379412779	1.591398127	2.95489685
GKN1	0	0	0	0	0	0	0	0	0	0	0	0
GKN2	0	0	0	0	0	0	0	0	0	0	0	0
GLA	39.65514182	43.07730131	43.17647384	43.07090574	22.62529056	26.8118445	30.43824467	37.42887994	35.34969538	31.33307044	33.35454879	37.200961
GLB1	18.08630334	20.10868131	17.53765186	22.57533269	13.33312341	14.44001544	14.67132844	20.59216821	40.6281018	44.65362359	37.3504089	67.55329933
GLB1L	0.534355123	0.81247976	0.471307359	0.558073561	0.4463698	0.512179027	0.3846536	0.488011252	1.279326371	1.523449586	1.630832443	1.631215855
GLB1L2	0	0	0	0	0	0	0.015044907	0	0	0.016042516	0	0
GLB1L3	0	0	0	0	0	0	0	0	0	0	0	0
GLCCI1	6.274414794	5.620302624	6.09444656	5.411290242	6.762574637	6.190001304	6.38305128	4.975339285	17.0397932	11.70487276	12.34436784	13.79530653
GLCE	1.59553924	1.553004953	1.557394234	2.275567552	1.559587442	1.661318456	1.449352062	1.929870118	1.663997915	1.666954979	1.589757624	1.637394703
GLDC	0.014126952	0.029290691	0.074760805	0	0	0.014507865	0.014527479	0	0.107615594	0.26334323	0.258689672	0.132315592
GLDN	0	0	0.006887505	0	0	0	0	0	0	0	0	0
GLE1	24.49498839	24.62734126	24.27376811	31.52402394	27.19269491	27.34900558	26.03428127	29.50171709	31.48437587	30.65825252	27.15203472	36.3066652
GLG1	14.22603726	14.77156962	13.42535544	15.99934442	12.01973752	12.81106692	13.66185312	13.54867326	23.80201327	24.11385849	20.9343461	31.01350433
GLI1	0.354182873	0.489572984	0.402640336	0.411004443	0.356132805	0.431090852	0.283285842	0.4749289	0.114204304	0.07192147	0.127170973	0.191122522
GLI2	0.011311659	0	0	0	0.015748525	0	0.015509822	0	0	0.004134565	0	0
GLI3	0.019319819	0.004005753	0.016358691	0	0.008069338	0	0.003973515	0.011342725	0.584489281	0.906716653	0.79080308	0.953018744
GLI4	19.15187646	20.06234608	21.4156028	15.5096944	18.82655204	18.59845664	17.51112908	22.2686994	13.29878511	15.11355531	12.25697978	19.13886416
GLIPR1	42.31179145	26.60193033	37.39584333	10.59540112	18.77130603	11.41882779	35.73555713	10.04614696	12.14443491	3.530176243	8.786050102	5.278930886
GLIPR1L1	0	0	0	0	0	0	0	0	0	0	0	0
GLIPR1L2	0	0	0	0	0.014018662	0	0	0	0.014610344	0.088329902	0	0
GLIPR2	11.20354572	15.85570284	11.14453993	15.88093908	7.087060562	10.83725624	11.32996526	14.3971074	14.97472492	12.9223403	14.39653547	16.88433321
GLIS1	0	0	0	0	0	0	0	0	0.084293804	0.058801835	0.070598908	0.055806676
GLIS2	0.011891616	0.012327983	0.03775875	0.012018822	0.012416972	0.036636768	0.012228766	0.058180031	0.543524154	0.899735211	0.935072788	0.556895127
GLIS3	0	0	0	0.004078684	0	0	0	0	0.043916497	0.048676175	0.021734552	0.041997074
GLMN	7.450311741	7.216426421	8.052578641	10.11446081	9.229864251	8.866961231	8.408218528	8.618461134	13.08929067	10.28119635	11.86778209	12.91145042
GLMP	9.75315707	10.48660599	9.791985185	8.646425044	11.28973478	11.64733642	13.58305001	13.19731606	28.99103768	18.85328792	22.66239579	39.96192781
GLO1	223.0980669	233.083338	214.4841187	172.613796	233.6130157	243.4213751	254.3201072	179.9253706	258.2914035	320.3300499	325.5426023	189.0338989
GLOD4	13.18300568	15.58673989	15.30739648	19.2458153	16.63101111	17.94754568	17.20999902	20.42854541	12.53249306	12.44335054	12.5499058	13.10612526
GLOD5	0	0	0	0	0	0	0	0	0	0	0	0
GLP1R	0	0.007581425	0	0	0	0	0	0	0	0	0	0
GLP2R	0	0	0	0	0	0	0	0	0	0	0	0
GLRA1	0	0	0	0	0	0	0	0	0	0	0	0
GLRA2	0	0	0	0.013948492	0	0	0	0	0	0	0	0
GLRA3	0	0	0	0	0	0	0	0	0	0	0	0
GLRA4	0	0	0	0	0	0	0	0	0	0.109368627	0	0.07784833
GLRB	0	0.033483867	0.034185343	0	0	0	0	0	2.442856462	2.53230058	2.417970231	2.840280283
GLRX	15.70797796	14.030144	13.98056815	9.873302623	10.30204269	10.83211643	13.34985956	10.09869192	16.14060523	19.5732243	15.27699909	16.00933537
GLRX2	11.61274711	10.92637711	13.06182021	12.55042352	13.52645152	10.90252637	14.38556715	13.23823625	6.756721969	4.959057103	7.142004796	6.620952427
GLRX3	24.56411598	24.1455651	26.36652423	27.1145941	31.92065496	31.65853709	32.38905978	29.32153991	13.81381446	14.27161253	15.98656932	13.4911305
GLRX5	35.65859883	35.34741102	38.81154524	46.58268497	42.46397676	44.07623254	39.88290494	49.41551514	41.45584552	41.01585032	45.04282693	41.0307901
GLS	17.37753806	14.17873103	16.70822745	13.24219078	16.52815512	13.19674587	16.73196345	12.49036977	11.77162102	11.647725	11.24744634	10.95061013
GLS2	0	0	0	0.019244575	0	0	0.019580739	0	0.020721252	0.020879113	0	0
GLT1D1	0	0	0	0.059320646	0	0.012055074	0.07242823	0.022972482	0.204391822	0.502000567	0.252887014	0.342052611
GLT6D1	0	0	0	0	0	0	0	0	0	0	0	0
GLT8D1	16.05656423	16.64576552	16.31470945	16.4920336	16.30485988	15.41768855	16.51179944	16.81122963	17.47355675	21.17202839	16.53886733	27.92634381
GLT8D2	0	0.020508868	0.041877045	0.059983642	0	0	0.061031435	0	0.150701397	0.260313416	0.191785049	0.43234444
GLTP	8.525670682	9.169709058	9.150483972	12.89503627	7.401229504	7.279206973	9.794016727	12.36708855	4.497795626	4.88236567	4.172382378	9.121898549
GLTPD2	0	0	0	0	0	0	0	0	0.041977245	0	0	0
GLUD1	76.81605533	69.81234876	78.50588444	70.47901147	85.08050107	78.50730668	80.35286043	71.49264261	49.88668805	55.1789434	54.75900638	44.4745675
GLUD2	0.414806037	0.215013745	0.463427343	0.41924327	0.409068769	0.402324538	0.308075881	0.270593208	3.009417922	2.931266563	3.599334917	2.638063757
GLUL	59.39490367	103.3027507	59.79814667	72.79684653	59.97445667	80.18975178	50.89800317	64.97684858	47.79107212	42.93919338	47.31081914	43.65831796

GLYAT	0	0	0	0	0	0	0	0	0	0	0	0
GLYATL1	0	0	0	0	0	0	0.014094541	0	0.029831002	0	0	0
GLYATL1B	0	0	0	0	0	0	0	0	0	0	0	0
GLYATL2	0	0	0.023360452	0	0	0.022666308	0	0	0	0	0	0
GLYATL3	0	0	0	0.025532943	0	0	0	0	0	0.027701584	0	0
GLYCTK	0.499498033	0.724958206	0.557512458	0.927072072	0.853470359	0.932665967	0.513659786	1.048615281	0.474396007	0.61742974	0.645649357	0.444210674
GLYR1	37.65579797	32.59977662	40.41100164	40.15072911	33.98303337	33.43293343	39.58913999	40.44186819	42.27231441	34.47080511	41.45760729	45.00113976
GM2A	18.70939474	27.499002	15.26458014	19.48433412	9.775589355	15.60471486	13.9612578	17.79355057	18.77355504	24.99290978	18.17390004	26.28457799
GMCL1	10.04066398	11.305343	9.908241503	13.19373955	15.68123643	14.66171451	9.906226978	12.07259018	19.56869917	16.57593624	18.09224626	22.63347736
GMCL2	0.018365038	0.019038949	0	0.018561491	0	0	0	0	0.019985753	0	0	0.038224504
GMDS	2.585321132	2.632329861	2.508311245	2.991443497	2.308532908	2.728781462	2.632244878	3.021645648	1.22252224	1.439954226	1.232169677	1.275856704
GMEB1	7.986065839	6.413936886	7.714177474	8.646703708	9.450730715	7.805469071	5.871455889	7.077875728	9.509972829	7.408259898	8.898930961	7.565869648
GMEB2	4.973705435	4.517603241	5.276312366	5.084557844	6.170184798	5.728718304	4.446052285	5.447239564	5.983693005	4.703337875	5.664690563	4.950506851
GMFB	13.73611335	12.54973695	13.91718874	14.63455446	14.28847315	12.48551452	14.89019063	12.9685758	32.65066454	29.19464922	32.71147785	30.31407057
GMFG	265.1284433	235.6646355	256.1578813	187.5834419	226.6229298	232.5299721	253.7524694	209.2534435	221.3484345	174.2375	202.8219858	177.0890822
GMIP	30.36894709	30.91427753	27.95035862	20.93236707	24.66208531	24.34690179	27.2785443	23.22709824	30.98243598	27.23278816	26.81547801	32.34565566
GML	0	0	0	0	0	0	0	0	0	0	0	0
GMNC	0	0	0	0	0	0	0	0	0	0	0	0
GMNN	12.70039325	13.73947192	15.40146881	21.90477646	20.95260216	21.33323006	16.62963239	21.6976353	6.826667331	6.258466844	7.200211635	6.742342335
GMPPA	16.42183819	17.62074608	19.08572628	17.2951231	14.05420725	14.70855605	16.76912891	18.62973733	13.08249677	13.78135307	15.70635155	12.81001
GMPPB	5.578597838	5.882448852	5.971944485	7.668051023	6.29105743	6.907557321	5.310602604	6.550423547	7.944218523	8.214471547	9.168113079	9.454790384
GMPR	0.129818	0.201872576	0.068700581	0.06560334	0	0	0.100123947	0.22229825	0.35318611	0.355876805	0.279670464	0.270199753
GMPR2	24.62497895	26.15993635	23.38600857	18.18099658	23.10408986	22.12984121	23.10716964	20.35220561	19.2022143	20.66694133	19.05705463	18.67171641
GMPS	14.05863382	14.42626119	15.5840289	19.26167573	19.35438488	19.12469071	16.81667339	18.62991293	20.85469705	17.45449484	20.04614965	22.19508008
GNA11	3.323340791	4.58923655	4.149515067	5.641887213	5.639012842	5.692693802	3.764660396	5.678132439	7.120231981	7.202946543	7.676954236	10.09196078
GNA12	14.03469114	14.837405	12.90328256	12.57199974	10.5253222	11.82771281	13.34200309	12.54980461	12.98657976	14.40058869	12.76887043	15.8641198
GNA13	16.76059499	19.12836926	15.95018726	19.41799069	19.04481803	20.35436912	15.79822054	16.53639982	22.24608433	22.856972	21.11281861	18.87449283
GNA14	0.019674428	0.020396388	0	0	0.061630858	0.020204921	0	0.019251528	1.73426648	1.013969139	1.3139399	1.576568633
GNA15	58.92311111	91.21457737	68.24899555	42.86328894	63.36322932	84.72146154	53.62252755	46.57052815	21.82533121	32.40867989	29.51145156	13.08431115
GNAI1	0.014799109	0.015342168	0.062654325	0	0.015452915	0.030396292	0	0.028962007	1.884300351	3.862222523	2.215807703	2.048364561
GNAI2	96.18070915	109.3490755	98.97988685	123.4029699	88.62260312	95.09900355	96.77352925	122.7502371	117.4543844	111.7374061	119.4613066	124.8879177
GNAI3	22.58798981	24.28720458	24.06354074	27.0263214	24.7231391	24.36213765	24.22007348	25.38841389	45.58234979	42.63293082	44.60472662	40.74471851
GNAL	0	0	0	0	0.006173017	0	0.006079452	0	0.083636271	0.350058912	0.191040553	0.135352205
GNAO1	0.006486987	0	0	0.019669139	0.006773574	0	0.006670906	0.00634755	0.042356786	0.092472196	0.027950189	0.067509221
GNAQ	20.95573213	18.52811143	19.52818817	18.0973381	20.37668234	17.01182289	18.61164164	15.04486507	52.37599324	35.57535619	42.32052763	37.53896909
GNAS	75.60360789	75.9875959	76.91112497	83.44798522	78.77499579	73.58509207	78.23039564	85.91308187	63.59260504	58.19918365	65.00917609	68.27158327
GNAT1	0	0.015499895	0	0	0	0	0	0	0	0.016394658	0.016104947	0
GNAT2	0.030027657	0	0.031781686	0.030348866	0.094062721	0.061674619	0	0.029382214	0.032677599	0.032926548	0.194068204	0.062498771
GNAT3	0	0	0	0	0	0	0	0	0	0	0	0
GNAZ	0.002770404	0.002872065	0	0.00280004	0	0.002845104	0.005697901	0.002710855	0.274355218	0.370619043	0.352133091	0.314260412
GNB1	105.047228	103.4257414	109.8142208	109.3151228	102.6767914	106.424621	109.3558484	108.8170565	119.5801844	120.8795969	128.6139349	111.1804125
GNB1L	2.656824927	2.607421093	2.137135309	2.97167149	2.367317388	2.764841903	3.315010057	2.807694005	0.462606376	0.738040232	0.992102893	1.179700305
GNB2	41.19794503	46.263274	49.79945721	52.62026529	45.61800778	45.36405433	47.91945728	59.01304767	85.02208477	80.74096177	94.29226671	89.31555139
GNB3	0	0	0.026902589	0.05137947	0.053081529	0.026103193	0.078415448	0.024871482	0.221287698	0.44594709	0.191654193	0.079356021
GNB4	16.30375534	13.74786303	15.8820367	17.2268	18.63745052	17.78215467	15.84866043	14.25432296	19.79678199	17.47598476	19.74953254	16.03047222
GNB5	3.166883493	3.235649855	3.342185669	2.682717961	2.456201267	2.866888903	2.927238852	2.543533193	5.607807234	6.583920662	6.0337749	5.943751127
GNE	1.728821232	1.69042793	1.694651945	1.439549005	1.353898762	1.38201585	1.666721924	1.018840891	9.171775203	13.00078128	11.37437595	8.331344022
GNG10	0.085752288	0.044449499	0.090761404	0.043334796	0	0.044032238	0.044091767	0.041954525	0.046659966	0	0	0.178482546
GNG11	17.88011614	15.72948505	17.61118499	9.691277423	10.01232261	8.283276284	18.271046	12.03180123	0	0	0	0
GNG12	0.045000678	0.058314992	0.095258677	0.011370514	0.023494375	0.023107028	0	0	0.771308843	1.073255398	0.727096429	1.346408816
GNG13	0	0	0	0	0	0	0	0	0	0	0	0
GNG14	0	0	0	0	0	0	0	0	0	0	0	0
GNG2	1.562352087	2.066492294	1.60799803	1.404823138	1.48511362	2.13561644	1.473683886	0.906718429	0.949783505	1.098799923	0.812438778	0.706433846
GNG3	0	0	0	0	0	0	0	0	0	0	0.055518873	0.160916376
GNG4	3.514498994	5.104967594	3.488620552	2.849702561	4.768621286	5.199784847	4.910740913	3.225234724	1.825893993	2.721603983	2.566569904	1.270823719
GNG5	82.31661109	74.26347715	89.52751106	92.50876693	84.62875129	82.54788104	93.36951404	102.5621557	80.35413438	93.04535955	88.95611343	92.89147363
GNG7	3.246192391	5.216860002	2.809960055	2.902819046	2.683962348	4.585415938	2.990754756	3.636936964	4.005442567	4.896079504	3.561674546	4.52110419
GNG8	0.02838057	0.014711003	0	0	0.014817194	0	0	0	0.015442578	0.093361352	0.045855778	0
GNGT1	0	0	0	0	0	0	0	0	0	0	0	0
GNGT2	0.135654355	0.14063224	0.2392974	0	0	0	0	0	0.049208619	0	0	0
GNL1	11.36055204	13.04685526	12.09616594	12.38276721	12.45201745	13.72778775	12.86490186	13.76564548	7.1883777	6.832870658	7.071181293	7.744980446
GNL2	29.98094013	27.61265851	31.71256769	32.51838578	34.70189843	34.54971676	33.69806597	33.90157458	37.72405852	40.21265305	41.94465256	39.71182705
GNL3	99.278891	89.40564416	100.983503	99.47763332	124.4923185	123.574975	115.5011837	108.6488997	57.84746589	69.88519968	67.86552417	53.72443376
GNL3L	10.78048855	9.083375397	11.17427988	8.974491573	9.491624901	9.691247279	9.6088341	8.306923444	7.533732036	6.674487915	8.33741422	7.372006936
GNLY	0	0	0.052250258	0.1995786	0	0.202790674	0.253831045	0.241527194	0.053723172	0.108264907	0.10635175	0.205500546
GNMT	0.100017771	0.103687959	0.264650471	0.050543837	0.261091072	0.102714607	0.051426736	0.048933948	0.054422172	0.054836779	0	0.104087173
GNPAT	29.70412175	29.96784283	31.02774357	39.76718976	37.51199562	36.43437026	32.68533218	36.21791023	49.05946464	51.94857669	49.12505664	46.632031
GNPDA1	5.44758118	7.130454012	4.8324829	9.447095929	5.094869941	7.365377944	5.340065363	10.39252992	8.871170771	14.58993816	10.76491947	15.78410331
GNPDA2	7.025718712	6.921765208	7.140642706	7.194925029	7.190355553	6.450637187	8.277547811	5.941381017	5.56974343	6.887670157	6.440189295	6.149468355
GNPNAT1	8.106761761	6.515215319	8.882062415	10.04766303	10.56172027	8.961843545	9.483843562	9.59421016	7.783486368	8.549585686	8.638844107	7.585199843
GNPTAB	18.55212629	15.96940145	15.68200608	17.57973001	12.66613479	14.03494954	14.82222828	14.07911375	6.449630185	4.712985907	4.74725342	8.972054463
GNPTG	11.92413831	14.20030587	13.0200608	15.25530676	10.59905248	11.1993496	11.95177539	14.99094593	18.80763095	17.66821027	19.91671444	30.5519939
GNRH1	0.900997298	1.297305156	1.218524623	1.315362217	1.724804051	1.182316788	1.106703358	1.37142511	1.498001925	1.811297073	1.347946597	1.250207974
GNRH2	0.08457963	0.280586591	0.161136445	0.205162535	0.211959001	0.086860199	0.104373155	0.099313917	0.073635029	0.092745008	0.182212209	0.158437626
GNRHR	0	0	0	0	0	0	0	0	0	0	0	0
GNS	35.58272638	40.55444729	30.26449474	39.50560707	19.72235521	22.43609102	24.19294976	31.50140096	35.18291537	33.39132105	26.31334753	54.79767441
GOLGA1	5.003185218	4.850793191	4.330683916	4.26851408	4.208986609	5.09648498	4.52013229	3.96408269	9.423547862	11.57209826	6.643831645	12.15259371
GOLGA2	13.7517437	13.2965594	12.07257856	13.47464604	11.70397182	11.56172017	12.22476868	13.5407049	19.08953505	24.47348218	17.67173759	19.71973152
GOLGA3	17.35585341	15.94635981	16.4110087	16.32452832	14.28373127	14.52462163	15.26994866	13.95032189	10.04254775	10.5736012	10.50369832	11.50894463
GOLGA4	6.453443085	6.330240765	6.107553292	6.709668536	6.448458012	6.698777757	6.654266719	5.131069491	8.818066031	9.602411357	7.674610864	9.275928171
GOLGA5	11.23675572	11.38604842	11.24093292	12.23620499	10.42739173	11.01858925	13.25136556	11.93513667	19.74294891	21.76146511	17.60911307	21.08682011
GOLGA6A	0	0	0	0.017123794	0	0	0	0	0.092188692	0.018578204	0.091249533	0.03526379
GOLGA6B	0	0	0	0	0.017679924	0	0	0	0.055278405	0.037133024	0.054715265	0.052862397
GOLGA6C	0	0	0	0	0	0	0	0	0	0.021328167	0.020951275	0.020241785
GOLGA6D	0	0	0	0	0	0	0	0	0	0	0	0
GOLGA6L1	0	0	0	0	0	0	0	0	0	0	0	0
GOLGA6L10	0.096692832	0.125301262	0.07675577	0.183238439	0.075723448	0.086887513	0.111863547	0.10644123	0.105225979	0.066267267	0.117173255	0.201253904
GOLGA6L2	0	0	0	0	0	0	0	0	0	0	0	0
GOLGA6L22	0	0	0	0	0	0	0	0	0	0	0	0
GOLGA6L3	0.032182752	0.166818546	0.034062668	0.032527015	0	0.033050513	0	0.031490986	0.105068643	0.035289698	0.034666091	0.033492164
GOLGA6L4	0.099565388	0.103218975	0.046836169	0.044724645	0.011551562	0.045444456	0.045505895	0.075775185	0.036117346	0.048523334	0.071498812	0.069077589
GOLGA6L6	0	0	0	0	0	0	0	0	0	0	0	0
GOLGA6L7	0	0	0	0	0.028192072	0	0.027764761	0	0.088145896	0.029605808	0.029082641	0.056195583
GOLGA6L9	0.390077146	0.284166764	0.424021491	0.575391674	0.330251559	0.313979878	0.303562833	0.422956517	0.172095191	0.312131291	0.238478796	0.164573566
GOLGA7	27.68366158	26.85389481	26.70986518	28.677043	23.79457477	25.57338839	27.34719835	26.63131983	35.30932126	29.81941499	30.44307843	41.06122731
GOLGA7B	1.059834713	1.124679873	1.024584775	0.565106436	0.731962017	0.771315334	0.789521626	0.612433254	0.145305849	0.100658827	0.215738351	0.173693855
GOLGA8A	16.59254004	15.75591258	16.04909946	11.67325795	19.1090926	17.350185	12.94061473	11.64253911	12.05053314	15.79905511	11.13926069	13.66416663
GOLGA8B	17.28847043	14.58315872	11.47048402	7.656795161	12.54933573	11.75666669	9.244558503	7.908158539	7.431250896	7.144303911	6.507494946	8.628077575
GOLGA8F	0	0	0.010106971	0	0.009971038	0	0	0	0	0	0	0
GOLGA8G	0	0	0	0	0.010426201	0	0	0	0.010866257	0.01094904	0	0
GOLGA8H	3.702539637	3.064362511	3.2368146	2.543005413	5.564212524	3.823380901	3.796995933	2.962415279	6.144109085	4.261863192	4.252654935	4.066067165
GOLGA8J	0.446849036	0.648544846	0.31214778	0.415498714	0.718548994	0.477253587	0.340043383	0.227366905	1.186531699	0.480188398	1.21295045	0.930059771
GOLGA8K	0.601571806	0.225803114	0.318355948	0.345934958	0.509016873	0.298244572	0.351977745	0.294320532	1.252884837	0.796126871	1.17308768	0.863514246
GOLGA8M	5.393662521	3.756491218	4.387047562	3.206160904	7.530614001	4.954137683	4.02363854	2.641006746	12.25427251	8.374287476	7.353174359	10.82040444
GOLGA8N	4.513581104	3.778958141	4.007748731	3.959738303	7.570298633	4.583434544	4.433874401	2.786287444	20.16408364	13.51929838	14.02001051	16.2879139
GOLGA8O	2.52677821	1.508505392	2.2373192	1.540234082	3.295443516	2.170838405	2.952287415	1.64510337	4.226294818	2.565876319	2.80647752	3.356045828
GOLGA8Q	5.66714701	4.112573479	5.428358829	3.207550755	9.379260224	4.044867689	4.807952975	2.966752675	8.418327635	4.567479215	7.600033813	5.042554872
GOLGA8R	3.30847648	2.577804541	3.31453735	2.134607352	4.606187668	2.820719463	3.231094489	1.974993831	4.562834029	2.715205161	3.496529687	3.226541348
GOLGA8S	0.193261635	0.124028319	0.17532924	0.10231518	0.240237723	0.189021579	0.189277126	0.12607166	1.071615103	0.403655716	0.495653358	1.139707365
GOLGA8T	0.589351314	0.462861946	0.652131084	0.442229217	0.634036227	0.788649093	0.587695112	0.655321876	1.681144785	1.263120526	1.03880916	1.41251786
GOLGB1	6.637755104	6.664439818	5.33051622	6.185899638	5.687791294	5.176029097	5.296466655	4.339976985	15.24601023	12.84698664	11.620203	13.84335849
GOLIM4	12.7068081	10.70689418	13.08059351	14.00385838	13.2318275	12.79916595	13.46087327	11.44579507	16.85901945	14.12440274	14.31230336	14.20200669
GOLM1	0.412018077	0.341709781	0.697736984	0.732908857	0.344176408	0.270801635	0.389803633	0.677311789	45.03515574	41.72774896	46.72441165	48.36658338
GOLPH3	47.85088549	44.82484652	47.06047696	45.90181599	50.37546027	41.26673367	50.31176731	43.87744027	77.41934829	60.16522488	70.01669543	70.35274812
GOLPH3L	8.640205022	9.341521567	8.712016902	10.25696529	9.355569083	9.188199762	9.226909228	9.279922284	18.84713499	14.98234589	14.93787375	16.99919067
GOLT1A	0	0	0	0	0	0	0	0	0	0	0	0.126551184
GOLT1B	9.89204066	8.969084055	9.854974996	11.14046932	11.42754556	11.81964287	11.33507086	10.34007025	24.07603858	31.07758847	24.89630224	23.98785629
GON4L	16.72500947	15.07161159	14.92443233	16.08829716	16.26104487	15.42990938	16.06525309	14.32207873	23.02107095	19.79196529	19.88016762	22.50213336
GON7	14.3675188	12.57040157	16.22379399	11.56147292	16.10114893	14.37899687	15.33951082	12.8050489	9.062587403	6.121202101	9.315273802	11.71405537
GOPC	29.79887458	28.17667371	27.83470328	28.34873159	32.47702072	31.67138527	29.65560336	24.45719566	38.71517421	38.13207866	32.33237891	49.05855912
GORAB	4.661997444	4.934973188	5.499094228	5.336331783	5.410471998	4.686756011	5.443987028	4.781633366	4.553851932	4.265190943	4.204946142	4.515568826
GORASP1	8.836982223	8.568405298	8.462404441	10.69818536	9.418921928	9.163906003	9.675609864	10.96979999	12.44671195	9.96223846	9.739704766	17.51720116
GORASP2	24.87065715	21.39501209	26.56750496	27.45061005	24.00880652	24.69552173	25.72822773	27.1403524	23.64197768	21.60062189	23.18822978	25.12837028
GOSR1	9.302035391	9.022475335	9.152064335	10.42303579	8.980116164	8.226600441	10.09505932	8.882320083	13.03557722	11.2364852	11.83426784	13.73190389
GOSR2	5.9820877	4.792606508	5.888111194	5.755591354	5.122225803	5.13783188	5.525501554	5.17663228	5.375527129	4.684667418	5.221066009	5.865537385
GOT1	22.51551912	24.31212074	26.96354056	35.9499516	25.72916872	27.35743435	30.3341823	36.68624343	14.70150883	15.75669235	15.94151229	15.48065469
GOT1L1	0	0	0	0	0	0	0	0	0	0	0	0
GOT2	22.4680051	21.32122604	24.93708296	26.50779948	22.73032146	23.90430072	25.75714963	27.16052761	30.04024316	36.28457464	35.05482402	33.59479025
GP1BA	0	0.022110235	0.022573437	0.021555754	0	0	0.02193229	0	0.046419546	0	0.022973327	0
GP1BB	3.145444059	5.648287896	4.161476713	6.301413063	6.627461634	7.498809618	3.119101321	6.430472457	3.239653053	2.155692173	2.722626999	3.857961715
GP2	0	0	0	0	0	0	0	0	0	0	0	0
GP5	0	0.015970261	0	0	0	0	0.031683463	0	0.01676446	0	0	0.016031749
GP6	0.142353336	0.049192347	0.100445822	0.239793512	0.32205837	0.170556971	0.121991111	0.232155769	0.103277349	0.104064152	0.07666892	0.074072618
GP9	0.338159063	0.350567929	0.313173189	0.128166148	0.308961187	0.434096297	0.391214855	0.330890359	0.460002019	0.139051943	0.546378982	0.439897084
GPA33	0.750220437	2.255475119	0.397021829	0.492859667	0.176256949	0.770449056	0.385745332	0.440456703	4.143369182	1.789257779	2.969804963	2.849717403
GPAA1	78.51457937	88.54751006	87.50945013	105.1393971	80.35228677	85.31017092	77.01948621	118.3226183	81.90223569	73.10604872	79.30125687	94.38808106
GPALPP1	11.90929791	11.46604646	11.32603359	12.57581382	13.67443228	13.41544614	12.35889649	11.54678825	7.439730009	8.069382371	6.836268103	8.088855709
GPAM	2.809419549	2.685658227	3.01088428	5.022584113	4.164441982	4.465491601	3.796444025	4.164989608	2.942124158	2.500119758	2.61561407	2.79149699

GPANK1	6.078003014	6.779597289	6.058460007	7.107241078	7.744362865	7.47446294	6.297797202	8.341356103	10.33184004	9.870620244	10.4254823	10.56885137
GPAT2	0	0	0.017416753	0	0	0.016899223	0	0	0.017907724	0	0	0
GPAT3	1.033887209	1.455323464	1.234830266	1.783120282	0.594254877	0.642903273	1.082708203	1.188008959	0.753525959	0.946483001	0.817369345	2.191390013
GPAT4	18.15947268	19.15520649	17.92826832	19.35929479	19.13633664	18.25415396	17.16972192	19.39715554	26.4676771	24.34984746	29.02588112	29.94844776
GPATCH1	7.458434075	7.715531407	8.046570827	10.40144658	10.11094978	9.500459563	8.34471449	10.21109734	6.322619665	5.405516746	5.378956437	6.112907755
GPATCH11	11.49730036	11.40715594	12.51741394	16.14083025	13.00806047	13.24447503	13.44579678	12.90144419	15.57273384	15.34534978	14.2909147	19.57534174
GPATCH2	2.861542854	2.315751503	2.66879648	2.987324544	2.490878882	2.406833117	2.689829277	2.549208468	3.444268474	3.67701769	2.907664698	3.604051238
GPATCH2L	6.76511677	5.802366682	5.473591532	4.659966285	4.73961889	4.90775952	5.371825053	3.345806609	7.633641173	8.120179326	6.118446021	7.686439085
GPATCH3	6.293368086	5.921658762	6.500481849	7.152582061	6.703356906	5.840114221	5.796027448	7.666701635	6.876263159	5.73692132	6.452289228	7.075483226
GPATCH4	36.60548317	27.85323199	39.72159179	39.87404473	41.84678042	38.31049889	40.98140507	41.92687935	35.59689872	30.03610147	44.2983415	32.35203204
GPATCH8	5.153725096	4.927324767	4.916979125	6.271040555	6.317307506	5.808020044	5.101870024	5.425876543	7.717325726	6.990547577	6.758232661	8.181422698
GPBAR1	0.066637227	0.069082504	0.03526488	0.084187567	0.069581175	0.034217002	0.068526524	0.13040973	0.163165422	0.073070433	0.287116798	0.277393926
GPBP1	21.4498978	21.05258381	22.00340593	25.91333283	26.88872815	25.08806414	26.19320811	23.86041319	33.04567754	34.69761814	28.69128462	31.01854076
GPBP1L1	37.19031017	34.92119691	35.30900551	36.20662507	34.45256918	33.52319569	36.83908183	34.11316483	59.33883745	57.29775889	50.62902334	54.36512815
GPC1	0.28434846	0.350931817	0.214970236	0.369501649	0.466573819	0.264204507	0.222788799	0.450477998	2.755509328	2.835892174	3.092068741	4.030117028
GPC2	0.311518106	0.47365908	0.483582087	0.419800607	0.260224463	0.469212693	0.363063626	0.223536168	1.536843489	2.004008083	1.208001543	2.528703653
GPC3	0.125527747	0.238579059	0.088573532	0.211450889	0.742749282	1.589919881	0.344231215	0.634619276	5.578061034	9.176419028	7.23394513	3.744871928
GPC4	0	0	0	0	0	0	0	0	6.669397614	11.10502361	7.7251059	11.03693967
GPC5	0	0.01915828	0	0.009338915	0	0.018978435	0.009502047	0.004520729	0.256415481	0.26850106	0.209014472	0.403872886
GPC6	0.003097666	0	0	0	0	0	0	0.009093247	1.621468059	1.773086374	1.38139113	1.766587794
GPCPD1	8.728240169	9.708400301	7.958062743	8.226530752	9.5209332	9.208714595	7.822034673	7.25602975	6.416038516	10.51312599	6.882041703	6.508226303
GPD1	0	0.018094104	0	0.01764034	0.018224716	0	0	0	0.094969603	0	0.075201691	0.072655075
GPD1L	7.272636365	7.470842492	7.823646472	8.461700284	8.76967758	9.822267623	8.4324148	8.710676473	8.404594748	10.21173687	8.846936551	7.692610919
GPD2	4.973246735	6.277283012	5.810636352	6.201459585	5.867368619	6.060824488	4.873479902	5.06384502	14.32107412	15.23578819	15.07960902	13.4683023
GPER1	0	0	0.036496496	0	0	0	0.017729948	0.016870531	0.506591778	0.283583984	0.371430334	0.107655685
GPHA2	0	0	0	0	0	0	0	0	0	0	0.030748914	0
GPHB5	0	0	0	0	0	0	0	0.096966719	0	0	0	0
GPHN	4.467024398	4.509343171	4.562429892	4.445654109	4.245905219	4.045406867	4.161445868	4.141456488	3.340107669	3.57992026	3.453485704	3.611191478
GPI	28.76553915	30.48334593	32.37175806	36.32688616	29.86455436	34.51107332	33.03985786	40.31247224	21.12394721	35.84625854	32.38745075	14.16718252
GPIHBP1	0.066105112	0.022843621	0.023322187	0	0.023008517	0.02262918	0.045319548	0	0.04795926	0	0.023735341	0
GPKOW	8.218502061	8.400502785	8.484926689	9.355647974	10.4183453	9.446991577	9.667344442	10.94819731	14.52972831	13.43883124	14.50595692	13.92469939
GPLD1	0.067574483	0.007005415	0.035760882	0.040978402	0.014111968	0.041637919	0.027796141	0.046285382	0.220613803	0.163015978	0.218366333	0.175809679
GPM6A	0.377089724	0.284310665	0.38702251	0	0	0	0	0	0	0.012530128	0	0
GPM6B	0.020255811	0.031498657	0.085756117	0.010236245	0.03172603	0	0.010415051	0	0.892757166	1.365996244	1.11275998	0.389979156
GPN1	21.94468125	22.49496832	21.43322039	26.68160125	26.93770336	25.09032502	25.23665859	27.40947259	27.24193168	28.46833704	27.75921105	26.64853642
GPN2	16.17850373	13.77978635	15.91660511	16.45961725	13.91646536	14.60193574	16.8204252	14.64518536	13.80578776	13.44205614	15.12378228	16.50298674
GPN3	32.9446291	32.4458667	36.64478842	36.96588274	40.83416998	40.72482774	40.43482505	37.60923452	20.34932629	28.43181304	25.72789907	20.15833377
GPNMB	0.319735245	0.857917286	0.278692394	0.247118907	0.157110936	0.173835766	0.116047189	0.01840368	1.555549646	2.660455886	4.153145491	0.332744243
GPR1	0	0	0	0.020105423	0	0	0	0.019465038	0	0	0	0
GPR101	0	0	0	0	0	0	0	0	0	0	0	0
GPR107	8.651075544	8.286359763	7.654246234	8.313282576	8.305757429	7.724936421	7.921135409	7.417239034	16.99670787	15.85287171	14.67631197	17.91884199
GPR108	36.82458011	37.58399711	34.93995943	34.99280647	31.80866887	31.59834482	32.58463467	36.15274957	20.8138102	17.41735795	17.10957452	21.68657621
GPR119	0	0	0	0	0	0	0	0	0	0	0	0
GPR12	0	0.01149951	0	0	0	0	0	0	0	0	0	0
GPR132	8.676270278	7.192836363	8.049915083	5.944425405	5.415420126	5.083391021	7.69258923	5.387741503	2.224305888	1.524660836	2.201646108	1.504879969
GPR135	2.451618549	1.940487719	1.961128801	1.465049049	1.783394522	2.330026363	2.177631343	1.048371728	0.953337753	0.919135838	1.011512514	0.787054095
GPR137	3.656430606	3.451147488	3.581209372	4.453957773	2.407598623	2.3398825	2.595589771	4.939549564	3.786094207	3.306279597	3.527081359	5.352835918
GPR137B	2.758555505	2.639232959	2.626973307	2.902740285	2.05850422	2.031848701	2.092935316	2.345372927	2.778198806	2.356131448	2.314496074	3.018390831
GPR137C	0.525651611	0.389243273	0.45416891	0.650540294	0.392053021	0.462707189	0.364047157	0.524849764	1.564355352	1.02340123	1.155536357	1.696936328
GPR139	0	0	0	0	0	0	0	0	0	0	0	0
GPR141	0.138505948	0.358971182	0.112390731	0.293973884	0.014462496	0.075861633	0.06646867	0.126493521	0.165802015	0.081001286	0.099462383	0.235430806
GPR142	0	0	0.038300458	0	0	0.03716238	0	0	0.078760265	0.079360288	0.077957907	0.037658977
GPR143	0.02859169	0.059281745	0	0	0.029854835	0.029362624	0.058804642	0	0	0.031351952	0	0
GPR146	0.076898265	0.159440148	0	0.038860428	0.100369415	0.177686362	0.039539241	0.037622671	1.966586697	1.412394813	1.408144283	1.0003376
GPR148	0	0	0	0	0	0	0	0	0	0	0	0
GPR149	0	0	0	0	0	0	0	0	0	0	0	0
GPR15	0	0	0	0	0	0	0.039795158	0	0	0	0	0
GPR150	0.442984278	0.567296153	0.44128063	0.184356504	0.516972966	0.160563076	0.482340446	0.280475647	0.397005133	0.514323844	0.196480351	0.461007884
GPR151	0	0	0	0	0	0	0	0	0	0	0	0
GPR152	0	0	0	0	0	0	0	0	0	0	0	0
GPR153	0.479275441	0.598493613	0.772436508	0.440365738	0.500449219	0.38033514	0.324842079	0.277120675	1.066850837	0.656931293	0.915184795	1.30361809
GPR155	0.129661592	0.162718427	0.158904399	0.082767536	0.106886744	0.091107922	0.14035553	0.113519313	0.386180009	0.70341296	0.404298504	0.504238658
GPR156	0.01022801	0.021206661	0	0.041349682	0.021359741	0.010503794	0	0	0.100175692	0.044861718	0.132206889	0.127729859
GPR157	0.345565987	0.486191886	0.418002065	0.0997893	0.103095043	0.253488342	0.228447941	0.169069036	1.128186604	1.028516617	1.276220998	1.387128687
GPR158	0.007774825	0.008060125	0.032915926	0	0	0	0	0	0.02538286	0.008525412	0.033499034	0.048546943
GPR160	9.675638777	13.01460923	9.517947434	12.9511976	7.776837042	9.134926212	8.311144888	10.69288844	22.75790831	31.7222067	25.73156321	20.1386188
GPR161	0.072392041	0.05772961	0.094302442	0.028140934	0.05814633	0.080062757	0.057264998	0.021795686	0.28482232	0.537347091	0.395888693	0.376687201
GPR162	1.753729726	2.283690197	1.697718044	1.642795236	1.406903145	1.735125686	1.561525008	1.988087516	3.933364644	2.884554072	2.879655388	4.162080319
GPR17	0	0	0	0	0	0	0	0	0	0	0	0
GPR171	0	0	0	0	0	0	0.021514451	0	0	0	0	0.021772511
GPR173	0.036439883	0.037777058	0	0	0	0	0	0	0	0	0	0
GPR174	0	0.044343499	0	0	0	0	0.04398662	0	0	0	0	0
GPR176	0.291456998	0.290750149	0.279380021	0.244552623	0.321559604	0.384027714	0.3845469	0.360525927	0.1555968	0.186932612	0.159935207	0.103012793
GPR179	0.006743053	0	0	0.006815184	0	0.006924869	0	0.006598111	0.080719402	0.073940319	0.072633714	0.175435147
GPR18	1.273001252	0.691279015	0.791307889	0.306337788	0.31648591	0.145258434	0.851949637	0.336124589	2.110999879	0.930598469	2.350681185	1.387881083
GPR180	3.06790285	2.859918352	2.990422094	3.695127512	4.279693056	3.960073425	3.260878062	3.304527445	4.196400168	4.846669172	4.571627331	4.315858795
GPR182	0	0	0	0	0	0	0	0	0	0	0	0
GPR183	27.43085156	21.13979286	28.92713427	5.291666777	5.310018679	4.502132332	17.15699339	3.529805251	0.245355826	0.082408344	0.377776467	0.026070251
GPR19	0.077501888	0.056242093	0.016405813	0.015666187	0.040462911	0.015918323	0.023909766	0.022750797	0.134946293	0.305942313	0.233750209	0.137113824
GPR20	0.809960667	0.803174527	0.559091439	0.213554327	0.367714651	0.433982659	1.050209338	0.37904595	0	0	0	0
GPR21	0.006192837	0.012840171	0.032772921	0.006259083	0	0.006359818	0.012736832	0.006059722	0.006739355	0.006790698	0.006670699	0
GPR22	0	0	0	0	0	0	0	0	0	0	0	0
GPR25	0	0	0	0	0	0	0	0	0	0	0	0
GPR26	0	0	0	0	0	0	0	0	0	0	0	0
GPR27	2.166963874	4.283011226	2.486459402	6.181528384	4.292781378	5.241112367	2.374184847	6.519688904	0.044078477	0.111035706	0.239961889	0.126455925
GPR3	0.763256182	1.081394321	0.861696928	1.517127635	1.062634489	1.201882314	0.654079999	1.26964508	0.304558311	1.115921973	0.98658219	0.847264663
GPR31	0	0	0	0	0	0	0	0	0	0	0	0
GPR32	0	0	0	0	0	0	0	0	0	0	0	0
GPR33	0	0	0	0	0	0	0	0	0	0	0	0
GPR34	0.035841182	0	0.075869602	0	0.024949733	0	0.03685735	0	0	0.013100438	0	0
GPR35	2.362977634	3.631554918	2.215805436	1.822615435	1.298616301	1.36235337	2.195501653	1.967385997	1.12785549	0.727326648	0.736801318	0.927562654
GPR37	0.019169776	0.019873218	0.010144778	0.009687419	0.010008336	0	0.009856638	0	0.229476604	0.189183958	0.216814353	0.189522458
GPR37L1	0	0	0	0	0	0	0	0	0	0	0	0
GPR39	0	0	0	0	0	0	0	0	0	0	0	0
GPR4	0	0	0	0.012505212	0	0	0	0	0	0	0	0
GPR42	0	0.049410205	0	0	0	0	0	0	0	0	0	0
GPR45	0	0	0	0	0	0	0	0	0	0	0	0
GPR50	0.01577993	0.03271796	0.016701695	0.03189746	0.016477067	0	0.016227322	0	0.343450188	0.692133423	0.492929445	0.328439289
GPR52	0	0.037896822	0.077381496	0	0.03817038	0	0.037591826	0	0	0.12025348	0	0.03804273
GPR55	0.345449781	0.584939431	0.475317456	0.360783271	0.348687601	0.473019119	0.485500082	0.416896715	2.26814563	1.149025907	1.351984956	2.025211621
GPR6	0	0	0	0	0	0	0	0	0	0	0	0
GPR61	0.019061127	0	0	0	0.039806446	0	0	0	0	0	0.082127812	0
GPR62	0	0.025460576	0	0.049644152	0.025644363	0.050443139	0.050511335	0	0.026726726	0.107721359	0.026454452	0.17891022
GPR63	1.916661657	1.69042793	1.786397712	1.53961203	2.023740355	2.027098161	1.86068547	1.62353527	3.533419552	4.493554825	3.497423373	3.646924565
GPR65	3.093871257	2.701619339	2.884163405	1.599563782	3.715137725	2.725139819	3.1815886	1.420534555	0.103597093	0.182676082	0.358895998	0.148603889
GPR68	1.561593935	1.157647143	1.181899482	0.299788543	0.054656419	0.035836873	0.843305088	0.247557542	0.455706281	0.401780759	0.253723411	0.37223652
GPR75	2.417567116	1.44692488	1.741029906	1.435828907	1.951834135	1.638105303	1.81972993	1.780298179	1.139160165	0.65590782	0.993322388	0.648435605
GPR75-ASB3	0	0	0	0	0	0	0	0	0	0	0	0
GPR78	0	0	0	0	0	0	0	0	0	0	0	0
GPR82	0.224942924	0.049970847	0.119041346	0.016239226	0	0	0.066091571	0	0.034970592	0.017618505	0.051921502	0.066884322
GPR83	0.037664182	0.039046282	0.026576193	0.088823187	0.039328137	0.05157299	0.025821357	0.036854593	0	0.01376677	0.027046992	0.052262155
GPR84	15.37416025	13.35641187	18.80683744	5.978253958	8.001680268	7.181154384	14.50485709	6.022164032	0.625455349	1.286699752	1.341347837	1.171316531
GPR85	1.550707806	2.280798924	1.795161514	1.557500326	1.072730666	1.413361873	1.495006337	1.631178615	2.457506105	2.391207534	2.380271692	1.805440002
GPR87	0	0	0	0	0	0	0	0	0	0	0	0
GPR88	0.03105891	0	0	0	0	0	0	0	0.01689993	0.017028679	0	0.113129089
GPR89A	4.538155735	5.322288394	8.159955641	4.303352644	6.970749032	4.67851489	8.234507236	4.457753619	5.777646333	4.93784572	4.756219252	5.032789345
GPR89B	2.692070984	2.080757648	3.203383199	2.833567103	3.775726255	3.893424849	2.21144099	3.740883007	3.57104824	3.580786484	7.566936446	4.542243423
GPRASP1	0.259301639	0.259547259	0.179811041	0.198815837	0.364121826	0.165285519	0.202288749	0.236229489	0.398951236	0.460818477	0.41414976	0.288462257
GPRASP2	0	0	0	0	0	0	0	0	0	0.014843868	0	0.014087773
GPRC5A	0	0.019566511	0.019976423	0	0.157662009	0.019382834	0	0	0	0	0	0
GPRC5B	0.090461576	0.078150913	0.09574578	0	0	0	0	0	0.803965984	0.727428543	0.812016022	0.784518056
GPRC5C	6.184321296	7.349238501	6.093063632	1.115438602	7.412670773	3.900497999	6.717517608	1.760934964	3.419144352	1.907938934	2.581073648	1.510687219
GPRC5D	0	0	0	0	0	0	0	0.012898807	0	0	0	0
GPRC6A	0	0	0	0	0	0	0	0	0	0.006703519	0.006585061	0
GPRIN1	0.549860056	0.543523999	0.798529966	0.891772014	0.78779019	0.735405342	0.841599511	1.001006242	2.337877183	1.738722058	2.217641228	2.861160468
GPRIN2	0	0	0	0	0	0	0	0	0	0.027459221	0	0.005212109
GPRIN3	0	0	0	0.007033323	0	0.00357326	0.010734271	0	0.140100583	0.35101212	0.149917113	0.057936138
GPS1	18.66107881	18.87063858	21.92658604	21.02773625	19.87150586	20.24242684	20.98448246	24.20674611	22.04584011	21.06256727	23.23883639	26.63756359
GPS2	33.11357591	36.78762736	38.24745987	46.25324624	33.41099175	30.8063922	33.76545248	51.24217571	20.09302338	16.472267	18.63591509	26.28124639
GPSM1	0.009476851	0.019649215	0.02006086	0	0	0	0	0	4.548114013	5.663505463	5.165308568	6.548656011
GPSM2	2.265873123	2.512430234	2.127126947	5.761787666	4.299905131	4.026668347	2.843415803	5.456162108	11.79313123	14.6628715	11.52442515	10.65571626
GPSM3	50.27291944	58.3900068	50.23971583	35.62656889	47.97699481	49.31718274	48.09140065	40.15465039	89.72995284	84.03556195	91.86977256	82.76643332
GPT	0.211621441	0.159554156	0.081448382	0.116664645	0.060264711	0.098785232	0.019783757	0.11294872	0.230297042	0.042191187	0.124336869	0.220231636
GPT2	10.99521931	10.61390692	13.59458687	13.32035264	14.59094448	15.06408257	11.77914206	13.26037771	5.537104565	6.30051322	11.34905129	5.450077332
GPX1	350.916742	331.9489278	336.572749	187.1457698	217.2331915	220.1042816	311.9368221	232.4978856	260.2102191	252.6529622	268.579032	258.5897255
GPX2	0	0	0	0	0	0	0	0	0	0	0	0
GPX3	0	0	0	0	0	0	0	0	0	0.032527218	0	0
GPX4	152.952622	176.4177819	161.4812288	214.5558861	121.9449764	142.4640618	144.5892555	230.5019856	136.1072532	125.242357	133.811802	174.7421091

GPX5	0	0	0	0	0	0	0	0	0	0	0	0
GPX6	0	0	0	0	0	0	0	0	0	0	0	0
GPX7	2.6707756	2.178712646	2.687763479	0.973534344	6.720471439	6.789527456	8.464614777	4.969682413	37.97472	38.36004484	46.1241222	28.38674856
GPX8	0	0	0	0	0	0	0	0	2.440570988	2.272392154	2.96612253	2.127101407
GRAMD1A	13.43255868	16.17328344	13.37760662	11.86585497	15.50313879	17.43352059	14.42521207	14.6574368	9.222565793	12.35177566	10.56697398	10.2228948
GRAMD1B	0.991060942	1.189943738	0.913966716	1.15741693	0.865607405	1.200602497	0.915330893	1.060754234	3.695311699	3.280610582	3.096709627	3.920918356
GRAMD1C	0.057219865	0.059319568	0.060562294	0.069398344	0.047798212	0.023505087	0.047073729	0.055989928	0.410978835	0.363914687	0.184905477	0.500202839
GRAMD2A	0	0	0	0	0	0	0	0	0.011787089	0	0	0.04508768
GRAMD2B	0.400638461	0.405450982	0.484618596	0.472411498	0.398417292	0.489810836	0.559139261	0.466698532	2.782062204	2.311640973	2.579044073	2.471853538
GRAMD4	12.32849479	15.36556435	11.46951305	11.38894082	10.13658224	14.33425248	10.77027073	12.06349471	14.94399884	15.80051469	17.12841297	15.64945513
GRAP	7.207329563	6.29345588	6.425301779	2.454251487	5.637561648	4.854855457	6.428761703	2.401357749	2.473896602	0.963105486	2.504346293	1.317304869
GRAP2	0.026487601	0	0.042052263	0.040156413	0	0	0.068096441	0.025918252	0.821516282	0.334014425	0.627692582	0.220522368
GRAPL	0.230553589	0.162187959	0.174300784	0.0665771	0.206347845	0.152209373	0.177817678	0.120855995	0	0.018057957	0	0
GRASP	0.122674647	0.092491808	0.094429482	0.0901723	0.093159459	0.045811779	0.080279	0.141862812	0.084954984	0.048915544	0.060063942	0.058029947
GRB10	2.697211453	3.147205722	2.585986137	4.067707283	3.724359123	4.231959322	2.765769206	3.795076993	3.915049721	3.240283125	4.93617369	3.663853589
GRB14	0.013269929	0	0	0	0	0	0	0	0	0	0	0
GRB2	102.3423972	103.8692242	100.8222814	100.5565965	78.94041139	85.13188078	97.59020074	97.24218179	141.7875474	136.8851481	142.5359692	143.8276802
GRB7	0	0	0	0	0	0	0	0	0.021616189	0	0	0.020671428
GREB1	0.417792046	0.273364563	0.297214285	0.148829775	0.160911725	0.087921559	0.323988764	0.134036602	0.115528923	0.135184718	0.092219346	0.181756752
GREB1L	0.035611887	0.016408301	0.046068137	0.007998407	0.004131686	0.008127136	0	0	0.701889543	0.681203528	0.588184065	0.901813284
GREM1	0	0	0	0	0	0	0	0	0.007243723	0	0	0
GREM2	0	0	0	0	0	0	0	0	0	0	0	0
GRHL1	0.412755517	0.456428508	0.262119679	0.292019571	0.186762563	0.127165463	0.297120564	0.269255578	0.583935573	0.331909035	0.414964895	0.415230904
GRHL2	0	0	0	0	0	0	0	0	0.007143864	0	0.007071087	0
GRHL3	0.015149088	0.031409978	0.016034004	0	0	0	0.015578595	0	0.115401971	0.182727511	0.163180472	0.299543659
GRHPR	11.77468938	11.28128548	11.61536987	10.46945608	9.514469333	10.68537471	10.24080481	12.2445168	15.82881949	17.26586812	18.43633137	17.76395275
GRIA1	0	0	0	0	0	0	0	0	0	0	0	0
GRIA2	0	0	0	0	0	0	0	0	0.009975853	0.010051853	0	0.009539846
GRIA3	0	0	0	0	0	0	0	0	0.817709996	1.266807129	0.526096804	0.801520284
GRIA4	0.003063105	0.003175506	0.009726097	0	0	0	0	0	0.003333424	0.003358819	0	0
GRID1	0	0.009529232	0.038915464	0	0	0.009439778	0	0.017988701	0.050015594	0.080634608	0.039604854	0.086093285
GRID2	0	0	0	0	0	0	0	0	0	0	0	0
GRID2IP	0	0	0	0	0.023939838	0.011772573	0	0	0.112276164	0.188552538	0.098784327	0.178948335
GRIFIN	0	0	0.025887628	0	0	0	0	0	0	0	0	0
GRIK1	0	0	0	0	0	0	0	0	0	0	0	0
GRIK2	0.003685586	0.00382083	0	0.003725012	0.011545233	0	0	0.00721273	0.308834643	0.230359544	0.202468965	0.356705315
GRIK3	0.029184055	0.018152985	0.055599852	0	0	0	0	0	0	0	0	0
GRIK4	0	0	0.006088602	0	0	0	0	0	0	0	0	0
GRIK5	0.040086586	0.027705052	0.070713659	0.047267964	0.02092878	0.027444976	0.05496416	0.091524832	0.167226212	0.11721753	0.079162992	0.104293951
GRIN1	0	0.02024832	0	0	0	0	0	0.009555885	5.38820986	4.347690741	4.618001435	6.667019535
GRIN2A	0	0	0	0	0	0	0	0	0	0	0	0
GRIN2B	0	0	0	0	0	0	0	0	0	0	0	0
GRIN2C	0.304116204	0.229888645	0.167646242	0.038421172	0.033078299	0.045546122	0.208492332	0.055796109	0.020684655	0.013894825	0.034123221	0.039561214
GRIN2D	1.13091965	1.821436888	1.207668284	0.438836977	0.579897528	0.653294969	0.986459176	0.454500789	10.26335374	10.13116955	10.10441409	11.47509805
GRIN3A	0.019328147	0.10519635	0.040914354	0.029302354	0.00504551	0.044660931	0.039752276	0.028369035	0.294473994	0.1271646	0.08327831	0.246403201
GRIN3B	0.136468579	0.113181074	0.144440226	0.02758568	0.11399807	0.056059304	0.168405279	0.040060559	2.153423098	1.870541921	1.528789546	3.365898814
GRINA	77.51529728	87.21938133	71.19097663	59.66340397	42.08299857	45.68534862	64.07969898	63.35474302	42.72848432	42.87411347	41.73360472	60.32401704
GRIP1	0	0	0	0.011795937	0	0.005992892	0	0.00571011	0.482640444	0.505514106	0.458866106	0.491910943
GRIP2	0.008253633	0.012834755	0.008735759	0.008341923	0.017236536	0.00847618	0.00848764	0.032304887	0.004491008	0.009050445	0.004445257	0.004294723
GRIPAP1	17.82620023	18.01892202	16.25841859	14.8449018	14.28801093	14.96661836	14.62303694	16.10297405	15.12703226	15.10461927	14.39515482	18.76527257
GRK1	0.035284958	0.109739256	0.037346086	0.095099748	0.04912507	0	0.012095119	0.023017674	0.038398857	0.025794262	0.02533845	0.024480393
GRK2	155.7986491	141.3472774	157.1934202	145.2207638	170.86881	161.1195786	134.9656724	156.1849394	219.2045191	228.1793376	210.9545654	238.5973351
GRK3	4.719517477	5.41735565	4.272704284	5.739467159	3.103054824	4.24243678	3.721689441	4.767784848	7.518306408	6.039817847	6.053524387	9.590362596
GRK4	0.863254987	0.847830727	0.945739965	0.551045896	0.822322975	0.808765507	0.872155756	0.489036486	0.412033894	0.33213833	0.44046327	0.551635649
GRK5	4.144051403	4.928542417	3.918565094	5.145117592	4.129444153	4.493422745	3.612577418	4.425480684	2.357896981	2.191327429	2.379194247	3.743476036
GRK6	12.3743241	15.00534563	13.99675787	17.850463	11.48533863	14.67194057	12.59662358	18.68842613	9.739305937	13.11665186	13.47727418	12.59162232
GRK7	0.027217785	0.018811034	0.00960256	0.009169646	0.028420232	0	0.027989463	0.062143062	0.04936626	0.049742349	0.029318009	0.037766919
GRM1	0.022336887	0.015437698	0.015761113	0.015050552	0	0.00764639	0.007656727	0.021856757	0.081027061	0.040822177	0.064161288	0.046491406
GRM2	0.018949253	0.039289204	0.033426917	0.02553594	0.013190938	0.006486731	0.012991001	0.012361293	0.020621525	0.006926209	0.020411446	0.032867061
GRM3	0.008828476	0	0.009344181	0	0	0	0	0	0.009607589	0	0.009509713	0
GRM4	0.005629793	0.00583638	0.00595865	0	0	0	0	0	0.098025959	0.129639241	0.121284168	0.140612426
GRM5	0.006529494	0	0	0	0	0	0	0	0	0	0	0
GRM6	0	0	0	0	0	0	0	0	0	0	0	0
GRM7	0	0	0	0	0	0	0	0	0	0	0	0
GRM8	0	0	0	0	0	0	0	0	0.067658298	0.051130307	0.066969039	0
GRN	94.264781	117.3621482	81.23658383	124.5864321	43.5324832	55.05907206	67.19270385	113.7295576	122.6010156	116.9550992	95.30729115	263.8315467
GRP	0	0	0	0	0	0	0	0	0	0	0	0
GRPEL1	22.66576358	20.36847888	25.00713406	20.36286413	23.18533491	22.64513968	25.50281049	21.36967723	16.38124876	14.56663904	17.74675957	15.92537459
GRPEL2	4.522362606	4.729080232	5.452483082	5.643868897	6.88476485	6.246249153	5.391977382	5.451277598	8.045519363	7.891206216	11.3099451	7.052723678
GRPR	0.040141411	0.062421621	0.084972445	0	0.041914807	0.061835649	0.041279499	0.098196435	0.087367784	0.088033382	0.064858303	0.083549271
GRSF1	20.15449091	19.91890404	20.66262248	18.4297068	20.07073922	19.42311289	21.92316079	18.68013333	17.41896284	16.40184643	16.90099827	16.0638767
GRTP1	0	0	0	0.009352566	0	0	0	0	0.211474359	0.131910037	0.289060947	0.048150386
GRWD1	26.6637767	19.19282689	26.50530498	26.92685872	28.43655973	26.66272125	27.62252273	33.05814962	13.63816085	11.91619287	15.85922836	15.91499649
GRXCR1	0	0	0	0	0	0	0	0	0	0	0	0
GRXCR2	0	0	0	0	0	0	0	0	0.165887414	0.083575601	0.08209873	0.105758071
GSAP	5.256733564	5.41822128	4.040568944	1.944514756	2.056386211	2.893706445	3.271504858	1.778815146	3.429087125	5.39876736	3.900013281	3.610289368
GSC	0	0	0	0	0	0	0	0	0.344750042	0.347376468	0.389986232	0.565169711
GSC2	0	0	0	0	0	0	0	0	0	0	0	0
GSDMA	0	0	0	0	0	0	0	0.021438489	0.095371754	0.048049165	0	0.045601709
GSDMB	4.997239483	6.290746725	4.502071712	4.449422307	5.559666642	6.0484084	3.945957245	4.656975418	4.499501234	6.882212818	4.069174331	4.983871041
GSDMC	0.011210325	0.023243384	0.011865163	0.011330243	0.023411166	0	0.03458448	0	0.036598911	0.024585156	0.084527485	0.011666437
GSDMD	8.547122179	9.370607269	9.459223368	10.21543035	6.162308832	6.112959646	5.824754514	7.118848488	23.36424628	19.73011068	20.47748812	28.78481059
GSDME	0	0.020059015	0.020479245	0	0	0.019870715	0	0	3.326934418	4.05243995	2.917885118	7.651773304
GSE1	18.58749636	16.16645871	17.39762392	16.25541367	20.54452511	17.26303587	16.68951607	15.69479459	26.69503341	16.67941306	22.23156316	23.83780545
GSG1	0.087694851	0.127277983	0.037126976	0	0	0.05403565	0.036072469	0	0.019086785	0.07692878	0.037784683	0
GSG1L	0.010012944	0	0.021195676	0	0	0	0	0	0.370483949	0.823470055	0.53927897	1.271281291
GSG1L2	0	0	0	0	0	0	0	0	0	0	0	0
GSK3A	32.00421793	30.95135527	34.21348817	34.89236609	33.3407667	31.16801449	35.50186539	38.03382707	34.26423945	32.73808563	33.56937587	34.82262869
GSK3B	12.55695248	12.24055612	11.51251397	14.35398026	10.47341641	10.50034255	10.96424379	11.01690528	15.34963601	13.98233174	13.04736492	17.18542058
GSKIP	4.73538324	4.706900183	4.767965211	5.467197239	4.166786319	4.42593653	5.799796765	4.407990703	4.168946449	4.045125203	3.285897505	4.688108209
GSN	44.3565299	36.68024218	38.45122877	17.67711383	31.05059924	29.90982146	34.90917063	19.56696661	90.06559808	70.47161851	89.13776226	60.13110777
GSPT1	31.26547291	27.94229096	33.08448165	34.20235262	34.6921319	34.58932228	35.16993369	33.01814067	22.7785142	25.29734753	25.50643018	23.50455453
GSPT2	0.037787613	0.019587121	0.039994931	0	0	0	0	0	7.813250591	6.629704983	6.878881965	9.103753568
GSR	29.31542178	26.51143879	29.8502516	41.69188833	30.8217683	28.35734058	30.42394099	37.65872035	33.4802065	33.79072566	33.3025578	48.87623188
GSS	21.76082341	22.55934451	21.83474999	24.24377219	18.82267128	22.60571311	22.11929634	23.61212401	20.04697089	23.56631168	19.30122798	21.18877253
GSTA1	0	0	0	0	0	0	0	0	0	0	0	0
GSTA2	0	0	0	0	0	0	0	0	0	0	0	0
GSTA3	0	0	0	0	0	0	0	0	0	0	0	0
GSTA4	1.453330476	2.485990569	1.768958771	1.432155014	2.42805882	2.537279664	1.494535257	2.275345991	5.416935326	7.410407582	5.792256679	4.688631638
GSTA5	0	0	0	0	0	0	0	0	0	0	0	0
GSTCD	5.939647023	6.098623582	5.551962821	8.257253342	8.150590793	7.735763073	6.985640775	7.415278127	7.677335458	9.220603223	7.893283391	8.052284037
GSTK1	131.2321264	126.1285362	130.7684171	113.1176019	106.782415	106.7347193	121.2318605	127.1228938	70.11706474	73.05761848	66.04608484	86.46485767
GSTM1	0	0	0.046003862	0.043929862	0	0	0.044697228	0	30.17784168	34.554258	32.96045184	29.03981668
GSTM2	0.719949526	1.050444313	0.451558226	1.104951394	0.751755978	1.287037439	0.411311952	1.069757216	3.627245854	3.45020623	3.015846821	7.353671056
GSTM3	0	0.01351687	0.027600088	0	0	0	0	0.01275816	11.63503048	11.25186397	11.74121257	10.44806206
GSTM4	8.216175037	9.620995672	7.840018945	10.00360103	7.356736688	9.093493182	6.960673808	9.89325196	13.85199277	14.86779528	12.33520434	16.03765932
GSTM5	0	0	0	0	0	0	0	0	0.035425443	0.28556261	0.035064552	0.101631395
GSTO1	59.31374495	69.07011099	65.21310836	67.83615686	70.42650638	75.93040108	72.90725172	73.67390658	46.84660565	57.56304612	51.10363949	46.89373437
GSTO2	0.061979981	0.041576349	0.042447361	0.040533698	0.041876468	0.033697685	0.056238737	0.039242646	0.035708674	0.051972144	0.031417686	0.09864973
GSTP1	60.77447271	73.48597242	77.10621853	79.76112931	82.40339465	95.8693968	105.4140724	129.1590622	207.2155443	222.0362939	221.2588208	209.6743738
GSTT2B	0	0	0	0	0	0	0	0	0.045570628	0	0	0
GSTT4	0	0	0	0	0	0	0	0	0	0	0	0
GSTZ1	2.045392664	1.675599615	1.574452221	1.879338628	1.105216135	1.189818486	1.676823269	1.833475176	6.304118583	6.242355394	5.068952819	6.058360401
GSX1	0	0	0	0	0	0	0	0	0	0	0	0
GSX2	0.044397327	0	0	0	0	0	0	0	0.048315394	0	0	0
GTDC1	1.114027647	1.037316725	1.033322314	1.256963544	1.429732586	1.210629635	1.216432208	1.296206246	1.701685404	1.865680737	1.627622936	1.943498635
GTF2A1	8.900517775	8.014614062	8.366887711	10.63893617	9.986646959	9.200138007	8.547223403	8.279017557	10.75117684	10.04174954	10.56123535	10.59205316
GTF2A1L	0	0	0	0	0	0	0	0	0	0	0	0
GTF2A2	12.93097974	14.06855369	17.10055087	19.75853929	18.61278446	17.30637617	18.61663788	21.41491193	20.48782431	24.48605302	23.99344942	24.85945948
GTF2B	30.03070368	23.34951821	28.46194296	25.08174442	22.83390447	22.87799807	29.78722127	25.19112705	28.75920489	24.99677959	26.0254225	25.42821637
GTF2E1	9.666388179	8.287290392	9.743846245	8.606720437	8.443240057	8.177980609	10.54003198	8.632858223	8.649323398	8.546969538	8.346353107	10.28323092
GTF2E2	15.04255047	15.10188349	16.30815035	18.17765146	15.87887787	14.56593609	17.38621953	18.9758006	9.965246871	12.26585499	8.938387725	10.19545169
GTF2F1	42.29357716	48.39155721	47.58354995	48.42061089	47.67105383	46.75884956	51.43684112	55.60036948	40.25043945	38.0180508	39.81832193	43.09740082
GTF2F2	14.67413728	14.18842165	15.50342541	17.06775671	17.08298749	17.08542452	18.07028493	16.17611384	7.396832471	8.146503582	7.53431203	7.443672477
GTF2H1	21.32924179	21.01020884	23.14548358	19.24281904	22.427832	22.88812874	23.82721554	19.24504807	14.1712611	13.42575194	14.65568529	13.97246516
GTF2H2	6.143374929	4.045334225	5.697396121	5.480989472	4.513331537	5.034887448	3.662945289	5.36515521	6.663751083	7.723889837	6.509644665	9.371329296
GTF2H2C	9.103647725	8.885966288	9.012829524	12.1453604	8.277388866	9.866911655	7.402987061	9.127238486	8.504817137	9.798227703	8.84059301	11.13564776
GTF2H3	37.07753683	35.40866586	36.88212232	32.83752662	34.74558818	35.94753334	40.51988352	31.08444411	10.85678635	11.35295845	11.81234136	11.44503042
GTF2H4	20.00611878	21.06533267	21.30750909	24.6571452	23.64036632	25.82685768	20.76681126	27.0015017	5.42585249	7.014506041	5.674572386	7.897280605
GTF2H5	3.708042108	3.858952146	4.015561273	3.603008376	2.959953739	3.396345864	4.262213942	3.453225178	3.349747311	3.29677228	3.415862009	3.352335304
GTF2I	21.29866051	17.38787739	18.62759628	18.12451925	19.45665828	18.99430792	17.7795533	15.61377401	21.16548446	17.15712166	18.47498368	20.40779682
GTF2IRD1	0.349687969	0.75799614	0.549564057	0.428398335	0.409395736	0.707351281	0.370499352	0.466597435	3.275018709	4.252776883	4.189040024	3.727378457
GTF2IRD2	0.48722372	0.386254904	0.455015563	0.188284206	0.374079884	0.279613505	0.486301076	0.392618374	1.356742574	0.675682582	0.571127306	1.401836615
GTF2IRD2B	1.936243613	1.478240324	1.350344884	1.68389225	1.238147027	1.418120555	1.589824907	1.365891864	3.021834744	1.925665774	2.457511564	4.233110623
GTF3A	138.5097956	136.0433281	154.6730449	185.8934645	162.637571	176.3690277	165.2566659	193.5857664	41.45791134	56.23066444	52.34717966	50.93347138
GTF3C1	15.96797015	15.31375003	15.31402351	17.18715192	13.8035951	15.27107649	15.08928596	17.29167507	24.47136956	23.07206364	24.31178255	26.68752925
GTF3C2	19.70154057	18.37302856	20.38055682	22.40682133	22.29761013	23.11358845	19.92468563	23.37411265	24.14980858	24.27417785	24.61820592	22.34750964
GTF3C3	18.34217509	16.67386034	17.75422889	17.79596185	18.68314086	18.65659736	18.6254477	15.41610799	21.91758725	23.49114659	20.18267147	22.56842337

GTF3C4	5.944459947	5.459355048	6.373572341	6.922186117	7.996506672	7.284138738	6.651479319	5.892376807	9.75215472	8.469277663	9.813045262	10.34768992
GTF3C5	18.69336649	20.75050492	21.51186578	25.08713635	23.96167856	24.8796575	21.0368848	27.41576071	22.07029762	21.73082038	23.31765383	21.01392517
GTF3C6	52.73336964	47.68074402	63.72716476	64.46073668	58.13855058	53.74806707	66.40220145	66.56438623	42.16194509	44.40855543	47.58960932	35.66245663
GTPBP1	7.129129615	6.582495274	6.490300238	7.190237531	7.459740749	7.367547903	6.251995145	7.760768761	13.89310557	14.6154608	13.61429818	14.60433806
GTPBP10	4.459158783	4.112433262	4.606363908	4.506858973	4.633809168	4.601374699	4.945094557	3.693105156	3.315350804	2.581734757	2.851205523	3.31152332
GTPBP2	34.12565729	31.18338526	31.35216227	28.56564564	29.35775647	26.96297896	30.00611738	28.98511916	42.77773883	36.61056268	36.85435438	40.09361336
GTPBP3	11.65302592	13.65149475	12.04733657	15.42402033	14.6918213	16.135387	11.79791042	15.65643561	5.849936508	7.832964211	7.597393943	5.744439505
GTPBP4	67.35416091	53.31993353	70.59455565	55.75204641	71.18563558	64.0356342	78.21939953	56.34224242	39.4476606	42.89388828	49.46389105	37.57428018
GTPBP6	1.761578471	3.698479595	3.885636799	1.376460938	1.499345108	7.069082045	2.953238671	3.259122111	0.676601595	1.655693586	1.722108389	1.263248887
GTPBP8	11.14258408	9.249393796	10.07271003	9.939220201	12.66997817	9.651237053	11.41772071	8.264608499	13.63626325	8.957185789	10.12300941	8.294604765
GTSCR1	0	0	0	0	0	0	0	0	0	0	0	0
GTSE1	4.678045628	4.500329531	5.494847823	12.44873752	10.71621167	9.212823245	7.401676944	13.30063534	16.58248288	15.07224773	17.94340841	13.66152677
GTSF1	0.078841847	0.367807396	0	0.039842614	0.04116249	0.080967705	0.121615754	0.115720722	77.60577804	90.68950724	83.0996733	79.01383089
GTSF1L	0	0	0	0	0	0	0	0	0	0	0	0
GUCA1A	0	0	0	0	0	0	0	0	0	0	0	0
GUCA1B	0.40297909	0.466915556	0.25089331	0.934370738	0.396030299	0.681626802	0.609418148	0.858219537	0.851287437	2.313387364	1.149020557	1.159448566
GUCA1C	0	0	0	0	0	0	0	0	0	0	0	0
GUCA2A	0	0	0	0	0	0	0	0	0	0	0	0
GUCA2B	0	0	0	0	0	0	0	0	0	0	0	0
GUCD1	20.59310863	21.26389422	19.84608712	22.37198668	19.27992355	21.98926185	20.78401817	24.14316369	30.13307217	28.97095327	30.44349183	32.57962819
GUCY1A1	0	0	0	0	0	0	0	0	0	0	0	0.005414707
GUCY1A2	0	0	0	0.006706353	0	0.003407143	0	0.01947824	0.426035781	0.447471364	0.400253456	0.473016163
GUCY1B1	0	0	0	0	0	0.013179217	0	0	1.075360313	1.632365234	1.271756913	1.482441634
GUCY2C	0	0	0.014663435	0	0	0	0	0.013556367	0.030153582	0	0	0
GUCY2D	0	0	0	0	0	0	0	0.007831761	0	0	0	0.041647255
GUCY2F	0.1455099	0.120679549	0.169410653	0.220599656	0.091163005	0.224150048	0.044890618	0.156620393	0.110845808	0.095734518	0.219433167	0.015143023
GUF1	17.45826951	16.50620307	17.73895029	17.33703699	18.53448431	18.16371373	18.9716845	15.33122075	12.94692942	15.39849883	14.83918086	12.14322511
GUK1	37.28622096	39.25549305	41.1517403	43.80111895	31.4419055	33.37954972	42.74036199	45.24251619	47.59583588	66.79215709	60.53777938	54.79218856
GULP1	0	0.00776397	0.015853245	0	0	0	0	0	0.008150071	0.106758089	0.016134086	0.031175448
GUSB	16.54068213	20.88588387	17.85160676	28.54457185	18.94755855	22.33831767	17.17710788	27.56709736	42.48195216	48.97898779	41.89881907	51.66521838
GVQW2	1.071429309	1.295870084	1.176016133	1.082890525	0.911585229	0.855803562	1.060998794	0.757177067	0.798914274	0.739730357	0.512935431	0.929185268
GVQW3	0.648596051	0.663100708	0.61688565	0.583032691	0.870750254	0.813421181	0.697721664	0.584935048	1.083147231	1.016017111	0.981965207	1.222438885
GXYLT1	4.137039472	3.983541659	4.601345375	5.768768209	5.586464341	4.522226737	4.549972756	4.981238723	7.317874449	6.697004177	6.329411997	8.391807879
GXYLT2	0.031699299	0.032862516	0.050326464	0.016019195	0	0.032554026	0.016299019	0	1.138393215	1.981295633	1.536540009	1.814397311
GYG1	30.75087604	25.46263268	31.84660178	27.97003186	26.89793551	23.23952131	33.90382793	28.74205728	18.94188302	17.16383895	18.01668648	24.29473503
GYG2	0.014590445	0	0.015442728	0	0	0.014983855	0	0	7.700855389	8.01550747	7.842432247	7.637593849
GYPA	0	0	0	0	0	0	0	0	0	0.020732387	0	0
GYPB	0	0	0	0	0	0	0	0	0	0	0	0
GYPC	11.77156844	14.15705985	12.80391106	8.300027893	6.923146394	8.314154596	8.80386588	7.102340526	14.78513711	23.11195986	21.55082799	26.8010294
GYPE	0	0	0	0.00962399	0.009942806	0.009778881	0.009792102	0	0	0.020882808	0.010256893	0.019819111
GYS1	22.88914156	29.75769897	21.50290195	16.02393304	17.7194962	19.77951737	17.89445727	19.00628093	28.14616059	39.77658072	31.94462054	32.44159535
GYS2	0.049285181	0.040874975	0.010432823	0	0.072047553	0	0.020273005	0.01929032	0.053634601	0.032425925	0.031852924	0.061548524
GZF1	2.475941429	2.17513313	2.363511862	2.713803548	2.289457066	2.279424632	2.553076953	2.330301366	4.412426341	3.920802207	4.43287854	6.318832617
GZMA	0	0	0	0.060697733	0	0	0.308789999	0.058764429	0.065355198	0.065853096	0	0
GZMB	0	0	0	0	0.057158493	0.224864531	0.112584268	0.053563508	0	0.120049592	0	0
GZMH	0.104789797	0.108635096	0	0.052955374	0.054709638	0.161422956	0.161641191	0.051268674	0.285093754	0.05745314	0.056437881	0.109053357
GZMK	0	0	0	0.052043224	0	0.052880822	0	0	0	0	0.055465745	0
GZMM	0	0	0.060523678	0.057795078	0	0.058725248	0.176413925	0.055954228	0	0.188111714	0.061595859	0.178529964
H1F0	0.484978875	1.436500988	0.855513882	0.466825482	1.422755845	1.778770126	0.569975978	0.813521639	382.0109474	247.2106019	289.2363641	369.0159196
H1FNT	0	0	0	0	0	0	0	0	0	0	0	0
H1FOO	0	0	0	0	0	0	0	0	0	0	0	0
H1FX	19.21004893	37.57192005	22.82646311	37.92887339	30.64734471	32.70891111	17.44140992	43.91820232	89.41111447	86.92084343	84.80793402	93.1952477
H2AFB1	0	0	0	0	0	0	0	0	0.113265488	0	0.112111612	0
H2AFB2	0	0	0	0	0	0	0	0	0	0	0	0.09427424
H2AFB3	0	0.107899655	0.110160119	0	0	0	0	0	0	0	0	0
H2AFJ	0	0	0	0.01483097	0	0.015069664	0	0	4.359532091	4.215747499	3.872543604	3.69559136
H2AFV	24.61695897	26.44527878	26.93876241	44.58103957	31.99924182	31.92983129	28.00872337	43.31777904	42.18833614	36.26310507	40.48940438	46.011
H2AFX	17.82399507	18.61803811	20.61590641	35.27871037	20.76162133	22.22205392	18.60705771	43.80036572	67.08116524	61.70658221	73.27028411	86.28186672
H2AFY	35.56550987	34.82845534	35.40415019	34.50106386	33.02731884	32.90532403	30.31724443	32.53599626	43.48452592	37.34482915	45.08281723	32.6530496
H2AFY2	0	0	0	0	0	0	0	0	3.251743508	4.874156669	3.511218395	5.114172037
H2AFZ	258.2517995	277.4479548	339.1306587	464.2552302	408.6150815	395.9846587	361.4607693	469.2615787	391.9754926	377.5781505	486.3397015	352.4914847
H2BFM	0	0	0	0	0	0	0	0	0	0	0	0
H2BFS	0	0	0	0	0	0.120131432	0.120293843	0	0	0	0	0
H2BFWT	0	0	0	0	0	0	0	0	0	0.066000419	0	0
H3.Y	0	0	0	0	0	0	0	0	0	0	0	0
H3F3A	253.340682	275.9265455	295.9185002	310.6919189	300.2448985	304.3487968	290.327265	326.0316918	280.9362709	274.2981131	298.9776014	272.6123418
H3F3B	143.918476	145.0270175	150.2550134	172.8121523	145.6025603	136.8666933	147.9601658	164.8785686	182.8998899	162.5765407	171.8794456	198.4525814
H3F3C	0.407262523	1.424949182	1.077630677	0.720333359	0.584725435	1.25473132	0.837618435	1.095898224	0.997207783	0.781514892	1.041884929	1.112560904
H6PD	5.734739162	5.686194695	4.728243467	6.107981317	4.791241312	5.168760035	4.099041247	5.557549477	11.64208675	10.8243841	8.796945943	16.70370649
HAAO	0.698825466	0.685757023	0.762231137	0.598468694	0.473468611	0.65740607	0.740581706	0.814301261	2.084109674	1.719766636	2.074370479	1.965263229
HABP2	0	0	0	0	0.035215794	0	0	0	0	0	0	0.017549012
HABP4	2.552438027	2.736104065	2.591268849	2.404249144	1.921845968	2.532102329	2.071274435	3.00728245	5.215256195	7.273207809	5.498786988	6.848526189
HACD1	15.68587811	9.048380872	13.68261158	9.861732944	12.5098381	9.470805447	12.53191254	9.507338244	7.302980797	6.952313427	7.760748386	5.869815716
HACD2	7.02764405	7.17049131	7.226755175	10.72151935	9.570448653	8.683352233	8.900487547	10.24249293	9.008343367	7.73854456	8.151611609	8.82248255
HACD3	23.94857616	20.64356849	26.50765253	28.19971705	24.12217188	22.75229992	30.04153096	26.83024218	17.67591834	18.77577468	19.03433725	22.55511203
HACD4	5.004482515	5.070211864	4.490253102	3.701548148	4.061696287	4.006412447	4.421199209	3.639295617	24.90365952	21.41418532	20.20267371	20.90341478
HACE1	5.166575366	5.90067104	5.842765413	7.226077182	7.487842288	8.134955952	5.357149719	6.188292373	6.392415313	8.874163871	7.262532192	6.92735382
HACL1	13.27004474	11.92760626	13.32301618	12.22298936	13.61061934	12.49219811	15.55772319	12.36319305	12.21350617	11.37775649	10.99929138	9.280097359
HADH	24.75192743	26.31878763	27.84136839	33.52999026	35.15667199	34.62537697	26.83283829	37.13562457	32.85100298	35.70706344	38.90302366	30.82755297
HADHA	76.06336674	77.93613051	71.05501757	84.98913736	73.04098814	78.87852746	75.17711645	82.29945695	167.6150572	149.7549284	174.9920516	143.2148328
HADHB	47.58293133	48.23026033	45.4079828	50.54674061	42.03058429	41.99522744	42.16553416	44.50760457	73.45613124	63.6530571	69.14020211	70.49840855
HAGH	8.839682937	10.16377367	8.845712747	10.10381567	7.317038292	7.262425857	8.330025272	11.57483729	13.74754781	12.1251522	13.08812663	19.16808186
HAGHL	6.644138014	6.228023938	7.369121426	4.966039425	6.501466903	7.497444841	6.362010059	7.279924286	2.251408035	3.381026996	2.935583512	2.546345492
HAL	0.54316515	2.127254529	0.587668896	0.74416673	0.252071775	1.103225844	0.35996408	0.649598712	2.508888986	3.957449065	1.846245383	7.084652074
HAMP	0	0	0	0	0	0	0	0	0.099588873	0	0	0
HAND1	0	0	0	0	0	0	0	0	0	0	0	0
HAND2	0.113618161	0.070672451	0.072153017	0	0.0711826	0.093345369	0.093471567	0.022235189	0.049457987	0.124586939	0.024477071	0.094592735
HAO1	0	0	0	0	0	0	0	0	0	0	0	0
HAO2	0	0.036071207	0	0	0	0	0	0.017023255	0	0	0	0.05431513
HAP1	0	0	0	0	0	0	0	0	0.029589822	0	0.01464419	0
HAPLN1	0	0	0	0	0	0	0	0	0.011317792	0	0.011202494	0
HAPLN2	0.032230944	0.016706835	0	0.032575722	0.016827433	0.033100005	0	0.047307213	0.157838968	0.353425423	0.138872006	0.402507808
HAPLN3	0	0	0	0	0	0	0	0	0.752124403	0.730788123	0.771050186	0.513751365
HAPLN4	0.422342484	0.243244717	0.46356915	0.094857852	0.359334177	0.240961302	0.434316725	0.183673006	0	0.017152434	0.101095995	0
HARBI1	1.792432687	1.365213039	1.471247919	1.55280563	1.069497187	1.164564389	1.974912518	1.557037649	2.189465764	1.203352298	1.260893621	2.265081224
HARS	38.19872122	36.11695303	40.45475099	39.14977445	34.98883037	33.78891872	42.18526675	38.45079414	30.90433601	31.65152258	32.92707363	35.86746469
HARS2	18.10294161	17.06112313	17.1836914	14.24106477	15.50446711	16.91995484	17.49427988	14.547407	21.73296138	20.82388057	19.75876633	22.5535084
HAS1	0	0	0	0	0	0	0	0	0	0	0	0
HAS2	0	0	0	0	0	0	0	0	0	0.018016603	0	0
HAS3	0.415147947	0.353133906	0.416865066	0.215173763	0.311222623	0.349818927	0.29555876	0.333312306	0.602379697	0.583623881	0.630641682	0.731142893
HASPIN	4.482526879	4.148408049	5.273782752	8.53596318	7.713882973	7.942332622	5.559235672	8.979423243	4.836237891	4.641030517	4.704078202	5.065327639
HAT1	18.2388671	17.82804618	22.09352769	26.68465903	28.68214481	24.94902708	24.37772544	25.762754	25.37079839	24.39474703	27.2512429	25.49927079
HAUS1	34.19896535	37.38775801	37.7153974	50.27606697	52.39106823	52.55883615	40.97261764	49.79796954	40.28808086	40.33276789	40.49591029	30.96116896
HAUS2	9.967040678	10.42319662	10.50969361	13.49870357	13.67268473	13.39608708	11.73582167	13.10532483	8.867122047	8.716089566	10.03828533	8.920408057
HAUS3	8.684664238	8.453528093	8.630626818	11.70342027	12.13727149	11.32442252	9.249040381	11.00630001	13.03432252	12.79437768	11.56853716	14.99436634
HAUS4	53.34965008	60.48388287	55.66630212	66.33795291	69.49600368	72.53356894	58.54447249	71.48969023	49.94254281	54.78977618	53.14925452	43.9991348
HAUS5	9.659699908	11.79559325	11.34119025	16.95814906	15.22580218	16.22267568	10.93071519	17.89526989	8.495221599	11.3190417	9.849787688	12.79901839
HAUS6	10.902693	11.64331377	12.17696821	13.42238609	15.39420282	13.77495442	13.05363045	11.69384803	20.50390135	22.24670742	21.79461154	17.76825526
HAUS7	18.225819	16.27718507	18.28835646	22.9661709	21.70853583	22.76860536	20.28415245	28.91278835	7.126591402	10.42525969	9.17868621	12.1111988
HAUS8	10.66337275	11.71138199	13.37217036	21.27032755	17.05080089	17.27566985	15.85140846	23.17555315	8.693737314	9.145870975	8.908437122	11.13385562
HAVCR1	0	0	0	0	0	0.033850204	0	0	0	0	0.035504872	0
HAVCR2	0.332157783	0.459128574	0.421872454	0.179045823	0.277465672	0.204668365	0.569291851	0.086671487	0.024098048	0.121408178	0.071557659	0.138268886
HAX1	92.00290023	93.95470369	98.49195882	93.72763099	101.8056973	99.9391272	106.5731786	101.8105984	110.8370755	134.4295405	161.1582007	111.5688699
HBA1	0	0	0	1.076934032	0	0.273566627	0.547872949	0.347544081	0.579784723	0.292100863	0.382585503	0.184814848
HBA2	0	0	0	0.539357044	0	0.822056408	0.731704699	0.174059267	0.677533553	0.195055783	0.287413407	1.203282124
HBB	0.085957765	0.356448062	0.090978884	8.166463016	0.628286886	9.533753235	7.071586949	4.794276221	18.33453416	1.885123781	13.24045307	14.67063803
HBD	0	0	0	0.070265076	0	0	0	0.068027039	0.075656663	0	0	0
HBE1	0	0	0	0	0	0	0	0.064525647	0	0	0	0
HBEGF	24.53150039	23.30252751	22.72797591	17.02131231	12.86720579	12.74880808	19.19599971	14.46950702	5.264779685	4.854473547	5.801086541	8.16947464
HBG1	0	0	0	0	0	0	0.189504	0.090159124	0	0.707244213	0	0
HBG2	0	0	0	0.093285023	0	0	0	0	0	2.530204732	0	0
HBM	0	0	0	0	0	0	0	0	0	0	0	0
HBP1	8.129922782	8.625316643	6.469093094	5.408959703	6.931740918	7.613329482	6.691345029	6.037917324	15.43519276	14.39835004	11.41908564	12.82800862
HBQ1	0	0	0	0	0	0	0	0	0	0	0	0
HBS1L	9.553907683	9.086473668	9.805563782	10.68884577	10.90660507	10.37117373	10.08747443	9.043039067	15.51216199	18.82985889	16.06955979	14.77510771
HBZ	0.039977385	0	0	0	0.041743535	0.082110637	0	0	0	0.087673662	0	0
HCAR1	0	0.018738368	0	0	0	0	0	0	0	0	0	0
HCAR2	1.198663341	1.701888459	1.516902165	0.842772588	0.027209103	0.10704205	0.267966915	0.178484503	0.028357509	0.028573547	0	0
HCAR3	2.039576242	2.140523154	1.67900104	1.170668112	0.078877116	0.05171779	0.362513968	0.197109699	0	0.027610844	0.027122931	0
HCCS	4.245391665	3.9815056	4.866913989	4.542588349	4.021084547	4.114686923	4.728680434	4.672134844	5.4898366	5.770682445	6.160667191	5.573964468
HCFC1	23.48084257	20.66165001	25.02515811	30.69295775	28.44298778	26.04979057	20.40856814	30.61831375	26.40418325	21.10163759	25.06239095	22.34568629
HCFC1R1	4.583437893	6.557247695	4.996709092	6.902376594	6.46100325	5.507217762	3.582174411	9.418326191	6.584062975	10.40366737	9.429885348	8.5381626
HCFC2	1.714275862	1.660857191	1.66266229	2.072836014	1.750955611	1.651668016	1.660311455	1.714026049	2.957761826	3.410934059	2.350161756	3.023110155
HCK	51.14851912	47.19068151	45.6868482	27.10928098	18.66336112	17.75106196	35.23530384	24.33325703	29.80448707	26.51969035	31.17502573	28.42832501
HCLS1	191.1321939	180.2719458	185.9488748	147.4910828	142.5071453	141.7032171	185.0388465	153.8250909	171.5371907	151.998511	160.8878104	170.4421833
HCN1	0	0	0	0	0	0	0	0	0	0	0	0
HCN2	2.226275845	2.09517828	2.005379623	1.851138368	1.928948192	1.199904323	2.22444777	1.900325758	4.141003507	4.657328844	3.520561226	6.638367116
HCN3	0.669327735	1.308151304	0.952309965	1.020276409	1.145734067	1.115576142	0.868843378	1.213250591	2.543415325	3.044067437	3.155745285	2.169614759

HCN4	0	0	0	0	0	0	0	0	0	0	0	0
HCRT	0	0	0.098704994	0	0	0	0	0	0	0	0	0
HCRTR1	0.016091376	0	0	0	0	0	0	0	0	0	0	0
HCRTR2	0	0	0	0	0	0	0	0	0	0	0	0
HCST	45.70919686	55.10931377	42.79113541	24.56104388	30.88763527	25.92190866	41.50137591	22.64642078	17.23690631	11.81673626	16.20434726	18.66629961
HDAC1	69.96761118	77.41424063	77.91122118	88.18683324	74.7798505	70.29504524	80.31517111	83.00104904	92.92376383	75.05755963	94.12644258	90.12640188
HDAC10	20.15622265	21.45226106	20.40790464	20.15084008	19.65603927	19.76066638	19.13325347	24.74123562	19.64200126	16.87084808	17.66472313	27.45125185
HDAC11	0.212156393	0.208365676	0.082728672	0.067713428	0.011659431	0.080270432	0.068896246	0.076482776	0.887062205	0.734646703	0.721664706	0.987737373
HDAC2	27.94329183	29.34489742	29.82073454	31.98025842	36.84512408	35.90462855	33.77755835	32.56336936	31.91336782	36.27130718	33.9253536	30.2212453
HDAC3	43.78045458	51.20646352	48.40116333	47.78583343	44.9298189	45.7327933	48.95870157	47.57379382	43.37554004	54.85784258	45.00823693	47.12609208
HDAC4	1.371074568	1.78026908	1.328428948	1.218555942	1.047025341	1.238167719	1.113577727	1.091304276	1.601564805	1.574497154	1.555858623	1.977016331
HDAC5	12.06845807	13.20037184	11.71818299	12.07279726	11.45151798	11.38948573	11.6294662	12.73816212	21.49306065	16.64866677	19.0853254	22.17425957
HDAC6	6.333667401	7.366152568	5.92436614	7.360735809	6.002150596	6.932609458	5.163156119	7.464806851	10.45476301	11.32230329	10.03293585	10.28400483
HDAC7	10.58002577	10.20281878	10.01629403	6.224808454	12.4671511	9.774147374	10.54664595	8.37900516	5.565778114	5.163870111	5.072618978	4.97580577
HDAC8	6.306993925	5.522613741	6.806872554	6.346563661	6.700913404	5.995171576	6.301311756	6.117408702	7.013774313	6.719143937	6.808530463	7.870581319
HDAC9	3.432329194	2.535274304	3.059003403	1.478286331	2.480266771	2.343242118	2.366464848	1.202208787	13.62086452	9.020022144	12.44437273	5.885648254
HDC	0	0	0.017523933	0	0	0	0	0	0.036035851	0.544655763	0	0.034460861
HDDC2	38.12381991	40.2042143	40.57213682	37.17276099	39.99526699	39.51997259	45.10384589	42.21589589	39.01716184	43.9666132	40.13228454	35.07315818
HDDC3	17.18708351	19.57882823	18.93137949	18.83534623	19.51147882	23.29456656	16.38989652	21.55983416	12.23361916	16.65490232	13.18534577	13.36278273
HDGF	121.8238479	128.9290509	134.0560541	162.3990618	139.5796652	154.0919713	150.7887333	176.877832	198.0731773	246.0709811	258.7030198	150.1633758
HDGFL1	0	0	0	0	0	0	0	0	0	0	0	0
HDGFL2	20.59557316	24.56531235	23.39132089	27.41247043	23.87181707	26.53172286	24.47947285	30.86795663	22.59070227	23.85621604	26.12963595	24.01839563
HDGFL3	0.019790203	0.010258206	0.073311782	0	0.010332254	0.010161909	0	0	0.204598544	0.227858011	0.18119694	0.236847126
HDHD2	9.0123886	8.276826535	9.043032459	7.863411962	9.123443078	8.167755136	9.320268625	8.559588461	11.24841615	12.36143876	10.28918964	11.7741818
HDHD3	3.141576158	3.646566837	2.898794258	2.768107384	2.607471194	3.005683625	2.650786485	2.36465247	0	0.029443301	0	0
HDHD5	14.7062954	15.11756117	15.27042353	14.20648248	16.42283873	18.09160007	14.6615908	15.64743238	7.698827238	13.07058925	12.3727227	9.118347739
HDLBP	55.0617733	52.04452766	54.27947212	51.78204404	50.77836423	50.37959299	56.79116397	53.14026205	77.20274774	78.53986997	80.58539124	71.48133933
HDX	0.2840197	0.252378774	0.32637697	0.221444662	0.254200567	0.208341346	0.408901105	0.198510512	0.008830984	0	0	0.008445016
HEATR1	32.92378954	26.0378503	31.97384457	32.77597443	38.84200542	36.5537121	33.224581	28.27519348	18.31843638	15.96818052	18.99263746	15.55744985
HEATR3	13.03077331	10.38521781	13.31595534	11.64747134	11.3993693	11.08442128	13.66614468	11.58452435	7.108722807	7.249179297	7.302738677	8.857326874
HEATR4	0	0	0	0	0	0	0	0	0.094798288	0.02729157	0	0.03885215
HEATR5A	1.427054527	1.102452998	1.241734745	1.463122603	1.210706231	0.979370616	1.298185757	1.013718243	3.270243095	2.723394557	2.793513194	3.841311689
HEATR5B	9.039324012	8.79638686	8.08561013	9.610057526	8.711476703	8.662726402	7.914419811	7.447343666	10.93520543	9.553534463	7.876729142	13.14184406
HEATR6	3.237084953	3.326941006	3.03482343	3.455041179	3.030430213	3.496318395	3.113634495	3.051453256	5.587822792	4.80419384	4.854565093	6.229360197
HEATR9	0.196624461	0.045297703	0.046246676	0.044161728	0.159686395	0.179489918	0.044933145	0.085510237	0.095100702	0.191650424	0.211796725	0.068208159
HEBP1	17.13538539	20.02506933	18.46607963	19.43291384	17.10235062	18.74010588	17.85005415	18.4655431	11.23698564	14.44606684	10.93074312	15.05346743
HEBP2	29.61381349	37.43306925	31.61861307	40.69510892	38.052025	43.21748967	38.96359062	43.6111496	36.83112444	48.18012361	43.49126454	38.73579002
HECA	10.54939265	10.05324947	9.068268861	9.578601123	10.74556281	10.32262613	10.37595925	8.102612169	21.99190191	22.49535211	18.46813934	18.63973309
HECTD1	21.56585419	20.11384418	20.15048796	26.38317869	22.02043311	21.38907553	20.23232231	20.00744585	33.78503622	25.88370063	26.49695361	37.6813363
HECTD2	0.022868492	0.007902553	0.032272436	0.015408746	0.023878792	0	0	0.022376935	2.762416717	2.223425921	2.192346632	2.609950095
HECTD3	12.35143681	13.65012402	12.6491986	12.64841066	10.96176727	12.53220099	11.16157148	14.5959895	19.54093395	16.58431023	17.72871068	21.83478695
HECTD4	10.52159763	10.07363751	8.544760328	9.944213676	8.578942263	8.365968452	8.226853114	7.923498923	12.44694603	10.96068313	9.435189948	12.84893828
HECW1	0.044030407	0.086727626	0.013980717	0.022250703	0.03218293	0.122087585	0.013583627	0.017233591	0.095832186	0.077249815	0.184969024	0.027493118
HECW2	0.012794538	0.008842692	0.013541915	0	0.004453261	0.004379841	0.008771525	0	0.204213625	0.144973893	0.128630237	0.208603332
HEG1	0.233311336	0.241872778	0.258985786	0.172541779	0.273328335	0.1227231	0.158000162	0.150341483	3.294521231	4.137045283	3.984254164	3.647982412
HELB	2.19607639	2.087923354	2.119621381	2.656581507	2.590129436	2.465628411	1.755186124	1.658973635	2.649919013	3.406258024	2.242967175	2.380160428
HELLS	5.581626009	7.567835626	7.107600075	9.514010921	11.44607005	12.17823349	6.048602626	10.00336063	5.74319714	7.14272598	6.671850781	5.052315862
HELQ	4.724545788	4.064227062	3.95786188	4.612528777	4.828307	4.23254026	4.899844867	3.895116685	4.94457915	4.320888239	4.970002238	5.136457936
HELT	0	0	0	0	0	0	0	0	0.119669463	0.040193715	0.039483449	0.152585555
HELZ	10.90443907	9.576841017	9.641785413	7.39312081	10.02787304	9.208140318	9.906898881	5.412504089	12.75717337	13.49935409	11.0757176	11.76012191
HELZ2	4.804060415	3.35551548	2.94490188	3.488081022	2.926617879	2.820695134	3.102759413	3.571806808	4.900226065	2.267245883	2.744107223	3.809412758
HEMGN	0	0	0	0.02121934	0	0	0	0.020543476	0.022847545	0	0.022614789	0.021848966
HEMK1	3.440876738	3.078843536	3.106610756	2.565669061	3.023411997	2.800447085	2.483020986	2.741076615	2.519737036	2.696597224	2.248399267	2.636638469
HENMT1	22.30420798	21.29144086	25.28134241	25.15413971	28.24480911	24.93624808	23.58725921	26.62313671	36.86788845	28.18680303	35.04396978	32.92438932
HEPACAM	0	0	0	0	0	0	0	0	0	0	0	0
HEPACAM2	0.020872599	0	0	0.021095876	0.021794724	0	0.021464379	0	0.045429214	0.022887655	0.022483205	0.065165515
HEPH	0.018716369	0	0.019809663	0	0	0.009610515	0.019247015	0	0.112024492	0.102616303	0.070562074	0.116867267
HEPHL1	0	0	0	0	0	0	0	0	0	0	0	0
HEPN1	0	0	0	0	0	0	0	0	0	0	0	0
HERC1	9.554806569	8.505783262	8.03378049	8.488913055	7.672049323	7.559818925	7.052521396	5.848061312	15.79531934	14.12695031	13.44738442	15.03272813
HERC2	10.89251629	10.15475141	9.543002843	11.54843074	9.155059593	9.463689695	8.160684525	9.467990992	12.71233277	10.80638237	11.20457254	14.33003301
HERC3	3.633549229	2.935233975	3.411273317	3.834576476	3.459013649	3.232370943	2.736914381	3.116787391	4.431796531	3.72561164	3.619111901	4.409980808
HERC4	14.10296345	12.50916668	13.55314177	11.62369466	13.60714307	11.80393469	13.45565121	10.32218817	9.118719313	11.15865039	8.402117092	10.04309256
HERC5	4.389621999	3.914432542	2.485417681	5.906447767	3.273964215	3.891388602	2.305060303	5.248320651	13.21299626	8.822126886	8.594371135	10.6776973
HERC6	1.519389371	1.228078295	1.076637885	1.028099622	0.914262337	0.912412453	1.138747173	0.844160373	4.61011404	2.047292242	2.149811933	3.229417234
HERPUD1	18.0578391	19.56217379	20.59426795	21.8729112	12.42424007	13.71448425	14.85585152	19.7790917	29.49240002	22.99390031	29.73477617	25.98908312
HERPUD2	8.057380558	7.737901965	7.404192114	7.275555056	6.098044889	7.087963635	7.691684682	7.364687015	13.13567403	11.57485695	10.71433698	13.1628288
HES1	3.60940244	2.825479128	3.781259265	2.717397199	4.499558823	3.63107736	4.544982988	3.135389982	5.811736669	3.473221214	6.863363951	4.676157192
HES2	0	0	0	0	0.0131832	0	0	0	0	0	0.054398596	0.013139113
HES3	0	0	0	0	0	0	0	0	0	0.08465477	0.083158829	0
HES4	0.410760007	0.475930989	0.409180288	0.390733138	0.630745382	0.446649418	0.54664288	0.212786868	0.36812555	0.264950042	0.442455753	0.226308976
HES5	0	0	0	0	0	0	0	0	0	0	0	0
HES6	4.959065444	6.522215054	6.463004756	5.647978307	7.844511776	9.235413639	7.535325475	8.365080085	5.960537858	4.139233964	7.932860405	5.045292811
HES7	0.145864898	0.136095716	0.123508336	0.029485047	0.076154512	0.089878762	0.030000091	0.071364771	0.015873748	0.03198936	0.015712037	0
HESX1	0.763574737	0.574293963	0.618018495	0.438834137	0.656606996	0.645781653	0.477292767	0.410206453	1.743387175	1.707416509	1.467589046	0.997197976
HEXA	28.28600922	27.10551683	23.94652374	21.90349885	17.61169718	18.44013133	21.52590045	21.30102198	35.77854463	39.91221417	34.38725803	38.34487144
HEXB	41.79790673	45.94121143	42.01932736	61.59337929	36.12831902	39.54626169	41.37081489	54.64064659	90.61271538	95.44326782	79.05814648	122.8207281
HEXDC	3.975988095	4.454596862	3.43452823	4.559127785	3.928235424	4.687434524	2.695251716	5.111765407	5.801497303	3.988367252	4.109941876	5.696375727
HEXIM1	11.39165834	13.75658591	10.9858761	12.54780065	11.1434215	10.89041012	12.70916395	12.89639122	16.12952619	12.33111272	13.60313338	16.92255125
HEXIM2	0.567186803	0.606967662	0.503492796	0.388333404	0.649558366	0.732797648	0.827863783	0.572898234	1.075194111	0.842633024	0.985408087	0.818753026
HEY1	0.062617798	0.021638527	0.088367388	0.042191752	0.021794724	0.021435399	0.021464379	0	0.090858428	0.022887655	0.022483205	0.065165515
HEY2	0.019959036	0.02069144	0.021124919	0.01008627	0.020840801	0	0.020524914	0.019530018	0	0.010942948	0	0.010385552
HEYL	0.051578779	0.12031083	0.027295845	0.065163154	0	0.013242382	0.053041139	0.088322669	11.77339508	12.31555477	11.58400691	13.11069355
HFE	1.09403236	1.221422746	0.837278924	0.306203639	1.564161739	1.17538667	1.453911199	0.922290893	0.824248223	0.719790616	0.833981348	0.490450162
HFM1	0	0	0.010492406	0	0	0	0	0	0.409950952	0.336981504	0.266956999	0.257916815
HGC6.3	0	0	0	0	0	0	0	0	0	0	0	0
HGD	0.021251801	0.110158218	0.134959197	0.042958269	0.022190679	0.043649651	0.043708663	0.124769972	1.248872781	1.840973765	1.304825082	2.034715124
HGF	0	0	0.022431186	0	0	0	0.014529386	0.006912555	0.207571608	0.333095457	0.258723635	0.044110988
HGFAC	0.100861407	0.104562552	0.026688276	0.101940335	0.052658668	0.025895248	0.129651284	0.172713448	0.164643647	0.02764966	0.135805304	0.393619251
HGH1	16.77429614	16.64046301	21.7370157	27.26779097	21.11167345	19.89027173	19.72039566	30.58072092	12.67875341	12.37902915	14.17554112	18.76217551
HGS	24.51748263	25.10353655	25.12827432	24.07517486	27.37235142	26.27269419	25.33433621	26.93780958	29.94472593	25.90365591	27.81198345	35.9592049
HGSNAT	21.55492252	21.46938113	18.92597392	20.5184427	14.69360383	15.97832656	14.67076512	18.25783319	39.08819182	32.09701563	34.5236949	36.40889215
HHAT	0.23922955	0.177148688	0.189902891	0.293600466	0.16058469	0.157937163	0.210867582	0.19228602	0.269639482	0.084318731	0.092031921	0.168939199
HHATL	0	0	0.028250388	0	0	0.027410942	0.027448	0	0	0	0	0
HHEX	26.82830294	28.76418327	28.84873686	37.00912269	37.85182284	36.19104506	33.72330868	37.3270049	17.67733627	13.85378431	15.65196659	18.71935902
HHIP	0	0	0	0	0	0	0	0	0.010587282	0	0.041917703	0.030373657
HHIPL1	0.005318201	0.005513355	0.005628858	0	0	0	0	0	2.233987668	1.947762506	2.411729318	1.914965309
HHIPL2	0.424440007	0.374827578	0.415956627	0.127105273	0.410362245	0.371308953	0.40414233	0.2768778	0.376360402	0.172376203	0.321727248	0.261753544
HHLA1	0.013108297	0	0	0	0	0	0	0.012826536	0	0.028747564	0	0
HHLA2	0.417012927	0.494074701	0.23644941	0.240842153	0.466538599	0.47414177	0.336942622	0.233171008	0.210699513	0.604251827	0.144382877	0.449479122
HHLA3	1.609687718	1.350897534	1.514413938	1.626906753	1.36064897	0.760946485	1.602775518	1.150063961	5.644504361	5.295264365	5.834701413	5.876427656
HIBADH	13.71565895	14.24558716	14.51684259	15.31610953	16.35987931	15.7736297	14.3422416	15.73304684	20.23683919	20.9542981	19.53267913	16.51900691
HIBCH	10.75152423	8.292234306	10.06019206	11.91644138	9.572364825	10.32133082	10.60234636	11.18112378	12.32210429	13.69744405	11.16154429	13.18892984
HIC1	0.286400293	0.296909852	0.285298842	0.11919104	0.369418526	0.224917333	0.381143924	0.148364544	0.073335325	0	0.018147058	0.035065059
HIC2	1.961104505	1.974813832	1.986448304	1.788985809	2.03014122	1.656197954	1.958920417	1.770493204	4.286689453	3.814088108	4.485131831	3.760159978
HID1	0	0.016681855	0	0	0	0	0	0	0.017511441	0.035289698	0.034666091	0.066984329
HIF1A	21.52382434	19.92743369	21.25200485	23.89482607	20.05849535	18.10581578	20.67357125	19.85117229	26.56772801	26.51504877	24.27929384	28.15871819
HIF1AN	20.95423949	20.13893206	19.27016797	19.68069139	18.30986897	17.20746637	18.1037748	17.91347475	20.75408107	19.2872876	17.73268989	23.05175555
HIF3A	0	0	0	0	0	0	0	0	0	0.007277303	0	0
HIGD1A	17.24371852	15.21053138	19.4627508	17.8198212	13.0765348	12.71623646	18.69260008	15.70108599	21.16147817	19.5651166	21.30638076	21.66634973
HIGD1B	0	0	0	0	0	0	0	0	0	0	0	0
HIGD1C	0	0	0	0	0	0.020391313	0	0	0	0	0	0
HIGD2A	77.96030865	74.92265614	76.68445357	76.83663865	74.38904611	71.04916101	70.39828444	92.27588563	39.52188328	45.66367779	50.39864671	42.32987629
HIGD2B	0	0	0	0	0	0	0	0	0	0.089063207	0	0
HIKESHI	11.97774341	11.88688499	12.35888095	15.75588218	12.94684622	12.64067537	13.27672652	14.78286914	20.92769925	22.24214111	24.18312692	22.64385003
HILPDA	9.450971029	21.92647843	9.156006626	7.048502729	16.71278713	17.72874485	7.563517989	7.196894594	10.03618853	29.12609697	12.80740194	8.209470297
HINFP	6.282877703	6.766994308	6.083592548	6.890848067	6.991425511	7.268634181	7.152699257	7.000446152	13.82440671	13.05807035	12.39919399	12.7747488
HINT1	167.2163388	167.5956098	167.2160149	162.1278791	134.5869828	150.841775	167.292368	170.9689562	156.8646334	180.1863234	190.2289635	129.1718786
HINT2	9.610685655	11.45519213	10.82483618	16.67396287	11.26955846	11.7698971	10.41167915	14.88564163	17.61781373	16.7939862	20.92600189	17.91750822
HINT3	9.345033203	9.770473386	7.801520042	10.32996398	8.461266421	9.907644347	8.562216805	10.25018545	9.944508744	13.80842015	9.808903696	16.02098674
HIP1	33.77044424	25.05290668	35.51497072	16.33861686	20.72583253	17.6302217	26.89179636	13.81457014	8.996226109	9.113423041	9.867420097	7.296746243
HIP1R	2.790194734	2.756688805	2.97300059	2.034593918	2.287752024	2.2404188	2.416761291	1.887333084	0.387195714	0.420946467	0.484106799	0.253344592
HIPK1	10.59416625	10.82258828	10.74081858	14.41693945	9.764055654	10.11011043	10.31952556	10.70403882	34.60672587	28.39985031	28.30807061	37.32847225
HIPK2	3.025643546	3.4663207	2.852228467	2.681439101	2.129685258	2.506891214	2.758005443	1.892023091	5.830309364	5.895858811	5.438775034	8.530364104
HIPK3	33.96447858	23.83217079	31.15347892	21.50576991	21.3769116	17.66055892	24.39419772	14.92737659	45.45667353	28.30008529	33.93192519	41.91670152
HIPK4	0.068569049	0	0	0	0	0.017604479	0.017628279	0.01677379	0	0	0.018465022	0
HIRA	21.03842537	19.97552526	22.89181068	24.54311994	23.11597422	23.10666571	23.30337247	24.4042977	19.81612586	17.59985948	21.92520961	20.58270017
HIRIP3	7.750130321	9.883013686	8.015991857	16.91507212	14.1388696	15.06608959	9.186218814	16.82544363	12.02672865	13.62830694	13.52059426	14.606012
HIST1H1A	0	0	0	0	0	0	0	0.067417322	0	0	0	0
HIST1H1B	0	0.141225625	0	0.275367942	0	0.139899895	0.070044516	0.066649276	0.222373128	0	0.14673849	0
HIST1H1C	3.675516452	3.810390826	2.411934727	1.337340211	1.688674276	1.132386446	2.267834749	1.007023219	4.239873804	2.579426201	3.246488866	4.590073675
HIST1H1D	0	0	0	0	0	0.142240563	0	0	0.075364552	0.075938706	0.074596787	0
HIST1H1E	0	0.142125151	0.145102628	0.138560939	0.286302159	0	0.140981319	0.134147588	0.298386023	0.075164808	0.221509696	0
HIST1H1T	0	0	0	0	0	0	0	0	0	0	0	0
HIST1H2AA	0	0	0	0	0	0	0	0	0	0	0	0
HIST1H2AB	0.338424911	0.116947844	0	0	0.235584061	0.115850018	0	0	0	0	0	0
HIST1H2AC	4.730510845	3.780242679	2.920655451	0.996065359	2.366843166	1.821773359	2.33096861	1.44650902	4.182732647	2.161332394	1.48619753	3.076862573
HIST1H2AD	0	0	0	0	0	0	0	0	0	0	0	0

HIST1H2AE	0.190814046	0.197816034	0.302940326	0	0.199243967	0.293938609	0.294335999	0	0.311480091	0.523088425	0.102768978	0
HIST1H2AG	2.485180522	2.016293555	1.601082205	1.419693156	0.902599175	0.998682986	2.222295899	1.374474033	0.940694893	0.710896076	0.465555853	0.56223794
HIST1H2AH	0.111870189	0	0.592024754	0.339200636	0.350437413	0.574433041	0.345125787	0	0.121742738	0.245340434	0	0.232843654
HIST1H2AI	0.573662696	0.356828071	0.121434502	0.463839391	0.359403829	0.235652275	0.353956298	0.112266372	0.249715382	0.125808901	0.123585722	0
HIST1H2AJ	0.122573032	0	0.129732987	0.123884211	0.255976304	0.125878038	0.126048218	0.119938333	0	0	0.132031216	0
HIST1H2AK	0.233954612	0.12126983	0.495241577	0.118228627	0.244290429	0.360394295	0.240587686	0.114462888	0.127300559	0.128270379	0.126003704	0
HIST1H2AL	0	0	0.12117613	0.115713125	0.11954638	0.235150888	0	0.112027507	0.498368145	0.125541222	0	0.35743978
HIST1H2AM	0.110491911	0.343639353	0.350838489	0.223347715	0.461493213	0.45388467	0.454498299	0.973052063	0.240485655	0.12115888	0.119017872	0.459949888
HIST1H2BA	0	0	0	0	0	0	0	0	0	0	0	0
HIST1H2BB	0	0	0	0	0	0	0	0	0	0	0	0
HIST1H2BC	4.176997875	1.273610084	2.340525259	2.731675135	1.539364346	1.387819736	2.021375996	2.163818972	2.406503715	0.538852734	0.926328597	1.662067774
HIST1H2BD	2.44588913	1.732688628	1.33752744	0.288406197	1.191841183	0.711687724	0.96417338	0.358997239	0.887246319	0.983406239	0.658655723	0.46665749
HIST1H2BE	0.24740028	0.25647872	0.130925934	0	0	0	0.127207282	0	0	0	0	0
HIST1H2BF	0.250277027	0.129730516	0.132448329	0	0	0.257025389	0.257372874	0	0	0.137219475	0	0
HIST1H2BG	0.483681446	0.125357577	0.255967556	0	0	0	0.124348692	0	0.657957945	0.662970498	0.130251019	0.377520666
HIST1H2BH	0	0	0	0	0	0	0	0	5.373581236	4.581516126	6.137121564	3.162290758
HIST1H2BI	0	0	0	0	0	0	0	0	0	0	0	0
HIST1H2BJ	0.671221133	0.115975305	0.592024754	0.113066879	0.350437413	0.459546433	0.230083858	0.43786219	0.486970953	1.226702169	0.241005005	0.116421827
HIST1H2BK	27.90574746	14.84222404	20.161413	3.004366698	7.538025972	5.170933552	15.03469612	3.442919778	6.799887799	3.259542664	4.378167018	6.628956458
HIST1H2BL	0.237569805	0	0.125723579	0.24011112	0.248065336	0.243975535	0.244305377	0.34869489	0	0.130252482	0	0
HIST1H2BM	0	0.125076506	0.127696819	0	0.125979369	0.37170712	0	0	0	0.132296803	0	0.125558069
HIST1H2BN	1.438117439	0.869685639	0.507374444	0.363375291	0.750825817	0.369223554	0.862686359	0.234534202	0.130419281	0.262825721	0.129090654	0.124719151
HIST1H2BO	0	0	0.365863691	0	0.240628688	0	0	0.33824151	0.125392413	0	0.124114997	0.239823978
HIST1H3A	0.114732539	0	0.242869003	0.115959848	0	0.353478413	0.235970865	0	0.124857691	0	0	0
HIST1H3B	0.456013228	0.118186699	0.120662672	0.576114074	0.238079655	0.117077243	0	0.446211257	0.124064104	0	0	0
HIST1H3C	0	0	0	0	0	0	0	0	0	0	0.252556443	0.366005875
HIST1H3D	0.292125738	0.181707237	0.185513946	0.118100258	0.183018888	0.30000249	0.120163231	0.17150791	0.127162339	0	0	0.121604558
HIST1H3E	0	0	0.123274418	0	0	0	0	0	0	0	0	0
HIST1H3F	0	0	0	0	0	0	0	0	0	0.126618829	0	0.120169311
HIST1H3G	0	0	0	0	0	0	0	0	0.125392413	0.37904309	0.124114997	0.239823978
HIST1H3H	1.478909707	1.061431491	1.444890858	0.22995843	0.118788158	0.700978333	1.520839709	0.33395092	0.247603624	0.748469865	0.1225406	0.35517272
HIST1H3I	0.112808304	0.233895688	0.358193593	0.114015029	0.117792031	0.347550054	0.348019924	0	0	0	0.121513006	0
HIST1H3J	0.112572303	0.116703183	0.357444234	0.227553007	0.235091208	0.462430616	0.11576395	0.11015257	0.122506814	0	0.121258794	0.117152508
HIST1H4A	0.144649357	0	0	0	0	0	0	0	0	0	0	0
HIST1H4B	0	0	0	0	0	0.1547912	0	0	0	0.16527836	0	0
HIST1H4C	0	0.429108628	0.146032773	1.255042352	0	0	0.993195321	0.810045051	0	0	0.297239506	0.43076076
HIST1H4D	0	0	0.15518469	0	0	0	0	0	0.319118567	0	0	0
HIST1H4E	0.428192792	0.443905478	0	0.432773225	0.298073202	0.293158931	0	0.41898882	0.310653884	0	0	0
HIST1H4F	0	0	0	0	0	0	0	0	0	0	0	0
HIST1H4G	0	0	0	0	0	0	0	0	0	0	0	0
HIST1H4H	0.431627494	0	0.456840492	0	0.150232082	0.295510474	0.147954994	0	0.46971864	0.631062827	0.309955635	0.149729676
HIST1H4I	0.436293737	0.452303689	0.153926436	0	0	0.448057772	0.299109016	0	0.15826556	0.159471282	0.313306506	0.151348375
HIST1H4J	0	0	0	0	0	0.155226007	0.155435865	0.147901484	0	0	0	0
HIST1H4K	0.760022046	0.157582265	0.160883563	0	0.15871977	0.15610299	0.156314034	0.297474171	0.496256416	0.166679024	0	0.474566939
HIST1H4L	0	0.153253082	0	0.149409804	0	0.151814447	0	0	0	0	0	0
HIST2H2AA3	0.201533936	0.208929295	0	0	0	0.413936019	0	0	0.328978972	0.220990166	0	0.209733704
HIST2H2AA4	0.100766968	0.104464647	0.106653149	0	0	0	0.10362391	0	0	0.331485249	0	0.104866852
HIST2H2AB	0.120110627	0.124518129	0	0	0	0	0	0.235057716	0	0.131706193	0.129378803	0
HIST2H2AC	0.36940202	1.148872072	0.781960384	0.995609494	1.414313009	1.390995525	1.013000785	1.325359752	2.948012941	1.620257419	3.18325146	1.922158999
HIST2H2BE	1.936466428	2.032619819	1.050411171	0.733942896	0.252752131	0.4225946	0.796547626	0.639509251	5.11034722	6.927639094	2.346627679	2.922119777
HIST2H2BF	0.252153519	0.261406381	0.160129657	0.050970167	0.052658668	0.310742972	0.311163081	0.098693399	0.713455803	0.22119728	0.434576972	0.367377968
HIST2H3A	0	0	0	0.107268577	0	0	0	0	0	0	0	0
HIST2H3C	0	0	0.112332902	0	0	0	0	0	0	0	0	0
HIST2H3D	0.11774521	0.122065912	0.124623154	0	0.122947043	0	0	0.115214285	0.12813623	0.516449666	0.253661723	0.490143535
HIST2H3PS2	0.020506692	0	0	0	0	0	0	0	10.89040756	6.273712059	7.20103483	5.996833298
HIST2H4A	0.135882729	0	0	0	0	0	0.139735272	0.13296194	0.591497546	0.149000945	0	0.282822721
HIST2H4B	0.135882729	0	0.431460464	0	0	0	0	0	0.295748773	0.447002836	0.292735877	0.282822721
HIST3H2A	0.976383161	0.562339936	0.459296623	0.438590069	0.113279836	0.22282443	0.557814192	0.636930585	4.014074073	2.974010804	3.272031658	1.806416092
HIST3H2BB	0.119047701	0	0	0	0	0	0	0	1.813751325	1.827569117	0.641169288	0.867239584
HIST3H3	0	0	0	0	0	0	0	0	0	0	0	0
HIST4H4	0.13060573	0	0.276469812	0	0	0	0	0.12779837	0	0	0	0.135919657
HIVEP1	3.422212404	2.553875178	3.039665861	1.685957608	1.643046365	1.417274465	3.032911886	1.522879518	0.95444906	0.801433656	0.89378466	0.612963338
HIVEP2	4.905575469	4.283668339	4.705042801	3.181663489	3.210382089	2.941258144	3.418485942	2.218024367	2.690557712	1.49779096	1.766642774	2.241790145
HIVEP3	0.540374915	0.618614076	0.547544706	0.339082247	0.302180203	0.42081164	0.308134532	0.205489511	5.192234206	3.900674693	6.002792212	4.701444841
HJURP	9.364787168	9.830880023	11.00122712	23.50039584	24.10270948	19.85841503	10.41113224	24.63410758	34.8154454	41.88959032	36.09656427	29.06183054
HJV	0	0	0	0	0	0	0	0	0.104896117	0.079271439	0.155741255	0.125389384
HK1	0.988782816	1.219829196	1.119799265	1.199231943	0.588505609	0.660038553	0.904966913	0.880451392	24.53377914	26.81327045	26.11578416	30.80865548
HK2	20.04699403	26.56683716	18.73488642	13.48229305	22.27528615	26.09350049	13.02959165	13.63413586	6.857437034	19.41801308	9.00690532	6.878567926
HK3	0.822090513	1.069195666	0.632808681	0.46831673	0.062429776	0.06140051	0.384271999	0.219387201	1.285028419	1.114527071	1.288037859	1.026646479
HKDC1	0	0	0.013356656	0.012754495	0	0.01295977	0	0	0.027466349	0.013837799	0.01359327	0.0262659
HKR1	8.548189268	7.862350109	7.753870479	7.833980973	7.974593598	8.365472918	7.994245472	7.881596635	4.535621209	5.028025128	4.677496401	4.384789624
HLA-A	133.6721679	169.9840183	135.2584365	155.2222255	75.29937818	112.8191485	122.6235564	168.3194171	108.9016376	150.5692011	105.5615385	166.9538865
HLA-B	198.8047835	262.126506	202.2866633	249.9152099	96.26043713	144.5141601	181.2561644	240.0921238	170.6586076	226.0552442	165.1782629	268.3497821
HLA-C	143.921915	178.5373277	142.058766	227.1763821	107.3430012	154.5271966	144.8623058	233.9503064	44.23252961	60.52970811	42.24699207	61.86006984
HLA-DMA	4.316274935	6.513119378	4.111564426	3.538428973	2.704177474	2.01932157	5.079788139	3.472653027	13.72622247	14.46185646	13.43141083	12.57729279
HLA-DMB	0.847394659	0.51910779	0.366911261	0.194649852	0.201098062	0.15822608	0.554539979	0.075379998	1.089845872	1.182621676	1.244703729	0.841787313
HLA-DOA	0	0	0	0.0156055	0	0	0	0	0.05040883	0.016930954	0	0
HLA-DOB	21.49244851	17.68665666	19.38553125	16.17284897	21.45909802	23.88444018	21.49682542	19.68728297	0.512171345	0.602085453	0.802676654	0.653048383
HLA-DPA1	0	0.031339394	0	0.183320793	0.031565617	0.279406813	0.093261519	0.177481781	10.16545024	15.48036114	12.24359583	6.354931216
HLA-DPB1	0.013269929	0.027513747	0.014045076	0.053647515	0.013856177	0.027255467	0.013646157	0.012984693	0.202174007	0.189163222	0.17152662	0.110478715
HLA-DQA1	0	0.03239496	0	0.03158256	0	0.064181717	0	0.030576613	0.034005956	0.171325129	0.033659526	0.065039371
HLA-DQA2	0.046709688	0	0	0	0	0	0	0	0	0	0	0
HLA-DQB1	2.522323165	4.592803289	2.464303037	3.922007351	0.675322099	1.560842279	1.39668092	2.373178861	3.448743505	6.772737681	4.946251154	5.148937213
HLA-DQB2	0	0	0	0.039697203	0	0	0	0	0	0	0	0
HLA-DRA	0.210193597	0.130744035	0.088988721	0.807279846	0.131687809	0.777100199	0.864611998	0.370215902	20.4038927	40.38111869	24.81485438	12.59975224
HLA-DRB1	0.088429845	0.275024429	0.140393052	0.134063686	0	0.090814229	0.409216525	0.302851683	61.44527056	81.79160191	66.58213781	50.15513536
HLA-DRB5	0.039134226	0	0	0	0	0.080378849	0.120731275	0	25.59528181	33.29992327	29.46562244	18.28618587
HLA-E	62.51733212	72.68138678	56.85825387	53.77587418	61.09440075	65.31511047	60.31762905	57.78713503	40.39547816	50.53320691	30.96501803	56.06860701
HLA-F	0.485844417	0.741254087	0.291070433	0.722664932	0.162721563	0.338905708	0.235669369	0.42158222	0.009975853	0.050259263	0.039496902	0
HLA-G	0	0	0.034706143	0	0.034239365	0	0.033720395	0.096257639	0	0	0	0
HLCS	3.182030519	3.01233362	3.477578959	2.740422521	3.236951089	3.086037003	3.125728812	2.741855932	2.715772493	2.173072922	2.413714531	2.682447453
HLF	0.009266327	0.009606358	0	0	0	0.01903236	0	0.009067148	0	0.02032181	0.009981351	0.019286688
HLTF	11.87315471	11.27062874	11.73096838	12.84689022	18.26975897	16.08961455	13.041592	10.57204466	15.41503152	21.78346891	16.58567719	15.0319163
HLX	33.59851036	24.74670778	31.28779385	14.67612053	20.7094826	17.67655739	22.0466575	17.30453853	26.18291529	16.85469403	26.50113875	21.5248071
HM13	25.52445492	24.86222678	27.16234683	25.82866849	21.24115431	21.3977872	24.83659298	28.23549123	24.25241658	22.93205263	27.70998043	27.86302333
HMBOX1	8.033137673	7.792257849	6.704111852	5.514085949	7.505748913	7.250906307	6.21989291	4.737692884	8.321993604	10.84168718	7.196103968	6.661414423
HMBS	14.84565231	13.64486009	15.8132447	15.24414598	17.73016651	17.85188018	16.75419142	18.14657997	18.8140015	19.3734063	23.19489948	17.22663349
HMCES	13.2415377	15.06812483	14.99697063	18.83874962	15.23560527	13.97390906	14.86012345	17.88108851	15.05259272	16.61617439	17.35217147	18.63704209
HMCN1	0	0	0	0	0	0	0	0	0.022466857	0.022638017	0.015884271	0.018415642
HMCN2	0.019302724	0.01667587	0.034050449	0.006503069	0.023514743	0.029734792	0.026466659	0.015739845	0.063018572	0.024693927	0.072772676	0.023436105
HMG20A	17.71898354	18.67555379	18.15451358	17.00003211	19.4146604	19.0692841	17.28432589	15.5067091	17.80867818	18.79498606	16.55290759	16.7099689
HMG20B	26.76337929	29.98429857	26.34032121	37.59930194	32.4557284	35.93381945	28.81764596	40.90475941	38.24612394	43.33001595	40.18271955	45.83187468
HMGA1	167.2719643	195.421941	183.2728722	179.7046919	204.8560844	221.1957019	185.9693053	219.1777625	109.8491409	142.8168727	162.1267701	86.80497564
HMGA2	5.334798099	3.174780928	5.233041766	2.027556599	7.933998619	4.968154061	6.033742324	2.275662689	0	0.155735729	0.019122964	0.027713081
HMGB1	83.54363138	100.0832772	108.0100233	198.3191007	179.4173915	179.119794	135.8466552	191.5957527	104.7621267	111.6390685	111.5329464	92.44510953
HMGB2	200.947622	199.5134467	242.6264283	445.1490207	387.3931906	322.1353773	270.0811461	445.9731697	349.082314	372.9804643	374.1387647	315.9720081
HMGB3	15.27655238	19.36540305	18.65224055	40.66509612	26.39729567	29.92061245	19.91217589	37.5912138	29.62986078	29.81318292	32.563535	27.9860333
HMGB4	0	0	0	0	0	0	0	0	0	0	0	0
HMGCL	12.61579878	14.36232984	13.45899061	11.16113535	12.54419198	12.61230615	13.00789394	11.95169082	21.63159496	25.57590107	18.38337616	24.27315453
HMGCLL1	0	0	0	0	0	0	0	0	0	0	0	0
HMGCR	22.38058964	32.36077101	22.53462242	34.09735208	24.85292582	31.94728119	22.49879369	27.32474779	58.96515383	95.10750751	86.45350002	27.82712801
HMGCS1	10.10404518	13.31651525	8.832637372	12.22069446	14.53367311	16.27239483	11.3771047	11.80164687	78.23787508	110.9703042	137.4201705	24.69117996
HMGCS2	0	0	0	0	0	0	0	0	0	0	0	0
HMGN1	64.58107974	73.62358753	69.98036462	92.16714357	85.92906294	82.48989229	76.27880651	89.00824192	73.62580234	88.85550504	81.20588609	63.18465354
HMGN2	161.6199812	186.9008892	197.2087674	456.1007055	313.5217656	282.2240586	213.8652248	433.7649316	429.9526269	388.4279839	415.534489	418.4417464
HMGN3	56.81118262	47.09394219	60.55591645	57.34490567	56.95124487	47.2533309	63.12726295	55.94821363	188.8205024	171.674632	169.5478406	162.8539405
HMGN4	48.2397993	48.590906	45.36239072	57.39066476	54.56033867	52.30353929	54.17021698	56.18621694	13.32970852	15.0098847	10.88053034	16.62774321
HMGN5	5.315149474	6.638467916	8.009822018	11.10214636	10.5449354	11.46277621	8.875490238	10.94665772	6.032106442	10.43539264	7.388345104	5.610425895
HMGXB3	24.68511116	24.23916054	23.35632929	20.47240438	20.16322407	20.78144881	22.10950104	21.89489139	25.08711124	24.27408963	26.48268739	26.02094596
HMGXB4	12.50110253	12.90411764	13.12894719	14.38393193	15.24204406	15.8848981	12.26968365	14.08355394	12.23570453	13.73154137	12.574193	12.3944826
HMHB1	0	0.14527115	0.148314535	0	0	0	0	0	0	0	0	0
HMMR	13.49950142	13.6929506	14.85015214	27.85919652	28.13811979	25.82691919	24.17059864	25.34582223	43.60641938	50.41952905	49.54701507	33.49950045
HMOX1	3.621146549	3.54351968	3.725213368	1.915452479	0.777427402	0.486570075	3.828219161	1.32460197	20.47697543	5.492231071	5.796170366	12.99597086
HMOX2	16.49457703	14.15563522	16.88079642	14.8271344	14.29709521	15.35549268	18.14204386	16.72894507	6.918102372	7.630764945	8.610179677	9.864888156
HMSD	0	0	0	0	0	0	0	0	0.091832081	0.37012676	0.151494259	0.234182536
HMX1	0	0	0	0	0	0	0	0	0	0	0	0
HMX2	7.638678359	10.12224761	10.46470681	11.92302208	16.434719	15.43623571	10.99101503	13.65299172	0.46927052	0.743043066	1.028513345	0.576977779
HMX3	31.06324742	31.21625432	35.05194455	42.38397218	50.69367586	46.82217625	29.52239404	38.87688283	3.107840459	3.516089347	4.101573071	3.024149099
HNF1A	0	0	0.01295854	0	0.012784255	0	0	0	0	0	0	0
HNF1B	0	0	0	0	0	0	0	0	0	0.011946624	0	0
HNF4A	0.009783557	0.010142568	0	0	0	0.010047356	0	0	0	0	0.010538492	0
HNF4G	0.044097161	0.068572983	0.011668261	0.033426656	0.149647282	0.124536989	0.170052759	0.151022536	6.298521811	6.539923483	5.462483852	6.367422425
HNMT	7.266446464	6.682756811	6.177211965	4.77212052	4.556873448	5.496887514	6.185600162	4.918526429	10.56268737	9.397804396	9.469608024	8.142110755
HNRNPA0	47.17177883	50.78291631	51.5396757	73.19176886	54.8801289	61.29496769	51.51187519	71.14445219	46.16372203	41.16584257	46.41624803	56.58399013
HNRNPA1	1109.250537	1180.02877	1114.540645	1278.06296	1417.404056	1368.730293	1225.75001	1302.319533	651.9898626	697.6737101	666.1797162	551.9147285
HNRNPA1L2	11.39898097	10.30767915	10.98117052	15.66016906	13.96948736	12.71107377	11.58177932	13.73651449	8.022357416	7.160361707	8.504317619	8.997708904
HNRNPA2B1	364.8282437	357.4204807	407.9522517	478.510495	519.3274166	468.9707502	457.3709102	467.0787741	448.2588903	427.7261693	456.0391923	384.6723713
HNRNPA3	119.4553728	112.4227678	130.2464036	152.4469631	162.5355464	151.3622956	141.2903985	146.6216398	118.9307114	127.2808647	125.4606515	103.072672

HNRNPAB	109.6043864	99.86463636	125.4152932	166.5695527	141.3333129	138.5163036	135.9916598	170.1362243	117.5679544	115.7668424	133.1267984	133.9224767
HNRNPC	199.0244175	203.1277645	221.9906761	223.4573026	233.1937374	229.5312981	241.747795	235.8156865	185.5588902	206.8524781	200.1525907	153.4862126
HNRNPCL1	0	0	0	0	0	0	0	0	0	0	0	0
HNRNPCL2	0	0	0	0	0	0	0	0	0	0	0	0
HNRNPCL3	0	0	0	0	0	0	0	0	0	0.05865246	0	0
HNRNPCL4	0	0	0	0	0	0	0	0	0	0	0	0
HNRNPD	164.5041958	167.5006614	190.0612976	224.504482	237.8167866	234.9732481	205.3555012	232.1441248	206.3938569	214.9986596	216.5667022	186.5554809
HNRNPDL	105.3315655	103.2814717	114.0156163	135.3455053	141.6205903	135.8819972	114.2657851	134.1250681	86.47951297	88.69184096	90.41465754	97.4434945
HNRNPF	169.238975	157.169155	185.3796972	206.6281265	190.3746828	189.007499	179.3588701	210.2366257	74.39279912	67.00823279	77.95324407	83.85706282
HNRNPH1	124.7986587	112.7412547	122.4742026	151.0189074	145.1055484	136.7559283	124.8298456	140.6729058	215.3813104	180.971213	188.2703939	211.8388671
HNRNPH2	0.044991272	0.046642242	0	0.045472549	0.023489464	0.046204397	0	0.066036281	0.024480877	0.049334761	0.024231481	0.023410911
HNRNPH3	53.58558766	51.19833022	57.62444088	72.09854817	74.27259437	63.98175883	62.83986807	66.56951916	74.25910087	78.46783602	75.33212057	64.77846952
HNRNPK	294.6729772	287.9241212	305.7170777	329.5484227	331.6210714	316.4547218	327.7831899	328.7617178	278.03942	239.5298781	273.4462086	265.5952683
HNRNPL	126.8327974	122.4524752	138.7341922	167.9960705	155.4599486	154.1699286	139.4291717	173.4146707	119.8144862	111.3360027	123.2267843	110.6100414
HNRNPLL	13.00771506	14.8479726	13.8002859	17.68026033	14.38989295	14.10499776	13.91729574	16.10310886	10.23380865	9.752088316	8.763396414	12.04000469
HNRNPM	117.1985945	114.0731992	140.7495487	162.739823	161.1523275	142.0404972	163.6616892	167.6677167	114.2445487	79.18612532	116.9386601	89.49123387
HNRNPR	33.91312755	32.22719054	38.19605121	47.99180822	52.547516	45.58653155	44.30058895	46.77201984	27.91652724	27.74552245	32.15496272	29.25965669
HNRNPU	306.0212449	257.3843196	304.5468037	281.9592155	350.2605909	316.3633001	294.9754041	266.8717441	218.9220028	186.9678488	216.8018325	189.305071
HNRNPUL1	70.97304533	76.47980342	73.36084332	94.66461302	92.24844807	94.80424623	76.56255901	101.5748687	101.9170597	109.7726499	112.0300372	101.2676651
HNRNPUL2	36.30864676	38.00950355	39.11561723	48.94348182	46.03453076	45.16820462	39.33774612	48.75536359	57.40916362	58.58021234	59.70719891	58.77327599
HOGA1	0.021645037	0.022439309	0.04581881	0	0	0	0	0	0	0	0	0
HOMER1	6.919401784	4.931651777	6.458999755	3.933798473	6.455753616	5.156765519	6.901454187	3.495044803	1.089415409	1.501674061	1.354366345	0.983460346
HOMER2	0.199872078	0.09117084	0.063464209	0.032321622	0.083480869	0.090314991	0.082215538	0.058672753	1.161507662	1.288707084	1.102310091	1.106582504
HOMER3	37.89838579	59.65665739	38.98613061	61.3386326	26.53814083	45.93707716	41.59862576	75.06975062	16.85222249	18.40646124	18.79426068	41.97457004
HOMEZ	5.465820136	6.188294708	5.419724711	5.495213507	3.950047592	4.859847866	4.92558431	4.644604744	5.901219704	5.693819604	5.67067189	7.364730882
HOOK1	0.007110143	0.007371052	0	0	0.00742426	0.014603715	0.029246917	0.006957311	0.030950453	0.054575928	0.015317575	0.073994317
HOOK2	5.50584476	6.612792065	4.633542976	3.895319039	4.711446054	5.17161766	4.736701418	4.69106449	4.398810925	4.491223902	4.180417359	4.597863169
HOOK3	9.487475204	8.290354928	8.720401625	7.091916376	8.202338747	7.661456929	8.066518586	5.163195741	8.78779384	9.827252099	8.088146321	7.418690684
HOPX	0	0	0	0	0	0.01311354	0	0.012494762	0	0	0	0
HORMAD1	0.326118551	0.234059252	0.371719785	0.076063173	0.078582935	0.051524903	0.051594562	0.098187279	0.354898528	0.247570801	0.216174186	0.234960415
HORMAD2	0	0	0	0	0	0	0	0	0.028775556	0.014497389	0.142412048	0.013758943
HOTS	0	0	0	0	0	0.014216737	0.014235958	0	0.03013031	0.166979192	0.014911681	0.014406714
HOXA1	3.293170457	2.757473258	2.859927818	3.072367312	2.53490853	2.059530625	2.843823849	2.933195693	3.308116523	3.24072672	3.570022548	3.602910714
HOXA10	23.16887743	24.30113431	23.83554752	24.66476336	40.4081644	35.22827366	28.4101212	27.21732468	23.80139192	17.48787758	24.14507375	17.09806907
HOXA11	11.60160905	11.43858357	12.42957421	14.73913917	19.37840963	16.76768532	16.31062996	17.08788967	18.165641	12.43250858	16.21092503	14.43771082
HOXA13	18.53583123	23.89797099	18.23667023	30.06976307	33.30084724	34.64219678	20.80021227	25.4049332	22.24468397	17.95479172	17.82195582	26.64068537
HOXA2	0.968451039	0.690242226	0.608606774	0.703520179	0.884831474	0.714842827	0.871419967	0.533044269	0.164674514	0.066371625	0.065198767	0.062990887
HOXA3	1.212132532	1.175540411	1.117397429	0.902357769	1.292901991	1.171197802	1.166079594	0.860862943	0.141839063	0.192941517	0.154433509	0.155985791
HOXA4	1.43243044	0.968474335	1.351310205	0.723876665	1.235589322	1.279177282	1.152815997	1.188347341	1.084412168	1.160965699	1.140450188	1.166643726
HOXA5	5.520594657	5.083525876	4.708829836	5.284255971	6.408753736	5.102504623	5.476745644	5.306601708	3.145253397	2.492641041	2.693452735	3.987851577
HOXA6	3.690181855	2.782250459	2.982564607	2.576853375	4.133442792	2.687229282	3.311830496	3.085645425	0.876183397	0.80928693	0.722714509	0.558592507
HOXA7	3.973054791	3.040759855	4.064024038	2.721953433	3.814465517	2.765761306	4.880901825	3.000538532	1.479916308	0.906410111	1.637173988	0.471744936
HOXA9	42.87499769	36.14984649	42.80456971	43.57158865	64.68173999	48.89355109	51.46143712	49.65808505	46.51637921	43.17043432	42.63298864	38.60303284
HOXB1	0	0	0	0	0	0	0	0	0	0	0	0
HOXB13	0	0	0	0	0	0	0	0	0	0	0	0
HOXB2	0.031953421	0.066251926	0.067639883	0	0	0	0	0	0	0	0	0
HOXB3	0.016089314	0.0285938	0.021894624	0.006969181	0	0.002360448	0	0.013494407	0.500262757	0.524236891	0.418415613	0.478398179
HOXB4	0.026468057	0.027439312	0.028014157	0	0	0	0	0	2.534740099	2.960376873	2.737001255	2.616770974
HOXB5	0	0	0	0	0	0	0	0	0.064314395	0	0.095488803	0.092255188
HOXB6	0	0	0	0	0	0	0.024825109	0	2.022873842	1.641216334	2.262300681	1.984707496
HOXB7	0	0	0	0	0	0	0	0	1.799156398	1.856026406	1.908029747	1.556662879
HOXB8	0	0	0	0	0.013727534	0	0.013519464	0	0	0	0	0
HOXB9	0.079688354	0.103265683	0.021085813	0	0.166417767	0.122755554	0.061460757	0.038987729	0.021680214	0.021845381	0.042918699	0
HOXC10	7.687080123	10.87221147	7.177212746	9.21218162	6.822682691	7.64060422	7.905023983	9.079943762	0	0	0	0
HOXC11	0.026416083	0	0	0	0	0	0.027165031	0	0	0	0	0
HOXC12	0	0	0	0	0	0	0	0	0	0	0	0
HOXC13	0	0	0	0	0	0	0	0	0	0	0	0
HOXC4	6.422283479	7.94198493	5.802094941	6.838714722	11.78342068	12.06025353	8.444156053	8.999925089	0.049921788	0	0	0.023869948
HOXC5	0.43367904	1.002938332	0.706172118	0.269734252	0.801175678	0.787966861	0.754726406	0.685499997	0	0	0	0
HOXC6	4.124440494	4.4249437	3.553203825	3.247599194	3.98115017	3.989391394	4.241376504	2.932983975	0	0	0	0
HOXC8	1.785819487	1.778271128	2.387540177	1.638679753	2.355429113	2.388989257	2.174744589	1.241597436	0	0	0	0
HOXC9	2.69757693	2.134221015	2.178932266	1.757831967	2.890877502	1.822574491	2.080543911	1.70184267	0.115880456	0.077842183	0.038233314	0.036938588
HOXD1	0	0	0	0	0	0	0	0	0	0	0	0
HOXD10	0	0	0	0	0	0	0	0	0	0	0	0
HOXD11	0	0	0	0	0	0	0	0	0	0	0	0
HOXD12	0	0	0	0	0	0	0	0	0	0	0	0
HOXD13	0	0	0	0	0	0	0	0	0	0.00928764	0	0
HOXD3	0	0	0	0	0	0	0	0	0	0	0	0
HOXD4	0	0	0	0	0	0	0	0	0	0	0	0
HOXD8	0	0	0	0	0	0	0	0	0	0	0	0
HOXD9	0	0	0	0	0	0	0	0	0	0	0	0
HP	0.297290391	0.231149676	0.157328125	0.262911727	0.038803038	0.0381633	0.30571916	0.181812598	0.485289423	0.203744387	0.160115203	0.928158544
HP1BP3	36.56217049	34.11344902	35.40216763	48.14889941	47.05885909	42.10882328	41.58175724	43.02517836	48.96173017	47.52955235	46.10543201	44.87614827
HPCA	0.026222983	0	0	0	0.027381481	0	0	0	0	0.115018274	0.028246444	0.054579823
HPCAL1	5.176513996	8.237632794	5.30834458	5.53725093	4.910955448	5.285063509	5.054007472	6.267501856	8.976083199	12.3464141	11.06512029	10.00916122
HPCAL4	0	0	0	0	0	0	0	0	0.304442048	0.294962879	0.220210432	0.403111449
HPD	0.221666574	0.258525796	0.11730748	0.196033049	0.08679732	0.028455437	0.113975629	0.162676401	0.060307165	0.030383303	0.119385589	0.086507053
HPDL	5.372002748	4.733760743	5.496275068	2.895716132	8.133529918	8.581768385	8.562679888	3.095513257	0.42221705	0.7526903	0.771536821	0.217411144
HPF1	13.18688251	15.73515933	14.84706552	22.85429371	21.99417446	22.81311694	17.38325175	22.42945466	24.0782307	24.55280709	23.26094686	26.89420799
HPGD	0	0	0.012600173	0.012032117	0	0.012225765	0	0.011648878	0	0.05221626	0	0
HPGDS	0	0	0	0	0	0	0	0	0	0	0	0
HPN	0	0	0	0	0	0	0	0	0	0.021597502	0	0.020497401
HPR	0	0	0	0	0	0.042970808	0	0	0	0	0	0
HPRT1	48.63775855	50.38311486	52.72660319	67.68359145	72.50678042	67.24983014	61.904997	66.75572679	49.37102521	59.62986242	57.01956996	42.1475811
HPS1	24.97075906	27.11336405	24.75173635	24.55641598	22.14617057	23.46959902	25.80037175	29.01756239	14.88092881	12.07585679	12.69066978	20.9272649
HPS3	12.28848976	11.92905486	11.93562881	12.5361241	12.9754175	11.58096771	12.04583125	10.40460558	9.432370396	10.56305612	9.138162209	9.678083563
HPS4	3.175293306	4.286794483	3.218368538	4.002509074	4.683013722	5.421033483	3.542041084	3.989124175	5.91048919	8.227564451	6.09182433	5.37679042
HPS5	13.35662573	11.7462257	12.83518095	11.14600495	10.65212443	10.22805041	13.32066143	9.67638117	10.7506729	9.583513031	10.39141072	10.61650162
HPS6	17.74184997	18.24675238	19.1192533	18.52189499	14.69856746	16.04869605	17.00231019	18.89751432	12.82057649	8.855072647	11.54027932	13.66440196
HPSE	1.881054564	2.659200716	1.930602756	3.456684445	2.071293	2.130805818	1.922662613	2.454161913	3.324917986	3.875287298	3.057725468	8.316785611
HPSE2	0	0	0	0	0	0	0	0	0	0	0	0
HPX	0.066308763	0.034370993	0	0	0	0	0	0.03244173	0.180401285	0.508971806	0.107138084	0.966093141
HR	0	0.019778097	0	0	0	0	0	0	0.373709848	0.104599139	0.318527356	0.198542453
HRAS	10.96837572	11.90596362	12.74722523	11.9116996	13.2943984	12.14758282	12.65056596	14.05761636	14.41722076	13.34791202	18.85724492	14.3242587
HRASLS	0	0	0	0	0	0	0	0	0	0	0	0
HRASLS2	0	0	0	0	0	0	0	0	0	0	0	0
HRASLS5	0	0	0	0	0	0	0	0	0	0.018415847	0	0.017477809
HRC	0.110947548	0.069011285	0.070457049	0	0.949962369	0.501331995	0.684558778	0.108562739	0.04829547	0.024331701	0.023901733	0.023092329
HRCT1	0	0	0	0	0	0	0	0	0	0	0	0
HRG	0	0	0	0	0	0	0	0	0	0	0	0
HRH1	0.810583681	0.703530699	0.718269444	0.562046759	0.511773258	0.387181353	0.988647245	0.581035993	1.477034335	1.27124506	1.401071134	1.942158341
HRH2	12.06026619	12.36074439	12.24561142	15.00330791	11.91521655	12.65212644	10.89642917	14.46896824	9.121272749	8.012256195	8.942884841	12.85872838
HRH3	0	0	0	0	0	0	0	0	0	0	0	0
HRH4	0	0	0.031053861	0	0	0	0	0	0.015964628	0.032172505	0.047405974	0.030533751
HRK	0.212161896	0.293263002	0.243268	0.214431419	0.212304316	0.19064722	0.118179264	0.233551678	0	0	0	0.009199753
HRNR	0	0	0	0	0	0	0	0	0	0	0.006017619	0
HS1BP3	2.733729071	2.881962846	2.243446165	2.429279834	1.654783614	2.014033157	2.064288997	2.54574227	2.615683589	3.062811431	3.250521361	3.126702537
HS2ST1	10.0434638	9.628807792	10.55174723	9.75414655	11.91794311	10.43597373	11.43263413	8.494044322	11.78419433	11.18362333	10.03658957	12.58722668
HS3ST1	0	0	0	0	0.004623286	0	0	0	0.033728939	0.063116668	0.033385331	0.032254776
HS3ST2	0	0	0	0	0	0	0	0	0	0	0	0
HS3ST3A1	0.004214738	0	0	0.004259824	0	0.004328382	0.004334234	0	0.073387022	0.184865276	0.036319702	0.114041777
HS3ST3B1	0.902976725	0.895411263	0.988965559	1.25388248	1.188834934	1.088597973	0.872055761	1.091013545	1.25610153	0.964320725	1.074148018	1.413659638
HS3ST4	0.352794498	0.748897044	0.497870083	0.271671151	0.122793503	0.31054894	0.19003648	0.361649836	0	0	0	0
HS3ST5	0	0	0	0	0	0	0	0	0.007070545	0.014248823	0.006998515	0
HS3ST6	0	0	0	0	0	0	0	0	0	0.01507136	0	0
HS6ST1	12.94860637	12.61918138	12.55209325	9.495386218	6.582613301	8.152036271	9.647249663	9.685900529	6.075122823	6.016893437	6.907885229	8.714403539
HS6ST2	4.458252981	3.318840986	4.945611127	1.404838101	3.73652247	2.89285995	5.443800522	1.909916609	0.442525988	0.632363451	0.41412594	0.32315935
HS6ST3	0	0	0	0	0	0	0	0	0.293111043	0.410796745	0.282212518	0.496895944
HSBP1	24.59169976	23.08211773	26.68677334	27.80780114	28.95498673	25.61595917	33.0786901	29.99284454	45.78083948	48.79949512	47.55832095	42.45668147
HSBP1L1	0	0.297515316	0.151874083	0	0	0.221041834	0	0	0.156155352	0	0.154564543	0.074665198
HSCB	4.327676095	3.694749633	4.176312763	3.698577403	4.086916754	4.117574086	4.515820915	3.207126917	2.493314316	2.477416073	2.810679894	2.318109355
HSD11B1	4.844922147	5.272062587	4.291461171	5.730238071	2.152750905	3.352326669	3.604206337	5.144251618	0.785263984	0.979638399	1.739591361	0.715183893
HSD11B1L	0.887528101	0.893807793	0.993050381	0.589471667	0.476608094	0.390625296	0.599768549	0.620321964	0.24836207	0.417090299	0.300461235	0.316676148
HSD11B2	0	0	0	0	0	0	0	0	0.062163755	0.06263734	0.030765235	0.089170221
HSD17B1	0.131082974	0.033973278	0.034685007	0.099363889	0.273748105	0.201926158	0.134799435	0.288597049	0.142651053	0.215606727	0.211796725	0.170520398
HSD17B10	69.39924672	68.75986755	77.59298969	76.74916316	55.01988693	60.82667298	75.79136701	80.20960109	58.55825706	57.53386035	69.21698775	56.28965117
HSD17B11	45.6719559	55.02515405	44.70390997	54.58438167	66.62442998	67.11644392	53.88736129	54.47139681	74.24733483	69.68753161	69.82095334	57.55198185
HSD17B12	16.69280167	17.36748762	16.8114587	14.29674162	15.94384511	16.12725162	15.57890691	11.80328803	59.38991151	60.20385063	70.29329419	40.21753275
HSD17B13	0.134692267	0.046544949	0.142560153	0.11344424	0.1406428	0.368864137	0.046170353	0.109830889	0.830613575	0.590782221	0.652885273	0.794310622
HSD17B14	0	0.133774872	0.045525804	0.173893425	0.044913508	0	0.044232748	0.042088672	0.982992325	1.320641472	1.019310536	0.58192301
HSD17B2	0	0	0	0	0	0	0	0	0	0	0	0
HSD17B3	0.051802225	0.067128907	0.027414094	0	0.013522695	0.013299749	0	0	0.014093443	0.014200812	0.027899737	0.094342309
HSD17B4	29.94507556	31.91732763	32.4696762	31.32052596	27.67266767	27.04715433	31.17076339	30.21539726	51.06407576	55.95173143	49.9914762	59.96342265
HSD17B6	0.255965142	0.383294705	0.4214265	0.172468822	0.475152631	0.379696597	0.46795068	0.16697546	1.392770385	1.153891042	1.378581747	0.947127253
HSD17B7	9.537429312	11.19509818	9.117655097	10.85215771	10.85828355	12.29063372	11.42138113	11.16880298	7.734109423	17.17165611	14.38272121	5.026773652
HSD17B8	7.679354414	9.530955466	7.326588952	9.291938354	12.36674261	14.05115178	7.00726749	10.79517324	1.647870551	3.024344816	2.097106866	1.125606007
HSD3B1	0	0	0	0	0	0	0	0	0	0	0	0
HSD3B2	0	0.089493243	0	0	0	0	0	0.028156646	0	0	0	0.029945935

HSD3B7	1.465279202	0.84131897	0.930523029	0.341758496	0.84739202	0.393560032	0.857729875	0.330873031	2.649472879	2.397747931	2.185401479	2.557134467
HSDL1	5.373976895	6.670941661	5.628796284	6.955094194	5.086690719	5.604886978	5.856484694	6.091622836	5.225432018	6.612163351	5.758581712	6.609467956
HSDL2	13.28072398	13.10473857	13.3957963	13.26505997	12.85712997	12.27654039	13.38443446	12.84255009	21.9933129	20.92875575	24.76148187	18.92074163
HSF1	29.17414017	29.95364795	29.88782914	26.81425269	30.04772275	27.63022928	28.7742873	33.88101475	35.36409524	37.81410773	38.88474293	37.714041
HSF2	16.32005745	15.62342126	17.43130187	17.24867566	20.19608672	18.5797729	17.58833586	16.44377415	14.89835726	17.3843044	13.94295402	15.00421102
HSF2BP	0.12918888	0.133929511	0.116478212	0.1450787	0.124903963	0.147413637	0.09348819	0.0936385	0.026035149	0.026233494	0.072155775	0.059753404
HSF4	9.310821646	8.182239393	8.484180561	7.831630467	8.92807801	8.337616934	6.425474039	8.245874948	5.524785903	4.868198953	4.405797948	9.561309313
HSF5	0	0.013500513	0	0	0.013597967	0	0	0	0	0	0	0
HSFX1	0	0	0	0	0	0	0	0	0	0	0	0
HSFX2	0.109703488	0.113729096	0.029027921	0.0554385	0.028637512	0.112661485	0	0.05367271	0.149231032	0.060147171	0.088626458	0.057083485
HSFX3	0	0	0	0	0	0	0	0	0	0	0	0
HSFX4	0	0	0	0	0	0	0	0	0	0	0	0
HSFY1	0	0	0	0	0	0	0	0	0	0	0	0
HSFY2	0	0	0	0	0	0	0	0	0	0	0	0
HSH2D	4.572715896	3.008739082	3.286080828	1.330211085	3.682358393	3.396378137	4.39002743	2.047338699	4.462106136	4.773637059	6.325077728	2.405722528
HSP90AA1	237.9428667	223.4788047	281.506666	398.877967	297.1666544	287.0044288	326.5915836	362.1988994	370.2940153	379.024172	405.3620178	398.0402829
HSP90AB1	1046.748591	1025.85483	1197.002548	1209.563392	1156.195684	1177.869533	1300.311432	1232.368388	731.2017611	1056.295161	923.9239543	669.0355181
HSP90B1	279.8209973	291.7069061	314.593473	386.9290018	263.3240846	274.4680009	328.4534703	352.2153434	241.2510164	391.2389979	273.465019	256.2213768
HSPA12A	0.005688114	0.005896842	0	0	0	0	0	0	0.006190091	0.006237249	0.01838109	0.065114999
HSPA12B	0	0.016076116	0	0	0	0	0	0	0.016875578	0.017004142	0	0
HSPA13	11.84971017	12.52239501	12.61331976	14.25445227	14.8112178	14.28982513	12.39385626	11.39690924	16.37633725	16.91547526	16.29673183	19.15788764
HSPA14	13.96502419	12.79498547	15.10992157	16.38332332	18.20464453	15.88850461	16.73946292	15.4525932	16.19156431	15.28124848	16.90593307	15.92889031
HSPA1A	0.443059373	0.436351714	0.445493143	0.738868079	0.48576483	0.546007001	0.410058881	1.018809235	1.374154241	0.777332227	0.954494914	2.282375869
HSPA1B	5.63719209	6.685239981	6.305486501	12.68987267	6.242977626	7.74084995	5.906809587	12.01406103	13.52416889	9.014557192	8.786258253	19.84405423
HSPA1L	0.212099176	0.197894007	0.179590954	0.257241633	0.177175557	0.174254501	0.109056302	0.311310179	0.877104363	0.581438454	0.73108968	0.595967784
HSPA2	0.019425834	0.060416016	0.143923996	0.078534539	0.020284043	0.09974812	0.119859569	0.038016555	1.01472792	0.830747509	1.653059418	0.58627006
HSPA4	69.91268794	60.0029504	76.7726857	69.42343088	72.37058849	65.39834476	81.45794432	63.63460259	68.28048468	70.61350679	72.37935639	69.89522824
HSPA4L	5.553849847	4.526013823	5.009309431	6.189229383	5.224333457	4.781105911	5.055753087	5.226542697	9.460138459	11.52338167	8.198496227	11.06709525
HSPA5	89.91753823	79.05326667	110.3621528	123.5378968	64.791729	66.17335517	101.4013467	111.2740727	111.9834478	136.4977768	113.251621	133.4804987
HSPA6	0.981260016	1.324813747	0.70043709	0.761115591	0.571875813	1.101459522	0.797877739	0.669884584	0.69534826	0.725668726	0.688264505	0.617460293
HSPA8	1086.388997	869.8948945	1151.066233	1318.33596	1123.418789	1052.622606	1288.974723	1335.792053	830.8897209	878.4276231	821.700636	840.9092708
HSPA9	145.5129625	135.800764	161.176696	158.6880419	161.0602756	157.2543055	166.0370696	156.7122952	100.6146436	99.109173	126.1568057	87.54419981
HSPB1	100.6913647	105.9960226	113.0838244	78.40880578	118.5472855	108.4356168	120.0054584	109.4549775	248.5963743	259.8550027	302.5459404	213.3676134
HSPB11	14.90298848	18.42585632	16.48463023	27.7790305	17.98487765	16.49659546	18.96846844	24.80245772	66.93321778	64.76416571	64.40920304	64.64345076
HSPB2	0	0.066330704	0	0	0	0	0	0	0	0	0	0
HSPB3	0	0	0	0	0	0	0	0	0	0	0	0
HSPB6	0.110567591	0.15283321	0.078017509	0.037250115	0.307872869	0.416345921	0.0758016	0.288509196	0.040108395	0.363725595	0.198498985	0.11506623
HSPB7	0	0.016562982	0	0	0	0	0	0	1.877758837	1.156261493	2.082353368	0.847964323
HSPB8	0	0	0	0	0.028065334	0	0	0	0	0	0	0
HSPB9	0	0	0	0	0.182424671	0	0	0	0.095062106	0	0	0.090907303
HSPBAP1	2.525317753	2.606297745	2.672831136	2.518148389	2.495621477	3.392272022	2.515761875	2.559287528	1.791222613	1.434005327	2.015848064	1.818526989
HSPBP1	8.826150668	9.818961035	10.58565614	12.53071551	11.93518292	12.59039142	12.53631855	16.30324076	8.025114458	7.555592261	10.17742976	7.890208873
HSPD1	322.2388645	290.3934512	361.0282631	356.7184827	407.2389641	361.847116	419.9203257	356.5312203	259.3294241	299.6966847	343.3683684	181.6516004
HSPE1	13.15595769	13.9313966	19.72131986	15.75058398	15.44694779	15.77213508	22.99341708	17.34839401	9.769950549	10.95883973	13.68455753	7.815166365
HSPE1-MOB4	0	0	0	0	0	0	0	0	0	0	0	0
HSPG2	0.316987749	0.339696796	0.188485509	0.046796875	0.152479398	0.149965502	0.172144089	0.059246742	5.29458427	6.975232498	5.635805049	6.134377653
HSPH1	55.35986251	42.87789483	65.24658602	84.86821229	73.82672598	60.23939983	80.85292209	78.92882655	40.10223227	40.74773888	45.49290343	41.28293188
HTATIP2	1.284343428	0.987126433	1.101555852	0.425233829	3.283343788	2.637947816	3.871184584	1.950108456	17.35059469	17.31280614	18.12794024	15.87787708
HTATSF1	27.41112239	29.25330018	28.35830674	43.45609166	39.12465117	38.42673036	30.81824661	40.12371476	33.97686431	34.74388359	34.5736478	36.88603703
HTD2	0	0.031876641	0	0.01553862	0	0	0.015810048	0	0	0.016858393	0	0
HTN1	0	0	0	0	0	0	0	0	0	0	0	0
HTN3	0	0	0	0	0	0	0	0	0	0	0	0
HTR1A	0	0	0	0	0	0	0	0	0	0	0	0
HTR1B	0	0	0	0	0	0	0	0	1.106612732	1.468140338	0.876271425	1.410995876
HTR1D	0	0	0	0	0	0	0	0	0	0	0	0
HTR1E	0	0	0	0	0	0	0	0	0	0	0	0
HTR1F	0	0	0	0	0	0	0	0	0	0	0	0
HTR2A	0	0	0	0	0	0	0	0	0	0.021684812	0	0
HTR2B	0	0	0	0	0	0	0	0	0.088724632	0.071520454	0.017564153	0.016969363
HTR2C	0	0	0	0	0	0	0	0	0	0	0	0
HTR3A	0	0	0	0	0	0	0	0	0	0.022409561	0	0
HTR3B	0	0	0	0.01095371	0	0	0	0	0	0	0	0
HTR3C	0	0	0	0	0	0	0	0	0	0	0	0
HTR3D	0	0	0	0	0	0	0	0	0	0.028532096	0	0
HTR3E	0	0	0	0	0	0	0	0	0	0	0	0
HTR4	0.027176546	0.014086899	0.014382015	0	0	0.013954661	0.013973527	0	0.014787439	0.014900095	0.043910382	0.028282272
HTR5A	0	0	0	0	0	0	0	0	0	0	0	0
HTR6	0	0	0	0	0	0	0	0	0.155556173	0.156741254	0.20529529	0.148757405
HTR7	3.797400803	2.749635682	3.791327384	0.682534855	1.062827766	1.346835476	2.556408265	0.660795208	1.267448634	1.459548001	1.349417619	1.497242293
HTRA1	0.177673078	0.15787959	0.134322597	0.076960144	0.053006414	0.13033127	0.156608966	0.024836287	0	0	0	0.026414575
HTRA2	11.76302028	11.45952888	12.00864846	11.27754465	13.87247703	14.22207151	11.06703358	14.26527012	10.84916546	10.33720047	10.82850433	11.67728976
HTRA3	0	0	0	0.031665309	0	0	0	0.030656727	0.630758519	0.858870078	1.012431505	0.228234231
HTRA4	0.019966442	0.020699118	0	0.020180025	0.020848534	0	0.02053253	0.039074529	0	0	0.043014251	0
HTT	10.25077126	9.885898524	9.695709114	10.65101743	9.156676051	9.530634932	8.718112164	9.674853472	16.20510134	16.06145047	15.79053166	18.26042015
HUNK	0.342457598	0.861122529	0.470429202	1.502312037	0.578225687	1.391800311	0.359659859	1.340385988	0.150657669	0.119846393	0.156971438	0.204735311
HUS1	6.474646348	5.206518025	5.926793501	6.933003607	5.408550372	5.193584561	5.41654814	6.215613978	7.464987567	6.98458285	7.238144457	7.448308821
HUS1B	0.052445966	0.163111467	0.055509533	0.106013974	0.32857777	0.323160576	0.377530385	0.256593218	0.057074325	0	0.056492889	0.054579823
HUWE1	43.19581375	34.30937641	39.11752853	28.84991364	29.86513554	28.65584701	31.3375494	24.1921874	35.58146676	34.46842774	32.88639951	31.54738071
HVCN1	19.76137806	20.52872557	16.71533975	10.90171685	14.3229585	15.25723705	16.09407871	11.82898617	10.8966197	10.62257269	12.91204367	7.306966755
HYAL1	0.198334077	0.173979399	0.161476556	0.185036015	0.12744383	0.203681872	0.125512147	0.044785593	0.066411406	0.100376027	0.131469699	0.111140429
HYAL2	4.717389657	3.835285195	4.972232635	3.640185894	3.822092159	3.618363966	4.710232551	4.864985011	3.237851973	4.357180063	4.090422157	5.622297592
HYAL3	13.18835236	13.13888724	12.12432133	11.16716094	5.655440225	6.118420173	7.435576157	11.12945641	26.72991402	19.89578803	27.47857315	24.43434589
HYAL4	0	0	0	0	0	0	0	0	0	0	0	0
HYDIN	0.002382219	0.002469635	0	0	0	0.002446452	0	0	0.00259245	0.0026122	0.007698119	0.007437431
HYI	3.898856519	4.401823995	3.222142466	7.098411584	6.524918551	6.883560844	4.393859484	6.924578663	18.10510876	17.1595848	17.25906809	16.84135618
HYKK	0.062802942	0.013021504	0.0398829	0.025389901	0	0.025798533	0.012916706	0	0	0.027546393	0.040589428	0.026143277
HYLS1	3.75812806	2.257928735	3.141443096	3.949399148	3.790379273	3.113882983	4.216012791	4.429532065	20.47215257	15.71108539	18.14753341	19.82183245
HYOU1	31.51718902	28.03766568	36.057806	43.66485138	24.6029895	26.05515044	32.38765412	42.39500765	50.54914068	78.64375057	60.11605389	69.24733325
HYPK	22.62311663	17.96157878	25.92595152	26.93092361	25.49408406	22.16962883	29.53120358	27.98282941	13.04665831	12.75298098	14.24373473	13.30543784
HYPM	0	0	0	0	0	0	0	0	0	0	0	0
IAH1	4.352243893	5.195579962	4.857737229	3.998909456	3.855956769	4.442507453	5.669142199	4.852328689	11.74035644	13.56522136	12.61519433	12.10564431
IAPP	0	0	0	0	0	0	0	0	0	0	0	0
IARS	73.61129654	70.00244304	77.16183273	77.78048625	93.83147675	94.39546498	78.56372519	75.10193787	49.56076595	54.45883362	71.935493	50.84110552
IARS2	77.93213379	70.35495315	81.63392462	73.49429941	88.89514633	78.81894654	89.18488596	68.14170183	64.0692342	68.43110871	65.70299629	56.07779275
IBA57	2.797960679	2.15778784	2.46301819	2.952042126	2.144860671	2.193598419	2.091297404	2.864693247	3.698416236	2.207519395	2.484598077	2.826577265
IBSP	0	0	0	0	0	0	0	0	0	0	0	0
IBTK	21.94049323	19.29355254	20.25131297	20.07837344	21.23502368	20.24038503	20.8683413	16.25732729	17.7107186	21.58300296	17.47395602	16.96386535
ICA1	0.251120585	0.260335543	0.26578949	0.112803046	0.203944823	0.200582427	0.272587034	0.382235414	5.465639757	5.828536849	4.192718517	7.21582907
ICA1L	0.870899244	0.946443409	0.915414723	1.195878805	1.078706258	1.171948558	0.877061484	1.14603427	0.830103655	0.915460211	0.672844869	1.337622988
ICAM1	34.08270398	29.79965096	31.26612948	16.57021927	13.24180559	13.22778075	22.36590889	15.73423921	2.760139658	2.581214158	2.285612467	3.139802707
ICAM2	9.398124221	7.581952194	8.077347841	3.46379603	6.566809032	5.442592768	9.192250475	4.528912417	2.845246896	2.092078933	3.23489482	1.654596486
ICAM3	2.047204933	3.563174265	1.649940959	2.790443206	2.078813492	2.546031599	3.669696997	4.024778814	4.435302542	5.601811456	4.673352023	9.069280649
ICAM4	20.04827156	20.11886356	17.01793242	15.64282793	17.50080613	17.08874091	15.05017606	15.30152164	2.356293503	3.033756952	1.598049952	3.129595688
ICAM5	19.02725463	23.49466387	17.4266715	12.77386521	26.52060283	26.58120513	16.25515067	15.33169245	0.207252519	0.645478999	0.279737952	0.234229628
ICE1	18.40150095	16.34667358	17.79300097	21.30607332	22.06633569	19.935269	18.3668516	16.75581377	37.33399236	33.26545829	30.34651523	44.45315374
ICE2	7.010304112	6.620283552	6.758976387	8.740374679	9.653263724	8.847297845	7.490663947	7.695634104	9.869971877	10.24544149	9.315168456	10.41892998
ICK	3.710238094	3.976902703	4.028864518	5.127145677	6.023880558	5.734432392	4.546531135	4.717458896	7.801320236	5.432690124	6.525278502	6.805261172
ICMT	17.80398442	18.27410883	20.79618184	27.87351519	22.46549338	22.36732847	21.12549513	26.75875421	26.57477553	27.38277718	31.77662366	33.43612412
ICOS	0	0	0	0	0	0	0	0	0	0	0	0
ICOSLG	2.14096396	1.895846311	1.756169981	1.595851267	1.853642727	1.401666141	1.825546818	2.243335971	1.067872725	0.743423815	0.759113824	1.318276465
ID1	6.218557741	7.334822605	6.178839481	8.43354914	5.565673449	5.082919538	5.122418253	9.406743681	12.15360052	8.210514355	11.58550563	25.25893727
ID2	73.49502338	85.76195249	75.44990896	118.1800878	115.3394693	94.68012586	83.12445673	121.7888481	65.77071101	69.3863757	59.95891779	111.7514682
ID3	0.173719325	0.360188034	0.459667323	0.087788811	1.043015632	0.71361367	0.17864461	0.254977861	23.15863274	16.47741205	21.33215244	32.22535502
ID4	0	0.014392188	0.0146937	0	0	0	0	0	0	0	0	0
IDE	15.35548214	12.99721842	16.7214227	17.01004801	15.03269909	13.56894899	17.11296836	14.92591111	8.426444658	7.12863324	7.620774752	8.829200033
IDH1	44.90902221	60.89917061	44.29660768	74.43004376	35.59911513	47.6499747	43.47621065	61.4946822	38.23868926	42.90069936	42.22596606	51.08565493
IDH2	75.9766659	92.22253829	86.85529168	126.8987267	107.3812323	113.6454988	85.16311782	132.6955867	139.4739747	135.2302731	146.7926184	127.1962064
IDH3A	27.29895609	22.76980504	27.05014553	32.37168298	24.86768362	27.62081524	28.03522816	30.30010083	21.10890493	22.6367719	23.75738654	24.68912269
IDH3B	41.13045667	41.85975781	48.87144413	44.27420251	43.15122857	42.66874351	49.80451671	49.65353831	20.13603454	20.35055066	20.77136144	20.06796375
IDH3G	60.44526904	59.34880212	60.2728986	57.67754428	52.50190208	55.66314128	55.11804558	59.4116282	25.86541848	25.30176366	26.83652871	29.91420997
IDI1	15.92979457	20.12576106	15.16891331	20.66362943	15.60057718	20.08445014	16.40752071	17.68886876	36.66792181	55.13812292	50.93963051	17.22027735
IDI2	0	0	0	0	0	0	0.040717563	0	0	0.086834988	0	0
IDNK	0.897876162	0.857337936	0.716911474	0.628868314	0.583908475	0.509573901	0.550759868	0.462408621	2.485640303	2.081389669	1.798577455	2.999941009
IDO1	0.027866163	0	0	0	0	0	0	0	0	0.183338294	0.030016418	0
IDO2	0	0	0	0	0	0	0	0	0.025526703	0.025721175	0	0.073233085
IDS	12.16210461	13.91567026	10.27615173	11.80469468	6.648303488	7.961072594	8.035610241	7.929292318	11.1282435	12.58064839	9.753064235	14.80109197
IDUA	7.509594948	6.225267557	6.910894191	5.901725582	4.208964905	3.983629774	4.556846816	6.389131631	3.87584154	3.239337124	3.078007352	7.54218109
IER2	111.477661	171.1620124	120.4620779	168.4879053	116.0462953	115.4269676	85.32412959	152.5137017	36.07759935	32.63949293	29.46150986	21.0064162
IER3	8.635566622	10.12211886	9.645229092	18.85937298	3.896826357	2.763022928	4.908764892	10.5730477	0.094448802	0.380673383	0.233716547	0.090320805
IER3IP1	28.08544234	29.30150009	30.25616507	40.06566942	41.0567099	37.51391502	35.90899192	38.78952432	38.31205116	39.27086351	40.42674677	38.35030971
IER5	21.39056412	23.72373408	18.65191429	24.19698791	16.36182607	16.58757574	22.96574858	22.05487732	19.70277821	15.82183907	20.17027689	17.98085906
IER5L	0.933228546	1.976116488	1.176884042	1.946627806	1.948914786	2.181870504	1.26597063	2.195491107	3.284448367	4.289247877	3.871242938	6.03382969
IFFO1	5.995962522	5.44624984	5.412071121	6.371602674	5.134487456	5.452672168	6.237992109	6.922605781	7.9734362	6.375114646	6.669896646	10.61369392
IFFO2	0.653557419	0.695607671	0.608726084	0.792658328	0.864412287	0.948600584	0.842349114	0.929420111	5.149333374	5.608998825	4.33656795	6.520357614
IFI16	14.42231177	8.623368342	8.870975928	3.65480267	9.169984965	5.196908329	9.486338537	3.662184474	12.37938075	6.369790413	8.165059744	6.297681805
IFI27	0.8448361	0	0.137567105	0.131365142	0.135716905	0.200219053	0.066829913	0.127180986	2.970346373	1.28270379	0.770022633	1.284998886
IFI27L1	2.141812451	2.840390792	2.72325266	2.698876291	2.889938725	3.027970332	2.102421731	3.415840013	5.327605708	6.71024159	6.501778079	4.892124013
IFI27L2	7.604777338	8.36164565	7.317273829	5.82282319	2.285972535	2.839937913	5.806045452	3.946150945	40.62714194	40.93665372	39.22033909	50.48294735

IFI30	8.479716789	10.85294196	9.4182615	9.046560146	3.552667229	5.698062848	6.674670055	6.965757055	10.59517102	11.76643652	6.935106566	16.99577474
IFI35	15.89327412	13.62258906	12.47056125	10.05346568	8.393525853	9.536763995	12.98451415	10.09240986	23.32743665	13.64425846	17.15919467	23.5684315
IFI44	2.450530286	0.571602006	0.626804804	0.103197663	0.383818738	0.272632243	0.420001274	0.09991068	8.222601561	2.19447009	1.715754416	2.018935632
IFI44L	0.508751546	0.037672883	0.048077648	0	0.037944824	0.009329809	0.009342422	0	1.838905253	0.328742926	0.283790209	0.576723422
IFI6	11.01122288	4.576882572	2.418843994	3.674673554	1.301624679	1.706886751	2.991090156	3.659276872	24.19202126	10.82126557	10.01461868	16.32400333
IFIH1	6.442200203	4.757145934	3.891773389	3.625677907	2.762517599	2.394619871	3.781236472	2.881285244	6.002221349	4.785910364	3.960716417	4.775461651
IFIT1	2.833879157	0.589933605	0.361375516	0.057513926	0.427818264	0.280509942	0.374518903	0.100227655	4.235813011	1.410214531	1.630479397	1.587109231
IFIT1B	0	0	0	0	0	0	0	0	0	0.029921082	0	0.028397008
IFIT2	6.358611913	2.074945871	1.303640202	0.700238222	1.736244421	0.80637579	1.820756597	0.331434742	12.86773717	4.05180253	5.356689637	5.784149494
IFIT3	8.851378979	2.515099534	1.865659972	0.938666172	1.523911058	1.206815228	2.338929252	1.020046163	15.8204238	4.634721902	7.656089775	9.093163918
IFIT5	1.347239873	0.71908139	0.720027726	0.606676397	0.738200379	0.589043064	0.960203706	0.743980395	3.512914777	2.530430531	2.629398059	2.748582541
IFITM1	18.08661669	10.89229712	6.592860318	3.640848107	8.620010948	9.171540543	9.338291929	6.315413997	53.08628604	32.09442956	34.51833677	21.00934977
IFITM10	0.391711551	0.255683491	0.353171736	0.777140452	0.121190183	0.119192146	0.223787414	0.298115798	2.747138509	2.497623827	1.593985919	5.133358211
IFITM2	53.3672905	59.29928553	53.52001092	55.3536716	55.57105289	61.54349701	62.38471666	67.00626084	93.77342809	94.64948269	96.94690434	84.535324
IFITM3	7.169237899	6.34669648	5.371295243	4.477820768	3.700926856	5.211689955	4.97022466	6.305739919	24.37005309	17.31266074	15.35811609	16.34698394
IFITM5	0	0	0	0	0	0	0	0	0	0	0	0
IFNA1	0.187055252	0	0	0	0	0	0	0	0	0	0	0
IFNA10	0	0	0	0	0	0	0	0	0	0	0	0
IFNA13	0	0	0	0	0	0	0	0	0	0	0	0
IFNA14	0	0	0	0	0	0	0	0	0	0	0	0
IFNA16	0	0	0	0	0	0	0	0	0	0	0	0
IFNA17	0	0	0	0	0	0	0	0	0	0	0	0
IFNA2	0	0	0	0	0	0	0	0	0	0	0	0
IFNA21	0	0	0	0	0	0	0	0	0	0	0	0
IFNA4	0	0	0	0	0	0	0	0	0	0	0	0
IFNA5	0.076870801	0	0	0	0	0	0	0	0	0	0	0
IFNA6	0	0	0	0	0	0	0	0	0	0	0	0
IFNA7	0	0	0	0	0	0	0	0	0	0	0	0
IFNA8	0	0	0	0	0	0	0	0	0	0	0	0
IFNAR1	10.60095903	10.70175333	10.08191995	10.57386691	8.87682665	9.369108339	9.441962704	8.740887217	12.54762925	11.85703131	12.99508489	13.1565054
IFNAR2	23.56188485	23.68576524	21.94923819	21.57868688	19.96647539	19.49752506	21.85065592	18.89379502	27.6406822	27.31027633	26.1679775	28.78729355
IFNB1	0	0	0	0	0	0	0.064418123	0	0.136340529	0.068689609	0	0
IFNE	0	0.037819744	0	0	0	0	0	0	0	0	0	0
IFNG	0	0	0	0	0	0	0.045731544	0	0	0	0	0
IFNGR1	25.85691764	26.19141679	24.36899829	22.43771662	20.35884807	20.58722966	24.88076042	19.43194293	29.30272171	31.83985712	26.54437409	34.62515851
IFNGR2	27.66279337	29.12416232	28.5853438	19.7678265	18.89830757	19.10570289	26.46464551	20.65826197	24.48244347	25.16151755	25.92652205	25.02907305
IFNK	0	0	0	0	0	0	0	0	0	0	0	0
IFNL1	0	0	0	0	0.065638783	0	0	0	0	0.068930344	0	0
IFNL2	0	0	0	0	0	0	0	0	0	0	0	0
IFNL3	0	0	0	0.163318825	0	0	0	0	0	0	0	0
IFNLR1	0	0	0	0	0	0	0	0	0	0	0	0
IFNW1	0	0	0	0	0	0	0	0	0	0	0	0
IFRD1	8.043299132	7.823731647	8.057703322	8.374669186	8.997721905	9.155310749	10.12076476	8.172701813	8.536994212	7.839826651	8.366831941	7.704585015
IFRD2	28.14222173	24.46672004	31.02832893	27.64386	31.30369293	33.35541193	28.96634677	31.20728326	14.02177327	16.7304959	19.40301697	13.96653428
IFT122	4.290848603	4.384183128	4.181447018	5.899540559	5.392641018	5.073481756	3.633358006	6.294143156	12.21646397	11.26678355	10.91778642	11.37678778
IFT140	1.480846969	1.475815292	1.50240346	1.608319187	1.541997438	1.751832995	1.320909435	1.625139798	2.350521494	2.278715385	1.947626047	2.720335743
IFT172	5.221582484	5.369710857	5.570985233	6.859963459	6.123762542	7.063690729	5.671531236	6.682503374	4.952120726	5.235124406	4.638141005	5.041210301
IFT20	9.426492414	9.184247124	9.930939157	8.777492742	8.658144152	8.29130968	11.17474193	10.33415138	11.87312593	12.63354001	9.683788284	13.12545155
IFT22	1.840909082	2.018143608	2.082818978	2.224167336	1.4361549	2.498998323	2.415337646	2.815886061	3.40803069	2.969940982	3.054214821	4.690430772
IFT27	0	0	0	0	0.222610137	0.109470005	0.306930408	0.229469973	5.12732758	5.376785301	4.661737656	9.384918466
IFT43	1.845894321	2.563542162	2.248621139	1.513632524	1.309206958	1.287622336	1.969860345	1.806216959	4.434508787	4.162768158	3.75156658	5.436786295
IFT46	0.300192808	0.359834816	0.387231254	0.445624638	0.440796014	0.510600471	0.347291849	0.688453561	2.6338965	2.283642103	2.253392593	2.74332123
IFT52	11.24206613	13.30586515	12.0697872	15.95858229	11.49768466	12.99130472	12.15427111	17.00886102	12.53563718	15.18774114	12.08467953	17.44109848
IFT57	6.182303589	7.785046019	6.205594966	7.352100532	5.33274642	6.60779133	6.063891329	6.213801719	2.559524193	3.168514639	2.859178638	4.598098781
IFT74	1.126838361	0.986051239	0.942587126	0.851107682	0.955213532	1.076340839	1.052875858	0.89513535	3.355792935	3.494291928	3.119308078	3.637847853
IFT80	7.408074712	6.702472694	6.569647071	7.612108496	7.993199138	6.201528308	6.567732691	6.589853357	12.6057825	9.514055355	10.57915951	14.50784989
IFT81	6.115343294	7.65581173	6.241400691	6.180759902	10.41921608	8.747469849	7.509973317	6.18424805	6.41734324	7.603810799	6.49900381	6.73350534
IFT88	2.498172665	2.869323561	2.387299283	3.060755147	3.040167461	3.156158455	2.56900078	2.734646077	2.924001146	2.581687722	2.78773725	3.226389462
IGBP1	41.90325631	42.78180762	36.45956784	30.5222885	42.42676631	38.84851786	40.89215414	33.70692297	33.39896294	33.17807731	29.75907019	31.03698367
IGDCC3	0.011113086	0.011520884	0.011762243	0	0	0	0	0	0	0.036557853	0	0
IGDCC4	0.007480823	0.031021338	0.015835613	0.007560846	0	0.007682533	0.007692919	0	0.065128049	0.024609081	0.064464567	0.06228155
IGF1	0	0	0	0.004345252	0	0	0	0	0	0.009428631	0	0
IGF1R	4.17532965	4.066334917	3.509885649	4.179416821	3.718398149	4.048777909	2.980824532	3.523813826	1.551009569	1.131397762	1.120053812	1.504111287
IGF2	0	0.008689116	0	0	0	0	0	0	0.018242448	0.009190713	0.018056606	0.01744514
IGF2BP1	14.5301253	13.22887635	13.98366127	11.57080171	14.1180027	13.15347706	12.93311472	10.34233198	0.006461957	0.110690175	0.031980635	0.030897649
IGF2BP2	16.04874778	12.52619985	14.50001294	9.821689889	17.45495105	14.61440443	14.61761723	10.16219621	22.50759141	12.7817818	18.89886017	12.81720947
IGF2BP3	10.32485326	8.181426595	9.963459274	5.731814053	11.31680834	8.186398088	10.25365574	5.782991799	0.038512501	0.08731328	0.03812016	0.018414633
IGF2R	57.25024625	49.12688815	50.23313173	36.79247272	36.40299635	33.2333974	41.3634714	30.76919558	42.26335044	39.41327512	41.259125	53.69418768
IGFALS	0.122294457	0.101425676	0	0.074161594	0.025539454	0.100473561	0.050304698	0.095732597	0.133086948	0.241381531	0.026346229	0.05090809
IGFBP1	0	0	0	0	0	0	0	0	0	0	0	0
IGFBP2	1.63546747	2.640504017	2.014772034	4.769202628	0.811867045	1.43176076	0.661706043	2.151240719	21.62016367	28.75250529	26.77154922	26.95313218
IGFBP3	0.020452133	0.063607892	0.021646819	0	0.021355682	0.021003595	0.021031991	0	0.244827377	0	0.132181764	0.085137056
IGFBP4	4.235457384	6.586318483	3.973449858	4.23211956	4.12103532	6.104352981	4.083319633	5.156972855	29.64577236	32.1674116	32.76547112	32.40723394
IGFBP5	0.103496511	0.053647176	0.109542134	0	0	0	0	0	0.01877168	0	0.009290223	0.017951242
IGFBP6	0.167110437	0.057747538	0.117914661	0.168898039	0.058164388	0.171616331	0.057282782	0.054506137	0.121238627	0.061081133	0.300008818	0.057969875
IGFBP7	33.42157994	32.27600663	36.32523637	30.10386326	27.38946448	27.52533092	35.46156544	35.42178777	146.4623377	114.4693823	131.8111636	109.3190349
IGFBPL1	0.044336359	0	0.015642071	0	0	0	0	0	2.991440762	3.630041157	3.024642597	3.214438302
IGFL1	0	0	0	0	0	0	0	0	0	0	0	0
IGFL2	0	0	0	0	0	0	0	0	0	0	0	0
IGFL3	0	0	0	0	0	0	0	0	0	0	0	0
IGFL4	0	0	0	0	0	0	0	0	0	0	0	0
IGFLR1	5.576627217	5.341752259	4.832357201	4.383774196	3.609576154	4.621783807	3.588401796	6.031153608	4.36525189	2.968098831	4.004626798	4.513850634
IGFN1	0	0	0.014201508	0	0	0	0	0	0	0	0	0
IGHMBP2	3.078298113	3.081213993	3.242063357	3.310468203	3.610142338	3.675205924	3.446265211	3.360825212	3.63874755	3.641526837	2.931978528	3.77166037
IGIP	2.567715025	2.875670502	2.142424375	2.595182201	2.700723863	2.636949782	2.871800771	2.292447245	5.119513244	5.466793304	4.078113598	5.07130397
IGLL1	0	0	0.099567799	0	0	0.096609193	0	0.046025287	0.153561863	0.103154501	0.151997475	0.048950086
IGLL5	0.050811672	0	0.107559549	0.102710422	0	0.104363472	0.104504566	0.049719479	0	0.111434135	0	0.052879036
IGLON5	0	0	0	0	0	0	0	0	0	0.018911658	0.037154938	0
IGSF1	0	0	0	0	0	0	0	0	5.051680627	6.90808256	6.326805681	2.888675591
IGSF10	0	0	0	0	0	0	0	0	0.005558449	0.005600795	0.005501823	0.00531551
IGSF11	0	0	0	0	0	0	0	0	0.0641664	0.064655243	0.025405086	0.061361932
IGSF21	0	0	0	0	0	0	0	0	0	0	0	0
IGSF22	0	0	0	0	0	0	0	0	0	0	0	0
IGSF23	0	0	0	0	0	0	0	0	0.014911703	0	0	0
IGSF3	0.006130048	0.012709984	0.019464382	0	0.006400866	0	0	0	5.416872264	5.10860384	5.632414356	4.535795976
IGSF5	0	0	0	0	0	0	0	0	0	0	0	0
IGSF6	0.154625175	0.106866134	0.054552473	0.104186147	0.215275091	0.105862947	0.212012137	0.100867679	4.767674186	5.143101595	5.163255211	5.900267119
IGSF8	1.125274625	1.588753117	1.531293662	1.364774197	1.354033586	1.331709916	0.903701208	1.226925436	2.810703038	3.548958585	3.289999112	4.260422098
IGSF9	0.012931882	0.013406422	0	0	0.06751598	0.026561143	0.013298526	0	0.070365606	0.184344356	0.153227287	0.053832155
IGSF9B	0	0	0	0	0	0	0	0	0	0	0	0
IHH	0	0.053845677	0.054973727	0.026247668	0	0	0	0.025411645	0	0	0	0.054052991
IK	72.05197529	75.09955402	77.37925687	85.27369588	80.14241045	75.70825616	82.13054375	86.28549649	84.4933611	75.10479278	81.05652202	83.17355827
IKBIP	10.38936426	10.71620865	10.38534384	11.8263214	12.27288434	11.23530533	13.08510796	10.74366187	7.822994849	7.206531338	8.534585327	8.081752343
IKBKB	23.2319528	21.6614372	21.38054282	22.46807259	22.57013792	20.89863195	20.61966902	21.12479505	24.87100369	21.61117107	22.279858	24.91786403
IKBKE	17.07692667	16.02491766	15.44024971	12.24386075	8.359919328	8.760992177	13.67575757	11.09100413	10.70332032	8.08864647	10.85267898	10.75041552
IKBKG	5.747614236	5.116112819	5.391110422	6.28985007	5.401382852	4.701718574	4.993412939	5.914969849	3.666631197	3.911892222	4.547271779	4.207652067
IKZF1	15.78455793	15.71053324	15.35151352	12.87909805	18.19259573	17.58086767	15.08043112	12.41563169	11.03402512	9.164569348	10.26755468	10.94522577
IKZF2	0.141942401	0.058305124	0.124722396	0.081204214	0.120248474	0.077010394	0.071606329	0.055032426	0.055375616	0.046987358	0.03461778	0.055742483
IKZF3	0	0.022295812	0.005690726	0.010868339	0.005614189	0.005521629	0.016587281	0.010522168	0.005851145	0	0	0
IKZF4	0.925553957	0.737020678	0.823447916	0.915118754	1.0784952	1.067602029	0.903515766	0.77440428	1.496253608	1.985688779	1.553255177	1.312201543
IKZF5	11.25127614	9.668823224	11.09582345	8.681346357	15.87469482	13.52072864	13.25475197	8.775488054	7.464618005	6.937728947	6.307856639	6.424531202
IL10	0	0	0.034961806	0	0	0	0	0	0	0	0	0
IL10RA	6.328805025	8.285223764	4.268343019	4.313922017	2.48967762	3.77875127	4.192516822	3.586044626	6.134522006	6.261952201	5.089088314	6.724156616
IL10RB	36.2206121	43.72268998	34.61892673	33.33552915	27.33411825	28.15155436	33.1559522	29.99149461	89.46483333	88.73222843	91.47958839	88.01050802
IL11	0	0	0.047899732	0	0	0	0.04653925	0.088566742	0.098500012	0.099250419	0.097496558	0.023548738
IL11RA	1.843649298	2.623991787	1.422165851	2.305526892	2.088243387	2.11799667	1.831651898	2.018056486	14.6905732	15.38499656	10.36713399	22.30850442
IL12A	1.37973233	1.584995835	2.012884162	0.79146815	1.362812164	1.914776804	1.879018175	1.021678443	0.568132778	0.736021302	0.763182515	0.232843654
IL12B	0.022926954	0	0.048532408	0	0	0	0	0	0.299403104	0.100561354	0.345745143	0.167018446
IL12RB1	1.659668383	1.397963532	1.701721658	0.432460377	0.961268121	0.639157111	1.506716619	0.482123199	1.509815303	1.350702545	1.382700882	1.227927661
IL12RB2	0	0	0	0.01028853	0	0.020908233	0	0	5.738399008	3.817536138	4.320263194	2.722610102
IL13	0	0	0	0	0	0	0	0	0.074691654	0.037630341	0.110896117	0.321422251
IL13RA1	16.15110862	16.65835181	14.21639105	14.64627927	9.501727268	9.598950799	14.06682675	11.5649836	15.03990238	13.06331806	12.47742647	19.27067772
IL13RA2	0	0	0	0	0	0	0	0	0	0	0	0
IL15	0.02304478	0.023890416	0	0	0	0	0.047396289	0.022549434	0.601883584	0.353773551	0.223406995	0.287788773
IL15RA	2.386485276	2.415152048	2.330433237	1.694152559	2.032099106	2.042361193	2.264242614	1.445592541	0.973909766	0.996906008	0.749768606	0.827861229
IL16	1.955849222	2.38398934	1.630986137	1.377749618	1.144901173	1.369490382	1.645610235	1.304869355	1.547965822	1.410281885	1.021464102	1.862723587
IL17A	0	0	0	0	0	0	0	0	0	0	0	0
IL17B	0	0	0	0	0	0	0	0	0.132185682	0	0.06541953	0.031602087
IL17C	0	0.053381935	0	0	0	0	0	0.050385577	0	0.112927032	0.055465745	0.428699704
IL17D	0.06324584	0.091793336	0.120492485	0.038353433	0.052831969	0.051960939	0.065038984	0.074263651	0.013765458	0.069351639	0.068126121	0.092146754
IL17F	0	0	0	0	0	0	0	0	0	0	0	0
IL17RA	52.30304318	48.84998947	47.56430585	39.5631884	25.34541579	28.33639221	32.61740727	34.02744427	29.3811701	23.34675872	25.37844576	53.59838842
IL17RB	0.041498376	0.057361565	0.073204089	0.013980763	0.014443907	0.014205773	0.014224979	0.013535457	0.030107073	0.060672879	0.044700543	0.028791208
IL17RC	0.020267255	0.042021937	0.042902283	0	0.232788996	0.291392249	0.18757684	0.099158057	0.198502566	0.400029657	0.283804952	0.147643048
IL17RD	0.017431021	0.024094211	0.018449233	0	0	0.005967008	0	0	0.202339297	0.254850985	0.156467184	0.241869771
IL17RE	0.546116698	0.530771852	0.325134812	0.224233172	0.463322792	0.455684086	0.40365013	0.26718898	0.724317166	0.692407818	1.158131091	0.355210269
IL17REL	0	0	0	0	0	0	0	0	0.011386011	0	0.011270018	0
IL18	18.56614129	18.67430786	16.18863304	8.520963916	11.73765314	9.698967471	15.68145597	7.528286841	12.33333154	9.446761989	11.16556791	10.45184927

IL18BP	2.425008997	2.59151965	2.498821653	2.159426984	1.952092468	1.897966911	2.043347474	1.808409093	2.476257446	1.991412277	1.749092507	3.579838282
IL18R1	0.140862725	0.504473257	0.257520905	0.69890507	1.056343906	1.301947977	0.30288169	0.801948456	0.055743224	0.098293817	0.068969186	0.213227601
IL18RAP	0.051287906	0.053169933	0.036189218	0.051836539	0.035702493	0.131677026	0.061532355	0.100370952	0.009302344	0.009373213	0.018415156	0.071166194
IL19	0	0	0.019214838	0	0	0.018643879	0.018669085	0	0	0	0	0
IL1A	0.036705021	0.057077887	0.058273651	0.037097659	0.019163301	0	0	0	0.01997212	0.04024855	0	0
IL1B	16.72087377	15.26573151	13.98329157	3.199127565	0.682576198	1.095315995	5.094798066	1.75060887	2.358804472	3.697205042	2.816553375	3.365662718
IL1F10	0	0	0	0	0	0	0	0	0	0	0	0
IL1R1	0	0	0	0	0	0.008213505	0.016449218	0.007825941	0.870366484	1.271645999	0.990724721	1.772854556
IL1R2	0.019043138	0.006580644	0.006718507	0.01283123	0	0.039113218	0.032638414	0.012422538	0.027631595	0.006960525	0.006837525	0.013211962
IL1RAP	8.883149752	8.993666056	8.383911969	7.909915274	7.781332187	7.640846897	7.382500327	5.845160659	5.402196304	4.759677516	3.965598794	8.076645418
IL1RAPL1	0	0	0	0	0	0	0	0	0	0	0	0
IL1RAPL2	0	0.011145679	0	0	0	0	0	0	0	0	0	0
IL1RL1	0	0	0	0	0.010886805	0	0	0	0	0.011432741	0	0
IL1RL2	0.020494977	0.021247047	0.021692166	0	0	0.010523797	0	0	0.022303659	0	0.022076444	0
IL1RN	10.39412023	8.786206389	9.541494936	5.858729158	6.532863288	6.322519034	7.893278033	5.65256177	11.63769225	9.205734482	11.19616861	11.08744914
IL2	0	0	0	0	0	0	0	0	0	0	0	0
IL20	0	0	0	0	0	0	0	0	0	0	0	0
IL20RA	0	0	0	0	0	0	0	0	0.029793059	0.015010016	0.029489547	0.028490918
IL20RB	0.50318701	0.244524204	0.532580071	0.413212853	0.459740024	0.322971704	0.53362377	0.446211257	0.684491608	0.517279728	0.592828646	0.523660071
IL21	0	0	0	0	0	0	0	0	0	0	0	0
IL21R	2.18879415	2.216096072	1.212452292	0.413496815	0.512633783	0.766776939	1.409411233	0.450367188	1.903338141	1.592590982	2.567017098	1.234721966
IL22	0	0	0	0	0	0	0	0	0	0	0	0
IL22RA1	0	0	0	0	0	0	0.018341123	0	0	0	0	0
IL22RA2	0.074296943	0.115534943	0.098296136	0	0	0	0.210109371	0	0.020213413	0.020367406	0	0
IL23A	4.159404623	5.21983238	3.84628222	4.425156111	2.697332074	3.417245607	3.241767361	3.64157763	4.860001807	6.001258579	5.753724855	3.417345332
IL23R	0.063492107	0.115188452	0.01680023	0.032085645	0.033148554	0.081505101	0.114261409	0.015531837	0	0.05221626	0.034195695	0
IL24	0	0.027643271	0	0	0	0	0	0	0	0	0	0
IL25	0	0	0	0	0	0	0	0	0	0	0	0
IL26	0	0	0	0	0	0	0	0	0	0	0	0
IL27	0	0	0	0	0	0	0	0	0	0	0.039918529	0.038566735
IL27RA	11.87715709	11.5199533	11.01555852	11.51590177	7.54622548	8.455491041	10.45426853	11.60216041	10.4716973	10.52940014	12.38164339	14.43443371
IL2RA	0	0	0	0	0	0	0	0	0	0	0	0
IL2RB	0.239209559	0.28714335	0.213206481	0.063623267	0.223485161	0.245659502	0.414301678	0.098554849	2.822414823	1.352931373	2.807223364	1.899822258
IL2RG	1.78897497	1.890987176	1.299444163	0.673610302	2.600562388	2.341544855	1.478971241	1.167014057	0.916165691	1.115467312	1.322463904	2.153803801
IL3	0	0	0	0	0	0	0	0	0	0	0	0
IL31	0	0	0	0	0	0	0	0	0	0	0	0
IL31RA	0.227465924	0.253067466	0.299473332	0.056072965	0.173791521	0.074068044	0.222504541	0.081430449	1.141098112	2.792350534	1.464132116	1.81293476
IL32	2.720120381	5.459107178	2.620639969	1.163130631	0.254897985	0.537204717	1.255172311	0.887217198	0.075901824	0	0.075128585	0.036292222
IL33	0	0	0	0	0	0	0	0	0.016602851	0.016729338	0.016433712	0.015877204
IL34	0	0	0	0	0	0	0	0	0	0	0.03023563	0
IL36A	0	0	0	0	0	0	0	0	0	0	0	0
IL36B	0	0	0	0	0	0	0	0	0	0.028518306	0	0
IL36G	0	0	0	0	0	0	0	0.08688602	0.048315394	0	0.047823188	0.184814848
IL36RN	0	0	0	0	0	0	0	0	0	0	0	0
IL37	0.033515765	0.034745638	0	0.067748575	0.017498224	0	0	0	0.109420599	0.128629904	0.090254911	0.052319121
IL3RA	5.712658859	0.700485546	0.682653201	3.600844496	0.673471901	0.66236851	4.232255991	0.571007784	3.542908247	0.370461254	0.297748478	0.319628418
IL4	0	0	0	0	0	0	0	0	0	0	0	0
IL4I1	21.5841393	12.99694108	17.64669845	1.828804709	0.359883418	0.287584452	8.74995649	1.665164667	1.312755162	0.850343265	2.041885477	0.582854832
IL4R	12.88652438	11.64387553	11.34425319	8.679711425	7.447878928	7.696225053	8.978028298	8.245050282	10.92921871	8.353217365	10.72568111	10.15462535
IL5	0	0	0	0	0	0	0	0	0.017263637	0	0	0
IL5RA	0	0	0	0	0	0	0	0	0	0.009215114	0	0
IL6	0.03288088	0	0	0.016616306	0	0	0	0	0	0	0	0
IL6R	42.07279133	38.77960546	41.24664331	53.63972231	35.86177672	37.90511788	37.74201409	46.36156234	99.71131108	69.64133695	82.50389105	127.7495737
IL6ST	2.689591365	2.684109118	2.759109805	2.61082266	1.740599496	2.057925459	2.139731857	2.007092841	6.883152264	7.02633657	5.374017125	7.972599461
IL7	0	0	0	0	0	0	0	0	0.110233071	0.111072865	0.060616716	0.105415204
IL7R	3.466331417	3.593529673	3.902730582	0.517282192	0.522272447	0.776465587	1.842113241	0.307312811	0.898754053	0.573972513	1.052482297	0.18157879
IL9	0	0	0	0	0	0	0	0	0	0	0	0
IL9R	0	0	0.008903045	0.008501668	0	0	0.008650175	0.008230878	0	0	0.02718229	0.008753931
ILDR1	0	0.015093107	0	0	0	0.014951423	0.029943273	0	0	0.015964387	0.031364558	0
ILDR2	0.186753431	0.203796225	0.208065691	0.307962412	0.051316831	0.060564938	0.040431212	0.081751738	0.385075761	0.215560779	0.248808117	0.255726088
ILF2	185.1432844	198.5224016	221.4465174	275.4925513	227.4002962	243.1054276	245.4953723	288.3203635	259.4567056	306.8165552	304.1925088	290.4832701
ILF3	90.03946795	82.59103964	90.51385507	99.16281914	106.4708148	103.8190721	91.51936079	106.3801339	63.9458245	59.56518135	69.33221656	66.32438329
ILK	26.36931165	29.43172774	25.15075885	27.97885129	25.27667471	24.88069464	23.91692761	26.33635018	56.07343429	59.80203018	57.65698573	52.73948114
ILKAP	8.651200441	8.217357994	9.172529873	11.97877591	10.49955328	9.473664474	9.765942928	12.2324985	10.30191559	10.6287341	10.58728089	12.50704924
ILVBL	11.97310448	14.26533651	13.03981771	15.71399905	13.89721849	16.35894904	13.18693617	15.90964006	7.081375814	8.847802023	7.32698737	8.108192705
IMMP1L	3.57658769	3.54118771	3.913111187	4.467788309	4.070290864	4.622234025	3.512688028	5.387192816	4.504481313	5.06759003	5.584062563	5.22767913
IMMP2L	0.128142271	0.14039248	0.131003881	0.105965203	0.130762446	0.152533401	0.121293226	0.112564104	0.107757462	0.185221969	0.138030198	0.113655655
IMMT	61.44590118	62.50399114	68.92602279	63.53910787	65.40291709	63.99635284	62.34794163	63.79171613	44.19892208	45.91825568	54.88189036	39.75367373
IMP3	38.52226028	37.594626	42.88696122	39.81222592	45.57524828	46.56329196	42.33877252	49.38071244	32.75292344	32.47859831	36.34886501	37.96535514
IMP4	18.24605912	15.70229358	19.39157199	18.89783144	21.54176701	21.31548472	21.88629774	20.77629541	8.576162648	9.687285341	11.19223196	9.716226851
IMPA1	13.86751819	13.86461073	15.56424354	17.22121408	15.19870866	12.59760245	17.42973178	14.31597551	17.90772387	16.94509833	17.82197505	17.31186419
IMPA2	7.211878806	10.80907917	8.402384359	12.62865993	10.79954052	13.09026967	8.68115361	12.58199846	13.17180535	15.9694956	16.59059674	15.41787864
IMPACT	10.78778215	11.17023398	10.02149902	8.876809971	10.39996032	10.53402491	10.64137844	8.783926023	11.6834984	14.29721378	11.49480902	15.07662661
IMPAD1	23.5109824	20.50047183	22.97804468	26.34258097	22.03175299	22.21118846	22.42490206	22.14627317	29.27912853	32.64419051	28.8525825	37.52623304
IMPDH1	35.48149491	36.31959968	38.67895057	42.19279019	33.05792934	35.84462177	36.66002806	48.69392436	21.36585055	23.04155575	24.16075173	22.56643082
IMPDH2	313.1068134	358.9540207	307.6273604	292.500473	300.9716948	355.3447222	332.9443715	341.0133512	77.29689932	130.6911347	104.2472665	76.56331374
IMPG1	0	0	0	0	0	0	0	0	0	0	0	0
IMPG2	0.051541725	0.0601122	0.027276236	0.026046537	0.026909386	0.019849303	0	0.02521692	0.154248282	0.1766175	0.16655662	0.160916376
INA	0.016654151	0.017265281	0.017626983	0	0	0	0	0	0	0.018261954	0	0
INAFM1	2.244737342	3.191463458	2.986796636	3.565177918	2.879657141	3.556692208	2.77005608	4.01643315	5.653419335	4.711910705	4.559561909	5.606564365
INAFM2	8.291912734	7.238022916	8.50571526	9.042664613	6.923254582	6.759889825	8.559864152	7.707213083	13.37033839	12.54368528	12.51132974	19.43667243
INAVA	4.507455591	5.233201013	5.067009781	3.541132951	5.039174765	5.124602167	4.526069337	4.013900366	2.573412194	4.529842551	4.044323358	3.334822316
INCA1	0.047409305	0.024574503	0.050178662	0	0	0	0.048753452	0.046390245	0.051593178	0.077979349	0.102135161	0.074007355
INCENP	10.75860183	10.50992757	11.87757174	20.22505962	18.83839142	17.72890135	11.267881	19.73472567	20.1108256	21.461911	21.91169911	17.96581098
INF2	5.497171456	6.358663169	5.828044299	4.679697401	6.934263801	7.255658427	5.909120601	6.191276796	11.47276103	13.35032124	13.77012706	10.50099337
ING1	4.308844248	5.405284476	4.965321848	6.081449794	5.484236208	5.694929987	4.457227453	5.588370502	5.257896097	5.926997092	5.067014606	5.333228461
ING2	2.516747349	3.52423247	2.425214422	4.347016967	4.334130017	4.474843416	3.244784713	4.263692625	4.292228266	4.880990129	4.511504334	4.182814782
ING3	7.295011213	7.19558266	7.833584266	10.29005329	8.726610383	8.637287864	8.011672875	9.113340013	6.628509519	4.679188621	5.721787133	6.136108362
ING4	11.64535272	10.53557752	10.20049814	9.584527417	10.96642453	11.38834938	10.26019473	10.09533758	9.378733479	8.569521648	6.887527358	7.972288698
ING5	4.514923293	4.178635369	4.41902155	5.288699007	5.242149813	4.798050708	4.027075916	5.219991947	2.67623576	2.612791381	2.845700503	3.425617381
INHA	0	0.038982615	0	0	0	0	0	0.073588998	0.491054567	0.494795592	0.405043352	0.860919479
INHBA	0.185344778	0.473502817	0.266232709	0.046831859	0.048383269	0.067979405	0.061264179	0.045340202	0	0	0.007130238	0
INHBB	0	0	0	0	0	0	0.017265263	0	0.475043583	0.478662631	0.397865049	0.803728345
INHBC	0.048902964	0.067596633	0.034506381	0.082376808	0.068084579	0.050221562	0.083815765	0.015950599	0.124176855	0.107248181	0.035117664	0.06785689
INHBE	0.218382958	0.158477618	0.323595348	0.220719028	0.182424671	0.067281402	0.112287272	0.085475533	1.021917635	0.263412385	0.752749398	0.068180477
INIP	9.648951294	9.49135939	10.23870226	13.41858322	10.29425639	10.54269698	10.30317733	11.92870745	17.53659338	15.04537131	16.82630517	18.02853794
INMT	0.042055147	0.043598376	0	0.042505016	0.04391309	0.021594552	0	0.020575587	0.297482353	0.092230362	0.022650138	0.065649354
INO80	9.546750119	8.239501942	8.658909922	9.265072675	10.19111799	9.468920968	8.755521177	8.885222398	7.13400086	5.734608625	7.054148161	7.099526112
INO80B	12.54560737	11.32492875	10.92987556	9.962707333	9.267925547	8.633077191	8.732512615	11.27382288	8.173079495	9.21797125	5.60771439	10.56918154
INO80C	5.370578157	5.191097149	6.645406497	5.428027917	6.014208918	4.582834558	5.576205442	5.509009376	5.73159411	3.641542872	5.058374331	4.266068475
INO80D	2.10071273	1.771998046	1.809120842	2.01109074	2.486442956	2.170755162	1.982485707	1.46187204	2.389046254	2.29636326	2.080099937	1.775425806
INO80E	9.587785634	9.854294248	10.3075388	11.90845267	11.21444388	11.0154674	10.55077889	13.42159784	11.73254908	11.92721612	11.73122424	15.55921482
INPP1	4.669692706	3.944558224	4.85093999	5.244050006	3.295810607	2.708628343	4.602000703	4.252004256	7.952065443	7.372583536	8.849115062	7.784499396
INPP4A	4.962413673	4.722881531	4.571256574	4.190317576	3.977349669	4.16424912	4.663023341	3.739073651	5.172442358	4.872378817	4.413576622	5.732867132
INPP4B	2.608575403	2.257671651	2.405185204	1.392405777	1.057889751	1.390505996	1.923050441	1.273935887	8.485434575	6.608531457	7.751345202	6.25713545
INPP5A	7.482464027	7.375675145	8.002383526	6.913838161	6.440028701	6.719671762	6.61607389	6.448547731	6.992920589	5.921893571	6.508572396	7.811337307
INPP5B	8.860208817	9.025314009	8.408404507	8.507758255	8.574692161	8.401618772	8.391812608	9.15309081	14.62556583	12.69457279	13.2683418	13.95421021
INPP5D	71.44017119	61.01685625	62.35989584	46.32581634	85.43545049	67.1984769	60.64113622	46.97555174	105.353817	119.9543165	106.0269709	100.6324726
INPP5E	5.824019971	6.849257887	5.965377151	6.614198567	7.192956473	7.010055262	6.971232962	8.701444076	9.081044811	9.4764081	9.376405945	10.42749353
INPP5F	3.786997457	3.630530693	2.975995634	3.827507452	3.392235812	3.271273468	3.295233017	2.943408374	1.771151238	2.270734425	1.814500785	2.115528601
INPP5J	0	0	0	0	0	0	0	0	0.638028515	0.552884745	0.656789843	0.866402662
INPP5K	7.966486404	7.352162611	8.39817904	8.774911955	7.770514704	7.609743999	7.750847983	9.880203749	3.789674438	2.667750968	3.733939538	4.914796972
INPPL1	44.13582159	42.76205715	42.16106606	35.47592491	57.099488	49.35014122	41.05447297	38.76654734	64.83197433	54.14747111	63.35693433	55.50830801
INS	0	0	0	0	0	0	0	0	0	0	0	0
INS-IGF2	0	0	0	0	0	0	0	0	0	0	0	0
INSC	0.028420543	0.019642296	0.030080694	0.009574854	0	0.009728954	0.009742107	0.027809645	0.010309552	0.010388094	0.020409051	0
INSIG1	45.8471659	87.22512525	36.3939545	43.91136767	68.45504401	108.1424431	31.83119459	43.58795669	143.093347	233.3481195	182.498333	59.72864014
INSIG2	4.999659126	9.478863774	5.085805127	6.331172917	6.581533896	8.33102358	4.421211895	5.653622456	5.080976752	7.210223619	4.945394817	5.587742617
INSL3	0	0	0	0	0	0	0	0	0	0	0	0
INSL4	0	0	0	0	0	0	0	0	0.190433356	0	0	0
INSL5	0	0	0	0	0	0	0	0	0.081557461	0.082178794	0	0
INSL6	0	0	0	0	0	0	0	0	0	0	0	0
INSM1	0.304331203	0.354936087	0.301976571	0.153793336	0.119166063	0.25393636	0.058679924	0.055835555	0.082797111	0.250283666	0.266349292	0.178151322
INSM2	0	0.018514478	0	0	0	0	0	0	0	0	0.019237207	0
INSR	14.79421781	16.6102763	15.23715578	14.61917465	14.40885041	15.81205852	13.52390567	13.30788298	15.68524743	11.6587257	13.8351108	13.47310785
INSRR	0	0	0	0.012970467	0.013400143	0	0	0	0.502765861	0.07036057	0.331762673	0.14690863
INTS1	12.82652641	12.45753785	13.10817982	13.51443161	14.5464454	15.59966147	12.72072825	16.22528662	18.17325219	18.60236433	20.06610704	19.53218297
INTS10	38.52257132	38.87076221	38.00251238	44.35531656	37.75967488	45.30005362	37.96345895	41.62268193	34.46042463	43.63259912	36.75578045	44.9528612
INTS11	22.41212215	24.16816836	24.02458519	24.92739755	25.98826441	26.29548637	24.68929324	28.35927977	23.29856861	24.97979028	24.36199412	25.68834994
INTS12	12.49371413	9.810311057	12.35067978	11.0020571	12.91650543	12.25892931	14.88325205	12.06377438	11.50972639	10.35402431	9.882359436	11.02813563
INTS13	22.19622944	23.02851055	24.40055406	19.43441951	27.95906684	29.84099994	26.65331166	19.16788146	18.98429947	21.57412094	21.30374381	16.13739386
INTS14	16.17656944	16.11023695	17.73581742	19.03670133	17.63413096	18.68934089	18.40654854	18.13723001	14.36449938	16.95811176	16.33576199	17.51670496
INTS2	5.325889024	6.271133889	5.436915887	6.828258529	7.20035498	7.503673082	5.527600396	5.444636089	6.162614803	6.461911778	6.029008736	5.901823766
INTS3	30.33493078	32.65212903	29.28375128	27.91380704	29.99986559	28.92910781	28.3305533	31.47425823	55.00213097	47.28336638	46.68106479	52.97719129
INTS4	11.53375265	10.45668133	11.44935022	13.72556958	12.21120318	13.37600342	11.62023493	13.10244019	12.04254295	10.98016746	10.61292808	12.62675524
INTS5	11.69559405	11.37758342	12.91623199	12.18492666	12.02414595	11.82590635	11.53868797	14.85822361	9.804274394	7.310435422	9.122131139	13.1942611
INTS6	16.47324668	13.95260688	15.28695799	15.43383687	16.6658004	13.97798263	18.66250687	12.48911768	10.92410002	8.475305303	8.866724826	8.90447453
INTS6L	11.15026356	10.90893398	10.7066921	9.10990139	10.77518656	10.1098948	9.949169012	7.716234375	7.981861387	9.31648016	8.375493579	6.274065092

INTS7	12.47642762	10.85406392	13.69936755	17.91086224	13.33878956	11.88451804	13.15704195	15.30890938	11.33978432	7.069196779	9.769066908	10.77180221
INTS8	11.90142384	11.26844574	12.64411438	13.29832672	14.52183814	13.55184423	13.96560514	12.96255394	19.91929278	20.29593058	19.02601897	22.70369839
INTS9	16.2075091	14.20345788	17.62148756	16.25340521	15.85346823	16.04544812	17.57495517	17.43270114	13.27983913	10.89151944	13.7389447	14.04484542
INTU	0.574406292	0.521048792	0.336549032	0.362844243	0.589072422	0.484556061	0.443018881	0.331213617	0.468823255	0.798572356	0.607680843	0.576427857
INVS	5.323462689	5.894088046	5.555544357	5.025301491	6.014455493	5.598210286	5.31016154	4.156810131	6.106094474	6.117588476	5.814519936	6.01650219
IP6K1	8.470721978	7.418517286	8.475048369	9.084163952	8.304798195	6.817633182	9.33150638	10.02862705	23.91353618	14.74813923	19.44439288	24.38245665
IP6K2	7.65032013	8.803159172	7.940078762	7.78217053	7.977967361	7.43988517	9.169944134	7.805455256	12.61205058	11.25391506	10.94844025	11.15446155
IP6K3	0	0.035106433	0	0.011408678	0	0	0	0.011045296	0	0	0	0
IPCEF1	5.711413191	5.592051319	4.750843256	2.197933608	2.270745062	2.463791479	5.300333929	2.044626873	11.95083658	12.36435689	11.32891633	16.32529382
IPMK	6.037048553	5.922375461	5.21033017	4.0761562	7.172043837	5.491033049	5.797750552	4.019555956	3.87311606	3.638192762	2.866287308	3.582683375
IPO11	6.806044175	6.3694799	7.117561056	9.203102133	8.998619595	8.981464559	8.718901911	8.353097846	10.4274902	11.1564498	11.04687769	10.00800523
IPO13	12.74733832	11.82472647	12.61637433	14.1251952	9.083074365	10.56809608	11.23975457	14.17814702	14.7452774	12.67226488	12.96138726	19.07067088
IPO4	30.17819538	23.64626936	29.96349105	26.6789466	32.60395065	30.82305578	33.58537273	33.40535786	13.76119041	15.11167587	20.93061521	12.273092
IPO5	70.87112894	71.32347267	78.63906982	80.99305998	105.007645	96.65259923	84.75195401	79.14836851	48.7014516	59.57245241	60.4170775	45.67432651
IPO7	67.67907946	58.14449636	69.2410429	68.86855733	76.80314335	70.13758784	73.99470123	62.71242674	59.25808119	61.95840472	62.35189084	55.67332384
IPO8	11.32553883	11.55796447	11.61309381	14.47354429	14.49178471	14.10770203	12.44609752	13.13946003	39.17663726	39.51384715	35.83709998	38.77907656
IPO9	16.09667575	17.38122993	16.65280168	21.40439405	20.19889818	21.01310885	16.72754593	18.7347753	18.27124314	16.31200118	18.70960739	19.39064884
IPP	0.678832036	0.691175196	0.705655096	0.980133699	0.594904153	0.647340357	0.648215528	0.593071957	1.991956931	2.405900373	1.553828055	2.787960496
IPPK	3.38938936	2.915676676	3.16757707	3.10980638	2.986923849	2.752531217	3.164128429	3.304773924	3.361508167	3.611167874	3.068332313	4.177715482
IQANK1	0	0	0	0	0	0	0	0.034190213	0	0	0.03763747	0.072725843
IQCA1	0	0	0	0	0	0	0	0	0	0	0	0
IQCA1L	0	0	0	0	0	0	0.021233756	0	0	0	0	0.064465348
IQCB1	13.64391577	12.68852395	14.40229656	14.25075774	15.59897901	15.90377265	15.24999711	12.86873265	14.68918991	16.06119071	14.73602635	14.95834993
IQCC	1.7130824	2.822048895	2.508604846	3.178199997	3.479513914	3.518546424	2.364957022	3.880656296	4.341432054	4.425971386	4.802757826	5.275081618
IQCD	0.366779171	0.411924784	0.242627583	0.571499926	0.351067756	0.32958524	0.298599315	0.717790559	4.057999069	1.390901185	2.535104335	2.322021934
IQCE	4.386094623	3.390896314	3.843395073	5.01720878	2.975138206	3.366048405	4.251710106	5.615957476	3.404119582	2.617279843	2.827400178	5.696842356
IQCF1	0	0	0	0	0	0	0	0	0	0	0	0
IQCF2	0	0	0	0	0	0	0	0	0	0	0	0
IQCF3	0	0	0	0	0	0	0	0	0	0	0	0
IQCF5	0	0	0	0	0	0	0	0	0	0	0	0
IQCF6	0	0	0	0	0	0	0	0	0	0	0	0
IQCG	2.115619215	1.905612923	1.541744644	2.103196981	1.557223552	1.460315051	2.282597966	1.645934272	1.868278263	1.958572529	1.998679437	2.147556868
IQCH	0.301121843	0.274332632	0.202816416	0.11989269	0.209616681	0.13119322	0.178288654	0.080358887	0.36741657	0.390227336	0.304699477	0.379846694
IQCJ	0	0	0	0	0	0	0	0	0	0	0	0
IQCJ-SCHIP	0	0	0	0	0	0	0	0	0	0	0	0
IQCK	0.494867479	0.950608511	0.785661846	1.044454144	0.820688971	1.031369121	0.748379333	1.082397228	1.077079113	1.117204816	0.768223825	1.090592566
IQCM	0	0	0	0	0	0	0	0	0	0	0	0
IQCN	1.331021294	0.727564439	0.691578635	0.232362987	0.353773412	0.074558748	0.398184253	0.177601512	0.513553413	0.358245583	0.508321665	0.831105762
IQGAP1	49.75705163	47.13410116	46.0598707	50.94555183	49.96027268	46.17045803	46.4400972	39.89499964	105.8840496	93.81777358	104.8616453	84.91483509
IQGAP2	10.82089053	11.03114235	10.55130687	13.32929235	8.664519259	10.20413095	9.417676616	10.01119006	4.671110778	4.311478107	4.279407258	5.36205014
IQGAP3	7.403516852	8.637702021	7.929169017	15.10294657	15.92084656	15.14052725	7.827419999	15.77316639	53.73284067	65.81493429	60.16878326	42.3302698
IQSEC1	3.067010892	4.96914207	2.47749329	1.688243917	3.190840828	4.43483539	2.763311436	1.885928184	8.657283851	8.949895237	7.503970563	10.2730538
IQSEC2	0.667658438	0.619097235	0.600660063	0.239929054	0.209146421	0.167605996	0.442467738	0.225028025	2.252395908	2.025517228	1.869861261	2.918257911
IQSEC3	0.215727225	0.320555557	0.091331468	0.050874807	0.090103112	0.073848	0.059158271	0.035181697	0.031302022	0.007885123	0.007745784	0.007483482
IQUB	0.019178886	0.039765324	0.013532798	0.006461348	0.033376974	0.006565339	0	0.012511091	0.020871423	0.014020286	0	0.026612284
IRAK1	92.81592214	92.51155586	96.86689408	103.8019199	80.52859493	80.04471703	90.00557198	99.09215703	44.78251802	39.7638175	56.97167456	52.89751055
IRAK1BP1	2.909004727	2.884632044	2.721953246	2.755477333	3.477741258	3.46370072	3.222706304	3.01148898	6.774956354	4.807877983	6.312361041	4.153483225
IRAK2	1.473597083	1.093490999	1.099981651	0.62709909	0.388723888	0.446034258	1.020885196	0.333918831	1.485478992	1.479786846	1.236427233	1.065415775
IRAK3	38.0936093	35.17261077	34.7149863	31.9115275	30.81385313	31.90192101	29.61731301	24.84985999	37.21649535	29.45975833	31.30793365	38.55628881
IRAK4	8.785234426	8.65382336	8.705070469	9.9891126	9.702586891	8.690884164	9.105387265	9.29522939	9.167747681	8.57234952	7.535765953	10.72506383
IREB2	22.84799379	20.33225354	21.33272483	20.20494915	22.87296408	21.32229268	22.45282827	15.22839538	26.31002126	29.353384	25.75733791	26.02975096
IRF1	8.229697543	7.297250653	9.396411257	9.627850575	7.239755566	6.60958426	7.858523842	8.185539277	11.10474511	9.354755149	10.89420256	13.16052553
IRF2	3.833931211	3.309012673	4.019054226	3.219849186	3.492520324	3.215153953	3.684819134	3.213025285	4.451758406	4.626479352	3.945347783	4.199917413
IRF2BP1	4.449152458	5.93957302	5.219931204	7.487505661	4.338918147	5.582082203	4.078918381	8.070437142	9.044098985	8.836848576	8.97456956	12.3616914
IRF2BP2	165.7560652	168.8357944	160.2567968	157.5572041	143.6781872	144.4498669	137.9878473	157.2361199	115.1072237	112.1753761	102.1135311	122.1455715
IRF2BPL	2.601809414	2.482574576	2.534583724	1.949336089	2.054460763	2.246576256	3.301207995	2.533217624	2.549685958	4.456909681	2.858347185	4.431955188
IRF3	29.81237586	32.09100724	29.91432071	32.26151814	33.35676861	33.71957193	30.37036349	36.92413945	39.5731119	39.87459371	41.7411797	47.06655915
IRF4	0	0.031415875	0.021382685	0.040837371	0	0.020747309	0	0.059304969	0.230847268	0.177223576	0.163211105	0.094610492
IRF5	4.059834896	4.514402279	3.60209324	4.347021693	5.23253553	6.425954719	4.932766459	6.961828919	17.86201815	16.96963455	17.75221502	18.43390046
IRF6	0.011944409	0.012382713	0.012642127	0	0.037416292	0	0.012283056	0.011687664	0.012998503	0.065487652	0.064330415	0
IRF7	7.827302819	3.757209319	3.249878072	2.874426591	3.521529942	2.662220408	3.364626672	3.300043216	10.3257544	4.664050171	5.449159231	4.845554857
IRF8	228.3886109	215.9709886	238.4386777	161.4741093	217.8401681	247.0754695	207.1861107	178.0548787	189.102005	187.9583585	190.5675005	226.8810741
IRF9	15.58489069	8.311102756	6.923937655	5.574641835	5.324017272	5.335038312	4.748667565	5.271568509	28.37178963	15.54227262	13.54051718	19.62297528
IRGC	0	0	0	0	0	0	0	0	0	0	0	0
IRGM	0.106553586	0.110463607	0.096666673	0.046154316	0.063577707	0.078161893	0.078267564	0.074473732	0	0.050074434	0.081982608	0.031682545
IRGQ	5.174207722	3.266329627	4.836272355	2.150731683	2.520540016	2.027233254	4.727181724	2.259782332	2.076406622	2.429875625	2.523164292	2.174492291
IRS1	0.006159519	0.006385545	0.026077281	0.006225408	0.032158195	0.006325602	0.044339077	0.012054242	2.33938092	1.472407693	1.671972221	3.525571699
IRS2	6.935727908	10.4824407	6.91767135	11.7194676	5.652401577	8.804717248	6.444435813	10.46587929	6.81980273	6.660968239	5.880652986	7.978122627
IRS4	0	0	0	0	0	0	0	0	0	0.028656811	0	0.02039785
IRX1	0	0	0.027687303	0	0	0	0	0	0	0	0	0
IRX2	0.017232846	0.008932606	0.027359222	0	0	0	0	0	0	0	0	0
IRX3	23.07491845	33.39106502	24.50388349	41.57271963	42.55000266	48.02350172	24.79251828	43.6963092	55.3490145	29.90114914	39.80999575	56.13839787
IRX4	0	0	0	0	0	0	0	0	0	0	0	0
IRX5	2.987250657	2.651567468	2.707116963	4.005874175	4.709415996	3.970091	2.650305573	4.527846159	15.87192236	7.750211725	11.12545038	14.30450827
IRX6	0	0	0	0	0	0	0	0	0.131044693	0.207496201	0.111179738	0.017902461
ISCA1	13.55979453	12.8325749	14.40871264	15.44169707	15.53267786	16.2417216	16.89876384	16.8941282	18.61357772	13.42614506	17.15134245	18.0794848
ISCA2	6.577823157	8.459908162	7.642560726	9.047532644	7.333203498	9.294727866	7.374631749	9.098116291	18.19181148	22.28933627	19.97760926	22.59514392
ISCU	26.99179971	23.898108	26.87715789	25.68144098	20.17173806	22.17892559	29.72577234	26.27227856	12.58632341	12.76895605	12.23654902	15.04939534
ISG15	16.61884488	8.196167806	8.026328999	5.789125893	6.402093997	5.13665432	6.802824237	9.630670548	19.75353499	8.580846045	9.993397183	14.10467017
ISG20	0.231604997	0.186747418	0.136185512	0.18206417	0.188095452	0.158566596	0.264634949	0.184658763	0.252044148	0.178715625	0.120118308	0.187466424
ISG20L2	29.83621971	28.06708234	33.9345009	36.12751217	27.22800082	30.89289754	32.49717931	37.87526675	42.32362333	38.96038979	44.02510463	53.12388407
ISL1	0	0	0	0	0	0	0	0	0	0	0	0
ISL2	3.117836693	3.343703598	3.47064901	3.341346522	3.508166748	3.753957225	3.759032383	3.918724436	3.188236773	2.917798732	4.400688789	3.412520309
ISLR	0	0	0	0	0	0	0	0	0.540160023	0.445316033	0.899196241	0.164357355
ISLR2	0	0	0	0	0	0.007643217	0	0	0	0.016322095	0	0.015490705
ISM1	1.349738149	1.455237957	1.885727541	1.637011764	1.747616145	2.16236577	1.961714982	1.672919127	0.685464548	0.473613707	0.639711111	0.449489489
ISM2	0	0	0.017859135	0	0	0	0	0	0	0	0.018175511	0
ISOC1	7.287288623	6.865296131	8.446138525	12.5461151	12.29628703	11.52445197	8.177751381	9.869034969	10.70452176	12.09255458	10.20746789	10.72687455
ISOC2	33.757451	31.13421493	33.92828083	36.67512652	31.50302556	36.60414991	40.81559819	48.37769055	24.32419909	26.52675291	31.7055473	28.76524633
ISPD	0.140075868	0.102861334	0.151346943	0.132725885	0.164547271	0.161834414	0.135044338	0.11136527	0.149261787	0.095999307	0.075442317	0.097183403
IST1	34.97431585	35.92964601	34.2708103	36.51264907	35.52813802	35.24137925	36.3433989	34.04914137	54.49458126	51.91163492	48.18552358	53.87802782
ISX	0	0	0	0	0	0	0	0	0	0	0	0
ISY1	3.611567821	3.947263554	4.607782254	4.301012293	3.127985147	3.522063566	3.901100545	4.547547399	4.311130788	4.113728493	3.664072318	4.428632999
ISY1-RAB43	0	0	0	0	0	0	0	0	0	0	0	0
ISYNA1	0.85791511	1.312224504	0.699629044	0.824448452	0.543364231	0.375528469	0.535128388	0.756903047	1.316259829	1.974009388	1.090758668	2.385734582
ITCH	7.121346497	6.041988827	6.83774046	6.54690077	6.877811497	6.669976306	6.77947421	5.944685456	9.876467411	9.258430455	9.689176055	11.19140565
ITFG1	10.08137949	10.12317738	9.614969549	11.30891594	8.428019795	8.874179523	9.227952996	10.94871186	19.92703042	21.81426428	16.33156238	31.27703203
ITFG2	5.671178264	5.771829873	6.56665255	7.093717643	7.266867187	6.858136162	5.862421473	7.157552722	1.836995406	1.704859468	1.674732769	2.696699861
ITGA1	0.302931223	0.511780933	0.344376701	0.464927421	0.585767292	0.645243052	0.299982144	0.428161844	0.012209812	0	0	0.023352335
ITGA10	0.046913305	0.038907844	0.029792213	0.009483029	0.05878305	0	0.019297356	0.018361963	0.510534063	0.452692672	0.47501309	0.361283218
ITGA11	0.01074794	0.022284679	0.022751536	0.010862912	0	0.011037743	0.011052665	0.005258457	0.61991164	0.842667086	0.590441803	0.665521718
ITGA2	0	0.007089099	0	0	0.042841631	0	0.007032046	0	0.014883278	0	0	0.007116393
ITGA2B	0.078416731	0.195106226	0.06639788	0.174362243	0.163762164	0.161062252	0.112896	0.092077403	0.699763492	1.221017365	0.861570063	0.538607887
ITGA3	0.021374205	0	0.03393413	0	0.11159245	0.010975265	0.010990103	0	1.407259008	0.914069752	1.082105292	2.357848372
ITGA4	14.95880509	15.92271915	14.39800801	15.21819388	15.53166791	21.26178959	13.46907963	13.57211347	25.59535407	27.01479316	22.64909172	25.24408726
ITGA5	42.6464753	50.46273755	37.76632685	44.72704766	31.62540113	37.36062492	35.207465	43.85102644	60.41574287	59.16831334	56.87467009	83.74551692
ITGA6	1.097973597	1.009750434	0.721633069	1.530337968	0.443798969	0.336454989	0.418860905	0.701807996	0.915424456	0.902979565	0.59134863	2.626244227
ITGA7	19.6910982	17.87802811	16.94029105	7.643998351	14.78228301	12.26981275	11.4982167	9.054891947	6.930334152	9.170767861	8.183114425	7.554103656
ITGA8	0	0	0	0	0	0	0	0	0	0	0	0
ITGA9	0	0	0	0	0	0	0	0	0.812898571	1.146728123	1.08257599	0.932843847
ITGAD	0	0	0	0	0	0	0	0	0.013659495	0.08258135	0	0
ITGAE	2.734016317	2.494609235	2.794620555	3.889386514	2.629937155	2.836581743	3.090758463	3.298251992	1.620108382	1.560582026	1.623774889	1.763668121
ITGAL	68.51178832	56.67347823	64.78026636	30.16747395	53.81865429	44.03013998	59.5940593	32.48715589	24.02637668	28.33983236	25.95902579	26.84499164
ITGAM	12.5688999	16.85505371	7.833653934	8.839555491	8.354914295	8.21716856	10.28534719	7.938173828	22.74846789	25.2249184	18.36291892	41.4035642
ITGAV	3.23902211	3.172189999	3.278142058	3.220869208	3.288603193	2.835834906	3.039213104	2.475636991	13.30352636	9.288483341	10.08903441	10.97454148
ITGAX	12.8184517	13.47819234	9.245729073	2.269668578	4.016548305	4.182051477	8.706891431	1.987101329	22.25166997	30.84533787	23.76105783	30.06809884
ITGB1	36.74638717	35.29673078	35.25582009	33.85267162	30.78107221	27.85332631	31.03210296	24.74829345	54.5607641	60.04384461	49.72198019	61.0137256
ITGB1BP1	9.819791915	11.0942159	10.95589979	13.4650597	12.73819677	12.87550078	13.05437967	13.69803455	11.42247483	12.60879111	13.59595518	12.65788802
ITGB1BP2	1.785348084	1.463472328	1.186516277	1.17498821	1.083850282	1.279177282	1.323603553	1.421954083	1.626618252	1.092673599	0.581405978	0.907389564
ITGB2	167.8376422	195.19447	162.034913	122.8942467	97.74825744	118.1419117	129.7155443	124.6381259	107.3975915	143.1252376	126.027776	157.9336108
ITGB3	0.637710366	0.752299148	0.453853386	0.544518443	0.608479838	0.350035598	0.463576192	0.20441472	0.43075138	0.361694162	0.260555268	0.56067553
ITGB3BP	5.421987342	6.699563635	6.498696995	9.863976658	10.81810093	11.49754639	7.293633239	8.70379289	11.48201119	14.59040629	12.26477226	9.79065715
ITGB4	0.031321048	0.056823174	0.049725944	0.04748414	0.114466703	0.096496726	0.032209062	0.06895756	0.093734113	0.07727581	0.143386054	0.057041952
ITGB5	0.034143122	0.011798672	0.024091701	0.011502785	0.035651522	0.023375828	0.035111147	0.022272812	4.50829221	4.729834577	4.683031049	3.683514708
ITGB6	0	0	0	0	0	0	0	0	0	0	0	0
ITGB7	5.345438224	5.89139239	4.943426232	3.015415289	2.392111229	2.64447739	7.304972657	3.110519528	11.13186669	10.65194481	8.914242213	25.55989343
ITGB8	0.025147005	0.005213957	0.031939124	0	0.010503187	0	0	0	0.005473246	0.005514943	0	0.015702093
ITGBL1	0	0	0	0	0	0	0	0	0	0	0	0
ITIH1	0.016526278	0.017132716	0	0	0	0	0	0.016171047	0	0.018121737	0.053404518	0
ITIH2	0	0	0	0	0	0	0	0	0	0	0	0
ITIH3	0	0	0	0	0	0	0	0	0	0	0	0
ITIH4	1.212886842	1.818123979	1.110258301	2.252934185	0.547662978	0.87527988	0.724767657	1.587767257	4.031119737	3.414813014	2.771851195	6.41351994
ITIH5	0	0	0	0	0	0	0.013991193	0	0.014806133	0	0	0
ITIH6	0	0	0	0	0	0	0	0	0	0	0	0
ITK	0.024065099	0	0	0	0	0.01235699	0.024747392	0.023547821	0.013094422	0	0	0.012522115
ITLN1	0	0	0	0	0	0	0	0	0	0.099838197	0	0.047376395
ITLN2	0	0	0	0	0	0	0	0	0	0	0	0
ITM2A	0	0	0	0	0	0	0	0	1.245241017	2.123385336	1.801426995	1.129748777

ITM2B	103.8161253	112.4803256	102.8514363	112.0976998	92.34075134	85.42637345	113.005153	109.082649	143.0291606	137.5212712	122.6489215	151.3092609
ITM2C	122.5780379	153.7554448	121.0340398	152.9806785	110.2761542	132.3387266	117.474836	146.3968771	135.6843936	136.1479942	120.0217096	142.5426516
ITPA	18.04503856	16.40898538	17.49111636	15.69978074	15.7989961	16.33455214	18.82766732	17.86953315	4.571964179	4.691791219	5.126546535	5.469209653
ITPK1	7.268871101	7.938192003	7.493232329	9.218507483	7.714767688	7.812544475	7.055131508	9.567945154	11.18284998	8.156230133	11.1225549	12.22773883
ITPKA	0.55665063	0.961795202	1.03805562	1.205582555	1.35623307	1.578870245	0.572432771	1.608118993	2.942336069	3.226347563	3.397755042	3.668893372
ITPKB	2.058889788	2.385105421	2.074464733	2.717777352	2.861364745	2.825476878	2.275493015	2.652721062	2.240586906	1.984488081	2.012559155	2.020657434
ITPKC	3.742189107	3.604711428	3.861764945	4.365311997	4.395953529	4.307465475	4.185012697	5.05016496	3.750036167	2.906619426	3.023212593	3.94312733
ITPR1	11.8754249	9.214098252	10.50130655	7.969171563	7.807787794	8.14691391	9.162465928	5.97510437	10.98324848	8.324208787	9.701177363	10.3892454
ITPR2	3.069170864	2.305648676	2.332551725	3.14695873	1.997171094	1.906105845	2.079175166	2.081268528	8.748314048	5.201182168	6.202560007	9.691550614
ITPR3	1.559611473	2.336121473	1.550290609	2.229588133	2.730700705	3.118068634	1.677008063	2.210796307	4.181810731	3.231780257	4.714099328	2.906709428
ITPRIP	4.842785628	5.633505683	5.608926021	5.053455644	5.103627696	4.796567832	5.426525714	4.746014405	2.899810755	2.117558573	3.087958339	3.076862573
ITPRIPL1	1.069118587	1.295917239	1.201204924	1.238482366	2.103892047	2.136771514	1.792916948	1.432404286	0	0.009017939	0	0
ITPRIPL2	1.85212802	2.318602279	1.78277962	1.575255565	1.787993982	2.146106911	1.609959424	1.484047986	3.468316044	4.958543993	3.560967117	4.335023212
ITSN1	1.542338381	1.683984768	1.713474947	1.694267843	1.930287944	1.746811263	1.602939762	1.589461779	0.761849561	0.872606238	0.898425553	0.614715767
ITSN2	7.512998452	6.577644181	6.826963739	6.9914528	7.400049164	6.158346694	7.08060253	5.370186287	9.312687229	7.806299824	7.298259809	9.730440365
IVD	20.15948639	18.83075162	19.5772213	17.64088816	15.77584909	17.50022205	19.24299641	19.32161074	19.57158383	17.86786883	20.32096475	18.74424474
IVL	0	0	0	0	0	0	0	0	0	0	0	0
IVNS1ABP	15.74099776	17.01076295	16.33434577	16.39600585	20.69299062	20.36501509	14.53026778	14.46039621	9.536151383	11.38977305	9.777839295	11.4910649
IWS1	20.31895522	17.47619428	21.04442324	21.85821777	20.81656743	18.15332521	22.30796603	20.19245619	20.18556173	19.60052013	19.75223292	21.3656491
IYD	0	0	0	0	0	0	0	0	0	0	0	0
IZUMO1	0	0	0	0	0	0.033329589	0	0	0.035318611	0.035587681	0.069917616	0.033774969
IZUMO1R	0	0	0	0	0	0	0	0	0	0	0	0
IZUMO2	0	0	0	0	0	0	0	0	0	0	0	0
IZUMO3	0	0	0	0	0	0	0	0	0	0	0	0
IZUMO4	4.214741269	4.242138532	3.378187788	2.812312901	4.400943163	3.65601513	3.70303785	4.004024968	1.514053795	1.794809866	1.939403012	2.044066269
JADE1	3.764309192	3.572141126	3.971707117	5.718791303	6.524322341	6.30163124	3.761820623	5.594373634	6.273181705	6.185107662	7.035483109	6.097248525
JADE2	11.33937357	10.74271951	10.96777607	8.827716312	9.278831189	9.906158051	10.90183176	9.445805054	8.07971648	7.442993967	7.833716	8.652869118
JADE3	2.755049516	2.744578787	2.767905171	2.653996228	2.573355377	2.541981094	2.888495763	2.664238173	1.756747712	1.510511983	1.42585791	2.195156834
JAG1	25.69593027	28.76325099	24.43823203	23.08213018	33.11895981	32.8313765	19.65891132	20.00758509	0.606415891	0.778577848	0.30980032	1.010168878
JAG2	0.322765147	0.267687302	0.22449254	0.102525596	0.240731785	0.189410312	0.180183066	0.090236381	2.97056454	3.074092511	3.248239434	2.66798524
JAGN1	21.97763992	24.66599116	24.87395569	21.55749454	22.7454992	21.93773626	23.80078908	24.39162764	19.3665561	19.37191808	21.57851377	21.72487401
JAK1	26.74072689	25.97123026	23.47157307	32.3423994	23.01482211	24.2408683	22.78506913	25.97708139	33.21731834	25.57632224	25.49857587	39.96047677
JAK2	5.724219056	4.968227131	5.303784369	5.814282913	4.636726446	4.345450444	4.977133849	4.298577997	19.45388289	19.58123609	16.09079987	22.98735501
JAK3	0.18765322	0.121587013	0.074480534	0.015805048	0.351065438	0.208772439	0.55479898	0.290731078	0.068071208	0.077163524	0.126333269	0.032548038
JAKMIP1	0	0	0	0	0	0	0	0.010836166	0	0.012143316	0.011928731	0.011524779
JAKMIP2	0	0	0.012284897	0	0	0.005959929	0	0	0	0	0	0
JAKMIP3	0	0.00465139	0	0.031743199	0.018739864	0.027646356	0.004613955	0.03512244	0.009765406	0	0.004832961	0.004669299
JAM2	0.010517897	0.098134694	0.022264574	0.031891225	0.098843078	0.075610479	0.0432644	0.010291816	0.206029833	0.542065206	0.294566907	0.164187545
JAM3	0.02934	0.045624963	0.077634653	0	0	0	0.015085924	0	0	0.032172505	0	0
JAML	0.955800968	0.990874422	0.624199039	0.123322378	0.233580796	0.271498852	0.543731808	0.039798132	0.464748072	0.133796767	0.394297304	0.08465442
JARID2	27.74218195	26.32942637	25.30534424	20.03426532	24.38984287	24.51518753	24.06609044	18.56582021	12.0777924	9.721152282	11.3052984	10.22527963
JAZF1	3.49712658	3.74516329	3.561731265	2.450833778	2.377001291	2.608862851	2.985588457	2.146793215	4.882846696	6.584179112	4.833103432	4.926941743
JCAD	0.587501704	0.667066028	0.550754774	0.446753491	0.858839492	0.90788733	0.690466897	0.394198902	3.09931921	4.000296566	3.821120944	3.99451436
JCHAIN	0	0	0.040420711	0.077196833	0	0.03921963	0.471271834	0.186845026	0	0.376890965	0	0.039743718
JDP2	0.978003536	1.144392635	1.086376609	1.585459297	1.218374885	0.96956896	0.945985415	1.430734601	1.127538871	1.056493656	1.783597503	2.060783662
JHY	0.490451727	0.417654557	0.475842465	0.436686467	0.377992786	0.305803319	0.378267749	0.388498169	2.948245581	1.959129617	2.270418297	2.953066931
JKAMP	12.2895028	12.2338538	13.39051266	12.34779307	13.04032662	11.6729122	13.81052626	10.57309055	19.49972233	19.19180289	18.09231786	18.76047872
JMJD1C	20.79150237	15.57930972	16.88802249	11.4278672	22.26319476	15.52445898	19.34137789	7.692461372	55.38982967	43.22278379	42.65985041	35.1420986
JMJD4	12.07355782	9.876055835	11.80236541	9.081086669	9.381917923	9.680363595	11.01341023	10.79355594	9.297732951	8.224985466	10.47760949	11.58381992
JMJD6	6.394240781	6.306710293	6.438833868	5.139655415	5.840909763	5.222374453	6.323742496	5.233962774	5.779988972	5.77239154	6.025420366	6.566199199
JMJD7	2.081241805	2.432219089	1.602047294	2.447715042	1.896600798	2.253942754	1.673285667	2.036505667	3.870939637	2.531129983	3.301616031	4.213700545
JMJD7-PLA2(	0	0	0	0	0	0	0	0	0	0	0	0
JMJD8	14.20047435	14.34060104	15.66896032	18.19913447	18.08940834	17.17117664	16.06069507	21.72896457	13.13990158	11.85844011	14.41974091	16.9908854
JMY	3.30673279	2.694796546	3.14428763	3.252744228	3.200474892	3.220348774	2.988305155	2.71079815	2.995586159	2.64353041	2.590467203	3.269516166
JOSD1	10.51693178	11.07150628	10.91856629	11.4708892	10.85165629	11.60636699	10.62813112	12.1449134	7.446052605	7.933008538	7.772207818	9.61033921
JOSD2	6.099450703	6.912729778	7.385807374	7.000588465	5.883151826	5.839241539	6.591316827	7.940931553	10.2378509	7.255100784	8.407509368	10.92005424
JPH1	0.058552297	0.121401788	0.049578047	0.118357277	0.036683438	0.02405243	0.06021237	0.05729372	1.236159072	2.195810231	1.400163026	1.267439713
JPH2	1.589095632	3.408692248	1.232375099	0.288652909	1.804584169	2.098212839	0.79071756	0.272293315	1.083821851	4.641334836	1.514513759	0.083830687
JPH3	0	0.012225317	0	0	0.036940696	0	0	0	0.01283328	0.077586291	0.012702543	0
JPH4	0	0	0.025864115	0	0	0	0	0.011955706	0.039889821	0.066989526	0.0263223	0.025430926
JPT1	22.36768819	22.45640332	24.58984915	31.7960533	24.5909414	24.56624716	27.99436643	34.68736223	19.30808673	22.04920682	23.23792712	22.92868299
JPT2	17.283929	18.34226755	20.9842314	26.34350822	25.64485705	25.73219833	22.80716395	27.2914581	33.32557882	30.32725513	37.15655236	35.60940024
JRK	4.045331197	4.117107387	3.733710374	4.111028413	5.011714171	4.841745786	4.156221722	4.121198829	6.193042717	5.761765802	5.65994921	6.060903358
JRKL	1.982159436	2.03812082	1.909557996	1.316495586	2.002145734	1.769730518	1.988438599	1.250813814	3.724273453	4.293808027	3.346458308	3.098420504
JSRP1	0.033609969	0.104529897	0.07114651	0.033969499	0	0.034516214	0.103688634	0	0	0	0	0.034977451
JTB	90.14981702	90.58717602	94.90924102	106.1789219	91.95501479	88.22714891	97.20567928	116.411811	108.187682	98.89043172	113.4929928	124.1297065
JUN	17.29419531	25.58381025	21.28659476	23.0273645	2.41790918	2.660750337	8.425999081	11.53515854	11.33101393	10.44073792	7.709621733	10.27966545
JUNB	123.2641923	168.8882715	131.3426476	125.541094	69.42906174	70.44491277	87.29914976	110.0318629	24.76472545	20.30712223	24.92736264	14.77063466
JUND	99.97120146	110.9146342	113.6734575	70.12168198	71.18521049	55.48566675	71.09286469	65.50099387	18.46539028	18.12513384	20.40322834	18.2288825
JUP	0.358647958	0.282061751	0.314150023	0	0.154962442	0.114305706	0.203484874	0.024202679	0.471050103	0.325466556	0.373001081	0.154444217
KAAG1	0	0	0	0	0	0	0	0	0	0	0	0
KALRN	0.005652562	0.008789977	0.008974124	0.002856514	0.005902285	0.014512437	0	0	0.384462531	0.288219277	0.298347968	0.314716223
KANK1	2.064829835	1.656122011	1.88765865	1.378425932	2.086340715	1.890334722	1.701639778	1.273861169	2.428683473	2.238024833	2.31661896	2.079363578
KANK2	11.10114194	12.31925677	11.95384915	16.03428338	15.45189989	13.97678642	13.3481209	17.13356898	28.49437273	19.65698768	21.30076199	30.41993787
KANK3	0.015226248	0	0	0.015389125	0	0	0.015657942	0	0.165699652	0.066784804	0.065604645	0.126766042
KANK4	0	0	0	0.008866183	0	0	0	0	0.152744068	0.125050027	0.122840259	0.146068207
KANSL1	4.616902062	4.063359541	4.067670976	4.084932728	4.804925358	4.380689081	4.08823821	4.094286925	7.60022029	5.665446974	6.195887346	6.83895359
KANSL1L	1.33867688	1.210923617	1.259443619	1.379526228	1.452634306	1.442163187	1.223672005	1.228568328	3.127867877	2.552059118	2.073426798	3.000266206
KANSL2	17.40300837	16.91985928	17.5844993	18.75114946	18.52493109	17.40924375	17.10823372	17.77891086	11.75090286	10.01121559	12.09255485	12.26955345
KANSL3	7.117578895	7.860920375	6.583984133	8.70464884	8.03040215	9.586781205	7.191265836	8.760589093	11.02655867	11.07413478	10.31483938	13.55244226
KARS	106.1730044	99.98584448	110.597512	98.16204683	113.7618277	112.3892811	121.5358889	107.5653451	77.82384749	80.71859794	88.13595858	75.07784747
KAT14	6.389342041	7.284679328	7.360617939	8.346673695	8.940871834	9.001770984	6.972767519	8.846372449	3.37411605	3.129741989	2.996404713	4.749502729
KAT2A	30.04407292	30.51396451	31.32317993	31.30677118	33.40020975	37.2855556	29.7069433	38.02449613	16.91566483	24.7216179	21.18862637	20.73847611
KAT2B	3.474252969	3.1408047	3.370764151	3.584572027	2.979930135	2.58037883	2.711465759	2.600269519	10.95998124	8.424337075	7.451264364	11.56779713
KAT5	16.6971592	16.60044656	16.87579234	20.74889858	17.48245451	18.34206827	15.78659092	21.98522018	14.09965664	13.08151241	12.67835485	13.79201366
KAT6A	12.78773342	10.90467421	12.00237657	15.70736892	15.19423847	12.95411982	11.19046982	11.20954623	19.79442111	18.06327046	16.90478804	19.81137053
KAT6B	6.173509183	5.320078485	5.243590139	5.473812481	7.367139365	6.254868756	6.652880705	4.795580756	4.154666792	3.342564381	3.780804122	2.858155215
KAT7	16.48365727	16.39103984	16.14616768	19.24693925	18.35730686	18.11473581	15.0558584	17.50327027	23.25458374	23.81666102	21.77305639	26.43927352
KAT8	10.29249734	11.94661741	10.01815102	13.39699005	11.55073622	12.94086534	10.64366118	13.51619683	12.54072077	14.7247241	12.16429033	12.79345597
KATNA1	12.95608844	11.67675402	13.80137302	17.46661531	15.53592257	14.59305314	14.97810175	15.0904315	19.7165083	18.95014275	19.42561175	20.35532789
KATNAL1	1.550244705	2.082411218	1.51073693	2.407564776	1.904971829	2.295845139	1.506710763	1.734286177	1.368159541	1.622166813	1.389859034	1.023080453
KATNAL2	4.416019277	4.141759206	4.435112305	3.180996032	4.923191492	4.547618785	4.534949714	3.205012753	2.396228837	2.039938129	2.34553709	1.713863374
KATNB1	5.165717843	5.470442902	6.011274207	5.964824943	6.466919275	7.344293203	5.911937936	8.044008663	5.289132896	5.649193002	6.19255363	7.167859051
KATNBL1	5.065828271	4.598337545	4.417773754	6.790634311	5.562803489	5.214633328	4.683617524	5.585283005	8.010510745	9.766690914	7.749575848	9.454841704
KAZALD1	2.43778121	4.283973987	2.517250002	3.560575955	4.827097893	5.526045857	2.323465613	3.01081662	4.723483719	6.503520819	6.500677261	3.960143122
KAZN	1.450068265	1.104944727	1.184674433	1.087365681	1.36760168	1.345054316	1.384667659	1.248892805	0.319970607	0.271116032	0.309512978	0.312940136
KBTBD11	16.65075616	18.8929567	16.1126129	23.4651369	18.92534982	24.36778272	14.95176035	21.43146336	20.2443228	24.65734928	18.2287949	36.79828935
KBTBD12	0.005712268	0.011843763	0.006045943	0	0	0	0.005874222	0	0	0	0	0
KBTBD13	0.120610607	0.089311754	0.036473123	0.017414399	0.19790419	0.017694671	0.088592968	0	0.056251928	0	0.074238493	0
KBTBD2	12.21393786	10.07311556	12.118535	12.23424919	14.2212592	11.29594968	12.47602441	11.11012842	23.83616601	19.21420661	22.12334872	16.21880357
KBTBD3	0.682522176	0.770743244	0.574042756	0.566640488	0.579048548	0.782282822	0.777073705	0.626110699	0.424431308	0.447711561	0.341337326	0.399539142
KBTBD4	6.268516709	7.770428067	7.689741401	8.873675532	7.406168683	8.524324389	6.64337427	8.928676125	9.262510202	7.146949511	7.784667716	7.979892959
KBTBD6	2.037929046	1.899306362	2.037140418	2.787916064	3.406888899	3.044187465	2.751940254	2.487619223	4.961994621	4.5934928	5.166441068	4.220268964
KBTBD7	2.888115769	3.722072527	2.457444784	4.590286804	3.701635018	4.047703553	3.144705394	4.255677642	5.33692387	4.83113063	4.208963958	5.021159946
KBTBD8	8.271324471	6.109576095	7.939830528	7.30321756	6.189664409	5.79267403	7.619808557	6.148836847	2.312826122	1.41086463	2.078899704	2.462803834
KCMF1	4.872597167	4.668613542	4.871099295	5.524482878	5.308175682	5.078463905	5.295523357	5.490459748	8.014896843	7.222812152	7.10938186	8.22039956
KCNA1	0	0	0.007134258	0	0	0	0	0	0	0	0	0
KCNA10	0	0	0	0	0	0	0	0	0	0	0	0
KCNA2	0	0	0	0	0	0	0	0	0	0	0.004502929	0
KCNA3	0	0	0	0.021063195	0.02176096	0	0.021431126	0	8.595499391	7.746895005	7.250825051	8.501769305
KCNA4	0	0	0	0	0	0	0	0	0	0.014119257	0.013869754	0
KCNA5	0	0	0.019844175	0	0	0	0	0	0	0	0	0
KCNA6	0	0.012061432	0	0.005879478	0.018222746	0.011948207	0.011964361	0.017076625	0	0	0	0
KCNA7	0	0	0	0	0	0	0	0	0	0	0.026514961	0.012808531
KCNAB1	0	0.003408537	0	0	0.003433142	0	0.003381105	0.003217214	0	0.014421208	0	0.006843321
KCNAB2	64.76633893	69.02538976	63.47774193	72.36136918	45.8280802	59.75406623	57.48481391	76.33157856	61.03553641	79.79916702	67.52180688	104.7064184
KCNAB3	0.272606838	0.31401138	0.320589819	0.505125405	0.537672714	0.388829571	0.280335779	0.444578624	0.412033894	0.232496831	0.44046327	0.252176296
KCNB1	0	0	0	0	0	0	0	0	0.038838174	0.004891757	0.038442516	0.055711058
KCNB2	0	0	0	0	0	0	0	0	0	0.014110253	0.009240606	0.031246895
KCNC1	0	0	0	0	0	0	0	0	0	0	0	0.020080887
KCNC2	0	0	0	0	0	0	0	0	0	0	0	0
KCNC3	1.541772682	1.229498904	1.326985459	1.575389015	1.167609795	1.148359655	0.940837237	1.409162125	3.171291	2.972512562	2.42723759	4.35507809
KCNC4	2.759692188	2.382600185	2.310419558	1.82958021	2.409064585	2.196202262	2.226546992	2.023108888	1.313311834	1.576304196	1.424190938	1.93927585
KCND1	0.442472405	0.416236023	0.312212597	0.579710949	0.376460962	0.30293536	0.21065619	0.416925883	1.774492638	2.048575811	1.368062139	2.822542335
KCND2	0	0	0	0	0.00610063	0.00600005	0.012016323	0.00571693	0.089013637	0.070472108	0.050346757	0.158085925
KCND3	0	0	0	0	0	0	0	0	0.009868815	0.02651733	0.006512185	0.044041603
KCNE1	0.143135913	0.13849578	0.020199603	0.048222352	0.05978379	0.009799691	0.039251759	0.074698249	0.010384511	0.020927248	0.051393601	0.019861287
KCNE1B	0.059738619	0.046448061	0.031614089	0.015094413	0	0.030674693	0	0.029227271	0.016252639	0	0.016087067	0.015542298
KCNE2	0	0.138939282	0	0	0	0.068817508	0	0.131140544	0.218773065	0.073479918	0.144362898	0.209211328
KCNE3	12.81255251	14.28178336	12.75835941	12.6781632	10.87113925	11.94119128	12.31475551	10.79029477	14.75131666	14.36131131	13.52893553	14.05726909
KCNE4	0	0	0.017937884	0	0	0	0	0	0.036887091	0	0.018255655	0
KCNE5	0.073260124	0.113922645	0.038769763	0.185109491	0.076496663	0.150470956	0.226011577	0.25089891	0.039862666	0	0.118369715	0
KCNF1	0	0	0	0	0	0	0.024174385	0	0.281407089	0.463992459	0.227896607	0.269107858
KCNG1	1.492119539	1.396482923	1.721853777	1.005387288	2.715749366	2.75610606	2.088521645	1.26740151	2.356764053	3.170079038	3.705620184	2.674316756
KCNG2	0.10990779	0.087131273	0.130013558	0.04574026	0.087760229	0.039836928	0.066484642	0.069588155	0.01407143	0.007089316	0.02089212	0
KCNG3	0	0	0	0	0	0	0	0	0.107193462	0.061720057	0.060629397	0.043932191
KCNG4	0	0	0	0	0	0	0	0	0	0	0.041955631	0
KCNH1	0	0.006604016	0	0	0	0	0	0	0	0	0	0

KCNH2	0.025187811	0	0	0	0.061368012	0.008622322	0.008633978	0	0.228421973	0.276194606	0.135656975	0.078637867
KCNH3	0	0	0.024789023	0	0	0.012026215	0	0	0.050975632	0.025681991	0	0.024373841
KCNH4	0.03615424	0.024987289	0.025510764	0.012180329	0	0.024752725	0.049572379	0.035377107	0.078689707	0.185008117	0.116832101	0.087792227
KCNH5	0	0	0	0	0	0	0	0	0	0	0	0
KCNH6	0	0	0	0	0	0	0	0	0	0.011481684	0	0
KCNH7	0	0	0	0	0	0	0	0	0	0	0	0
KCNH8	0	0	0	0	0	0	0	0	0.037489281	0.075549775	0.102045253	0
KCNIP1	0	0	0	0	0	0	0	0	0.025360874	0.063885204	0.012551257	0.012126223
KCNIP2	2.4442309	2.269184611	1.891990701	1.825129387	2.476028414	1.92943296	2.025829875	2.034723332	1.528469761	1.600118626	1.336066389	1.423700818
KCNIP3	0.035670905	0	0.018877289	0	0	0.01831636	0	0	0.155275458	0.273802201	0.096058508	0.167050079
KCNIP4	0	0.004782589	0	0	0	0	0	0	0	0	0	0
KCNJ1	0	0	0	0	0	0	0	0	0.684656376	0.852195228	0.478363441	0.365880013
KCNJ10	0	0	0	0	0	0	0	0	0	0	0	0
KCNJ11	0	0.028324002	0.01445869	0	0.042792687	0.028058116	0	0.01336708	0.163529025	0.599181258	0.220722405	0.213247901
KCNJ12	0	0	0.009728866	0	0.009598018	0	0.00945254	0	0	0	0	0
KCNJ13	0.029819651	0	0	0	0	0	0	0	0	0	0	0
KCNJ14	3.000954113	2.504513356	2.676840651	3.01397988	3.48827196	2.500385533	3.202491301	3.564368701	1.4377685	0.931321237	1.280809306	1.708840477
KCNJ15	0	0.007982845	0	0.031130606	0	0	0.023755796	0	0.025139492	0.025331014	0.016588925	0.016027161
KCNJ16	0	0	0	0	0	0	0	0	0	0	0	0
KCNJ18	0	0	0	0	0	0	0	0	0	0	0	0
KCNJ2	0.039947707	0.031060201	0.021140602	0.010093758	0.010428136	0	0	0.009772258	0.173892374	0.098559645	0.129090654	0.384550716
KCNJ3	0	0	0	0	0	0.011312274	0	0	0.035962082	0.072472108	0.035595724	0
KCNJ4	0	0	0	0	0	0	0	0	0.248245084	0.027792922	0.191112542	0.026377249
KCNJ5	0	0	0	0.017520995	0	0.008901491	0	0	0.301846686	0.342164542	0.308108283	0.279633677
KCNJ6	0	0	0	0	0	0	0	0	0	0	0	0
KCNJ8	0.020791948	0	0	0	0	0	0	0	0.090507352	0.113996086	0.179170645	0.043275811
KCNJ9	0	0	0	0	0	0	0	0	0.00539608	0.092432212	0.069434404	0.061922852
KCNK1	0	0	0	0	0	0	0	0	0.760230004	0.910944727	0.894847355	1.37541155
KCNK10	0.016468954	0.028455479	0.017430968	0.033290248	0	0.011275344	0.011290587	0.010743303	0.698971238	0.397295318	0.644544552	0.839812092
KCNK12	0	0	0.039966864	0	0.157717329	0	0	0.11084827	0.205467569	0.165626314	0.244049279	0.157189891
KCNK13	0.234696737	0.15483301	0.225823875	0.12938581	0.066836002	0.021911363	0.175527892	0.02087745	2.089707825	0.935834645	1.700700266	2.90874534
KCNK15	0	0	0	0	0	0	0	0	0.12743908	0.243978915	0.18921122	0.121869203
KCNK16	0	0	0	0	0	0	0	0	0	0	0	0
KCNK17	0	0.032489296	0	0	0	0	0	0	0	0	0	0
KCNK18	0	0	0	0	0	0	0	0	0	0	0	0
KCNK2	0	0	0	0	0	0	0	0	0.06843202	0.068953359	0.067734879	0.046743655
KCNK3	0	0	0	0	0	0	0	0	0	0	0	0
KCNK4	0.028040417	0	0.059356729	0	0	0	0.028835418	0	0.030514985	0.061494919	0	0.029181292
KCNK5	0.341377071	0.516110034	0.376373125	0.776314857	1.143638249	2.249566645	0.672856918	1.099545688	6.098850987	6.082925191	7.155198416	6.972107151
KCNK6	9.596775233	9.072671315	10.12983254	6.913146129	7.984011019	7.625355689	8.732205081	7.303716979	4.542581082	3.721638885	4.412261153	3.96512261
KCNK7	0.206562614	0.142761668	0.07287624	0.243567614	0.071896095	0.141421519	0.035403178	0.101061283	0.074930591	0.037750719	0.148334494	0.143311321
KCNK9	0	0.020722185	0.021156308	0.010101257	0.020871768	0.01026383	0.010277706	0	0.054381739	0.043836831	0.03229664	0.052004921
KCNMA1	0	0	0	0	0	0.004659791	0	0.001479971	0.339067402	0.336675043	0.31606292	0.374616688
KCNMB1	0.861228567	1.321390847	0.692767506	0.208905897	0.251797433	0.353780144	0.354258437	0.134834644	14.43337322	14.99663036	17.21781722	6.955048895
KCNMB2	0	0	0	0	0	0	0	0	0	0	0	0
KCNMB3	0.488337933	0.273652792	0.293355018	0.133395066	0.592600525	0.406625891	0.407175628	0.193719383	5.1276178	3.270784548	3.980690955	3.571183149
KCNMB4	0.00575688	0.005968131	0	0	0	0	0.005920099	0.005633137	0.100238805	0.227255534	0.111619842	0.161759952
KCNN1	0.036680001	0	0.019411309	0.055608557	0.019150238	0	0.018859975	0	0.059875518	0.020110557	0.019755182	0.076344784
KCNN2	6.682233048	3.646021026	6.05736652	2.606696534	5.063377575	3.414787695	5.753805335	2.648810417	12.17789065	8.285621195	10.54997141	6.355192916
KCNN3	0	0.008428514	0.008605089	0	0	0	0	0	0.004423831	0.008915068	0.017515057	0.012691448
KCNN4	21.42385783	20.40533856	21.90912567	9.790027147	19.81475506	17.8065411	20.84374037	12.07543135	7.784009187	12.56819633	12.03048814	2.834024989
KCNQ1	3.728464012	5.455932062	3.410346186	5.070986636	1.489983539	2.395092587	2.051203828	4.143771947	8.632625863	9.08536132	7.453871785	12.50274816
KCNQ2	0	0	0	0	0	0.005201964	0	0	0	0.011108797	0	0
KCNQ3	6.295885051	5.773198009	6.077250227	2.777689508	6.482564468	6.274486383	5.81681339	2.627075131	2.630974923	2.588585201	2.439052645	1.255713546
KCNQ4	0.013013195	0.013490719	0.02754669	0.052609595	0.040764304	0.026728154	0.026764289	0.038200432	2.02511022	3.153559064	3.238005694	1.340723334
KCNQ5	7.654470903	7.809145995	7.231843554	9.428198063	9.478344283	10.56449943	7.362894934	7.944099904	6.06117706	5.998889304	6.63871323	4.386791566
KCNRG	0.629157942	0.308958212	0.595813712	0.468549145	0.172882457	0.442083669	0.715100631	0.421223427	0.288286804	0.181551921	0.214012444	0.206765165
KCNS1	1.04882348	0.840966659	1.205487529	0.505176684	1.026711715	0.950880435	1.25551089	0.569264186	0	0	0	0
KCNS2	0	0	0	0	0	0	0	0	0	0	0	0
KCNS3	0.147171449	0.133500462	0.272594509	0.24171186	0.115254972	0.132247251	0.151344049	0.198011013	1.881872193	2.158450617	1.902332838	2.776013789
KCNT1	0	0	0	0	0	0	0	0	0	0	0	0
KCNT2	0	0	0	0	0	0	0	0	0	0	0	0
KCNU1	0	0	0	0	0	0	0	0	0	0	0	0
KCNV1	0	0	0	0	0	0	0	0	0	0	0	0
KCNV2	0	0	0	0	0	0	0	0	0	0	0	0
KCP	0.032371521	0.025169554	0.025696848	0.032717803	0.076053721	0.099733118	0.091545623	0.079189244	0.713373262	0.346092736	0.50560668	0.471640598
KCTD1	1.413430861	1.619539017	1.312276067	1.666642264	1.191056561	1.540671771	1.313253984	1.552897425	2.10485901	1.563479965	1.896442839	2.038669589
KCTD10	21.0227898	21.00438852	21.70410768	18.53298282	18.18958276	16.40441259	20.66271208	18.65028131	18.24060899	19.49820578	18.11042433	20.11767496
KCTD11	0.950823392	0.912698326	0.857273541	0.551682011	0.680272104	0.831800096	0.706175245	0.740862539	2.261084533	1.428770845	1.631121242	2.56539458
KCTD12	3.149028333	3.720730259	3.634312483	5.894560522	5.468235814	5.714766037	4.231982536	5.073658798	15.7544902	14.14326433	14.33011817	24.15216256
KCTD13	6.637302661	6.389370767	6.403753048	7.004928366	5.421843375	4.938316625	6.210261132	6.571951406	4.680238293	4.567361891	4.206995902	5.697391638
KCTD14	0	0	0	0	0	0	0	0	0	0	0	0
KCTD15	7.442426142	7.592760089	8.012148512	7.485211117	10.61528141	9.701218137	8.918076828	8.708635682	1.913279772	1.957822477	3.120335494	2.218341346
KCTD16	0	0.003280647	0	0.003198375	0	0	0.003254244	0.003096502	0.010331379	0.017350145	0.02045226	0.023052946
KCTD17	2.934435555	3.456131451	2.223712984	2.351601353	1.559233521	2.300290144	3.106911652	3.126214525	6.065569705	7.444566108	7.200792484	10.71550404
KCTD18	9.200692332	10.12037751	9.469765723	8.951311829	9.94757862	8.872177293	9.740926557	7.921864342	10.6827921	9.512990681	9.130285194	11.79916212
KCTD19	0	0	0.019098854	0	0	0	0	0	0.019637242	0.059360538	0.038874382	0.037557947
KCTD2	5.171794962	5.005575801	5.374774178	7.172826336	5.465472773	4.990646713	5.939086864	6.526885913	7.508049591	6.629576774	6.377917111	9.031731122
KCTD20	30.34251297	30.39615643	30.93719509	38.1464229	34.0823755	32.05255246	30.63529049	31.40763109	25.33127025	25.02825092	24.48852745	28.07476737
KCTD21	1.093716261	1.06091281	0.988363969	0.943805374	0.667856872	0.532045304	0.848476959	0.757280914	1.586982362	1.171250507	1.336570844	1.717308439
KCTD3	17.3979796	16.44455002	17.17062761	19.91873669	21.30358023	21.43228207	18.37182497	17.63807773	27.12860197	29.67788258	26.8953811	24.20597563
KCTD4	0	0	0	0	0	0	0	0	0	0	0	0
KCTD5	19.35658584	16.73750139	22.26777442	19.05578583	19.59580188	17.02910599	22.12957384	21.67684503	15.78671648	12.96035994	15.41409496	16.96109563
KCTD6	0.974516647	1.178656359	0.790772092	1.132682352	1.628111279	1.217631595	1.252682641	1.366782927	2.297788536	2.083764503	2.08193261	2.417097052
KCTD7	1.419093275	1.365073601	1.35756598	1.275675947	1.624266526	1.653539777	1.6066633	1.655626502	1.893287125	2.446358617	1.837254459	1.938341496
KCTD8	0	0	0	0	0	0	0	0	0.271843356	0.290026963	0.063311528	0.152918894
KCTD9	20.34070996	16.80738528	20.90947203	20.90999428	23.68367702	18.32269173	21.09632944	18.40221393	24.30460287	26.36291858	25.47111904	16.80625388
KDELC1	8.155137891	10.31386428	9.112445009	15.25201839	8.690224855	9.848641727	9.861956586	13.90037308	3.279262396	4.510556617	3.786831306	6.172323072
KDELC2	6.797121594	8.443598385	7.819746994	12.74801733	11.34351229	11.6178073	8.181144109	11.15057621	10.05987632	10.81055541	11.76551278	12.24053259
KDELR1	50.76830116	52.98954142	51.20995108	53.61672049	50.44903307	51.24990505	55.15746984	57.04912658	91.69237683	114.2191293	109.5576711	93.72712291
KDELR2	20.58309776	21.70899561	23.27895152	25.80443139	19.74396246	20.88690759	22.09537123	24.74319429	56.85883911	78.04669516	67.00048682	55.68561828
KDELR3	0	0	0.089972798	0	0	0	0	0.027726661	4.656291109	6.214257435	5.310867002	2.919373873
KDF1	0	0	0	0	0	0.027755127	0	0	0	0.059271094	0	0
KDM1A	33.04707417	33.99954761	34.87417005	36.30375598	44.07293067	43.50382823	38.51522698	36.36176574	49.08939145	51.57340106	50.33160636	45.85553779
KDM1B	50.51151779	34.74837922	41.56649416	23.91245296	22.28665671	19.69727278	39.33956404	20.03809552	7.687282605	7.630428763	6.727135352	9.335178311
KDM2A	21.04516059	20.9510995	19.11162409	18.65541688	17.78655273	17.27361334	18.38318287	16.87719656	22.17487483	18.74446331	19.46459001	25.12958412
KDM2B	20.8478544	19.22725711	20.88937514	20.9056826	20.68351353	20.49692383	19.15318835	19.36982954	7.363357558	6.315496756	7.168632526	6.408815782
KDM3A	18.42752	27.76740359	16.38585607	17.60175475	23.63612255	24.69741165	12.68484016	14.51142159	22.12564312	23.887432	16.61137094	18.2491756
KDM3B	26.06961408	25.68817302	25.68573359	28.48315077	27.91322102	28.55879779	24.85681861	25.73888017	34.96519839	34.22495906	31.76146717	32.58940534
KDM4A	17.21769028	15.53107544	15.53038099	16.69841479	12.84337763	12.64334919	15.12447655	15.25103904	105.618434	88.14372716	90.88778595	109.4210884
KDM4B	7.745678793	11.57543996	7.256531981	6.541726547	8.293685453	8.24995263	6.398522531	6.828565103	8.336478928	9.100961809	7.195720858	7.273592246
KDM4C	3.03044471	3.041249079	2.887145119	2.948667184	2.907303416	2.668746555	2.560314854	2.633633535	5.203714632	5.690260623	4.850788248	5.350026031
KDM4D	0.036186658	0.131300892	0	0.073147503	0.075570677	0.130068329	0.093031553	0.088522072	0.295350994	0.357121297	0.565194824	0.244783351
KDM4E	0	0	0	0	0	0	0	0	0	0	0	0
KDM5A	13.68081766	12.5818008	13.26060052	12.87356566	12.584474	12.36169727	12.82779086	9.693853083	10.13258878	8.418305901	8.03953863	9.116099811
KDM5B	4.660546228	5.120914675	4.347540558	3.566066056	4.503522028	4.596925992	4.305290513	3.375187713	16.36169739	13.98596542	12.81987182	18.04701251
KDM5C	15.49819267	15.7763251	15.60032988	13.77075861	15.00503757	15.87395462	14.5596662	14.5630066	21.09912553	23.58375832	21.38493043	20.41885906
KDM5D	16.5405106	14.70697228	14.94042321	13.70545458	12.59438645	12.29619904	13.01097262	12.83754831	24.47683332	16.6871293	16.94309017	22.57206147
KDM6A	5.477671219	5.252775576	5.254113752	5.605469899	5.54092813	5.51989311	5.140088761	4.798811505	0.055885087	0.046925699	0.009219294	0.035628375
KDM6B	6.43721991	5.599883225	5.462442979	2.936222043	4.015430215	3.345634754	4.754691425	2.870085763	1.924935944	1.503804402	1.416935437	1.677695086
KDM7A	2.415508725	2.182011298	2.295982685	2.091737254	3.269094625	2.727499208	2.664866442	1.612058494	3.304987705	2.372261292	2.058783547	4.380824729
KDM8	0.753497656	0.764166051	0.780175086	1.142336875	1.40253196	1.042967559	0.791705598	0.881555878	0.641734324	0.538852734	0.829284649	0.579592865
KDR	0	0.009563539	0	0	0	0	0	0	0.020078264	0.101156136	0.01987372	0
KDSR	5.906043207	6.181829615	5.42694581	5.911639996	5.363871194	6.396834449	5.292334743	5.695472924	5.04260269	6.153447192	5.942429826	5.770841172
KEAP1	25.93923545	25.55645019	28.06208817	30.20812145	27.15786096	26.15625973	27.26504877	33.97841737	23.74136901	20.69826905	24.99867853	27.22998508
KEL	0.060596352	0.020939986	0.085514687	0.122489118	0.147637984	0.145203908	0	0.118587676	0.373682571	0.310083048	0.195816566	0.294288507
KERA	0	0	0	0	0	0	0	0	0	0	0	0
KHDC1	1.568114564	1.806285646	1.782655864	2.083835386	2.577376084	1.55075221	1.522986272	1.846972661	5.688335818	4.999291297	4.62942911	5.228175661
KHDC1L	0.254218398	0.087849011	0	0.085645935	0	0.087024344	0	0	0.184435455	0.371681098	0.091278273	0.088187242
KHDC3L	0	0	0	0	0	0	0	0	0	0	0.056713996	0
KHDC4	40.46507752	41.68893587	40.3161734	36.42642977	45.16351962	43.42157022	45.6811045	37.60672053	48.22444699	54.62637598	50.87146852	51.41342751
KHDRBS1	77.97920801	81.30362135	85.80334306	94.5534674	95.47092962	92.19630449	92.78016219	90.0670942	91.21886171	93.75003462	95.30010405	81.90710307
KHDRBS2	0.189404686	0.312236573	0.200468532	0.30440631	0.346912144	0.312494226	0.22351193	0.291672309	0.378449209	0.609450837	0.347837114	0.39099058
KHDRBS3	0	0	0	0.044323691	0.030528008	0.045037049	0.030065291	0.028607948	0.111357729	0.192353299	0.062984736	0.167342539
KHK	0.353376221	0.532863252	0.425020756	0.633140769	0.603798433	0.742304667	0.412949014	0.880168354	0.821563607	1.109634503	1.003516063	1.153410155
KHNYN	12.11645437	12.58421888	10.67372319	11.56907183	10.54571034	10.81515199	10.1868753	10.79275792	24.15224378	24.02612422	19.77752738	24.61473036
KHSRP	82.92468382	87.68622324	88.50945702	102.7882169	98.62821887	103.7110934	97.07015871	114.2095125	66.41591456	69.45334616	80.54057818	63.33421785
KIAA0040	0	0	0	0.010672129	0.088205335	0.054219445	0.086868395	0.072325451	3.860983982	4.145126769	3.63966742	4.61529386
KIAA0100	61.45865047	55.48666442	58.90083456	55.22686909	53.55612527	55.98634052	55.95408119	53.23499347	61.04863943	55.74298027	57.62248183	67.8993021
KIAA0232	7.573649883	6.320396824	7.15803195	8.120455986	7.427023944	6.489155077	7.217382334	6.43831597	13.95222532	12.03711156	13.9237278	15.20886407
KIAA0319	0.006334263	0	0.006704271	0	0.006614102	0	0.006513851	0.006198108	0.013786523	0	0	0
KIAA0319L	6.172550326	6.52466272	6.340752484	5.707925501	5.503410474	5.882743338	5.724566404	5.81592891	13.16352113	12.88737023	11.81856104	17.75096443
KIAA0355	0.859827382	0.567241214	0.678403264	0.497714355	0.661117183	0.553888966	0.667173	0.298295207	1.046290764	0.934264498	0.724100307	0.732118085
KIAA0391	15.21148357	13.79626257	15.14663356	14.34352994	16.66437268	16.80853493	14.64651632	13.80351564	9.728882885	10.60438692	10.92960742	8.17166496
KIAA0408	0.013247061	0.006866583	0	0	0	0.006802124	0.006811321	0	0.021624172	0.021788912	0.014269253	0.020679062
KIAA0513	3.020238811	2.054609469	2.699849885	2.793775072	3.460619635	2.327473715	2.681676124	2.185173076	0.572734737	0.53550538	0.556685673	1.120076662
KIAA0556	4.586790501	4.610523521	4.190478221	4.698502687	3.778147583	3.779513005	3.920072368	4.556430514	4.932552971	3.806185703	4.131179742	7.006917652
KIAA0586	5.646270819	5.100183574	6.13836964	7.721184739	7.169524602	7.290930175	5.833774512	6.320699646	6.681386863	7.04660764	6.081957755	6.118809313

KIAA0753	3.352329899	3.624181866	3.646923029	3.642123084	4.579793752	4.091456044	4.26677255	3.863275157	2.421699532	2.290591373	1.94082012	2.06932964
KIAA0754	0.092522529	0.087924536	0.097927121	0.132475694	0.426694416	0.292969905	0.301294796	0.248967851	5.571383105	4.438644008	4.86682309	5.801290136
KIAA0825	0.100923094	0.086430589	0.102174116	0.086480534	0.105381747	0.087872376	0.090247359	0.109487864	0.066852777	0.057738929	0.054355345	0.089046606
KIAA0895	0.04352644	0.011280914	0	0.021996024	0.045449382	0.033525051	0	0.031943131	0.48551841	0.69206344	0.46885099	0.532244337
KIAA0895L	5.824093646	6.890979739	5.460766678	7.469962863	5.83351174	5.88361353	5.22429085	6.581616041	6.613638445	5.969854346	5.881408165	9.70098571
KIAA0930	65.46989199	71.41457498	65.73679523	85.48195757	39.8613567	55.58763713	55.5645025	86.12603773	75.19136768	67.80786892	69.20915681	117.6258694
KIAA1024	1.875592063	1.647721706	1.747150571	1.874994416	1.793025391	1.842190232	1.65022725	1.635246231	0.600654551	0.61643852	0.51746606	0.792462335
KIAA1024L	0	0	0	0	0	0	0	0	0	0	0	0
KIAA1107	0.310813348	0.248376956	0.212459232	0.157069079	0.135226952	0.19949624	0.206424814	0.145730127	2.755268172	2.222427096	1.841382643	2.318125295
KIAA1109	4.928709638	4.710094876	3.951980495	4.356489302	4.266878013	4.533971231	4.140768641	2.750438856	9.176749178	10.47168784	7.242799763	8.906230996
KIAA1143	14.64332545	14.77270043	14.1614691	15.35470407	19.53994325	18.8987365	17.97647486	14.46030191	16.57798232	13.86534663	14.50158098	16.75871588
KIAA1147	4.13148591	5.485624209	3.160254132	2.105932676	3.36447896	3.411956457	2.60660671	1.625479624	2.28310969	3.195579556	2.383255679	2.757098146
KIAA1191	13.19787303	12.35809232	11.77455162	13.18938557	10.8430664	10.96845015	11.32591155	11.95422521	24.55538884	23.34194376	22.61044787	24.0600704
KIAA1210	0	0	0	0	0	0	0	0	0	0	0	0
KIAA1211	0.01198031	0.018629897	0	0.024216929	0.012509584	0.012303342	0	0.005861397	0.202082374	0.348127779	0.174214182	0.292992123
KIAA1211L	0.083205615	0.091332926	0.067344566	0.108829699	0.040885427	0.065343457	0.070465013	0.052681664	0.106527664	0.032201769	0.063265458	0.086590984
KIAA1217	0	0.004151222	0.004238189	0	0	0	0	0	0	0.013172579	0	0
KIAA1257	0.006533855	0.023707659	0.010373283	0.016509371	0	0.010065046	0.006719102	0.00639341	0.042662806	0.025076232	0.014076062	0.040798178
KIAA1324	0.019199415	0.019903944	0.03386821	0.025873058	0.013365081	0.0197171	0.006581252	0.025048967	0.334300231	0.140352936	0.275745498	0.399611552
KIAA1324L	0.006320869	0.013105632	0.013380191	0	0.006600117	0	0.019500235	0	4.47114614	4.040825825	3.554094833	3.933671033
KIAA1328	0.864771199	0.891605352	0.765238916	1.194018807	1.011530141	1.179265077	0.806677603	0.850806956	1.259925615	1.518247272	1.114746035	1.352392832
KIAA1468	4.235222316	3.955763302	3.835366225	4.229446755	4.079308288	4.022434056	4.443652535	3.229300479	7.864967371	7.625624035	7.071687147	8.410091034
KIAA1522	0	0.008467535	0.017289855	0.008255186	0.008528658	0	0	0.007992248	1.244407406	1.325538464	1.416489722	1.249520056
KIAA1549	0.296658489	0.25196415	0.355600229	0.195071345	0.350817607	0.297316316	0.385931094	0.300773951	0.739028153	0.772093108	0.731499415	0.810877446
KIAA1549L	0.004372628	0.004533083	0	0	0	0	0	0	0.004758513	0.009589529	0.018840144	0
KIAA1551	71.7273247	49.48403266	56.81665276	27.77754578	37.78801426	29.5536961	54.26880447	21.21376571	34.70280935	30.58637272	30.11514869	28.70215445
KIAA1586	13.0782403	11.49533734	13.15818051	14.52448403	15.4140775	13.78018388	12.71449654	12.96353703	17.95243658	14.09839033	15.82772186	17.0085151
KIAA1614	0.9453906	0.644437507	0.890958071	0.104712719	0.351590076	0.279294736	0.412849628	0.240771513	0.46508363	0.426024364	0.571944609	0.188684617
KIAA1671	0.01966544	0.010193535	0.020814173	0	0.005133559	0	0	0	0.021400916	0.032345934	0.015887173	0.005116391
KIAA1755	0.032532987	0.236087577	0.063127831	0.076722326	0.056617089	0.105798943	0.022303574	0.04775054	0	0	0	0
KIAA1841	1.066111616	1.135375676	1.261742093	1.430157532	1.416814087	1.433268378	1.076404562	1.299252734	3.375115681	2.274304054	3.382491352	3.439413635
KIAA1958	1.985805183	1.78868484	1.842059894	2.126002495	2.38731139	2.206509375	1.715060583	1.516761105	2.346363521	2.345017484	2.114760595	2.483569927
KIAA2012	0	0	0	0	0	0	0	0	0.062615758	0.012618557	0.012395574	0.04790325
KIAA2013	17.2486384	17.3289122	18.27439835	17.81553142	17.14873528	16.84834691	17.98525591	22.48140372	25.15253995	27.45617419	26.52641727	32.35730556
KIAA2026	3.814702725	2.837486193	3.044973538	3.155091603	2.977465491	2.536620565	2.925797557	2.099647109	7.271782144	6.121089464	5.584896241	7.033358476
KIDINS220	9.176791566	8.974538982	8.05302485	11.59903635	9.427729919	8.842541563	7.95470206	8.747913857	16.06787054	12.73715795	12.33450552	17.802635
KIF11	23.49267358	24.26686192	28.96987674	58.72270174	53.69722996	46.11930355	29.60725702	54.34304792	44.40879243	43.67811012	42.40404275	37.25172908
KIF12	0	0	0	0	0	0	0	0	0.015565725	0.03136862	0.015407151	0.029770813
KIF13A	0.106366362	0.085765177	0.125088472	0.04180718	0.024681221	0.024274306	0.072921372	0.017346671	1.253992986	1.399620564	0.980244055	1.46362156
KIF13B	3.191890728	2.815134959	2.968654271	2.985285926	2.630258502	2.360617199	3.04355671	2.593022699	6.389823092	5.831220263	5.381791652	7.095920669
KIF14	8.526496135	7.353297771	9.582781373	18.27418206	18.61831968	16.11705508	11.97375711	14.27808737	11.08177119	15.45917574	12.55666965	8.01492156
KIF15	6.225067131	7.100936495	7.377634718	18.18268647	15.90311485	15.22714246	8.701149573	15.43513399	18.54746742	20.24616589	20.83236073	17.41186278
KIF16B	3.654159609	3.930665711	3.358716487	3.818208161	3.413954065	3.364722841	2.881892838	3.333654895	1.771547986	1.596748451	1.383563771	2.101567049
KIF17	5.646195991	7.391600313	6.017704808	3.623023677	5.127833745	5.151170125	3.807837321	3.67465532	22.02007666	21.91426495	25.51559624	12.85870273
KIF18A	2.966589155	3.275153684	3.873876533	8.060427136	6.986473052	6.739401987	5.388245464	8.004753065	10.18829819	12.64580624	10.87300694	7.965475825
KIF18B	6.146055846	6.033369704	7.162260586	13.78509261	12.21977705	11.29418721	6.926365625	15.30261128	17.99237666	19.15651504	19.53217591	16.94314424
KIF19	0	0	0	0	0	0	0	0	0	0.112084293	0.125832735	0
KIF1A	0	0	0.006107537	0	0.006025394	0	0	0	0.006279706	0.063275468	0.006215732	0
KIF1B	1.075116101	1.243439776	1.109469766	1.45334991	0.719174183	0.921237665	0.943213089	0.933655822	3.411267098	3.172000894	3.271564692	3.863560391
KIF1BP	10.16553417	7.967152897	9.884248443	12.4387237	10.31742474	8.86672849	11.59669009	10.58363647	7.817601069	6.450352259	6.934964544	8.406887584
KIF1C	3.959073463	3.472914129	3.617300284	4.227145541	2.946785943	3.343011014	3.507599624	4.622279458	8.278138717	8.074048458	8.864473859	11.99423025
KIF20A	9.813152413	9.289317874	10.61609032	27.48245051	25.80286863	22.00229148	13.85371964	28.65496445	36.25516981	41.03617392	35.15107641	27.41058171
KIF20B	19.20459056	16.6573982	20.02618729	31.26514809	33.46816749	30.12337555	26.30350771	25.06932448	21.35333653	21.2598707	22.38489982	16.99935565
KIF21A	0.186276324	0.134338643	0.23144568	0.114598489	0.05919741	0.133077564	0.049971554	0.126798142	3.234629793	3.943097864	3.06209482	3.270254779
KIF21B	17.02584779	16.43599864	17.70473932	18.63973069	21.55384318	19.13661367	17.21830639	17.63435367	1.871963285	2.382599439	3.212445967	2.333287452
KIF22	19.61909603	22.54667872	23.77032536	43.98243573	42.30995646	40.13419893	25.87309425	49.33438539	55.40396473	60.23678525	63.6325629	54.21366461
KIF23	10.64822024	9.903442373	12.9431469	23.55616295	22.83086355	19.9911579	13.41674645	22.90083561	21.30488507	27.92863584	25.09035356	18.98854699
KIF24	1.875277246	1.741953283	1.996958867	3.483479306	3.215635816	3.097836641	2.294082948	3.327633646	3.201575815	2.936698585	3.372811489	3.085519631
KIF25	0	0	0	0	0	0	0	0.013206152	0	0.014799191	0.014537673	0
KIF26A	0.007963528	0.008255753	0.008428708	0.008048715	0.016630694	0	0	0	0.485313363	0.157181995	0.317386863	0.082875387
KIF26B	2.5256014	2.589552033	2.094930527	2.288554141	4.038439069	3.813309066	2.528002593	2.176925308	0.896058079	0.768320037	0.750478911	0.869253855
KIF27	1.266551162	1.039247459	1.243560329	1.100340951	1.029866201	1.035026618	1.102934536	0.896534236	0.844590246	0.81556527	0.522491319	1.116163949
KIF2A	18.39685261	15.95292939	18.59709289	19.46600214	21.06361195	19.98189817	18.91843961	14.91269922	25.88425249	26.86564891	26.70966683	24.1241819
KIF2B	0	0	0	0	0	0	0	0	0	0	0	0
KIF2C	18.6334299	19.5545021	22.75849939	46.21042686	42.76953135	39.77624613	31.3555489	47.82964533	74.18597116	92.23826189	84.08227856	62.55804382
KIF3A	2.071875281	2.05585047	2.420023298	2.527803491	1.638008981	2.31011818	2.062132906	2.04906198	1.352831021	1.687693876	1.243402884	2.109969511
KIF3B	5.991548552	5.490850435	5.959741667	7.380590245	5.227319886	4.960430306	5.998563817	6.895846237	12.68634575	11.52881415	11.25874819	16.33453818
KIF3C	1.653608088	2.362129148	1.5365142	1.418659034	1.094222092	1.313139358	1.700491134	1.514583073	5.796189818	6.325285798	6.20315535	5.903046331
KIF4A	8.739122193	8.725176999	10.46433018	24.26247913	18.83712045	17.72852748	12.22020815	24.02779711	25.74689863	25.6415366	28.21463957	21.12555659
KIF4B	0.061454501	0.14016111	0.117079724	0.472050344	0.372182997	0.22720152	0.113754342	0.240534163	0.428018325	0.350414283	0.423657953	0.19186466
KIF5A	0.027684562	0.019133638	0	0	0	0	0	0	0	0.010119083	0.009940268	0.009603653
KIF5B	33.95011367	26.60529856	32.40819898	37.88479238	34.10320055	30.38372611	33.72256496	30.27262436	62.43265824	66.48284701	61.97743818	63.78597277
KIF5C	0.174249419	0.152852245	0.148961067	0.027094367	0.034989911	0.006882608	0.062027215	0.013115688	1.976496159	3.167378919	2.20902744	2.999069187
KIF6	0	0	0	0	0	0	0	0	0	0.004688468	0	0
KIF7	0.011397916	0.011816166	0	0	0	0	0	0.011152919	2.145854368	1.724762478	2.050329276	2.811213519
KIF9	2.075943712	2.052353992	2.22630954	2.000885099	2.713159157	2.6543092	2.219627327	2.219655895	2.199300917	1.914551748	1.8214843	1.230430583
KIFAP3	5.047072808	4.507807814	5.224487256	5.145906491	4.158124244	3.987046073	4.665447055	5.242499357	5.190692751	5.887057758	4.540393195	6.672307468
KIFC1	18.57961939	21.42957372	22.53058206	48.81392505	39.76583006	37.54514434	21.00702366	52.54187967	54.18270788	61.72437112	54.90557285	49.99238845
KIFC2	10.08998112	11.36237484	8.499197046	9.74145804	5.957986017	8.359770498	6.88716112	10.37968679	7.863708625	6.800299439	7.000492356	13.05685686
KIFC3	3.113517827	4.303692618	3.346312351	3.244076349	2.992705969	4.164474461	3.208860163	3.409763017	4.637389115	3.48946549	4.493901408	4.942551935
KIN	2.057586486	2.133090348	2.106481612	2.172435891	2.110122175	1.792333071	2.443387406	1.947445283	2.485743699	2.46439119	2.242742602	3.199208742
KIR2DL1	0	0	0	0	0	0	0	0	0	0	0	0
KIR2DL3	0	0	0	0	0	0	0	0	0	0	0	0
KIR2DL4	0.306704273	0.353287661	0.360688925	0.206656752	0.213502718	0.384968362	0.490622134	0.266765945	0.667541879	1.121045744	0.917696385	0.638366168
KIR2DS4	0	0.034691618	0.035418396	0.033821622	0	0.034365957	0.103237254	0	0	0	0	0
KIR3DL1	0	0	0	0	0	0	0	0	0	0	0	0
KIR3DL2	0	0	0	0	0	0.023365944	0	0	0	0.024948995	0.04901624	0
KIR3DL3	0	0	0	0	0	0	0	0	0	0.034245139	0	0.032500812
KIRREL1	0.020517118	0.01418	0.028954134	0	0	0	0	0	2.344414206	2.407270484	3.071940827	1.138767643
KIRREL2	1.917030121	2.705889159	2.247520008	1.341371656	0.939269585	1.51440007	0.925032952	0.880194198	3.642840327	4.301223233	3.653382253	3.990055823
KIRREL3	0	0	0.012308792	0.011753873	0	0	0	0.011379496	0.430296248	0.089265317	0.288117394	0.229950092
KISS1	0	0	0	0	0	0	0	0	0	0	0	0
KISS1R	0	0	0.025045198	0	0	0	0	0	0.025751212	0.051894788	0	0
KIT	0.114289471	0.064627289	0.076978079	0.084008756	0.119338634	0.064020612	0.064107165	0.040666483	0.067841194	0.205074095	0.111916786	0.086501485
KITLG	2.155150518	2.418712607	2.322894993	3.087454914	3.055537827	2.619364011	2.145089183	2.747277539	3.335120281	3.122039282	3.482174738	3.117337194
KIZ	8.385020252	8.12733192	7.816576826	8.635482852	8.755459753	9.871272793	8.015186901	8.182549672	18.71984822	16.84415416	13.68268258	18.98949146
KL	0	0	0	0	0	0	0	0	0.356451251	0.168359447	0.286666216	0.340872125
KLB	0	0	0	0	0	0	0	0	0	0	0	0
KLC1	27.4670716	24.04122534	24.05795132	27.02037034	26.27862621	23.23809024	25.89783981	23.44205739	33.85920754	34.2292634	30.02702571	35.26973078
KLC2	2.137659482	2.069339952	2.337446431	3.448257204	2.054713236	1.948145606	2.416637166	3.504654266	3.127420727	4.129219568	4.345984094	5.30376311
KLC3	0	0.045316102	0	0	0.022821608	0	0	0	0.047569665	0.047932067	0.023542528	0
KLC4	5.337588182	5.810125508	4.501847251	4.602340678	4.040712115	4.727801167	4.288218217	4.259892837	8.27729858	8.669582592	6.629841491	7.533639831
KLF1	0	0.130948642	0.033422994	0	0	0.032429847	0	0.061799212	0	0.138507921	0	0
KLF10	13.35507187	13.90722678	14.4521248	18.41590155	10.75584793	11.08591837	13.79530062	12.36479452	11.76379009	6.566986572	12.49869792	7.899677716
KLF11	2.46752409	2.908669288	2.956347819	1.506479298	2.707324794	2.971407339	2.820857022	1.59331487	8.628346536	9.792858219	9.822188143	6.947721853
KLF12	0.018132961	0.056395068	0.014394132	0.027490397	0.042601616	0.051210197	0.018647066	0.008871597	2.096652001	2.23689709	1.987397927	2.533395845
KLF13	30.42231316	28.75769004	27.02477855	26.51668381	21.13021299	21.29401746	27.62484508	23.35775882	30.4345049	23.52386165	26.5349184	31.82524134
KLF14	0	0	0	0	0	0	0	0	0	0	0.078326627	0
KLF15	0.259680749	0.384585465	0.274849686	0.39368788	0.290521193	0.457170288	0.19074515	0.308548701	0.807421676	0.955948154	0.699296666	1.216108455
KLF16	28.14424555	27.2834014	26.21761218	25.16750442	28.99838239	31.92741423	24.6296123	29.80079144	13.85358142	14.55182353	15.1981329	15.05200745
KLF17	0	0	0	0	0	0	0	0	0	0	0	0
KLF18	0	0	0	0	0	0	0	0	0	0	0	0
KLF2	8.90733882	9.692914961	11.52494609	8.905401222	3.716323828	2.291817374	6.251667161	5.402713775	3.056669836	1.03368407	3.812997309	1.061116067
KLF3	3.276311599	4.121006123	3.400452788	3.870895792	3.458961292	4.427174535	3.303870708	3.40126017	4.049398785	3.712030972	3.969042281	4.363550559
KLF4	9.710710756	10.53168173	8.984222189	7.67322293	4.268608377	3.417166166	6.452510742	5.873951115	6.045009374	6.061277224	5.851762106	7.70302874
KLF5	0.086118262	0.178556805	0.091148756	0.203092121	0.329717143	0.280061006	0.118079846	0.337068626	1.515110946	1.070231383	1.159543285	1.344332061
KLF6	61.15037611	66.74470846	56.65958809	33.25871207	18.58430791	17.4955892	32.8782529	19.94531777	10.58871958	9.846718842	11.53763642	4.372286396
KLF7	0.983698784	1.4495213	0.936389506	1.019233398	1.088528378	1.057874832	0.976596524	0.93228525	3.396681884	3.466655394	3.468705577	2.227722794
KLF8	0.031175875	0.053866475	0.016498488	0.015754684	0.021702124	0.021344325	0.005343295	0.020337168	1.193104697	1.675095216	1.220128563	0.80570065
KLF9	2.799998271	2.849189011	2.908878615	2.829950209	4.390942213	3.331755758	3.028134187	2.729702505	10.61424629	10.6724502	9.760448178	8.376179377
KLHDC1	0.357369	0.362921909	0.270174484	0.280107943	0.258925339	0.307085769	0.337501024	0.171275451	0.777813661	0.567811138	0.542065269	0.736228407
KLHDC10	5.750384882	6.013386216	5.590891237	6.411671787	6.292432714	6.008437558	5.715732625	5.340534668	12.14278863	11.26380492	10.68663284	10.99450266
KLHDC2	7.144705015	6.825624894	6.951664285	9.795482883	8.263256482	7.534769284	6.589481141	7.931640885	8.228489828	7.04398753	6.263798282	7.702140894
KLHDC3	59.60408705	64.38260329	64.07187897	67.49915984	76.16538159	71.89927063	65.54693661	73.77635818	116.2262704	116.8625489	128.1129886	96.57918195
KLHDC4	5.171446214	4.793017192	5.174123223	6.147034006	6.479896934	6.028083537	5.718058255	5.959892823	4.425299532	4.04219223	4.58018391	5.142662141
KLHDC7A	0	0	0	0	0	0	0	0	0	0	0	0
KLHDC7B	0.022322987	0	0	0	0	0	0.022955888	0	0.170050944	0.048956129	0.096182043	0
KLHDC8A	0.014646043	0.030366969	0.031003147	0	0	0	0	0	0	0	0	0
KLHDC8B	0.043013238	0.022295812	0.136577413	0.065210034	0.022456754	0.044173028	0.066349122	0.021044336	2.059602966	1.957379325	1.783793438	1.745769029
KLHDC9	0	0.041692169	0	0	0	0	0	0	0	0	0	0
KLHL1	0.081901919	0.053067087	0	0	0.02138006	0.021027572	0.010528	0.030053041	0.044564884	0.011226099	0.011027721	0
KLHL10	0.023294182	0.096595882	0.049309767	0.023543363	0.024323289	0	0	0.045586951	0.101399579	0.102172077	0.025091647	0.072725843
KLHL11	1.161879857	1.11417679	1.014543473	1.614674371	1.675746626	1.446765042	1.142548001	1.470871277	3.066208332	3.22493544	2.925462506	2.818837958
KLHL12	29.25932365	27.27016429	28.14305498	27.46541959	26.02632646	25.84365927	29.05558982	25.04976272	24.41559436	25.86785977	23.1814506	29.51335499
KLHL13	0.892767978	1.140550167	0.964007191	0.893203708	0.790965491	0.833491079	0.574959009	0.679449553	1.030436902	1.572263369	0.932516097	1.032324262
KLHL14	0	0	0	0	0	0	0	0	0	0	0	0
KLHL15	3.782846444	3.337221978	3.424430822	3.377396592	3.617249107	2.987051814	3.411187088	2.526317241	7.851038716	7.265798299	7.559839575	7.261320924
KLHL17	5.812811884	6.315504776	5.804769288	5.775416134	5.812427445	7.52095347	5.741213634	6.844720008	7.094180059	8.102523179	8.083154889	8.253728452

KLHL18	8.836263043	6.228210391	8.406402959	7.272701268	6.781165352	5.577169914	9.005344815	6.775355789	8.446376019	6.103900795	7.982530676	8.383350709
KLHL2	10.19800593	8.667188877	9.977129571	8.534898722	10.5167157	8.513798491	9.203294345	7.055162971	11.52541295	10.75183985	10.66761282	11.53261994
KLHL20	9.844188378	8.656633659	9.303887674	9.059700867	8.231630637	8.274000242	8.696702141	7.753058856	7.388733972	6.274882646	5.574898617	7.718241882
KLHL21	8.729973038	7.085302092	8.059139544	8.210321346	8.33527991	8.175611322	8.008451229	9.517377141	4.891641844	3.527435423	4.795140943	6.357362912
KLHL22	2.369662233	2.746372748	2.572393013	2.444139239	2.461661909	2.620753455	2.068195638	2.627890054	2.810214459	2.585105832	2.585238589	4.103803675
KLHL23	0.052819201	0.08213613	0.055904571	0.026692107	0.027576343	0.013560849	0.013579182	0	0.043110373	0.028959202	0.056894924	0.027484122
KLHL24	3.122952763	2.965288591	2.066615513	2.469692537	2.044782168	2.315955558	2.13121124	1.9050025	5.113362924	5.571765854	3.240935579	4.842345098
KLHL25	0.32142556	0.318074004	0.432983404	0.561128538	0.381392877	0.375104932	0.270440679	0.543255845	0.922177277	0.688891691	0.849832201	0.684211362
KLHL26	3.214753217	3.457307375	3.370739709	3.76536064	2.69797023	2.884898312	2.950591029	4.012911623	4.037936766	3.195658466	4.563149199	4.97142653
KLHL28	2.048428188	2.130819099	1.541257451	1.844997303	1.927942721	1.702963763	1.676606148	1.390806342	3.154245104	3.239395924	2.469170077	2.835111931
KLHL29	0.225110967	0.383396025	0.357390828	0.373781227	0.251845803	0.544926082	0.34723996	0.684417266	1.977314522	2.22159007	1.671389333	3.597717989
KLHL3	0.095661442	0.134590262	0.101249389	0.069060532	0.049943821	0.06315481	0.021080064	0.046802603	1.784632596	1.775750695	1.479403484	1.94129516
KLHL30	0.087613939	0.015138161	0.061821201	0	0	0	0.105114294	0	0.27014664	0.208156545	0.440414573	0.015196445
KLHL31	0	0	0	0	0	0	0	0	0.024549297	0.024736322	0	0.046952682
KLHL32	0.029254926	0.005054741	0.005160636	0	0.015273686	0.010014581	0	0.004771016	0.037142787	0.048118826	0.068276744	0.065964633
KLHL33	0	0.037457862	0.009560648	0.009129624	0	0.018553117	0	0.008838833	0.009830159	0	0.009730016	0.00940052
KLHL34	0	0	0	0	0	0	0	0	0	0	0	0
KLHL35	0.416482669	0.402981271	0.352648801	0.168375135	0.579843123	0.427712528	0.228421746	0.461867792	0.543884741	0.487136218	0.747699996	0.606799213
KLHL36	7.957177225	8.369028369	7.13349422	8.234761373	8.138279984	7.997121469	7.105729343	7.999092498	14.07707342	15.4968516	12.81281847	15.28079153
KLHL38	0	0	0.017347786	0	0	0	0	0	0.017836813	0	0.017655103	0
KLHL4	0.450327666	0.390631766	0.340452151	0.18577342	0.287891368	0.415279279	0.444193491	0.089928144	0	0.010077605	0	0
KLHL40	0	0	0	0	0	0	0	0	0	0	0	0
KLHL41	0.087353183	0.02263966	0.092455814	0.088287611	0.045606168	0.112135671	0.044914909	0.085475533	0.166358685	0.143679483	0.117617093	0.090907303
KLHL42	5.215364398	4.307747103	5.536393466	6.530286997	4.508517923	4.545438927	5.124510802	5.18654816	26.76972954	21.99300235	22.8962899	32.87136972
KLHL5	2.702882577	2.621578477	2.602792319	1.89598056	1.217994179	1.640426134	2.13946536	1.213789903	3.050353276	3.832444601	3.253694276	2.984977298
KLHL6	3.104658462	3.471574215	2.575717875	1.507273506	2.286260514	2.694103989	2.795339168	1.545494117	3.740115436	3.396951257	3.263905459	2.42676004
KLHL7	4.72837205	5.281688118	4.119988435	5.912940671	4.674263461	4.726532839	5.686571508	5.948660626	4.746956583	5.172298347	4.636936293	5.623719202
KLHL8	9.557974944	7.860273339	9.25057131	9.45586706	10.08579425	9.070076974	9.914020684	7.697849128	9.821384874	9.150464886	8.899670639	9.344308141
KLHL9	4.772073169	4.317896265	4.398470614	4.955260936	4.651354208	4.210229315	4.609663412	3.764839721	0.020325671	0	0	0
KLK1	0.434649118	0.660878125	0.64405407	0.820024082	0.907702724	1.220074745	0.536366732	0.567075157	2.774973948	2.287730185	2.74670432	1.749023227
KLK10	0	0	0	0	0	0	0	0	0.016616986	0	0	0.063562882
KLK11	0	0	0	0	0	0	0	0	0	0	0.032254704	0
KLK12	0	0	0	0	0	0	0	0	0	0	0	0.026290563
KLK13	0	0	0	0	0	0	0	0	0	0	0	0
KLK14	0.217412367	0.187825326	0.191760206	0.292984073	0.302689824	0.07442486	0.111788218	0.425478209	0.03943317	0	0	0.150838785
KLK15	0	0	0	0	0	0	0	0	0	0.027832252	0	0.079243725
KLK2	0	0	0	0	0	0	0	0	0	0	0	0
KLK3	0	0	0	0	0	0	0	0	0	0	0	0
KLK4	0	0	0	0	0	0	0	0	0	0	0	0
KLK5	0	0	0	0	0	0	0	0	0	0	0	0
KLK6	0	0	0	0	0	0	0	0	0	0	0	0
KLK7	0	0	0	0	0	0	0	0	0	0	0	0
KLK8	0	0	0	0	0	0	0	0	0	0	0	0
KLK9	0	0	0	0	0	0	0	0	0	0	0	0
KLKB1	0.227113009	0.319535216	0.223209574	0.147563014	0.440415063	0.566432195	0.18350523	0.238104891	0.123577871	0.071153903	0.157267209	0.118176754
KLLN	0.817774481	0.756483296	0.62585474	0.203451648	0.446656805	0.41345211	0.698643691	0.369321452	1.12269747	0.924314492	0.975740627	1.034349546
KLRB1	0	0	0	0	0.075928106	0.074676295	0.149554508	0.213457818	0	0	0.078326627	0
KLRC1	0	0	0	0	0	0	0	0	0	0	0	0
KLRC2	0.088140149	0.091374483	0.139933124	0.044541498	0.092034068	0	0.135958643	0.043122791	0.095918521	0	0.284824097	0
KLRC3	0.140985749	0.194879028	0.397923363	0.047497964	0.04907144	0	0.338293603	0	0.102285165	0.206128819	0.202486301	0.09781467
KLRC4	0.057984441	0	0.122743063	0	0	0	0.059628414	0	0	0.1271646	0	0.120687282
KLRC4-KLRK	0.027637165	0.028651321	0	0	0.05771628	0	0	0	0	0.030305277	0	0.028761633
KLRD1	0	0.007018636	0.007165675	0	0	0.020858251	0	0.006624676	0	0.007423802	0	0.007045659
KLRF1	0	0	0	0.076063173	0	0.077287355	0	0	0.450448131	0.701450604	0.486391919	0.469920829
KLRF2	0	0	0	0	0	0	0	0	0	0	0	0.076605881
KLRG1	0.536856709	0.513744789	0.611925509	0.306081788	0.574948055	0.381691579	0.424675118	0.255923673	0.149803676	0.18113392	0.148277574	0.114605063
KLRG2	0	0	0	0	0	0	0	0	0.037791712	0.133278677	0.1309235	0.14455992
KLRK1	0	0	0	0	0	0	0	0	0.03685227	0.037133024	0	0
KMO	0.427204284	0.348650761	0.423297699	0.137800258	0.294221412	0.177356201	0.261720389	0.168987439	1.13753371	0.598017308	0.910547034	0.567556407
KMT2A	6.511533591	5.948836641	5.875674641	6.056907586	7.270203691	6.839313086	6.016925026	4.621830604	14.140797	12.12166986	10.9669471	12.85550435
KMT2B	13.79779565	13.3874253	13.26493514	14.34879719	15.39478529	15.53195361	13.2228508	15.30377821	9.849922505	9.473827994	8.664821613	11.51546453
KMT2C	7.794500125	6.816158019	6.913661307	6.191085996	6.852593539	6.378621439	6.057195439	4.277078363	13.65827105	10.8764596	10.83242474	11.35628657
KMT2D	12.94498516	11.90978503	11.39933553	12.71406249	13.62784966	13.31000014	9.734767238	10.55138407	10.60079628	9.672522082	9.294981043	10.67159515
KMT2E	8.262560985	9.888726272	8.048081555	8.969095018	9.628513689	10.09182801	8.31539408	7.279339271	16.51150855	13.8515187	14.58227779	13.8681426
KMT5A	14.63148418	14.20531917	15.95888029	22.62437459	17.12466173	16.7322504	17.25090393	20.95978123	15.08672227	16.59253156	17.29999057	13.85098925
KMT5B	4.540891212	3.842167727	3.73889567	3.766061563	5.187761005	4.738766055	4.436152699	3.829066269	7.179890541	5.272170456	5.93427718	6.164187549
KMT5C	2.314942867	3.319848543	2.817692574	3.158603243	3.075233481	3.724533775	2.817015	3.750136864	4.534626025	4.978141526	4.640815183	5.420543506
KNCN	0	0	0	0	0	0	0	0	0	0	0	0
KNDC1	0.0043127	0	0	0.004358834	0.027019379	0	0.013304921	0.004219999	0.093865925	0.047290514	0.055745808	0.098739743
KNG1	0	0	0	0	0	0	0	0	0	0	0	0
KNL1	7.110208125	6.410428764	7.513653644	16.51252014	13.76960685	11.35662696	8.386041515	12.33752469	22.84022614	21.97960673	22.14172168	19.45729404
KNOP1	7.592447508	6.074975374	7.694378282	8.308845203	9.222580583	9.105416468	8.715987617	9.107892905	6.118302742	7.263796085	6.934181088	6.310481971
KNSTRN	14.79564413	14.26844234	15.71594211	25.49388376	25.17765546	22.83265381	20.54995167	26.52070763	29.58156911	35.95987202	33.89890096	21.26470728
KNTC1	15.58473717	18.3894731	17.94164081	26.75142801	34.95984452	32.59044332	18.72712796	22.53853408	13.50266193	16.32035886	14.21294967	11.19977977
KPNA1	13.87600264	10.81444155	13.78657731	13.4141364	12.34012135	12.06706681	13.19857491	12.20911255	13.04200275	13.891331	13.07506655	15.26135111
KPNA2	78.51195882	66.45962286	88.36921229	151.7049412	110.1360699	102.7983088	107.181282	163.0454617	90.77393536	93.03197076	98.44085806	84.76234503
KPNA3	25.5308453	21.70784826	25.30416614	29.35463205	28.72899658	27.64808981	28.18623936	26.70652732	18.49739385	16.84567895	17.14747234	18.2681174
KPNA4	11.01834789	8.428800038	11.58587367	11.23310185	10.45408535	9.217254975	12.51372074	9.984181821	12.02331879	10.32133082	11.44906606	11.39182587
KPNA5	2.303110152	2.411925105	2.053078917	2.345515875	3.102451199	2.7503844	2.278010614	1.875136959	2.366054604	2.493323595	2.354575851	2.152865529
KPNA6	11.32465967	10.33428612	10.87945016	11.18319449	9.555262893	9.944409456	11.75000679	10.39912387	12.52406016	13.01556862	12.10294436	13.11763158
KPNA7	0	0	0	0	0	0	0	0	0.014832385	0.014945384	0.014681283	0
KPNB1	108.7429235	99.34158658	114.8008455	119.5667641	125.710549	122.6576179	122.6494738	110.2601744	109.9627544	105.9110631	126.7529913	90.68225006
KPRP	0	0	0	0	0	0	0	0	0	0	0	0
KPTN	1.950529476	1.8922449	2.386448435	2.351204495	2.18618405	2.076631798	2.189852005	2.276315491	3.894795947	3.100329613	3.296126148	2.961032562
KRAS	10.5073526	9.517270888	9.544678497	11.57771833	11.7727414	10.90191231	11.07445987	9.477703756	26.62185483	24.52824017	25.54359931	23.71099156
KRBA1	0.050160392	0.18200366	0.119453515	0.050696964	0.039282311	0.090147567	0.064478173	0.036811649	3.111461806	2.736394895	2.782594023	3.614937072
KRBA2	0.554365017	0.474758483	0.408172229	0.499393485	0.47818552	0.34653815	0.42136522	0.318393967	0.262299024	0.237867579	0.350496304	0.41387764
KRBOX1	0	0	0	0	0	0	0	0	0	0	0	0.080923264
KRBOX4	4.025976798	4.442983144	4.971340926	4.134672914	4.701067625	4.090074166	4.496260624	4.066517393	6.053689487	5.625115334	6.13190992	6.262145566
KRCC1	18.72072164	20.54931367	20.23810461	19.35100185	20.41018127	17.73475182	20.92441464	17.43669068	33.06498794	23.71152532	28.57646785	32.97423532
KREMEN1	1.175079555	1.018108772	1.045446138	0.487682174	0.491982729	0.507190621	1.453577044	0.544359846	3.385370279	4.083433861	3.85229173	3.822268968
KREMEN2	0.052318484	0.027119165	0.11074921	0.026439071	0.027314924	0.026864589	0	0	0.056935593	0.057369348	0.197244495	0.027223577
KRI1	51.44980603	44.75702787	52.82796193	52.74096583	58.37080376	53.69994728	54.61820059	60.54212124	20.52457183	19.17152097	22.91509214	26.32506374
KRIT1	6.979268332	7.47448781	6.808294066	8.469892109	7.376803059	7.430641119	7.519749755	6.829249475	12.47581854	14.00405456	11.50216377	12.30393332
KRR1	24.30005787	24.37751163	24.24352829	29.07994568	30.12696978	30.04180426	26.50549596	24.51490228	20.79280382	20.52937421	20.20112996	21.71896523
KRT1	0	0	0	0	0	0	0	0	0	0	0	0
KRT10	3.719554898	3.83033831	5.222858746	6.942254241	4.816011311	4.354625998	3.978012069	6.478493946	3.80493744	4.051452432	4.433936778	4.799905615
KRT12	0	0	0	0	0	0	0	0	0	0	0	0
KRT13	0	0.032527185	0	0	0.163809909	0	0	0	0.034144756	0	0	0
KRT14	0	0	0	0	0	0	0	0	0	0	0	0
KRT15	0	0	0	0	0.018886319	0	0	0	0	0	0	0
KRT16	0	0	0	0	0	0	0	0	0	0	0	0
KRT17	1.504206276	1.13411175	1.302604909	0.138208815	0.642542805	0.140433186	1.01951707	0.301065029	0.409238137	0.074973792	0.294595698	0.142309781
KRT18	1.928029868	1.92609671	1.780933878	0.992042163	7.979623519	6.912057358	9.228536141	5.762665771	0	0.230635991	0	0
KRT19	0	0	0	0	0	0	0	0	0	0.040111743	0	0
KRT2	0	0	0	0	0	0	0	0	0	0	0	0
KRT20	0	0	0	0	0	0	0	0	0	0	0	0
KRT222	0	0	0	0	0	0	0	0	0	0	0	0
KRT23	0.021927286	0	0	0	0	0.022518524	0	0	0	0	0	0
KRT24	0	0	0	0	0	0	0	0	0	0	0	0
KRT25	0	0	0	0	0	0	0	0	0	0	0	0
KRT26	0	0	0	0	0	0	0	0	0	0	0	0
KRT27	0	0	0	0	0	0	0	0	0	0	0	0
KRT28	0	0	0	0	0	0	0	0	0	0	0	0
KRT3	0	0	0	0	0	0	0	0	0	0	0	0
KRT31	0	0	0	0	0	0	0	0	0	0	0	0
KRT32	0	0	0	0	0	0	0	0	0	0	0	0
KRT33A	0	0	0	0	0	0	0	0	0	0	0.02851043	0
KRT33B	0	0	0	0	0	0	0	0	0	0	0	0
KRT34	0	0	0	0	0	0	0	0	0	0	0	0
KRT35	0	0	0	0	0	0	0	0	0	0.016691478	0	0.015841273
KRT36	0	0	0	0	0	0	0	0	0	0.071781477	0	0
KRT37	0	0	0	0	0	0	0	0	0	0	0	0
KRT38	0	0	0	0	0	0	0	0	0	0	0	0
KRT39	0	0	0	0	0	0	0	0	0	0	0	0
KRT4	0	0	0	0	0	0	0	0	0	0	0	0
KRT40	0	0	0	0	0	0	0	0	0	0	0	0
KRT5	0	0.024222372	0	0	0	0	0	0.022862757	0	0	0	0
KRT6A	0	0	0	0	0.068800162	0	0	0	0	0	0	0
KRT6B	0	0	0	0	0	0	0	0	0	0	0	0
KRT6C	0	0	0	0	0	0	0	0	0	0	0	0
KRT7	0.196313611	0.183165668	0.207781034	0.079365441	0.266482445	0.080642771	0.222067438	0.057628159	0.02136383	0	0.042292378	0
KRT71	0	0	0	0	0	0	0	0	0	0	0	0
KRT72	0	0	0	0	0	0	0	0	0	0	0	0
KRT73	0	0	0	0	0	0	0	0	0	0	0	0
KRT74	0	0	0	0	0	0	0	0	0	0	0	0
KRT75	0	0	0	0	0	0	0	0	0	0	0	0
KRT76	0	0	0	0	0	0	0	0	0	0	0	0

KRT77	0	0	0	0	0	0	0	0	0	0	0	0
KRT78	0	0	0	0	0	0	0	0	0	0	0	0.018653864
KRT79	0.026045286	0.108004108	0.11026676	0	0	0.02674756	0.080351163	0	0	0	0	0
KRT8	0.804729225	0.695215874	0.539433123	0.216890004	1.680562272	1.98342623	1.765429085	1.181147445	0.554639524	0.647106797	0.664565894	0.167494214
KRT80	0	0	0	0	0.012807568	0	0.037840325	0	0	0	0.01321215	0
KRT81	0.22538036	0.204444425	0.238545681	0	0.235337376	0	0.376626797	0.027566978	0	0	0	0
KRT82	0	0	0	0	0	0	0	0	0	0	0	0
KRT83	0	0	0	0	0	0	0	0	0	0	0	0
KRT84	0	0	0	0	0	0	0	0	0	0	0	0
KRT85	0	0	0	0	0	0	0	0	0	0	0	0
KRT86	0.12326564	0.051115567	0.060884158	0	0.017161515	0	0.059154883	0	0	0.009011053	0	0
KRT9	0	0	0	0	0	0	0	0	0	0.025766102	0	0
KRTAP1-1	0	0	0	0	0	0	0	0	0	0	0	0
KRTAP1-3	0	0	0	0	0	0	0	0	0	0	0	0
KRTAP1-4	0	0	0	0	0	0	0	0	0	0	0	0
KRTAP1-5	0	0	0	0	0	0	0	0	0	0	0	0
KRTAP10-1	0	0	0	0	0	0	0	0	0	0	0	0
KRTAP10-10	0	0	0	0	0	0	0	0	0	0	0	0
KRTAP10-11	0	0	0	0	0	0	0	0	0	0	0	0
KRTAP10-12	0	0	0	0	0	0	0	0	0	0	0	0
KRTAP10-2	0	0	0	0	0	0	0	0	0	0	0	0
KRTAP10-3	0	0	0	0	0	0	0	0	0	0	0	0
KRTAP10-4	0	0	0	0	0	0	0	0	0	0	0	0
KRTAP10-5	0	0	0	0	0	0	0	0	0	0	0	0
KRTAP10-6	0	0	0	0	0	0	0	0	0	0	0	0
KRTAP10-7	0	0	0	0	0	0	0	0	0	0	0	0
KRTAP10-8	0	0	0	0	0	0	0	0	0	0	0	0
KRTAP10-9	0	0	0	0	0	0	0	0	0	0	0	0
KRTAP11-1	0	0	0	0	0	0	0	0	0	0	0	0
KRTAP12-1	0	0	0	0	0	0	0	0	0	0	0	0
KRTAP12-2	0	0	0	0	0	0	0	0	0	0	0	0
KRTAP12-3	0	0	0	0	0	0	0	0	0	0	0	0
KRTAP12-4	0	0	0	0	0	0	0	0	0	0	0	0
KRTAP13-1	0	0	0	0	0	0	0	0	0	0	0	0
KRTAP13-2	0	0	0	0	0	0	0	0	0	0	0	0
KRTAP13-3	0	0	0	0	0	0	0	0	0	0	0	0
KRTAP13-4	0	0	0	0	0	0	0	0	0	0	0	0
KRTAP15-1	0	0	0	0	0	0	0	0	0	0	0	0
KRTAP16-1	0	0	0	0	0	0	0	0	0	0	0	0
KRTAP17-1	0	0	0	0	0	0	0	0	0	0	0	0
KRTAP19-1	0	0	0	0	0	0	0	0	0	0	0	0
KRTAP19-2	0	0	0	0	0	0	0	0	0	0	0	0
KRTAP19-3	0	0	0	0	0	0	0	0	0	0	0	0
KRTAP19-4	0	0	0	0	0	0	0	0	0	0	0	0
KRTAP19-5	0	0	0	0	0	0	0	0	0	0	0	0
KRTAP19-6	0	0	0	0	0	0	0	0	0	0	0	0
KRTAP19-7	0	0	0	0	0	0	0	0	0	0	0	0
KRTAP19-8	0	0	0	0	0	0	0	0	0	0	0	0
KRTAP2-1	0	0	0	0	0	0	0	0	0	0	0	0
KRTAP2-2	0	0	0	0	0	0	0	0	0	0	0	0
KRTAP2-3	0	0	0	0	0	0	0	0	0	0	0	0
KRTAP2-4	0	0	0	0	0	0	0	0	0	0	0	0
KRTAP20-1	0	0	0	0	0	0	0	0	0	0	0	0
KRTAP20-2	0	0	0	0	0	0	0	0	0	0	0	0
KRTAP20-3	0	0	0	0	0	0	0	0	0	0	0	0
KRTAP20-4	0	0	0	0	0	0	0	0	0	0	0	0
KRTAP21-1	0	0	0	0	0	0	0	0	0	0	0	0
KRTAP21-2	0	0	0	0	0	0	0	0	0	0	0	0
KRTAP21-3	0	0	0	0	0	0	0	0	0	0	0	0
KRTAP22-1	0	0	0	0	0	0	0	0	0	0	0	0
KRTAP22-2	0	0	0	0	0	0	0	0	0	0	0	0
KRTAP23-1	0	0	0	0	0	0	0	0	0	0	0	0
KRTAP24-1	0	0	0	0	0	0	0	0	0	0	0	0
KRTAP25-1	0	0	0	0	0	0	0	0	0	0	0	0
KRTAP26-1	0	0	0	0	0	0	0	0	0	0	0	0
KRTAP27-1	0	0	0	0	0	0	0	0	0	0	0	0
KRTAP29-1	0	0	0	0	0	0	0	0	0	0	0	0
KRTAP3-1	0	0	0	0	0	0	0	0	0	0	0	0
KRTAP3-2	0	0	0	0	0	0	0	0	0	0	0	0
KRTAP3-3	0	0	0	0	0	0	0	0	0	0	0	0
KRTAP4-1	0	0	0	0	0	0	0	0	0	0	0	0
KRTAP4-11	0	0	0	0	0	0	0	0	0	0	0	0
KRTAP4-12	0	0	0	0	0	0	0	0	0	0	0	0
KRTAP4-2	0	0	0	0	0	0	0	0	0	0	0	0
KRTAP4-3	0	0	0	0	0	0	0	0	0	0	0	0
KRTAP4-4	0	0	0	0	0	0	0	0	0	0	0	0
KRTAP4-5	0	0	0	0	0	0	0	0	0	0	0	0
KRTAP4-6	0	0	0	0	0	0	0	0	0	0	0	0
KRTAP4-7	0	0	0	0	0	0	0	0	0	0	0	0
KRTAP4-8	0	0	0	0	0	0	0	0	0	0	0	0
KRTAP4-9	0	0	0	0	0	0	0	0	0	0	0	0
KRTAP5-1	0	0	0	0	0	0	0	0	0	0	0	0
KRTAP5-10	0.254298492	0.316356078	0.215322425	0.308422506	0	0.313386343	0.366111697	0.447898256	0.221392276	0.05576973	0.054784219	0.158787048
KRTAP5-11	0	0	0	0	0	0	0	0	0	0	0	0
KRTAP5-2	0	0	0	0	0	0	0	0	0	0	0	0
KRTAP5-3	0	0	0	0	0	0	0	0	0	0	0	0
KRTAP5-4	0	0	0	0	0	0	0	0	0	0	0	0
KRTAP5-5	0	0	0	0	0	0	0	0	0	0	0	0
KRTAP5-6	0	0	0	0	0	0	0	0	0	0.105177138	0	0
KRTAP5-7	0.47937248	0.559083625	0.317109027	0.121125097	0.250275272	0.307686295	0.431343179	0.05863355	0.130419281	0.06570643	0.129090654	0.436517029
KRTAP5-8	0.181942725	0.33008356	0.385141378	0.229861237	0.284971084	0.093424275	0.14032587	0.08901594	0.296998768	0.399015211	0.244977615	0.426027126
KRTAP5-9	0.588825161	0.563475977	0.383520413	0.32045133	0.520248135	0.279093225	0.326048969	0.177282587	0.887246319	0.347668872	0.390314503	0.989879525
KRTAP6-1	0	0	0	0	0	0	0	0	0	0	0	0
KRTAP6-2	0	0	0	0	0	0	0	0	0	0	0	0
KRTAP6-3	0	0	0	0	0	0	0	0	0	0	0	0
KRTAP7-1	0	0	0	0	0	0	0	0	0	0	0	0
KRTAP8-1	0	0	0	0	0	0	0	0	0	0	0	0
KRTAP9-1	0	0	0	0	0	0	0	0	0	0	0	0
KRTAP9-2	0	0	0	0	0	0	0	0	0	0	0	0
KRTAP9-3	0	0	0	0	0	0	0	0	0	0	0	0
KRTAP9-4	0	0	0	0	0	0	0	0	0	0	0	0
KRTAP9-6	0	0	0	0	0	0	0	0	0	0	0	0
KRTAP9-7	0	0	0	0	0	0	0	0	0	0	0	0
KRTAP9-8	0	0	0	0	0	0	0	0	0	0	0	0
KRTAP9-9	0	0	0	0	0	0	0	0	0	0	0	0
KRTCAP2	132.4089011	134.4121982	144.0999074	137.3051514	112.9899058	115.673172	129.2839937	154.3009947	179.4216432	163.745229	180.6667363	225.1217158
KRTCAP3	0.25191742	0.208929295	0.479939168	0.916603965	0.631312344	0.362194017	0.673555414	0.640906431	1.151426403	0.994455747	1.031153904	0.943801666
KRTDAP	0	0	0	0	0	0	0	0	0	0	0	0
KSR1	5.548386505	5.121778143	5.376298934	4.332068959	4.163578951	4.105516118	5.149428739	4.057980594	2.298600804	2.462987766	2.014370362	3.168535109
KSR2	0	0	0.002873066	0	0	0	0	0	0.002954056	0	0.002923962	0
KTI12	10.37483868	10.59108914	13.60016299	11.57608836	12.52276526	10.26358753	11.77829929	12.35605276	16.15876433	14.29881949	17.08776641	17.82983807
KTN1	17.72694836	17.51493024	19.05263913	21.60022378	22.24173896	19.54481298	20.98584905	17.66044807	19.02384561	18.2720214	19.2068476	18.33505192
KXD1	35.24066326	34.49258593	36.38649917	32.9593877	36.06220008	36.2499162	36.13634761	38.05524147	18.97199942	21.44897262	21.1163899	20.35643732
KY	0	0	0	0	0	0	0	0	0.061401625	0.004759185	0.023375425	0.027100612
KYAT1	1.383567583	1.796153096	1.543543065	1.940077824	2.238621581	2.342521176	1.474066321	1.963660287	0.895261748	1.681153126	1.033831638	0.713444391
KYAT3	12.07161218	13.60175856	11.76547279	14.01436783	15.42764415	15.02969596	13.80383573	12.19979067	18.08230285	18.47560098	17.2956318	18.86840333
KYNU	2.311949248	2.149531141	2.145704586	1.045868627	1.899939645	1.481460678	1.803891661	1.11042421	1.63266516	1.328737341	1.504153448	0.592496213
L1CAM	0.390073123	0.159626445	0.130376455	0.051874445	0.085748643	0.126502385	0.063336705	0.030133305	1.061242736	0.427731062	1.006202791	0.459357621
L1TD1	0	0	0	0	0	0	0	0	0.029359868	0.266251877	0.029060769	0
L2HGDH	2.672411039	3.051425795	3.115352195	3.644445406	3.76517579	3.710831017	3.243625537	3.182157951	1.540144422	1.948101895	1.954220842	1.410156938
L3HYPDH	6.8371694	5.510280716	6.0406314	3.915452464	6.260367535	6.015065985	6.058768114	3.926123646	5.721569578	5.00658501	4.88085484	5.699480812
L3MBTL1	1.177319985	1.267465318	1.022434223	1.067871473	1.008683061	0.992053113	0.760567525	1.270168818	3.285175712	3.392958413	2.698918039	4.33539862
L3MBTL2	14.58613035	12.93564774	14.9188779	16.22977801	14.30284943	14.54365938	15.46330221	18.12620524	15.5125989	13.26071696	16.19728239	18.353508
L3MBTL3	3.485147804	3.254638807	3.659060441	2.238111644	3.063492754	3.47356937	3.305313032	2.139396636	4.280782466	5.276480776	5.223497864	3.695013294
L3MBTL4	0	0	0	0	0	0	0	0	0	0	0	0
LACC1	4.543162853	3.192140367	3.498942155	2.863884602	2.445905926	2.172782643	4.011483998	2.541610548	3.186424379	2.164633007	2.635085352	3.027498831
LACRT	0	0	0	0	0	0	0	0	0	0	0	0
LACTB	12.86781176	11.66515476	12.13452728	11.20073791	7.487627102	8.397493967	12.3145159	9.582084138	3.917249748	3.7139967	3.159882185	4.793163582
LACTB2	19.99234811	20.15125066	20.79344945	19.96107283	26.48341056	22.95105974	23.37403099	19.39309401	23.26493536	20.17470881	26.01372019	21.88752839
LACTBL1	0	0	0	0	0	0	0	0	0	0	0	0
LAD1	0	0	0	0	0	0	0	0	0.020626367	0	0.020416239	0
LAG3	0	0	0	0	0	0	0.027488906	0	0.261810389	0.175869968	0.115174771	0.139093142
LAGE3	29.93830151	33.1530502	33.36748831	46.01821958	27.70855823	28.00872557	29.38769717	45.93954126	8.947257402	11.19155678	10.81056011	12.98183113
LAIR1	118.2824515	87.17652972	107.732055	93.39303456	69.63731775	57.55524402	88.06317404	83.3765149	108.2836045	54.50022363	79.37656211	144.9473788
LAIR2	0.381628091	0.395632069	0.323136348	0.231426249	0.47818552	0.156767258	0.392447999	0.224055014	0.830613575	0.167388296	0.246645547	0.476586373
LALBA	0	0	0	0	0	0	0	0	0	0	0	0
LAMA1	0	0	0	0.005572822	0	0.005662512	0	0	0	0.00604615	0	0
LAMA2	0.095051902	0.19356045	0.114976279	0.048034342	0.067349011	0.097614841	0.101237768	0.046504384	0.225351945	0.227068755	0.190146274	0.116583412

LAMA3	0.019027426	0.019725644	0.025173613	0	0.009934017	0.014655355	0.004891723	0.009309216	1.998186636	1.940384305	1.619156502	2.257381353
LAMA4	0.021313531	0.014730426	0.022558535	0.007180508	0	0	0	0	0	0	0	0
LAMA5	0.181333524	0.067482736	0.123029425	0.084587664	0.03883992	0.023874734	0.047814022	0.036397082	3.071361102	2.105660296	2.138568	3.508096575
LAMB1	0.048295791	0.100136045	0.093714375	0	0.042024532	0.008266336	0	0	4.081996678	4.880990129	3.511516826	4.448080072
LAMB2	0.036982516	0.038339603	0.136999818	0	0.009654089	0	0	0	0.392400692	0.415666555	0.368485058	0.163570667
LAMB3	1.659325665	1.16728889	1.542993855	0.814579618	0.779684651	0.499048194	1.621052275	0.858219537	2.205608361	1.338645497	1.429892249	2.51625008
LAMB4	0	0	0	0	0	0	0	0	0	0	0	0
LAMC1	38.5513548	40.69433646	35.31664421	36.19243696	24.91335044	29.13274777	29.57895842	26.58342167	29.20118941	35.51930204	27.97796521	32.68081256
LAMC2	0.091020324	0.102938561	0.035031697	0.016726178	0.03456054	0.016995374	0.017018351	0.032386854	0	0.009073408	0	0.008611241
LAMC3	0.675398047	0.638140572	0.334803449	0.06048557	0.205321952	0.237056305	0.290127191	0.117118048	0.595444622	0.18749404	0.359152597	0.124560627
LAMP1	92.8157099	82.83691381	89.55763656	108.7065797	57.02114734	59.9680709	78.06172569	98.61879072	108.146277	100.9461317	106.1385476	146.748189
LAMP2	21.08706587	19.57910961	19.03683721	25.3967383	16.500533	16.45805732	17.71809913	19.72521825	31.04435933	30.36952885	27.3385624	42.02303391
LAMP3	0	0.00915845	0	0	0	0	0.009084743	0	0.057683392	0.029061422	0.028547876	0.027581136
LAMP5	39.57980432	48.08033995	40.69251123	37.89916499	41.90621661	58.64319966	36.09522214	45.02057437	45.1087847	36.1770209	39.1142153	31.37254666
LAMTOR1	55.40696089	58.57569352	62.16982997	57.014477	42.4141228	47.43306537	58.66748079	62.92449195	42.31690841	36.83394361	45.91707482	47.16446696
LAMTOR2	82.49219022	76.91819423	87.8962895	77.70450668	54.94920395	69.94789322	93.76913911	85.74463658	96.65122017	101.9796602	103.4118501	100.403312
LAMTOR3	8.901207731	7.377059084	8.702597891	8.310257071	6.511367318	6.610596913	8.972571614	7.762616302	9.016329767	9.912183448	8.613000824	9.982981265
LAMTOR4	36.90928912	43.06862337	43.37265383	45.87320419	36.12008483	40.92292362	41.50137591	52.37638984	44.28775744	43.57150753	46.33283244	47.8814114
LAMTOR5	62.0392584	61.16564873	66.73525903	64.68635936	54.60034785	54.09023709	72.9773214	69.00630842	75.57459764	89.13131372	81.41914608	75.17028656
LANCL1	11.22580711	11.28755926	11.81004525	10.59480895	14.68451799	15.20558757	12.65564731	10.03699889	23.39144439	31.24923027	22.12223216	23.3768159
LANCL2	2.929672852	2.39642333	2.826051174	2.79859356	2.878395611	2.640518121	2.605952082	3.14490268	4.883217853	2.982072676	4.114442093	3.936518044
LANCL3	0	0.010468028	0.01068733	0.005102756	0.07380514	0.015554642	0.067494575	0.02470113	0.714259093	0.963291531	0.652599403	0.74609154
LAP3	55.55196569	43.75068973	51.14902168	46.14970021	42.35414392	42.99789967	46.03358966	44.27860544	73.92282832	76.9023679	71.51370199	61.46545801
LAPTM4A	15.21301466	17.63599995	15.61764654	18.241371	14.07057507	14.96572192	15.80109043	18.70446802	27.30506831	26.94477378	23.64443435	32.07642307
LAPTM4B	88.72085772	81.38300149	102.5557233	78.321447	97.58627626	90.7920939	109.4093467	85.94331866	56.51735266	71.18490203	71.63631471	42.76234053
LAPTM5	260.535151	219.6313348	228.6740461	166.2092185	139.8410296	138.9638002	209.7262045	158.0761043	410.2470407	310.4303224	354.1243125	399.778235
LARGE1	0.369026706	0.531344822	0.347184849	0.815017767	0.449551475	0.505302644	0.330296278	0.715502074	0.14873827	0.202326404	0.125139568	0.583173699
LARGE2	0.01572	0.016296851	0	0.031776318	0	0.016143868	0.016165693	0.0307642	0.017107291	0.086188102	0.067732052	0
LARP1	61.70871178	51.47077085	58.72579879	55.23171071	62.21646267	59.73612213	57.66005724	52.54631647	54.44539542	55.83835882	62.95688465	49.58545877
LARP1B	2.251585482	2.082528252	2.196692556	2.747163064	2.982314152	2.884259603	2.516124937	2.510609374	4.066545629	3.664284394	3.579022395	3.660932965
LARP4	11.26124814	8.23548844	10.76719249	10.61704838	11.3272975	9.807745242	11.42492272	8.262967662	5.649358133	4.447982795	5.109105395	5.317654465
LARP4B	10.2748272	9.722339607	10.40479394	9.727528092	10.07929511	9.888233373	10.6699727	8.856504719	13.04078276	12.98955415	13.55180567	13.75699136
LARP6	0	0.021563248	0.066044973	0	0	0	0.021389705	0	2.87471923	2.531691362	2.64378857	2.229565744
LARP7	14.50495644	13.99734032	15.2823995	19.60907477	18.57733739	17.18672099	18.41791092	18.68746316	18.78690219	16.59995521	17.81359202	17.07300252
LARS	66.79845384	57.06926048	68.07628069	58.58740691	74.95009652	72.283609	71.70444996	58.964839	35.17076969	44.05178774	47.00747343	32.40058612
LARS2	9.324892711	7.861972707	9.869595045	9.064194766	9.430181992	9.274708543	9.97758875	9.935289399	12.22532033	12.01940959	12.04428384	11.71283011
LAS1L	22.23699294	21.0634477	24.70225328	25.32802146	27.61504875	25.85009486	24.22982616	26.45973204	24.25330481	26.51531134	27.90057157	24.2498683
LASP1	69.82540241	78.1846573	69.23318146	74.06653772	64.06306926	65.29314343	69.77160009	72.57877489	64.62927617	67.51972942	65.32505467	61.47749639
LAT	1.526913445	2.696867681	1.3766831	1.000252707	1.771522815	2.119817906	1.77374947	1.604766076	7.877516452	8.34060783	6.700774989	10.65244424
LAT2	92.27867674	97.57327953	92.62320749	102.449254	116.1686705	119.2220157	85.81804983	102.7886772	115.4727736	152.0915386	128.7105726	133.2921158
LATS1	7.398518441	6.417261908	7.003110603	7.980562499	8.034637981	7.45201032	7.370712586	5.767246112	11.88699098	10.12635619	10.5216699	12.1380264
LATS2	4.511981811	4.613724351	4.282164008	3.342403301	4.215387475	3.477488811	4.232896137	3.218730826	2.498153824	2.430386129	2.40638652	2.489653561
LAX1	0.202215561	0.15722695	0.160520804	0.122627212	0.15836189	0.207668014	0.093576947	0.138508267	0.759210774	0.365867034	0.413856584	0.578718421
LAYN	0	0	0	0	0	0	0	0	0.07672225	0.212593553	0.17086647	0.018342253
LBH	0.323180546	0.485807666	0.205235248	0.114323177	0.151856213	0.348489378	0.249257513	0.284610423	2.813609949	2.055407635	2.732728971	2.236592656
LBHD1	9.620497245	9.93126409	8.715501381	4.449695613	6.810521046	6.572645451	9.180607398	5.02596134	7.097970553	5.051132046	6.937840286	5.302926026
LBHD2	0	0	0	0	0	0	0	0	0	0	0	0
LBP	0.056821078	0.029453074	0.120280425	0.02871445	0.059331361	0.087529765	0.058432067	0	0	0.031153313	0.0306028	0.059132945
LBR	54.06991062	62.64212126	58.38067158	85.37526577	87.89458954	81.49915002	66.83543919	76.21763234	54.41140006	62.45613674	60.15422907	57.46445172
LBX1	0	0	0	0	0	0	0	0	0	0	0	0
LBX2	0.801691908	1.263287619	1.018226126	0.745446292	0.703172093	0.757443711	0.791444594	0.690324448	1.465701309	1.54719456	1.27805902	1.268151464
LCA5	0.718373031	0.886588075	0.736198275	0.795205898	0.988239942	0.749453136	0.715288248	0.624828139	1.290540101	1.437911221	0.945762064	1.257868887
LCA5L	0.08175674	0.11300911	0.129798691	0.123946953	0.07114054	0.013993532	0.070062254	0.026666462	0.103800405	0.074707995	0.044032695	0.155985791
LCAT	0.993529558	1.318383969	1.261878463	0.803325976	1.161913118	0.612191195	1.798188616	1.049947611	2.075920487	3.094025538	1.88354133	2.150622407
LCE1A	0	0	0	0	0	0	0	0	0	0	0	0
LCE1B	0	0	0	0	0	0	0	0	0	0	0	0
LCE1C	0	0	0	0	0	0	0.07290536	0	0	0	0	0
LCE1D	0	0	0	0	0	0	0	0	0	0	0	0
LCE1E	0.091202646	0.047274679	0.048265069	0	0	0	0	0	0	0	0	0
LCE1F	0	0	0	0	0	0	0	0	0	0	0	0
LCE2A	0	0	0	0	0	0	0	0	0	0	0	0
LCE2B	0	0	0	0	0	0	0	0	0	0	0	0
LCE2C	0	0	0	0	0	0	0	0	0	0	0	0
LCE2D	0	0	0	0	0	0	0	0	0	0	0	0
LCE3A	0	0	0	0	0	0	0	0	0	0	0	0
LCE3B	0	0	0	0	0	0	0	0	0	0	0	0
LCE3C	0	0	0	0	0	0	0	0	0	0	0	0
LCE3D	0	0	0	0	0	0	0	0	0	0	0	0
LCE3E	0	0	0	0	0	0	0	0	0	0	0	0
LCE4A	0	0	0	0	0	0	0	0	0	0	0	0
LCE5A	0	0	0	0	0	0	0	0	0	0	0	0
LCE6A	0	0	0	0	0	0	0	0	0	0	0	0
LCK	0.021523824	0	0	0.021754067	0	0.022104183	0.022134067	0.021061171	0.023423303	0.094406999	0	0
LCLAT1	3.136411409	2.99305032	3.404739579	4.316025187	4.17349259	3.741292442	3.779430836	3.305070975	4.279627515	3.183467216	3.898186616	3.372822632
LCMT1	9.359686132	10.59826702	9.760200647	11.79857958	9.953502196	9.292837063	9.58953487	11.82832151	11.53875797	11.36156117	13.09532714	12.40026964
LCMT2	3.742287689	2.978288197	3.280736261	3.457574821	3.927352626	3.784962916	3.343044916	3.217987189	7.918837011	5.03620055	6.7795038	7.356370975
LCN1	0	0	0	0	0	0	0	0	0	0	0	0
LCN10	0	0	0	0	0	0	0	0	0	0	0	0
LCN12	0.017022955	0.017647618	0.036034661	0	0	0.034963909	0	0	0.166727084	0.186663633	0.091682543	0.177155643
LCN15	0	0	0.074741183	0	0	0	0	0	0	0.154867124	0	0
LCN2	0	0	0	0.064361146	0.066493253	0	0.065485406	0	0	0	0	0
LCN6	0	0	0	0	0	0	0	0	0	0	0	0
LCN8	0	0	0	0	0	0	0	0	0	0	0	0
LCN9	0	0	0	0	0	0	0	0	0	0	0	0
LCNL1	0	0.030383509	0.031020033	0	0	0	0	0	0.159472378	0.064274918	0.157847777	0.03050049
LCOR	4.488629951	4.055055008	4.165011478	4.369505831	3.91164993	3.178238868	3.703124945	2.896175247	3.298125617	3.130814143	2.541095764	3.290953852
LCORL	1.04733664	1.15460741	1.022262173	1.348342188	1.509618384	1.577718948	1.163938072	0.936222699	3.662354534	4.520976854	2.971561428	3.439465685
LCP1	607.8584798	582.5130322	613.725163	498.5843189	466.9760106	495.4258546	524.6667473	453.1617804	349.7685146	354.1779673	392.6760109	294.033817
LCP2	23.61760107	25.28914803	22.5451899	24.12337243	18.64611935	20.6021072	21.36398499	23.10994248	26.50589015	23.12389258	23.99671185	27.54984225
LCT	0.008477952	0	0	0	0	0	0	0	0	0.009296419	0	0.017645785
LCTL	0.015633225	0.016206892	0	0	0.016323881	0	0.032152916	0.01529719	0.017012858	0	0.016839542	0
LDAH	12.13749493	11.11488139	12.30408458	11.80389875	13.98333109	12.43706561	11.12250743	11.34875147	7.763738844	8.236951507	9.209954917	7.550728715
LDB1	36.52123453	37.84667924	35.0640442	37.5144331	35.5854665	36.98121429	35.55696886	36.90288329	23.84963042	19.75113454	19.63145014	29.29195895
LDB2	0	0	0	0	0	0	0	0	0	0.050046119	0	0
LDB3	0.027372856	0.011350925	0	0.0165994	0.005716431	0.028110926	0.045038289	0.042855166	0.017873107	0	0.011794018	0.005697314
LDHA	295.9842002	416.5782531	343.612883	285.7511365	560.5695056	474.5083324	372.2173278	320.0011108	335.5459235	514.0522108	491.9366216	166.8780346
LDHAL6A	0	0	0	0	0	0	0	0	0	0	0	0
LDHAL6B	0	0	0	0	0	0	0	0	0	0	0	0
LDHB	601.0431614	572.3935965	666.3038435	466.3215249	617.0201431	592.3186237	673.5606087	490.493673	455.4065168	595.3858275	592.0691673	372.972717
LDHC	0	0	0.0440811	0	0	0	0	0	0	0	0	0
LDHD	0.944732671	1.812931859	0.808444411	0.203157148	1.091413347	1.527558436	0.930176524	0.354035379	6.562374718	4.540493506	4.525213323	5.104120776
LDLR	39.63414286	45.73368948	32.93862598	32.09177814	34.40962914	39.53151654	30.62074771	32.41483267	21.57701107	27.30236474	27.32448975	9.26249765
LDLRAD1	0	0	0	0	0	0	0	0	0	0	0.024394656	0.02356856
LDLRAD2	0	0	0.013608789	0	0	0	0.013222262	0	0.013992415	0.014099014	0.027699739	0.04014258
LDLRAD3	2.575775539	1.376870618	2.555846581	1.532167552	3.375970698	2.36968309	3.421371636	2.257866784	2.905283759	2.647915079	3.352559274	3.113376824
LDLRAD4	1.004345696	1.100476783	0.868302972	1.002526323	0.783941473	0.86292608	0.777172143	0.705444639	1.195784228	1.084949919	0.870295851	2.012804033
LDLRAP1	5.415595621	6.043498818	6.035975409	7.33503844	7.198685458	6.819708028	5.86453734	8.143057686	5.157981192	5.067576192	4.978026676	5.627146374
LDOC1	0	0	0	0.036996713	0	0	0	0	0	0.04013903	0	0
LEAP2	0.91331645	1.341343803	0.483333363	1.615401047	0.476832803	1.1724284	0.704408078	1.415000903	2.816097228	2.587179073	1.885599978	2.296984535
LECT2	0	0	0	0	0	0	0	0	0	0	0	0
LEF1	0	0	0	0	0	0.026773478	0	0	1.744831787	2.958794935	1.727056577	2.007712458
LEFTY1	0.033093211	0	0	0.100341639	0.069110453	0.033985522	0.068062937	0.097145624	0.072027376	0.036288053	0.142587217	0.413276006
LEFTY2	0	0.045114534	0	0	0	0	0	0	0	0	0	0
LEKR1	0.132007509	0.119745125	0.122253747	0.091725982	0.129224468	0.084729314	0.110297023	0.064585009	0.278335782	0.235221364	0.186629221	0.248998477
LELP1	0	0	0	0	0	0	0	0	0	0	0	0
LEMD1	0	0	0	0	0	0	0	0	0	0	0	0
LEMD2	6.019879822	6.133297581	6.296080592	7.001173559	6.076077212	6.494967192	6.203882622	7.405903213	8.102533401	8.711387639	8.292209049	10.39189585
LEMD3	15.07295744	12.80081659	14.31535103	17.79589719	18.66647708	14.67316053	16.0884867	13.87105073	14.34054857	11.53524451	12.24951386	12.52330522
LENEP	0	0.085953963	0	0	0	0.085147086	0	0.081129319	0.180456878	0.090915831	0	0.086284898
LENG1	5.235524841	4.824572687	5.47978112	7.231757554	7.106870746	6.810478137	5.025031461	7.684481431	6.457234833	5.932331691	6.140807523	6.053935009
LENG8	54.04357714	49.98369049	47.56322814	38.53118956	48.40446793	46.62328094	39.65638415	42.653868	61.7145996	62.62021964	53.35562731	76.13134428
LENG9	7.273839386	6.230585617	6.985335911	4.938945374	5.982310501	5.191483581	4.967457659	5.825898124	2.56727223	1.847736149	2.662124176	2.922698269
LEO1	24.11189665	23.95918376	24.36258681	28.90976307	28.55514204	29.49455271	28.5292042	27.32850951	31.02675818	32.76886534	31.06165693	32.11701551
LEP	0	0	0	0	0	0	0	0	0	0	0	0
LEPR	0.717356847	0.74368051	0.585950945	0.890526598	0.806041598	0.736699346	0.416958228	0.60275052	5.082799012	7.113699385	4.434687659	6.329393
LEPROT	5.926301958	6.383420292	5.838744422	5.002033667	6.122287373	5.837904332	5.889019038	5.397927626	10.41721777	9.770691163	9.767809072	10.66177043
LEPROTL1	24.10914548	21.50179206	24.63067536	23.33014859	22.15691678	21.68626194	27.16645515	23.37341623	28.17197369	24.97420611	28.80588005	25.41036782
LETM1	12.94503167	10.39696739	13.01301191	11.75922448	12.17963559	12.19129487	13.52479845	13.63076541	9.273689557	8.577347717	10.43140877	9.914122149
LETM2	0.610887701	0.793297142	0.877976672	0.779902035	0.664733875	0.746227512	0.952230422	0.817982873	0.580824012	0.662812045	0.491787878	0.642434786
LETMD1	18.32006432	17.19785678	16.43561112	13.89461169	20.60252267	18.82431389	16.95655572	14.72480554	12.67419324	15.50263685	12.25408276	14.36937159
LEUTX	0	0	0	0	0	0	0	0	0	0	0	0
LEXM	0	0	0.019073269	0	0	0	0	0	0	0	0	0
LFNG	2.898487119	2.35085177	2.88733999	1.602604778	1.05038692	1.103108013	2.629998473	1.351358427	1.373038981	0.841317125	1.432513589	1.561439511
LGALS1	1230.580298	867.2531465	1145.485779	442.2937188	683.8074364	483.1823967	1047.599845	462.2700643	1069.475265	881.0473962	1190.604027	453.8118648
LGALS12	1.542758913	1.720535465	1.26176883	1.228509456	1.415653484	1.776400493	1.5624613	1.326615918	1.246461597	1.23032579	1.309299996	0.681133435
LGALS13	0	0	0	0	0	0	0	0	0	0	0	0
LGALS14	0	0	0	0	0	0	0	0.048617662	0	0	0	0
LGALS16	0	0	0	0	0	0	0	0	0	0	0	0

LGALS2	0.203824094	0.316955237	0.431460464	0.103002213	0.212828783	0.209319919	0.628808726	0.19944291	1.219963689	0.447002836	1.536863355	0.424234082
LGALS3	3.463247433	2.893700837	2.516645475	0.104486395	1.889085449	1.698688448	3.986683565	1.315058729	5.062673521	6.008130337	8.463188238	5.271750323
LGALS3BP	0.523809884	0.493664794	1.083614866	0.216578105	0.37292123	0.268966834	0.367268813	0.186382047	8.55054196	4.438382141	5.308881707	11.32367114
LGALS4	0.03001091	0.03111217	0.031763961	0.12132776	0	0.061640221	0.061723556	0.088097482	0.489890606	0.723980053	0.905146516	0.468479354
LGALS7	0	0	0	0	0	0	0	0	0	0	0	0
LGALS7B	0	0	0	0	0	0	0	0	0	0	0	0
LGALS8	8.323389737	10.55375275	7.418004626	9.155496619	8.270840209	8.316349055	7.599134953	7.451331933	17.73041472	15.05267673	13.47449199	19.13914693
LGALS9	41.84818889	39.5087296	35.94056344	25.20288301	27.37350054	37.29526472	36.26939222	30.52861976	29.55935483	28.41828465	29.36559299	25.70866396
LGALS9B	0	0	0	0	0	0.044457328	0	0.084719112	0	0.047469328	0	0
LGALS9C	0	0	0	0	0	0	0.031440436	0	0	0	0.065865572	0
LGALSL	0.056242029	0.014576462	0.014881835	0.014210914	0	0.014439629	0	0.027516555	1.729052273	2.960240364	2.074929031	2.341213435
LGI1	0	0	0	0	0	0	0	0	0	0	0	0
LGI2	0	0	0	0	0	0	0	0	0.916017495	2.162640348	1.138320739	1.329957862
LGI3	0.032951354	0	0	0	0	0.01691992	0.016942795	0	0	0	0	0.017146019
LGI4	0.105405604	0.16391025	0.055781372	0.159799712	0.110062289	0.144330287	0.072262707	0.103139918	0.420594729	0.154108715	0.132462268	1.554001437
LGMN	0.091746907	0.095113592	0.048553096	0.046364168	0.071850126	0.047110365	0.023587028	0.044887407	0.299530727	0.352114766	0.34589252	0.262569432
LGR4	51.32208928	46.72652792	54.47231633	37.06710249	38.0803747	38.38147609	40.53684218	31.5012362	10.1232517	12.08265316	9.43330879	12.39784599
LGR5	0	0	0.024703006	0	0	0	0	0	0	0	0	0
LGR6	0	0	0	0	0	0	0	0	0	0	0	0
LGSN	0.278546231	0.17326055	0.129719556	0.084457763	0.069804492	0.097259322	0.143221782	0.065414136	0.109126061	0.054978711	0.084011166	0.156534866
LHB	0	0	0.091711403	0	0	0.088986246	0.178213101	0	0	0.285045287	0.186672153	0
LHCGR	0	0	0	0	0	0	0	0	0	0	0	0
LHFPL1	0	0	0	0	0	0.022104183	0	0	0.023423303	0.047203499	0.023184681	0
LHFPL2	0.282338366	0.2284479	0.262388038	0.139199305	0.136620063	0.176799522	0.205364713	0.06064453	1.641189954	1.955737517	1.691229643	1.476295516
LHFPL3	0	0	0	0	0	0	0	0	0	0	0	0
LHFPL4	0	0	0	0	0	0	0	0	0	0	0	0
LHFPL5	0.025062674	0	0	0	0	0	0	0	0	0	0	0
LHFPL6	3.715303322	3.593138515	3.932328273	5.065532386	2.785907068	2.227831277	2.025708185	3.757437888	0.02713543	0	0.026858992	0.07784833
LHPP	1.443617525	1.78527422	1.163409668	1.903443869	1.277842213	1.362133052	1.122829908	1.921692059	0.470507581	0.650872167	0.370992792	0.861756172
LHX1	0.047091214	0	0	0	0.016390548	0	0.048426343	0	0.051247016	0.051637434	0.050724945	0.016335735
LHX2	0.480247602	0.497870457	0.785555604	0.110314744	0.296319831	0.179344287	0.404070191	0.1495215	3.23080039	2.36974972	3.621136861	3.316770479
LHX3	0	0	0.018189965	0	0	0	0	0.050449947	0.037405466	0	0	0
LHX4	0.014666002	0.030408352	0.023284048	0	0	0.030122899	0.007540906	0.021526136	0.015960277	0	0.007898842	0.015262714
LHX5	0	0	0.05465718	0	0	0	0.053104768	0	0	0	0	0
LHX6	0	0.010823462	0	0	0	0	0	0	0.022723421	0.022896536	0.04498386	0.032595401
LHX8	0	0	0	0	0	0	0	0	0	0	0	0
LHX9	0	0.006482003	0.006617799	0	0	0.006421155	0	0.006118165	1.741914224	0.74046856	1.629878258	0.260278405
LIAS	9.928165663	9.354218164	11.43700093	11.30945402	10.50996692	10.87183334	11.47880394	9.768433186	4.656008207	4.901994403	5.228961033	5.76543199
LIF	0.024952266	0.051735796	0.052819644	0.012609592	0.013027312	0	0.012829856	0.012207959	0.027154304	0.082083526	0.026877674	0
LIFR	0.034482256	0.026810696	0.063868867	0	0	0	0	0	0.469066462	0.694780762	0.496788072	0.488936236
LIG1	18.82698583	23.47416941	22.97297473	34.26394192	32.65940689	33.70496054	21.22489595	36.73568669	24.43388268	23.50410657	28.19295633	23.150845
LIG3	9.345204501	8.806538378	8.617603089	11.60302269	10.48543149	12.02202982	7.805347318	10.81739718	7.895716571	10.25731726	8.546135526	6.496173537
LIG4	9.794866119	6.990211304	8.6552778	6.487996377	6.373841522	6.498725322	9.211156182	5.323723949	7.53639714	7.448301879	6.476916639	9.028182519
LILRA1	2.732403816	2.99753475	2.341154094	2.703185435	1.871886897	2.895161048	2.690936428	3.635626701	2.312752227	2.488900261	2.289191413	2.63294124
LILRA2	8.768028718	13.65651236	7.160925499	11.03467189	10.18977194	15.38981044	8.648151972	12.43623806	22.04249316	22.39531482	23.72502167	29.23875133
LILRA4	0.027411901	0.05683558	0	0	0.028622923	0	0.028189082	0	0.089493006	0.030058265	0.029527103	0.085581608
LILRA5	0.100141863	0.207633207	0.105991528	0.236163883	0	0.102842044	0.137308109	0.097989321	0.217958773	0.10980963	0	0.312648939
LILRA6	0.147625681	0.229564287	0.039062264	0.149204852	0.115610697	0.113704647	0.3795279	0.216678717	0.120490241	0.121408178	0.039754255	0.192040119
LILRB1	1.551170244	1.80217093	1.344561189	0.689275248	0.600405651	0.892626692	1.512641269	1.360811269	1.964553853	1.803563132	2.376660314	1.753444646
LILRB2	0.481238206	0.665196549	0.471619587	0.486386072	0.111666377	0.237954939	0.641514036	0.453453507	4.150866847	1.83716833	3.359820517	6.121111831
LILRB3	0.171095583	0.177373996	0.271634887	0.069170326	0.017865437	0.052712679	0.211135776	0.167417896	0.130336343	0.131329291	0.073719178	0.106834147
LILRB4	26.46951604	31.75577403	23.58156112	11.67164008	15.5806163	21.84715804	20.22615085	13.8686429	44.74762667	43.01532245	45.15621099	36.87490684
LILRB5	0	0	0	0	0	0.022024894	0	0	0.023339281	0	0	0.02231921
LIM2	0	0	0.057878843	0	0	0	0	0	0	0	0	0
LIMA1	10.33554312	11.10822585	11.12829703	8.291453831	7.423976504	7.97786334	10.1809363	7.241006322	6.255445424	6.645794497	6.73274301	4.448782048
LIMCH1	0.005493014	0.011389163	0.005813881	0	0	0	0	0.005374942	0.340733019	0.487888355	0.378679975	0.291541838
LIMD1	6.683594294	8.427157957	6.579696174	7.992565829	7.545611592	8.743415272	6.269501779	7.583602625	17.74055571	17.6680938	14.03154543	16.02595744
LIMD2	37.28041362	31.22917442	37.41388889	14.87496403	17.43135239	15.89852926	37.5983528	18.64974339	5.18128151	3.381998085	5.289771508	4.534134547
LIME1	3.72102852	3.315101776	4.030693869	3.73145133	3.339031793	3.403399393	4.424421851	4.181512946	2.878877035	3.31521066	3.601078293	4.356478354
LIMK1	9.766908381	9.539939133	9.206774062	8.591190842	7.202618543	7.444332904	9.416080751	9.183650289	10.09739657	12.33301869	11.75343679	13.1818168
LIMK2	5.306084949	6.389469878	5.22555263	5.472514445	5.053299494	5.360008181	4.77585236	5.531795857	7.864448317	8.079042182	8.205105055	9.214579845
LIMS1	9.811429901	10.13800502	8.966924632	9.043807464	8.896874997	9.015351465	9.483895169	7.279352214	9.614828533	9.750010576	11.07260616	8.816740708
LIMS2	0.011715559	0	0.012399909	0	0.012233137	0.024062904	0	0.011463734	0.114745115	0.128465871	0.265011055	0.060961135
LIMS3	0	0	0	0	0	0	0	0	0	0.048523334	0	0
LIMS4	0.10357952	0.161070611	0.068518746	0.065429702	0.310947153	0.119668943	0.026629051	0.114022222	0.070450261	0.056789581	0.111572096	0.067371149
LIN28A	0	0	0	0	0	0	0	0	0	0.366577872	0.014404002	0.013916227
LIN28B	2.589666044	2.099814825	2.809461711	1.79476665	3.196601985	2.915943757	2.672599205	1.212700652	0.010065015	0.040566775	0	0
LIN37	8.796693428	9.410538791	9.409589954	9.978496152	7.96386798	7.92867449	7.458218279	10.89686691	7.485272859	6.721367861	5.59456444	9.592854844
LIN52	0.557581619	0.669312106	0.70818249	1.204209121	0.753815034	0.699194284	0.802746218	0.959101116	2.299408	2.600105501	2.301271829	2.901339109
LIN54	2.933356544	2.659764226	2.968930748	4.978680406	4.491729134	3.767758539	3.544194637	3.447712408	10.31191177	7.999162638	9.175120287	9.656517044
LIN7A	0	0	0.009333461	0	0	0	0	0	39.60503555	29.17341813	32.05846441	31.00037369
LIN7B	1.390427929	2.066078581	1.324483286	2.107952271	1.645435963	1.570710709	1.191541083	2.176865254	2.925390964	2.693567477	2.296501609	4.533933239
LIN7C	7.685495811	6.863792385	7.887936735	7.722873282	7.434444501	7.11825775	7.470019571	6.793226119	8.762014556	9.886759346	8.624969094	9.475493806
LIN9	4.113641751	4.396279066	4.871029598	7.85113656	7.66229994	7.355350668	5.667157197	7.151696093	5.571913329	5.153641558	5.809852991	5.23686815
LINC00452	0.016475677	0	0.034876167	0	0	0	0.016942795	0	0.017929656	0.018066251	0	0
LINC00483	0	0	0	0	0	0	0.026590662	0	0	0	0	0
LINC00694	0	0	0.015724125	0	0.031025289	0.015256891	0.045832552	0.043610929	0.161673819	0.211777158	0.160026791	0.092764603
LINC01638	0	0	0	0	0	0	0	0	0	0	0	0
LINC01835	0	0	0	0	0	0	0	0	0	0	0	0
LINC02054	0	0	0	0	0	0	0	0	0	0	0	0
LINC02210-(	0.096502082	0.060025956	0.061283481	0.058520626	0.020153084	0.118924947	0.019847621	0.094427777	0.21003679	0.35978277	0.103948536	0.180771194
LINGO1	0	0	0.0113384	0	0	0	0	0	0.128238269	0.364152022	0.253863723	0.156078954
LINGO2	0	0	0	0	0	0	0	0	0.011108673	0.033579907	0.008796404	0.025495572
LINGO3	0.126313523	0.130948642	0.133691975	0.306395316	0.131893893	0.103775509	0.181852664	0.148318108	0.412382092	0.055403168	0.21769654	0.131452814
LINGO4	0	0	0	0	0	0	0	0	0	0	0	0
LINS1	3.609444313	3.40666392	3.857285984	3.215234915	3.458631912	2.979774605	3.469120481	3.087170233	3.43341413	2.942072913	3.106604224	3.701730035
LIPA	52.1636597	57.86208868	48.27705478	51.98049428	38.11581736	41.4704471	45.68251594	44.75977281	35.20234412	36.91793986	33.87828227	35.93630122
LIPC	0	0	0.011491683	0	0	0	0.011165288	0	0.011815629	0.011905645	0	0
LIPE	0.146363678	0.281792727	0.232370003	0.147929349	0.120080588	0.182519486	0.096758599	0.092068458	0	0	0	0.032639731
LIPF	0	0	0	0	0	0	0	0.034503885	0.153494776	0.463992459	0.037982768	0.477054839
LIPG	0	0.01019633	0.01040994	0	0	0.010100614	0	0	0	0.010784934	0.010594353	0
LIPH	0.061655183	0.095876462	0.032628348	0.062314716	0.080473788	0.015829407	0.095104843	0.015082477	0.067096256	0.033803709	0.116222265	0.096245601
LIPI	0	0	0	0	0	0	0	0	0	0	0	0
LIPJ	0.193803569	0.130594918	0.225636807	0.127319862	0.141655894	0.228883585	0.129543883	0.104300776	0.632720228	0.456904033	0.43839214	0.877346335
LIPK	0	0	0	0	0	0	0	0	0	0	0	0
LIPM	0	0	0	0	0	0	0	0	0	0	0	0
LIPN	0	0	0.006934467	0.013243679	0	0	0.00673751	0.01282185	0.106949209	0.021552797	0.049401184	0.102274867
LIPT1	5.208261112	4.916277664	6.266836863	5.291679675	6.096682684	5.996167944	6.568056096	5.874167755	3.430565238	3.186176098	3.425143976	4.735515643
LIPT2	0.954392549	1.90815619	1.611417376	2.021070455	1.993112789	1.773562015	1.378705619	1.845520714	1.533197609	1.196034615	1.052514044	1.537131936
LITAF	28.17228343	32.5461318	28.09970133	29.97558028	24.40976724	27.27755752	26.99339125	27.72557307	17.88411843	20.40299786	16.53166252	22.69066544
LIX1	0	0	0	0	0	0	0	0	0	0	0	0.014116183
LIX1L	20.23894001	22.6779418	20.07259042	25.52524239	21.60922988	24.09036442	18.95077783	25.7490143	22.33153871	28.86978735	20.25481269	27.69237669
LKAAEAR1	0	0	0	0	0	0	0	0	0	0.058594215	0.115117584	0
LLCFC1	0	0	0	0	0	0	0	0	0	0	0	0.072915233
LLGL1	5.246369876	7.131272621	6.040846187	6.461230081	5.491160033	6.629207396	5.100369804	7.048029776	3.77007769	3.498175042	3.758517143	3.903582341
LLGL2	2.169878689	2.702960312	2.115077788	2.36645697	2.104542187	1.94653512	2.266678059	2.584810635	5.338750883	4.156827138	5.034926442	4.882275688
LLPH	60.92816341	57.75513011	69.4477629	74.58409728	66.66699854	60.11901984	80.88846644	77.78961802	30.45799238	29.67187929	32.95768195	33.6821643
LMAN1	30.44942265	31.68215061	29.97824786	33.05853372	34.39770919	34.74494189	30.62604121	29.1197374	35.36506942	52.98800278	36.85093631	40.56011246
LMAN1L	0.185977745	0.128534843	0.262455213	0.062655724	0.258925339	0.095496184	0.159375484	0.090990084	0	0	0.033388078	0
LMAN2	56.95236818	56.78896668	62.36205202	68.84236134	50.42090445	53.02832214	59.98670492	74.20843937	106.0888948	106.0597187	103.1098211	119.1504955
LMAN2L	4.445504049	5.307573051	4.125397236	5.493881558	4.795897456	5.517391125	4.723205402	5.463205171	11.76209314	11.22792715	11.00682313	14.86580008
LMBR1	11.24100289	11.44923677	10.09762166	12.82856935	9.615316474	10.63888959	10.01343662	10.32967451	11.74776365	12.74694616	11.22596111	14.28840664
LMBR1L	4.152287429	5.751304134	3.719402068	4.437499525	4.09584348	4.867184602	3.727511309	4.387744027	3.01865778	3.49883624	2.254677277	3.57741226
LMBRD1	14.66980109	14.74535042	13.70131013	14.56013186	13.98313993	13.18999633	13.12436674	13.79856153	31.59761156	25.49736539	26.33629446	30.78672493
LMBRD2	10.25182154	8.791257628	9.35350378	8.188101592	10.69727419	9.185620569	8.698465052	5.879064355	19.21880131	19.53756102	16.48353282	22.04417619
LMCD1	0	0	0	0	0	0	0	0	0	0.018273266	0	0
LMF1	1.245251388	0.999586913	1.148665979	1.166801126	1.119672644	1.06568984	0.978203048	1.129636952	1.18575177	1.175820397	0.950115512	1.480404799
LMF2	10.36697962	11.9415551	11.44280652	14.76881643	11.35763728	12.26883576	9.696345931	16.50435827	17.19141492	14.56163	17.14035701	21.66318258
LMLN	2.000746881	2.269105314	1.807140251	1.763678937	1.939913232	1.985174427	2.134820567	1.221748127	1.841712027	1.995954514	1.814847864	2.536153652
LMLN2	0	0.021538271	0	0	0	0	0	0	0	0	0	0
LMNA	32.47531653	31.86317955	32.20086066	25.14526618	19.32099132	18.82909447	37.14827149	28.51186563	61.83661508	51.37253442	63.15084267	58.57992917
LMNB1	81.79748996	66.71800626	91.56293462	114.8433211	113.6449412	98.00084262	93.20634213	109.0037849	110.5087061	106.7037672	130.0093106	92.08634672
LMNB2	45.35112429	46.83511854	52.63594689	66.61129177	58.47347067	59.88929543	55.3589987	69.9658098	47.29802374	46.09557927	55.31573441	53.04460723
LMNTD1	0	0	0	0	0	0	0	0	0	0	0	0
LMNTD2	0.715776631	0.982114819	0.445639916	0.404271598	0.791363361	1.059374988	0.671122928	0.453194219	3.413607317	3.832052481	2.403732624	4.600848496
LMO1	0	0.048962073	0.049987813	0.015911401	0.09863101	0.113172384	0.032378682	0.030809203	0.171323163	0.327993889	0.118704484	0.114684696
LMO2	55.6286909	47.03615716	47.69795434	34.00618071	36.88854876	34.58488359	44.07949169	32.5789994	55.23108337	48.62832102	54.89895455	52.21260965
LMO3	0	0	0.009473184	0	0	0	0	0	0.029220687	0.068701035	0.057846012	0.009314521
LMO4	15.83555283	16.84278683	15.99401497	19.29892032	11.04727707	12.34264153	13.04372091	17.22855912	15.83778251	14.0910774	14.28485057	22.70308212
LMO7	2.772675077	2.907036893	2.37268567	5.894840303	4.098408495	3.873321135	3.308300491	4.379405966	5.127378451	6.201453758	4.227874598	4.886908728
LMO7DN	0	0	0	0.017623191	0	0	0	0	0	0	0	0
LMOD1	0	0	0.014356638	0	0	0	0	0	0	0.014873802	0	0
LMOD2	0	0	0	0	0	0	0	0	0	0	0	0
LMOD3	0	0	0	0	0	0	0	0	0	0	0	0
LMTK2	2.328586841	2.221161214	2.623410515	3.578769738	2.675860252	2.656397239	2.690846888	3.030215371	2.788799439	2.040079863	2.178573961	2.791825539
LMTK3	0	0.02084219	0	0.010159755	0.073474237	0.030969807	0.020674451	0.009836153	0.196908019	0.330680222	0.259869398	0.219685574
LMX1A	0	0	0	0	0	0	0	0	0	0	0	0
LMX1B	0	0	0	0	0	0	0	0	1.908416611	2.024163786	2.21706002	2.362903793

LNP1	0.1213567	0.150971913	0.205512968	0.367964605	0.202748935	0.274183601	0.074878441	0.332494811	0.237719582	0.266145126	0.209153644	0.17681204
LNPEP	11.23863204	9.52205699	10.1694058	9.616769947	9.323254949	8.176841535	9.285736995	6.91352208	14.63005874	12.25125095	11.54968801	15.88137013
LNPK	5.626773802	6.634914813	5.875605315	7.459771928	8.015431841	7.59920081	6.031979394	6.570081487	4.200814328	5.446133396	4.171563203	4.710738073
LNX1	0.073914232	0.0425703	0.095616696	0.116207625	0.111481743	0.134946175	0.16046523	0.225012514	0.125124481	0.16209993	0.097310553	0.128202607
LNX2	3.49011109	3.778481149	3.740740972	4.599074192	4.589972466	4.536983462	4.009300957	4.095948242	5.048125609	4.166154511	3.307338591	5.275758326
LOC1001281	0.107791588	0.111747039	0.114088104	0.087155719	0.045021473	0.044279214	0	0.084379693	0.961894447	1.111059934	0.812764274	1.054466444
LOC1001282	0	0	0	0	0	0	0	0	0	0	0	0
LOC1001290	0.863254987	0.828641142	0.812160874	0.807860496	0.701083049	1.017869409	0.427425539	1.376547146	1.774492638	2.909900815	2.100810412	1.863302635
LOC1001290	0	0	0	0	0	0	0	0	0.024368813	0.024554463	0	0
LOC1001293	0	0	0	0	0	0	0	0	0	0	0	0
LOC1001294	0	0.168595759	0.06454792	0.123275788	0.021226596	0.020876637	0.083619445	0.218807031	0.0663675	0.06687311	0.175177042	0.148089268
LOC1001296	0	0	0	0	0	0.035332774	0	0	0.112324022	0	0.074119826	0.14321969
LOC1001299	0.645423249	0.47485033	0.418689435	0.178863971	0.347838568	0.203124146	0.267629947	0.183353204	0.04531496	0.011415046	0.044853321	0.010833604
LOC1001303	0.840409627	0.580832504	0.39533384	0.707832997	0.65815339	0.551405877	0.408111867	0.525387137	0.508097675	0.563165395	0.326898979	0.461596129
LOC1001303	1.76307162	2.863857662	2.028797048	3.149460187	4.575646522	3.994922188	2.245240644	3.60581536	3.29630726	2.635779748	2.208275213	4.768360373
LOC1001304	0.28561338	0.059218813	0.362756569	0	0.059646283	0.117325814	0.058742216	0.167684485	0.062163755	0	0	0
LOC1001304	0	0	0	0	0	0	0	0	0	0	0	0
LOC1001307	0.459434802	0.447427626	0.32418142	0.239210315	0.159910682	0.171571928	0.400875731	0.408690259	0.34847087	0.259527649	0.359917435	0.405684131
LOC1001308	0	0	0	0	0	0	0	0	0	0	0	0
LOC1001311	0	0	0	0	0	0	0	0	0	0	0	0
LOC1001322	0	0	0	0	0	0	0	0	0	0	0	0
LOC1001328	0	0	0	0	0	0	0	0	0	0	0	0
LOC1001328	0.026007521	0.269618761	0.096343516	0.131428634	0.162939	0.320505318	0.080234656	0.139966702	0.169816115	0.384997126	0.224114852	0.351853883
LOC1001343	0	0	0	0	0	0	0	0	0	0	0	0
LOC1001445	0	0	0	0	0	0	0	0	0	0	0	0
LOC1002869	0.074391098	0.025706969	0.052491043	0	0.025892534	0	0.025500077	0.024264022	0	0	0	0
LOC1002870	0.168999877	0.455523607	0.357743601	0.068323076	0.141172861	0.31240209	0.27806617	0.033073447	0	0	0	0.035175188
LOC1002873	0	0	0	0	0	0	0	0	0.20740823	0.052247085	0.102647645	0.165286006
LOC1002878	1.867945308	1.562395519	1.595127208	1.973741819	1.307700639	1.613125812	1.899076767	2.035497821	1.824892429	1.536208563	1.280416333	2.452022789
LOC1002885	0	0	0	0	0	0	0	0	0	0	0	0
LOC1002892	0	0	0	0	0	0	0	0	0.045910041	0.080954649	0.079524094	0.142686336
LOC1002895	9.410046358	8.701956587	9.11805612	6.920936891	9.410596815	9.164706593	7.814161638	9.207778106	8.365465295	7.993203422	7.709192112	8.091794906
LOC1005055	0	0	0	0	0	0	0	0	0	0	0	0
LOC1005055	3.393640317	1.716867519	2.183860433	1.344537468	2.296231427	1.644988424	2.875641923	1.487671032	1.920427199	2.351839341	1.930107185	1.384432918
LOC1005058	0	0	0	0	0	0	0	0	0	0	0	0
LOC1005062	0.925873011	1.468003203	2.190491589	2.091737254	2.047292258	1.789812423	2.520326472	2.344867253	1.24465931	2.090235933	1.114648147	0.396753332
LOC1005063	0	0.109703288	0.112001536	0	0.220990358	0.27168367	0.054410195	0	0.345476443	0	0.056992826	0
LOC1005064	0	0.021953609	0.022413531	0	0	0	0	0	0.023045359	0	0	0.044076268
LOC1005065	0	0	0	0	0	0	0	0	0	0	0.01269976	0
LOC1005065	0.160889702	0.158453926	0.076629546	0.154480222	0.083998802	0.115659504	0.107543307	0.055100986	0.288895572	0.229348742	0.199300222	0.343243363
LOC1005072	0	0	0	0	0	0	0	0	0	0	0	0
LOC1005075	4.988758121	6.993344703	4.449954924	1.99782252	2.29333872	2.867743914	4.58168737	2.456113275	1.604803546	3.4404883	2.095408529	1.92649273
LOC1005096	0	0	0	0	0	0	0	0	0	0	0	0.015325369
LOC1006527	0	0	0	0	0	0	0	0	0	0	0	0
LOC1006528	0	0	0	0	0	0	0	0	0	0	0	0
LOC1006528	0	0	0	0	0	0	0	0	0	0	0	0
LOC1006529	0.416383507	0.597687018	0.339004651	0.485581862	0.301000707	0.526290082	0.362313599	0.156705144	0.174280527	0.24585156	0.069002028	0.199996067
LOC1006531	0.075153018	0	0.079542991	0	0	0	0.038641877	0.036768805	2.085524518	1.153716817	1.740470152	0.938529031
LOC1009962	0.010546758	0	0	0.021319157	0.033038102	0.021662273	0.032537339	0.010320057	0.02295502	0.0231299	0.02272117	0.043903488
LOC1009963	0.040806037	0.028202286	0.043189672	0.02749503	0	0.041906313	0.027975312	0.013309638	0.088814343	0.104406122	0.04395478	0.056621738
LOC1009963	0	0	0	0	0	0	0	0	0	0	0	0
LOC1009963	0	0	0	0	0	0	0	0	0.054523517	0	0	0
LOC1009964	0.179965086	0	0.317462549	0	0.187915714	0.184817587	0.123378301	0	0.065282338	0	0	0.062429096
LOC1009964	0	0	0	0	0	0	0	0	0	0	0	0
LOC1009965	0.028500827	0.044320012	0	0.057611407	0	0	0.021981661	0	0.093048078	0.062504634	0.069075124	0.14088706
LOC1009965	0	0	0	0	0	0	0	0	0	0	0	0
LOC1009965	0	0	0	0	0	0	0	0	0	0	0	0
LOC1009966	0	0	0	0	0	0	0	0	0	0	0	0
LOC1009966	0	0	0	0	0	0	0	0	0	0	0.008294462	0
LOC1009966	0	0	0.069880713	0	0	0	0	0	0	0	0	0.068710305
LOC1009967	0	0	0	0	0	0	0	0	0	0	0	0
LOC1009967	0.334628412	0.299292934	0.277784569	0.397891735	0.226090032	0.269530343	0.323873681	0.359537128	1.178162714	0.805815136	0.749169685	1.126669712
LOC1009967	0.367079669	0.374153992	0.388522184	0.361653265	0.425169538	0.323123525	0.320388211	0.449741247	0.886229068	0.896363174	0.691127858	0.831444325
LOC1009967	0.052141047	0.054054381	0.220747214	0.105397614	0	0.107093912	0.107238697	0	0.453939977	0.514573032	0.561644415	0.379837492
LOC1009967	0.096346573	0	0	0	0.02515076	0.024736105	0.123847735	0.047137805	0.078636872	0.026411985	0.025945257	0.075199954
LOC1009967	0.619048045	0.756952684	0.672009219	0.529413146	0.580099691	0.831352032	0.734537627	0.419359606	0.794595819	0.974703529	0.820696689	0.759867064
LOC1009967	0.401219729	0.39203788	0.327034312	0.368223202	0.438206949	0.35520521	0.294033288	0.230142213	1.229582875	1.021501853	0.81965042	0.993466923
LOC1009967	0	0.043959119	0	0.085713426	0	0	0	0.041491669	0	0.046496749	0.045675101	0
LOC1009968	0.757253754	0.831220356	0.754341474	0.945437534	1.023269511	1.097889905	0.916145164	0.828150363	0.533229162	0.732670211	1.007612398	0.556280452
LOC1009969	0	0	0	0	0	0	0	0	0	0	0	0
LOC1010599	0.673555004	0.310342819	0.501670304	0.201706699	0.156291512	0.204953022	0.102615054	0.170871812	0.108592039	0.136774164	0.134357218	0.181730316
LOC1010599	0	0.030466478	0.031104741	0	0	0	0	0	0	0.032225218	0.031655764	0
LOC1010600	0	0	0	0	0	0	0	0	0	0	0	0
LOC1010600	0.197321455	0.429580695	0.146193425	0.179489005	0.185434979	0.283698724	0.162332726	0.154464036	0.236208591	0.281282313	0.276311752	0.246419797
LOC1010600	0	0	0	0	0	0.038833773	0	0	0.08230254	0.041464775	0.040732048	0.039352705
LOC1010601	0.924888882	0.860486672	1.154617867	0.886844971	1.089563305	1.193372618	1.536410567	0.951419154	0.309695498	0.104018289	0.332085565	0.345519869
LOC1010602	0	0	0	0	0	0	0	0	0.015695057	0.015814627	0.015535166	0
LOC1010603	0.391091293	0.214646043	0.243492011	0.093005846	0.096086872	0.094502708	0.141945706	0.067532614	0.525747498	0.403621201	0.223024939	0.335179386
LOC1010605	0	0	0	0.088001891	0	0	0.089539107	0.085198913	0.094754461	0	0	0
LOC1019272	0.142604843	0.098558519	0.301869866	0.09608687	0	0	0.146647971	0.046513188	0.103459818	0.46911605	0.460826266	0.296813951
LOC1019272	1.647548657	1.192381573	0.855443278	1.225315757	1.119840874	1.197150316	0.847129953	0.836484996	0.118402022	0.068173743	0.083711299	0.177928333
LOC1019273	0.133965712	0.046293877	0	0.04513292	0.046628049	0.091718603	0.137763903	0.262172257	0.24298032	0	0.048100999	0.046472115
LOC1019273	3.019374157	1.229710098	1.483739793	0	0.337796385	0.110742402	0.66535272	0	0.469404866	0.118245239	0.696934313	0.224444484
LOC1019273	0	0	0	0	0	0	0	0	0	0	0	0
LOC1019273	2.551088663	2.426582029	1.698005699	1.169573518	2.828563176	3.349118701	2.352961684	1.080851901	0	0	0	0
LOC1019274	0	0	0	0	0	0.020573514	0	0.019602728	0	0.065902131	0	0
LOC1019275	0.011894244	0	0	0.024042957	0.012419717	0	0	0	0.025887824	0.039127569	0.012812048	0.012378183
LOC1019275	0.314843175	0.267051646	0.282744304	0.173569687	0.29886595	0.205757027	0.304147199	0.130698758	0.093444027	0.094155917	0.061661387	0.059573297
LOC1019275	0.010875012	0	0.011510263	0	0.011355456	0	0	0	0.035504198	0	0.035142504	0.033952445
LOC1019275	0	0	0	0	0	0	0	0	0	0	0	0
LOC1019275	1.024944016	1.711893682	1.386152349	1.151008859	1.902621753	1.695823596	1.229670397	1.894391072	0.991462553	1.436085302	1.226702723	1.303678068
LOC1019277	0.134467874	0.371739253	0.047440884	0.045302098	0.140408493	0.253171614	0.207420454	0.131577497	0.58533868	0.835547159	0.748360189	0.5364326
LOC1019278	0.13777137	0.102019242	0.08332521	0	0.0616534	0.101061556	0.080958548	0.057775708	0.1499297	0.215817024	0.063600992	0.16385925
LOC1019278	0.753899277	0.521042584	0.26597913	0.095245479	0.065600465	0.129037848	0.161515376	0.030737261	0.512769326	0.413340626	0.338363711	0.261524338
LOC1019280	0	0	0	0	0	0	0	0	0	0	0	0
LOC1019280	0	0	0	0.006870284	0	0	0	0.006651456	0	0	0	0.021222423
LOC1019281	0.232941822	0.434681468	0.394478139	0.659214165	0.389172631	0.526290082	0.527001599	0.95732597	1.368894321	0.766290576	0.752749398	1.745420224
LOC1019281	0	0	0	0	0	0	0	0	0	0	0	0
LOC1019282	0.706986931	0.488620044	0.748284718	0.079394407	0.738220712	0.242016607	0.565468869	0.153731178	0.170973013	0.086137773	0	0
LOC1019282	0	0	0	0	0	0.007402607	0	0.007053306	0	0	0	0
LOC1019282	0	0.056290738	0.057470011	0	0	0.055762319	0	0	0	0.119080473	0	0.056507466
LOC1019284	0	0.018833262	0	0	0	0	0	0	0	0.01992045	0	0
LOC1019284	0	0	0	0.032507572	0	0	0	0	0	0	0.06929074	0.033472145
LOC1019285	0.547834896	1.325188411	0.837540902	0.246086736	1.588993174	1.875354929	0.876348813	0.357372817	1.788544051	3.40409852	2.950539213	0.063347171
LOC1019285	0	0	0	0	0	0	0	0	0	0	0	0
LOC1019285	0	0	0	0	0	0	0	0	0	0	0	0
LOC1019286	0.028546186	0.044390548	0.030213677	0.028851548	0.05961464	0.029315893	0.014677763	0.013966294	0	0	0.015374457	0
LOC1019287	0	0.013138041	0.013413279	0	0	0	0	0	0	0	0	0
LOC1019287	0.028328276	0.029367792	0.044974557	0.042946961	0.044369675	0.116368431	0.058262877	0.013859681	0.07707062	0.031063108	0.045771285	0.029480863
LOC1019288	0.043894321	0.030336693	0.025810197	0.024646591	0.035648291	0.035060565	0.045138812	0.038178503	0.265377762	0.085567841	0.210139413	0.38574425
LOC1019288	0	0	0	0	0	0	0	0	0	0	0	0
LOC1019290	0	0	0	0	0	0	0	0	0	0	0	0
LOC1019291	0.079895413	0	0.211406018	0.040375032	0.041712545	0.082049679	0	0	0.043473094	0.043804287	0	0.083146101
LOC1019292	0	0	0	0	0	0	0	0	0	0	0	0
LOC1019293	0.022752457	0.023587366	0	0.022995843	0	0	0	0	1.163737033	0.748469865	0.735243598	0.757701802
LOC1019293	0.029276148	0	0	0	0.030569531	0	0	0.02864686	0.06371954	0.032102489	0.063070407	0.060934602
LOC1019294	0	0.021098382	0	0	0	0	0.020928581	0	0	0	0	0
LOC1019294	0	0.043786595	0	0.042688515	0.044102668	0.086751112	0	0.041328829	0.045964095	0	0	0
LOC1019295	0	0	0	0	0	0	0	0	0	0	0	0.044024292
LOC1019295	0	0	0	0	0	0	0	0	0	0	0	0
LOC1019296	0	0	0	0	0	0	0	0	0	0.02831304	0	0.026870873
LOC1019296	0	0	0	0	0	0	0	0	0	0	0	0
LOC1019296	0.065184205	0.101364243	0.068991861	0.131762977	0.136127919	0.100412705	0.100548458	0.031891537	0	0.071477134	0	0
LOC1019297	0	0	0	0	0	0	0	0	0	0	0	0
LOC1019297	0	0	0	0	0	0	0	0.052811362	0	0	0	0
LOC1019297	0.394078009	0.445678735	0.505572848	0.398293512	0.423957202	0.429231258	0.528054199	0.292126766	0.818723967	0.824961292	0.604571698	0.633809108
LOC1019298	0.006998252	0.014510111	0	0	0.014614852	0	0.007196666	0.006847825	0.159932813	0.253237658	0.135688733	0.080112874
LOC1019298	3.034990475	2.721176668	2.517729661	3.288534077	3.939927953	3.509937663	2.052574825	3.959671439	3.451604583	3.148099241	3.328085627	2.817017777
LOC1019299	0.034895954	0.036176473	0.036934359	0	0.036437613	0	0	0.17072934	0.683559421	0.918356021	0.97730499	0.798946676
LOC1019299	0.058425148	0.423983553	0.061837982	0.236200515	0.122012592	0	0	0	0	0	0	0.060802279
LOC1019299	0.076870801	0.14799869	0.046492066	0.122089154	0.045866774	0.033832934	0.067757348	0.075218469	0	0.012041709	0	0.011428347
LOC1019299	0	0	0	0	0	0	0	0	0	0	0	0
LOC1019300	0	0	0	0	0	0	0	0	0	0	0	0.052433426
LOC1019303	2.852133249	2.391053054	2.321267206	2.664103169	2.612589374	2.865592092	2.117687251	2.156090061	3.809760314	3.703297893	2.85039377	4.693363508
LOC1019304	0	0	0	0.180801757	0.037358244	0.036742326	0	0.140034384	0.077870023	0.039231632	0.077076734	0.074466621
LOC1019304	0.213191604	0.088405898	0.090257974	0.215472142	0.044522027	0.21894001	0	0.375496319	0.092802309	0.280527929	0.459284498	0.221865685
LOC1027233	0.052780344	0.072956183	0.083795166	0.106689884	0.450082252	0.189712217	0.081415156	0.146329865	0.181887671	0.057875796	0.123181649	0.08239171
LOC1027233	0	0	0	0	0	0	0	0	0	0	0	0

LOC10272345	0	0	0	0	0	0	0	0	0	0	0	0
LOC10272353	0.502110988	0.433780107	1.062882388	0.507482133	0.262146805	0.429708076	0.516346823	0.655090866	0	0.183528381	0	0.087090045
LOC10272362	0	0	0	0	0	0	0	0	0	0	0	0
LOC10272372	0.122155643	0.443233657	0.129291217	0.493849431	0.255104648	0.125449395	0.251237993	0.418354709	0.066467942	0.937640455	0.26316324	0.889880345
LOC10272372	0.586010592	0.250153012	0.182424027	0.209039726	4.103328023	0.212404069	0.283588304	0.033730255	0.300106378	4.233497711	0.408442499	0.502232277
LOC10272373	0	0	0	0	0	0	0	0	0	0	0	0
LOC10272375	0.026180455	0.054282311	0.036946339	0.00882017	0.018224716	0.008962124	0.026922722	0.008539236	0.370381451	0.220094163	0.206804651	0.326947836
LOC10272389	0.041973136	0.130540066	0.088849893	0	0.131482368	0.043104882	0	0.082141854	0.045677268	0.092050506	0.090423875	0.174723553
LOC10272397	0	0	0	0	0	0	0	0	0	0	0	0
LOC10272399	0.958372898	0.556018206	1.153945242	0.577620255	0.762010504	0.614005095	0.777585692	0.653859894	1.04294935	0.85807015	1.108091393	2.223485689
LOC10272400	0	0	0	0	0	0	0	0	0	0	0	0
LOC10272413	0.033736402	0	0.071414146	0	0.035226833	0	0	0.03301124	0	0	0.072679252	0.035109027
LOC10272415	4.697006078	3.434183296	5.878434089	3.714515344	4.749634432	5.754534736	3.982776249	3.612329539	5.79305269	5.909473327	6.532896461	7.632315462
LOC10272420	0.551635759	0.571878228	0.743093139	1.098178303	0.855282116	0.669511614	0.842318492	1.161341383	0.727657745	0.568231005	0.450153026	1.078574628
LOC10272421	0.067941365	0	0.134831395	0.060084624	0.035471464	0.017443327	0.218336363	0.041550606	0.027726447	0.027937677	0.018295992	0.0883821
LOC10272423	0	0	0	0	0	0	0	0	0	0	0	0
LOC10272426	0	0	0	0	0	0	0	0	1.295861221	1.675280775	1.258458702	1.192460894
LOC10272433	0	0	0	0	0	0	0	0	0	0	0	0
LOC10272437	0.344271023	0.892260424	0.327943079	0.661112094	0.79085705	0.388909177	0.601854033	0.741116074	0.561979434	0.792765106	0.222501741	0.680728777
LOC10272447	0.08278394	0.042910863	0	0.125504235	0	0.08501609	0.127696541	0.121506758	0.225224066	0.226939901	0.089171852	0.172304304
LOC10272448	0	0	0	0	0	0	0	0	0	0	0	0
LOC10272448	0	0	0	0	0	0	0.005632651	0	0.005960735	0	0	0
LOC10272462	0	0	0	0	0	0	0	0	0	0	0	0
LOC10272464	0.212266512	0.286072419	0.134799482	0.107268577	0.177315341	0.152592982	0.240113155	0.270015017	0.138599425	0.139655324	0.09145831	0
LOC10272464	0	0	0	0.013586103	0	0	0	0.013153367	0.029257186	0.029480077	0	0.069946164
LOC10272465	0	0.02738543	0	0	0	0	0	0	0.028747303	0.02896631	0	0
LOC10272466	0	0	0	0	0	0	0	0	0	0	0	0
LOC10272473	0.049777577	0.034402789	0.017561758	0.067080072	0.086627812	0.034079839	0.017062956	0.113651094	0.126397718	0.05458314	0.071491463	0.086338111
LOC10272484	0.088292807	0.122043657	0.155750541	0.223093217	0.261214834	0.24179599	0.136194123	0.12959244	0.170817158	0.145224987	0.126807738	0.245027087
LOC10272487	0	0	0	0.095985119	0.019832968	0	0	0	0.020670052	0.041655047	0.020459479	0.019766643
LOC10272490	0	0	0	0	0	0	0	0	0	0	0	0
LOC10272495	0	0	0	0	0	0	0	0	0	0	0	0
LOC10272495	0	0	0	0	0	0	0	0	0	0	0.0374914	0
LOC10272496	0.176296341	0.162458309	0.124396319	0.197980228	0.061361629	0.160933261	0.201438543	0.134172005	0.426343335	0.644387051	0.379800024	0.448480442
LOC10272497	0	0	0	0	0	0	0	0	0	0	0	0
LOC10272518	0	0	0	0.02105504	0	0.021393906	0	0.020384409	0.068011913	0.137060103	0.067319052	0.151758533
LOC10536914	0.096495789	0.054565492	0.064993392	0.150725117	0.10075885	0.081079903	0.090210577	0.085837836	0.229116102	0.192384657	0.094492507	0.164326733
LOC10536915	0.252330883	0.065397564	0.066767622	0	0	0.064783656	0.12974248	0	0.137299548	0.207518315	0	0.262597415
LOC10536917	0	0	0	0	0	0	0	0	0	0	0	0
LOC10536919	0.079314998	0.035239496	0.083948088	0.080163441	0.035493872	0.034908691	0.069911772	0.077610128	0.184959751	0.186368839	0.207485568	0.165084141
LOC10536920	0	0	0.007039029	0	0	0	0	0	0.825069992	1.093889031	0.737863735	0.43603147
LOC10536920	2.394227344	3.1774543	2.415760287	3.210758742	4.105060426	4.544445606	2.385466898	3.965378085	3.893069722	2.175888458	2.819812799	2.520726056
LOC10536920	0	0	0.027781845	0	0	0	0	0	0	0	0	0
LOC10536921	0.571573484	0.592547575	0.748999653	0.632705553	0.397883248	0.614936818	0.391852479	0.719083998	0.148098779	0.149227047	0.117272036	0.141625945
LOC10536924	0	0.020218964	0.020642545	0	0	0	0	0	0.02122445	0	0.021008229	0.02029681
LOC10536926	0	0	0	0	0	0	0	0	0	0	0	0
LOC10536927	0	0	0	0	0	0	0	0	0	0	0	0
LOC10536953	0	0.017085489	0.017443425	0.016657019	0	0	0	0	0	0	0.008876218	0
LOC10536959	0	0	0	0.034312409	0	0	0	0	0	0	0	0
LOC10536960	0.226566572	0.176160384	0.119900592	0	0.177431996	0.058168904	0	0.055424135	0.431481894	0.062109868	0.122024639	0.235784837
LOC10536972	0	0	0	0.112715375	0	0	0	0.10912524	0	0.061144429	0	0
LOC10536980	0	0	0	0	0	0	0	0	0.103062532	0.066084899	0.083464853	0.089598238
LOC10536991	0	0	0	0	0	0	0	0	0	0	0	0
LOC10537004	0	0	0	0	0	0	0	0	0	0	0	0
LOC10537009	0.011129175	0.011537564	0.011779272	0	0	0.011429257	0.034334127	0.010889954	1.27168914	0.878658729	0.839155999	0.775993014
LOC10537029	3.291338716	4.126279272	3.078528719	4.564331362	3.756443152	4.166151215	3.38465465	3.520181552	0	0	0	0
LOC10537030	0	0	0.046078302	0.088001891	0.022729287	0.022354554	0	0.063899185	0	0	0.023447291	0
LOC10537037	0	0	0	0	0	0	0	0	0	0	0	0
LOC10537039	0.070523671	0.036555781	0.037321613	0.035639036	0.036819658	0.072425241	0	0.034503885	0	0	0	0
LOC10537057	0.07617435	0.047381757	0.04837439	0.061591357	0.063631709	0.015645656	0.062667234	0.029814795	0.033158696	0.083528276	0.032820897	0.031709456
LOC10537064	0.058026126	0.08020722	0.081887536	0.117293678	0.060589646	0.059590717	0.059671281	0.075705145	0.042097956	0.148465356	0.104172724	0
LOC10537068	0.124189557	0.018392391	0.093888528	0.161380322	0.037050312	0.036439472	0.127710575	0.121520112	0.598518288	0.680894528	0.535089912	1.126255466
LOC10537069	0	0	0	0	0	0	0	0	0	0	0	0
LOC10537070	0	0.126638188	0	0	0	0	0	0	0	0.066974318	0.06579081	0
LOC10537070	0	0	0	0	0	0	0	0	0	0	0	0
LOC10537070	0	0	0	0	0	0	0	0	0.103520784	0	0.068310788	0
LOC10537073	0	0	0	0	0	0	0	0	0	0	0	0
LOC10537084	0.405192476	0.361252595	0.377397948	0.073714837	0.507712036	0.565920359	0.525017406	0.174452473	0.070552117	0.044431005	0.130937584	0.033734276
LOC10537094	0	0	0	0	0	0	0	0	0	0	0	0
LOC10537098	0	0	0	0	0	0	0	0	0	0	0	0.085625228
LOC10537103	0.256032803	0.044238003	0.135494325	0.258771619	0.178229337	0.219113634	0.087763946	0.083509799	0.092875903	0.093583464	0.045964872	0.044408326
LOC10537104	0	0	0	0	0	0	0	0	0.317202052	0.361307992	0.273017916	0.09232037
LOC10537106	0	0	0	0	0	0	0	0	0	0	0	0.239311533
LOC10537109	0	0	0	0	0	0	0	0	0.031687369	0	0	0.060604869
LOC10537119	0	0	0	0	0	0	0	0	0	0	0	0
LOC10537121	0	0	0	0	0	0	0	0	0.478864252	0.068930344	0.101568406	2.943867345
LOC10537124	0	0	0.022134777	0.063410612	0	0	0	0	0.022758747	0	0	0.108820246
LOC10537125	0	0	0	0	0	0	0	0	0	0	0	0
LOC10537137	0.077759481	0.241838678	0	0.078591284	0.162389591	0.079856154	0	0	0.169243518	0	0	0.080923264
LOC10537140	0	0.073837355	0	0	0	0	0	0	0	0	0	0
LOC10537141	0	0	0	0	0	0	0	0	0.233241751	0.146886668	0.173149227	0.167285732
LOC10537144	0.022993082	0.035755232	0.030420244	0.005809761	0.018006666	0.023613058	0.047289963	0.011249424	0.08757778	0.075638553	0.105261079	0.095714388
LOC10537156	0	0	0	0	0	0	0	0.058928851	0	0	0	0
LOC10537159	0	0	0	0	0	0	0	0	0	0	0	0
LOC10537173	0	0.072259225	0	0	0	0	0	0	0	0.076430537	0.075079927	0.072537434
LOC10537176	0.895130691	1.286514716	0.968950911	0.904706018	1.210830821	1.065513568	0.962350746	1.074955815	0.819151422	1.56155244	0.745065378	1.439669233
LOC10537184	0	0	0	0	0	0	0	0	0	0	0	0
LOC10537191	0	0	0	0	0	0	0	0	0	0	0	0
LOC10537192	0.83031179	1.166218572	0.793766987	0.378990722	1.006831633	0.687661256	0.550872751	0.183459681	0.991030732	2.467081854	1.355998045	0.445984262
LOC10537193	0	0	0	0	0	0	0	0	0	0	0	0
LOC10537193	0.275710816	0.428742182	0.29181613	0.139330065	0.527800841	0.141572481	0.519800894	0.179856288	1.300183334	0.856596383	0.692966568	0.860785806
LOC10537193	0.276893109	0.0478423	0.244222904	0.27985507	0.43368884	0.189572757	0.189829049	0.451568854	0.301328939	0.303624568	0.497098659	0.192105999
LOC10537194	0	0	0.414503503	0.079163273	0.081785733	0.321749395	0.161092192	0.613134537	0.255712913	0	0	0.081512225
LOC10537210	0	0	0	0.020165061	0	0	0	0	0	0	0	0
LOC10537220	0	0	0	0	0	0	0	0	0	0	0	0
LOC10537226	0.49234825	0.36154406	0.303979771	0.165871654	0.342733045	0.231744203	0.253153646	0.040147105	1.071596012	0.584869894	0.751314497	0.555078677
LOC10537231	0	0	0	0	0	0	0	0	0	0	0	0
LOC10537231	0	0	0	0	0	0	0	0	0	0	0	0
LOC10537241	0.105336824	0.127402562	0.148653263	0	0.07332698	0.018029513	0.10832333	0.017178769	0.152843738	0.134757136	0.151286665	0.05481132
LOC10537247	0	0	0	0	0	0	0	0	0	0	0	0
LOC10537258	0	0	0	0	0	0	0	0	0	0	0	0
LOC10537270	0.578597429	0.333238481	0.374241693	0.747227526	0.369208354	0.495165399	0.264445247	0.314533622	1.853994997	1.973861985	1.073364883	1.505346743
LOC10537271	0	0	0	0	0	0	0	0	0	0	0	0
LOC10537279	0	0	0	0	0	0	0	0	0.011192327	0.011277594	0	0
LOC10537282	0.294041316	0.101610422	0.207478256	0	0.204687791	0.201313146	0.201585311	0.095906973	0.586649206	0.322428275	0.580672805	0.35700573
LOC10537283	0.233447119	0.363020315	0.370625475	0.707832997	0.304700643	0.539418793	0.300082255	0.45685838	0.317561047	0.25598427	0.188595565	0.182208998
LOC10537288	0	0.011848794	0.012097022	0	0	0	0	0	0.012438033	0.037598369	0.024622644	0
LOC10537297	0	0	0	0	0	0	0	0	0	0	0	0
LOC10537305	0	0	0	0	0	0	0	0	0	0	0	0
LOC10537309	0	0	0	0	0	0	0	0	0.395663899	0.558149487	0.35246982	0.718904782
LOC10537310	0.280258129	0.290542301	0.211877907	0.242790931	0.585279152	0.328931301	0.288203999	0.235057716	0.26142079	0.614628899	0.215631338	0.541656016
LOC10537313	6.879202276	6.395631787	5.622726817	3.488766502	7.157553966	7.341243813	5.362325186	4.479571245	6.926818397	9.073466119	8.095651967	5.452122089
LOC10537313	0	0.033185081	0	0.064705733	0	0	0	0	0	0	0	0
LOC10537315	0	0	0	0	0	0	0	0	0.207233468	0.149151604	0.087909561	0.169865214
LOC10537324	0	0	0	0	0	0	0	0	0	0	0	0
LOC10537324	0.016878783	0	0	0.034118675	0	0.017333895	0.05207199	0	0.018368337	0.018508273	0.054543636	0.017565527
LOC10537328	0	0.029484208	0	0	0	0	0	0	0.061900906	0.031186244	0.03063515	0.029597727
LOC10537329	0	0	0	0	0	0	0	0	0	0	0	0
LOC10537331	0.649560126	0.18855087	0.385001902	0.236343079	0.217042197	0.293512817	0.240471517	0.076271746	1.046188079	0.484343005	0.923580985	0.675988639
LOC10537331	0	0	0	0	0	0	0	0	0	0.140821896	0.046111141	0
LOC10537334	0	0	0	0	0	0	0	0	0.074124376	0.112033622	0.036684623	0.070884682
LOC10537338	0	0	0	0	0	0	0	0	0	0	0	0
LOC10537339	0.378735404	0.241620451	0.092505871	0.058890275	0.091261719	0.089757106	0.32955433	0.085521811	0.031704525	0	0	0
LOC10537339	0	0	0	0	0	0	0	0	0	0	0	0
LOC10537375	0	0	0	0	0	0	0	0	0	0	0	0
LOC10537375	0.035032266	0.163430044	0.037078634	0.070814022	0.036579947	0.035976861	0.09006375	0.137117001	0.152495461	0.288107297	0.113206452	0.109372849
LOC10537378	0.809047053	0.624940043	0.805935584	0.913901713	0.314725582	0.505033672	0.668850791	1.040019516	2.261536461	1.826491541	1.794215473	2.922112351
LOC10537392	0.010108879	0	0.010699377	0.020434029	0.010555476	0	0.02079097	0.019783178	0	0.022169594	0.010888917	0
LOC10537394	0	0	0.026991839	0	0	0	0	0	0.055505457	0	0.082410005	0.10615905
LOC10537398	0	0	0	0	0	0	0	0	0	0	0	0
LOC10537398	0	0	0	0	0	0	0	0	0.181014705	0	0.089585323	0.129827432
LOC10537401	0.820801003	0.557980712	0.46997794	0.258394149	0.337205093	0.221097109	0.442792041	0.276496998	3.558303692	2.19846256	1.956696673	4.074938625
LOC10537408	0.078098056	0.080963892	0.061995045	0.01973337	0.142709576	0.040101929	0.040156145	0.114629017	0.106237767	0.021409425	0.105155486	0.101594519
LOC10537410	0	0	0	0	0	0	0	0	0	0	0	0
LOC10537429	0	0	0	0	0	0	0	0	0	0	0	0
LOC10537430	0	0	0	0	0	0	0	0	0.010051194	0	0	0.01922379
LOC10537431	0	0	0	0	0	0	0	0	0	0	0	0
LOC10537437	0	0	0	0	0	0	0	0	0	0	0	0

LOC1053748	0.190814046	0.494540086	0.100980109	0.353567881	0.199243967	0.293938609	0.294335999	0.15559376	0.207653394	0.278980493	0.239794282	0.463347863
LOC1053751	0	0	0	0	0	0	0	0	0	0	0	0
LOC1053751	0	0	0	0	0	0	0	0	0	0	0	0
LOC1053751	0	0	0	0	0	0	0	0	0	0.03535313	0	0
LOC1053751	0.016480723	0	0	0	0	0	0	0	0	0.018071784	0	0
LOC1053751	0	0	0	0	0	0	0	0	0	0	0	0
LOC1053753	0	0.042910863	0	0	0	0	0	0	0.045044813	0	0	0
LOC1053756	0.04606983	0	0.048760943	0.046562644	0	0	0.047376	0	0.150406482	0.101034888	0.099249493	0.143832788
LOC1053757	0	0	0.097857013	0.093445307	0	0	0	0	0.100615562	0	0.099590556	0.096218039
LOC1053758	0	0	0	0	0	0	0	0	0	0	0	0
LOC1053758	0	0	0	0	0	0	0	0	0.639796943	0.54288096	0.666609588	0.483026729
LOC1053758	0	0	0	0	0	0	0	0	0.626770336	0.360883024	0.709011666	0.34250091
LOC1053759	0.119932156	0.372999328	0.169250464	0.363645258	0.166974142	0.041055318	0.246664939	0.195590373	0.043505392	0.131510493	0.258373122	0.12481181
LOC1053759	0	0	0	0	0	0	0	0	0	0	0	0
LOC1053762	0	0	0.007499708	0	0	0	0	0	0.015422243	0.054389073	0.05342796	0.014748196
LOC1053763	0	0	0	0	0	0	0	0	0	0	0	0
LOC1053763	0	0	0	0	0	0	0	0	0	0	0	0
LOC1053763	0	0	0	0	0	0	0	0	0	0	0	0
LOC1053765	0	0	0	0	0	0	0	0	0	0	0	0
LOC1053765	0	0	0	0	0	0	0	0	0.283322354	0.240404887	0.103318545	0.370759198
LOC1053766	0.056463338	0.17560584	0.059761575	0.171202	0.235831264	0.231943163	0.232256738	0.165748987	0.12289246	0.061914349	0.121640511	0.117521299
LOC1053766	0.189070839	0.176407974	0.240138221	0.171984019	0.197423748	0.155335091	0.155545096	0.222008131	0.16460508	0.207323873	0.224026262	0.216439876
LOC1053767	0.10981543	0.056922573	0.058115083	0.05549507	0.057333468	0	0.056464457	0	0	0	0	0
LOC1053767	0	0	0	0	0	0	0	0	0.033673523	0	0	0.032201782
LOC1053767	0	0	0	0	0	0	0	0	0	0	0	0
LOC1053767	0	0	0	0	0	0	0	0	0	0	0	0
LOC1053767	0	0	0	0	0	0	0	0	0	0	0	0
LOC1053768	0.059405565	0.036951284	0.025150268	0.012008207	0.012406005	0	0	0.011625729	0.077577731	0.013028124	0.025595806	0
LOC1053769	0	0	0	0	0	0	0	0	0.048839247	0	0	0
LOC1053769	0	0	0	0	0	0	0	0	0	0	0	0
LOC1053770	0.430285249	0.371728932	0.303613228	0.338246273	0.54913797	0.540084446	0.417902201	0.327472677	0.10405732	0.078637549	0	0.049754686
LOC1053770	0.025417837	0.026350553	0.026902589	0.102758939	0.053081529	0.052206385	0	0.124357412	0.027660962	0.111486773	0	0.105808028
LOC1053773	0.18044789	0.112241694	0.190988536	0.182378164	0.113051909	0.074125364	0.111338366	0.141255341	0.15709794	0.158294767	0.116623146	0.037557947
LOC1053773	0.26334206	0.18200366	0.650358025	0.088719688	0.183317451	0.180295134	0.180538884	0	0.573164017	0.866295871	0.756433327	1.187578605
LOC1053776	0	0	0	0	0	0	0	0	0.640564343	0.466154279	0.686875248	0.493457328
LOC1053776	0	0	0	0	0	0	0	0	0	0	0	0
LOC1053776	0	0.045352944	0	0	0	0	0.044987941	0	0.119020848	0.023985518	0.117808341	0.02276378
LOC1053777	0	0	0	0	0	0	0	0	0	0	0	0
LOC1053777	0	0	0	0	0	0	0	0	0	0	0	0
LOC1053777	0	0	0	0	0	0	0	0	0	0	0	0
LOC1053777	0.021285428	0.066199512	0.04505758	0.150591843	0.133354744	0.065578076	0	0.104139494	0	0.046680676	0.068783667	0.044302926
LOC1053778	0.105577948	0.164178252	0.10243306	0.133384161	0.082681685	0.072283137	0.063333253	0.103308558	0.239365014	0.366606642	0.274834762	0.366245251
LOC1053781	0	0	0	0	0	0	0	0	0.044666863	0	0	0
LOC1053781	0.082277616	0.085296822	0.029027921	0	0.057275024	0.056330743	0.084610348	0	0	0.030073585	0.029542153	0
LOC1053781	0	0	0	0	0.018284022	0	0	0	0.038111459	0	0	0
LOC1053781	0.083296534	0.037008484	0.0881622	0.108241158	0.049700839	0.085542506	0.036710638	0.046574903	0.012949637	0	0.012817714	0.012383657
LOC1053781	0	0	0	0	0	0	0	0	0	0	0	0
LOC1053782	0	0.073886254	0.037717074	0.108050004	0.0372098	0.03659633	0.073291613	0	0	0	0.076770468	0.074170727
LOC1053782	0	0	0	0.033969499	0	0	0	0	0	0	0.036203438	0
LOC1053784	0	0	0	0	0.03201527	0	0	0	0.033366528	0.201724357	0.132106447	0
LOC1053785	0	0	0	0	0	0	0	0	0	0.115694852	0	0.036600587
LOC1053785	0	0.018459339	0.018846056	0	0	0	0.018310777	0	0.038754637	0.039049884	0.019179915	0.01853041
LOC1053786	0	0	0	0	0	0	0	0	0	0	0	0
LOC1053788	0	0	0	0	0	0	0	0	0.083894351	0	0.041519845	0.080227649
LOC1053789	0.04593219	0	0.048615264	0.046423533	0	0.023585343	0.047234458	0.089889762	0.024992854	0	0.049476486	0
LOC1053789	0.046790922	0	0	0	0	0.048052573	0.144352612	0.137355465	0.356441565	0.256540758	0.100802963	0.146084084
LOC1053789	0	0	0	0	0	0	0	0	0	0.027469448	0	0
LOC1053789	0.009944476	0.020618785	0.031576112	0	0.010383811	0.030637844	0.030679265	0	0.021644153	0.010904523	0.010711828	0.04139634
LOC1053790	0.921479636	0.762558972	0.923974821	1.062050761	0.751182977	0.722196169	0.864482118	0.964947763	0.246301817	0.540674003	0.208965133	0.227124871
LOC1053791	0	0	0.018254097	0	0.018008589	0.017711685	0	0.016875939	0.056306016	0.018911658	0.037154938	0
LOC1053791	0	0	0	0	0	0	0	0.03208588	0.642320918	0.179781762	0.28256772	0.375373484
LOC1053792	0.444467552	0.340574634	0.337482813	0.341799408	0.625530888	0.506066329	0.407387661	0.236366171	0.357511356	0.519162209	0.228974229	0.321774962
LOC1053793	0	0	0	0	0.040306168	0	0	0	0	0	0	0
LOC1053794	0	0	0	0	0	0	0	0	0	0	0	0
LOC1053794	0	0	0	0	0	0	0	0	0	0	0	0
LOC1053794	0	0	0	0	0	0	0	0	0	0	0	0
LOC1053797	0.309553165	0.238977266	0.237012799	0.292894421	0.343860457	0.243497737	0.162551289	0.077336002	0.938939006	1.89940642	0.759106768	0.699129459
LOC1079839	0.100766968	0.470090913	0.106653149	0.050922443	0.473484258	0.879614041	0.31087173	0.098600989	0.383808801	1.160198372	0.596983839	0.681634536
LOC1079839	0	0	0	0	0	0	0	0	0	0	0	0
LOC1079839	0	0	0	0	0	0	0	0	0	0	0	0
LOC1079839	0	0	0	0	0	0	0	0	0	0	0	0
LOC1079839	0	0	0	0	0	0	0	0	0	0	0	0
LOC1079839	0.208969168	0.469380959	0.368626416	0.422409076	0.29093488	0.286138297	0.322340784	0.545273044	2.728928484	1.833145611	1.650689295	3.117090809
LOC1079839	0	0	0	0	0	0	0	0	0	0	0	0
LOC1079839	5.301773751	3.783184197	3.570935744	2.818425244	2.696103581	2.863785761	3.186286058	2.358096598	6.369100256	5.511605025	4.857954689	9.637686364
LOC1079840	0	0	0	0	0	0	0	0	0	0	0	0
LOC1079840	0	0.084012231	0.085772261	0.163810749	0.253856018	0.416117911	0.166672192	0.237889736	0.352760585	0.08886201	0	0
LOC1079840	1.550556333	1.716434514	1.821932686	1.36792976	1.605337104	1.889246447	1.689107689	1.375790801	0.714997034	0.619581953	0.438782128	0.615372515
LOC1079840	0.208295074	0.215938536	0.146974919	0.245610439	0.181247738	0.106955726	0.21420065	0.169848156	0.56669281	0.342606045	0.29915718	0.325154896
LOC1079840	0.039420924	0	0	0	0	0	0	0	0	0	0	0
LOC1079841	0.046608541	0.056372001	0.024665561	0.031404746	0.064890196	0.047865274	0.007988331	0.030404463	0.033814498	0	0.025102513	0.024252446
LOC1079841	2.346560299	2.371851454	2.219745905	2.223432894	2.036752308	2.470077734	1.945553736	1.994755257	2.505763521	3.119882427	2.496034118	4.120932866
LOC1079841	0	0	0	0	0	0	0	0	0.705521169	1.086091228	0.814722742	0.937063234
LOC1079841	0.092297703	0.0956846	0.488445809	0	0	0.189572757	0.284743574	0	0.301328939	0.202416379	0.298259196	0.288158999
LOC1079841	0	0	0	0	0	0	0	0	0	0	0	0
LOC1079841	0	0	0	0	0.072452352	0.106886767	0.071354182	0	0.037755163	0.038042795	0.074741075	0.036105028
LOC1079841	4.136264508	3.122171884	3.833166294	4.469486049	6.309321702	5.481023166	4.547558961	4.196567082	2.987031179	4.159359014	3.675219609	2.301052875
LOC1079841	0.529942645	0.71843187	0.60404465	0.906419477	0.553354835	0.544231789	0.712649889	0.717994477	0.22181158	0.223501418	0.219551908	0.169693633
LOC1079841	0.126461953	0.065551259	0.080309445	0.076688839	0.118843992	0.077923091	0.104037918	0.074246197	0.123860003	0.124803612	0.149842242	0.157928739
LOC1079841	0	0	0	0	0	0	0	0	0	0	0	0
LOC1079842	0	0	0.01893377	0	0.018679122	0.018371163	0.036792	0	0.058402517	0	0	0
LOC1079842	0	0	0	0	0	0	0	0	0	0	0	0
LOC1079842	0	0	0	0	0	0	0	0	0.072833653	0.146777051	0	0
LOC1079842	0.019595616	0.020314684	0.04148054	0	0.0818453	0.020123983	0	0.038348819	0.085299719	0.042974781	0.084430741	0.101964492
LOC1079842	0	0	0	0	0	0	0	0	0	0	0	0
LOC1079842	0.72750485	0.406519633	0.469646101	0.130370047	0.253215029	0.206650483	0.419165621	0.070681848	1.145448695	1.233383221	1.078202178	1.229623917
LOC1079843	0.118458032	0.122804891	0.156722018	0.179587788	0.092768517	0.121652083	0.213178963	0.057955892	0.096683969	0.162367568	0.127598687	0.123277708
LOC1079843	0	0	0	0	0	0	0	0	0	0	0	0
LOC1079843	0	0	0	0	0	0	0	0	0	0	0	0
LOC1079843	0	0	0	0	0	0	0	0	0	0	0	0
LOC1079843	0	0	0	0	0	0	0	0	0.024819832	0	0	0
LOC1079844	0	0	0	0	0	0	0	0	0	0	0	0
LOC1079845	0.024677625	0	0	0.012470802	0	0.012671511	0	0	0	0	0	0
LOC1079845	0.080049927	0.06638991	0.101671136	0.12944997	0.083586431	0.065766687	0.1317112	0.062663408	0	0	0	0
LOC1079845	0	0	0	0	0.035426733	0.03484266	0	0	0	0.223219575	0	0.105924777
LOC1079845	0	0	0	0	0	0	0	0	0	0	0	0
LOC1079846	0.233110011	0.040277344	0.123363425	0.078534539	0.081136171	0.159596992	0.119859569	0.076033109	0.54964429	0.724241418	0.251097633	0.525621433
LOC1079846	0.861483117	0.377848115	0.385763913	0.535814469	0.622760083	0.306246384	0.749614343	0.616013324	0.468754521	0.581323885	0.820886197	0.241374564
LOC1079847	0	0	0	0	0	0	0	0	0	0	0	0
LOC1079847	0	0	0	0	0	0	0	0	0.034680638	0.104834541	0.034327334	0
LOC1079847	0.018762051	0.008335942	0.017021154	0.002708964	0.008396115	0.022020506	0.011025138	0.010490721	0.023334631	0.038207654	0.03464537	0.0306828
LOC1079848	0.174611879	0.181019324	0.369623243	0.2353063	0.303876683	0.239093385	0.389052019	0.199335045	0.411713003	0.446761082	0.313475953	0.938867422
LOC1079848	0.713654653	0.59187397	0.868643216	0.46883766	0.819701306	0.952766527	0.770582577	0.558651759	1.70859636	1.838995752	1.422137291	1.448247384
LOC1079848	0.163741532	0.275843888	0.086653148	0.144806459	0.170975424	0.210195734	0.084191963	0.08011096	0.155917763	0.134661942	0.352752856	0.340807296
LOC1079848	0.066678514	0.155531938	0.088216824	0.016847946	0.052218214	0.034238202	0.034284491	0.097867897	0.108844344	0.146231411	0.053867754	0.277565793
LOC1079848	0.189675784	0.181510157	0.154427281	0.250690853	0.182820386	0.104886988	0.150041128	0.071384122	0.222292733	0.159990169	0.235744456	0.197393082
LOC1079848	0	0.051699835	0.10556586	0	0	0.051214512	0	0.048797895	0	0	0	0.051898887
LOC1079848	1.304474203	0.760692569	0.690336743	1.071223018	1.10670967	0.921007643	1.006093961	0.797771641	4.702405491	3.218420419	3.95193434	2.715098125
LOC1079848	0	0	0	0	0	0	0	0	0	0	0	0
LOC1079848	0.07242202	0.018769893	0.019163116	0.018299182	0	0.055781082	0.018618832	0.053148986	0	0.019853423	0	0.01884216
LOC1079848	0	0	0	0	0	0	0	0	0.015674052	0.015793462	0.015514375	0.029977997
LOC1079848	0.050336353	0.052183463	0.017758897	0.050874807	0.035040099	0.0172312	0.034508992	0	0.018259513	0.11039172	0.036146993	0.017461459
LOC1079849	0	0	0	0	0	0	0	0	0	0	0	0
LOC1079849	0.255883283	0.202445192	0.16392366	0.142921855	0.13359394	0.096814719	0.193891215	0.158136689	0.798754852	0.745769217	0.790617657	0.798883052
LOC1079850	0.005429277	0.016885518	0.011492843	0.010974709	0.01133827	0.005575669	0	0	0.035450463	0.017860269	0.011696439	0.005650176
LOC1079850	0	0	0	0	0	0	0	0	0	0	0	0
LOC1079850	0.048962294	0	0.103644734	0.098972099	0.051125385	0.100564984	0	0.047909853	0	0	0.105480807	0
LOC1079851	0	0	0	0	0	0	0	0	0	0	0	0
LOC1079851	0.056139344	0.058199397	0.133691975	0.056739873	0.058619508	0.115306121	0.072163756	0.123598423	0.397108681	0.323185149	0.408181012	0.350540838
LOC1079851	0	0.029084526	0	0.056710291	0	0	0	0	0	0	0.030219866	0
LOC1079851	0.226236834	0.180414365	0.128935792	0.070355975	0.10903001	0.071488303	0.125273666	0.034057522	0.378772685	0.152663323	0.281185496	0.452772468
LOC1079852	3.263143589	3.583849307	2.650159442	2.416392961	2.344630744	2.886616569	2.325705044	1.612308222	1.037207195	1.895367174	1.131044953	1.344915013
LOC1079853	0.079658861	0.041290986	0.021078009	0.120766474	0.124767132	0.081806748	0.081917347	0.1169199	0.02167219	0.021837296	0	0.062174943
LOC1079853	0	0	0	0	0	0	0	0	0	0	0	0
LOC1079853	0	0	0	0	0	0	0.044589176	0	0	0	0	0
LOC1079853	0	0	0	0	0	0	0	0	0	0	0	0
LOC1079853	0.593487804	0.732459581	0.628155676	0.714090974	0.796766577	0.725583752	0.523126587	0.746653921	2.706474066	1.766412467	1.339451135	3.117585755
LOC1079854	0	0.010450379	0	0.010188304	0	0	0	0	0	0.022107296	0.010858318	0

LOC1079854	0.116850295	0.181707237	0.061837982	0.47240103	0.122012592	0	0.240326462	0	0.190743508	0.448458871	0.377600675	0.486418231
LOC1079854	0	0.016159943	0	0	0	0.016008244	0	0	0	0	0	0
LOC1079854	0	0	0	0	0	0	0	0	0	0	0	0
LOC1079854	0.020328508	0	0.021515973	0	0.021226596	0	0	0.019891548	0.0221225	0.022291037	0	0
LOC1079855	0	0.020565575	0.010498209	0.010024916	0.020714027	0.01018626	0.020400062	0	0.010794149	0.010876382	0.02136837	0.020644755
LOC1079855	2.102508966	2.661214347	1.474924368	2.124999773	2.348562233	2.485590821	1.508455281	1.865937487	2.793556107	3.913965768	2.317414776	4.019911866
LOC1079855	0.033113576	0	0	0	0	0	0	0	0	0	0	0
LOC1079855	0	0	0	0	0	0	0	0	0	0	0	0
LOC1079855	0	0	0	0	0	0	0	0	0	0	0	0
LOC1079856	0	0	0.0237898	0	0	0	0	0.021993704	0.073381275	0	0.024211238	0
LOC1079856	0.967797857	0.777566445	0.563381829	0.415713969	0.404221573	0.397557256	0.422975654	0.071024634	0.605593486	0.795922316	0.36486684	0.25179361
LOC1079856	0.498341544	0.234831074	0.191800568	0.263283283	0.272005129	0.232626641	0.256235254	0.221649877	0.271160104	0.136612948	0.280597597	0.188588167
LOC1079856	0.007709106	0	0	0	0	0	0	0	0	0	0.008303969	0
LOC1079857	0.059034077	0.091800529	0.03124124	0.044749179	0.277389571	0.166721076	0.151769522	0.086647715	6.472566538	3.220480114	3.14767343	15.37435484
LOC1079857	0	0	0	0	0	0	0	0	0	0	0	0
LOC1079857	0.097658005	0.1518624	0.258406449	0.592215992	0.101972411	0.200582427	0.050213401	0.238897134	0.212552657	0.321257937	0.262984136	0.203262791
LOC1079857	0.122449064	0.08462825	0.014400198	0.027501982	0.085239138	0.111778424	0.1958767	0.173069044	0.148061333	0.074594658	0.087931788	0
LOC1079857	0	0	0	0	0	0	0	0	0	0	0	0
LOC1079857	0	0	0	0	0	0	0	0	0	0	0	0
LOC1079857	0	0	0	0	0	0	0	0	0	0	0	0
LOC1079857	0	0	0	0	0	0	0	0	0.075074689	0	0.074309876	0
LOC1079857	0	0	0	0	0	0	0	0	0	0	0	0
LOC1079858	1.540082835	0.967634375	1.506556661	1.532973096	0.998984711	0.910623342	1.439770196	1.050318605	2.361629621	1.151429682	1.608650925	3.108351713
LOC1079858	0	0	0	0	0	0	0	0	0	0	0	0
LOC1079858	0	0	0	0	0	0	0	0	0	0	0	0
LOC1079858	0	0	0	0	0	0	0	0	0	0	0	0
LOC1079858	0.074030449	0.186385627	0.044774199	0.021377818	0.011043003	0.076026574	0	0.093136076	0.138109097	0.023193543	0.011391844	0.088048583
LOC1079858	0	0	0.057066915	0	0	0.0276856	0	0	0	0	0	0.028055561
LOC1079859	1.253627762	0.69980081	0.56136254	1.072109058	0.453119344	1.188396958	0.743752256	0.802060736	0.524715565	0.15861391	0.415496084	0.10035645
LOC1079859	0	0	0	0	0	0	0	0	0	0	0	0.051850832
LOC1079859	2.182702825	1.884086934	1.75923391	1.781455794	2.126553988	2.832426763	1.624742709	1.483432808	0.427359989	0.63090217	0.383656898	0.646287359
LOC1079859	0.020085689	0	0.063776911	0.040601096	0.020973049	0.061881813	0.020655158	0.058961846	0.043716504	0	0.064906723	0.020902911
LOC1079859	2.618914677	2.320392159	2.385119308	1.708192902	1.701184905	1.42294899	2.270401625	1.445199221	3.380272903	2.738176965	2.000941666	3.581140673
LOC1079860	0	0	0	0	0	0	0	0	0	0	0	0
LOC1079860	0	0	0	0	0	0	0	0	0	0	0	0.074072618
LOC1079860	3.134954464	1.869858828	2.499922563	1.866370511	1.614305467	2.513843686	2.782215145	1.848945608	1.40203329	1.459804952	1.989108745	2.189901072
LOC1079861	0	0	0.02224718	0.021244206	0	0	0	0.02056755	0	0.023048584	0.02264129	0.04374914
LOC1079861	0.230777245	0.172788559	0.298537333	0.090706738	0.267747432	0.184333195	0.210951319	0.112908352	0.669715592	0.492054587	0.276205402	0.186796422
LOC1079861	0.075048202	0	0	0	0	0	0	0	0.040835605	0	0	0
LOC1079861	0	0	0	0	0	0	0	0	0.017212892	0.052032076	0	0
LOC1079862	0.172549498	0.134160947	0.155234453	0.200554574	0.198189766	0.141761638	0.097592889	0.151956503	0.403720547	0.614924536	0.260209669	0.565645443
LOC1079862	0	0	0	0	0	0	0	0	0	0.032671304	0	0.062014284
LOC1079862	0	0	0	0	0	0	0	0	0	0	0	0
LOC1079862	0.374979518	0.680294167	0.099220873	0.852729124	0.587318453	1.155270911	0.771221852	0.366919361	1.326232303	1.644721236	1.009785778	1.170708687
LOC1079862	0	0	0	0	0	0	0	0	0	0	0	0
LOC1079863	0	0	0	0	0.022252197	0	0	0	0.023191389	0	0	0.066533345
LOC1079863	0	0	0	0	0.013464366	0	0	0	0	0	0	0
LOC1079863	0	0	0	0	0	0	0	0	0	0	0	0
LOC1079863	0	0.015901973	0.032470229	0.046509551	0	0	0.015773993	0	0.016692776	0.050459841	0	0.063852792
LOC1079864	0	0	0	0	0	0	0	0	0	0	0	0
LOC1079865	0.122017145	0	0.04304821	0.041107459	0	0.083538108	0.041825524	0	0.088523442	0	0.131432435	0.08465442
LOC1079865	0.169212456	0.087710883	0.089548398	0	0.176688046	0	0.087004981	0	0	0.092774174	0	0.088048583
LOC1079865	0	0	0	0.005387871	0	0	0	0	0	0	0	0.005547741
LOC1079865	1.698436641	1.740752722	1.756793111	1.131398773	1.571940564	1.60548678	1.131314408	1.265332209	1.953342147	1.904732314	1.018695653	1.807711942
LOC1079866	0	0	0	0	0	0	0	0	0	0	0	0
LOC1079867	0.472013692	0.1223336	0.374689351	0.119265721	0.985733309	0.363555648	0.485396209	0	0.12841723	0	0.127108999	0
LOC1079867	0	0	0	0	0	0	0	0	0	0	0	0
LOC1079867	0.510902416	0.222111357	0.505859313	0.416425487	0.051626461	0.050775306	0.271167745	0.258023538	0.233156919	0.036143568	0.053257308	0.411630497
LOC1079867	0.234158228	0.194200598	0.198269038	0.473326097	0.195602432	0.336660757	0.240797075	0.091650006	0.560609946	1.437878574	0.403562776	0.82853027
LOC1079867	0	0.007719917	0	0	0	0	0	0	0.056726792	0.081655652	0.040106355	0.00774964
LOC1079867	0.123303302	0.102262368	0.182708281	0.17447121	0.077250411	0.227930397	0.050719677	0.217175231	0.483065365	0.243372763	0.18594497	0.128320116
LOC1079867	0.018358772	0.076129815	0.07772471	0	0.019169839	0.07541516	0.056637838	0.017964152	0.099894673	0	0.098877011	0
LOC1079867	0.229220707	0.237632041	0	0.115836355	0	0.235401314	0.058929891	0.224293624	0	0.062837459	0.123454108	0
LOC1079867	0.058744062	0	0	0	0	0.060328012	0	0	0	0	0.126553938	0.061134169
LOC1079867	0	0	0	0	0	0	0	0	0	0	0	0
LOC1079868	0.035319699	0.018307884	0.018691428	0	0.055320117	0.018136022	0.054481622	0	0.019218332	0	0	0
LOC1079868	0	0	0	0	0	0	0	0	0	0	0	0
LOC1079868	0	0	0	0	0	0	0	0	0	0	0	0
LOC1079868	0	0	0	0	0	0	0	0	0	0	0	0
LOC1079868	0	0	0	0	0.020145858	0	0.03968101	0	0	0	0.020782253	0
LOC1079868	0	0	0.021804281	0.041642549	0	0	0	0	0.089675738	0.158128109	0	0.064317265
LOC1079868	0	0	0	0	0	0	0	0	0	0	0	0.021840444
LOC1079868	0	0	0	0	0	0	0	0	0	0	0	0
LOC1079868	0.040306787	0.062678788	0.042661259	0.061106931	0.063131234	0.062090403	0.082899128	0.019720198	0.087727726	0.088396066	0.108542516	0.02097337
LOC1079869	0	0	0	0	0	0	0	0	0	0	0	0
LOC1079869	0	0	0	0	0.027995415	0.027533861	0	0.026234643	0.029177009	0.02939929	0	0.027901793
LOC1079869	0.170476092	0.088365886	0.219098729	0.110764091	0.101718576	0.050020782	0.087654713	0.011915123	0.145766198	0.106819415	0.039349425	0.038016903
LOC1079870	0	0	0	0	0	0	0	0	0	0	0	0
LOC1079870	0	0	0	0	0	0	0	0	0	0	0	0
LOC1079870	0	0	0	0	0	0	0	0	0	0	0	0
LOC1079870	0	0	0.02134662	0.020384246	0	0	0	0	0.043896744	0.066346748	0.086899106	0.104945463
LOC1079870	0	0	0	0	0	0	0	0	0	0	0	0
LOC1079870	0	0	0	0	0	0	0	0	0	0	0	0
LOC1079870	0	0	0	0	0	0	0	0	0	0	0	0
LOC1079870	0	0	0	0	0	0	0	0	0	0	0	0
LOC1079870	0	0	0	0	0	0	0	0	0	0.015785012	0.046518221	0
LOC1079870	0.29394683	0.170292145	0.338568912	0.419422894	0.343042794	0.257479643	0.222265295	0.35530575	0.432789175	0.398165765	0.344566683	0.440865367
LOC1079870	0	0	0	0	0	0	0	0	0	0	0	0
LOC1079871	0	0	0	0	0	0	0	0	0	0	0	0
LOC1079871	0.700011256	0.220864735	0.515409786	0.615216839	0.158899317	0.187535493	0.438174406	0.268029613	1.02675677	0.901084902	0.721242919	1.456984925
LOC1079871	0	0	0	0	0	0	0	0	0	0	0	0
LOC1079871	0.06385944	0.057927437	0.033794856	0.072610372	0.050010502	0.049185989	0.032834991	0.039054241	1.372527016	0.533936632	1.023207645	0.656269546
LOC1079871	0	0	0.052011672	0	0	0	0	0	0	0.107770547	0	0
LOC1079871	0.595319143	0.591974197	0.617234929	0.528011346	0.862655748	1.060541652	0.737135901	0.879728312	0.264431055	0.30641242	0.287910924	0.290804848
LOC1079872	0	0	0	0	0	0	0	0	0	0	0	0
LOC1079872	0	0	0	0	0	0	0	0	0	0	0	0
LOC1079872	0	0	0	0	0	0	0	0	0	0	0	0
LOC1079872	0	0	0	0	0	0	0	0	0	0	0	0
LOC1079872	0	0	0.02958586	0	0	0	0	0	0.091259621	0.030651623	0.030109976	0
LOC1079872	0.37576509	0.155821569	0.079542991	0.227870818	0.156946365	0.077179411	0	0.147075219	0	0	0.1619042	0.156421505
LOC1079872	0.041551784	0.043076542	0	0	0.043387489	0	0	0	0.045218731	0.182252894	0.134274217	0.216211965
LOC1079872	0	0	0	0	0	0	0	0	0	0	0	0
LOC1079872	0.049525597	0	0.131046437	0.025027689	0.025856787	0.127152459	0.152789235	0.048461048	0.053896233	0.054306833	0.08002076	0.128851585
LOC1079872	0.061743615	0.416060575	0	0.312020474	0.193414109	0.063408444	0.095241253	0.181249323	0.369558708	0.236965359	0.266031916	0.353406705
LOC1079872	0	0	0	0	0	0	0	0	0	0	0	0
LOC1079872	0.12627647	0.081818894	0.083532973	0.079767041	0.148337104	0.064840667	0.032464164	0.092671625	0.772989606	0.623102809	0.561084254	0.640644487
LOC1079872	0	0	0	0	0	0	0	0	0	0	0	0
LOC1079872	0	0	0	0	0	0	0	0	0	0	0.011608593	0
LOC1079872	0	0.065474321	0.200537962	0	0.263787787	0.194579079	0.12989476	0.061799212	0.206191046	0.138507921	0.068030169	0.065726407
LOC1079873	0.304307819	0.260609371	0.280072689	0.173839978	0.248675283	0.29892552	0.149664826	0.181249323	0.287967824	0.31917783	0.327789325	0.316689125
LOC1079873	0	0	0	0	0	0	0	0	0	0	0	0
LOC1101174	0	0	0	0	0	0	0	0	0	0	0	0
LOC1101174	0	0	0	0	0	0	0	0	0	0	0	0
LOC1122678	0.031522883	0	0	0	0.032915524	0	0	0.030845301	0	0	0	0
LOC1122678	0.42918892	0.16685181	0.340694604	0.271112505	0.392131197	0.385666211	0.331017953	0.209981768	0.058383108	0	0.115576677	0.167494214
LOC1122678	0	0.015405723	0.015728468	0	0	0	0	0	0	0	0	0
LOC1122678	0.024086643	0	0	0	0	0.024736105	0.024769547	0	0.078636872	0.10564794	0.103781027	0.075199954
LOC1122678	0	0	0	0	0.011110698	0	0	0	0.162115009	0.046671445	0.068770066	0.07751479
LOC1122678	0	0	0	0	0	0	0	0	0	0	0	0
LOC1122678	0	0	0	0	0	0	0	0	0	0	0	0
LOC1122679	0	0	0	0	0	0	0	0	0	0	0	0
LOC1122679	0	0	0	0	0	0	0	0	0.035914294	0	0	0.051516926
LOC1122679	0	0	0	0	0	0	0	0	0	0	0	0
LOC1122679	0	0	0	0	0	0	0	0	0	0	0	0
LOC1122679	0	0	0	0	0	0	0	0	0	0	0	0.005490088
LOC1122679	0	0	0	0	0	0	0	0	0	0	0	0
LOC1122679	0.016996071	0	0	0	0	0	0	0	0	0	0	0
LOC1122679	0	0	0	0	0	0	0	0	0	0	0	0
LOC1122679	0.01691061	0.052593452	0.143187382	0.085457525	0.035315398	0.01736658	0.034780118	0.082735588	0.073611888	0.037086345	0.054646484	0.052795945
LOC1122679	0	0	0	0	0	0	0	0	0	0	0	0
LOC1122679	0	0	0	0	0	0	0	0	0	0	0	0
LOC1122679	0.151519319	0.052359791	0.181752822	0.020418685	0.0632853	0.041494619	0.031163038	0.019768323	0.252832722	0.354447152	0.293779989	0.147171876
LOC1122679	0.047201369	0.19573376	0.04995858	0.047706288	0.098573331	0.096948173	0.048539621	0	0.154100676	0.103516446	0.152530799	0.098243682
LOC1122679	0.162370431	0.050498601	0.137484083	0.196928794	0.271269999	0.083374259	0.200368743	0.158880291	0.176699629	0.195850367	0.24485934	0.185874438
LOC1122679	0	0	0	0	0	0	0	0	0.199177745	0	0	0.063490815
LOC1122679	0	0	0	0	0	0	0	0	0	0	0	0
LOC1122679	0	0	0	0	0	0	0	0	0	0	0	0
LOC1122679	0	0	0	0	0	0	0	0	0	0	0	0

LOC1122680	0	0	0	0	0	0	0	0	0	0	0	0
LOC1122680	0	0	0	0	0	0	0	0	0	0	0	0
LOC1122680	0	0	0	0	0	0	0	0	0	0	0	0
LOC1122680	0	0	0	0	0	0	0	0	0	0	0	0
LOC1122680	0	0	0	0	0	0	0	0	0	0	0	0
LOC1122680	0	0	0	0	0	0	0	0	0	0	0	0
LOC1122680	0	0	0	0	0	0	0	0	0	0	0	0
LOC1122680	0	0	0	0	0	0	0	0	0.124393536	0.344688326	0.184689443	0.089217576
LOC1122680	0.084940112	0.176114039	0.269705357	0.171697454	0.532155946	0.174460801	0.393067491	0.457128817	0.323526282	0.186280582	0.137241603	0.265188156
LOC1122680	0.074049854	0.07676714	0.26125129	0.04989465	0.077321283	0.076046503	0.101532418	0.096610878	0.053723172	0.027066227	0.026587937	0
LOC1122680	0	0	0	0	0	0	0	0	0	0	0.042776165	0
LOC1122680	0	0	0	0	0	0	0	0	0	0	0	0
LOC1122680	0.023104148	0	0	0	0	0	0.023759196	0	0	0	0	0
LOC1122681	0	0.032245157	0.032920683	0	0.032477918	0.063884923	0.095956939	0	0.101546111	0	0.06700775	0.064738611
LOC1122681	2.116466637	1.595731729	1.96857051	1.1667855	1.607250497	1.31729341	0.527629729	2.008216575	2.512630816	1.828502662	1.381685427	3.070261438
LOC1122681	0	0	0	0	0	0	0	0	1.621470726	0.980294194	1.467384903	1.018967305
LOC1122681	0	0	0	0	0	0	0	0	0	0	0	0
LOC1122681	0	0	0	0	0	0	0	0	0	0	0	0
LOC1122681	1.340451694	1.64488005	1.592477362	1.714224938	2.39943624	1.460876383	2.391199425	1.90053783	3.959455104	3.329682538	3.388711704	2.334473668
LOC1122681	0.680444624	0.592547575	0.432115184	0.385125119	0.625245104	0.279516735	0.363863016	0.266327407	0.533155603	0.268608684	0.410452125	0.198276324
LOC1122681	0	0	0	0	0	0	0	0	0	0	0	0
LOC1122681	0	0	0	0	0	0	0	0	0	0	0	0
LOC1122681	0.057213781	0	0.030277927	0.028912902	0.089612119	0.029378234	0	0.027991987	0.0622629	0.03136862	0.092442909	0.029770813
LOC1122681	0	0	0	0	0	0	0	0	0.216721899	0.043674592	0.085805631	0.041449962
LOC1122681	0	0	0	0	0	0	0	0	0	0	0	0
LOC1122681	0	0	0	0	0	0	0	0	0	0	0	0
LOC1122681	0.29394683	0.340584291	0.23791329	0.297091217	0.090274419	0.248601035	0.231155907	0.135354572	0.28225381	0.094801373	0.242127939	0.305906581
LOC1122681	0	0	0.080441782	0	0	0	0	0.037184271	0.041354701	0.125009268	0	0.039547245
LOC1122681	0.091914818	0.122512712	0.152874718	0.092898043	0.109686283	0.067423693	0.067514846	0.051393781	0.157184194	0.100788341	0.183870705	0.122984404
LOC1122682	0	0	0	0	0	0.0386978	0	0	0.041007183	0	0	0
LOC1122682	0.282041876	0.229736181	0.085290575	0.20361351	0.126215197	0.227579575	0.207170228	0.197128148	1.183880899	0.618540802	0.781213527	1.278896604
LOC1122682	0	0	0	0	0	0	0	0	0.050481256	0	0	0
LOC1122682	0	0	0	0	0.047920511	0	0.023597087	0	0	0.02516178	0.049434289	0.143280765
LOC1122682	0	0	0	0	0	0	0	0	0.130807722	0.043934754	0	0.041696872
LOC1122682	0	0	0	0.024000516	0	0	0.024419756	0	0.232579132	0.703053004	0.076736589	0.4942533
LOC1122682	0.037148471	0	0	0	0.058184465	0.038150127	0.019100852	0	0	0.020367406	0	0
LOC1122682	0	0	0	0	0	0	0	0	0	0	0	0
LOC1122682	1.297227124	1.532654657	1.480388793	1.018119655	3.314453576	3.855207749	1.736443652	1.418260696	0.804436663	1.732384324	1.249006409	0.708942288
LOC1122682	0.127435313	0.165139496	0.269758585	0.225397383	0.066532621	0.327178559	0.294858799	0.654654526	0.416044455	0.524017534	0.17158586	0.331550615
LOC1122682	0	0	0	0	0	0	0	0	0	0	0	0
LOC1122683	0	0	0	0	0	0	0	0	0	0	0	0
LOC1122683	0	0	0	0	0	0	0	0	0	0	0	0
LOC1122683	0.59840893	0.555065888	0.766704109	0.350153266	0.394639499	0.566027524	0.615375001	0.416045966	0.634081215	0.259018325	0.237478446	0.868581106
LOC1122684	0.42236704	0.175146379	0.4470391	0	0.264616006	0.173502225	0.260605186	0.082657658	0.919281901	0.555771187	0.090991685	0.439551796
LOC1122684	0	0.070972165	0.036229505	0	0	0	0.035200488	0	0	0	0	0
LOC1122684	0	0	0	0	0	0	0	0	0	0	0	0
LOC1122684	0.040036876	0.041506043	0.042375581	0.013488385	0.041805654	0.013705471	0.054896	0	0.116187018	0.10243815	0.05750169	0.09722031
LOC1122684	0.108123699	0.074727558	0.114439614	0	0	0.037013033	0.074126146	0.387931823	0.352996861	0.158082718	0.11646692	0.262553444
LOC1122684	0.372706915	0.434681468	0.591717208	0.706300891	0.43781921	0.382756423	0.622820071	0.455869509	1.064695583	1.226064922	0.702566105	1.066645692
LOC1122684	0	0	0	0	0	0	0	0	0	0	0	0
LOC1122684	0	0	0	0	0	0	0	0	0	0	0	0
LOC1122684	0	0	0	0	0	0	0	0	0.628479983	0.699540193	0.499108643	0.461241398
LOC149373	0	0	0	0	0	0	0	0	0	0	0	0
LOC150051	0	0	0	0	0	0	0	0	0	0	0	0
LOC283710	0	0	0	0	0	0	0	0	0	0.024044162	0	0
LOC284898	0	0	0	0	0	0	0	0	0	0	0	0
LOC285500	0.08569766	0.115495076	0.12698502	0.086614379	0.062638571	0.096809212	0.202692923	0.100626714	0.121238627	0.075176779	0.110772487	0.098102865
LOC339862	12.60549456	7.429919421	12.58571099	1.048108174	13.35489218	8.022824606	12.01495936	0.856878491	0.777432963	0.252695393	0.695043984	0.071947193
LOC388282	0	0	0	0	0	0	0	0	0.017907724	0	0	0
LOC388436	0	0	0	0	0	0	0	0	0	0	0	0
LOC388692	0.031755421	0.016460349	0	0.016047556	0	0	0	0	0	0	0	0
LOC388780	0.044841301	0.023243384	0	0.022660487	0	0	0.02305632	0	0.073197821	0.073755468	0	0.046665749
LOC388813	0	0	0	0.032703048	0	0.03322938	0	0.031661412	0.105637265	0.106442046	0.069707401	0.033673421
LOC389199	0	0	0	0	0	0	0	0	0	0	0	0
LOC389602	0.119887622	0.04142898	0.063445356	0.040390025	0.041728035	0.041040073	0.123286672	0	0.195701565	0.109551382	0.043046197	0.041588488
LOC389895	0	0	0	0	0	0	0	0	0.094069489	0.189572287	0.186222341	0.08995807
LOC390877	0	0	0	0.034951908	0	0.035514434	0	0	0.150535365	0.075841098	0.037250452	0.071978019
LOC390937	0.301695576	0.40212823	0.25089331	0.261362444	0.382529266	0.287699624	0.243767259	0.231951226	0.351771668	0.212670953	0.580313413	0.313970598
LOC400499	0.142343158	0.143578207	0.057005715	0.08554184	0.060256093	0.102721951	0.067255155	0.097874894	0.075359164	0.101244369	0.161614817	0.056050946
LOC400927-	0.502725293	0.353653091	0.418071801	0.453663402	0.431196653	0.368771829	0.387733909	0.175685447	0.742480403	0.708761253	0.444817879	0.429754646
LOC401040	0	0	0	0	0	0	0	0	0	0	0	0
LOC401478	0	0	0	0	0	0	0	0	0	0	0	0
LOC402096	0.083555219	0	0.088435996	0.08444902	0.174493163	0.085808165	0	0.081759205	0	0.091621699	0	0.260864436
LOC403312	0	0	0	0	0	0	0	0	0	0	0	0
LOC440292	0	0	0	0.02526018	0.01304849	0.025666725	0	0.02445561	0.054396895	0.027405655	0.040382051	0.013004853
LOC441155	0.435453094	0.567514764	0.513562652	0.440111191	0.53263786	0.447194462	0.537358856	0.474789411	0.609276663	0.395636267	0.428849599	0.323693057
LOC441239	0	0	0	0	0	0	0	0	0.191054672	0.096255097	0.252144442	0.274056601
LOC643802	0.035960945	0.074561089	0.012687187	0.133267288	0.037549654	0	0.049307345	0.023458645	0.026089667	0	0.025823882	0
LOC644090	0	0	0	0	0	0	0	0	0	0	0	0
LOC644215	1.251385136	0.540543815	0.551868036	0.737783295	0.980002302	0.749657384	1.286864369	1.020405588	0.79439496	0.800446939	0.561644415	0.542624989
LOC644249	0	0	0.065312823	0	0	0	0	0.060381799	0	0.067665567	0.066469844	0
LOC645177	0.111176779	0.02305129	0.16473945	0	0.046435371	0.0456698	0	0	0.31456915	0.219437756	0.550875696	0.023140041
LOC645188	0	0	0	0	0	0	0	0	0	0	0	0
LOC645202	0	0	0	0	0	0	0	0	0	0	0	0
LOC646588	0.153147764	0.10206487	0.046312487	0.110561432	0.022844805	0.112340839	0.12374199	0.042815961	0.142854053	0.071971182	0.188531665	0.091073631
LOC646652	0	0	0	0	0.024302249	0.011950791	0	0	0.012663983	0.025520923	0	0
LOC647264	0	0	0	0	0	0	0	0	0	0	0	0
LOC653513	0.032601976	0.028165264	0.069012761	0.126311106	0.028368574	0.005580174	0.022350871	0.021267466	0.059131836	0.011916465	0.040970607	0.039583186
LOC728026	0.431339165	0.335375481	0.456535321	0.70842404	0.394095782	0.996681617	0.60990666	0.844135123	0.469404866	0.118245239	0.174233578	0.50500009
LOC728392	0.252288631	0.336274009	0.228879228	0.218560624	0.150533955	0.037013033	0.444756875	0.105799588	0.705993722	0.237124076	0.271756146	0.337568714
LOC728485	0.628016095	0.828623558	0.785555604	0.375070128	1.043256208	0.615633756	0.587110535	0.530719171	0.403850049	0.751249329	0.399735887	0.20795347
LOC729159	0	0	0	0	0	0	0	0	0	0	0	0
LOC729254	0	0	0	0	0	0	0	0	0	0	0	0
LOC729800	0	0	0	0	0	0	0	0	0	0	0	0
LOC730098	0.033401341	0.138508061	0.03535244	0.067517279	0	0.171509803	0.034348335	0.065366764	0.436188135	0.988900129	0.899467779	0.625686021
LOC730183	0.832106611	0.766792051	0.684999088	0.654117148	0.579245347	0.189898483	0.095077608	0.633282643	1.106771182	1.520731298	0.796724449	0.769744314
LOC730268	0.665342329	0.883751542	0.682201013	0.819560114	1.411183119	1.153038935	1.069072022	1.363116769	0.972954039	1.299555386	1.052627539	1.125171074
LOC732265	0	0	0	0	0	0	0	0	0	0	0	0
LOC79999	0	0	0	0	0	0	0	0	0	0	0	0
LONP1	46.05193619	52.79509682	51.23727128	53.01668449	57.36775266	63.25997433	49.86389906	60.80493588	27.7536322	31.54585556	41.09481358	24.55132303
LONP2	12.20631233	11.2472715	10.56802848	11.61022515	9.320884625	10.17104372	9.802656973	10.03101127	13.62637719	14.24396246	12.2606276	17.56218281
LONRF1	12.43077312	15.64743247	12.82204949	14.54889861	18.60964031	17.74778569	12.5120921	12.31836025	6.555885222	10.06052047	8.846188481	3.498216365
LONRF2	0.007712421	0.019988577	0.020407332	0.003897461	0.004026573	0.003960188	0.003965542	0.003773322	0.058751297	0.088798327	0.066460317	0.056183502
LONRF3	3.268191886	2.701425937	2.824159519	2.728416309	2.968876249	3.073947236	2.60899758	2.555908029	2.339338106	2.953302212	2.578020265	4.162036379
LOR	0	0	0	0	0	0	0	0	0	0	0.030394181	0
LOX	0	0.030208008	0	0	0	0	0	0.009504139	0.4228033	3.493399781	0.847454512	0.262810717
LOXHD1	1.195016436	1.85830198	1.199194327	0.945729938	0.941743954	1.354593265	0.834722834	0.706010353	0.914014278	1.489634844	1.007924032	0.99139052
LOXL1	0	0	0	0	0	0	0	0	0	0	0	0
LOXL2	0.409574085	0.351396042	0.47834357	1.084848507	0.516151693	0.449625778	0.334044321	0.552786649	3.565752136	2.988935498	3.331656982	1.807838466
LOXL3	0.888913339	1.110027576	0.86599874	0.449211079	0.685590747	0.50830908	0.685586837	0.573281367	2.539319951	3.101412581	2.40464361	3.279830381
LOXL4	0.047809472	0.037172893	0.037951653	0	0.012480408	0.012274647	0.012291241	0.011695453	0.247136136	0.196593873	0.193119848	0.161702729
LPA	0	0.008596721	0.008776819	0.008381132	0	0	0.008527534	0	0	0	0	0
LPAR1	1.07693393	0.972083556	1.257756385	0.806957298	1.027570851	0.896218617	1.498899475	0.735832849	3.788707108	2.382164182	2.980087593	2.714922459
LPAR2	17.81618846	20.51728549	15.35072993	11.13887234	11.10875537	10.59849363	12.57815971	10.22306264	25.67412139	24.47261146	22.8507269	23.31445867
LPAR3	5.37342325	5.622664623	5.541136212	3.667128727	4.4440795	4.95100702	4.325077284	3.243204171	1.256966787	1.294076339	1.20359114	0.90152217
LPAR4	0.329502329	0.50290159	0.32937482	0.185015032	0.344059321	0.413583973	0.254133277	0.098517131	0.747043592	0.712588974	0.650701215	0.323858234
LPAR5	0.497554159	0.382083025	0.624140069	0.055875173	0.115452326	0.24602259	0.303206399	0.090159124	0.802167905	0.626416303	0.813845839	0.441087217
LPAR6	1.879290587	2.071558896	1.800231593	0.661181316	2.471523636	1.53908439	1.981498054	0.756507591	2.096913714	3.364970951	2.139611961	1.621541942
LPCAT1	35.8766286	45.52894909	38.58753156	54.5048399	40.00176362	51.99943931	37.1816021	57.71915327	38.99588673	47.1893356	51.8281287	44.69606048
LPCAT2	8.750446011	11.40815724	8.279302597	11.84949643	7.268091692	9.822784786	9.806848654	10.87901053	16.29366498	17.4354707	13.89367131	17.44421875
LPCAT3	38.56843999	39.84243728	36.78250544	27.8009452	39.7505591	38.5353641	34.00928849	30.07151204	13.61992387	11.05865015	13.92157424	11.22814561
LPCAT4	17.04753823	20.41080838	18.04334957	14.34943953	19.62988737	23.33398372	17.12755196	18.06266637	10.84096013	20.09818582	12.95548284	12.68002277
LPGAT1	10.01339396	9.821912478	10.77559919	11.36374924	12.51085429	10.69193114	11.39538428	9.834009959	10.12807209	10.51517644	9.861383239	11.72817405
LPIN1	4.163874037	4.880007726	3.471632003	3.057056563	5.496311729	5.627570729	4.079950327	3.145466335	6.234149523	7.200558154	7.538759121	5.089570195
LPIN2	4.415343127	4.754970721	3.824325691	5.847782962	4.707982114	4.854283408	4.212199465	4.89192185	11.99130734	9.461193456	8.396478345	13.83359194
LPIN3	0.264108006	0.253518095	0.3313014	0.069204905	0.173636716	0.110500826	0.241418656	0.076572155	9.250522703	7.422473559	6.943603474	9.528262009
LPL	0	0	0	0	0	0	0.014767859	0	4.203942127	10.37733726	6.295813555	6.127448232
LPO	4.168346264	5.688409029	4.139103543	5.928749366	3.149626177	3.658086694	3.086299504	4.746179455	0.115466986	0.083104753	0.179599645	0.094646026
LPP	2.59213987	2.551017187	2.273506682	1.612226659	1.835984324	1.717290653	1.680000675	1.11301036	6.642914962	7.100998058	5.652657305	6.364368703
LPXN	19.26335791	17.26938253	17.71317604	8.976097841	5.551935286	5.828406897	15.47711499	8.519615292	6.481880506	4.715252378	5.257865127	4.213020107
LRAT	0	0	0	0	0	0	0	0	0	0.023663274	0	0
LRBA	5.157438111	5.716362236	4.823542493	6.399289274	5.348961434	6.27005688	4.852000739	4.906160203	6.710490425	7.433959884	6.599970944	6.697783278
LRCH1	6.517327735	6.314792942	5.798854264	6.560131063	6.680132808	6.959686604	5.853218921	5.595554303	5.839163082	5.825249379	4.983716942	5.195988387
LRCH2	0.008677562	0.017991976	0	0	0	0	0	0	0	0	0.009347154	0
LRCH3	5.212855645	4.961698749	4.692289687	5.73517924	5.352206377	4.848036346	4.82324181	5.015579203	5.682368907	4.737159223	5.169455927	6.231667684
LRCH4	25.45810806	24.58224893	22.0172835	23.56242471	24.30825652	22.95119169	21.80232974	27.53051753	36.6079586	31.78140435	30.46750863	46.61960243
LRCOL1	0	0	0.02125897	0.020300548	0	0	0	0	0	0	0	0.020902911
LRFN1	0.931965705	1.203745954	0.565970286	0.617662335	1.036951139	1.051235299	0.926966185	0.747486206	3.52480139	4.422815113	3.242037371	3.895437313

LRFN2	0	0	0	0	0	0	0	0	0	0	0	0
LRFN3	3.722808584	4.901904928	4.710210144	3.243648227	2.926628477	3.142045954	3.938367813	3.747464879	4.540302237	5.701019072	5.024115864	8.082544842
LRFN4	11.35935945	12.12255384	11.31567313	9.653466742	9.768048265	11.06016483	10.77196657	13.74976032	8.597669591	9.632927441	9.970767868	11.14660331
LRFN5	0	0	0	0	0	0	0	0	0	0	0.014042811	0
LRG1	33.9181614	34.78672758	36.25140518	20.2875011	15.4040212	14.43601867	29.28411693	20.97243044	8.783738559	6.397665309	8.238376981	13.6536641
LRGUK	0.020955919	0.038018582	0.013862521	0.047655196	0.041028234	0.018830845	0.016162545	0.038447762	0.279364677	0.114895092	0.152367442	0.264428643
LRIF1	12.04924943	13.1248092	11.26422846	13.72913155	12.72993748	12.6951674	12.72694249	11.44266174	20.93685769	17.87114269	18.87160829	19.01626192
LRIG1	6.129497785	7.721585364	6.634989192	8.785729687	5.711775008	7.134073698	4.822965098	6.82470645	17.64630943	16.96604896	16.51618446	20.62506906
LRIG2	1.986363342	1.787450592	1.904853946	1.9312596	1.92099551	2.163139595	1.891878727	1.661030524	5.546809881	5.247973505	4.810596431	5.401495899
LRIG3	0.012215564	0.012663819	0	0	0	0	0	0	0.757734541	0.964430182	0.697382587	0.55935336
LRIT1	0	0	0	0	0	0	0	0	0	0	0	0
LRIT2	0	0	0	0	0	0	0	0	0	0	0	0
LRIT3	0.014104734	0.043866937	0.074643226	0.042766843	0.014727863	0.043455144	0.072523156	0.110412432	0.046048433	0.015466415	0.121544857	0.044035831
LRMDA	13.10061446	14.38616664	10.73321126	14.07443047	7.44766402	8.37128678	10.02918733	13.6736189	9.6101017	8.885239419	8.257844164	13.07819188
LRMP	22.62904509	17.222917	19.89738053	11.7890502	30.54940335	23.59838887	22.37144909	12.1322477	53.59117708	40.09689528	55.17381541	18.65416682
LRP1	0.046412174	0.051816461	0.045344571	0.018041789	0.044734712	0.062329339	0.051399439	0.024453987	4.063955473	4.854393856	4.226374225	3.641117361
LRP10	7.586666752	7.912585202	7.5123007	7.413000405	5.688085677	5.17846126	6.646819825	7.610489995	18.12537277	15.21396334	15.99851653	23.20102042
LRP11	5.727936878	7.175235032	7.646168353	9.63940467	7.917143581	8.719877887	6.834299138	9.053932404	9.050457335	12.53163529	9.788824057	11.98884648
LRP12	5.524915143	5.91588661	5.957461337	5.361179551	4.292893508	4.501816099	5.828264248	3.946748785	4.71401732	5.546325861	4.847604524	6.100627205
LRP1B	0.002427134	0.002516199	0.002568912	0	0	0	0	0	0.018489301	0.018630159	0.007843262	0.025258863
LRP2	0	0	0	0	0	0	0	0	0.014842724	0.00373895	0.014691516	0.007097003
LRP2BP	0.123322141	0.186444257	0.135964432	0.129834723	0.182424671	0.105539455	0.13738678	0.08044756	0.167756657	0.202841623	0.110698441	0.058822373
LRP3	10.45138946	13.96465991	12.02049084	13.75975278	11.25837536	13.33160408	12.12258554	17.61193328	8.135264139	9.348008012	9.508011233	11.14592029
LRP4	0.015483281	0.016051445	0.032775435	0	0	0	0	0	0.685220349	0.679121899	0.544815553	0.365233562
LRP5	7.687080123	8.610907522	7.725394442	6.247197423	6.782163365	8.596493039	6.821883677	6.943243295	9.240219478	11.509095	10.67872734	9.980572973
LRP5L	1.796989546	1.770057333	1.561725469	1.683058787	1.271094945	1.417906194	1.327697202	1.567573226	1.250190974	1.490855801	1.095544884	1.881071619
LRP6	3.198523826	2.650603795	2.954105457	3.21214815	2.212371815	2.390348279	2.43548065	1.913745809	3.109435136	2.535540554	2.573027829	3.514176046
LRP8	12.48408616	11.28186969	12.45443138	14.62913626	12.97264317	14.1282343	11.41847736	12.04525895	23.7879246	24.57903265	24.91703624	26.89760309
LRPAP1	7.722100989	8.137831724	7.5839742	10.4165974	5.620812999	6.58237239	7.594405159	10.60954507	12.21075273	9.705256335	10.30393612	22.7429089
LRPPRC	86.47907306	77.73348646	88.69414482	68.22286347	94.50981371	90.99766153	88.98920769	66.69951904	58.29392548	66.55970426	71.50586944	44.38793245
LRR1	14.03057318	12.40357548	15.82928306	21.72290115	23.1830917	20.99580747	19.9143183	21.60429609	19.8326246	17.58296542	22.78609095	17.61799167
LRRC1	0.410533464	0.53327959	0.429279168	0.639884326	0.433835011	0.411443804	0.391654373	0.488826708	1.491004431	1.534905592	1.40655297	1.765568738
LRRC10	0	0	0	0	0	0	0	0	0	0	0	0
LRRC10B	0.024249464	0	0.025665967	0.024508864	0	0	0	0	0.052778961	0	0	0
LRRC14	10.64766284	9.320006409	12.03283615	13.07099162	12.10568338	11.88686509	9.996154587	15.13794598	12.65738867	14.22225174	13.15385687	16.3825529
LRRC14B	0.063206219	0.131051186	0.044598889	0.021294115	0.065998589	0.10818414	0	0.041231737	0	0	0	0
LRRC15	0	0	0	0	0	0	0	0	0.019716585	0.019866793	0.03903145	0
LRRC17	0	0.019987145	0.020405869	0.097429539	0.080525688	0.059398558	0.019826287	0.075461022	1.216903945	1.205033801	1.308343723	0.822628037
LRRC18	0	0	0	0	0	0	0	0	0.033040205	0.044389223	0.054506022	0.010532048
LRRC19	0	0	0	0	0	0	0	0	0	0	0	0
LRRC2	0	0	0	0	0	0	0	0	0.011005123	0.011088963	0	0.021048261
LRRC20	3.620393677	3.722977194	4.403565565	4.470620208	5.65526378	6.026778173	3.557904175	6.313780336	5.385580343	4.722270872	4.953319765	7.383468485
LRRC23	1.315344821	1.642532472	1.392179099	1.510699128	1.49831463	1.504312483	1.106703358	1.755097611	1.333826966	1.639010399	1.19143502	1.555524968
LRRC24	1.670161313	1.699967591	1.189194643	1.135581963	1.553698156	1.340970496	1.155418291	1.515405951	1.685367444	1.498418084	1.112132012	1.706512719
LRRC25	3.311357591	4.913293796	2.321921084	3.618705449	1.512721502	2.316688455	2.468799798	3.888216245	0.675672197	0.226939901	0.668788888	0.839983483
LRRC26	0.764212644	0.512636039	1.094330802	0.318041922	0.65715554	0.877150136	0.369825683	0.967722993	2.201438235	1.577393466	1.743209133	2.573048819
LRRC27	1.638842776	1.245181288	1.42946961	0.911814831	1.036780213	1.058062352	1.207711997	0.898442825	0.894640038	0.983406239	0.70152062	1.022202122
LRRC28	1.163998921	0.890861409	0.934329891	1.018537565	1.056357495	1.087077836	1.036329364	1.031960703	1.347486026	1.267805953	1.3632108	1.345501997
LRRC29	0.031689965	0.082132099	0.134164385	0.048043435	0.082724969	0	0.016294219	0.062017583	0.206919636	0.416992045	0.187744034	0.412241599
LRRC3	0.018336875	0.057029261	0.048520005	0.037066055	0.076587901	0.037662606	0.028285143	0.107656354	3.601896541	4.664854268	4.128129516	5.505429312
LRRC30	0	0	0	0	0	0	0	0	0	0	0	0
LRRC31	0	0.042175495	0	0	0	0	0	0	0	0.014870054	0.014607284	0
LRRC32	0	0	0	0	0	0	0	0	0.016343359	0	0.032353728	0.031258107
LRRC34	3.504557559	3.084400216	3.419485173	2.76722364	5.184127961	6.260852497	4.054408501	2.768386666	3.257650664	4.343266361	3.657013867	2.298462266
LRRC36	0	0	0	0	0	0	0	0	0.014171892	0.042839575	0	0
LRRC37A	0.343000871	0.355587409	0.165016751	0.178587573	0.26047579	0.181461811	0.149641173	0.223752062	0.475071817	0.524280707	0.380664077	0.378590199
LRRC37A2	1.398862067	1.248045578	1.337004004	1.585198707	1.133119619	0.922894061	1.203127961	1.119922061	2.05742734	1.868580313	1.634653372	2.796856929
LRRC37A3	3.72849446	3.007379473	3.334097004	2.023592349	2.96404643	2.019606639	2.937421678	2.150698412	1.578467984	0.790367839	1.437779982	1.009406313
LRRC37B	3.202581684	4.041862757	3.013028108	4.487792808	4.410292129	4.067474811	2.688799129	3.497074883	4.074496322	3.613551391	3.949661808	3.815911163
LRRC38	0	0	0	0	0	0	0	0	0	0	0	0
LRRC39	0.359467776	0.343992528	0.20486612	0.447154521	0.606332359	0.340763362	0.454965409	0.351741042	1.624946496	2.092138659	1.250972021	2.100678117
LRRC3B	0.0362965	0.004703552	0.019208358	0.004585596	0.009475008	0.013978194	0.051322668	0.017758155	0.118499002	0.228853391	0.112404653	0.066103253
LRRC3C	0	0	0.041571373	0	0	0	0	0	0.042743253	0.086137773	0.084615626	0.204375543
LRRC4	0	0	0	0.014137034	0	0.014364559	0	0.01368675	5.099302344	3.174916946	3.616014785	9.854758127
LRRC40	12.36980397	11.78190957	12.20278608	14.12721697	15.96887961	14.89874503	15.14436174	12.13967346	15.78786285	17.39076297	17.22121925	13.31043436
LRRC41	19.67663046	20.11483086	19.43504002	16.84316789	14.12293683	16.17699313	19.93417513	17.77125634	42.62755077	39.9500445	40.40410482	46.74806311
LRRC42	5.232250033	6.480550473	5.771059723	6.429362306	5.549668442	6.250031394	5.918654087	6.736556848	15.01416929	17.5442894	16.07740194	18.1695506
LRRC43	0	0	0	0	0	0	0.020019959	0	0.105930277	0.042694916	0.125821354	0.060780281
LRRC45	6.414912476	8.343867148	7.611978545	10.28908595	10.08907713	10.10687395	6.781170147	12.70999973	9.994578492	8.235708674	11.07302447	13.08230476
LRRC46	0.229085169	0.290267426	0.296348436	0.360166679	0.372098004	0.104560943	0.183229033	0.298881334	0.277002162	0.111644985	0.164508147	0.317874544
LRRC47	22.27163093	22.77289862	23.09942867	22.50987341	22.78875443	22.30869721	24.2404814	26.06797169	23.88327705	20.03207422	22.501749	27.89371132
LRRC49	0.203054947	0.294708567	0.2292439	0.656726564	0.353376092	0.208530032	0.13920797	0.490102729	1.104872775	0.697661785	1.035290807	1.098846317
LRRC4B	0.018967064	0.019663067	0	0.009584979	0.009902502	0.019478484	0.009752409	0	1.290056773	1.851036065	1.460790205	1.588971222
LRRC4C	0	0	0	0	0	0	0	0	0	0.007480271	0.003674043	0.003549626
LRRC52	0	0	0	0	0	0	0	0	0	0	0	0
LRRC53	0	0	0.006982074	0	0	0	0	0	0	0	0	0
LRRC55	0.009951833	0	0	0	0	0	0	0.009737919	0	0	0	0
LRRC56	1.144884274	0.837074167	0.969409043	1.035327958	0.629191474	0.816839925	1.003840671	0.955181902	1.062311046	0.647528408	0.856768744	1.166382439
LRRC57	7.174608115	7.395380705	7.788970853	11.78863274	7.042078762	7.494370762	8.094744558	10.26982831	12.71550725	11.17149488	12.78469578	15.2530334
LRRC58	12.33354636	13.82441334	13.60995789	16.95333578	14.35567951	16.06818488	12.36632608	12.70578278	21.25718846	22.34071651	20.75395637	24.83980831
LRRC59	60.6026718	53.90870354	68.89039687	77.03477226	62.79814406	64.28412178	73.33096175	80.05412636	67.6985682	69.36809293	79.97323229	75.42081538
LRRC6	0.265071728	0.382755233	0.310615099	0.143521733	0.266897179	0.243052685	0.369939546	0.240847315	0.422394184	0.394469779	0.448683139	0.394080921
LRRC61	0	0	0	0	0	0.021928753	0	0	0	0.023414434	0	0
LRRC63	0.170492272	0.210957942	0.17463036	0.094495898	0.08614084	0.152497177	0.152703346	0.086104543	0.359106237	0.247258723	0.248813528	0.211770161
LRRC66	0.01584965	0	0	0	0	0	0	0	0.206980585	0.156418076	0.273162668	0.115461647
LRRC69	3.089736157	1.247966928	2.548222877	1.216670438	2.220656471	1.60712743	2.971015725	1.256445717	1.310028122	2.156013679	1.383127902	1.211012727
LRRC7	0	0	0.00358577	0	0	0	0	0	0.036868512	0.0668689	0	0.038782842
LRRC70	0.526371871	0.099215868	0.379854033	0.411092871	0.274813155	0.221140119	0.319856462	0.42141072	0.286412107	0.760838976	0.335038749	0.473089852
LRRC71	0	0	0	0	0	0	0	0	0.036576051	0.0368547	0	0.017488725
LRRC72	0	0	0	0	0	0	0	0	0	0	0	0
LRRC73	0	0.031304221	0	0	0	0	0	0.029547098	0	0.099333964	0.065052417	0.062849494
LRRC74A	0	0	0	0	0	0	0	0	0.015657288	0	0	0
LRRC74B	0	0	0	0	0	0	0	0	0	0	0.012630574	0.012202854
LRRC75A	0.031212042	0.01617869	0.033035256	0	0.03259095	0	0	0	0.815196154	0.479153852	0.386635494	0.649639198
LRRC75B	0.284518498	0.516178258	0.188211439	0.071890507	0.482768263	0.840046627	0.877755472	0.313203143	2.012577242	1.715923643	1.723910552	2.627839932
LRRC8A	4.575915391	4.77497134	4.705440063	5.080266959	4.527146224	4.452508106	4.818916741	5.535709807	8.303199085	7.553965305	8.035256648	8.638460576
LRRC8B	1.605824256	1.450772325	1.586026821	1.748169209	1.784528932	1.649123006	1.884834257	1.195647624	2.371980033	2.380997385	2.401175295	2.130836504
LRRC8C	12.09792933	11.10185284	12.21288219	10.54382553	12.15093685	10.05058469	11.05992109	8.335585697	36.69926757	30.08363559	34.53737762	31.7063174
LRRC8D	19.82834029	17.99727037	18.8538567	23.462868	22.79592916	22.07724502	20.91774793	21.42051327	20.99429646	16.60437745	21.53817351	24.97801537
LRRC8E	0	0	0	0	0	0.012094651	0	0	0.179429984	0.284109485	0.215659655	0.085793892
LRRC9	0	0	0	0	0	0	0	0	0	0	0	0
LRRCC1	9.347791377	10.69510867	9.10818387	15.55133533	17.7874504	18.0368483	12.25214028	15.83157957	18.18222244	18.30694523	18.06475356	20.76554911
LRRD1	0	0.020963593	0.021402774	0	0	0.020766801	0.020794877	0.019786895	0	0	0	0.042088612
LRRFIP1	28.50183676	29.68186908	25.87059006	26.10846026	23.21856696	25.38861721	26.69759017	22.57125278	36.59057273	32.69474129	32.0729741	42.70261555
LRRFIP2	7.731449994	6.761711721	7.232099213	7.074220034	7.852947737	6.367882156	7.665477801	6.762064388	12.65080466	9.657693925	11.0164277	10.73571963
LRRIQ1	0	0	0.005293009	0	0.005221821	0.010271461	0.005142674	0.004893395	0	0	0	0
LRRIQ3	0.144068436	0.149355078	0.07624201	0.072804777	0.135389876	0.133157731	0.237044896	0.126874526	0.815268907	0.552919171	0.512111438	0.659692516
LRRIQ4	0.149589724	0.132924833	0.180946088	0.151189905	0.290082757	0.395031078	0.087903364	0.167284919	0	0.140598191	0.023018945	0.022239436
LRRK1	5.157320083	4.254028557	4.558330403	4.66733693	4.432037551	4.099048588	4.334993489	4.051786758	3.657351239	2.829880549	3.182106022	4.279043006
LRRK2	0.17058933	0.165059221	0.07222164	0.068965658	0.0475002	0.052556708	0.105255524	0.13353802	2.091587328	1.56505315	1.470021016	1.532676191
LRRN1	0	0	0.009124124	0	0	0	0	0	0.150101268	0.236319987	0.046428792	0.206340063
LRRN2	0	0	0	0	0	0	0	0	0	0	0	0
LRRN3	0.014433895	0.029927104	0.030554067	0	0	0	0	0	0	0	0	0
LRRN4	0	0	0	0	0	0	0	0	0	0	0	0
LRRN4CL	0.041632155	0.021579931	0	0.02103875	0.021735705	0	0.021406254	0.020368638	0	0	0.067266967	0.021663017
LRRTM1	0	0	0	0	0	0	0	0	0	0.072725194	0.047626708	0
LRRTM2	0.01912209	0.049559454	0.030358625	0.019326641	0.019966879	0.01963769	0.019664239	0.037422124	0.04161923	0.010484075	0.01029881	0.019900106
LRRTM3	0	0	0	0	0	0	0	0	0	0	0	0
LRRTM4	0	0	0	0	0	0	0	0	0	0.012115888	0	0
LRSAM1	6.69273145	6.829723747	5.728540624	4.258583395	4.071507147	6.108175282	5.745377586	5.216318662	2.457344121	4.377785075	3.14695817	3.234199868
LRTM1	0	0	0	0	0	0	0	0	0	0	0	0
LRTM2	0.024934922	0	0	0.025201654	0	0.012803628	0.025641876	0	0	0	0	0
LRTOMT	0.238436735	0.1817546	0.252364729	0.439447472	0.307551876	0.252067777	0.237985213	0.370553646	0.557116221	0.32297455	0.445684936	0.55466132
LRWD1	10.74302334	8.665019342	11.77040807	11.04885172	10.89713251	11.80861927	10.80480461	13.83901012	9.301487081	8.077825712	9.333894358	12.45785068
LSAMP	0.00656855	0.006809585	0.006952244	0	0	0	0	0	0.003574112	0	0.003537702	0.003417902
LSG1	21.31642133	17.12644207	22.39390678	23.46597746	21.97240707	19.03645883	22.54286015	23.73327926	18.88723227	14.1193551	16.77103899	20.74477409
LSM1	39.3027628	33.39359568	44.39471755	40.42584022	36.34477672	33.31810594	46.75607208	45.12460266	40.19890687	35.42295342	36.5942539	39.26193252
LSM10	24.08950136	21.60355954	25.55809819	24.75786532	20.06238441	18.12209213	23.39955319	27.49084931	18.31919584	17.56764544	19.44562011	26.15698295
LSM11	2.141267458	2.355404895	2.024141984	2.502841143	3.387934243	3.139035769	2.143145171	2.167215002	3.477563565	4.920018456	4.014358577	4.074038965
LSM12	9.692193773	8.652807734	10.99752979	9.778656462	11.73893229	11.1955345	10.28228666	10.08389416	8.712371263	10.1235742	9.320843767	7.746602973
LSM14A	37.35016577	34.98082474	38.311186	43.56072328	41.24590939	40.90431687	40.01432688	41.60472322	31.35060918	33.30109895	33.10496016	31.01824582
LSM14B	18.56305098	18.93383791	17.67560817	21.8213134	18.75788022	20.58388024	18.95250881	22.47718517	21.55885136	19.8626781	20.46128766	23.97479904
LSM2	53.19808858	62.75713691	64.26603618	76.13923604	68.82882832	72.78053577	66.5279632	79.81705271	47.51204039	58.73617265	52.89005459	50.90808985
LSM3	61.7842152	68.06465496	72.76844575	84.66870852	82.46120087	85.23627566	85.03303496	92.95704037	65.80431477	75.98405042	78.47764481	58.81898727
LSM4	68.8762379	66.29904655	80.67010393	88.35727392	79.15411467	79.51450093	79.89488622	98.78554881	45.7556573	54.54267371	59.13681765	43.01942804
LSM5	15.81377086	16.88145383	17.98151753	22.74328139	23.05042215	21.6389246	23.22846404	23.75445853	22.48841723	26.2918618	27.9449993	19.19263292
LSM6	20.65195094	20.0859358	22.0913407	26.03545305	24.82886355	23.87685935	24.72422395	26.49881417	21.22856619	26.07394611	23.38389518	18.74267563
LSM7	94.66214531	99.40003554	112.856857	112.621978	107.916854	120.6180831	122.6622347	126.0388794	55.82111474	63.3503056	68.30614369	44.49770857

LSM8	6.631641566	6.763601976	6.295738764	7.627823966	8.270948073	8.258172362	7.142302819	7.145153772	6.281448821	6.301026238	6.583190304	6.950662044
LSMEM1	0.225632919	0.274242357	0.197638339	0.212319195	0.121862634	0.223726553	0.208026947	0.144650902	0.465688857	0.546020815	0.603418546	0.445335372
LSMEM2	0	0	0.041180608	0	0	0	0	0.019035766	0.042341473	0.063996068	0.041910126	0.020245444
LSP1	0	0.714773769	0.032433247	0.092913158	0.047995557	0.251756075	0.23634041	0.059969167	1.483964943	0.873641078	0.891210705	0.430515452
LSR	0.04724281	0.048976402	0.025001221	0	0.024664969	0.048516645	0.072873355	0.023113665	0.05141199	0.025901832	0.025444119	0
LSS	16.81615465	25.87330173	14.0038566	16.25192328	23.41952225	24.76475042	14.08890652	18.30271334	34.87020543	30.01114817	47.46814121	9.83839468
LST1	263.8772767	151.6455509	304.4905682	116.9736867	146.148455	125.2158771	266.5362109	142.8394525	42.1357738	40.28119858	62.17121286	33.4742021
LTA	0.669511067	0.079323316	0.587142075	0.212668629	0.23968773	0.216091377	0.491780731	0.112306282	0.520425321	0.440487686	0.515123566	0.35832925
LTA4H	51.50013765	64.01135463	49.21982465	51.73275109	50.44316178	56.16601427	55.39993788	51.25597197	34.40840232	40.63313333	31.6607043	40.50798223
LTB	13.72324371	3.431909053	16.75456542	1.095003395	3.016740866	1.483502243	12.75060915	2.17914804	1.310028122	0.264001675	1.231848288	0.501108714
LTB4R	9.177907783	10.1304931	10.24410417	10.92412045	11.28600631	11.25543106	9.09077758	12.73015605	13.05519584	14.43180585	12.08162784	13.91487789
LTB4R2	3.587304057	2.86072419	2.884147258	3.590815619	3.385614789	2.975563154	2.447517041	3.948969624	5.180153509	6.013907385	5.424620983	6.533204864
LTBP1	0	0	0	0	0	0	0.00829364	0	1.105866365	1.78640342	1.103287824	1.619872224
LTBP2	0.036621752	0.0569484	0.025840645	0.049351333	0.025493103	0.031341004	0.043936726	0.011944857	0.591162078	0.78306622	0.5785651	0.533564826
LTBP3	0.152377461	0.200094074	0.193534088	0.164274627	0.159109303	0.104324068	0.208930217	0.178922543	1.470313043	1.002528543	1.192717708	0.761170609
LTBP4	2.359535798	3.093622096	2.699358005	1.885025189	2.500009096	3.109124624	2.80110313	2.402189056	4.389745209	5.51709449	4.251583938	4.224969314
LTBR	25.62556541	27.25037233	26.79488599	26.67125408	23.87077181	24.36715006	31.12603193	31.75730685	14.39257775	11.9230465	13.84591311	17.84320741
LTC4S	3.711004197	2.927202788	3.159299712	1.386128735	1.179333105	1.077040422	1.659225424	1.578798451	13.08122984	8.403921384	11.20998467	9.906851668
LTF	0.015738392	0.032631835	0	0.079533736	0.016433694	0.03232551	0.08092303	0.046200288	0	0.051773362	0.016952824	0
LTK	0.058109677	0.045181524	0.030752042	0.029365642	0.227538331	0.417735648	0.104575101	0.127936381	24.3307661	18.92472914	22.97186311	25.00598776
LTN1	9.763265902	8.052270707	9.631621109	9.538996152	10.81386122	9.784205392	9.508887583	6.789095285	14.27522337	12.99243038	12.89335361	13.41239329
LTV1	60.48016758	45.39748163	61.96418306	60.22820324	59.98357306	56.25444834	70.26517185	58.74500268	36.69498948	37.26139148	46.74694525	39.44696374
LUC7L	17.26084725	18.68630678	17.11388995	20.72341255	19.30963291	20.33636116	17.60591548	20.65837872	8.959362425	8.395000813	7.860352742	12.54336389
LUC7L2	2.352720117	2.254277193	2.810852737	2.612073351	2.177494349	2.086681774	1.887884164	2.389351169	3.433193607	2.755752495	2.572662567	3.691215922
LUC7L3	20.03372873	21.37214016	19.58788236	18.50372545	24.02441357	23.24524882	21.42517961	18.3474942	28.64856776	29.31948577	24.67082405	26.86332239
LUM	0.381447738	0.237267058	0.134576515	0.102807502	0	0	0.052301671	0	0	0	0	0
LURAP1	0.05820396	0.060339775	0.061603874	0.029413288	0	0	0	0	0	0.063823012	0.031347595	0
LURAP1L	0.069701504	0.198712869	0.129102807	0.228953106	0.163756861	0.304218846	0.089597098	0.255762281	0.208594828	0.363045049	0.225239781	0.163209226
LUZP1	10.98550575	9.284137039	10.30692762	11.13275403	9.873393701	8.635013154	9.977877729	8.860891929	12.04462346	9.265203014	9.728946432	13.19602263
LUZP2	0	0	0	0	0	0	0	0	0	0	0.006132216	0
LUZP4	0	0	0	0	0	0	0	0	0	0	0	0
LUZP6	0	0	0	0	0	0	0	0	0	0	0	0
LVRN	0.011644571	0.012071872	0	0.070614804	0.060795065	0.119585498	0.023949434	0.045577086	0.152066454	0.217068679	0.125431083	0.133301858
LXN	0	0	0	0.036871301	0	0	0	0	0	0.160011863	0	0
LY6D	0	0	0	0	0	0	0	0	0	0	0	0
LY6E	73.25604332	76.75060281	66.34805305	70.15501766	82.17797079	87.54271625	94.34269694	111.7979269	45.51211476	41.94528653	39.03543308	43.80866236
LY6G5B	22.87253944	19.9640849	23.98786396	21.00925764	22.43116379	22.27553369	23.09206618	22.99313924	30.71660513	26.60533159	31.22742949	30.74874936
LY6G5C	1.185885639	0.845213965	0.941368286	0.749107005	1.160884269	0.685047007	1.067069353	0.725246947	0.806587563	0.894005672	0.878207631	0.925601634
LY6G6C	0	0.126638188	0	0.061731179	0.063776162	0	0	0	0.199403827	0.401845909	0.39474486	0
LY6G6D	0	0	0	0	0	0	0	0	0	0	0	0
LY6G6F	0	0	0	0	0	0	0	0	0	0	0	0
LY6G6F-LY6(	0	0	0	0	0	0	0	0	0	0	0	0
LY6H	0	0	0	0	0	0	0	0	0	0	0	0
LY6K	0.04608956	0.191123329	0.048781826	0	0.120314344	0.189329194	0.165887013	0.067648302	0	0	0.024822999	0
LY6L	0	0	0	0	0	0	0	0	0	0	0	0
LY75	1.963909247	1.778460891	1.749991693	1.161137472	1.669717328	1.697991586	1.907833976	1.260875087	1.410737007	1.651305341	1.488341496	1.551037013
LY75-CD302	0	0	0	0	0	0	0	0	0	0	0	0
LY86	26.76454703	30.19736148	25.27465202	12.18908097	18.70104169	17.652075	25.01001259	16.34867544	22.5861334	20.42966052	20.15496322	18.38832047
LY9	0.264327667	0.234880512	0.19983432	0.114495092	0.105144886	0.155117077	0.155326787	0.049265898	0.191769731	0.179428507	0.230490985	0.013099158
LY96	2.245467259	1.227419977	1.555614664	0.247580434	0.468933828	0.293492572	1.511430989	0.439440221	0.133288901	0.134304342	0.219885067	0.212438918
LYAR	22.73905013	21.07033393	27.94280937	25.77127565	26.15401277	28.01171174	30.9964254	25.96298241	11.75207624	14.14010155	15.00487891	11.7630823
LYG1	0.130342093	0.247729245	0.206933804	0.197604569	0.181467254	0.133856581	0.268075097	0.255080799	0.591019954	0.786089767	0.397799339	0.791264215
LYG2	0	0	0	0	0	0	0	0	0.005743258	0.02893506	0.022738997	0.02746121
LYL1	9.009643018	12.87043019	9.323186568	14.42384058	9.24717912	11.57179016	8.450437635	15.93124199	5.056777637	5.043441358	5.298191325	7.161362145
LYN	26.98744229	21.54033555	27.33901484	24.43556303	22.01345877	18.25489893	25.54244692	21.04952388	28.26801447	18.75928677	23.90555211	24.85781278
LYNX1	0	0	0	0	0	0	0	0	0.105510373	0.070876125	0.092831557	0.213008824
LYNX1-SLURl	0	0	0	0	0	0	0	0	0	0	0	0
LYPD1	0	0	0	0.011976474	0	0	0	0	0.06447727	0.025987392	0.051056334	0.024663686
LYPD2	0	0	0	0	0	0	0	0	0	0	0	0
LYPD3	0.063082721	0	0	0.063757525	0.032934817	0	0.06487124	0	0.171624435	0.380450245	0.237826451	0.131298708
LYPD4	0	0	0	0	0	0	0	0	0	0	0	0
LYPD5	0	0	0	0	0	0	0	0	0.755866445	0.689773495	0.578770056	1.009234904
LYPD6	0.014883021	0.023143737	0.007876197	0	0	0	0	0	0.566875673	0.546714574	0.472927744	0.650519638
LYPD6B	0	0	0	0	0	0	0	0	0.107004581	0.026954945	0.13239311	0.204655638
LYPD8	0	0	0	0	0	0	0	0	0	0	0	0
LYPLA1	53.95970962	47.78716529	55.02588218	50.21190836	53.71747996	50.63453947	60.31475397	45.30686229	60.09012636	62.52364467	59.72056945	53.69409589
LYPLA2	14.52874767	13.21792442	14.42733416	14.80177125	12.23716189	12.59876998	13.86370749	17.25818874	15.35865315	14.67362414	14.96941126	17.09203338
LYPLAL1	3.962959603	3.972862266	3.757529108	3.226533465	3.29031502	3.108886558	3.940888442	2.94199331	2.800254206	3.372325193	2.779138073	2.756626525
LYRM1	10.46948853	8.458377392	9.017682917	5.546156073	6.766541667	6.265694396	8.575930077	6.323296994	6.148518772	6.749577877	5.658120015	7.251115381
LYRM2	11.90743784	11.85477694	12.11376618	14.29959242	12.86368163	13.05405791	13.48504091	12.90021325	13.48316171	10.75411193	12.4041293	12.84157755
LYRM4	5.659824248	4.763954667	6.149123086	5.386995664	5.933350984	5.442901115	5.820291411	6.315710684	1.182912688	1.49607079	1.542307801	1.232631097
LYRM7	8.768710254	8.670783123	8.92536784	9.280388943	9.686758784	10.62394921	9.663945599	8.377943133	7.911504556	9.284664637	7.747402362	8.722095177
LYRM9	0.33612302	0.464609564	0.166020045	0.211380444	0.101392057	0.138074438	0.245797525	0.168103464	1.121743403	1.523433319	1.094224278	1.298142158
LYSMD1	0.557834363	0.667274183	0.772087147	0.563801588	1.433793904	1.101683783	0.926665491	0.902741594	1.517658177	1.317482043	1.687083081	1.138733589
LYSMD2	7.408371682	6.06765067	6.418000833	6.821461618	7.047437751	5.577488714	7.564190024	6.320415208	3.328151798	2.775315991	2.925063928	3.210127959
LYSMD3	5.885242853	6.089402749	5.38563428	6.811089446	6.109586312	5.880264578	5.78285867	5.112691792	10.83953352	9.236986888	7.393887731	9.643135954
LYSMD4	0.689544699	0.662858855	0.550685094	0.728599067	0.595642903	0.701699672	0.799343059	0.778998357	1.889636208	1.505353912	1.512514168	2.087563796
LYST	52.73712476	48.06097847	45.58512876	45.2282359	44.76601521	48.70136339	39.57467533	33.90102494	65.70618081	49.58265747	50.96886698	57.0652922
LYVE1	0	0	0	0	0	0	0	0	0	0	0	0
LYZ	103.6437452	96.73908703	87.11972285	63.03083194	24.09064585	27.04700545	41.93938515	40.67056667	93.32239384	116.1794574	80.55759209	246.8236425
LYZL1	0	0	0	0	0	0	0	0	0	0	0	0
LYZL2	0	0	0	0	0	0	0	0	0	0	0	0
LYZL4	0	0	0	0	0	0	0	0	0	0	0	0
LYZL6	0	0	0	0	0	0	0	0	0	0	0	0
LZIC	7.3390402	6.804723545	7.543979263	9.462714087	7.495077161	7.164732982	7.981929735	8.481603608	7.866198049	7.959243013	7.872815128	8.737714056
LZTFL1	2.537573003	2.686901468	2.352946426	2.981210371	3.804668347	3.59716407	3.033286107	2.992367148	8.154727052	7.52716021	7.826348539	6.195766921
LZTR1	11.44778143	10.74312981	11.31136352	9.528432783	9.596679164	9.566526663	10.43866203	11.09189151	11.86093086	13.19564803	12.68038198	15.41754156
LZTS1	0.095328146	0.151533577	0.114349508	0.154156613	0.245531068	0.163164222	0.228738736	0.130590704	1.383211458	1.66553035	1.492341018	1.85185918
LZTS2	13.00551815	12.81181718	12.08534105	8.550245579	13.33873312	13.32454356	12.55024426	10.19283492	21.54849952	18.84017108	20.89741836	18.50593621
LZTS3	0.500304733	0.602020241	0.397147072	0.550804464	0.261203779	0.247722461	0.395054328	0.393388971	0.116669282	0.117558109	0.096233943	0.14876015
M1AP	0.213651121	0.427161454	0.145370117	0.123392328	0.04780499	0.235084197	0.062773872	0.134394882	0.365366323	0.78650187	0.509589567	0.381160968
M6PR	85.15532281	82.00389762	85.42917195	88.26016444	65.04526503	68.94263724	71.2885712	80.86959754	34.76219257	42.12414438	37.5185121	47.68818143
MAATS1	0.012005703	0.024892513	0.012707002	0	0	0	0	0	0.013065207	0	0.064660535	0.037482529
MAB21L1	0	0.019189584	0	0	0	0	0	0	0	0	0	0
MAB21L2	0	0	0	0	0	0	0	0	0	0	0	0
MAB21L3	0.029460477	0.076353848	0.046772062	0.029775619	0	0.030254836	0	0	0.288543287	0.258436898	0.12693502	0.398568675
MACC1	0.017625143	0.024362538	0.012436463	0	0	0.00603346	0.006041617	0	0.031967604	0.019326687	0.012656776	0
MACF1	13.0521707	10.9589018	10.2154899	8.056227778	10.73936946	10.30864839	9.270273517	5.502625353	13.01564076	13.82144988	11.68476258	11.509403
MACO1	8.887778546	9.836297956	9.517454843	9.457716683	10.11171588	10.38730424	9.354502064	9.373574919	9.643719754	8.686794865	9.433010709	9.61610972
MACROD1	2.738097123	3.59449678	2.520034571	4.030759177	4.008903221	5.593287565	2.876939038	4.208157159	1.246954036	2.039697675	1.699098614	1.115022322
MACROD2	0	0	0	0	0	0.010446211	0.010460334	0	0.387436483	0.290002596	0.252007407	0.359917308
MAD1L1	13.2860179	14.62362024	13.82312426	11.23015297	10.07590117	11.70379412	13.57715802	12.80275488	5.418707975	5.512113593	6.016342915	5.355018374
MAD2L1	39.06619022	34.02133152	47.17021038	66.67667054	80.91993043	69.67121793	56.98983562	70.48462241	59.65775146	88.91688401	76.05962087	52.10467778
MAD2L1BP	20.73153633	22.518989	21.55820004	20.58628775	21.16484317	19.66323066	21.62484782	22.32096532	29.45875509	23.63794874	27.87851268	28.96139369
MAD2L2	25.91861987	29.85256362	30.30605094	37.68735056	44.38732863	37.57901818	32.14164344	43.09411727	40.40241971	40.20134173	46.81426087	34.00019386
MADCAM1	0.035009474	0.108882476	0.111163529	0.035383974	0.036556147	0.035953454	0	0	0.266693428	0.076778626	0.075421866	0.182169482
MADD	16.83561372	17.46196229	15.77409395	16.79873322	17.49317392	17.23020589	15.91193871	15.83546717	18.92338336	16.85839267	16.84509233	20.7428789
MAEA	19.8379182	19.58029242	22.37191115	19.15331615	16.26372881	16.84174753	21.1336021	21.87670255	23.86473138	18.36502464	21.93191673	24.78396494
MAEL	0.079806542	0.062051303	0.063351258	0.080660243	0	0.040979205	0.020517304	0	0.151986577	0.240655587	0.085964707	0.18687063
MAF	0.006404756	0.003319891	0.003389441	0.001618317	0	0.001644363	0	0	0.505323292	0.417873031	0.303554718	0.641539489
MAF1	50.73235259	52.24911553	51.96464487	63.0430857	55.17364152	53.60919293	54.56543602	76.63293277	51.04614681	42.25454938	47.48022093	53.40235021
MAFA	0	0.023507847	0	0	0	0	0.023318655	0	0.172738221	0.024864886	0.048850993	0.212385204
MAFB	0.015858992	0.049322831	0.033570752	0.032057276	0.016559622	0.081433036	0.016308626	0.046554313	1.242615534	1.165132061	0.802888321	1.38635647
MAFF	2.0197188	2.159954139	1.237614766	0.945455321	1.465163457	1.441007612	1.071285352	1.227389479	0.994865687	1.748450445	1.534347746	0.442504139
MAFG	44.54087937	37.05492667	44.90966367	43.18517765	29.96486672	27.07278652	34.16583262	39.4846855	45.52921205	50.32329681	43.50988946	59.61207484
MAFK	7.001931749	5.621198193	6.612608038	5.868592644	6.865459129	5.875351585	6.219901177	6.69824346	8.951777991	7.116105449	8.308712876	7.005416332
MAG	0	0	0	0	0	0	0	0	0	0	0	0
MAGEA1	0	0	0	0	0.032877003	0	0	0	0	0	0	0
MAGEA10	0	0	0	0	0	0	0	0	0	0	0	0
MAGEA10-MA(	0	0	0	0	0	0	0	0	0	0	0	0
MAGEA11	0	0	0	0	0	0	0	0	0	0	0	0
MAGEA12	0.055388122	0.028710305	0.087935329	0	0	0	0	0	0	0	0	0
MAGEA2	0	0	0.024443254	0	0	0	0	0	0	0	0	0
MAGEA2B	0	0	0	0	0	0	0	0	0	0	0	0
MAGEA3	0	0	0	0	0	0	0	0	0	0	0	0
MAGEA4	0	0	0	0.025027689	0	0	0	0	4.33864675	4.996228661	4.774572	4.174791354
MAGEA5	0	0	0	0	0	0	0	0	0	0	0	0
MAGEA6	0	0	0	0	0	0	0	0	0	0	0	0
MAGEA8	0	0	0	0	0	0	0	0	0	0	0	0
MAGEA9	0	0	0	0	0	0	0	0	0	0	0	0
MAGEA9B	0	0	0	0	0	0	0	0	0	0	0	0
MAGEB1	0	0	0	0	0	0	0	0	0	0	0	0
MAGEB10	0	0	0	0	0	0	0	0	0	0	0	0
MAGEB16	0	0	0	0	0	0	0	0	0	0	0	0
MAGEB17	0	0	0	0	0	0.021747524	0	0	0	0	0.022810588	0
MAGEB18	0	0	0	0	0	0	0	0	0	0	0	0
MAGEB2	0.032434937	0	0	0	0	0.033309499	0	0	51.18111678	49.86385343	51.9524131	49.65303206
MAGEB3	0	0	0	0	0	0	0	0	0.024429811	0.172311481	0.072542808	0.186896617

MAGEB4	0	0	0	0	0	0	0	0	0	0	0	0
MAGEB5	0	0	0	0	0	0	0	0	0	0	0	0
MAGEB6	0	0	0	0	0	0	0	0	0	0	0	0
MAGEC1	0	0	0	0	0	0	0	0	0	0	0	0
MAGEC2	0	0.027975989	0	0	0	0	0	0	0	0	0	0
MAGEC3	0	0	0	0	0	0	0	0	0	0.015321832	0	0
MAGED1	0.125592855	0.217002548	0.192008858	0	0	0.057324127	0	0	8.868781671	8.921045188	8.988874167	9.730099124
MAGED2	11.27876428	13.21382126	10.59317096	13.01525402	11.18247641	11.67284412	12.29670397	13.22216393	27.16988728	34.36518506	26.75155553	25.1391068
MAGED4	0	0	0	0	0	0	0	0	0	0	0	0
MAGED4B	0	0	0.042454552	0	0	0	0	0	0	0	0	0
MAGEE1	0.014539195	0	0.015388485	0	0.015181518	0	0	0	0.838580823	1.418910921	0.736071352	1.180198354
MAGEE2	0	0	0	0	0	0	0	0	0	0	0	0
MAGEF1	16.21964766	17.97562067	15.54181131	13.32145628	16.10109212	16.49271712	15.69255355	14.30581941	20.50583437	25.25752053	21.02059407	23.27183727
MAGEH1	0.290862491	0.150767896	0.038481609	0.257227149	0.303712425	0.48539592	0.186943135	0.462491938	5.341462637	6.857265127	5.404537233	7.870115512
MAGEL2	0	0	0	0	0	0	0	0	0	0	0	0
MAGI1	0.00586864	0.006083992	0.012422899	0	0	0	0	0	0.08941167	0.025740811	0.050571887	0.036644497
MAGI2	0.004663817	0	0.001645416	0.001571235	0.001623286	0.003193046	0.003197363	0.003042379	0.055829384	0.051140647	0.045213244	0.05015358
MAGI3	0.194398703	0.228403196	0.160031097	0.327463684	0.166900413	0.270623633	0.093291468	0.22826414	3.013474854	3.22591353	2.503483989	2.949203407
MAGIX	0.446552372	0.570957815	0.378109751	0.436284893	0.435195121	0.412733715	0.41329171	0.291302508	0.32397376	0.212187238	0.43290899	0.216869871
MAGOH	50.74896415	50.76650228	55.67209707	70.21154041	53.8828426	60.59645523	66.82673052	72.71118673	76.76088988	90.76995683	87.24923117	84.88721967
MAGOHB	8.834405515	9.385659553	11.20509266	14.86921502	15.74297682	14.65864267	12.87650107	13.82407277	5.859798451	6.985253273	6.960123167	5.204782321
MAGT1	19.21196653	16.43564998	20.02268343	19.10647519	17.61600027	16.7897266	18.36709327	16.78295726	26.87681316	25.01304283	27.28033442	26.80205874
MAIP1	8.078551796	7.821583201	8.098444431	9.423885035	8.584094235	9.831391107	8.600373314	9.611248821	11.15395373	9.170653421	9.276939342	9.333149806
MAJIN	0	0	0	0	0	0	0	0	0.056523414	0.056954029	0.027973795	0.135132478
MAK	0.041466397	0.035823351	0.021944303	0.048894963	0.007216388	0.021292239	0.014214017	0.02028754	0.015041936	0.022734796	0.014888699	0.01438451
MAK16	27.35645554	22.74131654	28.23736107	25.4882925	32.99834648	30.28965818	31.7240512	25.57512131	17.35120152	15.74990854	18.08262439	16.6868571
MAL	0	0	0	0	0	0	0	0	0	0	0	0
MAL2	0	0.019795643	0	0.019299208	0.039877075	0	0	0	0	0	0	0
MALL	0	0	0	0	0	0	0	0	0	0	0	0
MALRD1	0	0	0	0	0	0	0	0	0.006144623	0	0	0
MALSU1	15.13823963	16.6448786	18.2074109	17.71111202	17.86673136	17.00684189	20.75658471	19.88512127	15.82520673	18.91359314	19.96293971	16.18382498
MALT1	6.623202721	6.077775332	7.188974947	7.975210236	7.632757097	6.693306426	8.071069834	5.819316578	6.966294421	6.984616751	6.223999195	7.6731903
MAMDC2	1.859063523	1.638190138	1.759961188	1.691055146	1.747075121	1.856157437	2.081706891	1.839315346	1.101479691	1.381676328	1.490761668	0.924358023
MAMDC4	1.014442541	0.975460052	0.762482591	0.846982134	1.381642589	1.479651623	0.710588221	1.409832507	1.695949521	1.853962582	2.137931692	0.933314981
MAML1	11.57427388	10.95602879	11.05618382	11.88814362	12.9495697	11.60633779	11.70904915	11.68429477	20.06513054	15.32072345	16.82236411	19.21751482
MAML2	1.519789051	1.757553424	1.290037832	0.572848998	1.225550977	0.922037853	1.041918709	0.471167144	1.233609815	1.04500663	1.0589573	0.548087656
MAML3	2.584010738	3.480124759	2.486320547	2.672922665	3.513158512	3.875469422	1.994902546	2.587786507	1.146260771	0.997116592	0.775434706	1.514123162
MAMLD1	0.010124094	0.020991203	0	0	0	0	0	0	0.033052638	0.155420741	0.109053064	0.221256232
MAMSTR	0.015817037	0.032794898	0.01674097	0.159862342	0.033031628	0.146191689	0.032530963	0.092862307	0.068851566	0.277504406	0.204450454	0.164605817
MAN1A1	0.425852941	0.525571148	0.418533724	0.420160526	0.804634765	0.718479676	1.032255817	0.912769815	6.2342972	8.594381265	6.258160203	10.15092155
MAN1A2	6.74260313	5.647359856	5.950912667	6.898752888	5.911995237	5.185442282	5.840384445	4.319954845	7.78522933	6.635473225	5.764121286	9.762696301
MAN1B1	9.272688659	9.286783294	8.186326369	9.142220156	7.610815342	8.391835108	7.342672106	9.731855195	14.53256729	12.90870428	12.37934714	20.7642498
MAN1C1	1.293864971	1.814759263	1.53055565	2.201945475	1.440432426	1.553467629	1.252281027	2.420396281	2.370905387	2.055138215	2.541460839	3.376171235
MAN2A1	12.09966649	11.16590847	11.53894728	13.21067333	11.34563121	11.60102177	11.26377416	10.41054642	12.64298914	12.77133629	11.4444672	14.69695622
MAN2A2	11.97184016	11.06667446	12.21890265	12.04686794	13.0721585	12.84124337	11.47869392	12.54339752	20.50110058	23.51790401	18.49961871	28.10086843
MAN2B1	111.2542269	115.8899138	105.1870395	100.1752222	70.70796072	88.22494036	93.1626796	109.3196838	48.2148401	56.11540901	47.86735056	56.27655064
MAN2B2	12.53627762	11.86328992	11.26807716	13.37212389	7.934623176	8.437618405	10.93481095	12.63167026	27.49509561	23.85553199	22.64453655	40.75809937
MAN2C1	40.073664	38.81081807	35.16789235	37.50800089	43.26501907	45.20119174	32.27398885	42.83251914	20.05419762	27.9011728	19.68193783	28.35349936
MANBA	8.86844218	9.790877524	7.557946149	8.187264965	5.828738956	6.868130624	6.727391985	7.686065308	6.484190565	7.897560379	5.05345333	10.31600719
MANBAL	5.532657999	6.059469272	5.950290584	5.276173073	5.707199695	5.269446712	4.909504944	5.937643658	10.31810085	9.736211024	9.708345057	9.867135989
MANEA	6.194732982	6.90939034	6.733497149	8.721054021	10.25346354	9.258352891	7.691706504	7.114431783	7.537201404	9.118128903	7.561689936	9.447300153
MANEAL	0.015858992	0	0.016785376	0	0	0	0	0	2.416196873	2.034633598	2.681988646	5.396887686
MANF	28.27376718	27.80516969	40.92557078	41.5089293	26.08047662	26.39648073	36.25803835	42.75658354	27.30286337	46.13737683	38.03924892	34.42507592
MANSC1	0.349270983	0.33945713	0.462091532	0.35300718	0.250732164	0.224180359	0.246931784	0.234962358	1.377841343	0.885664037	1.363804792	2.68067751
MANSC4	0.08248783	0.057009833	0.029102085	0	0	0.028237332	0.056551015	0	0.179534769	0.120601685	0.177705785	0.085843994
MAOA	0.009895101	0	0.020946223	0.01000095	0.010332254	0.010161909	0.020351294	0.009682407	0	0.021700763	0	0
MAOB	0	0	0	0	0	0	0	0	0	0	0	0
MAP10	0.315656452	0.317013341	0.396737982	0.209365452	0.60770323	0.577423673	0.44633316	0.299219208	6.934671689	5.289301385	5.4933457	6.816364588
MAP1A	0.109975745	0.048862004	0.138571244	0.105859209	0.054683016	0.064537762	0.086166685	0.117860569	0.490122638	0.281383391	0.276411044	0.517751376
MAP1B	0.083242278	0.122632412	0.166935363	0	0	0	0	0	9.244785902	7.1965928	10.67963975	4.778280897
MAP1LC3A	0	0	0	0	0	0.147263047	0.368655349	0.140314266	5.383770469	4.481344887	4.518000885	7.20039139
MAP1LC3B	19.80229469	20.18761047	19.14190945	19.91904339	14.12037116	11.90708307	20.99253746	17.60467228	30.20049971	27.95486967	30.1461658	29.98192792
MAP1LC3B2	1.396787118	1.654905959	2.534363569	1.478953905	1.944660521	1.09291389	2.325581839	1.692183111	2.822956768	2.479788292	2.865844433	2.699576087
MAP1LC3C	0	0	0	0	0	0	0	0	0.037227118	0.037510727	0.110543618	0
MAP1S	11.75295726	12.58941268	12.60200323	13.8678653	11.9078118	12.06985891	11.15315835	15.9256328	8.430566192	8.647852531	8.931064066	12.21662099
MAP2	0.034445992	0.040811429	0.036458113	0.00994699	0.005138253	0	0.005060372	0	0.021420487	0.016187757	0.015901702	0.035847489
MAP2K1	17.70685934	16.87094692	18.88702991	16.85174238	20.79127376	16.68427161	18.84654885	16.07228952	16.06040802	16.95336953	16.52019876	20.72174362
MAP2K2	48.32167164	48.44245439	51.22974216	48.08407482	41.75561863	44.83825734	49.77467857	54.39025779	29.39988984	29.69024011	31.46927143	33.53214902
MAP2K3	11.60560944	10.32965999	12.6876008	10.22495188	7.195258711	8.05677491	12.09168621	11.54292646	4.964676636	6.007185327	5.921592995	7.508898106
MAP2K4	7.233744077	7.589852639	7.246371988	8.371805614	7.005412321	6.466512913	7.104794018	7.579478886	4.785815297	4.493483814	4.346791335	5.447768427
MAP2K5	3.499541267	3.377754158	2.943431205	2.799644255	3.081396296	3.053126253	3.220833931	2.989569936	3.60542174	3.735139294	3.279178953	3.81888515
MAP2K6	0.191975348	0.339504623	0.211157566	0.292945644	0.408774191	0.386571938	0.197418227	0.224681961	1.949893694	1.989514406	1.982740335	0.936253013
MAP2K7	15.61478797	14.99217935	14.85654899	15.7664843	14.62504657	15.83322784	14.15369353	17.20945965	11.87348754	9.301592008	10.44487553	9.885874414
MAP3K1	19.2461269	21.23327508	17.55897153	15.34084563	24.36307522	21.6660157	19.18742663	11.90441594	15.69711793	14.50752548	13.80254192	13.85524871
MAP3K10	6.833186546	7.675492885	8.108070933	5.983521996	6.420071636	6.372825948	7.026920841	7.286539922	2.266570926	3.801183183	2.812033873	3.325491341
MAP3K11	10.33119472	9.539350226	11.18971522	12.23780452	9.749738357	9.155944996	11.47589124	13.13007533	33.31904187	28.66823226	29.73518131	38.55507729
MAP3K12	0.743295191	1.272740321	0.654121654	0.709041466	0.287779661	0.617531122	0.661308075	0.67011332	2.981081533	2.445160321	2.024116611	4.832436404
MAP3K13	0.278124786	0.325027304	0.235496887	0.255545384	0.221769152	0.254465038	0.306810911	0.31667958	0.54480476	0.54341027	0.468443428	0.699920453
MAP3K14	1.101941859	1.647429332	0.83483275	2.204012006	1.271742586	1.334160672	1.168968841	2.008960619	2.486737797	1.399112132	2.111560232	2.522907923
MAP3K15	0.029035241	0.060201399	0.0307313	0.036682294	0.045476972	0.089454405	0.037323059	0.049719479	0.24488142	0.437776958	0.226748874	0.226624439
MAP3K19	0	0	0	0	0	0	0	0	0	0	0	0.007896066
MAP3K2	7.011161737	5.928845659	5.871045479	5.973827413	5.427833542	4.934690329	5.340510633	4.427807431	7.854463078	7.030744022	6.869456843	7.899771613
MAP3K20	8.311324429	7.306545483	8.144794765	7.598199552	8.062508506	7.854523314	8.198001489	6.513290348	10.90830053	12.3367058	10.4035269	8.752714275
MAP3K21	0.559847043	0.827770525	0.563408036	0.640043772	0.555830517	0.754022972	0.55684375	0.404124471	6.801666904	7.155399993	6.870780154	5.549268331
MAP3K3	8.446973374	9.275295838	7.746839371	9.730837798	7.964795298	6.992228842	7.778431072	9.212700982	12.21410858	9.845727946	11.33335803	12.99776735
MAP3K4	10.63955726	11.45279546	11.37366468	15.97771554	15.26644108	16.11649558	11.98062458	14.45374503	14.4634746	14.51532887	14.51668966	15.16925188
MAP3K5	6.813888645	6.779697692	6.768103638	8.704234986	8.866281876	8.281201643	7.496327253	7.480140274	7.78378151	6.896959687	7.000919099	11.11082602
MAP3K6	1.741009483	2.100781104	1.832639002	2.653873149	2.294757865	2.403478219	1.555567838	2.569343744	3.986019973	3.494778184	4.437308643	4.197937254
MAP3K7	27.34567234	25.62821708	27.40584965	28.05758843	27.13474659	25.02541299	29.73185134	25.37735123	34.79401355	30.22452721	31.07605511	34.676781
MAP3K7CL	0.027736887	0.086264106	0.058714208	0.042050388	0.072405668	0.014242386	0.028523282	0.040711027	0.060369337	0.136865817	0.149385834	0.028865412
MAP3K8	2.177610853	2.133630811	1.714556279	1.919081828	1.02598966	1.111345434	1.679512807	1.396715557	3.09228057	2.511598908	2.595940583	3.053857284
MAP3K9	0.087819758	0.062074319	0.046474822	0.044379589	0.033345281	0.024596643	0.028734879	0.046872043	0.095569856	0.087543582	0.085996593	0.095547083
MAP4	15.52368011	16.2802467	15.83004325	17.71207068	16.10388222	16.22225949	16.00450794	16.5669969	17.6402203	17.89998673	20.1558556	16.49395484
MAP4K1	0.269586978	0.242215625	0.133156135	0.272470785	0.150131726	0.276856005	0.20330222	0.158275336	23.60715306	27.19640501	25.5929767	27.79370864
MAP4K2	5.03659779	4.904967978	4.492818238	4.155292974	3.346705254	3.644192623	4.070926196	3.780258105	5.532981921	5.742492735	6.421922494	6.323577125
MAP4K3	2.820708327	2.794512121	2.997514805	3.149764573	2.921571013	2.347781055	2.783718021	2.214739534	6.745772585	6.447952132	4.533043829	6.474613753
MAP4K4	21.17262916	19.62697155	19.88149105	18.51598076	18.10369843	17.07975164	20.4514574	16.6029864	36.564133	28.92675067	32.92323327	32.68352835
MAP4K5	8.188411435	6.614717988	7.412544376	7.38545062	8.930877366	7.239088869	7.764420789	6.228564926	6.703945014	6.72170103	6.292003122	6.14217171
MAP6	0	0	0	0	0	0	0	0	0.714818508	0.753003529	0.554772861	1.087508092
MAP6D1	2.013454101	1.852512928	1.86468414	2.187616698	2.233806307	1.990203606	1.578786361	2.117938183	2.93065178	3.28415367	2.683914248	3.038293856
MAP7	0.378148006	0.462028616	0.26682471	0.059148933	0.437159899	0.351359061	0.546268703	0.123339914	0.744654486	0.310991013	0.649784012	0.627779841
MAP7D1	19.51915443	19.21973941	20.34027904	19.77365514	19.70472602	17.70811333	20.69256278	21.85760218	15.38589499	15.80061117	16.88520219	16.84672755
MAP7D2	0.029651225	0.007684822	0.039229082	0.029968408	0.015480589	0.007612682	0	0.021760406	0.02420096	0.024385332	0.015969611	0.023143229
MAP7D3	5.832054201	6.638978555	6.829218237	6.863278013	7.208396172	7.76789979	6.776072953	6.652555615	8.59964823	9.044981936	8.477333005	10.61296496
MAP9	0	0.014436884	0.014739333	0	0	0	0	0	0	0	0	0
MAPK1	23.29889389	23.13559528	24.93147572	30.38092711	25.05814798	23.89985299	23.66827358	26.91957806	31.59507341	31.19537305	29.92924675	37.10237846
MAPK10	0.109166162	0.025867898	0.082530694	0.003152398	0.006513656	0	0.025659712	0	0.006788576	0.010260441	0.003359709	0.003245937
MAPK11	10.66894625	9.477105002	9.324231575	5.928453179	9.545515358	8.59253531	8.900061966	7.429022795	5.275301644	4.68442791	5.316931269	7.210059785
MAPK12	20.11149991	22.98715776	21.07701355	21.89681066	25.4241577	27.557709	19.60694925	26.94834914	13.80191991	17.34511792	16.39966314	16.37343131
MAPK13	0.176267174	0.232572276	0.186563608	0.315816196	0.28444776	0.32089903	0.107110956	0.368476419	0.392364736	0.491995974	0.431519533	0.508626091
MAPK14	20.92972921	21.12427873	21.0936227	22.70069564	18.80805807	19.01158996	20.45554368	19.40469137	24.51678612	24.37950068	23.11611671	29.64369254
MAPK15	0.052771717	0.072944259	0.055854313	0.071114964	0.055103104	0.054194631	0.0180893	0.051637393	0	0	0.018947925	0.018306276
MAPK1IP1L	12.7159455	11.56837039	13.57750743	13.0008341	13.84163835	12.72027761	13.77609106	12.62791816	13.98315002	11.45940013	13.0700922	13.33550419
MAPK3	9.074969912	14.60256885	9.575609893	10.83201754	10.9583151	12.55009897	10.10518068	10.84110992	21.14519577	30.73844023	27.19672282	19.06222216
MAPK4	0	0	0	0	0	0	0	0	0.0213888	0.008620699	0.004234181	0.012272386
MAPK6	24.83611798	22.13955983	24.20042426	21.81802105	25.32344859	23.93120266	24.41404625	18.08679288	33.11944842	36.72094939	32.70340358	26.61910569
MAPK7	3.343679064	4.233274014	3.695588779	4.68038432	4.016653188	4.634159984	2.784255079	4.980095503	3.815866627	3.19600268	4.111663334	5.081010893
MAPK8	9.031579053	8.987043996	9.129625886	11.06553174	10.23299365	11.03081041	9.474105283	9.851302058	7.329178516	6.599173447	6.668572132	7.512047405
MAPK8IP1	0.251838818	0.261080133	0.213239743	0.339377027	0.350619648	0.172419528	0.17265263	0.197140449	0.31060542	0.405022226	0.325525949	0.681421858
MAPK8IP2	0.453503351	0.594877115	0.577952201	0.458354534	0.386562082	0.285141685	0.247456891	0.579598798	0.503596982	0.284162794	0.279141332	0.549008812
MAPK8IP3	10.39941817	10.1218456	8.774061835	7.099201467	7.774937458	7.787117077	8.152083263	7.646449558	12.88216986	11.94423172	10.10729211	16.96973809
MAPK9	8.161591242	8.818846239	8.510500589	11.01306204	10.45000584	10.0207758	9.803649271	10.35836829	12.51533697	15.66638511	12.71310095	15.62258842
MAPKAP1	19.81929009	17.20238136	19.46252032	21.87077925	17.18238699	16.40927543	19.52929998	19.66347988	22.22399912	20.67286454	22.1327989	27.49654352
MAPKAPK2	32.36222906	33.031276	33.45340152	37.88228802	27.01308088	30.12284155	32.04941927	36.81002972	20.96016345	23.78670969	22.00368666	28.16784414
MAPKAPK3	47.51215269	46.49561428	46.06076509	60.04274402	46.56394063	49.70461798	48.10456554	62.83950818	22.76956015	30.6156538	29.15549064	22.07631819
MAPKAPK5	7.097138225	6.824917977	7.277921009	7.35262631	7.816814123	7.500549481	7.757589571	7.06675033	4.530609394	4.212341556	4.649970693	4.447529892
MAPKBP1	3.781286575	4.026758434	3.638992913	4.009007454	3.396574742	3.530862102	3.309806624	3.854454898	5.145598663	5.026772359	4.575729443	6.356207112
MAPRE1	53.78989362	48.58682822	58.28501011	64.84079677	53.84568202	50.99877847	55.1531443	62.15970705	61.29782464	64.02923518	61.09705897	66.92850848
MAPRE2	14.05784634	15.566304	14.13983643	16.71287995	13.19689932	14.39062392	14.25949166	15.56331061	12.18482469	11.0054213	10.45907234	12.97692715
MAPRE3	0.337496266	0.393615912	0.334885033	0.19187241	0.110127006	0.043324546	0.043383119	0.082560452	0.757515673	1.01771559	0.522586901	1.141490686
MAPT	0.083039446	0.305216154	0.11186012	0.099187316	0.055177833	0.155051792	0.085393778	0.059094196	0.15608963	0.579448121	0.357788294	0.345672215
MARC1	0.191663618	0.082790326	0.152144563	0.06457129	0.416939735	0.311649959	0.459894539	0.156286519	6.865690573	9.965416742	8.997913628	11.85135496
MARC2	0.023284102	0	0.073932646	0	0	0	0	0	1.925758346	2.936176136	1.906139973	2.616997436
43891	0.037264239	0.231789979	0.086770165	0.037662859	0.023346315	0.076538031	0.053649055	0.036463247	0.056773933	0.089895861	0.032111747	0.116341203
43900	0	0	0	0	0	0	0	0	0	0	0	0
43901	0	0	0	0	0	0	0	0	0	0	0	0
43892	8.433036467	9.013155034	7.516330187	7.124694576	3.816667544	4.021867436	6.980661644	7.536445345	7.216786654	6.384267579	6.327696906	10.40639411
43893	0.309218039	0.373020979	0.321330079	0.596640132	0.164374711	0.219402092	0.225480258	0.429101286	1.425564089	0.893912264	1.029515163	2.270146562

43894	0	0.012535758	0.012798378	0	0	0	0.012434869	0	0	0	0.013025102	0
43895	10.61163559	10.90537215	11.56635367	11.31136897	11.31444108	10.01781953	12.24097418	10.8349975	11.27554876	11.93964067	11.14648147	11.92137263
43896	25.6081093	26.45755772	23.04977602	24.83083055	27.99771335	28.3454532	22.52702977	20.31423861	38.1945035	40.08820699	34.19266714	36.32966708
43897	43.62788015	36.00546391	41.43881443	39.23551316	51.93131581	39.46972933	47.74878029	33.8087224	74.55465899	62.56433018	61.20577334	63.86318579
43898	4.623807593	4.793479845	4.38703333	4.581472696	4.552191143	4.129483401	4.364320437	4.32243619	4.061428908	3.558189216	3.344115479	3.394133043
43899	7.30079469	10.48405101	6.773278848	8.399183492	8.752717375	10.57076109	7.118493958	9.789740442	2.864155957	3.953391916	2.757307176	3.451858421
MARCKS	3.598967378	12.70883033	3.31982074	12.75639115	2.805425384	8.624019544	2.505888931	10.44254547	3.902977189	10.70190812	4.926610204	12.09451368
MARCKSL1	33.58791411	39.49887234	39.34190207	30.88325515	49.20841263	53.24391174	50.4109756	43.58529854	8.847470337	6.421730883	10.24163266	6.811645825
MARCO	0.286914769	0.919369916	0.220854217	0.263621758	0.027235482	0.187505191	0.187758689	0.357315073	0.198695007	0.543423709	0.280958331	0.217155206
MARCOL	0	0	0	0	0	0	0	0	0	0	0	0
MARF1	13.44375914	12.45532808	11.56156808	11.00544218	11.76656684	10.81383284	11.96455253	9.80359284	16.35844035	16.10449175	13.74480025	14.57997764
MARK1	0.012934991	0.013409645	0.020535859	0	0	0	0	0	0.014076504	0.028367488	0.027866204	0.033653184
MARK2	11.45232991	10.82124881	11.08047639	12.32026473	11.26303045	10.07795913	11.00804311	12.61946255	15.32792756	14.41131016	14.06837467	16.13979304
MARK3	22.11432707	21.99225868	22.28111554	25.85720087	23.43043455	22.64996573	23.80398991	24.86027151	30.65271728	27.43824815	27.47814099	33.07699018
MARK4	3.817882251	3.777105322	3.584668669	3.277839649	3.064929317	2.803601369	3.430083838	3.103138442	9.203128709	9.329511031	8.667170641	10.78750483
MARS	109.3727781	99.70335302	125.6477998	132.8378732	125.885414	119.0516912	123.5027795	137.8549396	48.07201	47.06180801	77.1497119	60.82075942
MARS2	15.56590147	11.70978648	14.88853078	13.70640142	13.15939599	11.33134768	12.79861026	12.72403127	6.070178899	5.351870689	6.721830227	6.657465459
MARVELD1	64.83079622	67.08915223	61.06984983	67.70844283	48.23698282	54.5434554	58.68570631	71.44011776	32.49015437	28.63635221	33.07237153	40.15243874
MARVELD2	0.034941273	0.012074485	0	0.058858408	0.024323289	0.023922276	0	0.056983689	0.025349895	0.01277151	0.025091647	0.048483895
MARVELD3	0	0	0	0	0	0	0	0	0	0	0	0
MAS1	0	0.049149006	0	0	0	0.048687629	0	0	0	0.051986233	0	0.049338237
MAS1L	0	0	0	0	0	0	0	0	0	0	0	0
MASP1	0	0	0	0	0	0	0	0	0.004921689	0	0	0.004706581
MASP2	0.020025888	0.04152149	0.021195676	0	0	0.020565857	0	0.019595433	0.021793173	0.021959201	0.043142318	0
MAST1	0.170505598	0.166364575	0.116771779	0.081096244	0.157092634	0.257504467	0.195967976	0.098141525	0.414764915	0.703873245	0.626613013	0.198318561
MAST2	4.092034202	4.306468491	4.221695093	6.085345968	3.503132881	4.358013376	3.641310497	5.729738712	7.324467693	9.072366354	7.910277314	12.08016189
MAST3	9.449306981	9.856197243	8.136111615	5.658402986	8.435592139	8.408228803	7.427739516	5.790402899	3.614512363	3.371678534	3.155866345	4.50557178
MAST4	0.001724444	0	0.003650351	0.005228673	0.00900314	0.001770942	0.001773336	0.00674951	0.148253503	0.141819256	0.128168105	0.141773907
MASTL	6.925983081	7.867926748	8.224266756	10.19023428	10.74823123	10.18906643	8.476524876	9.39352635	12.41614455	14.74833792	14.33612846	12.98237128
MAT1A	0	0	0	0	0	0	0	0	0.017202778	0.017333835	0.017027528	0
MAT2A	219.9640332	181.5674806	211.4414635	213.2528218	213.4014238	203.7063012	188.8317206	207.4588433	144.9735692	136.1850796	134.144901	163.6002427
MAT2B	36.30808142	32.7163338	37.74961687	37.82093832	39.81108356	37.61666205	36.65542333	36.51161312	35.50749186	33.94804361	33.02549556	31.95165798
MATK	12.96528167	14.02626139	12.73969152	7.416995921	17.51202759	19.87880455	17.07806196	11.54622591	1.446616841	2.975178267	3.099136778	0.928577341
MATN1	0.014049494	0	0.029740356	0.014199783	0	0.028856636	0.014447825	0.082485005	0.015289362	0.030811684	0	0.014621122
MATN2	0.025795571	0.013371074	0.013651194	0.013035755	0.040402779	0	0	0.012620548	4.968749521	5.614749908	4.473554309	9.90584548
MATN3	0	0	0	0	0	0	0	0.020384409	0	0	0	0
MATN4	0.019681624	0	0	0.059676483	0	0.040424622	0.080958548	0.077034277	0.021418529	0.064745107	0.021200331	0.020482406
MATR3	78.67031473	77.63577464	78.32077255	92.37941562	103.8456627	97.81534835	87.96896533	87.02810778	87.29472684	86.46224566	85.64086086	87.40652969
MAU2	20.11823989	18.82569738	19.92757772	19.47019062	21.56929382	20.53675427	20.48814187	19.80389644	16.89258209	12.41983035	14.74043326	16.18325665
MAVS	18.02947142	16.38785939	15.92316738	14.53074125	13.98106645	13.05575867	14.59652504	13.69217684	10.16349666	8.947651704	8.824005858	11.2844608
MAX	12.38540437	11.88557492	12.01057099	10.08197838	10.11886669	11.15522165	12.08251566	11.61148559	24.75293043	24.02386501	24.1221647	26.89340585
MAZ	81.20079768	95.87653461	86.83516899	120.9294691	111.650749	118.3603702	85.88880021	134.1221672	96.83695083	97.96449496	103.2266503	105.0076709
MB	0.109965043	0	0.077592345	0	0.114823158	0	0.339248304	0.035867117	0.797796418	0.040193715	1.026569683	0.305171111
MB21D2	0	0	0	0.016147615	0	0.032814999	0	0	0.139093247	0.017519114	0.034419064	0.216147769
MBD1	16.38986296	15.85732246	15.61066399	17.51915677	14.33223913	15.88964661	15.65713111	17.13378077	18.94983597	20.08083297	18.86130849	21.49072495
MBD2	7.62406004	9.037206669	9.409236904	11.25310653	9.923563971	10.32132607	10.11831799	10.63193153	10.66593098	11.14678966	12.01380527	11.46724911
MBD3	51.82857663	49.31057416	54.53701707	64.04783961	61.20066662	59.59461218	47.60728729	75.93483541	30.36874796	31.26122114	35.27801171	36.70516058
MBD3L1	0	0	0	0	0	0	0	0	0	0	0	0
MBD3L2	0	0	0	0	0	0	0	0	0	0	0	0
MBD3L2B	0	0	0	0	0	0	0	0	0	0	0	0
MBD3L3	0	0	0	0	0	0	0	0	0	0	0	0
MBD3L4	0	0	0	0	0	0	0	0	0	0	0	0
MBD3L5	0	0	0	0	0	0	0	0	0	0	0	0
MBD4	23.2579158	25.0223823	24.74051142	29.531413	22.74081915	22.60655123	24.42940727	26.54143257	28.75315616	25.00539218	22.30752558	36.13864702
MBD5	1.066698114	0.920652298	1.210037277	0.882088953	0.810053438	0.791456819	0.965728056	0.804052116	3.671346668	3.218013398	3.144654699	3.170951589
MBD6	18.34843135	18.31256486	17.73862514	17.97598765	18.49083695	17.61944086	16.1455698	19.94052873	16.56969341	13.87697711	15.49765462	18.5553046
MBIP	11.1341385	12.05045347	10.92055758	14.61931413	12.30790917	12.47384138	14.20314078	13.19518167	12.18779258	10.74108756	11.64158004	11.11143199
MBL2	0	0	0	0	0	0	0	0	0	0	0	0
MBLAC1	0.171258946	0.355086707	0.435030793	0.55389096	0.143059958	0.246227377	0.352228949	0.335155495	0.335470601	0.52581874	0.664106089	0.712907687
MBLAC2	7.317094491	5.682681556	6.826349722	6.048443984	7.53533234	7.540212104	7.369403199	5.068459456	3.283640583	3.7084525	3.602292798	4.631684623
MBNL1	70.74580156	58.61097177	66.06058539	56.5525429	69.07098813	56.11181017	65.00156084	47.44394878	96.2774094	69.23660733	77.83576375	83.9769583
MBNL2	1.447581475	1.177969593	1.095562614	0.998975472	1.194599277	1.094978713	1.216509331	1.005233807	0.14398183	0.187748949	0.125748562	0.178185678
MBNL3	1.693643057	1.64904563	1.537029862	1.812240854	2.087308982	1.990907976	1.814686799	1.535638028	4.296719357	4.123103427	3.881961677	3.924172258
MBOAT1	3.287734023	4.238499443	3.609402457	4.570182235	4.396971111	4.692108264	3.671573639	4.664282516	2.40917199	2.282907341	1.968587274	2.519558299
MBOAT2	0.006869598	0.021365041	0.014541754	0.006943083	0	0	0.007064365	0	4.463076659	5.559201873	4.861590617	4.568274781
MBOAT4	0	0	0	0	0.022628594	0	0	0	0	0	0	0.022552919
MBOAT7	11.24793401	12.88433262	12.21361581	10.17528961	9.555380722	10.13940285	12.43791773	11.91654662	8.024370194	9.16661506	9.438409035	14.46578006
MBP	15.44988121	13.45657106	14.43247059	11.52783034	12.69686358	12.45124378	12.89202415	9.40107374	14.34367523	13.82867934	13.33901593	16.73633635
MBTD1	14.10829474	12.80104334	12.81840674	8.947292601	14.74041839	13.16759905	13.08966538	7.767921794	11.81664734	9.939239529	10.16472154	9.855735734
MBTPS1	21.6354914	20.72395108	22.15006262	25.74654545	20.44670604	20.3451049	22.46421212	23.72650022	27.80893327	21.98588045	24.77629245	37.33259923
MBTPS2	6.445255688	6.348911088	6.960196257	5.817109746	6.096733288	6.240462835	6.114383191	4.887122629	6.599936155	8.488806122	6.673601681	7.994538928
MC1R	1.64953478	1.782067779	1.451845667	1.684729327	2.139413436	1.818834067	1.607023268	1.953884078	1.757314588	1.694543181	1.103498069	1.535949145
MC2R	0	0	0	0	0	0	0	0	0	0	0	0
MC3R	0	0	0	0	0	0	0	0	0	0	0	0
MC4R	0	0	0	0	0	0	0	0	0	0	0	0
MC5R	0	0	0	0	0	0	0	0	0	0	0	0
MCAM	0.00882558	0.036597751	0.037364462	0.008919988	0.018430966	0.054381294	0.03630321	0.008635875	0.17287988	0.135486508	0.199638474	0.165323959
MCAT	7.661370825	7.729285854	9.496665396	8.392933329	9.717926949	10.87783513	9.755698494	9.534381192	8.617268598	5.976553923	8.695640205	9.23058772
MCC	0.003975586	0	0.002103908	0	0	0	0	0	0.015142512	0.017437569	0.021411786	0.016549361
MCCC1	16.08394687	14.92419237	14.74805672	12.53804272	14.48319077	13.67201638	13.51036209	11.59330532	14.34928584	15.69841152	15.48961776	13.22495451
MCCC2	26.23657361	19.74925208	25.82090717	19.66174207	20.0497878	19.6027025	24.8152557	19.96073993	24.6005049	28.64504772	28.64133857	21.07175059
MCCD1	0	0	0	0	0	0	0	0	0	0	0	0
MCEE	4.032707581	5.241461099	4.650506751	5.596684017	7.227608325	7.29388603	4.889796714	5.418422154	7.336157484	8.646054854	6.353743802	8.581486735
MCEMP1	30.73068957	39.32183502	36.00913619	31.39205068	11.03976089	15.92751749	20.26417845	18.35606676	24.08588861	19.6230144	30.57612808	24.74569077
MCF2	0.022171224	0.022984805	0.011733165	0	0.046301441	0.034153559	0.011399911	0.043389311	0.060319589	0.0486233	0.107469166	0.03460995
MCF2L	0.005355251	0	0.005668071	0	0.005591839	0	0	0.00524014	0.046622816	0.023489003	0.057684817	0.016719416
MCF2L2	0.004353172	0	0.009214915	0	0.00454549	0.008941098	0.004476593	0.008519202	0.64901555	0.992873543	0.65178196	0.693134174
MCFD2	25.83913112	26.62100015	26.32974621	23.56767847	25.36542017	25.3120201	25.52299268	22.22276489	44.01845133	47.88451249	40.27449724	48.02120222
MCHR1	0	0	0	0	0	0	0	0	0.145006002	0.219166062	0.143528775	0.069334171
MCHR2	0	0	0	0	0	0	0	0	0	0	0	0
MCIDAS	0.015325993	0	0	0	0.016003076	0.031478473	0	0	1.300923968	1.260418136	1.485774232	1.435460238
MCL1	189.7334164	191.2326844	182.7121834	144.0755921	114.7226931	94.13489159	142.7574406	109.8162302	173.8119773	148.5365743	149.0330922	157.8428403
MCM10	12.2449361	13.71760669	15.83334584	20.33356817	22.25875796	21.57800828	16.80961313	19.00748865	14.28468511	13.22089716	17.91129301	15.22573249
MCM2	38.17966605	53.72555193	51.10942913	67.33908631	71.80035128	82.68212867	51.86995189	74.99767118	62.683692	65.07471535	81.4737059	59.74050824
MCM3	89.35272154	111.4986871	111.0827782	151.3392768	157.3685741	165.2646008	112.4814931	152.5096665	146.559927	147.9155793	188.0503154	154.8174045
MCM3AP	28.92867897	25.4572026	28.64826346	28.73375094	29.20588635	27.70751095	27.45986323	28.38436972	30.91269098	25.68466582	28.029471	29.42147357
MCM4	68.38936865	87.55701445	93.08698083	122.1232458	137.3467777	140.9838632	92.4804189	120.6540353	101.9290085	109.7870244	128.3740906	103.431652
MCM5	34.66788662	42.82328965	45.70527755	57.91977767	67.40651729	68.79363209	47.81236484	66.34103654	69.75511061	61.89438452	83.42117726	70.64476469
MCM6	42.65547972	47.31412698	58.57699582	67.42497137	72.83363995	73.23798298	61.09224634	61.39829191	57.59955309	53.1927473	67.19088436	60.76035789
MCM7	67.81801758	75.43760229	82.06937881	108.710786	120.4024801	121.261384	82.87399493	119.8491922	102.3847763	85.89796408	118.3986402	94.24616207
MCM8	5.331328055	5.511681095	6.240830752	9.768571513	9.743286513	9.562466349	6.088839128	8.596788955	3.957000295	4.525949635	4.599463107	4.581774574
MCM9	3.44899414	3.811506607	3.70605377	6.591337336	4.561121281	5.160159951	3.366740326	5.16507496	5.782473082	5.039417038	5.346394335	7.44283396
MCMBP	42.58454325	44.65897903	46.440527	62.57026715	53.21667952	54.89833236	51.18926081	60.42780372	29.59890931	26.03513241	31.27247882	36.85554439
MCMDC2	0.033380621	0.060559685	0.052995764	0.016868849	0.060996835	0.059991193	0.034327027	0.057160437	0.027244847	0.045754012	0.017978196	0.034738771
MCOLN1	12.31105301	10.68885459	12.81301848	11.66507429	9.319831231	9.113497933	13.02934839	12.9248132	5.442736	3.346768612	4.779492246	8.327767606
MCOLN2	6.328852023	6.739220663	5.971100541	6.353137045	6.997185141	9.911002947	7.421214638	7.299674204	27.59624089	23.30029046	23.104479	17.33015416
MCOLN3	0	0	0	0.013877308	0	0	0	0	3.60105638	2.589628065	2.381177415	4.686817764
MCPH1	1.875857436	1.634176489	1.84890591	1.929069242	1.903940763	1.930927774	1.857096078	1.826978742	2.124675912	1.745294349	2.086545971	2.131926223
MCRIP1	13.84077943	9.224146107	13.07970699	19.30602114	16.62745812	11.42194518	10.74751292	20.07306519	22.2475268	18.93250594	20.34744846	32.84974774
MCRIP2	20.34057746	23.07301369	24.92802365	26.25294526	21.35686691	21.52553988	20.8593303	31.04041743	9.197631999	11.18302802	11.3495692	10.20294073
MCRS1	25.40038023	25.27011672	26.4225829	32.1286774	26.81849627	27.87652792	28.56442621	34.54015031	13.09730871	10.37597183	13.17524949	12.54756526
MCTP1	7.961515165	8.162503791	7.624728107	7.103229437	5.177237385	5.446159587	6.350849563	5.639257691	3.386146857	4.695131233	3.205927041	4.047689105
MCTP2	0	0	0	0	0	0.010805721	0.016230495	0.00514792	0	0.005768906	0	0
MCTS1	4.430104408	4.738289855	4.557366134	4.608542399	5.364823845	5.198699767	5.016832991	4.419462659	5.3090468	5.450203253	5.650684162	4.627218247
MCU	12.69054034	16.12791519	13.96204724	19.68948436	13.585378	14.16165359	13.92322688	17.83691133	9.630993404	12.05416492	9.323035861	16.68237173
MCUB	2.479419979	2.294244152	2.971260868	2.609494	3.102469841	2.041227906	2.676148637	3.127896732	3.679403052	3.752372626	4.524809311	2.622949184
MCUR1	7.588779866	7.062184205	7.948835388	7.669958012	7.328712865	7.833780754	8.07687233	7.387184683	2.813449325	2.759430002	3.144798317	2.659794245
MDC1	19.50415193	20.19251181	20.44100805	28.83253913	32.81810437	29.73009961	21.16953407	28.62414037	12.64766474	12.9103712	12.48329411	12.69894127
MDFI	0	0	0	0	0	0	0	0	0	0.018256304	0	0
MDFIC	0.116998502	0.131399453	0.154791035	0.137958391	1.435466318	1.181506452	1.463840281	1.049433252	15.24700406	16.57125933	13.60036351	12.47012986
MDFIC2	0	0	0	0	0	0	0	0	0	0	0	0
MDGA1	1.389029717	2.619760225	1.245805189	4.251131777	2.114431671	2.320183052	0.894908375	3.597167714	3.023223307	3.60290032	3.004442446	3.134916584
MDGA2	0	0	0.004318531	0	0	0	0	0	0.435146284	0.639795688	0.395553028	0.658161156
MDH1	123.2992963	130.3998259	129.0204196	120.2882153	116.6681296	121.8135268	129.467845	116.8201913	141.4536241	156.7186249	154.7491345	121.9254156
MDH1B	0.126362961	0.130999894	0.089162867	0.127714682	0.065972758	0.108141798	0.1299456	0.206077997	0.068757249	0.254030574	0.090742393	0.284925904
MDH2	122.4244993	118.7111814	133.1085163	114.2642754	121.8583869	124.9664956	132.9809263	128.704566	99.49974118	116.074179	121.5218351	88.89856204
MDK	2.092594033	1.518567757	2.214830384	2.568188517	1.217380637	1.350811209	1.660055036	2.486388282	7.482443958	6.359360346	4.02511831	11.85310025
MDM1	3.970758539	3.78075506	3.957887797	5.634113325	5.410163158	5.138850496	4.670069334	5.522559612	4.136584143	3.804552007	3.645964738	4.693989944
MDM2	40.21215867	33.63968532	33.10228715	18.35107469	16.2405061	15.85499996	34.09722804	17.95136188	18.87839523	13.0907173	16.36121292	14.6840933
MDM4	9.458151781	10.28831632	8.975428801	8.845094512	11.75816237	12.04812624	8.504596129	9.044398137	5.929385755	6.502385373	5.510443658	4.679009629
MDN1	20.20612586	14.25768509	17.54857047	13.52720734	19.19182052	17.779523	16.76351763	10.857345	12.84058274	13.75633531	14.22808137	10.60804951
MDP1	0.964575995	1.214251048	0.583383163	0.76598829	0.503594866	0.990584405	1.062775311	0.876425183	0.449871373	0.377748875	0.667932565	1.14722456
MDS2	0	0	0.040392043	0	0	0	0	0	0	0	0	0
ME1	3.287597495	2.314430738	3.26924178	1.514562808	2.506771067	2.119966441	4.025519459	1.645871587	13.94481939	15.50981707	14.98867585	9.834418722
ME2	27.55049516	23.45948469	28.95048407	22.33025625	27.1518908	25.00759779	31.5978764	21.69869868	22.16983959	27.28872578	25.02692372	21.55200863
ME3	0	0	0	0	0.015469934	0	0	0	5.207686407	5.442308757	4.500330515	4.24000473
MEA1	17.98537462	19.18800139	20.7201753	27.25606893	21.70952959	22.0826813	20.86372672	29.0308169	25.92968737	26.81599581	30.31149016	29.45934289
MEAF6	12.07308744	11.30039493	12.13999718	14.008301	12.36156217	12.54772554	12.86330179	14.37081792	12.44575659	12.9079723	11.77760645	13.33141074

MECOM	0	0	0	0	0	0	0	0	0	0.006736428	0	0
MECP2	10.41709054	9.983265003	9.226330125	10.11363035	8.754288496	8.694272471	8.368314295	9.106281896	8.009583895	6.047657109	6.544231126	8.262626969
MECR	10.46980782	10.44570013	12.24510851	11.42768563	12.04614804	12.10033829	12.1673242	13.03878554	6.01833871	7.701699366	7.512572142	6.933684943
MED1	21.17411169	18.58558659	20.66502512	23.20789537	23.11547632	21.81280394	22.40548428	20.00875842	22.48263677	20.6641929	20.44118452	21.21160006
MED10	27.00946829	27.56647259	26.92514758	28.83260398	27.49217871	27.89900583	30.1974992	30.1678743	27.05756041	26.51753011	30.10851143	28.59865943
MED11	10.15170066	10.63923971	9.687844242	9.755690037	12.91717123	12.76117806	11.23817327	10.96483662	4.950285649	4.927169404	4.06329469	4.849389178
MED12	15.77456347	14.98462847	13.852054	11.78384339	15.94740819	16.04924209	13.10130966	12.39825734	13.32664596	11.54856746	11.90173596	11.96475253
MED12L	0.013531323	0.009351906	0.004773913	0.00455869	0	0	0	0	0.535025148	0.514371746	0.490706795	0.492865413
MED13	10.85133835	9.375440753	9.943328687	10.73417061	11.04458158	9.751555188	10.27398846	7.753040196	23.22552744	18.47840708	18.33831242	20.29910048
MED13L	48.64159751	41.82240074	40.65975386	33.9538784	47.12404099	38.60940907	38.70122512	24.96665395	55.8510532	43.66335772	42.41158106	42.47027052
MED14	11.33923621	10.24371423	10.24680027	12.06647551	12.96856232	12.66353837	10.77018742	9.987398646	13.87838747	14.64964177	11.85544062	13.41816806
MED14OS	0.119047701	0.246832397	0.315004321	0.060160585	0.124307077	0.244515303	0.306057344	0	0.129553666	0	0.192350786	0.309728423
MED15	11.49854992	11.94416069	11.47754396	10.42940017	12.38000739	11.65229016	10.49419603	11.70561495	17.75546643	14.58628855	15.68925199	17.34374041
MED16	9.863558345	10.54861586	9.818775925	10.22848827	13.66861132	12.46419883	11.89658976	14.19027813	11.43232753	13.34886016	12.20454271	9.978679418
MED17	11.40202591	10.14546794	11.43290034	11.46178703	11.35947001	10.5717034	12.23165135	10.78052522	9.645283405	7.289073411	8.618840693	10.34466361
MED18	9.5990552	9.212056794	9.666297744	8.537529016	8.992178782	8.224288431	8.996900384	8.694978989	13.10248463	10.97685863	11.61006602	12.90086762
MED19	7.37287017	6.423042222	6.852695045	7.357809223	5.854813214	4.962942738	7.996043257	7.608454237	7.113294324	5.129338242	5.038697324	7.866281702
MED20	14.61300779	13.66680521	15.11415274	15.34346766	15.3813151	13.86037712	16.6791114	15.60228746	14.85806522	13.44620981	14.70670093	14.29021772
MED21	8.076407286	8.269660924	8.653454017	11.88230486	10.59344447	10.54136659	9.491873529	10.1412849	28.4889709	26.9173375	24.81318035	32.19160358
MED22	11.63979576	11.6606285	12.83131368	13.33552325	14.56846345	13.81846864	12.77585449	14.2913265	7.520591888	8.007634198	9.315525084	7.151109684
MED23	14.30710009	14.39869854	13.25489454	17.61508569	14.56083991	14.61654394	14.08218879	13.41776972	26.3370614	24.13352259	21.26530046	25.53402829
MED24	20.87535015	17.96534791	20.60522421	22.93938522	23.09421492	22.71346541	22.46040276	24.43635904	23.49837751	25.31136366	24.86408467	27.58304735
MED25	12.62215591	12.92394912	12.82398692	11.99672837	13.41006043	13.56199297	12.61983337	14.06447555	15.79717687	15.33430944	16.54451715	16.05175765
MED26	4.512296718	4.853078427	4.775877075	4.850938405	5.876320334	5.050500453	5.630839947	4.894870221	4.892115697	3.910151692	5.133542849	6.138070256
MED27	3.331998897	2.929219072	3.968638327	3.744817587	3.228699789	2.60972443	3.435604875	3.868981688	4.97010306	5.27103146	4.708909875	5.677563389
MED28	33.69278686	32.90191305	31.56906466	31.9859304	32.24756112	32.17756025	34.32444624	32.0008399	44.02876471	46.3112863	42.32124394	48.84114485
MED29	20.07753623	17.38587349	20.02136835	20.85680872	18.07922666	15.4113434	21.2362499	21.34306081	20.90566566	16.69722257	20.61353132	22.53110571
MED30	11.27318682	11.81719712	12.73009987	14.48576921	15.09692019	14.54675623	14.13546345	15.1725728	22.57502901	26.51520561	23.74443623	19.49494375
MED31	4.256674199	4.749735	4.505322676	4.729145096	4.003648695	4.338079477	4.744923816	4.832877479	1.803424583	1.639010399	1.610047324	1.082104325
MED4	20.19179985	19.42972075	20.81182185	23.4605681	22.030019	21.17050967	22.70558951	22.37343067	17.43763984	16.41126438	17.79682958	18.92300707
MED6	6.751344985	5.65489227	7.618942908	6.552429952	6.792836893	5.532933326	6.643898428	5.840603183	11.7262484	10.44281823	10.57132859	10.67864336
MED7	6.493628362	5.635141785	7.027394032	8.001072262	6.818601325	6.144213698	6.902827722	7.103683211	7.979803166	8.800652445	7.662733704	8.352378132
MED8	14.33171719	15.45628348	17.39143468	17.2706072	14.60856764	15.41901576	15.76377465	16.5407248	75.01169905	64.84724654	73.54067864	70.25813064
MED9	6.32056498	6.276341325	6.125884218	6.951119659	8.066421586	7.560386771	7.869447817	8.530627451	4.928170149	4.939160049	4.904050535	5.771263652
MEDAG	0	0	0	0	0.016035045	0	0	0	0.016711831	0.033678296	0	0.015981421
MEF2A	4.491886332	3.951917172	3.953329108	3.304746652	3.976218509	3.860793112	4.082409543	2.926262618	5.843935258	4.521731027	5.392090504	4.615687232
MEF2B	0.222506799	0.115335882	0.157002843	0.112443491	0.154891244	0.228506376	0.076271768	0.326586047	0.080714345	0.203323137	0.119838119	0.154373257
MEF2C	34.98273735	17.45344729	31.93919533	13.53210141	39.19525687	18.81267111	34.54829353	13.71918842	40.53573364	34.2943633	37.28275464	25.95942318
MEF2D	67.66874911	57.61644686	67.54160394	46.49676719	64.4829933	59.43236389	56.59304761	50.24264308	80.27182975	82.83992661	82.64529049	73.76205319
MEFV	4.893060173	6.549079277	3.686409062	3.391947098	2.694492702	3.432056782	4.408252368	2.80098125	6.353018455	5.999396553	5.33138312	9.113028349
MEGF10	0.017900719	0.006185864	0.006315456	0	0.031152583	0.030638977	0.01227216	0.011677296	1.058438224	0.778611726	0.829126167	1.353710351
MEGF11	0.003680796	0	0.003895806	0	0	0	0	0	0.028039387	0.024216858	0.0198241	0.030644448
MEGF6	0.074554293	0.128816815	0.032878872	0.150703619	0.058386005	0.082944921	0.031945022	0.139824195	3.61045021	2.581994906	2.783982072	2.37288949
MEGF8	5.131833287	5.435042931	4.824691601	4.592568639	3.899415146	4.013274102	3.75111687	4.620745926	13.89100232	12.69701008	12.52454472	15.79121809
MEGF9	2.74680045	3.539282439	2.751342092	5.105725168	2.460999876	3.070025476	3.091997303	3.883259448	4.516812421	4.180664205	3.640106993	6.555748705
MEI1	0.326323142	0.31496681	0.250106317	0.181966269	0.082247509	0.219562675	0.370289706	0.1101065	1.420484697	1.406628749	1.745396346	1.112483352
MEI4	0	0	0	0	0	0	0	0	0	0	0	0
MEIG1	0.152004409	0.157582265	0.321767126	0.15363042	0.15871977	0.078051495	0.468942101	0.074368543	1.157931637	1.4167717	0.736801318	0.632755919
MEIKIN	0	0	0	0	0	0	0	0.033730255	0	0.037799087	0.037131136	0
MEIOB	0.057122676	0	0.060459428	0	0.029823142	0	0	0	21.44649542	29.50218717	28.79626042	14.50502263
MEIOC	0	0	0	0	0	0	0	0	1.105789872	1.3590964	1.190746765	0.650744622
MEIS1	11.23217492	8.084401164	10.77823777	8.428760805	16.42682261	11.58715054	12.94227127	8.772729334	0.036817515	0.018549002	0.018221221	0.052812542
MEIS2	12.80089655	13.27062996	13.42169902	16.02351269	18.19382051	15.56474922	15.07000317	14.84097737	4.319190592	4.292314162	4.145994203	4.323319242
MEIS3	0.016105825	0.066787335	0	0.016278111	0.01681736	0	0.04968737	0.031519263	1.244428689	0.865373943	0.954173511	1.156517216
MELK	10.80246751	12.53661659	12.5260999	22.93530063	19.28714362	19.04488569	13.32669511	21.59148859	28.34628888	29.55272192	34.15352185	25.2848745
MELTF	1.5229121	1.92963943	1.499935671	0.983379621	0.927612242	1.357617398	1.087562262	1.076239101	1.634290979	2.933983237	2.574556806	2.091153848
MEMO1	21.66721029	17.42479597	20.65644918	18.75905889	21.9590153	19.30639263	21.05275817	18.43437091	17.53280161	15.63550408	16.06710438	15.29503603
MEN1	13.55348359	13.80508842	14.65512097	14.93865735	14.34153839	13.77573494	14.52566081	17.86024441	13.62666879	13.82222193	13.12737212	14.54026863
MEOX1	0	0	0	0	0	0	0	0	0.021841946	0	0	0
MEOX2	0	0	0	0	0	0	0	0	0.024697704	0.02488586	0	0.023618262
MEP1A	0	0	0	0	0	0	0.01586899	0	0	0	0	0.032118669
MEP1B	0	0	0.012614127	0.012045442	0.024888947	0.012239304	0	0.011661778	0.012969714	0	0.012837587	0.037208571
MEPCE	18.0320798	18.42629486	19.70742219	19.90549325	17.25716005	17.82642923	20.23846177	23.57740344	17.51756065	11.67827654	15.13118529	14.94696234
MEPE	0	0	0	0	0	0	0	0	0	0	0	0
MERTK	0	0.034534124	0.023505069	0.033668078	0.023188939	0.011403314	0.022837461	0.010865235	0.954622845	0.87666425	0.885094113	1.999135266
MESD	10.44160215	9.791963241	11.23839744	13.98184438	12.07482877	10.89150355	11.58057298	14.21518009	14.54693051	11.85065592	13.5157945	18.92439937
MESP1	0.046307712	0.384055915	0.245063603	0.468030711	0.290121163	0.428006994	0.428585638	0.362498646	0.6551268	2.335801394	1.546310511	1.831289291
MESP2	0.066678514	0.034562653	0.070573459	0.067391783	0.034812143	0.034238202	0.034284491	0.032622632	0.108844344	0.40213638	0.395030198	0.173478621
MEST	0	0	0	0	0.01561612	0.01535866	0	0	2.392457973	2.968257854	2.883586702	1.21398391
MET	0.007653187	0	0	0.015470109	0.007991295	0	0.00787017	0	0.016657163	0.016784063	0	0.031858284
METAP1	22.5984776	20.92651939	23.05376964	21.94642656	26.29678678	25.00441971	24.23755478	20.10931775	15.86868095	17.14900269	16.21436726	15.06508425
METAP1D	3.347421588	3.33024606	2.695506322	2.934732117	3.233410247	3.407959438	3.025676982	3.33210536	2.204595551	2.168500671	2.244483326	1.817103028
METAP2	68.94254061	67.87651545	72.36869387	70.57972536	81.3634845	78.58774855	82.81335592	69.98364118	36.46111671	39.34746926	40.56988613	33.23836523
METRN	0.527060284	0.734226273	0.993666524	1.281805321	0.773922846	0.7442486	0.846880439	1.160395115	0.914132571	1.137825401	0.709662732	2.022610977
METRNL	17.48910895	19.91696676	17.28090721	16.38034304	14.16175081	13.969416	16.81068391	18.03451007	4.273052265	5.008561933	5.502693385	4.961927745
METTL1	18.56135808	16.88003136	19.48998868	21.65748176	22.79710272	21.96829705	20.75068795	22.29837128	8.599744036	6.085833693	9.422735977	7.458869722
METTL11B	0	0	0	0	0	0	0	0	0	0	0	0
METTL13	25.38067634	22.56252413	27.28116792	22.25210597	23.91278486	23.71317594	26.76617454	24.60330552	13.14853732	12.88501747	13.03160112	12.47524632
METTL14	10.9617676	10.65768673	10.40021246	13.68045603	11.7938525	11.47501925	11.28812513	11.08170804	11.35245895	9.662505872	9.638538791	12.08529978
METTL15	4.815242138	3.842409451	4.270643589	4.939389478	5.306709659	4.660393567	4.635019786	3.82765993	3.955280099	3.974154578	3.284607134	3.301595066
METTL16	9.100079477	7.652763443	9.785602059	9.234177132	10.80259788	10.4937921	9.750731559	9.224714761	5.451191019	4.914539036	5.591507483	4.597983584
METTL17	48.15630117	51.97926411	52.62963289	57.40656685	60.17365068	64.8048942	56.79616241	64.87489518	51.63305064	51.92904233	52.15915687	60.95809725
METTL18	3.177040553	2.982414689	2.938985925	4.19708879	4.597339172	5.112427362	4.579107337	4.528494533	2.722373643	2.605957956	2.721586271	2.525287395
METTL21A	6.085483543	6.869809294	7.143413367	7.347673632	8.017546977	8.504028823	7.465522649	7.643167015	4.233528642	4.982342806	4.52035298	3.591580229
METTL21C	0	0.005484625	0	0	0	0	0	0	0	0	0	0
METTL22	1.966723716	2.458322823	2.028080454	1.94232745	1.953864238	2.337143454	2.022484206	1.918950709	2.923020768	3.413578048	3.086932839	2.672462069
METTL23	12.35284014	12.41343967	12.69577057	11.14502477	10.56935868	10.91377848	10.73376741	11.88139994	10.78644469	10.98399773	11.31125934	14.4760548
METTL24	0	0	0	0	0	0	0	0	0.0180847	0	0	0
METTL25	0.628457624	0.566997702	0.6507862	0.782816821	0.769730764	0.596561769	0.677716071	0.631569216	0.698706476	0.305451938	0.556198166	0.597462999
METTL26	26.80344416	30.4659045	34.53633819	31.69907661	30.52710246	36.05979076	38.14062419	38.3741681	19.40913982	25.33521158	24.94208905	21.46097159
METTL27	0.827132447	0.857484364	0.29181613	0.650206969	0.383855157	0.896625715	0.472546267	0.494604792	2.150303206	0.856596383	1.534425972	1.243357276
METTL2A	7.449899645	7.19368025	8.268065957	9.680904221	8.134639359	8.284696913	8.47100869	9.039308106	7.574584675	7.445567807	7.451563959	8.173889902
METTL2B	20.19708082	17.10338103	20.59383339	21.2356387	18.84910018	20.28580537	20.00891267	21.18665035	13.63317809	11.89822397	12.53800372	13.75591649
METTL3	59.50453911	53.5494462	56.13595111	47.68788523	59.88256728	57.58347488	54.67837063	52.39252809	45.75768851	44.90985763	43.37094839	45.14253467
METTL4	3.196174079	3.555537998	3.39832164	4.897172762	5.120359192	4.871073782	3.541931006	4.626945695	4.81235473	6.129285429	5.47075478	4.73871886
METTL5	22.63018915	24.45591062	25.21019461	25.92190279	31.36340416	31.31582486	29.618654	28.36190022	23.78151816	31.28184332	28.06981401	22.59938568
METTL6	1.926284195	1.960280139	1.798001928	1.696502488	1.884683558	1.895148678	1.752133005	1.780987898	2.82805075	2.932755241	2.45614285	3.204296664
METTL7A	3.984129727	5.05183639	3.763251626	2.390380567	3.845232237	3.194999964	3.966503184	2.469562125	7.134088545	3.742165335	5.608094041	4.575721232
METTL7B	0	0.074082499	0	0	0	0.036693532	0	0	40.36087041	32.67572922	34.67695551	43.91414311
METTL8	3.853171137	3.548334649	3.787338001	3.595626991	3.573948255	3.589586457	3.807759239	3.136036016	1.794672875	1.882271387	1.63673662	1.915922233
METTL9	21.42059257	16.17003767	19.05254914	15.79713081	17.75206408	14.85870244	22.55870153	16.14626669	19.39654498	26.28312359	19.58118706	25.21293347
MEX3A	1.449800382	1.99489266	1.618188104	1.234411893	2.486385112	2.833406922	2.213768147	1.728157837	1.40562766	2.485814595	1.713107179	1.316760521
MEX3B	0.228652525	0.347663081	0.209741121	0.585449407	0.270587982	0.485290146	0.297838014	0.372896093	0.099532448	0.03343024	0.213456699	0.269683082
MEX3C	7.158152592	7.744053591	7.590037489	8.994167187	8.627427313	8.93879943	7.040471625	7.767006085	7.210215138	7.749488084	6.003276405	8.233541621
MEX3D	3.855258171	4.494800746	4.068055807	6.040164631	4.882084637	5.33109389	4.69963316	6.581616041	3.035031616	2.685521393	3.445893968	3.560905588
MFAP1	21.727273	23.71433579	23.76943514	27.6275602	26.2550043	25.07212274	27.9223993	28.35550333	23.38924082	20.87891094	25.17386645	26.92724559
MFAP2	0	0	0	0	0	0	0	0	0.049794436	0.050173788	0	0
MFAP3	6.99544422	6.907798085	6.434620259	7.833254736	6.831541173	6.739584546	7.142025035	6.254135706	9.913060726	7.6597523	8.532708713	10.4015912
MFAP3L	0.005128628	0	0.00542821	0	0.005355204	0	0	0.005018388	0.424173421	0.320553692	0.336986649	0.651149986
MFAP4	15.31144057	32.37242758	15.33455815	33.64059638	29.36841846	47.34195329	11.03317218	27.68239118	0.925704217	0.511511659	1.359632008	0.371231864
MFAP5	0	0	0	0	0	0	0	0	0	0	0	0
MFF	21.58197266	23.37090385	21.54074438	23.82459173	23.51625362	23.12854604	22.17086527	22.01531417	24.04636657	26.657421	22.21142592	27.99944942
MFGE8	0.034971552	0	0.024676248	0	0	0	0	0.011406614	1.357394607	0.971475834	1.004535592	1.067569995
MFHAS1	7.836651847	8.140666344	8.781339806	10.00481875	10.5598715	10.96411811	9.331284206	9.686151909	11.25589139	11.51559429	11.65385669	13.68605163
MFN1	9.293304169	8.439607648	8.92917027	10.30361514	8.716513242	7.935535374	9.268108843	8.153830745	9.550687725	9.267024197	8.530333103	11.40889153
MFN2	38.53883533	33.64106666	41.18481453	39.37242277	35.78543505	35.07152039	41.23344314	39.18107816	36.83021876	33.53806232	35.35604839	41.95636151
MFNG	49.04832163	44.50193978	51.80676689	37.68260746	41.92966152	44.91205809	51.70833103	47.13127292	0.137074572	0.414356561	0.135678145	0.209733704
MFRP	0	0	0	0	0	0	0	0	0	0	0	0
MFSD1	79.91834187	73.84440793	74.4045	68.91239876	51.57976611	57.45592903	67.73061969	60.26379117	72.12120079	56.88460604	67.05037317	76.30619182
MFSD10	33.51697223	35.14872414	35.86094619	31.17929369	32.23598532	31.70451733	32.66181731	38.12875191	39.15463121	38.50285445	46.34480288	48.47701285
MFSD11	2.829672278	3.0569243	3.082196129	3.813255525	3.050304419	2.924779206	2.693304631	3.333371228	3.009631319	2.801603207	2.692911011	3.516608862
MFSD12	28.28096041	27.26968393	27.37174522	26.60448515	22.06580763	21.94862704	24.33379679	28.37799433	16.90610854	14.36117453	17.33080806	17.28218677
MFSD13A	5.193018282	6.296222559	5.36026177	5.888394174	4.854374146	5.251775198	4.902858625	6.126710226	2.712625143	3.275610488	2.194873337	3.119056314
MFSD14A	23.01939587	20.66673993	21.79269089	24.38015883	23.83845923	23.35696616	22.02421187	21.66474957	40.44513624	34.72623761	37.36052176	38.44432889
MFSD14B	22.53865378	22.20354844	24.02695461	27.59510611	22.70267028	21.81333891	24.05744963	26.75946522	36.47049343	32.478285	35.54285392	43.9486972
MFSD14C	2.698078264	2.068267224	1.930602756	2.611717136	2.182396839	2.009826	1.738993623	1.375818042	3.391085508	3.500259495	3.172339007	3.341742198
MFSD2A	25.09329725	20.82136671	22.75022986	14.08165459	16.27462175	14.68285117	19.87865272	17.72545486	86.52756466	94.59896981	122.4974641	110.9856169
MFSD2B	0.264706616	0.20855929	0.156894714	0.171224458	0.210064773	0.239222764	0.250434644	0.31082012	0.103704115	0.139325559	0.250916466	0.165286006
MFSD3	8.27111435	9.594947418	9.114848925	6.886620013	8.181967863	9.018942235	8.155270961	9.148978752	2.698252436	4.628201013	3.792077693	5.200038443
MFSD4A	0.013162808	0.027291645	0.013931698	0.039910838	0.027488649	0	0	0	0.200541976	0.288671107	0.184320486	0.191777051
MFSD4B	3.953355492	3.870734975	3.966354412	4.536721972	3.511674914	3.481972772	3.712538049	3.089329978	4.302239294	4.590901576	3.400814245	4.799905615
MFSD5	7.06373431	7.193166905	7.230296596	9.272047687	6.759594917	6.684880996	7.245615202	8.329276797	9.088303771	6.510286568	8.398571883	10.81268203
MFSD6	1.651275395	1.129833889	1.479746977	2.225252398	1.149484424	1.040090464	1.505641843	1.486518844	3.402320991	2.812606224	2.430881598	4.536735667
MFSD6L	0	0	0	0	0	0	0	0	0	0	0.051751521	0
MFSD8	3.113753974	2.774074768	2.760098624	2.271785524	2.712816498	2.378298217	2.791774293	2.504108526	3.769753981	3.393018272	3.259690748	4.344218373
MFSD9	2.406631396	2.585485893	2.321249518	3.06008523	2.553484807	2.82032638	2.564677754	2.715732642	3.062559882	2.032432011	2.383276544	2.777222215
MGA	12.35008447	9.641100282	10.94326658	10.81158723	11.8725806	11.56309017	10.82958871	8.562197354	15.48912492	11.4215577	12.59179122	14.22250554
MGAM	0.011306905	0	0	0.022855713	0.017709644	0.017417669	0.029068695	0.011063864	0.116895029	0.322360524	0.146152646	0.335357978

MGAM2	0.063089445	0.267564003	0.042493015	0.017390269	0.029943934	0.070680612	0.070776169	0.039284854	0.137314182	0.163516706	0.123559377	0.125343943
MGARP	0.160745514	0.124983096	0.127601452	0	0.041961761	0.041269947	0.123977225	0.078645151	0.218664141	0.132198001	0.086574613	0.125464299
MGAT1	27.95529246	25.88334844	26.73445092	27.14539543	21.58499357	20.9773975	23.33439617	28.38232687	35.73196901	28.17106897	31.15598735	48.13961737
MGAT2	34.47702221	30.02177556	32.75152566	36.37045764	36.00017308	33.04212591	36.31179036	37.95050607	54.54061198	41.39665069	47.44264862	55.04871464
MGAT3	0	0	0.003221858	0	0	0	0	0	0.102693102	0.186923401	0.21968853	0.031678961
MGAT4A	5.978137942	8.326672775	5.511343722	6.751042068	4.810355397	5.704514748	6.082644408	5.602296308	7.214047171	4.462573938	4.213540172	9.61484323
MGAT4B	21.35101263	23.1990285	21.80296143	23.43991907	18.13616237	18.44158659	20.41104051	24.09021575	16.87110129	18.53756234	20.00400431	18.98775788
MGAT4C	0	0.009375483	0	0.004570182	0	0.004643736	0.004650014	0	0	0	0	0.023528949
MGAT4D	0	0	0	0	0	0	0	0	0	0	0	0
MGAT5	6.891466174	6.803078125	6.745685961	7.679702877	5.866057304	5.935608377	5.688350925	6.041013941	9.102928337	9.704861491	8.957876375	13.28225812
MGAT5B	0.067872806	0.063327081	0.05028626	0.048019195	0.170091217	0.069702899	0.048857994	0.033206943	0.376699181	0.215833357	0.453282748	0.275473897
MGEA5	49.19928985	46.94259397	41.34363613	50.64351096	55.45522321	46.11996478	49.98933346	45.06095329	48.16630789	42.92779269	39.46725232	42.41549772
MGLL	1.266857886	2.617242837	1.44697107	1.731777049	0.475836709	1.21677839	0.67481912	0.998836039	0.198368079	0.129921556	0.196347235	0.142273625
MGME1	8.120910263	8.312789219	8.649455372	10.8977256	11.95350091	10.51245249	9.631898277	9.832775771	6.813849189	5.369136157	5.549915823	6.320738106
MGMT	0	0	0	0.010980248	0	0	0	0	2.518253332	2.549351123	2.644729462	2.385577964
MGP	0	0	0	0.028135111	0.058134298	0	0	0	0.030293977	0	0	0
MGRN1	7.068382702	7.265659845	6.42882336	6.490137007	4.941285864	6.268552519	5.78422828	6.224803504	4.478406167	4.689872346	4.471497343	5.878544095
MGST1	43.76399888	49.56669637	48.98291841	52.34782947	36.54751205	43.96884784	49.08212259	52.53881981	12.69064395	21.68365088	14.78651511	24.01191173
MGST2	14.40283083	15.11325254	15.12034438	13.64062789	11.20682342	10.85687814	13.32254314	11.83260101	12.47545477	12.36205778	13.48239629	12.94974536
MGST3	44.70761959	56.94101858	46.69168396	51.81218266	38.67166568	46.80843768	38.51585891	54.3249915	16.96405762	26.72978462	21.82485407	19.92442998
MIA	0.240938332	0.083259883	0	0.324687574	0.25158268	0.164956593	0.247769408	0.392932301	0.262201151	0.264198691	0.778590049	0.668643568
MIA2	4.917702674	4.060427634	4.761464411	4.682088925	4.873654824	4.50642286	4.919479558	4.185583206	5.218689576	4.014033254	4.488490138	5.172297167
MIA3	13.05945098	12.35254069	12.66636706	16.04339112	15.00004096	14.49756232	14.19627535	14.03399572	17.09835706	16.29717041	14.65848498	17.90256893
MIB1	10.26059703	9.291570481	9.340586774	8.795445056	10.18577461	10.27373236	8.547522303	6.361000671	11.75697953	12.70700328	10.23513393	10.33361393
MIB2	3.594519681	3.667366036	3.689932475	2.826923331	3.253671274	2.995273638	3.690573339	3.483811158	4.46347079	4.441256634	3.743027655	5.786039092
MICA	5.766912903	7.979130001	5.082525104	8.096540947	5.646796693	8.203804525	5.353527501	8.716936007	7.423565533	7.504726512	5.583466866	9.224172244
MICAL1	72.81319058	61.95568149	72.36607832	53.80689592	65.58919709	67.3478095	61.94737022	60.35758467	110.2525969	101.2037054	113.5462438	124.9413406
MICAL2	4.127437907	4.701502681	4.122844438	3.32468255	4.69405704	5.70978223	4.593797081	3.22432705	5.594580957	4.097525996	4.616688728	6.557951853
MICAL3	1.667778715	1.479325642	1.640470038	1.675262049	1.631781907	1.718009876	1.736139575	1.689586445	2.76844169	2.354107108	2.646413722	2.935383147
MICALCL	0.178177354	0.434081742	0.198014637	0.162075006	0.325585754	0.384261467	0.238197743	0.130760584	0.329632573	0.302837021	0.220715097	0.417210339
MICALL1	7.939601199	8.067147715	7.452254188	7.61532091	6.001232667	6.480351078	7.281210382	7.208494133	7.705316629	6.518743505	7.116237796	9.115621815
MICALL2	1.211927046	1.353045225	1.460327726	1.479291661	1.119452849	1.091422778	1.332569012	1.523395535	6.685705371	6.961534811	4.830141972	12.6608737
MICB	11.00370685	13.40433389	12.31880418	13.77232944	11.27587314	12.79450069	9.819569014	12.05042267	7.670997879	7.504745252	8.012223316	7.804873181
MICU1	17.6474243	21.71554282	19.69600391	23.13370521	20.17921114	19.9820579	17.85693399	23.20861382	25.21657251	29.13005027	26.68594205	33.94467239
MICU2	31.15290366	32.79463758	32.60504312	40.26230704	42.85150084	40.11459306	36.02006211	36.73156122	23.34770627	22.47088139	21.81478221	21.35409846
MICU3	0.006100166	0.006324013	0.012912999	0	0	0	0	0	0	0	0	0
MID1	0.011827577	0.006130797	0.006259235	0.023908196	0.03087526	0.036439472	0.012162912	0.034720032	0.193070416	0.285327231	0.267544956	0.203095248
MID1IP1	26.47117631	31.43660015	25.86574136	24.29731046	21.95515857	25.73954781	19.82417111	24.98405793	21.52035286	22.68199859	22.95497854	18.65643631
MID2	0.00423931	0.004394873	0	0.004284658	0	0	0	0.004148186	1.102609583	1.106361073	0.853922523	1.222066886
MIDN	18.99160972	18.37839479	17.72586565	15.10035959	15.53893026	12.21406536	17.8175354	17.03477093	9.845396161	6.83808377	10.58475361	10.27547836
MIEF1	4.261910301	3.759218791	4.261645598	3.938626147	3.958461691	3.893199356	3.698696814	3.502131104	4.573962974	3.969772478	4.717591896	5.090808985
MIEF2	1.478376945	1.741621033	1.422485952	2.17336921	1.543689753	1.811535482	1.399359537	1.758933291	1.535714516	1.289511772	1.321012915	1.695876737
MIEN1	14.47218249	12.9439755	14.0410944	16.45536743	12.2225732	11.94820726	13.49638241	19.54093517	16.79158986	16.02491907	18.64555793	19.34306064
MIER1	6.194915911	5.826433973	6.068738991	7.173006589	6.475577388	6.10802386	6.294959485	6.035600204	12.11600301	9.870701968	9.991411411	11.61427733
MIER2	5.97538212	6.283376345	6.357366565	4.380066355	5.873778978	5.56919312	6.200803225	5.161753239	3.615438948	3.523540573	4.064694372	3.651750054
MIER3	6.694687729	7.003065681	6.637554801	7.856654486	9.980341972	7.651766701	8.097576562	6.639576684	13.67127381	10.67982492	11.66401906	12.68552908
MIF	235.7645287	279.3183562	296.1341588	273.7677649	308.6768509	319.249815	288.1176923	356.5569959	216.3837199	273.6709128	284.1259983	130.4644577
MIF4GD	6.785930411	7.748345145	6.716988772	9.292812112	6.906086084	8.146746112	5.641631681	9.988157629	15.04000705	13.70829869	12.58067529	20.37046977
MIGA1	0.663736173	0.660290501	0.702507519	0.697940738	0.86107354	0.85376238	0.820444179	0.603547147	2.130452412	2.220199483	1.819879058	2.637376496
MIGA2	6.401189882	7.031611979	6.834932	8.754755344	7.141389322	7.212302497	6.975021161	9.443789403	7.796490633	7.809402487	6.925571209	9.10276218
MIIP	14.18139886	13.25486541	15.8275446	14.7109488	13.89705904	13.87270428	15.7111894	18.24195942	9.981212876	12.7902025	11.86617555	13.04652437
MILR1	10.22505785	8.058778826	8.24403005	3.512767522	5.751532155	3.202812045	7.275904427	2.884676004	21.15729415	19.75319453	18.03364425	17.72975517
MINDY1	5.142210594	6.07977117	5.351876832	6.06342039	5.407739663	5.708361363	5.01101179	5.175441419	12.60434839	8.457964924	8.717334726	10.49899719
MINDY2	2.886533698	2.986530416	2.413868679	2.24149622	3.587026341	3.081765535	2.715620093	1.896042352	5.971523983	6.499632059	5.664411235	5.597510496
MINDY3	7.578811389	7.044593072	6.090915737	6.400546221	5.599901749	5.032672462	5.944336487	5.071725132	6.094867529	6.655422984	5.776799064	6.563728653
MINDY4	0.393776516	0.24493577	0.562650968	0.397988793	0.472849019	0.545932082	0.566917197	0.597234094	1.007039181	1.424913541	1.166444823	1.495762757
MINDY4B	0	0	0	0	0	0	0	0	0	0	0	0
MINK1	17.85471176	20.11069534	16.9169892	19.52367607	16.10218402	19.65936496	15.74244514	18.89181465	12.92020837	15.25008535	12.21579805	15.64393411
MINOS1	4.706810592	5.006087049	5.77136831	5.620851809	4.900587932	4.624772434	5.844576592	5.335638313	4.974573388	5.443811699	6.238882647	4.968896494
MINOS1-NBL	0.146089306	0.075725052	0.077311468	0.073826021	0.101695563	0.075014197	0.075115613	0.023824854	0.026496949	0.026698812	0.026227015	0
MINPP1	9.767465144	9.525338962	11.66646387	12.62048171	9.997352029	11.08844728	10.64010555	12.72235828	5.638683844	4.764109173	4.853252699	8.691216656
MIOS	6.61452137	8.077298592	6.728138277	6.693959928	7.606386532	7.392762497	6.978732858	5.690618211	8.142439639	7.733491593	7.939198873	7.411093093
MIOX	0	0.013313633	0.013592549	0	0.013409737	0	0	0	0.041927153	0.028164379	0.013833342	0.040094677
MIP	0.090833155	0.018833262	0	0.018360962	0	0	0	0.035552281	0.19769837	0.01992045	0.136979043	0.094528864
MIPEP	10.52944393	11.41199948	12.23001276	14.080023	14.9034234	16.37802028	12.16389332	14.70200889	2.697251358	2.980208234	2.614536823	2.330322664
MIPOL1	0	0	0	0	0.002192142	0	0	0	0	0.002302071	0	0
MIR1-1HG	0	0	0	0	0	0	0	0	0	0	0	0
MIS12	7.873085906	8.187221841	7.598855941	11.86831472	9.669415142	8.860168504	8.484225197	10.31149614	2.516071651	2.708703752	2.516654704	3.24190821
MIS18A	6.293379075	8.779935676	9.211493324	15.88992752	12.50766996	15.25669182	10.73021081	15.29224179	8.630977889	14.16104984	12.24755999	10.63979078
MIS18BP1	14.18331516	14.41783485	13.32509543	21.74415557	20.75564278	19.98541473	14.6988964	16.45141615	21.464913	17.97889297	19.07408154	19.44875755
MISP	0.016889379	0	0.017875951	0.017070047	0.01763553	0.034689553	0	0	0.165418805	0.055559675	0.145541001	0.017576553
MISP3	0.579487579	0.858217257	0.700957308	0.460182196	0.561867986	0.425080451	0.170262055	0.810045051	0.765761823	0.862371625	0.445859259	1.464586585
MITD1	16.40783958	18.24253475	18.91235036	20.49744044	16.74253091	15.28035407	17.32717378	18.51495017	14.12200391	14.11783957	12.88037859	16.368365
MITF	1.915939976	1.888269807	1.745957849	2.266410507	1.668648339	1.914660627	1.970260648	1.723431312	1.963473413	1.808851968	1.897197709	2.092246899
MIXL1	0	0	0	0	0	0	0.054517407	0	0	0	0	0
MKI67	38.24918823	36.86566105	41.28968861	99.12016042	85.30100277	76.54054583	37.17635238	79.50153735	77.10539919	73.93190039	77.05703555	63.28172308
MKKS	16.50249493	18.27531032	18.0356403	20.31376066	20.44272967	19.34633793	19.92549924	21.24534147	5.577847721	6.283726313	4.941769238	6.313429881
MKL1	18.97112542	22.17866178	19.10978945	19.55662506	22.6470425	23.58753957	17.95201141	21.74118523	19.95330594	14.31113413	18.20069254	20.83808208
MKL2	0.893688065	0.955109525	0.786471811	1.012348135	0.665801113	0.786304636	0.655709477	0.670597128	1.617284263	1.483711583	1.311473118	1.36111156
MKLN1	6.568717883	5.855525517	6.125806715	5.774459802	6.820082253	5.848299071	6.066551018	4.398737079	12.59698143	11.92321655	10.56079767	10.46441626
MKNK1	9.721954765	8.610316991	8.840506062	6.144786867	5.527778615	5.62994615	7.14735837	5.318245056	13.90854623	10.3818803	10.39103564	16.47400924
MKNK2	33.44127088	39.47119942	33.94340677	39.43664749	30.76234175	32.4562911	38.07201927	43.57348967	23.45517521	26.36365662	23.73246402	35.41147586
MKRN1	22.82443458	23.76773716	20.9861552	22.24379171	20.42430073	20.38875213	21.68823878	19.81347161	24.15875013	24.04917627	22.86877644	24.88102733
MKRN2	15.41734609	16.92327287	15.31805845	18.61851804	15.21068179	16.00768199	15.31043269	16.08428639	14.84517612	13.77735567	14.30726042	18.6400829
MKRN2OS	0	0	0	0	0	0.019288118	0	0.018377985	0	0	0	0
MKRN3	0	0	0	0	0	0	0	0.038800979	1.31615832	2.06537051	1.238680476	1.464967508
MKS1	6.883745624	7.498190052	7.675798273	9.093592155	8.038255516	9.558565806	6.799744954	10.37879344	7.849971758	7.697147212	7.394033548	8.172812263
MKX	0	0	0	0	0	0	0	0	0	0	0.012851819	0.012416607
MLANA	0	0	0	0	0	0	0	0	0	0	0	0
MLC1	42.47309604	35.01698997	45.60858354	29.41251996	42.85638986	45.24557536	42.12732436	35.83726963	62.66588678	55.3739879	69.57624662	39.81726827
MLEC	27.61140947	29.30236158	27.79462607	35.179829	28.55016175	30.37498674	28.72050432	33.01979246	53.85539186	57.64970897	48.51198871	64.19813014
MLF1	0.040780266	0.063415068	0.021581198	0	0	0	0	0	3.883173545	6.506355792	4.678227692	4.031750958
MLF2	48.08118967	54.02454368	49.17756178	52.05322692	44.00237704	46.92645151	56.34423698	55.45554846	27.18673147	30.88351186	29.25223487	30.80077432
MLH1	17.66957398	18.47694468	20.59533763	22.31892923	24.51722879	23.6230586	21.39462951	22.75846965	19.303086	19.87987787	19.91401155	18.86726674
MLH3	0.061162673	0.077497517	0.050349769	0.020605646	0.007096085	0.027916372	0.027954114	0.013299552	3.180102366	3.711060675	3.70404421	3.415946974
MLIP	0	0	0	0	0	0	0.00972584	0	0	0	0	0
MLKL	12.69791754	13.43934925	12.97760025	12.94884967	8.958177418	9.395252568	14.34615464	12.57355643	10.12118023	7.159613678	8.709589333	11.65409182
MLLT1	25.57964183	25.25257972	26.96878613	30.42800523	29.1143179	28.96051702	28.51991497	35.0728139	23.49459677	22.21507619	21.88666434	26.70303514
MLLT10	10.06832825	8.452309612	9.35661016	8.426256265	12.21353897	9.995331285	10.0032809	7.623186888	13.78291572	10.35216501	11.6203134	10.73133935
MLLT11	0.623373041	0.516998348	0.448654904	0.352823151	0.260365147	0.332894329	0.487195639	0.317186315	0.705521169	0.738238233	0.698333779	0.441140538
MLLT3	2.279609277	2.658667887	2.477979894	2.708750347	3.256856082	3.38506888	2.827344344	2.833476607	1.785159404	1.536968102	1.39366913	1.763239981
MLLT6	24.73804878	28.51684301	22.65201955	28.29479038	28.64777573	33.06049035	24.70161894	30.117475	18.28579174	23.09824574	19.10417753	23.138745
MLN	0	0	0	0	0	0	0	0	0	0	0	0
MLNR	0	0.045023504	0	0.043894406	0	0	0	0	1.465137989	1.571545079	1.918022478	1.807874054
MLPH	0.762580136	0.419986744	0.496045778	0.024085549	0.082944787	0.097892752	0.792369543	0.038863986	0.138312978	0.087104184	0.051338976	0.00826674
MLST8	9.70805808	11.48933214	11.41177337	10.11581502	10.89946147	11.85203023	10.41031498	13.12820485	5.033080636	5.982082657	8.806847469	4.649190111
MLX	38.0993534	34.18464913	38.76879414	33.64578151	34.73844224	33.79903926	36.58773395	34.71147382	32.4104639	33.27920411	33.07572629	31.29993097
MLXIP	17.60466787	17.97502611	15.40644072	15.8555703	13.72708962	15.0883532	14.73357665	15.50786662	7.382028039	5.939764644	6.174500034	8.693922355
MLXIPL	0	0.013615846	0	0	0	0	0	0	2.972935677	4.666198997	3.437806685	4.742889406
MLYCD	6.398312248	4.625380478	6.253129975	4.53416212	4.761159246	5.085472725	5.445283035	4.701582666	4.535263645	3.333276729	4.383436359	6.020838326
MMAA	1.505104391	1.241371276	1.469805977	1.41194689	1.615012293	1.48590941	1.547777065	1.26933346	0.82348963	0.738580486	0.680743236	0.830767159
MMAB	8.498232136	9.758554505	8.884833542	10.12393498	10.10475993	10.88742426	9.728514177	10.90898533	6.240739754	7.167402528	8.351362035	3.082147982
MMACHC	4.808144632	4.51671437	4.758313019	5.263080832	4.78494027	5.276482495	5.3728663	5.231323201	7.026990848	7.404097297	7.946362598	5.979237263
MMADHC	60.06300322	60.24840423	64.40380628	63.39259388	64.78881373	62.72081275	69.6599295	64.37800631	73.59513727	65.98471091	72.92606835	68.50804743
MMD	3.133795448	3.050694155	3.59999825	4.828228746	4.030445072	3.708887311	2.908240357	4.393976618	11.37477508	11.92240377	11.64997311	13.76109304
MMD2	0	0	0.023095207	0	0	0	0	0	0	0	0	0
MME	0	0	0.005405541	0.010323684	0	0.005244918	0.015756027	0	0	0.005600263	0	0
MMEL1	0	0	0	0	0	0	0	0	0.017777249	0	0.017596146	0.017000273
MMGT1	5.786726549	5.854017229	6.124751184	6.969867447	6.386760914	5.624578621	5.98162476	6.112199146	8.396540574	8.59201885	7.66506928	10.1929645
MMP1	0	0	0	0	0.027341508	0	0	0	0	0	0	0
MMP10	0	0	0	0	0	0	0	0	0	0	0	0
MMP11	0.046974737	0.024349246	0.049718709	0	0.02452501	0	0.024153281	0	0.357841815	0.463587402	0.632493493	0.342201913
MMP12	0	0	0	0	0	0	0	0	0	0	0	0
MMP13	0	0	0	0	0	0	0	0	0	0	0	0
MMP14	58.15024866	45.74453151	62.00644952	35.97438969	56.32746099	37.50640303	56.30488014	36.0148959	62.95623464	50.69944711	58.31640671	68.56944442
MMP15	0.050644293	0.065628378	0.013400654	0	0.013220423	0	0	0.012388924	3.251705569	2.138040859	2.291192051	4.400854638
MMP16	0.026835009	0.025037757	0.031242798	0	0	0	0	0	0.309553922	0.42078723	0.367102352	0.33791476
MMP17	16.6672908	19.3602661	15.91893437	13.32952669	11.42169724	13.57960449	14.8616485	17.42678136	4.407408219	4.877492508	5.238738735	7.799139015
MMP19	0.682137711	0.723614786	0.184693571	0.112233544	0.198774052	0.048874226	0.473089584	0.062090717	1.829945533	1.287241657	1.349933546	2.476366399
MMP2	0.130479052	0.094686918	0.193341159	0.026374961	0.08174607	0.174196402	0.322028135	0.421325566	6.105734854	5.508410311	3.949378935	12.08511638
MMP20	0	0	0	0	0	0	0	0	0	0	0	0
MMP21	0.056080835	0.087208111	0.059356729	0.028340369	0	0	0.028835418	0.054875381	0.030514985	0	0	0
MMP23B	0.025004443	0.051843979	0.026465047	0	0.052218214	0	0.051426736	0.195735793	0.081633258	0.054836779	0.080801631	0.156130759
MMP24	0.059092424	0.024504336	0.012508847	0.035834726	0.01234061	0.012137153	0.036460686	0.023128894	0.025722933	0.01295945	0.038191327	0.012299341
MMP24-AS1-	0.57668944	0.597851263	0.375616035	0.381099698	0.625327108	0.52390377	0.410565961	0.564293543	0.506893404	0.559398438	0.382270098	0.761732754
MMP24OS	15.04843651	15.85277583	14.73693437	16.80716792	10.38027297	13.48729836	13.31795566	16.56931134	9.164201812	11.96755389	8.743375637	9.61788997
MMP25	0.955620389	1.960295983	1.205122144	1.253540633	0.689994693	1.158872265	0.773626001	1.143980117	0.354026871	0.289838227	0.525630413	0.359713312
MMP26	0	0	0	0	0	0	0	0	0	0	0	0

MMP27	0	0	0	0	0	0	0	0	0	0	0	0
MMP28	0.015391751	0	0	0	0	0.015806767	0	0.015060906	0.033500147	0.016877681	0	0.016017992
MMP3	0	0	0	0	0	0	0	0	0	0	0	0
MMP7	0	0	0	0	0	0	0	0	0	0	0	0
MMP8	0.033349588	0.034573363	0	0	0	0.017124406	0.06859023	0.032632741	0.036292691	0	0	0.017353238
MMP9	3.455237213	2.961143446	3.827734017	0.279375866	0.144315407	0.118280091	2.842559994	0.293017152	0	0.025258722	0.049624746	0
MMRN1	0	0	0	0	0	0	0	0	0	0	0	0
MMRN2	0.8341841	1.107714662	0.783708365	0.454709649	1.262514723	1.135818518	1.069883929	0.476912084	4.539004423	8.006341686	3.765844882	4.721035889
MMS19	20.07772206	21.33935382	20.88212828	18.21367452	20.2295604	21.69960083	19.53245194	21.1401275	11.66516294	14.22628255	12.34732558	10.80131656
MMS22L	3.6431543	4.150962189	4.242470655	6.061612404	6.401481968	6.666551129	4.387051633	4.70408198	7.153224216	7.947335691	7.496843108	6.634919431
MN1	0.056971478	0.125506891	0.090449097	0.107964204	0.074360506	0.153582534	0.05126339	0.06271524	0.077499017	0.085898375	0.015341901	0.111167745
MNAT1	5.983954587	6.41032251	7.697638534	7.226543646	7.898514892	7.831322472	7.589454163	7.101464242	7.914631836	8.950763374	8.28024338	7.329197196
MND1	12.59615604	15.38600245	16.43333435	20.50020853	30.19941086	28.99806242	20.07563021	22.45383011	21.41062157	27.08192288	25.29163448	16.79174901
MNDA	6.611933583	16.26773651	7.094910468	15.05908689	1.622608567	9.700306996	5.828052789	14.66887924	9.052323997	11.79419261	9.682164541	27.17500357
MNS1	2.02151588	2.399020557	2.927874076	4.381998261	5.471477672	5.326638369	4.814132252	4.997213168	5.412948132	4.695849862	4.985336845	4.428978653
MNT	7.50707264	6.065357999	6.993871671	5.393076306	7.270433376	6.427612705	6.296382889	5.968026506	2.620185865	2.328778627	2.523398708	3.05358991
MNX1	0	0.021340521	0.021787598	0	0	0	0	0	1.926553216	2.415251743	2.173009548	1.628124067
MOAP1	10.41906267	9.8551179	9.30755011	7.492867664	6.834471989	5.83012699	8.860037223	7.537407199	8.86732399	9.374298614	8.321353401	13.0208445
MOB1A	72.35986508	71.6772514	71.24109151	65.5833662	54.50580915	57.2054884	71.56566295	57.85232778	44.43969411	47.91653631	40.9721468	53.62272343
MOB1B	9.290382948	8.126991882	8.711474996	7.442437193	7.651262609	7.049001093	8.705521544	6.085988023	10.05128861	11.98969943	9.741353798	10.92783703
MOB2	7.235929552	6.643237285	6.847314447	7.995084615	6.403053975	5.604764459	6.842012211	8.400467197	7.407369269	7.665525556	7.397961037	7.530336254
MOB3A	82.34155668	85.90230947	79.94780098	55.39146694	73.81402957	65.10387082	75.46121485	62.99157291	38.47282341	34.65957035	41.87456967	35.16749405
MOB3B	0.964861091	0.948971266	0.689543253	0.658456447	0.774990326	0.813027436	0.746282724	0.685900216	0.251284475	0.379798272	0.213192473	0.583590057
MOB3C	3.875730514	2.896662936	2.785630174	1.712631775	1.675251282	1.61060633	2.409906809	1.710999681	5.983339499	6.266126187	5.514614351	6.922476942
MOB4	0.013633028	0.014133297	0	0	0	0	0	0	0.014836143	0	0	0.028375424
MOBP	0	0	0	0	0	0	0	0	0	0	0	0
MOCOS	0	0.018386329	0	0	0.01851905	0	0	0	0.443915594	0.29171576	0.210144608	0.553713568
MOCS1	0.011652135	0	0	0	0	0	0	0	0	0.012777041	0	0
MOCS2	5.27670891	5.843923775	5.489588822	6.80302396	5.536704386	5.432204848	6.021885048	6.485591506	2.451015304	2.596700521	2.1765098	2.625157659
MOCS3	3.916630332	3.584815689	3.510532093	3.441422144	4.089662064	3.333745697	3.519679531	3.815179397	4.684660565	3.21138562	3.952798151	5.416286937
MOG	0	0	0	0	0.014793786	0	0	0	0	0	0	0.014744312
MOGAT1	0	0	0	0	0	0	0	0	0	0	0.052884766	0
MOGAT2	0	0	0	0	0	0	0	0	0	0	0	0
MOGAT3	0	0	0	0	0	0.029238338	0	0	0.185899229	0.156096228	0.337343249	0.059258094
MOGS	22.98416403	26.4750845	23.5082503	27.22893802	24.54416638	27.40859643	22.45217039	29.21252039	25.05166078	27.27374657	21.1157276	33.20255566
MOK	0.144313144	0.159582683	0.224023098	0.311161344	0.311423343	0.296408682	0.316596705	0.291836363	0.492086194	0.327040158	0.321261007	0.470578982
MON1A	4.745269094	4.084028876	5.401511704	5.067503231	4.487464782	4.459454643	5.501291648	6.307838335	1.705107319	2.626234632	2.338720988	2.865168285
MON1B	15.18036831	13.92928849	15.451775	15.22850009	14.18160194	14.90970956	13.65111284	15.3281334	16.78365411	14.6979684	15.49936313	18.70551453
MON2	8.214102485	6.889746638	7.48590211	7.779690506	8.342390644	7.623405224	7.629090786	5.768738368	10.27397896	9.287953707	8.097697722	9.923145164
MORC1	0.023243871	0.096387251	0.03690245	0	0.036406133	0.047741217	0.02390288	0	0.050590287	0.063719627	0.062593632	0.024189589
MORC2	8.990491825	7.670201496	9.045054178	7.891287065	9.749816322	8.666695617	8.79729491	8.15332901	14.19682087	14.44149371	14.42576859	13.52080502
MORC3	13.99003778	13.52799787	11.80786224	14.62648581	13.34494947	12.48076552	13.00445557	11.91376625	13.40842689	12.27006036	11.57062319	13.94144109
MORC4	0	0.013067257	0.026682025	0	0.013161583	0	0	0.02466757	6.447032284	8.486457215	7.915594573	7.778717969
MORF4L1	69.51128543	70.53758788	71.56688081	75.51956251	79.53073488	77.22130778	73.76313717	75.0895629	110.3629217	131.8101424	123.1988226	103.5940722
MORF4L2	66.33420003	76.84237453	68.27098376	69.58537906	69.57067761	71.05632467	70.54982838	66.27006496	91.90245516	134.6873899	107.6920038	83.28692849
MORN1	0.345959791	0.318804363	0.325483213	0.336710176	0.394692359	0.322391055	0.210825738	0.344792363	0.599596393	0.512837162	0.483071706	0.593392308
MORN2	4.502284445	5.269755086	6.994201213	5.798148879	6.369353691	6.040616928	7.691663011	6.537205676	9.325066577	8.759083911	10.63804548	9.068647585
MORN3	0.130923506	0.305387528	0.242499677	0.099243008	0.102530654	0.201680506	0.033658861	0.128109315	0.142477511	0.215344432	0.282052086	0.068125181
MORN4	2.193167658	3.35063727	2.138020035	2.722175046	2.229794297	2.825257624	2.571888389	3.049615441	0.314041422	0.232051526	0.207228115	0.040042116
MORN5	0	0	0.022808483	0	0	0.02213074	0.04432132	0	0.023451445	0	0	0.022426471
MOS	0	0	0	0	0	0	0	0	0	0	0	0
MOSMO	2.082169734	2.208775122	2.165358839	2.398086174	2.174157337	2.287497048	2.35283391	1.954495652	5.66480327	4.115040731	3.272978092	6.147910604
MOSPD1	4.043940388	3.891448978	3.788660389	4.048086983	3.232609774	3.934401581	3.720847319	3.843417637	5.264136737	5.283023809	4.689458225	5.557603768
MOSPD2	13.10662856	12.10018302	10.94456523	12.64589075	9.906584241	10.91138529	10.26153005	10.02973148	11.30935351	13.43649937	11.33337179	14.7025694
MOSPD3	8.651294608	10.12850997	8.288346551	10.55290867	6.658310856	7.161265247	7.36268346	10.98304326	14.56854767	16.43780907	14.29963029	16.84235765
MOV10	18.78617163	16.99080856	18.68360473	13.18871099	17.96356783	15.15911901	18.65371044	14.65228548	30.24792323	23.16956272	26.53846104	26.15446307
MOV10L1	0	0	0	0	0	0	0	0	0	0	0	0
MOXD1	1.182821712	2.434149667	1.289285404	1.623704181	0.866397485	0.815853227	0.871419967	1.295593709	2.132534952	1.645463195	1.996712232	1.451415029
MPC1	17.4320275	17.80552955	17.43481678	24.01032808	15.74697822	16.79185205	16.49493829	23.43068533	13.19104887	14.16579153	13.37689745	17.87408348
MPC1L	0	0	0	0	0	0	0	0	0	0	0	0
MPC2	16.04960703	16.45867678	15.5409242	18.50104083	17.21159491	17.84104285	16.90610933	17.8905355	13.68955627	16.40992273	15.88631942	12.57210264
MPDU1	17.65455673	14.79467325	18.68582632	20.90464391	20.40048064	17.82233856	18.04285646	24.08364166	15.32254597	11.16056371	15.34280391	13.26140251
MPDZ	0.018170721	0	0.032053569	0	0	0	0	0	0	0.01328329	0	0
MPEG1	1.705400533	1.211623211	1.312741413	0.49419147	0.149432975	0.306186051	0.735839998	0.268399236	2.777364143	1.961581594	2.569224453	2.5070429
MPG	31.37088126	34.18820095	31.43688847	26.48973175	24.72090049	26.91606483	30.03545873	29.66830711	30.0022776	26.90951938	27.19971964	33.99818269
MPHOSPH10	22.93131789	19.91167644	22.91862427	26.76581939	26.18282206	25.32863674	28.25767728	29.07034916	15.52913135	14.57894803	17.16692712	19.67285259
MPHOSPH6	16.18158017	16.85563389	18.60184368	19.52388427	18.75588098	17.29374063	19.86492717	20.49297426	13.94444826	14.22047871	15.55375213	15.51048637
MPHOSPH8	26.04123068	26.1841245	24.5546478	29.66238652	26.13012283	27.68683572	25.36877489	27.66286332	18.83515075	15.62078019	15.84612021	20.91832301
MPHOSPH9	16.5463075	15.23097204	15.33374925	18.0773883	19.86178435	18.98242226	14.45455152	16.77576068	5.1729618	5.735268346	5.079496607	4.316696661
MPI	10.75509436	13.00510408	9.543244	6.925194101	10.6073272	12.23536919	9.35982884	7.972157028	11.33325786	17.84896342	13.86160477	9.188289388
MPIG6B	0	0.010806688	0	0.010535678	0	0	0.02143943	0	0	0.057152629	0	0
MPL	0	0.011756401	0	0	0	0	0.011661785	0	0.037023134	0.062175315	0	0.047206659
MPLKIP	20.24291663	22.46778121	21.84904788	19.59253151	22.0328679	21.96324283	24.9331681	21.20665232	19.22901321	21.94636665	18.35556609	21.18555578
MPND	10.61902619	10.6047063	9.589514776	10.43722608	8.172008733	10.83865361	9.049988229	11.37582881	10.8140173	9.151553535	9.584494148	10.94969416
MPO	4.1173101	10.08104968	5.774994309	5.700715962	2.953520675	10.72551358	2.960388453	7.697317671	2.349615913	4.019271534	3.173020169	0.992826511
MPP1	28.38656397	34.30329923	29.50034826	34.70551041	26.11138889	30.5605176	24.78370742	33.14474277	20.01695542	22.93570127	18.91604075	22.14976927
MPP2	0.082369214	0.155257784	0.079255192	0.105954962	0.172016361	0.069210148	0.053902891	0.117234463	1.263085144	1.215230643	1.508327106	0.989682738
MPP3	0.046414227	0.064156552	0.065500611	0.015636909	0.064619665	0.095331441	0.095460324	0.015138852	2.677045104	2.86709007	2.933082186	2.093115828
MPP4	0	0	0	0.027010265	0	0	0	0	0.01454141	0.029304383	0	0.055623441
MPP5	1.521833256	1.413526052	1.424517826	1.449204264	1.855441119	1.626104715	1.474518941	1.489121563	5.916135829	5.710411904	5.249433354	6.289233857
MPP6	2.873269367	2.167431687	3.022564582	2.526248335	4.140529224	3.604464467	2.984759978	2.60007406	1.823987386	2.382203957	2.226876828	1.574095376
MPP7	0.198559265	0.300191307	0.11383551	0.476622788	0.07774926	0.152934848	0.127618007	0.226673123	0.216082129	0.244944358	0.151498918	0.611304092
MPPE1	5.335697934	6.198235744	5.874279789	6.1511335	4.750632376	5.210481485	5.609452609	5.535666435	9.046848922	7.078435347	7.88987334	11.89883641
MPPED1	0.030052813	0.077889028	0.063616623	0	0	0	0.030904869	0.014703415	0.114467411	0.164770663	0.307532077	0.125102259
MPPED2	0	0	0	0	0	0	0	0	0.598768943	0.813479426	0.586009872	0.598333823
MPRIP	10.99719453	11.21726373	10.44220026	13.46125868	12.83462149	11.56813159	10.37400574	12.22773087	10.32114772	9.315278499	9.801082613	12.27165344
MPST	20.08329482	29.32277232	20.11571123	26.99332257	23.22131492	28.02166878	21.62585945	31.63805659	14.27482671	14.47005175	15.91327673	19.91558298
MPV17	19.53189587	17.92968519	18.37143828	15.42134019	17.34144342	17.74854022	17.15570114	16.45862553	11.79052691	13.18211986	10.74162553	14.09726111
MPV17L	0	0.021356861	0	0	0	0	0	0	3.138650838	2.84630596	4.105250833	4.159183145
MPV17L2	19.38726825	14.19213684	20.93852254	26.39280241	16.60815665	15.80611646	20.42518697	27.2169181	7.621184927	7.982944765	10.73996274	13.3820898
MPZ	2.528233159	3.73545173	2.317723249	1.971789317	1.538225342	1.962635598	2.292837145	2.162217922	3.336282496	4.169380503	2.787651304	3.874137655
MPZL1	6.906097332	8.484531084	7.366347761	7.739845352	7.356993992	9.276256618	8.670280795	7.199053074	2.793497692	3.662746591	3.459191496	2.984372453
MPZL2	0.016878783	0	0	0	0	0	0	0	0	0	0	0
MPZL3	0.136140571	0.18818173	0.216139587	0.091731256	0.035538772	0.034952852	0.081666914	0.011101187	0.96300739	0.821060238	0.892094532	0.920918007
MR1	10.98497091	9.618912731	8.405317455	7.646110606	7.162782045	7.210285829	9.002587557	6.06143088	15.99037435	13.58522491	13.12081018	19.80832568
MRAP	0	0.041598898	0	0	0	0	0	0	0.043667604	0	0	0
MRAP2	0	0.012838693	0.039322979	0	0	0	0	0	2.06200537	1.792537955	1.800881472	1.043938045
MRAS	3.14107235	3.498621948	3.660967526	3.278605543	1.893717674	2.313719686	2.932110181	3.128440147	6.978972263	7.595941868	8.126319467	8.600247841
MRC1	0	0	0.010971447	0	0	0	0	0	0.033842183	0	0.011165807	0.01078769
MRC2	23.55976699	27.19669091	23.05209528	18.14906718	20.99337286	23.09647649	20.02597488	19.15821883	81.84988253	74.92603704	69.0308118	134.0068158
MRE11	11.80250419	11.10004661	12.12480755	15.52675806	17.57564999	16.58282463	12.66278905	12.94217017	13.09241439	14.60345754	13.71612835	12.57719119
MREG	2.870254752	2.767609135	2.41726746	2.573430691	2.707020984	2.741628716	2.681859298	2.295166362	0.940425121	0.592243505	0.681511285	0.465720696
MRFAP1	43.80900768	43.71000587	46.58585056	53.55327309	49.54751726	49.74497876	51.90713836	55.23020359	113.8247115	109.9309032	111.4238946	121.3345166
MRFAP1L1	12.29836551	12.64486666	12.76712317	15.63105347	14.46009658	13.77186131	13.27073845	15.39694272	52.03141313	43.48663031	43.95217478	57.85734695
MRGBP	8.521273689	9.681867711	9.240483075	10.38105021	11.04272182	11.48573688	10.42546641	12.06047987	8.817892368	9.031068962	9.752481775	8.749209362
MRGPRD	0	0	0	0	0	0	0	0	0	0	0	0
MRGPRE	0	0	0	0	0	0	0	0	0	0	0	0
MRGPRF	0	0	0	0	0	0	0	0	0	0	0	0
MRGPRG	0	0	0	0	0	0	0	0	0	0	0	0
MRGPRX1	0	0	0	0	0	0	0	0	0	0	0	0
MRGPRX2	0	0	0	0	0	0	0	0	0	0	0	0
MRGPRX3	0	0	0	0	0	0	0	0	0	0	0	0
MRGPRX4	0	0	0	0	0	0	0	0	0	0	0	0
MRI1	22.76497918	24.57758822	22.58156993	22.29394941	22.14662136	24.62115614	23.45887993	25.3656443	9.865142869	10.16425719	10.32310635	11.11802955
MRLN	0	0	0	0	0.102343896	0	0	0	0	0	0	0
MRM1	3.502043832	3.209259778	3.529503772	3.962313483	4.567829476	4.504795267	3.810284772	4.479358407	1.521838311	1.546538466	1.557833439	1.728975331
MRM2	36.33237682	35.36594848	40.05548516	39.23477185	40.3239423	38.0368678	42.78884312	42.19469522	18.76351397	21.30201647	20.38253539	19.23759798
MRM3	12.09410552	11.45595197	13.3203881	11.69606877	14.26304928	14.72141138	14.67818201	12.49493336	4.041956135	5.217158029	6.017701121	4.50418981
MRNIP	7.661477858	9.428956457	7.634802489	9.4641967	9.497019252	10.85884115	8.109067066	10.34645386	10.28792027	12.52798956	7.963098338	11.61009643
MRO	0	0	0.03118422	0.009926112	0	0.020171732	0	0	0.010687764	0	0.010578884	0
MROH1	7.520140468	8.066891385	6.598897885	6.127710616	4.73726202	5.456864393	4.107017442	5.461698748	3.747788297	3.05273015	3.257939398	5.451093627
MROH2A	0	0	0	0	0	0	0	0	0	0	0	0
MROH2B	0	0	0	0	0	0	0	0	0	0	0	0
MROH5	0	0	0	0	0	0	0	0	0	0	0	0
MROH6	2.413164152	2.280569891	2.201346354	2.762378484	1.71233306	1.519799529	1.727510196	3.07881345	7.065874923	10.51143339	7.080059441	8.741156161
MROH7	0.588350552	1.016567138	0.272439273	0.507925265	0.396763271	0.742680423	0.378144655	0.6116855	4.108415302	3.817139479	2.442577489	8.674089114
MROH8	0.337778357	0.213148937	0.186526576	0.148431137	0.306696499	0.256394049	0.15102393	0.244295792	0.863031081	0.644152558	0.711865902	0.382088556
MROH9	0	0	0	0	0	0	0	0	0	0	0	0
MRPL1	28.19161688	24.09512836	27.99445027	24.37127864	31.79370725	27.65056058	30.7824775	26.73327487	13.35765982	12.50423827	16.63360149	11.94972823
MRPL10	22.92036768	23.40225412	23.80789867	20.98367823	25.04612123	23.6478634	24.06353512	20.99336667	21.46265984	23.64266411	23.94257595	24.76821033
MRPL11	19.31050852	21.45900435	23.35490824	25.69823745	24.1168149	26.01980793	23.10623371	28.25546403	17.69912349	23.2381924	21.93471633	15.34024789
MRPL12	56.67233553	61.39001373	67.22111527	72.65644199	69.36163417	76.05799076	70.87262881	84.76343338	55.03899235	63.18990632	70.6390418	55.22650023
MRPL13	54.39128584	51.00980498	60.44178052	60.999581	65.34879012	63.12636168	70.09121265	63.98991019	49.74814091	53.10393691	57.7006149	44.64777069
MRPL14	30.14562711	28.98547735	32.84019963	30.15799086	30.99685113	30.52519985	31.71024588	34.78921209	15.86039749	17.32701513	20.36715603	17.48219365
MRPL15	34.45744564	31.97832685	36.44332036	39.22106204	37.41361024	35.93496739	40.48149333	40.78362454	28.83634398	27.46659203	33.03488403	29.6137844
MRPL16	44.29501076	47.80821936	50.68893903	49.64602108	50.67269657	49.18274316	49.15560598	53.76656894	48.8976309	52.91424434	53.54837597	47.13951298
MRPL17	23.90272547	23.86032593	26.33404174	25.56056952	23.08265776	25.06106173	24.95461713	27.08309965	15.44393593	22.14534422	20.4801286	18.3191664
MRPL18	99.20289374	106.1080418	112.7561624	126.5511592	111.9767178	114.3685172	119.5485312	129.5867731	106.8939324	108.4227706	124.9305742	120.3043952

MRPL19	9.136694313	8.053667755	9.241092661	8.497899731	10.54534396	9.905509565	10.91574028	8.193594825	7.982836372	9.257361914	7.79783748	7.12596183
MRPL2	27.32589873	29.39580674	27.99765713	26.89307178	30.29201317	31.49045262	30.73106715	31.86646792	20.36808941	22.09671063	23.45348936	22.1398545
MRPL20	47.57004777	42.58843428	55.89614343	52.41761612	47.5346571	46.44339097	55.7971498	61.98197221	51.76810666	47.78369091	54.62809918	51.94703046
MRPL21	41.49810315	44.12116923	48.58398011	43.55104231	42.38944867	42.83503001	49.11405433	47.8238136	22.4646568	24.26511253	26.06561821	24.07842199
MRPL22	8.174033512	7.928975381	8.489967084	8.672831803	9.013262056	9.195563228	8.981283157	8.64550131	7.529709701	8.591245178	9.389321047	7.518288971
MRPL23	11.22041548	11.33088821	11.32904111	11.6178338	12.30613951	13.37989501	12.80030436	14.73902854	8.099416893	10.6926619	9.825491319	7.947038449
MRPL24	47.72161784	46.31710834	53.1593935	59.74429105	58.80133032	63.98314044	64.72599016	71.77324354	40.76637786	50.44443318	51.82707079	47.67059545
MRPL27	31.0763609	32.85153113	37.99552551	36.90145864	35.32655049	36.15904828	35.18466578	43.21584586	24.7394059	24.00462456	24.48737694	21.34808661
MRPL28	47.14528052	47.75494267	52.99706511	41.73322143	48.42878878	48.87891607	59.03961426	48.07058142	38.76115593	44.38906489	49.13406344	42.3154859
MRPL3	75.22356978	70.29937785	79.45623287	78.12472632	93.80392402	93.57312343	91.94295751	81.48667479	62.44222309	83.58953033	84.08390004	60.95118241
MRPL30	21.25809812	18.60700532	20.88017533	17.97252375	20.7851517	20.85668101	21.70221862	18.27065899	12.74184297	11.60315298	12.30536169	12.16025471
MRPL32	45.69303492	41.26155147	42.38050542	38.82918741	43.75664653	42.66477863	49.83256459	45.65214791	25.97500341	28.08476366	28.17133083	29.72008598
MRPL33	92.86823824	77.8608494	95.10454793	64.67425455	92.08994602	83.21072912	99.24009838	70.54272636	65.68020725	82.01380218	70.60585909	65.18790733
MRPL34	45.11951317	48.65841272	52.32796603	46.1479989	32.70727876	38.36971621	49.07461969	50.29891967	17.1507304	19.32716277	20.41351972	25.29147347
MRPL35	16.41176153	16.90185192	19.21870133	20.83567344	20.17044753	19.59984673	22.0736784	19.52180657	13.52120582	12.82777466	13.03389258	11.30592357
MRPL36	25.46110929	22.67647223	28.5689968	23.47847522	26.95139121	28.57370053	27.93751159	29.23735776	18.90051061	21.1552269	20.78139131	20.12318153
MRPL37	54.33367996	57.38399099	59.94218595	60.97731025	72.24016967	69.61382443	63.0904755	70.40334734	135.7169995	144.0626283	159.2692268	114.6947143
MRPL38	27.09268683	24.26166563	29.32616231	27.62421262	29.32415776	29.0512125	29.37155578	32.72839164	30.97081151	26.52387114	31.86973674	36.19484382
MRPL39	7.088882099	8.049730796	8.480101628	8.114453771	9.433322809	9.294727866	9.714170096	8.646436759	7.983585905	10.48484593	9.251852821	7.01701888
MRPL4	29.15445507	29.03665946	32.56201905	31.4296269	30.71984177	33.29949879	34.28355973	40.923396	11.54542618	12.36160758	15.04035569	13.11421296
MRPL40	8.312045987	7.880074101	8.595008154	7.461379838	8.108257526	8.564247898	8.800766029	9.524615083	10.86065235	11.75290383	12.3404237	11.26807111
MRPL41	45.43522523	41.00687092	49.59795193	41.68929315	31.81437936	32.93669716	44.61626947	49.68902176	49.25098442	44.1555914	58.92133451	58.68758769
MRPL42	28.63449579	26.45395968	28.38575924	29.37352995	36.49817187	35.28086565	35.29334068	29.0244659	16.60579473	20.35669694	17.67260092	14.47202605
MRPL43	17.85307035	17.79446976	19.90614804	19.81524171	16.7629123	18.67189673	20.00046923	22.96580183	7.849769266	8.012516086	8.224865189	8.174308583
MRPL44	28.55982793	27.82116085	32.54444646	27.34465264	27.27930488	27.16668197	31.47943714	28.66867628	25.84065436	26.09751179	28.84825007	23.2877983
MRPL45	37.55623309	33.14329342	35.17377051	27.6232	39.77563985	43.49532868	39.49730165	36.87249057	27.16690987	31.07679071	28.55591265	22.76084275
MRPL46	16.89620211	15.56376995	17.94012611	18.54534249	21.96903826	19.61746279	18.92462741	22.90402382	21.1395308	18.52737355	20.5184431	20.94358817
MRPL47	41.83670595	38.84122856	48.95433655	46.56327464	41.68851303	45.20885231	53.22849905	51.98474395	37.20579615	37.08989014	40.65165172	34.58476832
MRPL48	19.06237238	20.33746139	21.40014224	27.3562542	27.92430749	26.18100831	24.97933202	31.32917145	13.09126985	13.29247298	13.75531402	10.64123529
MRPL49	19.3622218	17.39105174	21.46911128	19.95762256	19.30836202	16.49134104	22.36056998	20.95090342	25.7533696	22.07398298	30.59473077	26.54683638
MRPL50	27.66897106	27.31837497	30.51241075	25.40815418	31.36775242	29.82224551	30.02515475	26.6616689	29.82398989	30.68689792	31.96125285	28.90442673
MRPL51	105.3936155	100.3619797	114.410314	134.3964358	113.5355104	112.7248054	128.8156936	130.5823732	48.09215794	55.77961534	52.22546415	43.34331313
MRPL52	19.74307629	16.05298466	21.59288903	19.56301029	19.80685707	19.59957271	21.73597242	23.14456058	18.62068318	18.63519449	23.2680796	17.72627014
MRPL53	28.83535246	26.23131219	32.7804558	27.56622286	25.90597433	26.99747594	30.66666556	29.74287554	28.4298103	26.84473825	28.4941505	23.33999906
MRPL54	38.86725905	31.9661821	43.51448459	40.06868762	47.07786338	47.10000562	39.7027609	51.97680725	23.78047999	23.86693794	26.88753187	16.71877237
MRPL55	12.28379874	13.7863789	13.07804608	14.06323552	12.75080885	14.17793583	13.89900176	15.45904158	12.22428262	15.45636473	13.42681889	16.93165359
MRPL57	13.90941381	13.49283593	15.73141817	13.31512806	13.25825861	14.75380781	13.20033916	15.53533594	6.271009804	6.558462585	8.283301077	7.051560009
MRPL58	18.55502099	16.70486403	17.6233198	22.70161303	21.41420637	21.16144602	18.47853156	24.27194881	21.20212756	22.48805556	22.93221669	22.15564419
MRPL9	48.0302573	48.03256595	48.96971548	52.33912463	53.76612951	54.38863094	56.8461403	61.1834695	61.68515428	70.17510541	70.83426649	64.9016364
MRPS10	39.35252948	36.37653109	44.08912942	50.24932017	38.0975151	40.58976305	47.67492606	47.8815564	37.73221027	36.83743043	38.69392421	43.17219296
MRPS11	5.449996347	5.151138765	6.576584777	6.343526664	5.363086728	5.03832719	6.400549162	6.448550708	5.589444832	5.24202937	6.208572845	5.758829216
MRPS12	42.75386086	38.67218207	48.15619511	37.85864107	39.63752605	38.94433179	43.96601701	45.35263007	12.83065259	12.03824879	18.40450648	12.0687282
MRPS14	16.95816464	16.95629247	17.12566151	20.13138642	18.83371012	20.86758575	20.76681126	22.70580825	15.15143714	13.01122101	13.21364713	14.59365242
MRPS15	52.52705319	53.06717484	55.83143121	58.27384905	59.97140165	60.81513679	62.38825145	67.05746001	36.71268966	46.8366329	44.92782833	30.63981201
MRPS16	50.8678081	49.4093986	53.47161282	61.77692652	57.78665053	57.69410063	56.19649737	62.68776888	28.36762947	26.81406078	30.05910676	30.25301179
MRPS17	23.50724843	23.322713	25.6579083	28.2134663	26.65517068	25.36700231	30.97258543	30.45962918	19.18632313	26.2801053	23.34293867	24.75036655
MRPS18A	26.36142656	26.92901249	30.60980103	26.25368725	28.7705692	27.36022704	30.78581978	31.79756649	31.39638147	30.67458679	33.98406078	35.78822134
MRPS18B	36.68452121	35.94203161	38.29430381	31.92972197	44.99618819	42.817516	38.06990489	36.11503508	7.795587004	9.542569122	9.042387466	7.804318715
MRPS18C	19.64708762	20.87471221	25.91584326	27.61245163	24.80180212	23.13812116	26.63727427	27.45030051	20.74270686	19.93608289	23.90064801	21.46370146
MRPS2	24.38708538	23.43956673	27.27463472	24.91406195	30.03502263	30.55379483	26.39842871	30.8510736	24.46195081	27.86016942	29.70758879	26.30406991
MRPS21	68.69454328	68.64344845	70.8581357	61.97790318	62.57166211	70.83386053	72.51062908	67.73081234	51.37156188	55.7476394	55.81636787	52.8716876
MRPS22	31.08133479	33.06769958	32.56794425	33.73058471	33.99605578	36.029021	36.91674738	36.8760581	31.79480817	33.57071985	36.15805918	33.72063656
MRPS23	37.03915483	35.33243633	37.9812449	32.76961566	35.66280047	37.11191654	43.87500241	35.1137156	20.99776644	22.46279131	24.00826599	22.1068039
MRPS24	0.154625175	0.0801496	0.327314835	0	0.080728159	0	0.079504552	0	0	0	0	0.160916376
MRPS25	30.1844228	26.87206385	28.25324088	22.64667556	26.28328913	28.71282425	26.54739007	23.17462366	14.82439115	16.35014553	14.91308962	15.15163937
MRPS26	23.5643409	25.44915658	30.20306304	32.45313237	31.20287085	32.70951109	34.83079865	41.20002957	7.721348621	9.143122493	10.59934908	12.50408521
MRPS27	54.20984452	53.29428842	57.8001128	51.99630537	67.01415196	66.16377965	60.23555469	56.12209045	35.84437414	38.96002614	36.36208898	27.94985776
MRPS28	27.55171292	29.19395336	35.5250022	35.07727989	37.90820785	36.65792796	42.1862171	38.65186676	20.54094449	22.61695254	21.31547801	14.8907963
MRPS30	41.66213305	38.04677522	38.60511882	35.39920854	43.30084421	40.83317717	43.90365116	36.16341844	40.92065029	45.79022105	42.96801744	40.80877837
MRPS31	22.42399922	24.49668675	27.17910923	30.29972799	32.18467106	32.60325785	26.44847456	32.16586659	18.32405505	18.90431411	15.58343041	21.07800225
MRPS33	32.27256404	27.03857061	33.25150145	26.16079714	28.26532954	27.59641015	34.94852708	27.5831742	14.47829612	13.57750597	14.82741256	11.51507345
MRPS34	40.29662884	38.26057672	51.51894258	48.21727369	51.23062737	49.49068972	49.65721186	55.87560981	37.42246851	42.9653815	49.87523735	37.50061272
MRPS35	35.60250482	32.36544818	39.37158084	37.1590971	43.78558968	40.59101557	45.54231766	38.51684802	55.84968006	65.11543574	67.40342914	58.92694584
MRPS36	17.45065518	17.88114059	20.18416854	21.68859244	16.15000533	16.09164595	18.40341926	20.40350496	11.98483516	13.54125973	13.4764232	14.70550466
MRPS5	17.93155172	19.25402095	20.02024362	20.45846509	22.81713208	20.84895971	20.47861061	21.87794806	13.25265818	17.86475932	15.50997389	13.04572685
MRPS6	16.74075227	19.4399212	19.84718136	19.39188335	15.43718104	13.73138667	20.68082032	18.02963909	23.65990184	19.66812478	22.65775688	22.85207589
MRPS7	37.07725925	32.72142827	40.69205788	40.16431179	37.88141759	37.37403452	40.47484011	41.67581606	37.57660595	38.19647131	39.56961536	35.81555014
MRPS9	18.11791282	18.38453505	20.42981983	21.57936196	23.39723917	23.24160869	26.3014867	25.02658521	16.30295319	17.02386767	16.99888156	14.35783783
MRRF	13.59273702	13.17520655	13.55235995	11.95145769	14.33043987	14.56030285	12.40588669	12.27204767	5.940315977	5.854596079	6.085657232	5.854712842
MRS2	10.64642552	10.97474337	10.54256716	9.763311063	8.579383739	9.426275405	11.0101273	10.0291292	5.956630218	7.38708912	6.103277984	6.146983977
MRTO4	34.25349021	30.45250586	41.011145	39.30545857	42.78483086	43.32686067	43.30216297	44.76885554	20.44471879	20.68926896	26.32045761	16.83337854
MRVI1	0.064568245	0.133875198	0.03416996	0.057101572	0.067420787	0.116041161	0.124497903	0.071077899	0.158099389	0.106202564	0.243426984	0.117591808
MS4A1	0	0	0	0	0	0	0	0	0	0.016417466	0	0.031162437
MS4A10	0	0	0	0	0	0	0	0	0	0	0	0
MS4A12	0	0	0	0	0	0	0	0	0	0	0	0
MS4A13	0	0	0	0	0	0	0	0	0	0	0	0
MS4A14	7.108099581	8.3546903	4.801060069	2.126882318	4.68765875	4.546341319	4.279979671	1.891356637	32.8075519	26.6818738	26.12642262	26.29612254
MS4A15	0	0	0	0	0	0	0	0	0.029861426	0	0	0
MS4A18	0	0	0	0	0	0	0	0	0	0	0	0
MS4A2	0.078822599	0.245145066	0.027808975	0.318663097	0.01371748	0.080947937	0.067547812	0.231384939	0.042889348	0.244891202	0.070754033	0.437491397
MS4A3	12.49605916	32.8161332	11.60649098	43.33415389	3.262029778	15.9102979	5.277821107	28.91544109	8.91556402	11.74494655	9.854863122	28.92834111
MS4A4A	0.406826317	0.702924921	0.200942273	0.301530673	0.113279836	0.167118322	0.306797806	0.291926518	4.191165576	3.955434369	4.119254141	2.653173634
MS4A4E	0.010334081	0	0	0	0.021581255	0.0424509	0.031881218	0	1.405758044	1.246491488	0.645626812	2.086381097
MS4A5	0	0	0	0	0	0	0	0	0	0	0	0
MS4A6A	1.919660514	2.724910445	1.625436129	0.671606089	0.63217858	1.289006306	0.956672771	0.838054293	3.406792123	4.452854815	3.085776759	6.02403083
MS4A6E	0	0	0	0	0	0	0	0	0	0	0	0
MS4A7	38.77006579	37.13379719	30.98428149	13.57369944	21.24977629	19.22821539	23.77812299	11.16403066	94.38602418	55.80805895	72.4280511	71.2408723
MS4A8	0	0	0	0	0	0	0	0	0	0	0.085678793	0
MSANTD1	0.114769246	0.118980744	0.091105014	0.075398016	0.047935842	0.064825109	0.106220862	0.084226717	0.031224409	0.069217033	0.018543789	0.077635244
MSANTD2	0.920730315	0.731718068	0.770991154	0.919147304	1.169278791	0.973435304	0.786314922	0.940784265	1.117693126	0.922796067	1.025892426	1.087677258
MSANTD3	0.37195549	0.308483622	0.39368282	0.576432663	0.284817873	0.56024428	0.357001083	0.582336534	3.346186118	4.568080595	4.006569377	4.33539862
MSANTD3-TMI	0	0	0	0	0	0	0	0	0	0	0	0
MSANTD4	6.40620334	5.664233895	6.146856396	7.453020738	5.731718393	6.003443901	5.998463169	6.00679561	6.54189286	6.298455108	6.063685921	7.554881027
MSC	3.111437193	3.361142415	2.739711054	2.695474829	1.883813948	1.879607434	2.258578281	2.507282301	1.564967997	1.462207528	1.858830147	1.959145149
MSGN1	0.041745198	0	0.044183694	0	0	0	0	0	0	0	0	0
MSH2	5.688999784	5.952724781	6.835007156	9.543795968	9.588682159	9.180129386	6.602708474	8.352666726	8.533615351	8.81955226	9.891582495	9.352461674
MSH3	9.534952543	9.119643911	9.310697551	10.89660113	8.995952468	10.18969553	8.274766503	9.566801459	6.123136841	5.745197682	5.356928312	6.900696326
MSH4	0.016420373	0.119160467	0.017379549	0.04978807	0.034291607	0.067452497	0.033771845	0	0	0	0	0
MSH5	32.26415514	34.75320951	28.51446069	31.39143923	43.81143088	48.90698338	28.35187579	34.33351041	27.41982558	39.36254385	30.14318546	24.25620752
MSH6	14.85802336	17.54196755	17.68319372	28.97517326	25.63787772	24.57849955	19.57726142	24.87259904	29.55830973	28.78719575	31.67513841	35.41924789
MSI1	0.049411902	0.010245018	0.031378943	0	0	0	0	0	0	0	0	0
MSI2	4.418503769	4.533901076	3.881264283	3.891598759	4.654509126	5.158095535	4.179038486	3.664704988	5.115915208	5.524038175	4.72707448	4.839140683
MSL1	23.71752308	25.42409788	24.62400874	31.29881367	33.1984887	30.75188956	26.02877275	29.93612561	26.46362184	31.92924096	24.9648581	24.75417503
MSL2	7.695243394	9.152545277	8.154831872	10.18398378	11.90364566	10.4750356	9.098472736	8.471064044	18.50026351	17.55510678	16.66014279	15.83827263
MSL3	4.844363291	4.829675067	4.753082455	4.172478025	5.418375357	5.528769388	4.36354207	4.636433314	5.647067011	5.825797434	5.265783165	5.00466584
MSLN	0.024536963	0	0.025970261	0	0.102483901	0.025198568	0	0	0	0.053811559	0.132151627	0.025535294
MSMB	0	0	0	0	0	0	0	0	0	0	0	0
MSMO1	10.70837032	18.91849567	10.57829437	14.43055883	10.90191615	17.32044224	9.796068277	12.31187876	34.79020828	83.68738169	58.51440647	16.66965562
MSMP	0	0	0	0	0.165742769	0	0	0.077659186	0	0	0	0.082594246
MSN	504.3155518	388.2342133	518.8989933	336.7578801	401.9395041	323.0491345	482.6015424	315.2310911	200.9202258	204.8869844	205.3723468	193.7084899
MSR1	0	0.009959672	0	0	0.010031566	0.019732354	0.029638547	0	0.167279435	0.179088442	0.165575301	0.059988108
MSRA	2.733568782	3.01535793	2.893246898	3.239156688	2.573119157	2.572183507	2.866461399	4.229627125	5.568603024	4.400226115	4.815637041	7.357212685
MSRB1	29.4342897	33.61350923	31.15365815	46.17316308	18.20868474	23.30070618	26.99757122	45.19001327	33.24140376	24.12287099	27.82492042	55.16130847
MSRB2	3.317385548	4.143189621	3.621754539	2.877661834	1.636508698	2.387466414	2.686173198	2.147012611	16.43042358	16.3837389	14.49042591	15.35892128
MSRB3	9.472019261	15.05951209	8.762789383	18.5030134	5.942732976	12.27087395	5.634352995	12.7898246	1.793160981	1.363430029	1.263114339	2.461721271
MSS51	3.0646799	3.361321406	3.290709609	3.142353943	3.20007355	3.17012123	2.763332775	2.455543087	2.585940366	3.457953429	2.248617476	1.687131496
MST1	0.710848269	1.902315671	0.962335782	0.618203145	0.586897436	0.899786269	0.901002734	0.711744653	2.71652744	2.66471368	1.656048676	2.683818193
MST1L	0.184437227	0.143403912	0.048802726	0.023301272	0.072219536	0.082867012	0.118541491	0.056397738	0.639775302	0.695210066	0.608424053	0.47985346
MST1R	0.031839977	0.055013927	0.033699871	0.053634289	0.055411044	0.010899499	0.021828469	0.010385193	0.011549952	0.011637944	0.011432289	0.011045148
MSTN	0	0.019788621	0	0.038584724	0	0.019602859	0.019629361	0.037355749	0.020772706	0	0	0.079459239
MSTO1	9.035527519	7.59895004	9.025906546	8.987435314	8.496672912	9.813979334	7.993006345	10.61211935	8.798615445	10.06486424	11.30344378	10.45999272
MSX1	0	0.028754702	0.088071311	0	0	0.056969545	0.085569847	0.081422054	0.84517072	1.003682656	0.926192172	1.818520942
MSX2	1.907645478	1.760588363	1.649734694	2.304257035	1.724713663	2.006864903	2.488049053	1.821112955	1.164582717	0.841826353	1.202836911	0.968420213
MT1A	0	0	0	0	0	0	0	0	0	0	0	0
MT1B	0	0	0	0	0	0	0	0	0	0	0	0
MT1E	5.710920374	5.394219315	7.051936364	6.734012621	6.02947957	6.972722956	5.220298856	5.091438824	31.07454679	37.36479838	32.94521364	29.18810529
MT1F	11.36350975	18.59470723	11.67355145	13.9622379	10.70224736	9.267281871	9.394376327	14.06258334	26.9149753	20.52326064	26.16076894	27.8255398
MT1G	5.31452453	6.886928605	5.343717752	3.759962274	5.826779117	8.050289026	3.825641236	5.460303678	1.590471179	3.787935143	3.577882942	1.520957746
MT1H	0	0	0	0	0	0	0	0	0	0	0	0
MT1HL1	0	0	0	0	0	0	0	0	0	0	0	0
MT1M	0.122294457	0	0	0	0	0	0	0	0	0	0.263462289	0
MT1X	15.71665391	23.19735754	17.48055664	14.13475917	16.27191941	19.14966386	16.02528377	16.81244494	23.19138894	45.12958335	33.14146917	21.62333717
MT2A	18.45239366	15.42702481	15.49821261	26.95194196	18.64606149	11.49221926	9.916257961	22.24935688	38.99565349	70.49195166	50.65237377	33.6984524
MT3	0	0	0	0	0	0	0	0	0	0	0.096602839	0
MT4	0	0	0	0	0	0	0	0	0	0	0	0
MTA1	18.02988563	19.32602018	18.62192348	17.42590133	23.11598772	22.62834278	21.05872786	21.72301265	17.56634818	18.58745859	19.35409114	20.00500473
MTA2	54.82483559	54.68147059	58.06147576	70.20068184	63.91059036	65.24011011	61.71553315	75.77038652	59.99634544	54.02107589	63.49922491	62.90861621
MTA3	2.033552251	2.358710809	2.002611765	1.6144573	2.006451567	1.906785458	2.21243688	1.886023394	4.079641964	4.517916274	3.974589384	3.46580982

MTAP	8.997413366	7.423526963	8.088174346	5.701492685	8.264778994	7.252794846	9.07262911	5.423615331	0	0.083915201	0	0
MTBP	4.610641578	6.202121785	5.784545813	9.001683078	9.933966905	9.146558659	6.684164479	7.801656464	8.994841994	10.91303475	9.26660466	7.876125166
MTCH1	30.87845016	35.32355694	31.89970552	34.62451239	32.04963366	32.49737958	31.90324939	36.88030186	44.52208995	46.39573596	49.40111838	49.1707701
MTCH2	23.57550502	21.53798839	25.02734815	24.44597871	20.96870688	22.35064459	22.12680849	23.41409187	18.16663025	21.15591909	20.92146321	19.80896771
MTCL1	0.0087652	0	0.013915812	0.004429481	0	0	0	0	0.510321999	0.5766839	0.424869957	0.798159904
MTCP1	2.226602518	1.875500643	2.258472362	2.648932781	2.155441844	2.096086359	2.05121743	2.382998911	1.716362707	1.322511839	1.848778479	1.762034317
MTDH	48.32995842	47.59581955	49.77289777	54.31005096	56.41961686	52.33229263	52.18070201	45.69777004	59.01413501	54.55163339	62.42309547	55.04964578
MTERF1	7.959697057	8.407966079	9.168786537	9.364501733	9.884471808	9.128419194	10.04450784	9.041660114	8.525513861	6.690650008	7.492016759	7.89158537
MTERF2	0.79238613	0.738563081	0.815589681	0.705346688	1.017161715	1.037720041	1.00174446	1.002980667	0.624980047	0.581912906	0.328882944	0.839756521
MTERF3	18.42444455	15.63670827	21.18468329	22.54524139	22.89337922	21.04537866	22.05553114	21.48463663	19.11540976	20.55208545	21.59424796	25.10181273
MTERF4	5.043598376	4.776728357	5.473089167	4.765396177	4.727170519	4.683673417	5.186594322	4.777680646	5.744154096	4.986285156	5.447229784	5.611755536
MTF1	6.598199727	5.816472998	6.201254309	5.688978902	3.947816951	3.957398001	5.451894144	4.316105373	4.786994103	4.850038653	4.385797192	5.819950864
MTF2	5.818143998	5.678857887	5.757808632	5.627204972	6.825326527	6.936073368	6.454554497	5.20285853	9.983449887	11.49509902	9.978475114	9.65040312
MTFMT	5.624110707	5.849731975	5.127518427	5.703032513	5.019793324	5.261085396	6.890650433	5.684845314	3.979295151	3.480423599	4.618542099	4.732573375
MTFP1	14.44184615	18.76930542	18.31580056	20.08591516	21.80645706	24.81964258	17.27600312	23.77209675	10.95103555	14.35865448	16.10047324	10.95440118
MTFR1	7.986827901	7.241410515	7.321477043	8.469036318	8.452756121	7.826827213	8.213270201	7.907873763	9.015761842	10.79149186	9.696737845	9.875529078
MTFR1L	21.31781188	22.42507764	18.77095414	18.96578081	16.78823197	17.89123461	19.04377188	17.72626676	29.43752581	28.43406804	25.8089983	33.86034124
MTFR2	4.558566118	4.672625343	6.324464552	7.367576742	9.552077386	8.371838218	6.831111165	7.836154187	6.458056207	6.124476177	6.724044233	6.517711943
MTG1	19.72345839	20.43115543	19.0061254	21.17153698	20.95073545	21.49636612	18.99208756	23.22611177	10.0491567	8.613653267	10.46414863	12.931505
MTG2	5.197551224	4.219249479	4.986712274	4.283740848	4.642194853	4.41923937	5.511524008	4.724368493	4.090544273	2.970471935	4.111699802	4.323171643
MTHFD1	49.20578463	53.17935267	57.31616912	61.26217948	67.69140415	74.95741436	52.70321674	63.96842133	39.48606371	51.74861602	52.51518626	38.91338794
MTHFD1L	11.70459095	10.4563771	12.41399346	10.88930105	11.55842715	11.5465274	12.3193558	11.24318169	3.865725541	4.742532043	6.136096147	3.852555822
MTHFD2	138.7539042	105.9592366	142.1216572	90.00397418	97.7876276	93.24570979	124.782321	86.9928409	42.1255469	50.21304618	69.6970943	45.32634819
MTHFD2L	0.940782029	0.739697125	0.923014332	1.127126075	0.800224516	0.765320171	0.815271111	0.806782911	1.110081879	1.182289791	0.996296841	1.248575782
MTHFR	6.464686296	6.825657856	4.767675913	4.698776972	4.867554534	5.264744214	4.897146206	4.678211145	14.30284574	16.58184811	15.1180906	11.18670355
MTHFS	0.109324585	0.068001774	0.115710649	0.066296427	0	0	0.179878644	0.064184797	0.023794497	0.071927315	0.094208377	0.159281711
MTHFSD	4.729338572	4.110311957	4.328147927	4.258808841	3.592316384	3.633514375	3.921821745	4.15795469	4.334065614	4.357336087	4.05052051	5.338074447
MTIF2	14.07265163	13.30821496	13.52322901	14.06075388	15.74321164	15.39081654	15.25668153	13.01334275	11.92590227	12.1269024	11.86932778	9.679854457
MTIF3	8.346176287	7.351128659	8.041841822	8.233812023	9.122868123	8.323643921	9.087174951	8.565353549	5.807879041	6.399053112	5.283084375	5.874131363
MTM1	7.686188143	6.790153592	7.084402594	8.058698109	7.764966021	8.419236725	6.915290764	6.922228799	7.800519739	7.1958224	6.705479061	8.700687578
MTMR1	8.57864661	9.91618903	8.761966354	9.205088986	9.263692985	9.279821925	8.145524912	7.736699711	6.332725023	6.435843143	6.268211343	6.740401534
MTMR10	6.660436217	6.37894548	6.522525233	5.668286602	6.405373517	5.537622768	5.216653396	4.789136812	11.77707963	9.322443922	12.95233601	10.05985805
MTMR11	9.232429492	10.80659937	9.110485764	5.26194923	8.190636309	7.936784847	8.685158579	4.754725843	6.53445182	7.751380935	7.876111957	5.381962541
MTMR12	13.25456095	11.00300783	12.57358278	11.16695466	14.1293273	12.18980704	15.00356114	10.17246832	25.25324836	22.22425791	23.46170431	26.13625076
MTMR14	29.98251725	30.29704445	28.83833722	24.30611623	16.23259967	20.82470733	25.39600972	25.99184404	31.76347918	31.6608838	32.65448093	33.70321909
MTMR2	11.86032815	11.78600188	13.22037358	14.15785465	12.86405457	12.8604562	12.75697576	12.6300114	12.81397921	13.59115851	12.99419449	11.64234454
MTMR3	4.154360272	4.587795703	3.906734426	4.950898213	3.833607929	3.355406542	3.587993301	4.354390904	8.602202063	8.494814221	6.688501384	10.02123348
MTMR4	7.783115272	11.97143978	7.708073538	11.00688171	10.5291752	12.65682352	8.395521723	9.947339735	9.941247694	13.56347084	9.719286233	14.57494938
MTMR6	16.98829022	14.84886192	16.92419878	17.73495203	17.50951988	14.89398002	17.82919432	14.66976411	12.29603914	10.27733398	11.63319669	12.95318621
MTMR7	0.122968482	0.074988737	0.176087373	0.080418988	0.007553004	0.014856959	0.12645488	0.028311831	0.236153745	0.174498755	0.218164767	0.225832365
MTMR8	0.768419133	0.628907798	0.856110955	1.205834253	0.844596748	0.685304447	0.748615574	1.147639926	1.914531516	1.552163173	1.306915528	1.788766028
MTMR9	4.659506774	4.436461215	4.790142365	5.669716072	4.417039369	4.192421971	4.892946172	4.593787206	8.180538724	6.776434359	6.656687484	8.636063012
MTNR1A	0	0.021114353	0	0	0	0	0	0	0	0	0	0
MTNR1B	0	0	0	0	0	0	0	0	0	0	0	0
MTO1	25.6712493	22.82159002	28.22988134	20.01736888	24.22931842	23.61724632	27.51678274	21.38866951	27.84286477	24.13901304	31.01573691	31.8865163
MTOR	14.64522682	12.4475523	14.76993682	17.26981569	12.83047175	12.88411484	14.77759543	15.21475187	18.47889919	17.78256181	18.79855379	24.59371626
MTPAP	5.687345045	5.58833817	6.262779154	7.635213172	7.528231204	7.453278931	6.961203504	6.586300465	5.511978289	5.564469467	5.754916484	5.918744824
MTPN	0	0	0	0	0	0	0	0	0	0	0	0
MTR	9.567699118	9.570591837	9.124294742	10.09909243	13.00065588	13.1743616	9.507958625	8.814287351	8.071749348	9.125600679	7.803599981	8.573390147
MTREX	29.61829777	28.50244848	31.98513717	32.2507672	36.25946353	35.43820083	33.77498591	27.70415734	35.83977056	37.01106926	38.23211328	33.30096268
MTRF1	4.561092607	4.023599385	4.089901743	4.008594084	5.348306409	4.684075934	4.771980888	3.681325093	2.367734096	2.528670144	2.349716322	2.612141959
MTRF1L	6.283747684	5.730962983	6.664836442	6.056195173	6.907418703	5.554635894	6.720925786	6.057875715	7.285518555	6.933995212	7.168459038	6.580801859
MTRNR2L1	0	0	0	0	0	0	0	0	0	0.037944935	0	0.036012154
MTRNR2L10	0	0	0	0	0.050122033	0	0	0	0	0	0	0
MTRNR2L2	0.051939731	0.215382709	0	0.209981346	0	0.160020633	0.267061621	0.10164658	0.282617071	1.025172527	0.615423494	0.810794867
MTRNR2L3	0	0	0	0	0	0	0	0	0	0	0	0
MTRNR2L4	0	0.045316102	0.04626546	0	0.045643216	0	0.044951396	0.042772484	0.142708994	0	0	0.045490576
MTRNR2L5	0	0	0	0	0	0	0	0	0	0	0	0
MTRNR2L6	0	0	0	0	0	0	0	0	0	0	0	0
MTRNR2L7	0	0	0	0	0	0	0	0	0	0	0	0
MTRNR2L8	0.542266892	0.216217526	0.3090461	0.295113318	0.087111316	0.214187824	0.214477395	0.244897341	0.40854598	0.137219475	0.179726213	0.347279993
MTRNR2L9	0.102105429	0	0	0.206395327	0	0	0	0	0	0	0.329952772	0
MTRR	27.7876466	21.53215467	25.52931783	21.77008327	28.5661532	24.52662339	27.90037808	20.82194052	21.17409421	18.84069	20.61700481	28.34104977
MTSS1	0.04396655	0.031336193	0.043626377	0.016663825	0.020085159	0.028220028	0.022606544	0.016133059	0.622005795	0.738232648	0.748866869	0.237356174
MTSS1L	5.26975933	5.182460183	5.147753638	3.488859872	7.844949243	7.169461111	5.846734719	5.061871816	7.797245011	7.633989134	8.353151183	5.806175136
MTTP	0.039420924	0.027244992	0.055631532	0	0	0	0.013512862	0	0.071499703	0	0.042462787	0.082049669
MTURN	1.166151474	1.563951124	1.263658202	0.851229849	0.80211632	0.883939224	1.341977756	0.950904278	3.666186029	2.79088458	2.651842651	4.83513024
MTUS1	1.177933615	1.279308701	1.260441636	0.97684851	1.193930049	1.231850492	1.05603159	0.755743258	0.169039953	0.137208472	0.23238595	0.157161531
MTUS2	0	0	0	0	0	0	0.005684147	0.005408621	0.054137064	0.018183166	0.0357237	0
MTX1	12.34224151	11.20421376	12.02643683	11.35224393	11.08052764	13.91571014	11.68485341	12.74788738	15.03042642	17.40056185	16.49516931	15.18902171
MTX2	17.36196664	17.4142628	18.60833449	17.07257185	19.83063481	19.08529524	21.07699464	16.74344721	17.15480682	20.41269561	16.87877218	15.26353212
MTX3	1.017322174	1.040591128	1.098282775	0.836273074	2.131614115	2.124330711	1.89704634	1.645818783	7.941603806	8.879660067	7.385843509	6.655780389
MUC1	16.24826239	17.87641332	16.76357709	9.40940349	11.95268676	11.48503546	13.33703992	9.969107213	9.717229817	10.01597649	9.460560989	4.874768125
MUC12	0	0	0	0	0.003442608	0	0	0	0	0.003615243	0.010654072	0
MUC13	0	0	0	0	0	0	0.019220274	0	0.081359162	0	0.060397746	0.058352448
MUC15	0	0	0	0	0	0	0	0	0	0	0	0
MUC16	0	0	0	0	0.007623545	0	0	0	0.001324218	0	0	0
MUC17	0	0	0	0	0	0	0	0	0	0	0	0
MUC19	0.052229615	0.042865741	0.041460409	0.015396594	0.020451395	0.024584043	0.029093148	0.01064728	0.371821013	0.238632914	0.307084979	0.278567684
MUC2	0.004254393	0	0.004502908	0	0	0	0	0	0	0.004665115	0	0
MUC20	0.145375116	0.23682954	0.065943012	0.251880364	0.065056116	0.106639248	0.042713368	0.121928819	0.51981471	0.159409734	0.313185585	0.518708442
MUC21	0	0	0	0	0	0	0	0	0	0	0	0
MUC22	0	0	0	0	0	0	0	0	0	0	0	0
MUC3A	0	0	0	0	0	0	0	0	0	0	0	0
MUC4	0.012807226	0	0	0	0.02005955	0.003288139	0.003292584	0	0.003484366	0	0	0
MUC5AC	0	0	0	0	0	0	0	0	0	0	0.003321968	0
MUC5B	0	0	0	0	0	0	0	0	0	0.00329339	0.003235192	0
MUC6	0	0	0	0	0	0	0	0	0.014634076	0.007372782	0	0
MUC7	0	0	0	0	0	0	0	0	0	0	0	0
MUC8	0.780909727	0.809565485	0.568236367	0.098657902	0.917335488	0.300737189	0.677573484	0.167152153	2.57603217	2.461860517	1.393183807	2.361858318
MUCL1	0	0	0	0	0	0	0	0	0	0	0	0
MUL1	7.126640343	6.269275695	7.687990854	7.947402861	5.706331984	5.700219222	6.888268271	8.230686981	9.601242403	7.363806031	8.673034295	12.8899089
MUM1	10.63255755	10.82969837	10.95285687	12.32109811	10.33485096	9.238590596	10.20843654	10.893048	15.3354168	12.51868441	13.62749216	12.76827925
MUM1L1	0	0	0.012735416	0	0	0	0	0.011773911	0	0	0	0
MUS81	18.59150558	22.13904348	21.32301162	18.70308567	20.17944814	21.70946587	18.58568841	21.40345762	21.87710166	19.51027466	22.84044663	23.28044109
MUSK	0.011956352	0	0.006327384	0	0.012484568	0	0	0	0	0	0.006439474	0
MUSTN1	0.811941248	0.459128574	0.781245285	1.193638817	0.385368989	0.758030982	0.834961381	1.011167348	1.285229236	1.052204206	0.795085098	0.921792573
MUT	12.46232753	13.13496432	12.76527857	14.63893113	13.22977889	14.34837949	12.11791762	12.95321654	12.67305563	13.95394236	11.84292144	12.03270214
MUTYH	3.865629372	3.542612326	3.387708543	3.703817517	3.326000149	3.668151129	3.434596627	4.115401294	5.855825706	5.832616315	7.345146929	7.852719147
MVB12A	13.15480473	10.78978191	12.72796133	10.27241131	12.26994752	11.91093265	11.45623158	12.33446364	13.21961787	14.6925243	12.19727286	13.69003142
MVB12B	5.993290465	7.31991043	5.483067364	6.49007299	5.431956363	5.993505929	5.929166024	6.394706099	11.67626878	7.807829597	8.808077626	12.21486555
MVD	16.64702092	25.87389689	17.27602921	22.50072838	18.9461051	20.91490454	19.63423165	26.32646416	34.65692765	47.70158835	58.36496227	15.84384429
MVK	8.519342735	11.1693742	7.847824195	9.726931167	8.074087205	10.08549989	8.449661462	10.80895323	6.817524866	9.159284207	10.44810237	3.6062283
MVP	18.67237753	14.112906	14.6811614	6.712152432	8.298357952	6.801287208	19.46662829	10.72859668	29.42927791	19.2848485	24.98793447	34.82461435
MX1	0.243768145	0.308871815	0.143337537	0.118625368	0.160264359	0.296700449	0.129981938	0.194356447	8.341604068	2.098813316	2.431279516	4.021397433
MX2	3.462925094	1.892402233	1.164910967	1.356576917	0.798864436	1.488682845	1.02140245	1.549774393	11.91906654	6.181550817	6.216895129	8.548597179
MXD1	7.213565841	7.855659651	7.320617429	5.857758056	4.961483376	5.450292691	6.304879374	4.958767862	5.254292605	5.420376921	4.024401714	4.625865953
MXD3	3.149246681	3.247161782	3.423292771	6.882020702	3.697201555	4.353006701	2.765883114	7.895440691	5.409367625	5.39457899	6.032711327	6.997647909
MXD4	9.228473938	7.773281789	7.451962514	4.331917883	4.174291822	3.901218845	7.342570789	4.631822931	21.57922095	8.669095843	12.83277046	21.26736964
MXI1	18.15634205	31.53584768	16.84079635	13.9118254	21.00961388	26.84491116	16.66548081	14.10027572	8.535610352	16.00118626	9.219462642	8.805041982
MXRA5	0	0	0	0	0	0	0	0	0	0	0	0
MXRA7	2.652759124	3.111054083	2.895457993	2.253829973	1.367665103	1.694165961	2.207950775	1.60611305	4.718220373	5.844987322	5.063054379	5.823333341
MXRA8	0.078146665	0.081014285	0.035447789	0.03384969	0	0.057324127	0.011480325	0.021847688	0.048596064	0.061207857	0.01202525	0.058090144
MYADM	31.2637272	48.1039885	34.42171201	27.52348905	18.42058251	23.24604831	23.01340912	24.1090359	31.61950268	29.77672435	35.97193778	32.14825832
MYADML2	0.021945172	0	0.023227072	0	0.022914681	0	0.02256736	0	0	0	0	0
MYB	107.4143001	103.6814181	106.0548122	101.1995905	102.4744549	118.3637555	96.18209033	92.60788291	141.9377003	131.2759095	135.117903	175.6246052
MYBBP1A	33.98387693	28.0449539	33.09237567	29.48170162	33.92327466	35.64667404	36.24735155	33.73786891	6.041542966	6.445661881	7.648039989	6.106379533
MYBL1	1.495229201	2.053878743	1.661699767	3.721388751	2.21507525	2.725593997	1.18204683	2.926178719	2.572983508	3.863259661	2.818561484	3.734556644
MYBL2	42.41596281	53.79541876	56.4810802	81.70504984	75.52946319	79.17946152	52.36615815	92.35592865	76.207973	79.29575984	98.34913491	79.53174541
MYBPC1	0.062569257	0.038919155	0.066224164	0.025295427	0.026133395	0.025702539	0.038605931	0.024489734	0.013618199	0.027443895	0.013479466	0
MYBPC2	0	0	0	0.015115389	0.01561612	0	0	0	0.016275224	0.065596859	0.080547115	0.015563896
MYBPC3	0.255203039	0.304252976	0.121549687	0.128966489	0.119914913	0.117937901	0.104975419	0.162316355	0.569335675	0.587665099	0.439832705	0.650686754
MYBPH	4.111694019	3.459480415	5.707892257	4.848290224	1.33778092	2.019485197	2.389890972	4.577247922	15.0772921	4.900695544	18.19727906	8.185885544
MYBPHL	0	0	0	0	0	0	0.013359529	0	0.014137677	0	0.013993651	0
MYC	105.1299234	87.87419082	104.9554797	79.74806152	123.9765816	124.0363679	102.9052856	104.3851879	25.70186449	29.69808483	34.99768207	17.9598062
MYCBP	17.64282288	17.39855647	21.93736761	24.68005312	22.36519353	22.83668073	23.47160337	25.96723366	24.97572445	25.6030677	25.6477714	28.07586117
MYCBP2	20.00525157	16.54239385	16.77798152	21.40540074	16.04731697	17.63749897	15.17108751	14.06174057	27.01556297	25.29288706	22.76936819	28.15783725
MYCBPAP	0	0	0	0	0	0.011631332	0.023294114	0.022164988	9.441301812	9.215164337	6.197546929	10.38413595
MYCL	2.423658265	1.971767582	2.116605082	0.955667475	1.25969191	1.350538373	2.03413463	1.31871604	9.864186304	13.57422387	11.84755486	12.14761005
MYCN	0	0	0	0	0	0	0	0	0	0.058266334	0	0
MYCNOS	0	0	0	0	0	0	0	0	0	0	0	0
MYCT1	0	0	0	0	0	0	0	0	0.011504569	0	0	0
MYD88	18.64701368	16.48005231	15.93746372	18.36003264	15.36413865	15.54862379	16.33327853	20.65480321	17.01526662	9.922023593	13.06471317	17.25905239
MYDGF	54.2252246	49.71211407	58.95252784	57.59328248	47.19059773	45.1319616	63.80642251	65.70523429	39.88870039	48.43828203	51.32704236	41.60592343
MYEF2	0.058114907	0.055226833	0.046132214	0.024473571	0.060682349	0.044761419	0.014940645	0.018955244	10.24018481	13.03714688	9.009077691	8.411678711
MYEOV	0.051369509	0.013313633	0.013592549	0	0.053638949	0.039565961	0.026412968	0	0.139757177	0.140821896	0.221333475	0.040094677
MYF5	0	0	0	0	0	0	0	0	0	0	0	0
MYF6	0	0	0	0	0	0	0	0	0	0	0	0

MYH1	0	0	0	0	0	0	0	0	0	0	0	0
MYH10	0.044612925	0.052857157	0.047218935	0	0	0	0	0	2.420565168	3.074964434	2.251739761	2.739256807
MYH11	0.015486994	0	0	0.00782633	0	0.007952289	0	0	0.042134305	0.033964239	0.008341014	0.024175665
MYH13	0	0	0	0	0	0	0	0	0	0	0	0
MYH14	0.007422008	0.007694362	0	0	0	0	0	0	1.251935151	1.798616101	1.926726977	0.942326298
MYH15	0	0.007489812	0	0.014603966	0.015087755	0.044517018	0.022288601	0.035347025	0.070760515	0.15844354	0.077821836	0.135335685
MYH2	0	0	0	0	0	0	0	0	0	0	0	0
MYH3	0.455938922	0.536298384	0.436170885	0.434230611	0.48523714	0.513255033	0.450832393	0.428979374	0.104960213	0.067301714	0.103890947	0.191620804
MYH4	0	0	0	0	0	0	0	0	0	0	0	0
MYH6	0	0	0	0	0	0	0	0	0.009856633	0	0	0
MYH7	0	0	0	0	0	0	0	0	0.009671058	0	0	0
MYH7B	0	0.049410205	0.007206476	0.013763171	0.049766872	0.013984679	0.014003585	0.013324795	0.044457743	0.097058948	0.036670697	0.085029329
MYH8	0	0	0	0	0	0	0	0	0	0	0	0
MYH9	155.9113588	142.1867586	148.6634017	114.2201052	125.4141072	114.114919	143.7183239	110.7182019	143.8364636	121.2664249	146.0059362	153.703727
MYL1	0	0	0	0	0	0	0	0	0	0	0	0
MYL10	0	0	0	0	0	0	0	0	0	0	0	0
MYL12A	224.4742128	214.7726531	222.4404084	156.4772727	182.4736805	174.8914784	223.5931207	153.5650874	265.1807096	277.7688966	287.2525409	201.9227594
MYL12B	126.7128369	137.7081714	136.9670878	150.0901385	123.8685196	127.5493946	133.9693538	145.8906265	214.6970393	238.2714845	215.4128511	214.5356795
MYL2	0	0	0	0	0	0	0	0	0	0	0	0
MYL3	0	0	0	0	0	0	0	0	0	0.063513858	0	0
MYL4	0	0	0	0	0	0	0	0	0.048037947	0.2904235	0.095097135	0
MYL5	6.196591816	6.191785967	5.057201531	3.716225534	3.020795625	3.162669325	4.203399849	3.652648514	4.915400003	5.218909282	4.000825192	6.623525669
MYL6	637.267494	674.0294062	620.5670793	596.0184237	466.8574294	480.3356547	630.8482736	623.8948961	713.5566108	593.9092958	716.6760466	597.2292944
MYL6B	22.7072301	25.95219947	23.07015859	17.68540257	24.71374225	23.57918063	26.26340205	21.37788067	18.98740478	21.0175356	23.09752101	13.6313109
MYL7	0	0	0	0	0	0	0	0	0	0	0	0
MYL9	0	0	0	0.04543456	0	0	0	0	4.158272222	4.189951395	6.004387012	2.105221761
MYLIP	31.42518536	50.24736537	29.80540702	17.64895173	42.63833288	44.77231926	29.18267254	20.57625326	12.07969024	13.16307916	11.27133316	9.896507324
MYLK	0	0.012226657	0	0	0.006157457	0.012111881	0.006064128	0.005770184	0.757246502	0.827677799	0.800347908	0.601412831
MYLK2	0.07706346	0.099864163	0.1427388	0.292079316	0.140819044	0.13849739	0.118872541	0.452441919	0.335457255	0.084503221	0.228277386	0.260646504
MYLK3	0.039348856	0.054390369	0.062471058	0.019884888	0.047935112	0.013469947	0.06744079	0.038503055	0.135601083	0.093486516	0.077706123	0.068249724
MYLK4	0.305230315	0.415315489	0.309599142	0.546293941	0.331994792	0.287338712	0.287727179	0.360892205	0.352927335	0.251023101	0.219188669	0.443385278
MYLPF	0.115100665	0.178986487	0.243648262	0.116331911	0	0	0.177545993	0.056313292	0.187887456	0.189318848	0.185973381	0.179675611
MYMK	0	0	0	0	0	0	0	0	0	0.072132487	0	0.068458312
MYMX	0	0	0	0	0	0	0.050281843	0	0	0	0	0
MYNN	4.111100187	3.697994203	3.684986427	3.730929388	4.455019129	4.205988109	4.019871381	3.657720757	3.59434709	4.098946283	3.850719769	3.615180211
MYO10	2.050916061	2.485866435	1.946301525	2.567690651	2.061014536	2.620892934	1.847412826	2.546262116	6.536306384	7.905013615	6.071071124	8.561516702
MYO15A	0.011591892	0.040057534	0	0.003905297	0.040346689	0.0158726	0	0.015123633	0.071484301	0.08050288	0.074918187	0.112592932
MYO15B	9.066962058	8.260707509	6.626839752	6.699592306	6.972707079	6.824194769	5.212215813	6.150501457	8.40927684	6.104209657	6.062332269	8.666546849
MYO16	0	0.007224987	0.014752696	0	0	0	0	0	0	0.007642064	0	0
MYO18A	7.5116247	9.70066883	6.255469422	7.715424297	7.744283847	11.99284353	6.433672734	7.595832283	17.08010701	22.63446491	18.25830211	13.82671424
MYO18B	0	0	0	0	0	0	0	0	0	0	0	0
MYO19	13.50206862	14.58302112	14.67820953	15.51747862	16.85057926	18.03349075	13.7127967	16.5685308	12.189678	13.45230925	13.91306201	15.44459426
MYO1A	0.411084633	0.337996522	0.225050506	0.085961805	0.044404741	0.029115099	0.291544615	0.083223807	0.138836753	0.124350631	0.152691527	0.132768727
MYO1B	12.42134092	9.12720817	12.5380738	4.316126807	17.73462642	9.662319717	12.31230044	3.854282124	1.230795039	1.486067772	1.144741022	0.436302346
MYO1C	12.66430741	12.87252808	11.8716288	11.15118239	12.59227299	11.3965285	12.55218726	11.95271687	7.369644409	6.280268046	7.274825544	7.772326729
MYO1D	0.020837574	0.05040517	0.014703184	0.077221744	0.021758151	0.057065144	0.014285573	0.101948357	0.01511766	0.03808208	0.022445477	0.050599237
MYO1E	0.10406209	0.09807335	0.150191934	0.047806934	0.079025033	0.038861082	0.048642025	0.101825283	2.707598735	2.354780762	2.934769806	2.047779704
MYO1F	108.6557973	108.3440931	100.6484215	97.13398199	80.17952951	81.8260598	96.66095622	102.2984818	99.71627047	71.04062478	85.0245118	147.0415329
MYO1G	120.0933795	99.55267961	104.1333178	39.27909652	44.85371451	47.51020867	78.65102048	41.64819399	95.91653667	80.63512308	87.99175578	90.71923831
MYO1H	0	0	0	0	0	0	0	0	0	0.009347968	0	0
MYO3A	0	0	0	0	0	0	0	0	0	0.012892904	0.006332536	0
MYO3B	0.023311001	0.016110938	0.041121142	0.015706908	0	0	0.015981276	0	0.059192462	0.017040975	0.041849606	0.048518902
MYO5A	6.345589839	5.350887056	5.563982826	5.602470214	4.596937015	3.835180947	5.379188494	3.968280508	6.933909368	5.674046316	6.283934501	6.730159452
MYO5B	0.01130453	0	0.005982435	0.005712728	0	0	0	0	0.012302155	0.037187631	0.018265243	0.029411186
MYO5C	0.046287794	0.047986341	0.02449582	0.038985784	0.01611091	0.015845293	0.015866715	0.022646421	0.134327185	0.109972311	0.083099217	0.176627351
MYO6	16.42446551	9.316392566	14.36454109	10.62336001	14.56765803	7.456075524	16.329855	9.362517289	21.79113298	12.14993374	15.79275088	19.95319126
MYO7A	0.030580564	0.082427095	0.019420135	0.043270764	0.127726298	0.157025627	0.044026617	0.029923237	0.139773062	0.013413134	0.111996927	0.05728462
MYO7B	0	0	0	0	0	0	0	0	0.04925691	0.132352445	0.113761931	0.094208157
MYO9A	3.463192767	3.244732868	2.869580384	3.307150682	3.336064641	3.306109925	2.880037065	2.529631547	4.79064756	5.620525461	4.05442949	4.632028571
MYO9B	36.38833612	38.16269115	32.92547648	35.73597134	36.18273573	36.05739485	31.5402472	35.41277234	27.68515787	24.29682947	25.6086866	36.15723207
MYOC	0	0	0	0	0	0	0	0	0	0	0	0
MYOCD	0	0	0	0	0	0	0	0	0	0	0	0
MYOD1	0	0	0	0	0	0	0	0	0	0	0	0
MYOF	6.152201089	5.016888832	4.88703734	0.839752132	2.503372216	1.713048162	4.48081805	0.856447915	14.73619505	12.65770407	14.92083461	8.996522762
MYOG	0	0	0	0	0	0	0	0	0	0	0	0
MYOM1	0.208143273	0.225162953	0.124518358	0.08231862	0.311833898	0.185874398	0.148900553	0.044275924	0.482568886	0.248084319	0.370424611	0.555656749
MYOM2	0.246980623	0.356234663	0.193214385	0.206209979	0.224253836	0.165417457	0.187726572	0.199641915	0.116859423	0.08242479	0.057834468	0.122927138
MYOM3	0	0	0	0	0	0	0	0	0.010096251	0	0.009993397	0
MYORG	0.188023572	0.317652563	0.176894882	0.37303144	0.152701278	0.200244964	0.229160783	0.190796168	0.606271589	0.450531653	0.487577422	0.789941759
MYOT	0	0	0	0	0	0.018562465	0	0	0	0	0	0
MYOZ1	0.951779977	1.23338235	1.295199069	0.274846083	0.567901944	0.418904297	1.153544245	0.465660769	4.845945468	2.497342439	3.515049622	2.334761417
MYOZ2	0	0	0	0.032644159	0	0	0	0.031604399	0	0	0	0
MYOZ3	0	0	0	0	0	0	0	0	0.106830284	0.030755473	0.060423981	0.131350036
MYPN	0	0.008162734	0	0	0	0	0	0	0	0	0.008481373	0
MYPOP	4.601895104	5.033718726	5.004941387	4.614499154	4.407920566	4.111973516	4.769630632	5.389390643	3.391252598	2.999885699	3.122516022	4.468598998
MYRF	0.026121146	0.036106228	0.027646981	0.017600378	0.009091715	0	0.026861732	0.017039783	0.341116061	0.811548838	0.440809073	0.217471452
MYRFL	0	0.008570306	0	0.01671076	0	0	0	0.02426775	0.026989518	0.045325222	0.008904855	0.180669362
MYRIP	0	0	0	0	0	0	0	0	0.02106791	0.053071033	0.020853284	0
MYSM1	17.37745991	16.22120842	15.37055891	13.4533503	15.51825867	15.67934347	15.77582708	10.30916333	16.26738966	17.07733284	14.74671916	15.60488795
MYT1	0	0.010078432	0.010289572	0.019651371	0.040604733	0.079870581	0.00999732	0.028538173	0.021159262	0.042640921	0	0.010117236
MYT1L	0	0	0	0	0	0	0	0	0	0	0	0
MYZAP	0	0	0	0	0	0	0	0	0.170937364	0.196845286	0.072512557	0.070057004
MZB1	0	0	0	0	0	0.066820385	0	0.191002158	0.07080805	0.07134749	0	0.135426599
MZF1	3.558203508	3.633921399	3.234044366	3.582897046	4.143569221	4.863137701	2.870137764	4.374800829	3.62750265	3.17648918	3.063364377	3.661678243
MZT1	16.84337415	14.61673504	17.92170854	18.60189224	25.32133089	21.32980387	23.26279446	18.83892087	29.74040833	44.20435508	35.99777449	23.14714019
MZT2A	5.791767502	8.954084615	7.573508152	7.45328656	8.420987655	9.821007655	8.830079981	10.3131205	8.080160001	10.70792776	11.6612564	6.377847052
MZT2B	29.37685214	43.49270505	35.51292964	48.32080992	43.00470404	50.13370773	39.24304948	58.24231144	40.22288844	54.9514423	59.04751613	32.77009602
N4BP1	5.819090466	5.977710692	5.654431669	5.613656835	5.728509212	5.284363919	5.548301884	5.368214461	7.263167308	5.907905293	6.317018751	7.859218259
N4BP2	3.692602033	3.605020422	3.366240533	3.688604573	3.905169	3.332511058	3.337016442	2.425664778	4.107461525	3.001422226	3.620578306	3.9234612
N4BP2L1	8.304744682	8.753461629	7.535562426	4.7348409	6.844503343	7.653934815	6.997823192	4.457199025	7.576704974	8.131882975	6.302786771	6.031540033
N4BP2L2	17.77032117	16.65366424	16.98606189	17.02341603	19.66171888	18.57749888	17.08043694	15.23862802	18.45683012	18.28990379	16.51493163	18.09606808
N4BP3	0	0	0	0	0	0	0	0	0.055416161	0.05583834	0.054851617	0.010598826
N6AMT1	4.493152466	3.647855041	5.099393899	5.526058379	5.087335892	5.503808248	4.342196272	5.879753565	4.005997465	3.324190104	3.090513374	4.901312071
NAA10	152.4283356	137.2149684	170.1961023	119.0344433	117.1479674	123.6687543	154.027766	133.0451901	49.18265061	48.66013698	50.70352968	62.05960256
NAA11	0	0	0	0	0	0	0	0	0	0	0	0
NAA15	25.55690322	20.91531609	26.57254924	27.67253899	31.24382687	28.34450692	30.9872994	26.07770278	25.40064886	26.10961974	27.68397292	22.2937317
NAA16	5.87956989	5.332556133	6.403798264	7.443019656	9.479935992	8.06879605	7.02583013	6.691698008	6.448485883	5.251396521	5.625632329	5.783795436
NAA20	54.29396801	58.88091465	58.87541833	56.2619261	53.15195279	60.23514351	54.58795631	54.03541331	30.4871946	35.85412095	36.17714737	34.44793477
NAA25	13.40547513	12.69628001	13.54085839	14.43948904	18.18935786	17.47051647	14.80079396	12.6941859	6.037849098	7.810586627	6.934311476	6.342862385
NAA30	4.942062461	4.512592638	4.632583775	5.274449871	5.599846299	5.272897387	5.37894928	4.353426476	8.571096844	7.410158298	7.292165175	9.122502179
NAA35	7.124737374	6.332088858	6.633857506	7.70010013	7.113404929	6.899166443	7.505985115	6.666007122	7.287179513	7.88423952	7.682331353	8.094860393
NAA38	7.857233271	8.42437825	7.685880519	9.416924945	8.766023207	9.035805078	7.64538021	11.33513594	4.975674824	5.856199953	5.400929902	5.178048839
NAA40	8.549816732	9.102730577	9.264702129	9.656282142	8.814171185	9.797755958	8.87595631	8.790980719	18.94835909	15.50631982	16.19711668	20.16807756
NAA50	29.26331638	28.09128985	31.80479995	34.03487418	35.34477998	34.89662075	35.16837374	30.80538209	28.41406337	29.13819915	30.46753189	30.31758565
NAA60	9.123843343	9.251270079	9.268647597	10.53558202	8.656619533	8.251406762	9.851076975	11.70065074	10.01367965	9.236950796	9.588460271	11.68089381
NAAA	9.051461776	8.729888185	9.204770806	9.81216787	7.873833597	7.757510663	9.010878168	9.602474965	9.249802812	11.48107577	10.76876379	10.78689726
NAALAD2	0.010809474	0	0	0	0	0	0.011115944	0	0	0	0.011643572	0
NAALADL1	0	0.017351204	0.017714706	0	0.034952907	0.017188323	0.017211561	0.016377272	0.036428154	0.238586895	0.018028524	0.069672036
NAALADL2	0.034585155	0.028171212	0.013073359	0.037451911	0.028374565	0.045665607	0.02540408	0.031424482	0.137107295	0.132734108	0.101117654	0.161965413
NAB1	6.724901857	6.853692322	6.624963024	6.451768703	8.015882443	6.938104104	6.224009461	5.466752797	10.4371283	10.53933162	9.784729055	9.238041762
NAB2	12.40537644	13.33386605	12.43675637	10.47180303	13.98970457	13.20869685	11.55282369	12.8767582	13.08974688	10.25122107	11.5666845	13.4884843
NABP1	15.49613288	13.12447875	10.55335141	2.717770599	6.097236036	4.172502252	8.739787595	2.064632426	4.57102572	2.970127179	3.35105965	4.258898562
NABP2	24.15939467	25.29208386	28.33502964	32.57710596	29.50349272	30.35820007	30.49080679	37.98677896	15.05137771	17.9649281	22.34706611	15.50548661
NACA	130.6968559	141.6746304	126.7366512	117.8173714	147.4841412	147.3124	145.0321969	126.0998757	60.04383193	71.61997578	66.67461405	51.46838083
NACA2	1.188003292	2.752982629	1.701186974	1.130081425	1.167517894	1.363569757	1.79659636	1.641130233	0.532347791	0.383145288	0.677474458	0.436355056
NACAD	0	0	0	0	0.006587736	0	0	0.0061734	0.006865782	0.013836176	0	0.006565705
NACC1	28.12306715	28.48224411	29.5368741	29.55165319	24.72884916	25.60736442	29.15458485	34.11410633	18.54881736	19.58905276	20.0768745	24.1247901
NACC2	20.42401091	13.69954984	21.19494184	13.2629737	11.30783348	9.183821934	17.95080417	12.68293171	12.67816312	8.048344677	13.01164243	19.13189289
NADK	27.80213121	25.10122269	25.59376012	22.68965782	15.0138315	15.34833099	21.48865209	22.11073819	12.14110147	10.79502694	11.31649412	21.89931581
NADK2	11.10220011	11.73801469	11.01225298	15.41490448	14.55795351	14.30536894	12.92748349	13.01950252	17.61010369	18.85831346	18.32729028	19.8976979
NADSYN1	36.82405213	37.16648634	32.51434987	30.5790837	27.78163533	31.23022217	30.8858027	34.38779413	22.76864414	22.04479091	22.84639285	26.0775801
NAE1	43.80955398	49.83110507	48.29572137	59.42450228	56.04055501	61.21512537	56.37216218	57.12061563	52.21774823	55.22637432	54.22029228	61.45011908
NAF1	6.097300821	5.526345772	6.593354574	6.385158979	7.985940922	6.650800237	6.415146366	5.509287899	4.535460224	4.956483386	4.356381526	5.859390266
NAGA	31.27502783	36.67289482	30.57068605	36.94738438	20.76533272	30.26167969	24.38846288	37.69281059	37.05591293	41.00647921	38.62580199	45.06578049
NAGK	7.412832475	8.039534317	6.920436866	9.009764773	4.862509949	5.699771777	5.981123762	9.28075282	8.232492515	6.919622848	6.879241409	15.09260325
NAGLU	4.115165615	5.137276473	4.218724541	5.149986936	4.746912717	4.768219748	3.943807761	5.955736637	3.03635407	3.449304767	3.504391292	3.565095061
NAGPA	5.578265929	4.596066161	6.23928161	4.850103311	5.519481803	4.678001698	6.287517944	7.651240377	2.677403333	2.163582762	3.20114434	3.701149493
NAGS	0.022309105	0	0	0	0	0	0.022941612	0	0.072833653	0	0.048061114	0
NAIF1	3.59469403	4.022104125	4.08960525	3.889227772	3.852714562	3.756670373	3.550049028	4.385161482	3.860226481	3.472888425	4.042649725	3.675030736
NAIP	0.416131306	0.533368993	0.368367258	0.290584422	0.268609555	0.753692981	0.536856944	0.222101779	0.296414125	0.34845103	0.244495375	0.283458993
NALCN	0	0	0	0	0	0	0	0	0	0	0	0
NAMPT	12.11948206	10.64062033	12.11893535	10.24321268	12.34834269	12.54274922	12.0645641	9.790548872	7.22073663	10.94581366	7.982067876	5.010813733
NANOG	0	0	0	0.051721511	0	0	0	0.025037056	0	0.056114479	0.027561438	0.026628102
NANOGNB	0	0	0	0	0	0	0	0	0	0	0	0
NANOGP8	0.02691824	0	0	0.027206187	0	0	0	0	0.058587551	0.118067783	0.02899535	0.084040368
NANOS1	0.897495787	1.235943946	1.034989553	1.408030255	1.188916575	1.458204782	1.005593043	1.009279433	0.801768518	0.660990004	0.894604338	1.268583469
NANOS2	0	0	0	0	0	0	0	0	0	0	0	0
NANOS3	0.258389248	0	0.068370686	0	0	0	0.066428773	0	0	0.070833583	0.139163754	0.067225569

NANP	3.68073818	3.933213527	3.161546134	4.778912474	5.011135158	5.422823216	4.149045737	4.903745495	3.759067278	5.277949823	4.452727563	4.80285215
NANS	18.10803538	16.33106321	17.15495693	22.64215184	16.2629682	15.42577714	18.7638929	22.19207645	15.26951242	15.12543175	17.82125203	15.85845977
NAP1L1	189.4811748	211.0879181	195.2727677	202.8719012	235.1755182	217.7829478	221.6968176	208.7439175	89.35809471	93.2005931	92.39517198	78.21908755
NAP1L2	0	0.043310653	0.022108999	0	0	0	0	0	0	0.04581085	0	0
NAP1L3	0.058531067	0.020226295	0	0	0	0	0	0	4.31012552	5.09174804	2.774091691	5.603950718
NAP1L4	41.39361374	44.26396948	47.20431079	56.5882215	52.61654931	53.61449574	48.127092	57.04415745	60.62802866	63.29261919	68.2511721	65.58409558
NAP1L5	3.335590514	3.794184773	3.800121008	3.81609233	3.434578306	3.18764591	5.026138796	3.195894487	7.864905813	9.296427469	9.25690575	10.96835944
NAPA	31.95460076	27.74902464	30.55005602	33.91403334	25.5289762	25.81655269	29.65397458	40.09723003	45.61538076	37.4904717	45.0615091	49.73850912
NAPB	5.309899043	5.183302544	5.059430752	5.340584383	5.180247461	5.479608497	5.646445702	5.094629079	2.980636374	4.306681825	3.117268096	3.509174693
NAPEPLD	0.974920414	0.853998492	0.823895572	0.947157431	1.515149626	1.241808058	1.398922783	1.042705463	2.13836332	1.864604526	1.611856366	2.611184609
NAPG	8.608489437	8.89741912	8.794786673	9.502290212	8.907332021	8.53345409	9.467689429	8.588865785	10.99559346	10.50899562	9.777010429	11.32698848
NAPRT	15.03200534	17.94834607	16.24808047	20.9102789	11.48030392	11.80638877	9.242075517	15.02137379	43.21743347	42.87134279	41.59778054	62.57443719
NAPSA	0.395243364	0.380479319	0.298807877	0.256803	0.206352356	0.289928954	0.290320923	0.248623481	0.430123609	0.71201501	0.547382301	0.646367143
NARF	6.650508352	8.489559196	6.734317066	7.464040848	8.219171982	8.002113996	5.920383208	7.760503548	8.630888654	8.914329938	9.355559989	8.55323524
NARFL	8.011109509	7.754743375	7.278718687	9.389367673	8.868947587	8.251898073	8.436752053	10.43614095	7.851533122	9.313694964	8.759235037	10.49665458
NARS	90.76966327	84.19108301	92.56066791	91.53226565	93.68380531	93.31337622	95.88730763	86.15933726	66.02336402	73.5345355	83.25812117	72.24657656
NARS2	12.04722503	11.73951563	11.679257	12.99062015	13.16203808	13.5180154	12.98378939	12.51619149	5.262147524	6.79773898	5.631455847	5.440753228
NASP	59.69598131	60.62626306	67.8726814	90.72817068	91.02795854	85.53543631	75.74864687	91.84523882	144.2719484	137.9165861	151.1587474	135.1531093
NAT1	3.718698055	3.251182398	3.778484454	4.848435901	3.714722692	3.487990244	4.168302211	4.305871816	6.555934792	4.471881861	5.113875259	6.462899866
NAT10	49.77484249	46.1071434	50.92066292	47.49449641	58.24461754	57.12025128	55.11607293	50.9590702	27.17068349	28.75021358	33.83609476	27.81237069
NAT14	6.656441973	6.900702462	6.581210284	3.786819147	9.530945842	9.37381112	8.894615874	8.580477208	9.976591944	12.19423737	10.77658342	8.545017156
NAT16	0.01444552	0.029951206	0.015289337	0	0.015083704	0.029670045	0	0	0	0.0158401	0	0
NAT2	0	0	0	0	0	0	0	0	0.131962269	0.33241901	0.326544809	0.157743377
NAT6	8.962470434	11.56016983	8.677118391	11.97246126	8.088867718	10.55977775	7.108907946	12.23696204	11.07654572	15.57959271	13.5456015	15.36444933
NAT8	0	0	0	0	0	0	0	0	0	0	0	0
NAT8L	7.671368155	6.622642279	8.284393669	6.345399504	8.316526658	8.273538252	7.153703359	7.058057331	4.219067065	4.361760938	5.153467776	5.370018744
NAT9	15.46187589	13.32877873	14.4548207	9.87282625	11.5987271	12.97436078	14.42694211	10.96025686	8.828284951	9.119970724	9.259442286	11.26948794
NATD1	0.741890749	0.675888669	0.523484934	0.636216721	0.422545383	0.634912309	0.393021873	0.57195577	1.100949057	0.825839374	0.738597018	2.374718292
NAV1	0.30722174	0.515013757	0.221390792	0.257655682	0.204762386	0.231594487	0.131078204	0.124724502	0.928310562	1.308102322	1.383561075	1.261879948
NAV2	0.004779036	0.004954405	0.010116396	0.009660317	0.007485252	0.007361844	0.002457266	0.018705246	0.975147493	1.014018956	0.913735281	1.004642974
NAV3	1.698847434	3.567605614	1.61336871	1.333929033	0.062641746	0.366853498	0.263594273	0.400240169	0.050447999	0.023921096	0.023498385	0.059594429
NAXD	19.26468735	19.91315836	19.85290805	21.35135505	20.66528081	19.95784637	21.16381726	20.45059598	25.89408084	24.64870126	23.6462696	29.37839541
NAXE	22.44808268	22.36615138	24.6706048	21.37017213	21.81369829	23.26140244	26.1702794	24.34423627	29.33424219	30.75169157	35.24071582	32.82194282
NBAS	5.623241427	5.210644318	5.28306667	5.767592387	5.900664957	6.273926402	5.26865616	5.543128566	9.111244745	10.86301667	8.360364429	9.016465714
NBDY	2.062509673	1.892529328	2.108674173	1.428153995	1.044739038	1.171727225	2.084883996	1.657480862	5.052571846	5.110311985	4.45277482	4.850010648
NBEA	0.004018938	0	0.004253699	0	0	0	0	0	0.380504023	0.277636536	0.125542565	0.543719236
NBEAL1	3.033174006	2.88344935	2.689755382	2.396865257	2.79726431	2.919522568	2.676585464	2.162955595	1.561213666	2.144562929	1.709301017	1.691027197
NBEAL2	16.55716963	23.33211397	15.32394798	15.54076879	19.16930889	21.28381147	14.86319447	15.57011905	14.92979076	13.99891703	12.34057849	22.89009853
NBL1	0	0	0	0	0.017124901	0	0	0	0.035695372	0.017983656	0.017665865	0
NBN	25.73235933	22.62513896	25.74209847	25.77165507	25.54427178	23.69496743	25.32411192	21.88160745	19.49732573	16.6516778	18.83946716	17.34582001
NBPF1	5.781947382	6.192974977	4.592186576	6.554654508	3.20467906	3.724053989	3.428507261	4.933698258	16.81897317	10.67707742	12.12168034	22.06305283
NBPF10	0.857335208	0.703222754	0.777784635	0.76176372	0.742730158	0.536978981	0.673342234	0.539297261	1.948011703	1.528958663	1.334494271	1.838360069
NBPF11	4.328985498	4.628376909	3.682704199	5.416594723	4.718407678	5.002584341	3.847625042	4.934553914	12.71679986	12.75422066	10.35795309	13.07330692
NBPF12	4.205391907	4.134742993	3.944164245	5.112553744	4.281791913	4.264991587	3.80135905	4.444489986	12.69535847	10.92126578	10.02702814	11.8056363
NBPF14	2.327477037	3.309415156	2.254380927	3.355910205	2.335542522	2.236732847	2.903998394	2.914715675	4.444153438	4.647765202	3.484602422	5.960993894
NBPF15	4.75119821	4.542189001	4.044335365	4.167793012	3.404905956	3.578925992	2.465998735	3.574521998	8.072795955	8.736382791	6.831804305	11.19511514
NBPF19	2.0153088	2.630608317	1.999176478	2.684211126	2.151200402	2.543070383	1.795561171	2.407105063	2.863538727	2.527480781	2.276282221	3.120484507
NBPF20	0.524971325	0.417247089	0.524768175	0.55122095	0.408077562	0.4223157	0.380897904	0.496564202	0.60938674	0.556464018	0.48065803	0.761827838
NBPF26	1.547452085	1.338503979	1.256015819	1.285746752	1.149906253	1.735420188	1.230104297	1.068293359	1.570369632	1.83217535	1.646406896	1.758610443
NBPF3	4.404521792	4.723902843	4.107042674	5.18645677	5.066963459	5.634569931	4.703271928	4.235396591	3.854816922	2.975711574	3.624313913	4.48695026
NBPF4	0.139845812	0.080543058	0.115122573	0.188455681	0.162248913	0.207446134	0.111852779	0.167248689	1.538781806	1.550504784	1.087932642	1.148114877
NBPF6	0.600883589	0.460428905	0.433206137	0.440080665	0.745641283	0.626028823	0.617919823	0.468669859	0.597049716	0.33422125	0.47840215	0.262820532
NBPF7	0	0	0	0	0	0	0	0	0	0	0	0
NBPF8	2.535529046	2.966749928	2.432538193	2.075598195	3.382974787	3.304359192	1.928878131	1.559710608	3.324056477	3.698951547	2.866940151	3.826874321
NBPF9	3.089186152	2.759286115	2.62476068	2.582053098	1.930932889	2.360150694	2.341356925	2.437054489	6.025660937	5.661325548	4.904983266	7.275184712
NBR1	10.64113851	9.889541745	9.397122544	10.33433671	10.18247261	9.704520931	10.17918693	9.038992245	28.21346416	22.49575328	19.72491098	31.99937076
NCALD	1.229023106	1.729637856	1.118835704	1.435253562	0.797919034	1.477972028	1.159091682	1.663707913	6.638912458	4.161728652	4.451733216	6.222836214
NCAM1	10.65842011	11.19345986	10.87359305	13.39379079	13.89679352	14.43887671	8.527079933	11.97303014	7.547264514	6.705199806	6.912988463	7.118893672
NCAM2	1.461344983	0.683916111	1.287995787	0.579102562	1.193828819	0.569528489	1.173030961	0.529795498	1.13266594	1.069243535	0.747418051	0.869811451
NCAN	0	0	0	0	0	0	0	0	0.018282316	0.009210798	0.018096067	0.034966531
NCAPD2	41.12411923	41.6117512	42.95751815	66.67445139	69.93537857	60.8370966	45.38727253	70.2148601	51.47911696	58.23090877	57.63607611	40.17565609
NCAPD3	23.88834755	22.73931065	27.57101679	32.88045752	32.00466241	33.21545635	25.90826635	31.63933494	50.35657347	59.24133971	55.05197958	54.96812928
NCAPG	13.69266195	13.6676373	16.86204431	26.58119398	30.73213634	24.30345446	14.545975	24.14135104	39.46130078	34.88932747	39.94839	26.01671892
NCAPG2	13.52648951	16.2761884	16.78828439	30.8282478	27.62916187	27.28433257	17.05036219	26.89333345	32.02083962	37.52406381	35.14853437	28.60234192
NCAPH	9.048017398	10.21606445	11.68695084	20.52878894	18.51610409	18.01971475	12.47592965	19.91827674	18.75754446	18.74496675	20.60924213	17.71638417
NCAPH2	8.805200868	12.05516604	11.05031887	15.35498137	16.89982151	14.98491396	11.12452466	19.31063241	17.52184731	15.40244058	20.39950869	18.74872223
NCBP1	23.24335852	20.38758534	25.57383844	23.65180519	25.54977289	22.12448112	24.33934381	21.90245779	23.20821281	20.76260445	24.04692168	24.34332297
NCBP2	19.67764671	18.58462333	20.29174475	23.12254457	21.97907307	22.56568617	20.86571949	23.0713482	18.0268821	18.91083798	17.74893818	19.35464472
NCBP2-AS2	13.46696485	12.63150751	15.981333	12.13796829	13.27055482	14.60839858	15.46746838	14.14726568	13.25967034	16.87665745	15.19689305	15.34964399
NCBP2L	0	0	0	0	0	0	0	0	0	0	0.033026612	0.031908204
NCBP3	10.14140994	10.33557223	9.987503467	12.9641947	10.79431888	10.93749049	10.58657097	11.29734093	6.42566107	6.440997109	5.587465963	7.714524692
NCCRP1	0	0	0	0	0	0	0	0	0	0	0.047862678	0
NCDN	13.80536838	13.23265012	13.44949063	11.31522856	9.247813928	9.856954311	12.64217194	13.06205696	8.784514577	9.414427076	9.370961896	11.13150653
NCEH1	7.519621135	5.94685886	6.889768677	7.43621077	4.804850219	5.583675535	6.360658926	6.174364248	4.410529211	3.610001119	4.499923691	6.021666063
NCF1	65.72216412	43.96966412	54.49354537	14.94753434	11.82271911	7.878118836	38.38189849	12.23395662	5.89997518	1.577224537	5.680962045	2.418047037
NCF2	6.389493839	10.3472294	5.679807837	5.043870895	3.183264494	4.288743389	5.497013185	3.820759642	9.771071221	7.624546627	6.635119356	10.73475205
NCF4	40.80969753	46.27961866	40.73032022	30.38821784	29.65209819	34.2088553	43.65893979	34.50388488	46.81590669	40.29001184	43.12310237	65.27088777
NCK1	5.69171716	5.554163224	5.470107747	4.401697562	6.012952782	5.307566296	5.69272996	4.991728663	8.181913376	7.902262514	7.71462957	7.314283827
NCK2	10.64233537	9.297353616	10.02650199	7.58706673	8.823408378	7.640952296	9.823700174	8.177454794	19.93872506	18.86683081	20.92345944	24.68099615
NCKAP1	1.555809835	1.457172471	1.533125718	1.149517023	1.669358524	1.69693133	2.217820288	1.354382404	0.513771348	1.023605298	0.566325719	0.48015008
NCKAP1L	113.1978569	115.3013253	92.98678438	110.3970672	84.85702059	103.5310645	90.38778753	96.77728347	181.5083314	154.383562	150.156809	183.9233795
NCKAP5	0	0	0.003262087	0	0	0	0	0	0	0	0	0
NCKAP5L	6.678774808	6.385677753	5.726977266	2.502323156	4.336495033	3.64985589	5.410321609	2.92082107	2.009884519	1.445005086	1.602280862	1.402569822
NCKIPSD	8.187583571	10.36987212	8.598674224	8.441953512	8.310715438	10.6375405	7.884510828	9.564226188	6.838013033	10.69010604	8.709980003	9.234071783
NCL	551.6420715	540.122521	609.4807027	705.9262941	713.5123714	723.8367537	671.476071	714.5837611	426.2661789	459.2872702	511.1168414	401.3494873
NCLN	35.44540856	37.64270093	41.22325612	38.05359239	37.38762598	38.57721806	38.16985194	45.41720579	20.70637539	19.58382529	23.74054243	24.28669092
NCMAP	0	0	0	0	0.008898764	0	0	0	0	0	0	0
NCOA1	3.923303621	3.719970484	3.656072292	5.673272983	3.645767648	3.11165006	3.559885593	4.385315834	8.263616796	5.804110426	5.276535834	11.24161624
NCOA2	4.543279722	3.475485179	4.280926928	3.693661838	4.085533052	3.301289796	4.150288401	2.924524908	9.323485712	6.438829033	7.234945022	8.915991688
NCOA3	13.23449703	11.78097262	11.2188322	9.1884237	9.065774262	8.117627791	10.82434757	7.22944518	12.37413456	10.92455772	9.474925891	12.14030451
NCOA4	67.65082468	62.13875584	64.30557791	68.21461618	58.65064894	63.32886457	64.34983878	65.50013964	59.51940435	51.77698896	49.24330923	83.2575495
NCOA5	19.74571138	17.03555944	20.89913324	24.18053443	21.93927595	18.02120153	19.19267425	26.39162868	23.64434575	12.41428668	20.51614758	25.05396278
NCOA6	8.333973602	7.690615384	7.985998854	10.25714537	9.675463479	8.94952076	8.218599657	8.953588365	8.27114373	7.560011063	7.842296928	8.518077683
NCOA7	10.0604736	11.07425631	8.222733421	10.42229406	5.558860109	7.913825733	6.401406723	7.102881398	7.268461926	7.017398975	6.885868456	9.030204149
NCOR1	20.72076243	17.71185889	18.56861488	20.81308584	22.43786054	19.76597952	18.66285736	18.12521469	15.09693811	11.54443929	12.88037859	15.32794787
NCOR2	29.81004835	32.81973155	26.74811967	28.51837971	24.63589617	28.22446122	25.42140355	29.05070924	9.824547126	8.05903683	9.724461035	11.0787147
NCR1	0	0	0	0.026503493	0	0	0.026966456	0	0	0	0	0
NCR2	0	0	0	0	0.034449294	0	0	0	0	0	0	0
NCR3	0.34083649	0.353343605	0.811678593	0.129180923	0.489354541	0.043753332	0.219062422	0.041688779	0.185457663	0.140152908	0.183568341	0.088676008
NCR3LG1	17.83398927	15.55205775	15.06396657	16.01049622	18.12002509	20.93408583	13.43675209	10.81024752	12.07846865	15.29743039	15.31702041	9.336484265
NCS1	6.712769569	5.923583382	7.616439815	2.963503199	6.201856216	6.099607502	7.797730244	3.447137822	8.088286205	12.43685016	10.52797412	7.198261647
NCSTN	45.02546826	43.05252335	44.30976721	43.44310452	35.21350962	35.25062785	39.38333498	43.34593813	53.44373292	50.15448508	43.60311162	68.38648992
NDC1	15.80173109	15.14712395	17.96238039	23.39776592	27.477316	26.27515823	20.08878403	22.52655187	38.15159172	39.40682896	43.40194556	32.67718838
NDC80	12.93213203	13.58646426	16.44078908	30.67303478	27.26560118	23.68668827	16.73812399	31.10207506	45.99465311	47.86634788	49.82251415	32.87228178
NDE1	4.695592206	3.816808327	4.418336823	5.409893087	5.985372654	5.26428579	5.108309708	5.259750419	8.839214803	9.614607525	8.749166637	7.444906235
NDEL1	9.825658357	9.368721997	9.445762519	9.956065989	9.161885518	9.139378004	8.726969952	9.724685994	3.813982781	3.925389873	2.979654922	4.19438135
NDFIP1	6.597168846	8.281546007	6.713366492	11.82352011	4.514313262	6.91331842	6.230398385	11.25663105	23.14980304	29.05108339	22.62391769	35.29316199
NDFIP2	18.42953518	17.92548898	17.27616502	17.33614629	18.87707551	17.95391209	19.82743211	15.13888823	12.94604141	13.05633674	12.37861182	14.46204506
NDN	0	0	0	0	0	0	0	0	0	0	0	0.029519715
NDNF	0	0	0	0	0	0	0	0	0.173469234	0.30588387	0.079718805	0.272529554
NDOR1	12.22825967	11.08108705	13.31076802	15.60266272	14.68467002	12.69195274	13.64820853	17.88854943	13.34526304	12.56954346	14.06182596	20.60020911
NDP	0	0	0	0	0	0	0	0	0	0	0	0
NDRG1	11.45319425	43.0625432	9.286485435	8.495744696	13.60356926	17.64301984	6.498885167	9.095688302	39.45059318	70.58771124	43.06922488	40.14212209
NDRG2	0.249179053	0.312706507	0.194330719	0.026509953	0.397128238	0.35017595	0.728271768	0.11549509	10.19024995	11.87853113	10.97641817	9.362721083
NDRG3	14.2874169	15.04050971	15.15314635	14.96075461	16.68549721	16.3499634	16.47798683	15.26171835	10.2801206	14.74705993	11.7127971	13.07712869
NDRG4	0.041891445	0.0325715	0.022169241	0.010584891	0	0.032265741	0	0	0	0	0	0
NDST1	6.765721826	6.267477686	6.127054904	5.518574157	5.1129431	4.797149251	6.271769769	5.330546069	2.010203739	3.374719264	2.66758059	2.179744186
NDST2	10.11767602	8.945167306	8.249490206	10.89838217	8.149567063	8.850350185	8.406166818	10.32390096	10.97608155	9.438383728	9.396735471	15.27742284
NDST3	0	0	0	0	0	0	0	0	0.153627089	0.114992409	0.11296037	0.260245239
NDST4	0	0	0	0	0	0	0	0	0	0	0	0.007725052
NDUFA1	139.7277074	129.2446935	140.3897778	150.9580091	113.2984828	120.4132214	133.1004347	148.5332608	161.4569228	179.8055111	182.9490811	123.4049808
NDUFA10	10.39224256	10.6823432	10.84624764	11.20236619	9.938872897	10.89197261	11.68898043	11.68219545	8.757397948	10.76128384	9.29798097	9.912178028
NDUFA11	29.3680286	32.02935021	34.90328364	30.33835523	27.35567343	29.96379726	32.22321167	35.31370991	21.40347934	19.49363823	24.25012371	17.70582642
NDUFA12	147.307134	145.6319609	158.9402662	175.0959888	133.0942863	139.9994808	159.4564014	171.5961643	68.80344097	68.51794414	72.67582397	72.80817379
NDUFA13	227.0525197	218.813732	250.7183325	243.9305256	201.9407449	226.8533585	256.9716191	269.0933791	171.4611697	163.7316956	185.6485564	174.2991556
NDUFA2	39.88729414	42.64995592	45.45907641	48.75669061	38.23319027	38.81814877	45.09851975	55.85433535	43.63461328	48.31794174	46.639301	43.24882614
NDUFA3	50.5293412	48.76588589	62.12359154	61.22740122	43.72513512	50.09994882	51.89017688	61.80402093	54.0771453	59.69314136	61.87092362	61.01047344
NDUFA4	46.5931183	50.905044	58.80692919	65.19349414	41.88073328	45.51335144	54.44786927	59.12581018	54.14789271	57.16944725	58.2151805	50.17588409
NDUFA4L2	1.313533051	1.502602605	1.534081651	2.83828321	2.412059537	2.511839026	2.049450662	3.279727859	0.837954857	0.5463368	1.02457557	0.942742405
NDUFA5	7.578811389	8.090292367	8.061228981	10.07919245	10.22504145	10.46488673	10.27068867	9.662704459	11.04040769	12.51508205	11.4533167	11.61990817
NDUFA6	24.05351685	27.7809371	33.03715123	36.53123277	31.29447987	31.1307922	33.55383487	40.3182981	49.6462036	50.30651837	59.11631927	48.77035572
NDUFA7	82.66396306	89.63861584	93.68319823	98.72090387	90.59103402	93.20196907	97.03703353	117.7060028	50.60197214	61.99734986	60.58678082	51.63666578
NDUFA8	27.44168292	28.44866517	32.833089	29.82542136	24.4182096	27.03800707	29.61024504	29.69267133	28.65156406	30.39619285	31.8725111	28.18569853
NDUFA9	34.34581666	30.33629987	39.856328	39.1748977	40.92661777	38.46595003	39.9623773	39.53060125	16.81411732	20.13262991	21.61405979	14.44346987
NDUFAB1	74.27529294	69.13478472	80.23490147	74.2959095	77.04787393	72.27467479	88.26424579	80.85255617	61.05815678	67.78251542	79.10685299	60.7801761
NDUFAF1	14.00182908	15.10360607	17.37097348	14.07995472	13.10285027	14.99822723	19.39282563	14.11708948	11.68850586	10.92241712	11.68397979	10.14472805
NDUFAF2	22.07763624	17.53436927	23.60490797	17.39720275	24.69405893	24.74807741	28.70656136	21.60307908	11.15783201	14.60748071	15.15549414	8.48940191
NDUFAF3	20.21434182	16.40903284	19.78041075	19.33837655	19.34843686	18.34399156	19.28396282	20.49514458	35.4877565	33.56244093	36.83803493	35.85517209

NDUFAF4	15.02025486	13.11887059	18.40158301	18.51937927	17.78122246	18.3131922	19.41665368	20.48463159	6.387731899	8.002667114	8.774791305	6.596303306
NDUFAF5	3.777900857	2.928578179	4.178698691	4.076307703	3.62495475	3.512761598	4.270012955	3.680043377	1.981572899	2.425859793	2.584630049	1.8949659
NDUFAF6	4.189552184	3.215704563	4.285049239	4.071508037	4.017098461	3.857786084	4.39118682	4.257171072	3.16738467	2.76082605	2.918154274	3.626355792
NDUFAF7	7.07112819	6.577111418	6.910479416	6.36236748	8.309677636	6.882254913	7.676994445	5.954795465	4.812823783	5.119656108	4.790333109	5.038362464
NDUFAF8	15.97980898	16.29572525	17.39648593	15.49153287	13.14430562	13.9323338	18.98163941	17.48715438	14.76377875	16.37818391	21.4282662	13.64336808
NDUFB1	63.67464702	66.67530736	71.19097663	80.93031042	64.48104033	64.99682508	76.28348143	77.34965899	97.17882184	88.50656152	107.9191721	89.59823814
NDUFB10	86.31146204	93.48042201	100.0146403	102.6841921	87.06132356	91.88934215	97.49151529	117.8458654	86.28311335	82.70770981	98.29373562	92.6351942
NDUFB11	55.86811168	58.61303178	57.05412077	58.11084296	51.68785568	48.77079617	57.05823805	69.18894585	57.20370663	56.58960458	65.64523911	52.26231751
NDUFB2	85.11694173	78.99933194	97.33748077	100.9696362	66.62959757	62.96343158	91.10739762	94.66890143	61.87064332	52.56753351	75.05747888	60.63719147
NDUFB3	30.04092048	29.36366651	32.52947202	30.62919311	27.2316386	29.52874811	32.86162117	32.49622447	24.50106216	27.33024701	29.37200443	20.09776431
NDUFB4	51.84344229	50.70633626	57.39087962	54.52461775	58.67198395	59.4758397	61.25886854	58.49200307	46.28354549	55.978509	52.27699811	41.71200029
NDUFB5	79.01215027	82.43829127	73.19427323	71.69187475	63.93304282	68.56680128	77.69914508	73.08763422	70.33626344	84.1884888	78.86951367	83.65463452
NDUFB6	15.93662952	21.6398335	21.89473939	33.91966961	26.03778473	27.27722984	26.60715389	32.1054441	28.29295579	33.78531539	35.9483737	26.92629979
NDUFB7	37.88549654	41.41803012	45.8452735	42.63174737	37.57069065	36.86804691	45.79318486	53.28730095	22.97353308	23.72615655	25.57647466	23.10797934
NDUFB8	146.8305052	138.7270745	164.0910839	133.3037098	132.7568207	140.0977556	155.0082936	142.8992723	95.01092602	97.53404466	115.0091849	73.1930928
NDUFB9	247.9430809	272.480995	281.7538847	270.1104827	204.0385692	232.0324401	266.516949	292.0443368	179.3397525	187.9277013	215.6433342	184.8197315
NDUFC1	20.62761555	19.11940303	20.26468543	22.57003212	19.95346737	19.51040278	26.5060405	23.84420292	36.91628594	33.78640018	40.13040185	31.48733679
NDUFC2	2.141357623	1.802323869	1.772761908	2.33569085	1.970301449	2.199096263	2.681727994	2.344279315	3.137873507	2.324837445	2.809018735	3.155178303
NDUFC2-KCTI	0	0	0	0	0	0	0	0	0	0	0	0
NDUFS1	35.93277919	31.6156471	39.43857601	35.47186961	40.74829443	37.80655853	37.47371902	32.63224783	32.05020342	31.78670292	31.75587095	25.87792577
NDUFS2	67.84214444	65.76222437	71.10053005	58.16721978	64.4147551	64.77538206	71.1346441	62.68708718	35.57350488	35.25269708	37.90474502	29.69383268
NDUFS3	93.4793831	104.7042952	105.4725126	114.1578148	97.66777197	103.2027959	104.7848434	120.2946465	125.604103	101.7040989	128.5553115	110.7131514
NDUFS4	38.09874017	42.17256333	37.94872498	43.0431105	43.59017537	48.46094437	45.92992037	45.73502533	32.72911969	43.06888639	34.81328604	30.53954546
NDUFS5	198.1812723	197.9310518	245.3657478	233.2561785	202.3118353	204.4927302	242.4668827	224.7201162	145.7290425	179.6998371	189.7205326	131.5140514
NDUFS6	105.9437325	95.93642907	128.3002216	151.103115	115.2549716	123.6781692	131.3450139	168.6629517	151.7581204	171.3936588	178.6621745	153.9456908
NDUFS7	22.48167015	27.4304416	26.91589701	29.68282096	24.01215889	29.7947158	26.41597937	35.55379787	15.93714977	18.09843627	17.52281001	16.27036781
NDUFS8	43.3688998	43.64205541	49.01906052	53.1675902	42.55940344	47.69994807	50.51742938	63.39307788	50.98355714	54.74783988	56.23150355	63.7300901
NDUFV1	101.0207697	108.4558246	114.219833	110.0374349	100.2474457	115.5353548	109.4489587	126.9352018	80.24384092	92.14233076	92.50417819	74.98564995
NDUFV2	71.8157105	66.82833834	80.03457621	69.71266565	75.33742332	70.49140794	83.86199356	77.26244917	71.56109986	71.4191146	79.20232798	61.86834864
NDUFV3	13.17141823	11.46657825	12.75127223	12.03787863	10.60479312	10.20474591	12.68508688	12.32502321	10.85848475	9.889169211	10.11303286	13.82142969
NEB	0.015781665	0.016360779	0.016703532	0.011962863	0.016478879	0.016207195	0.004057277	0	0.17818438	0.084363038	0.135995492	0.110860452
NEBL	0.004590085	0	0	0.004639185	0	0.009427699	0	0	0.439574053	0.709683254	0.341154786	0.415585106
NECAB1	0	0	0	0	0	0	0	0	0	0	0.011276596	0
NECAB2	0	0.093128751	0.023769942	0	0.02345025	0.046127261	0	0.021975346	1.075360313	1.625329177	1.669180948	1.682771584
NECAB3	8.389786294	9.293382085	8.83120872	10.36129227	8.520424397	7.860629091	9.360412849	9.806354869	5.828310079	7.05733653	5.942250696	6.291204782
NECAP1	7.823449458	7.572699899	7.160280256	7.445713402	5.937208957	6.990138993	7.533094586	6.538466225	5.871633952	5.142687467	4.962398076	6.262892658
NECAP2	26.01516749	24.30273398	26.0333759	20.02385117	20.08464652	19.23498535	25.29395744	20.37418942	38.04455128	33.21962095	37.70877593	38.75884464
NECTIN1	0.264031211	0.31021592	0.33534513	0.68492934	0.496252392	0.551339217	0.543034032	1.222884498	5.50719624	6.842345668	5.915620393	12.28238851
NECTIN2	11.61985482	11.17242829	10.56215217	8.671201707	10.20006498	10.29467564	11.45399279	10.19183955	26.25703107	27.72792976	30.49677331	23.77799397
NECTIN3	0.021913892	0.030290706	0.027059626	0.003691385	0	0	0	0	1.037379019	1.389704602	0.940259769	0.935025393
NECTIN4	0.135578401	0.140553498	0.191330732	0.030450822	0.015729787	0.046411359	0.09294821	0.058961846	0.065574756	0.049555746	0.032453362	0.04703155
NEDD1	8.987436751	8.575036051	9.620676528	13.30879631	14.05671156	12.36762936	11.52980333	11.44628877	5.787653822	7.24762688	6.871677388	5.401651919
NEDD4	3.165664489	3.351655829	3.307809554	3.444598236	3.783764885	3.423948803	3.019919038	2.926261131	6.069124652	5.302934132	5.593750597	5.109925805
NEDD4L	0.643621802	1.075264027	0.82334285	1.071698099	0.464153917	0.551605989	0.228559337	0.403245041	2.443916589	2.528541298	2.21452512	3.42132503
NEDD8	49.07431951	44.62729736	53.94567445	48.11999718	40.81409053	41.46744811	56.1319943	51.97897084	35.50972708	44.9377315	43.7726786	35.57050054
NEDD8-MDP1	0	0	0	0	0	0	0	0	0	0	0	0
NEDD9	6.003466407	9.840143243	5.222031524	4.850253217	6.067449781	6.848180037	4.657508758	4.610902928	2.516830533	2.514871256	2.428910934	2.69765469
NEFH	4.410619743	4.6474275	5.663136007	10.80103187	11.65713747	10.30657303	7.078618627	11.75882382	4.42216346	6.485565469	5.732305011	5.432841301
NEFL	0.014009258	0	0.029655184	0	0	0	0	0	0	0.015361722	0	0
NEFM	0	0	0	0	0	0	0	0	0	0	0	0
NEGR1	0.040419243	0.047888505	0.030557346	0.052523502	0.048234187	0.07708831	0.041565208	0.067800745	0.527834917	0.715473152	0.572215553	0.817239
NEIL1	0.106185616	0.137602668	0.12643686	0.120736684	0.069297976	0.122679854	0.122845711	0.129878955	1.054453075	1.178923118	1.043710993	0.842607999
NEIL2	6.796614577	8.082197976	6.578132795	4.150978758	5.332148063	5.934760495	6.727679985	4.249932411	1.127261281	1.713737316	1.70302878	2.193810289
NEIL3	8.579959122	13.53960989	10.81201843	26.58126059	17.80106318	20.33732114	11.93322938	22.27118034	13.2865321	15.72139866	14.55075242	13.8948142
NEK1	3.357131089	3.30713081	3.334314222	4.317687099	3.630045979	3.823461651	3.223248986	3.588557061	3.861174253	4.038886062	3.556195597	4.942540302
NEK10	0.010416098	0.00809874	0.013780677	0.0131594	0.002719067	0.008022715	0.008033561	0.002548051	0.008501489	0.017132513	0.011219842	0.016259843
NEK11	0.050763737	0.026313265	0.053729039	0.025653381	0.053006414	0.060821259	0.087004981	0.066230099	0.331461832	0.194825764	0.33719859	0.237731174
NEK2	6.849385494	8.275666406	7.162559798	22.0628477	16.01906896	15.70436109	12.43183259	20.20290931	16.24830759	21.28912408	17.4805138	13.88006894
NEK3	10.86425662	11.22211665	9.832374826	11.83583589	13.25548102	13.15821453	11.7794688	11.42033156	11.15905338	9.388040542	9.158542787	12.45330303
NEK4	3.508239313	3.289370665	3.338565994	4.65028963	4.207850486	3.723991207	3.748181808	4.155850793	6.061245856	5.399315585	5.68514287	6.494218017
NEK5	0	0.024536671	0.012525353	0.01196067	0.049427577	0.036459506	0.024339199	0	0.064392189	0.051906201	0.050988963	0.061577852
NEK6	14.9387858	12.65192343	14.73140209	11.6470892	14.50486003	13.02218644	12.57808241	9.252537341	31.75915585	25.944022	32.05113676	29.79056464
NEK7	13.80418544	12.36862766	13.51406442	15.65329576	16.72172493	12.9706483	14.99456097	12.98372386	20.99754605	20.00260597	17.38944101	17.86969514
NEK8	3.367086993	3.077060094	2.769945473	2.967636721	1.816239932	1.589639526	2.182555554	2.685736557	2.188119926	0.979906573	1.54702056	2.540875303
NEK9	18.23826105	16.80215134	17.44574664	13.85883027	16.33165139	15.94113872	17.21736426	13.07080447	35.61808556	33.18780272	32.10956287	41.50766829
NELFA	8.083870854	8.237254745	8.61458351	8.5474379	10.18692343	9.678385399	9.22252798	10.30342357	5.774620111	7.091434822	7.174509801	6.025562048
NELFB	23.94556828	26.25517737	28.06689728	31.53621125	28.75943016	30.43298476	25.97935593	37.28473976	47.11060712	54.44088018	54.89804579	53.08251623
NELFCD	34.87699953	33.73668792	37.92734026	42.30481878	40.58612569	39.84506817	36.53801541	44.3381058	30.40964586	30.20614392	31.23142557	28.10877482
NELFE	47.78289004	51.97363708	52.04608014	62.55131084	54.14678563	59.40924376	53.18986599	70.11749963	55.96367583	61.80027393	58.9604284	64.3039663
NELL1	0	0	0	0	0	0	0	0	0	0	0	0
NELL2	0	0.025077151	0	0.0366724	0	0	0	0	1.237238967	1.114040783	1.042242367	1.069882311
NEMF	16.09852202	12.89861436	15.83448499	14.16500659	15.33320348	13.34465746	17.20027999	13.45183661	16.29906393	14.87266223	16.22314828	17.18890162
NEMP1	15.3472038	17.0351862	15.8442571	20.64474986	26.99330712	26.37871393	18.21597505	18.60239344	30.36575896	36.88204463	33.1560593	27.01758679
NEMP2	2.487446407	2.629957061	2.955302749	3.404796259	4.437836843	4.161663189	2.998415648	2.836955575	3.576418884	3.070792481	3.406902526	2.85437522
NENF	34.30430382	37.44413648	37.80848495	41.37628212	33.74760297	31.5607677	34.40226454	43.38734452	36.22096617	35.37775448	39.63872042	32.21854454
NEO1	0.02896491	0.090083362	0.038321075	0.080505565	0.030244542	0.066928291	0.037232652	0.077941355	1.371166294	1.206925703	0.616198976	3.579528589
NEPRO	11.44767734	10.03932483	10.23805087	9.112172996	10.21474169	9.832581594	9.710691106	8.768341891	11.02735908	9.922152527	10.05361798	10.6137978
NES	0.038725844	0.110403983	0.276668783	0	0.060655054	0	0.00995595	0.009473359	0.189645309	0.37156407	0.344141098	0.030226106
NET1	3.483952361	4.003507288	4.269694261	6.503307026	6.562088935	6.174289154	5.15981584	6.078683795	6.917662751	8.249888772	7.768927236	7.523189528
NETO1	0	0	0	0	0	0	0.004075955	0	0	0.008692453	0	0
NETO2	13.97165386	11.7568697	15.06625301	15.08677605	12.44900505	11.83136941	13.04349299	14.98562499	6.133095889	5.283973822	6.309264191	9.078565689
NEU1	36.79108555	46.03334813	39.32519424	47.05853314	21.70421965	26.33610459	35.29587483	46.06813429	49.42531789	45.98409474	50.60104308	67.84221979
NEU2	0	0	0	0	0	0	0	0	0	0	0	0
NEU3	11.4159294	10.71834653	11.02579318	10.68704186	10.89794893	13.2520532	9.605121935	8.909611234	15.02313364	15.56702016	14.06857945	15.85412784
NEU4	0.25465027	0.101536443	0.290258077	0.019798023	0.102269382	0.040233315	0.181294689	0.076669717	0.021317167	0	0	0
NEURL1	0.007286332	0	0	0	0	0	0	0	1.807892026	1.82166518	1.899354406	1.258743021
NEURL1B	0	0.01736741	0.026596878	0	0	0	0	0	4.904162874	6.649932601	4.403068398	5.029789015
NEURL2	0.111023853	0.076731942	0.039169726	0.187019149	0.347786236	0.190029087	0.190285997	0.325912211	0.040273904	0.08116145	0	0.192568428
NEURL3	0	0	0	0	0	0	0	0	0	0	0	0
NEURL4	11.24533718	10.80837523	11.1006867	11.03011324	11.81796748	12.28365312	11.70284927	12.97290195	6.096290498	5.403330984	6.581731917	6.499197436
NEUROD1	0	0	0	0	0	0	0	0	0.039012829	0.058965064	0.154461569	0.055961591
NEUROD2	0	0	0	0	0	0	0	0	0	0	0	0
NEUROD4	0	0	0	0	0	0	0	0	0	0	0	0
NEUROD6	0	0	0	0	0	0	0	0	0	0	0	0
NEUROG1	0	0	0	0	0	0	0	0	0	0	0	0
NEUROG2	0.305869826	0.195134663	0.199222672	0.09512054	0.171975335	0.096651436	0.193564208	0.138136235	0.435282191	0.670797435	0.506879787	0.4897149
NEUROG3	4.156479649	8.21039787	4.012852545	4.768636913	2.961830199	6.08557242	2.48372883	4.094617077	2.689523289	4.003428322	2.692375588	2.104342754
NEXMIF	0	0.004760956	0	0	0	0	0	0	1.194454505	1.495630211	1.15755216	1.390772345
NEXN	0.859472037	0.747078175	0.965657184	0.735025009	1.083834302	1.154229998	1.284967039	0.866874603	0.762645933	1.232429492	1.324594776	0.74307422
NF1	10.08016208	9.974672137	8.873638475	9.474339326	10.0551488	9.439286475	9.343547578	6.710667335	16.79464944	17.76360953	15.40400028	16.64763644
NF2	3.650953881	3.420687093	3.961229644	4.51601471	3.565010502	3.741552695	4.233591978	4.67112565	6.441823878	7.177679037	6.878067319	7.273100417
NFAM1	9.480451499	10.2473101	9.579759021	7.718251904	3.727617371	5.309016048	8.658295709	7.241734313	7.751570251	5.90874661	7.173792457	10.8913521
NFASC	0	0	0	0	0	0	0	0	0	0.005022931	0	0
NFAT5	6.768174927	5.286382186	5.437028351	4.166689455	4.515841286	3.737524328	5.384800441	2.756381804	7.287150318	6.647480462	4.975950613	7.000753943
NFATC1	8.132786731	7.30705925	8.748800946	4.806891338	7.022108296	5.314069199	8.519874708	5.519389517	5.072219544	4.401599076	4.62095251	4.028911407
NFATC2	2.688402057	2.846238462	2.197252365	1.841164562	1.338555099	1.444404347	2.57248755	1.376248416	8.014483678	6.704007811	7.552687273	7.756020325
NFATC2IP	23.41416796	24.60429407	23.51854601	26.86293471	26.76735029	26.69663113	22.16546704	26.1159611	27.62523915	27.62263076	27.9418169	30.07214531
NFATC3	16.08101819	17.11567994	16.20863063	19.11190676	19.91725138	19.96151737	15.81610545	17.27661602	20.43481246	18.28457394	19.373866	19.12115657
NFATC4	0.034235445	0.017745863	0.045294084	0	0	0	0	0	0.009314181	0.037540559	0.027657883	0
NFE2	19.28070916	30.12603856	22.64773364	24.81084507	11.02682371	19.46410531	22.08223093	26.58541299	2.194220405	1.851659523	1.737493693	4.301554758
NFE2L1	26.52096701	23.32464482	25.09492654	22.44467276	19.66594399	18.22029586	22.45871793	22.77712571	39.93303686	29.37770537	36.66133075	37.45636613
NFE2L2	23.33148928	20.1563721	25.29822205	20.66757802	16.8058728	15.91205169	21.66161845	19.84768587	34.45852794	23.08151028	27.86495966	36.95239837
NFE2L3	2.160502444	2.705777932	2.179276461	1.597408617	1.423217211	1.846482582	2.13229022	1.276950566	0.773202532	1.303788385	0.905895665	1.011028354
NFE4	7.215627184	8.292234306	8.465953978	7.292813669	7.00874827	6.950639799	8.167976963	7.553122774	0.121742738	0	0	0.058210914
NFIA	0.508885577	0.464252195	0.527839282	0.329170682	0.823619613	0.569641572	0.727405744	0.413296108	0.02215179	0.184144539	0.087704488	0.116509909
NFIB	0.018879223	0.031315204	0.01998203	0	0.01182797	0.019388274	0.003882897	0	0.008218126	0.004140367	0.004067203	0.007858943
NFIC	7.353705085	6.956170243	7.429478201	8.664840505	7.853095633	8.149535015	7.517638705	9.133856656	7.450297056	6.685759661	7.554424278	8.793109043
NFIL3	9.638898224	9.442723523	9.543752951	7.819485492	9.797358157	8.508833355	8.275823884	7.391454588	7.802459813	6.899219842	7.033422232	6.357454865
NFIX	2.764651623	3.106885085	3.568470006	3.294005617	4.412330051	4.724299855	3.937102588	3.804910861	5.422060839	6.309573865	5.617007205	6.970623164
NFKB1	27.38960517	21.73036643	28.31801251	21.64517611	18.04977469	16.78098464	24.81773047	21.44772231	11.21660942	7.35093429	9.516293059	10.36509095
NFKB2	21.53268553	21.35826808	21.48918241	10.43371287	4.996438248	5.221191948	15.5025038	9.732879711	5.014457583	5.550637933	4.796336943	4.011448496
NFKBIA	57.38083995	55.60612514	55.78973791	36.71953311	15.16720856	16.5040808	40.25659948	30.00578494	34.52956575	18.52594268	24.96754944	29.51824533
NFKBIB	18.37488738	14.42888687	20.18285936	17.09459135	17.54645443	15.34387478	19.79744295	21.51871205	7.354567248	6.769680424	9.286464402	6.5389074
NFKBID	4.533494793	5.727554171	5.719589585	5.119610344	4.418193557	4.38259759	4.425818864	5.500699095	2.565459476	2.744081215	2.799767757	2.440751825
NFKBIE	12.78003131	12.60059781	11.95986341	9.608538117	7.249207262	7.729184635	12.77790006	11.3017134	19.42110347	14.10526888	17.56143055	26.99055798
NFKBIL1	8.54385929	9.656280094	8.794700973	7.111385682	6.927139557	8.739823459	9.475199979	8.458565073	12.28775382	12.23440639	14.544562	11.95991426
NFKBIZ	8.461832301	8.386890825	7.707691156	5.235074604	1.539547735	1.500998875	4.284948668	4.064700685	10.40849649	7.605758047	7.775182073	8.93954306
NFRKB	10.05696407	10.49495286	10.09695697	10.16454469	10.43182529	11.13253127	10.67645027	10.15893495	14.24955116	16.05969101	14.35113316	16.3874833
NFS1	21.86224926	20.3489249	23.28258355	22.43815761	19.83617797	19.60182304	21.90205387	23.46753158	15.05082247	17.56524352	16.40425575	15.96614306
NFU1	10.5988529	10.842055	11.00968082	12.33185119	11.88905614	11.86627402	12.02687034	12.79185563	17.65314228	15.84547984	18.20009608	14.71653402
NFX1	16.79216532	14.8067771	15.87726636	12.54969823	15.88777194	15.71205826	16.15541309	12.09520285	25.29944116	21.84021138	25.25273254	21.48501441
NFXL1	7.913569263	12.81166431	7.443589397	7.203877785	7.881150047	9.685539955	6.967409705	6.218640993	5.308731439	5.453186952	5.035705808	4.512628463
NFYA	17.3914162	16.30736987	17.07026602	17.55996439	18.42175308	18.26020942	15.91809219	17.23771701	18.10701533	15.37016987	15.50864687	17.2525953
NFYB	12.39748383	13.34276702	12.68691381	18.11580911	18.04008247	17.79379096	14.92500711	17.22212368	9.414293005	7.779896687	8.46029031	9.300765525
NFYC	7.157493115	6.515244748	7.282130434	7.732242482	7.741768819	6.69663954	7.180899648	8.108952893	168.7014215	131.228431	154.3617509	166.5112295
NGB	0	0	0	0	0	0	0.029496358	0	0	0	0	0
NGDN	13.45996903	12.31964828	15.97886484	18.2305213	16.55532151	17.21635695	19.11011713	16.81927344	15.47257609	16.72067622	17.56811074	17.76190425
NGEF	0	0	0	0	0.015339011	0	0	0	0.415646924	0.805410515	1.076002143	0.183452576

NGF	0	0	0	0	0	0	0	0	0	0	0	0
NGFR	0.01573379	0.016311147	0.01665286	0.047706288	0.049286665	0.016158029	0	0.061582372	0.565035814	0.759120606	0.49148813	0.76957551
NGLY1	9.350366465	10.88963222	9.325601617	10.37422459	11.73492035	12.66481688	10.97149527	11.58335098	17.22013909	18.04143777	15.80186797	18.22966103
NGRN	22.17799543	15.7965415	21.19369623	23.06027905	22.86623606	19.5808	21.45314514	23.49671079	37.63529197	36.04121902	38.15418299	41.76048349
NHEJ1	5.398867019	5.782662164	6.499604143	6.930682446	6.358753245	6.963395873	5.920310401	7.010375621	7.685249144	5.386989955	6.724990819	7.16295948
NHLH1	0.126165442	0.087196751	0.111279369	0.191272574	0.109782725	0.172756416	0.108118734	0.185180287	0.137299548	0.069172772	0.135900829	0.175064944
NHLH2	0.035801438	0	0	0	0	0	0.024544319	0.011677296	0.038960916	0.013085911	0	0
NHLRC1	0	0	0	0	0	0	0	0	0	0	0	0
NHLRC2	8.48696818	6.448679279	6.432732241	6.685727435	7.810052665	6.914023053	5.706169418	4.608158002	8.231043621	10.16237586	7.161570873	8.351785848
NHLRC3	0.045396142	0.015687323	0.016015968	0	0	0	0	0	7.509157824	7.682515558	5.949387468	10.34625324
NHLRC4	0.459910777	0.34434643	0.243387954	0.129119584	0.160076349	0.104958136	0.315300102	0.125006952	0.500497924	0.308189989	0.275221765	0.478623067
NHP2	97.48656114	90.8204648	110.3085448	113.8590079	105.2763174	110.3916529	110.4114578	128.3992463	53.76401379	63.28305413	73.7571153	45.91254747
NHS	0.005416706	0.028077372	0.022932467	0.016423949	0	0.00556276	0	0.005300275	0.654315133	0.617722461	0.635980038	1.009039953
NHSL1	0.587434995	0.419349155	0.591752624	0.445289147	0.295932384	0.412766652	0.397427588	0.327741474	0.406557207	0.43225613	0.541179421	0.455820579
NHSL2	1.338156695	1.793171913	1.009221909	1.380405127	0.692625006	1.083306511	0.985294262	0.962325061	0.842168145	0.542992787	0.501145578	1.303916491
NICN1	8.037250801	7.070252796	6.635960883	4.348116981	6.250716055	5.925044519	6.344596255	4.699114768	7.04078207	6.198476264	5.711751399	5.934807376
NID1	0.036586477	0.066375803	0.096809079	0.009244462	0	0.018786489	0.018811888	0.017900027	37.95387288	52.03377429	43.36043818	28.55629721
NID2	0	0	0	0	0	0	0	0	0.215723916	0.228235745	0.245555201	0.061888634
NIF3L1	11.57235389	12.0165446	13.46519348	15.9821914	17.67276538	17.47817656	15.44733058	16.59812101	11.81420528	13.22690003	12.5465264	10.70942163
NIFK	51.85904168	46.66864957	52.13571637	47.31321917	55.8635144	53.09836928	58.00959486	49.80518652	25.90983871	23.28941358	29.94799188	26.77507764
NIM1K	0	0	0.023057806	0.022018287	0.022747692	0	0	0.04263395	0.782357281	0.525545035	0.844785964	0.838849902
NIN	19.69705745	16.54794231	18.48735995	19.25108718	17.54278172	16.01378094	17.74673881	13.91280929	14.1366393	11.2520611	11.97884239	16.39353913
NINJ1	18.91680278	19.65216104	19.81149187	22.89479033	11.86811256	11.67244546	17.12365978	22.7488649	15.2234169	16.77746243	16.01010131	26.42784074
NINJ2	6.463056383	6.202489852	6.606053562	5.897636677	4.241968325	3.830683813	5.81076231	7.15530529	6.832466507	5.062146049	7.399366425	5.188641965
NINL	0.066838244	0.024746749	0.060636445	0.072378452	0.049850766	0.073543331	0.044185654	0.051386941	1.589816934	1.732805244	1.558193258	1.137764869
NIP7	30.01274645	22.7705014	30.75941163	29.09137682	27.24575365	27.24912267	33.39191165	28.86832967	18.82950852	20.4989335	24.23398817	20.85476233
NIPA1	13.97300935	9.651525077	13.02592828	8.05583921	16.07408035	9.351253802	15.75486036	7.167958219	4.336990355	5.202844361	4.32799487	4.402402428
NIPA2	37.31291845	29.34048135	40.98483857	31.72684085	38.85428214	31.28968795	42.36475216	33.55087739	22.66726244	19.95860209	25.10109034	22.22347915
NIPAL1	0.04059567	0	0.010741754	0.010257482	0.021194568	0.020845137	0	0.009930767	0.02208912	0.033386104	0.032796136	0.021123689
NIPAL2	15.7327773	13.57305193	13.55964067	12.90458455	14.4958099	11.95661641	13.87551867	11.55124569	14.04785857	9.990284175	10.48975132	13.67035254
NIPAL3	3.776228474	4.554379597	3.523222328	3.830327485	2.690057135	3.035600345	3.395266065	3.058208386	6.935209983	6.415619889	5.009667218	9.475434187
NIPAL4	0	0	0	0	0	0	0	0	0	0	0	0
NIPBL	16.06230366	15.51040838	14.82192004	18.37849509	19.08422031	17.63898936	15.02555629	13.35618688	41.9184764	32.85318421	32.03182651	36.74584794
NIPSNAP1	10.70418175	13.31637058	10.87118356	6.102224997	10.07192948	12.54908938	14.6933794	9.650290842	34.00410189	46.554636	42.59679965	30.26493907
NIPSNAP2	28.16678111	31.56646407	32.36986921	31.26333045	32.15881139	35.15822588	33.71469061	32.63220408	45.35050647	59.94655997	49.92110804	41.80356919
NIPSNAP3A	26.24557498	26.22330507	25.53950934	34.42844576	26.79678089	28.87398319	28.03017906	31.1117303	25.69222675	22.86209376	21.86361699	27.8558625
NIPSNAP3B	1.894894393	1.785843829	1.602546669	1.548625694	1.628328452	1.84356711	1.370559339	1.179922404	3.867706279	3.628744168	2.802865872	4.0005953
NISCH	14.70486009	15.90352513	14.43749715	14.61073234	14.90714344	14.51234593	13.35946008	15.60587628	20.51456598	20.51930727	17.94602126	25.68024499
NIT1	7.422588783	7.590269921	7.072334744	7.553023102	6.520269719	6.136209819	6.81953586	6.307810932	9.396429738	8.471381866	8.085993066	9.879067546
NIT2	42.67508839	44.54829545	43.91323814	41.71954318	32.3151454	40.60839363	39.61841288	39.06507585	21.42375258	24.74376989	21.2967078	25.51011993
NKAIN1	0.047520955	0.082107921	0.067062445	0.016009764	0.016540123	0.01626743	0	0	0.637814398	0.833738584	1.109069985	0.478059484
NKAIN2	0	0	0	0	0	0	0	0	0.016162919	0	0	0.0154565
NKAIN3	0.016944691	0.010539889	0.007173798	0.01370076	0.010615972	0.006960632	0.010455064	0.006632186	0	0.007432217	0.010951323	0.007053646
NKAIN4	0	0	0	0	0	0	0	0	0	0	0	0
NKAP	11.53291484	13.35488525	12.13859279	13.24725301	12.57913402	13.5264406	13.37954805	13.20252531	11.36204988	14.58376775	11.0386767	12.73765997
NKAPD1	16.47579891	15.57589437	15.20949474	14.29372226	16.96597674	17.13921679	18.3036211	13.64354039	14.55440551	14.13483955	13.02682393	14.61420231
NKAPL	0	0	0.034267618	0.032722725	0	0	0	0	0	0	0	0
NKD1	0.002857195	0.005924082	0.003024095	0	0	0	0.008814608	0.011183121	1.032301882	0.943042355	0.778649765	0.951502556
NKD2	18.16467563	22.3609542	20.02449388	15.6805248	16.14499987	25.2330861	21.33273595	21.48363122	22.59439599	28.50146067	23.66864089	13.04083946
NKG7	53.41638035	57.82216372	54.21809661	20.77763226	20.52753603	28.14937764	37.54473812	21.6547534	7.090561398	8.068447849	12.88710113	1.987434823
NKIRAS1	1.737193248	1.699637283	1.942094439	1.832591436	1.97266	1.917836738	1.998586572	1.68922833	0.697122108	0.559565293	0.666629764	0.587559068
NKIRAS2	27.74007641	25.61493974	25.82412429	25.80637743	23.19401385	22.15501712	25.42999858	26.98235537	30.59214426	30.05627195	31.01362143	33.91270991
NKPD1	0.007769212	0.024162917	0.016446082	0.01570464	0.016224891	0.007978697	0.023968453	0.007602213	0.405832564	0.238539197	0.276167517	0.565972122
NKRF	9.413437282	8.343831017	9.440237968	9.966055387	10.12419026	9.896854486	10.1643431	9.67165095	7.772766682	7.573926906	8.162549127	6.208493705
NKTR	20.18906671	19.85911571	17.79421436	19.40731544	21.5108122	21.71198213	17.68075806	14.77250666	22.76712516	24.92995417	19.6202707	24.42960886
NKX1-1	0	0	0	0	0	0	0	0	0	0	0	0
NKX1-2	0	0	0	0	0	0	0	0	0	0	0	0
NKX2-1	0	0	0	0	0	0	0	0	0	0	0	0
NKX2-2	0.364039312	0.80197046	0.770608457	0.321941801	0.831517104	1.495420443	0.631733418	0.467531287	0.297124349	0.249489955	0.392129919	0.213103632
NKX2-3	0	0	0	0	0	0	0	0	0.149829229	0.07548534	0.123585722	0.071640382
NKX2-4	0	0	0	0	0	0	0	0	0.542540986	0.364449502	0.322208359	0.207531435
NKX2-5	0	0.02143894	0.043776158	0	0	0	0	0	0	0	0	0
NKX2-6	0	0	0	0	0	0	0	0	0	0	0	0
NKX2-8	0	0	0	0	0	0	0	0	0	0	0	0
NKX3-1	0	0	0	0	0	0	0	0	0.896482809	0.722650023	0.567904016	1.680309886
NKX3-2	0.552262338	0.746775391	0.457452059	0.364023886	0.576660584	0.394541427	0.37038265	0.516896217	7.081342108	7.582891483	6.285004011	7.071703869
NKX6-1	1.0125455	1.949445113	1.275823954	1.705628047	1.711784188	1.832111978	1.289170578	1.462581342	10.38936819	9.675448482	9.192850849	10.98903122
NKX6-2	0	0	0	0.065419208	0.022528789	0.022157361	0	0	0.023479654	0.02365853	0	0
NKX6-3	0	0	0	0	0	0	0	0	0.479985714	0.368489457	0.814450169	0.349719899
NLE1	8.397033383	8.078142764	8.354070393	7.24388439	10.15741114	9.938186629	8.832065012	8.571636327	3.532363952	4.189332686	4.147877632	3.157674888
NLGN1	0	0.002174565	0.006660365	0	0	0	0	0	0.10272177	0.124205209	0.076821343	0.237940201
NLGN2	0.801443008	1.767023784	0.848258333	1.277771949	1.343674228	1.495405738	0.708085849	1.214982613	2.616511171	3.094418625	2.808716917	2.408175847
NLGN3	0.349858982	0.375650651	0.297558941	0.265201101	0.260939504	0.307964938	0.160615256	0.18339578	0.326343474	0.321979054	0.275911976	0.325083588
NLGN4X	0	0	0	0	0	0	0	0	0	0.022583966	0	0
NLGN4Y	0.003051813	0.0126552	0.016150403	0.01850675	0	0.0031341	0.003138338	0	1.295242755	1.305110367	1.308346079	1.886532776
NLK	5.53832938	4.627760377	5.221205485	4.848171666	4.992977606	4.45999764	6.131061908	4.782591633	12.03770695	10.93472517	10.49700145	12.51943886
NLN	15.54075603	12.56049461	14.88944619	12.84723214	13.23437198	14.23270394	15.62159695	12.2158157	10.60687946	11.43474758	11.73514589	10.49460372
NLRC3	8.962128419	10.13771335	7.632124392	8.634101018	8.436087091	9.392691098	9.193522354	7.027110358	4.428102852	3.65499543	3.804405546	4.847162993
NLRC4	0.223029404	0.169556601	0.125897278	0.045083036	0.062102016	0.030539076	0.168192	0.014549027	2.071140344	1.989094687	2.914901925	0.835573511
NLRC5	11.52054801	10.82498483	10.96967879	9.249469188	7.825808038	9.528663977	9.875065337	8.237368504	9.613927686	8.303288529	8.45274845	11.88655872
NLRP1	1.702778365	2.222584288	1.223284858	0.722282121	1.133132683	1.150693267	1.487943521	0.664744299	2.054675686	1.083536633	1.216445002	1.285431356
NLRP10	0	0	0	0	0	0	0	0	0	0	0	0
NLRP11	0	0.013804534	0.014093735	0.013458344	0.013904182	0.041024839	0.013693434	0	0.028982062	0.014601429	0	0
NLRP12	2.058218919	2.41380015	1.96060256	2.119240373	0.913388072	1.453179642	1.40222993	1.988802935	2.533838042	1.49521006	2.965289166	4.431182289
NLRP13	0	0	0	0	0	0	0	0	0	0	0	0
NLRP14	0.119783836	0.137977051	0.126780864	0.282485417	0.055589215	0.095677272	0.068433302	0.130232323	0.014483863	0.014594206	0.028672621	0.013850828
NLRP2	0	0.015179353	0	0	0	0	0	0	0.366487051	0.305056629	0.567788117	0.106664569
NLRP2B	0	0	0	0	0	0	0	0	0.018333831	0	0	0.017532529
NLRP3	31.06407874	31.6283102	29.82472643	32.67291897	29.78741651	29.95604381	28.7760788	30.34308779	27.80984001	25.52637644	25.34505091	34.40158982
NLRP4	0	0.015179353	0.015497355	0	0.015288925	0	0	0	0	0	0	0
NLRP5	0	0	0	0	0	0	0	0	0	0	0	0
NLRP6	0.11352228	0.151313169	0.017164792	0.016390949	0.067735743	0.049964248	0.016677266	0.031737751	0.17648661	0.160048032	0.174688679	0.135018442
NLRP7	0	0.014399618	0	0	0	0	0	0	0.030231418	0	0	0
NLRP8	0	0	0	0	0	0	0	0	0	0	0	0
NLRP9	0	0	0	0	0	0	0	0	0.015882359	0	0	0
NLRX1	5.708365538	5.76642018	5.346166985	4.723796594	5.261554995	5.324803291	4.505917312	4.716254154	17.94762233	15.89554622	15.90308675	20.78583872
NMB	6.398558097	6.033955857	7.425076072	7.051372146	9.055895195	6.17523749	8.720441929	9.768702318	4.278611906	4.015340661	4.857821868	1.845235922
NMBR	0.018834288	0	0	0.019035761	0	0	0.019368277	0	0.04099283	0.061957691	0.060862832	0.019600595
NMD3	21.03999597	21.90696006	23.00255711	17.23408502	23.34858461	23.12478971	23.35776102	17.13299417	15.04157417	19.78761521	16.87439149	12.94166532
NME1	34.86400258	29.59642538	41.26452729	30.80595389	36.13176284	35.10727484	44.27886857	37.12771958	16.86886746	21.8047203	25.63582625	11.78638317
NME1-NME2	0	0	0	0	0	0	0	0	0	0	0	0
NME2	0.908360754	0.538110499	1.052985506	0.393461831	0.677493553	0.88843181	0.800669632	0.719533586	0.188290216	0.237155845	0.279558056	0.405136728
NME3	1.514340362	1.726900578	1.763078596	1.632575418	1.633950054	1.399655142	1.66109322	2.272077582	16.31501158	15.7750907	18.75871272	20.85512461
NME4	36.86228262	43.53158602	44.19557872	26.75845015	38.13743516	40.82376402	47.94643719	34.23586631	29.37828378	44.18905249	49.29689165	21.40257866
NME5	0	0.020653137	0	0	0	0	0	0	0	0	0	0
NME6	3.197331535	3.114814454	3.23635323	3.94368054	3.054007585	3.611214649	3.62976827	3.531876477	4.774360675	4.110990381	4.697081853	5.326640161
NME7	6.008022964	6.432702258	5.802998461	7.200476868	6.033481732	6.507180295	7.52882394	7.324507418	7.252792058	7.812049563	6.259439626	7.755796239
NME8	0.046548063	0.217152723	0	0.094091987	0	0	0	0.022773758	0	0.051041846	0	0
NME9	0.004370142	0.004530506	0.004625419	0.00883378	0	0.004487977	0	0	0	0.019168155	0	0.009095898
NMI	33.10476443	35.09144856	36.28125557	39.27656934	42.76057586	40.90862047	40.72833272	38.52995021	46.84037273	43.49178205	41.9212109	45.9852586
NMNAT1	4.967036387	4.001148022	4.463866643	3.685980242	2.616599354	2.619414668	4.176022025	3.338699197	5.928871349	5.606028914	5.398512108	6.438127039
NMNAT2	0.367270184	0.42717989	0.36976169	0.135804483	0.056121324	0.193186221	0.147388494	0.078887357	0.126728374	0.058935616	0.173682481	0.177123203
NMNAT3	0	0	0	0	0	0	0	0	1.585023552	2.423737902	1.482154555	2.292664384
NMRAL1	14.64026165	14.56702348	14.93667283	14.20171195	15.47787283	16.28619552	15.70265896	16.03430685	9.36932113	10.13093975	10.36748964	12.31975774
NMRK1	0.664201677	0.774646641	0.693236323	0.391595591	0.385302922	0.492635667	0.474328543	0.279828695	1.847200291	3.024568478	2.076803714	2.198482399
NMRK2	0	0	0	0	0	0	0	0	0	0	0	0
NMS	0	0	0	0	0	0	0	0	0	0	0	0
NMT1	26.25124525	24.33367382	26.93602659	28.51918721	27.03251365	27.22979118	27.34567084	29.51315395	30.45650929	29.12279591	30.30004553	33.61762839
NMT2	0.084769131	0.234346039	0.079751838	0.380781856	0.068844318	0.125745836	0.135601672	0.129028706	1.650250199	2.230867296	1.724748752	3.058227978
NMU	0	0	0	0	0.042373151	0	0	0	0.044161581	0.08899604	0	0
NMUR1	0.008516866	0	0	0	0	0	0	0	1.047338248	1.223420867	1.302716353	0.576120358
NMUR2	0	0	0	0	0	0	0	0	0	0	0	0
NNAT	0	0	0	0	0	0.042410175	0	0	0	0.04528348	0	0
NNMT	0	0	0	0	0	0	0	0	0	0.037391872	0	0
NNT	31.02557523	31.16505534	29.23599882	41.83946033	35.61898243	36.99717113	30.52616337	37.3219254	55.22149977	66.75585696	56.58978606	45.61964145
NOA1	24.02296901	24.85552393	22.07607809	21.9641594	28.340049	27.65448761	25.1170165	23.76084737	17.71143069	15.61880497	14.45225974	16.91274908
NOB1	52.35442907	50.69283848	51.24146014	38.63644935	55.83859875	58.09352996	51.65654827	44.22845979	31.04412389	40.42215167	39.54457641	30.06588766
NOBOX	0	0	0	0	0	0	0	0	0.021640154	0	0	0
NOC2L	59.82470106	55.8837362	66.55339301	61.96024565	60.70180923	64.06266018	69.80136177	74.6355259	56.25775411	57.7399949	65.39322209	60.45881114
NOC3L	28.52587184	23.25145266	28.24338274	26.84871577	31.24894355	29.51426426	31.36528484	25.84993754	10.68655522	12.30416038	11.68432062	11.46915211
NOC4L	19.3905459	19.83802198	21.97231563	21.68970629	26.39782137	24.16418869	26.42336123	32.93144689	11.19190357	10.54397695	14.67905868	14.77840762
NOCT	8.163648094	9.362959942	9.157226429	11.4033418	8.297747983	8.77371192	8.060415079	9.739730189	9.592456424	9.308653816	9.056516197	12.05218236
NOD1	3.061215011	3.743645707	3.123623843	3.612709207	3.674966901	4.423848667	3.487312573	3.659069112	5.046921832	5.226073013	5.173212863	5.532168511
NOD2	1.87169441	2.447754602	2.177029865	0.889039752	0.711312716	0.937308663	1.543889271	0.783063462	1.065219032	1.377929096	1.189725235	0.970482391
NODAL	0	0	0	0	0	0	0	0	0	0	0	0
NOG	3.242225126	2.063894566	2.227540072	0.7761097	1.752125327	1.723238401	1.550086627	0.612243353	3.404549829	1.808802173	2.879704093	3.048565846
NOL10	9.74590815	8.319438023	9.789215873	10.51986073	10.55124079	10.34893144	11.62161556	10.30185565	7.48127553	6.983243894	7.4248873	6.857398934
NOL11	34.06161608	29.97546173	37.76594492	41.97026159	49.46322593	42.62468298	43.2938642	42.3189885	29.4576598	28.80510806	31.54318324	29.06650161

NOL12	10.06087212	8.603325811	10.26754367	11.22172845	10.96038255	10.19594376	10.77477037	14.03460097	6.556875263	6.170527881	6.081897074	7.611117941
NOL3	2.955255247	2.001144683	2.097308772	2.261732408	1.444803393	1.80692928	1.141836797	2.340130148	3.085927198	1.723302362	1.766451921	5.457670458
NOL4	0	0	0	0	0	0	0	0	0	0	0	0
NOL4L	3.21083547	3.718553491	2.964681606	3.738767589	2.942392334	3.257058115	2.840069121	3.361526407	1.514964297	1.975840201	1.55999578	2.967602818
NOL6	35.86310344	25.43508707	37.55833463	28.78446781	35.40339001	29.77839669	38.33097225	32.40853372	26.38636145	21.98907247	29.78899747	27.41515805
NOL7	49.58144952	45.97696407	51.5440378	59.42709491	60.60170254	56.47875019	62.09175141	61.99889751	14.06854678	14.70940028	17.79152351	14.6249244
NOL8	23.28963698	18.99051475	22.80636874	20.29897276	25.67234214	24.23618338	24.84152326	19.24770018	11.34809682	9.245326949	10.43567702	10.97628084
NOL9	3.136243718	3.097171434	3.79160584	3.463544841	3.161874863	3.553995074	3.517095207	3.472275766	4.104447612	3.713251042	4.375704547	4.290833851
NOLC1	125.5375699	114.2520639	135.1229842	116.154117	139.8442714	150.0801673	141.7528104	120.5579643	49.6196415	57.63701857	57.87410411	51.49923921
NOM1	14.18696152	12.1936016	14.3693366	16.46761437	15.94115586	15.24207056	14.18873795	14.34099495	9.939493633	11.01479686	10.17189767	11.1445177
NOMO1	10.13601335	8.485176034	10.29897246	8.105912814	7.196990453	7.260498206	9.602547381	8.554396172	37.61406293	40.41528726	37.38099984	39.01595047
NOMO2	12.68527773	11.46883516	15.66605892	10.88416308	9.182373908	9.296603255	10.90502981	10.83658695	14.90504031	23.10155972	15.62699198	16.85962041
NOMO3	3.590628714	2.66254149	4.011502668	3.37702159	2.134994202	2.151009509	2.794964457	2.452094206	5.725575644	5.687168612	5.412086787	6.630082786
NONO	182.263584	199.6821932	189.1228779	232.3115375	214.9695043	225.9015949	198.728569	249.7332645	160.6803635	173.5476704	175.0198491	158.460526
NOP10	180.8403559	172.461565	206.3074583	164.5786691	148.6062057	153.9029594	185.2435246	174.0007052	110.1114142	134.8828969	141.70824	124.6629692
NOP14	41.44631384	37.68785027	43.25981228	41.83016797	45.8746154	46.56933065	45.90578889	48.85085691	19.47670694	18.81813471	22.86126277	19.37598661
NOP16	40.59883035	30.51880365	42.69133362	38.19100027	40.19023048	43.4382701	48.25636306	40.87265966	15.33289148	15.9957422	21.07698315	13.68726488
NOP2	46.37261384	34.26391373	45.69978292	43.07392924	45.59405095	42.6734571	47.52030519	49.96817653	9.762983482	8.966448031	11.70511426	9.693200872
NOP53	194.6265654	220.2409903	178.1551851	158.7246511	186.5386948	205.2842528	201.9260988	195.2171318	88.45297831	91.10399456	88.42777654	91.24656315
NOP56	171.8537838	158.235139	186.5606756	200.276828	202.3059694	204.7629212	187.254428	213.1750451	52.10026746	47.10383983	55.83097039	55.49816353
NOP58	75.7973971	76.00962393	87.78708965	100.0569858	96.37921705	96.4688946	94.24058968	98.54688202	53.25042876	50.50496913	65.51575225	50.92305548
NOP9	24.09266191	18.64894556	23.56189445	22.08543713	18.7926677	19.04699325	22.0318437	22.23521815	21.5026755	19.52470141	19.99683473	24.15638773
NOS1	0.004293088	0	0	0	0	0	0.004414805	0	0	0.004707545	0	0
NOS1AP	0.054708341	0.032409076	0.033088036	0.015798161	0.008160755	0.016052421	0.040185307	0.038237421	0.773972606	0.462779407	0.538787078	0.577475936
NOS2	0.012793524	0.03978896	0.027081684	0.012930378	0	0.026276966	0	0	0.25060595	0.561144787	0.385860129	0.226338868
NOS3	0.105035254	0.087111648	0.044468305	0.021231766	0.054837789	0.010786738	0.09721189	0.020555506	0.411496634	1.002026267	0.848551195	0.535613126
NOSIP	17.51695256	19.2471531	19.53935577	19.40055722	18.59202547	18.60866319	21.84282943	22.99075233	16.52301698	13.88845654	15.76151591	18.93919873
NOSTRIN	0	0	0	0	0	0	0	0	0.04501019	0.030235396	0.014850552	0.014347655
NOTCH1	9.73729857	9.249398214	9.247329954	9.911588861	7.275423635	7.339558004	7.932206528	8.74719828	12.57876164	9.022482774	11.30458759	13.07002023
NOTCH2	18.19741513	20.16248086	15.46514848	21.92883585	14.79150141	17.46370686	14.27717524	16.84893706	31.06930273	28.55662024	24.28264524	43.05166929
NOTCH2NL	0.879307249	0.78583943	0.695328848	0.878498216	0.474907201	0.903016509	0.810695547	0.553824941	2.51875298	1.895144255	1.643917591	2.072085475
NOTCH3	0.020001076	0.020735022	0.035282357	0	0	0	0	0.006523718	0.174129373	0.168145287	0.179536934	0.062444566
NOTCH4	0.023933089	0.041352203	0.059105926	0	0.041650703	0.016385607	0.024611639	0.046837297	0.173634565	0.096226556	0.111712698	0.116231962
NOTO	0	0	0	0	0	0	0	0	0	0	0	0
NOTUM	0.09708536	0.201295883	0.128445605	0.073592921	0.228092552	0.12462891	0.174716362	0.023749629	0.34337273	0.319374151	0.23529785	0.303106354
NOV	0.124128168	0.08578873	0.109482471	0.041818661	0.064805996	0.084983404	0.021274574	0.02024334	0.225137474	0.294908445	0.200559528	0.107648787
NOVA1	0.017249418	0.008941196	0.045642556	0	0	0	0	0	0	0	0	0
NOVA2	0.006378563	0	0	0	0	0	0	0	0.118005023	0.321740306	0.158027404	0.212418772
NOX1	0	0	0	0	0	0	0	0	0	0	0	0
NOX3	0	0	0	0	0	0	0	0	0	0	0	0
NOX4	0	0	0	0	0	0	0	0	0	0	0	0
NOX5	0.05341783	0.018459339	0	0	0	0	0	0	0.019377319	0.039049884	0.03835983	0.03706082
NOXA1	0.638148117	1.512149	0.627180141	0.345522037	0.880521622	1.006437831	0.492180655	0.75823785	2.083394152	3.623733283	1.963971323	2.537857762
NOXO1	0.036730076	0	0	0.055684473	0.019176382	0	0.075542891	0	0.139900273	0.040276023	0.039564303	0.057336756
NOXRED1	0.945255786	0.74403025	0.629926664	0.459991667	0.913903686	0.719069077	0.612035038	0.359697949	0.533386857	0.518255728	0.433675727	0.710460981
NPAP1	0	0	0	0	0	0	0	0	0	0	0	0
NPAS1	0.667562221	0.796916024	0.513859681	0.429356594	0.211228566	0.581689038	0.395251199	0.435475347	0.330215735	0.266185147	0.174320913	0.231574394
NPAS2	0	0	0	0	0.006487334	0	0	0.006079313	1.338706258	1.410218853	1.391991036	1.506493872
NPAS3	0.008943665	0	0.009466098	0	0	0	0	0	0	0	0	0
NPAS4	0.175602297	0.303410141	0.309766487	0.152126346	0.183360182	0.283386967	0.146184593	0.196374558	0.200199169	0.220062935	0.423341115	0.078320138
NPAT	4.538295577	4.121238499	4.158437548	5.730620117	5.768963794	4.833502146	4.446149706	4.247669369	5.582991722	4.575511773	5.044768642	5.544325834
NPB	0.076434035	0	0.080898837	0	0	0.07849497	0.078601091	0.224373274	0.249538027	0.335252127	0.329327862	0.238631671
NPBWR1	0	0	0	0	0	0	0	0	0	0	0	0
NPBWR2	0	0	0	0	0	0	0	0.052547833	0	0	0	0
NPC1	5.665292321	5.762087982	4.86200722	7.307194719	5.16960322	5.055026016	5.010433417	5.830130407	9.951482878	9.408424534	9.157518236	9.865976999
NPC1L1	0.01994424	0.010338051	0.021109259	0.010078793	0.020825352	0.020482008	0	0.029273311	0.021704321	0.065609015	0	0.041511415
NPC2	15.32134825	23.03756476	14.65121168	25.1583299	14.3683798	17.08434024	14.48852106	25.96472649	33.21281298	44.72621659	33.44965494	34.53977883
NPDC1	0.422588174	0.36507933	0.186363813	0.177961939	0.661886371	0.904130539	0.615639957	1.171596573	25.5234288	33.093422	28.07045858	40.16674943
NPEPL1	10.47619769	12.03601783	10.65614619	8.777715792	8.381848595	9.361443044	8.954363449	9.540985748	11.79555283	16.56001404	13.16534272	17.29759496
NPEPPS	18.53934363	20.76979221	17.85789425	17.99223941	18.9469565	17.64889974	18.24418978	17.16212609	23.23199346	21.18219372	19.94814172	24.28791895
NPFF	2.559525572	2.221491572	1.953026792	2.767386898	2.423987994	1.956122427	2.632093162	2.620997538	2.591073321	2.219191071	2.308209438	3.407012651
NPFFR1	0	0	0	0	0	0	0	0	0	0	0	0
NPFFR2	0	0	0	0	0	0	0	0	0	0.024093252	0	0
NPHP1	0.069431691	0.107969268	0.091859325	0.122805219	0.072499095	0.124781681	0.107100325	0.084924078	0.207787364	0.22840403	0.224367885	0.108384965
NPHP3	2.442264248	3.069263458	1.962433739	1.85381086	2.352392829	2.497879194	1.804184799	1.375335845	7.27910161	8.329257735	5.486926745	8.70379328
NPHP4	4.038990152	4.126124817	4.392708436	4.446086776	3.923506376	4.430248442	3.453399163	4.772071971	3.889636053	3.682389786	3.793600556	5.436389449
NPHS1	0	0.011103527	0	0	0	0	0	0	0	0	0	0
NPHS2	0	0	0	0	0	0	0	0	0	0	0	0
NPIPA1	6.874567059	5.024672726	6.543288293	6.048350552	7.643057167	6.856766458	5.391110106	7.210741103	8.450042609	11.49717588	7.884496454	10.39685438
NPIPA2	0.042394769	0.06592569	0.033653406	0.085696543	0.033200787	0.021768942	0.032697558	0.072596119	0.184544438	0.185950362	0.616492416	0.275747975
NPIPA3	0.019714072	0.020437487	0.010432823	0.019924956	0.020585015	0.020245634	0.010136503	0.0482258	0.053634601	0.086469133	0.010617641	0.133355136
NPIPA5	0.547746378	0.405604375	0.496921989	0.738140921	0.735358004	1.071458237	0.402340048	0.638062631	4.030670142	3.517953488	3.00625414	4.234526524
NPIPA7	1.074974265	0.672857857	0.79429058	0.881478421	0.804786411	0.895665178	0.792588157	1.389259323	2.295536651	1.401159285	1.092380393	1.941914321
NPIPA8	1.009830901	1.221368169	1.374195796	1.263641093	2.008464652	1.950659619	1.335164909	2.023302876	2.459551458	2.741936967	3.988428223	2.226944592
NPIPB11	0.645011082	0.628153931	0.548219653	0.572891351	0.857190535	0.772803362	0.773848152	0.784151857	0.478590913	0.546535251	0.452661325	0.56954928
NPIPB12	1.4239054	1.767742504	1.841988026	1.439137098	1.964059933	2.419111875	1.807748052	1.496505967	1.147826012	1.156570552	1.874618968	1.317190695
NPIPB13	0.413495601	0.342935174	0.350119557	0.362196307	0.316626427	0.339715934	0.226783475	0.566450561	0.104996875	0.317390333	0.20785447	0.200815723
NPIPB15	1.085980336	1.252684939	1.05228277	0.94300605	0.750647964	0.863935538	0.880832689	0.92793677	0.749039672	0.603796895	0.477796875	0.684466359
NPIPB2	0.273776674	0.309625097	0.276597689	0.188662703	0.29886595	0.255598791	0.230349913	0.20700735	1.367803877	0.95520495	0.898111504	1.036057333
NPIPB3	2.083140033	1.961873418	2.142716418	1.5864812	2.665349771	2.606341148	1.885740454	2.440293843	1.564533586	1.335158962	1.327367259	1.465620035
NPIPB4	0.940270873	0.955471981	0.650325876	0.715099103	1.137345232	1.099472792	0.708443326	0.737869757	0.861150839	0.571668679	0.78218216	0.658810575
NPIPB5	7.189645281	7.526545107	6.371191411	6.995839084	7.919438738	6.905747639	7.451474008	8.662834422	4.387650385	4.06553588	6.377761472	4.768048761
NPIPB6	0.220422583	0.173668411	0.158642845	0.124757064	0.165715611	0.153928854	0.090668799	0.198429846	0.220684895	0.183693776	0.056983487	0.128458886
NPIPB8	0.148235705	0.192094083	0.156894714	0.168549076	0.212828783	0.247378086	0.171493289	0.235705258	0	0.040636621	0.099796322	0.057850102
NPIPB9	0.364481108	0.264499109	0.321476526	0.294704007	0.202977823	0.21210833	0.237382748	0.27342907	0.14543708	0.119900512	0.078521161	0.164367958
NPL	10.6735326	10.73042644	10.59544794	9.069920727	7.152015113	7.540277353	11.44805274	7.849710098	1.257726305	1.621408897	1.757524811	2.175573139
NPLOC4	26.59250748	23.83121306	28.05310079	28.88139273	22.24132285	21.37952989	26.54645806	28.82991738	24.81362919	24.34185755	24.83229984	32.13294582
NPM1	862.5397749	876.360033	885.6622352	784.8475753	966.3300912	919.6981532	1014.303243	833.8238618	438.770637	588.2726801	565.9430802	325.2300449
NPM2	0.902265202	0.995078874	1.239429062	1.008929278	0.721628523	0.867449225	0.829139155	1.164638443	0.396934315	0.378907862	0.248141436	0.539411441
NPM3	80.6223067	81.79974972	87.279975	81.17467012	85.78577351	90.42047667	88.83912927	97.44093805	15.82475134	25.56631956	21.08299141	19.15583769
NPNT	0	0	0	0	0.011863767	0	0	0.011117595	0.012364497	0	0	0
NPPA	0	0	0	0	0	0	0	0	0	0	0	0
NPPB	0	0	0	0	0	0	0	0	0	0	0	0
NPPC	0	0	0	0	0	0	0	0	0.020040471	0	0.019836312	0
NPR1	0	0	0	0	0	0	0	0	0.274406078	0.29032143	0.271610608	0.170568342
NPR2	0.911874931	0.926800493	0.870519335	0.668633075	0.644108507	0.550860195	0.799827148	0.839787526	0.418342757	0.137242273	0.211855371	0.307021708
NPR3	0	0.018820554	0.025619785	0.030580954	0.435997425	0.329375203	0.423165926	0.171720077	0.296347455	0.637024284	0.325920511	0.188930158
NPRL2	15.6248259	16.40030079	15.97743658	14.52523136	15.00641206	16.93281132	15.6954824	15.01644074	6.061931373	8.21541237	5.610164899	7.391159008
NPRL3	9.469941572	9.326572201	10.2915931	10.03271338	10.59461948	12.03504016	11.07746761	12.45997958	5.999368888	8.010650446	8.597713428	6.405907975
NPS	0	0	0	0	0	0	0	0	0	0	0	0
NPSR1	0	0	0	0	0	0	0	0	0.029725004	0	0	0
NPTN	20.51119937	20.30603331	20.82922805	24.97011347	17.67414407	17.97103512	20.90270765	21.82420484	28.97748916	25.79415129	26.55251122	36.53774306
NPTX1	0.009896921	0	0	0	0	0	0	0	0.3877317	0.347276068	0.607654425	0.113295547
NPTX2	0.138837791	0.143932492	0.08397019	0.06013841	0.642016497	0.672169234	0.611889066	0.52400629	21.13104816	11.61383557	13.28867662	27.34925948
NPTXR	1.378783245	1.438971325	1.057764468	1.720872059	0.801978381	1.178382951	1.113364513	1.738497375	3.725976804	4.90096523	2.840771375	9.572296723
NPVF	0	0	0	0	0	0	0	0	0	0	0	0
NPW	0.370735163	0.823584474	0.448447097	0.160586128	0.110603934	0.21756086	0.217854992	0.31094249	0.172908239	0.174225515	0.285244605	0.440936211
NPY	0	0	0	0	0	0	0	0	0	0.210729908	0.310509127	0.099998034
NPY1R	0	0	0	0	0	0	0	0	0.014813624	0.01492648	0.029325426	0.014166177
NPY2R	0	0	0	0	0	0	0	0	0	0	0	0
NPY4R	0	0	0	0	0	0	0	0	0.006924235	0	0.013707391	0
NPY4R2	0	0	0	0	0	0	0	0	0	0.007222078	0	0
NPY5R	0	0	0	0	0	0	0	0	0	0	0.005401836	0
NQO1	12.19874411	8.105566561	14.55772881	17.6250147	7.971729132	5.99057843	14.2284418	12.05669945	32.67590837	29.93983849	31.68161588	47.52131735
NQO2	6.412957178	6.341009327	6.103102253	11.43804247	5.289493311	7.360682012	6.317685667	9.386300693	5.483422169	4.579708575	4.905121642	6.561713735
NR0B1	0	0	0	0	0	0	0	0	0	0	0	0
NR0B2	0	0	0.105663787	0	0	0	0	0	0	0	0	0
NR1D1	3.141314904	1.889624303	2.319158302	1.861834601	2.55118437	2.668432954	2.672040539	2.88405229	2.19461576	2.870483076	2.548226251	1.331865702
NR1D2	3.513441926	2.079875433	2.831264216	2.421153861	3.147544646	3.136653742	2.740584274	2.656575126	10.51463417	10.68229816	6.708978498	10.75104068
NR1H2	16.9152543	18.44345842	16.24108022	18.00695534	15.1354869	17.90015811	16.9447404	21.48944874	11.6556148	11.26447147	9.401443799	13.37007202
NR1H3	0.888964424	1.316550411	1.152112906	0.990154789	0.587252447	0.894302768	0.802229339	0.585821522	6.120384251	5.371268063	5.569482651	5.456399786
NR1H4	0	0	0	0	0	0	0	0	0	0	0	0
NR1I2	0.011800342	0.03670008	0.037468935	0.011926572	0.012321666	0	0	0.011546695	0.025683446	0.012939556	0.0254218	0
NR1I3	0	0	0.031275553	0	0	0.030346216	0	0	0.032157198	0	0	0.030751729
NR2C1	16.57489864	15.4745715	14.97642597	15.72898554	16.66797177	16.15138321	14.93843736	14.69812657	9.697790548	9.487686534	8.559210232	11.01356597
NR2C2	7.042465847	6.78053716	6.602749631	8.432651784	8.407162331	7.947701589	5.883246004	6.795866249	11.84988145	9.929538725	9.323746353	11.9449393
NR2C2AP	15.09846328	15.61327738	18.26333915	22.67960969	22.75920957	24.05500974	19.6902918	25.32672502	5.065165695	4.190887348	6.358667912	5.592013808
NR2E1	0	0	0	0	0.016762171	0	0	0	0	0	0	0
NR2E3	0	0	0	0	0	0	0	0	0	0	0	0
NR2F1	7.127370184	5.790141214	8.778933756	6.024073671	11.85246953	7.219672614	9.358258446	6.362398775	1.723635762	1.782471417	1.406764819	1.604925838
NR2F2	0.228976855	0.438238599	0.130497371	0.42724846	0.137938131	0.361770596	0.19924283	0.465345029	0.65171219	0.898102588	0.616613869	1.191465933
NR2F6	15.12275352	15.80137815	15.78513894	12.39041258	16.91210181	14.82597669	16.31835328	15.4689867	7.92029641	8.503956392	9.06053239	9.591829125
NR3C1	6.343744418	5.17428508	5.918324818	4.7847793	5.940417149	4.617308438	5.975563853	3.957374081	9.835761744	7.558350301	8.060030784	10.57422272
NR3C2	0.08561061	0.095579207	0.069701115	0.093182274	0.103145512	0.11497097	0.067721415	0.077326538	0.121832134	0.144423876	0.049655113	0.102800573
NR4A1	0.832759082	1.125765982	1.064735666	0.740667147	1.252151016	1.183615121	1.226321041	0.840934475	0.311750038	0.153409912	0.22246042	0.034665655
NR4A2	3.280161649	2.318542049	3.3928645	3.119348822	1.18320773	1.0258938	1.180606242	1.269272586	1.979525453	1.25889078	0.947559024	1.58267877
NR4A3	0.03858976	0.048006989	0.008168787	0.062404095	0	0	0.039683855	0.007552055	0.142784046	0.016926097	0	0.032127882
NR5A1	0.069543859	0.036047898	0.018401545	0	0.018154054	0	0.017878891	0.034024509	0.018920277	0.019064418	0	0

NR5A2	0.084504022	0.316725504	0.055040137	0	0.162899634	0.30040114	0.153745936	0.006360586	0.014147924	0.021383562	0.007001897	0.03382393
NR6A1	2.408596113	2.418000483	2.2081453	2.049364881	2.34366629	2.268916672	2.404566759	2.064370965	1.345653696	1.14384433	1.028943334	1.445408302
NRAP	0	0	0	0	0	0	0	0.009512724	0	0	0	0
NRARP	2.360968905	2.554023122	3.085576095	1.92972937	2.272339052	2.930638589	2.280121378	2.470931013	1.362859092	1.463290474	1.5037756	1.645141248
NRAS	31.02446167	30.0212258	30.84196419	31.88138195	31.72649272	31.22823849	33.48187639	28.11150955	55.7841681	58.73942431	56.90761789	54.8924994
NRBF2	17.35383672	17.41284025	16.91958804	17.15539687	17.08882859	14.4395266	17.8719045	17.79887125	18.69232499	13.97325814	15.82758386	16.53074839
NRBP1	29.48342479	29.81796405	28.56443171	37.14109075	23.0460452	23.17863961	28.49447497	35.27076957	23.63653454	23.20852237	23.05724934	32.04883733
NRBP2	2.888691445	3.546905891	1.940709662	1.853216291	1.829039133	2.198636178	2.180540596	1.543603838	8.148883669	8.62656758	7.183146599	15.91592537
NRCAM	0.00860196	0	0.027313299	0	0	0	0.004422921	0.004208531	0.08424975	0.056594396	0.101922906	0.062663623
NRDC	31.88401009	33.19199146	30.99296846	34.80552953	33.76009128	32.91768715	33.17372556	32.60763764	125.0963441	123.4487742	129.7002278	107.6911587
NRDE2	3.976829399	3.372206712	3.788217972	4.483143517	4.69554258	4.649543984	3.942774895	4.120837483	5.670507743	4.371936777	4.195825431	4.92391303
NREP	25.70480196	36.96025434	26.53785702	27.0489615	39.02410574	45.36935536	24.68000445	27.63969741	63.83331132	53.18704167	68.25126666	21.85403036
NRF1	5.070600374	5.498353723	6.014509805	8.939019047	8.398351704	7.646383517	6.877563514	9.067766698	6.818318586	6.9501497	7.235403571	6.92973105
NRG1	0.003344286	0	0	0	0	0	0.006878206	0	0.010918258	0.018335729	0.014409373	0.006960708
NRG2	0	0	0.013175889	0	0	0	0	0	0.101604842	0.095553643	0.060341855	0.142507319
NRG3	0	0	0	0	0	0	0	0	0.107177236	0.284194077	0.156336355	0.178014032
NRG4	0.748493007	0.707087102	0.609396401	0.470014626	0.469398746	0.534433835	0.516938274	0.348867092	0.03614856	0.033995689	0.028624241	0.032264068
NRGN	18.3783065	27.61726222	22.07037174	47.33027809	29.38504986	29.85335118	19.80799113	49.23783691	45.91390435	39.19091859	44.58958648	57.83794313
NRIP1	9.536187543	5.790627339	8.765523172	6.541106453	11.8473208	7.312941687	10.51894458	5.410634945	9.046578335	5.810103668	7.186390769	7.715923431
NRIP2	0	0	0.031870611	0.030433782	0	0.015461796	0	0.014732213	0	0	0	0
NRIP3	7.352455112	7.527821017	8.016086752	7.996658406	8.180036947	8.098630687	9.032947598	7.640086335	4.10686688	7.520025461	5.424710838	3.494006264
NRK	0	0	0	0	0	0	0	0	0	0.007215013	0	0
NRL	0.190847884	0.20774367	0.25249504	0.183244938	0.1992793	0.254791971	0.147194098	0.270781153	0.145383153	0.08370899	0.205574406	0.248266088
NRM	10.66283275	15.2116281	13.68264978	18.90725064	23.72112542	20.59245497	12.42069529	23.08326538	4.929059779	6.078925021	6.63218091	4.148975845
NRN1	0	0.034087456	0.017400789	0	0.017166758	0.016883733	0.016906559	0	3.900305542	3.100749278	3.789735589	3.0112454
NRN1L	0.45823227	0.475047265	0.277142488	0.132324011	0.205061309	0.403361011	0.067317722	0.192163972	0.997342578	1.148503637	0.423078129	0.953752535
NRP1	12.40647113	9.027013372	12.07816569	5.749633027	5.513906756	5.414267432	8.062424903	4.509779891	3.211080152	2.741353675	2.702070572	3.486657102
NRP2	0.046527938	0.032156865	0.041038176	0.03526924	0.008097247	0.031854998	0.011961774	0.034145868	0.004219503	0.008503296	0	0.008070168
NRROS	30.08616011	30.46940085	30.23804677	26.04270211	21.42754184	27.63022928	23.37770013	30.0373205	36.31804197	33.40654867	41.78053893	27.67056004
NRSN1	0	0	0	0	0	0	0	0	0.048636426	0	0.024070475	0.046510713
NRSN2	0.018315031	0.028480661	0.038769763	0.018510949	0	0.018808869	0	0.017921351	3.029562653	4.207425604	4.389543589	3.059163806
NRTN	0	0	0	0	0	0.045745413	0	0	0.678655297	1.172272338	0.86366777	0.927134087
NRXN1	0	0	0	0	0	0	0	0.00186792	0.031161269	0.064890578	0.034956328	0.095357852
NRXN2	0.006962034	0	0.022106138	0.007036508	0	0.007149755	0.007159421	0	0.227293015	0.24429292	0.412458753	0.246340091
NRXN3	0	0	0	0	0	0	0	0	0	0	0	0
NSA2	80.83009634	83.38474737	83.73144	79.45516027	93.24176786	91.05918219	95.84173515	81.48744531	77.59778871	84.7893222	78.83789195	69.55978768
NSD1	12.08469652	10.63507466	11.05036376	10.35336359	11.53344687	10.78299333	11.34718265	8.476475011	17.14579951	14.60254484	15.50789409	16.87151263
NSD2	4.890583638	5.823854131	5.637558066	9.584752332	9.309962035	9.509594156	5.594552363	8.89585679	12.28653243	14.67364269	13.87174152	11.03840885
NSD3	17.65423562	15.66827521	16.22261983	19.30045045	18.96792394	17.55046465	15.92661148	15.36363625	19.31185395	19.13271007	17.18388237	18.85685308
NSDHL	28.34903262	31.58116021	31.67545391	30.99984707	24.53314008	29.51098092	29.42838757	31.61506764	17.92070623	24.09807873	27.20062241	13.01579276
NSF	19.96749459	19.2856479	19.00332572	21.13689204	14.64270574	14.73427241	20.43636253	18.94342111	35.27021308	32.3983346	31.37470075	45.87607505
NSFL1C	8.362423879	8.005421735	9.090118402	11.57374256	7.817379228	7.785206361	8.744777143	10.5692875	4.611693328	4.491932594	4.260398239	5.262759656
NSG1	0	0	0	0	0	0	0	0	0.020868944	0	0	0
NSG2	0	0.022750457	0	0	0	0.022536892	0	0	0	0	0	0.02283805
NSL1	3.126723656	4.057362657	3.403597718	4.481107538	4.306043204	4.39596765	4.204154383	4.094453394	2.813586673	2.745206828	3.038098564	2.812916229
NSMAF	36.41355427	30.50947602	35.10808512	34.25848712	30.12075803	30.35454214	31.79980306	28.96398198	34.53435673	27.95026139	29.99973983	30.10884489
NSMCE1	19.84803239	21.19242172	20.8816382	22.07239035	20.10438736	21.39016094	20.68575851	23.52082773	21.14693546	22.67644646	20.22738637	21.9233679
NSMCE2	5.404556771	5.50501201	5.432995585	6.476128629	6.222441515	6.035023765	5.788349798	6.02737469	7.255573518	7.401425905	6.584246161	7.048984196
NSMCE3	12.645892	13.44437465	13.14557602	16.88939888	15.52884542	15.80290092	14.13236302	18.56110423	16.851297	13.22999761	15.25490882	18.96845194
NSMCE4A	26.29552363	27.79100266	29.4823501	34.85281412	36.31275437	36.13953903	29.89803228	33.55193395	20.26200561	23.78346611	21.65688656	24.60096552
NSMF	12.0850127	11.28642927	11.39882627	7.015318215	9.72254623	10.51179101	11.47682451	9.684468908	17.0062702	22.16234003	23.29970711	15.70733876
NSRP1	10.48293095	10.3855749	10.6255189	14.09827623	13.35153699	11.04775075	13.21436845	12.69791751	13.40905886	11.03145412	13.31798768	15.17644941
NSUN2	73.90037851	59.47328514	74.88986723	61.82038886	67.35084225	60.17686947	76.9802113	59.86694164	74.04875686	72.14562142	79.96377385	73.28681512
NSUN3	1.800238266	1.735430143	1.911206145	2.113499514	2.06316274	1.967146067	1.89078756	1.815247835	2.030784109	2.521708777	1.820910315	1.890619698
NSUN4	8.611014395	7.830363004	7.640845638	7.202588288	8.109734515	8.40484988	8.015440769	7.200167643	8.320745615	7.987313996	8.305945118	7.94742089
NSUN5	16.55039043	16.6619498	18.72751812	22.04406563	14.65459392	16.97529543	18.32389446	24.19023639	10.61198708	9.917659521	11.57890293	13.91317309
NSUN6	2.760090957	2.844492209	2.766204085	2.896592427	3.5451498	3.796073262	2.938817359	2.740597268	3.366944724	2.642653171	2.394241958	3.380777824
NSUN7	0	0.011072672	0	0	0	0	0	0	0	0	0.011504903	0.005557652
NT5C	15.66063528	16.73028672	18.59682655	17.80667898	21.63715227	21.42752475	20.86729933	25.22855483	12.62613706	11.04695917	13.26896144	9.391119681
NT5C1A	0.097216912	0	0	0	0	0	0	0	1.851435408	2.505154105	2.199089028	3.085756847
NT5C1B	0	0	0	0	0	0	0	0	0	0	0	0
NT5C1B-RDH	0	0	0	0	0	0	0	0	0	0	0	0
NT5C2	25.95242947	24.38446097	24.05959946	29.11416092	24.00360209	23.83547389	24.3520323	24.98794526	22.33339113	18.09852286	18.91271963	23.75754545
NT5C3A	11.76151677	12.24466627	12.39591497	13.92300527	17.57784782	15.34729002	16.41644075	12.23864277	14.69368465	11.96992622	14.62434852	10.99793531
NT5C3B	10.23209496	11.2941391	11.42560413	7.831464278	7.330216191	8.195551086	8.683364806	7.679227085	7.567528605	9.186527206	10.05858488	6.306337531
NT5DC1	6.671653635	7.295856879	5.899372586	7.440084535	6.496093381	7.328551528	5.500225947	5.674341216	9.619300402	11.20512053	9.187756322	13.27099198
NT5DC2	24.7790382	29.75531483	33.6038791	32.54995679	30.7893749	29.73833426	35.23887598	40.45830301	10.30577668	11.02455418	16.5689102	7.899471657
NT5DC3	3.581685727	4.568069928	3.637995092	5.153175725	5.053606231	6.552867891	3.460382464	4.388234881	3.471962887	3.505014295	3.248552806	2.756406252
NT5DC4	0.042091334	0.04363589	0.017820019	0	0	0	0.025970823	0	0	0.027692917	0.018135702	0
NT5E	0.039682567	0	0	0.013369019	0.013811897	0	0.013602549	0	6.089022305	5.192617015	4.887134797	10.40687501
NT5M	4.303450838	4.495423726	4.972068209	4.083867972	3.893285497	4.470092039	4.205878145	4.291310092	0.73287196	0.954588474	0.690020282	1.470056563
NTAN1	17.44123176	25.08635059	19.80803505	22.38337643	23.60510475	22.74362069	21.97399172	23.85132651	16.86293004	22.5776107	21.72134851	12.26011395
NTF3	0.032221294	0	0	0	0	0	0	0	0	0	0	0
NTF4	0	0	0	0	0	0	0	0	0	0	0	0
NTHL1	9.110162994	9.620995672	11.17427988	10.45537656	12.5353459	12.41611569	12.7393464	12.41342772	10.56272358	10.03634532	11.09709833	8.860585259
NTM	0	0.005211034	0	0	0	0	0.005169096	0	0.005470178	0.016535556	0.005414452	0.005231098
NTMT1	8.439139493	8.123901218	8.847033989	8.407565273	9.273549289	9.719072436	9.091663132	9.987934126	6.60365707	6.72006504	7.640209631	6.043197074
NTN1	0.061768929	0.045739687	0.056037502	0	0.018427943	0.018124125	0.027222943	0.008634459	0.729817569	0.193520414	0.437231612	0.440791595
NTN3	0.027094442	0.140443408	0.114708522	0.136921371	0.509245909	0.19477503	0.139313111	0.212096388	0.029485527	0	0	0
NTN4	0	0	0	0	0.013451472	0	0.013247586	0	0	0	0	0
NTN5	0.035100822	0	0	0.01773815	0.018325766	0	0.018048	0.017173166	0.401083952	0.230936886	0.245760648	0.803637165
NTNG1	0	0	0	0	0	0	0	0	0.004407848	0.004441428	0	0.029506383
NTNG2	0.399507748	0.692659985	0.291616904	0.884141886	0.13665504	0.233435117	0.120417032	0.343740315	4.152748901	3.043876454	2.441039491	9.032080458
NTPCR	14.14121743	14.66013439	14.45262598	12.85914378	12.52356355	11.85936046	15.20883756	14.66986708	21.07744494	26.08099981	25.09637018	22.77063658
NTRK1	0	0.019525419	0	0	0	0.019342128	0	0	1.414252621	0.185873073	1.359269914	0.333210109
NTRK2	0	0	0	0	0	0	0	0	0.045064312	0.011351907	0.03345392	0.026934202
NTRK3	0	0	0	0	0.001779133	0	0	0.001667234	0.001854224	0	0	0
NTS	0	0.045023504	0.045966732	0	0	0	0	0	0	0.095245156	0.046781036	0
NTSR1	14.30348572	16.77737749	15.08522678	17.89729108	16.56688184	17.84615376	13.65090898	17.87415272	11.86095008	8.789367378	13.32736057	11.97710159
NTSR2	0	0	0	0	0	0	0	0	0	0	0	0
NUAK1	3.526297274	3.467595309	3.8157779	3.21319791	5.593144153	3.7834092	4.591658851	3.134010981	0.197454906	0.57957676	0.41637934	0.32018136
NUAK2	0	0	0	0	0	0.015561943	0	0.029655268	0.016490638	0.03323254	0	0
NUB1	14.87001878	14.72775859	13.64759754	16.01984647	14.0781945	14.71340685	14.56270253	16.04084448	26.39060464	26.19476709	23.36012608	25.72370942
NUBP1	8.663536197	9.302213707	9.437549228	10.49039582	9.075651216	10.19704228	8.706683498	10.01864397	6.367024291	7.371691306	7.180827897	6.996203253
NUBP2	9.725524259	9.848836559	10.65132267	10.15215115	11.17462499	10.64332628	11.15970936	12.49266967	10.62466632	11.17912605	11.91187839	14.4002751
NUBPL	0.803935401	0.821415382	0.940894965	0.976604809	0.992813709	0.976445396	1.017512066	0.854730772	0.719254558	0.796783679	0.915929809	0.607372053
NUCB1	29.71264408	34.13248354	28.0291175	37.16794419	24.18091543	26.52116249	26.94330164	38.22620974	41.2667089	44.95865592	40.80584861	47.73099859
NUCB2	15.09602258	19.64291438	18.68063173	44.90269951	29.8612063	30.86124194	19.73147045	37.6467057	60.40067732	64.96050548	57.36901712	62.1802141
NUCKS1	87.17415243	94.51542401	91.52622094	151.2755773	136.407709	135.3812843	105.5083453	132.5934974	168.4093439	176.1459486	169.4734219	145.4550606
NUDC	36.79801808	38.62779428	45.28980516	50.80826663	48.0191362	53.58777652	53.18462324	60.30688539	38.11976226	39.27010116	46.02713762	47.02149689
NUDCD1	21.40006493	17.97547764	21.96789121	19.23155459	26.00692261	22.9700277	22.36474083	17.60984378	10.6762898	13.55658721	12.49773198	10.08892475
NUDCD2	2.453047293	2.335850211	2.72455336	2.748642582	2.991485339	2.456987971	2.734369507	2.82704264	2.346548797	2.723074458	2.674954806	2.155743292
NUDCD3	14.0909605	13.25959943	13.18822506	14.44174384	14.0639228	15.83571733	13.11411493	15.61418946	14.14465519	14.67616248	13.31017577	15.66478218
NUDT1	31.3354006	36.01337689	39.01061631	55.09905506	48.8114432	49.12773464	45.03411037	56.10866944	15.30341086	17.39150413	18.26001165	14.43408371
NUDT10	0	0	0	0	0	0	0	0	5.86993613	6.170577944	6.676070927	4.695811276
NUDT11	0.02245808	0	0.047539884	0	0	0	0	0	5.914481723	6.402811908	5.201071071	5.188545719
NUDT12	2.518484013	2.403939034	2.486808088	1.567609026	2.469396972	2.996428975	2.795183994	1.713023353	7.620595097	7.594455717	7.675294092	6.408549781
NUDT13	2.751234471	2.329290126	2.135425971	1.622054453	2.370043913	2.378059785	2.499159691	1.615258134	3.967091126	3.268243999	2.988225881	2.43369735
NUDT14	1.810709436	2.570884906	1.916479839	1.511804248	2.055113337	1.576560266	1.538212421	2.195476894	5.011935681	4.14368686	5.512085937	5.079636764
NUDT15	12.03691783	9.969451468	13.31185491	17.5168294	15.38931593	14.11471954	15.84496976	17.17035253	12.11144674	11.65869565	13.05884167	9.603024018
NUDT16	0.004424764	0.013761398	0.004683232	0.008944194	0.023101225	0.031808503	0.054602583	0.056285508	7.945162746	10.6888115	6.863321542	8.615569421
NUDT16L1	5.395133078	5.388981311	5.918687753	5.468766222	2.927766439	3.801583069	4.843447071	5.602851126	4.979851779	5.199155936	5.081829697	7.983449571
NUDT17	3.361668273	4.267378625	2.977132214	3.282062873	4.059394716	2.818212932	3.692146773	3.625063489	5.873246352	5.140627599	4.286160832	4.854983155
NUDT18	10.33143569	9.941970134	7.973389382	7.420554115	8.865028228	8.080307053	9.738989547	9.149908878	9.49945059	8.627750738	8.732896685	11.29933337
NUDT19	14.60565501	13.47883289	14.24195111	13.35599894	13.72434068	13.51264708	14.91757889	13.52781646	7.02822658	7.719907591	7.889274357	7.178967247
NUDT2	15.6824656	17.74949327	17.41069874	17.54672998	16.77591581	14.97253066	16.57095936	17.78561483	24.16441446	25.92616449	22.52344278	25.70359439
NUDT21	38.11968437	41.06278438	41.84549713	52.47237052	55.93180976	56.20090204	48.89383792	46.97783394	59.94016283	64.65444151	63.26203644	57.0154434
NUDT22	14.87873512	15.25075261	14.35660028	16.2533638	12.67415312	12.80985682	15.30057657	17.62395314	12.47863066	14.10707495	13.43602902	18.19091049
NUDT3	1.36994291	1.559906575	1.568816179	1.9520553	0.984910494	1.406881458	1.316404244	2.219509917	0.879840256	1.034300385	1.016023186	1.098475895
NUDT4	13.16858451	12.31801281	13.1127571	13.40123986	15.09378135	12.78529597	14.40582219	12.3671435	13.08707039	10.54775806	10.57332344	15.31109133
NUDT4B	0.235877527	0.183399852	0.140431516	0.357601109	0.338660156	0.257377478	0.242565119	0.173105518	0.224607013	0.129324656	0.174679107	0.230132461
NUDT5	37.40106007	35.46894508	40.533672	30.1304573	39.51302933	36.95981587	41.0213882	29.39355293	28.14760791	28.09573594	33.10929476	21.12003604
NUDT6	5.643537337	6.378810711	4.272495611	4.357151162	6.17932017	6.399426739	5.843845115	6.17415984	6.098929905	7.219763212	6.754459276	8.197945132
NUDT7	15.9880206	17.49775328	14.62744095	11.93343628	20.13023433	20.23566432	14.49064828	14.73524397	26.45653628	22.20092646	18.93137659	18.12913991
NUDT8	1.956711304	3.14419595	2.381661764	2.125965682	2.298550853	3.014206831	2.263711413	2.393314924	2.235860724	1.984692592	2.529237978	1.985415504
NUDT9	3.044882386	3.032420716	3.05368345	4.096545167	2.887521933	3.106478469	2.915619235	3.243185938	3.118037064	2.791487098	3.172301035	3.916806097
NUF2	15.18016453	16.34054939	16.14389339	39.1161465	37.27218007	33.29573371	22.76746655	37.34822406	31.87849235	40.52395967	35.55019319	24.98373585
NUFIP1	7.221931311	6.767044301	7.284468156	8.593698852	8.443327354	8.510142314	9.299228098	8.561574522	4.147946514	4.382601938	3.407556424	4.014833585
NUFIP2	17.00694461	15.13581296	15.22131422	16.98506661	17.48053646	16.3243501	15.79933313	13.51797394	30.99731053	36.1402969	28.07043438	28.39682639
NUGGC	0.010770529	0.011165757	0	0	0	0	0	0	0.187536452	0.023620646	0.150821086	0.134504961
NUMA1	20.58747718	18.39930522	19.42186373	23.46243771	23.02706425	21.01146249	18.20120607	24.06566046	30.74143077	25.93525607	29.5440955	28.29051714
NUMB	14.88410633	12.60313446	13.2469526	10.96697452	12.57421287	11.37932089	13.68250217	9.676678478	34.03532732	33.01978591	32.40536966	31.35281106
NUMBL	2.535300187	2.265805105	2.282429362	2.415155064	1.704013389	2.169717436	1.917926209	2.880013951	2.489479112	2.843969302	2.746628254	3.548264914
NUP107	22.44852874	23.59242386	23.74167434	35.38980683	33.55264589	33.73944784	26.69284521	31.63540361	19.80880304	22.85678777	21.21474121	20.65701449
NUP133	35.06009037	38.15259354	38.02315183	45.84265411	43.09001967	43.36024471	39.11944242	44.1552255	37.43796483	36.91664573	36.02799422	38.76950143
NUP153	38.37362156	34.20974758	40.82067481	37.93671019	44.6858761	39.39924773	37.42860953	33.0667644	12.30328478	10.58730286	11.51248534	11.09507459
NUP155	19.83499138	18.68209552	21.24964828	23.32539209	27.63416925	26.29783572	20.83831516	19.1289143	40.20346214	44.80185024	44.1167442	36.21506228
NUP160	23.13419589	22.80954676	24.81887259	27.50227523	32.44827615	31.37136757	24.72664493	24.71905904	30.03866303	30.24884171	32.92320118	26.54677314

NUP188	28.71418006	28.22999802	33.6416429	40.76738977	39.48368185	38.79390928	31.87966388	40.8094865	24.98622733	25.68425707	26.31939696	28.70250846
NUP205	24.12773396	24.64879796	26.36266048	32.52021709	36.20191151	35.79870739	28.09066264	29.50509746	34.68909812	41.00606647	39.48744928	29.81018157
NUP210	12.94308839	16.97374692	14.62536668	23.88059078	16.79168293	20.2304398	12.6423707	21.38895733	39.85463609	49.88312456	41.73425108	58.4176674
NUP210L	0.044901169	0.009309767	0.009504803	0.009076296	0	0.018444746	0.009234841	0.017574409	0.00977274	0.039388768	0.029019545	0.056073664
NUP214	15.92206607	15.715316	17.0308041	18.39227373	17.90165413	16.97548031	15.42218696	17.74038305	18.42942762	15.41562397	15.55779698	20.41370324
NUP35	5.473870493	5.004656564	6.5632522	7.920962994	9.132463892	8.318109567	7.186924957	8.301137348	7.715446032	8.26149836	8.463627148	7.567418762
NUP37	46.31867072	45.12775816	51.62309334	64.5219247	69.96190703	65.6030966	63.29521523	66.4982459	32.60784616	39.65568547	38.19286274	30.40311445
NUP43	12.60360845	13.19062044	14.46702346	15.94327115	19.85768553	18.74908416	16.41115916	16.6734273	15.50225287	16.46327409	15.95671901	16.48300921
NUP50	15.08291753	15.85132371	16.52170105	24.76974206	25.95110285	24.25463055	19.14388935	23.36373317	31.77506965	29.09013222	31.64677382	30.72792362
NUP54	15.84445723	15.61118701	16.28667458	15.57388586	21.15737355	19.42350044	17.52771747	15.91953546	15.439872	14.90538764	15.98036314	13.72619545
NUP58	31.43404384	29.89024875	33.48378439	33.64860935	37.95686041	33.75392131	34.54216223	28.9398866	22.11969687	26.73653908	24.27367972	20.14722569
NUP62	34.92197405	32.18255461	35.92382008	31.75096559	31.49936515	31.04108609	33.86432809	36.39610041	32.95822367	31.38426539	35.88791483	36.83771804
NUP62CL	0	0	0	0	0	0	0	0	0	0.033487159	0	0.031781441
NUP85	15.28606946	17.07505534	18.60068219	22.56754885	23.89125032	23.13073477	18.37274422	23.92881875	23.92835896	21.58392825	26.11422957	24.72327741
NUP88	19.55698251	16.46230876	19.5641576	18.40057348	24.50809104	22.4160338	22.23147309	20.60865328	11.0658886	10.41702907	12.04847218	8.56726617
NUP93	26.26132839	25.3191007	29.14246129	35.34678632	29.10490936	32.66248918	30.35072151	36.75326613	23.97733627	26.53258463	28.68380762	27.75661582
NUP98	30.10254836	24.86159798	29.76287849	32.44835075	30.24367194	28.12196827	30.57035597	29.27201379	29.58157628	25.90758341	27.77977857	31.44580355
NUPL2	19.96807847	17.20911057	19.96742444	17.54752459	18.0241556	18.09759429	19.85386239	18.18526995	16.51587206	14.92696356	15.61337993	18.46461456
NUPR1	0.236006846	1.039835602	0.437137576	0.05963286	0	0.121185216	0.060674526	0	4.109351373	2.846702271	5.465686968	2.27188515
NUPR2	0	0	0	0	0	0	0	0	0.097597095	0.098340624	0.386411358	0.093331498
NUS1	25.10739552	22.58066945	26.55030804	26.34935952	29.4556807	26.87693895	30.03415564	22.39804069	23.6182939	24.69465074	25.83849516	23.99619827
NUSAP1	30.04782469	34.06714812	35.80180815	82.28458135	65.30225476	63.36708694	39.61250739	76.81632776	68.14592508	75.92824629	75.31384799	57.20353604
NUTF2	23.72956542	22.55517211	29.45079229	26.64189649	25.0525635	25.044719	31.55109258	32.31977933	24.74004576	26.61789513	29.66894468	24.32354405
NUTM1	0	0	0	0	0	0	0	0	0	0.014062053	0	0
NUTM2A	0.319994259	0.157138371	0.240645555	0.204263544	0.149479746	0.147015304	0.147214062	0.197757477	0.082476418	0.036935446	0.072565513	0.18403394
NUTM2B	0.032691106	0	0.121102512	0.033040807	0.034135358	0.016786288	0	0.04798262	0.729310006	0.125464952	0.21128203	0.527328634
NUTM2D	0.252399167	0.251597157	0.174670266	0.313968139	0.182457582	0.199388268	0.279520963	0.484448736	0.433138831	0.127738137	0.303245428	0.40410535
NUTM2E	0.79221324	0.67688224	0.939845283	1.055851841	0.818121967	0.670528133	0.859436356	0.758147649	0.75791305	0.410481815	0.468869954	0.98752305
NUTM2F	0	0.021782164	0.022238493	0	0	0	0	0	0	0.023039584	0	0
NUTM2G	0	0.010627571	0	0	0	0	0	0	0	0	0	0
NVL	14.14609285	12.43156767	14.45414369	13.04364205	15.10055721	14.85159746	13.85337469	13.77813736	10.17220279	9.569566881	10.20923031	9.614371105
NWD1	0	0.012495043	0	0.024363386	0	0.006188874	0.006197241	0.005896845	0.01967463	0.013216345	0.051931194	0.062715756
NWD2	0	0	0	0	0	0	0	0	0	0	0	0
NXF1	30.90773426	30.48150947	31.29862276	37.90044303	26.3794784	25.03843782	32.9182202	39.14371305	35.61223157	23.78956207	31.05640355	48.1263746
NXF2	0	0	0	0	0	0	0	0	0	0	0	0
NXF2B	0	0	0	0	0	0	0	0	0	0	0	0
NXF3	42.79789967	48.42862042	41.21266664	40.07177243	36.65773729	48.67350517	50.46123695	48.1818727	34.71491611	25.61213085	22.1024851	43.59407947
NXF5	0.018873925	0	0.019976423	0.019075822	0	0.038765667	0	0.036936463	0.30809325	0.08278411	0.142312145	0.058925534
NXN	0.113303164	0.088095648	0.159895508	0.181315705	0.059154377	0.087268666	0.097096276	0.138584651	4.59300595	3.426995597	3.457971441	4.981828691
NXNL1	0.059261631	0	0	0	0	0	0.060941815	0	0	0	0	0
NXNL2	0	0	0.02080847	0.019870358	0	0	0	0	0.085580208	0.129348281	0.127062558	0.081839823
NXPE1	0	0	0	0	0	0	0	0	0	0	0	0
NXPE2	0.00475896	0.004933592	0.020147796	0	0.004969205	0.009774557	0	0.009313333	0	0	0	0.004952587
NXPE3	2.992376942	3.255453195	3.135876847	4.488763778	1.86486572	2.684374402	2.779225379	3.263682995	7.465389405	6.893246503	6.975279207	10.9794522
NXPE4	0	0	0	0	0	0	0	0	0	0	0	0
NXPH1	0	0	0	0	0	0	0	0	0	0	0	0.01917114
NXPH2	0	0	0	0	0	0	0	0	0	0	0	0
NXPH3	0	0	0	0	0	0	0	0	0.010545337	0.031877026	0.073065357	0.070591084
NXPH4	0.23862333	0.154612311	0.220991945	0.422057849	0.404893782	0.490114932	0.214715175	0.55454858	0.649204624	0.490612869	0.77110914	1.21061921
NXT1	13.79618584	14.30244175	16.1514895	18.83080858	13.94247847	15.12487407	15.73836185	19.57664524	10.71717483	12.40405232	13.28388592	13.9881833
NXT2	7.893619953	8.338330729	7.844638808	9.32173211	7.270620329	8.908572999	6.647739439	8.650820836	10.25402463	9.001902696	8.914431261	7.090657357
NYAP1	0	0	0.029050131	0	0	0	0	0	0	0.06019319	0.044347134	0.01428179
NYAP2	0.015233792	0.011844601	0.008061828	0	0	0.003911137	0	0	0.049734524	0.025056709	0.061534826	0.003963401
NYNRIN	0.011836683	0.012271034	0.012528109	0	0.006179806	0.012155842	0	0	1.706768381	1.564018282	1.625630712	1.016258063
NYX	0	0	0	0	0	0	0	0	0	0.017400287	0.017092806	0
OAF	19.5312217	19.09227816	19.49225532	18.91862145	18.01760526	18.74610291	18.34156622	18.86142879	57.29459161	59.9466108	69.2688638	81.88358622
OARD1	11.79387635	12.82100818	11.09582345	11.56133214	13.25563742	14.6632267	11.62291197	11.75407837	10.01224385	11.19616236	9.263285973	9.205298439
OAS1	7.954742656	2.01054562	1.600383592	0.913617677	1.32143662	0.911443116	1.69013952	0.997092717	16.47286339	5.26245489	7.347008863	7.217939923
OAS2	5.189931709	1.157459487	0.803192085	0.211581139	0.491828031	0.421015003	0.58304197	0.162166581	19.36437744	6.150075005	7.601072825	7.552453207
OAS3	2.064616013	0.654703806	0.608433264	0.441889724	0.338169116	0.241130499	0.516217335	0.522884934	23.04971419	12.10983548	11.79118618	15.48690582
OASL	1.156158873	0.249705111	0.178455447	0.048688602	0.150904562	0.04947221	0.148617282	0.070706708	3.119262574	0.924419473	1.323208096	1.403732469
OAT	7.309746281	7.374202389	8.386882178	8.890360894	5.336463066	5.652211287	7.100083184	7.994108981	31.48509027	25.94575913	31.97156987	27.90995346
OAZ1	895.6261963	898.8281556	1026.500341	1168.014747	806.2163333	844.956275	1038.951746	1258.320279	549.8823631	601.0852056	611.9106869	624.6176921
OAZ2	24.26160138	23.16165963	25.67881349	23.48067	19.26155506	19.0011401	24.89224201	20.69091289	27.82576951	33.62089996	30.98883226	30.02629684
OAZ3	0.367300757	0.14279212	0.58313428	0.232018637	0.239704772	0.23575281	0.519357378	0.673885601	0.499643832	0.453105264	0.544009164	1.051173869
OBP2A	0	0	0	0	0	0	0	0	0	0	0	0
OBP2B	0	0	0	0	0	0	0	0	0	0	0	0
OBSCN	0.229628282	0.256810388	0.209163195	0.180041422	0.502796646	0.408754459	0.163150372	0.166136228	1.091956636	0.695874685	0.986390819	0.53732294
OBSL1	3.098373854	3.565044477	3.128006465	2.649745623	3.036163378	3.587524076	3.480331364	3.471548425	8.685178091	5.645065427	6.660241321	7.653608042
OC90	0	0.031304221	0	0	0	0	0	0	0	0	0.032526209	0
OCA2	0	0	0	0	0	0	0	0	0.010157547	0	0	0
OCEL1	8.830286911	9.583426035	8.177835264	8.738813416	6.819252483	7.556985787	8.087447613	9.135508077	7.107070515	5.749144167	5.598010695	11.34336669
OCIAD1	48.21125894	48.56928425	48.54115061	49.54797286	49.21789337	47.52715082	50.70697146	48.76464598	59.70505279	59.7506557	58.60020468	58.32933037
OCIAD2	0.913131942	0.946639641	0.621303069	0.197764249	4.154417838	3.884977693	6.975584799	3.510195222	6.67209232	6.365320384	6.252838334	5.498073704
OCLM	2.069598495	1.262084201	1.675081803	1.476520414	1.779672355	1.500283943	1.251926875	0.893432042	0.861150839	1.134699507	0.458972767	0.823513218
OCLN	0.155471663	0.212074672	0.025982108	0.157134763	0.222149751	0.252100632	0.176709022	0.192163972	0.142477511	0.071781477	0.061698894	0.14476601
OCM	0.073914232	0.076626541	0	0	0	0.22772167	0.076009846	0.144650902	0.241311499	0	0.079617725	0.230764693
OCM2	0	0	0	0	0	0	0	0	0	0	0	0
OCRL	8.042288802	8.242982099	7.509515341	9.481503666	7.456068065	7.810169753	7.558476723	7.833771834	12.34996417	13.71242484	11.15478285	18.12286638
OCSTAMP	1.247600705	0.646690927	1.521420065	0.054823759	1.047838483	0.278530537	0.557814192	0.159232646	0.088545752	0.059480216	0.262931115	0
ODAM	0	0	0	0	0	0	0	0	0	0	0	0
ODAPH	0.01867739	0	0.039536815	0.018877185	0.019502533	0.019180999	0.038413862	0	0.081302682	0.143363631	0.060355818	0.097186565
ODC1	52.96942682	47.98794018	59.59872164	77.62209136	68.36131821	64.07772138	64.54364783	80.48318899	83.75659312	84.39468014	97.58137543	98.75510301
ODF1	0	0	0	0	0	0	0	0	0	0	0.058311573	0
ODF2	25.558737	24.89548505	26.18897079	43.90271733	33.83303143	33.6497311	29.33855821	43.1130615	33.93040727	34.60053206	34.91334831	38.01719144
ODF2L	2.749202049	2.598406839	2.673676479	2.480191813	2.82955101	2.796377308	2.70569471	2.195744603	3.120346096	2.827547753	2.897731801	2.908856347
ODF3	0	0	0	0	0	0	0	0	0	0	0	0
ODF3B	1.4977919	0.74762225	0.645856283	0.616739025	0.289622673	0.284847725	0.684558778	0.434250955	1.207386744	1.27741429	0.776806338	1.385539765
ODF3L1	0.161798508	0.047924503	0.097857013	0.046722653	0	0	0.213924618	0.067851712	0.100615562	0.177418651	0.224078751	0.120272549
ODF3L2	0.020428839	0.10589241	0.021622165	0.020647368	0.085325435	0	0.021008036	0.059969167	0.444633691	0.537625279	0.462109255	0.488980514
ODF4	0	0	0	0	0	0	0	0	0	0	0	0
ODR4	12.56050045	11.56993667	11.02863466	12.84453319	12.49688866	11.66870829	12.66742022	11.68455027	7.398528851	7.189174458	6.917121002	7.929791529
OFCC1	0	0.097100299	0	0	0	0	0	0	0	0	0	0
OFD1	5.31459577	7.860625835	4.989857622	6.491227094	6.567679411	8.456479826	5.376264032	5.606901644	7.730317903	6.194668363	5.867651287	5.34466702
OGDH	33.29170389	30.33736162	33.5834455	32.79874159	27.38487884	26.93339003	28.93023589	33.98832943	20.79310409	23.19547462	24.51422735	21.06123517
OGDHL	0.156309545	0.148541597	0.110293452	0.039495402	0.272025169	0.227409295	0.107160819	0.140203876	1.034798539	0.971265421	1.220689474	0.610009791
OGFOD1	20.62725074	17.631739	23.03437848	21.30710799	21.12737489	20.68573315	24.07772855	20.00603806	17.75042017	17.47047617	19.85347407	18.6295239
OGFOD2	10.72718388	11.92088079	10.33277423	11.11493227	11.12051375	13.07705366	10.31706249	12.34672468	3.54834903	2.961854575	3.408289494	4.276717624
OGFOD3	3.505431175	3.16435523	3.432562933	4.916718101	3.847046483	4.224431244	3.617078335	5.285126641	3.256840798	3.425469993	3.429154637	4.131981678
OGFR	15.64768825	16.81509108	17.37700403	20.50843577	20.52139516	19.77623773	19.98402995	23.43001473	26.82423227	26.90792532	25.48418464	36.55388243
OGFRL1	16.32226445	16.86821645	14.94899831	12.02035295	12.42825974	11.61720126	14.36190875	9.522821896	19.86671565	20.81308212	19.94467329	22.90160529
OGG1	2.97525218	3.194588328	3.407719734	3.855504646	3.605985299	3.81934449	3.147023766	3.940651626	3.896945622	3.413957679	3.960258583	4.467526685
OGN	0.028268747	0.01465304	0.044880048	0	0.014758812	0.014515487	0.058140444	0	0.046145199	0.030997833	0.030450068	0
OGT	47.13644734	42.33807297	38.78824711	33.90029281	48.87047931	43.36108136	40.64358723	29.78719215	93.7328223	76.91855475	73.20687329	84.81276291
OIP5	5.616046401	5.686730853	7.003901908	13.1562827	10.54638939	10.81960435	8.55098468	13.24843099	10.18610459	7.303939543	9.80096769	10.1939743
OIT3	0	0.023311376	0.023799741	0.022726773	0	0	0	0	0.073411939	0.123285362	0.121106777	0.187209022
OLA1	41.7701467	41.93023866	40.78436811	35.0176547	42.84739463	40.84413935	44.4042974	36.86544742	47.83124893	43.82644347	47.48924273	33.17319138
OLAH	0.320136207	0.193598836	0.112945526	0.080890186	0.083569854	0	0.109737566	0.052209151	0.087097061	0.087760596	0.057473182	0.02776346
OLFM1	0	0	0	0	0	0.014334749	0	0	0	0.015305934	0	0
OLFM2	0	0.048214453	0	0.047005332	0	0	0.02391321	0	0.025306075	0.076496596	0.025048273	0
OLFM3	0	0	0	0	0	0	0	0	0	0	0	0
OLFM4	0	0	0	0.018631438	0	0	0	0	0.040122136	0.0404278	0	0
OLFML1	0	0	0	0	0	0	0	0	0	0	0	0
OLFML2A	0	0.016523733	0.042174749	0.008054675	0.033286018	0.016368619	0.008195374	0	0.026018183	0.034955198	0.034337502	0.082936758
OLFML2B	0.015449199	0.048048339	0.010901097	0.020819282	0.016131725	0.031731529	0.037070167	0.01007808	0.016812592	0	0.011094211	0.010718518
OLFML3	0	0.023617325	0.048224201	0.02302505	0	0.023395622	0.070281754	0.022291672	3.49564532	1.274014857	3.141023738	1.114287995
OLIG1	5.33609511	8.640541886	6.97227994	4.080677416	1.873715093	3.806885474	4.103397706	3.604149547	2.620861001	2.744389505	2.56872842	2.45716976
OLIG2	5.206745431	5.702196507	5.365867718	2.829057515	2.207411514	3.417343745	4.770620148	3.945617765	2.151467429	3.133735415	2.972935691	2.220400136
OLIG3	0	0	0	0	0	0	0	0	0	0	0	0
OLR1	0.085547792	0.022171749	0.045272481	0.086462907	0.022331796	0	0.02199331	0	0	0.023451659	0	0
OMA1	12.2142695	11.04721748	11.83816861	12.99170005	11.24317015	10.8292212	11.9311091	12.76847124	8.720154102	10.12898257	9.35059542	10.22110201
OMD	0	0	0	0	0	0	0	0	0	0	0	0
OMG	0	0.088312594	0.030054238	0.057398595	0.029650026	0.116644767	0.029200616	0.111140746	0.309014549	0.435916222	0.367039812	0.295508701
OMP	0	0	0.115757686	0	0	0	0.112469853	0	0.357062543	0	0.117808341	0.5690945
ONECUT1	0.018953702	0.029473823	0.02006086	0.009578226	0.019791053	0.019464762	0.029236616	0.037092588	0.051565919	0.093525778	0.020416239	0.059174603
ONECUT2	6.675295976	4.539682897	6.249191795	3.2788974	8.733663154	6.118342547	6.18496294	2.857667305	8.038048808	10.81490672	7.657761369	6.033375963
ONECUT3	0	0	0	0	0	0	0	0	0.078866339	0.039733585	0	0
OOEP	0	0	0	0	0	0	0	0	0.08624191	0.173797863	0	0
OOSP2	0	0	0	0	0	0	0	0	0	0	0	0
OPA1	21.98051975	20.30061763	22.93412259	20.71302025	23.20889591	22.67441805	24.5380343	16.85440249	21.86437136	24.93117206	24.06591878	20.10523738
OPA3	4.674993281	3.742704651	4.613509853	5.044486421	4.012929141	4.123319798	4.05475671	4.981489045	3.995214488	3.083089539	3.458706216	4.397728632
OPALIN	0.01361578	0	0	0	0	0	0	0	0	0	0	0
OPCML	0	0	0	0	0	0	0	0	0	0.003091338	0	0
OPHN1	1.138934294	1.16491457	1.10859614	1.187090045	1.008739652	0.992108771	0.982992683	1.000022545	2.240961364	1.962538011	1.467800867	2.672157603
OPLAH	5.97609625	7.334626373	5.854714553	3.082408592	4.950833106	6.365477329	6.653425417	4.421976082	3.816095687	2.65370752	3.31171735	5.422564321
OPN1LW	0	0	0	0	0	0	0	0	0	0	0	0
OPN1MW	0	0	0	0	0	0	0	0	0	0	0	0
OPN1MW2	0	0	0	0	0	0	0	0	0	0	0	0

OPN1MW3	0	0	0	0	0	0	0	0	0	0	0	0
OPN1SW	0	0	0	0	0	0	0	0	0.053331746	0	0	0
OPN3	26.4345601	29.24851366	28.75769238	30.8292825	33.64376696	32.66917687	27.58348794	32.66802126	8.165228093	8.586122863	6.848818327	8.595575657
OPN4	0	0	0.026832877	0	0	0	0.013035375	0	0	0	0	0
OPN5	0	0	0	0	0	0	0	0	0	0	0	0
OPRD1	0	0	0	0	0	0	0	0	0	0	0	0
OPRK1	0	0	0	0	0	0	0	0	0	0	0	0
OPRL1	4.454035239	3.591371494	3.496728059	2.041302625	2.136858113	2.211517233	3.026034535	1.910844528	2.925729473	2.508015912	2.535734488	3.855753164
OPRM1	0	0.002405629	0	0	0	0	0	0	0.00252526	0	0	0
OPRPN	0	0	0	0	0	0	0	0	0	0	0.052740404	0
OPTC	0	0	0	0	0	0	0	0	0	0	0	0
OPTN	19.07344734	17.67970496	15.89674518	8.058741969	5.88891384	5.884002122	11.76853028	6.030860848	9.84924813	10.07191711	7.557419799	10.04150396
OR10A2	0	0	0	0	0	0	0	0	0	0	0	0
OR10A3	0	0	0	0	0	0	0	0	0	0	0	0
OR10A4	0	0	0	0	0	0	0	0	0	0	0	0
OR10A5	0	0	0	0	0	0	0	0	0	0	0	0
OR10A6	0	0	0	0	0	0	0	0	0	0	0	0
OR10A7	0	0	0	0	0	0	0	0	0	0	0	0
OR10AC1	0	0	0	0	0	0	0	0	0	0	0	0
OR10AD1	0	0	0	0	0	0	0	0	0	0	0	0
OR10AG1	0	0	0	0	0	0	0	0	0	0	0	0
OR10C1	0	0	0	0	0	0	0	0	0	0	0	0
OR10D3	0	0	0	0	0	0	0	0	0	0	0	0
OR10G2	0	0	0	0	0	0	0	0	0	0	0	0
OR10G3	0	0	0	0	0	0	0	0	0	0	0	0
OR10G4	0	0	0	0	0	0	0	0	0	0	0	0
OR10G6	0	0	0	0	0	0	0	0	0	0	0	0
OR10G7	0	0	0	0	0	0	0	0	0	0	0	0
OR10G8	0	0	0	0	0	0	0	0	0	0	0	0
OR10G9	0	0	0	0	0	0	0	0	0	0	0	0
OR10H1	0	0	0	0	0	0	0	0	0	0	0	0
OR10H2	0	0	0	0	0	0	0	0	0	0	0	0
OR10H3	0	0	0	0	0	0	0	0	0	0	0	0
OR10H4	0	0	0	0	0	0	0	0	0	0	0	0
OR10H5	0	0	0	0	0	0	0	0	0	0	0	0
OR10J1	0	0	0	0	0	0	0	0	0	0	0	0
OR10J3	0	0	0	0	0	0	0	0	0	0	0	0
OR10J4	0	0	0	0	0	0	0	0	0	0	0	0
OR10J5	0	0	0	0	0	0	0	0	0	0	0	0
OR10K1	0	0	0	0	0	0	0	0	0	0	0	0
OR10K2	0	0	0	0	0	0	0	0	0	0	0	0
OR10P1	0	0	0	0	0	0	0	0	0	0	0	0
OR10Q1	0	0	0	0	0	0	0	0	0	0	0	0
OR10R2	0	0	0	0	0	0	0	0	0	0	0	0
OR10S1	0	0	0	0	0	0	0	0	0	0	0	0
OR10T2	0	0	0	0	0	0	0	0	0	0	0	0
OR10V1	0	0	0	0	0	0	0	0	0	0	0	0
OR10W1	0	0	0	0	0	0	0	0	0	0	0	0
OR10X1	0	0	0	0	0	0	0	0	0	0	0	0
OR10Z1	0	0	0	0	0	0	0	0	0	0	0	0
OR11A1	0	0	0	0	0	0	0	0	0	0	0	0
OR11G2	0	0	0	0	0	0	0	0	0	0	0	0
OR11H1	0	0	0	0	0	0	0	0	0	0	0	0
OR11H12	0	0	0	0	0	0	0	0	0	0	0	0
OR11H2	0	0	0	0	0	0	0	0	0	0	0	0
OR11H4	0	0	0	0	0	0	0	0	0	0	0	0
OR11H6	0	0	0	0	0	0	0	0	0	0	0	0
OR11H7	0	0	0	0	0	0	0	0	0	0	0	0
OR11L1	0	0	0	0	0	0	0	0	0.241726551	0.060892027	0.119631999	0
OR12D1	0	0	0	0	0	0	0	0	0	0	0	0
OR12D2	0	0	0	0	0	0	0	0	0	0	0	0
OR12D3	0	0	0	0	0	0	0	0	0	0	0	0
OR13A1	0	0	0	0	0	0	0	0	0.018572235	0	0	0.008880257
OR13C2	0	0	0	0	0	0	0	0	0	0.061655564	0	0
OR13C3	0	0	0	0	0	0	0	0	0	0	0	0
OR13C4	0	0	0	0	0	0	0	0	0	0	0	0
OR13C5	0	0	0	0	0	0	0	0	0.061189401	0	0	0
OR13C8	0	0	0	0	0	0	0	0	0	0	0	0
OR13C9	0	0	0	0	0	0	0	0	0	0	0	0
OR13D1	0	0	0	0	0	0	0	0	0	0	0	0
OR13F1	0	0	0	0	0	0	0	0	0	0	0	0
OR13G1	0	0	0	0	0	0	0	0	0	0	0	0
OR13H1	0	0	0	0	0	0	0	0	0	0	0	0
OR13J1	0	0	0	0	0	0	0	0	0	0	0	0
OR14A16	0	0	0	0	0	0	0	0	0	0	0	0
OR14A2	0	0	0	0	0	0	0	0	0	0	0	0
OR14C36	0	0	0	0	0	0	0	0	0	0	0	0
OR14I1	0	0	0	0	0	0	0	0	0	0	0	0
OR14J1	0	0	0	0	0	0	0	0	0	0	0	0
OR14K1	0	0	0	0	0	0	0	0	0	0	0	0
OR1A1	0	0	0	0	0	0	0	0	0	0	0	0
OR1A2	0	0	0	0	0	0	0	0	0	0	0	0
OR1B1	0	0	0	0	0	0	0	0	0	0	0	0.026377249
OR1C1	0	0	0	0	0	0	0	0	0	0	0	0
OR1D2	0	0	0	0	0	0	0	0	0	0	0	0
OR1D4	0	0	0	0	0	0	0	0	0	0	0	0
OR1D5	0	0	0	0	0	0	0	0	0	0	0	0
OR1E1	0	0	0	0	0	0	0	0	0	0	0	0
OR1E2	0	0	0	0	0	0	0	0	0	0	0	0
OR1E3	0	0	0	0	0	0	0	0	0	0	0	0
OR1F1	0.782071132	0.953846481	1.006290237	0.774938282	0.912695219	1.212611943	0.630779913	0.750255462	0.250320278	0.571715226	0.479022344	0.303214328
OR1F12	0	0	0	0	0	0	0	0	0	0	0	0
OR1G1	0	0	0	0	0	0	0	0	0	0	0	0
OR1I1	0	0	0	0	0	0	0	0	0	0	0	0
OR1J1	0	0	0	0	0	0	0	0	0	0	0	0
OR1J2	0	0	0.013146995	0	0.012970175	0	0	0	0	0.013620585	0.013379895	0
OR1J4	0	0	0	0	0	0	0	0	0	0	0	0
OR1K1	0	0	0	0	0	0	0	0	0	0	0	0
OR1L1	0	0	0	0	0	0	0	0	0	0	0	0
OR1L3	0	0	0	0	0	0	0	0	0	0	0	0
OR1L4	0	0	0	0	0	0	0	0	0	0	0	0
OR1L6	0	0	0	0	0	0	0	0	0	0	0	0
OR1L8	0	0	0	0.022179922	0	0	0.045134721	0	0	0.024063774	0	0.02283805
OR1M1	0	0	0	0	0	0	0	0	0	0	0	0
OR1N1	0	0	0	0	0	0	0	0	0	0	0	0
OR1N2	0	0	0	0	0	0	0	0	0	0	0	0
OR1Q1	0	0	0	0	0	0	0	0	0	0	0	0
OR1S1	0	0	0	0	0	0	0	0	0	0	0	0
OR1S2	0	0	0	0	0	0	0	0	0	0	0	0
OR2A1	0	0	0	0	0	0	0	0	0	0	0	0
OR2A12	0	0	0	0	0	0.059228787	0	0	0	0	0	0
OR2A14	0	0	0	0	0	0	0	0	0	0	0	0
OR2A2	0	0	0	0	0	0	0	0	0	0	0	0
OR2A25	0	0	0	0	0	0	0	0	0	0	0	0
OR2A4	0	0	0	0	0	0	0	0	0	0	0	0.031853754
OR2A42	0	0	0	0	0	0	0	0	0	0	0	0
OR2A5	0	0	0	0	0	0	0	0	0	0	0	0
OR2A7	0	0	0	0	0	0	0	0	0.376580431	0.063241559	0.062124012	0.060020256
OR2AE1	0.055359631	0.057391072	0.058593396	0.111903639	0	0	0	0.054169679	0	0	0.119262765	0.057612036
OR2AG1	0	0	0	0	0	0	0	0	0	0	0	0
OR2AG2	0.113164166	0.058658382	0.179661771	0.285936743	0.236327229	0.174323213	0.232745186	0.166097566	0	0.062044558	0	0
OR2AJ1	0	0	0	0	0	0	0	0	0	0	0	0
OR2AK2	0	0	0	0	0.055740872	0.054821883	0	0	0	0	0	0
OR2AP1	0	0	0	0	0	0	0	0	0	0	0	0
OR2AT4	0	0	0	0	0	0	0	0	0	0	0	0
OR2B11	0	0	0	0	0	0	0	0	0	0	0	0
OR2B2	0	0	0	0	0	0	0	0	0	0	0	0
OR2B3	0	0	0	0	0	0	0	0	0	0	0	0
OR2B6	0.114245352	0.059218813	0.060459428	0.115467449	0.298231415	0.175988722	0.117484433	0.167684485	0.12432751	0	0	0.059446814
OR2C1	0	0	0	0	0	0.053340211	0	0.05082329	0.056523414	0.170862088	0.05594759	0
OR2C3	0	0	0	0	0	0	0	0	0	0	0	0
OR2D2	0.058046991	0	0.122875472	0	0.1818343	0	0.059692738	0	0	0	0	0
OR2D3	0	0	0	0	0	0	0	0	0	0	0	0
OR2F1	0	0	0	0.011958041	0.024708355	0	0	0.011577161	0	0	0	0
OR2F2	0	0	0	0	0	0	0	0	0	0	0	0
OR2G2	0	0	0	0	0	0	0	0	0	0	0	0
OR2G3	0	0	0	0	0	0	0	0	0	0	0	0
OR2G6	0	0	0	0	0	0	0	0	0	0	0	0

OR2H1	0	0	0	0	0	0	0	0	0	0	0	0
OR2H2	0	0	0	0	0	0	0	0	0	0	0	0
OR2J1	0	0	0	0	0	0	0	0	0	0	0	0
OR2J2	0	0	0	0	0	0	0	0	0	0	0	0
OR2J3	0	0	0	0	0	0	0	0	0	0	0	0
OR2K2	0	0	0	0	0.032704772	0	0	0	0	0	0	0
OR2L13	0	0.025994465	0	0.025342576	0	0	0	0.024535381	0	0.027495049	0	0.026094547
OR2L2	0	0.040837571	0	0.039813447	0	0	0	0.038545336	0	0	0	0
OR2L3	0.057305177	0	0	0	0	0	0	0	0	0	0	0
OR2L5	0	0	0	0	0	0	0	0	0	0	0	0
OR2L8	0	0	0	0	0.059836846	0.058850329	0	0	0	0	0	0
OR2M2	0	0	0	0	0	0	0	0	0	0	0	0
OR2M3	0	0	0	0	0	0	0	0	0	0	0	0
OR2M4	0	0	0	0	0	0	0	0	0	0	0	0
OR2M5	0	0	0	0	0	0	0	0	0	0	0	0
OR2M7	0	0	0	0	0	0	0	0	0	0	0	0
OR2S2	0	0	0	0	0	0	0	0	0	0	0	0
OR2T1	0	0	0	0	0	0	0	0	0	0	0	0
OR2T10	0	0	0	0	0	0	0	0	0	0	0	0
OR2T11	0	0	0	0	0	0	0	0	0	0	0	0
OR2T12	0	0	0	0	0	0	0	0	0	0	0	0
OR2T2	0	0	0	0	0	0	0	0	0	0	0	0
OR2T27	0	0	0	0	0	0	0	0	0	0	0	0
OR2T29	0	0	0	0	0	0	0	0	0	0	0	0
OR2T3	0	0	0	0	0	0	0	0	0	0	0	0
OR2T33	0	0	0	0	0	0	0	0	0.060808159	0.122542834	0.300943425	0.407053263
OR2T34	0	0	0	0	0	0	0	0	0	0	0	0
OR2T35	0	0	0	0	0	0	0	0	0	0	0	0
OR2T4	0	0	0	0	0	0	0	0	0	0	0	0
OR2T5	0	0	0	0	0	0	0	0	0	0	0	0
OR2T6	0	0	0	0	0	0	0	0	0	0	0.06252611	0.060408737
OR2T7	0	0	0	0	0	0	0	0	0	0	0	0
OR2T8	0.015471409	0	0	0	0.016154916	0.015888573	0.015910054	0.030277705	0.084183808	0.30537054	0.166652397	0.386421384
OR2V1	0	0	0	0	0	0	0	0	0	0	0	0
OR2V2	0	0	0	0	0	0	0	0	0	0	0	0
OR2W1	0	0	0	0	0	0	0	0	0	0	0	0
OR2W3	0.113882668	0.118061633	0.120534987	0.057550443	0	0.233906703	0.117111466	0.278586922	1.797025878	3.059486077	1.472043268	2.07403329
OR2W5	0	0	0	0	0	0	0	0	0	0	0	0
OR2Y1	0	0	0	0	0	0	0	0	0	0	0	0
OR2Z1	0	0	0	0	0	0	0	0	0	0	0	0
OR3A1	0	0	0	0	0	0	0	0	0	0	0	0
OR3A2	0	0	0	0	0	0	0	0	0	0	0	0
OR3A3	0	0	0	0	0	0	0	0	0	0	0	0
OR4A15	0	0	0	0	0	0	0	0	0	0	0	0
OR4A16	0	0	0	0	0	0	0	0	0	0	0	0
OR4A47	0	0	0	0	0	0	0	0	0	0	0	0
OR4A5	0	0	0	0	0	0	0	0	0	0	0	0
OR4B1	0	0	0	0	0	0	0	0	0	0	0	0
OR4C11	0	0	0	0	0	0	0	0	0	0	0	0
OR4C12	0	0	0	0	0	0	0	0	0	0	0	0
OR4C13	0	0	0	0	0	0	0	0	0	0	0	0
OR4C15	0	0	0	0	0	0	0	0	0	0	0	0
OR4C16	0	0	0	0	0	0	0	0	0	0	0	0
OR4C3	0	0	0	0	0	0	0	0	0	0	0.058547175	0
OR4C45	0	0	0	0	0	0	0	0	0	0	0	0
OR4C46	0	0	0	0	0	0	0	0	0	0	0	0
OR4C5	0	0	0	0	0	0	0	0	0	0	0	0
OR4C6	0	0	0	0	0	0	0	0	0	0	0	0
OR4D1	0	0	0	0	0	0	0	0	0	0.063241559	0	0
OR4D10	0	0	0	0	0	0	0	0	0	0	0	0
OR4D11	0	0	0	0	0	0	0	0	0	0	0	0
OR4D2	0	0	0	0	0	0	0	0	0	0	0	0
OR4D5	0	0	0	0	0	0	0	0	0	0	0	0
OR4D6	0	0	0	0	0	0	0	0	0	0	0	0
OR4D9	0	0	0	0	0	0	0	0	0	0	0	0
OR4E1	0	0	0	0	0	0	0	0	0	0	0	0
OR4E2	0	0	0	0	0	0	0	0	0	0	0	0
OR4F15	0	0	0	0	0	0	0	0	0	0	0	0
OR4F16	0.009309613	0	0.009853422	0.009409199	0	0.019121266	0	0.009109503	0	0	0	0.009688391
OR4F17	0	0	0	0	0	0	0	0	0	0	0	0
OR4F21	0.057305177	0.05940801	0	0	0	0	0	0	0	0	0	0
OR4F29	0	0	0	0	0	0	0	0	0	0	0	0
OR4F3	0	0	0	0	0	0	0	0	0	0	0	0
OR4F4	0	0	0	0	0	0	0	0	0	0	0	0
OR4F5	0	0	0	0	0	0	0	0	0	0	0	0
OR4F6	0	0	0	0	0	0	0	0	0	0	0	0
OR4K1	0	0	0	0	0	0	0	0	0	0	0	0
OR4K13	0	0	0	0	0	0	0	0	0	0	0	0
OR4K14	0	0	0	0	0	0	0	0	0	0	0	0
OR4K15	0	0	0	0	0	0	0	0	0	0	0	0
OR4K17	0	0	0	0	0	0	0	0	0	0	0	0
OR4K2	0	0	0	0	0	0	0	0	0	0	0	0
OR4K3	0	0	0	0	0	0	0	0	0	0	0	0
OR4K5	0	0	0	0	0	0	0	0	0	0	0	0
OR4L1	0	0	0	0	0	0	0	0	0	0	0	0
OR4M1	0	0	0	0	0	0	0	0	0	0	0	0
OR4M2	0	0	0	0	0	0	0	0	0	0	0	0
OR4N2	0	0	0	0	0	0	0	0	0	0	0	0
OR4N4	0	0	0	0	0	0	0	0	0	0	0	0
OR4N5	0	0	0	0	0	0	0	0	0	0	0	0
OR4P4	0	0	0	0	0	0	0	0	0	0	0	0
OR4Q2	0	0	0	0	0	0	0	0	0	0	0	0
OR4Q3	0	0	0	0	0	0	0	0	0	0	0	0
OR4S1	0	0	0	0	0	0	0	0	0	0	0	0
OR4S2	0	0	0	0	0	0	0	0	0	0	0	0
OR4X1	0	0	0	0	0	0	0	0	0	0	0	0
OR4X2	0	0	0	0	0	0	0	0	0	0	0	0
OR51A2	0	0	0	0	0	0	0	0	0	0	0	0
OR51A4	0	0	0	0	0	0	0	0	0	0	0	0
OR51A7	0	0	0	0	0	0	0	0	0	0	0	0
OR51B2	0	0	0	0	0	0	0	0	0	0	0	0
OR51B4	0	0	0	0	0	0	0	0	0.188290216	0	0	0
OR51B5	0	0	0	0	0	0	0	0	0	0	0	0
OR51B6	0	0	0	0	0	0	0	0	0	0	0	0
OR51D1	0	0	0	0	0	0	0	0	0	0	0	0
OR51E1	0	0	0	0	0	0	0	0	0	0	0	0
OR51E2	0	0	0	0	0	0	0	0	0	0	0	0
OR51F1	0	0	0	0	0	0	0	0	0	0	0	0
OR51F2	0	0	0	0	0	0	0	0	0	0	0	0
OR51G1	0	0	0	0	0	0	0	0	0	0	0	0
OR51G2	0	0	0	0	0	0	0	0	0	0	0	0
OR51H1	0	0	0	0	0	0	0	0	0	0	0	0
OR51I1	0	0	0	0	0	0	0	0	0	0	0	0
OR51I2	0	0	0	0	0	0	0	0	0	0	0	0
OR51J1	0	0	0	0	0	0	0	0	0	0	0	0
OR51L1	0	0	0	0	0	0	0	0	0	0	0	0
OR51M1	0	0	0	0	0	0	0	0	0	0	0	0
OR51Q1	0	0	0	0	0	0	0	0	0	0	0	0
OR51S1	0	0	0	0	0	0	0	0	0	0	0	0
OR51T1	0	0	0	0	0	0	0	0	0	0	0	0
OR51V1	0	0	0	0	0	0	0	0	0	0	0	0
OR52A1	0	0	0	0	0	0	0	0	0	0	0	0
OR52A5	0	0	0	0	0	0	0	0	0	0	0	0
OR52B2	0	0	0	0	0	0	0	0	0	0	0	0
OR52B4	0	0	0	0	0	0	0	0	0.052098093	0.052494995	0	0.049821084
OR52B6	0	0	0	0	0	0	0	0	0	0	0	0
OR52D1	0	0	0	0	0	0	0	0	0	0	0	0
OR52E1	0	0	0	0	0	0	0	0	0	0	0	0
OR52E2	0	0	0	0	0	0	0	0	0	0	0	0
OR52E4	0	0	0	0	0	0	0	0	0	0	0	0
OR52E5	0	0	0	0	0	0	0	0	0	0	0	0
OR52E6	0	0	0	0	0	0	0	0	0	0	0	0
OR52E8	0	0	0	0	0	0	0	0	0	0	0	0
OR52H1	0	0	0	0	0	0.114767308	0	0	0	0	0	0.116300932
OR52I1	0	0	0	0	0	0	0	0	0	0	0	0.057434768
OR52I2	0	0	0.051078728	0	0	0	0	0	0	0	0	0
OR52J3	0	0	0	0	0	0	0	0	0	0	0	0
OR52K1	0.156726876	0	0	0	0	0	0	0	0	0	0	0.054367863

OR52K2	0	0	0	0	0	0	0	0	0	0	0	0
OR52L1	0	0	0	0	0	0	0	0	0	0	0	0
OR52M1	0	0	0	0	0	0	0	0	0	0.061849449	0	0
OR52N1	0	0	0	0	0	0	0	0	0	0	0	0
OR52N2	0	0	0	0	0	0	0	0	0	0	0	0
OR52N4	0.097894895	0.135316244	0.069075538	0.065961393	0	0.100534491	0	0	0.035511375	0.035781913	0	0
OR52N5	0	0	0	0	0	0	0	0	0	0	0	0
OR52R1	0	0	0	0	0	0	0	0	0	0	0	0
OR52W1	0	0	0	0	0	0	0	0	0	0	0	0
OR52Z1	0	0	0	0	0	0	0	0	0	0	0	0
OR56A1	0	0	0	0	0	0	0	0	0	0	0	0
OR56A3	0	0	0	0	0	0	0	0	0	0	0	0
OR56A4	0	0	0	0	0	0	0	0	0	0	0	0
OR56A5	0	0	0	0	0	0	0.058742216	0	0	0	0	0
OR56B1	0	0	0	0	0	0.101302399	0	0	0	0	0	0
OR56B4	0	0	0	0	0	0	0	0	0	0	0	0
OR5A1	0	0	0	0	0	0	0	0	0	0	0	0
OR5A2	0	0	0	0	0	0	0	0	0	0	0	0
OR5AC2	0	0	0	0	0	0	0	0	0	0	0	0
OR5AK2	0	0	0	0	0	0	0	0	0	0	0	0
OR5AL1	0	0	0	0	0	0	0	0	0	0	0	0
OR5AN1	0	0	0	0	0	0	0	0	0	0	0	0
OR5AP2	0	0	0	0	0	0	0	0	0	0	0	0
OR5AR1	0	0	0	0	0	0	0	0	0	0	0	0
OR5AS1	0	0	0	0	0	0	0	0	0	0	0	0
OR5AU1	0	0	0	0	0	0	0	0	0	0	0	0
OR5B12	0	0	0	0	0	0	0	0	0	0	0	0
OR5B17	0	0	0	0	0	0	0	0	0	0	0	0
OR5B2	0	0.053178381	0	0	0	0	0	0	0	0	0	0
OR5B21	0.057859743	0.059982927	0	0	0	0	0	0	0	0	0	0
OR5B3	0	0	0	0	0	0	0	0	0	0	0	0
OR5C1	0	0	0	0	0	0	0	0	0	0	0	0
OR5D13	0	0	0	0	0	0	0	0	0	0	0	0
OR5D14	0	0	0	0	0	0	0	0	0	0	0	0
OR5D16	0	0	0	0	0	0	0	0	0	0	0	0
OR5D18	0	0	0	0	0	0	0	0	0	0	0	0
OR5F1	0	0	0	0	0	0	0	0	0	0	0	0
OR5H1	0	0	0	0	0	0	0	0	0	0	0	0
OR5H14	0	0	0	0	0	0	0	0	0	0	0	0
OR5H15	0	0	0	0	0	0	0	0	0	0	0	0
OR5H2	0	0	0	0	0	0	0	0	0	0	0	0
OR5H6	0	0	0	0	0	0	0	0	0	0	0	0
OR5I1	0	0	0	0	0	0	0	0	0	0	0	0
OR5J2	0	0	0	0	0	0	0	0	0	0	0	0
OR5K1	0	0	0	0	0	0	0	0	0	0	0	0
OR5K2	0	0	0	0	0	0	0	0	0	0	0	0
OR5K3	0	0	0	0	0	0	0	0	0	0	0	0
OR5K4	0	0	0	0	0	0	0	0	0	0	0	0
OR5L1	0	0	0	0	0	0	0	0	0	0	0	0
OR5L2	0	0	0	0	0	0	0	0	0	0	0	0
OR5M1	0	0	0	0	0	0	0	0	0	0	0	0
OR5M10	0	0	0	0	0	0	0	0	0	0	0	0
OR5M11	0	0	0	0	0	0	0	0	0	0	0	0
OR5M3	0	0	0	0	0	0	0	0	0	0	0	0
OR5M8	0	0	0	0	0	0	0	0	0	0	0	0
OR5M9	0	0	0	0	0	0	0	0	0	0	0	0
OR5P2	0	0	0	0	0	0	0	0	0	0	0	0
OR5P3	0	0	0	0	0	0	0	0	0	0	0	0
OR5R1	0	0	0	0	0	0	0	0	0	0	0	0
OR5T1	0	0	0	0	0	0	0	0	0	0	0	0
OR5T2	0	0	0	0	0	0	0	0	0	0	0	0
OR5T3	0	0	0	0	0	0	0	0	0	0	0	0
OR5V1	0	0	0	0	0	0	0	0	0	0	0	0
OR5W2	0	0	0	0	0	0	0	0	0	0	0	0
OR6A2	0	0.040306446	0.041150854	0.039295642	0.121792194	0.079856154	0	0	0	0	0.041879844	0
OR6B1	0	0	0	0	0	0	0	0	0	0	0	0
OR6B2	0	0	0	0	0	0	0	0	0	0	0	0
OR6B3	0	0	0	0	0	0	0	0	0	0	0	0
OR6C1	0	0	0	0	0	0	0	0	0	0	0	0
OR6C2	0	0	0	0	0	0	0	0	0	0	0	0
OR6C3	0	0	0	0	0	0	0	0	0	0	0	0
OR6C4	0	0	0	0	0	0	0	0	0	0	0	0
OR6C6	0	0	0	0	0	0	0	0	0	0	0	0
OR6C65	0	0	0	0	0	0	0	0	0	0	0	0
OR6C68	0	0	0	0	0	0	0	0	0	0	0	0
OR6C70	0	0	0	0	0	0	0	0	0	0	0	0
OR6C74	0	0	0	0	0	0	0	0	0	0	0	0
OR6C75	0	0	0	0	0	0	0	0	0	0	0	0
OR6C76	0	0	0	0	0	0	0	0	0	0	0	0
OR6F1	0	0	0	0	0	0	0	0	0	0.063650889	0	0
OR6J1	0	0	0	0	0	0	0	0	0	0	0	0
OR6K2	0	0	0	0	0	0	0	0	0	0	0	0
OR6K3	0	0	0	0	0	0	0	0	0	0	0	0
OR6K6	0	0	0	0	0	0	0	0	0	0	0	0
OR6M1	0	0	0	0	0	0	0	0	0	0	0	0
OR6N1	0	0	0	0	0	0	0	0	0	0	0	0
OR6N2	0	0	0	0	0	0	0	0	0	0	0	0
OR6P1	0	0	0	0	0	0	0	0	0	0	0	0
OR6Q1	0	0	0	0	0	0	0	0	0	0	0	0
OR6S1	0	0	0	0	0	0	0	0	0	0	0	0
OR6T1	0	0	0	0	0	0	0	0	0	0	0	0
OR6V1	0	0	0	0	0	0	0	0	0	0	0	0
OR6X1	0	0	0	0	0	0	0	0	0	0	0	0
OR6Y1	0	0	0	0	0	0	0	0	0	0	0	0
OR7A10	0	0	0	0	0	0	0	0	0	0	0	0
OR7A17	0	0	0	0	0	0	0	0	0	0	0	0
OR7A5	0	0	0	0	0	0	0	0	0	0	0	0
OR7C1	0	0	0	0	0	0	0	0	0	0	0	0
OR7C2	0	0	0	0	0	0	0	0	0	0	0	0
OR7D2	0	0.034605535	0	0.050606546	0.052283001	0.068561363	0	0.04899466	0.072652924	0.054904815	0.017978196	0.052108156
OR7D4	0	0	0	0	0	0	0	0	0	0	0	0
OR7E24	0	0	0	0	0	0	0	0	0	0	0	0
OR7G1	0	0	0	0	0	0	0	0	0	0	0	0
OR7G2	0	0	0	0	0	0	0	0	0	0	0	0
OR7G3	0	0	0	0	0	0	0	0	0	0	0	0
OR8A1	0	0	0	0	0	0	0	0	0	0	0	0
OR8B12	0	0	0	0	0	0	0	0	0	0	0	0
OR8B2	0	0	0	0	0	0	0	0	0	0	0	0
OR8B3	0	0	0	0	0	0	0	0	0	0	0	0
OR8B4	0	0	0	0	0	0	0	0	0	0	0	0
OR8B8	0	0	0	0	0	0	0	0	0	0	0	0
OR8D1	0	0	0	0	0	0	0	0	0	0	0	0
OR8D2	0	0	0	0	0	0	0	0	0	0	0	0
OR8D4	0	0	0	0	0	0	0	0	0	0	0	0
OR8G1	0	0	0	0	0	0	0	0	0	0	0	0
OR8G2P	0	0	0	0	0	0	0	0	0	0	0	0
OR8G5	0	0	0	0	0	0	0	0	0	0	0	0
OR8H1	0	0	0	0	0	0	0	0	0	0	0	0
OR8H2	0	0	0	0	0	0	0	0	0	0	0	0
OR8H3	0	0	0	0	0	0	0	0	0	0	0	0
OR8I2	0	0	0	0	0	0	0	0	0	0	0	0
OR8J1	0	0	0	0	0	0	0	0	0	0	0	0
OR8J3	0	0	0	0	0	0	0	0	0	0	0	0
OR8K1	0	0	0	0	0	0	0	0	0	0	0	0
OR8K3	0	0	0	0	0	0	0	0	0	0	0	0
OR8K5	0	0	0	0	0	0	0	0	0	0	0	0
OR8S1	0	0	0	0	0	0	0	0	0.108441217	0.05463368	0	0
OR8U1	0	0	0	0	0	0	0	0	0	0	0	0
OR9A2	0.057673699	0.059790055	0	0	0.060221649	0	0	0	0	0	0.124248025	0
OR9A4	0	0	0.060267493	0	0	0	0	0	0	0	0	0
OR9G1	0	0	0	0	0	0	0	0	0	0	0	0
OR9G4	0	0	0	0	0	0	0	0	0	0	0	0
OR9I1	0	0	0	0	0	0	0	0	0	0	0	0
OR9K2	0	0	0	0	0	0	0	0	0	0	0	0
OR9Q1	0	0	0	0	0	0	0	0	0	0	0	0
OR9Q2	0	0	0	0	0	0	0	0	0	0	0	0
ORAI1	23.58431053	22.66346238	23.74615631	26.04973386	20.20325848	22.15579426	21.59511221	27.74904161	4.570591112	5.589473754	5.684036316	8.181293426
ORAI2	0.826231348	0.856550199	0.605824609	0.487962386	0.509324416	0.751390936	0.946906489	0.832823119	3.9649102	3.776803908	3.994216005	4.604848864

ORAI3	14.60826287	18.3057584	12.70244848	13.24830661	8.808076751	10.15297273	12.3163742	12.99478121	22.40094203	20.25241551	17.85474784	26.2334878
ORAOV1	5.938028901	5.690530427	6.414477075	5.960300995	6.690426534	6.559167058	7.428384311	6.828707428	5.307327811	4.475113716	5.165339537	6.009741513
ORC1	14.81072159	17.44102102	17.16551286	25.90242519	28.93073299	28.67224523	17.9738319	25.8620679	28.05026	30.30972879	33.40554393	29.83882931
ORC2	12.89889294	13.1061085	12.20901901	14.49622562	16.16584993	16.47598645	14.51846981	13.32806683	7.350335785	9.482217583	6.75701435	7.463180614
ORC3	9.449557611	10.47029876	10.60963585	15.15877697	12.25090074	14.00531993	10.85247181	13.24090218	21.7351665	23.0944348	23.90777774	19.55791831
ORC4	4.34893017	4.383928045	4.388321507	4.266396532	4.878306637	4.489333736	4.302301579	4.365695062	4.160546182	4.332258641	3.883531233	4.142855442
ORC5	4.736425462	4.310401001	5.554284748	4.596695921	6.519298466	5.541246283	5.839320041	5.661605196	6.15015453	5.562552921	6.667262736	6.091403099
ORC6	13.25523004	15.50257722	16.95099692	28.54148363	22.70909505	24.07910624	21.08600535	26.80134965	23.00738736	26.60851154	26.49771233	28.59921215
ORM1	0	0	0	0	0	0.131729341	0.065953716	0	0.209385901	0.070327025	0.276337085	0.066744814
ORM2	0	0	0	0	0	0	0	0	0	0	0.068675004	0
ORMDL1	16.47390792	14.7149642	15.60169053	15.3717801	15.0331481	14.52872184	15.35125377	14.46962366	17.53688952	14.75965487	14.11197602	26.43941045
ORMDL2	9.801891521	9.074328642	9.605978856	11.37440932	9.181950601	8.74059727	9.955352542	12.47250776	10.05235447	6.413518951	10.34099361	10.24267715
ORMDL3	7.535041355	8.385271458	8.320633133	9.480115096	7.334510897	7.213588341	7.500600287	8.831029118	8.09191105	7.857913778	7.18407192	11.28247856
OS9	101.6214802	103.869177	98.30424166	113.4069484	85.10474216	89.01143047	95.53146907	119.1221911	98.78381031	87.76436813	84.67431272	118.356862
OSBP	16.4191676	15.88031165	15.75361214	17.58985189	17.82988514	16.62271122	16.7105022	16.81206033	20.82890592	23.19314184	19.53342482	24.06090794
OSBP2	0.01029454	0.010672302	0.021791766	0	0.064496038	0.010572118	0	0	0.42571528	0.846628673	0.565534128	0.439249063
OSBPL10	0.00761097	0	0	0	0.007947213	0	0.007826756	0	0.198783334	0.200297735	0.229551302	0.253460362
OSBPL11	5.800944465	4.113447525	6.053842428	4.831110277	4.870007126	3.753135931	5.201839844	4.370715266	5.820473589	4.847060506	5.386264398	3.622179744
OSBPL1A	8.954554193	9.415761074	8.520190241	8.764055867	8.424680454	9.4587438	7.479503079	6.920252424	10.04310403	10.89111138	9.438830669	13.36974898
OSBPL2	4.169546042	4.13663292	4.175841322	4.268524314	3.099121871	3.425577502	3.882037273	3.843023264	6.830658214	6.784372321	6.055988701	6.672090096
OSBPL3	4.296777743	3.503758056	3.725882889	3.901739693	4.610159261	3.911371105	3.749345487	3.813646678	2.38224905	2.473382927	2.413743294	3.263267332
OSBPL5	6.063186185	7.012111517	6.242247327	7.623169771	5.995697448	6.666436221	5.60457479	7.713668284	16.69159217	13.10496866	15.36839529	19.12206927
OSBPL6	0.048060342	0	0.031792331	0	0	0.006169528	0.006177868	0	0.653770873	0.632401467	0.640639574	0.975307382
OSBPL7	7.896527335	8.132962293	8.344181391	7.967999128	7.332216074	7.779256715	8.14686028	8.695787159	6.689972963	7.714004008	4.973291495	9.997891356
OSBPL8	37.89530566	30.52140541	33.50676165	30.66486322	34.37201121	29.77964455	32.82208381	21.40942302	25.71270532	22.70040011	22.6335098	24.20665593
OSBPL9	19.3403143	20.59575399	18.4957246	19.47780177	17.07591137	19.57973713	17.6173429	17.69281726	36.489939	40.06826187	38.32474196	33.73987248
OSCAR	3.62555936	5.70550154	3.80973525	1.55536837	1.82477727	2.491140401	3.40647907	1.684485346	4.229365149	3.775364718	5.00105829	4.424537329
OSCP1	0	0.157493285	0.032158544	0.092126203	0	0.093608908	0.249961233	0.118922481	0.099195241	0.266535853	0.392738816	0.158099658
OSER1	4.884851576	5.880327492	5.474850437	6.092234116	6.152613569	6.520664557	5.049464659	5.981028044	6.412294984	6.516845625	6.03691753	6.863301163
OSGEP	31.25921566	32.88688528	29.33506335	35.24158925	29.63205319	35.53672566	28.74023489	39.49779669	28.9007521	31.66265503	30.35409835	36.25282559
OSGEPL1	2.908990592	3.113923816	3.207800606	3.938377812	5.128440509	4.988308933	3.90977676	3.892371367	3.651608688	4.866340152	3.891318802	4.33685211
OSGIN1	0.443790193	0.345056423	0.499070764	0.616739025	0.231698139	0.398786814	0.484895801	0.352828901	1.297940749	0.760365649	1.314595341	2.164905883
OSGIN2	4.228460566	4.011471632	4.214797406	4.421352625	4.519875121	4.115277343	4.700334251	3.941515463	7.118205633	6.389737537	5.975572428	7.593365539
OSM	4.473539306	3.583675272	4.438927215	3.4681142	1.353578994	1.409572396	3.18889489	2.437405279	0.221287698	0.585305556	0.383308385	0.370328098
OSMR	0.030530247	0.015825283	0.032313635	0.007714208	0.031879035	0.031353452	0.05494272	0.014937001	0.980124019	0.334776592	0.781044234	0.698993347
OSR1	0	0	0.018765332	0	0	0	0	0	0	0.01944131	0	0
OSR2	4.947197099	4.22938439	6.799591319	3.838953078	3.525446189	2.793121217	2.676341453	2.684266934	1.658512727	1.799697693	1.439571725	2.07403329
OST4	160.5447473	174.4542451	172.9633748	164.3839182	129.6795927	140.250363	165.4373808	173.9600856	138.2792518	172.2879267	158.8888591	132.2355927
OSTC	70.71846801	76.8968622	71.83367295	78.27309856	79.74428451	73.29685824	78.29541983	74.82017679	53.37341139	67.25064542	55.84851655	52.64479813
OSTF1	33.32772506	31.00492204	35.73755457	33.68423598	26.61917067	26.95632125	36.03125122	31.91086565	52.15277144	45.26721402	50.63616683	59.55832626
OSTM1	14.68409552	11.90111215	12.99191496	15.1211953	8.930518699	9.253374302	13.00692328	12.56503729	16.28147644	13.57879938	13.02743463	22.82829523
OSTN	0	0	0	0	0	0	0	0	0	0	0	0
OTC	0	0	0	0	0	0	0	0	0	0	0	0.032633391
OTOA	0	0	0	0	0	0	0	0	0	0	0	0.032633391
OTOF	0.021107307	0	0	0	0	0	0.021705741	0	0.084223435	0.030860028	0.045472048	0.007322032
OTOG	0	0	0	0	0	0	0	0	0	0	0	0
OTOGL	0	0	0	0	0	0	0	0	0.006385851	0.006434501	0.006320796	0.01832025
OTOL1	0	0	0	0	0	0	0	0	0	0	0	0
OTOP1	0	0.028666044	0	0	0	0	0	0	0	0	0	0
OTOP2	0	0	0	0	0	0	0	0	0.024739441	0	0	0
OTOP3	0	0	0	0	0	0	0	0	0	0	0	0
OTOR	0	0	0	0	0	0	0	0.0315287	0.035064825	0	0	0
OTOS	0	0	0	0	0	0	0	0	0	0	0	0
OTP	0.040186379	0.062491548	0.085067635	0.020308129	0.062942642	0.020634973	0.061988612	0.058983863	0.021866414	0.044066	0.12986192	0.041821433
OTUB1	23.83195895	26.38438188	27.95502692	27.61932058	25.59511969	25.07678176	25.66548327	30.13657144	29.840716	27.67598378	28.65238533	30.02556476
OTUB2	0.318265252	0.459565008	0.384978665	0.172323094	0.166162903	0.17509651	0.350666463	0.100100624	0.828771277	1.63277842	1.10193353	1.052788761
OTUD1	1.019840697	1.308140342	0.987937742	1.100631424	0.992699622	0.869824115	0.924326608	1.082488729	6.771915369	6.14115557	5.567153676	6.31404224
OTUD3	2.891460713	3.369354754	3.258059691	4.470427633	3.877372802	4.327533828	3.188571879	4.013670876	5.862330372	6.816389816	5.488736725	7.511099178
OTUD4	14.94475927	11.80916802	14.52437153	12.44995898	14.27769472	12.4628129	13.47630165	9.730689964	15.1248748	12.51397916	13.93441114	13.90105789
OTUD5	12.93169505	13.80253869	12.41743639	11.96420417	11.35573466	11.24974059	12.20030649	12.4215507	16.74117736	15.98697649	17.12440338	17.66942227
OTUD6A	0	0	0	0	0	0	0	0	0	0	0	0
OTUD6B	21.20766578	15.59294385	20.75678737	18.6820489	20.95156365	18.42015286	20.40866576	15.94290238	13.60908883	13.8157421	12.93095599	14.64310938
OTUD7A	0.018935361	0.01472265	0.025051808	0.023922393	0.024714876	0.034030369	0.004868054	0.018528346	0.046364416	0.025954242	0.005099121	0.049264449
OTUD7B	3.262780218	2.902411026	3.119174331	4.691219433	3.632222386	3.1724097	3.063051836	3.584003727	5.051186575	3.906695495	4.098416797	5.202831677
OTULIN	6.115188946	5.924981037	5.654757793	7.203219193	6.59236546	6.59952116	5.627722257	6.161014736	11.50557039	11.17717217	10.5083663	12.22641439
OTULINL	6.892203327	11.43376679	7.238254191	9.912757681	4.327578958	8.206765974	6.130037747	8.55890154	44.72854103	38.114315	40.81161659	41.67747833
OTX1	1.567622708	1.96529426	1.331213384	1.713353889	1.370409723	1.740929081	1.443363119	1.854981215	44.29559215	35.19874771	36.55985509	56.70078205
OTX2	0	0	0	0	0	0	0	0	0	0.023157133	0	0
OVCA2	0.700124398	1.555319378	1.323252354	0.606526044	0.522182143	1.078503374	0.874254511	1.12548081	0.27211086	0.219347117	0.37707428	0.41634869
OVCH1	0.029693885	0.021988223	0.049387512	0.025724163	0.013288167	0.008712725	0.02181126	0.008301605	0.161571856	0.218620859	0.155356557	0.167753895
OVCH2	0	0	0	0	0	0	0	0	0	0	0	0
OVGP1	2.472071708	2.488141021	1.676576078	2.42574347	2.330674519	2.267601333	2.418753993	2.395449906	1.201462901	1.710653137	1.111665146	2.647583977
OVOL1	0.068397844	0.060778052	0.020683777	0.019751287	0.030608389	0.010034585	0.060288906	0.038244364	0.138234491	0.192859767	0.084200768	0.244048224
OVOL2	0.202618034	0.270068368	0.398271198	0.438825997	0.302242058	0.356710868	0.386959216	0.254909282	1.070995557	1.460032932	1.278337735	1.295294594
OVOL3	0	0.069383236	0	0.135286489	0.069884078	0.068731914	0	0.130977434	0	0	0.072091671	0.069650372
OXA1L	130.6893058	129.6761782	132.5253783	123.4185821	130.9717872	133.0237217	132.0769016	131.4638559	85.87863063	95.45733861	85.81299349	88.28215
OXCT1	41.68048053	42.6768913	49.74964707	51.05296884	56.81846584	55.78852366	50.1717964	49.35287127	49.30875159	50.44724755	53.79132813	39.17404699
OXCT2	0.590017115	0.2140838	0.343465238	0.685777894	0.554474986	0.424148257	0.424721683	0.230933896	2.985700607	5.046426753	4.131042476	3.592034629
OXER1	0.946169249	0.854322907	0.419958115	0.401025066	0.478049903	0.470168394	0.784673396	0.866100352	6.576593816	5.187565527	5.950691073	6.543263279
OXGR1	0	0	0	0	0	0	0	0	0	0	0	0
OXLD1	8.942340317	9.243611688	9.162923389	8.645041251	8.25480423	9.662594634	8.209649184	8.293597092	9.393015222	9.066664459	9.269405402	13.05561996
OXNAD1	0.974503858	1.026446334	1.114036999	1.138189397	1.187536979	1.090094417	0.997546541	1.012472389	0.965859358	1.039239913	1.052102205	1.151075583
OXR1	15.67773477	15.99775151	13.83276875	14.24153991	16.90365552	17.1120098	14.57472049	11.99415859	12.94236732	15.6711618	12.27019794	13.17397824
OXSM	2.812346386	2.89394979	3.252239738	2.421329612	2.784324516	2.952358994	2.399356873	2.792664026	3.445936924	2.901105514	2.737641443	3.295328155
OXSR1	15.92904244	16.99148147	15.74870019	16.08952399	18.22025424	17.46657586	15.7855519	15.40429274	15.23197828	16.5956525	15.93260101	14.12731972
OXT	0	0	0	0	0	0	0	0	0	0	0	0
OXTR	0.317010287	0.377330956	0.223685373	0.723869798	0.50265301	0.699346846	0.325998152	0.769746061	0.30665463	0.180244652	0.265589303	0.391002567
P2RX1	36.13508545	34.22924948	30.48480108	22.0143491	28.53213754	26.36355378	25.98104608	23.78002531	8.466609459	8.664874117	9.402351929	7.602873167
P2RX2	0	0	0	0.019776425	0	0	0.020121879	0.019146519	0.021293912	0	0	0
P2RX3	0	0	0.010465414	0.019987199	0	0	0.010168168	0	0	0	0	0
P2RX4	22.00593313	25.98422239	20.65772848	21.01006995	17.45688108	17.96763465	20.7373606	21.63436795	7.108227736	7.56029074	8.013010092	8.577866007
P2RX5	3.985030276	4.10710294	4.094483195	2.237588141	5.085769127	5.193382204	5.583843272	3.762552262	0.456495724	0.689960202	0.727972891	0.266776911
P2RX6	0.026625216	0.041403356	0.028180495	0	0.013900742	0.027343126	0.027380093	0.026052909	0.101412123	0.116782532	0.100379002	0.124688295
P2RX7	12.6664249	13.24650194	9.618739506	6.324332024	8.750489586	8.647747884	8.867348901	6.182243134	0.363032591	0.443391879	0.479112335	0.11572194
P2RY1	0.087728645	0.08267989	0.050647204	0.056424512	0.066621371	0.040951874	0.07381303	0.078039022	3.489020207	2.92966732	3.09266538	2.166253534
P2RY10	0	0	0	0	0	0	0	0.014405726	0	0.048430403	0	0
P2RY11	0	0	0	0	0	0	0	0	0	0	0	0
P2RY12	0	0.02326277	0	0	0	0	0	0	0	0.024605661	0	0
P2RY13	0.038865699	0.020145945	0.020567996	0	0	0.079827315	0.019983809	0.038030284	0	0.021308911	0.02093236	0
P2RY14	0.009311224	0	0	0.009410827	0	0.009562287	0.009575215	0	0.010132939	0	0	0.009690067
P2RY2	13.36434955	15.39366327	13.1919457	16.0844215	8.327245692	15.4688998	9.96592287	15.69424539	11.01696643	10.20637343	11.42812151	12.24265546
P2RY4	0.065661453	0.136141847	0.104245481	0.099545763	0.27424917	0.236011721	0	0.064250065	0.107184119	0.108000685	0	0.239165523
P2RY6	2.905500493	3.547429108	2.797880835	3.372784017	1.722139059	2.825549085	1.989276302	2.564021144	2.440626297	2.924020843	2.689715266	2.694876827
P2RY8	0.798692136	1.530837996	1.435123588	1.154534991	0.950346266	1.611842854	0.792685353	1.644836042	2.172941883	1.435902103	1.740651784	2.464570108
P3H1	15.23767583	18.13025923	15.92209866	19.50861299	15.70795544	16.46339271	15.96943304	19.7428675	138.6683493	159.7894567	138.0057175	169.6313318
P3H2	0.250829651	0.711671818	0.293426989	0.026685559	0.234341407	0.366053087	0.190061911	0.025835588	0.64649695	0.65142219	0.412386999	0.302251171
P3H3	0.040276617	0.041754582	0.042629327	0	0.021027993	0.062043928	0	0.039410874	0.679380976	2.009505264	1.258150753	1.613740722
P3H4	0.331645984	0.309434265	0.386120551	0.553069511	0.242408376	0.35761776	0.392206121	0.551679372	2.905355743	3.254786751	2.87575787	3.5549378
P4HA1	29.76926949	52.47222857	30.39370651	24.7484084	46.44029923	43.64018303	22.50113294	25.37469481	43.62250261	92.72395544	60.51855493	22.51100013
P4HA2	0.029838943	0.041245192	0.010527316	0.030158134	0	0.010214503	0.010228312	0.048662595	2.835908198	7.045623998	3.589125827	3.902326222
P4HA3	0	0	0	0	0.048879338	0	0	0	0.127355931	0.102660938	0.176481916	0.194863502
P4HB	306.9828785	343.0910944	323.8874655	381.5618797	209.5626476	232.3988652	273.0546841	375.3127551	368.1278191	476.8563639	399.8475928	507.5952539
P4HTM	5.303176169	5.742123784	4.939400218	6.050737111	5.414408979	5.155706384	5.502007494	7.149543487	11.95275856	13.25853878	10.40924157	17.31722408
PA2G4	126.9626958	126.3746154	144.3338765	150.9778671	173.4387623	182.5952014	163.2661389	172.6558895	89.8220333	110.3017798	110.1184081	87.98608401
PAAF1	8.002987525	8.296659978	8.136111615	8.258882746	8.048676438	8.716228885	8.143257582	7.624885902	9.557257611	12.28584233	9.596008081	11.33128403
PABPC1	1290.456886	1289.956916	1259.770976	1232.163385	1192.909987	1205.742102	1246.743454	1235.175108	543.4702891	692.691256	627.9285296	506.8124185
PABPC1L	8.424345918	6.792706873	8.889667792	4.909239479	12.38909664	8.937281498	9.495691714	8.812619879	1.872102247	1.858623922	1.553275556	2.922368487
PABPC1L2A	0	0	0	0	0	0	0	0	0	0	0	0
PABPC1L2B	0	0	0	0	0	0	0	0	0	0	0	0
PABPC3	1.616501211	1.423298578	1.195305287	1.521889492	0.878641295	1.569124132	1.366300441	1.49508315	0.554255108	0.582759253	0.524756165	0.322627401
PABPC4	96.21565985	92.10299743	98.67193789	96.09064901	86.19167143	90.23805219	94.3668406	100.7209106	193.9331041	223.2184836	224.8458223	230.9342854
PABPC4L	0	0	0	0	0	0	0	0	1.78052712	1.805895034	1.658036332	1.422656562
PABPC5	0	0	0	0	0	0	0	0	0	0	0	0
PABPN1	5.773111594	6.211415832	5.185532572	6.150265538	5.680590699	5.982187666	5.241490868	6.534540882	6.74670814	7.083262415	6.050516137	8.930812206
PABPN1L	0.295386427	0.272200655	0.434223706	0.21561671	0.257030186	0.219086905	0.236258722	0.256921883	0.125010003	0.071978499	0.123736482	0.153702376
PACRG	0	0	0	0	0	0	0	0	0.028992312	0.008346624	0.018448044	0.055450337
PACRGL	2.103975186	2.049982423	2.302221793	2.598144906	2.279517334	2.672451987	2.505252346	2.089709047	2.341297357	2.471886916	2.325965765	2.881025223
PACS1	5.407400371	6.636240202	5.095927559	6.829250963	5.008347073	5.309723777	4.876171377	6.290512535	6.152536753	7.479286903	6.875646935	7.468417791
PACS2	6.804485351	6.71750165	6.735470472	6.025429658	5.215788463	6.027114248	5.430941178	6.991134613	9.146835095	9.691334945	8.329027685	11.99803968
PACSIN1	0	0	0	0	0	0	0	0	0.06271859	0.115860071	0.072426263	0.039984933
PACSIN2	18.04991592	16.62736093	16.39325129	18.23288564	16.15187629	15.73145446	17.2240742	17.14304156	22.38671932	20.83475398	22.90230946	22.26211337
PACSIN3	2.390976745	3.444108543	3.036771314	1.551681611	4.152251723	4.187181613	4.244605955	2.462718818	1.807691752	3.09096816	2.602583513	1.912029755
PADI1	0.011119975	0	0	0	0	0	0	0	0.012101314	0.036580517	0.011978033	0.011572411
PADI2	0.691519079	1.190774107	0.707102708	0.959529222	0.538492516	0.722201593	0.735230939	0.722529838	0.229590204	0.719722275	0.416627362	0.317135999
PADI3	0.031357553	0.021672153	0.022126178	0.031692989	0.010914297	0.02146871	0.042995468	0.010227842	0	0	0	0.010877797
PADI4	0.035706411	0.11105001	0.037792157	0.05413255	0	0.055003774	0.055078137	0.069877808	0.019428752	0	0	0
PADI6	0	0	0	0	0	0	0	0	0.024440007	0.049252399	0.024191028	0
PAEP	0	0	0	0	0	0	0	0	0	0	0	0

PAF1	31.19497041	31.85227045	34.70910001	36.77948492	36.27963669	35.15038343	35.89896212	40.89860488	23.15212872	20.5961097	23.52399419	22.48273833
PAFAH1B1	24.72554198	22.59984074	23.72437063	27.70436443	25.01107598	22.58799171	26.02828056	22.82140724	16.73559556	13.23547168	14.45608363	16.3788777
PAFAH1B2	13.94178507	13.53767283	14.3350538	11.85503379	13.46090118	11.99679875	14.32069562	11.936844	14.69585363	15.04341044	14.76900553	15.90858987
PAFAH1B3	7.119284432	7.063573851	7.304009291	6.665714658	11.53836043	12.87317501	9.521960706	9.530521929	9.411148457	8.046051047	11.10305362	5.818067412
PAFAH2	3.052613138	3.104465789	3.415201295	3.343350529	2.920840912	2.991884189	3.234648511	2.93021042	3.536736837	3.220040274	2.96309831	3.96195833
PAG1	10.06066924	9.286292429	8.923140989	7.993280934	8.114099775	6.8222164	8.203804518	5.502850201	19.90884277	16.63159815	20.25238259	15.7895678
PAGE1	0	0	0	0	0	0	0	0	0	0	0	0
PAGE2	0	0	0	0	0	0	0	0	0	0	0	0
PAGE2B	0.042436562	0.043993787	0.089830885	0.128671535	0	0.043580803	0.043639722	0.083048783	0	0.232667091	0.137133368	0.220815847
PAGE3	0	0	0	0	0	0	0	0	0	0	0	0
PAGE4	0	0	0	0	0	0	0	0	0	0	0	0
PAGE5	0	0	0	0	0	0.073582501	0	0	0	0	0	0
PAGR1	9.115972664	10.32553155	9.70802965	13.02400377	9.944696124	11.83223936	9.128494361	14.10960824	9.063134165	8.299177359	10.18307578	12.51740092
PAH	0	0	0	0	0	0	0	0	0	0	0	0
PAICS	92.37384404	90.31035559	105.2631116	95.2629097	143.3330183	133.2438167	115.1833205	101.8046389	66.91183622	80.98639616	95.81222796	46.86466527
PAIP1	18.80589244	18.32590619	20.35642212	21.68932319	21.78671784	21.97574359	21.73881595	21.86409864	38.45790295	40.23937774	36.8339398	43.61818991
PAIP2	42.4071909	47.45584209	45.76597768	43.59045161	45.47902896	42.79026221	52.9177044	44.42987037	63.51365137	61.29797404	59.97550898	57.71334741
PAIP2B	0.008254266	0	0.008736429	0	0	0.033907322	0.008488291	0	0.305411987	0.64263086	0.382321408	0.592717291
PAK1	21.39057936	24.06252961	23.66002124	27.28276532	20.00016489	22.63931676	21.49544328	25.36606916	30.77448088	33.94414814	29.79459673	36.98180651
PAK1IP1	27.81749956	25.70306367	31.60518902	35.16448623	35.84575508	35.95427304	36.61927312	36.23054663	7.1752396	9.02244104	9.039091001	9.413485779
PAK2	42.64956341	39.73494347	41.06254118	42.76190778	42.22447687	41.475918	42.56416802	38.24536921	65.50082627	58.49137394	64.69610963	56.22905555
PAK3	0	0	0.004571583	0	0	0	0	0	0.004700454	0.004736264	0.013957707	0.004495015
PAK4	5.12760969	5.048407969	5.330793175	6.62373635	5.781838596	5.733253102	4.851772543	7.897197031	10.43383661	10.62977029	11.50409906	11.50921828
PAK5	0	0	0	0	0	0.008681926	0	0	0.082800363	0.101971425	0.081956847	0.131969125
PAK6	0	0	0	0	0	0	0	0	0	0	0.012480987	0
PALB2	6.25469549	6.086544763	7.262889338	6.967763185	7.977412796	8.031916156	7.440114651	6.912651778	4.278811259	4.381512947	5.141949156	5.899289937
PALD1	0.053320787	0.027638706	0.084653184	0	0.01855881	0.009126418	0.063971292	0.008695777	7.011517154	6.753101804	8.433403951	7.842620349
PALLD	0.027895055	0.005783735	0.029524511	0.028193452	0.023301938	0.028647205	0.011474374	0.016377272	0.048570872	0.085646578	0.102161634	0.081284042
PALM	0.938368364	1.504803283	1.024218955	0.429746564	1.163541334	1.189530307	0.919739848	0.588220725	3.39861274	5.803972518	4.92753448	3.295847997
PALM2	0	0	0	0	0	0	0	0	0.02450649	0.030866486	0.012128417	0.011717702
PALM2-AKAP:	0	0	0	0	0	0	0	0	0	0.01569474	0	0
PALM3	0	0.022658051	0	0	0	0	0	0	0.142708994	0.239660334	0.188340223	0.159217015
PALMD	0	0	0	0	0	0	0	0	0	0	0	0
PAM	8.531527203	9.322924723	7.537190601	5.710398709	6.408622073	5.9989917	6.392057898	5.562588933	10.54446531	14.20458001	10.40891297	9.938649414
PAM16	0.790026179	1.274025618	1.021991182	1.419515004	0.274976176	0.811328103	0.812424977	0.858938472	0.764218689	0.866295871	0.094554166	0.913522004
PAMR1	0	0	0	0	0	0	0	0	0	0	0	0
PAN2	13.27831184	14.80825804	10.89104229	13.5767432	17.56089914	17.59912519	11.70551628	14.55431412	15.6604397	20.15947441	13.1361805	17.52230102
PAN3	11.82098703	9.310796383	11.18880775	7.671719518	16.46609325	12.25957218	11.48607271	7.108573551	10.55699646	8.525934808	8.432888387	8.683725863
PANK1	3.280041832	3.13252663	3.175826398	3.144575605	3.358873693	3.086873911	3.232042342	2.827695484	2.691476143	2.232035329	2.368681931	2.601281659
PANK2	3.053987373	2.968176111	3.052805556	3.433905096	3.100327845	2.800920295	3.258345452	3.050599268	1.961789315	1.646503903	2.170251398	2.004059599
PANK3	22.49611402	19.02292901	18.79929421	16.94016914	18.36397581	18.42015286	16.27216628	9.171900712	23.99730168	36.32244479	27.32944811	18.03802735
PANK4	3.711829966	4.555579142	4.068055807	5.639405556	4.851018662	5.619499236	4.235713785	5.694108404	4.730006078	5.88205601	5.558854979	5.744435328
PANO1	0.835252885	0.965814644	0.71403487	0.844187692	0.402532289	0.560852415	0.693754344	0.660126266	0.594322609	0.352264921	0.415248026	0.735507925
PANX1	5.965331645	6.545538605	6.49393816	7.896867379	6.211946106	6.359239395	5.934423788	6.780878704	7.073915072	5.714687861	6.19864582	6.99248187
PANX2	0.01303842	0	0.013800044	0.013177894	0	0	0.053632341	0	0.397293724	0.228754541	0.365157329	0.244240412
PANX3	0	0	0	0	0	0	0	0	0.036598911	0	0	0
PAOX	0.347719295	0.600798295	0.613384828	0.234292595	0.211797302	0.446368809	0.208587063	0.198476305	1.545155948	2.478374367	1.779115298	1.477623071
PAPD4	14.97538236	15.43694948	15.76034866	14.42381797	14.47638426	15.67542809	16.08925411	13.27531258	15.59522172	18.23534748	15.54601986	14.99308266
PAPD5	2.648259415	2.168498311	2.119141767	2.747705261	2.538157808	2.404342348	2.973311446	2.459890731	3.243954802	3.072267709	2.880170248	2.942405288
PAPD7	6.213575157	6.033617474	6.086947245	6.887113345	7.663146901	7.295741835	5.559075757	6.640727296	10.4434151	12.9303915	10.95427117	10.56176306
PAPLN	0.84998984	1.043029999	0.697680751	0.578565623	1.593951734	1.897240115	1.105989816	1.069353477	6.503326743	6.086847128	4.437751328	7.663292249
PAPOLA	23.53729508	21.9943832	24.62488265	23.19304876	29.09280788	25.96957968	28.6325923	22.34440613	29.00663425	29.54581048	29.83537986	21.32897086
PAPOLB	0	0	0	0	0	0	0	0	0	0.013860553	0.013615622	0.02630909
PAPOLG	3.912230461	3.617735485	3.462198593	4.042439421	3.894887747	3.493993251	4.165495984	3.67843366	6.770748921	5.663972571	5.454018961	6.64162407
PAPPA	0.00977467	0.015200033	0.010345646	0	0	0	0	0	0	0	0	0
PAPPA2	0.008971251	0.013950681	0.004747648	0.004533609	0.018735178	0.009213147	0.009225603	0.004389207	0.087866674	0.004918671	0.048317526	0.135375797
PAPSS1	21.24513701	18.0867993	21.55679945	19.50457803	21.8847481	17.42694135	20.53231132	18.62184091	18.2176628	20.65885686	17.02457986	28.04911144
PAPSS2	0.013947527	0	0.029524511	0	0	0	0	0	3.855312933	3.548215357	4.176607988	2.235311151
PAQR3	5.107247299	4.955390095	5.290121964	7.106355425	6.078446516	6.151366932	6.414637043	5.938737953	10.77057511	11.85307188	9.731498716	11.92014894
PAQR4	8.130195017	11.54429729	10.5218887	15.1102366	15.12798875	15.45235164	10.71834079	18.41815645	18.28242039	16.30912173	21.33355938	25.30276059
PAQR5	0.031836209	0.01980267	0.02021753	0.019306059	0	0	0.006547766	0	0.006929151	0.01396388	0	0
PAQR6	7.393540071	7.778965487	6.271990577	6.545504438	4.655094465	4.389937554	3.226155372	5.15219585	13.77606457	17.76367629	12.03500768	18.65357114
PAQR7	3.216638612	3.998591358	3.219673057	2.544937562	2.097316259	2.3018963	2.110429719	1.637837911	0.459342563	0.255361101	0.407628968	0.15147119
PAQR8	9.426326618	14.12852386	8.101767539	9.228720041	6.344435894	9.680494008	7.556321996	8.690289141	33.7037499	22.81469266	27.17031318	37.71475015
PAQR9	0	0	0	0	0	0	0	0	0.02780544	0.308189989	0.137610882	0.079770511
PARD3	0.008773775	0	0.06500399	0	0	0	0	0.008585183	5.232337334	5.291440713	4.498576707	6.309349274
PARD3B	0	0	0	0	0	0	0	0.002227847	0	0	0	0
PARD6A	0.373677506	0.602606253	0.615230662	0.713385699	0.433540113	0.341113942	0.768543998	0.690663412	0.677757605	0.956089399	0.849747199	1.728361075
PARD6B	0.104937388	0.169225938	0.098726381	0.223904269	0.121748209	0.155663264	0.119902856	0.159727193	0.355283033	0.357989704	0.251188315	0.351889072
PARD6G	0.042082555	0.189049422	0.14846919	0.056710291	0.073236182	0.187274755	0.072126131	0.164711976	1.068581333	1.368975317	1.420333718	1.445227056
PARG	11.99428533	11.2327211	11.15809593	11.38265547	12.76625221	12.36781692	13.07465871	10.39925183	7.303183987	7.733453146	8.043664998	8.571260026
PARK7	150.5323665	147.2481856	178.3775336	161.8941192	142.3966948	153.9011165	170.8430116	159.6966604	156.5036023	155.5904822	177.1942537	132.7788342
PARL	19.71481432	22.24828648	21.20008894	21.56780008	20.07937418	22.31337128	24.18887465	23.01637696	40.21756817	36.93423883	38.84139402	32.98357854
PARM1	0	0	0	0	0	0	0.010725948	0	0.590236357	0.766290576	0.449402626	0.73811303
PARN	13.78342171	13.00296423	13.94852075	14.43667525	13.77180784	13.49121833	14.42081007	14.33392062	12.00438597	11.52420255	12.30886015	14.70228791
PARP1	110.9678723	115.557823	117.6928868	137.1434277	142.7863763	155.2562344	127.9721223	143.9928765	167.8278178	181.6475946	176.6341102	174.8542781
PARP10	14.72900212	14.81048541	11.77794764	7.592443998	10.7873722	10.06389042	11.41306809	8.607006385	22.67807432	14.82391851	16.77169428	22.48556991
PARP11	3.184236186	3.342868716	2.783647176	3.615493418	4.387620792	4.077269789	3.233065988	3.224252352	0.997902883	0.640871482	0.727234859	0.639687796
PARP12	6.587055029	4.439378874	5.960013619	4.460404434	3.897113071	4.357359108	5.386728438	5.010293253	8.978267693	5.471079734	5.910429261	9.839670227
PARP14	17.07588027	12.93721179	12.74588478	11.24210561	12.58807593	12.93629056	12.3369331	9.786694784	20.25733962	16.45087427	13.72315145	13.84875228
PARP15	4.973993021	4.047303421	3.761350689	2.767856847	3.88366557	3.79347449	3.615222236	2.53013243	3.597080769	2.409339466	2.785235139	2.180570212
PARP16	1.262349788	1.844754731	1.867304305	2.01369618	2.50918999	2.545917113	1.454542287	1.904910917	1.837186697	2.36818009	1.949531582	2.358348199
PARP2	11.00252403	13.69658923	13.79831612	19.43274927	18.40917061	18.08319884	15.02597242	19.73414631	17.61508546	25.68848981	19.20275955	19.57685081
PARP3	5.095522266	5.041394102	5.124631402	2.927610255	4.106382925	4.559802082	4.913849878	3.455025159	2.646050909	2.43436515	2.596319928	2.156342666
PARP4	53.06208077	45.98105429	47.63063568	49.1332452	48.11846838	45.29970311	46.56489106	43.15337071	39.59420226	31.6994212	35.46577215	37.8918072
PARP6	17.88213423	18.14929126	16.54359142	16.65525175	16.88329819	16.92330599	14.61398764	15.48615539	18.66122746	15.8156055	15.32522911	21.42832333
PARP8	16.03428671	12.59331984	15.19480877	11.39207785	18.1147991	14.87001792	15.3976671	9.870160263	33.32758405	23.38662258	26.02362205	22.3944232
PARP9	4.162138714	2.336076041	2.616502947	2.257396455	2.221451208	1.851317247	2.664866442	1.919604235	7.024971349	3.800872987	4.07638044	5.179846413
PARPBP	6.509851418	5.834238916	7.209212823	11.73699426	12.83703368	11.58187656	9.117269827	11.3522292	5.456040137	7.138317129	6.519037847	5.345402255
PARS2	2.728816791	2.665293325	2.721130372	2.210964524	2.307756189	3.196120403	2.551076474	2.912902992	4.638520782	3.264282236	4.518389305	4.787835441
PARVA	0.006031786	0	0.01276825	0	0	0.006194424	0	0	2.710969641	2.162810269	2.098602206	2.535988693
PARVB	21.30460021	23.22272325	22.78111252	19.49809008	21.64232244	23.90526504	22.42099024	23.16728846	10.90918641	14.94777483	14.76950078	10.26646479
PARVG	51.84429398	44.01177811	45.1828328	37.19397204	42.45802839	40.67783756	39.77926806	38.76517678	36.28262378	31.89999868	31.14994336	34.79046814
PASD1	0	0.015491286	0.015815824	0	0	0.015345865	0	0	0	0.016385552	0	0
PASK	6.494508973	6.717697324	7.06696432	9.491959857	9.935935098	9.742147564	6.370973356	9.946505174	4.748825295	4.456934705	4.91267491	6.204380302
PATE1	0	0	0	0	0	0	0	0	0	0	0	0
PATE2	0	0	0	0.033161688	0	0	0	0	0.321356289	0.215869662	0.424110027	0.20487402
PATE3	0	0	0	0	0	0	0	0	0	0	0	0
PATE4	0	0	0	0	0	0	0	0	0	0	0	0
PATJ	0	0.005669694	0.005788473	0	0.005710621	0.005616471	0	0	0.005951647	0.011993978	0.017673047	0
PATL1	18.54019494	20.76033778	18.94748705	25.29634598	22.13460566	23.5224906	18.06097005	23.08824533	25.84786989	26.97342827	28.47561664	31.10447311
PATL2	0.629289215	0.497578885	0.428980315	0.258720326	0.267291014	0.328605304	0.372922836	0.260916394	2.716081695	1.824515837	1.895675144	2.708370925
PATZ1	10.9250053	11.02480462	10.51090359	10.83864685	11.6992649	12.18322753	11.48747517	12.11115727	12.3146179	11.2325122	12.30949197	13.74106258
PAWR	0	0.005283588	0.010788555	0	0.010643455	0	0	0.004987017	1.397677664	1.844235986	1.213254074	1.71316957
PAX1	0	0	0	0	0.010498281	0.010325198	0	0	0	0	0	0
PAX2	0	0	0	0	0	0	0	0	0.055321924	0.083615079	0.041068756	0.066130018
PAX3	0	0	0	0	0	0	0	0	0	0	0	0
PAX4	0	0	0	0	0	0	0	0	0	0	0	0
PAX5	5.768114336	4.307944295	5.063037691	3.784802394	5.102157379	5.45223292	4.086939102	2.789559117	0.210333845	0.112591129	0.214697073	0.087999168
PAX6	0.028389554	0	0.036057475	0.034431889	0.035572522	0.017493023	0.005838891	0.072226239	0.210085548	0.186781812	0.250757608	0.20090351
PAX7	0	0	0	0	0	0	0	0	0	0	0	0
PAX8	0.026539857	0.068784367	0.014045076	0.013411879	0	0.013627733	0	0.012984693	0.28882001	0.34922441	0.300171585	0.538583737
PAX9	0	0	0	0	0	0	0	0	0	0.018990787	0	0
PAXBP1	20.66564432	18.88382973	19.27943996	19.82364764	25.49747437	23.53049735	23.04607725	17.76539274	20.16110583	18.59778036	18.6553773	22.27766055
PAXIP1	8.174804629	8.44528737	8.983563494	10.71600608	12.45734802	11.68709029	9.011225788	10.67163687	7.853865637	7.393745182	9.13249261	7.974464269
PAXX	22.07236841	27.95976003	29.51315245	41.97690198	32.25285892	38.22628809	33.71147363	49.58576745	42.9948368	43.75203001	49.80204635	60.82404667
PBDC1	17.15845206	15.32577468	18.63751595	18.25255658	14.96604225	16.35945082	20.17164796	16.86977384	21.4802739	18.14137537	21.65844482	29.8746028
PBK	7.007756763	6.875717325	7.602534062	14.69664324	15.94137077	14.57333954	10.44933868	15.33057355	48.42631677	57.57729181	55.2118926	43.4447271
PBLD	1.49775424	1.041455089	1.411253576	1.12610159	0.724744857	1.087952002	1.296037537	1.304705963	4.949424986	3.525040694	3.875918405	6.576924303
PBOV1	0	0	0	0	0	0	0	0	0	0	0	0
PBRM1	13.21710573	11.61051243	12.81250808	15.45114267	15.58219372	14.46751184	12.3215716	12.06389788	11.45816355	9.249264628	10.42905162	9.855512366
PBX1	0.062685882	0.025994465	0.019904281	0	0.013091053	0	0	0	0.573030475	0.673628691	0.297100997	0.313134569
PBX2	52.63723927	52.67632282	49.73840782	43.99223566	47.2857465	51.28748442	51.75293479	43.03348577	62.27624164	64.62383857	54.98514558	58.57836881
PBX3	26.25933747	13.57624931	24.95279686	14.09413618	29.44131494	13.79767132	25.51915413	12.49842238	80.18488609	56.11748103	66.57911604	59.64024138
PBX4	0.080997834	0.027990026	0.057152816	0.081864278	0	0.083181824	0	0.052837861	0.881458962	1.332261338	1.308718848	1.685867501
PBXIP1	17.53622497	18.07739804	15.3046453	13.05177541	12.19585051	12.34955525	14.90378566	14.16526351	30.00416962	19.53584146	21.67140433	34.22250039
PC	2.753087191	3.716570429	3.513026198	4.10879342	4.594170816	4.588890487	3.986531062	4.473065157	0.886680654	1.24140539	0.997746891	0.722969526
PCBD1	17.55960948	15.82503823	17.00135561	12.77278373	12.11940218	10.99744602	16.55267074	13.7001748	23.12653045	29.53865465	25.07613879	22.70412709
PCBD2	3.959913015	3.331095577	4.191226547	4.871337642	4.016718153	3.973733564	3.560252601	4.36191206	4.383250529	3.697078124	4.362970965	4.803942541
PCBP1	158.2326835	162.5417412	163.2472926	182.0707358	197.9212872	195.1631501	153.9651273	198.7039123	140.6267681	147.3929945	147.9993016	149.3197365
PCBP2	154.9464208	167.9504919	157.9267758	171.7823553	173.9115196	174.6342022	158.0132277	183.9448565	169.155987	178.1109654	173.9033897	171.7052329
PCBP3	0.014724192	0.053425756	0.015584288	0.096731043	0.007687344	0.037803023	0.015141652	0.036019242	0.528778898	0.524734483	0.396509123	1.501680691
PCBP4	5.7003227	6.101989847	5.404421966	2.64606928	4.769479801	4.500161567	6.721179811	4.305984822	7.739065719	7.736943496	8.120238677	9.642322471
PCCA	2.859116402	3.348971998	2.528323586	3.252477018	2.345693836	2.859943227	2.33559593	2.912715184	5.354090209	6.426353365	5.359541086	6.401714255
PCCB	49.61980879	42.35618808	45.96799549	38.88608747	38.14411673	47.63929609	44.5778986	40.80936887	41.72089562	56.42199461	49.9975001	36.81963221
PCDH1	0.005672524	0	0	0	0	0	0	0	0.265444345	0.255026286	0.219968515	0.247939464
PCDH10	0	0	0	0	0	0	0	0	0.110608101	0.152511555	0.09795695	0.116907931
PCDH11X	0.854282409	0.742444818	0.974569886	1.271865337	0.854633309	0.803769385	0.578654669	0.615677363	0.295045881	0.252419132	0.209387274	0.351357289

PCDH11Y	0.182001897	0.217708293	0.214860239	0.286535622	0.29237309	0.280363976	0.230353242	0.253435456	0.091413957	0.080596583	0.05278157	0.116558135
PCDH12	0.58221256	0.544069459	0.607542623	0.472410029	0.128436754	0.193689507	0.556556093	0.473410968	1.07978499	0.602452466	1.236610563	1.775033672
PCDH15	0	0	0.00321858	0	0	0	0	0	0	0	0	0
PCDH17	0.037138857	0.02887626	0.171973716	0.004692017	0	0	0	0	0.075780679	0.061086403	0.03000347	0.072468595
PCDH18	0.00901937	0	0.019092451	0	0	0	0	0	0	0.069230744	0	0
PCDH19	0	0	0	0	0	0	0	0	0.168063878	0.181440266	0.160410619	0.269777393
PCDH20	0	0	0	0	0	0	0	0	0.992720557	0.950886736	0.970476411	1.324377474
PCDH7	0.706013514	0.614452138	0.558134489	0.440472735	0.623438197	0.411756879	0.501946935	0.319832318	0.109082361	0.205490248	0.117359892	0.30387351
PCDH8	0	0	0	0	0	0	0	0	0.512000749	0.427461118	0.637100915	0.643504708
PCDH9	0.016292762	0.026274314	0.022992645	0.020126391	0.024573691	0.031605026	0.013031428	0.014171156	0.20882705	0.188582141	0.220349634	0.237379264
PCDHA1	0.327168288	0.369100948	0.346279429	0.34039358	0.241145059	0.326107856	0.247385407	0.169483675	0.010471791	0.021103138	0.020730223	0
PCDHA10	0.009665809	0.0100205	0.030691278	0.019538412	0.010092833	0	0.009939854	0.009458044	0	0	0	0
PCDHA11	0.023213788	0.024065626	0.016379862	0.007820703	0.008079781	0.031786286	0	0.007571603	0	0	0	0
PCDHA12	0.019732146	0	0.010442387	0.009971611	0	0	0.040583181	0.009654002	0	0.01081855	0	0
PCDHA13	0	0.021078451	0.021520038	0	0.021230606	0	0.010454405	0	0	0	0	0
PCDHA2	0.151275432	0.073800723	0.094183531	0.044968719	0.065041771	0.082246424	0.036603385	0.008707281	0	0	0	0.018521217
PCDHA3	0.171271772	0.135778615	0.149286452	0.101825817	0.052599512	0.093118166	0.113964959	0.029574758	0	0.011047439	0	0
PCDHA4	0	0.020433744	0.010430912	0	0.010290622	0	0.010134646	0.019286787	0	0	0	0
PCDHA5	0.040481144	0.020983307	0.021422901	0.030685632	0.021134775	0.010393165	0.010407216	0.009902751	0	0	0	0
PCDHA6	0	0	0	0.019965187	0.010313289	0.010143256	0	0	0	0	0	0
PCDHA7	0.02055369	0.010653958	0	0	0.010730863	0.031661836	0.021136428	0.02011189	0	0	0	0
PCDHA8	0.030395158	0.031510519	0.021447103	0.020480199	0	0.010404907	0	0	0	0	0	0
PCDHA9	0.034888412	0.006028109	0.018463188	0.023507745	0.006071623	0.005971521	0	0.005689748	0	0	0	0
PCDHAC1	0.019007263	0.00985237	0.010058775	0	0	0	0	0	0.010342327	0	0	0
PCDHAC2	0.017567601	0.009106125	0.027890686	0.008877762	0	0.009020643	0	0.008594993	1.347814927	1.14618357	0.889389511	1.74596632
PCDHB1	0	0	0	0	0	0	0	0	0	0	0	0
PCDHB10	0.148462921	0.102607213	0.139675736	0.033344677	0.034449294	0.050822004	0.050890712	0.016141302	0.664210764	0.52456372	0.444219065	0.600846554
PCDHB11	0.016450492	0.017054149	0	0.033252931	0.017177254	0	0.016916896	0	0.089511246	0.072154539	0.070879491	0.085599051
PCDHB12	0.063156762	0.098211482	0.050134491	0.079790447	0.016486737	0	0.048710535	0.030899606	0.343651743	0.554031684	0.527233807	0.410790044
PCDHB13	0.58371776	0.366267428	0.504006914	0.434708741	0.176435851	0.157751809	0.473895243	0.270554585	3.777952069	2.779252574	3.127251496	3.836635947
PCDHB14	0.51596349	0.57712566	0.273051437	0.370527265	0.382801807	0.334658341	0.390962579	0.425156121	3.797494844	2.888430135	3.320037025	2.769564515
PCDHB15	0	0.019329217	0	0	0	0	0	0	0.182614107	0.122670217	0.080335002	0.058210914
PCDHB16	0.201845219	0.283105649	0.301603431	0.180003868	0.074386759	0.10974054	0.024419756	0.081326235	1.498843296	1.223833007	1.138259405	1.346840241
PCDHB2	1.84279318	1.769647696	1.909375869	1.764479154	0.344331498	0.438258864	0.33911242	0.246751286	8.549421092	6.083363758	7.083279575	8.801557279
PCDHB3	0.210076367	0.155560852	0.190583763	0.06066388	0.078341883	0.077050277	0.092585334	0.058731655	0.914462464	0.691071869	0.614206564	0.577791204
PCDHB4	0.069449614	0.08639771	0.102909001	0.11230804	0.043510686	0	0.042851188	0.02718272	0.468586982	0.365540781	0.433889883	0.419196713
PCDHB5	0.110491911	0.057273226	0.058473081	0.093061548	0	0.037823722	0.018937429	0.018019483	5.230563003	4.866548329	5.236786368	4.810309243
PCDHB6	0.008880931	0	0.0187994	0	0	0	0	0	1.90394069	1.996351996	1.922809446	1.580427744
PCDHB7	0	0	0.015163147	0	0	0	0	0	0.109134132	0.062837459	0.061727054	0.11927348
PCDHB8	0.25803183	0.308654307	0.252096413	0.401218507	0.186529394	0.122302749	0.163290794	0.09710979	1.879221086	1.784713647	1.881456998	2.272179591
PCDHB9	0.317100767	0.341380618	0.245263564	0.184899712	0.152819942	0.187875539	0.163045599	0.107406245	1.818332098	1.283866713	1.445101406	1.370779935
PCDHGA1	0	0.023977701	0.012240013	0	0	0	0.011892364	0	0.616667654	0.342385151	0.323877992	0.252735198
PCDHGA10	0.113666162	0.023567436	0.0721835	0	0.059343894	0.035019302	0.023377764	0.022244583	1.59569395	0.884940976	1.248858	1.727046732
PCDHGA11	0.43673294	0.189527027	0.419244709	0.277161108	0.912054489	0.584104507	0.605783265	0.327962747	1.22687175	0.679363314	1.115910489	0.856155305
PCDHGA12	0.737883029	0.301347842	0.72182	0.090397122	0.408588812	0.137777998	0.333413644	0.076578121	0.219000338	0.122593755	0.228812044	0.151254038
PCDHGA2	0.010945802	0	0	0	0	0	0	0	0.54794138	0.228047826	0.330131754	0.353125684
PCDHGA3	0.011519923	0	0	0	0	0	0	0	0.325950478	0.101056518	0.161314953	0.119886317
PCDHGA4	0.132129063	0.057073994	0.081577546	0.08902831	0.114971964	0.1017688	0.090583455	0.086192639	0.335508737	0.108663673	0.225347323	0.13750497
PCDHGA5	0.19787206	0.144799797	0.11087498	0.09411234	0.121537527	0.215160773	0.167573513	0.05694671	0.266001168	0.076579331	0.238215957	0.133244189
PCDHGA6	0.290047223	0.264607736	0.306989981	0.211067925	0.424005596	0.333612083	0.369855585	0.261107665	0.277766635	0.152663323	0.287434063	0.181108987
PCDHGA7	0.042511998	0.110179976	0.078741748	0.064450131	0.088780247	0.152803954	0.087434593	0.083196411	0.069395525	0	0.057240474	0.066362511
PCDHGA8	0.075935171	0.05904123	0.020092708	0.009593432	0.069378654	0.058486991	0.039044042	0.018575738	0.878012321	0.416330036	0.725927141	0.395123647
PCDHGA9	0.03458198	0.035850978	0.109806134	0.023301272	0.048146357	0.059190723	0	0	0.464150709	0.29072421	0.471839061	0.095970692
PCDHGB1	0.128284605	0.072541121	0.074060834	0.035360968	0.097419677	0.035930077	0.08395019	0.034234674	0.164988587	0.05115247	0.087934964	0.121367358
PCDHGB2	0.396258408	0.27789361	0.456411719	0.365159243	0.316408223	0.203471474	0.263672015	0.125445573	0.329762764	0.166137506	0.326403356	0.291092392
PCDHGB3	0.260168152	0.304895347	0.335227639	0.21722056	0.295284836	0.267183214	0.232647332	0.166027733	0.332367656	0.173651722	0.219321141	0.235437876
PCDHGB4	0.046862235	0.036436396	0.086799362	0.023681763	0.061165685	0.060157259	0.10842946	0	4.589804603	2.941868436	3.117034793	6.096113525
PCDHGB5	0.046407556	0.024055249	0.036838798	0.011725996	0.036343337	0.023829434	0.023861651	0.011352507	0.505030246	0.241716928	0.524879593	0.09659146
PCDHGB6	0.081108296	0.02402417	0.024527468	0.035132538	0.036296382	0.059496618	0.119154108	0.045351359	0.491768309	0.29222666	0.386910597	0.229108329
PCDHGB7	0.130027131	0.123565312	0.160559593	0.065709024	0.214971642	0.055638802	0.089142437	0.063616103	0.365546913	0.190106724	0.408509792	0.157870436
PCDHGC3	0.259688208	0.168260974	0.229047984	0.043744354	2.033706767	1.300115353	2.325568085	1.238767869	10.49173679	5.790093441	9.440776184	12.13891272
PCDHGC4	0.067304016	0.02325792	0.023745166	0	0.117129036	0.023039591	0.092282957	0.043904881	0.524912456	0.307506641	0.471233363	0.221800933
PCDHGC5	1.139181239	0.358926478	1.158441795	0.022575828	1.189508813	0.240861276	0.884351998	0.076498651	0.546932662	0.379646244	0.625572559	0.37193125
PCED1A	11.90793943	11.78799244	10.92147761	7.194044765	11.89645612	12.38992811	11.3708706	9.067517607	7.039657359	8.246867629	6.775057707	7.756919015
PCED1B	0.264238661	0.372551589	0.190178213	0.202969594	0.187620424	0.173672626	0.249991919	0.175820032	0.851170894	1.587821506	1.309290104	0.824969046
PCF11	20.49644231	21.16010452	19.46293113	21.62987676	21.44649926	19.54183676	20.7265567	18.42768995	20.27159454	16.64585822	18.47494836	21.03732816
PCGF1	15.24350166	17.11977295	18.17756382	15.04707686	15.01498018	15.81106605	16.35496463	16.30798913	11.33563994	16.32510074	11.93168215	11.95066207
PCGF2	0.295116422	0.890024212	0.350216538	0.253080392	0.588294551	0.76227656	0.441430623	0.341277082	3.464640105	4.177474401	3.053657979	4.299732635
PCGF3	8.297245234	7.839538248	7.32752928	7.209484777	9.376651821	8.520968562	7.749443647	6.91133916	9.077101331	8.599206151	8.41896574	8.589291087
PCGF5	2.410908055	2.57704144	2.515696833	3.964796496	2.382303195	2.790649661	2.325183614	2.745602641	5.225107104	5.899690639	4.951797237	6.102398671
PCGF6	12.20970305	11.28190085	13.22939046	15.60829951	17.58671993	16.4542352	15.03726984	14.94587607	8.271682051	7.752592683	8.811218628	7.809711901
PCID2	9.766821367	8.95366165	9.146650233	9.674905979	10.59342473	10.19296304	9.780805118	9.161599521	9.788460912	11.52275865	9.958639192	9.440468169
PCIF1	8.256207328	9.418826578	8.604926086	10.07537629	11.21853668	11.23721888	7.415097873	11.18323503	4.060823857	4.368498067	4.252466701	3.658219522
PCK1	0	0.020745304	0	0	0.020895053	0	0	0.019580858	0	0.021942869	0	0.020825176
PCK2	11.97528054	14.31962147	12.1047713	11.8152059	15.0184319	16.999464	12.55914467	14.24434534	15.30754556	13.54825198	24.17772567	10.66567193
PCLAF	63.88772481	71.17921021	75.0826872	100.1320576	109.6218944	98.04982754	72.76702751	100.9500552	43.75398559	43.9701691	48.17994692	32.68764313
PCLO	0.099592844	0.082128644	0.098223364	0.105233574	0.120537026	0.132496788	0.060518839	0.106311385	0.569005064	0.819057062	0.419358643	0.709025725
PCM1	32.38985352	29.81531289	30.09931951	31.75577259	37.17124884	33.98250891	35.88269066	28.79471489	56.31790158	42.08148387	48.18417089	49.62083604
PCMT1	29.41837583	29.0502087	32.71336227	37.63679488	32.5893153	31.9564157	33.96219862	37.80443816	36.67489456	37.82200812	38.4322196	37.90582901
PCMTD1	10.55679597	10.8055011	8.825498325	7.807939059	10.68561863	9.479109114	9.239723495	6.043033414	13.6114283	11.68597097	10.69895959	10.16263422
PCMTD2	13.60369522	11.36501661	12.60764535	11.51097127	14.16160109	13.47609342	12.19297411	10.79438868	14.2503734	10.15080366	10.71224738	12.13882061
PCNA	115.5674133	138.4158027	156.5214094	209.3942136	182.7824358	179.2707525	143.5567705	210.1005198	68.30443022	68.12918397	77.73781892	89.24096361
PCNP	46.14660653	48.80289972	48.9315775	48.79516696	65.13612788	61.1138614	59.763718	48.72822986	51.36769823	62.03676864	50.20770564	49.29836367
PCNT	7.222149545	6.708185763	7.203313619	9.930868197	9.200382129	8.488376051	6.831380683	9.216486965	10.44808891	7.331593966	10.00655224	7.929400245
PCNX1	6.162797453	5.549735724	5.278508458	4.89663863	5.109821496	4.474142057	4.902692527	3.697805444	13.20969452	13.1096051	11.89643373	14.39342728
PCNX2	2.661059297	3.327084793	2.285754021	2.500312175	1.668569194	2.149170419	2.172702914	1.975793285	2.823136648	2.998606997	2.283034302	4.898512026
PCNX3	9.349845728	8.618019246	10.13071862	10.90368718	8.749069335	8.22320925	9.109531981	11.70586377	11.40770679	9.253526296	11.13654921	16.46659534
PCNX4	12.43353139	12.03046527	11.35643821	13.61374567	12.16539157	13.31263936	11.12859602	11.73820097	19.89527433	17.52205212	17.69605287	17.88753872
PCOLCE	0.353700006	1.051146903	0.474190554	0.476644773	0.88638245	1.186574264	0.436473717	0.8306334	1.46267338	2.352935173	2.082760605	1.521442475
PCOLCE2	0.026507173	0	0	0.026790723	0	0	0	0	0.230771456	0.377860525	0.25697307	1.379283223
PCOTH	0.684848956	0.456415541	0.517752557	0.247205312	0.510789078	0.351657464	0.452742283	0.478662985	0.159704337	0.107280681	0.158077374	0.305448539
PCP2	0.770492938	1.07941412	0.462851551	0.273609594	0.804532333	0.898196308	0.749508853	0.652048648	3.036689801	2.831479264	2.646857984	4.182580292
PCP4	0	0	0	0	0	0	0	0	0	0	0	0
PCP4L1	0.031652683	0	0	0	0	0	0	0	0	0	0	0
PCSK1	0	0	0	0	0	0	0	0	0	0.011253934	0.033165194	0
PCSK1N	0	0.052775896	0	0	0	0	0	0	0	0	0	0
PCSK2	0	0	0	0	0	0	0	0	0	0	0	0
PCSK4	0.978355652	1.897641676	0.801681379	0.271128407	1.235780029	1.231611393	0.762684132	0.617629658	1.390973262	1.851456908	1.835737242	1.06742769
PCSK5	0	0	0	0	0	0	0	0.006217136	0.245462053	0.421509582	0.253227422	0.740568741
PCSK6	1.155185521	1.14474125	1.033870537	0.403489017	0.576502275	0.671674082	0.925892312	0.498685983	3.891558993	5.038889743	5.114537071	5.392159162
PCSK7	10.74490177	10.5317757	11.55259262	10.22945479	8.555313385	8.181342917	10.48588798	10.68038852	8.361057779	9.005058186	8.499828338	13.17852554
PCSK9	0.014503925	0	0	0	0.045434069	0	0	0	0	0.031808288	0	0
PCTP	13.03401444	14.01275784	12.34771724	14.53957558	9.022820235	8.973214414	12.12938937	12.83257673	14.62205283	12.61606688	9.983834123	17.28195682
PCYOX1	10.90801959	10.93480831	9.8293065	10.80218711	8.892498723	9.393353009	10.13671943	9.841211264	18.25249002	16.87720434	15.9321923	21.55806595
PCYOX1L	9.220714583	9.116945825	8.265079768	8.100551212	4.422464609	6.56963637	5.686259394	6.604999754	24.00584466	25.89806647	21.57525017	39.79151052
PCYT1A	18.00798053	16.07590227	17.52199336	17.04501964	12.70980704	11.98655365	15.82404535	14.31115449	22.70785002	18.64945697	19.47451663	23.17794175
PCYT1B	0.009025421	0.009356612	0.01910526	0	0	0.009268779	0	0	0	0.009896742	0.009721856	0
PCYT2	9.129597057	9.888124559	8.666523248	9.622198504	7.798827801	9.001827464	7.890672561	11.23621659	15.31850758	14.35425776	17.82186069	16.27563308
PDAP1	35.57954601	40.26663452	41.08902779	44.63669286	44.92436484	45.02627918	46.7539983	50.67526162	29.79088719	36.24961935	37.87233668	30.69630974
PDC	0	0	0	0	0	0	0	0	0	0	0	0
PDCD1	0	0	0	0	0	0	0	0	0	0	0	0
PDCD10	20.41984053	20.34879906	21.42879038	22.0427692	20.53599276	19.95957022	22.74537388	21.32179199	16.78193952	16.23255205	16.4654481	17.83609116
PDCD11	61.82129808	53.55617131	63.19274938	58.48798239	58.800538	60.83301284	62.72730278	58.93744004	18.76373226	19.57482534	21.82765917	22.38242139
PDCD1LG2	0.255324132	0.220577784	0	0.064513842	0	0.065552145	0.109401281	0.020819663	0.347320623	0.373297742	0.481295285	0.309997858
PDCD2	30.24374432	27.02080188	29.91884185	28.38634908	34.63943411	35.03639036	33.90417736	29.96038302	19.54001782	18.86695381	19.85475835	20.86071263
PDCD2L	12.77860901	11.561476	15.14806273	10.37920748	14.31356269	14.36390158	15.2912381	14.04987466	4.045475445	5.7577671	5.355701461	3.820304843
PDCD4	62.90392958	63.83948162	51.26982395	44.20817112	47.14646732	51.19587102	50.36727287	42.47260281	68.69273097	44.89948876	55.75169819	46.18621997
PDCD5	40.04019632	37.96054276	45.35824713	45.00488033	38.89651611	42.70836572	43.39195068	46.52414732	14.26702116	18.96370561	17.72721285	13.61707436
PDCD6	110.7007265	103.9009985	115.9628877	106.3265983	105.6410822	103.9480873	116.6670103	113.7952715	131.4078244	116.9170378	130.8351408	142.2914751
PDCD6IP	28.23814701	27.72921514	27.08060287	26.53907616	25.84669026	25.92327011	27.44594274	24.18531657	35.91414082	32.47043167	32.35309759	41.30916733
PDCD7	15.96734075	16.24020379	17.51932276	19.95330984	17.77639157	18.49122325	17.04113553	20.53667355	18.8142278	20.19932282	17.0571271	22.88274891
PDCL	4.547138925	4.376506035	4.445962631	4.447186894	5.460777853	5.457024207	5.0324333	4.356916786	6.319811896	6.40250432	6.244117959	6.218456955
PDCL2	0	0	0	0	0	0	0	0	0	0	0	0
PDCL3	11.67837671	10.23387537	13.16426818	10.87703372	11.62014084	11.37478052	13.2212007	11.6835695	7.437326079	10.07811941	8.574383412	6.785268034
PDE10A	0.007212594	0.007477263	0.003816955	0	0.003765619	0	0	0	0.011773659	0.027681162	0.019422862	0.015012103
PDE11A	0	0	0.005817444	0	0	0	0	0	0.053832923	0.036162027	0.047364007	0.045760081
PDE12	6.961826146	5.344497859	7.103982806	7.534251064	6.777308911	6.284263932	7.269349283	6.483800091	6.51165332	6.5064537	6.283799348	7.081601724
PDE1A	0	0	0	0	0	0	0	0	0.010741678	0	0.005316124	0
PDE1B	15.44640107	19.82588178	11.82360176	15.18184865	11.80858818	14.36394235	13.35513449	15.31680657	9.480384206	7.372396197	8.71530102	11.06343046
PDE1C	0.003941226	0	0.004171448	0.003983386	0	0	0	0	0.162983503	0.185833743	0.140096405	0.127149042
PDE2A	0.011113086	0	0	0	0.023208095	0	0	0	0.06046908	0.024371902	0.023941224	0.069391448
PDE3A	0.482480008	0.337962691	0.483060062	0.174628252	0.336998247	0.187482472	0.375471877	0.095697798	0.024833866	0.014298891	0.017557768	0.047496946
PDE3B	10.23983054	7.092803279	8.791646376	6.822818756	8.347131468	6.578307674	8.100115308	6.032219105	38.7365775	21.80006766	29.34547905	28.43302425
PDE4A	10.46104895	10.14582676	10.96231234	6.099107918	12.39467778	11.35574012	10.11751623	7.173792291	2.916622701	2.483795964	2.9241605	2.537225172
PDE4B	0.096088502	0.307899373	0.110946977	0.035315045	0	0.008970854	0.053897891	0.042737766	0.446791896	0.287358966	0.338737229	0.390901404
PDE4C	0.02610003	0.090192598	0.027624631	0.026379225	0.045421826	0.026803779	0.008946672	0.034052015	0.123081219	0.085859235	0.065599341	0.045269926
PDE4D	0.830453835	0.76453033	0.831452236	0.839337306	0.733220647	0.668446734	0.768269224	0.756128931	1.109613023	0.703186442	0.797828241	0.870916017
PDE4DIP	1.583940306	1.972010905	1.598124812	2.663152685	1.943738632	2.059915306	1.750631171	2.085838151	3.999198711	4.070246731	3.240912316	5.549822093

PDE5A	0.051932569	0.053838253	0.054966148	0.037491499	0.108453768	0.099046734	0.0762928	0.050816283	0.250283465	0.374217733	0.263716562	0.378318771
PDE6A	0.008030079	0.008324746	0.008499147	0	0	0.008246599	0	0	0	0.008805309	0.00864971	0
PDE6B	0	0.007411203	0.01513293	0	0.0149294	0.007341631	0.014703114	0	0.140035688	0.148941559	0.130908591	0.052078158
PDE6C	0	0	0	0	0	0	0	0	0	0	0	0.018854848
PDE6D	19.03211926	19.24373715	19.87880674	23.09550499	20.59202044	20.02749604	20.40866576	25.23910002	17.23704367	17.95188353	20.77045343	21.10953255
PDE6G	0.065621416	0.136058833	0.092606148	0.176862337	0.045680324	0.022463601	0.022493971	0.085614518	0.142825017	0.071956554	0.070685004	0
PDE6H	0.051004323	0	0.215934716	0.051549923	0.106515258	0	0.052450396	0	0.055505457	0	0	0.10615905
PDE7A	16.64484434	17.98536693	15.92390133	19.27339088	19.03580228	20.46710695	17.30686342	17.37240513	38.61842772	28.44965143	36.8923793	28.66539827
PDE7B	0	0	0	0	0	0.010258114	0	0.009774072	1.358786362	1.423903595	1.344943931	1.351374949
PDE8A	3.0731857	3.414948017	2.907102526	4.096116571	3.229011094	3.432204102	2.548005232	3.016525074	5.518250889	5.402328046	5.28617358	5.896725025
PDE8B	0	0.010357245	0	0	0	0.010260018	0	0.009775887	0.086978473	0.24101304	0.139900139	0.093574098
PDE9A	0.010615419	0	0.011235506	0	0	0	0	0	0	0.023280479	0.022869088	0
PDF	1.440497743	1.541530133	3.000103333	1.69073063	2.668630332	1.431618098	2.245900596	2.728131001	1.061937304	1.070027514	1.051118978	1.644181831
PDGFA	7.427008251	4.075247214	7.588020263	2.312183815	3.095320643	2.068130934	4.721713427	2.017836774	2.52466737	2.526235189	3.314576467	2.347259233
PDGFB	0.013954762	0	0	0	0	0	0.014350407	0	0.04555881	0.061207857	0.045094686	0.014522536
PDGFC	3.39979994	3.508536086	3.892809291	3.701690109	3.856589567	3.713655171	3.829918282	2.873077651	2.926231111	4.033038798	2.913066669	2.717924728
PDGFD	0	0	0	0	0	0	0	0	1.56917944	1.773234391	1.103202975	4.431668428
PDGFRA	0.059604437	0.03432869	0.042057439	0	0.006915298	0.013602574	0.006810482	0.012960721	0.194593593	0.108931153	0.121273718	0.227441681
PDGFRB	0.018722881	0.116459544	0.049541389	0.028384744	0.048875086	0.067297009	0.04813428	0.027480652	0.407503529	0.420873234	0.473938774	0.243558189
PDGFRL	0.338957864	0.234264028	0.179378839	0.342583739	0.176966295	0.203056803	0.203331326	0.386950654	1.32178743	2.10619289	2.09940029	2.263472551
PDHA1	7.472271497	8.828111646	8.184830846	6.3581178	9.580754372	11.03003165	10.46113952	10.23932054	22.92571627	26.07835194	22.92354804	26.26694856
PDHA2	0	0	0	0	0	0	0	0	0	0	0	0
PDHB	37.67021648	35.76467265	41.47445502	40.35258417	38.00979894	40.49538516	41.49231645	41.06721522	51.92395551	52.08768607	49.42117758	45.03382303
PDHX	5.349691654	4.393584795	4.669466253	4.300957491	4.878711695	4.958814552	4.92979546	4.418902958	13.81732922	14.85584635	13.5643109	12.77960667
PDIA2	0.03141247	0	0.033247391	0	0	0	0	0.030737261	0.205107731	0.241115365	0	0.392286507
PDIA3	85.78872031	89.19249058	101.8026304	124.5124751	85.66003092	86.60005066	105.3796124	122.1327257	116.6946799	160.3396538	129.6641648	133.3778619
PDIA4	36.62972807	33.58719983	43.99760932	47.64896986	33.67794612	33.42110758	40.11472852	47.12641442	40.69235616	52.25362075	45.6573123	54.27905416
PDIA5	10.69255506	9.976429928	10.70541002	11.06980607	9.022477331	12.13830696	10.57965616	12.30076467	10.158065	16.35137334	13.35422711	10.46595961
PDIA6	55.2329305	55.04632135	62.83146114	70.03771425	63.24649702	63.44307391	61.52417889	68.07941883	121.6792765	162.9321481	147.6873542	105.7023493
PDIK1L	1.606013423	1.42109083	1.5195421	2.393800005	2.354526684	2.490635682	1.64320592	2.047702066	2.065515851	2.205771004	2.376482272	3.224537135
PDILT	0	0	0	0	0	0	0	0	0	0	0	0
PDK1	5.470421238	7.390864755	5.661741681	3.852361267	8.641128712	8.201976618	4.207159456	3.383137584	5.222982748	6.744524951	5.701809435	3.472048109
PDK2	4.092826268	4.374580298	4.298323117	3.158572587	2.898788255	3.421195843	3.964164444	3.725443042	9.771218602	6.610159862	8.270478942	9.740374503
PDK3	1.441912715	2.314429531	1.500126548	2.061800697	1.40295904	2.086570299	1.251107252	2.004146176	3.316636971	4.662495476	3.794691318	3.491405157
PDK4	0.014543125	0	0	0.044096083	0	0.044805777	0.044866352	0.014230521	0.633062239	0.271101179	1.190564724	0.272427075
PDLIM1	204.2719912	237.2590492	219.7007608	157.6825679	255.8633014	254.2680594	245.1207848	179.7530983	49.14446131	42.34870918	49.56092509	28.46019985
PDLIM2	6.160418928	8.832565251	6.772920759	7.321258834	6.576376704	7.523945732	7.392498968	8.974624705	6.214508769	4.751759886	5.280933246	9.583159108
PDLIM3	0	0	0	0	0	0	0	0	0	0	0	0
PDLIM4	0.070339295	0.024306807	0.024816027	0	0	0	0	0	0.051031161	0.128549835	0	0
PDLIM5	7.293136705	6.263018383	7.111096879	5.824799469	4.843254051	4.49014515	5.397946248	4.656985232	4.389565909	4.396482377	4.364389914	5.103967966
PDLIM7	45.75625315	48.88493051	48.51127185	30.04531932	37.7462805	36.88462754	45.58893523	34.7641086	29.02550759	25.83925922	31.77015422	33.95505897
PDP1	3.601650253	3.675106313	3.392472555	3.628730466	3.15762476	2.954358341	3.796940587	3.324748158	6.606446177	6.818228061	5.453353302	7.967850056
PDP2	5.709063165	5.850529836	5.425176864	5.718548893	7.324078629	7.105985797	5.758456495	5.208216487	3.776928233	6.24422986	4.068314701	4.105068329
PDPK1	4.389536664	4.181305352	4.265707083	4.653129607	4.025501675	3.571591577	4.15696756	3.846168799	7.004387571	4.869582286	5.658290191	7.02813828
PDPN	0.068766212	0.124756822	0.145566214	0	0	0	0	0	0.093543542	0.037702476	0.074072465	0
PDPR	7.451260026	6.68839022	6.750858014	6.863607558	6.133386369	6.635022251	6.163022106	5.810442452	12.19069638	10.59916669	9.483295358	12.34982106
PDRG1	10.6992062	10.76678241	11.65603172	12.12080232	11.74480059	11.75240634	13.42069257	12.77015669	14.37300264	12.63458562	17.47716338	14.07109985
PDS5A	29.62423339	27.93910857	29.68461431	33.5893866	36.37889259	33.85034707	32.74733368	29.14459481	34.00132352	35.12547937	34.30171667	31.81772129
PDS5B	12.75654802	13.36371456	13.24615355	19.68522119	19.65102293	17.66020388	14.77351734	16.06560745	15.50994594	13.42913967	14.40553871	14.8530047
PDSS1	7.728816491	8.039962258	9.937220232	7.798070818	9.498508653	8.973687497	9.682288753	8.758162313	4.248797526	4.761705432	5.078086081	4.118379036
PDSS2	6.756881638	6.77456529	7.349544231	7.998861425	7.519241355	7.335246618	7.092711069	7.378044486	8.320030441	8.844887747	7.996020383	8.309200067
PDX1	0	0	0	0	0	0	0	0	0	0	0	0
PDXDC1	12.66843518	13.13330789	12.60629466	15.29868356	12.27702242	12.21742404	12.61290039	13.47109421	13.12812715	15.44680998	12.41020067	15.75986583
PDXK	8.768491949	10.0346252	8.372921516	10.64982314	8.54956039	9.240165778	8.441886318	10.60773281	12.220652	11.12794111	10.89686915	17.28032548
PDXP	18.57167886	17.50035539	20.25303395	21.75406744	20.09273547	19.40317348	19.45700417	24.05490391	17.72811074	21.74774695	22.25960689	21.28237452
PDYN	0	0.094031389	0	0.171887385	0.011838769	0.186297374	0.023318655	0.122035864	0.037015333	0	0	0.023598356
PDZD11	9.855356712	9.825410076	12.21195566	10.23843314	9.322634106	8.745752217	10.66446758	10.61794853	17.67584913	17.54692945	16.0162206	16.15284127
PDZD2	0	0.004401114	0	0	0.017731534	0	0	0	0.203279157	0.139655324	0.096031225	0.357862785
PDZD3	0.033453255	0.017340417	0	0.016905554	0	0	0	0.01636709	0.072811013	0.055024284	0.090086577	0.069628721
PDZD4	0.012839313	0.013310456	0.013589306	0	0	0.013185507	0	0	0.964094426	1.281173483	1.189383564	1.563319295
PDZD7	4.529821944	5.727951044	4.160526115	2.949877463	2.977134024	4.565333385	3.435135049	3.433707193	12.22755893	16.85310708	12.92076228	14.56375187
PDZD8	8.780057604	7.125708976	9.466148109	10.5859209	8.826180503	9.075622757	9.373992855	8.062879991	6.411488	6.191152418	6.266844784	8.839242244
PDZD9	0	0	0	0	0.025667793	0.025244613	0.025278743	0.024053416	0.026751145	0.134774724	0	0.025581955
PDZK1	0.012655118	0.026239004	0.053577405	0.025580982	0.02642841	0.05198538	0.039041746	0.024766194	0.123947393	0.180399075	0.163579596	0.144870152
PDZK1IP1	0	0	0	0	0	0	0	0	0	0	0.06483412	0
PDZRN3	0	0	0	0	0	0	0	0	0	0	0	0
PDZRN4	0	0	0	0	0	0	0	0	0	0	0.01415772	0
PEA15	55.6888684	43.73665992	54.96369628	40.8373308	33.13939834	29.97377688	51.72635258	39.1425547	26.67051052	22.6681096	25.07680265	36.20171151
PEAK1	2.851377184	2.886907957	2.645594066	2.743398843	3.143614843	3.141224883	2.418128938	2.036401192	2.466289541	2.277988714	1.966493697	2.289129854
PEAK3	2.868447408	2.474470768	2.526310143	1.375500373	1.049403243	0.989097702	2.325368923	1.126813641	0.546847537	0.137753403	0.42851065	0.413999641
PEAR1	0.058370824	0.095091483	0.097083619	0.016855778	0.069656654	0.068508239	0.042875537	0.024478349	0.22686447	0.265167652	0.251499721	0.078101672
PEBP1	154.5217837	152.2323703	163.9220052	159.3241924	151.3185962	169.7733252	179.8145187	185.1182656	95.33633851	122.7290986	116.0964273	76.92744252
PEBP4	0.026377236	0.082035473	0	0.053318793	0.055085097	0.05417692	0.081375247	0.077430777	0.229640224	0.115694852	0.0284126	0.164702644
PECAM1	18.03410745	16.98866274	15.2452494	12.48669268	7.67295237	7.351024004	11.76399176	7.942341881	33.34124919	29.52626404	31.00698864	38.63253739
PECR	1.653336012	1.962085542	1.945627661	1.736983351	1.476507747	2.010689821	1.89036656	1.351712532	2.272727988	2.957971465	2.448756027	2.252634095
PEF1	6.480094923	7.839784489	6.831021746	8.723765838	7.651315927	7.900089788	7.146509445	9.364489796	5.916838671	6.819744745	6.362164225	5.373289057
PEG10	0.714322123	0.605891803	0.953651942	0.008204129	0.008475909	0	0.008347438	0.007942816	0.008833649	0.008900946	0	0
PEG3	0.017876931	0	0.006307063	0	0.006222237	0	0.012255851	0	0.01945457	0.013068521	0.006418793	0.006201428
PELI1	1.656659067	1.833048404	1.879880141	2.044676262	2.278742277	1.995789208	1.87562958	1.94058306	3.527710276	2.786026557	2.067609618	3.439837628
PELI2	0.87695624	1.294905173	0.830347118	1.051627054	0.888475563	1.355771282	0.911569078	0.927682303	1.782308753	1.580172647	1.342899887	1.600814225
PELI3	1.723185013	2.302084917	1.729830267	2.387991886	1.61381693	2.225743131	1.406671485	1.877357629	2.435899766	1.986941625	3.157372434	3.47566622
PELO	3.317027724	3.305018169	3.881371055	4.470013857	3.655990322	4.163459207	4.150137591	5.012847286	1.885094577	1.535730291	1.86589046	2.320502315
PELP1	13.73335075	14.4819681	15.34442103	17.34464337	18.61158367	18.57019169	16.50347112	19.64037803	8.316544445	8.256668925	8.776573758	8.455974073
PEMT	10.23436746	11.41204581	11.94891686	11.41022165	10.98029594	10.76314814	11.35636779	12.76747478	1.530934825	2.043942379	1.969940255	1.939831136
PENK	0	0	0	0.080750065	0.083425091	0.041024839	0	0	4.825513388	6.351621589	5.464837687	6.610115008
PEPD	26.20873636	24.33963917	25.16122875	31.17000708	18.67302561	21.11444387	22.93243525	29.75807292	12.43998794	10.56794764	11.6211774	15.740984
PER1	5.835365457	5.242215503	5.174680258	3.875404027	4.765430988	4.302197269	4.216785096	4.57180583	2.445737793	3.372296171	2.495145697	3.138974729
PER2	4.582264227	4.019579411	4.341197562	4.145482555	4.876716332	4.376740205	4.489752344	4.178057287	5.405109331	4.805030932	5.298270961	5.760791886
PER3	3.139996683	2.711591685	3.022502894	3.058646913	3.324896855	3.205197432	3.404441466	2.763397788	0.990065635	1.77352587	1.088866101	0.907343905
PERM1	0	0	0	0	0	0.01354423	0	0	0.014352514	0.028923713	0.0142063	0.041175661
PERP	0.076325618	0.011303773	0.034621752	0.022040595	0.034156109	0.078383629	0.0897024	0.064015718	3.286856577	6.037934964	4.310424568	3.687870744
PES1	28.7908243	24.18680257	31.22256465	24.23320545	27.85402876	29.53502433	32.26456051	29.93590193	16.99822373	18.43029741	21.43819791	15.53958599
PET100	102.0635623	106.1379307	111.2175257	125.7757291	90.16406624	107.7497437	102.345576	134.5057107	87.75101648	79.54277014	100.022409	78.29934528
PET117	0.557130915	0.288787515	0.294837522	0.281545308	0.290872124	0.476794293	0.334207226	0.136288857	0.151574436	0.050909728	0.200040392	0
PEX1	5.701352611	5.56957158	4.848142579	6.611916581	5.495290244	5.217027671	5.236608597	4.815889301	6.54919153	5.557124684	4.86841568	6.199561135
PEX10	6.735910363	5.801333792	7.156801117	6.441382511	6.438352467	5.933116158	6.953528559	7.858646019	4.933892627	5.511241514	6.362671369	5.607978314
PEX11A	0.019624202	0	0.020770526	0	0	0	0	0	2.221027985	1.743017623	1.543108814	1.980996786
PEX11B	19.49095666	21.76941335	20.3930561	18.42943181	16.94059678	17.63631656	18.40676835	18.71679082	30.99606757	29.08881974	30.10880403	36.81920334
PEX11G	0.559406326	0.193311313	0.3665278	0.215386806	0.278152468	0.191496639	0.575266597	0.156394837	1.014623266	1.139193366	0.717347818	1.524722493
PEX12	0.381059134	0.41583393	0.67927283	0.567567917	0.293184935	0.432526884	0.371238547	0.333619002	2.029786771	2.221185915	2.095521899	2.087174761
PEX13	3.682647687	4.040178914	3.582411936	3.987041153	3.79556447	4.283756478	3.958639917	3.755092563	6.757220804	6.534260725	6.367442971	6.833974144
PEX14	3.614742698	3.47839136	3.153521146	3.364031047	3.195192074	3.390606786	3.33998436	3.545799131	4.868494934	4.532760384	4.500850617	5.354068313
PEX16	14.43946287	15.40787848	16.47690528	14.36589359	13.57553153	13.67030212	15.25487333	15.56407315	9.69832769	8.65892286	8.900827658	13.64753038
PEX19	26.54546338	25.98393676	26.40411038	26.51814299	25.22203798	25.64964866	27.43381585	25.97487061	28.05816733	31.52046163	27.0456355	32.09748821
PEX2	9.076882541	8.869441943	9.908103332	9.665014547	10.0346826	9.139089272	10.17504188	8.812205577	14.21079042	13.29255119	12.39392518	11.99888319
PEX26	9.482167338	8.436116637	9.631179087	9.208690762	9.707413292	10.24973176	10.1920656	10.03831141	9.675610333	9.317149159	10.40114157	9.699076834
PEX3	8.302268222	8.265059127	9.505817499	8.195025405	9.864084691	8.605810418	9.953946926	7.459481194	6.459562603	6.466232805	5.829601774	9.164924323
PEX5	5.723155137	6.27250032	5.406577396	5.082632346	5.955282804	6.447902354	6.089418474	5.697192429	2.871243572	3.404307681	2.767203917	3.385739874
PEX5L	0.051852142	0.031357011	0.03201393	0	0.004511909	0	0.026661126	0	0.047023414	0.085286978	0.102397613	0.242828277
PEX6	19.72696076	16.04355335	18.77606241	11.73395312	16.98933862	15.23744068	16.96713966	13.63461013	16.78897209	17.76205987	13.82882187	18.51172193
PEX7	2.582858921	2.774653705	2.614875524	2.780737316	2.833768974	2.88315436	2.328888805	2.454084311	3.971777436	5.233431464	3.487782516	4.012442838
PF4	0	0	0	0.410067247	0	0.156250118	0.156461361	0.248128786	0.055191571	0	0.054629315	0
PF4V1	0	0	0	0	0	0.07427481	0	0	0	0	0	0
PFAS	20.75850846	20.02434104	22.35773645	22.54972283	27.02535426	27.96786956	22.83092212	25.55793873	5.267460326	5.327618322	6.59102876	4.257893191
PFDN1	27.44260023	24.10354413	29.07481813	28.48879667	27.70461316	26.11488611	30.1209371	27.63536576	30.73483097	30.45484622	29.70871536	29.56374465
PFDN2	79.04009302	67.00552968	84.56375343	77.44573937	78.05980707	75.17341686	95.13484724	85.5705333	31.5248504	40.5601705	35.73326448	32.41614983
PFDN4	9.728960781	9.058693869	10.2019212	8.922566723	11.13075531	11.53315374	11.88841497	9.637366345	12.90765153	16.75961303	14.32870241	9.531062581
PFDN5	216.0214976	230.9316479	200.453894	209.0702042	178.947184	195.0605706	213.5277002	215.2102223	194.9384773	217.2082427	196.7491891	192.8579253
PFDN6	134.4137871	117.1999283	140.2452527	108.8445325	113.5233953	127.4835408	144.1130088	121.7554072	76.77283088	86.29289127	93.62436171	77.08443237
PFKFB1	0.039546468	0.109327039	0.083713054	0.079939003	0.013764527	0.054150376	0.040667688	0.051595226	0.358637047	0.245731103	0.184591413	0.274369911
PFKFB2	0	0	0	0.004651088	0.014415496	0.009451887	0.004732333	0	0.010015951	0.005046128	0.004956957	0.004789096
PFKFB3	13.41081766	14.15645044	12.392743	13.38620583	8.75333329	8.809929497	11.67862751	11.84954104	12.78466947	10.77974845	12.18028166	14.42472999
PFKFB4	1.481159826	7.815477739	1.455032323	0.654378986	4.361955193	6.421398268	0.985033481	0.9546435	5.48229603	12.18600314	8.60779545	2.335210385
PFKL	36.6128279	41.01490005	37.69658036	47.36036169	45.5572388	45.97986785	37.7602047	55.03264823	47.04618677	49.28446383	49.44423918	59.4288141
PFKM	0.451211216	0.435133555	0.466461937	0.40301022	0.262964736	0.312510394	0.399259207	0.215622366	0.570965845	0.49477147	0.440816389	0.589691992
PFKP	42.44483547	43.71830294	45.91852518	31.99282711	40.55967496	43.57589992	40.46820231	35.59562662	40.0706599	50.33399344	51.76356804	37.97985452
PFN1	536.9899327	556.3638293	610.3829817	534.7626578	462.5276738	473.4988607	538.9999092	580.4083757	431.1117711	441.4551021	506.6488097	409.4823589
PFN2	0.306607184	0.34434643	0.27043106	0.025823917	0.106717566	0.052479068	0.052550017	0	2.196629776	2.241381742	2.449473706	1.568820053
PFN3	0	0	0	0	0	0	0	0	0	0	0	0
PFN4	0.109636432	0.022731916	0.046416285	0.044323691	0.022896006	0	0.067646904	0.042911922	0.214761334	0.216397461	0.283431314	0.387930432
PGA3	0	0	0	0	0	0	0	0	0	0	0	0
PGA4	0	0	0	0	0	0	0	0	0	0	0	0
PGA5	0	0	0	0	0	0	0	0	0.042402793	0	0	0
PGAM1	181.1250124	210.6113727	212.7132653	192.1694802	213.403822	204.6200942	195.9700191	195.262888	126.2317145	152.9660573	160.6250042	113.160039
PGAM2	0.062788286	0	0.066455988	0.12691988	0	0	0	0	0.068329355	0.068849912	0	0
PGAM4	1.956128255	2.194131128	2.070393122	2.171517459	1.43982857	2.338195803	1.945634627	1.882703039	0.942236556	0.984578356	1.796191055	1.034544615
PGAM5	34.14773915	30.75498289	37.92768196	40.83065977	38.91980458	41.4708174	43.32944307	43.85047196	14.72054526	15.34041202	20.14587795	16.88227405
PGAP1	1.987388277	2.385629275	1.776164326	3.075023478	2.04194755	2.77890237	1.973583574	1.909069156	1.430302256	2.029646751	1.121807821	1.760614466

PGAP2	4.76098205	4.461102586	4.623779522	4.626837387	4.725481849	4.32519875	4.088938025	5.132188687	5.081501646	7.113799144	5.015645723	6.96923583
PGAP3	5.244633483	6.10445526	5.490873356	6.1044577	4.250584696	5.055495858	4.283510532	7.392159888	7.644304493	6.519132141	6.118406438	10.36432822
PGBD1	2.52301176	3.003936119	2.460490757	4.031005535	2.991107216	3.598039686	2.220657843	3.191086565	1.858421344	1.606793978	1.388517471	1.972115192
PGBD2	5.188843206	4.988369908	5.016861616	6.169822263	4.574434056	4.535893496	4.689733948	6.166559173	4.943356369	2.756293763	4.486858608	6.408949717
PGBD3	0	0	0	0	0	0	0	0	0	0	0	0
PGBD4	2.692799422	2.382497001	2.211281414	1.736196064	2.496652398	2.312452321	2.124603081	1.408318186	4.041984957	3.487316344	3.825772502	3.8653252
PGBD5	0.905503759	1.149946135	1.01829752	1.487183828	0.543666962	0.557951622	0.325911801	0.708832018	5.099520072	2.991176739	3.584505864	5.500943575
PGC	0	0	0.031413558	0	0	0	0.030521328	0	0	0.03254516	0	0.030887424
PGD	131.8704248	130.6597837	125.2513623	140.0124262	118.1357798	122.4198292	118.8890758	135.1183791	124.5944841	130.2269433	142.9797939	164.4761371
PGF	0	0.058382126	0	0.056918021	0	0.028917037	0	0	0.3064273	0.185257062	0.454958427	0.029303453
PGGHG	8.811061885	9.617098415	7.885186495	4.754328029	7.11840737	8.386543967	8.00499877	5.397304834	7.366880798	7.370637399	5.542821007	11.50543162
PGGT1B	3.692852098	3.700199282	3.621543494	4.163631451	3.868490027	3.743278673	3.789349672	3.137401199	5.121080807	4.788393234	4.368713601	5.200223838
PGK1	278.9302866	331.596659	313.2520497	297.4911369	357.9676477	339.6819108	307.0039875	299.9456746	315.2672156	429.6385376	408.387208	203.9580199
PGK2	0	0	0	0	0	0	0	0	0	0	0	0
PGLS	46.76727877	48.62436075	50.7653897	39.60031731	43.12670395	43.72808394	44.20661972	52.06760017	18.8487164	16.54746224	20.06254018	19.77929777
PGLYRP1	0	0	0	0.057067333	0	0	0.058064185	0.055249662	0	0	0	0
PGLYRP2	0	0	0	0	0	0	0	0	0	0	0	0
PGLYRP3	0	0	0	0	0	0	0	0	0	0	0	0
PGLYRP4	0	0.021944973	0	0	0	0	0	0	0.046072586	0	0	0.066088394
PGM1	9.019079393	12.67745325	9.477975535	15.57105931	8.613186548	10.53129527	8.796195789	12.67250616	11.03752415	15.9961158	13.13775568	14.49658342
PGM2	35.76241886	33.63430304	37.60054401	36.95955364	42.91978362	39.89139711	38.51522698	35.96790346	49.7874168	44.96809146	44.83733552	48.15411918
PGM2L1	1.46941026	1.781415757	1.317458832	1.515813207	1.147575102	1.471616816	1.436141798	1.420001114	0.680602627	0.739052759	0.71291195	0.840425812
PGM3	6.106649315	7.768741249	7.430180522	8.770698363	8.672655809	8.416753277	7.75840455	7.556133851	9.848685171	10.53583295	8.746186028	8.150735669
PGM5	0.023018492	0.007954388	0.00812103	0	0	0.007879717	0	0	0.367398162	0.395437843	0.438039397	0.127760214
PGP	8.802393087	9.801355566	11.16899251	12.86791936	10.4454412	11.08381009	9.881279978	14.75826658	13.50051654	12.71905518	15.35911216	16.6068163
PGPEP1	32.15296447	31.23757877	26.11474345	16.39382944	17.97669472	18.52115438	22.55884706	16.2181124	8.958329662	11.16998505	7.444841169	11.66036016
PGPEP1L	0	0	0	0	0	0	0	0	0	0	0	0
PGR	0	0	0	0	0	0	0	0	0.132185682	0.378547727	0.182486058	0.206245202
PGRMC1	9.337458972	10.69752829	12.82105343	16.29571232	14.55176598	14.36944702	11.64950903	15.63948366	33.68854393	31.27016608	35.36902292	34.55064941
PGRMC2	9.594892896	10.91094391	9.955682782	11.60434852	11.03191839	12.35852479	9.631340264	10.77321373	15.54514212	16.18076381	15.19807256	17.44618837
PGS1	8.925012189	7.51103466	8.716377443	8.39933378	6.973437698	7.391436971	7.633163386	8.465895963	8.278413498	7.32313174	7.120168673	10.97947953
PHACTR1	0.339451312	0.345140144	0.297096882	0.059379658	0.136326092	0.261453098	0.161111735	0.057488336	0.51148787	0.472435849	0.513308791	0.176631247
PHACTR2	3.071372234	2.550685597	2.895410424	2.987401344	2.661056441	2.617184157	3.237695993	2.41828609	4.252901239	2.746214232	3.753044028	4.410715232
PHACTR3	24.26558533	20.55704965	23.4589327	19.35043346	19.31230736	18.55716933	25.09933928	20.05592714	17.72265242	13.74299933	17.17383812	21.16555312
PHACTR4	7.150471502	6.810761144	6.713945763	8.324727237	8.064939977	7.653116493	7.508332283	8.030051306	13.71612665	11.04988003	12.66642781	11.1935001
PHAX	9.14806871	8.579815477	9.150611662	10.67986695	8.888655867	9.53132875	10.6080602	10.42646562	11.19377833	9.366802629	10.79319832	10.65840069
PHB	85.75898762	85.93696482	102.7819725	91.61564046	91.87968194	97.73114303	105.8879506	107.7739627	63.81935047	75.0807615	83.43315546	54.98719998
PHB2	211.6583974	236.1257209	232.3499817	211.6988857	236.0390667	256.1205453	252.5937289	250.3841955	57.58431516	78.22270834	74.51071399	60.50054282
PHC1	1.790215315	2.030810213	1.507894576	1.695688065	1.066776006	1.029937131	0.751810328	0.990509712	2.764202694	2.980537983	2.130276863	4.174800331
PHC2	16.44681887	15.67509786	16.82138155	15.60742392	15.03514621	14.64177301	15.9992539	16.81163389	12.67033614	14.34859845	13.45688189	18.1480395
PHC3	5.614405903	5.033767936	4.994395093	5.087739437	5.862443195	5.007806063	5.121178749	3.562400483	9.042979668	9.225543311	7.132992274	7.184878367
PHETA1	1.722131801	1.668255476	1.912370411	1.098546165	0.648535976	0.840793966	1.306444152	1.022118756	1.321093942	1.129936864	1.611736775	2.130073539
PHETA2	0	0.045169329	0.069173419	0.022018287	0.022747692	0.022372655	0.022402902	0	0.047415593	0.095553643	0.023466277	0.090686476
PHEX	0.015583423	0.008077631	0	0	0	0	0	0.007624229	0	0.008543929	0	0
PHF1	14.35214225	17.65435499	15.02791578	18.07663638	16.88812014	16.51954165	12.70542395	19.58379716	16.50234732	20.69484582	16.73608735	18.68152498
PHF10	105.304732	83.14300098	106.7269265	94.02071561	153.1207738	105.9984065	116.6203538	83.28850311	185.7842911	127.5255833	150.5827056	116.07896
PHF11	0.372682094	0.445797456	0.364109417	0.289745171	0.449015439	0.382730933	0.471690296	0.39272296	0.062395586	0.157177342	0.24703976	0.089502769
PHF12	9.0275161	8.405277745	8.957669567	9.850574203	9.59582068	8.881995567	8.886055399	9.514131547	12.67556642	9.49222914	11.50575617	12.49156706
PHF13	5.475212916	6.782613457	6.235170545	6.276289421	7.49736262	7.416460307	6.556975071	6.483281746	5.294680121	5.000628326	5.449773786	5.885510233
PHF14	3.104648602	3.262525905	3.658784738	3.975061414	4.195280964	4.075877459	4.094802423	3.701660416	4.17715567	3.894288707	4.187293986	3.835076102
PHF19	4.696729973	5.822333359	4.808879507	9.985951547	8.914206451	8.822787236	4.885514068	9.491464379	10.45788273	11.89181812	12.44880283	10.39483664
PHF2	7.882416987	8.057011598	8.257727633	11.65541636	10.79223262	10.41812457	8.416073739	10.08394087	11.37903351	10.53964534	10.5808267	13.51841878
PHF20	12.26622885	11.87741708	12.59506656	11.39875942	11.19822376	11.62595369	12.40376725	9.918788144	10.93853781	13.1048183	10.74452351	12.57914159
PHF20L1	12.34785809	11.58912902	12.14501144	13.90250409	11.83719042	10.80905937	12.82060593	11.22341193	14.37216311	13.24171586	12.3139069	16.1075038
PHF21A	7.871096597	10.05899175	6.994922376	5.030938849	7.245548915	7.531589024	7.002597932	5.162051796	9.685745635	13.27296764	9.266342375	8.225027725
PHF21B	0	0	0.022560024	0	0	0	0	0	0.011597991	0.070118092	0.034439515	0.044364348
PHF23	17.36112307	16.06467516	16.33822413	14.0575233	17.50657996	15.38408784	19.85291974	16.67731026	8.291434628	5.004058297	8.292456128	8.403964538
PHF24	0	0	0	0	0	0	0	0.014431391	0.248774287	0.307272335	0.150921251	0.406734499
PHF3	17.34784808	17.0425319	16.53409681	19.98982083	21.35169501	20.20657334	17.50684184	16.2137236	23.63711833	20.59798125	20.02602551	22.89359794
PHF5A	35.06463403	29.54201374	36.7385547	38.35048039	41.20365233	36.06198939	39.1243018	42.8639332	39.03883804	41.66318264	42.72294589	33.96740718
PHF6	9.616233333	9.69256892	9.648587439	9.894339757	11.35016505	10.33437006	9.305963913	8.633018239	8.505425366	10.02540553	8.181730136	8.237785713
PHF7	3.090987034	4.669844163	3.650563565	5.695679654	5.78596105	6.677708653	2.887894926	5.090090444	7.465921391	8.266812518	5.562699405	9.33641886
PHF8	10.1402282	9.106696184	10.17531932	11.43684889	9.81254505	9.524855879	9.426484167	10.47977464	13.18561478	10.7553879	10.02929358	14.29807027
PHGDH	11.49954035	13.24613338	15.00027047	25.1787066	27.42631179	25.35696822	14.59552946	26.97186971	19.89522609	24.37878078	37.87485988	18.64473669
PHGR1	0	0	0	0	0	0	0	0	0	0	0	0
PHIP	23.80046803	19.4565815	22.24563895	23.38225847	32.29408657	25.65908303	24.89968461	16.83590954	59.48070053	42.95518453	43.8070971	44.90534379
PHKA1	3.109268571	4.518142627	3.47577435	3.292593392	2.699446907	4.296341447	2.371122272	2.384380296	2.661306927	4.386301485	3.339783282	3.381202786
PHKA2	10.95124211	11.64235281	9.175725877	10.97774699	9.187582072	10.61205473	9.09956094	10.08206072	8.252376597	8.774169	7.867764589	9.333149806
PHKB	11.78324723	12.44947451	11.23012589	13.213298	9.307940484	10.24783152	10.01117474	11.40636878	19.92198249	21.75563702	17.52262772	22.88217915
PHKG1	0.438664898	0.505290957	0.309525985	0.525460566	0.390186102	0.417123027	0.250612173	0.333850089	0.424335196	0.676982556	0.367510802	0.439604882
PHKG2	6.757933959	7.542499213	6.790921473	8.409467086	6.995307416	7.963122342	8.365552603	9.336099995	7.343694307	6.510827514	6.92174247	8.648831744
PHLDA1	5.205321971	6.235972339	4.507678277	2.612106863	1.615382338	1.102779319	2.844899549	1.620638078	1.297333278	1.676430727	0.950834645	1.145927069
PHLDA2	13.04296964	10.06539587	17.08583439	6.088774311	8.855528045	8.409200217	14.73599579	8.413022243	2.673311735	3.335029854	3.213094442	1.582577576
PHLDA3	13.99522328	16.67793807	13.72633064	10.93289523	6.513216569	6.932896856	15.87384493	11.85946368	0.601478796	0.562770995	1.020602265	0.883328191
PHLDB1	0.851564989	0.586747975	0.643006409	0.493313312	0.807858052	0.623899802	0.875708533	0.518247485	5.899336136	3.365008708	4.60874687	4.139260495
PHLDB2	0	0.008421516	0.008597944	0	0.050893839	0.008342461	0	0	0.008840317	0	0.017500514	0.01690788
PHLDB3	1.025222989	1.199495268	0.883589694	0.562503105	0.51996493	0.797170469	0.783186916	0.730892582	1.944503909	2.537477177	1.877366014	2.179597859
PHLPP1	2.711274753	2.994423939	2.298981438	3.293004053	3.096466858	3.923445097	2.479230969	3.361466652	25.5238896	14.67085575	20.41021915	20.15992422
PHLPP2	1.143237323	0.913583049	1.05876588	1.51053196	1.175091838	0.960041164	1.047063595	1.130316266	1.542200418	1.527832643	1.564972413	2.41048707
PHOSPHO1	0	0.02429622	0	0.142121521	0	0.072204432	0.072302049	0.02293246	0.153026804	0.256987693	0.2272018	0.243897643
PHOSPHO2	1.254119408	1.422027866	1.037013498	1.425976744	1.227679504	1.126943073	0.926954742	0.843674015	1.279495784	1.117344307	1.013168891	1.509074477
PHOSPHO2-KI	0	0	0	0	0	0	0	0	0	0	0	0
PHOX2A	0	0	0	0	0	0	0	0	0	0	0	0
PHOX2B	0	0	0	0	0	0	0	0	0	0	0	0
PHPT1	27.18392021	26.89035076	27.90822643	28.29938542	21.56891472	21.47792763	27.7779893	33.53314988	33.55533006	39.32055274	39.08829975	28.8262488
PHRF1	12.17800416	12.61485261	13.02245871	13.32500176	15.31173588	14.26460875	12.37406954	14.54746555	14.68430138	12.16574103	14.43051583	16.29736064
PHTF1	7.347541557	6.915058543	8.209531762	8.665981508	7.458660754	7.086356596	7.634702574	7.827293548	14.74038292	9.892445569	12.67697199	12.67322503
PHTF2	6.011498606	5.36148601	5.481214484	6.097023713	6.291693799	5.720812202	5.678169782	4.882499401	8.453591539	8.479624614	7.553339455	6.940688073
PHYH	22.93068265	23.74342184	24.68051562	22.67214954	22.06409025	22.29758081	23.12514479	21.27254181	16.33483033	20.49817431	17.00369073	15.30386788
PHYHD1	0	0	0	0	0	0	0	0.024638712	0.109608343	0.138054222	0.054245862	0.05240889
PHYHIP	0	0	0	0	0	0	0.024441329	0	0.038797432	0.013031001	0.01280073	0
PHYHIPL	0	0	0	0	0	0	0	0	0	0	0	0
PHYKPL	4.204272502	4.402843971	3.468539246	2.595322084	3.841260411	4.023649397	3.822581869	2.688250986	8.169513868	6.760625074	6.208538847	7.741311964
PI15	0.015884741	0.008233819	0.016812629	0.016054662	0.024879763	0.01631305	0	0.02331495	3.077009522	3.440107434	2.275691981	2.760683721
PI16	0	0.045059872	0	0	0	0	0.022348614	0	0.023650346	0.023830523	0	0
PI3	0	0	0	0	0.101420214	0	0	0.190082774	0	0	0.104624014	0
PI4K2A	17.49549341	17.29853762	18.0824061	17.44895359	14.04334642	15.40802473	16.47241689	18.52719416	12.02198641	9.888057005	13.24166875	10.23995142
PI4K2B	8.854377475	9.961450303	9.733200191	11.16478593	10.88061815	12.01695686	10.39364264	9.555531437	23.02052116	26.4427011	24.05870715	22.57733382
PI4KA	26.61067483	29.18889329	24.29686502	35.78910308	22.03741074	25.83009629	22.77605511	32.11667469	17.60759136	17.07093472	17.73234455	22.15959923
PI4KB	26.53321792	27.25427544	26.57452621	30.38772835	24.47439451	24.89660687	26.87207043	30.90990336	48.03182371	40.93036065	42.84468246	52.53779416
PIANP	0	0	0.009493713	0	0	0.009211612	0	0.008776951	0.009761336	0.009835702	0	0
PIAS1	11.09826448	10.12909207	10.96825676	10.37743063	10.81363036	10.00603773	10.64972673	9.527228585	16.70129209	12.89390795	13.6635017	15.41865163
PIAS2	1.809845382	1.705913794	1.68368109	1.776254843	1.832610665	1.861090855	1.80238465	1.619480668	2.697784812	2.778396809	2.336834485	2.577396654
PIAS3	14.48148416	16.41614057	13.52186985	20.22108784	16.49591745	17.38552676	14.56790774	19.25043313	31.15573705	33.60931454	28.98085183	34.30945628
PIAS4	8.422989927	8.523813795	8.869446475	10.94954728	7.641404614	8.473270314	9.81276977	11.34495096	6.293060692	5.790295936	5.827083275	6.600403543
PIBF1	10.84533785	9.833893403	9.97450376	10.02448413	12.63114078	12.24837037	11.86774918	9.493551245	11.06268537	11.09614275	10.13456145	10.17723312
PICALM	23.80224719	22.85390031	21.54970103	22.24402391	19.75396231	18.68151989	22.64504505	18.09647942	41.9403733	41.7283188	37.76648844	44.73605725
PICK1	0.018987142	0.059051646	0.020096253	0	0.158607759	0	0	0.018579015	5.785572328	5.100942737	4.192713215	5.730303692
PID1	0.006440402	0	0	0	0	0	0	0	0.049061376	0.077683796	0.027749469	0.040214649
PIDD1	6.234529	7.35660987	6.48068574	7.366792808	8.267123159	7.922609075	6.369590726	8.272897932	9.046301958	8.726155588	8.211605341	9.494915025
PIEZO1	28.23854443	33.8125292	28.08562797	31.40476233	34.78594233	37.92196879	23.29973814	34.21887059	58.62341027	48.58797596	51.50559104	51.51981767
PIEZO2	0	0	0	0	0	0	0	0	5.615626075	5.581046834	6.030666114	4.184971088
PIF1	2.847552726	3.675771761	3.130555749	9.228210582	8.363756983	7.867398847	4.647473984	10.3903696	8.156971281	10.41490664	8.667540527	6.11800875
PIFO	0.145994933	0.151352268	0.088298886	0	0	0.021418782	0.107238697	0.020408112	0.068090997	0.137219475	0.06739733	0.065114999
PIGA	2.914132628	2.989378231	3.192202853	3.769167148	3.723798432	3.432196631	3.227273567	2.861463819	4.191445024	4.592084427	4.060941177	4.400595156
PIGB	8.674645327	9.385973232	8.520494841	9.421052828	8.988024978	9.732985864	10.22771856	8.248158685	17.73977866	18.14390624	17.07864538	15.71125342
PIGBOS1	7.561599042	7.878665875	6.386472282	8.491651591	6.061315395	9.098954143	7.108350168	7.660646066	10.88876036	9.882918628	10.94237982	11.16798479
PIGC	11.94572251	14.87576579	12.83336005	17.43951073	14.53365191	18.0885901	14.12891287	16.88403902	12.33627282	12.35158236	12.40380458	11.87176655
PIGF	20.52132138	19.00923892	19.67954386	16.93041475	18.78857916	19.71073681	19.51710141	16.05578945	16.92408226	16.95905982	15.6902701	18.27989532
PIGG	7.795552561	6.725931261	7.599061914	6.753379356	6.941862233	6.818748964	7.218385023	6.530019803	12.5232773	10.97196425	10.93256969	12.70467335
PIGH	4.443939405	3.714179678	4.338912276	4.804835638	4.604295919	4.45762982	5.030469807	5.730472353	8.097684715	6.572830433	8.126512869	10.36087181
PIGK	8.689092513	7.862353512	8.704197229	10.43969514	8.672151799	8.708360202	9.043101365	10.30067015	13.02560452	14.46410733	11.66501213	15.55524968
PIGL	1.364672651	1.384902754	1.151854004	1.367631313	1.719574576	1.673484192	1.498105669	1.205749241	0.651691678	0.814506612	0.601635661	0.880881555
PIGM	10.61998043	8.712235573	9.355963611	8.997083653	9.061128794	9.282529597	9.704779559	7.918649563	8.942260449	7.481350565	7.147984278	11.11685683
PIGN	10.45729118	9.685920827	10.50553774	11.89158987	10.97264988	9.941423682	9.27158398	9.682377397	8.510081051	8.11534328	7.366333539	9.201946712
PIGO	3.356357879	3.675156314	3.57381556	3.807779214	4.004294642	4.235896874	3.21587148	3.996452225	4.694048662	4.818493496	4.602670356	4.783473072
PIGP	10.73865156	10.16814322	9.683614112	7.013649265	9.877218203	9.63474854	9.767374734	7.928286758	9.83002442	11.90289972	9.562845921	8.432110848
PIGQ	6.74196843	7.849022148	7.250270317	9.055085023	8.338610316	7.719802755	7.489248852	11.5183793	10.31880845	9.586975397	9.864169333	15.43956239
PIGR	0.012464573	0.012921965	0.013192676	0	0.026030483	0	0.012817968	0	0.054258288	0.164014939	0.201395774	0
PIGS	26.38972198	30.96960463	28.40700857	34.31963476	28.00539354	28.33228523	27.08014133	36.10381789	42.86391041	44.68970365	40.24838107	56.38872758
PIGT	26.52145319	24.88677201	27.71566784	40.6072697	24.36595809	25.71112164	27.42121187	40.83766747	39.44730318	47.0773993	43.71644078	45.55208734
PIGU	7.318248871	8.973213915	9.10222221	11.01970796	9.193145513	10.18608384	8.76729101	11.52293979	7.256615218	8.574677805	7.902959249	7.731984651
PIGV	3.332368744	3.341383939	3.160831179	2.742264	2.928178338	3.627928583	2.996151222	2.51237323	4.775817243	4.812201089	4.354483321	5.040848423
PIGW	5.980800371	4.846817131	6.535565068	9.789330348	7.037166255	6.75808231	7.275213875	8.873313167	3.321501138	3.308114103	3.895786639	4.626794265
PIGX	7.458578872	7.047364001	7.765451926	8.979263701	6.626229887	7.498995993	8.063379875	9.101556269	10.68995646	9.684724449	11.59234073	12.1225403
PIGY	0	0	0	0	0	0	0	0	0	0	0	0
PIGZ	0.768855444	0.904057975	0.693613658	0.505884288	0.457985988	0.577616991	0.642074733	0.464525259	1.813801019	1.73708697	1.550759045	1.868777982
PIH1D1	25.82628843	31.78293625	28.308972	31.42189591	33.06342346	38.69118157	29.72305918	34.67044773	46.53988683	48.56586665	51.33219827	40.79449445

PIH1D2	0.203486876	0.237323137	0.323059974	0.192809626	0.278875625	0.182851907	0.287727179	0.298669411	0.262965465	0.167348734	0.30138442	0.357355299
PIH1D3	0	0	0	0	0	0.028632362	0	0	0	0	0	0
PIK3AP1	94.39555944	85.98144993	94.54138902	81.69203032	82.91403904	76.69227139	84.31498264	70.88177883	32.1677838	25.32807344	32.84921848	30.30238592
PIK3C2A	6.520460088	5.929473618	5.513066607	5.640557156	5.373072505	5.30391604	5.298116976	3.375266822	12.59280461	11.99034163	10.16860077	13.4336981
PIK3C2B	2.606474206	2.756379129	2.310019434	2.98348751	2.328136973	2.61225395	2.314378192	2.668240021	2.067566123	2.175144235	2.311477337	2.766991597
PIK3C2G	0	0	0	0	0	0	0	0	0	0	0	0
PIK3C3	13.20053114	12.27444484	11.44332085	9.993182881	10.75802051	10.39933355	11.62027845	8.882196363	11.29119838	8.64395293	8.59189122	12.01906495
PIK3CA	5.297254873	4.724949579	4.526611427	5.180091703	6.387312395	5.675589395	5.500501985	4.398028533	6.164027059	6.09154472	5.631906428	5.220438577
PIK3CB	16.2790549	17.9517178	15.83884	24.27966473	11.62428471	14.84779243	15.16913151	18.94296789	20.35235479	18.70150417	16.21324289	28.50208815
PIK3CD	27.02747324	25.05742795	26.31515319	27.09515159	27.67245587	29.58324549	26.70327401	28.39313448	32.37871054	28.90637753	31.52021637	30.88147744
PIK3CG	9.16868675	9.456920495	8.529907855	8.695986336	8.664935361	9.020166221	8.615587936	7.762389976	15.01734781	15.67815948	14.14153926	14.9348835
PIK3IP1	2.241158418	1.579008701	1.496939258	0.857671482	0.999684246	1.005548174	2.036190973	0.8303535	2.296866533	0.835080106	1.968776024	0.5661029
PIK3R1	9.118863734	9.036502204	8.491014702	10.56406562	8.428595125	9.22893794	8.017536612	8.114136507	17.01103577	12.28301178	13.12239676	18.69879266
PIK3R2	13.07927803	16.92090227	13.13963563	17.01764857	12.6370712	15.73097774	12.85014867	19.10427732	10.23219167	11.08377683	9.528752089	13.54083308
PIK3R3	0.435352434	0.333982606	0.377842077	0.343207375	0.29093488	0.214603723	0.358156426	0.230037065	0.900167382	0.343714802	0.506461488	0.942376291
PIK3R4	11.97073184	10.86013562	12.62477807	14.01991149	12.9232982	12.52380307	13.26550562	13.27033518	12.73662428	13.47768106	11.65215436	13.80246723
PIK3R5	4.24689509	4.850267303	3.754042827	2.735767371	2.229441489	2.707906252	3.683412086	2.602963499	3.948746302	4.055591212	3.983924558	4.383261596
PIK3R6	0.146221633	0.037896822	0.012896916	0.012315482	0.01272346	0	0.012530609	0	0.159125699	0.093530485	0.183755401	0.07608546
PIKFYVE	17.326862	15.06308607	14.73158479	17.15691931	15.65591255	16.32909403	14.07217013	11.89451685	23.385602	22.05541279	20.41994881	22.29458148
PILRA	2.220199435	2.762004525	2.264439167	1.958355658	0.927314006	1.16075982	1.826517169	0.947989707	1.537538633	0.575436509	0.956607262	0.966222561
PILRB	27.3538171	27.27137521	26.57532424	30.67202485	24.57684029	28.43723181	28.62960007	36.43231132	27.40591586	21.50089858	22.28986239	27.53214289
PIM1	68.84604575	42.66692512	59.76993623	30.89655758	25.49043024	14.31998594	41.69032028	25.42267636	4.25838931	5.771930307	5.306202476	5.395242745
PIM2	16.8047234	15.89095008	16.22386043	12.78188233	10.19943258	10.15761515	11.18342213	13.16925093	17.29704347	13.51677711	16.4317587	18.28221937
PIM3	18.90911849	19.41540679	20.73186575	18.31127366	14.17069322	12.72918508	16.00277238	19.03384546	17.05795382	17.75835731	19.71039019	15.4885563
PIMREG	0.927351329	1.249795057	0.981521436	3.702221731	2.130305161	3.476099505	2.217221289	5.467193333	4.995491124	7.39779101	5.793673093	4.946046644
PIN1	30.23253419	34.27318714	34.57683004	42.29469054	33.93010394	31.67313268	34.7578217	49.16259678	35.59887576	36.20397747	42.49900179	40.01861485
PIN4	3.480324037	3.716060779	4.080660163	4.423270878	3.285904955	3.916601053	3.729015961	4.71062217	6.713107704	5.964423588	5.297816446	5.985939612
PINK1	7.464559383	8.661218895	6.102083711	8.484904124	2.598111365	3.220064315	5.491911398	7.10616589	23.68747541	12.48851983	15.49277117	22.33640288
PINLYP	0.106729707	0.073764128	0.056482097	0	0	0	0.036585235	0	0.058074305	0.175550205	0.15328715	0.074048131
PINX1	13.12679096	12.08063782	15.12694892	15.20706307	14.17195685	15.09983231	15.33171849	16.8355223	8.280212145	8.493623314	8.158940451	9.701720053
PIP	0	0	0	0	0	0	0	0	0	0	0	0
PIP4K2A	27.5843433	24.60235369	25.85578716	25.07236303	26.19911564	23.67958591	25.19433407	22.21530317	24.37247905	17.6896488	21.25983859	24.40189222
PIP4K2B	26.17810616	24.99424442	24.17780045	26.95377619	25.30500724	26.20636825	24.5730323	26.80195457	33.2816606	30.83675173	28.98565396	35.32523743
PIP4K2C	5.003750295	4.263899758	4.388146035	5.546331316	4.535823719	3.952822501	4.568855063	5.88619493	8.748433663	8.175958677	8.161784962	9.922551664
PIP4P1	11.88003292	12.40653356	11.21797207	14.86174791	11.21911726	11.48269269	12.42645815	15.58503885	23.03671693	25.70266307	22.89612753	27.4540056
PIP4P2	7.41638779	7.266087794	6.771364381	6.651423498	5.999499134	5.733194111	7.123800484	6.120578946	10.68726994	10.72400594	9.634679253	10.51702152
PIP5K1A	25.68002333	25.53573388	25.7451171	25.07958865	23.30508967	24.04409112	26.10348381	24.81588364	1.376236827	2.048849755	1.61993328	0.592831874
PIP5K1B	0.085646819	0.056502511	0.074167998	0.157387263	0.07317048	0.135932252	0.072061425	0.198086548	0.652436087	0.503727114	0.369022567	1.029063833
PIP5K1C	15.89380647	14.37343431	15.19171805	13.23790836	14.35672267	10.9907609	14.22286192	13.63969161	9.65262227	7.277875913	8.984100618	12.03457228
PIP5KL1	2.75789321	2.28727606	2.221677403	2.152488165	2.287788213	2.360213208	1.937991358	2.548689583	2.817866015	2.503317704	1.980468234	4.305154523
PIPOX	1.835588699	1.8174206	1.178785048	1.334093825	1.141395296	0.847228188	1.272560396	1.432870031	4.264495532	2.872194535	4.26548375	3.004923663
PIR	2.375470057	3.640422529	2.696420868	5.776031982	2.839895772	2.863785761	2.01798117	3.975077122	9.25392802	15.10028774	11.7925923	13.07715808
PIRT	0	0	0	0	0	0	0	0	0	0	0	0
PISD	12.20161288	10.55919262	12.02916086	12.09062449	11.71143097	12.63797332	12.3325395	13.48838547	12.98585828	11.62728443	13.04661162	15.62000925
PITHD1	24.1180954	26.24123604	27.80924956	40.97328978	28.82084862	29.23148795	30.32858461	39.24623326	27.18518346	32.1577478	26.94397322	39.70328833
PITPNA	26.06977901	22.82843761	24.4016311	26.82663082	19.64454673	18.89570747	26.0034255	27.04228947	10.53437473	8.572731126	8.86697856	15.47236809
PITPNB	37.59685325	33.44289125	37.0298677	34.23442164	39.88290373	37.89337378	37.15800983	34.02264312	31.66778426	29.6115896	32.36613057	29.36654244
PITPNC1	10.36245255	9.857630821	9.400939447	6.697212902	8.113144722	6.764781027	10.68846693	7.388271165	2.233226106	2.499311927	2.23578624	4.043006379
PITPNM1	9.538912999	9.59003655	8.849996827	8.912416555	6.96219541	7.414944318	8.092104256	9.780584141	8.994882978	7.627491125	8.307973599	13.06515947
PITPNM2	6.119600129	5.425549466	5.759023042	3.064544429	5.970705734	5.012044679	5.986990375	3.833983974	0.452012791	0.470638262	0.529432775	0.360214196
PITPNM3	0.089347548	0.144728419	0.118208346	0.220114319	0.128280362	0.212187315	0.057425454	0.114747976	0.048616237	0.067356592	0.036090725	0.075548535
PITRM1	17.83402589	14.81570835	16.82406384	16.61956705	14.11300706	16.37906263	16.24997973	15.06985675	17.63294598	16.22803538	16.67569015	17.90573486
PITX1	0.022580596	0.046818398	0.143397687	0	0	0	0	0	0.933786726	1.584674762	1.556671857	0.892974467
PITX2	0	0	0	0	0	0	0	0	0.323195613	0.277412226	0.379144423	0.194599608
PITX3	0	0	0	0	0	0	0	0	0	0	0	0
PIWIL1	0	0	0	0	0	0	0	0	0	0	0	0
PIWIL2	0.01060078	0.032969339	0.022440024	0.064285069	0.066414656	0.065319691	0.010901333	0.031118752	0.057681498	0.197611183	0.034256326	0.033096276
PIWIL3	0	0	0	0	0	0	0	0	0	0	0	0
PIWIL4	14.44901931	14.00270388	12.92520138	11.73046775	8.852187093	9.569957501	9.150454457	9.842591791	19.40348624	14.12858729	16.72355501	23.019241
PJA1	0.541832384	0.503606654	0.45483124	0.339907304	0.273130271	0.422128503	0.614835199	0.493621203	6.059152988	5.162882755	5.494286493	7.952621397
PJA2	37.59342368	35.16387968	36.1286366	31.54862504	38.52294724	34.28051675	34.10525565	25.71495638	44.31150449	46.04326128	38.85778611	42.48694988
PJVK	0.458289946	0.557022067	0.334524413	0.223610091	0.462035349	0.324584191	0.373776464	0.231951226	0.309559068	0.346574886	0.221292848	0.345367658
PKD1	6.15675633	6.469667808	6.157457481	7.448764564	8.108042347	7.532742647	5.195199635	7.485193111	7.681704958	6.984989284	8.07795818	8.746758028
PKD1L1	0.627823459	0.562907383	0.531597606	0.469902125	0.545709321	0.51231631	0.561866929	0.537952472	0.635218227	0.960086313	0.668957604	0.685153026
PKD1L2	0	0	0	0	0	0	0	0	0	0	0	0
PKD1L3	0.024774199	0.017122198	0.034961806	0	0.017245795	0	0.016984398	0.00808056	0.206697347	0.144884897	0.142324625	0.051564364
PKD2	3.599575337	4.675180097	3.604195396	5.353766122	3.406231236	3.686130931	2.881382744	3.57223402	13.46550571	11.15723156	10.54149571	16.09090366
PKD2L1	0.033956391	0.035202433	0.023959942	0.034319627	0	0	0.058198536	0	0.591248704	0.397168696	0.487687873	0.259145093
PKD2L2	0	0	0	0.012413871	0	0	0	0	0.013366413	0.04040473	0.039690735	0.051128874
PKDCC	0.150606528	0.133828361	0.227720037	0	0.988492259	0.243048798	0.243377388	0.063158251	3.090639717	3.77477005	4.496029792	1.947982487
PKDREJ	0.007024747	0	0	0	0	0	0.007223912	0.013747501	0.015289362	0.023108763	0.007566802	0.014621122
PKHD1	0	0	0	0	0	0	0	0	0.0035357	0.001781318	0.00349968	0.001690584
PKHD1L1	0	0	0	0	0	0	0	0	0.002540709	0	0.002514826	0.002429664
PKIA	0.011756513	0	0	0	0	0	0	0	0	0	0	0
PKIB	0.757100891	0.842313437	0.605880652	0.242624294	0.501323529	0.512022094	0.702608515	0.180689528	3.576997171	3.705490908	3.242195503	2.286845903
PKIG	3.177040553	3.112084892	3.839215848	2.376664737	2.272548341	2.389224847	3.601545097	2.423356534	2.477360015	2.084766365	2.802425467	3.150100771
PKLR	0	0.017487185	0	0	0	0	0	0	0.055070461	0	0.054509439	0.017554514
PKM	417.8313999	396.1733301	436.68875	423.5935725	331.8960538	293.1628071	399.7086369	417.2345684	281.5528319	319.598263	315.7414731	251.0467568
PKMYT1	8.11364982	10.29006761	11.90058117	15.65160612	16.55715076	16.62254896	11.4778649	20.17052478	11.67579484	11.60667754	15.48307277	15.19449647
PKN1	43.77897564	56.53185151	46.65583207	55.33453563	43.5762952	48.68019199	46.14138843	56.87107127	50.76387559	47.17774944	51.69215529	57.88414855
PKN2	6.483649814	6.567373041	5.86057644	6.293345933	5.838231244	5.82529523	5.638499956	4.591171285	12.16928724	10.00828061	10.29750584	11.9047791
PKN3	4.136595902	5.830807861	5.272963832	5.946597823	7.720682845	7.500791192	5.517289466	7.157886569	7.200187871	8.182005827	10.06498792	7.131824569
PKNOX1	5.449323989	6.285094429	6.56574478	7.509461808	6.949861864	6.814630541	5.366022446	7.663795061	5.78798916	4.740635458	5.393297538	7.031251872
PKNOX2	0.015865227	0.010964938	0.01119465	0	0	0	0.005438346	0.015524205	0.028775556	0.023195823	0.045571855	0.038525041
PKP1	0	0.010241256	0	0	0	0	0	0	0	0	0	0
PKP2	0	0	0	0	0.02531507	0	0	0	0.356177729	0.67790563	0.496180387	0.290149735
PKP3	0	0	0.038572829	0	0	0.018713328	0	0	0.257791176	0.179830467	0.176652669	0.132743749
PKP4	9.816957498	10.77192192	10.2880683	14.18262003	13.7236323	15.31114526	11.00339946	13.89487316	21.80853153	21.80504251	20.92675789	22.35126149
PLA1A	0	0	0	0	0	0	0	0	0.029664771	0.059781534	0	0.028368236
PLA2G10	0.034339222	0.035599312	0	0	0	0	0	0	0.07473932	0.037654355	0	0
PLA2G12A	3.001411991	3.0582699	2.839479641	3.573734097	3.058880155	3.12456461	2.991375838	3.178285645	2.225996782	2.569818023	1.904376892	2.62076986
PLA2G12B	0	0	0	0	0	0	0.03070764	0	0	0	0	0
PLA2G15	6.95731127	7.331175753	6.274287986	5.471267404	5.473386335	5.36357267	6.19408893	6.919672833	19.3119863	17.2226725	17.76013945	22.79232546
PLA2G16	0.060904992	0	0.032231342	0	0	0	0.062631769	0.059595844	0	0.200354412	0	0
PLA2G1B	0	0	0	0	0	0	0	0	0	0	0	0
PLA2G2A	0	0	0	0	0	0	0	0	0.056742497	0	0	0
PLA2G2C	0.673086932	0.969147354	0.514514355	0.226762343	0.234274351	0.614431784	0.538354656	0.402489376	0.366243442	0.86107843	0.483349857	0.233490892
PLA2G2D	0	0.141584065	0.057820083	0	0	0.028050994	0	0	0.208075025	0.149757295	0.235377477	0.028425837
PLA2G2E	0	0	0	0	0	0	0	0	0	0	0	0
PLA2G2F	0	0	0	0	0	0	0	0	0	0	0	0
PLA2G3	0.017683063	0.073327797	0.037431996	0	0	0	0	0	0	0	0	0.01840253
PLA2G4A	25.56128621	22.66456047	24.63521733	20.79121718	27.82011722	23.09686482	25.69389118	17.15829863	6.930533018	9.623605803	6.653191665	7.2631516
PLA2G4B	0.255767565	0.285549435	0.312355291	0.278388432	0.225979812	0.181844288	0.080928948	0.288772916	0.342571156	0.647214344	0.254310948	0.511872933
PLA2G4C	1.311449133	0.957250534	1.033959969	0.351657395	0.041920019	0.123686677	0.550461755	0.248795235	0.684466074	0.777724904	0.634248933	0.80774338
PLA2G4D	0	0	0	0	0	0	0	0	0	0	0	0
PLA2G4E	0	0	0	0	0	0	0	0	0.082811748	0.020860659	0.040984058	0.01979809
PLA2G4F	0	0	0	0	0	0.015824874	0	0	0	0	0	0.01603634
PLA2G5	0	0	0	0	0	0	0	0	0.050875984	0.051263575	0.06714359	0.048652388
PLA2G6	3.225730635	3.525474873	3.124822384	3.868077425	4.578521896	4.581643385	2.934866656	4.52594771	3.712695835	4.700206207	3.180178823	2.970069619
PLA2G7	0	0	0	0.014053015	0	0.014279188	0	0	0	0	0	0.01447
PLA2R1	0	0	0	0	0	0	0	0	0.035149014	0.099167016	0.055665502	0.020167671
PLAA	11.57662556	9.45199637	11.06652831	10.82969081	10.65566522	9.947397951	12.54137646	9.993661664	9.567033759	9.172139647	10.12730529	10.55039101
PLAC1	0	0	0	0	0	0	0	0	0.036191753	0	0	0.03460995
PLAC8	4.338216174	5.581121452	4.093740473	2.29796487	4.256989114	7.461101254	5.21370693	3.605664349	73.88749483	81.24205987	81.86140565	46.45270482
PLAC8L1	0.100918156	0.104621384	0.320439505	0.244794457	0.210753183	0.10363927	0.249070523	0.296246784	0.109824188	0.044264347	0.108705371	0.084019353
PLAC9	0	0	0	0	0	0	0	0	0	0	0	0
PLAG1	5.622966256	5.055040522	5.062131636	3.687130075	5.226504557	4.852713431	4.571262367	2.923210754	0.507979008	0.503974371	0.433185027	0.343780775
PLAGL1	11.56160561	10.60739082	11.29872913	9.521693556	12.58442493	11.39880122	10.24078817	9.144593756	10.76544193	8.913734786	10.03613095	7.586251104
PLAGL2	15.58900576	14.48459238	15.26728202	17.11232803	11.49908636	12.38897133	12.43897989	14.44691918	9.520666287	7.066339047	9.841731801	10.43405478
PLAT	0.050117567	0.069275531	0.123771956	0.01688456	0	0.06862522	0.034358999	0	0.127261037	0.054955953	0.125964584	0.034771126
PLAU	147.0130111	112.0650738	141.5805158	68.97442117	30.26520824	35.02257064	81.96780479	60.36235246	22.21750758	22.91227932	22.48674574	23.83993021
PLAUR	15.86859622	15.01229246	17.52127435	12.55676981	13.3307271	11.90380024	13.02794001	12.34851747	10.02028428	14.39305733	11.85260566	11.53618092
PLB1	2.118050633	2.033123379	1.450559647	0.596972951	0.587838902	0.758225999	1.119895345	0.577958565	3.525246245	2.818406356	3.285540358	1.935301967
PLBD1	0.02785174	0	0.029478665	0.197047712	0	0.343232662	0.085924174	0.163518411	1.667031128	1.740812287	2.250066136	0.927517993
PLBD2	13.40089003	14.20736043	12.06072399	12.08681059	7.188921445	9.430910418	9.900791674	10.9910203	13.12044768	14.12395662	13.45393565	16.01096866
PLCB1	0	0	0.023707277	0	0	0	0	0	2.860067502	1.932153787	2.010604401	2.773915205
PLCB2	30.21343182	31.99076967	25.66598722	18.52346652	22.88929826	23.13988692	26.11709667	21.49885289	25.2865201	20.06920267	22.45745336	28.11277222
PLCB3	8.37704624	10.26857818	8.731604571	8.383920514	8.37673367	8.911169282	8.380715081	10.35080386	10.19523407	11.61935364	11.63936848	13.17621149
PLCB4	0	0.005196956	0	0	0	0	0	0	0.076375591	0.137424013	0.091796997	0.07825447
PLCD1	0.04885856	0.050651442	0.034475049	0.065841608	0.085028448	0.050175961	0.016747932	0.063744465	1.435598917	1.339385011	1.052573311	1.338956722
PLCD3	1.256481736	1.181417728	1.097922203	1.269922482	0.90008719	0.795222456	1.232007539	1.572582709	1.87615377	1.377783381	1.526550436	2.615207874
PLCD4	0.02284907	0.011843763	0.024183771	0.04618698	0	0.011732581	0	0.011178966	0.223789517	0.425933912	0.14767313	0.487463875
PLCE1	0.007850826	0	0.004154711	0.011902211	0	0.012093768	0	0.003841036	0.081164786	0.081783128	0.105707805	0.220596771
PLCG1	7.773819281	8.306736583	7.669556935	7.062225001	9.021483761	8.51497632	7.543843642	7.947611823	12.51105936	13.16301803	13.65949139	13.67342702
PLCG2	26.20334651	25.69132055	22.50769731	20.89959505	23.65046709	24.96780108	22.47852174	20.36090613	33.12270771	21.40746739	24.04475406	28.86448352
PLCH1	0.016446721	0.01278768	0.004351859	0.004155664	0.004293329	0	0.004228255	0	0.08501619	0.126241498	0.084150101	0.0599056
PLCH2	0	0.028237976	0.007207388	0.013764912	0.007110453	0.006993224	0.014005358	0	0.014821123	0	0.014670135	0
PLCL1	0.007022913	0.007280621	0.014866296	0.007098038	0.007333177	0.014424552	0	0.006871956	0.213995197	0.200223667	0.143731711	0.255802853

PLCL2	0.043366193	0.06947982	0.041726706	0.075706514	0.123496517	0.186239365	0.129732975	0.13115976	1.724699197	1.456844762	1.690142725	1.222629632
PLCXD1	1.358032077	2.224019482	1.416528577	1.223367911	3.339559172	3.041952822	1.416774598	1.608090532	3.202070019	4.240804521	2.098832722	1.198220769
PLCXD2	0.12525503	0.025970261	0.02651433	0.151913879	0.026157727	0	0.051522503	0.024512536	0.354402859	0.41204172	0.215872267	0.286772759
PLCXD3	0	0	0	0	0	0	0	0	0	0	0.006422349	0.006204864
PLCZ1	0	0	0	0	0	0	0	0	0	0	0	0
PLD1	4.10798721	4.469038967	3.634794444	5.001355884	2.541909834	3.906252962	3.203732623	3.424177243	3.776680373	4.043789759	3.121675167	6.393842226
PLD2	3.398179203	3.900327652	2.970472101	2.897884653	2.582026551	2.27460585	3.291717063	2.894649288	3.466939381	2.977666481	2.810660566	3.69431698
PLD3	30.59795551	33.57767655	26.13104619	31.09323641	18.54897058	22.60482725	24.81917104	34.20452756	22.08494321	22.19244828	18.27961759	37.03152988
PLD4	8.990500094	13.8509242	9.833446741	11.81773192	10.15403087	13.77229036	9.02241113	13.49782137	44.99873505	34.67833037	41.97102906	35.4810134
PLD5	0	0	0.004746461	0	0	0	0	0	0	0	0	0
PLD6	7.384957164	5.375078604	7.274792667	4.774862663	6.179657409	6.23541149	6.840163383	5.039995041	2.858311121	3.328931421	3.784504264	2.183158338
PLEC	19.07400095	17.04565353	16.68163739	17.60528472	14.28333029	11.9547813	15.14334884	16.00875633	13.47648183	10.48817023	11.67927384	18.37720629
PLEK	71.73350294	76.36087346	73.18791847	63.44300699	39.48048543	47.20002702	55.54859244	58.09914637	74.49135926	81.92753241	69.48494559	98.46931195
PLEK2	0	0	0	0	0	0	0	0	0	0	0	0
PLEKHA1	3.064592361	2.623529442	2.732680685	2.29900604	2.420988877	2.313473051	2.745322316	2.075485825	0.023878588	0.064161342	0.015756886	0.030446594
PLEKHA2	7.857875153	11.60907689	7.061140287	8.467704337	8.891160638	9.944258233	8.822092572	7.447853158	12.59360074	17.36305792	10.74493591	15.05397806
PLEKHA3	1.021379769	0.913367493	0.990267877	1.178089213	0.887395646	0.908796935	1.118489592	1.110048695	1.531517119	1.440590752	1.30175528	1.736399964
PLEKHA4	0.413665136	0.463152302	0.385289418	0.033447213	0.328274654	0.288876936	0.612566434	0.097145624	1.584602282	1.016065487	1.711046602	0.964310681
PLEKHA5	3.70893368	2.930462015	3.123847833	2.362136937	5.517398939	3.481646059	3.505352266	1.884658464	0.578042881	0.709065443	0.542302635	0.754663272
PLEKHA6	0.00936796	0.00323724	0.006610119	0.003156057	0.003260608	0	0.003211187	0	0.088353916	0.044513514	0.047090521	0.090991711
PLEKHA7	0	0	0.008467556	0	0.012530508	0.01232392	0.016454109	0	0.156712552	0.254404825	0.172351185	0.149863244
PLEKHA8	1.520157847	1.461136957	1.289295891	1.302395057	1.342035789	1.350926084	1.183658409	0.935833151	0.503962549	1.059760512	0.647031179	0.642581689
PLEKHB1	0.057782079	0.074878016	0.015289337	0.014600045	0	0	0	0	0	0	0	0
PLEKHB2	45.74236704	38.60709604	44.95858839	43.99241573	32.57638857	33.41102193	39.32110925	39.72827334	38.51970456	44.00917283	41.41589818	46.01485561
PLEKHD1	0.009392487	0.019474296	0.019882277	0.01898592	0.009807436	0.009645742	0.009658783	0.009190597	0.102213749	0.092693205	0.070820724	0.068422463
PLEKHF1	0.551488911	0.620731076	0.333544839	0.207029924	0.115170597	0.12945349	0.259257009	0.231272013	0.360094699	0.362838027	0.271562887	0.54113138
PLEKHF2	8.783752937	10.27255664	8.490094211	11.20719663	11.23735973	11.38756836	10.39517319	11.20539554	13.56813578	12.29838224	11.20136803	14.16498284
PLEKHG1	0	0.007123499	0.043636405	0	0	0	0	0	0.299109984	0.218506813	0.199842421	0.278886101
PLEKHG2	19.36886883	19.97958501	19.69687942	11.46217002	21.15820869	16.97780974	17.72842199	13.07983085	14.57481066	11.92784184	14.27389235	14.81477144
PLEKHG3	10.75716811	9.67031336	10.82065186	6.964466157	6.278754002	6.266587157	8.67165624	7.0501772	2.736246528	2.503491748	2.567842723	2.968423004
PLEKHG4	1.106669997	1.702111603	1.132911024	1.017659089	1.487074745	1.704764652	1.166031016	1.349164718	0.937800813	0.994679271	1.250690841	1.085615874
PLEKHG4B	0.003683064	0	0	0	0	0	0	0	0	0.012115888	0	0
PLEKHG5	0	0	0.009498463	0	0.037482854	0.009216221	0	0	0.078129763	0.019681246	0.019333457	0.009339376
PLEKHG6	0	0	0	0.014460295	0	0	0	0	0	0	0	0
PLEKHG7	0	0	0	0	0	0	0	0	0	0	0.017741568	0
PLEKHH1	0.504932163	0.536385786	0.468448502	0.289819017	0.325456433	0.384108841	0.205135006	0.23179	2.889923252	2.419780876	2.55831894	2.925799742
PLEKHH2	0.392293275	0.572376569	0.56898958	0.242299253	1.44885629	1.335442431	1.1355401	0.462054859	0.845920549	1.744560278	0.939031246	0.680424044
PLEKHH3	8.692651387	10.99904494	9.215911035	8.637731428	10.66586496	9.422982736	8.999301167	10.78260947	3.185110528	3.947532252	4.066934877	4.370868634
PLEKHJ1	20.95111922	23.96239837	24.96590145	28.10872037	22.82319807	24.2028961	24.19324721	32.67626218	16.56759264	14.25411953	16.06595461	19.61676586
PLEKHM1	5.38496873	4.634117194	4.578307103	5.483128109	3.46087216	3.733480646	4.794889267	5.347747083	7.240088755	4.690429758	5.705402597	10.14745147
PLEKHM2	19.71739474	21.16120734	19.60129116	18.971852	16.99863298	18.09620092	20.39960775	20.14923949	23.71270472	21.35821743	22.42596638	27.4135304
PLEKHM3	1.866124812	1.842478973	1.561295007	1.963925498	1.085630646	1.308047847	1.334184935	1.281107251	1.244274316	0.919202793	0.906150162	1.134404645
PLEKHN1	0.066267932	0.085874572	0.087673617	0.033488404	0.086494456	0.153123192	0.136293517	0.081054385	0.16226118	0.090831857	0.089226761	0.086205201
PLEKHO1	31.88176083	33.38671308	36.66869763	26.58830465	29.8313093	27.36471357	30.89131444	29.20419178	3.552182561	1.665588946	3.376747901	3.245581825
PLEKHO2	11.71344915	11.70851896	12.25992417	10.40640689	8.908952215	8.168033381	11.62915917	10.62680709	15.09200982	12.01970324	14.43980094	19.38365539
PLEKHS1	0.022171224	0.011492402	0	0.011204196	0	0	0	0	0	0	0	0
PLET1	0	0	0	0	0	0.035860129	0	0	0	0	0	0
PLG	0	0	0	0	0	0	0	0	0	0	0	0
PLGLB1	0.2680649	0.074107103	0.13240434	0.090310808	0.093302555	0.073411436	0.036755342	0.017486858	1.303023322	0.352732892	0.96249923	1.320465565
PLGLB2	0.285461861	0.129472431	0.226602578	0.126225524	0.167666176	0.164901899	0.256860859	0.157120807	0.349485619	0.215201631	0.307489144	0.352778209
PLGRKT	5.374361777	5.537394433	5.513810935	8.797600803	5.060943259	6.602812145	6.679551515	7.87212899	10.90791063	14.46185646	11.08091393	11.87231556
PLIN1	0	0	0	0	0	0	0	0	0	0.019714124	0.038731509	0.037419912
PLIN2	6.794366004	10.38885037	8.081137888	5.856510977	8.185998956	7.934356382	8.038554793	6.248088201	29.89735337	43.33133741	40.90118531	16.69561764
PLIN3	46.77292598	43.697562	46.90085879	36.28002059	37.77001464	36.39160968	52.45017195	44.50747531	51.80153509	46.29573987	52.80955222	49.46569397
PLIN4	0.113882668	0.118061633	0.040178329	0.031972468	0.033031628	0.032487042	0.032530963	0.024763282	0	0.02081283	0.01363003	0.026336931
PLIN5	0.881401488	0.616777758	0.746309993	0.200436739	0.207076653	0.271550165	0.385216156	0.107806979	0.191837042	0.120811577	0.142412048	0.321042008
PLK1	32.11558771	27.51232704	37.06808164	66.01695477	69.98057133	62.46834447	50.30469807	78.5516019	70.54251169	75.64065381	82.55151733	49.3587132
PLK2	2.283650021	2.297302717	2.076869736	1.094200186	0.777183005	0.694881592	3.548687283	1.423499477	2.945401173	1.354077123	1.803900868	2.218126769
PLK3	7.800985595	7.595755457	8.439138599	4.55206257	3.71278405	3.208957346	5.828253565	4.533592146	3.283202238	3.14280408	3.38903033	4.216176605
PLK4	9.396502323	9.754178604	11.16721202	18.84348864	18.92335087	17.85926239	12.11374263	16.9558219	21.42408205	21.23340807	21.11223301	18.09791402
PLK5	0.167023986	0.144294159	0.11785366	0.028135111	0.087201446	0.085763774	0.085879723	0.081716909	0.060587954	0.030524767	0.059970723	0.057939885
PLLP	0	0	0.03804461	0	0	0	0	0	0.469404866	0.512396036	0.464622875	0.18703707
PLN	0	0	0	0	0	0	0	0	0	0	0	0
PLOD1	25.90651808	28.74334457	27.45389907	25.01197012	22.67982985	23.24841554	24.43887127	24.62493326	39.72219293	51.76565697	43.37588894	36.7964039
PLOD2	0.025690886	0.026633622	0.027191588	0	0	0	0	0	4.72492024	6.33849808	5.631514297	1.617538018
PLOD3	17.35209218	18.66341458	18.99701439	14.37726829	14.36283297	17.83852895	17.30501891	18.07567778	44.47437663	59.26203537	51.7118861	51.40913353
PLP1	0	0	0	0	0	0	0	0	0	0.055316601	0	0
PLP2	140.4485964	132.307812	147.1325427	124.0127009	155.7590185	116.368306	131.9814967	115.6959007	29.4745088	33.13019685	44.31390499	17.50966522
PLPBP	15.35070615	17.16216304	16.39220657	22.47035316	16.34317255	17.45067113	19.71130694	21.75464239	36.01638033	39.53128055	37.06424732	41.14844079
PLPP1	0	0.030600176	0.062482481	0.119331143	0	0	0.030353904	0	3.437045258	3.463229871	3.529209603	3.809030969
PLPP2	0.312442611	0.323907803	0.367437299	0.140348822	0.543743213	0.071303818	0.392701191	0.101908894	0.037779521	0.114202015	0	0.072256644
PLPP3	0	0.016863398	0.017216681	0	0.016985127	0	0	0	0.088510062	0.178368725	0.087608379	0.236996549
PLPP4	0	0	0	0	0	0	0	0	0	0	0	0
PLPP5	6.643389329	5.817795316	6.470541105	7.010961763	6.620674478	6.336584415	6.442469422	6.620971465	5.96293191	5.686668509	4.597384515	6.785656226
PLPP6	2.888134766	3.499372948	3.152367969	2.919029512	3.034577182	2.873321395	3.508335032	2.561111911	10.15586008	7.917393405	6.474755893	12.07893054
PLPP7	0.040873195	0.021186526	0.043260753	0	0.02133946	0.020987641	0.105080076	0.039994628	0.177921024	0.156866928	0.198122041	0.106340484
PLPPR1	0	0	0	0	0	0	0	0	0.043765513	0.022049467	0.043319659	0
PLPPR2	17.33734773	20.33796104	17.0408286	13.1860592	9.344284399	10.30178807	15.423883	13.61713664	11.00070706	12.58929539	10.38896915	22.34726171
PLPPR3	38.55225643	42.5109665	42.42285825	35.585801	39.92122176	44.01113851	39.84034559	45.9132607	50.63695946	28.41673548	41.92295067	42.62529092
PLPPR4	0.010054103	0	0	0	0	0	0	0	0.426713757	0.650459284	0.476516248	0.585937656
PLPPR5	0.021089383	0.010931633	0.022321294	0	0	0	0	0	0.034425783	0.011562684	0.011358359	0
PLRG1	23.47558269	22.39338774	24.0158244	29.15226429	24.94774174	23.53259552	24.7343916	27.3926938	26.82030952	23.93208989	27.70355401	29.23349305
PLS1	0.713004533	0.877762577	1.073023416	1.39622379	1.140022001	1.16699105	0.607197494	0.926604329	0.35159202	0.769622274	0.744021869	0.63766862
PLS3	0.126135867	0.03922934	0.120153547	0	0.026341678	0.012953694	0	0.012342459	13.49338178	18.14668053	16.05971255	10.842731
PLSCR1	25.00469356	19.37175308	20.03938231	20.29500859	15.02697498	14.06335949	19.14647681	16.63418881	27.18688826	16.05175416	18.38400881	23.86915036
PLSCR2	0	0.008342175	0.008516941	0	0.008402392	0	0	0	0	0.061766206	0.008667819	0.041871466
PLSCR3	4.419142679	5.039435148	4.154526559	4.282503614	2.850055003	3.470463581	3.849402983	3.866301983	1.867074863	2.422884937	2.492073249	3.165155152
PLSCR4	0	0	0	0	0	0	0	0	1.273747289	0.974047741	1.013119699	1.402963284
PLSCR5	0	0	0	0	0	0	0	0	0	0	0	0
PLTP	1.475891732	1.304305004	0.998722334	0.758066649	0.631596208	0.844809169	1.194284199	0.87597048	8.557299256	10.45311308	11.4151197	6.773248106
PLVAP	0.046709688	0	0.049438178	0.047209348	0	0	0	0	0.355822743	0.256095375	0.226412905	0.170135543
PLXDC1	0.025820327	0.044613021	0.02732859	0.008698843	0	0	0.008850795	0.016843547	0.121762211	0.084939119	0.037083624	0.098526533
PLXDC2	4.201920183	3.392083232	3.764289453	3.906698338	1.938350353	1.746042346	2.219401239	2.427575687	4.066755407	2.549195951	2.436873277	6.980002028
PLXNA1	4.660208478	4.752115952	5.118792735	3.161790452	3.530060647	4.406129495	3.494662108	3.646881488	7.115390311	6.738392228	8.920589427	10.00503886
PLXNA2	0.012800625	0.008846899	0.022580597	0.004312518	0.00445538	0.004381925	0	0.012525477	0.018573708	0.018715209	0.009192245	0.035523844
PLXNA3	7.534612874	8.843220003	6.566935559	5.312938318	6.378815061	6.028264944	5.430316208	4.933289466	3.068327857	2.777292931	3.517510139	2.378875661
PLXNA4	0.023235268	0.041293533	0.017566091	0.006709662	0.010397902	0.013635299	0.0204806	0.006495951	0.021673527	0.029118191	0.025028186	0.034543766
PLXNB1	0.614858303	0.583485144	0.595708972	0.348959946	0.844505807	0.786867741	0.924117245	0.536849757	1.950740918	1.75815091	1.808596713	3.17968609
PLXNB2	42.44516012	40.78883424	39.50087009	24.470104	32.02947096	28.55505627	40.24748363	29.09479792	65.83467556	56.90706246	62.34831956	56.65409922
PLXNB3	0.531598296	0.778545841	0.464410323	0.187623288	0.625570441	0.537266494	0.590056675	0.305497624	0.220385474	0.333096672	0.272675413	0.201971879
PLXNC1	5.586025281	4.890183225	4.687817747	7.32194897	7.403778578	5.354201485	5.25421129	4.848908597	2.415386261	2.308559078	1.797096284	5.110538982
PLXND1	29.07963766	39.48238305	25.63239255	29.45251637	31.16256029	36.87432503	27.64006965	30.45177883	28.94220434	35.70942388	25.72416	35.433882
PM20D1	0	0	0	0	0	0	0	0	0	0	0	0
PM20D2	8.955898655	9.950923615	9.982764102	10.63840364	15.56011618	15.47495957	10.52550402	9.718958858	3.773395614	5.38806313	5.452634424	2.849473028
PMAIP1	14.95327673	15.12740903	13.79692894	21.37764117	9.867516288	9.533570848	12.86199667	15.4205632	35.63100559	21.85234319	29.07066852	20.85496181
PMCH	0.036717544	0	0	0	0	0	0	0.035928303	0	0	0	0
PMEL	0.146620057	0.076000166	0.10345646	0.049396157	0.153097544	0.050191152	0.025129504	0.119557058	0.398898209	0.428732965	0.500123692	0.279740185
PMEPA1	0.195068192	0.303339433	0.216786008	0.187297119	0.122211634	0.080131171	0.10029938	0.11452513	0.053070742	0.042780043	0.073542129	0.040600978
PMF1	6.846111066	7.368567129	6.969100467	7.977079025	8.059208556	7.702430205	8.789054217	9.557743877	9.158625294	7.937379369	10.66232312	7.578457136
PMF1-BGLAP	0	0	0	0	0	0	0	0	0	0	0	0
PMFBP1	0.404823463	0.640562078	0.439746282	1.07672082	1.001150639	1.323800334	0.438211585	0.88606195	0.881098305	0.957901148	1.032776347	0.609768251
PML	4.06041693	4.047034355	3.659974974	3.306218825	3.617040888	3.934802021	4.082610348	4.00261289	6.700239444	4.868587539	5.502857669	5.974804142
PMM1	6.225308238	9.346807625	7.762845856	11.46283141	9.750501624	11.94125601	7.395191984	13.05820629	17.8322352	20.75353326	18.76818463	24.31335169
PMM2	25.98980942	23.7736894	28.53768293	22.75608432	27.28312908	25.31086006	28.6561373	25.18183529	15.50293934	15.01144436	18.93798238	15.86193519
PMP2	0	0	0	0	0	0	0	0	0	0	0	0
PMP22	1.279487577	0.982547278	1.554853563	0.455005814	1.68238763	0.608327373	2.095475314	0.69554727	2.475382069	2.520222066	2.501209581	1.824710926
PMPCA	29.90685602	25.7581959	32.4115919	23.23744881	27.09946895	25.81717825	31.02249811	25.42689039	30.35042413	29.99284949	34.58072008	28.31271813
PMPCB	12.49431755	11.72282909	12.77964849	13.49813355	14.08781195	12.80964386	13.55036794	13.20175861	15.21943384	12.96369278	14.2161681	15.67969167
PMS1	5.86418507	5.514052566	5.816038627	5.731894913	7.647501591	8.417441225	6.323772693	5.401563274	3.441918134	3.560132971	3.108641419	3.631983461
PMS2	5.166254596	5.345895524	5.427456002	4.514337116	5.694743217	5.374458564	5.450721025	4.351791725	3.869810005	3.384290798	3.572274575	4.079720275
PMVK	25.4834589	26.86929417	27.75076479	29.57552674	27.02832948	29.02118551	28.40699109	33.14941976	35.41155011	30.87736681	37.89665149	35.0123631
PNCK	0.216570988	1.13986131	0.12342708	0.016837513	1.095903503	0.479038031	0.171316309	0	1.903596592	2.155577763	1.920093589	0.01733712
PNISR	49.14298653	47.23399715	43.72876576	42.0968243	52.76711386	48.80488387	45.39815356	38.48458935	56.91985304	54.40688715	45.472575	56.16119639
PNKD	14.05899667	13.31716433	13.49040682	9.376753207	8.688833847	9.674244736	11.72753293	10.9076756	5.560704517	7.105566566	7.041501399	6.357434669
PNKP	15.07042902	15.42896103	15.22270738	16.46407486	15.76886911	13.83919677	13.24700126	17.56117423	19.87101809	16.38819927	17.47944462	22.61431417
PNLDC1	0.018008555	0.018669385	0.019060502	0.091005972	0.037608299	0	0.037038265	0.035242924	0	0.039494226	0.058194482	0.037482529
PNLIP	0	0	0	0	0	0	0	0	0	0	0	0
PNLIPRP1	0	0	0	0	0	0	0	0	0	0	0	0
PNLIPRP2	0	0	0	0	0	0	0	0	0	0	0	0
PNLIPRP3	0.063906842	0	0	0	0	0	0	0	0	0	0	0
PNMA1	7.858679717	7.978205827	7.391147933	7.716397361	6.972860367	6.648817943	6.762489302	7.829209547	13.93611188	14.64505092	13.72834903	17.07722757
PNMA2	0.086337041	0.134257814	0.228450787	0	0	0	0	0	6.999743524	5.419976625	5.998443459	4.975433651
PNMA3	0.018768595	0.029185972	0	0.009484682	0.029396651	0.038549326	0.028951082	0.027547748	0.112337082	0.092612377	0.111192665	0.097661142
PNMA5	0.069918868	0.057987652	0.044401857	0.042400079	0.043804677	0.043082478	0.100661688	0.04104958	0.13696058	0.21467288	0.060251251	0.130974555
PNMA6A	0	0	0	0	0	0	0	0	0.359883933	0.595742147	0.508882385	0.29498981
PNMA6E	0	0	0	0	0	0	0	0	0	0	0	0
PNMA6F	0	0	0	0	0	0	0	0	0	0	0	0
PNMA8A	0.042694706	0.044261403	0.060251554	0	0	0	0	0	0	0.031210989	0	0

PNMA8B	0	0	0.012061157	0	0	0	0.011718587	0	0	0	0	0
PNMT	0	0.043210009	0	0	0	0	0	0	0	0.091408791	0	0.043376374
PNN	97.88982786	90.21991063	105.9454401	108.8457465	122.174345	111.2105235	108.8442445	105.9921261	77.70417639	88.57201285	79.61295569	75.14660884
PNO1	11.81915285	9.013004525	13.21332677	12.56722463	13.48691459	11.54033673	15.64154421	12.49226103	8.466254033	8.567209127	9.579706969	9.151503703
PNOC	0.024184072	0.175500608	0.051193511	0.073328317	0.025252494	0.024836161	0.099478953	0.047328475	0.447411402	0.901639878	0.625204893	0.553696977
PNP	52.05369332	53.24952715	55.89417942	42.41189521	41.31040957	47.38567203	52.54669597	45.25846503	53.7449183	74.69987913	67.27483985	63.95578492
PNPLA1	0.123355797	0.438453879	0.102584017	0.329499138	0.018400786	0.099535786	0.099670353	0.112082539	0.306838747	0.541058615	0.436587255	0.605195239
PNPLA2	20.92370275	21.69150541	21.20809034	20.82186657	13.96258768	14.58032309	17.20940288	24.88796724	25.02751855	23.82699099	22.82025159	35.87953399
PNPLA3	0.076733777	0.099436937	0.081216087	0	0.040061889	0.019700698	0.019727333	0.018771097	12.48407762	14.61962216	14.67123337	6.24871848
PNPLA4	2.716210564	2.697319381	2.375554374	2.022827737	1.627088483	1.967295815	2.219875226	1.986375086	3.127048265	2.696267903	2.263647832	2.618439478
PNPLA5	0	0	0	0	0	0	0	0	0	0	0	0
PNPLA6	17.05895784	16.75298802	15.74470287	13.45567815	12.71690072	12.8699278	16.71720764	17.3520092	15.37328944	15.1383538	15.5855655	20.76013274
PNPLA7	0.784991595	0.546247395	0.421113691	0.304313493	0.471591835	0.41964377	0.431269294	0.231487694	2.761740341	1.792299141	1.841708809	1.891249781
PNPLA8	4.738439315	4.679842063	4.612772829	5.114314641	3.909355078	3.804851287	4.090731744	4.37899875	6.599607881	5.217273905	4.442435341	7.325820794
PNPO	11.98835747	11.46414167	10.39699707	9.678591981	6.964553219	9.729899807	10.66954088	10.13814148	6.325493607	8.014906913	8.233285478	9.013055281
PNPT1	21.06656556	15.38205133	20.3958367	19.53527517	23.0569282	21.62261602	24.84264745	18.73042605	13.49391443	13.34089799	14.43702526	11.89657942
PNRC1	23.96581473	30.99117872	22.74907158	14.89026651	22.73286789	19.1973745	21.76568488	14.6628408	58.69552443	31.53538995	37.44548881	34.83850856
PNRC2	60.991315	56.00636919	59.04029361	74.557516	78.28097004	70.93593526	71.62707587	74.07071311	134.7073212	97.06337119	105.0469283	131.2069817
POC1A	6.795824457	8.36514336	8.035041905	13.22230363	10.28678745	12.0621764	7.594060306	14.20274198	11.67354784	13.68217376	13.49182513	12.88587498
POC1B	6.662705204	7.944823551	6.593367519	7.881470852	9.40606318	10.23285005	8.188130061	8.30284933	5.104745341	7.944786663	6.340064198	5.923259427
POC1B-GALN'	0	0	0	0	0	0	0	0	0	0	0	0
POC5	5.066549646	4.837253575	5.256527638	5.687470181	6.691393957	7.198050055	5.127821661	6.172561378	10.74403451	10.67217191	9.189313643	11.29564688
PODN	0	0	0	0	0	0	0	0	0	0.014408883	0.014154262	0
PODNL1	0.060829257	0.037836845	0.064382525	0.024591982	0.012703323	0	0.025021555	0.059521737	1.482822697	0.827101788	1.035264433	1.443335851
PODXL	2.337982257	1.985631337	2.179509846	2.826501911	1.793395478	1.84694046	1.322347762	2.208536933	2.514951883	2.977827716	2.21812001	3.265978559
PODXL2	11.88730905	11.96997451	12.607474	7.164365805	12.97205401	13.63374827	12.37463691	10.79754483	13.28098089	15.7327191	16.24187169	16.78192441
POF1B	0	0	0	0	0	0	0	0.01304582	0	0.014619518	0.014361175	0
POFUT1	17.30501444	15.89415562	17.09975928	17.95995951	13.66433636	15.51976693	16.00074781	16.33227752	16.21686695	16.22500074	16.8925063	23.12978152
POFUT2	2.625996284	2.586724643	2.33429253	2.814654436	2.849347899	2.629622377	2.834990365	2.935323027	4.332375392	5.656549955	4.046648988	4.862703963
POGK	13.30012847	11.99113074	12.07705298	13.96326182	13.39308037	14.09711031	12.93313008	13.48285782	8.967565148	9.789349135	9.240677917	9.79994273
POGLUT1	6.237669653	6.352110698	6.45013034	7.509482656	6.132822501	6.768669217	6.278282281	6.416873087	4.373246937	4.27339847	4.15031485	6.066355886
POGZ	6.732160985	6.698822537	6.583779686	7.321389603	7.497479585	7.259504412	6.663996762	6.818364819	13.10303761	11.01934457	11.61290466	11.26379743
POLA1	4.833436433	5.448347117	6.107110319	7.672475166	9.266867508	8.104708923	5.327206449	7.060369029	5.788502901	5.154244799	6.370992032	4.771368516
POLA2	9.639054076	10.58430031	12.47763075	15.03547551	15.08670336	14.74379589	12.71000921	16.03217359	12.9291664	11.44110572	13.62061622	13.9864699
POLB	27.0167282	25.18409378	25.75118822	25.83484084	25.36588482	24.29620716	28.78042712	24.46425935	24.32482384	26.88340773	23.19272053	29.78582206
POLD1	17.43776569	23.03559495	22.7041493	24.49311257	30.4442503	30.31570375	22.15995159	29.80199529	23.99853726	25.10139314	28.34821369	27.88302759
POLD2	32.53709606	37.92934226	38.30517947	43.3764061	44.32730134	48.95044081	38.91651513	51.4231454	61.54695703	73.55130055	74.70842427	56.09578275
POLD3	10.38589083	11.35408244	13.01846359	15.86567958	17.77494387	17.86572603	12.49729218	15.3381715	21.06420992	20.60371649	21.16681874	21.66406568
POLD4	17.88530235	19.87966416	17.46638696	16.24411375	11.29511886	11.92944223	16.05383511	14.31341741	23.67746773	13.37791926	19.26539779	24.11374627
POLDIP2	47.40038408	44.47239808	48.83747816	49.73550493	47.60584071	49.12265097	54.04125091	55.11532322	35.4088109	37.59723378	41.31650853	37.03251987
POLDIP3	27.72924517	28.88792583	29.10884352	30.36517988	31.608036	32.03459813	29.01323815	31.42596762	46.07943816	41.63648569	43.49164663	42.30223255
POLE	16.90740889	21.01827761	19.72184634	28.1016655	31.08232886	31.94920797	18.65546376	29.46042545	26.83203624	23.77101912	29.26234644	23.59745782
POLE2	5.507865428	6.473063178	7.092233143	9.568711268	12.77454573	12.2511394	7.960955756	8.245647266	8.894225837	9.657791462	9.897234304	7.316837254
POLE3	58.17506315	56.34983369	65.8924595	89.15522789	75.36165848	78.23691907	72.02355184	91.23721707	56.98327749	64.9880631	66.65084795	67.10979146
POLE4	27.40372786	26.49944725	28.59825823	36.61882964	24.60677201	24.27991617	24.54955379	37.25513901	25.72887757	21.63213154	28.19535085	22.60725873
POLG	25.94908005	24.29268069	24.31504759	30.29821218	23.92486435	25.28215674	24.15937411	31.21532644	24.36855527	27.40625841	25.34521439	27.621759
POLG2	25.14938698	21.24276152	20.54444746	19.95942511	24.39226139	23.47009439	18.46819907	18.03544471	14.87835264	15.65799896	13.52682168	20.6570593
POLH	7.207231487	6.673802304	6.647675868	6.458418839	6.201085846	6.240343149	7.240572029	6.447961468	7.905313001	8.496921646	7.6558987	8.039396919
POLI	2.821797969	3.056419356	2.487844447	2.526706007	3.432537522	3.346435972	2.618122329	2.491215128	4.721935713	4.436513889	3.57811222	4.766754499
POLK	3.96638158	3.728269643	3.898539767	4.356446065	4.700796973	4.386318461	4.128086802	2.952381206	3.833128082	4.22994382	3.381372954	3.579507248
POLL	8.842778314	9.978394334	8.156200779	7.325958239	7.923186419	9.293459836	8.926496217	8.753820182	7.213349087	8.385258138	7.012650566	7.65087836
POLM	7.853860954	8.753327007	8.262709012	7.556475321	8.471732952	9.397790017	7.251902343	8.435084507	6.275473965	6.193972236	6.846670671	6.676178165
POLN	0.231367891	0.34265431	0.192408045	0.283952047	0.258845817	0.339437706	0.186943135	0.226394655	0.521556958	0.489286888	0.498442169	0.808337913
POLQ	5.357078821	5.909092877	6.000486459	9.591194826	8.681496477	8.444054597	6.258181462	7.680174549	10.18056853	12.37957291	11.00672243	9.62092812
POLR1A	15.16915536	12.29097167	14.93396721	13.10038848	13.65336122	13.57129938	13.71551733	12.61704416	8.924519058	8.788870256	10.28389471	9.50518606
POLR1B	20.58919607	17.28051008	20.52949093	17.16334263	22.8360953	22.73068281	20.65015168	17.82202134	8.160280372	7.268344079	9.319787016	7.467876407
POLR1C	20.26897688	17.90383669	22.19582702	20.20634723	21.25326731	20.4005013	22.94331664	22.10174304	16.78052821	13.66662584	17.57178921	19.21227043
POLR1D	49.6942767	50.15199717	49.11883614	38.39129096	54.53288186	52.78247927	52.06224873	44.17921084	23.87437055	25.22497874	22.4671363	21.9990172
POLR1E	26.11874761	22.83982612	26.3608658	27.1925843	29.47696406	30.39786494	28.10638535	27.66696025	21.55608628	22.95161807	23.65882562	23.83926411
POLR2A	53.52206543	45.70025998	51.55164933	59.55087176	50.04110694	47.15755963	54.4071664	58.08388487	64.29415045	52.20021898	51.7681111	66.01354311
POLR2B	63.86301658	56.55027823	65.86724695	60.95311461	65.91271618	58.79356863	67.12812338	53.07684487	56.2980165	53.08018426	55.72448895	55.97237793
POLR2C	33.22829867	32.87057725	37.28449818	38.91976312	31.36369524	37.49379229	40.15173767	40.65716694	41.22449361	42.94497978	40.1940639	38.30523346
POLR2D	22.56529972	22.217676	25.79409837	32.81823292	29.9662926	30.56556977	29.05988816	31.75028194	34.12750035	32.83942383	34.40307572	36.4655195
POLR2E	47.92386146	47.59308228	55.31045715	58.53120399	47.3220601	49.41753968	57.16928017	68.72629491	31.34623306	29.49240852	33.71653627	35.00627219
POLR2F	9.504450507	10.29339787	10.63003354	11.53724821	9.020581631	9.324678289	11.57084391	11.77699793	9.276907492	9.061066288	10.34339355	8.514551841
POLR2G	67.18159032	67.38082083	70.15330648	67.51038253	64.98067034	63.1170829	74.04466908	71.83947927	78.84725891	81.84477732	76.31278068	85.6375036
POLR2H	26.61175807	27.86444495	30.67555745	28.00028482	30.62458678	33.37512844	30.21519315	31.77421764	17.7124233	22.05361516	22.66819103	19.93228132
POLR2I	27.58357076	26.61260852	31.74203178	36.79730444	28.48000573	32.63662404	29.31748573	43.77680394	15.37825788	17.9990408	21.13741028	20.99958706
POLR2J	20.87033843	21.30050161	21.71558456	26.80581888	21.02394435	22.55158758	24.42859982	28.16989272	27.67742566	25.7579271	31.29551505	28.61212884
POLR2J2	0.568696919	0.505341817	1.003194437	0.821114775	0.50898962	0.584031017	0.334183198	0.450477998	0.559943072	0.386037678	0.291704598	0.338191639
POLR2J3	1.95624938	1.580286734	1.183155041	1.206847462	1.883504581	1.252361667	1.515316212	1.04410906	1.188859799	0.612887741	0.766254817	1.348415411
POLR2K	60.79205795	54.63511816	64.75372869	74.10049233	65.73450386	63.62635703	77.33246428	78.19312322	61.93545829	75.9582574	63.99067592	67.36015844
POLR2L	53.68670798	44.19426993	63.71429872	54.3851686	36.81646394	37.40805015	53.88230388	67.91987329	59.56096694	52.60549813	64.5783365	71.85246837
POLR2M	0.424779445	0.336751141	0.42314581	0.45457768	0.391363817	0.3720811	0.475365965	0.378973944	0.516650515	0.424689019	0.444099424	0.403056853
POLR3A	16.34156219	13.89768699	15.96566288	14.44260242	14.96338707	14.76666059	16.23776516	13.75245287	9.346374413	9.382006773	9.653004151	9.638393741
POLR3B	7.719025815	6.087569999	8.196221413	8.672161716	8.483800132	7.910147217	8.304107847	7.990732235	1.703480158	1.271450271	1.802103146	1.956556878
POLR3C	16.05978356	15.26083329	15.53916031	17.02191562	16.00379659	16.53296553	15.88184655	16.9579731	23.5190384	17.73882644	22.59506195	25.4309259
POLR3D	26.38872192	22.09386676	25.21393221	20.89134781	25.02045621	25.98301499	24.21092882	20.58077136	13.32663289	17.86342904	16.07543362	13.64410703
POLR3E	10.49026348	8.098983655	9.98152039	9.940432714	11.31581605	10.00149008	10.51031384	10.84918018	6.01726451	6.802509323	5.904083312	6.907132349
POLR3F	6.784079747	6.93700997	7.327401725	8.330946653	7.30138261	6.871890071	7.262145526	8.178875699	4.384310124	4.54465706	4.888336453	5.349292402
POLR3G	4.679092249	3.845140944	5.773792039	3.321936067	7.126130558	5.848349698	6.900759006	3.472971518	1.838095874	2.915804713	2.163575789	1.270812763
POLR3GL	29.5223109	30.51667263	30.52015075	33.95820336	27.91417508	30.14206831	33.88948081	33.33845701	49.17212495	48.74683559	44.09526738	60.46452075
POLR3H	8.437897564	7.449234107	8.680898943	9.534523072	8.124683968	7.853689743	8.962143748	9.974123965	7.149424746	5.999490944	8.768338612	8.38594727
POLR3K	35.85127295	29.11064603	42.23076381	38.67683107	37.02600929	35.20842324	42.03861055	40.83157913	26.294363	29.35897267	32.91999674	32.07703914
POLRMT	22.94260638	19.34861244	24.37215543	20.57048229	22.79511501	22.1880209	22.84041196	27.07707483	7.842486952	9.723451697	8.550981131	10.31211739
POM121	13.90072244	12.86320947	13.76880427	14.02002815	13.18886662	13.1406603	13.17836281	13.64461131	11.79393468	10.57635606	10.3007063	12.77654574
POM121C	21.65786625	20.89168182	22.32051504	22.90097187	22.70096215	22.3078391	20.5441606	23.06092254	22.80984489	22.68159963	22.49835793	24.07732897
POM121L12	0	0	0	0	0	0	0	0	0	0	0	0
POM121L2	0	0.070390059	0.053898531	0.017156205	0.070898169	0.104593928	0	0.04982927	0.184726363	0.167520306	0.109706695	0.176652678
POMC	0	0	0	0	0	0	0	0	0.197033166	0.11912054	0.312041473	0.414527515
POMGNT1	9.07508295	8.064082563	7.631734365	8.480199543	6.038483773	6.9112972	8.478534115	8.495166562	12.41232141	16.61195163	12.92919432	21.02611605
POMGNT2	0.058067871	0	0.040973224	0	0	0.019877863	0	0	4.486657825	6.240030956	5.316631091	7.312086431
POMK	5.371009772	4.502600088	4.45656391	3.853539365	4.015815552	4.800816179	3.648097944	2.660221887	4.004908524	5.235138574	4.071247207	4.002385869
POMP	96.79832084	84.24852306	104.673135	94.85785221	94.19551535	88.86274835	121.2301934	96.37430656	57.79724004	62.15235054	63.512072	58.41198408
POMT1	7.264001314	9.090671678	7.412642385	8.209449975	6.507112211	7.667908736	7.469950056	7.202256062	5.763442655	5.807350577	4.75913594	5.983388976
POMT2	3.452735889	3.887817366	3.744378317	5.057337495	4.048999309	4.898706001	3.801902946	4.709136531	2.497252424	3.36668592	2.918111438	3.117806411
POMZP3	2.808359813	2.803583436	2.862317617	3.924702889	3.294457909	2.49241759	2.388824902	3.630066579	2.490235159	2.243078664	2.091401678	3.066950001
PON1	0	0	0	0	0	0	0	0	0	0	0	0
PON2	16.01177768	15.11089607	16.38548696	10.0000571	10.2661494	11.57136052	14.73250699	9.925871059	3.770282212	5.407593473	5.782722175	4.092726945
PON3	0	0	0	0	0	0	0	0	0	0.049541876	0	0
POP1	10.46389683	9.061029067	11.45175784	8.148222202	11.20484825	11.25993225	10.88635665	8.661238055	10.27401534	11.31557334	11.76242881	8.100687991
POP4	18.83542069	18.66639076	21.67016004	25.34759786	19.5375838	18.98113669	22.14259995	25.87990887	11.4678468	9.007606878	10.41254969	13.01747725
POP5	61.03152613	53.82032423	63.40617676	65.57331659	54.20882552	53.43668106	61.05739646	69.74051232	22.67602474	16.81202745	22.06243066	23.47148565
POP7	24.01800141	22.26576531	27.25422796	29.11823941	30.92685375	29.29041473	29.03315139	32.3713819	17.84393241	17.66332666	23.1971196	20.18844422
POPDC2	0.395434873	0.409945496	0.193169411	0.553382156	0.190571392	0.312382468	0.312804793	0.327406564	0.66204926	1.134058071	0.622539497	0.886359055
POPDC3	0.772225395	0.46409419	0.841024849	0.531635384	0.43238593	0.344803194	0.586957896	0.240924381	0.024358676	0.012272124	0.024110526	0
POR	21.812359	23.81819042	20.12377187	24.85308625	18.95377001	21.49386384	23.78150072	28.52823728	19.70688043	18.7708994	19.66847727	24.40288149
PORCN	0.928091508	1.842411359	1.086805368	0.878145842	0.371142156	1.013953368	1.563599239	0.830853548	1.009995626	1.559014662	1.063517498	1.500153987
POSTN	0	0	0	0	0	0	0	0	0	0	0	0
POT1	10.77217138	12.28420602	10.88563646	13.20745634	13.37717155	14.06534074	13.24034999	11.82055731	13.188168	14.01986683	11.60339158	12.19804422
POTEA	0	0	0	0	0	0	0	0	0	0	0	0
POTEB	0	0	0	0	0	0	0	0	0	0	0	0
POTEB2	0	0	0	0	0	0	0	0	0	0	0	0
POTEB3	0	0	0	0	0	0	0	0	0	0	0	0
POTEC	0	0	0	0	0	0	0	0	0	0	0	0
POTED	0	0	0	0	0	0	0	0	0	0	0	0
POTEE	0.01235865	0.012812155	0	0	0	0.025383766	0.012709042	0	0	0	0	0
POTEF	0	0	0.025747189	0.036879635	0	0	0.012507949	0.023803313	0.013236496	0	0.013101651	0.025315958
POTEG	0	0	0	0	0	0	0	0	0	0	0	0
POTEH	0	0	0	0	0	0	0	0	0	0	0	0
POTEI	0	0	0	0	0	0.013777227	0	0	0	0	0	0
POTEJ	0	0.014373646	0.029349539	0.014013184	0.043432207	0.014238716	0.042773899	0.040700537	0.030176891	0.015203395	0.014934734	0.043286961
POTEM	0	0	0	0	0	0	0	0	0	0	0	0
POU1F1	0	0	0	0	0	0	0	0	0	0	0	0
POU2AF1	0	0	0	0	0	0	0	0	0	0	0	0
POU2F1	3.756127039	3.59971521	3.386423768	3.546696842	4.491710782	4.368445622	3.555582098	2.975658711	6.193584663	5.234324207	5.118004933	5.071279919
POU2F2	4.500396565	3.861718219	4.218086886	1.874216814	2.751590501	2.288598193	3.758152272	1.883493506	2.997540769	1.373439185	2.622410816	1.512263693
POU2F3	0.02751	0.019012993	0.009705655	0	0.009575119	0.04708628	0.009429988	0.017945783	0.179626555	0.140773899	0.118531091	0.37218082
POU3F1	0	0	0	0	0	0	0.018898623	0	0.019999405	0.020151767	0	0
POU3F2	2.185560828	2.634288106	2.689475604	2.594839216	4.399259986	4.177998947	2.112132662	2.512190121	0.544461407	0.938410651	0.496368884	0.822102748
POU3F3	0.011939108	0.099017744	0.063182584	0	0	0	0	0	0	0	0	0
POU3F4	0	0	0	0	0	0	0	0	0	0	0	0
POU4F1	3.214194361	3.770579879	3.431789285	2.963614113	3.738917174	4.270850217	2.681950762	3.627906772	2.193825193	2.3187467	1.83740271	2.303334325
POU4F2	0.017131347	0	0	0	0	0	0	0	0	0.018785219	0	0
POU4F3	0.045524163	0	0	0	0	0	0	0.089091249	0	0.049919098	0	0.047376395
POU5F1	0.015260794	0.015820795	0.016152235	0	0	0	0	0	0	0.451820223	0.065753493	0
POU5F1B	0	0	0	0	0	0	0	0	0	0.073801594	0.03624872	0.0350212
POU5F2	0	0	0	0.083992538	0.043387489	0.042672169	0.085459719	0.040658632	0.045218731	0.045563223	0	0

POU6F1	1.858590741	1.838577794	1.924496813	1.299088089	3.858019881	3.467863983	2.095505733	1.818640471	0.419143579	0.918336912	0.689847992	0.321591679
POU6F2	0	0	0.009090628	0	0	0	0	0	0	0	0	0
PP2D1	0	0	0	0	0	0	0	0	0	0	0	0
PPA1	173.7858705	185.2187082	183.281224	178.4435025	197.4737254	208.4787807	198.5181023	181.7375268	73.84996782	101.9207664	87.74386936	81.97688263
PPA2	42.19853189	44.61395985	48.23795045	51.94710067	53.02985956	55.16136629	51.13459028	50.16662746	49.28274115	51.17474428	50.85940285	40.06615775
PPAN	4.959037939	3.917948347	5.079400785	4.425994769	3.987989052	3.922239906	4.708938478	5.106805757	1.545015329	1.469079555	1.550814814	1.852063172
PPAN-P2RY1	0	0	0	0	0	0	0	0	0	0	0	0
PPARA	1.311377717	1.533157841	1.160027299	0.933588681	0.919538464	0.924038621	1.038487986	0.903852634	3.312554611	3.395519897	2.386931317	4.771586328
PPARD	9.461442629	10.76169922	8.412672803	7.859184357	9.969431969	9.205159142	7.907837659	7.974308952	25.04279974	25.24408541	28.83127989	15.79609444
PPARG	0	0.023495966	0.009595279	0.018325387	0	0	0.004661374	0.004435425	0.651140589	1.222734065	0.751925058	1.792568575
PPARGC1A	0.040542993	0.126092196	0.038143345	0.081953373	0.004703792	0.069393627	0.013897489	0.013223841	0.029413942	0.009879343	0.033966674	0.032816433
PPARGC1B	4.831528273	3.804330355	4.472912878	3.915665982	3.537038643	3.604764955	4.101861832	3.378626283	2.542139799	2.247486623	2.692511285	2.580045062
PPAT	16.08619602	14.16070062	17.07199224	13.32091491	21.60680563	19.23595223	18.00671815	12.32786629	8.982200921	9.464919893	11.11337013	8.181313603
PPBP	0	0.042681042	0.130725588	0.041610688	0	0.253682289	0.338700339	0.080570663	0.044803563	0	0.044347134	0
PPCDC	2.799911192	2.60625706	2.921725681	2.69036195	2.604481505	2.460295242	2.666388632	2.681845745	2.521288093	2.324283709	2.761092516	2.349532399
PPCS	6.920521341	8.092943409	7.731707185	7.231081752	7.714993785	7.862469718	8.371614401	8.636454227	61.08686773	57.42799156	52.32578777	75.44803487
PPDPF	26.44317881	42.45335732	32.52450501	51.91917443	38.35299858	44.62823798	33.63510204	61.23483944	83.45986883	107.0900471	99.37306792	96.48829873
PPDPFL	0	0	0	0	0	0	0	0	0	0	0	0
PPEF1	0	0.017410775	0	0	0	0	0	0	0.073106438	0.055247542	0.054271258	0
PPEF2	0	0.014136878	0.028866083	0.027564708	0.02847785	0.042012513	0.014023104	0.066716838	0.029679806	0.10467071	0.058754895	0.014191307
PPFIA1	9.989302089	9.328367269	9.812894008	11.35029117	10.02317075	9.280871794	9.148961767	9.744037207	14.98989467	15.32658948	14.2739627	18.80978241
PPFIA2	0	0	0	0	0	0	0	0	0.051530751	0.038942498	0.025502895	0.018479452
PPFIA3	3.234020423	3.976177379	3.146695213	2.626360947	2.547482435	3.227993889	3.022313049	2.798088979	3.778748769	3.496463347	4.779212596	3.424647968
PPFIA4	1.744593937	3.017211253	1.653975978	0.810596397	1.908002842	1.927492946	1.41994054	0.84141127	5.488711864	8.957640113	6.546074399	2.925572465
PPFIBP1	13.92851414	11.93247435	11.40655423	8.386926281	13.80995753	10.44671029	11.78203855	7.025320493	16.90951917	13.18482578	13.03324263	16.91764486
PPFIBP2	3.264365083	3.909904316	3.220599268	4.218370785	3.019028504	4.088711212	2.901334769	3.895780527	2.055343439	2.186057418	2.072079104	2.67528051
PPHLN1	13.08714955	12.70647185	13.85975801	13.34371742	13.58503334	13.29788686	14.22520249	13.1143261	11.80704497	11.12128748	12.85461047	11.31501043
PPIA	538.4393177	519.9127652	562.5350301	565.736988	595.5840532	577.6070997	573.0076415	610.8898125	499.2621307	619.6924994	598.0874446	330.2504258
PPIAL4A	0.081529638	0	0	0	0.085131513	0	0.083841163	0.079777164	0	0.178801134	0.087820763	0
PPIAL4C	0	0	0	0	0	0	0	0	0	0	0	0
PPIAL4D	0	0	0	0	0	0	0	0	0	0	0	0
PPIAL4E	0	0	0	0	0	0	0	0	0	0	0	0
PPIAL4F	0	0	0	0	0	0	0	0	0	0	0	0
PPIAL4G	0	0	0	0	0	0	0	0	0	0	0	0
PPIB	118.0879079	118.6769204	133.4069041	137.4971335	159.5420868	179.704001	227.3694058	252.6623496	271.9996863	332.2726878	326.9085193	264.6874022
PPIC	6.791497973	5.145137438	6.358805679	5.852125747	1.681967273	1.341273266	3.626333817	4.259945657	39.7494965	38.8588679	34.42062337	53.05397293
PPID	23.38398037	22.20414877	27.38950551	33.44954754	28.76739581	26.57564038	31.19769007	34.45120719	25.70305176	29.34132465	28.22235625	29.70933946
PPIE	4.753711363	4.808123893	5.319725681	5.940311416	5.916645546	6.119845747	5.953030338	5.704455973	39.45141151	41.3127704	40.12789761	37.76966611
PPIF	117.2254242	96.53142756	119.5681237	95.81690994	72.47426655	72.01197186	104.8996333	97.0926513	91.65415224	78.27270517	103.4499872	81.78363264
PPIG	14.94264905	14.06231656	16.91792714	17.62092419	20.00337039	17.84692296	18.76913526	16.28352176	18.93813793	18.73977524	17.66640968	18.9275611
PPIH	14.4324074	15.40629643	18.11709464	18.96920029	20.05825278	20.38773067	20.94673959	21.22644405	72.53597174	81.35388789	82.97118085	62.47897755
PPIL1	28.84093331	26.52716438	32.39455839	32.93793746	31.15019406	33.2453536	33.48144009	35.79914125	27.37438384	30.80999858	36.13847154	35.59170082
PPIL2	12.10089603	11.53883199	11.95711486	15.28792608	14.50390518	14.01561581	10.98594498	14.78614713	11.07301409	9.81882008	9.490136628	12.03301687
PPIL3	15.21145159	18.39354154	14.92130724	16.49973365	19.97987759	19.78043696	15.04408609	17.23727098	13.63421319	19.01128713	15.97627392	12.32713295
PPIL4	19.3663455	19.15240169	19.62106475	21.56949268	23.39268846	21.72036967	22.01177949	19.92666072	20.84433618	20.6771446	18.2302596	21.45814158
PPIL6	0.1610855	0.261385967	0.303926075	0.155730016	0.153575787	0.100695877	0.237675458	0.130210287	0.259141057	0.245755562	0.233868647	0.262392341
PPIP5K1	3.934533858	4.402199493	3.693854866	5.686636618	5.009008065	4.940053324	4.312185709	5.310739256	3.444178621	3.96515216	3.373356859	4.046282949
PPIP5K2	15.41169392	14.02408966	14.57562546	14.77991601	15.56395065	14.87300659	13.93758248	11.28053091	15.94190492	14.72122952	15.18586972	13.93136831
PPL	0.008405117	0	0	0	0.008776445	0	0	0	0.009146869	0	0	0
PPM1A	4.606297081	4.301946559	4.027478207	4.509831608	4.341364968	3.866668978	4.28791944	3.899781426	5.191520334	4.409735883	4.517336886	5.056324571
PPM1B	4.555741355	4.297132907	4.514223506	5.728451883	5.317820537	4.821338741	4.567945399	5.311045729	7.646404633	7.212723544	6.506738927	8.061842411
PPM1D	5.889995883	4.856884601	5.316228644	5.463558168	6.467714367	5.100431609	6.068570106	5.785430592	6.728439332	4.705036967	6.295965718	6.492965667
PPM1E	1.248883756	1.400023487	1.233291766	1.618570259	1.509961718	1.988271913	1.185973059	1.18695663	1.098983392	1.52670952	1.435364788	1.138012047
PPM1F	26.61509835	20.48590323	25.03771436	13.85529059	14.16536484	13.32768389	22.20006238	14.45186388	14.00664621	16.33200423	15.50573984	16.39987629
PPM1G	80.87796724	79.53235769	86.27035117	92.49258691	97.75331386	96.90984849	96.82452485	104.4595672	81.65595359	82.38056436	88.57918048	92.00166612
PPM1H	0.08180853	0.07774298	0.064940458	0.117135166	0.092541287	0.098016777	0.056085309	0.106733416	2.715358176	3.057492602	2.195685974	3.788599009
PPM1J	0.816247714	0.781107888	0.830699844	0.9518991	0.983432882	0.548090896	0.548831887	0.491509249	0.375811451	0.137699823	0.202899779	0.7514438
PPM1K	2.558159451	2.633742139	2.334130381	2.680624157	2.27203448	2.439915329	2.122693325	1.910466908	3.398298852	3.765962253	2.983602451	3.060049117
PPM1L	1.849643319	1.805427979	1.56124596	2.560220352	2.141025481	2.113658013	1.608234158	1.772100709	3.441637067	3.1672244	2.46238454	4.512864255
PPM1M	13.01594717	13.1270233	12.65357482	6.633427135	10.91431448	9.894685805	11.5669817	6.897857961	6.084287079	4.846355183	5.689054281	7.405193193
PPM1N	0.563778868	0.746818859	0.430958182	0.221592654	0.654095444	0.321655754	0.611972171	0.183886828	2.147363327	2.404136324	1.990535807	1.727556251
PPME1	13.42329348	16.10781596	15.11075957	14.3314918	14.98854758	15.83708167	13.00595871	16.3425275	18.06988754	23.41882342	21.70952058	19.37957566
PPOX	2.835410749	3.420260547	2.97872529	3.046847839	3.389918507	3.518050741	2.894121415	3.413931298	2.569451436	3.097585176	2.531921629	2.830110852
PPP1CA	109.0869807	117.7664791	119.8168293	140.8659091	116.5978875	119.2714089	122.5620585	159.0798054	139.8696892	110.1179441	142.6482647	136.811681
PPP1CB	25.29688329	27.60428993	24.70134256	26.78498668	30.41765369	29.47511319	27.44676032	26.54683363	37.35553989	40.44274909	36.76060551	34.2294223
PPP1CC	113.1050255	116.1674662	114.7302335	140.3343062	149.2669384	147.328563	137.2096317	134.8064335	95.36482112	112.254776	97.082428	92.91779461
PPP1R10	23.1301126	21.07097246	21.11810522	17.15386401	15.39959905	14.39179933	21.29575795	17.63674752	22.23806749	19.10343213	22.03512538	19.14521506
PPP1R11	24.56899563	24.09539233	25.08852424	34.10002533	25.47375758	25.11300582	26.51106114	35.07373521	12.64682615	10.62458188	11.61719034	12.84433478
PPP1R12A	27.47655138	24.7157262	26.46054767	27.93785958	27.94928837	24.92916481	26.1631616	22.05511786	19.59380051	17.26113604	16.26801444	19.48430102
PPP1R12B	3.028929072	3.398386345	2.925656574	3.530891228	3.246757899	3.158578208	2.490242699	2.793662992	1.369899415	1.787321392	1.308415846	1.874553145
PPP1R12C	23.30235283	25.07933701	24.49065638	28.39401045	22.77791971	23.25971409	25.64267139	33.52739584	26.9190234	24.73606655	27.48538123	38.1699071
PPP1R13B	1.08375228	0.775142362	0.978111779	1.154782659	0.385982692	0.465896138	0.54428034	0.723412598	0.694836462	0.340852748	0.524867886	1.0404167
PPP1R13L	0.698825466	0.97144722	1.042229174	1.284183438	1.34330894	1.353783371	0.865632791	1.181116456	1.002473114	0.92303183	0.718533516	1.008244637
PPP1R14A	0.069252974	0	0.073298303	0	0	0	0	0	0	0.379693529	0	0
PPP1R14B	67.05750746	71.72244218	81.59192782	83.86446465	79.46987932	80.45519652	85.77791981	90.528895	36.90297456	65.87925079	64.59764339	27.97108118
PPP1R14C	2.046878879	2.705893062	2.122825139	2.762994269	2.524604687	2.158501445	2.542846622	2.567451956	1.151132447	1.039392859	0.799063568	1.615490316
PPP1R14D	0	0	0	0	0	0	0	0	0	0	0	0
PPP1R15A	41.43539412	43.16656333	40.05575387	30.60449479	13.22735797	13.03247072	24.31217825	24.63617922	16.63614773	13.766862	15.95588653	12.7131365
PPP1R15B	21.01194505	19.1538657	21.90174906	26.77193857	19.98258271	19.5892214	18.97571465	21.05508165	22.60380893	21.22349044	22.34839723	23.42125406
PPP1R16A	5.17314595	5.683838201	4.835760669	6.762768158	5.986487173	6.114824777	5.334970992	7.41265548	7.875131257	6.286689023	7.747277838	11.02799459
PPP1R16B	4.700945152	5.869593025	4.66261781	5.543103723	2.925109434	3.947175612	4.150137591	4.542038322	2.203978356	3.40409852	2.858276321	3.723003714
PPP1R17	0	0	0	0	0	0	0	0	0	0	0	0
PPP1R18	34.00845795	38.92867787	36.4940152	29.27243387	35.60404289	34.62447866	37.27884267	31.61918689	8.722032605	11.37332719	10.21158123	8.513211711
PPP1R1A	0	0	0	0	0	0	0	0	0.031929257	0	0	0.030533751
PPP1R1B	0	0	0	0	0	0	0	0	0.024298032	0.048966286	0	0.069708173
PPP1R1C	0	0	0.015023155	0.014345864	0	0	0	0.013888929	0.092679911	0.093385979	0.198760788	0.029543075
PPP1R2	8.209750579	8.14096635	8.374483044	8.342666825	8.634566069	8.828232463	9.130761532	7.669456392	12.4626473	12.37819794	12.06333965	12.44419974
PPP1R21	8.022029148	9.427568653	7.249824946	9.089584668	7.694426205	7.705161979	8.025579904	7.980695952	13.77841343	10.46762944	11.32896044	17.32427807
PPP1R26	0.69958259	0.758830595	0.589615889	0.59576867	0.601977258	0.618661689	0.512917771	0.475378551	2.044287294	2.443421786	1.883912362	2.777362023
PPP1R27	905.7381527	770.2258205	976.394491	712.1121826	480.9983763	489.7363043	769.9829743	754.4064123	29.82879928	73.84777766	59.12238859	23.2686288
PPP1R2B	0.184369518	0.16383002	0.195139241	0.239582241	0.385029457	0.270486826	0.27085251	0.154634151	0.200640137	0.433218608	0.255337902	0.383741842
PPP1R2C	0	0	0	0	0	0	0	0	0	0	0	0
PPP1R32	1.013955338	1.268644743	0.629108046	1.342843669	1.204785156	0.897668268	0.791016045	1.47113445	3.500558577	6.172647349	2.862306354	6.367646067
PPP1R35	18.90179291	20.57790008	19.67155753	19.90220456	24.13503385	25.95403142	16.78463996	26.27494466	22.05961739	21.2461935	22.23188633	25.86250514
PPP1R36	0.069075174	0.071609912	0.036555059	0.03490704	0.072126828	0.035468844	0.035516796	0.202771226	0.225513185	0.037871871	0.148810536	0.251599674
PPP1R37	3.741561414	3.589646135	3.456420701	4.196232893	3.889723479	3.79188874	3.307622112	4.303441534	5.679025845	4.480661547	5.037842496	6.438421733
PPP1R3A	0.012333156	0.025571452	0	0	0.012878019	0.012665702	0	0.024136112	0.402646737	0.20285712	0.371975178	0.102679622
PPP1R3B	2.219518277	2.538667205	1.689071748	1.928091881	2.11646199	1.940285404	1.839161025	1.534626	6.787048714	7.351661437	5.660824305	6.919925287
PPP1R3C	0	0	0	0	0	0	0	0	0.683027882	0.75705457	0.833819221	0.435450224
PPP1R3D	4.191743056	3.492543107	4.091529874	5.13097234	3.647440765	3.045224347	4.182866124	3.752242728	5.963954052	4.647488228	4.44830155	6.5434374
PPP1R3E	1.161185184	1.749846754	1.267025168	1.718063168	1.874976595	1.807182961	1.41569395	1.979609541	1.472098565	2.323420302	1.250786486	2.192615394
PPP1R3F	1.382285443	1.238702604	1.326645826	1.195798852	1.088630691	1.136848446	1.174524626	1.375498291	1.115456078	0.989079531	0.921128631	1.036226494
PPP1R3G	0.061992582	0.064267421	0.032806902	0	0.226559672	0.350152675	0.255000774	0.090990084	4.722440086	6.559818116	6.176794457	2.741881568
PPP1R42	0	0	0	0	0	0	0	0	0.035793556	0	0	0
PPP1R7	17.09539775	17.89596202	17.26270638	20.75756901	17.06543139	17.21311799	20.86238938	22.92528121	22.05936827	21.54942368	21.18662274	21.8604397
PPP1R8	26.5651689	25.54490285	27.79743043	34.09561061	31.02882463	28.81216756	31.47042156	32.75115528	23.940718	20.46119433	24.32684407	24.1706327
PPP1R9A	0.012822486	0.003323253	0.010178622	0	0	0	0	0	0	0.003515094	0	0
PPP1R9B	30.62291716	32.44746775	29.32539611	29.69615806	29.56312713	28.87884831	29.5196986	32.85748087	39.35360835	27.44981301	34.17055997	46.44348951
PPP2CA	42.77472606	40.60678865	47.12780244	53.07406716	48.14754786	43.73777059	52.28601649	51.08685813	63.12338105	58.4501692	67.23485036	62.81905277
PPP2CB	25.75757152	26.13641844	29.60388226	27.05455088	25.55501095	23.00181524	30.56074246	26.24628204	45.83595553	45.01704297	43.83905505	45.36763581
PPP2R1A	55.37596554	58.29376131	62.79312606	61.14958256	59.62528945	60.06810018	61.50385844	71.13283469	81.067253	85.15914167	90.2499964	82.59976511
PPP2R1B	4.911469934	4.585139379	5.155714241	6.491395803	5.628147775	5.24048348	5.646445702	5.545267213	7.126543174	8.230342425	7.618257998	8.1728406
PPP2R2A	19.22353712	18.06524518	20.68160035	20.85384217	21.45934678	20.57056933	22.67817529	20.81929569	21.83151421	24.00272859	23.44654337	19.30410028
PPP2R2B	0	0	0	0	0	0	0	0	0	0	0	0
PPP2R2C	0.017717998	0	0.009376487	0	0	0	0.009110169	0	0.163893706	0.087428279	0.229022207	0.092194433
PPP2R2D	5.756458088	5.832793931	5.954989109	6.212584898	7.091286423	7.146187813	6.646082462	6.990882814	4.531454255	4.55918185	4.44524351	5.358715446
PPP2R3A	0	0	0.02379477	0.005680506	0	0	0.005779733	0	1.492397611	1.189455217	1.186598487	1.585095215
PPP2R3B	0.279207868	0.707552999	0.558199644	1.285553135	1.765454324	0.923928105	0.654004579	1.062469006	1.839968296	1.922021995	1.704264564	0.710277183
PPP2R3C	16.66688243	13.61697351	16.3699543	17.45178297	19.21101609	17.09257427	19.10974266	17.39285921	23.01130994	19.9465667	23.39852559	19.1707942
PPP2R5A	31.59243617	34.04219901	30.43596658	38.96940192	36.70651471	32.15301059	35.04830692	36.15552472	25.75534469	23.73997375	23.7617526	25.26919564
PPP2R5B	2.674537093	2.350351408	3.074475357	2.112392987	1.793982708	1.400610701	2.713936805	1.958453226	2.602160864	3.10753782	2.728282957	4.534473046
PPP2R5C	17.44203745	19.02773466	17.97328355	18.52261992	19.48258	18.0448107	18.02183036	16.94089937	28.07587667	27.14807429	27.02563683	25.94743498
PPP2R5D	25.28908866	26.92712663	28.97825916	34.98471518	31.44920354	30.17322036	30.10565433	35.58884287	46.47966096	54.38262179	50.22464857	43.82681443
PPP2R5E	6.691162841	6.077176452	6.383704762	7.128611509	7.364762668	6.943534182	7.565354983	6.51305754	7.453215661	8.303892527	7.798666725	7.382667327
PPP3CA	26.21751426	23.19666367	25.00627772	22.77435871	25.31699972	22.09955525	25.01436923	20.57168431	29.25460665	21.94132513	25.60703407	28.18705042
PPP3CB	27.79098741	25.41209936	27.34223946	23.65081605	31.13559089	27.85031938	26.82772832	22.89750597	14.82551352	15.9546812	15.08626474	11.01894371
PPP3CC	4.996245205	5.334971713	5.728769398	5.554659745	5.321312405	5.216477828	4.881003666	4.970332138	6.669587929	6.957804589	5.866133425	7.972607077
PPP3R1	35.0092759	31.5881526	35.10606513	33.84849085	37.33968252	33.25538058	38.33582205	31.45885688	33.7230215	27.98347611	31.72397464	29.55239132
PPP3R2	0	0	0	0	0	0	0	0	0	0	0	0.049600442
PPP4C	65.10739598	67.15867679	66.64931809	65.62086628	59.70319989	55.74389461	65.79895131	72.95584079	56.66673866	58.9646675	65.68473012	61.65154677
PPP4R1	10.98076402	9.959094793	11.85113444	12.22991141	12.40919128	11.02857106	11.61920763	12.48779549	15.7853897	13.50584875	15.13117069	15.002779
PPP4R2	17.5552449	17.37453698	17.49639973	19.96468481	20.11715877	17.03779018	19.6179024	17.34334903	24.73301615	23.24183396	22.44542868	22.65833448
PPP4R3A	23.91793802	23.9177879	25.26596765	29.14454174	29.64977	26.36158543	27.17256142	26.12783811	27.6570686	23.16166538	23.76442344	26.08614365
PPP4R3B	23.40540049	22.69648089	23.227801	25.6996581	26.79889954	25.96880373	25.09070112	22.26565008	31.37459557	27.67999326	25.51451465	32.1353158
PPP4R3C	0	0	0	0	0	0	0	0	0	0	0	0
PPP4R4	0.01090587	0.011306064	0.011542923	0	0	0	0	0	0.106814818	0.083711111	0	0.090796755
PPP5C	23.91536038	25.96456071	28.39921886	28.64801395	29.46380904	31.56135947	26.82406004	33.76422538	26.30360734	28.72265784	31.39592282	30.31376705
PPP5D1	0.272575249	0.282577494	0.220615661	0.356517792	0.167421927	0.230526347	0.148395861	0.141202728	0.296630027	0.19339932	0.189981746	0.250293052

PPP6C	16.2837426	15.29434794	17.71629499	19.50735794	18.08031182	17.08734031	19.22072931	18.43015302	24.34797042	21.35882939	22.98872765	25.76572711
PPP6R1	76.78625814	75.38602093	77.65434217	85.81701473	82.97478566	86.68158266	76.84848308	93.42022174	90.48595125	88.70935357	90.72751689	105.9159816
PPP6R2	15.6059151	16.78261156	17.54532604	16.80051387	15.49610434	15.85071752	17.36419918	17.42800749	20.15348932	19.31735568	20.1327701	23.23181493
PPP6R3	16.88692383	15.3574779	16.78814284	18.04156051	17.44575526	16.43889318	17.06033237	16.0689018	21.90751954	18.46190597	21.06875338	20.47773144
PPRC1	18.16563642	14.03934328	17.58122659	15.92850285	21.24093391	19.7394795	17.89809946	18.43172765	8.949368666	7.581918541	10.70916514	9.639713121
PPT1	46.60062448	54.53854322	50.20837299	70.69204531	46.73272007	51.36049797	49.51041337	66.2780282	1242.051452	1622.455609	1283.70845	1266.606672
PPT2	8.315367601	10.71334182	7.783631567	8.137128844	10.69029755	11.82213473	8.032125753	9.571121853	11.97841971	11.01555537	9.552420121	13.10559312
PPTC7	18.7531306	15.28332101	17.34742251	18.00057051	19.45711258	16.35326624	17.89140702	15.80436406	13.42451582	13.70508534	12.13179091	14.21406377
PPWD1	18.71431946	16.7132454	17.93650887	19.39094491	20.08253512	18.71061519	18.27539053	18.49656089	24.34156196	24.78545554	21.35163941	27.22679707
PPY	0	0	0	0	0	0	0	0	0	0	0	0
PQBP1	31.37752946	30.12200988	32.30697112	30.60891151	27.84383866	29.27822519	30.37114227	34.1776305	26.9382845	22.57591227	26.40641068	28.35477238
PQLC1	12.12361391	13.64126577	13.76524828	14.52331207	10.35084599	11.5931032	13.56775751	15.7636608	15.11304668	12.75247514	14.08509932	18.33180736
PQLC2	13.58554163	12.19826386	13.86744287	11.71844275	7.743117521	8.827199792	10.17048083	10.70377026	4.146427522	2.31812526	3.812921575	4.5365333
PQLC2L	0	0	0	0	0	0	0	0	0	0.025609537	0	0
PQLC3	3.695535541	4.240833388	3.380087656	4.425946631	3.45730599	3.597743233	3.36096891	4.065500023	8.031710129	8.385695124	6.902942079	10.40392404
PRAC1	0	0	0	0	0	0	0	0	0	0	0	0
PRAC2	0	0	0	0	0	0	0	0	0.492086194	0.413195899	0.446483712	0.117644745
PRADC1	15.59012738	15.52551938	17.10083512	24.49481255	14.30568183	15.81642624	15.93497372	25.33257658	13.9326494	14.40141885	16.02979513	24.72997903
PRAF2	1.974662784	1.748584854	1.611049624	0.997893002	0.902081629	0.718216969	0.80379831	1.127126906	9.580632272	11.54825675	8.463826758	15.62660403
PRAG1	6.982697397	6.754990337	6.381840667	5.63215283	5.554704293	5.443150175	5.850546396	5.947601699	6.688743623	4.404266511	6.473944128	4.453192751
PRAM1	26.29031562	49.16373858	23.38966182	41.60953356	14.39041988	23.16792472	17.26403076	31.52304192	3.892738948	2.622951388	3.309395804	8.975353688
PRAME	1.22360669	3.03535692	1.541764518	0.32389651	1.262453786	1.780182612	1.66275139	0.44186269	43.38764201	45.0599188	47.6528679	32.71402915
PRAMEF1	0	0	0	0	0	0	0	0	0	0	0	0
PRAMEF10	0	0	0	0	0	0	0	0	0	0	0	0
PRAMEF11	0	0	0	0	0	0	0	0	0	0	0	0
PRAMEF12	0	0	0	0	0	0	0	0	0	0	0	0
PRAMEF13	0	0	0	0	0	0	0	0	0	0	0	0
PRAMEF14	0	0	0	0	0	0	0	0	0	0	0	0
PRAMEF15	0	0	0	0	0	0	0	0	0	0	0	0
PRAMEF17	0	0	0	0	0	0	0	0	0	0	0	0
PRAMEF18	0	0	0	0	0	0	0	0	0	0	0	0
PRAMEF19	0	0	0	0	0	0	0	0	0	0	0	0
PRAMEF2	0	0	0	0	0	0	0	0	0	0	0	0
PRAMEF20	0	0	0	0	0	0	0	0	0	0	0	0
PRAMEF25	0	0	0	0	0	0	0	0	0	0	0	0
PRAMEF26	0	0	0	0	0	0	0	0	0	0	0	0
PRAMEF27	0	0	0	0	0	0	0	0	0	0	0	0
PRAMEF33	0	0	0	0	0	0	0	0	0	0	0	0
PRAMEF4	0	0	0	0	0	0	0	0	0	0	0	0
PRAMEF5	0	0	0	0	0	0	0	0	0	0	0	0
PRAMEF6	0	0	0	0	0	0	0	0	0	0	0	0
PRAMEF7	0	0	0	0	0	0	0	0	0	0	0	0
PRAMEF8	0	0	0	0	0	0	0	0	0	0	0	0
PRAMEF9	0	0	0	0	0	0	0	0	0	0	0	0
PRAP1	0	0	0	0	0	0	0	0	0	0	0	0
PRB1	0	0	0	0	0	0	0	0	0	0	0	0
PRB2	0	0	0	0	0	0	0	0	0	0	0	0
PRB3	0	0	0	0	0	0	0	0	0	0	0	0
PRB4	0	0	0	0	0	0	0	0	0	0	0	0
PRC1	28.40605031	30.09544622	33.86815866	58.32340495	57.5430317	53.64941698	39.63663279	60.22900798	71.05925175	82.36566739	76.78562999	56.08667141
PRCC	26.46968996	25.04935739	29.72939468	33.8296611	27.95604911	27.07949512	30.63308278	36.7521004	58.88859699	54.01152582	59.72736669	68.65603994
PRCD	0	0.006716123	0	0.006547697	0	0	0	0.006339144	0	0	0	0.006741982
PRCP	11.26537718	16.40794275	10.24531812	13.32570169	8.756950006	12.65463073	10.42629552	12.96930491	15.72551799	16.15033146	13.7975521	19.91586581
PRDM1	0.985939804	1.539730867	0.99001781	1.303097768	0.798520394	0.869732376	0.799415745	0.859614404	1.464988108	0.720748759	1.068826342	0.703768167
PRDM10	3.293355031	3.045271524	3.262288	2.578738518	3.268798172	3.444099873	3.151021778	2.467091586	3.400199211	4.689395966	3.579968469	2.912620677
PRDM11	0.561176244	0.605227224	0.574796784	0.57632727	0.486731729	0.539126425	0.511931747	0.433977037	0.349675042	0.089325377	0.18524346	0.438006526
PRDM12	0.151150452	0.156696971	0.137125477	0.152767328	0.112734347	0.133050863	0.133230741	0.169030267	0.281981976	0.42619532	0.232591106	0.292129087
PRDM13	1.108436647	1.131700347	1.244286733	1.171216178	1.806254762	1.862712087	1.450734738	1.166778374	0.530021678	0.423564485	0.506531742	0.38451179
PRDM14	0	0	0.022212473	0	0	0	0	0	0	0.06903788	0	0.021840444
PRDM15	2.460967087	1.724084401	2.248885233	1.893785444	2.410546592	2.366200908	2.115864883	1.965054306	1.73664941	1.553264103	1.424417076	1.418166049
PRDM16	0	0	0	0	0	0	0	0	0	0	0	0
PRDM2	4.555476309	3.820830253	4.148011639	4.511659949	4.871466673	4.22693374	4.350352686	3.875162342	7.299229694	4.884817174	5.927845469	6.475155579
PRDM4	6.318875441	7.154445265	6.46505208	9.980423526	8.034078275	8.677787875	7.071383415	9.735275488	9.734990007	7.608208528	8.181101622	10.5086582
PRDM5	0	0.008370967	0	0	0	0	0	0	0.984172388	1.292712883	0.948053076	1.008383532
PRDM6	0.020836229	0.005400205	0.005513338	0.021059117	0.010878373	0.016048536	0	0.030582533	0.464838052	0.337004655	0.291772371	0.720992599
PRDM7	0	0.027670695	0.028250388	0.013488385	0.013935218	0.041116413	0.013724	0.013058762	0	0.029268043	0	0.013888616
PRDM8	1.169773062	1.354179766	0.990483153	0.87686232	0.885552805	1.241358127	0.962350746	0.6963159	1.442739667	1.678204125	1.396541048	1.897064145
PRDM9	0	0	0	0	0	0	0	0	0	0	0	0
PRDX1	271.123544	198.3481419	276.313939	156.6196027	183.4407276	185.0487463	278.687281	180.3792455	263.8596835	333.934994	335.9026921	199.7349448
PRDX2	54.48347636	65.35018496	64.96348079	57.79079091	56.89048686	68.67629244	58.16056662	59.75447935	11.79063172	16.48484447	18.09209247	4.80144701
PRDX3	199.99165	204.5532084	222.9454604	233.9865889	251.5110353	242.6773058	241.7129921	221.0969922	199.2492385	220.4594909	217.846231	185.4124573
PRDX4	20.6750194	20.58603623	26.37465994	26.95646924	23.90581592	24.07148774	26.42634645	30.02207491	44.87206529	46.66554353	46.76360317	37.48117325
PRDX5	71.40915204	88.57655567	75.89508757	79.80497668	74.62600216	82.98227977	79.91498831	90.00450842	89.6814854	93.95061153	98.24668885	84.21491858
PRDX6	126.3396935	132.8797821	134.8791916	145.7001463	131.9885096	143.577922	141.1212455	150.7071841	95.06073946	110.518373	98.18782015	100.3956306
PREB	36.61832535	33.68681033	37.52152305	37.38666484	34.13750092	37.40158461	37.29886662	40.64436572	49.23111085	46.28274793	51.48583464	49.01842668
PRELID1	145.1547777	129.5348498	163.6106215	134.4890108	109.0632988	111.7363868	156.3990055	151.5891456	77.00433804	81.20633453	97.66524301	87.01164328
PRELID2	0	0.007308283	0	0	0	0.003619839	0	0	0.874576353	1.209771333	0.903634578	0.652941438
PRELID3A	2.575873502	2.03674278	2.841862921	2.316609616	3.669807132	3.564467713	2.49176618	1.92241929	1.496620769	1.579833147	1.787054555	0.840551423
PRELID3B	41.59061259	35.5860472	40.77579153	37.4945739	40.73159261	38.42143471	45.90033824	36.05379993	30.04452128	34.07197582	30.2585874	31.39696357
PRELP	0	0	0	0	0	0	0	0	0	0	0	0
PREP	19.98127028	18.47732512	19.92184181	20.47101098	18.52724738	19.32303447	18.76697116	18.84139322	16.24322986	16.35967305	16.03470822	18.85344781
PREPL	10.54016747	9.505305812	10.36066592	11.22058108	11.71415159	11.17644085	11.55910176	9.692649677	11.39889618	15.59692693	10.9292029	12.9274942
PREX1	24.9893605	29.08180931	21.12847249	23.32076545	15.94125302	20.81604537	18.47552992	21.02004859	49.59597651	39.87979642	39.20989986	60.50707093
PREX2	0	0	0	0	0	0	0	0	0	0.004990643	0.004902453	0
PRF1	0.633799303	0.306626503	0.402493154	0.128115827	0.352959866	0.325444397	0.782122514	0.165380222	0.620758909	0.046332449	0.295839084	0.043972437
PRG2	0.592462782	0.8671107	0.553297746	1.056706644	0.8005891	0.787389954	1.0034875	0.920744213	1.213642633	1.528610734	1.051118978	2.466272747
PRG3	0	0	0	0	0	0	0.063676833	0	0	0	0	0
PRG4	0	0	0.011262168	0	0	0.010927518	0	0	0.023159287	0.011667861	0.011461678	0.011073541
PRH1	0.550012547	0.332614014	0.291070433	0.602220777	0.622170683	0.470702373	0.282803243	0.269095034	0.29927559	0.100518525	0.39496902	0.333894627
PRH1-TAS2R	0.156879186	0.097581554	0.29887757	0.126845874	0.458667744	0.257774734	0.193592424	0.15350708	0.068289513	0.068809766	0.168984559	0.097957257
PRH2	0	0	0	0.017118404	0.017685489	0	0	0	0	0	0.018244162	0
PRICKLE1	2.935622945	3.592839814	3.387606238	2.843399609	2.096760623	2.282019316	3.155121336	3.022132465	7.543022315	5.610948271	7.202666717	11.85958873
PRICKLE2	0.028159142	0.031846311	0.024385111	0	0.093555564	0.034176306	0.018427506	0.002504897	0.022286682	0.011228235	0.02205964	0.005328154
PRICKLE3	5.28785996	4.960733314	4.493160601	5.607882437	5.249285685	4.608225092	5.169721567	6.176243149	5.551890116	5.716686788	5.294771545	6.568728964
PRICKLE4	6.047854653	5.354733431	5.639916982	2.973672645	2.321204318	2.920814031	4.269481361	3.070887679	1.778804893	1.07541387	0.80991445	2.279420548
PRIM1	30.69574128	40.6361762	37.24135357	53.08319808	71.0982062	68.60310007	39.24936326	50.31199786	44.25914778	46.93713226	47.85210418	44.04838144
PRIM2	5.619305136	6.333773279	5.923600186	8.278986978	8.372100777	8.179849208	6.763704288	8.148140296	8.495626025	8.386660441	8.888436193	8.94852042
PRIMA1	0	0	0	0	0	0	0	0	0.023752673	0.007977876	0.015673798	0
PRIMPOL	4.390957743	5.7322558	5.42202527	5.383042755	5.561368055	6.32562106	5.163500743	5.132019459	7.234057446	8.515176049	8.211423828	8.927667287
PRKAA1	24.70104414	23.62553333	24.80625081	23.66365222	21.96689169	21.39114525	24.58701008	18.3936943	26.76774795	26.0594613	22.54880013	32.54046314
PRKAA2	0.009502792	0	0.015086829	0	0	0	0.004886108	0	0	0	0	0
PRKAB1	17.6534185	15.07282356	15.238775	18.84371494	16.97639463	16.82110915	17.84200452	18.75797672	15.31625213	11.39731244	13.52657571	20.68655301
PRKAB2	5.701618543	6.639697085	5.263322683	6.100567032	6.490667465	6.471707624	5.79273507	4.681378905	11.95237847	16.03911132	12.44015533	10.45741068
PRKACA	22.65831582	24.55154756	23.2480753	30.4971004	17.50593082	18.3662139	20.25445384	28.32311254	16.78727194	15.20464112	16.32771857	19.20196502
PRKACB	10.11553205	9.337044954	8.794857587	8.326302862	11.94372696	10.42895744	10.83406054	6.975951051	5.672211563	4.916307358	5.80214316	4.591695371
PRKACG	0	0	0	0	0	0	0	0	0	0	0	0
PRKAG1	30.33133266	31.51013478	30.17781022	35.1023769	36.24313544	35.58043677	30.88676823	35.7435563	10.79552774	11.50399751	9.888120829	12.7450324
PRKAG2	2.512572577	2.548023463	2.045201607	2.356875058	1.731902338	2.012537555	2.105325818	2.463921366	1.924142119	1.656684366	1.662787429	2.233119872
PRKAG3	0	0	0	0	0	0	0.020818348	0	0	0.011099393	0	0.010534029
PRKAR1A	36.82118361	40.64488867	34.11994055	34.93296206	33.53856863	33.36707798	34.41736872	33.03600625	56.88820323	59.32592407	51.40218661	54.34080247
PRKAR1B	2.547384237	2.265924289	3.728060494	2.574633931	2.265861546	2.083167491	3.169401784	2.584947971	3.935826746	2.879523821	4.115924602	4.811126902
PRKAR2A	11.75855356	10.33997422	11.01279189	13.60393481	10.88836359	10.53023915	11.50094341	11.7869751	19.14088054	17.87709824	17.52046439	17.91083386
PRKAR2B	13.17512466	13.42309757	11.92232298	13.35657605	8.036651604	8.645167345	12.93031859	11.59750371	11.4877137	11.97084029	10.0896832	18.82853465
PRKCA	4.894119944	6.471207352	4.584757965	5.258881789	3.734131423	4.646922238	3.880613736	4.557876754	10.46517439	9.8764303	9.257964794	14.17620121
PRKCB	28.34167162	35.06171961	27.77283121	29.14024382	19.69412495	28.08533164	22.91016808	24.27001712	28.23422826	32.94055039	28.01892188	37.78213701
PRKCD	84.02530605	85.78507294	88.64526543	80.53684727	58.4975496	65.50488398	76.21933595	79.40414721	63.61564529	53.21780173	65.82806585	66.18534833
PRKCE	0.660494861	0.764402023	0.617737737	0.734736133	0.672324375	0.748694212	0.670677528	0.684912836	0.915431895	0.799418512	0.727122156	0.927298707
PRKCG	0.028239077	0	0.014944314	0	0	0	0	0	0.046096765	0.12386119	0.091254322	0.058776068
PRKCH	0.623205996	0.627072561	0.620809243	0.339637138	0.37640753	0.301181107	0.590610399	0.209248608	2.553238414	2.385097907	2.494321073	2.041063532
PRKCI	8.659769623	8.463561461	7.80127164	9.47620362	8.375100205	8.304909211	8.678693406	6.953550117	8.75256504	10.38979565	8.141222095	9.562465526
PRKCQ	0.907078433	1.059260594	0.99316999	0.958932444	1.27375614	0.845877975	0.782690807	0.80596422	2.575610222	2.400875531	2.796447241	2.224331382
PRKCSH	125.7040911	146.4759755	132.0427384	155.5231863	126.3444781	137.4651134	125.3454055	173.4950934	94.78051107	106.2248596	97.4857873	123.1814569
PRKCZ	0.478078384	0.747244933	0.467081312	0.453457529	0.407039411	0.657143233	0.453814936	0.590150372	0.616318452	0.685534694	0.681343154	0.665924576
PRKD1	0.012215564	0.006331909	0	0	0	0	0	0	0	0	0.006579081	0.006356288
PRKD2	6.406808097	6.800048853	6.565782489	8.929291797	7.353528571	7.780589016	7.006768245	9.179745122	8.646092763	8.753779138	7.394122366	11.93267346
PRKD3	17.2401213	16.6841715	17.33931741	18.04255436	20.25420582	19.28359752	16.5935636	14.51778185	19.42699753	18.43886698	17.97581245	19.30265074
PRKDC	88.18490398	82.04300317	90.76441104	98.51247876	109.977891	111.5926649	87.49829899	77.11438203	94.00358003	97.75534105	107.3250467	85.46371288
PRKG1	0	0	0	0	0	0	0	0	0	0	0	0
PRKG2	0	0	0	0	0	0	0	0	0.007339967	0.007395885	0	0
PRKN	0	0	0	0	0.02132731	0.010487846	0	0.009992964	0.288957048	0.694300856	0.220010263	1.009659402
PRKRA	8.013735235	9.947208357	8.617557457	12.8511419	10.48760737	10.27080803	9.493563353	11.91905049	12.00002408	18.23089883	13.09777974	14.92266129
PRKRIP1	13.7669578	13.50482176	13.31770677	11.96922555	13.80840168	13.85945476	14.23339256	13.85730438	14.60600268	12.58002479	13.18157039	17.1000764
PRKX	5.598529261	5.950673074	5.457506821	5.747807924	5.337930573	6.339875095	5.666306638	5.625312855	4.706605474	6.817895327	5.725671252	4.970316465
PRL	0.071035724	0.14728481	0.263148164	0.071795602	0.111260987	0.109426651	0.620922676	0.069508816	0	0	0	0
PRLH	0	0	0.215730232	0	0	0	0.209602909	0	0	0	0	0
PRLHR	0	0	0	0	0	0	0	0	0	0	0	0
PRLR	0.016521204	0.102764731	0.013114702	0.03757035	0.004312772	0.012725006	0.004247403	0.00404152	1.065267648	2.486444697	1.348050062	2.351197242
PRM1	0	0	0	0	0	0	0	0	0	0	0	0

PRM2	0	0	0	0	0	0	0	0	0	0	0	0
PRM3	0	0	0	0	0	0	0	0	0	0	0	0
PRMT1	125.9838606	116.3203362	145.0240926	108.4162287	114.2637663	119.7859642	145.6488873	127.9897552	47.088083	66.46396156	68.05704108	57.30543853
PRMT2	7.619013459	7.727790444	6.320231689	6.278327557	6.002743879	5.844328538	6.91460638	5.786419134	8.30108361	7.138728509	6.556905735	7.409983386
PRMT3	10.61148739	8.102342805	10.66025928	9.964830345	14.03389501	12.6607067	13.29994463	9.519444653	9.982127685	11.1877027	11.13090146	9.64794155
PRMT5	59.01362007	56.70106254	63.70177297	61.16252524	69.88984814	71.2935962	68.34400895	72.19650378	50.25355012	51.9446002	56.05623481	46.06637244
PRMT6	4.670263773	3.999616273	5.136496125	5.992629898	5.173425987	4.712778731	4.823556143	5.38450701	23.88734939	21.08571415	23.62212828	28.02058108
PRMT7	7.33766739	7.056424485	8.226134381	7.749561255	8.014683896	8.072587922	8.480644001	7.912110192	5.130852069	4.428857046	5.399243627	5.308508054
PRMT8	0	0	0	0	0	0	0	0	0	0	0	0
PRMT9	2.951257239	2.646338949	3.042867275	3.17139675	2.869113122	3.091798706	3.04365226	3.01229519	3.193247745	2.975791929	2.886666408	3.847993679
PRND	0	0	0	0	0	0	0	0	0	0	0	0
PRNP	16.18024837	14.91021182	14.90229904	10.56040158	12.88040074	12.22932411	15.63223069	11.14716313	12.37801067	12.31616282	13.32563151	14.59713274
PROB1	0.297930512	0.223067851	0.227741051	0.133830006	0.311092702	0.23797183	0.153188715	0.145763259	1.20682966	0.998228419	0.855786458	0.94738217
PROC	0	0.024520493	0	0	0	0	0	0	0.205919146	0	0.025477672	0.221534105
PROCA1	1.706683119	1.769310458	1.370354927	1.249094284	1.823049609	1.611679433	1.311259902	1.574021189	5.379759672	5.141102978	5.261561871	4.756742045
PROCR	7.385626001	9.738404547	7.70897862	5.228681611	2.843101765	4.788540685	6.545019857	4.762409077	7.037361696	10.44985757	9.531968976	7.154824519
PRODH	0	0	0	0	0	0.022948695	0	0	1.094319588	0.686097376	0.746184723	0.83719284
PRODH2	0	0	0	0	0	0	0	0	0	0.034851964	0	0
PROK1	0	0	0	0	0	0	0	0.03837677	0	0.043006104	0	0.040815524
PROK2	14.55283616	9.716432994	14.29365852	10.28990391	16.88417989	12.76187552	13.98005911	10.46026456	0	0	0.032254704	0
PROKR1	0	0.034799826	0	0	0.035051028	0	0	0	0	0.036808718	0.036158268	0.034933811
PROKR2	0	0	0	0	0	0	0	0	0	0.014163316	0	0
PROM1	0	0	0	0	0	0	0	0	0	0.06383452	0	0
PROM2	0	0	0	0.010814311	0.011172559	0.032965078	0.02200643	0	0.069864693	0.082129771	0.057627464	0.122487152
PROP1	0	0	0	0	0	0	0	0	0	0	0	0
PRORY	0	0	0	0	0	0	0	0	0	0	0	0
PROS1	5.825953543	5.159576	5.856406783	8.344188	6.54187971	5.30656743	5.313741638	6.302309157	18.86098378	20.78636147	16.09872128	21.3728115
PROSER1	0.041368104	0.032164591	0.032838429	0	0	0	0	0	14.58610439	13.34771259	13.31236515	14.41140218
PROSER2	2.695312685	2.606266243	2.814378232	3.286076	3.836654713	3.202425496	3.094891465	3.240544106	1.801994883	1.391612603	1.692502578	1.471669174
PROSER3	5.014511048	4.921676025	4.789246842	6.747539528	7.218926982	7.374155486	4.714245072	7.142332709	5.521622254	7.369446259	7.095394658	7.920439786
PROX1	0.191493099	0.147472004	0.069489921	0.193541525	0.085694151	0.067425064	0.129406087	0.123133437	0.101219153	0.08999142	0.064827528	0.113876764
PROX2	0.031172865	0.016158382	0.01099793	0.015753163	0.005425007	0.010671132	0.016028339	0.005083801	0.407086464	0.27915558	0.425324851	0.329818753
PROZ	0	0	0	0	0	0	0	0	0	0	0	0
PRPF18	3.363506939	2.940295155	3.067754805	3.412727257	3.122248761	3.077499671	2.98735812	2.836143735	3.995362518	4.187407212	3.217350806	4.420606926
PRPF19	82.2428914	88.88285943	91.10985305	112.7894035	97.73286802	106.2719284	93.69867812	121.5673121	79.1949773	69.51476482	91.43638258	78.62809931
PRPF3	45.34206651	42.61680363	47.04076715	45.8665345	44.51347172	42.21674627	46.56741285	45.57017401	88.30449279	65.59369534	85.36636703	73.19653021
PRPF31	25.84706877	24.70331643	28.43778732	32.73431748	27.42057954	28.66651895	29.83048137	35.4033246	25.21736059	23.16981532	26.50575875	31.98491104
PRPF38A	30.20887627	30.01916753	31.64723114	38.30133428	35.92897327	33.37787096	36.57311848	34.38558584	68.68487295	59.57477109	61.32360699	68.30943371
PRPF38B	22.36810544	19.35548373	22.31734608	24.56243762	25.77785598	22.07125763	23.34297847	22.35788694	34.18127458	29.92992494	31.72640621	29.18393457
PRPF39	19.56881868	19.43108005	19.81829698	19.55059582	22.3728466	23.71883699	21.06903882	19.9926914	18.92730275	22.42495399	20.65441462	18.78345212
PRPF4	27.49499667	27.09191735	30.5234616	32.17590299	27.40125684	30.53033097	32.30841729	30.70680396	25.98856161	26.96318693	29.18625105	28.68927723
PRPF40A	27.00384842	24.87582636	27.08016452	27.93563748	30.29296004	29.51508213	29.38876503	26.10197493	23.51976507	26.56340341	24.71235427	25.22663573
PRPF40B	0.746772324	0.808969799	0.817036626	0.81412384	0.630820131	0.922012953	0.811087756	0.878506679	0.584395517	1.012081893	0.723052595	0.98672627
PRPF4B	27.8968336	24.19322457	27.0158605	25.682304	28.94754666	26.65228105	27.95650705	21.93993659	11.47246142	9.85314707	10.17717743	10.21549083
PRPF6	44.1790203	44.88274222	44.2802824	49.35177828	45.35167913	46.10087272	48.2331373	54.96211834	53.25194612	45.72315755	48.50390874	53.52490685
PRPF8	129.5191335	120.5686425	126.974645	138.7995982	124.5275065	127.1407178	120.7816145	127.6156855	82.51135358	76.078841	80.30417257	70.4764757
PRPH	0	0.030383509	0.031020033	0.029621552	0	0	0	0.028678066	0.063788951	0.064274918	0.094708666	0.03050049
PRPH2	0	0	0	0	0	0	0	0	0	0	0	0.036999603
PRPS1	23.03628232	24.37321087	28.76696607	31.0015641	35.83341749	34.294292	29.84916523	31.84574144	28.11122266	31.69158882	31.10468049	31.45392694
PRPS1L1	0	0	0	0	0	0	0	0	0.214892686	0	0.053175875	0
PRPS2	12.05418903	14.51704899	14.91086597	14.81674672	18.73630647	18.66672183	14.77056843	14.61429703	30.8468299	34.63603234	35.16495486	32.56313921
PRPSAP1	9.394279809	9.829601631	9.411198534	15.3682815	12.52397582	12.09313324	10.6716219	16.50347571	14.59795771	13.65509272	12.6136716	18.71177172
PRPSAP2	14.31377634	12.24678852	14.64976409	12.17845707	15.19284456	13.56742323	14.86132939	11.86761941	8.891941705	7.2109261	7.953032884	6.515788449
PRR11	12.2224919	11.80289512	12.98758622	20.37714532	21.36325832	18.81154321	16.60965835	18.36510189	29.22217377	36.03125119	35.80217159	17.89819661
PRR12	5.872243953	5.839085636	6.288962766	6.740476683	6.616389371	6.411961188	5.386327628	7.691642729	7.434499063	7.194206966	7.333759773	7.802003303
PRR13	162.1041164	165.0649827	168.8566562	149.0017088	151.445756	156.3015481	183.5090128	161.0430569	172.2445804	171.532382	178.4444416	150.3300983
PRR14	13.22458161	15.93832048	11.00477103	16.81382546	14.54074337	15.45277261	12.92675654	18.65600934	14.06110124	15.14180861	15.30209111	15.92673849
PRR14L	7.922089476	7.147880908	7.361816969	7.275108141	7.662555801	7.255952012	7.448964907	5.952940966	14.09094154	13.86577859	13.249206	12.7492562
PRR15	0.070500571	0.109631422	0.111928165	0.089068406	0.055211397	0.162903415	0.181248503	0.086231458	0.134263937	0.11596012	0.17086647	0.256791544
PRR15L	0	0	0	0	0	0	0.033761542	0	0	0	0	0
PRR16	0	0	0	0	0	0	0	0	0.084540313	0.198763532	0.223144191	0.026948459
PRR18	0	0	0	0.020112858	0	0.02043656	0.020464189	0	0.02165616	0.065463433	0.021435541	0.041419304
PRR19	0.076073837	0.078865394	0.053678399	0.102516812	0.132391137	0.286458551	0.234692041	0.397006057	0.055191571	0.08341806	0.136573289	0.079169037
PRR20A	0	0	0	0	0	0	0	0	0	0	0	0
PRR20B	0	0	0	0	0	0	0	0	0	0	0	0
PRR20C	0	0	0	0	0	0	0	0	0	0	0	0
PRR20D	0	0	0	0	0	0	0	0	0	0	0	0
PRR20E	0	0	0	0	0	0	0	0	0	0	0	0
PRR22	2.319377623	2.644936805	2.823090453	3.594422063	2.865849643	2.739203765	1.987613789	4.009490232	0.631015701	0.5086584	0.791143944	0.60343641
PRR23A	0	0	0	0	0	0	0	0	0	0	0	0
PRR23B	0	0	0	0	0	0	0	0	0	0	0	0
PRR23C	0	0	0	0	0	0	0	0	0	0	0	0
PRR23D1	0	0	0	0	0	0	0	0	0	0	0	0
PRR23D2	0	0	0	0	0	0	0	0	0	0	0	0
PRR25	0	0.046140713	0.047107346	0	0	0	0	0	0.145305849	0.244021399	0.191767423	0.277910168
PRR26	0	0	0	0	0	0	0	0	0	0	0	0
PRR27	0	0	0	0	0	0	0	0	0	0	0	0
PRR29	0	0	0	0	0	0	0.016652172	0	0	0.035512714	0.017442583	0
PRR3	8.773297967	10.25877209	10.12233628	10.40092384	12.08247256	11.94820726	9.217109338	11.60096834	3.956638991	5.269485841	4.937982979	4.836567643
PRR30	0	0	0	0	0	0	0	0	0	0	0	0
PRR31	0	0	0	0	0	0	0	0	0	0	0	0
PRR32	0	0	0	0	0.045754722	0.045000373	0.27036727	0	0.143057631	0.144147494	0.283200506	0.045601709
PRR34	0.820625111	0.911505256	0.403260434	0.266593964	0.887482113	0.752457224	0.843891449	0.51620518	1.49904035	0.771299011	1.262782215	0.976015666
PRR35	0	0	0	0	0	0	0	0	0	0	0	0
PRR36	0.14292048	0.074082499	0.100846005	0.072224659	0.037308631	0.036693532	0.048990852	0.093232277	0.025922203	0.039179531	0.012829062	0.049578485
PRR4	1.908140456	3.066148533	2.120582283	2.507117701	2.789415535	2.939386094	2.158463995	3.547537724	2.180360635	2.824677495	1.64430365	1.588621244
PRR5	0.121153723	0.125599502	0.091593408	0.034985634	0.036144612	0.053323054	0.142387051	0.06774259	0.828743426	0.891992793	0.950803116	1.00866463
PRR5-ARHGAI	0	0	0	0	0	0	0	0	0	0	0	0
PRR5L	0.485230971	1.045786809	0.297332983	0.116152472	0.37333421	0.458973908	0.367675534	0.149937148	1.153378105	1.694240459	1.375455711	1.049813244
PRR7	0.841111372	1.129097453	1.05573793	0.768367612	1.002129274	1.317834583	0.992484474	1.086823971	0.28164292	0.567577148	0.348467156	0.448888969
PRR9	0	0	0	0	0	0	0	0	0	0	0	0
PRRC1	11.91342812	11.60561052	11.3080824	12.60090793	12.55727487	13.70174846	12.36694259	11.01348461	14.33099099	19.9750931	14.62332282	15.79502328
PRRC2A	119.9611191	115.137978	114.1185008	140.8508609	133.2598317	137.4677413	115.0627736	149.2438697	145.4188296	150.4043054	145.2793349	156.3765626
PRRC2B	21.62208444	21.4170281	20.12036425	21.86321142	22.90239333	23.42899572	19.28877304	20.95740765	30.85133992	32.64389541	30.32745803	26.71363128
PRRC2C	67.01863915	56.27820072	60.82892622	58.0307901	60.56607923	58.44955272	60.0711071	44.25869865	51.30262368	51.42391305	49.03454214	48.80978046
PRRG1	0.008832824	0	0	0	0	0	0	0	3.018268667	3.041262893	2.683059821	2.831198431
PRRG2	0.097894895	0.067658122	0.103613307	0.065961393	0	0.167557485	0.201340817	0.191581304	0.106534124	0.071563826	0.386645688	0.16979654
PRRG3	0	0	0	0	0	0	0	0	0	0	0	0
PRRG4	3.096614575	3.249276286	2.331109118	4.956213546	3.508603658	3.905059517	3.290879321	4.383906574	9.648745523	7.451663159	7.725523559	11.78357098
PRRT1	0.161408507	0.125498586	0.064063871	0.061175668	0.147471912	0.124320492	0.082992378	0.118454282	0.329349027	0.376105873	0.173863378	0.398942286
PRRT1B	0	0	0	0	0	0	0	0.01359838	0	0	0	0
PRRT2	0.011436676	0.011856349	0.012104736	0.011559016	0.011941934	0.023490099	0.011760928	0	0.062229816	0.037622343	0.049276687	0.047607991
PRRT3	0.16328765	0.130215037	0.1994145	0.126949507	0.222963487	0.180589734	0.077500235	0.135196595	1.380575155	1.046762944	1.285331897	2.522826208
PRRT4	0.521762184	1.22292341	0.852370884	1.157862991	0.343469047	0.80374613	0.524890926	1.387355826	0.629525951	1.082078535	0.818599103	1.876860227
PRRX1	0	0.013070319	0.013344138	0	0	0	0	0	0.013720304	0	0	0
PRRX2	0	0	0	0	0	0	0	0	0	0	0	0
PRSS1	0	0	0	0	0	0	0	0	0	0	0	0.03701183
PRSS12	7.239627219	6.455325341	6.392051439	5.516237038	5.708766748	5.633908725	6.615532444	5.533237327	16.52245001	9.121101437	13.44493335	12.11129896
PRSS16	0.093192866	0.193225222	0.039454646	0.207217476	0.058386005	0.095705678	0.038334027	0.091189692	0.567935988	0.674452495	0.321228701	0.737082839
PRSS2	0	0	0	0	0	0	0	0	0	0	0	0
PRSS21	0	0	0	0	0	0.031150202	0	0	0	0.033260639	0	0
PRSS22	0	0	0	0	0	0	0	0	0	0	0	0
PRSS23	0.012959914	0	0.027433902	0	0.027064932	0	0	0	0.338487035	0.170532874	0.181479323	0.18882095
PRSS27	0.502065057	0.642956397	0.437617419	0.388039073	0.277541815	0.363954722	0.485929026	0.52017163	0.77134916	1.068685156	0.795303288	1.075719791
PRSS3	0	0.041723352	0	0	0	0	0	0	0	0	0	0
PRSS33	0	0	0	0	0	0	0	0	0	0	0	0
PRSS35	0	0	0	0	0	0	0	0	0	0	0	0
PRSS36	0.376649806	0.301727671	0.398651349	0.069214341	0.089384026	0.193402814	0.211270129	0.134019544	3.055537435	1.989966173	3.430123093	1.674800029
PRSS37	0	0	0	0	0	0	0	0	0	0	0	0
PRSS38	0.040982148	0	0	0	0	0	0	0	0	0	0	0
PRSS41	0	0	0	0	0	0	0	0	0.033848704	0	0.033503875	0
PRSS42	0	0	0	0	0	0	0	0	0	0.067704388	0.033253989	0
PRSS43	0	0	0	0	0	0	0	0	0	0	0	0
PRSS45	0	0	0	0	0	0	0	0	0	0	0	0.118390907
PRSS47	0	0	0	0	0	0	0	0	0	0	0	0
PRSS48	0	0	0	0	0	0	0	0	0	0	0	0
PRSS50	0	0	0	0	0	0	0	0	0	0	0.086060436	0
PRSS53	1.598443843	2.082372576	1.751702264	2.07304139	2.333731384	2.353363377	1.323738243	2.214634209	2.06277772	3.7381846	1.935104197	2.163995302
PRSS54	0	0	0	0	0	0	0	0	0	0	0	0
PRSS55	0	0	0	0	0	0	0	0	0	0	0	0.057054405
PRSS56	0	0	0	0	0	0	0	0	0	0.054684314	0.026858992	0
PRSS57	118.700497	138.5805917	133.6118589	126.5792097	136.5057752	149.8388872	113.082908	134.5140674	3.422235802	1.91572644	2.419551637	3.740186193
PRSS58	0	0	0	0	0	0	0	0	0	0	0	0
PRSS8	0	0	0	0	0	0	0	0.028126564	0.03128112	0.031519431	0	0
PRTFDC1	0.081365263	0.056233994	0.086118117	0	0	0	0	0	0.708366013	0.773242809	0.934866188	0.564505029
PRTG	0.007380272	0	0	0	0	0	0	0	0.020078952	0.052602995	0.043723683	0.030722205
PRTN3	61.85585034	142.2755777	71.64340527	175.4048755	57.18450814	121.9345259	54.04229012	193.8808763	222.2477401	319.8367955	292.5170091	351.8510251
PRUNE1	12.49680363	13.62486008	13.57777952	18.57527815	14.61622202	15.50447508	15.848509	17.77868906	18.00623668	18.58360282	16.35636788	21.76019488

PRUNE2	0.017543545	0.029099698	0.025995662	0.007092484	0.010991158	0.018016581	0.021649127	0.010299869	0.045820232	0.038474423	0.098265799	0.003651467
PRX	0.374149917	0.617080992	0.360004939	0.876625663	0.532744614	0.777209357	0.367268813	0.790459575	0.573737664	0.568784266	0.457978063	0.557511161
PRY	0	0	0	0	0	0	0	0	0	0	0	0
PRY2	0	0	0	0	0	0	0	0	0	0	0	0
PSAP	242.4774756	240.6075498	203.7595881	241.991465	110.7244682	130.21318	149.878818	216.3206289	156.2631943	160.1430878	144.4839567	187.1593031
PSAPL1	0	0	0	0	0	0	0	0	0	0	0.012384979	0
PSAT1	42.2653111	36.62069337	54.20107334	56.33782758	72.79876436	57.80792651	57.29740787	53.1897268	38.98692469	38.34238156	91.87546644	28.74411563
PSCA	0.42079813	0.1635898	0.612395498	0.106324865	0.494312011	0.162054131	0.540910732	0.617629658	0.686900376	0.461422282	0.566585569	0.602138697
PSD	0.18335834	0.164741841	0.142317264	0.222383697	0.063819626	0.12553489	0.050281843	0.167455928	0.093118537	0.147443916	0.092169906	0.152654881
PSD2	0	0.027211767	0.006945461	0.019897013	0.013704097	0.00673908	0.006748191	0	0.064271258	0.050369588	0.028274002	0.061462206
PSD3	0.077795746	0.296585667	0.066403299	0.071018782	0.05240817	0.126283111	0.12903453	0.063845543	3.572157459	3.318686478	2.943769018	2.444500015
PSD4	26.58599142	27.67698195	24.2293006	22.87102445	22.89952842	23.36383503	23.46827225	23.60815895	36.56449378	30.15137956	33.67382031	39.37932721
PSEN1	7.516543615	6.699898337	7.058101722	7.829933239	6.782647509	6.53554689	7.788915919	7.18580356	24.77257968	17.4684003	21.6114859	25.95175407
PSEN2	0	0	0	0	0	0.020489603	0	0	3.582540755	4.52870059	4.642094175	6.893449913
PSENEN	35.30410254	34.64389528	33.84839757	38.99570937	27.90579732	31.18202837	36.44361223	39.24796744	27.66608806	29.40368238	31.10966231	30.85081238
PSG1	0	0	0	0	0	0	0	0	0	0	0	0
PSG11	0	0	0	0	0	0	0	0	0	0	0	0
PSG2	0	0	0	0	0	0	0	0	0	0	0	0
PSG3	0	0	0	0	0	0	0	0	0	0	0	0
PSG4	0	0	0	0	0	0	0	0	0	0	0	0
PSG5	0	0	0	0	0	0	0	0	0	0	0	0
PSG6	0	0	0	0	0	0	0	0	0	0	0	0
PSG7	0	0	0	0	0	0	0	0	0	0	0	0
PSG8	0	0	0	0	0	0	0	0	0	0	0	0
PSG9	0	0	0	0	0	0	0	0	0	0	0	0
PSIP1	15.22778108	17.83745671	16.03139187	27.24385237	30.29680318	27.97404435	20.42727351	26.36617225	39.22652476	41.81701417	42.99044913	40.55274707
PSKH1	8.563773834	8.461366348	8.704073443	8.546017588	7.666971948	6.88969808	8.29789073	9.211615441	20.00165689	16.4418969	18.31596496	24.24313144
PSKH2	0	0	0	0	0	0	0	0	0.102089012	0.041146705	0.060629397	0.253830434
PSMA1	85.5145622	80.32696571	91.28503644	79.10569978	82.51838535	79.2774007	91.22097923	74.94787269	64.02407396	66.57712828	68.78197308	63.10425256
PSMA2	66.54620317	72.90748784	79.52410868	76.27278761	79.18275987	75.87947005	83.15373454	76.50118508	73.73706858	80.41660297	85.87504799	62.83641786
PSMA3	108.8053044	109.4441987	126.7456185	119.9961335	138.3839189	130.4545495	140.2230857	126.0399256	96.28667319	97.25670922	110.8125557	79.73390305
PSMA4	104.9515089	101.6914513	118.7457423	131.6876002	117.0690946	121.7138995	131.7149428	133.9815662	127.3600619	136.0192907	135.0113621	122.4678467
PSMA5	27.12759435	25.22123722	30.18615324	27.17850946	28.28243358	26.98666804	31.93515123	29.61045892	36.78076861	36.16003066	39.60116399	36.30363395
PSMA6	55.58122416	56.95646751	58.92767635	51.43255875	56.18679863	56.15081972	62.73903272	52.04433055	47.25682111	46.60415557	53.43439721	38.95405012
PSMA7	104.0735305	108.4871436	123.066572	124.6853768	94.24355468	106.1836137	120.4290572	124.2854006	127.9543311	118.0087487	145.4097391	134.4082097
PSMA8	0	0	0	0	0	0	0	0	0	0	0	0
PSMB1	222.1624836	199.2290061	242.3443322	212.7960577	228.5460839	210.6767319	250.9694255	229.4450399	208.5619057	212.7889487	234.691173	186.0116094
PSMB10	56.2700378	54.39783637	60.97231159	48.76284501	51.77451524	55.31538944	58.6354761	61.55054047	83.99559139	75.72032533	85.96308336	90.84513211
PSMB11	0.028410539	0	0	0	0	0	0	0	0	0	0	0
PSMB2	28.34828908	26.0962864	32.87183849	31.41231674	29.29922413	29.14687002	33.46829902	32.05244295	25.27937965	26.33645516	29.71045644	22.86872076
PSMB3	119.7871129	112.8296934	136.7296044	132.4784446	118.0910981	121.1425882	145.7066732	152.8654741	127.4892707	131.2031942	150.5111074	117.9775796
PSMB4	320.2977752	317.3362685	346.7039043	313.3173659	272.9159851	281.9776913	342.3601792	338.5725596	333.5604935	337.6325983	362.3076438	353.3681865
PSMB5	38.19191844	34.58110712	40.01788835	31.53425742	32.36098098	31.93461536	39.59665859	37.13273743	50.98770023	57.28802215	64.44352314	48.21624645
PSMB6	58.00571928	57.76727281	67.01095598	79.58196689	58.63530591	62.67499257	74.56286956	86.22520831	47.14208309	47.63655911	53.3088146	41.74229845
PSMB7	106.0817057	96.8939966	111.156118	110.8064322	95.71130525	105.7298626	122.1189912	117.0295629	80.07769537	82.89678603	88.5700516	79.39154229
PSMB8	56.02507014	68.99863445	66.52521719	56.13359195	62.90512988	72.99492045	69.91019518	62.42326866	81.51256539	82.66602701	81.07446423	87.20415098
PSMB9	20.38396533	24.6450843	21.8463913	25.27249724	19.0327419	23.30641478	21.59549968	26.77863626	52.85888471	60.74973275	49.9975001	73.95274427
PSMC1	32.52489012	33.48818226	37.04642822	39.17458652	35.26390505	36.01578458	41.59799287	40.70154936	45.48954128	46.41677449	49.79186352	49.33236326
PSMC2	26.77067561	25.54599966	30.64095119	31.66538321	25.76541046	26.27023669	29.90857205	30.59118733	30.13652357	29.0058887	31.29209733	32.63985661
PSMC3	56.74676397	57.82426152	66.4656367	76.23423197	69.12939089	71.57918569	71.10206615	86.30826624	75.14763229	69.85834496	88.77758759	74.49198497
PSMC3IP	6.868632181	7.350378388	8.643422104	11.64482434	12.26194252	10.66201789	8.625776169	12.29600738	8.301494089	7.462317932	9.546633544	11.56212558
PSMC4	55.91808597	58.84437916	64.5107784	66.46129015	53.69156463	55.28933028	67.76678867	70.2314138	35.64282627	37.76403269	42.30538141	41.1360772
PSMC5	65.99551267	67.10433049	70.26757902	85.47459814	68.49970063	72.51490091	75.73856398	87.26837336	65.84741249	57.89341333	69.45097442	76.88133339
PSMC6	23.9805353	22.55331114	24.9796104	29.29371056	28.46269376	25.51300428	29.59251684	25.37266793	27.63634319	29.22788021	28.13530598	28.18796767
PSMD1	32.47449842	29.39191088	38.38036497	43.10146988	32.66433089	31.93449994	38.56732218	39.95449535	40.39479122	38.01989688	42.16369488	46.44819811
PSMD10	14.56345167	14.92065771	14.94377298	17.37975845	18.34816677	15.79873339	15.29275605	16.82618993	21.91284206	27.81527685	24.59874082	22.66283263
PSMD11	20.63726563	19.47862686	24.55007895	27.09783662	20.9337077	22.73408114	25.69821385	26.65407073	20.69505352	19.89818996	21.72481974	22.38283513
PSMD12	14.54710742	14.19701532	16.22129295	16.44747079	16.86043605	15.28526769	16.74559951	16.85058504	19.35904821	18.90633122	22.30255694	23.03710568
PSMD13	58.91268044	59.01713191	66.25486887	60.71768823	58.43663883	61.63666532	67.63835062	62.25140735	55.90493245	57.60542975	59.85507859	59.42138159
PSMD14	43.90634523	43.76308208	49.75490504	57.01415462	51.0164351	46.63506375	52.8536257	53.9408858	43.75669412	46.01447714	49.72486284	42.23562842
PSMD2	69.84191975	64.08564502	80.23774149	87.72078537	66.23473798	65.92843335	77.31188384	87.40552462	89.16026565	82.04684092	90.92570574	101.5411313
PSMD3	68.86738036	68.35914319	81.48388326	80.42141529	67.82494211	71.45705388	80.05508512	89.41608404	71.0624665	66.80147731	78.15686515	78.0194992
PSMD4	156.6877521	161.4954419	175.8343946	161.1989175	148.5526152	148.5378405	177.8282157	169.4017121	227.095972	209.0713	229.1232104	242.3740886
PSMD5	13.65340294	14.21853906	15.0892139	13.09619865	15.32220457	14.24385958	15.50338755	12.89088935	6.781315401	7.341636234	7.261868619	5.326332045
PSMD6	34.30858176	32.50221831	38.87095628	41.90954772	38.10312772	37.13753452	43.99302706	43.67460518	31.79460534	31.18765884	33.16430667	33.1646335
PSMD7	63.95926845	56.28517668	74.00451223	70.9938129	63.05033571	61.54757661	73.36051598	72.45295167	64.13356417	62.00759101	68.54752379	62.16883739
PSMD8	105.3464632	106.3849234	119.0124397	122.3047879	111.0227775	113.2857354	128.1805546	139.0393117	90.06328425	92.62804966	106.6585736	93.36788461
PSMD9	17.799251	16.72100587	18.81475811	20.48693001	15.7667037	15.36579077	20.8918491	20.34928225	4.531293701	4.415293319	4.361913923	5.166565071
PSME1	70.71787298	72.08903483	70.57630578	82.36678047	70.54167075	76.22267073	73.70190088	77.65654821	77.58036604	77.04392829	77.1592106	79.89651809
PSME2	120.7750794	109.6951144	132.849982	114.7053399	99.99245591	119.8566294	144.6959479	125.8587311	141.6335598	152.4950409	166.6475006	139.086457
PSME3	52.18297546	44.66568407	55.70707043	61.60266455	49.94535528	48.91820392	58.57177883	61.37449964	43.34663806	39.02206227	45.26967172	52.15921027
PSME4	16.7866798	14.01924805	16.19353743	16.25994817	15.43235088	15.36115538	17.19380942	13.18141836	20.54150521	18.146413	19.74765974	20.00734352
PSMF1	11.27039965	11.40371579	11.00905438	14.31839188	10.51822247	11.60734034	11.99369907	14.92042909	6.703379427	6.345087624	6.323094945	7.810372733
PSMG1	31.26610309	30.18371056	32.79738257	37.71939525	40.09183855	40.12621011	38.17348369	40.76607182	22.75579938	23.54060531	26.25652305	26.07202618
PSMG2	44.19905258	46.06160868	48.40249317	53.0712905	56.52614944	55.16509971	48.84199891	51.92605442	48.80696835	52.23338057	50.71023944	52.47178959
PSMG3	16.14286826	16.22810813	19.03736326	17.34240341	19.09565324	20.59708001	19.27056895	21.76075569	12.16209954	10.39797366	14.87548618	13.23610336
PSMG4	2.080045458	2.126732589	2.012412304	1.553242727	2.254040009	2.121449592	2.065513041	1.87446663	0.8634387	0.82298875	0.862342031	0.855456079
PSORS1C1	0	0	0.062723327	0	0	0	0	0	0.064491473	0.129965582	0.063834475	0.061672796
PSORS1C2	0	0	0	0	0	0	0	0	0	0	0	0
PSPC1	9.970999389	8.610420042	10.86680107	8.875394482	10.38942376	9.376578104	10.90610599	8.78411007	10.38491393	8.516155032	10.35884643	8.852645729
PSPH	12.05732753	9.211267009	13.2889823	9.064194766	11.77494947	10.98386892	12.67243659	8.775488054	5.114810725	4.443539303	9.141490917	4.493738796
PSPN	0.314675795	0.163111467	0.0832643	0.556573363	0.410722212	0.080790144	0.242698105	0.230933896	0.428057435	0.776373347	0.254217999	1.064306557
PSRC1	3.921911594	3.87221669	3.780379844	8.446807097	7.654080161	8.007372304	3.456947581	9.091481767	26.59891329	37.11771922	34.17351639	24.70754018
PSTK	3.249822491	3.82260532	3.671172314	4.926879385	3.915456351	4.68526536	3.470498334	4.219572653	1.734303781	1.267805953	0.875147674	1.463385858
PSTPIP1	2.581200924	5.11745094	2.432856904	3.941782178	1.278761173	2.612101508	1.782505408	4.000590142	10.06726338	14.08998015	11.91299722	16.64673148
PSTPIP2	0.11871003	0.140647013	0.089745952	0.085699919	2.603044248	2.629791757	2.197362481	1.808751714	4.687613392	5.467156022	4.621591877	6.971183436
PTAFR	2.29103138	4.13489188	1.89716964	1.043792117	0.359456687	0.865539942	1.025403508	0.789852002	0.129182125	0.117149651	0.166225932	0.11118246
PTAR1	7.231654354	7.031839252	6.179594907	6.60236896	8.019680751	7.158729045	6.315280965	4.594941869	11.70256645	16.7750243	11.21803166	10.88701563
PTBP1	139.9904455	148.2901665	156.1891309	178.34931	175.5513136	170.3604234	143.4909895	189.1885362	122.6899142	112.9378238	130.9714433	123.4331808
PTBP2	5.941714469	5.783235079	5.412359346	6.416392732	7.402580381	7.489403402	6.707745782	6.240452358	13.40642602	14.86260293	13.55152143	12.48787539
PTBP3	25.17731	22.89186982	23.97975462	22.02074553	24.56424914	22.81782741	23.32552956	18.30933847	32.90037285	30.97389478	29.49792735	32.33921115
PTCD1	0.048873352	0	0.020691292	0.039516925	0.020413006	0.040152922	0.020103603	0.028693694	0.021274571	0	0.031586759	0
PTCD2	4.720136918	5.15784909	4.644797717	4.667480093	5.488136803	6.157519091	5.509902918	3.470249899	5.580943418	6.043122266	6.788307636	6.053935009
PTCD3	21.38318754	19.58325864	20.9153888	19.5686462	22.11927579	23.04297114	20.65002627	18.20255675	18.55707166	19.22417543	18.93610752	18.54433389
PTCH1	4.152675325	3.669290975	3.399981517	2.892514123	3.98851257	3.958743369	3.243350771	2.583211072	2.339025138	2.27999102	2.069836156	2.856776561
PTCH2	0.116119035	0.144456077	0.036870596	0.058680588	0.04849961	0.035775006	0.047764495	0.056811533	0.505466181	0.432919449	0.437777218	0.555880308
PTCHD1	0	0	0	0	0	0	0	0	0	0	0	0
PTCHD3	0	0	0	0	0	0	0	0	0	0	0	0
PTCHD4	0.018427231	0.019103424	0.002167071	0	0.004275849	0.004205354	0.012633119	0.00200346	0.006684478	0.004490269	0.006616381	0.00426155
PTCRA	1.707728578	1.569972287	1.398241936	0.4233576	0.471027054	0.363991044	0.894640439	0.504459194	0.561037193	0.035331961	0.798274961	0.301790473
PTDSS1	90.52938995	89.84660724	93.15101803	102.6591673	92.6654693	98.46840198	95.79482633	101.7107484	83.6220136	79.86194874	91.9517312	95.52367761
PTDSS2	6.271077823	6.501197238	6.626478484	5.462493453	6.397346825	6.948602802	6.894356742	6.550076765	4.355109017	4.829378541	4.821809352	5.753385044
PTEN	31.60195855	30.33784928	27.40623626	26.36094111	31.90745635	26.57316487	31.15530443	24.70130042	8.181370302	8.769750707	7.260748342	8.304091307
PTER	10.13576455	8.019390101	8.753469902	6.974485864	11.38719119	9.20337819	10.1682563	6.471523554	18.73864226	15.14803211	19.36272185	18.93780943
PTF1A	0	0	0	0	0	0	0	0	0	0	0	0
PTGDR	0.012652142	0	0	0	0	0	0.013010855	0	0.013768694	0	0	0
PTGDR2	0.018491258	0	0	0.074756245	0.019308178	0.11393909	0.019015522	0.054281369	0.020123112	0.020276417	0.059754334	0
PTGDS	0.463875525	0.343498286	0.631250039	0.46883766	0.069195565	0.136109504	0.068146758	0.518748062	1.153857282	0.799320342	0.92795831	0.689641611
PTGER1	0.03786739	0.039256947	0	0	0	0	0.07788201	0	0.782974584	0.581323885	0.774998149	0.827569934
PTGER2	0.021697404	0	0	0.021929503	0	0	0.044625135	0.021231019	0.070836601	0.309297124	0.233716547	0.293542615
PTGER3	0	0.005597443	0	0	0	0.005544899	0.005552395	0	0.023503214	0.017761702	0.011631889	0.011237989
PTGER4	37.86598727	41.49712079	33.06046225	28.81847835	7.616704728	11.94567647	15.77943059	17.62745095	32.77334552	24.88153477	25.14339699	28.25023122
PTGES	0	0	0.02651433	0.02531898	0.078473182	0.02572647	0	0	0	0.027469448	0.026984033	0
PTGES2	17.99684491	18.03186296	18.0903827	19.22584809	18.43498102	19.39072145	19.04472706	21.66325638	11.0289094	11.75236604	13.75399171	13.09989188
PTGES3	82.19137738	78.76444717	95.8793339	125.2604348	114.1768898	99.82120419	105.1555133	123.6276527	67.13921787	74.0295592	75.09565322	79.13864652
PTGES3L	3.299389823	4.282529781	4.201900116	2.033343791	3.361124544	4.021947632	3.282594705	1.83734047	3.123496264	3.941468675	3.669559504	1.507447925
PTGES3L-AA	0.047346732	0.073626205	0.050112434	0.071779809	0.024719225	0.048623369	0.073033658	0.046329017	0	0.051917619	0.051000179	0
PTGFR	0.026555138	0.018353059	0.009368775	0	0.00924277	0.018180773	0.009102676	0.025984337	0.048164383	0.097062633	0.047673716	0.101330463
PTGFRN	0.083907003	0.130479	0.222020822	0	0.175227814	0.258508305	0.371029506	0.14778617	3.278093604	3.845911193	3.280851151	3.326926627
PTGIR	1.694182954	2.494817607	1.528249946	0.252954273	0.683488785	1.42352523	0.831512362	0.320250369	0.6075812	0.802206163	0.891718518	0.180318458
PTGIS	0	0	0	0	0	0	0	0	0	0.010530854	0	0
PTGR1	1.589048989	2.713298334	2.011650063	3.338058988	1.789388491	2.527838	1.409813194	2.865880291	28.04150468	34.74664083	28.46063967	32.58766261
PTGR2	3.933858235	3.870140483	3.228952912	3.326806285	4.50584174	4.287271683	4.14858961	3.240482347	4.89259589	7.504845823	5.621052947	4.093914275
PTGS1	3.847890194	4.608977232	3.334307291	2.327835182	1.520437086	1.734174284	2.231853669	1.348963798	5.561196756	5.846405237	5.92796928	3.946830508
PTGS2	7.353454344	9.175265064	5.450633421	6.015897795	1.450627341	1.316018043	2.290750328	2.14455506	0.899292174	0.669758089	0.619221406	0.66057014
PTH	0	0	0	0	0	0	0	0	0	0	0	0
PTH1R	0	0	0	0	0	0.011548685	0	0	0.01223788	0.123311127	0.121132087	0.023406018
PTH2	0	0	0	0	0	0	0	0	0	0	0	0
PTH2R	0	0	0	0	0	0	0	0	0	0	0	0
PTHLH	0	0	0	0	0	0	0	0	0.092217728	0.018584055	0.127789583	0.123462139
PTK2	13.95571781	12.85842865	14.06061904	13.32048457	14.82554747	12.70450813	15.43231729	12.36611976	1.16148609	1.915093142	1.576418988	1.506206295
PTK2B	62.49826827	49.21747194	57.29460826	37.28516593	46.27660046	42.36813474	50.53552505	38.65632221	75.30376446	57.95337678	64.57147006	64.92185987
PTK6	0.466986327	0.484122555	0.112332902	0.128722293	0.243808594	0.108994987	0.240113155	0.083081544	0.485097988	0.768104281	0.548749857	0.839431225
PTK7	0.121029152	0.100376287	0.153718708	0.012232382	0	0	0	0	2.02833369	2.999322673	3.024542341	2.582045493
PTMA	708.5789508	759.8212979	802.094148	834.5171841	988.5376161	933.3587279	836.5668058	931.5205932	667.6656766	920.4227644	827.4898714	437.9567059
PTMS	10.133785	16.67011886	13.38501164	14.30520388	18.36455675	15.95457598	14.59814935	18.93046917	21.14477142	17.90794241	21.33531971	19.91556169
PTN	0.639743788	0.786037822	1.45454043	0	0	0	0	0	0	0.025981671	0	0
PTOV1	9.771413477	11.73841935	10.23344801	11.23634761	12.50979706	12.46182959	12.0877281	14.13595899	12.82534292	11.83016862	13.14846361	13.74042104

PTP4A1	38.44835152	35.65087822	40.10789279	35.84615849	37.83300537	34.91277568	41.34081898	33.58078001	42.46624632	48.65486129	45.78779286	40.78841546
PTP4A2	44.98832138	44.95088747	45.56222504	48.33627595	47.72689478	46.29892645	49.27830461	46.45143588	54.49683949	47.47136297	51.98280409	57.31236607
PTP4A3	8.790447669	8.707993306	8.492819105	4.996570921	13.08567162	9.428690361	10.92487118	6.940011776	5.968657869	7.167523832	6.895192895	5.051003721
PTPA	16.96636888	18.66487594	18.44697004	21.90228698	19.30611383	19.91461809	20.55637683	22.78315389	20.5874627	21.3504298	21.11896037	22.30564105
PTPDC1	1.57517298	1.559582396	1.598499289	1.490658058	1.70636369	1.684289824	1.625899023	1.102588456	1.566518444	1.565514592	1.474302735	1.019166452
PTPMT1	9.878178508	9.222703332	9.727701332	11.11521694	10.97078002	11.01175561	10.82468977	11.08786118	11.39107701	13.99738808	13.58080794	11.11802955
PTPN1	22.75047688	20.75069097	23.72766071	23.80305386	21.56298533	18.25984271	23.1314051	21.65540146	28.95033427	26.22232583	28.63913488	30.89214941
PTPN11	57.24172016	51.618537	57.6350097	60.0030516	58.54685396	57.63318516	58.68385803	50.27748507	40.29296375	48.17283083	44.12050653	42.60899412
PTPN12	27.40301711	29.64509722	26.5747686	28.65750467	26.17005772	27.3201257	25.68023784	26.53329385	68.72872876	55.4945743	55.65234285	75.81220171
PTPN13	11.33259132	6.968978501	10.26033666	8.708427483	10.74035018	8.087208478	10.94274247	6.431496976	1.396665619	1.202358445	2.301825212	0.966057187
PTPN14	2.60005012	3.745745614	2.274425259	2.0878913	1.27687878	2.523846788	1.92494921	1.386316713	0.938861516	1.119594659	0.801412097	0.601297288
PTPN18	16.60191412	19.00521998	16.29883357	26.51814299	21.10259158	24.1435037	17.94735617	31.15549397	24.88207216	21.59794896	21.97457885	35.69948879
PTPN2	14.93546825	14.68764813	13.92425328	15.10699128	15.43740187	15.66071361	15.45461239	13.99980584	8.493858731	7.601947063	7.943735434	8.522097878
PTPN20	0.006312712	0.00654436	0.03340731	0.038281442	0.0395496	0.006482926	0	0.006177021	0	0.013844292	0.013599649	0.013139113
PTPN21	0.029161122	0.0226734	0.038580667	0.014736531	0.045674135	0.044921115	0.037484872	0.028534306	0.17454026	0.079940895	0.188467808	0.22002006
PTPN22	7.071838437	7.780811245	6.098077568	7.468831569	5.321900891	6.639170582	6.063585093	5.43027515	2.736563132	3.13774381	2.833222041	2.757331635
PTPN23	7.844256316	7.382260709	7.18109662	8.237056963	7.595110606	7.114182474	6.757134283	8.08434776	9.955568883	8.076516973	8.954326048	9.785494818
PTPN3	1.922624075	1.705602868	1.923567407	0.507549791	1.13362397	1.404718794	1.352517213	0.482023964	0.619361176	0.461504306	0.530624431	0.47283756
PTPN4	0.707099835	0.733047113	0.600282549	0.666275079	0.642200765	0.658087728	0.677913562	0.56937668	0.917790765	0.884399287	0.912407901	0.916004163
PTPN5	0	0	0	0	0	0	0	0	0	0	0	0
PTPN6	132.1544762	108.8672966	131.1455582	86.97894864	104.8334703	91.15974236	121.471558	95.14863906	37.84073417	23.68723072	36.61553296	29.84615348
PTPN7	77.23661726	69.90653278	79.37984652	67.65211862	75.02413048	69.72412843	75.06129525	71.03912773	45.50346878	41.46116594	50.08437887	40.90980648
PTPN9	23.29701646	22.37021053	24.48464312	25.75196323	24.05564078	22.52441505	24.9253722	24.11757705	25.01157494	19.63821128	24.24253765	25.56502327
PTPRA	28.79319143	32.30567249	28.19699965	36.32518808	31.5494175	32.61409706	29.44248555	34.92975396	19.97609977	17.90170452	18.73730546	23.20256261
PTPRB	0.004075864	0	0.004313951	0.004119464	0.004255931	0	0.004191423	0.003988254	0.527831585	0.402241607	0.430256549	0.462345097
PTPRC	68.37566104	66.55453316	56.39043423	43.81876565	53.61772277	46.56384075	56.02444082	35.44054768	52.18916819	41.82202806	45.02751823	35.82126128
PTPRCAP	0.533942721	0.615039931	0.502339857	0.899423957	0.49558367	0.487413085	0.366054032	0.870776102	0.58106319	0.910762118	0.575143697	0.308703963
PTPRD	0.054290419	0.056282625	0.057461728	0	0	0	0	0	0	0.008504522	0	0
PTPRE	10.29112715	8.072160054	9.497277793	4.168349871	3.884933203	4.435221186	6.446767118	4.049859183	4.991001122	5.935635272	5.333063478	5.734522088
PTPRF	0.025854444	0.026803182	0.061570574	0	0.006749165	0	0.006646867	0.006324676	25.25918332	56.90644104	39.7899263	24.11484112
PTPRG	0.005034577	0.020877291	0.02664333	0.005088433	0	0	0.005177317	0	6.777373127	6.210696215	6.556483881	9.18993905
PTPRH	0.227347489	0.520999135	0.240627717	0.036280966	0.249885695	0.196612706	0.369147217	0.140501477	1.015686914	1.535137158	1.121340498	0.8841276
PTPRJ	9.776438248	10.83096761	8.618984469	7.749928921	6.710178945	7.656396556	6.959313287	6.583668306	25.19204095	21.20134312	20.74234962	33.0487213
PTPRK	0.012713423	0.013179946	0.013456062	0.032123549	0.019912628	0.006528111	0	0.012440148	3.991507894	5.039593934	3.745428459	4.194128986
PTPRM	0.302555867	0.33718263	0.232166244	0.05351366	0.0789806	0.225267543	0.085561828	0.066611801	0.024694233	0.008294121	0.008147555	0.023614942
PTPRN	0	0	0	0	0	0	0.028282733	0	0	0.030158126	0	0.014310989
PTPRN2	0.29713605	0.421250683	0.292989105	0.513358208	0.503846127	0.589430982	0.428306944	0.58895337	4.518725236	4.372138367	3.610980217	5.494700334
PTPRO	0.414507653	0.51505226	0.360933273	0.130733578	0.107437607	0.156989939	0.341612433	0.135199281	0.435091945	0.602809113	0.522491319	0.198859726
PTPRQ	0	0	0	0	0	0	0	0	0	0	0.012789432	0
PTPRR	0	0	0	0	0	0.013347937	0	0	0.028289013	0	0.014000412	0
PTPRS	2.48461033	2.443315093	2.674827196	1.356041803	2.045851771	2.259992369	2.029442832	1.444829695	5.894452525	6.523174895	5.941456958	5.429972381
PTPRT	0	0	0	0	0	0	0	0	0	0	0	0
PTPRU	0.261574254	0.503606654	0.415280697	0.273814217	0.146319788	0.268627229	0.1537088	0.383927602	6.658968468	6.904331304	6.661570802	12.69697255
PTPRZ1	0.110719261	0.108030253	0.1861202	0	0	0	0	0	0	0.05713326	0	0
PTRH1	1.706885356	1.990710132	2.303403526	2.415201778	1.73773604	2.23495907	2.23798062	3.173372365	2.22902168	1.778085825	2.02245437	2.486866245
PTRH2	12.78216437	10.09970531	12.46475463	9.991581242	12.4470755	12.41393753	12.38148997	11.50026148	8.570136376	8.162971718	9.48839277	7.896641873
PTRHD1	18.90860443	18.43216609	23.39843288	19.87150391	19.44927645	19.70827543	20.89579766	23.56494694	20.37254048	15.67948409	18.84768685	15.4682273
PTS	8.229697543	9.366959472	9.74593049	12.15668475	10.93689556	10.28376448	10.59357759	11.09371859	5.887140282	9.150410995	6.137121564	7.426591931
PTTG1	20.57808055	22.14460589	23.98920182	57.51643588	45.32401756	46.82067944	35.85048149	61.84325763	64.91094071	103.8119386	75.6663695	50.50082513
PTTG1IP	24.84836409	30.81352228	27.28735523	28.55894912	23.69945646	22.8785338	23.47730583	29.23299524	55.38790557	50.44393787	48.09631815	73.1988437
PTTG2	0.186838753	0	0.296629069	0.094418695	0.292639576	0.095938296	0.096068	0	0.203327281	0	0.100627958	0
PTX3	6.358302083	6.332562835	5.906867411	3.92875315	1.855497994	2.822902682	5.225147431	4.048135356	2.266186419	2.009436897	1.854295989	2.976205666
PTX4	0	0	0	0	0	0	0	0	0.074930591	0.151002877	0.259585365	0.143311321
PUDP	6.069243481	5.630845141	6.67979086	4.533949487	4.454531645	4.200427173	4.907123592	4.518641172	13.71225227	12.9968769	16.20180029	13.09005727
PUF60	26.48769404	25.98841506	29.42235491	29.42076667	26.70767698	27.88055349	28.63124249	33.42353191	29.50902304	27.52428737	32.31245368	32.87553634
PUM1	27.38941622	24.20937649	25.95396942	24.90507442	25.73011057	24.77228356	27.31306894	21.334947	30.21975792	25.94658467	26.43527283	32.01868329
PUM2	12.29694062	11.08218784	11.39259734	13.00670261	13.60202454	13.17972829	11.6077595	10.93502758	15.99141986	14.66122311	13.88283548	15.49318477
PUM3	41.19264763	37.13083528	46.82841199	44.61933499	51.28120055	50.11243205	50.70315113	43.38771906	27.72425132	31.99832182	34.35443912	24.84919633
PURA	5.641160989	4.720681247	5.680216949	6.112424503	6.84557709	6.169046281	6.313268115	5.171802555	11.94614991	11.5133205	10.93761654	11.84714142
PURB	7.704833195	7.551990961	7.810416616	7.278868381	7.952535999	7.432480021	7.46078476	5.593613633	17.8514779	17.00763941	16.17165316	18.16746416
PURG	0.047944367	0.033135801	0.016914993	0.016152411	0.033374992	0.049237118	0.098607368	0.015637935	0.034783639	0.087621584	0.034429286	0
PUS1	53.42197699	41.58696227	53.19239358	47.4863697	53.48875308	52.96070359	54.49373675	55.09700183	11.83350133	11.09726128	14.35706065	11.56279784
PUS10	1.420337316	1.337058742	1.278672881	1.336528719	1.116576247	1.341273266	1.267537978	1.18213492	1.838828764	1.843886698	1.406837467	2.268159282
PUS3	9.142813339	7.600256454	10.42586423	9.412267475	9.310279626	10.23234162	10.07152451	9.694121469	6.068372773	4.904098447	5.976062029	7.305484961
PUS7	14.41218703	11.38741556	13.96407379	14.65874307	16.70332349	16.67826977	16.79481879	12.58184357	6.31673158	5.496160578	6.76110473	5.691847305
PUS7L	3.580148871	3.842658903	3.493114739	3.847020403	4.206605311	4.424615717	3.815674776	3.071858406	4.500951658	4.984982759	3.860535185	4.559534219
PUSL1	9.36259319	9.285673524	10.83963111	11.00001526	10.48208492	10.10100091	9.732315959	11.67492695	8.018655804	6.041277022	8.009783169	8.899322491
PVALB	0	0	0	0	0	0	0	0	0	0	0	0
PVALEF	0	0	0	0	0	0	0	0	0	0	0	0
PVR	7.881242882	6.852928842	7.454210182	7.323310385	6.874750169	7.096758907	7.079125955	7.167776039	7.097679509	7.403369916	8.593960982	9.074448426
PVRIG	0.203438793	0.105452026	0.071774142	0.102807502	0.17702205	0.348207048	0.278942245	0.099532946	0.258290989	0.22307892	0.182614063	0.211716064
PWP1	35.29043097	35.89321407	39.01473376	36.44203268	39.94594297	40.19492479	38.37110181	38.29827962	19.44818027	19.91935995	18.40389861	19.1499884
PWP2	2.773844968	2.074226497	2.310197326	2.221366041	3.13379891	3.050999966	2.40045517	2.358257916	0.362896241	0.149588555	0.146945164	0.110420364
PWWP2A	2.088216222	1.954778323	2.216154252	2.628237227	2.439071279	2.29481193	2.303702596	2.47292519	4.385743939	3.351399087	3.904533175	3.877748016
PWWP2B	8.971532037	8.03893764	9.204699787	7.559558277	9.244890982	9.253757929	9.044201098	8.721061459	5.533231879	4.692795917	5.392278159	5.393545893
PXDC1	0	0	0	0	0	0	0	0	0.852790151	0.62059617	1.008233518	0.521025353
PXDN	0	0	0	0.015768388	0	0.016022168	0	0	0	0.051323028	0	0
PXDNL	0.010744721	0.05569501	0.022744721	0	0	0.03310331	0.011049355	0	0.058464714	0.058910118	0.057869113	0.055909444
PXK	29.12782259	33.93940624	26.70335341	35.44513077	25.65479984	33.93818791	25.23705477	29.3677017	19.47610482	20.34846065	20.1023687	31.89181512
PXMP2	15.0126988	20.51829764	17.49646573	30.97169999	24.83491726	27.42812729	18.38723447	30.48037857	27.10690478	23.12083634	33.2507579	24.98592456
PXMP4	0.987072657	1.00380233	0.875584339	1.168654042	1.30552834	1.496396065	1.372745255	1.526971716	2.956557703	2.618293341	2.48089053	3.277363926
PXN	2.853640756	4.416301946	3.063686789	3.436160107	5.146773485	4.83756869	5.370642404	4.656063314	6.805298588	6.857143733	6.713909597	8.141935812
PXT1	0.051052714	0.052926112	0	0	0.026654079	0.078643916	0.052500159	0.02497767	0	0.02799069	0	0.026564943
PXYLP1	0.238706901	0.573079933	0.412219524	0.457124929	0.249252667	0.658016201	0.219635268	0.57779305	1.435586503	2.23177881	2.043478228	2.732610286
PYCARD	76.22441028	80.10397812	80.97167743	78.58762397	70.28801329	75.92057826	84.11231857	98.97115764	68.63361048	54.19547967	72.13345268	70.41517422
PYCR1	22.53232772	22.20533	23.53599531	17.83760068	29.19626387	29.50801186	26.41792945	21.07006165	7.613314975	12.67759668	14.45984249	6.169570535
PYCR2	63.31966394	58.27251561	70.07346723	69.43244845	62.18301825	61.2202257	71.23895131	79.70779268	80.08362428	87.32380868	88.3662337	100.9308216
PYCR3	6.29669349	5.417703533	6.765997185	4.47421791	4.188561466	5.104247881	5.784965576	5.692208467	6.069745089	4.749089827	6.093983693	6.735834283
PYDC1	0.098372141	0	0	0	0	0	0	0	0	0	0	0
PYDC2	0	0	0	0	0	0	0	0	0	0	0	0
PYDC5	0	0	0	0	0	0	0.161798736	0	0	0.17252741	0	0
PYGB	19.30422881	16.98775722	19.68738119	20.82724201	16.72954789	17.13595919	18.53882168	22.30516208	20.15730853	17.43992764	19.76955712	27.12510205
PYGL	259.6509836	248.0688138	249.4435766	185.2650069	216.6133361	229.5464305	258.6714945	202.5824192	76.21215185	94.45675669	87.39060801	77.50515198
PYGM	0.104166738	0.092562149	0.063000864	0.210562047	0.108768692	0.137539858	0.153028672	0.160172072	0.534408873	0.293716465	0.43278927	0.247782738
PYGO1	0	0.022970608	0.005862959	0.005598638	0	0	0	0	1.302098366	1.33631484	1.253032507	0.847420026
PYGO2	17.02277303	18.75258682	17.7843119	23.73792894	19.84206757	20.50545318	16.96597128	24.72038427	29.20547035	28.63010994	27.50446881	44.86955794
PYHIN1	0.063831033	0.099260003	0.013511929	0.051611073	0.019995302	0.019665644	0.072204845	0.074950788	0.333427798	0.153985319	0.233771996	0.352068996
PYM1	16.74168939	17.53090345	15.57721056	16.62242614	13.03392821	15.67734013	18.9076279	19.47663179	29.23323648	28.02245365	31.66089926	27.47281401
PYROXD1	4.576084488	3.880439507	4.465537738	4.056473171	6.054709302	5.277184925	5.106321282	4.276594902	9.371204789	8.754569415	9.322348799	8.027184333
PYROXD2	0.313380312	0.274467513	0.223030271	0.098296318	0.197463395	0.14981749	0.238920797	0.17975696	1.175986687	0.491752492	0.72168403	0.820949817
PYURF	0	0	0	0	0	0	0	0	0	0	0	0
PYY	0	0	0.107661212	0.154211253	0.10621323	0.156693172	0.052301671	0	0.055348069	0	0.219136876	0
PZP	0	0	0	0	0	0	0	0	0.007399325	0	0	0
QARS	159.7501142	166.4031847	154.8687648	130.4974145	144.7990167	155.2621037	154.0400827	145.6774568	79.48229929	86.78098163	83.82975928	79.79991965
QDPR	14.61614811	13.09391362	16.50355853	15.00280513	11.11499283	12.0349456	16.26914192	15.03459296	11.30071628	14.49230247	13.92062695	11.6198562
QKI	5.23461063	5.173112566	5.032947035	5.136620335	6.132865622	5.781986313	5.31977703	4.327450586	9.321375673	9.984011598	8.684393763	9.299450873
QPCT	2.780529275	2.915318163	2.541639095	3.576711029	0.494892531	0.746324455	2.014551033	2.442502253	4.401325252	4.954565785	3.879996604	6.70803016
QPCTL	8.304082003	9.462759576	8.412140226	7.987891954	7.369141566	9.808331289	7.62375296	9.171238239	4.700166268	6.518067005	5.443651937	4.193546003
QPRT	31.82510993	33.81776716	28.67583619	13.5010652	28.94603945	27.50121654	27.43074081	15.22222792	104.2200264	83.1341788	96.00893871	76.80782817
QRFP	0	0	0	0	0.060286265	0	0.059372498	0.056494558	0	0.063309415	0	0
QRFPR	0	0	0	0	0	0	0	0	0.074520561	0.090105942	0.073761394	0.042758131
QRICH1	23.79917126	23.24882618	23.4291821	25.45444908	25.33733883	26.49810797	25.12172571	27.88595736	29.25856253	25.7513823	28.33622974	26.73419685
QRICH2	0.531501058	0.423457298	0.489625155	0.76598829	0.24665871	0.217322089	0.430170959	0.78974577	0.273136191	0.10792825	0.143128407	0.655556891
QRSL1	10.67528693	9.693547662	11.73033138	11.88388035	12.84953776	12.27138024	13.49318724	12.05383089	12.40758524	12.50211057	12.56934672	12.75353709
QSER1	5.118257636	4.220740608	4.863199124	4.800708451	6.219922133	5.335904484	4.859645891	3.789379367	11.60932479	11.69245393	11.41274403	10.39576027
QSOX1	100.079355	86.59172898	93.79207414	74.06561537	36.98707874	43.74900913	65.22333377	71.45754558	77.22752717	82.97031236	68.53560331	167.7876414
QSOX2	6.758860956	6.034044069	7.392546447	8.427900299	8.62027043	8.429354717	7.048210125	8.403546838	11.46604283	10.85003175	10.90140652	13.63505197
QTRT1	36.22262793	33.45374615	37.18581148	32.4109513	38.91799245	43.70298635	41.85396131	45.7456851	11.58874053	11.41164062	14.6859489	14.98621206
QTRT2	11.07688206	9.439123396	11.35974069	12.14504011	12.78228941	12.96568556	12.94238678	11.08482702	13.26417972	14.42458143	14.3835118	12.56050067
R3HCC1	18.21871853	19.1681123	18.42273731	28.13380024	21.53414749	21.94420979	20.44162589	28.62940848	29.22385013	26.32731366	27.79535443	32.21082035
R3HCC1L	5.394515011	4.879604598	5.29960186	5.647991771	7.008180607	6.106897329	6.035477274	6.055655363	5.364678338	4.460373871	4.256367007	4.727048876
R3HDM1	15.65420699	14.97197916	15.83692264	16.29066472	18.12573071	17.09748084	16.80895807	15.12312197	15.54133257	14.55898149	14.39351705	15.6702304
R3HDM2	18.54501987	19.34934342	17.04610829	15.48442657	20.73602841	20.37663707	15.52683056	15.78419269	17.23885278	18.40846682	15.23470271	17.88804394
R3HDM4	29.9891498	28.48727604	30.86473309	33.60274685	29.36213348	29.78305608	33.20110073	38.6208576	21.38902094	18.82869117	20.9122375	23.42782716
R3HDML	0	0	0	0	0	0	0	0	0	0	0	0
RAB10	36.73874792	37.89769186	34.94728454	40.49446157	33.0700281	35.89839338	34.79200485	38.17440329	46.91688167	49.87586039	50.3554587	51.30919194
RAB11A	15.74221683	16.4104233	15.49476156	19.18762593	18.23876604	18.7789142	18.43382951	19.21741085	25.41207382	26.45560302	24.27124292	25.43751968
RAB11B	36.30246003	39.62076661	40.94027434	45.8718365	38.66507137	39.14706518	38.11298856	53.13776192	35.55194367	25.89817536	34.62046511	38.32951025
RAB11FIP1	15.95802626	18.21855476	13.70199866	16.8275738	14.89736493	14.01900183	14.46036066	14.59008625	21.24040597	13.44488988	15.54494546	24.92877189
RAB11FIP2	5.680774754	4.666614286	5.458817285	5.607237633	6.634187522	5.970415341	5.95286495	4.307154192	7.384179043	6.547946466	6.253315459	6.75893485
RAB11FIP3	9.81332067	10.24681203	9.712224011	10.81264082	8.435873565	9.114658764	9.245277185	11.16093153	12.23935944	10.09119377	11.13291726	12.91078049
RAB11FIP4	0.01187849	0	0	0.012005556	0	0	0.024430538	0	0.413656562	0.514497304	0.319876952	0.543918664
RAB11FIP5	0.037178416	0.077085382	0.052466864	0.087677609	0	0.038180879	0.038232497	0.036379269	3.735752466	4.579565674	4.218309156	5.352266932
RAB12	4.815783056	4.446150868	4.808576604	4.940766752	5.199318752	5.412202967	5.027071989	5.210168186	9.018090247	8.787885541	8.084492945	9.928882773
RAB13	111.2700149	112.2502009	104.3322039	73.96515901	59.44947948	60.09068202	94.57037249	65.39776917	9.449498695	7.285381185	8.715512897	8.796893033
RAB14	22.05168343	21.13480476	22.20414417	24.56868377	24.68716318	21.50636228	24.46517049	22.54312861	37.72799922	36.1220912	33.84974122	37.52940183
RAB15	0.573850936	0.518638266	1.012286241	0.252815933	0.952579031	0.71021098	1.059191072	0.331150493	7.798160019	4.372487134	6.957940363	7.641085731
RAB17	2.474002798	1.878855743	1.705081932	0.726880094	1.727207122	1.093096481	1.627069892	0.872622709	1.127023392	0.741300613	1.177516567	0.359255165

RAB18	15.67771685	12.99802914	14.65778851	13.3558862	13.58859511	11.88805543	14.87200583	11.36848099	12.21785246	11.34877848	11.8542502	12.78403152
RAB19	0	0	0	0	0	0	0	0	0	0	0	0
RAB1A	31.3329225	33.93903431	33.37868265	35.3719655	27.18990369	24.2326415	32.80749454	31.19730827	51.2689394	55.0305648	50.70278428	56.44383901
RAB1B	63.34414846	64.27529573	68.54731548	71.12103811	61.42948766	64.06254857	68.47792217	79.92228187	87.53383195	84.94739097	86.91524083	82.41496752
RAB20	20.01065141	21.14811972	20.54341454	18.11647863	13.84365932	15.86650486	15.30624552	17.98917393	3.308810846	2.985109609	2.894276924	2.759472676
RAB21	9.355777557	9.297353616	9.973898143	9.853737109	9.287392641	8.445728521	9.608381345	9.305147141	6.981740068	7.001204912	7.18226872	7.431952624
RAB22A	3.800444074	3.836730136	3.199520485	4.054841492	3.312364733	3.391897295	3.524410762	3.657890408	5.834841936	5.360933792	5.07190032	6.712617974
RAB23	4.27883255	3.886965909	4.517339079	5.449437576	5.054555379	4.793678334	4.289031085	5.032689535	3.268914752	2.962066985	3.130863109	4.03686841
RAB24	44.51488039	39.78189061	39.70181547	29.49078544	30.05179174	31.09039988	38.36935762	33.16387404	19.50737743	18.8551918	17.30627056	25.460326
RAB25	0	0	0	0.048514869	0	0	0	0	0	0.052635481	0	0
RAB26	0.923916148	2.14622535	0.941667608	1.158602956	1.554292999	1.651663944	0.967705639	1.171925273	0.521345373	0.769214419	0.645042807	0.427336589
RAB27A	19.28619904	27.99750338	18.87490016	27.14852791	19.7033094	23.6958438	21.10805054	22.81468919	19.2172282	19.04497627	17.63371362	34.39572328
RAB27B	0.01588865	0.010981126	0.016816766	0.010705742	0.005530197	0	0	0.005182375	0.011527216	0.029037586	0.034229353	0.033070216
RAB28	9.967906975	10.89626156	10.36879193	11.83541355	12.01872603	12.05522743	11.57221664	11.06717602	13.83143545	18.54065266	14.45966068	16.97206541
RAB29	22.28506111	21.00940868	20.49623582	20.07141616	17.56595918	19.33519285	21.27356346	19.91542996	8.932347527	12.44125712	10.78175319	10.9155521
RAB2A	23.95457103	22.84339296	23.20243163	26.8073798	20.73841177	20.65743795	22.22406664	25.24909574	41.18433557	37.72131697	38.08868512	42.96872484
RAB2B	7.045885212	8.463313396	6.955517515	7.001427119	6.614372895	6.38356572	6.984388517	6.397239639	6.930407509	7.63323823	5.692178641	6.662758502
RAB30	0.335857639	0.455314994	0.366414091	0.381228856	0.312928204	0.30246266	0.478218274	0.323582429	0.331758898	0.492930725	0.294984664	0.172072668
RAB31	20.24326028	18.57235782	19.72811487	13.71395237	12.95267786	12.06165778	17.99723128	11.42922939	43.60900971	40.77826352	42.84428816	43.74913972
RAB32	0	0.046140713	0.094214692	0.089967194	0	0	0.091538739	0.043550809	40.87937879	43.04537483	47.03096054	58.63904543
RAB33A	0.040703147	0.042196764	0	0	0.085002721	0.041800649	0.293000132	0.159312945	0.088590404	0.223163292	0.175375805	0.296514593
RAB33B	2.147417308	2.174744306	1.734202337	2.408754872	2.151559071	2.256309381	1.927476436	1.991944693	2.71515794	2.300285874	2.005595282	3.216545746
RAB34	18.35462099	21.08700482	20.42246198	13.5434705	19.07819037	18.11959092	21.08927727	14.83037025	0	0.160462557	0.022518144	0
RAB35	28.78570747	29.21802571	30.70608567	31.84634873	27.88914082	27.73840132	31.86104884	34.51299329	29.23000248	23.03414614	28.44597034	31.6324876
RAB36	0.008481961	0.00879321	0.026932274	0	0	0	0	0	0.046152472	0.027902447	0.036545841	0.052962389
RAB37	9.778373179	16.52096637	7.588675231	8.16858029	4.152613727	7.377820164	6.185812144	6.918106609	4.514027233	4.032616843	3.930647983	3.486024166
RAB38	0.101743438	0.10547695	0.094225826	0.141393726	0.119518125	0.130608505	0.104628065	0.124445588	0.069201438	0.181294461	0.15069221	0.291178391
RAB39A	1.795582763	2.131686028	2.482871521	2.195310896	1.723706955	2.498319978	2.174094325	1.785325341	2.489829014	2.731095906	2.776421758	3.556442445
RAB39B	0.015321629	0.031767723	0.016216623	0.015485526	0.015998519	0	0.015756027	0.029984583	0	0.03360158	0.016503902	0
RAB3A	2.460413412	2.153187595	2.096836366	1.970009076	2.402285927	2.526754934	2.004421164	2.376260423	1.634345654	2.382599439	1.445600685	1.795689155
RAB3B	0.00407463	0	0.004312644	0.004118217	0	0	0	0	0.886843209	0.741687577	0.886586714	0.839601845
RAB3C	0.005900818	0	0	0	0	0	0.006068118	0	0.077058788	0.103527798	0.057205322	0.257917946
RAB3D	11.72401151	14.32136239	10.15633498	13.52947209	7.718617083	9.065663809	10.82616437	12.7781621	13.53979826	12.82823865	11.82836546	23.57641446
RAB3GAP1	13.30762967	12.07012318	12.44337286	14.41144264	13.64721627	13.64521315	13.34466481	12.77875643	18.25163201	17.07543962	15.13642492	18.03500278
RAB3GAP2	17.18763569	17.14092537	16.38101555	16.51813144	17.30298874	18.75191165	18.21100081	12.75193721	24.41725114	23.78208785	23.67817448	22.67768205
RAB3IL1	0.099384413	0.206062719	0.120216953	0.014349649	0.059300051	0.058322384	0.073001541	0.027785186	4.913331331	4.45257284	5.077384067	2.039010037
RAB3IP	1.675604227	1.997235196	1.833455464	3.38296611	1.639750122	1.741566915	1.444249963	2.57423117	0.766388622	1.216035996	0.815256757	1.874352011
RAB40A	0	0	0	0	0	0	0	0	0	0.043836831	0.086124374	0.24962362
RAB40AL	0.052293062	0	0.166043094	0.105704895	0.054603303	0	0	0	0.170723782	0.114682943	0.168984559	0.217682794
RAB40B	0.21504709	0.148625546	0.303478408	0.084524019	0.037424599	0.036807588	0.184286749	0.058451297	1.781190324	1.19213989	1.338369711	2.088768873
RAB40C	9.503359197	10.06130255	8.427380527	10.36526057	9.118621257	10.66396848	9.037008324	11.79439956	6.907997594	6.920390309	7.15381409	10.53917223
RAB41	0	0	0	0.018676225	0	0	0	0	0.020109292	0	0	0
RAB42	0	0	0	0	0	0	0	0	0	0.079663872	0	0
RAB43	0.17028342	0.141225625	0.192245675	0.114736643	0.082976285	0.069949948	0.081718602	0.133298553	0.123540627	0.174274523	0.122282075	0.22446816
RAB44	14.74143989	19.26573859	14.15948294	21.18783365	14.9785446	21.47792086	14.53907743	21.49132117	15.62872401	22.26255341	15.96795092	25.6432304
RAB4A	8.844193304	9.297353616	9.379575313	9.171675337	9.160891081	7.990560379	8.183626607	9.191716736	16.99269581	17.04441248	16.64776205	20.06811658
RAB4B	26.61061546	21.39407863	24.4288633	27.58137651	15.68882855	15.75550501	21.8269081	24.66583774	15.16900183	13.94468393	14.77072852	15.35377714
RAB5A	17.3634007	18.66072394	17.90586458	18.75054728	17.88668501	17.30840399	19.99487723	17.80022074	25.64089363	27.72158179	23.20754604	28.40811988
RAB5B	8.140444821	9.607211327	7.751381856	8.692516914	5.333383084	6.836444796	5.291165939	6.43117254	9.788659966	10.01766816	9.901327496	10.14253306
RAB5C	61.5944502	57.3351411	62.77688759	63.09909759	43.98033578	49.94795454	61.64577609	67.86322289	64.29818047	62.2854847	64.1348163	82.65247463
RAB5IF	11.07289562	9.281071035	12.86674043	13.4772353	6.445245727	7.742270903	9.884741022	13.09407324	7.230047501	6.716785171	7.257901597	9.463911975
RAB6A	26.75362998	26.57580629	25.2767962	27.9411442	24.16347997	23.90480511	27.27899428	25.01014095	24.85436135	30.62923702	26.03392251	27.08710781
RAB6B	2.952098292	3.462598023	2.914235864	1.310140337	6.441672711	6.636696536	4.320171362	3.370083684	4.993978315	4.16049835	4.994062738	3.298686673
[truncated: 890,480 more chars]
